# Role of solvent accessibility for aggregation-prone patches in protein folding

**DOI:** 10.1038/s41598-018-31289-6

**Published:** 2018-08-27

**Authors:** Avinash Mishra, Shoba Ranganathan, B. Jayaram, Abdul Sattar

**Affiliations:** 1 Institute for Integrated and Intelligent Systems, Griffith University, Brisbane, Australia; 2 Novo Informatics Pvt. Ltd, Delhi, India; 3 Department of Molecular Sciences, Macquarie University, Sydney, Australia; 4 Department of Chemistry, Indian Institute of Technology, Delhi, India

## Abstract

The arrangement of amino acids in a protein sequence encodes its native folding. However, the same arrangement in aggregation-prone regions may cause misfolding as a result of local environmental stress. Under normal physiological conditions, such regions congregate in the protein’s interior to avoid aggregation and attain the native fold. We have used solvent accessibility of aggregation patches (SAAP_p_) to determine the packing of aggregation-prone residues. Our results showed that SAAP_p_ has low values for native crystal structures, consistent with protein folding as a mechanism to minimize the solvent accessibility of aggregation-prone residues. SAAP_p_ also shows an average correlation of 0.76 with the global distance test (GDT) score on CASP12 template-based protein models. Using SAAP_p_ scores and five structural features, a random forest machine learning quality assessment tool, SAAP-QA, showed 2.32 average GDT loss between best model predicted and actual best based on GDT score on independent CASP test data, with the ability to discriminate native-like folds having an AUC of 0.94. Overall, the Pearson correlation coefficient (PCC) between true and predicted GDT scores on independent CASP data was 0.86 while on the external CAMEO dataset, comprising high quality protein structures, PCC and average GDT loss were 0.71 and 4.46 respectively. SAAP-QA can be used to detect the quality of models and iteratively improve them to native or near-native structures.

## Introduction

The folding of a protein is a self-assembly process where the information of three dimensional (3D) structure is cryptically encoded in the primary sequence^1^. Successful prediction of a protein’s 3D structure from its primary sequence has been considered as a grand challenge in modern biology^2–4^. Protein folding involves a deep insight of the protein folding pathway, involving several intermediates^5,6^. Exhaustive sampling of all possible conformations is not a feasible theoretical solution for protein structure prediction (PSP) due to the large degrees of freedom available for proteins^7^. The dedicated pathway of protein folding is therefore via the thermodynamic folding hypothesis^8^, with the native state of a protein considered its most stable thermodynamic conformation. The hydrophobic effect is known to be a principal factor in the thermodynamic protein folding hypothesis, in addition to electrostatic interactions and conformational entropy^9^. Clustering of hydrophobic groups in a polar solvent is an “entropy-driven” process, which leads to the collapse of side chains to functional native conformations. This “hydrophobic collapse” is considered as the most popular protein folding model^10–12^. In contrast to protein folding which leads to the native state, protein misfolding is also a self-assembly process that results in an aggregated form. The same protein sequence can undergo folding or misfolding, depending on the physiological environment^13^. In the crowded cellular environment, there is always a possible chance for the protein to move in the direction of misfolding, suggesting that protein misfolding information is also encoded in its primary sequence.

The primary sequence of proteins may have several aggregation patches that are responsible for the formation of fibrils or amyloids. However, under physiological conditions, these patches self-assemble in the core of globular structures^8,14^ ruling out misfolding or aggregation and leading to the native structures. Aggregation-prone regions can be detected from protein sequences using several prediction programs^15–18^ and from protein structures^19^.

In this study, we have studied the packing and spatial positions of these ‘aggregation-prone’ patches in the native and non-native states of proteins. We developed a hypothesis on solvent accessibility of aggregation patch (SAAP), based on the first (http://predictioncenter.org/casp12/target.cgi?id=3view=all) CASP12 target^20^, which was applied to 1557 native structures from the Protein Databank (PDB)^21^ for validation. We then used the predicted structures from the CASP 12 homology model dataset to test our hypothesis, by comparing SAAP with the global distance total (GDT) score, which measures the deviation of the model from the experimental 3D structure. We observed that SAAP decreases exponentially as we move from non-native to native folds. Here, we have used folds in the context of structural domains, rather than complete protein structures which could comprise several domains. Our results support folding as a preferred pathway for globular proteins, accompanied by burying aggregation-prone residues from the solvent in their native states while these residues are more exposed in their non-native states or in aggregates. Thus, the SAAP score for the entire protein fold, SAAP_p_ provides a direct structural metric to identify near native folds from misfolded structures. Moreover, minimizing SAAP_p_ at an early stage of structure prediction can filter out non-native states, opening a new avenue for improved protein structure prediction. Quality assessment of predicted protein structure is broadly classified into single model quality assessment^22–30^ and consensus model quality assessment^31–33^. These scoring functions have been used to detect the quality of predicted protein models. Therefore, SAAP_p_ was trained using a machine learning method (random forest) to evaluate the quality of these models applying the protein folding with aggregate formation paradox. The scoring function developed using SAAP_p_, SAAP-QA, showed excellent results comparable with the state-of-art methods in this field.

## Results

We calculated the SAAP_p_ score for the target ‘T0859’ from CASP12 as a case study, followed by validation on 1557 PDB native structures. Our hypothesis that SAAP_p_ is a measure of protein folding was then tested on CASP12 TBM (template based model) predictions. Based on the results obtained, the SAAP_p_ score was formulated into a scoring function, SAAP-QA, using random forest machine learning approach for evaluating the quality of protein models. Here 10978 CASP11 and CASP12 TBM models were used as training and test sets, with the remaining 4305 CASP12 models forming the blind test set. Furthermore, we validated SAAP-QA additionally on 51 targets from the CAMEO platform^34^.

### Aggregation patches in T0859-a case study

**Figure 1:**
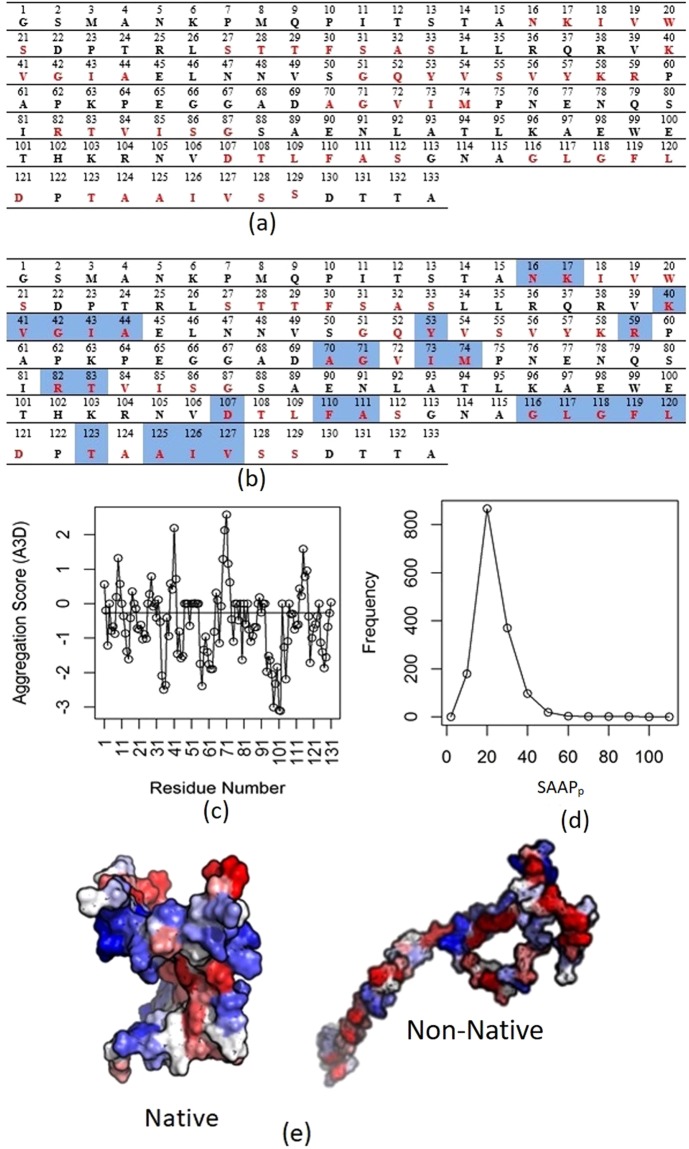
Aggregation-prone regions. (**a**) Aggregation-prone regions predicted by Aggrescan server for the CASP 12 target T0859, highlighted in red. (**b**) Solvent accessibility of aggregation-prone residues predicted from side chain solvent accessible surface area (SASA) and aggregation propensity. (**c**) Individual aggregation score for each residue predicted by the A3D server, where positive scores correspond to aggregation. (**d**) Frequency of loss in SASA of aggregation-prone residues on native protein structures collected from PDB, as measured by SAAP_p_ scores. (**e**) SAAP_p_ scores mapped to the 3D surface of the native T0859 structure and its corresponding decoy, coloured by predicted aggregation propensity from red (highly aggregation-prone) to blue (least aggregation-prone or hydrophilic).

In order to demonstrate the concept of minimum solvent accessibility of aggregation patches, the first CASP12 protein target T0859 was chosen as a case study. This is the Acinetobacter phage 205 (AP205) coat protein of 133 amino acids. Initially, the complete primary sequence of this protein was submitted to the Aggrescan server^35^ to predict aggregation-prone regions in the polypeptide sequence. The Aggrescan server uses aggregation propensity values per amino acid derived from experimental data, change in hydrophobicity, *β*-sheet propensity and the charge of the protein^35^. A window size that depends on the protein length is selected to calculate average aggregation propensity and the resultant value is assigned to the central residue as its aggregation value. Aggregation-prone “hot spot” patches have a high propensity to nucleate and initiate the aggregation process when exposed to a polar solvent. Figure 1a shows the primary sequence of the selected protein, T0859 with the corresponding aggregation-prone residues shown in red. These nine hot spot regions constitute 62 residues. Thus, 47% region of this protein has been predicted as aggregation-prone at sequence level. Many predicted aggregation-prone regions are shielded because they are buried in the protein’s hydrophobic core or involved in non-covalent interactions at the protein secondary and tertiary structural levels. Further, native structure of 131 residues (missing residues 1 and 2) of T0859 (PDB code: 5JZR) was submitted to the Aggrescan-3D (A3D) server^19^ to detect aggregation-prone residues for the given structure. The residue-wise scores are shown in Fig. 1c, with residues having positive scores considered aggregation-prone. The aggregation propensity of the same amino acids in the native 3D structure showed a reduction in aggregation-prone patches compared to the sequence-based prediction, due to protein folding. The A3D server detected 27 aggregation-prone residues out of 131 that constitute 21% of the complete structure. The results indicate that 35 residues having a high propensity to participate in aggregation from sequence-based analysis, this propensity was diminished in the native structure. In summary, structure-based prediction lowered aggregation-prone regions by 55.3% (47-21/47) in the native structure, from the sequence-based method. To illustrate further, aggregation prone patches on 3D structure of native and non-native conformations of AP205 are shown in Fig. 1d. It shows that non-native has bigger area for aggregation-prone regions than native. As the A3D server is only accessible via RESTful URIs, and is therefore unsuited for local installation, large-scale structure analysis using this method is unfeasible. Moreover, A3D score has major contribution of solvent exposed surface of individual residues. We therefore determined the spatial location of all AP205 residues, by calculating their solvent accessible surface areas (SASA; described in the Methods section), using a local copy of the naccess program^36^. The side chain accessible surface area for each residue is used as a marker to represent the exposure of any given residue to the solvent. Residues that showed greater than 50% of side chain solvent accessible surface area (SC_sasai_; described in the Methods section) are considered surface exposed residues, compared to earlier studies with a minimum cut-off 20% for solvent exposure^37,38^. Figure 1b shows the residue-wise solvent accessibility from naccess and aggregation propensity predicted by Aggrescan. Residues with SC_sasai_ > 50% are given a score of ‘1’ and considered as surface accessible while all others are classified inaccessible and marked as ‘0’. As noted earlier, 62 residues (in red) are predicted as aggregation-prone using sequence-based prediction, and 27 of these (highlighted in blue) are solvent accessible as shown in Fig. 1c, consistent with A3D results. The reduction in solvent accessibility of aggregation-prone residues by 21% in their native structure suggests close packing of these residues in the interior of the protein. Figure 1a–c collectively demonstrates reduction of aggregation propensity as we move from primary sequence to structure. This can be quantified as solvent accessibility of aggregation patches (SAAP_p_) score (as defined in Eq. 1 in the Method section), computed as 43.5 for AP205 protein. Thus, SASA of predicted aggregation-prone patches may act as marker of their 3D location. The arrangement of aggregation-prone residues in tertiary structures could be perceived as a strong driving force for native protein folding.

### Validation on native crystal structures

As shown in the case study, the native protein structure leads to considerable loss in solvent accessibility for aggregation-prone residues. In order to examine the universality of this phenomenon, native crystal structures from the PDB database were analyzed. Aggregation-prone residues are predicted using the Aggrescan server for their primary sequences and their corresponding SASA was calculated using the naccess program. The SAAP_p_ score was computed as per Eq. 1 (see Methods section). SAAP_p_ score is intrinsically normalized with respect to the number of aggregation-prone residues in the polypeptide, to address the amino acid length of different native proteins. Among the 1557 selected native structures, 51 did not show any aggregation patches in their polypeptide sequence. Therefore, SAAP_p_ was calculated for the remaining 1505 structures. Figure 1d shows the frequency distribution curve of these structures for different ranges of SAAP_p_. From the plot, 1358 structures have SAAP_p_ ≤ 30, i.e. 90% of native structures have only 30% predicted aggregation-prone residues as solvent-exposed. This distribution confirms that native structures tend to move their aggregation-prone residues into the core, in order to reduce their solvent accessibility. In this calculation. 10 proteins with SAAP_p_ > 50 are either aggregation-prone (coiled coil: 1M5I, 3K29, 3QFL, 5APZ and 5VO5), multimeric (trimeric: 2WB3, 2WH7, 3EMI and 3WP8) or phosphoinositide-binding (2WWE).

### SAAP as a measure for CASP12 models

We then applied this concept of non-native/decoy structures being unfolded and thus aggregation-prone, to investigate the potential of SAAP_p_ as a structural metric to detect the quality of predicted models for the CASP12 template based model (TBM) category. These predicted models contains near native structures in pools of ‘decoys’. Targets selected under the CASP12 TBM category with their different domains, are shown in Table 1, comprising 37 structural domains. Of these, 30 were selected, as five domains are small (<100 amino acids in length) and therefore have no significant hydrophobic core, while another two domains have very small aggregation-prone patches (<20%) of their complete sequence. The best models predicted by different servers were taken with their corresponding GDT (global distance total) scores, resulting in 6149 models from 30 target domains. Aggregation-prone residues for each target sequence were predicted followed by SAAP_p_ calculation for each model structure. Inferences drawn from the case study and their extension to native crystal structures suggest that low SAAP_p_ scores are representative of native or near native structures. Plotting SAAP_p_ against GDT gives an exponential relation from curve fitting. (*y* = *e*^(*a* + *bX*)^) as shown in Supplementary Figure S1. Overall, the SAAP_p_ score decreases from high quality models (near native) to poorly predicted models. Native and near native structures have the least SAAP_p_ scores, maximizing their chance of attaining the native state. Each plot in Supplementary Figure S1 shows the top 10 models in red colour as per their GDT score. It can be seen that the top ten models can be detected using SAAP_p_ scores. Pearson correlation coefficient (PCC) values between GDT and SAAP_p_ scores are shown for each target in Table 2 with the sequence length of the model domain, to provide some indication of the globularity of the protein fold. The average PCC for 30 models (Table 2) is 0.76, where the maximum is 0.86 for T0910-D1 targets and the minimum is 0.52 for T0911-D1. These correlations clearly suggest that SAAP_p_ can be used as structural metric to rank predicted protein structural models in the absence of their native structure. There is no observed correlation found between PCC values and the length of the proteins. A total of 3996 models out of 6149 have GDT > 50 where 99.7% of them showed SAAP_p_ ≤ 50. This shows that the GDT cut-off score of 50 for SAAP_p_ can be used to screen good models from set of decoys. Similarly, of 786 high quality models (GDT > 80), 89.4% have SAAP_p_ < 40. This again confirms the possible application of SAAP_p_ in screening high quality models from poor predictions.

**Table 1:** CASP12 models under TBM (template based modeling) category for specific domains.

S. No.	Target	Type	Domain	Residue in Domain	Category
1	T0860	Server only	T0860-D1: 1–136	136	TBM
2	T0861	Server only	T0861-D1: 2–313	312	TBM
3	T0865*	Server only	T0865-D1: 11–72	62	TBM
4	T0867	Server only	T0867-D1: 1–104	104	TBM
5	T0871	Server only	T0871-D1: 33–143, 160–305, 313–374	319	TBM
6	T0872*	All groups	T0872-D1: 1–88	88	TBM
7	T0873	Server only	T0873-D1: 16–281, 306–501	462	TBM
8	T0877	Server only	T0877-D1: 1–142	142	TBM
9	T0879	Server only	T0879-D1: 4–223	220	TBM
10	T0881	Server only	T0881-D1: 1–202	202	TBM
11	T0882*	All groups	T0882-D1: 5–83	79	TBM
12	T0883	Server only	T0883-D1: 15–231	217	TBM
13	T0885	Server only	T0885-D1: 2–115	114	TBM
14	T0889	Server only	T0889-D1: 4–242	239	TBM
15	T0891	Server only	T0891-D1: 12–64, 72–130	112	TBM
16	T0893*	Server only	T0893-D1: 1–73	73	TBM
17	T0893	Server only	T0893-D2: 74–242	169	TBM
18	T0895	All groups	T0895-D1: 1–120	120	TBM
19	T0902	Server only	T0902-D1: 26–49, 72–188, 214–303	231	TBM
20	T0903#	Server only	T0903-D1: 15–155, 168–350	324	TBM
21	T0906	Server only	T0906-D1: 2–34, 39–224, 234–264, 268–284, 288–353	333	TBM
22	T0910	Server only	T0910-D1: 29–345	317	TBM
23	T0911	All groups	T0911-D1: 27–443	417	TBM
24	T0912	All groups	T0912-D1: 24–113, 299–622	414	TBM
25	T0913	All groups	T0913-D1: 49–386	338	TBM
26	T0917	Server only	T0917-D1: 19–409	391	TBM
27	T0920	Server only	T0920-D1: 1–321	321	TBM
28	T0920	Server only	T0920-D2: 322–562	241	TBM
29	T0921	Server only	T0921-D1: 5–142	138	TBM
30	T0922*	Server only	T0922-D1: 23–96	74	TBM
31	T0928	Server only	T0928-D1: 6–98, 137–386	343	TBM
32	T0942#	All groups	T0942-D2: 270–483	214	TBM
33	T0943	Server only	T0943-D2: 10–60, 152–551	451	TBM
34	T0944	All groups	T0944-D1: 2–147, 165–271	253	TBM
35	T0946	All groups	T0946-D2: 1–49, 130–292	212	TBM
36	T0947	All groups	T0947-D1: 42–216	175	TBM
37	T0948	All groups	T0948-D1: 1–125, 138–161	149	TBM

The TBM category has 37 domains. Of these, five domains are smaller than 100 residues (marked*) and two are very small aggregation patches (marked^#^), and were not selected.

**Table 2:** Individual pearson correlation coefficient (PCC) between SAAP_p_ and GDT scores for the selected CASP 12 models under TBM category along with sequence length.

Target	PCC	Sequence Length	Target	PCC	Sequence Length
T0860-D1	0.6	136	T0911-D1	0.52	417
T0861-D1	0.85	312	T0912-D1	0.71	414
T0867-D1	0.68	104	T0913-D1	0.84	338
T0871-D1	0.77	319	T0917-D1	0.82	391
T0873-D1	0.85	462	T0920-D1	0.83	321
T0877-D1	0.75	142	T0920-D2	0.73	241
T0879-D1	0.75	220	T0921-D1	0.83	138
T0881-D1	0.75	202	T0928-D1	0.83	341
T0883-D1	0.85	217	T0943-D2	0.69	447
T0885-D1	0.59	114	T0944-D1	0.81	253
T0889-D1	0.78	239	T0946-D2	0.75	212
T0891-D1	0.78	112	T0947-D1	0.7	175
T0893-D2	0.83	169	T0948-D1	0.73	149
T0895-D1	0.83	120			
T0902-D1	0.8	231			
T0906-D1	0.82	333			
T0910-D1	0.86	317			

### SAAP Based Scoring Function, SAAP-QA

SAAP_p_ has the potential to be developed further, as a scoring function for quality assessment of protein structure models by predicting GDT score of a given protein model. Machine learning approaches have been extensively used to build quality assessment scoring functions^39–42^. This requires strong descriptors/features for representing the quality of models. For 30 CASP12 target domains, SAAP_p_ correlates with GDT with an average PCC of 0.76, suggesting its importance as a major descriptor in building a scoring function. In addition to SAAP_p_ other physico-chemical descriptors were added to build a robust universal scoring function. The random forest^43,44^ machine learning method is used to for formulating a decision based prediction algorithm to predict the GDT score using input descriptors. Protein are structurally diverse in nature, therefore physico-chemical descriptors of proteins may not follow any strict rules to represent structural folds. In such a case, any linear or logistic regression approach would not fit the prediction method. Random forest is a decision tree method and it is highly applicable when individual descriptors have diversity as well as belong to multiple classes. Earlier methods in protein quality assessment used support vector machine (SVM)^33,45^ this technique is intrinsically suited for binary class problem. Protein quality assessment cannot be purely represented as binary problem (good vs bad models) as it is an example of multiclass problem where models span across continuous quality spectrum. Random forest (RF) is a fast machine learning method as one of the most powerful scalable and interpretable prediction model. In addition to this, RF is equally competent for classification and regression problem. Overfitting can result in biased prediction model specially when the dataset size is moderate and RF is less prone to overfitting/overtraining than support vector machine (SVM) or neural network (NN). SVMs are also best designed for a binary classification problem. These reasons made random forest more suitable for building the SAAP_p_ scoring function, SAAP-QA.

### Physico-chemical Descriptors for SAAP-QA

Here, SAAP_p_ is used as major descriptor to build a scoring function for quality assessment. In addition, other descriptors that influence the SAAP_p_ score are also included to build a robust scoring technique. TBM category models from CASP11 and CASP12 are used as dataset for training and testing. This dataset comprises 10978 models with 9135 from CASP11 and 1843 models from CASP12. Moreover, 4305 models from CASP12 are additionally used for blind testing. These models belong to different domains/targets, with varied secondary structure (helix, sheet and loop) composition. Residues involved in these secondary structural elements differ in their propensity for solvent accessibility. In order to account for this effect, helix, sheet and loop fractions were used as descriptors in addition to the SAAP_p_ score. Moreover, loop residues show high degrees of freedom in the tertiary structure of proteins and are therefore more susceptible to solvent accessibility changes. This attribute makes the loop element another critical component that influences the SAAP_p_ score. To provide a higher weightage to loops in scoring function, the total loop content and loop to SAAP_p_ ratio were added in the descriptor list. In summary, six descriptors (detailed in Method section) are used to build the scoring function: (1) SAAP_p_ (2) helix fraction (3) sheet fraction (4) loop fraction (5) loop content and (6) SAAP_p_/loop ratio. Density plot and individual correlation for all six descriptors for CASP11 and CASP12 models are shown in Supplementary Figure S2. As expected, SAAP_p_ has highest correlation with GDT (PCC = 0.57) followed by loop fraction (PCC = 0.47) and then the helix fraction (PCC = 0.27). Supplementary Figure S2 shows, SAAP_p_, loop fraction and total loop content follow normal distribution pattern for the dataset with bell shaped curve while the SAAP_p_/loop ratio has skewed distribution. Helix and sheet fraction are multimodal distribution with more than one peak value. The GDT distribution also shown in Supplementary Figure S2, shows a multimodal pattern with two major peaks at values ‘13’ and ‘75’. From Supplementary Figure S2, the six descriptors that were selected to build a scoring function using RF machine learning method, showed uniform distributions on the combined CASP11 and CASP12 dataset. These six are distinct in nature, contributing individually to GDT score prediction with maximum PCC of 0.71 with SAAP_p_ (shown in Supplementary Figure S3). Further, after the data is divided into training, test and blind test sets, the individual correlation of these features with their GDT was re-calculated. Here again, SAAP_p_ has maximum PCC of 0.57, 0.58 and 0.64 with GDT while SAAP_p_/loop has low individual PCC of 0.28, 0.26 and 0.15 with GDT on training, test and blind datasets respectively.

### Training and Test Dataset Performance (CASP11 and CASP12 Targets)

**Figure 2:**
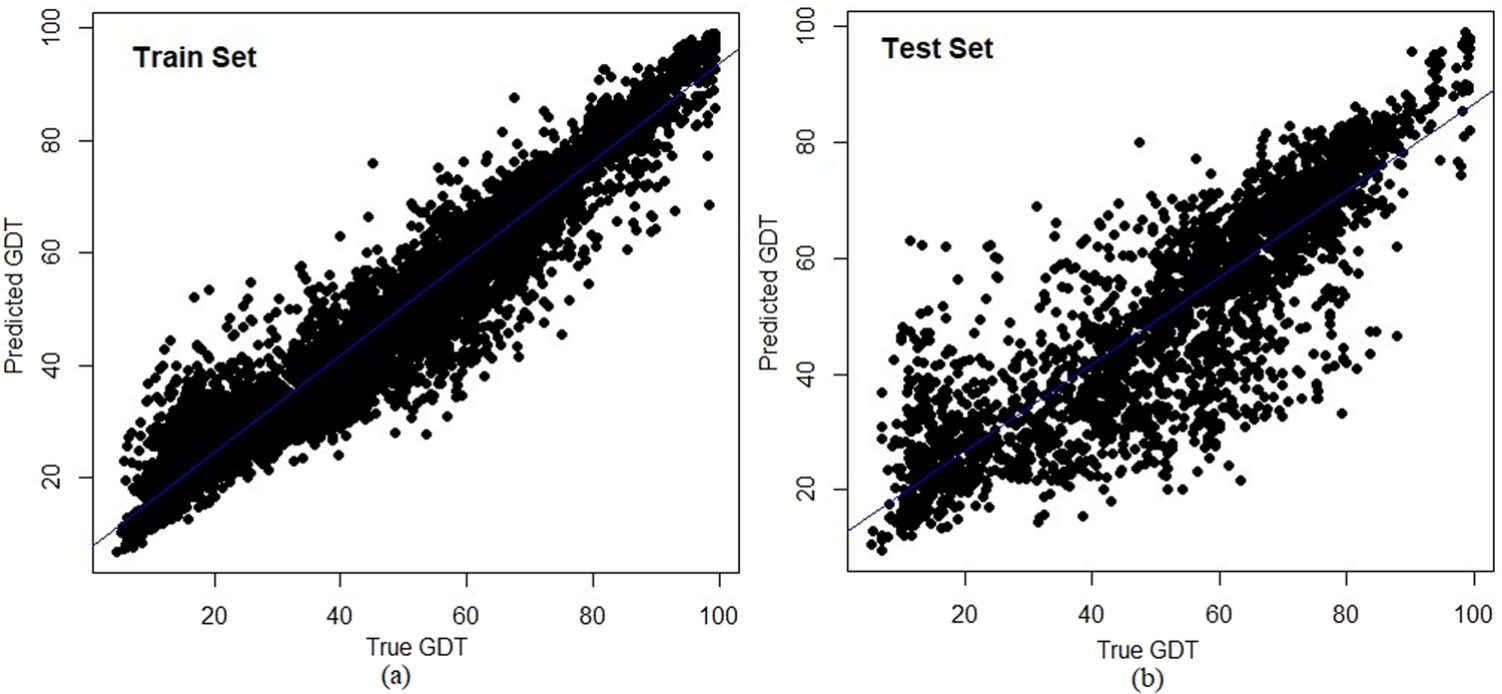
Evaluation of GDT predicted by SAAP-QA on CASP11/CASP12 train and test set. Compared to true GDT values, SAAP-QA predicted GDT values show correlation coefficient values (r) of (**a**) 0.96 for the training set with with 38 targets consisting of 7907 models and (**b**) 0.86 for the test set with 15 targets with 3071 models.

**Figure 3:**
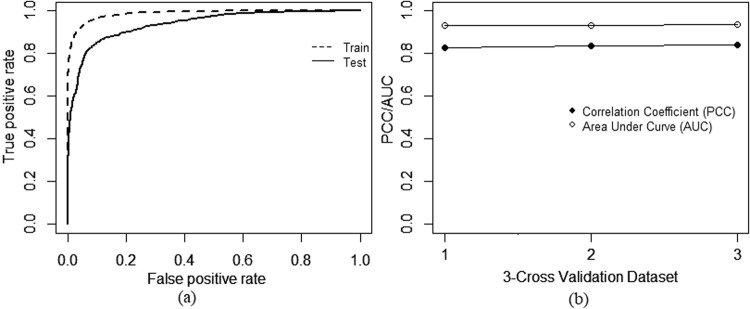
SAAP-QA performance on train and test set from CAP11/CASP12. (**a**) Classification of good and bad models representing the ROC curve for training set (38 targets with 7907 models) with an AUC of 0.98 and for test set (15 targets with 3071 models) with an AUC of 0.94. (**b**) Shows correlation coefficients (PCCs) and area under the curve (AUC) for 3-fold validation test of prediction model, average PCC for three distinct dataset is 0.83 while average AUC is 0.93. Standard deviation of these 3 runs are 0.007 and 0.002 for PCC and AUC respectively.

Structures from CASP11 and CASP12 are used as datasets for building the prediction model, with 53 unique targets divided randomly into training and test sets, comprising fractions of 70% and 30% respectively. T0852 has two domains so it was counted once. The resulted in 38 targets consisting of 7907 protein models in the train set and 15 targets with 3071 protein models in the test set. These targets are listed in Supplementary Table S1 and their division into train and test sets is shown in Supplementary Table S2. As the models are randomly split into train and test sets based on targets, the two datasets do not have any common target protein, making the learning process unbiased. A complete list of models with their individual GDT and the six features that were used in prediction model building are shown in Supplementary Table S2. The prediction model showed a high correlation between observed and predicted GDT on train and test set with Pearson correlation coefficient (PCC) values of 0.96 and 0.86 respectively. Figure 2 shows the relation between predicted GDT using SAAP-QA and the other descriptors with the true GDT score for the training and the test sets. Structural metrics for accessing the quality of models are evaluated on their classification ability characterizing good and bad models. SAAP-QA was tested on this parameter using receiver operating characteristic (ROC) values, with GDT cut-off set as 50 based on earlier study^46^. Hence, models with GDT > 50 score are considered as high quality models while GDT < 50 were considered as low quality models. True positive and false positive rates (TPR and FPR) on different cut-off values of predicted GDT were calculated and plotted to determine its ability to categorize good and bad models. Figure 3(a) shows ROC curves for training and test datasets have good classification for all targets. The area under the curve (AUC) for each ROC was computed as 0.98 and 0.94 for training and test sets, respectively (shown as label in Fig. 3). Figures 2 and 3(a) collectively show the high performance of SAAP-QA as a structural scoring metric to rank protein models and for further classifying them as good and poor quality models. Furthermore, 3-fold cross validation was performed to assess how the results of prediction model could be generalized to an independent set. Here, the complete data set was randomly divided into three groups based on targets having 18 (3812 protein models), 18 (3869 protein models) and 17 (3297 protein models) targets respectively. The complete list of these three sets used in cross validation is given in Supplementary Table S2, again with none of the sets having any common target and thus comprised of distinct protein models. In each run, two sets were used to train the machine learning model while the third independent set was used for testing. The average AUC of 3-fold cross validation is 0.93 while PCC is 0.83 as shown in Fig. 3(b). Consistent AUC and PCC values with standard deviation values (*σ*) of 0.007 and 0.002, respectively on the three distinct cross-validation datasets confirms the robustness of prediction model.

#### Training and Test Set Per Target Evaluation

Top model for each target is selected based on the prediction made by SAAP-QA. The GDT loss for each target is calculated as the difference between the GDT score of best model selected by SAAP-QA with the best GDT score model available in the decoy set, as shown in Supplementary Table S3. This table shows that the average GDT loss on train set for 38 targets is 0.80 while it is 2.02 on the test set for 15 targets. Pearson correlation co-efficient (PCC) between predicted GDT and actual GDT for each target is calculated as shown in Supplementary Table S4. The average PCC for the train set is 0.96 while for the test set, the average PCC is 0.86.

### Blind Test Set Performance (CASP12 and CAMEO)

CAMEO dataset is a repository of high quality models. Here, models are deposited more frequently than CASP experiment but the number of models for each target is lesser than CASP. A dataset of 51 targets from the CAMEO platform used in the blind test set comprise 1489 models, as shown in Supplementary Table S5. These structures are completely unknown for the prediction model as they are not from train/test set. Similarly, 18 CASP12 targets were also excluded from training and test sets for further use of blind testing. Although the number of targets for the blind set from CASP12 is 18 but it comprises of 4305 models, which is larger than the prediction model test set of 3071 models. CASP12 models considered in the blind test is shown in the Supplementary Table S5 with their corresponding six physico-chemical features used in building the SAAP-QA and their respective GDT score. In summary, 5794 models were used in the blind test set from 69 targets (CAMEO + CASP). Further, CASP12 targets were separately tested for domains and full protein structure.

#### CAMEO-Complete Structures

**Figure 4:**
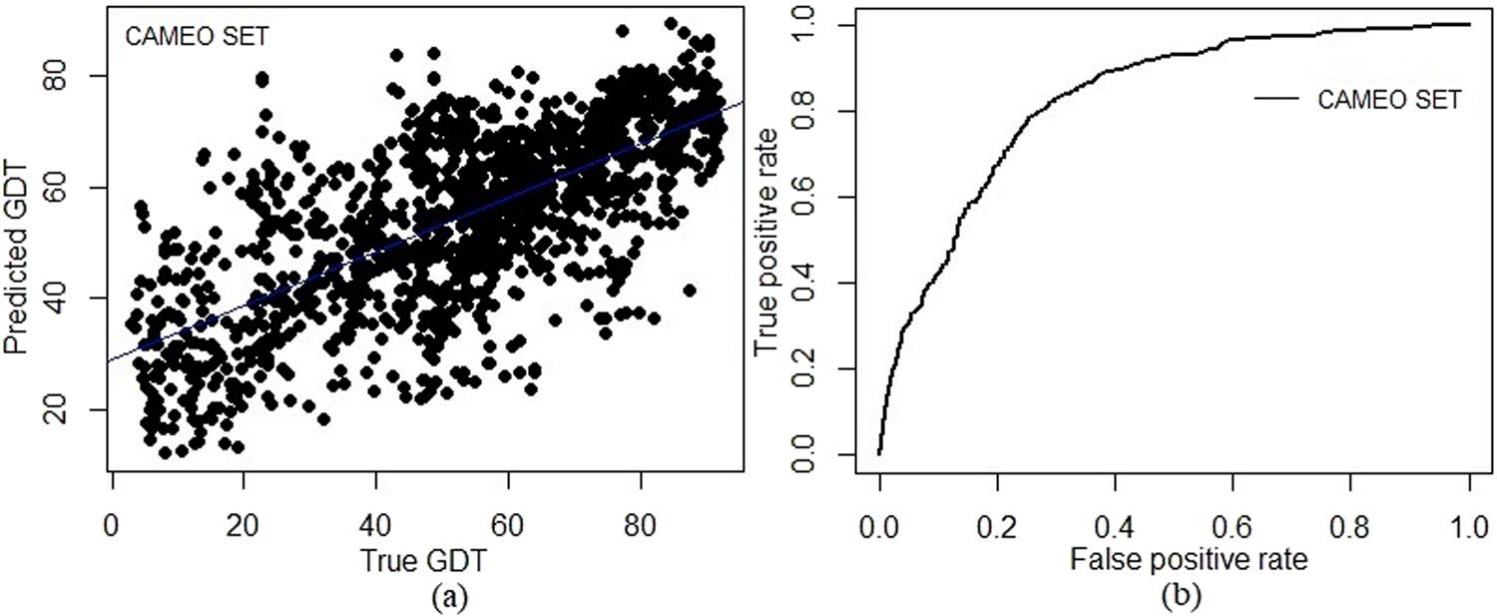
SAAP-QA performance on CAMEO Set. (**a**) Comparison between predicted GDT by SAAP-QA and true GDT on CAMEO dataset showed overall correlation coefficient (PCC) 0.71 (**b**) classification of good and bad models representing the ROC curve for blind test on CAMEO models (51 targets) with an AUC of 0.82.

Continuous Automated Model Evaluation (CAMEO) is a continuous blind prediction assessment for protein structures which are going to be published in the subsequent weekly release of PDB^34^. This platform releases targets every week. For validation of SAAP-QA, recently submitted models were collected from CAMEO. This set consisted of 61 targets. Four targets less than 50 residues in length while six are above 500. These 10 targets were not considered in the study, being too small to have a hydrophobic core or multi-domain in nature. The selected 51 targets with their residue length are shown in Supplementary Table S6. The values of SAAP_p_, helix fraction, sheet fraction, loop fraction, loop content and SAAP_p_/loop ratio were calculated for each model for all 51 targets. Further, SAAP-QA was used to predict the GDT using these six physico-chemical features. Top 1 model was selected for each target using the predicted GDT score. Individual GDT loss of the selected model with the best available model was calculated to evaluate the performance of SAAP-QA on CAMEO targets. Further, GDT loss for top 5 and top 10 were also computed. Table 3 shows the list of 51 targets with respective GDT loss on top 10, top 5 and top 1 model selected by SAAP-QA. The average GDT loss for top 1 model for 51 targets is 4.46 while for top 5 and top 10, the average GDT loss is 2.12 and 1.01, respectively. Here, target 5VH2 and 5VO3 shown high GDT loss due to the extended sheet component in their 3D structure. Similarly, correlation coefficient was also calculated for 51 CAMEO targets between predicted and true GDT. Overall correlation coefficient (PCC) on CAMEO dataset is 0.71, with individual PCC values for each target is shown in Supplementary Table S4. Predicted GDT compared to true GDT is shown in Fig. 4(a). AUC value for CAMEO data was 0.82 as shown in Fig. 4(b).

**Table 3:** GDT loss for top 10, top 5 and top 1 models selected by SAAP-QA on 51 CAMEO targets.

Name	Number of Models	Best GDT	Top10 GDT Loss	Top 5 GDT Loss	Top 1 GDT Loss
5MM8_A	31	91.42	0.61	7.23	7.23
5NVA_A	22	58.52	0.00	0.00	1.97
5O6C_A	32	18.54	0.00	0.00	0.00
5OJY_A	22	64.67	0.00	0.00	0.39
5OUN_A	35	62.85	0.47	0.70	0.70
5OVY_A	30	57.32	0.00	0.00	0.00
5TOS_B	31	55.13	0.00	0.51	4.24
5TXR_A	26	77.34	2.09	2.09	3.56
5U7Z_C	32	49.03	1.36	1.94	2.71
5U7Z_D	28	63.92	0.00	5.10	5.79
5U81_A	26	53.84	0.78	0.78	1.43
5U84_B	26	56.06	0.00	0.00	3.00
5UD7_F	21	64.50	0.00	0.00	0.00
5V8C_A	32	50.00	0.00	0.00	1.45
5VFX_H	32	56.54	0.00	0.00	9.11
5VG2_C	33	62.23	0.11	0.22	0.76
5VGU_F	36	82.20	0.40	0.40	0.40
5W35_B	31	65.77	0.46	1.85	8.62
5WEE_D	30	79.49	0.13	0.13	0.13
5WJD_A	31	70.60	2.83	6.76	6.92
5WLY_A	36	74.29	0.00	0.00	0.00
5 × 2B_L	30	77.74	0.00	3.53	8.57
5 × 7Y_D	15	86.02	0.28	2.26	2.26
5XB6_L	38	83.50	1.72	1.72	6.70
5XBV_A	30	72.48	0.67	0.67	0.67
5XCA_A	27	84.74	1.05	3.55	5.26
5XD6_B	20	74.67	1.15	3.69	7.05
5XDY_A	25	68.69	4.75	4.75	8.98
5XEO_B	36	91.94	0.00	0.00	0.48
5XEP_F	31	91.86	0.72	0.92	5.32
5XFL_D	31	78.73	0.00	0.00	4.07
5XJV_B	38	85.27	0.00	0.95	2.30
5XOM_B	31	75.70	0.00	1.65	1.65
5XPW_A	33	65.00	0.00	0.00	5.42
5XVS_B	37	81.73	3.17	3.23	3.23
5Y4B_A	30	69.09	5.38	5.38	5.65
5Y8E_A	20	65.75	0.00	1.30	3.25
5YH0_L	27	61.25	0.00	4.20	7.41
5Z11_B	36	59.96	1.76	1.76	4.69
5Z4G_B	36	71.43	1.40	5.28	5.59
5Z9Y_B	31	90.18	0.00	0.00	0.79
5ZB8_E	24	35.63	0.00	0.00	7.75
5ZHZ_A	20	80.81	0.00	0.58	3.00
5ZI9_D	31	76.44	2.11	2.11	3.65
6AU1_B	32	89.15	0.00	2.66	4.83
6CK0_B	20	68.07	0.00	0.11	0.34
6CKG_B	22	63.18	0.00	0.00	4.85
6CKP_A	20	83.20	5.47	5.47	5.86
5VH2_D	31	68.07	1.59	4.43	15.00
5Z68_D	35	73.53	6.10	7.91	16.42
5OV3_B	29	59.51	4.86	12.20	17.85
**Average GDT Loss**			**1.01**	**2.12**	**4.46**

#### CASP12 - Domain Structures

Single or multiple domains are predefined for each targets during CASP. These domains assist in detailed categorization of targets into (1) High Accuracy Modeling category - that will include domains where the majority of submitted models are of sufficient accuracy for detailed analysis and (2) Topology category (formerly Free Modeling) - that will assess domains where all submitted models are of relatively low accuracy. As SAAP-QA performs better on high quality models where high GDT score structures are available, we have considered specific domains of CASP targets instead of complete structures under the high accuracy modeling category for model development. Moreover, CASP itself gives weightage to these domains and reports domain-wise detailed quality analysis http://predictioncenter.org/casp12/results.cgi^20^. Implementation of a scoring function on the blind dataset of domain-wise models produces an overall correlation coefficient (PCC) of 0.76 between observed and predicted GDT (individual PCC for each target is shown in Supplementary Table S4). Based on CASP quality assessment (QA) results, the models generated by servers are classified into stage 1, which are closer to the experimental structure, based on their GDT values, and stage 2, which are the rest of the predicted structures. Stage 1 has 20 models for each target while stage 2 has 150 models selected by the Davis-QAconsensus method^47^. Stage 1 models for each target were ranked using SAAP-QA. The GDT loss of top ranked models for each target by SAAP-QA and the best available model in the pool are ‘0’ for stage 1 models. These results show that the SAAP-QA is able to capture the best model every time at first position in the decoy set for stage 1 models. As a next step, stage 2 models were also tested using SAAP-QA for ranking. Stage 2 is considered more important than stage 1 for structure prediction, and is used for QA method evaluation, as it is essential to eliminate model structures that are far from the experimental structure efficiently. For stage 2, 150 models were preselected by CASP organizers for quality assessment servers (QA). These 150 models for each of 18 target domains (listed in Table 4 as the blind test test) as ranked by different QA servers in the CASP12 competition, were evaluated using SAAP-QA. CASP allows the submission of the top 5 models in the 3D structure prediction category. Table 4 tabulates the GDT loss of the best model available in the pool with the top 5 and the top 10 captured by our scoring function. Average GDT loss for these targets in the top 5 models is 3.14 for stage 2 models, i.e. SAAP-QA selects the top 5 models that have 3.14 average GDT deviation from the best model available in the pool. Similarly, the top 10 models selected by SAAP-QA and their GDT loss were also shown in Table 4. The average GDT loss for the top 10 models is 1.72. Lastly, the top model (referred to as top 1) selected by SAAP-QA was also evaluated, with an average GDT loss of 6.41 (shown in the last column of Table 3). Data presented in Table 4 shows the ranking ability of SAAP-QA on an independent dataset. Individual PCC for each target in the blind test set between the predicted and actual GDT is shown in Supplementary Table S4 while GDT loss for the top 1 model is shown in Supplementary Table S3.

**Table 4:** GDT loss predicted bt SAAP-QA for the CASP12 blind test set of stage 2 models of 18 target domains.

Stage 2 (150 models) - Protein Domains			
Targets	GDT Loss for top 10 ranked	GDT Loss for top 5 ranked	GDT Loss for top 1 ranked
T0893-D2	0	5.77	7.55
T0895-D1	2.09	4.8	3.54
T0902-D1	0	0.1	3.35
T0906-D1	0.22	0.22	3.97
T0910-D1	1.89	2.44	2.60
T0911-D1	0.37	0.37	3.49
T0912-D1	2.17	2.17	8.31
T0913-D1	1.77	1.77	1.77
T0917-D1	2.5	5.12	9.36
T0920-D1	4.52	4.52	6.24
T0920-D2	4.99	8.33	7.06
T0921-D1	1.45	2.71	19.21
T0928-D1	0.37	0.44	13.01
T0943-D2	0	0.62	4.03
T0944-D1	2.96	6.52	10.47
T0946-D2	0	0	3.66
T0947-D1	3.72	8.72	6.00
T0948-D1	1.85	1.85	1.68
**Average GDT Loss**	**1.72**	**3.14**	**6.41**

GDT loss corresponding to the difference between the best model available in the pool of decoys and the model captured by SAAP-QA, are listed for top 10, top 5 and top 1 category models.

#### CASP12-Complete Structures

SAAP-QA is designed for structural domains but it can also be implemented for complete protein structures. In order to compare with CASP QA servers, we tested our prediction model on complete structures from “stage 2” models for 17 targets (T0920 is duplicated in domain study as it has two domains). Here, we calculated the GDT loss for top 10, top 5 and top 1 structures selected by SAAP-QA. For comparison with other scoring functions, the top three QA servers from CASP12 were selected. These servers, outperforming others during competition, are: (a) SMQA^48^ (b) ProQ3^49^ and (c) MESHI_CON_SERVER^50^. The average GDT loss for these three servers on the selected 17 targets (with full structure) were calculated afresh. SVMQA has 3.8 average GDT loss while ProQ3 and MESHI_CON_SERVER have 3.69 and 5.14 GDT loss, respectively for the best predicted model from stage 2. Comparatively, SAAP-QA showed 6.08 average GDT loss for full protein structures on these 17 CASP12 targets, with the individual GDT loss for each target shown in Table 5. Moreover, SAAP-QA captured models among top 5 and top 10 categories with average GDT loss of 3.12 and 2.28, respectively. Detailed scores are shown in Table 5. SAAP-QA showed comprarable performance with the state-of-art QA servers, as well as efficiently capture native/near-native models in the top5/top10 bin. Thus, it can be integrated with protein structure prediction programs to screen high quality models. It should also be noted that although SAAP-QA was modeled on domains, it can be applied to full length protein structures.

**Table 5:** GDT loss predicted by SAAP-QA for the CASP12 blind test set of stage 2 models of 17 target full length strutures.

Stage 2 (150 Models) - Full Length Protein				
Targets	Best GDT	Top10 GDT Loss	Top 5 GDT Loss	Top 1 GDT Loss
T0893	61.98	0.52	0.52	4.86
T0895	72.92	0.00	0.00	0.00
T0902	50.75	2.42	2.92	3.17
T0906	91.90	1.05	1.96	4.05
T0910	87.91	3.28	3.81	5.15
T0911	65.99	0.74	3.98	6.62
T0912	47.79	5.47	5.47	6.05
T0913	66.57	0.37	0.37	5.55
T0917	84.18	4.90	4.90	4.90
T0920	50.42	4.45	4.45	8.29
T0921	70.65	0.18	0.18	0.18
T0928	63.27	0.59	0.59	11.44
T0943	60.61	8.55	8.94	14.10
T0944	74.11	1.19	3.36	12.85
T0946	45.21	0.00	0.09	2.41
T0947	66.43	2.00	8.57	8.57
T0948	76.68	3.02	3.02	5.21
**Average GDT Loss**		**2.28**	**3.12**	**6.08**

GDT loss corresponding to the difference between the best model available in the pool of decoys and the model captured by SAAP-QA, are listed for top 10, top 5 and top 1 category models. These full protein targets correspond to the 18 target domains listed in Table 3.

## Conclusions

The primary sequence of a protein has been considered to encode its native (or 3D) structure but it also encodes residue-level information about aggregation. Under standard physiological conditions, protein selects its native folding pathway and avoids aggregation. To the best of our knowledge, protein aggregation has focussed on understanding disease propensity including amyloid and fibril formation and has not been applied extensively to protein structure prediction to address the protein folding problem. Folding and the spatial conformation of aggregation-prone patches can help in solving the protein folding problem. Here, we have examined solvent accessibility of aggregation patches (SAAP_p_) on native and decoy structures. Native structures showed smaller SAAP_p_ values, suggesting the close packing of these aggregation-prone residues in the core of their respective structures. However, non-native structures showed higher SAAP_p_ values, indicating the exposure of a larger proportion of aggregation-prone residues to the solvent compared to native structures. CASP12 models under the TBM category were examined to uncover the relevance of SAAP_p_ scores for predicted protein structures. The results showed a high overall correlation of 0.76 between SAAP_p_ and GDT scores on 30 target domain structures of CASP12. Furthermore, SAAP_p_ along with 5 other structural descriptors were trained using random forest machine learning approach to build the SAAP_p_ scoring function, SAAP-QA. So as to add diversity in the data set, 21 CASP11 targets domain structures were also added to the 13 CASP12 targets during scoring function formulation. Train and test set was divided based on protein targets to avoid any similarity between train and test set protein models. SAAP-QA showed correlation co-efficient of 0.96 and 0.86 on train and test sets respectively while the average area under the ROC curve (AUC) values for distinguishing true positives from false positives was 0.98 and 0.94, respectively. Generalized effect of prediction model was tested using 3-fold validation. Average PCC in 3-fold validation was 0.83 while AUC was 0.93. Small standard deviation in PCC and AUC during 3-fold validation showed robustness of prediction model on independent dataset. Further, CAMEO was used as an external validation dataset for blind testing the prediction model, 51 targets from CAMEO platform were tested for this purpose. The average GDT loss for top 10, top 5 and top 1 models were 1.01, 2.12 and 4.46, respectively, for CAMEO targets. In addition to the 51 CAMEO targets, 18 CASP12 targets (both domains and full structures) were also added to the blind test set that were not part of model training/testing. The result showed 0.76 PCC between predicted and actual GDT. Ranking ability was further tested using GDT loss between models captured by SAAP-QA and the best model available. Average GDT loss on stage 2 (150 models) domains structures was computed as 6.08, 3.12 and 2.28 for top 1, top 5 and top 10 ranked models respectively. This combined showed its performance on an external unseen sample set. SAAP_p_ is thus a computational measure of the degree of protein folding, which naturally tends to minimize the solvent accessible area for aggregation-prone residues. SAAP_p_ has shown noteworthy performance in classifying good and bad models and can serve as an independent metric for separating near-native prediction model structures from poorly predicted model structures and for incorporation in protein structure prediction algorithms, to eliminate decoy structures and iteratively improve near-native models.

## Methods

The first CASP12 target “T0859” from Acinetobacter phage AP205 (listed in the category Human and Server) was selected as a case study. High quality PDB structures were then selected, applying the criteria of: (1) number of chains = 1, (2) 100 ≤ sequence length ≤500; (3) resolution ≤ 2 Å; (4) type of macromolecule = only protein; (5) no ligands were present and (6) sequence identity ≤30%, resulting in 1557 structures. Test structures were used from CASP 12 automatic evaluation results for the template-based model (TBM) category predicted by different participating servers, comprising 37 domains from different targets where 5 domains are small (<100 amino acids in length) and therefore have no hydrophobic core while another 2 proteins have very small aggregation-prone patches (<20%) of their complete sequence. Thus 30 shortlisted targets are: T0860-D1, T0861-D1, T0867-D1, T0871-D1, T0873-D1, T0877-D1, T0879-D1, T0881-D1, T0883-D1, T0885-D1, T0889-D1, T0891-D1, T0893-D2, T0895-D1, T0902-D1, T0906-D1, T0910-D1, T0911-D1, T0912-D1, T0913-D1, T0917-D1, T0920-D1, T0920-D2, T0921-D1, T0928-D1, T0943-D2, T0944-D1, T0946-D2, T0947-D1 and T0948-D1. Here ‘D1/D2’ represents the domain name assigned by CASP organizer. Solvent accessible surface area (SASA) was calculated using the naccess program based on Lee and Richards’ algorithm^51^. The solvent accessibility of aggregation patches (SAAP) score for each protein p (SAAP_p_), was calculated from residue scores i, using side chain solvent accessible surface area (SC_sasai_) values and predicted aggregation patches (PREDAggregation_i_), as shown in Equation 1. SAAP_p_ scores were then computed as the percentage solvent accessible aggregation-prone residues, as shown in Equation. 1.

1 $${{\rm{SAAP}}}_{{\rm{p}}}=\frac{{\sum }_{i=1}^{n}{{\rm{SAAP}}}_{{\rm{i}}}}{{{\rm{AggreProne}}}_{{\rm{T}}}}\times 100$$

SAAP_p_ = SAAP score for protein ‘p’; SAAP_i_ = SAAP score for residue ‘i’ AggreProne_T_ = Total number of aggregation-prone residue

2 $$\begin{array}{rcl}{{\rm{SAAP}}}_{{\rm{i}}} & = & \{\begin{array}{llllll}\mathrm{1,} & {\rm{if}}\,{{\rm{SC}}}_{{\rm{sasai}}} &  >  & {\rm{50}}\,{\rm{and}}\,{{\rm{PREDAggregation}}}_{{\rm{i}}} & = & {\rm{TRUE}}\\ \mathrm{0,} & {\rm{if}}\,{{\rm{SC}}}_{{\rm{sasai}}} &  <  & {\rm{50}}\,{\rm{and}}\,{{\rm{PREDAggregation}}}_{{\rm{i}}} & = & {\rm{TRUE}}\\ \mathrm{0,} & {\rm{if}}\,{{\rm{SC}}}_{{\rm{sasai}}} &  >  & {\rm{50}}\,{\rm{and}}\,{{\rm{PREDAggregation}}}_{{\rm{i}}} & = & {\rm{FALSE}}\end{array}\end{array}$$

SC_sasai_ is toal surface area for side chains of amino acids exposed to water solvent. Aggregation-prone regions were predicted consistently using the Aggrescan server^35^ for protein sequences and the Aggrescan3D (A3D)^19^ server for 3D structures. Multiple protein sequences were submitted to the the aggregation server available at http://bioinf.uab.es/aggrescan/http://bioinf.uab.es/aggrescan/ to calculate individual aagregation score of amino acids, Supplementary Figure S4 shows that Aggrescan server can handle multiple sequences and produce the result in very short time. However, Aggregation3D (A3D) server available at http://biocomp.chem.uw.edu.pl/A3D/http://biocomp.chem.uw.edu.pl/A3D/ used only once for a case study (CASP Target T0859), here 3D structure of protein was submitted to server that gives A3D scores for individual residues.

Random forest, a decision based machine learning approach was used to build SAAP_p_ scoring function. This scoring function was designed to predict GDT of a given protein model using SAAP_p_ and other descriptors. Multiple descriptors were used to build scoring function, SAAP_p_ served as major descriptors. Additional descriptors along with SAAP_p_ were added to build robust scoring function in order to capture diversity among protein’s structures. Following descriptors were used in machine learning method to build SAAP_p_ scoring function: (A) SAAP_p_ Score - calculation described above. (B) Helix fraction-ratio of total number of residues involved in helix formation to the length of protein. (C) Sheet fraction - ratio of total number of residues involved in sheet formation to the length of protein. (D) Loop fraction - ratio of total number of residues involved in loop formation to the length of protein. (E) Loop content-total number of residues involved in loop formation. (F) SAAP_p_/Loop -ratio of SAAP score with total number of residues involved in loop formation. Secondary structure of protein was assigned using STRIDE program, it implements a knowledge-based algorithm that makes combined use of hydrogen bond energy and statistically derived backbone torsional angle information^52^.

Model training was done using the ‘R’ package^53^ where 700 trees were grown in the forest using 2 features at every split. All protein Models from CASP11 and CASP12 were mixed, resulting in a total of 10978 models and 53 unique targets. These models were then randomly split into train (38 targets, 7907 models) and test (15 targets, 3071 models) sets based on targets following 70/30 rule. Although, data division is based on non-overlapping targets i.e. train set has 70% while test set has 30% unique targets, but the number of protein models in train and test set are also in 70% and 30% proportion. Later, these 53 targets divided into 3 non overlapping sets for conducting 3-fold validation. These sets have 18, 18 and 17 targets comprising 3812, 3869 and 3297 protein models respectively. In each run of 3-fold validation, any two sets were used for training while the third one was used as test set. All these data points belong to the CASP11 and CASP12 TBM category models and the complete list of the names of the targets used for training and testing is provided in Supplementary Table S1. In addition to training and testing, a blind validation was also performed to evaluate the performance of prediction algorithm on an unknown set. The blind validation dataset is comprised of 4305 models belong to 18 targets from CASP12 that were not used in building the prediction algorithm. These 18 cases were preselected from the total list of 30 and they are the last 18 TBM category targets. Furthermore, SAAP_p_ scoring function, SAAP-QA, was also validated on CAMEO targets for blind prediction testing that has high quality models thn CASP experiment. Here, April 2018 targets were selected from the CAMEO website https://www.cameo3d.org/ https://www.cameo3d.org/. This constituted 51 targets after ignoring those with residue length less than 50 and more than 500. The GDT score was given for each model in its downloaded score file while the fasta sequence is provided separately with every target.

## Electronic supplementary material

Supplementary Information
