# Supplementary material for: Role of solvent accessibility for aggregation-prone patches in protein folding
Source: Sci Rep. 2018 Aug 27;8:12896. doi: 10.1038/s41598-018-31289-6 (PMC6110721; doi:10.1038/s41598-018-31289-6)
Supplement: Supplementary file 1 — Supplementary Information [file 41598_2018_31289_MOESM1_ESM.pdf]

Role of solvent accessibility for aggregation-prone patches in protein folding

Avinash Mishra, Shoba Ranganathan, B. Jayaram, and Abdul Sattar

<u>SUPPLEMENTARY INFORMATION</u>

Supplementary Figure S1 SAAP score is plotted against GDT for 31 TBM category domains of CASP12, Y axis: GDT score, X axis: SAAP score. Red marked data points represent top 10 models predicted based on GDT. SAAP has exponential relation with GDT score that implies lowest SAAP score correspond to highest GDT score.

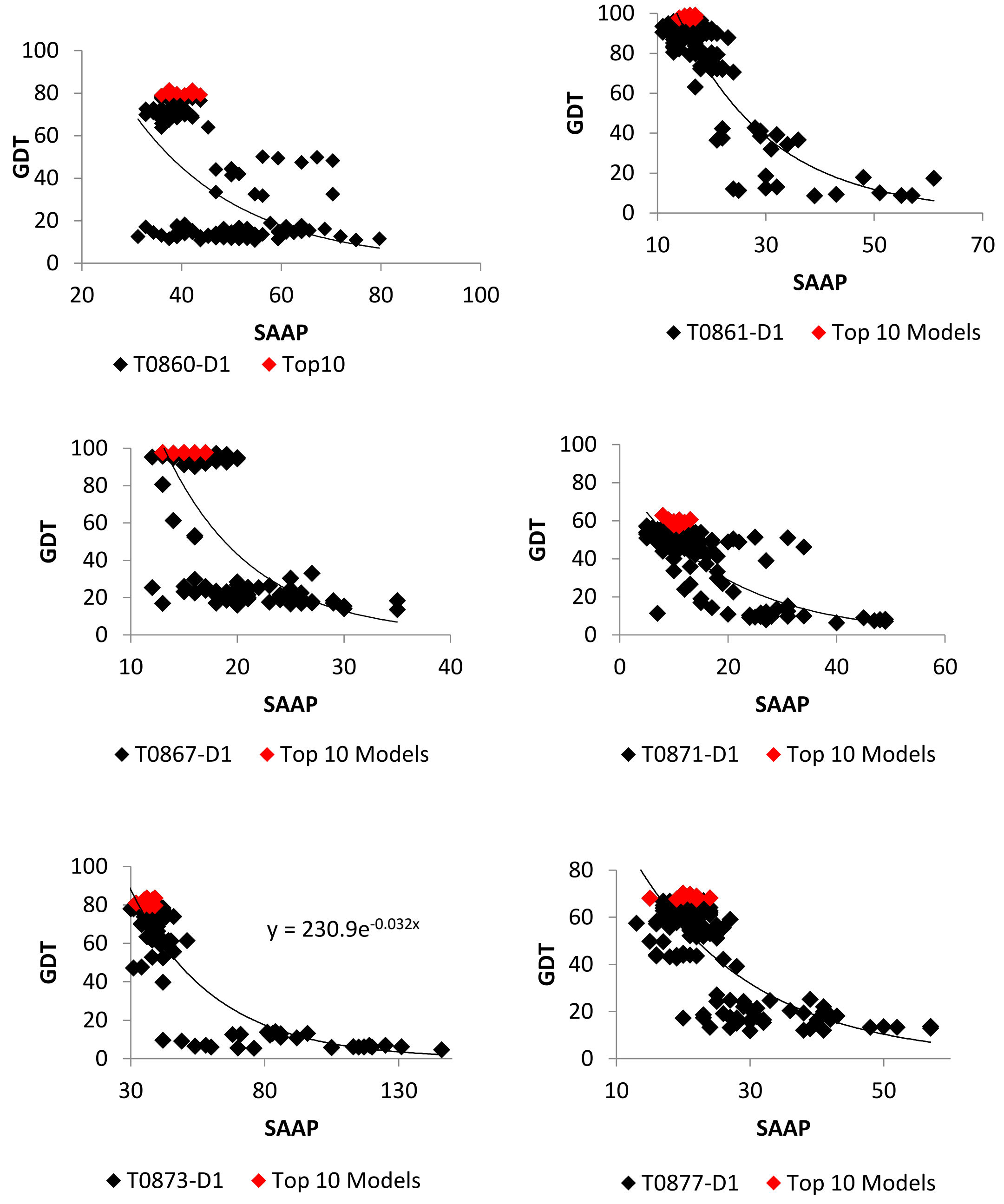

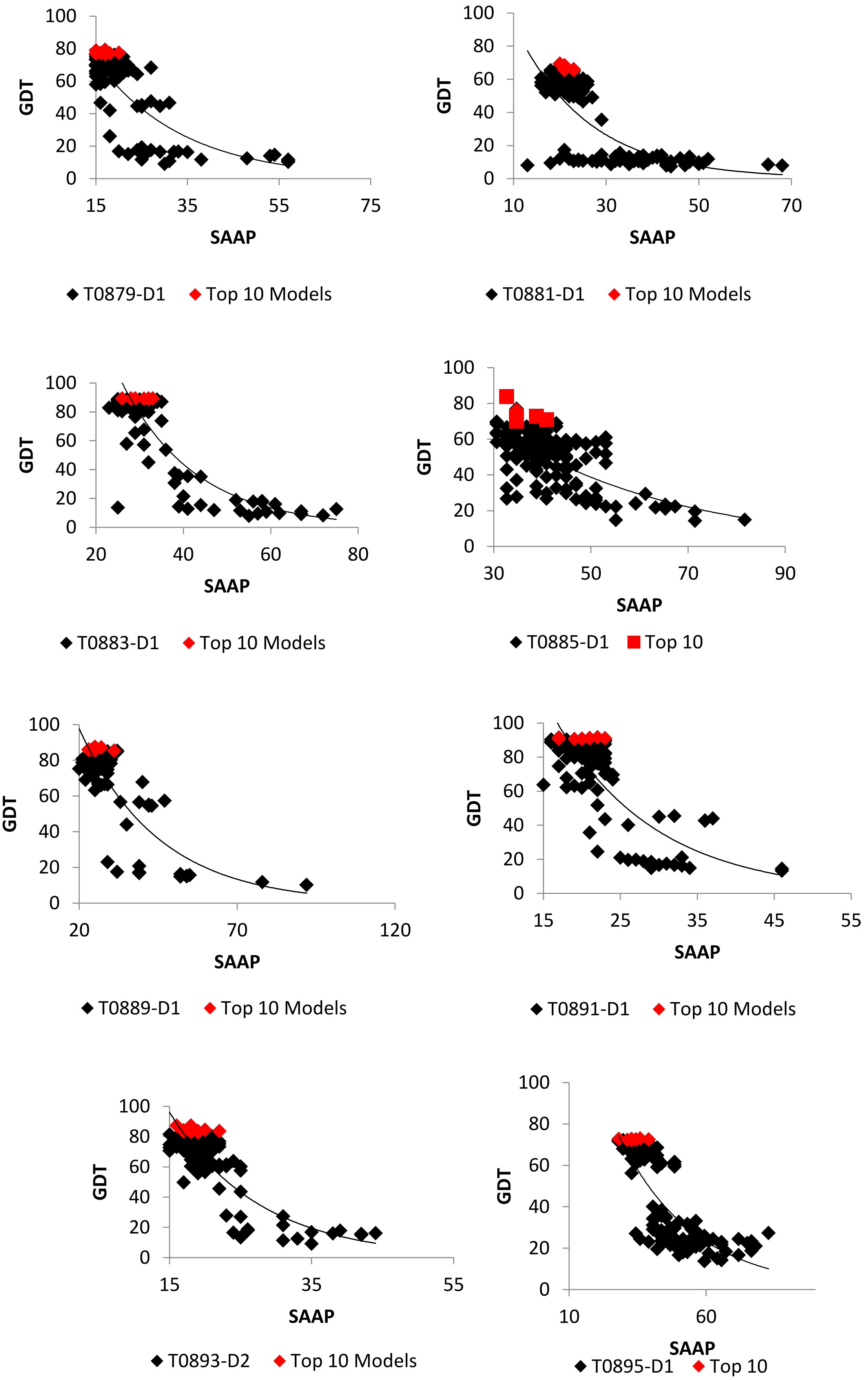
GDT
SAAP
T0879-D1
Top 10 Models
GDT
SAAP
T0881-D1
Top 10 Models
GDT
SAAP
T0883-D1
Top 10 Models
GDT
SAAP
T0885-D1
Top 10
GDT
SAAP
T0889-D1
Top 10 Models
GDT
SAAP
T0891-D1
Top 10 Models
GDT
SAAP
T0893-D2
Top 10 Models
GDT
SAAP
T0895-D1
Top 10

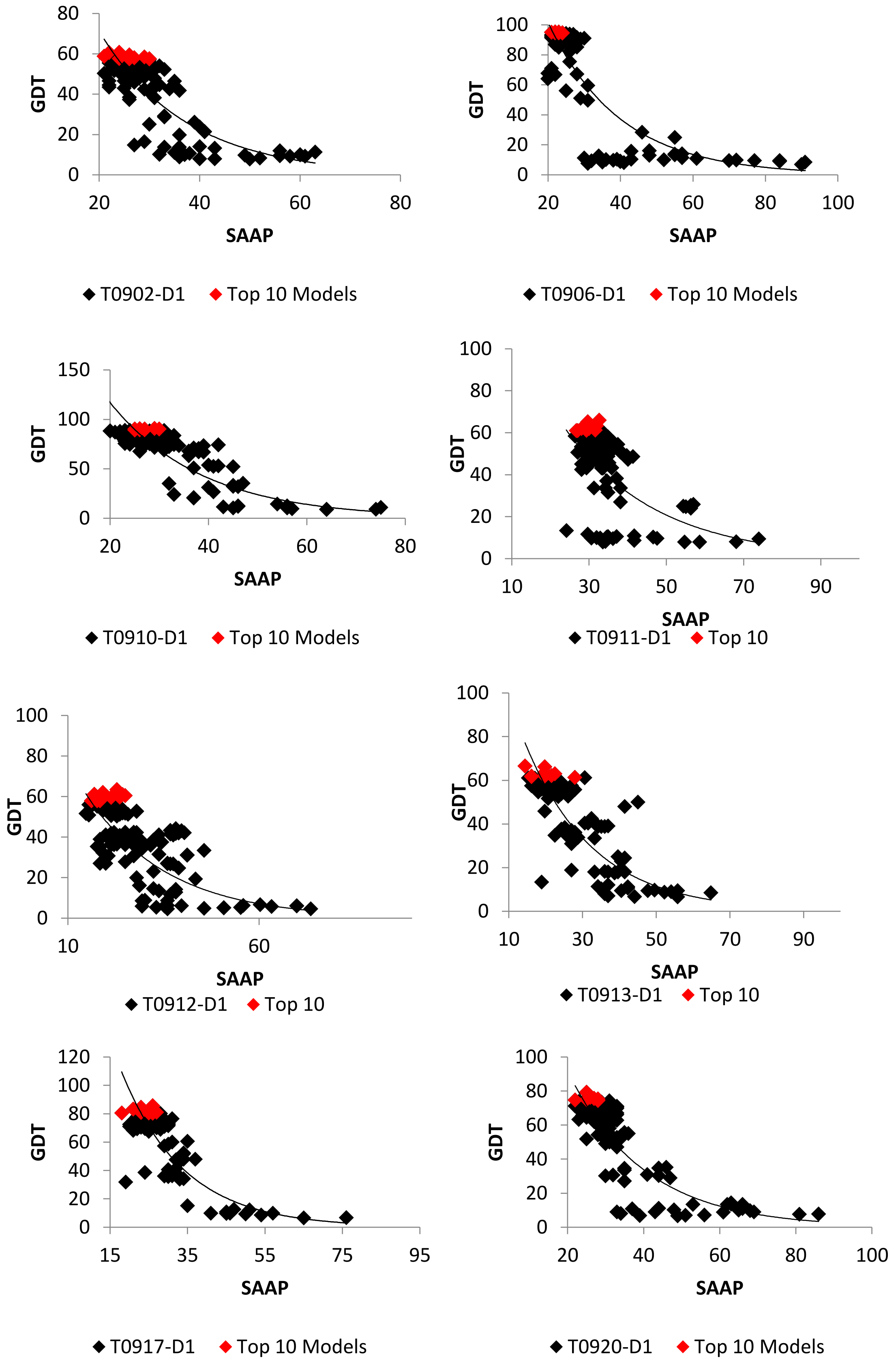
GDT
SAAP
T0902-D1
Top 10 Models
GDT
SAAP
T0906-D1
Top 10 Models
GDT
SAAP
T0910-D1
Top 10 Models
GDT
SAAP
T0911-D1
Top 10
GDT
SAAP
T0912-D1
Top 10
GDT
SAAP
T0913-D1
Top 10
GDT
SAAP
T0917-D1
Top 10 Models
GDT
SAAP
T0920-D1
Top 10 Models

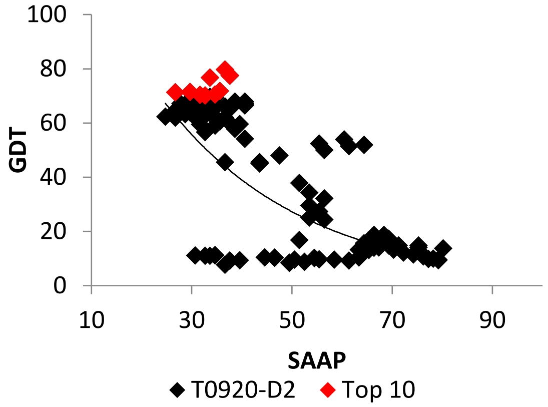
GDT
SAAP
T0920-D2
Top 10

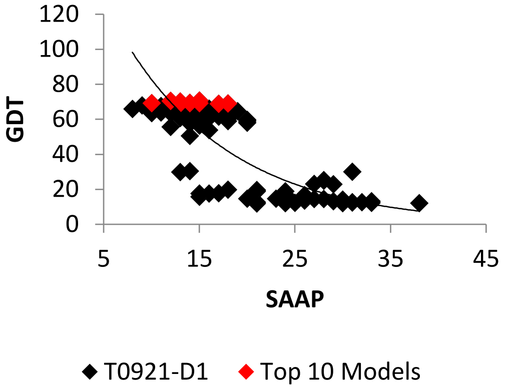
GDT
SAAP
T0921-D1
Top 10 Models

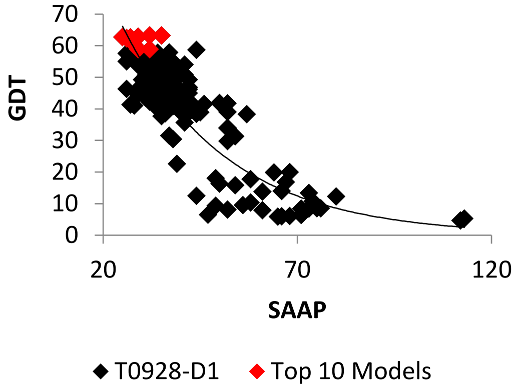
GDT
SAAP
T0928-D1
Top 10 Models

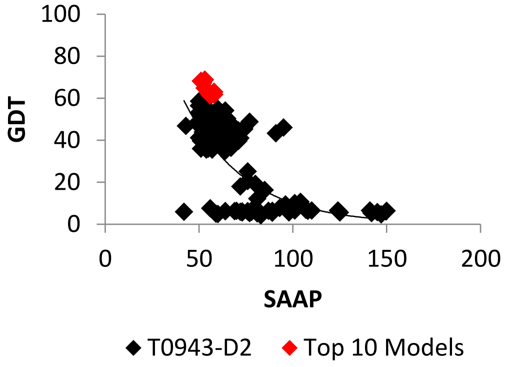
GDT
SAAP
T0943-D2
Top 10 Models

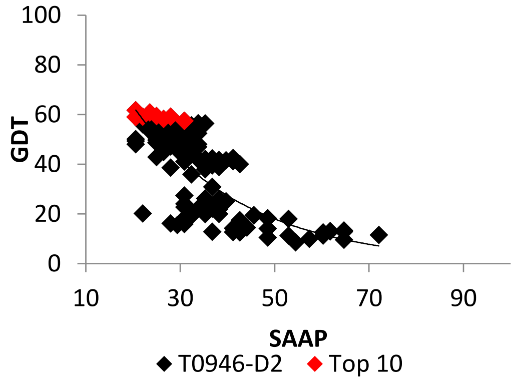
GDT
SAAP
T0946-D2
Top 10

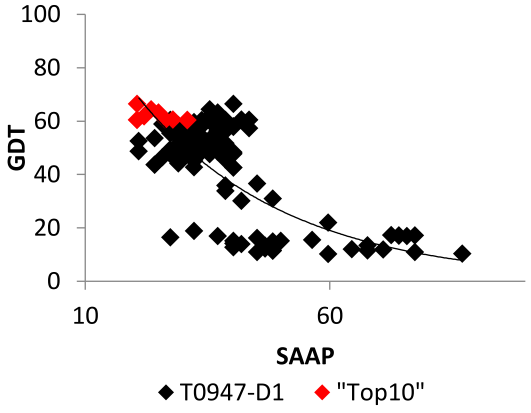
GDT
SAAP
T0947-D1
"Top10"

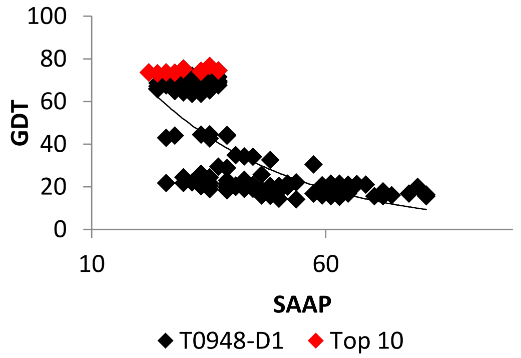
GDT
SAAP
T0948-D1
Top 10

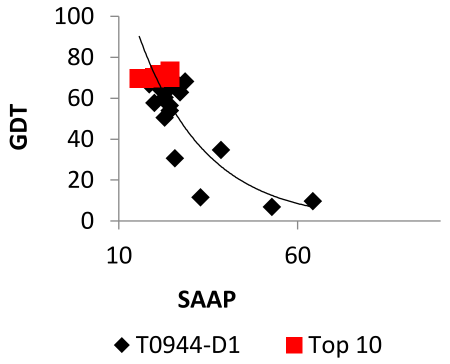
GDT
SAAP
T0944-D1
Top 10

Supplementary Figure S2. Data analysis of 6 features selected for building SAAP scoring function.

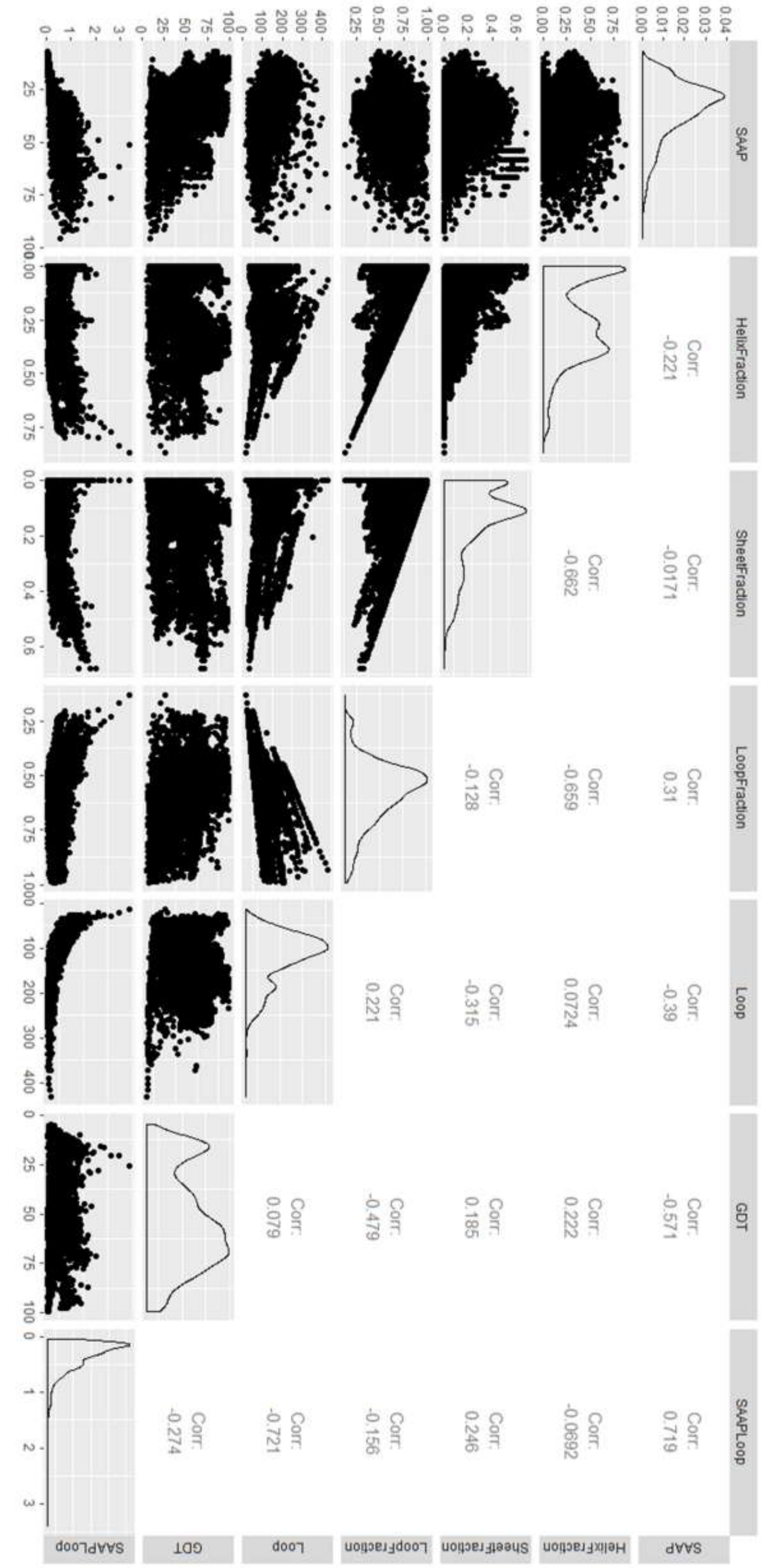

Supplementary Figure S3. Interdependency of descriptors used in random forest method for SAAP based GDT prediction. Numbers shown in the bar represent Pearson correlation coefficient value.

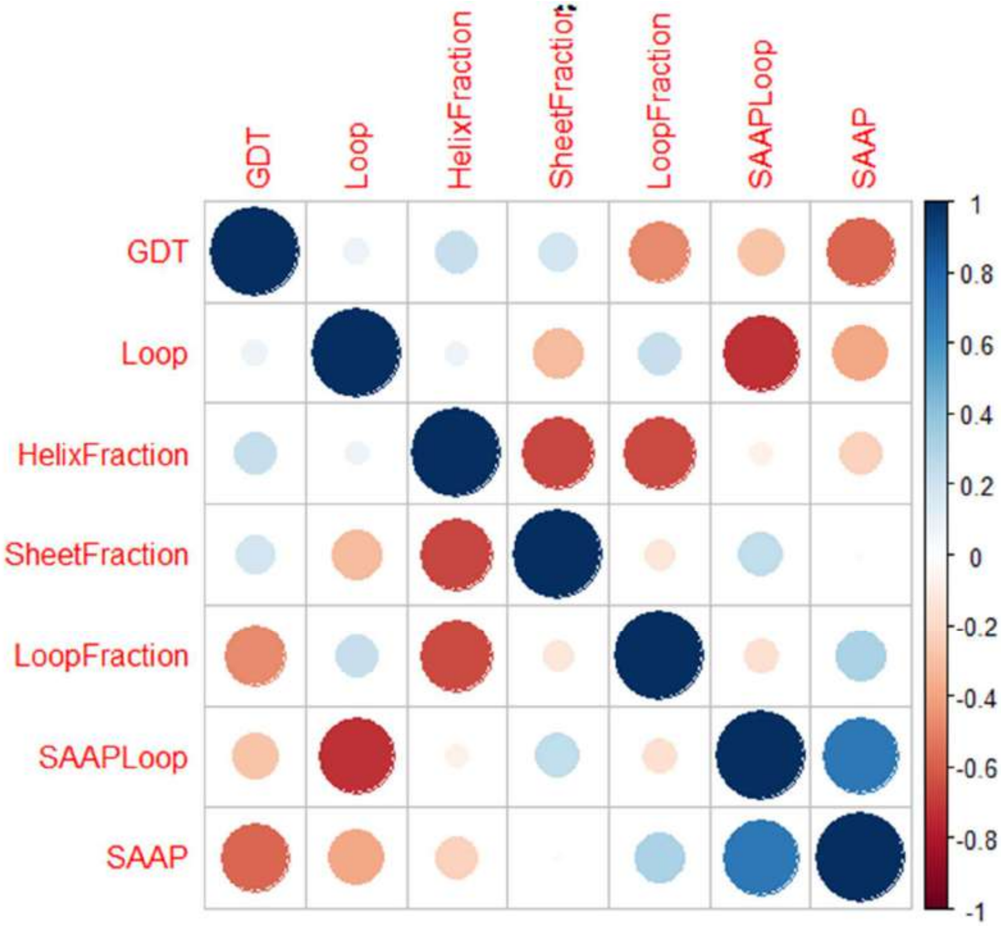

Supplementary Figure S4. Use of Aggrescan server for multiple protein sequences and the corresponding results of aggregation prone residues.

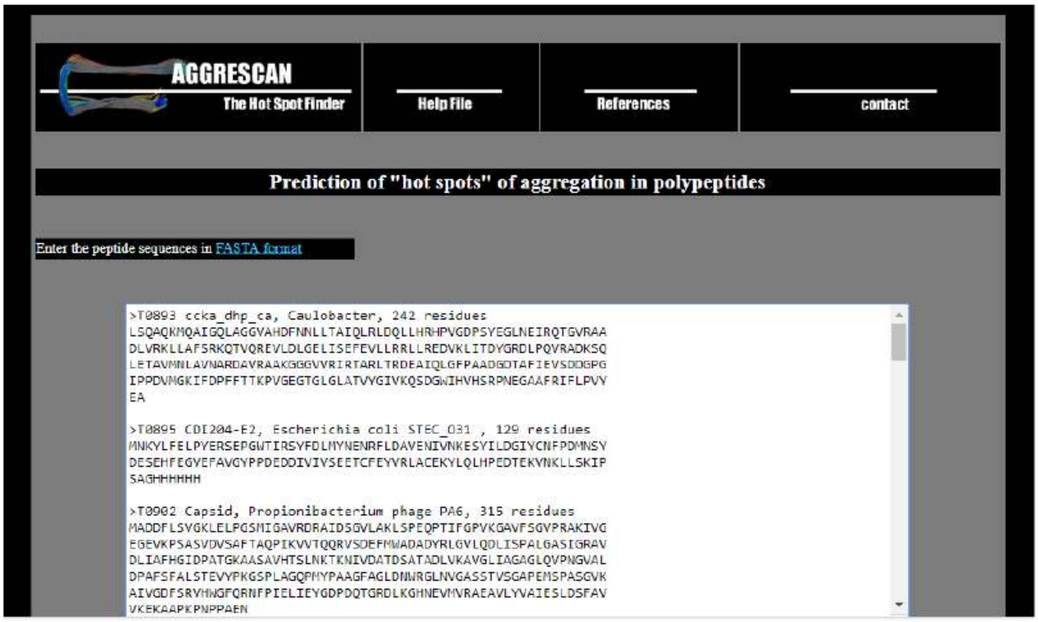

RESULTS

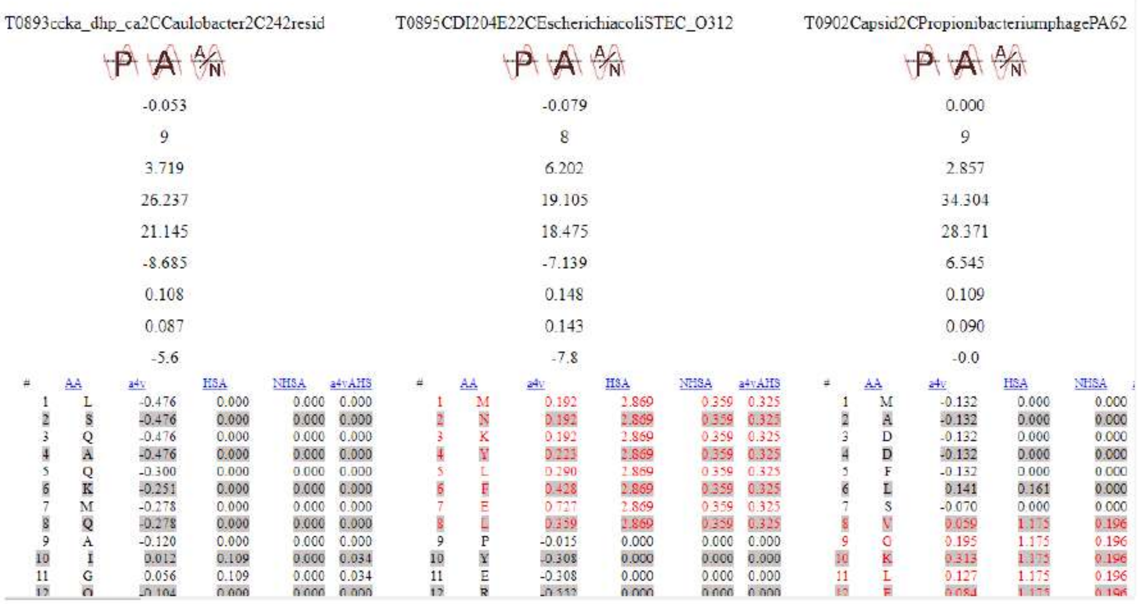

T0893ccka_dhp_ca2CCaulobacter2C242resid	T0895CDI204E22CEscherichiacoliSTEC_O312	T0902Capsid2CPropionibacteriumphagePA62
-0.053	-0.079	0.000
9	8	9
3.719	6.202	2.857
26.237	19.105	34.304
21.145	18.475	28.371
-8.685	-7.139	6.545
0.108	0.148	0.109
0.087	0.143	0.090
-5.6	-7.8	-0.0

#	AA	a4v	HSA	NHSA	a4vAHS
1	L	-0.476	0.000	0.000	0.000
2	S	-0.476	0.000	0.000	0.000
3	Q	-0.476	0.000	0.000	0.000
4	A	-0.476	0.000	0.000	0.000
5	Q	-0.300	0.000	0.000	0.000
6	K	-0.251	0.000	0.000	0.000
7	M	-0.278	0.000	0.000	0.000
8	Q	-0.278	0.000	0.000	0.000
9	A	-0.120	0.000	0.000	0.000
10	I	0.012	0.109	0.000	0.034
11	G	0.056	0.109	0.000	0.034
12	Q	-0.104	0.000	0.000	0.000

#	AA	a4v	HSA	NHSA	a4vAHS
1	M	0.192	2.869	0.359	0.325
2	N	0.192	2.869	0.359	0.325
3	K	0.192	2.869	0.359	0.325
4	Y	0.223	2.869	0.359	0.325
5	L	0.290	2.869	0.359	0.325
6	F	0.428	2.869	0.359	0.325
7	E	0.727	2.869	0.359	0.325
8	L	0.359	2.869	0.359	0.325
9	P	-0.015	0.000	0.000	0.000
10	Y	-0.308	0.000	0.000	0.000
11	E	-0.308	0.000	0.000	0.000
12	R	[illegible]	0.000	0.000	0.000

#	AA	a4v	HSA	NHSA
1	M	-0.132	0.000	0.000
2	A	-0.132	0.000	0.000
3	D	-0.132	0.000	0.000
4	D	-0.132	0.000	0.000
5	F	-0.132	0.000	0.000
6	L	0.141	0.161	0.000
7	S	-0.070	0.000	0.000
8	V	0.059	1.175	0.196
9	Q	0.195	1.175	0.196
10	K	0.313	1.175	0.196
11	L	0.127	1.175	0.196
12	F	0.084	1.175	0.196

Supplementary Table S1 List of targets from CASP11 and CASP12 used as train and test set for building SAAP based scoring function.

CASP11 Targets	
T0760-D1	T0811-D1
T0762-D1	T0817-D2
T0764-D1	T0819-D1
T0766-D1	T0821-D1
T0768-D1	T0822-D1
T0770-D1	T0823-D1
T0772-D1	T0826-D2
T0776-D1	T0827-D1
T0782-D1	T0830-D2
T0783-D2	T0833-D1
T0786-D1	T0835-D1
T0794-D1	T0838-D1
T0795-D1	T0839-D1
T0796-D1	T0845-D2
T0799D4	T0847-D1
T0801-D1	T0848-D1
T0803-D1	T0849-D1
T0805-D1	T0851-D1
T0807-D1	T0852-D1
T0808-D1	T0852-D2
T0810-D2	T0858-D1
**CASP12 Targets**	
T0860-D1	T0879-D1
T0861-D1	T0881-D1
T0867-D1	T0883-D1
T0871-D1	T0885-D1
T0873-D1	T0889-D1
T0877-D1	T0891-D1
T0893-D1	

Supplementary Table S2. Complete model list of CASP11 and CASP 12 used in building SAAP scoring function, 6 Features used for training is listed for each models with their corresponding GDT. 3-Cross Validation datset is given at the bottom

TARGETS: T0852-D1,T0852-D2,T0873-D1,T0826-D2,T0776-D1,T0881-D1,T0796-D1,T0871-D1,T0801-D1,T0808-D1,T0879-D1,T0838-D1,T0823-D1,T0783-D2,T0891-D1,T0799D4,T0805-D1,T0847-D1,T0821-D1,T0849-D1,T0848-D1,T0822-D1,T0861-D1,T0830-D2,T0807-D1,T0827-D1,T0858-D1,T0795-D1,T0760-D1,T0766-D1,T0867-D1,T0764-D1,T0817-D2,T0877-D1,T0851-D1,T0883-D1,T0762-D1,T0772-D1,T0839-D1

TRAIN SET MODELS							
**Name**	**SAAP**	**HelixFraction**	**SheetFraction**	**LoopFraction**	**Loop**	**SAAP/Loop**	**GDT**
T0852TS008_3-D1.rsa	79.412	0.651	0	0.349	44	1.805	29.56
T0852TS216_1-D2.rsa	78.947	0.495	0	0.505	56	1.41	17.12
T0852TS499_5-D1.rsa	71.429	0.061	0.061	0.878	115	0.621	10.69
T0852TS448_5-D1.rsa	71.154	0.212	0.024	0.764	126	0.565	16.3
T0852TS349_1-D2.rsa	71.154	0.606	0	0.394	65	1.095	14.08
T0852TS237_5-D1.rsa	66.667	0	0.602	0.398	43	1.55	70.83
T0852TS414_5-D1.rsa	66.667	0	0.583	0.417	45	1.481	56.25
T0852TS156_5-D1.rsa	65.789	0.288	0	0.712	79	0.833	27.48
T0852TS492_3-D2.rsa	65.789	0.171	0.054	0.775	86	0.765	23.65
T0852TS117_3-D1.rsa	65.789	0.378	0	0.622	69	0.953	15.54
T0852TS448_4-D1.rsa	65.385	0.345	0	0.655	108	0.605	13.77
T0852TS277_2-D1.rsa	64.286	0.413	0.079	0.508	64	1.004	15.08
T0852TS349_2-D1.rsa	63.158	0.27	0	0.73	81	0.78	16.22
T0852TS335_1-D1.rsa	63.158	0.252	0	0.748	83	0.761	16.22
T0852TS452_2-D1.rsa	63.158	0.167	0	0.833	90	0.702	16.67
T0852TS145_1-D1.rsa	61.765	0.69	0	0.31	39	1.584	37.5
T0852TS216_2-D2.rsa	61.765	0.571	0	0.429	54	1.144	33.93
T0852TS492_2-D2.rsa	60.526	0.144	0.036	0.82	91	0.665	20.27
T0852TS300_3-D1.rsa	60.465	0.254	0.045	0.701	94	0.643	41.79
T0852TS228_3-D1.rsa	60.345	0.594	0.066	0.34	87	0.694	12.79
T0852TS410_1-D2.rsa	60.227	0.091	0.301	0.608	180	0.335	23.9
T0852TS022_2-D1.rsa	59.615	0.285	0.152	0.564	93	0.641	17.25
T0852TS022_5-D1.rsa	58.333	0	0.556	0.444	48	1.215	54.63
T0852TS335_1-D2.rsa	57.895	0.207	0.072	0.721	80	0.724	25.68
T0852TS237_2-D1.rsa	57.895	0.243	0	0.757	84	0.689	18.47
T0852TS410_3-D1.rsa	57.143	0.135	0	0.865	109	0.524	14.88
T0852TS041_3-D1.rsa	57.143	0.27	0.175	0.556	70	0.816	19.44
T0852TS156_3-D1.rsa	56.338	0.452	0	0.548	92	0.612	22.76
T0852TS277_4-D1.rsa	56	0.152	0.152	0.696	96	0.583	14.49
T0852TS263_2-D2.rsa	55.263	0.243	0.054	0.703	78	0.709	19.82
T0852TS210_1-D1.rsa	55	0	0.421	0.579	66	0.833	21.27
T0852TS008_1-D2.rsa	54.167	0	0.382	0.618	68	0.797	45.23
T0852TS210_1-D2.rsa	54.167	0	0.602	0.398	43	1.26	66.2
T0852TS420_5-D1.rsa	54.167	0.056	0.648	0.296	32	1.693	70.6

T0852TS268_2-D2.rsa	54.167	0.019	0.62	0.361	39	1.389	68.52
T0852TS022_3-D1.rsa	53.571	0.031	0.198	0.771	101	0.53	13.74
T0852TS156_4-D1.rsa	53.571	0	0.206	0.794	104	0.515	15.84
T0852TS011_1-D1.rsa	52.632	0.189	0	0.811	90	0.585	20.27
T0852TS410_5-D1.rsa	52.632	0.162	0.081	0.757	84	0.627	23.2
T0852TS381_1-D2.rsa	52.632	0.234	0.072	0.694	77	0.684	14.87
T0852TS268_5-D1.rsa	52.632	0.234	0.171	0.595	66	0.797	21.17
T0852TS452_1-D1.rsa	51.786	0.357	0	0.643	81	0.639	25.4
T0852TS349_3-D2.rsa	51.563	0.059	0.287	0.654	89	0.579	13.05
T0852TS452_1-D2.rsa	50.704	0.5	0.048	0.452	76	0.667	20.99
T0852TS454_4-D1.rsa	50	0.045	0.018	0.937	104	0.481	22.97
T0852TS492_4-D1.rsa	50	0.015	0.206	0.779	102	0.49	14.7
T0852TS160_5-D1.rsa	50	0.135	0.018	0.847	94	0.532	19.82
T0852TS335_2-D2.rsa	50	0.189	0	0.811	90	0.556	28.38
T0852TS117_4-D1.rsa	50	0.153	0.162	0.685	76	0.658	32.21
T0852TS008_2-D1.rsa	50	0	0.583	0.417	45	1.111	62.5
T0852TS346_1-D1.rsa	50	0.046	0.221	0.733	96	0.521	12.02
T0852TS345_5-D1.rsa	50	0.056	0.352	0.593	64	0.781	26.62
T0852TS216_1-D1.rsa	50	0.659	0	0.341	43	1.163	37.1
T0852TS145_2-D2.rsa	46.875	0.809	0	0.191	26	1.803	12.32
T0852TS184_1-D2.rsa	46.512	0.321	0.194	0.485	65	0.716	24.63
T0852TS268_3-D2.rsa	46.479	0.375	0.048	0.577	97	0.479	21.95
T0852TS216_4-D1.rsa	46.429	0	0.366	0.634	83	0.559	51.15
T0852TS268_1-D2.rsa	46.429	0	0.16	0.84	110	0.422	20.23
T0852TS381_4-D1.rsa	46.429	0	0.336	0.664	87	0.534	15.84
T0852TS041_2-D1.rsa	46.429	0	0.374	0.626	82	0.566	17.56
T0852TS454_2-D1.rsa	46.429	0	0.374	0.626	82	0.566	14.12
T0852TS145_4-D2.rsa	46.429	0	0.321	0.679	89	0.522	14.31
T0852TS448_1-D2.rsa	46	0.127	0	0.873	219	0.21	8.66
T0852TS492_3-D1.rsa	45.833	0	0.491	0.509	55	0.833	62.5
T0852TS499_2-D2.rsa	45.833	0.018	0.391	0.591	65	0.705	20.23
T0852TS479_3-D1.rsa	45.313	0.081	0	0.919	125	0.363	12.32
T0852TS038_2-D1.rsa	45.313	0.059	0.324	0.618	84	0.539	13.05
T0852TS237_3-D1.rsa	45.07	0.405	0	0.595	100	0.451	17.79
T0852TS345_2-D1.rsa	45.07	0.518	0.113	0.369	62	0.727	34.45
T0852TS492_4-D2.rsa	44.444	0.413	0.094	0.493	105	0.423	75.13
T0852TS216_3-D2.rsa	44.231	0.382	0.085	0.533	88	0.503	15.35
T0852TS452_3-D1.rsa	44.186	0.284	0.03	0.687	92	0.48	22.39
T0852TS008_5-D1.rsa	44.186	0.343	0.037	0.619	83	0.532	36.01
T0852TS499_2-D1.rsa	44.186	0.261	0.127	0.612	82	0.539	17.91
T0852TS454_5-D1.rsa	44	0.072	0.261	0.667	92	0.478	35.87
T0852TS171_2-D1.rsa	43.75	0	0.257	0.743	101	0.433	58.46
T0852TS454_3-D1.rsa	43.75	0	0.25	0.75	102	0.429	13.79
T0852TS160_4-D2.rsa	43.75	0.018	0.236	0.745	82	0.534	15.68
T0852TS022_4-D1.rsa	43.662	0.405	0.119	0.476	80	0.546	41.51
T0852TS011_3-D1.rsa	42.857	0	0.198	0.802	105	0.408	39.88
T0852TS251_3-D1.rsa	42.857	0.119	0	0.881	111	0.386	18.25

T0852TS228_5-D1.rsa	42.857	0.015	0.313	0.672	88	0.487	16.41
T0852TS381_3-D2.rsa	42.5	0	0.404	0.596	68	0.625	26.97
T0852TS038_4-D1.rsa	42.5	0	0.325	0.675	77	0.552	20.61
T0852TS479_5-D2.rsa	42.5	0	0.368	0.632	72	0.59	15.79
T0852TS038_1-D2.rsa	42.5	0	0.316	0.684	78	0.545	16.01
T0852TS277_3-D1.rsa	42.308	0.248	0.194	0.558	92	0.46	17.56
T0852TS251_1-D2.rsa	42.308	0.297	0.188	0.515	85	0.498	19.46
T0852TS251_1-D1.rsa	42.188	0.118	0.25	0.632	86	0.491	14.71
T0852TS345_1-D2.rsa	42.188	0.096	0.316	0.588	80	0.527	13.05
T0852TS335_4-D2.rsa	42.105	0.126	0	0.874	97	0.434	29.28
T0852TS216_5-D1.rsa	42.105	0.27	0.081	0.649	72	0.585	41.67
T0852TS420_2-D2.rsa	41.86	0.239	0.134	0.627	84	0.498	29.48
T0852TS117_1-D1.rsa	41.667	0.018	0.509	0.473	52	0.801	61.14
T0852TS300_2-D2.rsa	41.667	0	0.464	0.536	59	0.706	51.82
T0852TS008_2-D2.rsa	41.071	0.23	0.087	0.683	86	0.478	19.05
T0852TS414_3-D1.rsa	41.071	0.143	0.317	0.54	68	0.604	42.26
T0852TS300_2-D1.rsa	40.625	0.015	0.382	0.603	82	0.495	70.96
T0852TS171_5-D1.rsa	40.278	0.362	0.094	0.545	116	0.347	70.94
T0852TS145_4-D1.rsa	40	0.036	0.087	0.877	121	0.331	44.02
T0852TS349_1-D1.rsa	40	0.051	0.196	0.754	104	0.385	37.14
T0852TS279_1-D2.rsa	40	0	0.421	0.579	66	0.606	38.82
T0852TS145_3-D2.rsa	40	0.08	0.239	0.681	94	0.426	13.95
T0852TS212_1-D2.rsa	39.726	0.761	0	0.239	61	0.651	41.47
T0852TS420_3-D1.rsa	39.583	0.045	0.164	0.791	87	0.455	32.05
T0852TS008_4-D1.rsa	39.535	0.313	0.075	0.612	82	0.482	26.31
T0852TS011_2-D2.rsa	39.474	0.24	0.175	0.585	127	0.311	63.36
T0852TS479_2-D1.rsa	39.474	0.253	0.203	0.544	118	0.335	64.98
T0852TS228_2-D1.rsa	39.474	0.261	0.036	0.703	78	0.506	36.94
T0852TS436_3-D1.rsa	39.474	0.241	0.37	0.389	42	0.94	69.91
T0852TS184_4-D2.rsa	39.474	0.324	0.018	0.658	73	0.541	38.51
T0852TS184_5-D2.rsa	39.474	0.27	0.108	0.622	69	0.572	37.16
T0852TS251_5-D2.rsa	39.286	0	0.382	0.618	81	0.485	17.56
T0852TS171_1-D1.rsa	38.636	0.071	0.493	0.436	129	0.3	43.33
T0852TS479_1-D1.rsa	37.719	0.267	0.166	0.567	123	0.307	52.07
T0852TS041_5-D1.rsa	37.5	0.034	0.135	0.831	246	0.152	44.76
T0852TS117_4-D2.rsa	37.5	0.408	0.07	0.521	111	0.338	54.82
T0852TS160_2-D1.rsa	37.5	0	0.324	0.676	73	0.514	62.96
T0852TS414_2-D2.rsa	37.5	0.051	0.235	0.713	97	0.387	18.57
T0852TS452_4-D2.rsa	37.5	0.151	0.278	0.571	72	0.521	46.23
T0852TS184_2-D1.rsa	37.5	0.037	0.265	0.699	95	0.395	14.15
T0852TS268_3-D1.rsa	37.5	0	0.404	0.596	68	0.551	26.97
T0852TS160_1-D1.rsa	37.5	0.096	0.213	0.691	94	0.399	14.34
T0852TS038_1-D1.rsa	37.5	0.143	0.373	0.484	61	0.615	43.25
T0852TS145_5-D2.rsa	37.5	0	0.518	0.482	55	0.682	16.45
T0852TS381_5-D2.rsa	37.209	0.276	0	0.724	97	0.384	18.1
T0852TS263_1-D2.rsa	36.842	0.063	0	0.937	104	0.354	29.28
T0852TS268_4-D1.rsa	36.842	0.234	0.099	0.667	74	0.498	37.61

T0852TS184_5-D1.rsa	36.842	0.241	0.435	0.324	35	1.053	70.14
T0852TS038_5-D2.rsa	36.842	0.259	0.37	0.37	40	0.921	52.31
T0852TS133_1-D1.rsa	36.765	0.449	0.086	0.465	113	0.325	25.78
T0852TS022_1-D2.rsa	36.207	0.637	0	0.363	93	0.389	52.44
T0852TS216_3-D1.rsa	36.111	0.413	0.094	0.493	105	0.344	59.01
T0852TS420_5-D2.rsa	35.938	0.051	0.059	0.89	121	0.297	16.54
T0852TS349_5-D2.rsa	35.938	0.074	0.309	0.618	84	0.428	13.23
T0852TS448_3-D1.rsa	35.714	0	0.405	0.595	78	0.458	54.77
T0852TS263_4-D1.rsa	35.714	0	0.443	0.557	73	0.489	58.59
T0852TS345_5-D2.rsa	35.714	0.175	0.325	0.5	63	0.567	51.59
T0852TS436_1-D1.rsa	35.616	0.702	0	0.298	76	0.469	49.71
T0852TS345_4-D1.rsa	35.616	0.765	0	0.235	60	0.594	50.78
T0852TS228_4-D2.rsa	35.526	0.268	0.03	0.702	233	0.152	15.66
T0852TS038_3-D1.rsa	35.294	0.571	0	0.429	54	0.654	46.23
T0852TS381_2-D1.rsa	35.211	0.399	0.125	0.476	80	0.44	42.63
T0852TS300_4-D2.rsa	35.211	0.417	0.095	0.488	82	0.429	33.65
T0852TS335_5-D2.rsa	35	0	0.193	0.807	92	0.38	19.52
T0852TS499_4-D2.rsa	35	0	0.456	0.544	62	0.565	16.01
T0852TS160_3-D1.rsa	34.884	0.254	0.06	0.687	92	0.379	41.42
T0852TS499_3-D1.rsa	34.884	0.349	0.142	0.509	86	0.406	50.15
T0852TS452_3-D2.rsa	34.884	0.299	0.082	0.619	83	0.42	23.88
T0852TS448_4-D2.rsa	34.694	0.378	0.094	0.528	197	0.176	26.46
T0852TS008_5-D2.rsa	34.375	0.015	0.338	0.647	88	0.391	63.6
T0852TS011_4-D1.rsa	34.375	0.015	0.404	0.581	79	0.435	73.9
T0852TS184_4-D1.rsa	34.375	0.074	0.235	0.691	94	0.366	12.87
T0852TS452_4-D1.rsa	34.247	0.761	0	0.239	61	0.561	59.51
T0852TS335_2-D1.rsa	34.211	0.259	0.324	0.417	45	0.76	83.8
T0852TS420_2-D1.rsa	33.929	0.167	0.262	0.571	72	0.471	48.61
T0852TS381_1-D1.rsa	33.929	0.167	0.278	0.556	70	0.485	42.46
T0852TS038_5-D1.rsa	33.929	0.175	0.389	0.437	55	0.617	52.78
T0852TS022_5-D2.rsa	33.684	0.517	0.051	0.432	102	0.33	51.06
T0852TS133_5-D1.rsa	33.684	0.547	0.055	0.398	94	0.358	58.69
T0852TS263_5-D1.rsa	33.621	0.582	0	0.418	107	0.314	53.22
T0852TS263_3-D1.rsa	32.877	0.757	0	0.243	62	0.53	62.84
T0852TS041_1-D2.rsa	32.877	0.749	0	0.251	64	0.514	55.98
T0852TS420_1-D1.rsa	32.877	0.769	0	0.231	59	0.557	52.74
T0852TS212_1-D1.rsa	32.813	0.081	0.184	0.735	100	0.328	14.71
T0852TS277_1-D1.rsa	32.813	0.081	0.162	0.757	103	0.319	13.23
T0852TS050_1-D1.rsa	32.759	0.613	0	0.387	99	0.331	21.97
T0852TS414_5-D2.rsa	32.692	0.236	0.115	0.648	107	0.306	16.61
T0852TS263_2-D1.rsa	32.558	0.32	0.201	0.479	81	0.402	72.04
T0852TS300_5-D1.rsa	32.558	0.328	0.015	0.657	88	0.37	37.69
T0852TS268_5-D2.rsa	32.558	0.358	0.097	0.545	73	0.446	51.31
T0852TS268_1-D1.rsa	32.558	0.239	0.134	0.627	84	0.388	24.07
T0852TS499_3-D2.rsa	32.558	0.366	0.119	0.515	69	0.472	26.49
T0852TS268_4-D2.rsa	32.5	0.018	0.088	0.895	102	0.319	23.03
T0852TS414_3-D2.rsa	32.5	0.132	0.035	0.833	95	0.342	17.32

T0852TS184_2-D2.rsa	32.5	0	0.377	0.623	71	0.458	19.3
T0852TS277_2-D2.rsa	32.5	0	0.482	0.518	59	0.551	24.56
T0852TS145_5-D1.rsa	32.394	0.494	0.107	0.399	67	0.483	50.32
T0852TS011_3-D2.rsa	32.353	0.354	0.086	0.56	136	0.238	66.22
T0852TS381_4-D2.rsa	32.143	0.175	0.341	0.484	61	0.527	52.18
T0852TS345_3-D1.rsa	32.143	0.159	0.302	0.54	68	0.473	40.67
T0852TS410_5-D2.rsa	32	0.012	0.353	0.635	209	0.153	54.86
T0852TS251_5-D1.rsa	32	0.036	0.348	0.616	85	0.376	57.43
T0852TS160_4-D1.rsa	31.579	0.241	0.37	0.389	42	0.752	93.98
T0852TS448_1-D1.rsa	31.25	0	0.036	0.964	106	0.295	19.32
T0852TS156_2-D1.rsa	31.169	0.371	0.027	0.603	135	0.231	15.83
T0852TS133_3-D1.rsa	31.034	0.586	0	0.414	106	0.293	55.57
T0852TS414_2-D1.rsa	30.986	0.53	0.101	0.369	62	0.5	62.34
T0852TS454_2-D2.rsa	30.986	0.518	0.077	0.405	68	0.456	38.14
T0852TS277_1-D2.rsa	30.769	0.364	0.206	0.43	71	0.433	15.66
T0852TS041_1-D1.rsa	30.357	0.175	0.167	0.659	83	0.366	56.55
T0852TS263_4-D2.rsa	30.357	0.167	0.317	0.516	65	0.467	48.81
T0852TS499_1-D2.rsa	30.357	0.143	0.389	0.468	59	0.515	48.81
T0852TS228_3-D2.rsa	30.303	0.201	0.101	0.698	201	0.151	46.09
T0852TS216_2-D1.rsa	30.233	0.29	0.154	0.556	94	0.322	69.82
T0852TS237_4-D1.rsa	30.233	0.337	0.213	0.45	76	0.398	72.78
T0852TS349_4-D2.rsa	30.233	0.306	0.104	0.59	79	0.383	30.41
T0852TS038_3-D2.rsa	30.233	0.366	0.09	0.545	73	0.414	36.75
T0852TS133_1-D2.rsa	30.137	0.396	0.113	0.491	182	0.166	79.43
T0852TS335_5-D1.rsa	30	0.026	0.316	0.658	75	0.4	42.54
T0852TS492_1-D2.rsa	30	0.044	0.36	0.596	68	0.441	46.93
T0852TS349_5-D1.rsa	30	0	0.14	0.86	98	0.306	17.32
T0852TS349_3-D1.rsa	29.825	0.29	0.194	0.516	112	0.266	65.21
T0852TS117_2-D2.rsa	29.6	0.018	0.395	0.587	193	0.153	54.94
T0852TS277_5-D1.rsa	29.577	0.482	0.155	0.363	61	0.485	53.2
T0852TS117_1-D2.rsa	29.524	0.359	0.205	0.436	102	0.289	71.05
T0852TS492_2-D1.rsa	29.524	0.376	0.201	0.423	99	0.298	55.45
T0852TS171_3-D1.rsa	29.487	0.405	0.125	0.47	139	0.212	55.38
T0852TS436_1-D2.rsa	29.167	0.385	0.085	0.531	113	0.258	65.1
T0852TS193_1-D1.rsa	29.07	0.343	0.201	0.456	77	0.378	65.68
T0852TS454_4-D2.rsa	28.947	0.211	0.081	0.708	235	0.123	35.17
T0852TS414_1-D1.rsa	28.947	0.241	0.5	0.259	28	1.034	93.98
T0852TS381_3-D1.rsa	28.947	0.176	0.426	0.398	43	0.673	79.4
T0852TS216_4-D2.rsa	28.947	0.25	0.491	0.259	28	1.034	94.21
T0852TS251_4-D2.rsa	28.947	0.25	0.509	0.241	26	1.113	93.75
T0852TS346_1-D2.rsa	28.947	0.287	0.306	0.407	44	0.658	70.14
T0852TS237_3-D2.rsa	28.767	0.396	0.113	0.491	182	0.158	82.15
T0852TS160_3-D2.rsa	28.767	0.773	0	0.227	58	0.496	52.16
T0852TS420_1-D2.rsa	28.571	0.198	0.079	0.722	91	0.314	45.44
T0852TS452_5-D2.rsa	28.571	0.175	0.23	0.595	75	0.381	46.83
T0852TS160_5-D2.rsa	28.571	0.175	0.405	0.421	53	0.539	54.37
T0852TS133_2-D1.rsa	28.448	0.602	0	0.398	102	0.279	56.15

T0852TS381_2-D2.rsa	28.421	0.525	0.055	0.419	99	0.287	35.8
T0852TS145_2-D1.rsa	28.169	0.506	0.113	0.381	64	0.44	52.88
T0852TS448_5-D2.rsa	28	0.483	0.025	0.493	199	0.141	39.36
T0852TS022_4-D2.rsa	28	0.072	0.326	0.601	83	0.337	65.58
T0852TS171_4-D1.rsa	27.941	0.354	0.095	0.551	134	0.209	61
T0852TS041_4-D2.rsa	27.941	0.42	0.099	0.481	117	0.239	65
T0852TS011_5-D1.rsa	27.907	0.302	0.189	0.509	86	0.324	68.49
T0852TS268_2-D1.rsa	27.907	0.306	0.03	0.664	89	0.314	28.36
T0852TS335_3-D2.rsa	27.5	0	0.079	0.921	105	0.262	21.49
T0852TS050_1-D2.rsa	27.5	0	0.14	0.86	98	0.281	16.01
T0852TS436_5-D1.rsa	27.397	0.361	0.102	0.536	199	0.138	79.22
T0852TS228_1-D2.rsa	27.397	0.418	0.094	0.488	181	0.151	78.2
T0852TS345_2-D2.rsa	27.397	0.757	0	0.243	62	0.442	42.94
T0852TS237_5-D2.rsa	27.273	0.236	0.174	0.59	170	0.16	66.15
T0852TS436_4-D1.rsa	27.211	0.378	0.08	0.542	202	0.135	74.72
T0852TS133_4-D2.rsa	26.857	0.431	0.017	0.552	223	0.12	46.16
T0852TS008_1-D1.rsa	26.786	0.151	0.333	0.516	65	0.412	50.79
T0852TS479_3-D2.rsa	26.786	0.119	0.349	0.532	67	0.4	44.84
T0852TS041_3-D2.rsa	26.733	0.271	0.14	0.589	189	0.141	74.45
T0852TS237_1-D2.rsa	26.712	0.407	0.113	0.48	178	0.15	81.06
T0852TS436_2-D2.rsa	26.712	0.41	0.113	0.477	177	0.151	78.61
T0852TS499_5-D2.rsa	26.563	0.096	0.287	0.618	84	0.316	16.73
T0852TS038_4-D2.rsa	26.563	0.103	0.272	0.625	85	0.313	16.18
T0852TS237_4-D2.rsa	26.531	0.397	0.126	0.477	178	0.149	86.32
T0852TS279_1-D1.rsa	26.316	0.25	0.491	0.259	28	0.94	93.98
T0852TS349_2-D2.rsa	26.316	0.25	0.5	0.25	27	0.975	93.98
T0852TS300_1-D1.rsa	26	0.422	0.068	0.51	128	0.203	38.05
T0852TS410_3-D2.rsa	25.85	0.397	0.107	0.496	185	0.14	85.42
T0852TS237_2-D2.rsa	25.714	0.52	0.025	0.455	184	0.14	49.88
T0852TS277_5-D2.rsa	25.581	0.366	0.052	0.582	78	0.328	36.01
T0852TS008_3-D2.rsa	25	0.37	0.082	0.547	133	0.188	66.33
T0852TS117_3-D2.rsa	25	0.457	0.099	0.444	108	0.231	69.22
T0852TS160_2-D2.rsa	25	0.096	0.294	0.61	83	0.301	16.73
T0852TS277_4-D2.rsa	25	0.018	0.377	0.605	69	0.362	21.93
T0852TS041_2-D2.rsa	24.8	0	0.359	0.641	211	0.118	47.04
T0852TS410_2-D2.rsa	24.752	0.212	0.118	0.67	215	0.115	68.85
T0852TS228_2-D2.rsa	24.752	0.287	0.15	0.564	181	0.137	74.3
T0852TS263_1-D1.rsa	24.675	0.424	0.125	0.451	101	0.244	76.67
T0852TS041_5-D2.rsa	23.529	0.337	0.058	0.605	147	0.16	63
T0852TS436_2-D1.rsa	23.377	0.433	0.125	0.442	99	0.236	77.26
T0852TS420_3-D2.rsa	23.256	0.385	0.106	0.509	144	0.161	73.76
T0852TS345_1-D1.rsa	23.256	0.306	0.015	0.679	91	0.256	24.63
T0852TS160_1-D2.rsa	23.214	0.159	0.341	0.5	63	0.368	50.2
T0852TS117_5-D1.rsa	23.129	0.373	0.115	0.512	191	0.121	82.08
T0852TS479_5-D1.rsa	22.951	0.033	0.443	0.524	110	0.209	73.88
T0852TS420_4-D2.rsa	22.84	0.428	0.105	0.467	213	0.107	54.86
T0852TS022_3-D2.rsa	22.5	0	0.167	0.833	95	0.237	46.49

T0852TS251_2-D1.rsa	22.5	0	0.298	0.702	80	0.281	46.05
T0852TS345_3-D2.rsa	22.5	0	0.386	0.614	70	0.321	44.3
T0852TS454_5-D2.rsa	22.093	0.375	0.067	0.558	158	0.14	74.38
T0852TS448_2-D1.rsa	22.059	0.428	0.103	0.469	114	0.193	70.44
T0852TS448_3-D2.rsa	21.143	0.517	0.01	0.473	191	0.111	51.24
T0852TS436_4-D2.rsa	20.988	0.441	0.09	0.469	214	0.098	74.72
T0852TS479_4-D1.rsa	20.779	0.388	0.121	0.491	110	0.189	78.45
T0852TS145_1-D2.rsa	20.588	0.73	0	0.27	34	0.606	59.92
T0852TS041_4-D1.rsa	20.37	0.443	0.083	0.474	216	0.094	66
T0852TS022_1-D1.rsa	20	0.043	0.319	0.638	88	0.227	68.66
T0852TS479_4-D2.rsa	20	0.026	0.158	0.816	93	0.215	19.52
T0852TS216_5-D2.rsa	18.812	0.274	0.125	0.601	193	0.097	68.22
T0852TS011_1-D2.rsa	18.644	0.019	0.229	0.752	161	0.116	58.06
T0852TS011_4-D2.rsa	17.442	0.392	0.117	0.491	139	0.125	81.09
T0852TS117_2-D1.rsa	17.284	0.408	0.088	0.504	230	0.075	68.1
T0852TS448_2-D2.rsa	16.832	0.252	0.115	0.632	203	0.083	68.38
T0852TS277_3-D2.rsa	16.667	0	0.345	0.655	72	0.231	16.59
T0852TS133_4-D1.rsa	16	0.043	0.355	0.601	83	0.193	67.39
T0852TS300_1-D2.rsa	16	0	0.341	0.659	91	0.176	40.04
T0852TS133_2-D2.rsa	15.789	0.337	0.105	0.557	185	0.085	98.27
T0852TS008_4-D2.rsa	14.474	0.325	0.142	0.533	177	0.082	99.4
T0852TS228_5-D2.rsa	14.474	0.346	0.117	0.536	178	0.081	99.4
T0852TS011_5-D2.rsa	13.158	0.325	0.111	0.563	187	0.07	97.89
T0852TS133_3-D2.rsa	13.158	0.334	0.123	0.542	180	0.073	99.32
T0852TS410_4-D2.rsa	11.842	0.223	0.096	0.681	226	0.052	50.6
T0873TS464_5-D1.rsa	80.663	0.065	0	0.935	432	0.187	4.54
T0873TS321_1-D1.rsa	72.376	0.21	0.065	0.725	335	0.216	6.22
T0873TS434_4-D1.rsa	69.061	0.264	0.009	0.727	336	0.206	6.98
T0873TS321_5-D1.rsa	66.298	0.223	0.054	0.723	334	0.198	6.06
T0873TS434_3-D1.rsa	65.746	0.281	0	0.719	332	0.198	6.98
T0873TS464_2-D1.rsa	64.641	0.097	0	0.903	417	0.155	6.12
T0873TS321_4-D1.rsa	64.641	0.216	0.054	0.729	337	0.192	5.95
T0873TS321_3-D1.rsa	63.536	0.21	0.093	0.697	322	0.197	6.06
T0873TS321_2-D1.rsa	62.431	0.21	0.071	0.719	332	0.188	6.17
T0873TS434_2-D1.rsa	62.431	0.251	0.004	0.745	344	0.181	6.12
T0873TS434_5-D1.rsa	58.011	0.273	0	0.727	336	0.173	5.63
T0873TS434_1-D1.rsa	58.011	0.266	0	0.734	339	0.171	5.84
T0873TS451_5-D1.rsa	53.039	0.234	0.013	0.753	348	0.152	13.15
T0873TS432_4-D1.rsa	50.829	0.225	0.05	0.725	335	0.152	10.82
T0873TS451_1-D1.rsa	47.514	0.232	0.026	0.742	343	0.139	11.09
T0873TS432_1-D1.rsa	47.514	0.234	0.022	0.745	344	0.138	12.93
T0873TS432_3-D1.rsa	46.409	0.225	0.032	0.742	343	0.135	14.12
T0873TS432_2-D1.rsa	45.856	0.232	0.035	0.734	339	0.135	13.1
T0873TS451_4-D1.rsa	45.304	0.247	0.032	0.721	333	0.136	12.12
T0873TS451_2-D1.rsa	44.751	0.236	0.03	0.734	339	0.132	13.69
T0873TS455_5-D1.rsa	41.989	0.121	0.032	0.846	391	0.107	5.41
T0873TS432_5-D1.rsa	39.227	0.255	0.048	0.697	322	0.122	12.61

T0873TS455_4-D1.rsa	38.674	0.126	0	0.874	404	0.096	5.46
T0873TS451_3-D1.rsa	37.569	0.24	0.041	0.719	332	0.113	12.55
T0873TS467_5-D1.rsa	33.149	0.424	0.028	0.548	253	0.131	5.95
T0873TS180_4-D1.rsa	32.044	0.028	0.206	0.766	354	0.091	6.93
T0873TS026_5-D1.rsa	29.834	0.208	0.121	0.671	310	0.096	6.44
T0873TS357_1-D1.rsa	28.177	0.182	0.058	0.76	351	0.08	61.42
T0873TS467_4-D1.rsa	27.072	0.286	0.084	0.63	291	0.093	9.09
T0873TS016_1-D1.rsa	25.414	0.232	0.175	0.593	274	0.093	55.74
T0873TS357_2-D1.rsa	24.862	0.193	0.024	0.784	362	0.069	61.2
T0873TS357_5-D1.rsa	24.862	0.188	0.004	0.807	373	0.067	61.09
T0873TS275_5-D1.rsa	24.309	0.255	0.158	0.587	271	0.09	58.55
T0873TS357_3-D1.rsa	24.309	0.169	0.035	0.797	368	0.066	61.36
T0873TS026_3-D1.rsa	24.309	0.247	0.147	0.606	280	0.087	60.77
T0873TS359_1-D1.rsa	23.757	0.312	0.171	0.517	239	0.099	75.27
T0873TS026_1-D1.rsa	23.757	0.305	0.173	0.522	241	0.099	73.75
T0873TS421_4-D1.rsa	23.204	0.284	0.13	0.587	271	0.086	75.87
T0873TS287_2-D1.rsa	23.204	0.307	0.165	0.528	244	0.095	77.44
T0873TS180_5-D1.rsa	23.204	0.24	0	0.76	351	0.066	9.52
T0873TS455_1-D1.rsa	23.204	0.221	0.123	0.656	303	0.077	52.49
T0873TS455_2-D1.rsa	23.204	0.19	0.022	0.788	364	0.064	39.66
T0873TS407_5-D1.rsa	23.204	0.273	0.11	0.617	285	0.081	75.27
T0873TS048_1-D1.rsa	23.204	0.316	0.165	0.519	240	0.097	74.41
T0873TS220_1-D1.rsa	23.204	0.288	0.184	0.528	244	0.095	78.41
T0873TS183_5-D1.rsa	22.652	0.279	0.136	0.584	270	0.084	72.4
T0873TS183_1-D1.rsa	22.652	0.312	0.167	0.522	241	0.094	76.79
T0873TS313_1-D1.rsa	22.652	0.305	0.158	0.537	248	0.091	77.22
T0873TS359_5-D1.rsa	22.652	0.299	0.171	0.53	245	0.092	76.3
T0873TS258_5-D1.rsa	22.652	0.303	0.169	0.528	244	0.093	74.13
T0873TS359_3-D1.rsa	22.652	0.314	0.169	0.517	239	0.095	75.65
T0873TS452_3-D1.rsa	22.652	0.268	0.188	0.543	251	0.09	59.36
T0873TS421_5-D1.rsa	22.652	0.284	0.123	0.593	274	0.083	72.94
T0873TS444_3-D1.rsa	22.652	0.331	0.169	0.5	231	0.098	73.75
T0873TS382_5-D1.rsa	22.652	0.301	0.175	0.524	242	0.094	76.46
T0873TS444_1-D1.rsa	22.652	0.333	0.167	0.5	231	0.098	73.05
T0873TS258_4-D1.rsa	22.652	0.307	0.169	0.524	242	0.094	73.48
T0873TS405_1-D1.rsa	22.652	0.327	0.165	0.509	235	0.096	78.14
T0873TS407_2-D1.rsa	22.652	0.288	0.139	0.574	265	0.085	78.79
T0873TS452_1-D1.rsa	22.652	0.323	0.171	0.506	234	0.097	73.21
T0873TS275_3-D1.rsa	22.652	0.29	0.162	0.548	253	0.09	75.43
T0873TS479_4-D1.rsa	22.099	0.271	0.149	0.58	268	0.082	64.39
T0873TS479_3-D1.rsa	22.099	0.301	0.167	0.532	246	0.09	68.83
T0873TS313_4-D1.rsa	22.099	0.305	0.158	0.537	248	0.089	77.38
T0873TS287_1-D1.rsa	22.099	0.314	0.165	0.522	241	0.092	78.9
T0873TS287_5-D1.rsa	22.099	0.312	0.16	0.528	244	0.091	78.41
T0873TS467_1-D1.rsa	22.099	0.305	0.165	0.53	245	0.09	66.4
T0873TS464_4-D1.rsa	22.099	0.301	0.158	0.541	250	0.088	77.92
T0873TS251_4-D1.rsa	22.099	0.305	0.158	0.537	248	0.089	70.29

T0873TS446_5-D1.rsa	22.099	0.29	0.152	0.558	258	0.086	74.89
T0873TS236_3-D1.rsa	22.099	0.303	0.16	0.537	248	0.089	79.06
T0873TS349_1-D1.rsa	22.099	0.292	0.156	0.552	255	0.087	76.84
T0873TS405_5-D1.rsa	22.099	0.325	0.167	0.509	235	0.094	77.6
T0873TS220_2-D1.rsa	22.099	0.292	0.175	0.532	246	0.09	78.52
T0873TS479_1-D1.rsa	21.547	0.32	0.156	0.524	242	0.089	77.38
T0873TS452_5-D1.rsa	21.547	0.201	0.154	0.645	298	0.072	71.16
T0873TS446_2-D1.rsa	21.547	0.301	0.169	0.53	245	0.088	78.08
T0873TS479_5-D1.rsa	21.547	0.284	0.141	0.576	266	0.081	72.24
T0873TS313_5-D1.rsa	21.547	0.305	0.158	0.537	248	0.087	78.19
T0873TS313_3-D1.rsa	21.547	0.305	0.158	0.537	248	0.087	78.63
T0873TS345_2-D1.rsa	21.547	0.301	0.173	0.526	243	0.089	76.95
T0873TS464_1-D1.rsa	21.547	0.301	0.158	0.541	250	0.086	77.92
T0873TS236_2-D1.rsa	21.547	0.316	0.156	0.528	244	0.088	76.41
T0873TS119_1-D1.rsa	21.547	0.292	0.156	0.552	255	0.084	76.84
T0873TS287_4-D1.rsa	21.547	0.305	0.165	0.53	245	0.088	78.9
T0873TS382_1-D1.rsa	21.547	0.299	0.169	0.532	246	0.088	76.79
T0873TS236_1-D1.rsa	21.547	0.316	0.162	0.522	241	0.089	78.46
T0873TS444_2-D1.rsa	21.547	0.318	0.175	0.506	234	0.092	73.7
T0873TS452_4-D1.rsa	21.547	0.301	0.136	0.563	260	0.083	78.68
T0873TS005_3-D1.rsa	21.547	0.301	0.184	0.515	238	0.091	83.5
T0873TS407_1-D1.rsa	21.547	0.275	0.169	0.556	257	0.084	77.92
T0873TS077_5-D1.rsa	21.547	0.29	0.175	0.535	247	0.087	78.68
T0873TS275_1-D1.rsa	21.547	0.29	0.158	0.552	255	0.084	77.38
T0873TS345_4-D1.rsa	21.547	0.314	0.173	0.513	237	0.091	76.84
T0873TS220_4-D1.rsa	21.547	0.299	0.182	0.519	240	0.09	77.87
T0873TS220_5-D1.rsa	21.547	0.286	0.18	0.535	247	0.087	78.41
T0873TS077_4-D1.rsa	21.547	0.323	0.171	0.506	234	0.092	73.59
T0873TS180_2-D1.rsa	20.994	0.307	0.145	0.548	253	0.083	52.65
T0873TS382_4-D1.rsa	20.994	0.301	0.165	0.535	247	0.085	76.57
T0873TS345_1-D1.rsa	20.994	0.314	0.171	0.515	238	0.088	75.81
T0873TS236_5-D1.rsa	20.994	0.312	0.165	0.524	242	0.087	76.95
T0873TS425_2-D1.rsa	20.994	0.29	0.165	0.545	252	0.083	79.49
T0873TS180_1-D1.rsa	20.994	0.318	0.167	0.515	238	0.088	71.59
T0873TS452_2-D1.rsa	20.994	0.292	0.16	0.548	253	0.083	75.65
T0873TS407_4-D1.rsa	20.994	0.279	0.149	0.571	264	0.08	78.25
T0873TS005_5-D1.rsa	20.994	0.299	0.184	0.517	239	0.088	82.52
T0873TS405_4-D1.rsa	20.994	0.327	0.169	0.504	233	0.09	78.03
T0873TS425_1-D1.rsa	20.994	0.279	0.177	0.543	251	0.084	79.11
T0873TS446_3-D1.rsa	20.994	0.292	0.158	0.55	254	0.083	78.41
T0873TS357_4-D1.rsa	20.994	0.199	0.009	0.792	366	0.057	61.63
T0873TS236_4-D1.rsa	20.994	0.312	0.16	0.528	244	0.086	77.17
T0873TS275_2-D1.rsa	20.994	0.277	0.162	0.561	259	0.081	77.71
T0873TS275_4-D1.rsa	20.994	0.297	0.162	0.541	250	0.084	75.11
T0873TS479_2-D1.rsa	20.442	0.314	0.16	0.526	243	0.084	71.32
T0873TS287_3-D1.rsa	20.442	0.288	0.143	0.569	263	0.078	79.49
T0873TS183_4-D1.rsa	20.442	0.275	0.136	0.589	272	0.075	68.72

T0873TS258_2-D1.rsa	20.442	0.281	0.145	0.574	265	0.077	75.92
T0873TS345_5-D1.rsa	20.442	0.316	0.182	0.502	232	0.088	75.87
T0873TS382_2-D1.rsa	20.442	0.299	0.175	0.526	243	0.084	76.35
T0873TS382_3-D1.rsa	20.442	0.303	0.165	0.532	246	0.083	76.52
T0873TS313_2-D1.rsa	20.442	0.305	0.158	0.537	248	0.082	77.44
T0873TS077_3-D1.rsa	20.442	0.264	0.16	0.576	266	0.077	79.33
T0873TS425_4-D1.rsa	20.442	0.279	0.167	0.554	256	0.08	79.55
T0873TS077_2-D1.rsa	20.442	0.29	0.165	0.545	252	0.081	78.63
T0873TS405_3-D1.rsa	20.442	0.288	0.149	0.563	260	0.079	79.11
T0873TS028_1-D1.rsa	20.442	0.297	0.16	0.543	251	0.081	75.97
T0873TS446_4-D1.rsa	20.442	0.284	0.158	0.558	258	0.079	77.92
T0873TS251_2-D1.rsa	20.442	0.305	0.169	0.526	243	0.084	71.54
T0873TS166_1-D1.rsa	20.442	0.333	0.171	0.496	229	0.089	73.16
T0873TS183_3-D1.rsa	19.89	0.273	0.143	0.584	270	0.074	63.37
T0873TS183_2-D1.rsa	19.89	0.314	0.13	0.556	257	0.077	70.29
T0873TS407_3-D1.rsa	19.89	0.262	0.156	0.582	269	0.074	76.68
T0873TS446_1-D1.rsa	19.89	0.294	0.165	0.541	250	0.08	76.3
T0873TS258_1-D1.rsa	19.89	0.301	0.139	0.561	259	0.077	76.68
T0873TS345_3-D1.rsa	19.89	0.301	0.169	0.53	245	0.081	76.73
T0873TS425_5-D1.rsa	19.89	0.271	0.158	0.571	264	0.075	79.22
T0873TS005_2-D1.rsa	19.89	0.305	0.173	0.522	241	0.083	83.5
T0873TS258_3-D1.rsa	19.89	0.284	0.165	0.552	255	0.078	76.57
T0873TS005_4-D1.rsa	19.89	0.301	0.184	0.515	238	0.084	82.63
T0873TS251_5-D1.rsa	19.89	0.316	0.16	0.524	242	0.082	73.92
T0873TS005_1-D1.rsa	19.337	0.305	0.182	0.513	237	0.082	82.52
T0873TS444_5-D1.rsa	19.337	0.297	0.167	0.537	248	0.078	73.7
T0873TS444_4-D1.rsa	18.785	0.325	0.169	0.506	234	0.08	69.7
T0873TS251_3-D1.rsa	18.785	0.307	0.169	0.524	242	0.078	70.35
T0873TS467_3-D1.rsa	18.785	0.221	0.134	0.645	298	0.063	47.67
T0873TS077_1-D1.rsa	18.232	0.297	0.158	0.545	252	0.072	78.95
T0873TS251_1-D1.rsa	17.68	0.312	0.167	0.522	241	0.073	80.95
T0873TS467_2-D1.rsa	17.127	0.249	0.154	0.597	276	0.062	47.19
T0873TS250_2-D1.rsa	17.127	0.316	0.167	0.517	239	0.072	78.25
T0873TS425_3-D1.rsa	17.127	0.288	0.158	0.554	256	0.067	78.57
T0873TS250_1-D1.rsa	17.127	0.307	0.162	0.53	245	0.07	77.87
T0873TS250_5-D1.rsa	17.127	0.305	0.167	0.528	244	0.07	78.03
T0873TS250_3-D1.rsa	16.575	0.307	0.162	0.53	245	0.068	77.92
T0873TS250_4-D1.rsa	16.022	0.303	0.162	0.535	247	0.065	77.92
T0826TS290_2-D2.rsa	89.474	0.144	0	0.856	95	0.942	25.68
T0826TS368_1-D2.rsa	81.579	0.216	0	0.784	87	0.938	16.89
T0826TS391_1-D2.rsa	76.471	0.317	0	0.683	86	0.889	34.13
T0826TS008_5-D2.rsa	75	0.053	0.099	0.847	111	0.676	14.31
T0826TS144_5-D2.rsa	75	0.218	0.024	0.758	125	0.6	16.77
T0826TS064_2-D2.rsa	72.093	0.157	0.097	0.746	100	0.721	17.16
T0826TS153_1-D2.rsa	71.875	0.015	0.125	0.86	117	0.614	39.52
T0826TS049_3-D2.rsa	71.875	0.103	0.169	0.728	99	0.726	36.77
T0826TS347_5-D2.rsa	71.053	0.207	0.018	0.775	86	0.826	35.59

T0826TS184_2-D2.rsa	71.053	0.279	0.036	0.685	76	0.935	37.61
T0826TS445_4-D2.rsa	70.833	0	0.528	0.472	51	1.389	61.81
T0826TS011_4-D2.rsa	69.643	0.056	0.016	0.929	117	0.595	15.08
T0826TS008_4-D2.rsa	67.857	0	0.015	0.985	129	0.526	19.08
T0826TS445_3-D2.rsa	67.442	0.266	0	0.734	124	0.544	13.46
T0826TS038_5-D2.rsa	66.667	0	0.454	0.546	59	1.13	64.12
T0826TS290_4-D2.rsa	66.667	0	0.519	0.481	52	1.282	66.9
T0826TS381_2-D2.rsa	66.667	0	0.491	0.509	55	1.212	66.2
T0826TS144_2-D2.rsa	65.116	0.209	0.015	0.776	104	0.626	36.75
T0826TS268_5-D2.rsa	64.286	0	0.366	0.634	83	0.775	45.23
T0826TS442_5-D2.rsa	63.462	0.261	0.036	0.703	116	0.547	60.13
T0826TS445_2-D2.rsa	62.5	0	0.157	0.843	91	0.687	36.34
T0826TS197_4-D2.rsa	60.714	0.015	0.29	0.695	91	0.667	48.09
T0826TS335_1-D2.rsa	60.714	0	0.321	0.679	89	0.682	46.37
T0826TS410_3-D2.rsa	60.526	0.162	0.081	0.757	84	0.721	35.81
T0826TS169_3-D2.rsa	60.526	0.072	0	0.928	103	0.588	17.34
T0826TS197_5-D2.rsa	60	0.065	0.268	0.667	92	0.652	19.38
T0826TS162_5-D2.rsa	58.333	0	0.417	0.583	63	0.926	59.26
T0826TS184_3-D2.rsa	58.14	0.284	0	0.716	96	0.606	40.67
T0826TS056_1-D2.rsa	57.692	0.303	0.061	0.636	105	0.549	58.07
T0826TS011_5-D2.rsa	57.692	0.285	0.085	0.63	104	0.555	53.32
T0826TS116_5-D2.rsa	57.534	0.28	0.005	0.714	265	0.217	7.02
T0826TS032_3_2-D2.rsa	57.143	0	0.099	0.901	118	0.484	16.98
T0826TS162_3-D2.rsa	55.814	0.179	0.097	0.724	97	0.575	36.38
T0826TS169_1-D2.rsa	55.814	0.201	0.104	0.694	93	0.6	36.01
T0826TS448_1-D2.rsa	55.769	0.291	0.073	0.636	105	0.531	66.14
T0826TS171_3_2-D2.rsa	55.769	0.279	0.085	0.636	105	0.531	52.53
T0826TS169_5-D2.rsa	54.167	0	0.537	0.463	50	1.083	68.06
T0826TS335_4-D2.rsa	54.167	0	0.537	0.463	50	1.083	63.89
T0826TS345_5-D2.rsa	53.571	0	0.153	0.847	111	0.483	16.6
T0826TS216_3-D2.rsa	52.632	0.045	0	0.955	106	0.497	15.99
T0826TS038_3-D2.rsa	51.786	0.175	0.024	0.802	101	0.513	21.43
T0826TS425_4-D2.rsa	51.429	0.162	0.09	0.748	175	0.294	19.44
T0826TS041_4-D2.rsa	51.163	0.299	0.015	0.687	92	0.556	43.1
T0826TS483_4-D2.rsa	51.163	0.216	0.03	0.754	101	0.507	36.38
T0826TS347_2-D2.rsa	51.163	0.321	0.015	0.664	89	0.575	42.35
T0826TS132_1-D2.rsa	51.163	0.328	0.067	0.604	81	0.632	42.91
T0826TS204_4-D2.rsa	50.505	0.274	0	0.726	209	0.242	11.29
T0826TS157_4-D2.rsa	50	0.283	0	0.717	238	0.21	10.39
T0826TS296_2-D2.rsa	50	0.055	0	0.945	156	0.321	14.56
T0826TS282_2-D2.rsa	50	0.218	0.055	0.727	120	0.417	42.41
T0826TS263_5-D2.rsa	50	0.261	0.121	0.618	102	0.49	48.73
T0826TS268_1-D2.rsa	50	0.242	0.085	0.673	111	0.45	47.31
T0826TS067_3-D2.rsa	50	0	0.083	0.917	99	0.505	35.19
T0826TS197_1-D2.rsa	50	0.015	0.389	0.595	78	0.641	58.59
T0826TS064_1-D2.rsa	50	0.255	0.048	0.697	115	0.435	17.41
T0826TS132_4-D2.rsa	49.296	0.292	0	0.708	119	0.414	22.11

T0826TS290_5-D2.rsa	48.837	0.291	0	0.709	95	0.514	44.78
T0826TS184_4-D2.rsa	48.438	0.066	0.015	0.919	125	0.388	13.97
T0826TS457_2-D2.rsa	48	0.043	0.203	0.754	104	0.462	47.65
T0826TS277_1-D2.rsa	48	0.043	0.203	0.754	104	0.462	47.46
T0826TS041_5-D2.rsa	47.887	0.238	0	0.762	128	0.374	20.51
T0826TS277_5-D2.rsa	47.475	0.007	0.278	0.715	206	0.23	7.38
T0826TS128_4-D2.rsa	46.552	0	0.035	0.965	138	0.337	57.17
T0826TS457_3-D2.rsa	46.512	0.007	0	0.993	133	0.35	13.25
T0826TS430_1-D2.rsa	46.479	0.238	0.012	0.75	126	0.369	20.99
T0826TS340_1-D2.rsa	45.313	0.015	0.316	0.669	91	0.498	63.42
T0826TS439_1-D2.rsa	44.737	0.189	0.18	0.631	70	0.639	30.41
T0826TS482_1-D2.rsa	44.186	0.261	0.03	0.709	95	0.465	42.35
T0826TS358_4-D2.rsa	44.186	0.239	0.052	0.709	95	0.465	40.48
T0826TS032_2_2-D2.rsa	43.86	0.295	0.138	0.567	123	0.357	27.65
T0826TS065_1_2-D2.rsa	43.75	0.066	0.375	0.559	76	0.576	60.11
T0826TS050_2-D2.rsa	43.75	0.073	0.309	0.618	68	0.643	60.45
T0826TS448_5-D2.rsa	43.103	0	0.098	0.902	129	0.334	59.09
T0826TS452_5-D2.rsa	43.103	0.049	0.21	0.741	106	0.407	31.29
T0826TS345_1-D2.rsa	42.857	0.038	0.435	0.527	69	0.621	61.07
T0826TS118_2-D2.rsa	42.647	0.379	0.025	0.597	145	0.294	17.56
T0826TS282_5-D2.rsa	42.623	0.01	0.376	0.614	129	0.33	31.47
T0826TS041_3-D2.rsa	42.466	0.553	0	0.447	114	0.373	44.02
T0826TS340_3-D2.rsa	42.188	0.015	0.324	0.662	90	0.469	64.52
T0826TS338_3-D2.rsa	41.667	0.073	0.3	0.627	69	0.604	64.09
T0826TS335_5-D2.rsa	41.053	0.525	0.055	0.419	99	0.415	60.17
T0826TS483_3-D2.rsa	40.698	0.296	0.124	0.58	98	0.415	59.17
T0826TS438_1-D2.rsa	40.625	0.015	0.301	0.684	93	0.437	63.23
T0826TS381_1-D2.rsa	40.625	0.029	0.353	0.618	84	0.484	70.59
T0826TS457_1-D2.rsa	40.517	0.605	0	0.395	101	0.401	53.71
T0826TS333_2-D2.rsa	40.351	0.249	0.037	0.714	155	0.26	7.83
T0826TS228_5-D2.rsa	40	0.036	0.014	0.949	131	0.305	18.48
T0826TS064_3-D2.rsa	40	0.029	0.312	0.659	91	0.44	56.88
T0826TS457_4-D2.rsa	39.773	0.068	0.466	0.466	138	0.288	34.71
T0826TS263_4-D2.rsa	39.655	0	0.245	0.755	108	0.367	76.57
T0826TS116_2-D2.rsa	39.655	0	0.035	0.965	138	0.287	46.68
T0826TS032_5_2-D2.rsa	39.583	0.018	0.127	0.855	94	0.421	60.91
T0826TS310_4-D2.rsa	39.437	0	0.28	0.72	121	0.326	12.34
T0826TS235_2-D2.rsa	39.394	0.247	0.108	0.646	186	0.212	10.42
T0826TS340_2-D2.rsa	39.063	0.015	0.375	0.61	83	0.471	68.57
T0826TS358_5-D2.rsa	39.063	0.015	0.375	0.61	83	0.471	71.69
T0826TS216_2-D2.rsa	38.947	0.513	0.051	0.436	103	0.378	51.38
T0826TS276_5-D2.rsa	38.889	0.338	0.094	0.568	121	0.321	70.43
T0826TS044_5-D2.rsa	38.889	0.362	0.085	0.554	118	0.33	66.5
T0826TS454_5-D2.rsa	38.636	0.088	0.341	0.571	169	0.229	40.29
T0826TS160_3-D2.rsa	38.158	0.205	0.099	0.696	231	0.165	42.77
T0826TS042_4-D2.rsa	37.931	0	0.203	0.797	114	0.333	82.34
T0826TS338_4-D2.rsa	37.931	0	0.042	0.958	137	0.277	46.68

T0826TS452_2-D2.rsa	37.895	0.534	0.034	0.432	102	0.372	61.23
T0826TS445_5-D2.rsa	37.895	0.534	0.055	0.411	97	0.391	58.79
T0826TS235_4-D2.rsa	37.5	0.054	0.422	0.524	155	0.242	35.39
T0826TS011_1-D2.rsa	37.5	0.394	0.085	0.521	111	0.338	69.54
T0826TS425_2-D2.rsa	37.5	0.044	0.36	0.596	81	0.463	65.81
T0826TS364_1-D2.rsa	37.209	0.254	0.015	0.731	98	0.38	44.03
T0826TS268_2-D2.rsa	37.209	0.343	0.097	0.56	75	0.496	53.17
T0826TS268_4-D2.rsa	36.842	0.508	0.055	0.436	103	0.358	61.23
T0826TS310_1-D2.rsa	36.842	0.513	0.055	0.432	102	0.361	59
T0826TS118_3-D2.rsa	36.842	0.53	0.059	0.411	97	0.38	63.98
T0826TS235_1-D2.rsa	36.207	0	0.217	0.783	112	0.323	79.55
T0826TS042_1-D2.rsa	36.207	0	0.21	0.79	113	0.32	79.2
T0826TS417_1-D2.rsa	36.207	0	0.112	0.888	127	0.285	51.4
T0826TS335_3-D2.rsa	36	0.094	0.362	0.543	75	0.48	65.94
T0826TS414_3-D2.rsa	35.938	0.015	0.309	0.676	92	0.391	62.5
T0826TS439_4-D2.rsa	35.714	0.143	0.254	0.603	76	0.47	39.29
T0826TS326_4-D2.rsa	35.354	0.201	0.174	0.625	180	0.196	62.5
T0826TS251_2-D2.rsa	35.354	0.212	0.184	0.604	174	0.203	49.91
T0826TS420_1-D2.rsa	35.294	0.329	0.041	0.63	153	0.231	13.89
T0826TS338_2-D2.rsa	35.211	0.226	0.06	0.714	120	0.293	33.97
T0826TS322_2-D2.rsa	34.884	0.272	0.142	0.586	99	0.352	66.12
T0826TS358_2-D2.rsa	34.884	0.358	0.097	0.545	73	0.478	52.98
T0826TS457_5-D2.rsa	34.722	0.423	0.07	0.507	108	0.322	59.65
T0826TS049_5-D2.rsa	34.615	0.145	0	0.855	141	0.245	13.13
T0826TS347_4-D2.rsa	34.426	0.043	0.452	0.505	106	0.325	68.78
T0826TS282_1-D2.rsa	34.375	0	0.294	0.706	96	0.358	66.91
T0826TS080_2-D2.rsa	34.286	0.47	0.01	0.52	210	0.163	37.38
T0826TS263_2-D2.rsa	34.211	0.3	0.101	0.599	130	0.263	43.43
T0826TS251_4-D2.rsa	33.824	0.305	0.045	0.65	158	0.214	55.22
T0826TS032_1_2-D2.rsa	33.824	0.407	0.025	0.568	138	0.245	23.67
T0826TS133_4-D2.rsa	33.684	0.5	0.03	0.47	111	0.303	49.26
T0826TS067_4-D2.rsa	33.684	0.492	0.03	0.479	113	0.298	49.05
T0826TS117_5-D2.rsa	33.333	0.115	0.063	0.823	237	0.141	50.78
T0826TS454_3-D2.rsa	33.333	0.282	0.167	0.551	129	0.258	69.12
T0826TS448_3-D2.rsa	33.333	0.258	0.198	0.544	118	0.282	64.29
T0826TS410_1-D2.rsa	33.333	0.385	0.075	0.54	115	0.29	69.67
T0826TS011_3-D2.rsa	32.955	0.064	0.439	0.497	147	0.224	32.01
T0826TS414_1-D2.rsa	32.877	0.549	0	0.451	115	0.286	47.74
T0826TS169_4-D2.rsa	32.759	0.598	0	0.402	103	0.318	55.18
T0826TS008_1-D2.rsa	32.759	0.602	0	0.398	102	0.321	55.27
T0826TS347_3-D2.rsa	32.632	0.496	0.03	0.475	112	0.291	49.36
T0826TS038_4-D2.rsa	32.632	0.53	0.068	0.403	95	0.343	60.17
T0826TS410_4-D2.rsa	32.558	0.302	0.148	0.55	93	0.35	71.89
T0826TS345_2-D2.rsa	32.558	0.358	0.097	0.545	73	0.446	52.98
T0826TS210_3-D2.rsa	32.203	0.056	0.313	0.631	135	0.239	53.51
T0826TS116_1-D2.rsa	32	0.537	0.015	0.448	181	0.177	7.98
T0826TS263_1-D2.rsa	32	0.014	0.304	0.681	94	0.34	63.95

T0826TS216_1-D2.rsa	32	0.014	0.159	0.826	114	0.281	47.83
T0826TS310_5-D2.rsa	32	0.022	0.362	0.616	85	0.376	66.3
T0826TS345_3-D2.rsa	32	0.014	0.275	0.71	98	0.327	57.25
T0826TS169_2-D2.rsa	32	0.072	0.326	0.601	83	0.386	57.25
T0826TS296_5-D2.rsa	31.944	0.385	0.094	0.521	111	0.288	68.02
T0826TS210_1-D2.rsa	31.897	0.594	0	0.406	104	0.307	56.84
T0826TS041_2-D2.rsa	31.579	0.166	0.124	0.71	154	0.205	31.8
T0826TS041_1-D2.rsa	31.579	0.3	0.212	0.488	106	0.298	66.47
T0826TS410_2-D2.rsa	31.579	0.432	0.059	0.508	120	0.263	52.97
T0826TS128_5-D2.rsa	31.429	0.235	0.184	0.581	136	0.231	68.91
T0826TS032_4_2-D2.rsa	31.429	0.359	0.226	0.415	97	0.324	70.83
T0826TS228_2-D2.rsa	31.2	0.021	0.289	0.69	227	0.137	54.79
T0826TS210_2-D2.rsa	31.169	0.424	0.121	0.455	102	0.306	79.52
T0826TS204_3-D2.rsa	31.148	0.048	0.405	0.548	115	0.271	66.05
T0826TS235_3-D2.rsa	31.148	0.048	0.429	0.524	110	0.283	68.03
T0826TS425_1-D2.rsa	31.034	0.602	0	0.398	102	0.304	64.26
T0826TS346_1-D2.rsa	30.882	0.346	0.091	0.564	137	0.225	66.11
T0826TS117_4-D2.rsa	30.882	0.44	0.008	0.551	134	0.23	44.44
T0826TS436_1-D2.rsa	30.857	0.458	0.027	0.515	208	0.148	29.02
T0826TS436_3-D2.rsa	30.822	0.418	0.102	0.48	178	0.173	82.97
T0826TS042_2-D2.rsa	30.769	0.341	0.125	0.534	158	0.195	54.08
T0826TS080_3-D2.rsa	30.693	0.265	0.14	0.595	191	0.161	74.14
T0826TS290_1-D2.rsa	30.526	0.542	0.021	0.436	103	0.296	58.9
T0826TS442_1-D2.rsa	30.303	0.205	0.17	0.625	180	0.168	60.33
T0826TS436_4-D2.rsa	30.286	0.394	0.042	0.564	228	0.133	24.57
T0826TS282_3-D2.rsa	30.233	0.266	0.148	0.586	99	0.305	69.82
T0826TS067_1-D2.rsa	30.233	0.284	0.154	0.562	95	0.318	73.52
T0826TS067_5-D2.rsa	30.233	0.249	0.189	0.562	95	0.318	72.34
T0826TS133_3-D2.rsa	30.233	0.29	0.183	0.527	89	0.34	71.89
T0826TS437_1-D2.rsa	30.233	0.302	0.201	0.497	84	0.36	71.45
T0826TS381_5-D2.rsa	30.233	0.296	0.183	0.521	88	0.344	67.6
T0826TS368_3-D2.rsa	30.172	0.594	0.008	0.398	102	0.296	65.14
T0826TS445_1-D2.rsa	30.172	0.582	0	0.418	107	0.282	54.69
T0826TS425_3-D2.rsa	30.172	0.629	0	0.371	95	0.318	68.65
T0826TS160_1-D2.rsa	29.703	0.296	0.162	0.542	174	0.171	53.74
T0826TS206_1-D2.rsa	29.6	0	0.374	0.626	206	0.144	37.99
T0826TS492_1-D2.rsa	29.6	0	0.395	0.605	199	0.149	36.09
T0826TS133_1-D2.rsa	29.524	0.368	0.197	0.436	102	0.289	67.95
T0826TS414_5-D2.rsa	29.524	0.359	0.171	0.47	110	0.268	54.06
T0826TS434_1-D2.rsa	29.508	0.038	0.448	0.514	108	0.273	63.93
T0826TS347_1-D2.rsa	29.412	0.42	0.099	0.481	117	0.251	67
T0826TS097_3-D2.rsa	29.412	0.453	0.123	0.424	103	0.286	64
T0826TS492_2-D2.rsa	29.293	0.222	0.17	0.608	175	0.167	59.81
T0826TS155_5-D2.rsa	29.293	0.222	0.188	0.59	170	0.172	60.68
T0826TS157_2-D2.rsa	29.143	0.399	0.01	0.592	239	0.122	34.41
T0826TS157_3-D2.rsa	29.143	0.416	0.022	0.562	227	0.128	34.9
T0826TS038_2-D2.rsa	29.07	0.308	0.178	0.515	87	0.334	70.56

T0826TS133_5-D2.rsa	29.07	0.302	0.201	0.497	84	0.346	72.19
T0826TS499_5-D2.rsa	29.07	0.32	0.183	0.497	84	0.346	72.19
T0826TS414_4-D2.rsa	29.07	0.284	0.183	0.533	90	0.323	68.94
T0826TS251_1-D2.rsa	28.814	0	0.313	0.687	147	0.196	63.08
T0826TS228_3-D2.rsa	28.8	0.012	0.362	0.626	206	0.14	52.74
T0826TS349_1-D2.rsa	28.713	0.274	0.156	0.57	183	0.157	73.99
T0826TS362_2-D2.rsa	28.571	0.453	0.027	0.52	210	0.136	38.55
T0826TS049_4-D2.rsa	28.571	0.406	0.107	0.487	109	0.262	68.57
T0826TS162_2-D2.rsa	28.448	0.602	0	0.398	102	0.279	64.26
T0826TS155_4-D2.rsa	28.283	0.247	0.17	0.583	168	0.168	46.79
T0826TS042_3-D2.rsa	28.283	0.257	0.198	0.545	157	0.18	63.54
T0826TS044_4-D2.rsa	28	0	0.225	0.775	107	0.262	63.41
T0826TS216_5-D2.rsa	28	0	0.014	0.986	136	0.206	17.21
T0826TS251_5-D2.rsa	27.941	0.333	0.041	0.626	152	0.184	68.56
T0826TS296_3-D2.rsa	27.941	0.403	0.107	0.49	119	0.235	66.89
T0826TS144_3-D2.rsa	27.941	0.416	0.111	0.473	115	0.243	68
T0826TS483_2-D2.rsa	27.907	0.296	0.172	0.533	90	0.31	70.86
T0826TS216_4-D2.rsa	27.907	0.302	0.195	0.503	85	0.328	73.52
T0826TS268_3-D2.rsa	27.907	0.325	0.201	0.473	80	0.349	68.05
T0826TS171_5_2-D2.rsa	27.778	0.315	0.094	0.592	126	0.22	65.48
T0826TS204_2-D2.rsa	27.723	0.308	0.156	0.536	172	0.161	72.35
T0826TS279_1-D2.rsa	27.619	0.35	0.205	0.444	104	0.266	71.15
T0826TS097_1-D2.rsa	27.397	0.388	0.113	0.499	185	0.148	80.18
T0826TS362_1-D2.rsa	27.273	0.149	0.188	0.663	191	0.143	63.28
T0826TS097_5-D2.rsa	27.273	0.226	0.191	0.583	168	0.162	61.02
T0826TS263_3-D2.rsa	27.273	0.233	0.212	0.556	160	0.17	68.92
T0826TS349_5-D2.rsa	27.211	0.391	0.102	0.507	189	0.144	85.14
T0826TS420_5-D2.rsa	27.193	0.217	0.134	0.65	141	0.193	41.36
T0826TS357_1-D2.rsa	27.119	0.014	0.322	0.664	142	0.191	60.75
T0826TS251_3-D2.rsa	27	0.343	0.092	0.566	142	0.19	76.49
T0826TS235_5-D2.rsa	26.923	0.378	0.128	0.493	146	0.184	56.77
T0826TS326_1-D2.rsa	26.733	0.259	0.162	0.579	186	0.144	72.74
T0826TS210_4-D2.rsa	26.667	0.346	0.218	0.436	102	0.261	70.41
T0826TS197_3-D2.rsa	26.667	0.38	0.222	0.397	93	0.287	72.11
T0826TS160_4-D2.rsa	26.531	0.373	0.099	0.528	197	0.135	82.57
T0826TS362_5-D2.rsa	26.531	0.381	0.126	0.493	184	0.144	74.17
T0826TS499_3-D2.rsa	26.263	0.25	0.222	0.528	152	0.173	71.88
T0826TS153_3-D2.rsa	25.974	0.357	0.08	0.563	126	0.206	70.95
T0826TS276_3-D2.rsa	25.714	0.441	0	0.559	226	0.114	35.95
T0826TS128_1-D2.rsa	25.714	0.463	0.027	0.51	206	0.125	35.58
T0826TS008_3-D2.rsa	25.714	0.286	0.192	0.521	122	0.211	63.03
T0826TS414_2-D2.rsa	25.714	0.419	0.107	0.474	111	0.232	67.63
T0826TS277_4-D2.rsa	25.714	0.406	0.167	0.427	100	0.257	68.48
T0826TS116_3-D2.rsa	25.424	0	0.07	0.93	199	0.128	54.56
T0826TS128_3-D2.rsa	25.424	0.009	0.271	0.72	154	0.165	62.15
T0826TS276_2-D2.rsa	25.342	0.41	0.105	0.485	180	0.141	59.81
T0826TS290_3-D2.rsa	25.143	0.569	0.005	0.426	172	0.146	8.23

T0826TS117_1-D2.rsa	25	0.473	0.091	0.436	106	0.236	66.67
T0826TS132_2-D2.rsa	24.675	0.415	0.129	0.455	102	0.242	82.14
T0826TS277_2-D2.rsa	24.675	0.388	0.121	0.491	110	0.224	81.91
T0826TS133_2-D2.rsa	24.675	0.42	0.143	0.438	98	0.252	71.43
T0826TS349_3-D2.rsa	24.658	0.396	0.1	0.504	187	0.132	81.06
T0826TS153_4-D2.rsa	24.59	0.024	0.257	0.719	151	0.163	71.77
T0826TS282_4-D2.rsa	24.59	0.062	0.433	0.505	106	0.232	63.31
T0826TS349_2-D2.rsa	24.571	0.527	0.01	0.463	187	0.131	43.69
T0826TS034_1-D2.rsa	24.571	0.535	0.03	0.436	176	0.14	50.93
T0826TS296_4-D2.rsa	24.561	0.356	0.091	0.553	121	0.203	61.99
T0826TS310_2-D2.rsa	24.561	0.457	0.091	0.452	99	0.248	81.39
T0826TS050_1-D2.rsa	24.561	0.438	0.096	0.466	102	0.241	66.1
T0826TS322_3-D2.rsa	24.359	0.416	0.057	0.527	156	0.156	57.55
T0826TS362_3-D2.rsa	24.242	0.191	0.198	0.611	176	0.138	52.95
T0826TS050_4-D2.rsa	24.211	0.525	0.021	0.453	107	0.226	59.53
T0826TS439_5-D2.rsa	24	0.422	0.116	0.462	116	0.207	89.24
T0826TS430_2-D2.rsa	24	0.418	0.068	0.514	129	0.186	38.35
T0826TS483_1-D2.rsa	24	0.051	0.348	0.601	83	0.289	68.12
T0826TS080_4-D2.rsa	23.81	0.413	0.131	0.456	170	0.14	88.19
T0826TS118_1-D2.rsa	23.81	0.286	0.231	0.483	113	0.211	60.26
T0826TS276_1-D2.rsa	23.729	0.019	0.215	0.766	164	0.145	57.71
T0826TS360_1-D2.rsa	23.529	0.362	0.115	0.523	127	0.185	68.78
T0826TS499_1-D2.rsa	23.529	0.412	0.107	0.481	117	0.201	71.33
T0826TS064_4-D2.rsa	23.529	0.354	0.008	0.638	155	0.152	10.22
T0826TS333_1-D2.rsa	23.377	0.406	0.125	0.469	105	0.223	78.57
T0826TS204_5-D2.rsa	23.256	0.403	0.117	0.481	136	0.171	80.39
T0826TS162_4-D2.rsa	23.256	0.308	0.16	0.533	90	0.258	68.64
T0826TS296_1-D2.rsa	23.077	0.385	0.135	0.48	142	0.163	58.68
T0826TS358_1-D2.rsa	23.077	0.409	0.132	0.459	136	0.17	59.03
T0826TS153_2-D2.rsa	23	0.458	0.135	0.406	102	0.225	84.56
T0826TS499_2-D2.rsa	22.807	0.466	0.059	0.475	104	0.219	81.16
T0826TS448_4-D2.rsa	22.772	0.259	0.156	0.586	188	0.121	74.84
T0826TS349_4-D2.rsa	22.449	0.383	0.107	0.509	190	0.118	84.58
T0826TS326_2-D2.rsa	22.449	0.381	0.115	0.504	188	0.119	82.36
T0826TS160_2-D2.rsa	22.368	0.208	0.06	0.732	243	0.092	41.19
T0826TS360_3-D2.rsa	22.368	0.337	0.012	0.651	216	0.104	8.96
T0826TS157_5-D2.rsa	22.222	0.406	0.088	0.507	231	0.096	67.27
T0826TS155_3-D2.rsa	22.093	0.367	0.117	0.516	146	0.151	73.85
T0826TS044_1-D2.rsa	21.795	0.399	0.132	0.47	139	0.157	59.29
T0826TS155_2-D2.rsa	21.212	0.177	0.108	0.715	206	0.103	11.89
T0826TS044_3-D2.rsa	21.053	0.443	0.091	0.466	102	0.206	82.08
T0826TS499_4-D2.rsa	21.053	0.447	0.1	0.452	99	0.213	80.59
T0826TS008_2-D2.rsa	21.053	0.429	0.091	0.479	105	0.201	79.8
T0826TS144_4-D2.rsa	21.053	0.425	0.082	0.493	108	0.195	76.14
T0826TS492_5-D2.rsa	20.93	0.413	0.117	0.47	133	0.157	78.71
T0826TS442_3-D2.rsa	20.93	0.392	0.12	0.488	138	0.152	82.95
T0826TS492_3-D2.rsa	20.93	0.396	0.12	0.484	137	0.153	82.77

T0826TS442_4-D2.rsa	20.93	0.41	0.124	0.466	132	0.159	77.21
T0826TS362_4-D2.rsa	20.792	0.227	0.14	0.632	203	0.102	73.13
T0826TS023_1-D2.rsa	20.779	0.384	0.147	0.469	105	0.198	77.74
T0826TS162_1_2-D2.rsa	20.779	0.397	0.138	0.464	104	0.2	77.62
T0826TS237_4-D2.rsa	20.513	0.389	0.125	0.486	144	0.142	57.99
T0826TS063_1-D2.rsa	20.395	0.465	0.011	0.524	239	0.085	60.09
T0826TS026_1-D2.rsa	20.339	0.061	0.238	0.701	150	0.136	56.08
T0826TS050_3-D2.rsa	20	0.014	0.341	0.645	89	0.225	60.69
T0826TS345_4-D2.rsa	20	0.065	0.326	0.609	84	0.238	71.38
T0826TS439_3-D2.rsa	19.767	0.399	0.042	0.558	158	0.125	78.18
T0826TS157_1-D2.rsa	19.737	0.496	0.024	0.48	219	0.09	64.53
T0826TS097_4-D2.rsa	19.481	0.393	0.125	0.482	108	0.18	80.83
T0826TS193_1-D2.rsa	19.481	0.42	0.121	0.46	103	0.189	80.59
T0826TS171_1_2-D2.rsa	19.298	0.425	0.041	0.534	117	0.165	68.84
T0826TS340_4-D2.rsa	19.298	0.402	0.091	0.507	111	0.174	67.58
T0826TS049_2-D2.rsa	19.298	0.466	0.091	0.443	97	0.199	80.14
T0826TS228_4-D2.rsa	19.079	0.465	0.015	0.52	237	0.081	57.51
T0826TS425_5-D2.rsa	18.605	0.371	0.113	0.516	146	0.127	73.41
T0826TS160_5-D2.rsa	18.421	0.461	0.02	0.52	237	0.078	58.5
T0826TS153_5-D2.rsa	18.421	0.28	0.127	0.593	197	0.094	71.39
T0826TS097_2-D2.rsa	18.182	0.357	0.121	0.522	117	0.155	74.52
T0826TS204_1-D2.rsa	18.182	0.384	0.134	0.482	108	0.168	82.14
T0826TS326_5-D2.rsa	17.901	0.425	0.094	0.48	219	0.082	75.33
T0826TS049_1-D2.rsa	17.544	0.452	0.082	0.466	102	0.172	80.36
T0826TS210_5-D2.rsa	17.544	0.457	0.082	0.461	101	0.174	80.59
T0826TS410_5-D2.rsa	17.544	0.447	0.082	0.47	103	0.17	80.82
T0826TS197_2-D2.rsa	17.544	0.429	0.087	0.484	106	0.166	65.64
T0826TS322_1-D2.rsa	17.442	0.382	0.106	0.512	145	0.12	70.67
T0826TS063_2-D2.rsa	17.105	0.43	0.033	0.537	245	0.07	65.46
T0826TS439_2-D2.rsa	17.105	0.43	0.004	0.566	258	0.066	58.94
T0826TS042_5-D2.rsa	17.105	0.34	0.142	0.518	172	0.099	67.77
T0826TS063_3-D2.rsa	16.923	0.266	0.231	0.502	230	0.074	69.5
T0826TS300_1-D2.rsa	16	0.036	0.225	0.739	102	0.157	63.04
T0826TS171_4_2-D2.rsa	15.789	0.406	0.078	0.516	113	0.14	78.08
T0826TS237_1-D2.rsa	15.789	0.411	0.068	0.521	114	0.139	77.85
T0826TS368_2-D2.rsa	15.789	0.47	0.091	0.438	96	0.164	83.9
T0826TS338_5-D2.rsa	15.789	0.438	0.082	0.479	105	0.15	81.96
T0826TS132_3-D2.rsa	15.789	0.457	0.091	0.452	99	0.159	82.99
T0826TS276_4-D2.rsa	14.894	0.346	0.195	0.459	118	0.126	86.38
T0826TS063_4-D2.rsa	14.615	0.284	0.159	0.557	255	0.057	75.17
T0826TS300_4-D2.rsa	14.474	0.343	0.136	0.521	173	0.084	94.8
T0826TS338_1-D2.rsa	14.035	0.42	0.073	0.507	111	0.126	79.45
T0826TS212_1-D2.rsa	14.035	0.447	0.105	0.447	98	0.143	80.59
T0826TS436_2-D2.rsa	13.158	0.31	0.111	0.578	192	0.069	91.27
T0826TS442_2-D2.rsa	13.158	0.334	0.123	0.542	180	0.073	99.32
T0826TS116_4-D2.rsa	13.158	0.346	0.133	0.521	173	0.076	96.76
T0826TS454_2-D2.rsa	12.766	0.315	0.171	0.514	132	0.097	80.93

T0826TS237_5-D2.rsa	12.766	0.339	0.183	0.479	123	0.104	83.66
T0826TS144_1-D2.rsa	12.766	0.35	0.206	0.444	114	0.112	84.92
T0826TS454_4-D2.rsa	10.638	0.311	0.101	0.588	151	0.07	75.49
T0826TS300_3-D2.rsa	10.638	0.35	0.125	0.525	135	0.079	84.34
T0826TS420_3-D2.rsa	10.638	0.366	0.198	0.436	112	0.095	85.31
T0826TS322_4-D2.rsa	10.638	0.377	0.187	0.436	112	0.095	81.91
T0826TS011_2-D2.rsa	8.511	0.342	0.198	0.459	118	0.072	84.05
T0776TS171_1-D1.rsa	84.211	0.306	0	0.694	77	1.094	13.96
T0776TS210_3-D1.rsa	79.412	0.373	0	0.627	79	1.005	29.36
T0776TS410_1-D1.rsa	76.471	0.429	0.016	0.556	70	1.092	17.46
T0776TS145_2-D1.rsa	72.093	0.231	0.045	0.724	97	0.743	38.25
T0776TS160_1-D1.rsa	71.429	0.015	0.13	0.855	112	0.638	28.24
T0776TS499_4-D1.rsa	70.588	0.532	0	0.468	59	1.196	30.95
T0776TS345_2-D1.rsa	64.706	0.571	0	0.429	54	1.198	34.13
T0776TS300_4-D1.rsa	63.158	0.171	0	0.829	92	0.686	17.12
T0776TS022_5-D1.rsa	63.158	0.117	0.126	0.757	84	0.752	19.82
T0776TS277_5-D1.rsa	62.5	0.045	0	0.955	105	0.595	14.09
T0776TS216_3-D1.rsa	61.765	0.651	0	0.349	44	1.404	49.8
T0776TS479_4-D1.rsa	61.538	0.285	0.097	0.618	102	0.603	42.41
T0776TS008_3-D1.rsa	60.526	0.297	0.018	0.685	76	0.796	36.26
T0776TS381_4-D1.rsa	60.465	0.261	0.134	0.604	81	0.746	20.15
T0776TS171_4-D1.rsa	58.824	0.421	0	0.579	73	0.806	41.27
T0776TS156_5-D1.rsa	58.333	0	0.546	0.454	49	1.19	57.64
T0776TS277_4-D1.rsa	58.333	0	0.602	0.398	43	1.357	51.16
T0776TS160_3-D1.rsa	58.14	0.261	0.015	0.724	97	0.599	41.79
T0776TS038_5-D1.rsa	57.895	0.288	0.126	0.586	65	0.891	31.98
T0776TS420_5-D1.rsa	57.5	0	0.289	0.711	81	0.71	34.43
T0776TS479_3-D1.rsa	57.143	0	0.244	0.756	99	0.577	29.58
T0776TS335_3-D1.rsa	56.25	0.272	0	0.728	99	0.568	10.48
T0776TS335_2-D1.rsa	56	0.268	0	0.732	101	0.554	13.41
T0776TS436_3-D1.rsa	55.769	0.261	0.097	0.642	106	0.526	73.42
T0776TS479_1-D1.rsa	55.769	0.376	0.03	0.594	98	0.569	15.51
T0776TS210_4-D1.rsa	55.357	0.087	0.302	0.611	77	0.719	17.46
T0776TS210_2-D1.rsa	55.263	0.099	0.054	0.847	94	0.588	27.25
T0776TS414_1-D1.rsa	54.167	0.037	0.463	0.5	54	1.003	48.61
T0776TS335_4-D1.rsa	53.125	0	0.265	0.735	100	0.531	11.21
T0776TS499_2-D1.rsa	53.125	0.059	0.243	0.699	95	0.559	12.5
T0776TS133_1-D1.rsa	52.941	0.492	0	0.508	64	0.827	33.53
T0776TS381_3-D1.rsa	51.923	0.224	0.218	0.558	92	0.564	14.24
T0776TS452_4-D1.rsa	50.704	0.214	0.214	0.571	96	0.528	14.26
T0776TS210_5-D1.rsa	50.704	0.452	0	0.548	92	0.551	25.16
T0776TS184_2-D1.rsa	50	0.153	0	0.847	94	0.532	19.37
T0776TS349_4-D1.rsa	50	0.253	0.23	0.516	112	0.446	11.87
T0776TS349_3-D1.rsa	50	0	0.427	0.573	63	0.794	47.95
T0776TS454_2-D1.rsa	50	0.126	0	0.874	97	0.515	17.12
T0776TS022_4-D1.rsa	50	0.028	0.38	0.593	64	0.781	34.72
T0776TS448_1-D1.rsa	47.917	0.036	0.382	0.582	64	0.749	67.27

T0776TS420_4-D1.rsa	47.887	0.03	0.321	0.649	109	0.439	10.58
T0776TS184_5-D1.rsa	47.5	0.035	0.105	0.86	98	0.485	32.9
T0776TS011_1-D1.rsa	47.368	0.279	0.126	0.595	66	0.718	38.51
T0776TS452_2-D1.rsa	47.368	0.306	0.117	0.577	64	0.74	37.61
T0776TS381_2-D1.rsa	47.368	0.234	0.072	0.694	77	0.615	23.2
T0776TS492_5-D1.rsa	47.368	0.189	0.162	0.649	72	0.658	31.08
T0776TS228_4-D1.rsa	46.429	0.071	0.063	0.865	109	0.426	18.25
T0776TS300_5-D1.rsa	46.429	0	0.237	0.763	100	0.464	15.46
T0776TS133_4-D1.rsa	46.429	0	0.282	0.718	94	0.494	16.03
T0776TS008_2-D1.rsa	45.833	0.019	0.556	0.426	46	0.996	71.76
T0776TS041_3-D1.rsa	45.313	0.059	0.243	0.699	95	0.477	14.15
T0776TS452_3-D1.rsa	45	0	0.316	0.684	78	0.577	21.71
T0776TS228_1-D1.rsa	44.737	0.117	0	0.883	98	0.456	24.32
T0776TS381_5-D1.rsa	44.737	0.234	0.018	0.748	83	0.539	34.91
T0776TS228_5-D1.rsa	44.231	0.061	0.012	0.927	153	0.289	12.5
T0776TS349_5-D1.rsa	44	0	0.167	0.833	115	0.383	13.59
T0776TS228_3-D1.rsa	43.662	0.024	0.173	0.804	135	0.323	11.7
T0776TS448_5-D1.rsa	42.5	0.07	0.053	0.877	100	0.425	37.28
T0776TS263_4-D1.rsa	42.105	0	0.036	0.964	107	0.394	23.2
T0776TS414_5-D1.rsa	42.105	0.117	0	0.883	98	0.43	29.95
T0776TS145_3-D1.rsa	42.045	0.064	0.402	0.534	158	0.266	36.06
T0776TS277_3-D1.rsa	41.86	0.239	0.112	0.649	87	0.481	25
T0776TS492_4-D1.rsa	41.667	0.073	0.282	0.645	71	0.587	52.5
T0776TS479_5-D1.rsa	41.071	0.175	0.27	0.556	70	0.587	50.79
T0776TS050_1-D1.rsa	40.845	0.458	0.107	0.435	73	0.56	50.8
T0776TS277_2-D1.rsa	40.625	0	0.221	0.779	106	0.383	13.97
T0776TS263_3-D1.rsa	40	0	0.014	0.986	136	0.294	13.77
T0776TS022_3-D1.rsa	39.583	0.055	0.345	0.6	66	0.6	59.32
T0776TS263_5-D1.rsa	39.535	0.239	0.015	0.746	100	0.395	21.83
T0776TS452_5-D1.rsa	39.535	0.313	0.09	0.597	80	0.494	44.4
T0776TS038_1-D1.rsa	39.474	0.162	0	0.838	93	0.424	17.57
T0776TS268_2-D1.rsa	39.474	0.297	0.081	0.622	69	0.572	40.77
T0776TS279_1-D1.rsa	39.437	0.411	0.113	0.476	80	0.493	38.14
T0776TS492_2-D1.rsa	39.437	0.393	0.125	0.482	81	0.487	41.35
T0776TS499_1-D1.rsa	39.286	0.167	0.302	0.532	67	0.586	48.81
T0776TS410_5-D1.rsa	39.286	0.159	0.27	0.571	72	0.546	43.45
T0776TS171_5-D1.rsa	39.063	0.015	0.36	0.625	85	0.46	63.23
T0776TS216_4-D1.rsa	39.063	0.147	0.118	0.735	100	0.391	13.79
T0776TS117_5-D1.rsa	38.4	0.015	0.313	0.672	221	0.174	41.11
T0776TS335_1-D1.rsa	38.028	0.399	0.143	0.458	77	0.494	41.03
T0776TS492_1-D1.rsa	37.5	0.19	0.19	0.619	78	0.481	50
T0776TS216_1-D1.rsa	37.5	0.079	0.158	0.763	87	0.431	33.33
T0776TS268_3-D1.rsa	37.5	0.143	0.23	0.627	79	0.475	46.23
T0776TS038_2-D1.rsa	37.5	0.018	0.263	0.719	82	0.457	19.74
T0776TS335_5-D1.rsa	37.209	0.313	0	0.687	92	0.404	26.31
T0776TS038_3-D1.rsa	37.209	0.261	0.082	0.657	88	0.423	24.63
T0776TS073_1-D1.rsa	36.986	0.667	0	0.333	85	0.435	37.84

T0776TS492_3-D1.rsa	36.62	0.405	0.143	0.452	76	0.482	40.7
T0776TS268_1-D1.rsa	36.62	0.482	0.095	0.423	71	0.516	50.48
T0776TS212_1-D1.rsa	36	0.072	0.312	0.616	85	0.424	35.51
T0776TS345_3-D1.rsa	35.714	0	0.229	0.771	101	0.354	16.6
T0776TS011_5-D1.rsa	35.714	0.143	0.254	0.603	76	0.47	45.63
T0776TS133_3-D1.rsa	35.616	0.753	0	0.247	63	0.565	58.53
T0776TS251_2-D1.rsa	35.417	0.045	0.236	0.718	79	0.448	65.23
T0776TS454_3-D1.rsa	35.227	0.088	0.453	0.459	136	0.259	49.24
T0776TS300_3-D1.rsa	34.884	0.358	0.097	0.545	73	0.478	51.49
T0776TS420_1-D1.rsa	34.884	0.313	0.06	0.627	84	0.415	34.33
T0776TS410_3-D1.rsa	34.247	0.741	0	0.259	66	0.519	57.26
T0776TS499_5-D1.rsa	34.211	0.207	0	0.793	88	0.389	38.51
T0776TS277_1-D1.rsa	34.211	0.25	0.417	0.333	36	0.95	93.06
T0776TS346_1-D1.rsa	34.211	0.241	0.157	0.602	65	0.526	64.81
T0776TS414_4-D1.rsa	33.684	0.572	0.055	0.373	88	0.383	58.48
T0776TS041_2-D1.rsa	33.333	0.018	0.409	0.573	63	0.529	68.86
T0776TS436_1-D1.rsa	32.955	0.074	0.48	0.446	132	0.25	47.38
T0776TS008_1-D1.rsa	32.877	0.729	0	0.271	69	0.476	56.67
T0776TS008_5-D1.rsa	32.877	0.749	0	0.251	64	0.514	57.45
T0776TS073_2-D1.rsa	32.813	0.103	0.404	0.493	67	0.49	61.77
T0776TS184_4-D1.rsa	32.759	0.668	0	0.332	85	0.385	60.35
T0776TS448_4-D1.rsa	32.632	0.5	0.03	0.47	111	0.294	49.36
T0776TS038_4-D1.rsa	32.632	0.525	0.034	0.441	104	0.314	59.11
T0776TS184_1-D1.rsa	32.558	0.299	0	0.701	94	0.346	39.74
T0776TS008_4-D1.rsa	32.5	0	0.368	0.632	72	0.451	43.42
T0776TS133_5-D1.rsa	32.5	0.07	0.14	0.789	90	0.361	38.6
T0776TS117_3-D1.rsa	32.5	0	0.447	0.553	63	0.516	46.27
T0776TS041_5-D1.rsa	32.143	0.175	0.333	0.492	62	0.518	52.58
T0776TS436_4-D1.rsa	31.944	0.362	0.089	0.549	117	0.273	68.27
T0776TS022_1-D1.rsa	31.579	0.25	0.491	0.259	28	1.128	93.98
T0776TS171_3-D1.rsa	31.25	0.082	0.282	0.636	70	0.446	61.59
T0776TS300_2-D1.rsa	31.034	0.664	0	0.336	86	0.361	62.6
T0776TS420_3-D1.rsa	30.986	0.524	0.095	0.381	64	0.484	61.06
T0776TS300_1-D1.rsa	30.508	0.019	0.318	0.664	142	0.215	52.34
T0776TS145_1-D1.rsa	30.476	0.342	0.145	0.513	120	0.254	65.49
T0776TS410_4-D1.rsa	30.357	0.222	0	0.778	98	0.31	43.85
T0776TS117_4-D1.rsa	30.357	0.183	0.31	0.508	64	0.474	53.37
T0776TS414_2-D1.rsa	30	0	0.289	0.711	81	0.37	42.98
T0776TS381_1-D1.rsa	30	0.026	0.36	0.614	70	0.429	46.05
T0776TS452_1-D1.rsa	30	0	0.351	0.649	74	0.405	48.03
T0776TS345_5-D1.rsa	29.577	0.345	0.024	0.631	106	0.279	20.67
T0776TS145_5-D1.rsa	29.524	0.325	0.201	0.474	111	0.266	64.64
T0776TS268_5-D1.rsa	28.947	0.25	0.5	0.25	27	1.072	93.98
T0776TS268_4-D1.rsa	28.767	0.757	0	0.243	62	0.464	62.55
T0776TS499_3-D1.rsa	28.169	0.524	0.06	0.417	70	0.402	60.74
T0776TS448_2-D1.rsa	28	0.051	0.319	0.63	87	0.322	57.97
T0776TS263_1-D1.rsa	28	0	0.29	0.71	98	0.286	46.38

T0776TS436_2-D1.rsa	27.941	0.387	0.058	0.556	135	0.207	67.44
T0776TS436_5-D1.rsa	27.869	0	0.338	0.662	139	0.2	50.62
T0776TS041_1-D1.rsa	27.778	0.493	0.085	0.423	90	0.309	57.11
T0776TS345_1-D1.rsa	27.368	0.576	0.047	0.377	89	0.308	55.19
T0776TS117_1-D1.rsa	26.786	0.19	0.063	0.746	94	0.285	31.35
T0776TS160_4-D1.rsa	26.23	0.038	0.438	0.524	110	0.238	66.29
T0776TS145_4-D1.rsa	25.974	0.339	0.138	0.522	117	0.222	71.67
T0776TS251_1-D1.rsa	25.581	0.349	0.207	0.444	75	0.341	69.23
T0776TS448_3-D1.rsa	25	0.029	0	0.971	132	0.189	13.05
T0776TS410_2-D1.rsa	25	0	0.088	0.912	104	0.24	40.13
T0776TS479_2-D1.rsa	24	0.051	0.326	0.623	86	0.279	52.35
T0776TS133_2-D1.rsa	24	0.08	0.355	0.565	78	0.308	66.67
T0776TS420_2-D1.rsa	24	0.036	0.13	0.833	115	0.209	17.21
T0776TS251_3-D1.rsa	23.377	0.388	0.116	0.496	111	0.211	71.07
T0776TS011_4-D1.rsa	21.053	0.231	0.343	0.426	46	0.458	88.19
T0776TS454_1-D1.rsa	17.647	0.379	0.07	0.551	134	0.132	68.22
T0776TS251_5-D1.rsa	17.544	0.457	0.091	0.452	99	0.177	81.62
T0776TS117_2-D1.rsa	17.105	0.465	0.024	0.511	233	0.073	61.29
T0776TS022_2-D1.rsa	16	0.058	0.268	0.674	93	0.172	47.28
T0776TS041_4-D1.rsa	16	0.051	0.312	0.638	88	0.182	47.46
T0776TS011_3-D1.rsa	12	0.036	0.261	0.703	97	0.124	48.91
T0881TS464_2-D1.rsa	85	0.015	0	0.985	199	0.427	7.92
T0881TS464_5-D1.rsa	81.25	0	0.01	0.99	200	0.406	8.54
T0881TS321_1-D1.rsa	65	0.015	0.282	0.703	142	0.458	11.88
T0881TS321_3-D1.rsa	60	0.015	0.257	0.728	147	0.408	13.24
T0881TS446_4-D1.rsa	58.75	0.02	0.381	0.599	121	0.486	8.04
T0881TS321_4-D1.rsa	57.5	0.015	0.252	0.733	148	0.389	12.38
T0881TS321_5-D1.rsa	55	0.015	0.223	0.762	154	0.357	10.4
T0881TS455_5-D1.rsa	55	0.01	0.069	0.921	186	0.296	7.3
T0881TS434_5-D1.rsa	55	0.035	0	0.965	195	0.282	9.78
T0881TS455_4-D1.rsa	53.75	0	0.01	0.99	200	0.269	7.92
T0881TS455_3-D1.rsa	53.75	0	0.03	0.97	196	0.274	8.79
T0881TS451_2-D1.rsa	52.5	0	0.059	0.941	190	0.276	12.75
T0881TS434_1-D1.rsa	52.5	0.045	0	0.955	193	0.272	14.11
T0881TS451_1-D1.rsa	51.25	0	0.069	0.931	188	0.273	12.87
T0881TS321_2-D1.rsa	51.25	0.025	0.277	0.698	141	0.363	13.86
T0881TS451_3-D1.rsa	50	0	0.104	0.896	181	0.276	12.62
T0881TS434_2-D1.rsa	48.75	0.04	0.03	0.931	188	0.259	11.26
T0881TS451_5-D1.rsa	47.5	0	0.104	0.896	181	0.262	13.98
T0881TS284_5-D1.rsa	47.5	0.005	0.134	0.861	174	0.273	9.28
T0881TS432_4-D1.rsa	47.5	0.05	0.203	0.748	151	0.315	12.99
T0881TS434_4-D1.rsa	46.25	0.045	0	0.955	193	0.24	10.52
T0881TS434_3-D1.rsa	46.25	0.03	0.01	0.96	194	0.238	10.77
T0881TS432_3-D1.rsa	46.25	0.05	0.233	0.718	145	0.319	13.12
T0881TS432_2-D1.rsa	45	0.05	0.183	0.767	155	0.29	10.52
T0881TS455_1-D1.rsa	43.75	0.01	0.153	0.837	169	0.259	8.66
T0881TS467_5-D1.rsa	43.75	0	0.243	0.757	153	0.286	9.78

T0881TS446_5-D1.rsa	43.75	0.02	0.366	0.614	124	0.353	12.01
T0881TS451_4-D1.rsa	43.75	0	0.03	0.97	196	0.223	13.12
T0881TS407_3-D1.rsa	42.5	0	0.099	0.901	182	0.234	12.13
T0881TS180_4-D1.rsa	41.25	0.035	0.188	0.777	157	0.263	15.59
T0881TS284_1-D1.rsa	41.25	0.02	0.252	0.728	147	0.281	10.77
T0881TS432_5-D1.rsa	41.25	0.045	0.218	0.738	149	0.277	10.89
T0881TS407_2-D1.rsa	40	0	0.188	0.812	164	0.244	13.37
T0881TS284_4-D1.rsa	40	0.025	0.233	0.743	150	0.267	10.64
T0881TS455_2-D1.rsa	38.75	0	0.084	0.916	185	0.209	8.66
T0881TS180_2-D1.rsa	36.25	0	0.317	0.683	138	0.263	11.88
T0881TS432_1-D1.rsa	36.25	0.04	0.287	0.673	136	0.267	12.01
T0881TS180_3-D1.rsa	36.25	0	0.213	0.787	159	0.228	14.48
T0881TS407_1-D1.rsa	36.25	0	0.129	0.871	176	0.206	10.89
T0881TS345_5-D1.rsa	36.25	0.015	0.391	0.594	120	0.302	35.64
T0881TS452_3-D1.rsa	35	0.025	0.332	0.644	130	0.269	10.15
T0881TS407_4-D1.rsa	35	0.01	0.124	0.866	175	0.2	11.63
T0881TS495_2-D1.rsa	33.75	0.035	0.233	0.733	148	0.228	49.13
T0881TS430_3-D1.rsa	32.5	0.025	0.297	0.678	137	0.237	58.79
T0881TS220_2-D1.rsa	32.5	0.025	0.272	0.703	142	0.229	56.81
T0881TS430_2-D1.rsa	31.25	0.025	0.302	0.673	136	0.23	56.44
T0881TS430_5-D1.rsa	31.25	0.03	0.277	0.693	140	0.223	58.29
T0881TS183_4-D1.rsa	31.25	0.035	0.233	0.733	148	0.211	52.72
T0881TS407_5-D1.rsa	31.25	0	0.059	0.941	190	0.164	10.89
T0881TS251_1-D1.rsa	31.25	0.035	0.361	0.604	122	0.256	53.71
T0881TS357_2-D1.rsa	30	0	0.03	0.97	196	0.153	55.07
T0881TS430_1-D1.rsa	30	0.02	0.337	0.644	130	0.231	58.66
T0881TS446_1-D1.rsa	30	0.059	0.376	0.564	114	0.263	53.22
T0881TS357_1-D1.rsa	30	0.01	0.035	0.955	193	0.155	55.2
T0881TS275_1-D1.rsa	30	0.05	0.292	0.658	133	0.226	55.69
T0881TS467_4-D1.rsa	30	0.01	0.302	0.688	139	0.216	48.89
T0881TS275_3-D1.rsa	30	0.01	0.297	0.693	140	0.214	56.31
T0881TS287_5-D1.rsa	30	0.035	0.371	0.594	120	0.25	59.53
T0881TS467_1-D1.rsa	30	0.05	0.361	0.589	119	0.252	57.92
T0881TS357_4-D1.rsa	30	0.01	0.059	0.931	188	0.16	55.2
T0881TS026_1-D1.rsa	30	0	0.347	0.653	132	0.227	56.31
T0881TS005_3-D1.rsa	30	0.054	0.401	0.545	110	0.273	58.79
T0881TS026_3-D1.rsa	30	0	0.297	0.703	142	0.211	11.39
T0881TS180_5-D1.rsa	30	0	0.04	0.96	194	0.155	10.77
T0881TS479_4-D1.rsa	28.75	0.04	0.302	0.658	133	0.216	62.5
T0881TS479_5-D1.rsa	28.75	0.045	0.248	0.708	143	0.201	54.08
T0881TS357_5-D1.rsa	28.75	0	0.069	0.931	188	0.153	55.07
T0881TS077_3-D1.rsa	28.75	0.03	0.238	0.733	148	0.194	57.18
T0881TS077_4-D1.rsa	28.75	0.02	0.257	0.723	146	0.197	57.8
T0881TS275_5-D1.rsa	28.75	0.059	0.277	0.663	134	0.215	66.09
T0881TS425_4-D1.rsa	28.75	0.02	0.252	0.728	147	0.196	57.55
T0881TS077_2-D1.rsa	28.75	0.01	0.238	0.752	152	0.189	59.03
T0881TS236_3-D1.rsa	28.75	0.04	0.391	0.569	115	0.25	65.59

T0881TS220_1-D1.rsa	28.75	0.01	0.381	0.609	123	0.234	60.4
T0881TS287_3-D1.rsa	28.75	0.02	0.396	0.584	118	0.244	65.59
T0881TS251_3-D1.rsa	28.75	0.02	0.351	0.629	127	0.226	51.86
T0881TS251_2-D1.rsa	28.75	0.03	0.366	0.604	122	0.236	57.05
T0881TS220_5-D1.rsa	28.75	0.03	0.332	0.639	129	0.223	59.9
T0881TS382_5-D1.rsa	28.75	0.03	0.272	0.698	141	0.204	60.27
T0881TS345_4-D1.rsa	28.75	0.064	0.401	0.535	108	0.266	61.63
T0881TS464_1-D1.rsa	28.75	0.015	0.248	0.738	149	0.193	51.98
T0881TS016_1-D1.rsa	28.75	0.03	0.282	0.688	139	0.207	49.63
T0881TS119_1-D1.rsa	28.75	0.01	0.267	0.723	146	0.197	55.69
T0881TS452_2-D1.rsa	28.75	0.03	0.351	0.619	125	0.23	64.6
T0881TS467_3-D1.rsa	28.75	0.025	0.302	0.673	136	0.211	49.75
T0881TS026_4-D1.rsa	28.75	0	0.287	0.713	144	0.2	10.64
T0881TS359_4-D1.rsa	27.5	0.01	0.337	0.653	132	0.208	60.02
T0881TS275_2-D1.rsa	27.5	0.015	0.297	0.688	139	0.198	55.32
T0881TS220_4-D1.rsa	27.5	0.025	0.342	0.634	128	0.215	59.9
T0881TS452_1-D1.rsa	27.5	0	0.356	0.644	130	0.212	54.08
T0881TS405_2-D1.rsa	27.5	0.02	0.351	0.629	127	0.217	61.14
T0881TS180_1-D1.rsa	27.5	0	0.272	0.728	147	0.187	11.88
T0881TS464_4-D1.rsa	27.5	0.015	0.243	0.743	150	0.183	51.98
T0881TS357_3-D1.rsa	27.5	0.01	0.084	0.906	183	0.15	55.07
T0881TS425_3-D1.rsa	27.5	0.02	0.257	0.723	146	0.188	59.78
T0881TS444_1-D1.rsa	27.5	0	0.332	0.668	135	0.204	50.37
T0881TS258_3-D1.rsa	27.5	0.01	0.332	0.658	133	0.207	55.82
T0881TS345_2-D1.rsa	27.5	0.025	0.366	0.609	123	0.224	60.27
T0881TS251_4-D1.rsa	27.5	0.05	0.337	0.614	124	0.222	63.74
T0881TS467_2-D1.rsa	27.5	0.04	0.297	0.663	134	0.205	49.63
T0881TS345_1-D1.rsa	27.5	0.04	0.347	0.614	124	0.222	61.76
T0881TS284_3-D1.rsa	26.25	0.01	0.347	0.644	130	0.202	58.17
T0881TS425_5-D1.rsa	26.25	0.02	0.233	0.748	151	0.174	58.17
T0881TS183_5-D1.rsa	26.25	0.02	0.307	0.673	136	0.193	61.76
T0881TS183_2-D1.rsa	26.25	0.025	0.248	0.728	147	0.179	59.16
T0881TS287_4-D1.rsa	26.25	0.02	0.332	0.649	131	0.2	65.59
T0881TS495_3-D1.rsa	26.25	0.025	0.327	0.649	131	0.2	61.51
T0881TS425_2-D1.rsa	26.25	0.01	0.267	0.723	146	0.18	58.66
T0881TS284_2-D1.rsa	26.25	0.02	0.356	0.624	126	0.208	59.78
T0881TS236_4-D1.rsa	26.25	0.02	0.342	0.639	129	0.203	63.86
T0881TS236_5-D1.rsa	26.25	0	0.317	0.683	138	0.19	64.6
T0881TS250_5-D1.rsa	26.25	0.03	0.342	0.629	127	0.207	64.6
T0881TS236_1-D1.rsa	26.25	0.045	0.401	0.554	112	0.234	65.72
T0881TS382_3-D1.rsa	26.25	0.03	0.243	0.728	147	0.179	58.79
T0881TS077_1-D1.rsa	26.25	0.02	0.252	0.728	147	0.179	59.03
T0881TS005_1-D1.rsa	26.25	0.02	0.371	0.609	123	0.213	63.49
T0881TS005_4-D1.rsa	26.25	0.03	0.342	0.629	127	0.207	68.44
T0881TS382_4-D1.rsa	26.25	0.04	0.243	0.718	145	0.181	58.54
T0881TS446_2-D1.rsa	26.25	0.035	0.376	0.589	119	0.221	52.1
T0881TS005_2-D1.rsa	26.25	0.01	0.401	0.589	119	0.221	62.87

T0881TS430_4-D1.rsa	26.25	0.03	0.292	0.678	137	0.192	57.43
T0881TS287_1-D1.rsa	26.25	0.03	0.396	0.574	116	0.226	66.46
T0881TS005_5-D1.rsa	26.25	0.059	0.351	0.589	119	0.221	17.33
T0881TS479_1-D1.rsa	25	0.02	0.332	0.649	131	0.191	62.5
T0881TS495_4-D1.rsa	25	0.03	0.371	0.599	121	0.207	62.25
T0881TS183_1-D1.rsa	25	0.01	0.213	0.777	157	0.159	62.13
T0881TS359_5-D1.rsa	25	0.035	0.297	0.668	135	0.185	54.58
T0881TS452_5-D1.rsa	25	0.01	0.366	0.624	126	0.198	12.01
T0881TS382_1-D1.rsa	25	0.035	0.302	0.663	134	0.187	59.03
T0881TS405_1-D1.rsa	25	0	0.366	0.634	128	0.195	61.02
T0881TS220_3-D1.rsa	25	0.03	0.312	0.658	133	0.188	58.66
T0881TS258_2-D1.rsa	25	0.02	0.312	0.668	135	0.185	56.56
T0881TS048_1-D1.rsa	25	0.03	0.376	0.594	120	0.208	69.43
T0881TS359_2-D1.rsa	23.75	0	0.337	0.663	134	0.177	54.7
T0881TS479_3-D1.rsa	23.75	0.02	0.312	0.668	135	0.176	54.83
T0881TS359_1-D1.rsa	23.75	0.015	0.361	0.624	126	0.188	54.95
T0881TS077_5-D1.rsa	23.75	0.02	0.248	0.733	148	0.16	56.68
T0881TS479_2-D1.rsa	23.75	0.01	0.347	0.644	130	0.183	57.92
T0881TS444_5-D1.rsa	23.75	0.01	0.322	0.668	135	0.176	50.87
T0881TS313_2-D1.rsa	23.75	0.02	0.267	0.713	144	0.165	56.68
T0881TS345_3-D1.rsa	23.75	0.03	0.401	0.569	115	0.207	57.05
T0881TS313_5-D1.rsa	23.75	0.02	0.267	0.713	144	0.165	56.19
T0881TS421_2-D1.rsa	23.75	0	0.173	0.827	167	0.142	58.91
T0881TS495_1-D1.rsa	23.75	0.01	0.332	0.658	133	0.179	60.02
T0881TS250_4-D1.rsa	23.75	0.04	0.371	0.589	119	0.2	64.85
T0881TS183_3-D1.rsa	23.75	0.025	0.238	0.738	149	0.159	54.95
T0881TS250_2-D1.rsa	23.75	0.05	0.337	0.614	124	0.192	64.73
T0881TS444_4-D1.rsa	23.75	0.02	0.361	0.619	125	0.19	55.69
T0881TS258_1-D1.rsa	23.75	0.02	0.332	0.649	131	0.181	56.81
T0881TS313_3-D1.rsa	22.5	0.02	0.272	0.708	143	0.157	56.19
T0881TS421_1-D1.rsa	22.5	0.02	0.272	0.708	143	0.157	54.7
T0881TS444_3-D1.rsa	22.5	0	0.356	0.644	130	0.173	55.32
T0881TS425_1-D1.rsa	22.5	0.01	0.272	0.718	145	0.155	57.55
T0881TS382_2-D1.rsa	22.5	0.04	0.312	0.649	131	0.172	58.66
T0881TS313_4-D1.rsa	22.5	0.03	0.267	0.703	142	0.158	56.44
T0881TS250_3-D1.rsa	22.5	0.04	0.366	0.594	120	0.188	65.47
T0881TS313_1-D1.rsa	22.5	0.02	0.267	0.713	144	0.156	56.31
T0881TS250_1-D1.rsa	22.5	0.04	0.332	0.629	127	0.177	64.23
T0881TS452_4-D1.rsa	22.5	0.01	0.163	0.827	167	0.135	9.41
T0881TS444_2-D1.rsa	21.25	0	0.307	0.693	140	0.152	52.1
T0881TS258_5-D1.rsa	21.25	0.02	0.307	0.673	136	0.156	56.19
T0881TS495_5-D1.rsa	20	0.05	0.297	0.653	132	0.152	60.89
T0881TS251_5-D1.rsa	20	0	0.342	0.658	133	0.15	58.17
T0881TS258_4-D1.rsa	20	0.035	0.332	0.634	128	0.156	56.19
T0796TS335_1-D1.rsa	91.549	0.554	0	0.446	75	1.221	14.42
T0796TS160_2-D1.rsa	75	0	0.366	0.634	83	0.904	18.32
T0796TS038_1-D1.rsa	71.429	0.031	0.023	0.947	124	0.576	15.27

T0796TS454_5-D1.rsa	65	0	0.254	0.746	85	0.765	17.76
T0796TS492_2-D1.rsa	62.791	0.201	0	0.799	107	0.587	38.43
T0796TS263_4-D1.rsa	61.538	0.291	0.139	0.57	94	0.655	74.68
T0796TS277_3-D1.rsa	59.091	0.095	0.28	0.625	185	0.319	21.11
T0796TS268_5-D1.rsa	58.333	0.065	0.463	0.472	51	1.144	45.83
T0796TS335_4-D1.rsa	57.895	0.225	0.018	0.757	84	0.689	35.36
T0796TS156_4-D1.rsa	57.692	0.297	0.164	0.539	89	0.648	62.34
T0796TS381_4-D1.rsa	57.143	0	0.328	0.672	88	0.649	15.08
T0796TS410_5-D1.rsa	56.338	0.411	0.131	0.458	77	0.732	19.39
T0796TS184_1-D1.rsa	55.814	0.007	0.075	0.918	123	0.454	12.87
T0796TS420_2-D1.rsa	54.167	0	0.491	0.509	55	0.985	62.96
T0796TS268_3-D1.rsa	54.167	0	0.241	0.759	82	0.661	25.23
T0796TS237_3-D1.rsa	53.846	0.121	0.036	0.842	139	0.387	51.42
T0796TS145_3-D1.rsa	53.571	0	0.313	0.687	90	0.595	14.88
T0796TS263_1-D1.rsa	52.113	0.429	0	0.571	96	0.543	16.83
T0796TS133_3-D1.rsa	50.704	0.446	0.012	0.542	91	0.557	18.59
T0796TS420_4-D1.rsa	50	0.037	0.446	0.517	153	0.327	24.58
T0796TS050_1-D1.rsa	50	0.018	0.255	0.727	80	0.625	31.82
T0796TS436_3-D1.rsa	48.837	0.336	0.09	0.575	77	0.634	43.1
T0796TS184_2-D1.rsa	48.611	0.127	0	0.873	186	0.261	9.77
T0796TS145_4-D1.rsa	47.917	0.082	0.164	0.755	83	0.577	48.18
T0796TS216_5-D1.rsa	47.887	0.357	0.155	0.488	82	0.584	16.51
T0796TS414_2-D1.rsa	46.875	0.088	0.257	0.654	89	0.527	16.18
T0796TS133_4-D1.rsa	46.479	0.363	0.125	0.512	86	0.54	23.56
T0796TS420_1-D1.rsa	46.429	0	0.221	0.779	102	0.455	19.47
T0796TS454_1-D1.rsa	46.429	0.214	0.254	0.532	67	0.693	22.82
T0796TS492_4-D1.rsa	46.154	0.048	0.115	0.836	138	0.334	11.08
T0796TS335_2-D1.rsa	46.154	0.255	0.085	0.661	109	0.423	19.78
T0796TS492_1-D1.rsa	45.833	0	0.291	0.709	78	0.588	58.41
T0796TS210_1-D1.rsa	45.313	0.096	0.25	0.654	89	0.509	14.71
T0796TS268_4-D1.rsa	45.07	0.446	0.048	0.506	85	0.53	19.87
T0796TS145_5-D1.rsa	44.186	0.358	0	0.642	86	0.514	40.86
T0796TS133_5-D1.rsa	43.75	0	0.436	0.564	62	0.706	46.59
T0796TS300_1-D1.rsa	43.103	0	0.084	0.916	131	0.329	59.09
T0796TS448_1-D1.rsa	42.188	0.118	0.228	0.654	89	0.474	14.71
T0796TS499_4-D1.rsa	42.105	0.47	0.038	0.492	116	0.363	58.37
T0796TS448_2-D1.rsa	42.105	0.27	0.036	0.694	77	0.547	41.89
T0796TS184_5-D1.rsa	41.86	0.254	0.015	0.731	98	0.427	44.59
T0796TS041_2-D1.rsa	41.86	0.463	0.09	0.448	60	0.698	40.67
T0796TS381_2-D1.rsa	39.535	0.052	0.09	0.858	115	0.344	15.11
T0796TS499_5-D1.rsa	39.344	0.043	0.429	0.529	111	0.354	69.78
T0796TS452_2-D1.rsa	39.286	0	0.145	0.855	112	0.351	20.23
T0796TS184_4-D1.rsa	39.286	0	0.45	0.55	72	0.546	61.07
T0796TS436_1-D1.rsa	39.286	0	0.26	0.74	97	0.405	15.46
T0796TS216_2-D1.rsa	39.063	0.147	0.103	0.75	102	0.383	14.34
T0796TS410_1-D1.rsa	38.028	0.452	0.077	0.47	79	0.481	37.66
T0796TS171_2-D1.rsa	37.5	0.436	0.015	0.548	250	0.15	21.22

T0796TS436_5-D1.rsa	37.5	0.081	0.446	0.473	140	0.268	47.3
T0796TS452_1-D1.rsa	37.5	0.183	0.27	0.548	69	0.543	60.52
T0796TS448_3-D1.rsa	37.5	0.105	0.158	0.737	84	0.446	33.33
T0796TS479_1-D1.rsa	36.842	0.342	0.063	0.595	66	0.558	57.88
T0796TS251_1-D1.rsa	36.765	0.35	0.082	0.568	138	0.266	65.89
T0796TS011_1-D1.rsa	36.62	0.387	0.137	0.476	80	0.458	41.35
T0796TS277_1-D1.rsa	36.111	0.315	0.085	0.601	128	0.282	73.73
T0796TS038_4-D1.rsa	36.066	0.019	0.414	0.567	119	0.303	74.13
T0796TS117_4-D1.rsa	36	0.029	0.268	0.703	97	0.371	47.65
T0796TS345_4-D1.rsa	34.884	0.358	0.082	0.56	75	0.465	54.48
T0796TS414_5-D1.rsa	34.884	0.328	0	0.672	90	0.388	42.72
T0796TS381_5-D1.rsa	34.884	0.358	0.03	0.612	82	0.425	44.22
T0796TS420_5-D1.rsa	34.884	0.299	0.075	0.627	84	0.415	41.98
T0796TS492_5-D1.rsa	34.211	0.3	0.147	0.553	120	0.285	36.52
T0796TS210_2-D1.rsa	34.211	0.241	0.472	0.287	31	1.104	92.13
T0796TS263_3-D1.rsa	34.211	0.25	0.5	0.25	27	1.267	92.36
T0796TS410_4-D1.rsa	33.684	0.534	0.059	0.407	96	0.351	62.39
T0796TS237_2-D1.rsa	33.663	0.28	0.143	0.576	185	0.182	64.49
T0796TS479_4-D1.rsa	33.333	0.265	0.222	0.513	120	0.278	68.91
T0796TS156_3-D1.rsa	33.333	0.323	0.152	0.525	114	0.292	61.98
T0796TS414_1-D1.rsa	33.333	0.373	0.143	0.484	105	0.317	37.21
T0796TS346_1-D1.rsa	32.632	0.551	0.055	0.394	93	0.351	54.34
T0796TS117_3-D1.rsa	32.558	0.331	0.148	0.521	88	0.37	73.82
T0796TS345_1-D1.rsa	32.558	0.358	0.097	0.545	73	0.446	52.98
T0796TS011_2-D1.rsa	32.5	0.044	0.316	0.64	73	0.445	46.71
T0796TS212_1-D1.rsa	32.5	0	0.439	0.561	64	0.508	42.33
T0796TS277_4-D1.rsa	32.203	0.047	0.271	0.682	146	0.221	57.01
T0796TS420_3-D1.rsa	32	0.572	0	0.428	173	0.185	33.73
T0796TS279_1-D1.rsa	31.944	0.305	0.075	0.62	132	0.242	64.47
T0796TS345_3-D1.rsa	31.897	0.625	0	0.375	96	0.332	57.72
T0796TS011_5-D1.rsa	31.579	0.445	0.059	0.496	117	0.27	53.39
T0796TS008_1-D1.rsa	31.25	0.018	0.255	0.727	80	0.391	35.68
T0796TS479_2-D1.rsa	30.857	0.54	0	0.46	186	0.166	32.24
T0796TS228_4-D1.rsa	30.822	0.394	0.073	0.534	198	0.156	76.09
T0796TS008_2-D1.rsa	30.476	0.35	0.179	0.47	110	0.277	70.62
T0796TS008_5-D1.rsa	30.233	0.355	0.201	0.444	75	0.403	73.97
T0796TS335_3-D1.rsa	30.233	0.337	0.154	0.509	86	0.352	63.46
T0796TS216_4-D1.rsa	30	0.018	0.228	0.754	86	0.349	47.81
T0796TS410_3-D1.rsa	30	0.07	0.123	0.807	92	0.326	20.18
T0796TS414_3-D1.rsa	29.577	0.327	0	0.673	113	0.262	18.11
T0796TS349_3-D1.rsa	29.524	0.286	0.205	0.509	119	0.248	71.05
T0796TS499_2-D1.rsa	29.524	0.35	0.184	0.466	109	0.271	68.8
T0796TS210_5-D1.rsa	29.508	0.024	0.429	0.548	115	0.257	72.39
T0796TS479_3-D1.rsa	29.412	0.399	0.074	0.527	128	0.23	60.56
T0796TS228_2-D1.rsa	29.293	0.233	0.205	0.563	162	0.181	63.8
T0796TS300_2-D1.rsa	29	0.394	0.108	0.498	125	0.232	83.17
T0796TS268_1-D1.rsa	28.947	0.231	0.157	0.611	66	0.439	63.89

T0796TS349_2-D1.rsa	28.713	0.308	0.156	0.536	172	0.167	75.86
T0796TS160_5-D1.rsa	28.713	0.209	0.115	0.676	217	0.132	36.84
T0796TS210_3-D1.rsa	28.571	0.167	0.333	0.5	63	0.454	48.02
T0796TS145_2-D1.rsa	28.448	0.617	0	0.383	98	0.29	57.62
T0796TS073_2-D1.rsa	28.169	0.464	0.161	0.375	63	0.447	60.1
T0796TS479_5-D1.rsa	28	0.454	0.116	0.43	108	0.259	82.97
T0796TS300_5-D1.rsa	27.941	0.354	0.107	0.539	131	0.213	68.44
T0796TS436_4-D1.rsa	27.907	0.296	0.183	0.521	88	0.317	72.63
T0796TS117_1-D1.rsa	27.907	0.302	0.201	0.497	84	0.332	72.19
T0796TS452_3-D1.rsa	27.907	0.302	0.195	0.503	85	0.328	71.15
T0796TS160_1-D1.rsa	27.907	0.358	0.045	0.597	80	0.349	52.98
T0796TS171_1-D1.rsa	27.891	0.378	0.131	0.491	183	0.152	77.92
T0796TS381_1-D1.rsa	27.5	0	0.158	0.842	96	0.286	35.97
T0796TS133_1-D1.rsa	27.5	0	0.316	0.684	78	0.353	41.89
T0796TS041_4-D1.rsa	27.5	0	0.43	0.57	65	0.423	42.76
T0796TS171_5-D1.rsa	27.397	0.402	0.105	0.493	183	0.15	72.41
T0796TS251_4-D1.rsa	27.273	0.264	0.181	0.556	160	0.17	57.73
T0796TS160_4-D1.rsa	27.273	0.366	0.121	0.513	115	0.237	76.67
T0796TS454_4-D1.rsa	26.923	0.402	0.111	0.486	144	0.187	62.41
T0796TS349_1-D1.rsa	26.857	0.438	0.032	0.53	214	0.126	39.48
T0796TS436_2-D1.rsa	26.744	0.26	0.154	0.586	99	0.27	65.09
T0796TS216_3-D1.rsa	26.744	0.32	0.16	0.521	88	0.304	73.97
T0796TS345_2-D1.rsa	26.667	0.355	0.209	0.436	102	0.261	70.41
T0796TS228_3-D1.rsa	26.543	0.408	0.094	0.498	227	0.117	76.82
T0796TS268_2-D1.rsa	26.316	0.25	0.5	0.25	27	0.975	93.75
T0796TS300_4-D1.rsa	26	0.414	0.12	0.466	117	0.222	86.16
T0796TS345_5-D1.rsa	25.862	0.617	0	0.383	98	0.264	65.14
T0796TS349_5-D1.rsa	25.253	0.267	0.226	0.507	146	0.173	70.4
T0796TS171_3-D1.rsa	25.17	0.389	0.097	0.515	192	0.131	81.74
T0796TS156_5-D1.rsa	25	0.383	0.107	0.51	124	0.202	67
T0796TS263_5-D1.rsa	24.571	0.406	0.022	0.572	231	0.106	41.77
T0796TS454_3-D1.rsa	24.419	0.272	0.136	0.592	100	0.244	53.85
T0796TS263_2-D1.rsa	24.359	0.365	0.132	0.503	149	0.163	59.81
T0796TS452_5-D1.rsa	24	0.065	0.37	0.565	78	0.308	68.3
T0796TS145_1-D1.rsa	24	0.029	0.384	0.587	81	0.296	63.04
T0796TS335_5-D1.rsa	24	0.065	0.333	0.601	83	0.289	67.75
T0796TS038_2-D1.rsa	23.529	0.358	0.086	0.556	135	0.174	68.33
T0796TS499_3-D1.rsa	23.077	0.378	0.135	0.486	144	0.16	56.42
T0796TS251_5-D1.rsa	22.857	0.46	0.047	0.493	199	0.115	34.65
T0796TS237_5-D1.rsa	22.772	0.265	0.137	0.598	192	0.119	73.75
T0796TS038_3-D1.rsa	22.222	0.253	0.219	0.528	152	0.146	70.23
T0796TS251_2-D1.rsa	19.767	0.389	0.074	0.537	152	0.13	77.39
T0796TS251_3-D1.rsa	19.767	0.417	0.113	0.47	133	0.149	83.3
T0796TS237_4-D1.rsa	18.421	0.343	0.117	0.539	179	0.103	93.75
T0796TS300_3-D1.rsa	16.949	0.009	0.336	0.654	140	0.121	65.42
T0796TS038_5-D1.rsa	16.279	0.392	0.117	0.491	139	0.117	82.16
T0796TS228_1-D1.rsa	16.154	0.26	0.223	0.517	237	0.068	75.83

T0796TS499_1-D1.rsa	14.474	0.34	0.117	0.542	180	0.08	98.87
T0796TS184_3-D1.rsa	14.035	0.457	0.078	0.466	102	0.138	81.16
T0796TS041_5-D1.rsa	13.846	0.266	0.203	0.531	243	0.057	71.61
T0796TS349_4-D1.rsa	13.158	0.307	0.145	0.548	182	0.072	92.92
T0796TS381_3-D1.rsa	12	0.051	0.29	0.659	91	0.132	48.73
T0796TS237_1-D1.rsa	10.638	0.307	0.167	0.525	135	0.079	83.56
T0796TS008_3-D1.rsa	8.511	0.327	0.183	0.49	126	0.068	81.23
T0871TS321_1-D1.rsa	72.059	0.254	0.078	0.668	213	0.338	8.23
T0871TS321_3-D1.rsa	72.059	0.257	0.072	0.671	214	0.337	7.21
T0871TS464_5-D1.rsa	70.588	0.176	0.006	0.818	261	0.27	8.07
T0871TS321_2-D1.rsa	70.588	0.257	0.107	0.636	203	0.348	7.92
T0871TS321_5-D1.rsa	69.118	0.26	0.085	0.655	209	0.331	7.45
T0871TS321_4-D1.rsa	66.176	0.257	0.094	0.649	207	0.32	9.01
T0871TS464_2-D1.rsa	58.824	0.122	0.006	0.871	278	0.212	6.35
T0871TS434_5-D1.rsa	50	0.282	0	0.718	229	0.218	9.88
T0871TS451_2-D1.rsa	45.588	0.273	0.038	0.69	220	0.207	15.2
T0871TS451_4-D1.rsa	45.588	0.273	0.013	0.715	228	0.2	12.38
T0871TS451_1-D1.rsa	42.647	0.27	0.031	0.699	223	0.191	13.64
T0871TS455_5-D1.rsa	39.706	0.144	0.05	0.806	257	0.154	7.92
T0871TS451_3-D1.rsa	39.706	0.273	0.013	0.715	228	0.174	12.07
T0871TS451_5-D1.rsa	38.235	0.282	0.031	0.687	219	0.175	11.52
T0871TS434_3-D1.rsa	36.765	0.313	0	0.687	219	0.168	9.33
T0871TS434_2-D1.rsa	35.294	0.288	0	0.712	227	0.155	9.17
T0871TS467_4-D1.rsa	35.294	0.257	0.129	0.614	196	0.18	10.03
T0871TS434_4-D1.rsa	35.294	0.266	0.009	0.724	231	0.153	10.42
T0871TS455_3-D1.rsa	30.882	0.097	0.078	0.824	263	0.117	22.57
T0871TS434_1-D1.rsa	29.412	0.266	0	0.734	234	0.126	10.81
T0871TS455_2-D1.rsa	27.941	0.072	0.056	0.871	278	0.101	27.04
T0871TS180_1-D1.rsa	26.471	0.248	0.201	0.552	176	0.15	29.78
T0871TS180_3-D1.rsa	26.471	0.248	0.122	0.63	201	0.132	33.15
T0871TS479_5-D1.rsa	25	0.273	0.154	0.574	183	0.137	44.12
T0871TS432_3-D1.rsa	25	0.323	0	0.677	216	0.116	14.34
T0871TS180_2-D1.rsa	23.529	0.223	0.163	0.614	196	0.12	37.3
T0871TS183_4-D1.rsa	22.059	0.276	0.11	0.614	196	0.113	44.2
T0871TS432_2-D1.rsa	22.059	0.335	0.019	0.646	206	0.107	19.04
T0871TS452_5-D1.rsa	22.059	0.223	0.15	0.627	200	0.11	44.91
T0871TS432_1-D1.rsa	22.059	0.326	0.025	0.649	207	0.107	17.01
T0871TS119_1-D1.rsa	22.059	0.172	0.144	0.683	218	0.101	53.92
T0871TS405_1-D1.rsa	20.588	0.219	0.144	0.636	203	0.101	51.41
T0871TS251_3-D1.rsa	20.588	0.216	0.135	0.649	207	0.099	50.47
T0871TS444_5-D1.rsa	20.588	0.285	0.179	0.536	171	0.12	43.34
T0871TS349_1-D1.rsa	20.588	0.172	0.144	0.683	218	0.094	53.92
T0871TS236_1-D1.rsa	19.118	0.276	0.132	0.592	189	0.101	55.17
T0871TS313_2-D1.rsa	19.118	0.172	0.144	0.683	218	0.088	53.37
T0871TS287_2-D1.rsa	19.118	0.251	0.135	0.614	196	0.098	54.55
T0871TS467_5-D1.rsa	19.118	0.16	0.157	0.683	218	0.088	35.89
T0871TS444_4-D1.rsa	19.118	0.282	0.166	0.552	176	0.109	45.3

T0871TS405_5-D1.rsa	19.118	0.241	0.132	0.627	200	0.096	53.37
T0871TS313_1-D1.rsa	19.118	0.172	0.147	0.68	217	0.088	53.53
T0871TS455_4-D1.rsa	19.118	0.16	0.078	0.762	243	0.079	26.65
T0871TS444_3-D1.rsa	19.118	0.276	0.135	0.589	188	0.102	43.65
T0871TS005_4-D1.rsa	19.118	0.32	0.135	0.545	174	0.11	60.5
T0871TS452_3-D1.rsa	17.647	0.263	0.135	0.602	192	0.092	45.38
T0871TS313_5-D1.rsa	17.647	0.172	0.144	0.683	218	0.081	53.68
T0871TS287_3-D1.rsa	17.647	0.238	0.15	0.611	195	0.09	54
T0871TS345_3-D1.rsa	17.647	0.266	0.16	0.574	183	0.096	52.51
T0871TS077_3-D1.rsa	17.647	0.169	0.15	0.68	217	0.081	56.03
T0871TS407_3-D1.rsa	17.647	0.226	0.138	0.636	203	0.087	49.37
T0871TS464_4-D1.rsa	17.647	0.251	0.166	0.583	186	0.095	57.29
T0871TS180_4-D1.rsa	17.647	0.166	0.132	0.702	224	0.079	24.06
T0871TS275_5-D1.rsa	17.647	0.229	0.147	0.624	199	0.089	55.09
T0871TS479_1-D1.rsa	17.647	0.27	0.119	0.611	195	0.09	59.17
T0871TS467_3-D1.rsa	17.647	0.163	0.132	0.705	225	0.078	46.4
T0871TS345_2-D1.rsa	16.176	0.248	0.157	0.596	190	0.085	51.72
T0871TS467_2-D1.rsa	16.176	0.166	0.125	0.708	226	0.072	45.06
T0871TS479_3-D1.rsa	16.176	0.317	0.141	0.542	173	0.094	55.49
T0871TS026_1-D1.rsa	16.176	0.191	0.15	0.658	210	0.077	50.23
T0871TS444_2-D1.rsa	16.176	0.263	0.135	0.602	192	0.084	47.34
T0871TS026_4-D1.rsa	16.176	0.201	0.125	0.674	215	0.075	49.45
T0871TS251_5-D1.rsa	16.176	0.216	0.138	0.646	206	0.079	53.6
T0871TS425_2-D1.rsa	16.176	0.163	0.141	0.696	222	0.073	56.82
T0871TS479_2-D1.rsa	16.176	0.307	0.129	0.564	180	0.09	57.37
T0871TS452_2-D1.rsa	16.176	0.241	0.141	0.618	197	0.082	49.69
T0871TS407_2-D1.rsa	16.176	0.266	0.129	0.605	193	0.084	49.92
T0871TS026_3-D1.rsa	16.176	0.176	0.141	0.683	218	0.074	45.45
T0871TS425_5-D1.rsa	16.176	0.191	0.138	0.671	214	0.076	56.35
T0871TS464_3-D1.rsa	16.176	0.276	0.141	0.583	186	0.087	57.45
T0871TS357_1-D1.rsa	16.176	0.172	0.044	0.784	250	0.065	54.23
T0871TS313_4-D1.rsa	16.176	0.172	0.147	0.68	217	0.075	54
T0871TS357_3-D1.rsa	16.176	0.188	0	0.812	259	0.062	54.23
T0871TS005_1-D1.rsa	16.176	0.31	0.169	0.52	166	0.097	60.34
T0871TS357_4-D1.rsa	16.176	0.185	0.038	0.777	248	0.065	54.08
T0871TS357_5-D1.rsa	16.176	0.172	0	0.828	264	0.061	54.47
T0871TS251_4-D1.rsa	14.706	0.213	0.144	0.643	205	0.072	45.69
T0871TS275_1-D1.rsa	14.706	0.238	0.179	0.583	186	0.079	57.68
T0871TS407_4-D1.rsa	14.706	0.27	0.132	0.599	191	0.077	47.41
T0871TS444_1-D1.rsa	14.706	0.232	0.138	0.63	201	0.073	46.47
T0871TS236_2-D1.rsa	14.706	0.238	0.15	0.611	195	0.075	53.92
T0871TS077_2-D1.rsa	14.706	0.188	0.144	0.668	213	0.069	56.27
T0871TS236_3-D1.rsa	14.706	0.254	0.141	0.605	193	0.076	53.29
T0871TS313_3-D1.rsa	14.706	0.172	0.147	0.68	217	0.068	53.76
T0871TS026_2-D1.rsa	14.706	0.191	0.125	0.683	218	0.067	54.39
T0871TS275_2-D1.rsa	14.706	0.219	0.176	0.605	193	0.076	55.96
T0871TS005_5-D1.rsa	14.706	0.313	0.147	0.539	172	0.085	59.48

T0871TS251_2-D1.rsa	14.706	0.245	0.141	0.614	196	0.075	43.65
T0871TS407_5-D1.rsa	14.706	0.232	0.129	0.639	204	0.072	49.06
T0871TS048_1-D1.rsa	14.706	0.295	0.135	0.571	182	0.081	54.94
T0871TS183_1-D1.rsa	14.706	0.273	0.122	0.605	193	0.076	58.78
T0871TS236_4-D1.rsa	14.706	0.263	0.129	0.608	194	0.076	53.84
T0871TS455_1-D1.rsa	14.706	0.113	0.103	0.784	250	0.059	33.62
T0871TS479_4-D1.rsa	13.235	0.285	0.088	0.627	200	0.066	51.49
T0871TS452_1-D1.rsa	13.235	0.241	0.141	0.618	197	0.067	52.27
T0871TS425_3-D1.rsa	13.235	0.176	0.125	0.699	223	0.059	55.56
T0871TS425_1-D1.rsa	13.235	0.166	0.147	0.687	219	0.06	55.09
T0871TS287_5-D1.rsa	13.235	0.254	0.144	0.602	192	0.069	53.29
T0871TS005_2-D1.rsa	13.235	0.292	0.141	0.567	181	0.073	60.42
T0871TS407_1-D1.rsa	13.235	0.273	0.113	0.614	196	0.068	49.45
T0871TS251_1-D1.rsa	13.235	0.194	0.141	0.665	212	0.062	50.08
T0871TS382_5-D1.rsa	13.235	0.216	0.141	0.643	205	0.065	50.86
T0871TS220_2-D1.rsa	13.235	0.329	0.16	0.511	163	0.081	56.98
T0871TS345_4-D1.rsa	13.235	0.245	0.15	0.605	193	0.069	50.7
T0871TS183_3-D1.rsa	11.765	0.317	0.116	0.567	181	0.065	56.03
T0871TS077_1-D1.rsa	11.765	0.191	0.135	0.674	215	0.055	56.51
T0871TS287_1-D1.rsa	11.765	0.226	0.144	0.63	201	0.059	53.29
T0871TS345_5-D1.rsa	11.765	0.238	0.172	0.589	188	0.063	48.43
T0871TS077_4-D1.rsa	11.765	0.185	0.135	0.68	217	0.054	55.33
T0871TS382_2-D1.rsa	11.765	0.213	0.141	0.646	206	0.057	50.86
T0871TS275_4-D1.rsa	11.765	0.219	0.144	0.636	203	0.058	47.26
T0871TS357_2-D1.rsa	11.765	0.166	0.013	0.821	262	0.045	54.15
T0871TS425_4-D1.rsa	11.765	0.194	0.135	0.671	214	0.055	55.09
T0871TS005_3-D1.rsa	11.765	0.301	0.176	0.524	167	0.07	62.7
T0871TS452_4-D1.rsa	11.765	0.216	0.15	0.633	202	0.058	44.04
T0871TS382_1-D1.rsa	11.765	0.223	0.144	0.633	202	0.058	50.86
T0871TS220_4-D1.rsa	11.765	0.329	0.163	0.508	162	0.073	56.03
T0871TS236_5-D1.rsa	11.765	0.219	0.132	0.649	207	0.057	53.21
T0871TS183_5-D1.rsa	10.294	0.216	0.132	0.652	208	0.049	48.75
T0871TS250_5-D1.rsa	10.294	0.266	0.144	0.589	188	0.055	54.31
T0871TS016_1-D1.rsa	10.294	0.266	0.141	0.592	189	0.054	50.23
T0871TS382_3-D1.rsa	10.294	0.204	0.144	0.652	208	0.049	50.94
T0871TS287_4-D1.rsa	10.294	0.232	0.135	0.633	202	0.051	52.82
T0871TS250_3-D1.rsa	10.294	0.266	0.135	0.599	191	0.054	54.23
T0871TS077_5-D1.rsa	10.294	0.166	0.135	0.699	223	0.046	55.25
T0871TS382_4-D1.rsa	10.294	0.219	0.144	0.636	203	0.051	50.78
T0871TS220_1-D1.rsa	10.294	0.32	0.135	0.545	174	0.059	55.33
T0871TS180_5-D1.rsa	10.294	0.257	0	0.743	237	0.043	11.29
T0871TS183_2-D1.rsa	8.824	0.301	0.113	0.586	187	0.047	56.19
T0871TS220_3-D1.rsa	8.824	0.32	0.154	0.527	168	0.053	55.17
T0871TS250_4-D1.rsa	7.353	0.266	0.135	0.599	191	0.038	54
T0871TS250_2-D1.rsa	7.353	0.266	0.138	0.596	190	0.039	53.6
T0871TS250_1-D1.rsa	7.353	0.266	0.135	0.599	191	0.038	53.92
T0871TS464_1-D1.rsa	7.353	0.251	0.154	0.596	190	0.039	57.29

T0871TS275_3-D1.rsa	7.353	0.226	0.182	0.592	189	0.039	52.98
T0871TS345_1-D1.rsa	7.353	0.248	0.182	0.571	182	0.04	50.86
T0871TS220_5-D1.rsa	7.353	0.313	0.147	0.539	172	0.043	56.82
T0801TS110_5-D1.rsa	92.105	0.207	0	0.793	88	1.047	14.41
T0801TS184_1-D1.rsa	88.732	0.53	0.012	0.458	77	1.152	13.78
T0801TS133_4-D1.rsa	84	0.232	0.051	0.717	99	0.848	10.33
T0801TS038_4-D1.rsa	82.813	0.125	0.191	0.684	93	0.89	12.5
T0801TS414_4-D1.rsa	76.923	0.358	0.012	0.63	104	0.74	15.03
T0801TS479_4-D1.rsa	76.316	0.018	0	0.982	109	0.7	13.06
T0801TS160_3-D1.rsa	74.419	0.172	0.097	0.731	98	0.759	17.54
T0801TS133_5-D1.rsa	73.684	0.234	0.036	0.73	81	0.91	19.37
T0801TS011_1-D1.rsa	71.053	0.207	0	0.793	88	0.807	20.95
T0801TS349_1-D1.rsa	67.647	0.325	0	0.675	85	0.796	30.95
T0801TS345_2-D1.rsa	64.706	0.46	0	0.54	68	0.952	27.18
T0801TS160_2-D1.rsa	63.158	0.288	0.081	0.631	70	0.902	34.69
T0801TS237_4-D1.rsa	62.5	0.118	0.118	0.765	104	0.601	37.32
T0801TS499_5-D1.rsa	62.5	0.118	0.184	0.699	95	0.658	38.42
T0801TS228_2-D1.rsa	60.714	0	0.237	0.763	100	0.607	19.47
T0801TS263_1-D1.rsa	60.526	0.288	0.081	0.631	70	0.865	34.23
T0801TS454_2-D1.rsa	60	0.491	0.094	0.415	97	0.619	10.15
T0801TS436_3-D1.rsa	59.615	0.255	0.103	0.642	106	0.562	18.04
T0801TS279_1-D1.rsa	58.333	0	0.556	0.444	48	1.215	71.3
T0801TS277_3-D1.rsa	58.333	0	0.145	0.855	94	0.621	30
T0801TS277_1-D1.rsa	58.333	0	0.546	0.454	49	1.19	67.36
T0801TS436_2-D1.rsa	58.14	0.261	0.03	0.709	95	0.612	36.94
T0801TS452_4-D1.rsa	55.814	0.231	0.104	0.664	89	0.627	31.34
T0801TS216_3-D1.rsa	55.814	0	0.134	0.866	116	0.481	14.93
T0801TS448_4-D1.rsa	55.263	0.144	0	0.856	95	0.582	30.63
T0801TS300_4-D1.rsa	53.846	0.133	0	0.867	143	0.377	11.08
T0801TS410_3-D1.rsa	53.846	0.291	0.048	0.661	109	0.494	49.37
T0801TS268_4-D1.rsa	53.571	0.038	0.359	0.603	79	0.678	26.34
T0801TS038_1-D1.rsa	52.632	0.045	0	0.955	106	0.497	12.84
T0801TS349_4-D1.rsa	51.563	0	0.25	0.75	102	0.506	44.3
T0801TS011_2-D1.rsa	51.163	0.082	0.097	0.821	110	0.465	14.93
T0801TS300_3-D1.rsa	50	0.015	0.243	0.743	101	0.495	42.28
T0801TS145_1-D1.rsa	50	0.364	0.048	0.588	97	0.515	45.89
T0801TS452_5-D1.rsa	50	0.015	0.359	0.626	82	0.61	52.1
T0801TS133_1-D1.rsa	50	0	0.13	0.87	94	0.532	37.73
T0801TS041_4-D1.rsa	48.837	0.134	0.06	0.806	108	0.452	27.05
T0801TS454_1-D1.rsa	48.077	0.261	0.036	0.703	116	0.414	16.46
T0801TS448_5-D1.rsa	47.368	0.56	0	0.44	146	0.324	10.02
T0801TS335_4-D1.rsa	46.429	0	0.458	0.542	71	0.654	58.02
T0801TS008_4-D1.rsa	46.429	0.015	0.115	0.87	114	0.407	39.88
T0801TS263_3-D1.rsa	45.455	0.557	0	0.443	131	0.347	7.09
T0801TS216_1-D1.rsa	44.737	0.072	0	0.928	103	0.434	14.19
T0801TS381_3-D1.rsa	44.186	0.313	0.09	0.597	80	0.552	43.47
T0801TS038_5-D1.rsa	44.186	0.351	0.037	0.612	82	0.539	42.91

T0801TS193_3-D1.rsa	44.186	0.306	0.03	0.664	89	0.496	40.86
T0801TS268_2-D1.rsa	43.836	0.678	0	0.322	82	0.535	47.06
T0801TS454_5-D1.rsa	43.103	0	0.098	0.902	129	0.334	58.92
T0801TS349_5-D1.rsa	42.857	0	0.366	0.634	83	0.516	51.34
T0801TS251_2-D1.rsa	42.857	0	0.267	0.733	96	0.446	58.59
T0801TS410_2-D1.rsa	42.857	0	0.092	0.908	119	0.36	16.6
T0801TS420_4-D1.rsa	42.857	0.167	0.032	0.802	101	0.424	21.23
T0801TS041_3-D1.rsa	41.86	0.358	0.06	0.582	78	0.537	45.9
T0801TS110_1-D1.rsa	41.86	0.396	0.03	0.575	77	0.544	42.72
T0801TS479_3-D1.rsa	41.667	0.045	0.173	0.782	86	0.484	56.36
T0801TS420_2-D1.rsa	41.667	0.091	0.318	0.591	65	0.641	59.09
T0801TS210_3-D1.rsa	41.379	0	0.084	0.916	131	0.316	58.04
T0801TS420_5-D1.rsa	41.071	0.032	0.016	0.952	120	0.342	12.5
T0801TS156_1-D1.rsa	40.625	0.015	0.456	0.529	72	0.564	67.1
T0801TS171_1-D1.rsa	40	0.53	0.059	0.411	97	0.412	51.16
T0801TS448_1-D1.rsa	40	0.508	0.051	0.441	104	0.385	59
T0801TS346_1-D1.rsa	40	0	0.368	0.632	72	0.556	49.34
T0801TS277_5-D1.rsa	39.437	0	0.06	0.94	158	0.25	11.06
T0801TS452_2-D1.rsa	39.286	0	0.389	0.611	80	0.491	58.59
T0801TS216_2-D1.rsa	39.286	0.175	0.071	0.754	95	0.414	25.79
T0801TS156_4-D1.rsa	39.063	0.015	0.353	0.632	86	0.454	71.14
T0801TS117_3-D1.rsa	39.063	0.015	0.382	0.603	82	0.476	71.88
T0801TS300_1-D1.rsa	39.063	0.015	0.39	0.596	81	0.482	55.7
T0801TS499_2-D1.rsa	38.947	0.525	0.055	0.419	99	0.393	59.85
T0801TS345_4-D1.rsa	37.895	0.53	0.059	0.411	97	0.391	54.02
T0801TS133_3-D1.rsa	37.5	0.015	0.441	0.544	74	0.507	76.84
T0801TS210_2-D1.rsa	37.5	0.026	0.254	0.719	82	0.457	24.56
T0801TS268_1-D1.rsa	36.986	0.761	0	0.239	61	0.606	66.37
T0801TS171_3-D1.rsa	36.842	0.521	0.051	0.428	101	0.365	63.03
T0801TS436_1-D1.rsa	36.62	0.095	0.054	0.851	143	0.256	18.91
T0801TS300_5-D1.rsa	36.111	0.39	0.103	0.507	108	0.334	61.93
T0801TS499_3-D1.rsa	36	0.087	0.217	0.696	96	0.375	41.3
T0801TS335_2-D1.rsa	35.938	0.118	0.074	0.809	110	0.327	35.85
T0801TS381_4-D1.rsa	35.938	0.015	0.39	0.596	81	0.444	71.32
T0801TS008_5-D1.rsa	35.938	0.015	0.36	0.625	85	0.423	67.46
T0801TS216_4-D1.rsa	35.616	0.749	0	0.251	64	0.557	59.22
T0801TS335_1-D1.rsa	35.417	0	0.255	0.745	82	0.432	61.36
T0801TS022_5-D1.rsa	35.354	0.222	0.139	0.639	184	0.192	39.41
T0801TS073_1-D1.rsa	35.238	0.299	0.205	0.496	116	0.304	49.25
T0801TS381_2-D1.rsa	35.211	0.268	0.012	0.72	121	0.291	19.55
T0801TS492_2-D1.rsa	35.211	0.44	0	0.56	94	0.375	43.75
T0801TS145_2-D1.rsa	35	0.018	0.237	0.746	85	0.412	42.76
T0801TS171_2-D1.rsa	34.483	0.602	0	0.398	102	0.338	56.15
T0801TS008_2-D1.rsa	34.483	0.594	0	0.406	104	0.332	55.96
T0801TS268_3-D1.rsa	34.483	0.637	0	0.363	93	0.371	54.3
T0801TS110_2-D1.rsa	34.375	0.015	0.456	0.529	72	0.477	70.77
T0801TS193_1-D1.rsa	34.211	0.323	0.166	0.512	111	0.308	36.87

T0801TS237_3-D1.rsa	33.898	0.051	0.304	0.645	138	0.246	22.08
T0801TS277_2-D1.rsa	33.721	0.361	0.207	0.432	73	0.462	68.64
T0801TS011_3-D1.rsa	33.684	0.508	0.055	0.436	103	0.327	59.64
T0801TS345_1-D1.rsa	33.684	0.538	0.055	0.407	96	0.351	60.27
T0801TS073_2-D1.rsa	33.621	0.563	0	0.438	112	0.3	56.54
T0801TS193_2-D1.rsa	33.333	0.332	0.166	0.502	109	0.306	37.1
T0801TS436_4-D1.rsa	32.955	0.078	0.52	0.402	119	0.277	46.79
T0801TS212_1-D1.rsa	32.759	0.594	0	0.406	104	0.315	54.69
T0801TS171_5-D1.rsa	32.558	0.272	0.189	0.538	91	0.358	73.37
T0801TS117_1-D1.rsa	32.558	0.302	0.189	0.509	86	0.379	72.78
T0801TS038_2-D1.rsa	32.558	0.351	0.045	0.604	81	0.402	53.17
T0801TS345_5-D1.rsa	32.558	0.351	0.045	0.604	81	0.402	53.17
T0801TS160_5-D1.rsa	32.5	0	0.105	0.895	102	0.319	50
T0801TS133_2-D1.rsa	32.5	0	0.096	0.904	103	0.316	43.86
T0801TS414_1-D1.rsa	32.5	0.079	0.044	0.877	100	0.325	37.72
T0801TS050_1-D1.rsa	32.456	0.332	0.171	0.498	108	0.301	36.06
T0801TS448_2-D1.rsa	32.381	0.376	0.214	0.41	96	0.337	69.02
T0801TS022_4-D1.rsa	32.203	0.047	0.21	0.743	159	0.203	52.8
T0801TS410_4-D1.rsa	32	0.087	0.341	0.572	79	0.405	56.7
T0801TS156_5-D1.rsa	32	0.087	0.37	0.543	75	0.427	66.85
T0801TS117_2-D1.rsa	31.973	0.273	0.059	0.668	249	0.128	41.94
T0801TS171_4-D1.rsa	31.944	0.474	0.094	0.432	92	0.347	74.75
T0801TS479_2-D1.rsa	31.897	0.59	0	0.41	105	0.304	54.98
T0801TS008_1-D1.rsa	31.897	0.602	0	0.398	102	0.313	54.4
T0801TS251_3-D1.rsa	31.579	0.458	0.055	0.487	115	0.275	58.58
T0801TS452_3-D1.rsa	31.579	0.47	0.059	0.47	111	0.284	55.3
T0801TS420_3-D1.rsa	31.395	0.337	0.154	0.509	86	0.365	56.36
T0801TS156_2-D1.rsa	31.25	0.074	0.265	0.662	90	0.347	62.13
T0801TS011_4-D1.rsa	31.034	0.609	0	0.391	100	0.31	66.31
T0801TS492_1-D1.rsa	31.034	0.656	0	0.344	88	0.353	65.14
T0801TS420_1-D1.rsa	30.986	0.274	0.113	0.613	103	0.301	37.5
T0801TS184_2-D1.rsa	30.986	0.423	0.089	0.488	82	0.378	51.92
T0801TS008_3-D1.rsa	30.882	0.403	0.053	0.543	132	0.234	17.11
T0801TS041_1-D1.rsa	30.526	0.492	0.059	0.449	106	0.288	59.96
T0801TS228_1-D1.rsa	30.476	0.346	0.201	0.453	106	0.288	69.87
T0801TS448_3-D1.rsa	30.476	0.368	0.201	0.432	101	0.302	72.01
T0801TS160_1-D1.rsa	30.233	0.314	0.189	0.497	84	0.36	73.08
T0801TS268_5-D1.rsa	30.172	0.617	0	0.383	98	0.308	55.57
T0801TS228_4-D1.rsa	29.87	0.384	0.116	0.5	112	0.267	70.12
T0801TS237_2-D1.rsa	29.524	0.338	0.218	0.444	104	0.284	69.66
T0801TS041_2-D1.rsa	29.474	0.564	0.055	0.381	90	0.327	62.08
T0801TS436_5-D1.rsa	29.412	0.346	0.091	0.564	137	0.215	65.22
T0801TS011_5-D1.rsa	29.07	0.308	0.183	0.509	86	0.338	73.37
T0801TS110_3-D1.rsa	28.448	0.617	0	0.383	98	0.29	56.54
T0801TS263_2-D1.rsa	28.283	0.243	0.184	0.573	165	0.171	66.15
T0801TS228_5-D1.rsa	28	0.014	0	0.986	136	0.206	13.77
T0801TS492_3-D1.rsa	28	0.022	0.326	0.652	90	0.311	58.51

T0801TS414_2-D1.rsa	28	0.022	0.326	0.652	90	0.311	58.33
T0801TS216_5-D1.rsa	27.907	0.358	0.082	0.56	75	0.372	52.98
T0801TS117_4-D1.rsa	27.619	0.385	0.244	0.372	87	0.317	75.21
T0801TS110_4-D1.rsa	26.744	0.302	0.189	0.509	86	0.311	69.08
T0801TS117_5-D1.rsa	26.286	0.423	0.012	0.564	228	0.115	35.46
T0801TS228_3-D1.rsa	25.714	0.359	0.201	0.44	103	0.25	72.11
T0801TS414_5-D1.rsa	25.714	0.385	0.244	0.372	87	0.296	74.89
T0801TS193_5-D1.rsa	25.581	0.278	0.178	0.544	92	0.278	73.52
T0801TS237_5-D1.rsa	25	0.432	0.107	0.461	112	0.223	71.44
T0801TS022_2-D1.rsa	24.571	0.535	0.022	0.443	179	0.137	49.2
T0801TS251_5-D1.rsa	24.359	0.351	0.139	0.51	151	0.161	51.91
T0801TS206_1-D1.rsa	24	0.454	0.139	0.406	102	0.235	71.81
T0801TS160_4-D1.rsa	24	0.051	0.355	0.594	82	0.293	70.29
T0801TS479_1-D1.rsa	23.377	0.433	0.134	0.433	97	0.241	75.71
T0801TS210_1-D1.rsa	23.158	0.508	0.047	0.445	105	0.221	63.24
T0801TS251_1-D1.rsa	22.059	0.379	0.111	0.51	124	0.178	67.67
T0801TS492_4-D1.rsa	22.059	0.3	0.037	0.663	161	0.137	22.78
T0801TS237_1-D1.rsa	19.298	0.452	0.055	0.493	108	0.179	80.71
T0801TS349_2-D1.rsa	17.544	0.429	0.096	0.475	104	0.169	74.09
T0801TS349_3-D1.rsa	16.883	0.411	0.112	0.478	107	0.158	79.88
T0801TS492_5-D1.rsa	10.638	0.346	0.187	0.467	120	0.089	83.17
T0808TS064_3-D1.rsa	83.333	0.098	0	0.902	267	0.312	6.42
T0808TS263_4-D1.rsa	80.198	0.065	0	0.935	300	0.267	6.7
T0808TS038_5-D1.rsa	78.947	0.124	0	0.876	190	0.416	11.98
T0808TS403_5-D1.rsa	76.563	0.051	0.118	0.831	113	0.678	40.26
T0808TS116_3-D1.rsa	75.41	0.01	0	0.99	208	0.363	7.71
T0808TS317_3_1-D1.rsa	73.714	0.386	0.01	0.604	244	0.302	6.68
T0808TS310_3-D1.rsa	69.318	0	0.034	0.966	286	0.242	6.59
T0808TS391_1-D1.rsa	68.421	0.18	0.072	0.748	83	0.824	23.87
T0808TS340_1-D1.rsa	67.442	0.194	0	0.806	108	0.624	39.37
T0808TS317_5_1-D1.rsa	67.442	0.194	0	0.806	108	0.624	38.81
T0808TS436_3-D1.rsa	67.308	0	0.012	0.988	163	0.413	9.18
T0808TS116_2-D1.rsa	65.385	0	0.091	0.909	150	0.436	11.23
T0808TS169_3-D1.rsa	65.385	0.158	0	0.842	139	0.47	14.08
T0808TS279_1-D1.rsa	64.286	0	0.252	0.748	98	0.656	49.43
T0808TS235_1-D1.rsa	59.615	0.224	0.085	0.691	114	0.523	72.15
T0808TS482_1-D1.rsa	59.615	0.273	0.085	0.642	106	0.562	65.35
T0808TS212_2-D1.rsa	58.333	0	0.583	0.417	45	1.296	69.91
T0808TS391_4-D1.rsa	57.692	0.23	0.127	0.642	106	0.544	77.69
T0808TS454_4-D1.rsa	57.692	0.273	0.127	0.6	99	0.583	75.79
T0808TS008_2-D1.rsa	57.692	0.321	0.145	0.533	88	0.656	76.27
T0808TS479_4-D1.rsa	55.769	0.261	0.024	0.715	118	0.473	62.02
T0808TS410_1-D1.rsa	55.769	0.236	0.061	0.703	116	0.481	59.81
T0808TS049_1-D1.rsa	53.947	0.361	0.024	0.614	204	0.264	7.76
T0808TS499_1-D1.rsa	53.409	0.074	0.179	0.747	221	0.242	18.41
T0808TS301_2-D1.rsa	52.381	0.218	0.043	0.739	173	0.303	33.87
T0808TS038_1-D1.rsa	52.113	0.036	0.042	0.923	155	0.336	17.47

T0808TS420_4-D1.rsa	51.429	0.355	0.06	0.585	137	0.375	13.14
T0808TS448_5-D1.rsa	50	0.436	0.015	0.548	250	0.2	16.78
T0808TS436_4-D1.rsa	49.6	0.03	0.267	0.702	231	0.215	25.08
T0808TS335_2-D1.rsa	48.276	0	0.105	0.895	128	0.377	60.66
T0808TS282_1-D1.rsa	48.276	0	0.112	0.888	127	0.38	58.74
T0808TS452_4-D1.rsa	46.552	0	0.035	0.965	138	0.337	56.99
T0808TS454_5-D1.rsa	46.552	0	0.175	0.825	118	0.395	63.99
T0808TS346_1-D1.rsa	44.828	0	0.077	0.923	132	0.34	44.93
T0808TS358_5-D1.rsa	44.828	0	0.084	0.916	131	0.342	42.31
T0808TS479_2-D1.rsa	44.318	0.068	0.358	0.574	170	0.261	23.65
T0808TS452_5-D1.rsa	43.103	0	0.098	0.902	129	0.334	58.04
T0808TS008_3-D1.rsa	43.103	0	0.203	0.797	114	0.378	41.08
T0808TS204_1-D1.rsa	42.105	0.295	0.101	0.604	131	0.321	59.91
T0808TS414_5-D1.rsa	41.379	0.014	0.196	0.79	113	0.366	76.22
T0808TS038_4-D1.rsa	41.379	0	0.14	0.86	123	0.336	57.52
T0808TS065_1_1-D1.rsa	41.379	0	0.182	0.818	117	0.354	58.57
T0808TS448_1-D1.rsa	40.404	0.174	0.125	0.701	202	0.2	50.69
T0808TS483_1-D1.rsa	40.404	0.198	0.122	0.681	196	0.206	11.98
T0808TS414_4-D1.rsa	38.889	0.319	0.061	0.62	132	0.295	72.46
T0808TS437_1-D1.rsa	38.889	0.329	0.075	0.596	127	0.306	73.98
T0808TS335_1-D1.rsa	38.889	0.329	0.103	0.568	121	0.321	70.69
T0808TS425_4-D1.rsa	38.636	0.074	0.307	0.618	183	0.211	48.48
T0808TS296_2-D1.rsa	38.636	0.047	0.47	0.483	143	0.27	34.63
T0808TS499_4-D1.rsa	38.4	0.024	0.261	0.714	235	0.163	24.77
T0808TS448_2-D1.rsa	38.4	0.012	0.313	0.675	222	0.173	25.15
T0808TS169_5-D1.rsa	37.714	0.505	0.04	0.455	184	0.205	24.2
T0808TS008_1-D1.rsa	37.5	0.037	0.148	0.815	88	0.426	62.04
T0808TS410_2-D1.rsa	36.842	0.24	0.175	0.585	127	0.29	62.56
T0808TS420_3-D1.rsa	36.842	0.458	0.059	0.483	114	0.323	56.14
T0808TS434_1-D1.rsa	36.364	0.064	0.345	0.591	175	0.208	25.17
T0808TS296_3-D1.rsa	35.965	0.29	0.166	0.544	118	0.305	51.96
T0808TS067_3-D1.rsa	35.616	0.369	0	0.631	161	0.221	45.59
T0808TS425_1-D1.rsa	35.294	0.214	0.123	0.663	161	0.219	11.89
T0808TS414_1-D1.rsa	35.227	0.041	0.426	0.534	158	0.223	44.26
T0808TS157_3-D1.rsa	35.2	0	0.337	0.663	218	0.161	42.63
T0808TS438_1-D1.rsa	35.088	0.253	0.157	0.59	128	0.274	64.98
T0808TS050_1-D1.rsa	35.088	0.235	0.203	0.562	122	0.288	64.86
T0808TS391_3-D1.rsa	34.884	0.276	0.082	0.642	86	0.406	23.88
T0808TS326_3-D1.rsa	34.653	0.287	0.168	0.545	175	0.198	64.72
T0808TS044_4-D1.rsa	34.426	0.024	0.39	0.586	123	0.28	72.64
T0808TS044_3-D1.rsa	34.375	0.015	0.309	0.676	92	0.374	71.88
T0808TS251_2-D1.rsa	34.247	0.369	0.092	0.539	200	0.171	62.12
T0808TS097_5-D1.rsa	34.247	0.388	0	0.612	156	0.22	42.35
T0808TS237_2-D1.rsa	33.333	0.249	0.212	0.539	117	0.285	65.32
T0808TS345_5-D1.rsa	33.143	0.431	0.03	0.54	218	0.152	26.24
T0808TS439_5-D1.rsa	32.877	0.356	0.113	0.531	197	0.167	70.23
T0808TS011_4-D1.rsa	32.8	0.006	0.28	0.714	235	0.14	39.21

T0808TS206_1-D1.rsa	32.787	0	0.1	0.9	189	0.173	50.75
T0808TS156_3-D1.rsa	32.787	0.029	0.424	0.548	115	0.285	72.64
T0808TS212_1-D1.rsa	32.787	0.024	0.424	0.552	116	0.283	73.13
T0808TS345_1-D1.rsa	32.203	0.042	0.215	0.743	159	0.203	52.57
T0808TS483_4-D1.rsa	32.203	0.061	0.252	0.687	147	0.219	39.14
T0808TS445_5-D1.rsa	32	0.45	0.04	0.51	206	0.155	35.58
T0808TS454_1-D1.rsa	32	0.015	0.429	0.556	183	0.175	49.77
T0808TS237_3-D1.rsa	31.818	0.057	0.311	0.632	187	0.17	45.02
T0808TS420_1-D1.rsa	31.313	0.208	0.188	0.604	174	0.18	66.93
T0808TS038_3-D1.rsa	31.169	0.397	0.094	0.509	114	0.273	79.05
T0808TS338_4-D1.rsa	31.148	0	0.424	0.576	121	0.257	73.88
T0808TS381_2-D1.rsa	31.034	0	0.203	0.797	114	0.272	81.29
T0808TS381_1-D1.rsa	31	0.426	0.108	0.466	117	0.265	82.97
T0808TS345_2-D1.rsa	30.682	0.057	0.331	0.611	181	0.17	49.66
T0808TS228_5-D1.rsa	30.612	0.365	0.102	0.534	199	0.154	83.06
T0808TS333_3-D1.rsa	30.556	0.366	0.085	0.549	117	0.261	70.69
T0808TS216_5-D1.rsa	30.508	0	0.318	0.682	146	0.209	64.95
T0808TS445_2-D1.rsa	30.508	0	0.29	0.71	152	0.201	65.54
T0808TS414_2-D1.rsa	30.508	0	0.294	0.706	151	0.202	62.27
T0808TS132_3-D1.rsa	30.508	0.009	0.313	0.678	145	0.21	62.03
T0808TS097_3-D1.rsa	30.476	0.312	0.184	0.504	118	0.258	69.98
T0808TS216_1-D1.rsa	30.4	0.012	0.383	0.605	199	0.153	56.23
T0808TS430_1-D1.rsa	30.233	0.313	0.149	0.537	72	0.42	30.04
T0808TS145_1-D1.rsa	30.137	0.388	0.1	0.512	190	0.159	78.13
T0808TS328_2-D1.rsa	29.932	0.332	0.115	0.552	206	0.145	67.71
T0808TS439_2-D1.rsa	29.545	0.078	0.297	0.625	185	0.16	52.2
T0808TS067_2-D1.rsa	29.545	0.091	0.331	0.578	171	0.173	51.27
T0808TS156_4-D1.rsa	29.508	0.029	0.39	0.581	122	0.242	63.18
T0808TS420_2-D1.rsa	29.508	0.014	0.429	0.557	117	0.252	64.8
T0808TS160_4-D1.rsa	29.452	0.391	0.1	0.509	189	0.156	75.68
T0808TS282_3-D1.rsa	29.452	0.394	0.102	0.504	187	0.157	74.66
T0808TS204_3-D1.rsa	29.452	0.399	0.1	0.501	186	0.158	72.96
T0808TS362_3-D1.rsa	29.412	0.325	0.045	0.63	153	0.192	61.22
T0808TS290_5-D1.rsa	29.412	0.358	0.074	0.568	138	0.213	60.33
T0808TS454_2-D1.rsa	29.293	0.198	0.219	0.583	168	0.174	49.83
T0808TS340_3-D1.rsa	29	0.39	0.139	0.47	118	0.246	87.85
T0808TS322_1-D1.rsa	29	0.434	0.124	0.442	111	0.261	88.45
T0808TS145_4-D1.rsa	28.8	0.018	0.328	0.653	215	0.134	49.85
T0808TS439_1-D1.rsa	28.571	0.322	0.078	0.601	224	0.128	67.43
T0808TS310_1-D1.rsa	28.571	0.378	0.099	0.523	195	0.147	83.96
T0808TS448_3-D1.rsa	28.571	0.378	0.075	0.547	204	0.14	74.93
T0808TS499_5-D1.rsa	28.571	0.396	0.064	0.54	218	0.131	33.79
T0808TS345_3-D1.rsa	28.283	0.243	0.184	0.573	165	0.171	65.97
T0808TS347_1-D1.rsa	28.283	0.247	0.188	0.566	163	0.174	70.31
T0808TS117_3-D1.rsa	28.283	0.208	0.17	0.622	179	0.158	60.24
T0808TS097_2-D1.rsa	28.283	0.271	0.219	0.51	147	0.192	73.26
T0808TS268_2-D1.rsa	28.205	0.405	0.044	0.551	163	0.173	59.29

T0808TS042_5-D1.rsa	28.082	0.342	0.086	0.571	212	0.132	63.62
T0808TS282_2-D1.rsa	27.941	0.391	0.091	0.519	126	0.222	70.11
T0808TS157_2-D1.rsa	27.941	0.391	0.103	0.506	123	0.227	67.22
T0808TS080_2-D1.rsa	27.891	0.357	0.105	0.539	201	0.139	82.85
T0808TS153_1-D1.rsa	27.891	0.378	0.097	0.525	196	0.142	75.42
T0808TS110_3-D1.rsa	27.891	0.383	0.105	0.512	191	0.146	86.18
T0808TS156_1-D1.rsa	27.869	0.014	0.362	0.624	131	0.213	69.53
T0808TS479_1-D1.rsa	27.869	0	0.41	0.59	124	0.225	69.9
T0808TS116_5-D1.rsa	27.869	0.014	0.333	0.652	137	0.203	54.48
T0808TS362_2-D1.rsa	27.429	0.433	0.035	0.532	215	0.128	46.35
T0808TS064_1-D1.rsa	27.429	0.525	0.022	0.453	183	0.15	37.31
T0808TS436_1-D1.rsa	27.397	0.394	0.111	0.496	184	0.149	76.97
T0808TS197_2-D1.rsa	27.273	0.25	0.174	0.576	166	0.164	68.49
T0808TS310_4-D1.rsa	27.273	0.25	0.194	0.556	160	0.17	68.49
T0808TS445_1-D1.rsa	27.273	0.041	0.341	0.618	183	0.149	34.46
T0808TS162_2-D1.rsa	27.273	0.229	0.212	0.559	161	0.169	69.18
T0808TS335_3-D1.rsa	27.273	0.264	0.219	0.517	149	0.183	67.19
T0808TS041_2-D1.rsa	27.273	0.415	0.094	0.491	110	0.248	81.43
T0808TS197_3-D1.rsa	27.211	0.373	0.105	0.523	195	0.14	82.43
T0808TS228_3-D1.rsa	27.211	0.367	0.088	0.544	203	0.134	77.08
T0808TS235_3-D1.rsa	27.211	0.37	0.107	0.523	195	0.14	79.65
T0808TS263_3-D1.rsa	27.211	0.397	0.107	0.496	185	0.147	84.17
T0808TS492_3-D1.rsa	27.2	0.006	0.365	0.629	207	0.131	46.51
T0808TS452_1-D1.rsa	27.119	0	0.313	0.687	147	0.184	63.32
T0808TS333_4-D1.rsa	27	0.406	0.116	0.478	120	0.225	89.04
T0808TS403_4-D1.rsa	27	0.414	0.112	0.474	119	0.227	87.35
T0808TS300_4-D1.rsa	26.974	0.434	0.015	0.55	251	0.107	61.62
T0808TS483_2-D1.rsa	26.923	0.348	0.101	0.551	163	0.165	57.38
T0808TS118_3-D1.rsa	26.923	0.355	0.139	0.507	150	0.179	54.25
T0808TS044_5-D1.rsa	26.923	0.395	0.128	0.476	141	0.191	59.12
T0808TS041_4-D1.rsa	26.857	0.488	0.02	0.493	199	0.135	35.02
T0808TS362_4-D1.rsa	26.733	0.234	0.14	0.626	201	0.133	55.45
T0808TS368_5-D1.rsa	26.712	0.396	0.092	0.512	190	0.141	72
T0808TS235_2-D1.rsa	26.712	0.426	0.113	0.461	171	0.156	75.55
T0808TS439_4-D1.rsa	26.712	0.434	0.111	0.456	169	0.158	74.86
T0808TS425_2-D1.rsa	26.667	0.321	0.171	0.509	119	0.224	62.39
T0808TS436_5-D1.rsa	26.531	0.386	0.102	0.512	191	0.139	86.18
T0808TS263_2-D1.rsa	26.531	0.386	0.102	0.512	191	0.139	85.9
T0808TS042_2-D1.rsa	26.531	0.391	0.107	0.501	187	0.142	86.53
T0808TS277_4-D1.rsa	26.471	0.37	0.07	0.56	136	0.195	69.11
T0808TS282_5-D1.rsa	26.263	0.208	0.146	0.646	186	0.141	70.57
T0808TS216_3-D1.rsa	26.263	0.208	0.174	0.618	178	0.148	60.42
T0808TS454_3-D1.rsa	26.263	0.226	0.205	0.569	164	0.16	69.27
T0808TS008_5-D1.rsa	26.136	0.074	0.409	0.517	153	0.171	29.98
T0808TS333_5-D1.rsa	26	0.355	0.116	0.53	133	0.195	81.97
T0808TS338_2-D1.rsa	26	0.426	0.12	0.454	114	0.228	89.84
T0808TS326_2-D1.rsa	26	0.45	0.112	0.438	110	0.236	89.64

T0808TS328_5-D1.rsa	25.85	0.386	0.105	0.509	190	0.136	88.19
T0808TS118_5-D1.rsa	25.743	0.277	0.143	0.579	186	0.138	74.92
T0808TS381_4-D1.rsa	25.714	0.545	0.027	0.428	173	0.149	49.63
T0808TS358_4-D1.rsa	25.641	0.365	0.128	0.507	150	0.171	57.99
T0808TS381_5-D1.rsa	25.424	0	0.308	0.692	148	0.172	63.55
T0808TS144_3-D1.rsa	25.342	0.407	0.113	0.48	178	0.142	78.88
T0808TS328_3-D1.rsa	25.17	0.378	0.097	0.525	196	0.128	82.64
T0808TS403_1-D1.rsa	25	0.367	0.03	0.602	200	0.125	40.36
T0808TS038_2-D1.rsa	25	0.296	0.062	0.642	156	0.16	59.56
T0808TS144_1-D1.rsa	25	0.358	0.045	0.597	145	0.172	69
T0808TS492_5-D1.rsa	25	0.398	0.12	0.482	121	0.207	90.34
T0808TS044_1-D1.rsa	25	0.402	0.12	0.478	120	0.208	86.25
T0808TS301_5-D1.rsa	25	0.418	0.124	0.458	115	0.217	89.14
T0808TS403_3-D1.rsa	25	0.398	0.139	0.462	116	0.216	89.94
T0808TS056_1-D1.rsa	25	0.358	0.095	0.547	133	0.188	66.11
T0808TS296_1-D1.rsa	24.675	0.424	0.112	0.464	104	0.237	81.07
T0808TS425_3-D1.rsa	24.59	0.033	0.357	0.61	128	0.192	55.47
T0808TS452_2-D1.rsa	24.359	0.429	0.111	0.459	136	0.179	62.41
T0808TS368_3-D1.rsa	24.359	0.382	0.122	0.497	147	0.166	58.42
T0808TS268_3-D1.rsa	24.242	0.191	0.191	0.618	178	0.136	51.04
T0808TS073_1-D1.rsa	24.242	0.212	0.174	0.615	177	0.137	60.33
T0808TS064_4-D1.rsa	24.242	0.24	0.205	0.556	160	0.152	64.5
T0808TS300_2-D1.rsa	24.074	0.414	0.094	0.491	224	0.107	65.78
T0808TS317_4_1-D1.rsa	24	0.502	0	0.498	201	0.119	45.3
T0808TS391_2-D1.rsa	24	0.52	0	0.48	194	0.124	50.56
T0808TS160_3-D1.rsa	24	0.465	0.017	0.517	209	0.115	38.43
T0808TS333_1-D1.rsa	24	0.53	0.027	0.443	179	0.134	49.94
T0808TS067_5-D1.rsa	24	0.43	0.108	0.462	116	0.207	79.38
T0808TS049_3-D1.rsa	23.81	0.397	0.11	0.493	184	0.129	85.21
T0808TS410_4-D1.rsa	23.81	0.391	0.11	0.499	186	0.128	70.9
T0808TS338_1-D1.rsa	23.729	0.023	0.294	0.682	146	0.163	59.46
T0808TS184_3-D1.rsa	23.684	0.39	0.029	0.581	265	0.089	61.18
T0808TS391_5-D1.rsa	23.684	0.268	0.012	0.72	239	0.099	50.38
T0808TS011_1-D1.rsa	23.684	0.328	0.009	0.663	220	0.108	67.92
T0808TS204_5-D1.rsa	23.529	0.379	0.037	0.584	142	0.166	64.89
T0808TS268_5-D1.rsa	23.529	0.362	0.103	0.535	130	0.181	67.56
T0808TS204_2-D1.rsa	23.429	0.505	0.015	0.48	194	0.121	43.69
T0808TS338_3-D1.rsa	23.429	0.493	0.04	0.468	189	0.124	34.78
T0808TS132_5-D1.rsa	23.404	0.292	0.128	0.58	149	0.157	72.86
T0808TS425_5-D1.rsa	23.377	0.402	0.121	0.478	107	0.218	82.5
T0808TS499_3-D1.rsa	23.288	0.402	0.108	0.491	182	0.128	62.12
T0808TS333_2-D1.rsa	23.232	0.247	0.205	0.549	158	0.147	72.4
T0808TS347_5-D1.rsa	23.214	0.198	0.024	0.778	98	0.237	41.67
T0808TS290_2-D1.rsa	23.077	0.409	0.132	0.459	136	0.17	59.29
T0808TS133_1-D1.rsa	22.951	0.014	0.414	0.571	120	0.191	65.17
T0808TS328_4-D1.rsa	22.857	0.196	0	0.804	325	0.07	38.24
T0808TS144_2-D1.rsa	22.368	0.286	0.127	0.587	195	0.115	68.22

T0808TS097_4-D1.rsa	22.286	0.418	0.027	0.554	224	0.099	40.84
T0808TS204_4-D1.rsa	22.286	0.438	0.027	0.535	216	0.103	40.22
T0808TS492_2-D1.rsa	22.286	0.475	0.01	0.515	208	0.107	42.14
T0808TS345_4-D1.rsa	22.286	0.488	0.005	0.507	205	0.109	42.27
T0808TS347_3-D1.rsa	22.286	0.624	0	0.376	152	0.147	34.03
T0808TS184_2-D1.rsa	22.222	0.432	0.081	0.487	222	0.1	69.54
T0808TS296_5-D1.rsa	22.222	0.08	0.069	0.851	245	0.091	37.5
T0808TS184_1-D1.rsa	22.093	0.36	0.113	0.527	149	0.148	72.7
T0808TS381_3-D1.rsa	22.093	0.353	0.127	0.519	147	0.15	72.08
T0808TS133_2-D1.rsa	22.059	0.383	0.111	0.506	123	0.179	67.56
T0808TS132_4-D1.rsa	22.034	0.023	0.187	0.79	169	0.13	64.72
T0808TS162_5-D1.rsa	22.034	0.023	0.243	0.734	157	0.14	59.7
T0808TS067_1-D1.rsa	22.034	0.056	0.182	0.762	163	0.135	54.21
T0808TS335_5-D1.rsa	22.034	0.019	0.332	0.65	139	0.159	65.77
T0808TS358_3-D1.rsa	21.795	0.331	0.101	0.568	168	0.13	53.04
T0808TS277_5-D1.rsa	21.714	0.515	0.01	0.475	192	0.113	45.17
T0808TS063_5-D1.rsa	21.711	0.399	0.02	0.581	265	0.082	59.48
T0808TS483_3-D1.rsa	21.605	0.434	0.09	0.476	217	0.1	74.94
T0808TS011_3-D1.rsa	21.605	0.432	0.092	0.476	217	0.1	69.81
T0808TS448_4-D1.rsa	21.143	0.49	0.007	0.502	203	0.104	38.86
T0808TS197_1-D1.rsa	21.143	0.52	0.005	0.475	192	0.11	44.62
T0808TS310_2-D1.rsa	21.053	0.316	0.117	0.566	188	0.112	94.8
T0808TS160_2-D1.rsa	21.053	0.283	0.09	0.627	208	0.101	55.72
T0808TS155_2-D1.rsa	20.988	0.425	0.09	0.485	221	0.095	73.01
T0808TS044_2-D1.rsa	20.779	0.42	0.121	0.46	103	0.202	80.36
T0808TS492_4-D1.rsa	20.588	0.284	0.07	0.646	157	0.131	63.11
T0808TS216_4-D1.rsa	20.571	0.527	0	0.473	191	0.108	45.48
T0808TS118_2-D1.rsa	20.571	0.527	0.005	0.468	189	0.109	44.49
T0808TS403_2-D1.rsa	20.571	0.537	0.015	0.448	181	0.114	44.62
T0808TS317_2_1-D1.rsa	20.37	0.417	0.088	0.496	226	0.09	71.14
T0808TS228_1-D1.rsa	20.37	0.421	0.09	0.489	223	0.091	71.36
T0808TS110_2-D1.rsa	20.37	0.423	0.096	0.48	219	0.093	71.8
T0808TS011_2-D1.rsa	20.37	0.421	0.096	0.482	220	0.093	69.76
T0808TS282_4-D1.rsa	20.37	0.445	0.09	0.465	212	0.096	74.67
T0808TS301_1-D1.rsa	20.37	0.432	0.101	0.467	213	0.096	69.48
T0808TS117_1-D1.rsa	19.767	0.367	0.11	0.523	148	0.134	73.14
T0808TS290_1-D1.rsa	19.767	0.371	0.11	0.519	147	0.134	73.14
T0808TS301_3-D1.rsa	19.767	0.389	0.113	0.498	141	0.14	68.02
T0808TS300_3-D1.rsa	19.737	0.406	0.024	0.57	260	0.076	55.32
T0808TS442_2-D1.rsa	19.737	0.425	0.004	0.57	260	0.076	59.16
T0808TS169_1-D1.rsa	19.481	0.393	0.125	0.482	108	0.18	80.48
T0808TS322_3-D1.rsa	19.136	0.303	0.033	0.664	303	0.063	66.28
T0808TS116_1-D1.rsa	19.136	0.425	0.088	0.487	222	0.086	69.81
T0808TS237_1-D1.rsa	19.136	0.436	0.096	0.467	213	0.09	71.19
T0808TS049_4-D1.rsa	19.079	0.425	0.029	0.546	249	0.077	63.87
T0808TS237_5-D1.rsa	19.079	0.423	0.029	0.548	250	0.076	64.14
T0808TS110_5-D1.rsa	19.079	0.452	0.02	0.529	241	0.079	62.06

T0808TS145_3-D1.rsa	19.079	0.445	0.033	0.522	238	0.08	61.51
T0808TS362_1-D1.rsa	18.812	0.265	0.137	0.598	192	0.098	68.69
T0808TS118_1-D1.rsa	18.644	0.009	0.215	0.776	166	0.112	62.62
T0808TS414_3-D1.rsa	18.605	0.375	0.11	0.516	146	0.127	82.6
T0808TS335_4-D1.rsa	18.605	0.42	0.12	0.459	130	0.143	81.01
T0808TS439_3-D1.rsa	18.519	0.404	0.088	0.509	232	0.08	50.33
T0808TS436_2-D1.rsa	18.421	0.404	0.022	0.575	262	0.07	50.71
T0808TS317_1_1-D1.rsa	18.421	0.434	0.024	0.542	247	0.075	59.05
T0808TS026_1-D1.rsa	18.421	0.307	0.114	0.578	192	0.096	98.19
T0808TS197_5-D1.rsa	18.421	0.292	0.114	0.593	197	0.094	55.57
T0808TS228_2-D1.rsa	17.901	0.441	0.09	0.469	214	0.084	79.97
T0808TS034_1-D1.rsa	17.692	0.269	0.236	0.496	227	0.078	69.5
T0808TS169_2-D1.rsa	17.544	0.411	0.096	0.493	108	0.162	73.29
T0808TS358_1-D1.rsa	17.544	0.42	0.087	0.493	108	0.162	80.82
T0808TS338_5-D1.rsa	17.442	0.364	0.057	0.58	164	0.106	76.24
T0808TS410_3-D1.rsa	17.442	0.378	0.113	0.509	144	0.121	79.77
T0808TS347_4-D1.rsa	17.442	0.399	0.117	0.484	137	0.127	82.07
T0808TS162_4-D1.rsa	17.442	0.382	0.117	0.502	142	0.123	74.12
T0808TS116_4-D1.rsa	17.105	0.43	0.011	0.559	255	0.067	59.92
T0808TS153_3-D1.rsa	17.105	0.404	0.024	0.572	261	0.066	56.41
T0808TS216_2-D1.rsa	17.105	0.301	0.096	0.602	200	0.086	98.19
T0808TS340_2-D1.rsa	17.105	0.268	0.117	0.614	204	0.084	98.12
T0808TS349_1-D1.rsa	16.923	0.249	0.166	0.585	268	0.063	61.17
T0808TS042_4-D1.rsa	16.923	0.264	0.223	0.513	235	0.072	77.5
T0808TS145_5-D1.rsa	16.923	0.262	0.242	0.496	227	0.075	72.72
T0808TS064_2-D1.rsa	16.923	0.26	0.245	0.496	227	0.075	71.78
T0808TS296_4-D1.rsa	16.832	0.218	0.125	0.657	211	0.08	51.48
T0808TS110_4-D1.rsa	16.447	0.393	0	0.607	277	0.059	56.09
T0808TS011_5-D1.rsa	16.447	0.45	0.024	0.526	240	0.069	52.41
T0808TS162_3-D1.rsa	16.279	0.406	0.124	0.47	133	0.122	82.86
T0808TS132_2-D1.rsa	16.279	0.385	0.131	0.484	137	0.119	73.41
T0808TS063_3-D1.rsa	16.154	0.258	0.203	0.539	247	0.065	71.89
T0808TS153_2-D1.rsa	16.154	0.266	0.236	0.498	228	0.071	75
T0808TS160_5-D1.rsa	15.789	0.301	0.148	0.551	183	0.086	99.1
T0808TS479_5-D1.rsa	15.789	0.343	0.09	0.566	188	0.084	98.04
T0808TS410_5-D1.rsa	15.789	0.331	0.142	0.527	175	0.09	99.4
T0808TS235_4-D1.rsa	15.385	0.247	0.203	0.55	252	0.061	72.78
T0808TS042_3-D1.rsa	15.385	0.242	0.205	0.552	253	0.061	71.83
T0808TS445_3-D1.rsa	15.385	0.26	0.216	0.524	240	0.064	53.33
T0808TS268_4-D1.rsa	14.894	0.319	0.117	0.564	145	0.103	85.7
T0808TS445_4-D1.rsa	14.894	0.327	0.187	0.486	125	0.119	79.57
T0808TS080_1-D1.rsa	14.615	0.255	0.216	0.528	242	0.06	78.17
T0808TS322_5-D1.rsa	14.615	0.262	0.218	0.52	238	0.061	73.67
T0808TS263_5-D1.rsa	14.615	0.264	0.242	0.493	226	0.065	78.56
T0808TS160_1-D1.rsa	14.615	0.279	0.247	0.474	217	0.067	77.83
T0808TS041_5-D1.rsa	14.615	0.284	0.234	0.483	221	0.066	71.33
T0808TS326_4-D1.rsa	14.474	0.289	0.133	0.578	192	0.075	89.01

T0808TS153_5-D1.rsa	14.474	0.31	0.148	0.542	180	0.08	99.02
T0808TS277_2-D1.rsa	14.474	0.343	0.117	0.539	179	0.081	99.4
T0808TS042_1-D1.rsa	14.474	0.349	0.117	0.533	177	0.082	99.32
T0808TS452_3-D1.rsa	14.474	0.211	0.087	0.702	233	0.062	48.87
T0808TS118_4-D1.rsa	14.474	0.352	0.123	0.524	174	0.083	99.02
T0808TS268_1-D1.rsa	14.474	0.343	0.142	0.515	171	0.085	94.8
T0808TS153_4-D1.rsa	13.158	0.34	0.123	0.536	178	0.074	98.49
T0808TS237_4-D1.rsa	13.158	0.346	0.123	0.53	176	0.075	99.17
T0808TS326_1-D1.rsa	13.158	0.328	0.133	0.539	179	0.074	82.91
T0808TS483_5-D1.rsa	13.158	0.355	0.123	0.521	173	0.076	87.05
T0808TS041_1-D1.rsa	13.158	0.343	0.136	0.521	173	0.076	94.73
T0808TS144_5-D1.rsa	12.766	0.307	0.175	0.518	133	0.096	81.52
T0808TS277_1-D1.rsa	12.766	0.268	0.171	0.56	144	0.089	73.73
T0808TS067_4-D1.rsa	12.766	0.331	0.171	0.498	128	0.1	82.98
T0808TS145_2-D1.rsa	11.842	0.214	0.136	0.651	216	0.055	92.92
T0808TS049_2-D1.rsa	11.842	0.343	0.133	0.524	174	0.068	97.36
T0808TS364_1-D1.rsa	10.638	0.339	0.183	0.479	123	0.086	85.02
T0808TS358_2-D1.rsa	10.638	0.331	0.183	0.486	125	0.085	82.39
T0879TS464_5-D1.rsa	81.429	0.195	0	0.805	177	0.46	11.71
T0879TS464_2-D1.rsa	81.429	0.282	0	0.718	158	0.515	10.91
T0879TS321_5-D1.rsa	81.429	0.323	0.009	0.668	147	0.554	10.23
T0879TS321_4-D1.rsa	77.143	0.291	0.018	0.691	152	0.508	14.54
T0879TS321_1-D1.rsa	75.714	0.327	0.036	0.636	140	0.541	13.98
T0879TS321_3-D1.rsa	68.571	0.336	0.018	0.645	142	0.483	12.5
T0879TS321_2-D1.rsa	54.286	0.314	0.023	0.664	146	0.372	11.59
T0879TS434_2-D1.rsa	50	0.323	0	0.677	149	0.336	16.25
T0879TS434_1-D1.rsa	47.143	0.341	0	0.659	145	0.325	16.59
T0879TS434_5-D1.rsa	45.714	0.355	0.009	0.636	140	0.327	16.48
T0879TS455_3-D1.rsa	44.286	0.136	0.05	0.814	179	0.247	10.68
T0879TS451_4-D1.rsa	44.286	0.341	0.077	0.582	128	0.346	46.59
T0879TS455_1-D1.rsa	42.857	0.173	0	0.827	182	0.235	8.98
T0879TS434_3-D1.rsa	41.429	0.336	0	0.664	146	0.284	16.48
T0879TS451_5-D1.rsa	41.429	0.336	0.077	0.586	129	0.321	44.66
T0879TS451_3-D1.rsa	38.571	0.341	0.077	0.582	128	0.301	47.61
T0879TS434_4-D1.rsa	38.571	0.341	0	0.659	145	0.266	17.61
T0879TS382_2-D1.rsa	37.143	0.355	0.059	0.586	129	0.288	15.46
T0879TS451_2-D1.rsa	35.714	0.332	0.073	0.595	131	0.273	45.23
T0879TS451_1-D1.rsa	35.714	0.336	0.073	0.591	130	0.275	44.2
T0879TS455_4-D1.rsa	35.714	0.186	0.068	0.745	164	0.218	19.2
T0879TS455_2-D1.rsa	35.714	0.045	0.036	0.918	202	0.177	11.59
T0879TS382_3-D1.rsa	34.286	0.382	0.023	0.595	131	0.262	17.5
T0879TS251_2-D1.rsa	34.286	0.277	0.068	0.655	144	0.238	44.66
T0879TS357_5-D1.rsa	31.429	0.309	0.036	0.655	144	0.218	69.09
T0879TS382_1-D1.rsa	31.429	0.273	0	0.727	160	0.196	15
T0879TS357_3-D1.rsa	31.429	0.268	0.009	0.723	159	0.198	69.66
T0879TS275_2-D1.rsa	31.429	0.368	0.105	0.527	116	0.271	69.66
T0879TS357_1-D1.rsa	30	0.3	0	0.7	154	0.195	69.2

T0879TS251_5-D1.rsa	30	0.368	0.114	0.518	114	0.263	69.2
T0879TS464_3-D1.rsa	30	0.377	0.1	0.523	115	0.261	74.89
T0879TS026_5-D1.rsa	30	0.341	0.105	0.555	122	0.246	65.23
T0879TS183_1-D1.rsa	28.571	0.364	0.073	0.564	124	0.23	73.64
T0879TS048_1-D1.rsa	28.571	0.377	0.109	0.514	113	0.253	75.23
T0879TS467_4-D1.rsa	28.571	0.336	0.114	0.55	121	0.236	62.5
T0879TS026_1-D1.rsa	28.571	0.332	0.064	0.605	133	0.215	67.84
T0879TS258_4-D1.rsa	28.571	0.35	0.123	0.527	116	0.246	74.2
T0879TS382_5-D1.rsa	28.571	0.35	0.018	0.632	139	0.206	16.82
T0879TS446_4-D1.rsa	28.571	0.364	0.132	0.505	111	0.257	77.39
T0879TS467_2-D1.rsa	28.571	0.359	0.136	0.505	111	0.257	71.7
T0879TS313_3-D1.rsa	27.143	0.377	0.064	0.559	123	0.221	73.18
T0879TS258_3-D1.rsa	27.143	0.359	0.127	0.514	113	0.24	75.57
T0879TS275_4-D1.rsa	27.143	0.386	0.109	0.505	111	0.245	72.39
T0879TS180_4-D1.rsa	27.143	0.345	0.114	0.541	119	0.228	61.14
T0879TS026_2-D1.rsa	27.143	0.305	0.095	0.6	132	0.206	70.57
T0879TS258_2-D1.rsa	27.143	0.359	0.123	0.518	114	0.238	76.25
T0879TS251_4-D1.rsa	27.143	0.327	0.109	0.564	124	0.219	65.8
T0879TS275_1-D1.rsa	27.143	0.368	0.109	0.523	115	0.236	69.89
T0879TS275_5-D1.rsa	27.143	0.314	0.105	0.582	128	0.212	70.68
T0879TS220_1-D1.rsa	27.143	0.368	0.118	0.514	113	0.24	73.07
T0879TS455_5-D1.rsa	27.143	0.277	0.095	0.627	138	0.197	60.11
T0879TS357_2-D1.rsa	27.143	0.318	0.009	0.673	148	0.183	68.75
T0879TS357_4-D1.rsa	27.143	0.286	0.032	0.682	150	0.181	69.43
T0879TS183_3-D1.rsa	25.714	0.345	0.095	0.559	123	0.209	75.34
T0879TS405_4-D1.rsa	25.714	0.345	0.118	0.536	118	0.218	74.77
T0879TS028_1-D1.rsa	25.714	0.355	0.105	0.541	119	0.216	71.48
T0879TS452_3-D1.rsa	25.714	0.355	0.109	0.536	118	0.218	69.66
T0879TS250_5-D1.rsa	25.714	0.345	0.109	0.545	120	0.214	69.32
T0879TS258_1-D1.rsa	25.714	0.359	0.095	0.545	120	0.214	75.45
T0879TS258_5-D1.rsa	25.714	0.359	0.109	0.532	117	0.22	75.91
T0879TS180_2-D1.rsa	25.714	0.373	0.105	0.523	115	0.224	69.89
T0879TS026_4-D1.rsa	25.714	0.282	0.109	0.609	134	0.192	26.02
T0879TS180_5-D1.rsa	25.714	0.327	0.018	0.655	144	0.179	42.05
T0879TS405_3-D1.rsa	25.714	0.341	0.127	0.532	117	0.22	72.16
T0879TS220_5-D1.rsa	25.714	0.395	0.114	0.491	108	0.238	72.61
T0879TS464_4-D1.rsa	25.714	0.355	0.114	0.532	117	0.22	74.89
T0879TS236_4-D1.rsa	25.714	0.373	0.109	0.518	114	0.226	77.16
T0879TS467_3-D1.rsa	25.714	0.373	0.114	0.514	113	0.228	71.59
T0879TS446_1-D1.rsa	25.714	0.418	0.114	0.468	103	0.25	72.16
T0879TS250_3-D1.rsa	24.286	0.341	0.109	0.55	121	0.201	69.66
T0879TS345_5-D1.rsa	24.286	0.373	0.132	0.495	109	0.223	73.18
T0879TS005_4-D1.rsa	24.286	0.4	0.118	0.482	106	0.229	73.64
T0879TS405_1-D1.rsa	24.286	0.359	0.132	0.509	112	0.217	74.2
T0879TS446_5-D1.rsa	24.286	0.4	0.123	0.477	105	0.231	71.7
T0879TS313_1-D1.rsa	24.286	0.377	0.064	0.559	123	0.197	73.98
T0879TS313_4-D1.rsa	24.286	0.377	0.064	0.559	123	0.197	74.77

T0879TS287_4-D1.rsa	24.286	0.35	0.118	0.532	117	0.208	77.27
T0879TS183_2-D1.rsa	24.286	0.368	0.127	0.505	111	0.219	67.95
T0879TS250_2-D1.rsa	24.286	0.341	0.109	0.55	121	0.201	69.32
T0879TS479_3-D1.rsa	24.286	0.368	0.105	0.527	116	0.209	68.98
T0879TS464_1-D1.rsa	24.286	0.35	0.1	0.55	121	0.201	75.23
T0879TS287_2-D1.rsa	24.286	0.368	0.118	0.514	113	0.215	76.59
T0879TS345_2-D1.rsa	24.286	0.377	0.136	0.486	107	0.227	77.84
T0879TS236_2-D1.rsa	24.286	0.341	0.118	0.541	119	0.204	76.36
T0879TS287_5-D1.rsa	24.286	0.359	0.123	0.518	114	0.213	76.48
T0879TS467_1-D1.rsa	24.286	0.35	0.114	0.536	118	0.206	65.23
T0879TS407_2-D1.rsa	24.286	0.355	0.059	0.586	129	0.188	70.45
T0879TS251_1-D1.rsa	24.286	0.341	0.118	0.541	119	0.204	71.25
T0879TS016_1-D1.rsa	24.286	0.382	0.109	0.509	112	0.217	79.2
T0879TS220_2-D1.rsa	24.286	0.368	0.109	0.523	115	0.211	72.95
T0879TS005_5-D1.rsa	24.286	0.395	0.114	0.491	108	0.225	75.57
T0879TS467_5-D1.rsa	24.286	0.377	0.095	0.527	116	0.209	59.43
T0879TS220_4-D1.rsa	24.286	0.373	0.109	0.518	114	0.213	72.16
T0879TS444_1-D1.rsa	22.857	0.391	0.105	0.505	111	0.206	67.95
T0879TS183_4-D1.rsa	22.857	0.4	0.073	0.527	116	0.197	62.61
T0879TS452_4-D1.rsa	22.857	0.35	0.091	0.559	123	0.186	66.14
T0879TS313_2-D1.rsa	22.857	0.377	0.064	0.559	123	0.186	74.2
T0879TS444_2-D1.rsa	22.857	0.423	0.123	0.455	100	0.229	63.98
T0879TS250_1-D1.rsa	22.857	0.341	0.109	0.55	121	0.189	69.55
T0879TS005_3-D1.rsa	22.857	0.391	0.109	0.5	110	0.208	73.86
T0879TS452_2-D1.rsa	22.857	0.336	0.105	0.559	123	0.186	74.89
T0879TS236_3-D1.rsa	22.857	0.359	0.114	0.527	116	0.197	76.14
T0879TS444_4-D1.rsa	22.857	0.35	0.123	0.527	116	0.197	58.18
T0879TS005_1-D1.rsa	22.857	0.423	0.114	0.464	102	0.224	75.11
T0879TS345_3-D1.rsa	22.857	0.386	0.123	0.491	108	0.212	77.27
T0879TS180_3-D1.rsa	22.857	0.355	0.118	0.527	116	0.197	67.27
T0879TS407_5-D1.rsa	22.857	0.332	0.095	0.573	126	0.181	67.5
T0879TS349_1-D1.rsa	22.857	0.373	0.064	0.564	124	0.184	73.75
T0879TS251_3-D1.rsa	22.857	0.305	0.077	0.618	136	0.168	46.59
T0879TS220_3-D1.rsa	22.857	0.355	0.109	0.536	118	0.194	72.73
T0879TS479_5-D1.rsa	21.429	0.405	0.068	0.527	116	0.185	62.84
T0879TS275_3-D1.rsa	21.429	0.382	0.118	0.5	110	0.195	73.07
T0879TS421_4-D1.rsa	21.429	0.336	0.077	0.586	129	0.166	66.25
T0879TS407_3-D1.rsa	21.429	0.395	0.086	0.518	114	0.188	69.77
T0879TS345_4-D1.rsa	21.429	0.373	0.118	0.509	112	0.191	78.75
T0879TS236_1-D1.rsa	21.429	0.364	0.118	0.518	114	0.188	76.36
T0879TS444_3-D1.rsa	21.429	0.318	0.127	0.555	122	0.176	69.55
T0879TS236_5-D1.rsa	21.429	0.359	0.109	0.532	117	0.183	76.93
T0879TS479_4-D1.rsa	21.429	0.318	0.127	0.555	122	0.176	64.66
T0879TS250_4-D1.rsa	21.429	0.368	0.109	0.523	115	0.186	69.32
T0879TS425_2-D1.rsa	21.429	0.323	0.1	0.577	127	0.169	70.11
T0879TS287_3-D1.rsa	21.429	0.364	0.123	0.514	113	0.19	76.93
T0879TS183_5-D1.rsa	21.429	0.314	0.127	0.559	123	0.174	66.02

T0879TS452_5-D1.rsa	21.429	0.355	0.127	0.518	114	0.188	70.11
T0879TS005_2-D1.rsa	21.429	0.418	0.109	0.473	104	0.206	75.23
T0879TS119_1-D1.rsa	21.429	0.373	0.064	0.564	124	0.173	73.75
T0879TS444_5-D1.rsa	21.429	0.377	0.1	0.523	115	0.186	57.95
T0879TS479_2-D1.rsa	20	0.377	0.055	0.568	125	0.16	75.57
T0879TS479_1-D1.rsa	20	0.395	0.091	0.514	113	0.177	74.43
T0879TS425_4-D1.rsa	20	0.314	0.114	0.573	126	0.159	69.09
T0879TS077_1-D1.rsa	20	0.327	0.123	0.55	121	0.165	70.34
T0879TS446_2-D1.rsa	20	0.423	0.114	0.464	102	0.196	72.5
T0879TS077_4-D1.rsa	20	0.318	0.114	0.568	125	0.16	68.98
T0879TS407_4-D1.rsa	20	0.314	0.086	0.6	132	0.152	70
T0879TS405_2-D1.rsa	20	0.359	0.132	0.509	112	0.179	73.64
T0879TS345_1-D1.rsa	20	0.405	0.118	0.477	105	0.19	76.25
T0879TS287_1-D1.rsa	20	0.359	0.123	0.518	114	0.175	76.48
T0879TS077_2-D1.rsa	20	0.355	0.114	0.532	117	0.171	68.52
T0879TS405_5-D1.rsa	20	0.332	0.127	0.541	119	0.168	74.09
T0879TS452_1-D1.rsa	20	0.332	0.118	0.55	121	0.165	65.91
T0879TS425_3-D1.rsa	20	0.3	0.095	0.605	133	0.15	69.43
T0879TS425_1-D1.rsa	20	0.332	0.114	0.555	122	0.164	70.45
T0879TS077_3-D1.rsa	18.571	0.314	0.1	0.586	129	0.144	69.09
T0879TS077_5-D1.rsa	18.571	0.318	0.105	0.577	127	0.146	70
T0879TS180_1-D1.rsa	18.571	0.318	0.086	0.595	131	0.142	64.89
T0879TS425_5-D1.rsa	18.571	0.314	0.109	0.577	127	0.146	69.09
T0879TS421_5-D1.rsa	18.571	0.341	0.068	0.591	130	0.143	61.59
T0879TS407_1-D1.rsa	15.714	0.323	0.082	0.595	131	0.12	70.91
T0838TS116_2-D1.rsa	92	0.072	0	0.928	128	0.719	13.22
T0838TS368_3-D1.rsa	84	0.087	0.261	0.652	90	0.933	18.48
T0838TS203_4-D1.rsa	82.353	0.516	0	0.484	61	1.35	27.98
T0838TS452_2-D1.rsa	79.412	0.278	0	0.722	91	0.873	28.57
T0838TS197_4-D1.rsa	73.529	0.54	0	0.46	58	1.268	28.97
T0838TS049_3-D1.rsa	71.429	0.015	0.221	0.763	100	0.714	12.4
T0838TS433_2-D1.rsa	71.053	0.333	0.072	0.595	66	1.077	18.92
T0838TS032_2-D1.rsa	70.833	0	0.333	0.667	72	0.984	21.76
T0838TS310_2-D1.rsa	70.833	0.045	0.136	0.818	90	0.787	29.09
T0838TS173_5-D1.rsa	70.588	0.325	0	0.675	85	0.83	32.14
T0838TS457_5-D1.rsa	68.421	0.315	0.018	0.667	74	0.925	19.82
T0838TS153_3-D1.rsa	68	0.014	0.014	0.971	134	0.507	10.51
T0838TS064_5-D1.rsa	67.857	0	0.298	0.702	92	0.738	17.18
T0838TS156_2-D1.rsa	67.647	0.579	0	0.421	53	1.276	33.73
T0838TS403_5-D1.rsa	66.667	0.167	0.111	0.722	78	0.855	34.95
T0838TS173_1-D1.rsa	66.667	0	0.407	0.593	64	1.042	57.18
T0838TS044_3-D1.rsa	66.667	0	0.556	0.444	48	1.389	72.92
T0838TS483_5-D1.rsa	66.667	0	0.454	0.546	59	1.13	50.23
T0838TS073_2-D1.rsa	66.197	0.446	0	0.554	93	0.712	19.07
T0838TS008_4-D1.rsa	66.071	0.103	0.159	0.738	93	0.71	21.43
T0838TS358_4-D1.rsa	64.583	0.1	0.227	0.673	74	0.873	26.82
T0838TS499_3-D1.rsa	63.462	0.279	0.097	0.624	103	0.616	70.73

T0838TS277_5-D1.rsa	63.38	0.202	0.042	0.756	127	0.499	12.66
T0838TS120_3-D1.rsa	62.791	0.216	0.06	0.724	97	0.647	18.1
T0838TS067_4-D1.rsa	62.791	0.388	0.045	0.567	76	0.826	42.35
T0838TS097_5-D1.rsa	62.791	0.425	0.052	0.522	70	0.897	41.79
T0838TS338_3-D1.rsa	62.5	0	0.583	0.417	45	1.389	75
T0838TS434_1-D1.rsa	62.5	0	0.62	0.38	41	1.524	71.76
T0838TS153_2-D1.rsa	62.5	0	0.565	0.435	47	1.33	69.91
T0838TS317_5-D1.rsa	62.5	0.019	0.519	0.463	50	1.25	71.3
T0838TS144_5-D1.rsa	62.5	0.331	0.044	0.625	85	0.735	11.4
T0838TS403_2-D1.rsa	60.526	0.279	0.018	0.703	78	0.776	16.67
T0838TS011_2-D1.rsa	59.615	0.461	0	0.539	89	0.67	17.88
T0838TS326_4-D1.rsa	59.155	0.173	0	0.827	139	0.426	17.15
T0838TS290_3-D1.rsa	58.333	0	0.5	0.5	54	1.08	68.06
T0838TS204_3-D1.rsa	58.333	0.037	0.5	0.463	50	1.167	71.99
T0838TS328_5-D1.rsa	58.14	0.291	0	0.709	95	0.612	37.13
T0838TS336_4-D1.rsa	58.14	0.209	0.097	0.694	93	0.625	22.95
T0838TS368_4-D1.rsa	57.895	0.126	0	0.874	97	0.597	15.77
T0838TS144_3-D1.rsa	57.895	0	0.135	0.865	96	0.603	18.92
T0838TS210_1-D1.rsa	57.692	0.273	0.085	0.642	106	0.544	65.19
T0838TS448_5-D1.rsa	57.692	0.279	0.097	0.624	103	0.56	77.69
T0838TS347_3-D1.rsa	57.692	0.261	0.048	0.691	114	0.506	55.22
T0838TS077_5-D1.rsa	57.692	0.242	0.085	0.673	111	0.52	60.13
T0838TS212_1-D1.rsa	57.534	0.404	0	0.596	152	0.379	39.61
T0838TS452_1-D1.rsa	57.5	0.07	0.184	0.746	85	0.676	39.25
T0838TS077_1-D1.rsa	57.143	0	0.016	0.984	124	0.461	16.67
T0838TS454_2-D1.rsa	57.143	0.145	0.244	0.611	80	0.714	12.79
T0838TS452_4-D1.rsa	55.882	0.302	0	0.698	88	0.635	31.15
T0838TS216_2-D1.rsa	55.882	0.556	0	0.444	56	0.998	33.53
T0838TS277_4-D1.rsa	55.814	0.299	0.06	0.642	86	0.649	42.35
T0838TS279_1-D1.rsa	55.769	0.267	0.103	0.63	104	0.536	74.84
T0838TS349_5-D1.rsa	55.769	0.285	0.115	0.6	99	0.563	64.24
T0838TS428_3-D1.rsa	55.769	0.327	0.115	0.558	92	0.606	67.72
T0838TS184_5-D1.rsa	55.263	0.153	0.036	0.811	90	0.614	27.93
T0838TS358_3-D1.rsa	55	0	0.351	0.649	74	0.743	16.01
T0838TS336_5-D1.rsa	54.167	0	0.602	0.398	43	1.26	66.44
T0838TS145_5-D1.rsa	53.846	0.273	0.109	0.618	102	0.528	63.45
T0838TS097_4-D1.rsa	53.571	0	0.275	0.725	95	0.564	16.03
T0838TS310_1-D1.rsa	53.571	0	0.359	0.641	84	0.638	19.85
T0838TS492_4-D1.rsa	53.571	0	0.282	0.718	94	0.57	12.98
T0838TS153_5-D1.rsa	53.125	0.191	0.044	0.765	104	0.511	13.05
T0838TS381_1-D1.rsa	52.632	0.054	0	0.946	105	0.501	17.12
T0838TS410_2-D1.rsa	52	0.065	0.275	0.659	91	0.571	13.77
T0838TS153_1-D1.rsa	51.923	0.267	0.139	0.594	98	0.53	18.2
T0838TS296_2-D1.rsa	51.563	0	0.338	0.662	90	0.573	12.5
T0838TS044_2-D1.rsa	51.163	0.403	0.06	0.537	72	0.711	43.28
T0838TS216_4-D1.rsa	51.163	0.381	0.052	0.567	76	0.673	36.94
T0838TS034_2-D1.rsa	50	0.339	0.042	0.618	102	0.49	47.78

T0838TS063_5-D1.rsa	50	0.285	0.036	0.679	112	0.446	16.3
T0838TS368_1-D1.rsa	50	0	0.211	0.789	90	0.556	40.13
T0838TS120_1-D1.rsa	50	0	0.267	0.733	96	0.521	15.08
T0838TS011_3-D1.rsa	50	0.135	0.373	0.492	62	0.806	44.44
T0838TS492_1-D1.rsa	50	0.031	0.344	0.626	82	0.61	16.79
T0838TS438_1-D1.rsa	50	0	0.366	0.634	83	0.602	17.37
T0838TS024_5-D1.rsa	49.495	0.285	0	0.715	206	0.24	11.02
T0838TS258_1-D1.rsa	49.296	0.381	0.083	0.536	90	0.548	22.11
T0838TS132_4-D1.rsa	48.837	0.239	0.045	0.716	96	0.509	22.76
T0838TS228_1-D1.rsa	48.077	0.176	0.073	0.752	124	0.388	45.41
T0838TS237_1-D1.rsa	48	0.179	0.109	0.711	234	0.205	9.04
T0838TS067_2-D1.rsa	47.368	0.278	0.269	0.454	49	0.967	68.98
T0838TS340_3-D1.rsa	47.368	0.261	0.144	0.595	66	0.718	36.71
T0838TS184_3-D1.rsa	47.368	0.306	0.108	0.586	65	0.729	39.41
T0838TS162_1-D1.rsa	46.512	0.284	0.045	0.672	90	0.517	38.06
T0838TS203_2-D1.rsa	46.512	0.418	0.075	0.507	68	0.684	44.4
T0838TS044_4-D1.rsa	46.479	0.208	0.167	0.625	105	0.443	15.54
T0838TS296_3-D1.rsa	46.429	0.023	0.252	0.725	95	0.489	16.03
T0838TS116_4-D1.rsa	46.154	0.339	0.03	0.63	104	0.444	17.72
T0838TS041_3-D1.rsa	45.833	0.019	0.528	0.454	49	0.935	64.58
T0838TS173_4-D1.rsa	45.833	0	0.63	0.37	40	1.146	71.76
T0838TS024_4-D1.rsa	45.455	0.051	0.405	0.544	161	0.282	35.22
T0838TS454_4-D1.rsa	45	0	0.316	0.684	78	0.577	22.15
T0838TS328_3-D1.rsa	44.828	0	0.091	0.909	130	0.345	60.84
T0838TS445_5-D1.rsa	44.737	0.054	0.018	0.928	103	0.434	20.05
T0838TS241_2-D1.rsa	44.737	0.306	0.108	0.586	65	0.688	39.41
T0838TS258_4-D1.rsa	44.231	0.285	0.158	0.558	92	0.481	21.68
T0838TS237_5-D1.rsa	44	0.032	0	0.968	391	0.113	5.07
T0838TS210_3-D1.rsa	43.86	0.29	0.074	0.636	138	0.318	58.3
T0838TS336_3-D1.rsa	43.75	0.073	0.318	0.609	67	0.653	62.5
T0838TS228_5-D1.rsa	43.421	0.352	0.012	0.636	211	0.206	9.56
T0838TS425_2-D1.rsa	43.103	0	0.07	0.93	133	0.324	59.62
T0838TS364_1-D1.rsa	42.857	0	0.45	0.55	72	0.595	62.21
T0838TS439_1-D1.rsa	42.857	0	0.443	0.557	73	0.587	57.44
T0838TS340_2-D1.rsa	42.857	0	0.298	0.702	92	0.466	37.41
T0838TS499_2-D1.rsa	42.857	0	0.45	0.55	72	0.595	60.88
T0838TS333_1-D1.rsa	42.857	0.23	0.238	0.532	67	0.64	20.44
T0838TS173_3-D1.rsa	42.466	0.729	0	0.271	69	0.615	52.16
T0838TS024_2-D1.rsa	42.308	0.206	0.012	0.782	129	0.328	22.63
T0838TS050_1-D1.rsa	42.188	0	0.221	0.779	106	0.398	15.81
T0838TS145_3-D1.rsa	42.105	0.252	0.036	0.712	79	0.533	50.68
T0838TS169_2-D1.rsa	42.105	0.25	0.324	0.426	46	0.915	68.98
T0838TS056_1-D1.rsa	41.86	0.299	0.09	0.612	82	0.51	44.78
T0838TS064_4-D1.rsa	41.86	0.276	0.015	0.709	95	0.441	41.98
T0838TS260_1-D1.rsa	41.667	0.018	0.436	0.545	60	0.694	73.64
T0838TS118_1-D1.rsa	41.667	0.036	0.436	0.527	58	0.718	73.41
T0838TS403_3-D1.rsa	41.667	0.019	0.296	0.685	74	0.563	56.02

T0838TS404_3-D1.rsa	41.667	0	0.436	0.564	62	0.672	45.45
T0838TS038_1-D1.rsa	41.379	0	0.14	0.86	123	0.336	58.92
T0838TS391_3-D1.rsa	41.071	0.127	0.135	0.738	93	0.442	21.63
T0838TS492_5-D1.rsa	41.071	0.151	0.294	0.556	70	0.587	44.84
T0838TS197_2-D1.rsa	40.845	0	0.149	0.851	143	0.286	12.34
T0838TS454_3-D1.rsa	40.625	0.066	0.11	0.824	112	0.363	34.93
T0838TS111_1-D1.rsa	40.404	0.233	0.042	0.726	209	0.193	38.63
T0838TS290_4-D1.rsa	40	0.065	0.275	0.659	91	0.44	53.8
T0838TS184_4-D1.rsa	40	0.08	0.029	0.891	123	0.325	17.39
T0838TS169_4-D1.rsa	40	0	0.447	0.553	63	0.635	24.56
T0838TS290_1-D1.rsa	40	0	0.43	0.57	65	0.615	22.37
T0838TS338_1-D1.rsa	39.583	0.064	0.3	0.636	70	0.565	59.77
T0838TS448_3-D1.rsa	39.535	0.299	0.06	0.642	86	0.46	43.84
T0838TS210_5-D1.rsa	39.474	0.225	0.153	0.622	69	0.572	31.08
T0838TS300_2-D1.rsa	39.394	0.229	0	0.771	222	0.177	48.7
T0838TS381_4-D1.rsa	39.286	0	0.336	0.664	87	0.452	57.63
T0838TS410_3-D1.rsa	39.286	0.015	0.359	0.626	82	0.479	51.91
T0838TS203_1-D1.rsa	39.286	0.015	0.237	0.748	98	0.401	53.05
T0838TS197_1-D1.rsa	39.063	0.015	0.478	0.507	69	0.566	69.67
T0838TS117_1-D1.rsa	38.889	0.357	0.085	0.559	119	0.327	66.88
T0838TS347_2-D1.rsa	38.889	0.394	0.094	0.512	109	0.357	69.16
T0838TS034_4-D1.rsa	38.372	0.302	0.172	0.527	89	0.431	69.97
T0838TS206_1-D1.rsa	37.931	0	0.189	0.811	116	0.327	78.5
T0838TS414_4-D1.rsa	37.5	0.054	0.422	0.524	155	0.242	36.23
T0838TS282_5-D1.rsa	37.5	0.385	0.085	0.531	113	0.332	77.28
T0838TS011_5-D1.rsa	37.5	0.19	0.143	0.667	84	0.446	27.78
T0838TS317_3-D1.rsa	37.5	0.167	0.262	0.571	72	0.521	44.84
T0838TS483_2-D1.rsa	37.209	0.358	0.082	0.56	75	0.496	52.98
T0838TS428_4-D1.rsa	37.209	0.313	0.104	0.582	78	0.477	44.78
T0838TS204_5-D1.rsa	37.209	0.119	0.164	0.716	96	0.388	18.66
T0838TS153_4-D1.rsa	37.143	0.559	0.02	0.421	170	0.218	11.14
T0838TS277_3-D1.rsa	37.069	0.598	0	0.402	103	0.36	55.27
T0838TS276_3-D1.rsa	36.842	0.244	0.021	0.735	244	0.151	40.89
T0838TS067_3-D1.rsa	36.842	0.036	0.117	0.847	94	0.392	16.44
T0838TS420_3-D1.rsa	36.364	0.233	0.194	0.573	165	0.22	38.54
T0838TS038_2-D1.rsa	36.207	0.021	0.07	0.909	130	0.279	47.9
T0838TS008_3-D1.rsa	36.207	0.168	0	0.832	119	0.304	21.85
T0838TS420_4-D1.rsa	36.047	0.325	0.183	0.491	83	0.434	66.72
T0838TS436_1-D1.rsa	36	0.058	0.123	0.819	113	0.319	47.46
T0838TS403_1-D1.rsa	36	0.058	0	0.942	130	0.277	15.04
T0838TS483_1-D1.rsa	36	0.13	0.167	0.703	97	0.371	23.73
T0838TS184_2-D1.rsa	36	0.036	0.341	0.623	86	0.419	58.51
T0838TS439_3-D1.rsa	36	0.014	0.275	0.71	98	0.367	14.67
T0838TS276_4-D1.rsa	35.965	0.253	0.171	0.576	125	0.288	64.4
T0838TS335_3-D1.rsa	35.965	0.295	0.171	0.535	116	0.31	58.87
T0838TS428_2-D1.rsa	35.938	0.015	0.346	0.64	87	0.413	71.88
T0838TS008_2-D1.rsa	35.714	0.048	0.135	0.817	103	0.347	22.02

T0838TS457_4-D1.rsa	35.714	0.119	0.341	0.54	68	0.525	43.65
T0838TS204_1-D1.rsa	35.616	0.663	0	0.337	86	0.414	51.77
T0838TS445_4-D1.rsa	35.616	0.745	0	0.255	65	0.548	60.39
T0838TS433_1-D1.rsa	35.616	0.741	0	0.259	66	0.54	58.33
T0838TS133_2-D1.rsa	35.417	0.1	0.209	0.691	76	0.466	57.95
T0838TS011_4-D1.rsa	35.417	0.018	0.5	0.482	53	0.668	62.95
T0838TS340_1-D1.rsa	35.345	0.641	0	0.359	92	0.384	55.86
T0838TS145_4-D1.rsa	35.294	0.342	0.074	0.584	142	0.249	51.56
T0838TS077_2-D1.rsa	35.294	0.3	0.132	0.568	138	0.256	14
T0838TS210_2-D1.rsa	35.227	0.041	0.334	0.625	185	0.19	48.56
T0838TS063_3-D1.rsa	35.227	0.074	0.304	0.622	184	0.191	36.91
T0838TS111_3-D1.rsa	35.227	0.071	0.297	0.632	187	0.188	35.39
T0838TS410_1-D1.rsa	35.211	0.327	0	0.673	113	0.312	22.44
T0838TS336_2-D1.rsa	35.211	0.393	0.131	0.476	80	0.44	40.55
T0838TS258_2-D1.rsa	35.211	0.399	0.065	0.536	90	0.391	45.99
T0838TS216_5-D1.rsa	35.211	0.482	0.137	0.381	64	0.55	42.63
T0838TS212_2-D1.rsa	35.088	0.379	0.073	0.548	120	0.292	56.85
T0838TS026_1-D1.rsa	35.088	0.258	0.217	0.525	114	0.308	61.06
T0838TS358_5-D1.rsa	35	0	0.36	0.64	73	0.479	48.9
T0838TS381_2-D1.rsa	34.884	0.284	0.189	0.527	89	0.392	70.86
T0838TS160_1-D1.rsa	34.884	0.381	0.067	0.552	74	0.471	53.55
T0838TS322_2-D1.rsa	34.884	0.366	0	0.634	85	0.41	37.87
T0838TS032_3-D1.rsa	34.694	0.378	0.094	0.528	197	0.176	26.04
T0838TS042_1-D1.rsa	34.483	0	0.217	0.783	112	0.308	77.97
T0838TS445_1-D1.rsa	34.483	0.715	0	0.285	73	0.472	76.56
T0838TS263_5-D1.rsa	34.4	0.024	0.334	0.641	211	0.163	23.78
T0838TS439_2-D1.rsa	34.375	0.044	0.132	0.824	112	0.307	52.39
T0838TS430_1-D1.rsa	34.375	0.154	0.191	0.654	89	0.386	16.54
T0838TS067_1-D1.rsa	34.247	0.761	0	0.239	61	0.561	44.61
T0838TS401_3-D1.rsa	34.211	0.241	0.5	0.259	28	1.222	93.52
T0838TS203_3-D1.rsa	34.211	0.287	0.25	0.463	50	0.684	84.26
T0838TS492_3-D1.rsa	34.211	0.25	0.5	0.25	27	1.267	94.21
T0838TS044_1-D1.rsa	34.211	0.25	0.5	0.25	27	1.267	93.98
T0838TS268_2-D1.rsa	34.091	0.064	0.28	0.655	194	0.176	48.73
T0838TS277_1-D1.rsa	33.803	0.292	0.089	0.619	104	0.325	33.17
T0838TS317_4-D1.rsa	33.803	0.405	0.113	0.482	81	0.417	42.31
T0838TS118_5-D1.rsa	33.684	0.508	0.059	0.432	102	0.33	48.94
T0838TS300_4-D1.rsa	33.333	0.115	0.146	0.74	213	0.156	50.87
T0838TS044_5-D1.rsa	33.333	0.115	0.167	0.719	207	0.161	49.74
T0838TS410_5-D1.rsa	33.333	0.064	0.227	0.709	78	0.427	60
T0838TS454_5-D1.rsa	32.955	0.02	0.24	0.74	219	0.15	43.58
T0838TS448_2-D1.rsa	32.955	0.078	0.503	0.419	124	0.266	45.44
T0838TS404_4-D1.rsa	32.877	0.769	0	0.231	59	0.557	64.71
T0838TS296_4-D1.rsa	32.877	0.765	0	0.235	60	0.548	54.51
T0838TS073_1-D1.rsa	32.759	0.625	0	0.375	96	0.341	53.12
T0838TS014_1-D1.rsa	32.692	0.242	0	0.758	125	0.262	12.03
T0838TS063_1-D1.rsa	32.673	0.1	0.072	0.829	266	0.123	30.3

T0838TS118_3-D1.rsa	32.558	0.296	0.136	0.568	96	0.339	53.99
T0838TS492_2-D1.rsa	32.558	0.254	0.015	0.731	98	0.332	38.06
T0838TS173_2-D1.rsa	32.558	0.261	0.112	0.627	84	0.388	28.92
T0838TS008_1-D1.rsa	32.5	0.018	0.263	0.719	82	0.396	42.1
T0838TS144_1-D1.rsa	32.5	0.018	0.237	0.746	85	0.382	44.52
T0838TS204_2-D1.rsa	32.5	0.044	0.333	0.623	71	0.458	46.49
T0838TS169_3-D1.rsa	32.5	0.018	0.421	0.561	64	0.508	51.75
T0838TS425_1-D1.rsa	32.143	0.159	0.349	0.492	62	0.518	43.85
T0838TS401_1-D1.rsa	31.818	0.095	0.439	0.466	138	0.231	52.03
T0838TS133_5-D1.rsa	31.818	0.068	0.52	0.412	122	0.261	46.88
T0838TS347_1-D1.rsa	31.579	0.241	0.389	0.37	40	0.789	90.74
T0838TS454_1-D1.rsa	31.579	0.25	0.407	0.343	37	0.853	92.82
T0838TS241_1-D1.rsa	31.579	0.25	0.509	0.241	26	1.215	94.21
T0838TS391_4-D1.rsa	31.579	0.25	0.5	0.25	27	1.17	93.98
T0838TS410_4-D1.rsa	31.429	0.333	0.179	0.487	114	0.276	63.35
T0838TS077_4-D1.rsa	31.429	0.385	0.218	0.397	93	0.338	70.83
T0838TS160_4-D1.rsa	31.25	0.055	0.373	0.573	63	0.496	63.18
T0838TS041_4-D1.rsa	31.148	0.01	0.148	0.843	177	0.176	73.51
T0838TS063_4-D1.rsa	31.034	0	0.084	0.916	131	0.237	50.35
T0838TS162_4-D1.rsa	31.034	0.645	0	0.355	91	0.341	59.08
T0838TS317_2-D1.rsa	31.034	0.719	0	0.281	72	0.431	63.28
T0838TS268_1-D1.rsa	30.857	0.408	0.02	0.572	231	0.134	38.98
T0838TS420_1-D1.rsa	30.769	0.405	0.122	0.473	140	0.22	54.25
T0838TS433_3-D1.rsa	30.693	0.265	0.128	0.607	195	0.157	74.77
T0838TS326_1-D1.rsa	30.556	0.347	0.085	0.568	121	0.253	71.7
T0838TS228_4-D1.rsa	30.508	0	0.294	0.706	151	0.202	64.95
T0838TS041_2-D1.rsa	30.476	0.368	0.124	0.509	119	0.256	64.32
T0838TS006_2-D1.rsa	30.357	0.175	0.381	0.444	56	0.542	52.58
T0838TS132_3-D1.rsa	30.233	0.325	0.16	0.515	87	0.348	55.77
T0838TS204_4-D1.rsa	30.233	0.269	0	0.731	98	0.308	40.48
T0838TS197_3-D1.rsa	30.172	0.563	0	0.438	112	0.269	48.93
T0838TS335_1-D1.rsa	30.172	0.566	0	0.434	111	0.272	49.71
T0838TS345_5-D1.rsa	30.137	0.372	0.1	0.528	196	0.154	71.46
T0838TS338_5-D1.rsa	30	0.018	0.202	0.781	89	0.337	27.41
T0838TS300_3-D1.rsa	29.932	0.279	0.062	0.66	246	0.122	41.04
T0838TS414_5-D1.rsa	29.545	0.057	0.348	0.595	176	0.168	33.95
T0838TS193_2-D1.rsa	29.412	0.362	0.086	0.551	134	0.219	65.78
T0838TS006_3-D1.rsa	29.412	0.579	0	0.421	53	0.555	53.77
T0838TS228_2-D1.rsa	29.293	0.212	0.108	0.681	196	0.149	46.62
T0838TS049_2-D1.rsa	29.252	0.367	0.091	0.542	202	0.145	78.06
T0838TS160_2-D1.rsa	29.07	0.367	0.254	0.379	64	0.454	74.41
T0838TS439_5-D1.rsa	28.947	0.325	0.006	0.669	222	0.13	12.2
T0838TS169_1-D1.rsa	28.947	0.25	0.5	0.25	27	1.072	94.21
T0838TS317_1-D1.rsa	28.947	0.25	0.5	0.25	27	1.072	93.98
T0838TS290_2-D1.rsa	28.947	0.241	0.5	0.259	28	1.034	93.98
T0838TS006_1-D1.rsa	28.846	0.23	0.012	0.758	125	0.231	12.5
T0838TS145_2-D1.rsa	28.814	0	0.28	0.72	154	0.187	58.53

T0838TS111_2-D1.rsa	28.8	0	0.337	0.663	218	0.132	55.02
T0838TS080_5-D1.rsa	28.767	0.391	0.108	0.501	186	0.155	82.97
T0838TS276_1-D1.rsa	28.713	0.255	0.153	0.592	190	0.151	74.92
T0838TS439_4-D1.rsa	28.571	0.338	0.214	0.449	105	0.272	70.62
T0838TS117_3-D1.rsa	28.571	0.167	0.095	0.738	93	0.307	52.38
T0838TS483_4-D1.rsa	28.571	0	0.313	0.687	90	0.317	53.05
T0838TS282_1-D1.rsa	28.448	0.664	0	0.336	86	0.331	56.35
T0838TS120_2-D1.rsa	28.421	0.504	0.055	0.441	104	0.273	60.8
T0838TS414_2-D1.rsa	28.283	0.215	0.17	0.615	177	0.16	60.42
T0838TS160_5-D1.rsa	28	0.022	0.29	0.688	95	0.295	59.96
T0838TS404_2-D1.rsa	28	0.029	0.304	0.667	92	0.304	68.48
T0838TS448_1-D1.rsa	27.941	0.391	0.107	0.502	122	0.229	64.78
T0838TS391_1-D1.rsa	27.907	0.254	0.015	0.731	98	0.285	43.47
T0838TS260_2-D1.rsa	27.907	0.308	0.207	0.485	82	0.34	72.78
T0838TS368_5-D1.rsa	27.907	0.336	0.097	0.567	76	0.367	53.17
T0838TS144_2-D1.rsa	27.907	0.306	0	0.694	93	0.3	38.43
T0838TS349_2-D1.rsa	27.723	0.252	0.159	0.589	189	0.147	73.91
T0838TS155_1-D1.rsa	27.429	0.428	0.02	0.552	223	0.123	46.53
T0838TS111_5-D1.rsa	27.397	0.396	0.113	0.491	182	0.151	81.95
T0838TS493_4-D1.rsa	27.397	0.388	0.094	0.518	192	0.143	75
T0838TS322_1-D1.rsa	27.368	0.453	0.059	0.487	115	0.238	46.5
T0838TS006_5-D1.rsa	27.368	0.517	0.059	0.424	100	0.274	62.5
T0838TS445_2-D1.rsa	27.368	0.551	0.047	0.403	95	0.288	57.1
T0838TS381_5-D1.rsa	27.273	0.393	0.08	0.527	118	0.231	78.81
T0838TS310_4-D1.rsa	27.273	0.42	0.121	0.46	103	0.265	82.98
T0838TS118_2-D1.rsa	27.273	0.42	0.116	0.464	104	0.262	80.36
T0838TS038_3-D1.rsa	27.119	0.009	0.299	0.692	148	0.183	64.6
T0838TS328_1-D1.rsa	27	0.474	0.096	0.43	108	0.25	86.65
T0838TS457_1-D1.rsa	26.786	0.151	0.333	0.516	65	0.412	51.39
T0838TS442_3-D1.rsa	26.712	0.402	0.105	0.493	183	0.146	76.36
T0838TS120_5-D1.rsa	26.712	0.412	0.1	0.488	181	0.148	72
T0838TS132_1-D1.rsa	26.667	0.385	0.214	0.402	94	0.284	60.04
T0838TS156_5-D1.rsa	26.471	0.342	0.078	0.58	141	0.188	61.56
T0838TS008_5-D1.rsa	26.471	0.391	0.095	0.514	125	0.212	70.33
T0838TS335_5-D1.rsa	26.471	0.403	0.091	0.506	123	0.215	66.67
T0838TS499_1-D1.rsa	26.471	0.395	0.107	0.498	121	0.219	58.22
T0838TS322_4-D1.rsa	26.4	0.012	0.304	0.684	225	0.117	54.48
T0838TS110_2-D1.rsa	26.4	0.015	0.353	0.632	208	0.127	52.74
T0838TS499_4-D1.rsa	26.263	0.24	0.191	0.569	164	0.16	66.06
T0838TS156_3-D1.rsa	26.263	0.229	0.212	0.559	161	0.163	69.18
T0838TS479_1-D1.rsa	26	0.442	0.12	0.438	110	0.236	89.54
T0838TS499_5-D1.rsa	25.85	0.365	0.102	0.534	199	0.13	83.4
T0838TS345_3-D1.rsa	25.85	0.375	0.097	0.528	197	0.131	78.4
T0838TS237_2-D1.rsa	25.714	0.423	0.017	0.559	226	0.114	46.16
T0838TS120_4-D1.rsa	25.424	0	0.304	0.696	149	0.171	62.38
T0838TS155_4-D1.rsa	25.424	0.019	0.327	0.654	140	0.182	62.5
T0838TS433_5-D1.rsa	25.253	0.212	0.181	0.608	175	0.144	64.32

T0838TS401_2-D1.rsa	25	0.313	0.049	0.638	155	0.161	58.33
T0838TS263_1-D1.rsa	25	0.367	0.116	0.518	130	0.192	79.58
T0838TS335_2-D1.rsa	25	0.442	0.112	0.446	112	0.223	90.04
T0838TS024_1-D1.rsa	25	0.374	0.049	0.576	140	0.179	65.89
T0838TS034_1-D1.rsa	25	0.412	0.14	0.449	109	0.229	70.78
T0838TS296_5-D1.rsa	25	0.026	0.079	0.895	102	0.245	24.78
T0838TS235_2-D1.rsa	24.752	0.234	0.109	0.657	211	0.117	63.94
T0838TS328_2-D1.rsa	24.675	0.388	0.121	0.491	110	0.224	82.5
T0838TS133_3-D1.rsa	24.675	0.411	0.121	0.469	105	0.235	80.83
T0838TS133_4-D1.rsa	24.675	0.424	0.125	0.451	101	0.244	78.33
T0838TS282_4-D1.rsa	24.675	0.379	0.125	0.496	111	0.222	73.45
T0838TS032_1-D1.rsa	24.675	0.429	0.134	0.438	98	0.252	75.59
T0838TS110_3-D1.rsa	24.571	0.433	0.02	0.547	221	0.111	43.5
T0838TS276_2-D1.rsa	24.359	0.351	0.128	0.52	154	0.158	60.24
T0838TS049_4-D1.rsa	24.359	0.395	0.128	0.476	141	0.173	58.16
T0838TS080_4-D1.rsa	24.242	0.198	0.139	0.663	191	0.127	54.17
T0838TS479_2-D1.rsa	24	0.058	0.058	0.884	122	0.197	48.91
T0838TS064_2-D1.rsa	24	0.072	0.384	0.543	75	0.32	73.55
T0838TS403_4-D1.rsa	24	0.036	0.283	0.681	94	0.255	36.77
T0838TS346_1-D1.rsa	23.81	0.256	0.145	0.598	140	0.17	43.91
T0838TS420_2-D1.rsa	23.81	0.325	0.197	0.479	112	0.213	61.22
T0838TS479_5-D1.rsa	23.684	0.428	0.015	0.557	254	0.093	64.75
T0838TS277_2-D1.rsa	23.457	0.428	0.077	0.496	226	0.104	65.07
T0838TS006_4-D1.rsa	23.214	0.167	0.119	0.714	90	0.258	21.23
T0838TS482_1-D1.rsa	23.077	0.409	0.101	0.49	145	0.159	58.85
T0838TS258_3-D1.rsa	23.077	0.412	0.111	0.476	141	0.164	55.3
T0838TS442_4-D1.rsa	23.026	0.463	0.015	0.522	238	0.097	62.06
T0838TS042_2-D1.rsa	22.84	0.39	0.099	0.511	233	0.098	44.26
T0838TS326_2-D1.rsa	22.772	0.084	0.019	0.897	288	0.079	28.97
T0838TS310_3-D1.rsa	22.5	0	0.404	0.596	68	0.331	46.49
T0838TS338_4-D1.rsa	22.5	0	0.053	0.947	108	0.208	20.18
T0838TS110_4-D1.rsa	22.368	0.286	0.051	0.663	220	0.102	51.35
T0838TS345_2-D1.rsa	22.286	0.47	0.025	0.505	204	0.109	40.28
T0838TS203_5-D1.rsa	22.286	0.512	0.02	0.468	189	0.118	42.88
T0838TS442_1-D1.rsa	22.034	0.009	0.047	0.944	202	0.109	38.44
T0838TS235_1-D1.rsa	22.034	0.056	0.136	0.808	173	0.127	51.52
T0838TS042_3-D1.rsa	22	0.434	0.116	0.45	113	0.195	87.15
T0838TS067_5-D1.rsa	21.795	0.412	0.057	0.53	157	0.139	58.42
T0838TS333_3-D1.rsa	21.711	0.5	0.015	0.485	221	0.098	65.46
T0838TS493_1-D1.rsa	21.605	0.419	0.09	0.491	224	0.096	73.45
T0838TS349_1-D1.rsa	21.605	0.395	0.092	0.513	234	0.092	64.79
T0838TS300_5-D1.rsa	21.605	0.417	0.079	0.504	230	0.094	53.31
T0838TS251_5-D1.rsa	21.053	0.423	0.015	0.561	256	0.082	65.35
T0838TS445_3-D1.rsa	21.053	0.498	0.015	0.487	222	0.095	64.97
T0838TS116_3-D1.rsa	21.053	0.438	0.082	0.479	105	0.201	65.3
T0838TS251_3-D1.rsa	20.988	0.425	0.09	0.485	221	0.095	75.66
T0838TS436_5-D1.rsa	20.988	0.425	0.088	0.487	222	0.095	71.8

T0838TS448_4-D1.rsa	20.93	0.403	0.124	0.473	134	0.156	75.8
T0838TS268_5-D1.rsa	20.792	0.199	0.125	0.676	217	0.096	53.82
T0838TS335_4-D1.rsa	20.779	0.379	0.134	0.487	109	0.191	76.55
T0838TS420_5-D1.rsa	20.588	0.189	0.008	0.802	195	0.106	21
T0838TS260_5-D1.rsa	20.513	0.429	0.152	0.419	124	0.165	59.63
T0838TS401_5-D1.rsa	20	0.537	0.02	0.443	179	0.112	46.78
T0838TS333_5-D1.rsa	20	0.051	0.391	0.558	77	0.26	73.91
T0838TS156_1-D1.rsa	20	0.065	0.196	0.739	102	0.196	17.57
T0838TS210_4-D1.rsa	19.767	0.304	0.085	0.611	173	0.114	70.94
T0838TS157_5-D1.rsa	19.737	0.434	0.024	0.542	247	0.08	62.61
T0838TS493_5-D1.rsa	19.737	0.428	0.024	0.548	250	0.079	63.54
T0838TS251_1-D1.rsa	19.737	0.439	0.007	0.555	253	0.078	51.86
T0838TS391_2-D1.rsa	19.481	0.371	0.129	0.5	112	0.174	78.93
T0838TS401_4-D1.rsa	19.298	0.429	0.1	0.47	103	0.187	80.82
T0838TS414_1-D1.rsa	19.298	0.443	0.087	0.47	103	0.187	80.36
T0838TS345_1-D1.rsa	19.136	0.417	0.094	0.489	223	0.086	69.43
T0838TS097_3-D1.rsa	19.118	0.395	0.074	0.531	129	0.148	67.89
T0838TS155_3-D1.rsa	19.079	0.48	0.024	0.496	226	0.084	65.46
T0838TS263_4-D1.rsa	18.605	0.417	0.11	0.473	134	0.139	81.36
T0838TS425_5-D1.rsa	18.605	0.389	0.127	0.484	137	0.136	71.38
T0838TS038_4-D1.rsa	18.519	0.379	0.101	0.52	237	0.078	62.8
T0838TS251_2-D1.rsa	18.421	0.441	0.029	0.531	242	0.076	68.09
T0838TS237_3-D1.rsa	18.421	0.361	0.133	0.506	168	0.11	67.55
T0838TS276_5-D1.rsa	17.901	0.425	0.099	0.476	217	0.082	64.24
T0838TS251_4-D1.rsa	17.763	0.452	0.024	0.524	239	0.074	61.18
T0838TS493_2-D1.rsa	17.692	0.269	0.234	0.498	228	0.078	69.94
T0838TS260_4-D1.rsa	17.544	0.443	0.078	0.479	105	0.167	82.42
T0838TS326_3-D1.rsa	17.544	0.452	0.091	0.457	100	0.175	75.57
T0838TS077_3-D1.rsa	17.442	0.442	0.127	0.431	122	0.143	75
T0838TS282_2-D1.rsa	17.284	0.432	0.092	0.476	217	0.08	70.58
T0838TS300_1-D1.rsa	16.923	0.262	0.218	0.52	238	0.071	54.39
T0838TS479_4-D1.rsa	16.154	0.255	0.207	0.537	246	0.066	65.61
T0838TS282_3-D1.rsa	16	0	0.225	0.775	107	0.15	41.85
T0838TS347_4-D1.rsa	16	0.051	0.319	0.63	87	0.184	67.75
T0838TS349_4-D1.rsa	15.789	0.441	0.013	0.546	249	0.063	53.62
T0838TS483_3-D1.rsa	15.789	0.454	0.029	0.518	236	0.067	64.09
T0838TS442_2-D1.rsa	15.789	0.325	0.105	0.569	189	0.084	97.97
T0838TS442_5-D1.rsa	15.789	0.352	0.123	0.524	174	0.091	99.02
T0838TS457_2-D1.rsa	15.789	0.443	0.082	0.475	104	0.152	80.48
T0838TS041_1-D1.rsa	15.789	0.447	0.082	0.47	103	0.153	80.48
T0838TS024_3-D1.rsa	15.116	0.399	0.131	0.47	133	0.114	71.47
T0838TS428_5-D1.rsa	14.894	0.358	0.171	0.471	121	0.123	84.73
T0838TS155_5-D1.rsa	14.615	0.271	0.238	0.491	225	0.065	76.72
T0838TS157_2-D1.rsa	14.474	0.271	0.072	0.657	218	0.066	98.34
T0838TS110_5-D1.rsa	14.474	0.28	0.102	0.617	205	0.071	96.61
T0838TS157_1-D1.rsa	14.474	0.346	0.123	0.53	176	0.082	99.25
T0838TS038_5-D1.rsa	13.953	0.385	0.117	0.498	141	0.099	76.86

T0838TS237_4-D1.rsa	13.846	0.262	0.234	0.504	231	0.06	76.22
T0838TS436_4-D1.rsa	13.846	0.266	0.227	0.507	232	0.06	73.56
T0838TS345_4-D1.rsa	13.158	0.328	0.127	0.545	181	0.073	98.12
T0838TS228_3-D1.rsa	13.158	0.331	0.142	0.527	175	0.075	97.59
T0838TS155_2-D1.rsa	13.158	0.349	0.123	0.527	175	0.075	98.87
T0838TS326_5-D1.rsa	13.077	0.266	0.225	0.509	233	0.056	75.89
T0838TS145_1-D1.rsa	12.766	0.362	0.16	0.479	123	0.104	83.85
T0838TS433_4-D1.rsa	12.308	0.277	0.245	0.478	219	0.056	71.61
T0838TS156_4-D1.rsa	11.842	0.31	0.099	0.59	196	0.06	83.13
T0838TS111_4-D1.rsa	11.842	0.355	0.123	0.521	173	0.068	98.42
T0838TS268_4-D1.rsa	10.526	0.322	0.108	0.569	189	0.056	99.4
T0838TS042_5-D1.rsa	10.526	0.434	0.091	0.475	104	0.101	82.42
T0838TS263_2-D1.rsa	6.383	0.331	0.175	0.494	127	0.05	84.83
T0823TS414_4-D1.rsa	84.211	0.252	0.09	0.658	73	1.154	18.92
T0823TS041_5-D1.rsa	80	0.203	0.094	0.703	97	0.825	12.14
T0823TS145_2-D1.rsa	79.167	0.037	0.019	0.944	102	0.776	16.67
T0823TS452_3-D1.rsa	76.471	0.524	0	0.476	60	1.275	32.74
T0823TS156_4-D1.rsa	76	0.275	0.022	0.703	97	0.784	11.23
T0823TS237_2-D1.rsa	70.588	0.286	0	0.714	90	0.784	31.75
T0823TS184_2-D1.rsa	70.588	0.683	0	0.317	40	1.765	40.28
T0823TS420_1-D1.rsa	69.767	0.299	0.164	0.537	72	0.969	19.4
T0823TS145_4-D1.rsa	67.188	0.066	0	0.934	127	0.529	11.21
T0823TS251_2-D1.rsa	64.286	0	0.344	0.656	86	0.748	24.81
T0823TS346_1-D1.rsa	64	0.203	0.029	0.768	106	0.604	12.32
T0823TS133_5-D1.rsa	62.857	0.59	0.043	0.368	86	0.731	12.07
T0823TS410_5-D1.rsa	61.538	0.364	0.115	0.521	86	0.716	39.4
T0823TS335_5-D1.rsa	60.714	0	0.267	0.733	96	0.632	15.84
T0823TS038_3-D1.rsa	54.93	0.369	0.113	0.518	87	0.631	20.67
T0823TS454_4-D1.rsa	54.167	0.009	0.361	0.63	68	0.797	55.32
T0823TS237_1-D1.rsa	53.571	0	0.405	0.595	78	0.687	51.53
T0823TS268_5-D1.rsa	53.571	0.381	0	0.619	78	0.687	17.46
T0823TS216_3-D1.rsa	52.632	0.225	0.126	0.649	72	0.731	36.71
T0823TS436_5-D1.rsa	51.948	0.21	0.058	0.732	164	0.317	9.17
T0823TS345_4-D1.rsa	51.923	0.248	0.079	0.673	111	0.468	12.82
T0823TS381_3-D1.rsa	51.786	0.317	0.048	0.635	80	0.647	24.21
T0823TS277_2-D1.rsa	51.163	0.187	0.06	0.754	101	0.507	34.52
T0823TS171_3-D1.rsa	50.704	0.208	0.167	0.625	105	0.483	13.3
T0823TS251_3-D1.rsa	50	0.212	0.152	0.636	105	0.476	42.88
T0823TS110_3-D1.rsa	50	0.291	0.152	0.558	92	0.543	39.24
T0823TS268_3-D1.rsa	50	0.207	0.072	0.721	80	0.625	25.9
T0823TS410_3-D1.rsa	48.837	0.343	0.03	0.627	84	0.581	42.72
T0823TS300_4-D1.rsa	48.438	0.081	0.015	0.904	123	0.394	13.6
T0823TS414_3-D1.rsa	48.077	0.291	0.145	0.564	93	0.517	17.25
T0823TS381_5-D1.rsa	47.368	0.261	0	0.739	82	0.578	28.15
T0823TS268_2-D1.rsa	47.368	0.198	0.036	0.766	85	0.557	31.31
T0823TS499_3-D1.rsa	47.368	0.241	0.37	0.389	42	1.128	61.11
T0823TS184_3-D1.rsa	47.059	0.548	0	0.452	57	0.826	42.46

T0823TS171_2-D1.rsa	46.429	0.016	0.198	0.786	99	0.469	18.45
T0823TS117_1-D1.rsa	46.429	0.214	0.063	0.722	91	0.51	21.23
T0823TS210_2-D1.rsa	46.429	0	0.153	0.847	111	0.418	14.12
T0823TS335_2-D1.rsa	46.429	0	0.359	0.641	84	0.553	17.75
T0823TS184_4-D1.rsa	46.154	0.297	0.061	0.642	106	0.435	16.93
T0823TS038_1-D1.rsa	45.833	0	0.445	0.555	61	0.751	46.36
T0823TS268_1-D1.rsa	44.737	0.288	0.108	0.604	67	0.668	39.64
T0823TS110_4-D1.rsa	44.231	0.327	0.036	0.636	105	0.421	40.51
T0823TS008_2-D1.rsa	44.186	0.313	0.06	0.627	84	0.526	34.52
T0823TS381_4-D1.rsa	44	0	0.159	0.841	116	0.379	14.86
T0823TS263_2-D1.rsa	43.75	0.055	0.318	0.627	69	0.634	58.18
T0823TS038_2-D1.rsa	43.662	0.387	0	0.613	103	0.424	18.75
T0823TS251_5-D1.rsa	42.857	0	0.382	0.618	81	0.529	60.69
T0823TS263_1-D1.rsa	42.857	0	0.412	0.588	77	0.557	58.4
T0823TS008_4-D1.rsa	42.857	0.015	0.298	0.687	90	0.476	55.53
T0823TS228_4-D1.rsa	42.857	0	0.26	0.74	97	0.442	16.79
T0823TS117_3-D1.rsa	42.857	0.111	0.063	0.825	104	0.412	19.05
T0823TS277_3-D1.rsa	42.105	0.297	0.081	0.622	69	0.61	40.99
T0823TS110_5-D1.rsa	41.667	0.091	0.255	0.655	72	0.579	60
T0823TS414_2-D1.rsa	41.667	0	0.528	0.472	51	0.817	68.29
T0823TS160_3-D1.rsa	41.071	0.143	0.159	0.698	88	0.467	33.53
T0823TS184_5-D1.rsa	41.071	0.159	0.056	0.786	99	0.415	16.86
T0823TS300_3-D1.rsa	40.845	0.03	0.101	0.869	146	0.28	12.18
T0823TS011_2-D1.rsa	40.845	0.399	0.131	0.47	79	0.517	40.87
T0823TS050_1-D1.rsa	40.845	0.464	0.113	0.423	71	0.575	34.13
T0823TS228_3-D1.rsa	40.625	0	0.228	0.772	105	0.387	13.6
T0823TS038_4-D1.rsa	39.726	0.741	0	0.259	66	0.602	52.06
T0823TS011_3-D1.rsa	39.583	0.082	0.255	0.664	73	0.542	62.95
T0823TS216_5-D1.rsa	39.535	0.455	0.075	0.47	63	0.628	39.55
T0823TS160_5-D1.rsa	39.474	0.36	0.108	0.532	59	0.669	53.15
T0823TS110_1-D1.rsa	39.474	0.25	0.241	0.509	55	0.718	69.21
T0823TS300_2-D1.rsa	39.063	0.118	0.162	0.721	98	0.399	15.44
T0823TS479_5-D1.rsa	37.5	0.044	0.11	0.846	115	0.326	57.17
T0823TS041_2-D1.rsa	37.5	0.14	0	0.86	117	0.321	11.77
T0823TS008_1-D1.rsa	36.842	0.27	0.081	0.649	72	0.512	55.63
T0823TS210_3-D1.rsa	36.62	0.405	0.131	0.464	78	0.469	40.06
T0823TS452_2-D1.rsa	36.538	0.273	0.048	0.679	112	0.326	19.78
T0823TS448_1-D1.rsa	36	0	0.232	0.768	106	0.34	62.14
T0823TS492_4-D1.rsa	36	0	0.297	0.703	97	0.371	49.82
T0823TS263_4-D1.rsa	36	0.072	0.326	0.601	83	0.434	55.8
T0823TS251_1-D1.rsa	35.965	0.295	0.138	0.567	123	0.292	43.66
T0823TS300_1-D1.rsa	35.938	0.015	0.412	0.574	78	0.461	64.89
T0823TS345_1-D1.rsa	35.714	0	0.427	0.573	75	0.476	55.53
T0823TS145_3-D1.rsa	35.616	0.741	0	0.259	66	0.54	58.43
T0823TS041_1-D1.rsa	35.417	0.064	0.318	0.618	68	0.521	60.68
T0823TS349_4-D1.rsa	35.345	0.621	0	0.379	97	0.364	58.69
T0823TS436_2-D1.rsa	35	0.018	0	0.982	112	0.313	23.46

T0823TS117_4-D1.rsa	35	0.018	0.351	0.632	72	0.486	48.47
T0823TS452_1-D1.rsa	35	0	0.421	0.579	66	0.53	50.22
T0823TS216_4-D1.rsa	35	0.018	0.404	0.579	66	0.53	50.22
T0823TS452_4-D1.rsa	34.884	0.284	0.045	0.672	90	0.388	42.91
T0823TS145_5-D1.rsa	34.884	0.343	0.075	0.582	78	0.447	50.56
T0823TS499_1-D1.rsa	34.737	0.513	0.051	0.436	103	0.337	63.14
T0823TS251_4-D1.rsa	34.375	0.081	0.301	0.618	84	0.409	60.29
T0823TS345_3-D1.rsa	34.375	0.088	0.074	0.838	114	0.302	13.42
T0823TS133_3-D1.rsa	34.247	0.741	0	0.259	66	0.519	55.69
T0823TS268_4-D1.rsa	33.929	0.159	0.206	0.635	80	0.424	38.69
T0823TS492_1-D1.rsa	33.684	0.487	0.059	0.453	107	0.315	48.62
T0823TS448_3-D1.rsa	33.621	0.578	0	0.422	108	0.311	67.19
T0823TS499_2-D1.rsa	33.621	0.645	0	0.355	91	0.369	52.34
T0823TS216_2-D1.rsa	32.813	0.096	0.118	0.787	107	0.307	13.05
T0823TS335_3-D1.rsa	32.759	0.684	0	0.316	81	0.404	66.89
T0823TS277_4-D1.rsa	32.558	0.302	0.195	0.503	85	0.383	72.19
T0823TS011_5-D1.rsa	32.558	0.239	0	0.761	102	0.319	36.57
T0823TS145_1-D1.rsa	32.5	0.018	0.272	0.711	81	0.401	43.42
T0823TS335_1-D1.rsa	32.394	0.387	0.024	0.589	99	0.327	42.95
T0823TS133_1-D1.rsa	32.394	0.339	0	0.661	111	0.292	16.99
T0823TS133_2-D1.rsa	32.143	0.135	0.206	0.659	83	0.387	45.24
T0823TS133_4-D1.rsa	32	0.072	0.181	0.746	103	0.311	19.02
T0823TS452_5-D1.rsa	31.897	0.645	0	0.355	91	0.351	57.03
T0823TS436_1-D1.rsa	31.897	0.691	0	0.309	79	0.404	66.5
T0823TS381_2-D1.rsa	31.579	0.09	0	0.91	101	0.313	37.16
T0823TS454_3-D1.rsa	31.579	0.25	0.5	0.25	27	1.17	93.75
T0823TS237_5-D1.rsa	31.395	0.249	0.183	0.568	96	0.327	72.34
T0823TS228_5-D1.rsa	31.395	0.325	0.225	0.45	76	0.413	76.33
T0823TS171_4-D1.rsa	31.034	0.652	0	0.348	89	0.349	64.65
T0823TS184_1-D1.rsa	30.986	0.464	0.161	0.375	63	0.492	62.98
T0823TS345_5-D1.rsa	30.526	0.504	0.059	0.436	103	0.296	51.16
T0823TS160_1-D1.rsa	30.357	0.19	0	0.81	102	0.298	29.96
T0823TS263_5-D1.rsa	30.357	0.206	0.063	0.73	92	0.33	38.49
T0823TS263_3-D1.rsa	30.357	0.167	0.325	0.508	64	0.474	53.77
T0823TS381_1-D1.rsa	30.233	0.391	0.183	0.426	72	0.42	73.97
T0823TS420_4-D1.rsa	30.172	0.684	0	0.316	81	0.372	67.87
T0823TS041_3-D1.rsa	30	0	0.272	0.728	83	0.361	46.49
T0823TS110_2-D1.rsa	30	0	0.325	0.675	77	0.39	42.33
T0823TS011_4-D1.rsa	30	0.018	0.36	0.623	71	0.423	48.25
T0823TS479_4-D1.rsa	29.508	0.019	0.395	0.586	123	0.24	65.3
T0823TS492_2-D1.rsa	29.508	0.048	0.386	0.567	119	0.248	66.54
T0823TS492_3-D1.rsa	29.412	0.453	0.099	0.449	109	0.27	64.33
T0823TS160_4-D1.rsa	28.947	0.286	0.184	0.53	115	0.252	62.9
T0823TS436_3-D1.rsa	28.448	0.605	0	0.395	101	0.282	61.03
T0823TS410_2-D1.rsa	28	0.094	0.341	0.565	78	0.359	62.14
T0823TS420_2-D1.rsa	28	0.072	0.072	0.855	118	0.237	16.85
T0823TS349_5-D1.rsa	28	0.08	0.297	0.623	86	0.326	65.4

T0823TS345_2-D1.rsa	27.907	0.337	0.207	0.456	77	0.362	69.67
T0823TS008_3-D1.rsa	27.907	0.358	0.082	0.56	75	0.372	52.8
T0823TS206_1-D1.rsa	27.586	0	0.154	0.846	121	0.228	52.8
T0823TS008_5-D1.rsa	27.368	0.551	0.038	0.411	97	0.282	62.82
T0823TS349_1-D1.rsa	26.667	0.389	0.231	0.38	89	0.3	56.52
T0823TS448_2-D1.rsa	26.471	0.407	0.107	0.486	118	0.224	66.22
T0823TS410_1-D1.rsa	26.316	0.25	0.5	0.25	27	0.975	94.44
T0823TS479_3-D1.rsa	25	0.407	0.095	0.498	121	0.207	71.11
T0823TS171_5-D1.rsa	24.419	0.308	0.16	0.533	90	0.271	68.49
T0823TS160_2-D1.rsa	24.419	0.349	0.172	0.479	81	0.301	70.71
T0823TS228_2-D1.rsa	24	0.058	0.304	0.638	88	0.273	56.16
T0823TS420_5-D1.rsa	24	0	0.087	0.913	126	0.19	17.39
T0823TS479_1-D1.rsa	23.729	0.065	0.271	0.664	142	0.167	59.35
T0823TS117_2-D1.rsa	23.684	0.452	0.024	0.524	239	0.099	64.09
T0823TS448_5-D1.rsa	23.529	0.444	0.107	0.449	109	0.216	68.11
T0823TS237_4-D1.rsa	23.377	0.379	0.125	0.496	111	0.211	70.48
T0823TS156_2-D1.rsa	23	0.422	0.068	0.51	128	0.18	35.76
T0823TS499_4-D1.rsa	21.795	0.453	0.149	0.399	118	0.185	59.9
T0823TS349_3-D1.rsa	20.588	0.395	0.025	0.58	141	0.146	11.44
T0823TS454_5-D1.rsa	20	0.036	0.348	0.616	85	0.235	69.38
T0823TS277_5-D1.rsa	19.767	0.428	0.124	0.449	127	0.156	78.89
T0823TS228_1-D1.rsa	19.298	0.47	0.091	0.438	96	0.201	79.91
T0823TS038_5-D1.rsa	16	0.029	0.174	0.797	110	0.145	52.35
T0823TS479_2-D1.rsa	14.894	0.327	0.198	0.475	122	0.122	84.73
T0823TS499_5-D1.rsa	14.894	0.35	0.206	0.444	114	0.131	84.73
T0823TS448_4-D1.rsa	14.035	0.457	0.078	0.466	102	0.138	81.28
T0783TS169_2-D2.rsa	86.842	0.505	0	0.495	55	1.579	17.57
T0783TS064_2-D2.rsa	80.282	0.637	0	0.363	61	1.316	13.78
T0783TS216_4-D2.rsa	76.471	0.357	0	0.643	81	0.944	18.06
T0783TS228_4-D2.rsa	76	0.366	0.02	0.614	248	0.306	5.63
T0783TS326_2-D2.rsa	75	0	0.176	0.824	108	0.694	43.13
T0783TS360_3-D2.rsa	70.833	0.019	0.491	0.491	53	1.336	70.37
T0783TS301_3-D2.rsa	69.767	0.44	0.045	0.515	69	1.011	15.86
T0783TS301_1-D2.rsa	69.231	0.509	0.036	0.455	75	0.923	13.29
T0783TS097_3-D2.rsa	68.75	0.463	0.029	0.507	69	0.996	10.11
T0783TS326_1-D2.rsa	68	0.072	0.203	0.725	100	0.68	15.76
T0783TS290_4-D2.rsa	67.442	0.56	0	0.44	59	1.143	16.6
T0783TS026_5-D2.rsa	64.789	0.196	0.048	0.756	127	0.51	14.1
T0783TS026_3-D2.rsa	64.286	0.015	0.061	0.924	121	0.531	11.45
T0783TS064_1-D2.rsa	64.286	0.222	0.214	0.563	71	0.905	17.46
T0783TS290_3-D2.rsa	62.5	0	0.528	0.472	51	1.225	73.84
T0783TS499_4-D2.rsa	62.5	0	0.537	0.463	50	1.25	56.02
T0783TS049_4-D2.rsa	61.616	0.181	0	0.819	236	0.261	10.42
T0783TS157_1-D2.rsa	61.538	0.255	0.024	0.721	119	0.517	69.62
T0783TS335_2-D2.rsa	60.714	0	0.298	0.702	92	0.66	13.93
T0783TS067_2-D2.rsa	60.714	0.238	0.159	0.603	76	0.799	19.84
T0783TS067_5-D2.rsa	60.714	0	0.557	0.443	58	1.047	15.65

T0783TS401_2-D2.rsa	60.526	0.288	0.081	0.631	70	0.865	34.69
T0783TS439_2-D2.rsa	60.465	0.187	0.045	0.769	103	0.587	41.98
T0783TS414_3-D2.rsa	60	0.275	0.072	0.652	90	0.667	13.22
T0783TS296_5-D2.rsa	58.929	0.143	0.31	0.548	69	0.854	17.66
T0783TS420_1-D2.rsa	58.333	0.019	0.574	0.407	44	1.326	73.61
T0783TS065_2-D2.rsa	58.333	0	0.5	0.5	55	1.061	26.82
T0783TS347_2-D2.rsa	58.333	0.082	0.264	0.655	72	0.81	24.32
T0783TS358_3-D2.rsa	58.14	0.336	0.157	0.507	68	0.855	25.19
T0783TS065_1_2-D2.rsa	57.895	0.324	0.018	0.658	73	0.793	23.42
T0783TS235_2-D2.rsa	57.143	0	0.069	0.931	122	0.468	16.03
T0783TS360_5-D2.rsa	55.882	0.5	0	0.5	63	0.887	35.12
T0783TS160_4-D2.rsa	55.769	0.303	0.085	0.612	101	0.552	74.84
T0783TS290_2-D2.rsa	55.357	0.413	0	0.587	74	0.748	18.25
T0783TS184_3-D2.rsa	55.263	0.204	0	0.796	86	0.643	16.9
T0783TS310_5-D2.rsa	54.167	0	0.583	0.417	45	1.204	75.93
T0783TS282_5-D2.rsa	54.167	0	0.5	0.5	54	1.003	55.09
T0783TS282_3-D2.rsa	54.167	0.036	0.227	0.736	81	0.669	19.55
T0783TS454_2-D2.rsa	53.571	0.015	0.336	0.649	85	0.63	15.46
T0783TS322_4-D2.rsa	53.488	0.291	0.045	0.664	89	0.601	42.16
T0783TS296_3-D2.rsa	52.941	0.587	0	0.413	52	1.018	32.14
T0783TS184_4-D2.rsa	52.113	0.53	0	0.47	79	0.66	15.22
T0783TS333_2-D2.rsa	52.113	0.452	0.036	0.512	86	0.606	15.38
T0783TS499_5-D2.rsa	52	0.094	0.181	0.725	100	0.52	14.86
T0783TS349_1-D2.rsa	51.282	0.385	0.054	0.561	166	0.309	11.29
T0783TS347_4-D2.rsa	50	0	0.305	0.695	91	0.549	18.51
T0783TS080_2-D2.rsa	50	0.159	0.063	0.778	98	0.51	18.65
T0783TS335_1-D2.rsa	50	0.091	0.364	0.545	60	0.833	48.64
T0783TS132_2-D2.rsa	50	0.056	0.676	0.269	29	1.724	77.55
T0783TS097_4-D2.rsa	50	0.243	0.027	0.73	81	0.617	19.37
T0783TS338_5-D2.rsa	50	0.207	0	0.793	88	0.568	14.41
T0783TS132_5-D2.rsa	50	0.234	0.171	0.595	66	0.758	20.5
T0783TS368_4-D2.rsa	50	0.619	0	0.381	48	1.042	36.91
T0783TS438_1-D2.rsa	50	0.278	0.151	0.571	72	0.694	20.24
T0783TS008_1-D2.rsa	48.529	0.346	0.037	0.617	150	0.324	15.33
T0783TS358_1-D2.rsa	48.438	0.096	0.353	0.551	75	0.646	14.15
T0783TS204_5-D2.rsa	48.214	0.317	0	0.683	86	0.561	24.01
T0783TS162_1-D2.rsa	48.214	0.111	0.175	0.714	90	0.536	15.87
T0783TS340_1-D2.rsa	48.077	0.212	0.255	0.533	88	0.546	14.24
T0783TS006_5-D2.rsa	48.077	0.352	0.067	0.582	96	0.501	15.35
T0783TS097_2-D2.rsa	48	0.297	0.181	0.522	72	0.667	13.77
T0783TS241_3_2-D2.rsa	47.917	0	0.455	0.545	60	0.799	30.45
T0783TS067_3-D2.rsa	47.887	0.488	0	0.512	86	0.557	15.22
T0783TS117_3-D2.rsa	47.368	0.304	0.078	0.618	134	0.353	53.57
T0783TS452_2-D2.rsa	47.368	0.144	0.072	0.784	87	0.544	18.69
T0783TS006_2-D2.rsa	47.368	0.252	0.126	0.622	69	0.686	37.84
T0783TS042_4-D2.rsa	47.368	0.27	0.018	0.712	79	0.6	35.13
T0783TS268_2-D2.rsa	47.368	0.306	0	0.694	77	0.615	17.79

T0783TS049_5-D2.rsa	46.429	0	0.122	0.878	115	0.404	46.76
T0783TS301_4-D2.rsa	46.429	0.159	0.222	0.619	78	0.595	47.62
T0783TS006_1-D2.rsa	46.429	0	0.282	0.718	94	0.494	15.46
T0783TS118_4-D2.rsa	46.429	0.015	0.366	0.618	81	0.573	17.37
T0783TS310_3-D2.rsa	46.429	0.341	0.079	0.579	73	0.636	25.2
T0783TS414_1-D2.rsa	45.313	0.059	0.221	0.721	98	0.462	15.99
T0783TS335_3-D2.rsa	45.313	0.169	0.125	0.706	96	0.472	12.13
T0783TS169_3-D2.rsa	45.313	0.132	0.235	0.632	86	0.527	13.6
T0783TS482_1-D2.rsa	45.313	0.154	0.235	0.61	83	0.546	12.5
T0783TS338_2-D2.rsa	45.205	0.773	0	0.227	58	0.779	41.18
T0783TS425_2-D2.rsa	45.07	0.512	0.113	0.375	63	0.715	33.81
T0783TS452_4-D2.rsa	45	0.088	0.07	0.842	96	0.469	26.75
T0783TS499_2-D2.rsa	44.737	0.225	0.036	0.739	82	0.546	25
T0783TS345_1-D2.rsa	44.186	0.216	0.09	0.694	93	0.475	44.22
T0783TS042_1-D2.rsa	44.186	0.261	0.134	0.604	81	0.546	25.56
T0783TS065_3-D2.rsa	44.118	0.651	0	0.349	44	1.003	42.46
T0783TS184_1-D2.rsa	43.75	0.059	0.199	0.743	101	0.433	12.68
T0783TS434_1-D2.rsa	43.75	0.051	0.25	0.699	95	0.461	14.34
T0783TS300_3-D2.rsa	43.421	0.307	0	0.693	230	0.189	19.13
T0783TS442_5-D2.rsa	43.103	0	0.14	0.86	123	0.35	60.49
T0783TS041_2-D2.rsa	42.982	0.267	0.134	0.599	130	0.331	53.46
T0783TS381_3-D2.rsa	42.857	0.19	0.23	0.579	73	0.587	47.62
T0783TS067_1-D2.rsa	42.857	0.183	0.119	0.698	88	0.487	25
T0783TS368_3-D2.rsa	42.857	0	0.397	0.603	79	0.542	17.94
T0783TS401_1-D2.rsa	42.857	0.373	0	0.627	79	0.542	17.46
T0783TS184_2-D2.rsa	42.5	0	0.263	0.737	84	0.506	19.3
T0783TS358_2-D2.rsa	42.5	0	0.368	0.632	72	0.59	16.01
T0783TS345_3-D2.rsa	42.188	0.051	0.279	0.669	91	0.464	13.79
T0783TS162_5-D2.rsa	42.105	0.261	0	0.739	82	0.513	40.54
T0783TS193_1-D2.rsa	41.667	0.329	0.094	0.577	123	0.339	70.05
T0783TS038_5-D2.rsa	41.379	0	0.098	0.902	129	0.321	58.22
T0783TS322_5-D2.rsa	41.176	0.317	0.008	0.675	164	0.251	11.89
T0783TS210_4-D2.rsa	41.176	0.387	0.041	0.572	139	0.296	14
T0783TS216_2-D2.rsa	41.176	0.587	0	0.413	52	0.792	46.03
T0783TS347_5-D2.rsa	41.096	0.757	0	0.243	62	0.663	45.39
T0783TS160_3-D2.rsa	40.909	0.098	0.466	0.436	129	0.317	50.25
T0783TS340_2-D2.rsa	40.845	0.429	0.143	0.429	72	0.567	40.87
T0783TS277_3-D2.rsa	40.625	0.088	0.25	0.662	90	0.451	12.68
T0783TS044_4-D2.rsa	40.625	0.015	0.324	0.662	90	0.451	13.97
T0783TS333_4-D2.rsa	40.385	0.273	0.212	0.515	85	0.475	19.3
T0783TS064_5-D2.rsa	40	0	0.246	0.754	86	0.465	18.86
T0783TS184_5-D2.rsa	40	0.053	0.36	0.588	67	0.597	25.22
T0783TS097_1-D2.rsa	39.773	0.074	0.432	0.493	146	0.272	27.62
T0783TS277_1-D2.rsa	39.535	0.149	0.187	0.664	89	0.444	14.74
T0783TS116_2-D2.rsa	39.474	0.194	0.143	0.664	144	0.274	33.52
T0783TS414_5-D2.rsa	39.474	0.243	0.036	0.721	80	0.493	38.51
T0783TS064_3-D2.rsa	39.474	0.225	0.108	0.667	74	0.533	23.65

T0783TS080_1-D2.rsa	39.286	0	0.443	0.557	73	0.538	56.11
T0783TS041_1-D2.rsa	39.286	0	0.435	0.565	74	0.531	58.59
T0783TS042_3-D2.rsa	39.286	0.222	0.048	0.73	92	0.427	20.24
T0783TS216_3-D2.rsa	39.286	0.015	0.336	0.649	85	0.462	16.79
T0783TS282_2-D2.rsa	39.286	0.135	0.254	0.611	77	0.51	24.21
T0783TS290_1-D2.rsa	39.063	0	0.346	0.654	89	0.439	13.42
T0783TS080_3-D2.rsa	38.947	0.525	0.051	0.424	100	0.389	55.61
T0783TS349_3-D2.rsa	37.5	0.081	0.405	0.514	152	0.247	35.64
T0783TS160_2-D2.rsa	37.5	0.071	0.419	0.51	151	0.248	35.22
T0783TS049_1-D2.rsa	37.5	0.385	0.085	0.531	113	0.332	71.7
T0783TS038_3-D2.rsa	37.5	0.015	0.441	0.544	74	0.507	70.96
T0783TS268_1-D2.rsa	37.5	0.175	0.183	0.643	81	0.463	29.76
T0783TS204_3-D2.rsa	37.5	0.143	0.27	0.587	74	0.507	40.87
T0783TS282_1-D2.rsa	37.5	0.159	0.357	0.484	61	0.615	46.43
T0783TS368_5-D2.rsa	37.5	0	0.439	0.561	64	0.586	24.12
T0783TS162_4-D2.rsa	37.5	0.071	0.31	0.619	78	0.481	16.07
T0783TS338_1-D2.rsa	37.5	0	0.412	0.588	67	0.56	32.02
T0783TS277_5-D2.rsa	37.5	0	0.43	0.57	65	0.577	31.8
T0783TS452_1-D2.rsa	37.209	0.358	0.075	0.567	76	0.49	44.78
T0783TS381_1-D2.rsa	37.209	0.261	0.03	0.709	95	0.392	37.13
T0783TS050_1-D2.rsa	37.209	0.343	0.134	0.522	70	0.532	24.44
T0783TS210_2-D2.rsa	36.364	0.081	0.351	0.568	168	0.216	53.88
T0783TS326_3-D2.rsa	36.364	0.064	0.443	0.493	146	0.249	42.91
T0783TS153_5-D2.rsa	35.965	0.304	0.152	0.544	118	0.305	58.64
T0783TS310_4-D2.rsa	35.938	0.088	0.265	0.647	88	0.408	12.87
T0783TS044_1-D2.rsa	35.938	0.096	0.279	0.625	85	0.423	15.07
T0783TS335_5-D2.rsa	35.714	0.222	0.183	0.595	75	0.476	39.09
T0783TS132_3-D2.rsa	35.714	0.135	0.325	0.54	68	0.525	44.64
T0783TS162_2-D2.rsa	35.417	0.155	0.373	0.473	52	0.681	63.18
T0783TS364_1-D2.rsa	35.294	0.508	0	0.492	62	0.569	46.43
T0783TS401_3-D2.rsa	35	0	0.14	0.86	98	0.357	20.18
T0783TS032_4_2-D2.rsa	35	0.009	0.377	0.614	70	0.5	29.61
T0783TS454_1-D2.rsa	34.884	0.325	0.201	0.473	80	0.436	68.05
T0783TS338_4-D2.rsa	34.884	0.179	0.067	0.754	101	0.345	24.25
T0783TS268_5-D2.rsa	34.884	0.276	0.067	0.657	88	0.396	24.25
T0783TS268_4-D2.rsa	34.884	0.396	0.037	0.567	76	0.459	25.19
T0783TS032_1-D2.rsa	34.884	0.291	0.134	0.575	77	0.453	20.52
T0783TS235_1-D2.rsa	34.722	0.394	0.085	0.521	111	0.313	71.95
T0783TS144_3-D2.rsa	34.375	0	0.324	0.676	92	0.374	15.62
T0783TS442_3-D2.rsa	34.343	0.208	0.135	0.656	189	0.182	37.59
T0783TS241_2_2-D2.rsa	34.247	0.745	0	0.255	65	0.527	42.74
T0783TS032_2-D2.rsa	34.211	0.241	0.5	0.259	28	1.222	92.59
T0783TS251_2-D2.rsa	34.211	0.25	0.5	0.25	27	1.267	93.98
T0783TS049_3-D2.rsa	34.211	0.27	0.081	0.649	72	0.475	37.16
T0783TS116_5-D2.rsa	33.929	0.19	0.262	0.548	69	0.492	48.41
T0783TS333_3-D2.rsa	33.929	0.151	0.198	0.651	82	0.414	28.18
T0783TS301_5-D2.rsa	33.929	0.175	0.373	0.452	57	0.595	52.18

T0783TS118_5-D2.rsa	33.929	0.183	0.278	0.54	68	0.499	37.5
T0783TS056_1-D2.rsa	33.929	0.175	0.349	0.476	60	0.565	51.98
T0783TS368_2-D2.rsa	33.929	0.135	0.317	0.548	69	0.492	42.86
T0783TS425_4-D2.rsa	33.929	0.135	0.349	0.516	65	0.522	36.91
T0783TS144_5-D2.rsa	33.929	0.151	0.349	0.5	63	0.539	44.44
T0783TS296_1-D2.rsa	33.929	0.19	0.365	0.444	56	0.606	50.79
T0783TS358_4-D2.rsa	33.803	0.375	0.131	0.494	83	0.407	36.7
T0783TS492_2-D2.rsa	33.333	0.107	0.103	0.791	185	0.18	66.88
T0783TS345_5-D2.rsa	33.333	0.437	0.075	0.488	104	0.321	70.94
T0783TS157_5-D2.rsa	33	0.446	0.06	0.494	124	0.266	34.66
T0783TS132_4-D2.rsa	32.813	0.125	0.162	0.713	97	0.338	15.44
T0783TS436_4-D2.rsa	32.653	0.378	0.094	0.528	197	0.166	26.53
T0783TS044_2-D2.rsa	32.558	0.239	0.149	0.612	82	0.397	16.42
T0783TS381_4-D2.rsa	32.5	0	0.386	0.614	70	0.464	47.81
T0783TS452_5-D2.rsa	32.5	0.114	0.088	0.798	91	0.357	17.98
T0783TS268_3-D2.rsa	32.394	0.423	0.167	0.411	69	0.469	51.92
T0783TS263_5-D2.rsa	32.192	0.278	0.054	0.668	248	0.13	58.86
T0783TS118_1-D2.rsa	32.143	0.151	0.341	0.508	64	0.502	43.45
T0783TS457_1-D2.rsa	32.143	0.159	0.294	0.548	69	0.466	42.06
T0783TS011_2-D2.rsa	32	0.006	0.292	0.702	231	0.139	41.11
T0783TS024_1_2-D2.rsa	32	0.021	0.365	0.614	202	0.158	53.42
T0783TS210_3-D2.rsa	32	0.065	0.341	0.594	82	0.39	64.86
T0783TS277_2-D2.rsa	31.897	0.652	0	0.348	89	0.358	58.98
T0783TS117_5-D2.rsa	31.683	0.255	0.146	0.598	192	0.165	74.77
T0783TS169_4-D2.rsa	31.579	0.269	0.444	0.287	31	1.019	93.29
T0783TS347_3-D2.rsa	31.579	0.25	0.5	0.25	27	1.17	93.75
T0783TS153_1-D2.rsa	31.507	0.399	0.102	0.499	185	0.17	78.81
T0783TS457_4-D2.rsa	31.507	0.753	0	0.247	63	0.5	58.63
T0783TS228_1-D2.rsa	31.429	0.321	0.171	0.509	119	0.264	68.7
T0783TS041_4-D2.rsa	31.313	0.229	0.208	0.563	162	0.193	70.23
T0783TS326_4-D2.rsa	31.293	0.349	0.102	0.55	205	0.153	45.83
T0783TS118_2-D2.rsa	31.25	0.132	0.25	0.618	84	0.372	13.23
T0783TS301_2-D2.rsa	30.986	0.5	0.113	0.387	65	0.477	53.53
T0783TS425_5-D2.rsa	30.986	0.47	0.137	0.393	66	0.469	38.78
T0783TS038_4-D2.rsa	30.508	0.019	0.266	0.715	153	0.199	56.19
T0783TS160_1-D2.rsa	30.476	0.248	0.175	0.577	135	0.226	69.12
T0783TS362_5-D2.rsa	30.476	0.303	0.158	0.538	126	0.242	66.88
T0783TS401_5-D2.rsa	30.357	0.175	0.294	0.532	67	0.453	52.18
T0783TS360_4-D2.rsa	30.357	0.19	0.333	0.476	60	0.506	54.37
T0783TS204_1-D2.rsa	30.357	0.19	0.23	0.579	73	0.416	41.87
T0783TS044_5-D2.rsa	30.357	0.183	0.333	0.484	61	0.498	42.86
T0783TS132_1-D2.rsa	30.357	0.175	0.397	0.429	54	0.562	52.58
T0783TS065_4-D2.rsa	30.357	0.159	0.365	0.476	60	0.506	53.37
T0783TS144_4-D2.rsa	30.357	0.19	0.317	0.492	62	0.49	44.05
T0783TS457_2-D2.rsa	30.357	0.143	0.333	0.524	66	0.46	41.27
T0783TS425_3-D2.rsa	30.357	0.175	0.397	0.429	54	0.562	54.56
T0783TS038_2-D2.rsa	30.233	0.355	0.16	0.485	82	0.369	72.34

T0783TS333_1-D2.rsa	30.233	0.299	0.127	0.575	77	0.393	29.66
T0783TS204_2-D2.rsa	30.233	0.306	0.112	0.582	78	0.388	26.31
T0783TS425_1-D2.rsa	30.233	0.336	0.037	0.627	84	0.36	26.68
T0783TS032_3_2-D2.rsa	30.137	0.741	0	0.259	66	0.457	62.84
T0783TS034_1-D2.rsa	29.6	0	0.264	0.736	242	0.122	49.7
T0783TS171_4-D2.rsa	29.6	0.015	0.325	0.66	217	0.136	54.86
T0783TS169_5-D2.rsa	29.577	0.5	0.071	0.429	72	0.411	51.92
T0783TS296_2-D2.rsa	29.577	0.494	0.071	0.435	73	0.405	52.24
T0783TS216_5-D2.rsa	29.524	0.389	0.162	0.449	105	0.281	70.51
T0783TS492_1-D2.rsa	29.508	0.019	0.338	0.643	135	0.219	69.78
T0783TS300_4-D2.rsa	29.452	0.391	0.119	0.491	182	0.162	75.14
T0783TS483_3-D2.rsa	29.452	0.41	0.119	0.472	175	0.168	75.55
T0783TS340_3-D2.rsa	29.252	0.365	0.097	0.539	201	0.146	81.32
T0783TS338_3-D2.rsa	28.947	0.241	0.509	0.25	27	1.072	93.52
T0783TS436_2-D2.rsa	28.8	0.012	0.255	0.733	241	0.12	25.76
T0783TS145_4-D2.rsa	28.767	0.402	0.108	0.491	182	0.158	81.4
T0783TS235_4-D2.rsa	28.767	0.404	0.105	0.491	182	0.158	81.27
T0783TS026_1-D2.rsa	28.767	0.475	0	0.525	134	0.215	49.9
T0783TS049_2-D2.rsa	28.571	0.397	0.121	0.482	108	0.265	80.83
T0783TS401_4-D2.rsa	28.571	0.159	0.325	0.516	65	0.44	46.83
T0783TS204_4-D2.rsa	28.571	0.167	0.333	0.5	63	0.454	47.22
T0783TS144_2-D2.rsa	28.571	0.175	0.325	0.5	63	0.454	49.8
T0783TS041_5-D2.rsa	28.283	0.24	0.167	0.594	171	0.165	65.8
T0783TS454_4-D2.rsa	28.283	0.247	0.208	0.545	157	0.18	61.81
T0783TS445_4-D2.rsa	28.283	0.212	0.188	0.601	173	0.163	49.57
T0783TS241_1_2-D2.rsa	28.169	0.506	0.113	0.381	64	0.44	53.53
T0783TS251_1-D2.rsa	28	0.428	0.022	0.55	222	0.126	46.29
T0783TS210_1-D2.rsa	28	0.036	0.326	0.638	88	0.318	56.7
T0783TS117_1-D2.rsa	27.941	0.337	0.103	0.56	136	0.205	66.67
T0783TS171_2-D2.rsa	27.891	0.365	0.097	0.539	201	0.139	82.08
T0783TS276_3-D2.rsa	27.869	0.019	0.433	0.548	115	0.242	67.04
T0783TS391_1-D2.rsa	27.5	0.009	0.158	0.833	95	0.289	28.07
T0783TS333_5-D2.rsa	27.5	0.018	0.377	0.605	69	0.399	37.5
T0783TS276_4-D2.rsa	27.273	0.424	0.107	0.469	105	0.26	79.17
T0783TS251_4-D2.rsa	27.16	0.366	0.077	0.557	254	0.107	53.86
T0783TS169_1-D2.rsa	26.761	0.393	0.155	0.452	76	0.352	41.51
T0783TS197_3-D2.rsa	26.733	0.283	0.14	0.576	185	0.145	74.69
T0783TS117_2-D2.rsa	26.712	0.402	0.105	0.493	183	0.146	72.41
T0783TS290_5-D2.rsa	26.563	0.096	0.235	0.669	91	0.292	15.99
T0783TS063_5-D2.rsa	26.531	0.389	0.102	0.509	190	0.14	86.6
T0783TS008_5-D2.rsa	26.531	0.391	0.105	0.504	188	0.141	86.04
T0783TS349_2-D2.rsa	26.471	0.362	0.095	0.543	132	0.201	67.67
T0783TS263_1-D2.rsa	26.4	0	0.292	0.708	233	0.113	54.86
T0783TS216_1-D2.rsa	26.316	0.287	0.343	0.37	40	0.658	85.88
T0783TS362_3-D2.rsa	26.286	0.431	0.027	0.542	219	0.12	35.09
T0783TS155_4-D2.rsa	26.263	0.184	0.177	0.639	184	0.143	52.69
T0783TS026_2-D2.rsa	26.23	0.033	0.414	0.552	116	0.226	60.57

T0783TS251_5-D2.rsa	26.027	0.399	0.113	0.488	181	0.144	74.59
T0783TS310_1-D2.rsa	26.027	0.714	0	0.286	73	0.357	35.78
T0783TS326_5-D2.rsa	25.974	0.388	0.125	0.487	109	0.238	76.79
T0783TS206_1-D2.rsa	25.85	0.346	0.062	0.592	221	0.117	83.68
T0783TS157_4-D2.rsa	25.85	0.37	0.102	0.528	197	0.131	82.43
T0783TS340_4-D2.rsa	25.85	0.397	0.102	0.501	187	0.138	86.39
T0783TS420_2-D2.rsa	25.85	0.378	0.107	0.515	192	0.135	83.4
T0783TS442_1-D2.rsa	25.85	0.389	0.094	0.517	193	0.134	82.22
T0783TS492_5-D2.rsa	25.743	0.305	0.14	0.555	178	0.145	76.71
T0783TS041_3-D2.rsa	25.714	0.448	0.01	0.542	219	0.117	37.99
T0783TS145_1-D2.rsa	25.714	0.493	0.005	0.502	203	0.127	35.89
T0783TS117_4-D2.rsa	25.641	0.365	0.108	0.527	156	0.164	54.25
T0783TS349_4-D2.rsa	25.641	0.368	0.152	0.48	142	0.181	56.25
T0783TS335_4-D2.rsa	25.581	0.355	0.225	0.42	71	0.36	72.93
T0783TS197_4-D2.rsa	25.253	0.264	0.222	0.514	148	0.171	73.61
T0783TS080_4-D2.rsa	25.143	0.545	0.027	0.428	173	0.145	50.06
T0783TS153_2-D2.rsa	25	0.395	0.115	0.49	119	0.21	66.22
T0783TS212_1-D2.rsa	25	0.434	0.135	0.43	108	0.231	84.86
T0783TS452_3-D2.rsa	25	0.018	0.289	0.693	79	0.316	41.23
T0783TS026_4-D2.rsa	24.675	0.379	0.125	0.496	111	0.222	72.02
T0783TS155_3-D2.rsa	24.359	0.382	0.111	0.507	150	0.162	58.07
T0783TS483_4-D2.rsa	24.242	0.26	0.208	0.531	153	0.158	73.61
T0783TS345_2-D2.rsa	24	0.022	0.145	0.833	115	0.209	61.41
T0783TS228_2-D2.rsa	24	0.051	0.377	0.572	79	0.304	63.23
T0783TS300_1-D2.rsa	23.429	0.502	0.01	0.488	197	0.119	50.99
T0783TS160_5-D2.rsa	23.429	0.436	0.057	0.507	205	0.114	38.92
T0783TS362_4-D2.rsa	23.429	0.537	0.03	0.433	175	0.134	50.93
T0783TS276_1-D2.rsa	23.377	0.366	0.138	0.496	111	0.211	70.71
T0783TS171_1-D2.rsa	22.84	0.346	0.077	0.577	263	0.087	61.53
T0783TS310_2-D2.rsa	22.5	0	0.386	0.614	70	0.321	38.6
T0783TS439_3-D2.rsa	22.368	0.217	0.045	0.738	245	0.091	47.06
T0783TS442_4-D2.rsa	22.286	0.49	0.007	0.502	203	0.11	42.76
T0783TS277_4-D2.rsa	22.078	0.379	0.138	0.482	108	0.204	77.62
T0783TS349_5-D2.rsa	22.059	0.374	0.086	0.539	131	0.168	68.44
T0783TS300_2-D2.rsa	22.034	0.014	0.224	0.762	163	0.135	60.16
T0783TS008_2-D2.rsa	21.605	0.432	0.086	0.482	220	0.098	68.76
T0783TS346_1-D2.rsa	21.053	0.428	0.009	0.564	257	0.082	55.54
T0783TS420_4-D2.rsa	20.37	0.43	0.092	0.478	218	0.093	70.86
T0783TS155_1-D2.rsa	20.339	0	0.304	0.696	149	0.137	62.38
T0783TS445_3-D2.rsa	20	0.421	0.025	0.554	224	0.089	41.34
T0783TS360_2-D2.rsa	20	0.08	0.261	0.659	91	0.22	66.85
T0783TS360_1-D2.rsa	20	0	0.319	0.681	94	0.213	48.37
T0783TS381_2-D2.rsa	20	0	0.29	0.71	98	0.204	42.39
T0783TS322_1-D2.rsa	19.767	0.41	0.071	0.519	147	0.134	72.7
T0783TS011_1-D2.rsa	19.753	0.414	0.101	0.485	221	0.089	69.87
T0783TS153_3-D2.rsa	19.737	0.175	0.12	0.705	234	0.084	98.49
T0783TS263_2-D2.rsa	19.737	0.419	0.004	0.577	263	0.075	59.59

T0783TS063_2-D2.rsa	19.737	0.406	0.029	0.566	258	0.076	61.35
T0783TS008_3-D2.rsa	19.737	0.467	0.029	0.504	230	0.086	64.42
T0783TS276_2-D2.rsa	19.298	0.438	0.082	0.479	105	0.184	79.8
T0783TS436_5-D2.rsa	18.519	0.432	0.092	0.476	217	0.085	33.44
T0783TS063_4-D2.rsa	18.421	0.13	0.081	0.789	262	0.07	44.73
T0783TS145_2-D2.rsa	18.421	0.328	0.114	0.557	185	0.1	94.5
T0783TS492_3-D2.rsa	18.286	0.507	0.027	0.465	188	0.097	41.95
T0783TS145_5-D2.rsa	17.901	0.452	0.094	0.454	207	0.086	70.03
T0783TS420_5-D2.rsa	17.714	0.324	0	0.676	273	0.065	23.76
T0783TS322_2-D2.rsa	17.442	0.389	0.106	0.505	143	0.122	68.55
T0783TS417_1-D2.rsa	17.105	0.419	0.029	0.553	252	0.068	58.88
T0783TS436_1-D2.rsa	17.105	0.325	0.111	0.563	187	0.091	98.87
T0783TS362_1-D2.rsa	17.105	0.22	0.117	0.663	220	0.078	47.52
T0783TS153_4-D2.rsa	16.923	0.271	0.164	0.566	259	0.065	73.17
T0783TS011_3-D2.rsa	16.923	0.262	0.214	0.524	240	0.071	73.78
T0783TS228_5-D2.rsa	16.923	0.269	0.236	0.496	227	0.075	70.5
T0783TS011_4-D2.rsa	16.447	0.399	0.024	0.577	263	0.063	61.95
T0783TS157_2-D2.rsa	16.279	0.389	0.113	0.498	141	0.115	76.5
T0783TS155_2-D2.rsa	16.279	0.417	0.138	0.445	126	0.129	78.44
T0783TS116_4-D2.rsa	16.154	0.271	0.159	0.57	261	0.062	75.78
T0783TS228_3-D2.rsa	16.154	0.269	0.234	0.498	228	0.071	75.17
T0783TS144_1-D2.rsa	16	0	0.203	0.797	110	0.145	13.95
T0783TS420_3-D2.rsa	15.789	0.304	0.148	0.548	182	0.087	99.1
T0783TS251_3-D2.rsa	15.385	0.251	0.236	0.513	235	0.065	75.11
T0783TS492_4-D2.rsa	14.474	0.334	0.123	0.542	180	0.08	99.32
T0783TS322_3-D2.rsa	14.035	0.356	0.073	0.571	125	0.112	65.3
T0783TS454_5-D2.rsa	13.953	0.389	0.113	0.498	141	0.099	81.09
T0783TS157_3-D2.rsa	13.158	0.316	0.151	0.533	177	0.074	92.47
T0783TS145_3-D2.rsa	13.158	0.337	0.133	0.53	176	0.075	81.7
T0783TS454_3-D2.rsa	12.766	0.331	0.191	0.479	123	0.104	79.67
T0783TS457_5-D2.rsa	12	0.029	0.29	0.681	94	0.128	49.27
T0783TS008_4-D2.rsa	11.842	0.283	0.087	0.63	209	0.057	91.87
T0783TS300_5-D2.rsa	11.842	0.331	0.127	0.542	180	0.066	98.19
T0783TS276_5-D2.rsa	10.638	0.339	0.187	0.475	122	0.087	81.91
T0891TS451_1-D1.rsa	74	0	0.268	0.732	82	0.902	43.97
T0891TS451_3-D1.rsa	72	0	0.277	0.723	81	0.889	42.63
T0891TS451_4-D1.rsa	72	0	0.259	0.741	83	0.867	42.86
T0891TS455_1-D1.rsa	68	0.036	0.125	0.839	94	0.723	14.96
T0891TS321_1-D1.rsa	66	0	0.429	0.571	64	1.031	16.3
T0891TS434_5-D1.rsa	66	0.063	0	0.938	105	0.629	21.2
T0891TS451_2-D1.rsa	64	0	0.259	0.741	83	0.771	45.54
T0891TS321_3-D1.rsa	64	0	0.429	0.571	64	1	16.74
T0891TS455_5-D1.rsa	62	0	0.036	0.964	108	0.574	17.41
T0891TS455_2-D1.rsa	58	0.018	0.098	0.884	99	0.586	14.96
T0891TS321_4-D1.rsa	58	0	0.438	0.563	63	0.921	18.53
T0891TS321_2-D1.rsa	56	0	0.33	0.67	75	0.747	18.97
T0891TS434_2-D1.rsa	54	0.054	0.018	0.929	104	0.519	19.87

T0891TS434_1-D1.rsa	52	0.063	0.071	0.866	97	0.536	19.64
T0891TS180_5-D1.rsa	52	0	0.107	0.893	100	0.52	40.18
T0891TS434_4-D1.rsa	50	0.116	0.018	0.866	97	0.515	20.98
T0891TS251_4-D1.rsa	48	0.036	0.402	0.563	63	0.762	66.96
T0891TS180_3-D1.rsa	48	0.063	0.518	0.42	47	1.021	69.64
T0891TS183_1-D1.rsa	46	0.054	0.455	0.491	55	0.836	90.18
T0891TS479_2-D1.rsa	46	0.054	0.5	0.446	50	0.92	89.51
T0891TS180_1-D1.rsa	46	0	0.464	0.536	60	0.767	43.53
T0891TS183_2-D1.rsa	46	0.054	0.491	0.455	51	0.902	87.72
T0891TS467_5-D1.rsa	46	0.018	0.393	0.589	66	0.697	70.31
T0891TS357_5-D1.rsa	46	0.054	0.205	0.741	83	0.554	72.99
T0891TS180_2-D1.rsa	46	0.054	0.527	0.42	47	0.979	82.59
T0891TS357_3-D1.rsa	46	0.027	0.268	0.705	79	0.582	79.24
T0891TS467_3-D1.rsa	46	0.054	0.518	0.429	48	0.958	76.79
T0891TS220_3-D1.rsa	46	0.071	0.527	0.402	45	1.022	91.07
T0891TS434_3-D1.rsa	44	0.054	0	0.946	106	0.415	24.55
T0891TS077_4-D1.rsa	44	0.054	0.482	0.464	52	0.846	89.29
T0891TS432_3-D1.rsa	44	0.036	0.384	0.58	65	0.677	51.79
T0891TS421_1-D1.rsa	44	0.036	0.375	0.589	66	0.667	89.29
T0891TS026_5-D1.rsa	44	0.054	0.473	0.473	53	0.83	79.02
T0891TS357_1-D1.rsa	44	0.036	0.205	0.759	85	0.518	76.34
T0891TS432_1-D1.rsa	44	0.036	0.455	0.509	57	0.772	60.71
T0891TS005_1-D1.rsa	44	0.045	0.464	0.491	55	0.8	86.83
T0891TS077_5-D1.rsa	44	0.054	0.464	0.482	54	0.815	89.73
T0891TS359_2-D1.rsa	44	0.036	0.509	0.455	51	0.863	85.94
T0891TS220_2-D1.rsa	44	0.071	0.527	0.402	45	0.978	91.74
T0891TS180_4-D1.rsa	42	0	0.268	0.732	82	0.512	35.71
T0891TS236_2-D1.rsa	42	0.071	0.491	0.438	49	0.857	90.18
T0891TS421_3-D1.rsa	42	0.036	0.366	0.598	67	0.627	89.29
T0891TS016_1-D1.rsa	42	0.054	0.438	0.509	57	0.737	87.28
T0891TS048_1-D1.rsa	42	0.054	0.536	0.411	46	0.913	84.15
T0891TS275_3-D1.rsa	42	0.054	0.455	0.491	55	0.764	85.71
T0891TS464_4-D1.rsa	42	0.071	0.446	0.482	54	0.778	87.28
T0891TS359_3-D1.rsa	42	0.054	0.473	0.473	53	0.792	89.06
T0891TS077_1-D1.rsa	42	0.071	0.527	0.402	45	0.933	89.73
T0891TS251_5-D1.rsa	42	0.036	0.384	0.58	65	0.646	64.95
T0891TS446_3-D1.rsa	42	0.071	0.509	0.42	47	0.894	90.4
T0891TS446_5-D1.rsa	42	0.071	0.464	0.464	52	0.808	91.07
T0891TS220_5-D1.rsa	42	0.071	0.5	0.429	48	0.875	91.3
T0891TS251_2-D1.rsa	42	0	0.384	0.616	69	0.609	68.75
T0891TS357_2-D1.rsa	42	0.054	0.25	0.696	78	0.538	75.89
T0891TS467_2-D1.rsa	42	0.054	0.527	0.42	47	0.894	83.04
T0891TS357_4-D1.rsa	42	0.054	0.232	0.714	80	0.525	73.66
T0891TS382_5-D1.rsa	42	0.045	0.509	0.446	50	0.84	86.16
T0891TS382_2-D1.rsa	42	0.045	0.509	0.446	50	0.84	86.61
T0891TS432_2-D1.rsa	40	0.018	0.357	0.625	70	0.571	62.05
T0891TS425_5-D1.rsa	40	0.071	0.5	0.429	48	0.833	89.06

T0891TS425_4-D1.rsa	40	0.054	0.464	0.482	54	0.741	90.62
T0891TS421_4-D1.rsa	40	0.071	0.455	0.473	53	0.755	86.16
T0891TS455_4-D1.rsa	40	0.018	0.473	0.509	57	0.702	83.04
T0891TS464_1-D1.rsa	40	0.089	0.455	0.455	51	0.784	87.5
T0891TS421_2-D1.rsa	40	0.036	0.402	0.563	63	0.635	89.51
T0891TS407_4-D1.rsa	40	0.071	0.491	0.438	49	0.816	90.85
T0891TS452_3-D1.rsa	40	0.054	0.464	0.482	54	0.741	79.46
T0891TS359_5-D1.rsa	40	0.08	0.42	0.5	56	0.714	88.17
T0891TS236_5-D1.rsa	40	0.054	0.455	0.491	55	0.727	87.5
T0891TS251_1-D1.rsa	40	0.018	0.393	0.589	66	0.606	70.54
T0891TS220_1-D1.rsa	40	0.071	0.527	0.402	45	0.889	90.85
T0891TS444_4-D1.rsa	40	0.036	0.491	0.473	53	0.755	85.94
T0891TS382_3-D1.rsa	40	0.045	0.509	0.446	50	0.8	85.94
T0891TS452_4-D1.rsa	40	0.036	0.464	0.5	56	0.714	84.82
T0891TS313_4-D1.rsa	40	0.071	0.446	0.482	54	0.741	87.72
T0891TS183_4-D1.rsa	38	0.071	0.509	0.42	47	0.809	81.47
T0891TS183_3-D1.rsa	38	0.036	0.5	0.464	52	0.731	84.15
T0891TS479_3-D1.rsa	38	0.071	0.42	0.509	57	0.667	87.72
T0891TS183_5-D1.rsa	38	0.054	0.473	0.473	53	0.717	87.95
T0891TS479_1-D1.rsa	38	0.054	0.491	0.455	51	0.745	90.4
T0891TS026_4-D1.rsa	38	0.036	0.446	0.518	58	0.655	87.28
T0891TS275_1-D1.rsa	38	0.054	0.455	0.491	55	0.691	80.36
T0891TS359_1-D1.rsa	38	0.054	0.446	0.5	56	0.679	88.39
T0891TS464_3-D1.rsa	38	0.071	0.446	0.482	54	0.704	85.94
T0891TS425_1-D1.rsa	38	0.054	0.518	0.429	48	0.792	88.62
T0891TS275_2-D1.rsa	38	0.054	0.455	0.491	55	0.691	86.38
T0891TS028_1-D1.rsa	38	0.054	0.473	0.473	53	0.717	88.39
T0891TS287_2-D1.rsa	38	0.054	0.446	0.5	56	0.679	90.4
T0891TS407_1-D1.rsa	38	0.054	0.446	0.5	56	0.679	89.06
T0891TS407_2-D1.rsa	38	0.018	0.491	0.491	55	0.691	89.51
T0891TS005_2-D1.rsa	38	0.045	0.554	0.402	45	0.844	79.91
T0891TS432_5-D1.rsa	38	0.054	0.438	0.509	57	0.667	63.17
T0891TS444_5-D1.rsa	38	0.054	0.5	0.446	50	0.76	85.94
T0891TS236_4-D1.rsa	38	0.054	0.464	0.482	54	0.704	90.62
T0891TS005_3-D1.rsa	38	0.045	0.5	0.455	51	0.745	80.13
T0891TS313_3-D1.rsa	38	0.089	0.455	0.455	51	0.745	87.72
T0891TS345_1-D1.rsa	38	0.054	0.527	0.42	47	0.809	89.06
T0891TS345_5-D1.rsa	38	0.071	0.536	0.393	44	0.864	88.84
T0891TS220_4-D1.rsa	38	0.071	0.518	0.411	46	0.826	89.51
T0891TS382_1-D1.rsa	38	0.045	0.509	0.446	50	0.76	86.61
T0891TS407_3-D1.rsa	36	0.018	0.5	0.482	54	0.667	88.17
T0891TS005_5-D1.rsa	36	0.063	0.473	0.464	52	0.692	84.82
T0891TS455_3-D1.rsa	36	0.054	0.438	0.509	57	0.632	85.49
T0891TS258_2-D1.rsa	36	0.08	0.455	0.464	52	0.692	87.72
T0891TS287_3-D1.rsa	36	0.071	0.446	0.482	54	0.667	89.95
T0891TS432_4-D1.rsa	36	0.054	0.348	0.598	67	0.537	62.28
T0891TS446_4-D1.rsa	36	0.027	0.527	0.446	50	0.72	86.83

T0891TS345_4-D1.rsa	36	0.054	0.598	0.348	39	0.923	89.51
T0891TS236_3-D1.rsa	36	0.054	0.438	0.509	57	0.632	89.73
T0891TS345_2-D1.rsa	36	0.054	0.589	0.357	40	0.9	89.95
T0891TS005_4-D1.rsa	36	0.045	0.455	0.5	56	0.643	79.24
T0891TS345_3-D1.rsa	36	0.089	0.473	0.438	49	0.735	86.38
T0891TS119_1-D1.rsa	36	0.071	0.455	0.473	53	0.679	86.61
T0891TS452_1-D1.rsa	36	0.054	0.491	0.455	51	0.706	89.06
T0891TS250_2-D1.rsa	36	0.071	0.527	0.402	45	0.8	90.18
T0891TS250_1-D1.rsa	36	0.071	0.527	0.402	45	0.8	90.4
T0891TS452_2-D1.rsa	36	0.071	0.42	0.509	57	0.632	86.83
T0891TS026_3-D1.rsa	36	0.027	0.464	0.509	57	0.632	87.05
T0891TS077_2-D1.rsa	36	0.054	0.491	0.455	51	0.706	88.17
T0891TS467_4-D1.rsa	36	0.045	0.491	0.464	52	0.692	67.86
T0891TS275_5-D1.rsa	36	0.071	0.473	0.455	51	0.706	86.38
T0891TS313_2-D1.rsa	36	0.071	0.455	0.473	53	0.679	87.28
T0891TS446_1-D1.rsa	36	0.054	0.5	0.446	50	0.72	88.62
T0891TS407_5-D1.rsa	36	0.054	0.518	0.429	48	0.75	89.06
T0891TS382_4-D1.rsa	36	0.045	0.509	0.446	50	0.72	86.16
T0891TS479_4-D1.rsa	34	0.054	0.429	0.518	58	0.586	84.15
T0891TS452_5-D1.rsa	34	0.054	0.42	0.527	59	0.576	74.78
T0891TS250_3-D1.rsa	34	0.071	0.518	0.411	46	0.739	90.18
T0891TS258_4-D1.rsa	34	0.054	0.455	0.491	55	0.618	89.51
T0891TS467_1-D1.rsa	34	0.089	0.527	0.384	43	0.791	83.93
T0891TS258_5-D1.rsa	34	0.054	0.5	0.446	50	0.68	91.52
T0891TS313_5-D1.rsa	34	0.071	0.5	0.429	48	0.708	86.38
T0891TS250_4-D1.rsa	34	0.071	0.527	0.402	45	0.756	90.85
T0891TS444_1-D1.rsa	34	0.054	0.536	0.411	46	0.739	86.38
T0891TS258_3-D1.rsa	32	0.071	0.518	0.411	46	0.696	88.62
T0891TS287_4-D1.rsa	32	0.054	0.482	0.464	52	0.615	90.18
T0891TS258_1-D1.rsa	32	0.071	0.491	0.438	49	0.653	89.06
T0891TS250_5-D1.rsa	32	0.071	0.527	0.402	45	0.711	90.4
T0891TS251_3-D1.rsa	30	0.045	0.464	0.491	55	0.545	63.84
T0799TS204_5-D4.rsa	95.775	0	0.012	0.988	166	0.577	8.49
T0799TS381_4-D4.rsa	88	0.065	0	0.935	129	0.682	13.59
T0799TS044_2-D4.rsa	82.353	0.278	0	0.722	91	0.905	31.94
T0799TS317_4-D4.rsa	78.947	0.477	0	0.523	58	1.361	20.5
T0799TS162_5-D4.rsa	77.941	0.407	0.008	0.584	142	0.549	10
T0799TS360_5-D4.rsa	75	0	0.229	0.771	101	0.743	18.89
T0799TS153_1-D4.rsa	75	0.532	0	0.468	59	1.271	15.68
T0799TS145_3-D4.rsa	73.239	0.327	0	0.673	113	0.648	17.63
T0799TS360_4-D4.rsa	67.857	0	0.344	0.656	86	0.789	58.4
T0799TS133_4-D4.rsa	65.714	0.256	0.009	0.735	172	0.382	9.62
T0799TS279_1-D4.rsa	64.912	0.233	0	0.767	168	0.386	9.7
T0799TS041_1-D4.rsa	64.789	0.024	0	0.976	164	0.395	11.7
T0799TS041_5-D4.rsa	62.791	0.201	0	0.799	107	0.587	38.81
T0799TS032_1_2-D4.rsa	62.791	0.194	0.015	0.791	106	0.592	39.55
T0799TS026_1-D4.rsa	61.702	0.136	0.016	0.848	218	0.283	20.04

T0799TS076_1-D4.rsa	61.538	0.279	0.073	0.648	107	0.575	72.47
T0799TS032_3_2-D4.rsa	61.538	0.279	0.139	0.582	96	0.641	64.4
T0799TS277_2-D4.rsa	60.714	0.038	0.275	0.687	90	0.675	59.35
T0799TS340_1-D4.rsa	60.526	0.054	0	0.946	105	0.576	15.09
T0799TS169_2-D4.rsa	60.345	0.395	0	0.605	155	0.389	11.33
T0799TS042_1-D4.rsa	59.615	0.273	0.061	0.667	110	0.542	71.04
T0799TS439_5-D4.rsa	59.155	0.256	0.054	0.69	116	0.51	24.68
T0799TS011_5-D4.rsa	57.692	0.285	0.115	0.6	99	0.583	78.96
T0799TS277_5-D4.rsa	57.692	0.285	0.115	0.6	99	0.583	75.47
T0799TS156_1-D4.rsa	57.692	0.279	0.085	0.636	105	0.549	51.9
T0799TS345_1-D4.rsa	55.769	0.267	0.085	0.648	107	0.521	65.19
T0799TS454_3-D4.rsa	53.846	0.267	0.097	0.636	105	0.513	69.46
T0799TS499_1-D4.rsa	53.846	0.236	0.127	0.636	105	0.513	71.36
T0799TS067_2-D4.rsa	53.571	0	0.038	0.962	126	0.425	16.22
T0799TS184_1-D4.rsa	51.563	0	0.015	0.985	134	0.385	11.03
T0799TS041_2-D4.rsa	51.515	0.288	0.104	0.608	175	0.294	15.54
T0799TS032_2_2-D4.rsa	50	0	0.085	0.915	151	0.331	13.29
T0799TS216_4-D4.rsa	50	0.038	0.374	0.588	77	0.649	14.5
T0799TS338_2-D4.rsa	48	0.006	0.374	0.62	204	0.235	21.43
T0799TS144_3-D4.rsa	46.591	0.037	0.341	0.622	184	0.253	29.9
T0799TS117_3-D4.rsa	46.552	0	0.098	0.902	129	0.361	58.39
T0799TS282_2-D4.rsa	45.07	0.494	0.083	0.423	71	0.635	23.88
T0799TS482_1-D4.rsa	44.737	0.325	0	0.675	224	0.2	9.04
T0799TS156_2-D4.rsa	44.737	0.346	0	0.654	217	0.206	9.49
T0799TS310_4-D4.rsa	44.643	0.183	0.222	0.595	75	0.595	20.44
T0799TS117_4-D4.rsa	43.103	0	0.063	0.937	134	0.322	58.39
T0799TS454_5-D4.rsa	43.103	0	0.133	0.867	124	0.348	55.59
T0799TS499_5-D4.rsa	43.056	0.343	0.061	0.596	127	0.339	65.36
T0799TS310_3-D4.rsa	42.982	0.341	0.147	0.512	111	0.387	38.48
T0799TS162_4-D4.rsa	41.667	0.404	0.042	0.554	118	0.353	68.66
T0799TS210_3-D4.rsa	41.667	0	0.473	0.527	58	0.718	32.73
T0799TS116_1-D4.rsa	41.667	0	0.3	0.7	77	0.541	16.14
T0799TS338_5-D4.rsa	41.379	0	0.077	0.923	132	0.313	58.22
T0799TS008_3-D4.rsa	41.379	0	0.126	0.874	125	0.331	59.27
T0799TS391_1-D4.rsa	41.228	0.318	0.074	0.608	132	0.312	54.26
T0799TS268_1-D4.rsa	40.909	0.084	0.422	0.493	146	0.28	45.52
T0799TS499_3-D4.rsa	39.655	0	0.147	0.853	122	0.325	59.27
T0799TS290_1-D4.rsa	38.889	0.343	0.08	0.577	123	0.316	68.78
T0799TS042_2-D4.rsa	38.636	0.057	0.389	0.554	164	0.236	34.71
T0799TS381_3-D4.rsa	38.636	0.051	0.449	0.5	148	0.261	43.75
T0799TS420_2-D4.rsa	38.596	0.244	0.129	0.627	136	0.284	52.99
T0799TS420_5-D4.rsa	37.719	0.295	0.101	0.604	131	0.288	42.28
T0799TS210_2-D4.rsa	37.209	0.366	0.06	0.575	77	0.483	41.42
T0799TS064_3-D4.rsa	37.209	0.284	0.037	0.679	91	0.409	25.56
T0799TS448_1-D4.rsa	36.8	0	0.146	0.854	281	0.131	43.54
T0799TS237_4-D4.rsa	36.765	0.399	0	0.601	146	0.252	48.33
T0799TS381_1-D4.rsa	36.364	0.068	0.399	0.534	158	0.23	47.63

T0799TS064_4-D4.rsa	36.207	0.586	0	0.414	106	0.342	54.2
T0799TS479_4-D4.rsa	36.066	0.029	0.39	0.581	122	0.296	68.03
T0799TS097_2-D4.rsa	36.066	0.029	0.414	0.557	117	0.308	70.03
T0799TS160_4-D4.rsa	35.938	0.037	0.029	0.934	127	0.283	57.17
T0799TS360_2-D4.rsa	35.938	0.015	0.301	0.684	93	0.386	68.02
T0799TS452_3-D4.rsa	35.593	0.023	0.271	0.706	151	0.236	57.48
T0799TS063_3-D4.rsa	35.2	0.009	0.38	0.611	201	0.175	55.47
T0799TS333_4-D4.rsa	35.088	0.253	0.18	0.567	123	0.285	56.45
T0799TS169_5-D4.rsa	35.088	0.267	0.194	0.539	117	0.3	61.87
T0799TS328_2_2-D4.rsa	34.737	0.453	0	0.547	129	0.269	59.32
T0799TS301_1-D4.rsa	34.722	0.474	0.047	0.479	102	0.34	57.74
T0799TS073_2-D4.rsa	34.483	0.582	0	0.418	107	0.322	55.66
T0799TS065_1_2-D4.rsa	34.426	0.024	0.338	0.638	134	0.257	65.3
T0799TS479_3-D4.rsa	34.426	0.024	0.39	0.586	123	0.28	67.91
T0799TS216_2-D4.rsa	34.375	0.088	0.36	0.551	75	0.458	12.87
T0799TS157_1-D4.rsa	34.343	0.212	0.184	0.604	174	0.197	57.9
T0799TS499_2-D4.rsa	34.211	0.244	0.18	0.576	125	0.274	58.87
T0799TS300_4-D4.rsa	34.091	0.047	0.436	0.517	153	0.223	47.89
T0799TS333_3-D4.rsa	33.803	0.482	0.137	0.381	64	0.528	38.94
T0799TS317_1-D4.rsa	33.803	0.482	0.137	0.381	64	0.528	38.94
T0799TS263_1-D4.rsa	33.714	0.428	0.02	0.552	223	0.151	13.12
T0799TS116_4-D4.rsa	33.663	0.296	0.14	0.564	181	0.186	42.76
T0799TS006_5-D4.rsa	32.895	0.313	0.042	0.645	214	0.154	10.24
T0799TS067_3-D4.rsa	32.571	0.47	0.005	0.525	212	0.154	32.43
T0799TS044_3-D4.rsa	32.456	0.3	0.212	0.488	106	0.306	66.47
T0799TS358_5-D4.rsa	32.353	0.358	0.091	0.551	134	0.241	66.33
T0799TS338_4-D4.rsa	32.353	0.305	0.033	0.663	161	0.201	46.56
T0799TS132_5-D4.rsa	32.203	0	0.336	0.664	142	0.227	65.07
T0799TS006_2-D4.rsa	32.051	0.355	0.091	0.554	164	0.195	35.59
T0799TS157_2-D4.rsa	32	0.418	0	0.582	235	0.136	12.99
T0799TS184_5-D4.rsa	32	0.043	0.145	0.812	112	0.286	35.33
T0799TS362_1-D4.rsa	31.818	0.081	0.365	0.554	164	0.194	53.63
T0799TS153_3-D4.rsa	31.683	0.246	0.156	0.598	192	0.165	76.56
T0799TS317_5-D4.rsa	31.579	0.231	0.491	0.278	30	1.053	92.59
T0799TS276_2-D4.rsa	31.507	0.377	0.105	0.518	192	0.164	69.96
T0799TS144_2-D4.rsa	31.313	0.198	0.167	0.635	183	0.171	45.92
T0799TS034_1-D4.rsa	31	0.359	0.084	0.558	140	0.221	60.56
T0799TS162_3-D4.rsa	31	0.43	0.108	0.462	116	0.267	82.07
T0799TS073_1-D4.rsa	30.882	0.358	0.091	0.551	134	0.23	66.22
T0799TS335_3-D4.rsa	30.682	0.061	0.392	0.547	162	0.189	51.18
T0799TS328_4_2-D4.rsa	30.526	0.525	0.051	0.424	100	0.305	63.88
T0799TS414_2-D4.rsa	30.508	0.037	0.215	0.748	160	0.191	56.89
T0799TS347_4-D4.rsa	30.508	0	0.318	0.682	146	0.209	62.5
T0799TS301_2-D4.rsa	30.508	0.014	0.304	0.682	146	0.209	63.44
T0799TS420_4-D4.rsa	30.476	0.321	0.192	0.487	114	0.267	69.23
T0799TS251_1-D4.rsa	30.4	0.018	0.41	0.571	188	0.162	57.07
T0799TS067_4-D4.rsa	30.357	0.183	0.048	0.77	97	0.313	35.91

T0799TS216_5-D4.rsa	30.357	0.175	0.349	0.476	60	0.506	50
T0799TS282_5-D4.rsa	30	0	0.325	0.675	77	0.39	21.93
T0799TS290_5-D4.rsa	29.87	0.393	0.107	0.5	112	0.267	78.81
T0799TS439_2-D4.rsa	29.87	0.411	0.121	0.469	105	0.284	81.31
T0799TS362_5-D4.rsa	29.6	0.021	0.395	0.584	192	0.154	53.19
T0799TS277_1-D4.rsa	29.524	0.235	0.197	0.568	133	0.222	69.66
T0799TS171_5_2-D4.rsa	29.508	0	0.4	0.6	126	0.234	63.43
T0799TS116_2-D4.rsa	29.412	0.387	0.128	0.486	118	0.249	65.33
T0799TS360_3-D4.rsa	29.412	0.436	0.099	0.465	113	0.26	64.33
T0799TS160_3-D4.rsa	29.252	0.375	0.105	0.52	194	0.151	82.99
T0799TS276_4-D4.rsa	29.252	0.378	0.091	0.531	198	0.148	75.07
T0799TS008_4-D4.rsa	29.167	0.3	0.085	0.615	131	0.223	65.36
T0799TS454_4-D4.rsa	29.167	0.418	0.103	0.479	102	0.286	73.22
T0799TS251_3-D4.rsa	28.814	0.028	0.313	0.659	141	0.204	66.12
T0799TS133_2-D4.rsa	28.814	0.037	0.266	0.696	149	0.193	54.09
T0799TS235_2-D4.rsa	28.8	0.018	0.374	0.608	200	0.144	53.34
T0799TS317_3-D4.rsa	28.767	0.412	0.105	0.482	179	0.161	74.32
T0799TS417_1-D4.rsa	28.713	0.262	0.14	0.598	192	0.15	73.83
T0799TS110_2-D4.rsa	28.713	0.271	0.146	0.583	187	0.154	74.06
T0799TS042_3-D4.rsa	28.713	0.299	0.162	0.539	173	0.166	74.06
T0799TS063_4-D4.rsa	28.571	0.303	0.086	0.611	228	0.125	52.71
T0799TS326_1-D4.rsa	28.571	0.389	0.01	0.601	243	0.118	34.28
T0799TS145_1-D4.rsa	28.571	0.411	0.121	0.469	105	0.272	75.71
T0799TS263_3-D4.rsa	28.571	0.406	0.121	0.473	106	0.27	79.88
T0799TS310_2-D4.rsa	28.571	0.393	0.197	0.41	96	0.298	69.77
T0799TS290_3-D4.rsa	28.283	0.226	0.177	0.597	172	0.164	70.31
T0799TS156_4-D4.rsa	28.283	0.208	0.184	0.608	175	0.162	70.23
T0799TS204_2-D4.rsa	28.205	0.351	0.139	0.51	151	0.187	54.86
T0799TS235_3-D4.rsa	28.205	0.389	0.128	0.483	143	0.197	57.2
T0799TS050_2-D4.rsa	28.205	0.399	0.108	0.493	146	0.193	55.73
T0799TS277_4-D4.rsa	28.082	0.388	0.116	0.496	184	0.153	70.64
T0799TS349_2-D4.rsa	28	0.41	0.124	0.466	117	0.239	90.64
T0799TS268_3-D4.rsa	27.941	0.272	0.07	0.658	160	0.175	49.33
T0799TS381_5-D4.rsa	27.869	0.019	0.276	0.705	148	0.188	67.79
T0799TS155_5-D4.rsa	27.869	0.019	0.386	0.595	125	0.223	71.89
T0799TS340_2-D4.rsa	27.723	0.271	0.1	0.629	202	0.137	72.04
T0799TS156_3-D4.rsa	27.723	0.29	0.103	0.607	195	0.142	50.31
T0799TS425_3-D4.rsa	27.586	0.695	0	0.305	78	0.354	27.25
T0799TS349_3-D4.rsa	27.429	0.441	0.022	0.537	217	0.126	13.8
T0799TS097_4-D4.rsa	27.273	0.215	0.184	0.601	173	0.158	69.97
T0799TS145_4-D4.rsa	27.273	0.222	0.174	0.604	174	0.157	61.37
T0799TS335_2-D4.rsa	27.273	0.215	0.194	0.59	170	0.16	65.36
T0799TS391_2-D4.rsa	27.273	0.208	0.233	0.559	161	0.169	63.98
T0799TS452_5-D4.rsa	27.273	0.264	0.184	0.552	159	0.172	62.33
T0799TS184_4-D4.rsa	27.273	0.257	0.191	0.552	159	0.172	61.46
T0799TS328_1_2-D4.rsa	27.273	0.371	0.121	0.509	114	0.239	77.26
T0799TS492_1-D4.rsa	27.211	0.399	0.102	0.499	186	0.146	86.25

T0799TS436_3-D4.rsa	27.211	0.367	0.11	0.523	195	0.14	80.69
T0799TS442_4-D4.rsa	27.2	0.015	0.267	0.717	236	0.115	35.94
T0799TS011_4-D4.rsa	27.119	0.023	0.229	0.748	160	0.169	58.76
T0799TS452_1-D4.rsa	27.119	0.033	0.206	0.762	163	0.166	59.35
T0799TS116_3-D4.rsa	27.119	0	0.299	0.701	150	0.181	62.85
T0799TS110_4-D4.rsa	27.119	0.009	0.28	0.71	152	0.178	60.4
T0799TS479_1-D4.rsa	27.119	0.047	0.262	0.692	148	0.183	51.87
T0799TS171_2_2-D4.rsa	27.119	0	0.29	0.71	152	0.178	52.1
T0799TS479_5-D4.rsa	27	0.414	0.096	0.49	123	0.22	87.25
T0799TS420_3-D4.rsa	27	0.394	0.116	0.49	123	0.22	83.86
T0799TS437_1-D4.rsa	26.923	0.405	0.111	0.483	143	0.188	61.81
T0799TS301_4-D4.rsa	26.923	0.382	0.084	0.534	158	0.17	53.04
T0799TS184_3-D4.rsa	26.923	0.389	0.135	0.476	141	0.191	54.43
T0799TS310_1-D4.rsa	26.923	0.426	0.078	0.497	147	0.183	58.68
T0799TS024_1_2-D4.rsa	26.923	0.405	0.132	0.463	137	0.197	58.59
T0799TS322_3-D4.rsa	26.733	0.283	0.146	0.57	183	0.146	72.9
T0799TS425_1-D4.rsa	26.733	0.293	0.153	0.555	178	0.15	74.38
T0799TS251_4-D4.rsa	26.712	0.394	0.116	0.491	182	0.147	76.09
T0799TS362_4-D4.rsa	26.712	0.415	0.108	0.477	177	0.151	72.21
T0799TS206_1-D4.rsa	26.667	0.299	0.179	0.521	122	0.219	66.67
T0799TS442_5-D4.rsa	26.531	0.394	0.107	0.499	186	0.143	84.86
T0799TS439_3-D4.rsa	26.531	0.389	0.099	0.512	191	0.139	82.5
T0799TS310_5-D4.rsa	26.471	0.354	0.074	0.572	139	0.19	59.89
T0799TS425_2-D4.rsa	26.471	0.412	0.095	0.494	120	0.221	71.22
T0799TS333_1-D4.rsa	26.471	0.354	0.091	0.556	135	0.196	69.44
T0799TS169_3-D4.rsa	26.471	0.383	0.107	0.51	124	0.213	66.11
T0799TS454_1-D4.rsa	26.471	0.457	0.107	0.436	106	0.25	67.56
T0799TS436_1-D4.rsa	26.4	0.006	0.307	0.687	226	0.117	55.02
T0799TS041_4-D4.rsa	26.4	0.015	0.35	0.635	209	0.126	52.28
T0799TS235_1-D4.rsa	26.4	0.012	0.389	0.599	197	0.134	52.58
T0799TS155_2-D4.rsa	26.316	0.34	0.018	0.642	213	0.124	9.71
T0799TS345_3-D4.rsa	26.286	0.433	0	0.567	229	0.115	30.88
T0799TS144_5-D4.rsa	26.286	0.49	0.01	0.5	202	0.13	41.65
T0799TS414_1-D4.rsa	26.263	0.271	0.212	0.517	149	0.176	73
T0799TS110_5-D4.rsa	26.263	0.233	0.222	0.545	157	0.167	69.01
T0799TS169_4-D4.rsa	26.23	0.01	0.4	0.59	124	0.212	63.31
T0799TS326_4-D4.rsa	26.027	0.38	0.108	0.512	190	0.137	82.36
T0799TS349_1-D4.rsa	26.027	0.35	0.113	0.536	199	0.131	76.02
T0799TS155_3-D4.rsa	26.027	0.356	0.105	0.539	200	0.13	72.96
T0799TS328_3_2-D4.rsa	26	0.43	0.116	0.454	114	0.228	88.05
T0799TS296_1-D4.rsa	26	0.47	0.135	0.394	99	0.263	82.67
T0799TS054_4_2-D4.rsa	25.974	0.393	0.121	0.487	109	0.238	79.41
T0799TS492_5-D4.rsa	25.85	0.357	0.102	0.542	202	0.128	83.61
T0799TS210_4-D4.rsa	25.743	0.243	0.15	0.607	195	0.132	73.52
T0799TS132_3-D4.rsa	25.641	0.375	0.061	0.564	167	0.154	47.83
T0799TS008_2-D4.rsa	25.641	0.429	0.139	0.432	128	0.2	62.85
T0799TS157_4-D4.rsa	25.581	0.353	0.11	0.537	152	0.168	72.88

T0799TS171_4_2-D4.rsa	25.424	0.014	0.29	0.696	149	0.171	63.55
T0799TS301_3-D4.rsa	25.424	0.014	0.322	0.664	142	0.179	61.33
T0799TS133_5-D4.rsa	25.424	0	0.336	0.664	142	0.179	66.12
T0799TS290_2-D4.rsa	25.342	0.41	0.108	0.482	179	0.142	71.94
T0799TS268_4-D4.rsa	25.309	0.364	0.088	0.548	250	0.101	55.74
T0799TS381_2-D4.rsa	25.253	0.205	0.191	0.604	174	0.145	70.14
T0799TS438_1-D4.rsa	25.253	0.267	0.219	0.514	148	0.171	72.05
T0799TS155_4-D4.rsa	25.17	0.378	0.107	0.515	192	0.131	84.58
T0799TS492_3-D4.rsa	25.17	0.389	0.07	0.542	202	0.125	82.5
T0799TS492_4-D4.rsa	25.17	0.383	0.091	0.525	196	0.128	77.36
T0799TS362_3-D4.rsa	25.17	0.383	0.123	0.493	184	0.137	70.42
T0799TS326_5-D4.rsa	25.143	0.423	0.012	0.564	228	0.11	34.65
T0799TS162_1-D4.rsa	25.143	0.542	0.005	0.453	183	0.137	36.63
T0799TS080_4-D4.rsa	25	0.398	0.12	0.482	121	0.207	89.74
T0799TS133_3-D4.rsa	25	0.329	0.095	0.576	140	0.179	69.22
T0799TS064_1-D4.rsa	25	0.426	0.112	0.462	116	0.216	87.65
T0799TS347_3-D4.rsa	25	0.387	0.086	0.527	128	0.195	65.78
T0799TS301_5-D4.rsa	25	0.426	0.12	0.454	114	0.219	81.77
T0799TS132_4-D4.rsa	24.752	0.29	0.137	0.573	184	0.135	70.09
T0799TS282_1-D4.rsa	24.752	0.302	0.112	0.586	188	0.132	76.95
T0799TS011_2-D4.rsa	24.571	0.441	0.05	0.51	206	0.119	44.24
T0799TS235_5-D4.rsa	24.571	0.502	0.02	0.478	193	0.127	47.22
T0799TS067_1-D4.rsa	24.571	0.49	0.022	0.488	197	0.125	39.54
T0799TS064_5-D4.rsa	24.571	0.545	0	0.455	184	0.134	44.49
T0799TS054_5_2-D4.rsa	24.419	0.378	0.117	0.505	143	0.171	71.2
T0799TS116_5-D4.rsa	24.359	0.365	0.132	0.503	149	0.163	59.46
T0799TS008_1-D4.rsa	24.242	0.267	0.205	0.528	152	0.159	71.79
T0799TS153_2-D4.rsa	24.242	0.243	0.222	0.535	154	0.157	70.92
T0799TS414_3-D4.rsa	24.242	0.281	0.205	0.514	148	0.164	69.01
T0799TS056_1-D4.rsa	24	0.465	0.012	0.522	211	0.114	39.79
T0799TS097_3-D4.rsa	24	0.382	0.108	0.51	128	0.188	77.99
T0799TS296_2-D4.rsa	24	0.051	0.203	0.746	103	0.233	64.67
T0799TS276_1-D4.rsa	23.81	0.378	0.099	0.523	195	0.122	74.17
T0799TS442_3-D4.rsa	23.762	0.237	0.131	0.632	203	0.117	60.51
T0799TS282_3-D4.rsa	23.762	0.28	0.146	0.573	184	0.129	70.25
T0799TS414_5-D4.rsa	23.729	0	0.322	0.678	145	0.164	62.38
T0799TS212_1-D4.rsa	23.729	0.042	0.21	0.748	160	0.148	55.61
T0799TS425_5-D4.rsa	23.684	0.274	0.024	0.702	233	0.102	15.96
T0799TS364_1-D4.rsa	23.529	0.296	0.049	0.654	159	0.148	69.11
T0799TS064_2-D4.rsa	23.529	0.3	0.041	0.658	160	0.147	49.22
T0799TS349_4-D4.rsa	23.429	0.502	0.03	0.468	189	0.124	41.89
T0799TS117_1-D4.rsa	23.256	0.367	0.12	0.512	145	0.16	72.7
T0799TS155_1-D4.rsa	23.232	0.226	0.16	0.615	177	0.131	68.14
T0799TS144_4-D4.rsa	23.232	0.212	0.146	0.642	185	0.126	65.1
T0799TS479_2-D4.rsa	23.232	0.253	0.201	0.545	157	0.148	67.45
T0799TS300_5-D4.rsa	23.232	0.267	0.198	0.535	154	0.151	69.18
T0799TS237_5-D4.rsa	23.077	0.375	0.118	0.507	150	0.154	58.25

T0799TS358_2-D4.rsa	23	0.438	0.124	0.438	110	0.209	87.55
T0799TS044_5-D4.rsa	22.951	0.029	0.395	0.576	121	0.19	60.82
T0799TS442_1-D4.rsa	22.857	0.515	0.01	0.475	192	0.119	47.46
T0799TS050_1-D4.rsa	22.807	0.237	0.096	0.667	146	0.156	59.25
T0799TS042_5-D4.rsa	22.772	0.199	0.112	0.688	221	0.103	57.09
T0799TS499_4-D4.rsa	22.772	0.287	0.087	0.626	201	0.113	64.49
T0799TS333_5-D4.rsa	22.5	0.044	0.237	0.719	82	0.274	23.46
T0799TS345_5-D4.rsa	22.286	0.448	0.025	0.527	213	0.105	42.08
T0799TS300_2-D4.rsa	22.286	0.515	0.027	0.458	185	0.12	45.36
T0799TS204_4-D4.rsa	22.222	0.243	0.233	0.524	151	0.147	70.05
T0799TS346_1-D4.rsa	22.093	0.406	0.12	0.473	134	0.165	82.33
T0799TS268_2-D4.rsa	22.034	0.056	0.22	0.724	155	0.142	54.09
T0799TS347_2-D4.rsa	21.795	0.399	0.061	0.541	160	0.136	58.77
T0799TS326_2-D4.rsa	21.782	0.277	0.134	0.589	189	0.115	68.61
T0799TS006_4-D4.rsa	21.714	0.495	0.035	0.47	190	0.114	42.2
T0799TS492_2-D4.rsa	21.605	0.432	0.103	0.465	212	0.102	66.94
T0799TS322_1-D4.rsa	21.277	0.315	0.136	0.549	141	0.151	76.07
T0799TS210_1-D4.rsa	21.143	0.572	0.03	0.399	161	0.131	44.43
T0799TS448_5-D4.rsa	20.988	0.382	0.077	0.542	247	0.085	66.61
T0799TS335_4-D4.rsa	20.93	0.378	0.106	0.516	146	0.143	72.44
T0799TS410_5-D4.rsa	20.792	0.131	0	0.869	279	0.075	29.98
T0799TS044_1-D4.rsa	20.779	0.371	0.125	0.504	113	0.184	73.69
T0799TS237_1-D4.rsa	20.571	0.507	0.025	0.468	189	0.109	43.44
T0799TS076_5-D4.rsa	20.513	0.392	0.139	0.47	139	0.148	59.72
T0799TS448_3-D4.rsa	20.395	0.425	0.011	0.564	257	0.079	58.28
T0799TS410_3-D4.rsa	20.37	0.401	0.07	0.529	241	0.085	72.63
T0799TS144_1-D4.rsa	20.339	0	0.248	0.752	161	0.126	62.85
T0799TS054_2_2-D4.rsa	20	0.52	0	0.48	194	0.103	44.62
T0799TS345_2-D4.rsa	20	0.53	0	0.47	190	0.105	44.49
T0799TS328_5_2-D4.rsa	20	0.058	0.348	0.594	82	0.244	66.3
T0799TS041_3-D4.rsa	19.767	0.428	0.12	0.452	128	0.154	78.62
T0799TS296_3-D4.rsa	19.767	0.392	0.113	0.495	140	0.141	66.61
T0799TS063_1-D4.rsa	19.753	0.41	0.09	0.5	228	0.087	56.02
T0799TS011_1-D4.rsa	19.737	0.504	0.024	0.471	215	0.092	47.26
T0799TS080_5-D4.rsa	19.429	0.413	0.007	0.579	234	0.083	40.97
T0799TS452_2-D4.rsa	19.136	0.443	0.092	0.465	212	0.09	34.49
T0799TS153_5-D4.rsa	18.644	0.009	0.299	0.692	148	0.126	64.84
T0799TS160_2-D4.rsa	18.605	0.367	0.102	0.53	150	0.124	77.3
T0799TS420_1-D4.rsa	18.605	0.378	0.127	0.495	140	0.133	72.53
T0799TS076_4-D4.rsa	18.519	0.377	0.083	0.539	246	0.075	65.07
T0799TS263_2-D4.rsa	18.421	0.247	0.087	0.666	221	0.083	53.24
T0799TS251_2-D4.rsa	18.421	0.226	0.117	0.657	218	0.085	50.23
T0799TS156_5-D4.rsa	17.763	0.447	0.011	0.542	247	0.072	62.5
T0799TS436_2-D4.rsa	17.763	0.458	0.024	0.518	236	0.075	61.51
T0799TS063_2-D4.rsa	17.692	0.251	0.194	0.555	254	0.07	73.06
T0799TS110_1-D4.rsa	17.442	0.371	0.12	0.509	144	0.121	79.42
T0799TS080_1-D4.rsa	17.442	0.399	0.117	0.484	137	0.127	83.04

T0799TS347_1-D4.rsa	17.442	0.424	0.113	0.463	131	0.133	83.13
T0799TS410_1-D4.rsa	16.923	0.271	0.227	0.502	230	0.074	75.83
T0799TS439_1-D4.rsa	16.667	0.414	0.103	0.482	220	0.076	77.87
T0799TS410_2-D4.rsa	16.571	0.203	0.01	0.787	318	0.052	10.64
T0799TS335_5-D4.rsa	16.279	0.378	0.117	0.505	143	0.114	80.74
T0799TS153_4-D4.rsa	16.279	0.389	0.131	0.481	136	0.12	73.85
T0799TS038_1-D4.rsa	16.279	0.399	0.12	0.481	136	0.12	81.98
T0799TS277_3-D4.rsa	16.279	0.41	0.124	0.466	132	0.123	82.86
T0799TS276_3-D4.rsa	16.154	0.273	0.229	0.498	228	0.071	71.61
T0799TS145_2-D4.rsa	15.789	0.337	0.133	0.53	176	0.09	98.72
T0799TS038_2-D4.rsa	15.789	0.346	0.117	0.536	178	0.089	99.32
T0799TS157_5-D4.rsa	15.789	0.34	0.151	0.509	169	0.093	99.17
T0799TS251_5-D4.rsa	15.789	0.331	0.136	0.533	177	0.089	67.39
T0799TS436_4-D4.rsa	15.385	0.26	0.24	0.5	229	0.067	71.56
T0799TS268_5-D4.rsa	15.254	0	0.206	0.794	170	0.09	62.5
T0799TS452_4-D4.rsa	15.116	0.346	0.095	0.558	158	0.096	70.85
T0799TS080_2-D4.rsa	15.116	0.396	0.102	0.502	142	0.106	80.48
T0799TS171_3_2-D4.rsa	15.116	0.371	0.12	0.509	144	0.105	68.99
T0799TS434_1-D4.rsa	14.894	0.28	0.152	0.568	146	0.102	79.57
T0799TS276_5-D4.rsa	14.615	0.258	0.238	0.504	231	0.063	73.33
T0799TS160_5-D4.rsa	14.474	0.319	0.12	0.56	186	0.078	99.1
T0799TS436_5-D4.rsa	14.474	0.328	0.108	0.563	187	0.077	99.4
T0799TS011_3-D4.rsa	14.474	0.331	0.114	0.554	184	0.079	98.64
T0799TS345_4-D4.rsa	14.474	0.334	0.145	0.521	173	0.084	98.95
T0799TS110_3-D4.rsa	14.474	0.34	0.117	0.542	180	0.08	95.71
T0799TS300_1-D4.rsa	14.474	0.352	0.123	0.524	174	0.083	98.95
T0799TS241_1-D4.rsa	14.035	0.411	0.082	0.507	111	0.126	81.05
T0799TS425_4-D4.rsa	14.035	0.457	0.096	0.447	98	0.143	81.62
T0799TS006_3-D4.rsa	13.559	0	0.182	0.818	175	0.077	55.61
T0799TS160_1-D4.rsa	13.158	0.337	0.117	0.545	181	0.073	98.87
T0799TS042_4-D4.rsa	13.158	0.313	0.151	0.536	178	0.074	95.41
T0799TS097_5-D4.rsa	13.158	0.337	0.114	0.548	182	0.072	97.82
T0799TS338_1-D4.rsa	13.158	0.328	0.154	0.518	172	0.076	99.32
T0799TS063_5-D4.rsa	13.077	0.216	0.138	0.646	296	0.044	73.11
T0799TS008_5-D4.rsa	12.766	0.307	0.187	0.506	130	0.098	82
T0799TS204_3-D4.rsa	12.766	0.323	0.187	0.49	126	0.101	85.6
T0799TS169_1-D4.rsa	12.766	0.342	0.206	0.451	116	0.11	84.53
T0799TS210_5-D4.rsa	11.842	0.325	0.13	0.545	181	0.065	99.02
T0799TS439_4-D4.rsa	11.842	0.343	0.133	0.524	174	0.068	96.69
T0799TS132_1-D4.rsa	11.842	0.346	0.123	0.53	176	0.067	97.97
T0799TS362_2-D4.rsa	10.638	0.28	0.132	0.588	151	0.07	74.81
T0799TS157_3-D4.rsa	10.638	0.327	0.148	0.525	135	0.079	83.27
T0799TS326_3-D4.rsa	8.511	0.249	0.058	0.693	178	0.048	74.9
T0805TS073_2-D1.rsa	88.235	0.056	0	0.944	119	0.741	11.51
T0805TS454_5-D1.rsa	81.579	0.252	0	0.748	83	0.983	12.84
T0805TS499_1-D1.rsa	79.688	0.125	0.228	0.647	88	0.906	10.48
T0805TS499_2-D1.rsa	73.529	0.524	0	0.476	60	1.225	32.54

T0805TS050_1-D1.rsa	72	0	0.326	0.674	93	0.774	16.49
T0805TS210_4-D1.rsa	71.429	0.015	0.237	0.748	98	0.729	16.41
T0805TS117_4-D1.rsa	69.767	0.239	0	0.761	102	0.684	40.67
T0805TS251_4-D1.rsa	67.647	0.563	0	0.437	55	1.23	35.52
T0805TS206_1-D1.rsa	67.442	0.164	0.104	0.731	98	0.688	17.54
T0805TS110_3-D1.rsa	66.197	0.19	0.054	0.756	127	0.521	12.82
T0805TS300_5-D1.rsa	64	0.696	0	0.304	123	0.52	7.67
T0805TS133_2-D1.rsa	63.462	0.103	0.024	0.873	144	0.441	12.18
T0805TS216_3-D1.rsa	62.5	0.037	0.676	0.287	31	2.016	71.3
T0805TS216_5-D1.rsa	61.765	0.603	0	0.397	50	1.235	35.91
T0805TS117_1-D1.rsa	61.538	0.273	0.085	0.642	106	0.581	57.44
T0805TS228_3-D1.rsa	60.714	0	0.336	0.664	87	0.698	53.63
T0805TS381_2-D1.rsa	58.333	0	0.241	0.759	82	0.711	52.08
T0805TS263_2-D1.rsa	58.333	0	0.602	0.398	43	1.357	71.99
T0805TS263_1-D1.rsa	57.895	0.369	0.054	0.577	64	0.905	29.95
T0805TS011_2-D1.rsa	57.143	0.015	0.313	0.672	88	0.649	58.4
T0805TS335_3-D1.rsa	56.338	0.357	0	0.643	108	0.522	22.76
T0805TS479_1-D1.rsa	55.814	0.276	0.09	0.634	85	0.657	42.35
T0805TS335_5-D1.rsa	55.769	0.291	0.097	0.612	101	0.552	60.44
T0805TS349_5-D1.rsa	55	0.044	0	0.956	109	0.505	19.52
T0805TS038_2-D1.rsa	54.93	0.44	0.06	0.5	84	0.654	19.71
T0805TS414_1-D1.rsa	52.632	0.144	0	0.856	95	0.554	33.33
T0805TS448_1-D1.rsa	51.923	0.261	0.103	0.636	105	0.495	61.71
T0805TS268_5-D1.rsa	51.163	0.284	0	0.716	96	0.533	39.55
T0805TS011_3-D1.rsa	51.163	0.216	0	0.784	105	0.487	32.84
T0805TS479_5-D1.rsa	51.163	0.246	0.075	0.679	91	0.562	30.6
T0805TS452_3-D1.rsa	51.163	0.306	0.045	0.649	87	0.588	33.4
T0805TS210_1-D1.rsa	51.163	0.216	0.134	0.649	87	0.588	22.57
T0805TS212_1-D1.rsa	50	0.066	0.029	0.904	123	0.407	13.79
T0805TS277_2-D1.rsa	47.368	0.36	0.135	0.505	56	0.846	41.89
T0805TS452_5-D1.rsa	46.875	0.015	0.213	0.772	105	0.446	42.28
T0805TS452_1-D1.rsa	46.491	0.304	0.147	0.548	119	0.391	21.08
T0805TS345_1-D1.rsa	46.154	0	0.109	0.891	147	0.314	10.44
T0805TS268_3-D1.rsa	46.154	0.261	0.097	0.642	106	0.435	42.56
T0805TS454_4-D1.rsa	44.737	0.207	0.018	0.775	86	0.52	25.68
T0805TS216_4-D1.rsa	44.643	0.151	0.357	0.492	62	0.72	48.02
T0805TS110_1-D1.rsa	43.056	0.376	0.094	0.531	113	0.381	68.78
T0805TS410_5-D1.rsa	42.857	0.015	0.206	0.779	102	0.42	19.08
T0805TS277_5-D1.rsa	42.105	0.53	0.059	0.411	97	0.434	51.06
T0805TS414_4-D1.rsa	42.105	0.126	0	0.874	97	0.434	22.52
T0805TS210_3-D1.rsa	42.105	0.216	0	0.784	87	0.484	28.6
T0805TS410_1-D1.rsa	41.667	0.055	0.373	0.573	63	0.661	62.95
T0805TS008_3-D1.rsa	41.379	0	0.147	0.853	122	0.339	60.49
T0805TS251_5-D1.rsa	40.625	0.066	0.206	0.728	99	0.41	12.68
T0805TS345_5-D1.rsa	40.385	0	0.133	0.867	143	0.282	10.6
T0805TS454_3-D1.rsa	40	0.043	0.355	0.601	83	0.482	24.27
T0805TS008_4-D1.rsa	39.655	0	0.231	0.769	110	0.361	79.37

T0805TS237_5-D1.rsa	39.655	0	0.161	0.839	120	0.33	59.27
T0805TS041_1-D1.rsa	39.437	0.28	0.042	0.679	114	0.346	40.7
T0805TS452_4-D1.rsa	39.286	0	0.252	0.748	98	0.401	56.49
T0805TS414_3-D1.rsa	39.063	0.015	0.419	0.566	77	0.507	71.32
T0805TS210_2-D1.rsa	39.063	0.015	0.419	0.566	77	0.507	58.64
T0805TS145_2-D1.rsa	38.889	0.347	0.094	0.559	119	0.327	69.92
T0805TS008_2-D1.rsa	37.719	0.313	0.152	0.535	116	0.325	60.37
T0805TS420_2-D1.rsa	37.5	0.432	0.07	0.498	106	0.354	74.75
T0805TS345_3-D1.rsa	37.5	0.118	0.483	0.399	118	0.318	48.14
T0805TS410_4-D1.rsa	37.5	0	0.173	0.827	91	0.412	61.82
T0805TS263_5-D1.rsa	37.5	0.015	0.397	0.588	80	0.469	67.65
T0805TS479_2-D1.rsa	37.209	0.328	0.03	0.642	86	0.433	45.52
T0805TS156_3-D1.rsa	37.209	0.448	0.03	0.522	70	0.532	47.2
T0805TS184_5-D1.rsa	36.842	0.241	0.5	0.259	28	1.316	92.82
T0805TS184_1-D1.rsa	36.842	0.054	0.054	0.892	99	0.372	15.99
T0805TS345_4-D1.rsa	36.207	0	0.154	0.846	121	0.299	59.09
T0805TS300_3-D1.rsa	36.111	0.437	0.066	0.498	106	0.341	76.27
T0805TS492_3-D1.rsa	36	0.094	0.362	0.543	75	0.48	66.49
T0805TS184_4-D1.rsa	35.938	0.015	0.449	0.537	73	0.492	68.38
T0805TS237_3-D1.rsa	35.593	0.009	0.308	0.682	146	0.244	57.94
T0805TS414_2-D1.rsa	35.294	0.457	0.008	0.535	130	0.271	46.56
T0805TS420_3-D1.rsa	35	0.053	0	0.947	108	0.324	35.53
T0805TS038_4-D1.rsa	35	0.018	0.404	0.579	66	0.53	51.32
T0805TS171_4-D1.rsa	34.737	0.547	0.051	0.403	95	0.366	60.17
T0805TS448_2-D1.rsa	34.722	0.286	0.103	0.61	130	0.267	65.99
T0805TS160_5-D1.rsa	34.722	0.39	0.085	0.526	112	0.31	65.86
T0805TS345_2-D1.rsa	34.286	0.372	0.205	0.423	99	0.346	71.8
T0805TS251_1-D1.rsa	34.247	0.776	0	0.224	57	0.601	66.67
T0805TS038_5-D1.rsa	33.803	0.333	0.048	0.619	104	0.325	31.57
T0805TS171_3-D1.rsa	33.684	0.517	0.055	0.428	101	0.334	64.51
T0805TS436_2-D1.rsa	33.333	0.24	0.157	0.604	131	0.254	63.48
T0805TS228_1-D1.rsa	33.333	0.446	0.103	0.451	96	0.347	71.19
T0805TS381_1-D1.rsa	32.759	0.602	0	0.398	102	0.321	55.47
T0805TS251_3-D1.rsa	32.759	0.602	0	0.398	102	0.321	55.08
T0805TS156_2-D1.rsa	32.759	0.625	0	0.375	96	0.341	64.26
T0805TS436_5-D1.rsa	32.558	0.237	0.03	0.734	124	0.263	72.19
T0805TS499_5-D1.rsa	32	0.065	0.377	0.558	77	0.416	72.1
T0805TS414_5-D1.rsa	31.897	0.594	0	0.406	104	0.307	60.55
T0805TS110_5-D1.rsa	31.395	0.243	0.136	0.621	105	0.299	64.79
T0805TS300_1-D1.rsa	31.169	0.393	0.125	0.482	108	0.289	78.93
T0805TS237_4-D1.rsa	31.034	0.014	0.147	0.839	120	0.259	79.72
T0805TS073_1-D1.rsa	31.034	0.598	0	0.402	103	0.301	61.82
T0805TS216_1-D1.rsa	31.034	0.621	0	0.379	97	0.32	57.91
T0805TS171_1-D1.rsa	30.882	0.37	0.025	0.605	147	0.21	18.22
T0805TS436_1-D1.rsa	30.508	0.009	0.22	0.771	165	0.185	41.59
T0805TS479_3-D1.rsa	30	0	0.088	0.912	104	0.288	48.68
T0805TS454_1-D1.rsa	30	0	0.105	0.895	102	0.294	20.83

T0805TS454_2-D1.rsa	30	0	0.333	0.667	76	0.395	42.98
T0805TS300_4-D1.rsa	29.825	0.281	0.23	0.488	106	0.281	68.78
T0805TS110_2-D1.rsa	29.688	0.081	0.426	0.493	67	0.443	61.58
T0805TS041_3-D1.rsa	29.6	0	0.264	0.736	242	0.122	41.19
T0805TS499_4-D1.rsa	29.474	0.551	0.055	0.394	93	0.317	63.45
T0805TS410_2-D1.rsa	29.412	0.37	0.091	0.539	131	0.225	64.89
T0805TS133_4-D1.rsa	29.412	0.358	0.066	0.576	140	0.21	58
T0805TS251_2-D1.rsa	29.07	0.302	0.154	0.544	92	0.316	63.02
T0805TS228_4-D1.rsa	29.07	0.331	0.166	0.503	85	0.342	68.94
T0805TS145_5-D1.rsa	28.571	0.411	0.121	0.469	105	0.272	75.83
T0805TS038_3-D1.rsa	28.421	0.559	0.034	0.407	96	0.296	56.57
T0805TS279_1-D1.rsa	28	0.414	0.12	0.466	117	0.239	84.66
T0805TS041_5-D1.rsa	27.941	0.407	0.091	0.502	122	0.229	66.33
T0805TS448_5-D1.rsa	27.869	0.024	0.343	0.633	133	0.21	64.06
T0805TS171_5-D1.rsa	27.619	0.342	0.167	0.491	115	0.24	72.01
T0805TS008_1-D1.rsa	27.273	0.388	0.125	0.487	109	0.25	71.67
T0805TS160_3-D1.rsa	27.273	0.415	0.121	0.464	104	0.262	79.88
T0805TS160_4-D1.rsa	27.193	0.267	0.226	0.507	110	0.247	67.05
T0805TS193_2-D1.rsa	27	0.426	0.124	0.45	113	0.239	88.45
T0805TS448_3-D1.rsa	26.923	0.378	0.115	0.507	150	0.179	56.86
T0805TS117_3-D1.rsa	26.857	0.522	0.059	0.418	169	0.159	29.33
T0805TS277_1-D1.rsa	26.744	0.367	0.178	0.456	77	0.347	69.23
T0805TS228_2-D1.rsa	26.667	0.393	0.111	0.496	116	0.23	62.71
T0805TS228_5-D1.rsa	26.471	0.354	0.103	0.543	132	0.201	69.78
T0805TS038_1-D1.rsa	26.316	0.572	0.059	0.369	87	0.302	49.58
T0805TS349_4-D1.rsa	26.027	0.402	0.089	0.509	189	0.138	66.89
T0805TS171_2-D1.rsa	26	0.474	0.104	0.422	106	0.245	86.85
T0805TS479_4-D1.rsa	25.714	0.244	0.235	0.521	122	0.211	54.81
T0805TS156_4-D1.rsa	25	0	0.351	0.649	74	0.338	42.98
T0805TS133_5-D1.rsa	24.561	0.283	0.11	0.607	133	0.185	59.13
T0805TS349_1-D1.rsa	24.359	0.348	0.098	0.554	164	0.149	56.86
T0805TS145_3-D1.rsa	24	0.434	0.139	0.426	107	0.224	83.77
T0805TS335_4-D1.rsa	24	0.051	0.391	0.558	77	0.312	73.91
T0805TS263_3-D1.rsa	24	0.036	0.348	0.616	85	0.282	48.91
T0805TS349_2-D1.rsa	23.729	0.037	0.252	0.71	152	0.156	59.11
T0805TS436_3-D1.rsa	23.729	0.019	0.243	0.738	158	0.15	42.41
T0805TS448_4-D1.rsa	23.529	0.37	0.095	0.535	130	0.181	70.22
T0805TS420_4-D1.rsa	23.529	0.412	0.103	0.486	118	0.199	71.78
T0805TS008_5-D1.rsa	23.529	0.379	0.078	0.543	132	0.178	67.22
T0805TS145_4-D1.rsa	23	0.402	0.12	0.478	120	0.192	90.44
T0805TS011_5-D1.rsa	22.951	0.029	0.443	0.529	111	0.207	65.05
T0805TS492_1-D1.rsa	22.078	0.379	0.121	0.5	112	0.197	77.5
T0805TS436_4-D1.rsa	22	0.442	0.131	0.426	107	0.206	89.34
T0805TS011_4-D1.rsa	21.311	0.024	0.405	0.571	120	0.178	59.33
T0805TS277_3-D1.rsa	21.053	0.438	0.087	0.475	104	0.202	80.48
T0805TS041_4-D1.rsa	20.571	0.441	0.022	0.537	217	0.095	43.01
T0805TS335_2-D1.rsa	19.298	0.443	0.082	0.475	104	0.186	80.25

T0805TS011_1-D1.rsa	19.298	0.397	0.091	0.511	112	0.172	79.34
T0805TS381_4-D1.rsa	19.298	0.452	0.091	0.457	100	0.193	81.28
T0805TS335_1-D1.rsa	19.298	0.438	0.105	0.457	100	0.193	65.75
T0805TS160_1-D1.rsa	18.644	0.009	0.304	0.687	147	0.127	66.24
T0805TS499_3-D1.rsa	17.544	0.447	0.068	0.484	106	0.166	80.71
T0805TS210_5-D1.rsa	17.544	0.447	0.091	0.461	101	0.174	82.19
T0805TS110_4-D1.rsa	17.544	0.443	0.1	0.457	100	0.175	80.82
T0805TS156_5-D1.rsa	16.279	0.385	0.11	0.505	143	0.114	69.44
T0805TS381_3-D1.rsa	14.035	0.457	0.078	0.466	102	0.138	80.94
T0805TS145_1-D1.rsa	12.766	0.331	0.195	0.475	122	0.105	84.73
T0805TS133_1-D1.rsa	10.638	0.358	0.191	0.451	116	0.092	85.89
T0805TS268_4-D1.rsa	10.638	0.385	0.187	0.428	110	0.097	81.52
T0805TS156_1-D1.rsa	8.511	0.319	0.195	0.486	125	0.068	83.56
T0805TS492_4-D1.rsa	8.511	0.339	0.198	0.463	119	0.072	81.13
T0847TS110_4-D1.rsa	84.507	0.506	0.012	0.482	81	1.043	14.42
T0847TS251_3-D1.rsa	76.563	0.11	0.235	0.654	89	0.86	11.4
T0847TS216_5-D1.rsa	71.053	0.108	0.09	0.802	89	0.798	19.82
T0847TS452_3-D1.rsa	67.857	0.313	0	0.687	90	0.754	12.6
T0847TS011_4-D1.rsa	67.606	0.423	0	0.577	97	0.697	24.36
T0847TS251_4-D1.rsa	67.308	0.285	0.024	0.691	114	0.59	16.3
T0847TS300_4-D1.rsa	66.667	0.019	0.5	0.481	52	1.282	55.09
T0847TS454_1-D1.rsa	66.197	0.536	0.048	0.417	70	0.946	22.92
T0847TS145_5-D1.rsa	65.789	0.117	0.126	0.757	84	0.783	20.27
T0847TS420_1-D1.rsa	63.158	0.207	0	0.793	88	0.718	16.67
T0847TS492_3-D1.rsa	63.158	0.18	0	0.82	91	0.694	18.92
T0847TS452_1-D1.rsa	62.5	0	0.537	0.463	50	1.25	69.68
T0847TS335_3-D1.rsa	62.5	0	0.63	0.37	40	1.563	64.35
T0847TS410_3-D1.rsa	61.765	0.548	0	0.452	57	1.084	34.52
T0847TS499_1-D1.rsa	60.714	0.015	0.282	0.702	92	0.66	15.65
T0847TS414_3-D1.rsa	58.824	0.333	0	0.667	84	0.7	33.73
T0847TS041_1-D1.rsa	58.333	0	0.676	0.324	35	1.667	70.6
T0847TS268_5-D1.rsa	57.692	0.291	0.073	0.636	105	0.549	19.15
T0847TS263_2-D1.rsa	57.5	0.088	0.14	0.772	88	0.653	19.08
T0847TS335_4-D1.rsa	57.143	0.053	0.229	0.718	94	0.608	17.56
T0847TS110_1-D1.rsa	54.167	0	0.455	0.545	60	0.903	50
T0847TS251_1-D1.rsa	53.571	0	0.206	0.794	104	0.515	14.5
T0847TS263_5-D1.rsa	52.632	0.054	0.054	0.892	99	0.532	22.3
T0847TS479_5-D1.rsa	52.083	0.018	0.373	0.609	67	0.777	41.59
T0847TS171_5-D1.rsa	51.163	0.194	0.045	0.761	102	0.502	43.66
T0847TS117_3-D1.rsa	51.163	0.246	0.075	0.679	91	0.562	41.42
T0847TS448_1-D1.rsa	51.163	0.261	0.045	0.694	93	0.55	41.79
T0847TS381_4-D1.rsa	51.163	0.187	0.112	0.701	94	0.544	15.48
T0847TS420_4-D1.rsa	50	0	0.458	0.542	71	0.704	58.21
T0847TS414_4-D1.rsa	50	0.175	0.254	0.571	72	0.694	54.17
T0847TS414_1-D1.rsa	50	0	0.351	0.649	85	0.588	17.94
T0847TS216_1-D1.rsa	50	0.056	0.454	0.491	53	0.943	57.18
T0847TS410_5-D1.rsa	48.438	0.059	0.316	0.625	85	0.57	13.05

T0847TS110_3-D1.rsa	48.214	0.27	0.056	0.675	85	0.567	18.45
T0847TS237_5-D1.rsa	47.917	0	0.236	0.764	84	0.57	51.59
T0847TS381_1-D1.rsa	47.887	0.333	0	0.667	112	0.428	13.62
T0847TS073_2-D1.rsa	47.368	0.25	0.38	0.37	40	1.184	71.06
T0847TS145_3-D1.rsa	47.059	0.374	0.091	0.535	130	0.362	43.78
T0847TS448_4-D1.rsa	46.512	0.195	0.101	0.704	119	0.391	16.12
T0847TS335_5-D1.rsa	46.429	0.023	0.229	0.748	98	0.474	13.55
T0847TS011_1-D1.rsa	45.313	0.015	0.309	0.676	92	0.493	13.6
T0847TS171_1-D1.rsa	45.07	0.161	0.095	0.744	125	0.361	17.47
T0847TS345_3-D1.rsa	45.07	0.298	0	0.702	118	0.382	19.23
T0847TS184_1-D1.rsa	45.07	0.399	0	0.601	101	0.446	16.35
T0847TS436_2-D1.rsa	44.737	0.153	0	0.847	94	0.476	29.95
T0847TS216_4-D1.rsa	44.737	0.288	0.036	0.676	75	0.596	38.74
T0847TS011_2-D1.rsa	44.643	0.175	0.286	0.54	68	0.657	49.01
T0847TS300_1-D1.rsa	44.186	0.366	0.06	0.575	77	0.574	46.45
T0847TS448_5-D1.rsa	42.857	0	0.175	0.825	104	0.412	21.82
T0847TS041_3-D1.rsa	42.857	0.143	0.198	0.659	83	0.516	33.53
T0847TS210_3-D1.rsa	42.857	0.302	0.032	0.667	84	0.51	26.59
T0847TS156_2-D1.rsa	42.5	0.018	0.105	0.877	100	0.425	37.06
T0847TS210_1-D1.rsa	42.5	0.035	0.193	0.772	88	0.483	36.62
T0847TS454_5-D1.rsa	42.308	0.242	0	0.758	125	0.338	15.35
T0847TS381_5-D1.rsa	42.105	0.045	0	0.955	106	0.397	20.72
T0847TS420_3-D1.rsa	41.667	0.091	0.191	0.718	79	0.527	51.36
T0847TS268_4-D1.rsa	41.667	0.091	0.182	0.727	80	0.521	51.14
T0847TS277_1-D1.rsa	41.667	0	0.091	0.909	100	0.417	27.95
T0847TS228_5-D1.rsa	40.625	0.015	0.346	0.64	87	0.467	70.59
T0847TS133_4-D1.rsa	40.625	0.015	0.397	0.588	80	0.508	70.96
T0847TS414_2-D1.rsa	40.625	0	0.228	0.772	105	0.387	13.6
T0847TS268_3-D1.rsa	40.385	0.279	0.091	0.63	104	0.388	16.93
T0847TS133_2-D1.rsa	40	0.547	0.059	0.394	93	0.43	58.9
T0847TS410_2-D1.rsa	40	0	0.307	0.693	79	0.506	21.49
T0847TS436_1-D1.rsa	39.655	0.621	0	0.379	97	0.409	54.59
T0847TS145_2-D1.rsa	39.583	0.064	0.273	0.664	73	0.542	51.82
T0847TS133_5-D1.rsa	39.535	0.269	0.03	0.701	94	0.421	43.47
T0847TS492_4-D1.rsa	39.286	0	0.443	0.557	73	0.538	57.82
T0847TS184_4-D1.rsa	39.286	0	0.366	0.634	83	0.473	16.98
T0847TS499_2-D1.rsa	39.063	0.015	0.441	0.544	74	0.528	72.24
T0847TS492_2-D1.rsa	38.356	0.737	0	0.263	67	0.572	50.98
T0847TS436_3-D1.rsa	37.5	0.045	0.173	0.782	86	0.436	53.86
T0847TS479_3-D1.rsa	37.5	0.015	0.419	0.566	77	0.487	66.18
T0847TS263_3-D1.rsa	37.5	0	0.386	0.614	70	0.536	40.57
T0847TS345_2-D1.rsa	37.209	0.299	0.03	0.672	90	0.413	40.48
T0847TS277_3-D1.rsa	36.62	0.482	0.083	0.435	73	0.502	52.4
T0847TS216_2-D1.rsa	36.62	0.464	0.089	0.446	75	0.488	45.03
T0847TS346_1-D1.rsa	36.207	0.637	0	0.363	93	0.389	54.49
T0847TS145_4-D1.rsa	35.938	0.015	0.463	0.522	71	0.506	71.32
T0847TS216_3-D1.rsa	35.211	0.476	0.167	0.357	60	0.587	51.44

T0847TS110_5-D1.rsa	35	0	0.404	0.596	68	0.515	51.75
T0847TS349_1-D1.rsa	34.857	0.542	0.042	0.416	168	0.207	35.15
T0847TS008_4-D1.rsa	34.722	0.441	0.075	0.484	103	0.337	67.13
T0847TS381_2-D1.rsa	34.483	0.648	0	0.352	90	0.383	57.81
T0847TS228_2-D1.rsa	34.375	0.015	0.338	0.647	88	0.391	64.34
T0847TS492_1-D1.rsa	34.375	0	0.213	0.787	107	0.321	13.05
T0847TS454_2-D1.rsa	34.375	0.213	0	0.787	107	0.321	14.71
T0847TS038_4-D1.rsa	34.211	0.194	0.472	0.333	36	0.95	93.29
T0847TS454_3-D1.rsa	32.877	0.761	0	0.239	61	0.539	58.14
T0847TS210_2-D1.rsa	32.813	0.132	0.132	0.735	100	0.328	15.99
T0847TS335_1-D1.rsa	32.759	0.609	0	0.391	100	0.328	55.57
T0847TS452_4-D1.rsa	32.558	0.291	0	0.709	95	0.343	40.86
T0847TS008_5-D1.rsa	32.353	0.37	0.086	0.543	132	0.245	58.67
T0847TS210_4-D1.rsa	31.579	0.551	0.059	0.39	92	0.343	51.06
T0847TS184_2-D1.rsa	31.579	0.25	0.491	0.259	28	1.128	93.29
T0847TS110_2-D1.rsa	31.507	0.671	0	0.329	84	0.375	53.63
T0847TS038_5-D1.rsa	31.507	0.757	0	0.243	62	0.508	53.63
T0847TS492_5-D1.rsa	31.395	0.361	0.172	0.467	79	0.397	69.38
T0847TS414_5-D1.rsa	31.395	0.367	0.172	0.462	78	0.403	72.63
T0847TS237_1-D1.rsa	31.034	0.035	0.105	0.86	123	0.252	51.4
T0847TS237_3-D1.rsa	31.034	0.574	0	0.426	109	0.285	61.52
T0847TS448_3-D1.rsa	31.034	0.582	0	0.418	107	0.29	53.42
T0847TS345_5-D1.rsa	30.702	0.341	0.078	0.581	126	0.244	35.71
T0847TS436_5-D1.rsa	30.693	0.33	0.156	0.514	165	0.186	74.3
T0847TS050_1-D1.rsa	30.556	0.465	0.075	0.46	98	0.312	66.37
T0847TS184_5-D1.rsa	30.526	0.517	0.034	0.449	106	0.288	51.7
T0847TS349_5-D1.rsa	30.303	0.24	0.184	0.576	166	0.183	59.38
T0847TS011_3-D1.rsa	30.233	0.134	0.045	0.821	110	0.275	15.11
T0847TS300_2-D1.rsa	30	0.018	0.289	0.693	79	0.38	42.76
T0847TS499_3-D1.rsa	29.688	0.015	0.441	0.544	74	0.401	72.79
T0847TS420_5-D1.rsa	29.524	0.355	0.214	0.432	101	0.292	72.22
T0847TS011_5-D1.rsa	29.524	0.363	0.197	0.44	103	0.287	68.8
T0847TS349_3-D1.rsa	29.508	0	0.381	0.619	130	0.227	64.92
T0847TS300_3-D1.rsa	29.31	0.586	0	0.414	106	0.277	54.49
T0847TS420_2-D1.rsa	29.31	0.605	0	0.395	101	0.29	56.25
T0847TS038_3-D1.rsa	28.947	0.241	0.509	0.25	27	1.072	93.75
T0847TS277_4-D1.rsa	28.571	0.384	0.098	0.518	116	0.246	78.93
T0847TS171_4-D1.rsa	28.571	0.397	0.125	0.478	107	0.267	80.95
T0847TS160_1-D1.rsa	28.421	0.462	0.064	0.475	112	0.254	55.4
T0847TS263_1-D1.rsa	28	0.014	0.297	0.688	95	0.295	63.95
T0847TS381_3-D1.rsa	28	0.014	0.362	0.623	86	0.326	60.87
T0847TS212_1-D1.rsa	28	0.051	0.428	0.522	72	0.389	70.83
T0847TS263_4-D1.rsa	28	0.051	0.326	0.623	86	0.326	54.17
T0847TS022_1-D1.rsa	27.941	0.416	0.111	0.473	115	0.243	67.33
T0847TS008_2-D1.rsa	27.619	0.338	0.201	0.462	108	0.256	70.94
T0847TS499_4-D1.rsa	27.368	0.462	0.051	0.487	115	0.238	56.89
T0847TS041_4-D1.rsa	27.273	0.191	0.069	0.74	213	0.128	59.55

T0847TS345_1-D1.rsa	26.923	0.399	0.111	0.49	145	0.186	56.51
T0847TS171_2-D1.rsa	26.744	0.325	0.095	0.58	98	0.273	74.7
T0847TS008_3-D1.rsa	26.724	0.668	0	0.332	85	0.314	62.5
T0847TS448_2-D1.rsa	26	0.454	0.143	0.402	101	0.257	71.91
T0847TS251_5-D1.rsa	25.641	0.378	0.135	0.486	144	0.178	57.2
T0847TS160_3-D1.rsa	25.641	0.422	0.135	0.443	131	0.196	56.34
T0847TS160_4-D1.rsa	25	0.362	0.095	0.543	132	0.189	69.44
T0847TS237_4-D1.rsa	25	0.366	0.099	0.535	130	0.192	67.11
T0847TS133_1-D1.rsa	24	0.065	0.37	0.565	78	0.308	69.93
T0847TS041_5-D1.rsa	24	0	0.138	0.862	119	0.202	26.63
T0847TS073_1-D1.rsa	24	0.036	0.087	0.877	121	0.198	15.22
T0847TS277_2-D1.rsa	24	0	0.159	0.841	116	0.207	17.21
T0847TS300_5-D1.rsa	23.377	0.415	0.107	0.478	107	0.218	64.76
T0847TS237_2-D1.rsa	23.077	0.409	0.135	0.456	135	0.171	57.47
T0847TS268_1-D1.rsa	22.5	0.114	0	0.886	101	0.223	33.77
T0847TS479_1-D1.rsa	22.078	0.406	0.107	0.487	109	0.203	78.93
T0847TS117_4-D1.rsa	20.779	0.362	0.134	0.504	113	0.184	72.98
T0847TS499_5-D1.rsa	19.298	0.361	0.1	0.539	118	0.164	73.17
T0847TS349_4-D1.rsa	18.605	0.406	0.131	0.463	131	0.142	69.7
T0847TS041_2-D1.rsa	16.279	0.371	0.092	0.537	152	0.107	70.05
T0847TS117_5-D1.rsa	14.474	0.316	0.111	0.572	190	0.076	91.34
T0847TS436_4-D1.rsa	14.474	0.325	0.12	0.554	184	0.079	98.72
T0847TS117_2-D1.rsa	13.846	0.258	0.231	0.511	234	0.059	70.28
T0847TS251_2-D1.rsa	12.766	0.35	0.187	0.463	119	0.107	55.45
T0847TS184_3-D1.rsa	12	0.014	0.297	0.688	95	0.126	48.73
T0847TS349_2-D1.rsa	10.638	0.323	0.179	0.498	128	0.083	85.31
T0821TS277_1-D1.rsa	76.471	0.77	0	0.23	29	2.637	20.44
T0821TS038_5-D1.rsa	67.647	0.698	0	0.302	38	1.78	20.64
T0821TS454_1-D1.rsa	65.789	0.748	0	0.252	28	2.35	16.22
T0821TS011_2-D1.rsa	63.158	0.486	0	0.514	57	1.108	16.22
T0821TS349_3-D1.rsa	63.158	0.703	0	0.297	33	1.914	18.69
T0821TS117_4-D1.rsa	61.765	0.706	0	0.294	37	1.669	22.62
T0821TS041_3-D1.rsa	61.538	0.285	0.073	0.642	106	0.581	76.74
T0821TS216_4-D1.rsa	60.714	0.656	0	0.344	45	1.349	10.3
T0821TS410_4-D1.rsa	60.526	0.396	0	0.604	67	0.903	13.74
T0821TS300_4-D1.rsa	60.526	0.712	0	0.288	32	1.891	19.37
T0821TS210_3-D1.rsa	58.929	0.706	0.016	0.278	35	1.684	15.28
T0821TS454_4-D1.rsa	58.824	0.675	0	0.325	41	1.435	35.91
T0821TS410_5-D1.rsa	57.895	0.25	0.454	0.296	32	1.809	46.06
T0821TS156_5-D1.rsa	57.143	0.031	0.374	0.595	78	0.733	17.37
T0821TS414_4-D1.rsa	55.882	0.675	0	0.325	41	1.363	16.67
T0821TS300_1-D1.rsa	55.263	0.694	0	0.306	34	1.625	19.59
T0821TS499_3-D1.rsa	54.167	0	0.445	0.555	61	0.888	17.5
T0821TS156_4-D1.rsa	52.632	0.25	0.444	0.306	33	1.595	48.61
T0821TS228_5-D1.rsa	52.5	0.175	0.351	0.474	54	0.972	15.57
T0821TS345_4-D1.rsa	52.5	0.175	0.333	0.491	56	0.938	15.79
T0821TS038_3-D1.rsa	51.163	0.701	0	0.299	40	1.279	22.95

T0821TS492_3-D1.rsa	50	0.378	0	0.622	69	0.725	27.03
T0821TS448_5-D1.rsa	50	0.063	0.444	0.492	62	0.806	17.26
T0821TS133_1-D1.rsa	50	0.018	0.535	0.447	51	0.98	18.64
T0821TS184_5-D1.rsa	50	0.706	0	0.294	37	1.351	21.82
T0821TS349_5-D1.rsa	48.837	0.687	0	0.313	42	1.163	22.2
T0821TS160_4-D1.rsa	47.5	0	0.307	0.693	79	0.601	17.54
T0821TS145_4-D1.rsa	47.368	0.315	0.09	0.595	66	0.718	18.92
T0821TS436_3-D1.rsa	45.313	0.088	0.228	0.684	93	0.487	13.79
T0821TS011_5-D1.rsa	45.313	0.11	0.419	0.471	64	0.708	15.07
T0821TS228_3-D1.rsa	45.313	0.316	0.154	0.529	72	0.629	13.6
T0821TS251_3-D1.rsa	45	0.018	0.544	0.439	50	0.9	21.27
T0821TS414_2-D1.rsa	44.737	0.559	0	0.441	49	0.913	18.47
T0821TS335_5-D1.rsa	44.231	0.661	0	0.339	56	0.79	14.4
T0821TS277_4-D1.rsa	44.231	0.727	0	0.273	45	0.983	14.4
T0821TS436_5-D1.rsa	44.118	0.329	0	0.671	163	0.271	25.44
T0821TS171_2-D1.rsa	44.118	0.643	0	0.357	45	0.98	54.17
T0821TS022_5-D1.rsa	44.118	0.754	0	0.246	31	1.423	47.42
T0821TS008_5-D1.rsa	43.75	0.018	0.373	0.609	67	0.653	20.68
T0821TS335_1-D1.rsa	43.75	0.118	0.441	0.441	60	0.729	13.42
T0821TS381_1-D1.rsa	43.75	0.11	0.471	0.419	57	0.768	13.05
T0821TS212_3-D1.rsa	43.662	0.53	0.113	0.357	60	0.728	33.97
T0821TS279_1-D1.rsa	42.5	0	0.465	0.535	61	0.697	16.45
T0821TS300_5-D1.rsa	42.5	0	0.561	0.439	50	0.85	17.11
T0821TS452_2-D1.rsa	42.5	0	0.544	0.456	52	0.817	16.89
T0821TS251_2-D1.rsa	42.188	0.125	0.434	0.441	60	0.703	12.68
T0821TS251_5-D1.rsa	42.105	0.495	0.018	0.486	54	0.78	20.27
T0821TS479_5-D1.rsa	41.176	0.762	0	0.238	30	1.373	52.98
T0821TS145_2-D1.rsa	41.071	0.214	0.246	0.54	68	0.604	25
T0821TS499_2-D1.rsa	40.845	0.53	0.036	0.435	73	0.56	20.51
T0821TS345_3-D1.rsa	40.625	0.654	0	0.346	47	0.864	14.34
T0821TS160_2-D1.rsa	40	0	0.43	0.57	65	0.615	26.75
T0821TS349_2-D1.rsa	40	0	0.482	0.518	59	0.678	22.37
T0821TS492_1-D1.rsa	40	0	0.439	0.561	64	0.625	26.32
T0821TS410_3-D1.rsa	40	0	0.404	0.596	68	0.588	16.45
T0821TS193_3-D1.rsa	40	0.283	0.152	0.565	78	0.513	14.67
T0821TS133_4-D1.rsa	40	0.018	0.535	0.447	51	0.784	28.51
T0821TS420_1-D1.rsa	40	0	0.474	0.526	60	0.667	24.34
T0821TS479_2-D1.rsa	40	0	0.509	0.491	56	0.714	16.45
T0821TS210_4-D1.rsa	40	0.018	0.535	0.447	51	0.784	21.05
T0821TS345_2-D1.rsa	40	0	0.474	0.526	60	0.667	16.89
T0821TS268_1-D1.rsa	40	0	0.588	0.412	47	0.851	17.32
T0821TS300_2-D1.rsa	40	0	0.544	0.456	52	0.769	17.11
T0821TS184_3-D1.rsa	40	0	0.535	0.465	53	0.755	16.01
T0821TS499_5-D1.rsa	40	0	0.535	0.465	53	0.755	16.23
T0821TS212_1-D1.rsa	39.286	0.175	0.349	0.476	60	0.655	30.16
T0821TS184_2-D1.rsa	39.063	0.699	0	0.301	41	0.953	11.77
T0821TS184_1-D1.rsa	38.235	0.675	0	0.325	41	0.933	20.04

T0821TS492_4-D1.rsa	37.5	0.103	0.346	0.551	75	0.5	12.32
T0821TS133_2-D1.rsa	37.5	0.018	0.325	0.658	75	0.5	24.56
T0821TS228_1-D1.rsa	37.5	0	0.474	0.526	60	0.625	20.61
T0821TS210_1-D1.rsa	37.5	0.018	0.491	0.491	56	0.67	19.96
T0821TS479_1-D1.rsa	37.5	0.214	0.421	0.365	46	0.815	38.29
T0821TS263_3-D1.rsa	37.5	0	0.509	0.491	56	0.67	16.01
T0821TS335_3-D1.rsa	37.5	0	0.57	0.43	49	0.765	17.11
T0821TS414_3-D1.rsa	37.5	0	0.57	0.43	49	0.765	17.11
T0821TS452_3-D1.rsa	37.5	0.018	0.553	0.43	49	0.765	23.9
T0821TS133_5-D1.rsa	36.986	0.78	0	0.22	56	0.66	38.43
T0821TS277_3-D1.rsa	35.714	0.19	0.333	0.476	60	0.595	38.29
T0821TS263_4-D1.rsa	35	0.035	0.263	0.702	80	0.438	20.18
T0821TS160_5-D1.rsa	35	0	0.439	0.561	64	0.547	26.32
T0821TS349_1-D1.rsa	35	0.018	0.421	0.561	64	0.547	16.89
T0821TS381_5-D1.rsa	35	0	0.351	0.649	74	0.473	14.47
T0821TS335_4-D1.rsa	35	0.026	0.412	0.561	64	0.547	26.75
T0821TS171_4-D1.rsa	34.247	0.741	0	0.259	66	0.519	37.74
T0821TS492_5-D1.rsa	33.803	0.518	0.107	0.375	63	0.537	24.04
T0821TS420_2-D1.rsa	32.813	0.088	0.346	0.566	77	0.426	13.05
T0821TS454_2-D1.rsa	32.692	0.685	0	0.315	52	0.629	15.03
T0821TS345_1-D1.rsa	32.558	0.403	0.067	0.53	71	0.459	21.27
T0821TS277_2-D1.rsa	32.5	0.026	0.281	0.693	79	0.411	23.68
T0821TS011_3-D1.rsa	32.5	0	0.263	0.737	84	0.387	16.45
T0821TS251_4-D1.rsa	32.5	0	0.474	0.526	60	0.542	25.22
T0821TS184_4-D1.rsa	32.5	0.053	0.404	0.544	62	0.524	20.18
T0821TS479_4-D1.rsa	32.353	0.579	0	0.421	53	0.61	46.43
T0821TS145_1-D1.rsa	32.353	0.563	0	0.437	55	0.588	40.28
T0821TS436_2-D1.rsa	32.143	0.198	0.365	0.437	55	0.584	50.59
T0821TS171_1-D1.rsa	32.143	0.175	0.397	0.429	54	0.595	52.18
T0821TS216_5-D1.rsa	32	0.217	0.254	0.529	73	0.438	11.59
T0821TS251_1-D1.rsa	31.579	0.278	0.361	0.361	39	0.81	66.2
T0821TS277_5-D1.rsa	30.769	0.364	0.206	0.43	71	0.433	15.66
T0821TS420_4-D1.rsa	30.357	0.159	0.373	0.468	59	0.515	41.47
T0821TS210_2-D1.rsa	30.233	0.433	0.03	0.537	72	0.42	23.13
T0821TS263_1-D1.rsa	30	0.018	0.377	0.605	69	0.435	36.84
T0821TS145_5-D1.rsa	30	0	0.351	0.649	74	0.405	23.25
T0821TS448_4-D1.rsa	30	0	0.43	0.57	65	0.462	19.3
T0821TS499_4-D1.rsa	30	0.035	0.377	0.588	67	0.448	27.19
T0821TS237_5-D1.rsa	30	0	0.342	0.658	75	0.4	15.35
T0821TS452_1-D1.rsa	30	0.026	0.482	0.491	56	0.536	21.27
T0821TS454_5-D1.rsa	30	0.044	0.561	0.395	45	0.667	16.67
T0821TS414_1-D1.rsa	29.688	0.426	0	0.574	78	0.381	15.26
T0821TS008_2-D1.rsa	29.577	0.595	0	0.405	68	0.435	23.08
T0821TS216_1-D1.rsa	29.577	0.643	0	0.357	60	0.493	25.16
T0821TS237_3-D1.rsa	29.412	0.73	0	0.27	34	0.865	55.95
T0821TS210_5-D1.rsa	29.31	0.688	0	0.313	80	0.366	29
T0821TS011_4-D1.rsa	28.947	0.287	0.38	0.333	36	0.804	69.68

T0821TS237_2-D1.rsa	28.947	0.25	0.389	0.361	39	0.742	58.56
T0821TS335_2-D1.rsa	28.947	0.278	0.435	0.287	31	0.934	56.94
T0821TS410_1-D1.rsa	28.571	0.198	0.365	0.437	55	0.519	51.39
T0821TS268_3-D1.rsa	28.448	0.723	0	0.277	71	0.401	24.51
T0821TS022_4-D1.rsa	28.169	0.494	0.167	0.339	57	0.494	51.6
T0821TS410_2-D1.rsa	28.125	0.096	0.324	0.581	79	0.356	16.18
T0821TS212_2-D1.rsa	28.125	0.096	0.324	0.581	79	0.356	15.62
T0821TS263_2-D1.rsa	28.125	0.096	0.257	0.647	88	0.32	13.05
T0821TS041_4-D1.rsa	28.125	0.169	0.25	0.581	79	0.356	12.32
T0821TS300_3-D1.rsa	27.083	0.355	0.036	0.609	67	0.404	29.32
T0821TS171_5-D1.rsa	26.786	0.151	0.341	0.508	64	0.419	42.26
T0821TS160_3-D1.rsa	26.786	0.159	0.333	0.508	64	0.419	47.82
T0821TS008_4-D1.rsa	26.786	0.151	0.389	0.46	58	0.462	50.2
T0821TS228_2-D1.rsa	26.786	0.159	0.381	0.46	58	0.462	40.87
T0821TS228_4-D1.rsa	26.563	0.096	0.324	0.581	79	0.336	16.36
T0821TS268_5-D1.rsa	25.581	0.396	0.134	0.47	63	0.406	28.55
T0821TS436_1-D1.rsa	25	0.382	0.112	0.506	127	0.197	88.35
T0821TS263_5-D1.rsa	25	0.175	0.413	0.413	52	0.481	54.17
T0821TS050_1-D1.rsa	25	0.096	0.316	0.588	80	0.313	16.36
T0821TS117_3-D1.rsa	25	0.175	0.437	0.389	49	0.51	53.77
T0821TS117_1-D1.rsa	24.658	0.749	0	0.251	64	0.385	38.73
T0821TS160_1-D1.rsa	24	0.043	0.203	0.754	104	0.231	65.94
T0821TS156_3-D1.rsa	23.438	0.096	0.309	0.596	81	0.289	15.44
T0821TS448_3-D1.rsa	23.438	0.096	0.309	0.596	81	0.289	15.26
T0821TS452_5-D1.rsa	23.256	0.455	0.082	0.463	62	0.375	28.92
T0821TS349_4-D1.rsa	22.535	0.679	0	0.321	54	0.417	25.64
T0821TS038_2-D1.rsa	22.5	0	0.316	0.684	78	0.288	29.39
T0821TS452_4-D1.rsa	22.5	0.026	0.36	0.614	70	0.321	18.42
T0821TS145_3-D1.rsa	21.875	0.096	0.294	0.61	83	0.264	16.54
T0821TS216_2-D1.rsa	20.588	0.619	0	0.381	48	0.429	45.04
T0821TS436_4-D1.rsa	20.339	0.028	0.238	0.734	157	0.13	56.08
T0821TS346_1-D1.rsa	16	0.029	0.355	0.616	85	0.188	49.64
T0821TS041_2-D1.rsa	15.254	0	0.308	0.692	148	0.103	64.95
T0821TS117_2-D1.rsa	14.615	0.236	0.188	0.576	264	0.055	72.33
T0821TS117_5-D1.rsa	8.511	0.327	0.191	0.482	124	0.069	83.46
T0849TS448_2-D1.rsa	86.842	0.279	0.09	0.631	70	1.241	19.37
T0849TS133_4-D1.rsa	73.077	0.624	0	0.376	62	1.179	17.88
T0849TS117_5-D1.rsa	69.118	0.342	0.016	0.642	156	0.443	9.22
T0849TS448_5-D1.rsa	67.308	0.358	0.012	0.63	104	0.647	11.08
T0849TS145_3-D1.rsa	62.5	0.1	0.318	0.582	64	0.977	26.14
T0849TS160_1-D1.rsa	61.765	0.571	0	0.429	54	1.144	31.55
T0849TS381_3-D1.rsa	61.765	0.516	0	0.484	61	1.013	30.36
T0849TS117_4-D1.rsa	60.714	0.31	0.048	0.643	81	0.75	17.66
T0849TS145_2-D1.rsa	60.714	0	0.328	0.672	88	0.69	15.08
T0849TS279_1-D1.rsa	60.526	0.225	0.072	0.703	78	0.776	38.96
T0849TS335_1-D1.rsa	58.824	0.587	0	0.413	52	1.131	33.53
T0849TS184_2-D1.rsa	58.824	0.54	0	0.46	58	1.014	33.73

T0849TS008_4-D1.rsa	57.813	0.316	0	0.684	93	0.622	10.29
T0849TS448_1-D1.rsa	57.143	0.015	0.321	0.664	87	0.657	17.75
T0849TS237_2-D1.rsa	56	0.188	0.261	0.551	76	0.737	12.5
T0849TS345_4-D1.rsa	55.357	0.31	0.159	0.532	67	0.826	24.21
T0849TS420_5-D1.rsa	55.263	0.216	0.171	0.613	68	0.813	29.28
T0849TS349_5-D1.rsa	54.167	0.019	0.278	0.704	76	0.713	21.3
T0849TS345_2-D1.rsa	54.167	0	0.218	0.782	86	0.63	18.41
T0849TS011_5-D1.rsa	53.571	0	0.321	0.679	89	0.602	15.27
T0849TS228_5-D1.rsa	53.571	0	0.382	0.618	81	0.661	17.56
T0849TS160_3-D1.rsa	53.571	0.023	0.366	0.611	80	0.67	16.79
T0849TS110_3-D1.rsa	53.571	0.175	0.325	0.5	63	0.85	21.03
T0849TS008_1-D1.rsa	53.488	0.418	0.082	0.5	67	0.798	25.19
T0849TS414_1-D1.rsa	53.125	0.022	0.324	0.654	89	0.597	14.15
T0849TS160_4-D1.rsa	52.941	0.524	0	0.476	60	0.882	29.96
T0849TS410_2-D1.rsa	51.563	0.154	0.184	0.662	90	0.573	13.23
T0849TS237_5-D1.rsa	51.163	0.313	0.097	0.59	79	0.648	28.55
T0849TS206_1-D1.rsa	50	0.333	0	0.667	84	0.595	26.19
T0849TS499_1-D1.rsa	50	0	0.278	0.722	78	0.641	21.3
T0849TS073_2-D1.rsa	50	0.135	0.159	0.706	89	0.562	15.68
T0849TS479_3-D1.rsa	50	0	0.39	0.61	83	0.602	12.5
T0849TS263_2-D1.rsa	50	0.008	0.382	0.611	80	0.625	17.37
T0849TS251_1-D1.rsa	50	0.056	0.287	0.657	71	0.704	25
T0849TS454_3-D1.rsa	50	0	0.359	0.641	84	0.595	17.37
T0849TS335_5-D1.rsa	47.5	0	0.316	0.684	78	0.609	19.52
T0849TS228_2-D1.rsa	47.368	0.279	0.108	0.613	68	0.697	37.16
T0849TS499_3-D1.rsa	47.368	0.396	0.081	0.523	58	0.817	42.79
T0849TS479_4-D1.rsa	46.875	0.059	0.294	0.647	88	0.533	13.6
T0849TS216_4-D1.rsa	46.575	0.702	0	0.298	76	0.613	28.23
T0849TS448_4-D1.rsa	46.429	0	0.267	0.733	96	0.484	19.47
T0849TS277_2-D1.rsa	46.429	0	0.359	0.641	84	0.553	17.56
T0849TS110_5-D1.rsa	46.429	0.023	0.252	0.725	95	0.489	16.22
T0849TS216_1-D1.rsa	45.833	0.056	0.491	0.454	49	0.935	54.4
T0849TS499_2-D1.rsa	45	0	0.333	0.667	76	0.592	16.67
T0849TS110_4-D1.rsa	44.737	0.241	0.426	0.333	36	1.243	71.99
T0849TS452_1-D1.rsa	44.737	0.241	0.37	0.389	42	1.065	67.59
T0849TS160_2-D1.rsa	44.231	0.412	0.103	0.485	80	0.553	17.09
T0849TS452_5-D1.rsa	43.836	0.698	0	0.302	77	0.569	30.59
T0849TS228_3-D1.rsa	43.75	0.027	0.382	0.591	65	0.673	62.5
T0849TS160_5-D1.rsa	43.75	0.091	0.264	0.645	71	0.616	48.64
T0849TS041_3-D1.rsa	43.75	0.132	0.257	0.61	83	0.527	13.05
T0849TS420_2-D1.rsa	43.75	0.118	0.294	0.588	80	0.547	13.79
T0849TS117_2-D1.rsa	43.103	0.014	0.063	0.923	132	0.327	55.59
T0849TS237_4-D1.rsa	42.857	0	0.328	0.672	88	0.487	17.56
T0849TS381_2-D1.rsa	42.857	0.046	0.176	0.779	102	0.42	12.02
T0849TS041_5-D1.rsa	42.857	0.373	0.016	0.611	77	0.557	19.05
T0849TS038_1-D1.rsa	42.857	0	0.336	0.664	87	0.493	18.13
T0849TS300_1-D1.rsa	42.5	0	0.404	0.596	68	0.625	26.97

T0849TS277_4-D1.rsa	42.5	0.018	0.404	0.579	66	0.644	33.11
T0849TS008_5-D1.rsa	42.254	0.411	0.131	0.458	77	0.549	41.19
T0849TS145_4-D1.rsa	42.188	0.096	0.257	0.647	88	0.479	12.68
T0849TS448_3-D1.rsa	41.667	0.064	0.291	0.645	71	0.587	50
T0849TS436_1-D1.rsa	41.667	0	0.463	0.537	58	0.718	56.94
T0849TS251_4-D1.rsa	40.625	0.088	0.191	0.721	98	0.415	14.34
T0849TS251_3-D1.rsa	39.726	0.706	0	0.294	75	0.53	39.8
T0849TS216_3-D1.rsa	39.726	0.718	0	0.282	72	0.552	39.22
T0849TS410_5-D1.rsa	39.583	0.127	0.373	0.5	55	0.72	52.73
T0849TS335_2-D1.rsa	39.286	0.19	0.31	0.5	63	0.624	42.86
T0849TS263_3-D1.rsa	39.286	0.015	0.344	0.641	84	0.468	16.6
T0849TS499_5-D1.rsa	37.5	0.19	0.325	0.484	61	0.615	42.86
T0849TS038_4-D1.rsa	37.5	0	0.211	0.789	90	0.417	20.83
T0849TS268_4-D1.rsa	37.5	0.183	0.294	0.524	66	0.568	37.7
T0849TS038_2-D1.rsa	37.5	0.035	0.386	0.579	66	0.568	25.66
T0849TS041_2-D1.rsa	37.5	0.184	0.147	0.669	91	0.412	12.32
T0849TS414_2-D1.rsa	37.209	0.299	0.104	0.597	80	0.465	26.12
T0849TS300_4-D1.rsa	37.209	0.418	0.037	0.545	73	0.51	35.45
T0849TS436_5-D1.rsa	37.069	0.664	0	0.336	86	0.431	55.27
T0849TS011_2-D1.rsa	36.842	0.259	0.352	0.389	42	0.877	62.27
T0849TS041_4-D1.rsa	36.62	0.214	0.012	0.774	130	0.282	34.45
T0849TS479_5-D1.rsa	36.62	0.429	0.012	0.56	94	0.39	43.91
T0849TS410_1-D1.rsa	36.62	0.494	0.137	0.369	62	0.591	51.44
T0849TS420_4-D1.rsa	36	0.043	0.196	0.761	105	0.343	14.86
T0849TS038_5-D1.rsa	35.714	0.167	0.063	0.77	97	0.368	20.04
T0849TS452_2-D1.rsa	35.211	0.399	0.071	0.53	89	0.396	32.53
T0849TS420_3-D1.rsa	35.211	0.53	0.101	0.369	62	0.568	51.92
T0849TS216_2-D1.rsa	35	0.018	0.158	0.825	94	0.372	15.57
T0849TS237_3-D1.rsa	34.211	0.25	0.435	0.315	34	1.006	93.06
T0849TS038_3-D1.rsa	33.929	0.175	0.333	0.492	62	0.547	51.39
T0849TS210_2-D1.rsa	33.929	0.206	0.183	0.611	77	0.441	39.68
T0849TS345_3-D1.rsa	32.877	0.765	0	0.235	60	0.548	49.8
T0849TS345_5-D1.rsa	32.394	0.488	0.071	0.44	74	0.438	34.3
T0849TS210_3-D1.rsa	32.394	0.476	0.071	0.452	76	0.426	37.66
T0849TS263_5-D1.rsa	32.143	0.198	0.048	0.754	95	0.338	35.91
T0849TS335_4-D1.rsa	32.143	0.159	0.333	0.508	64	0.502	49.01
T0849TS414_3-D1.rsa	32.143	0.175	0.357	0.468	59	0.545	51.19
T0849TS349_3-D1.rsa	31.579	0.241	0.509	0.25	27	1.17	93.52
T0849TS216_5-D1.rsa	31.579	0.259	0.463	0.278	30	1.053	89.35
T0849TS145_1-D1.rsa	31.507	0.765	0	0.235	60	0.525	55.29
T0849TS268_3-D1.rsa	31.507	0.765	0	0.235	60	0.525	50.69
T0849TS110_1-D1.rsa	30.986	0.476	0.161	0.363	61	0.508	62.82
T0849TS133_3-D1.rsa	30.986	0.512	0.089	0.399	67	0.462	51.44
T0849TS436_3-D1.rsa	30.526	0.521	0.055	0.424	100	0.305	58.58
T0849TS349_2-D1.rsa	30.357	0.127	0.278	0.595	75	0.405	49.21
T0849TS184_5-D1.rsa	30.357	0.175	0.325	0.5	63	0.482	53.37
T0849TS117_1-D1.rsa	30.357	0.159	0.183	0.659	83	0.366	21.43

T0849TS073_1-D1.rsa	30.357	0.127	0.262	0.611	77	0.394	42.66
T0849TS133_5-D1.rsa	30.357	0.175	0.341	0.484	61	0.498	48.41
T0849TS011_1-D1.rsa	30.233	0.306	0.104	0.59	79	0.383	30.6
T0849TS277_5-D1.rsa	30.137	0.745	0	0.255	65	0.464	59.12
T0849TS479_1-D1.rsa	28.947	0.241	0.481	0.278	30	0.965	92.13
T0849TS381_5-D1.rsa	28.947	0.241	0.472	0.287	31	0.934	91.67
T0849TS414_5-D1.rsa	28.947	0.287	0.046	0.667	72	0.402	60.19
T0849TS251_2-D1.rsa	28.947	0.25	0.509	0.241	26	1.113	94.68
T0849TS008_3-D1.rsa	28.947	0.25	0.5	0.25	27	1.072	94.44
T0849TS251_5-D1.rsa	28.947	0.25	0.509	0.241	26	1.113	88.89
T0849TS184_1-D1.rsa	28.947	0.269	0.278	0.454	49	0.591	70.37
T0849TS335_3-D1.rsa	28.571	0.151	0.31	0.54	68	0.42	49.6
T0849TS300_5-D1.rsa	28.571	0.151	0.286	0.563	71	0.402	50.59
T0849TS133_2-D1.rsa	28.571	0.159	0.302	0.54	68	0.42	48.81
T0849TS345_1-D1.rsa	28	0.109	0.275	0.616	85	0.329	19.57
T0849TS145_5-D1.rsa	27.5	0.035	0.368	0.596	68	0.404	46.93
T0849TS381_1-D1.rsa	27.5	0.044	0.368	0.588	67	0.41	46.27
T0849TS117_3-D1.rsa	27.5	0.053	0.175	0.772	88	0.313	22.81
T0849TS212_1-D1.rsa	27.5	0	0.404	0.596	68	0.404	47.81
T0849TS237_1-D1.rsa	27.5	0.07	0.07	0.86	98	0.281	17.76
T0849TS263_4-D1.rsa	26.786	0.175	0.302	0.524	66	0.406	50.79
T0849TS349_1-D1.rsa	26.316	0.241	0.509	0.25	27	0.975	93.98
T0849TS184_4-D1.rsa	26.316	0.25	0.426	0.324	35	0.752	94.44
T0849TS210_1-D1.rsa	26.316	0.25	0.5	0.25	27	0.975	93.98
T0849TS300_2-D1.rsa	25	0.018	0.307	0.675	77	0.325	35.75
T0849TS228_4-D1.rsa	24	0.051	0.297	0.652	90	0.267	56.52
T0849TS228_1-D1.rsa	24	0.08	0.362	0.558	77	0.312	56.88
T0849TS133_1-D1.rsa	23.256	0.306	0.015	0.679	91	0.256	24.63
T0849TS454_5-D1.rsa	23.158	0.547	0.051	0.403	95	0.244	34
T0849TS414_4-D1.rsa	20	0	0.217	0.783	108	0.185	40.94
T0849TS268_1-D1.rsa	20	0.029	0.319	0.652	90	0.222	49.09
T0849TS011_4-D1.rsa	12	0.058	0.275	0.667	92	0.13	46.56
T0848TS296_4-D1.rsa	82.813	0.051	0.066	0.882	120	0.69	11.4
T0848TS133_2-D1.rsa	75	0.133	0	0.867	143	0.524	18.51
T0848TS454_4-D1.rsa	75	0	0.374	0.626	82	0.915	60.69
T0848TS097_3-D1.rsa	68.75	0.14	0.169	0.691	94	0.731	37.13
T0848TS216_1-D1.rsa	68.421	0.135	0.018	0.847	94	0.728	36.71
T0848TS067_3-D1.rsa	67.308	0.206	0.133	0.661	109	0.618	43.83
T0848TS116_4-D1.rsa	63.462	0.279	0.073	0.648	107	0.593	62.18
T0848TS169_3-D1.rsa	63.462	0.218	0.176	0.606	100	0.635	43.83
T0848TS050_1-D1.rsa	62.791	0.216	0	0.784	105	0.598	39.55
T0848TS492_2-D1.rsa	61.538	0.261	0	0.739	122	0.504	75
T0848TS277_2-D1.rsa	61.538	0.279	0.085	0.636	105	0.586	77.22
T0848TS333_4-D1.rsa	61.538	0.291	0.073	0.636	105	0.586	71.2
T0848TS153_3-D1.rsa	61.538	0.285	0.103	0.612	101	0.609	65.51
T0848TS420_5-D1.rsa	60.714	0	0.061	0.939	123	0.494	15.08
T0848TS290_4-D1.rsa	60.714	0	0.321	0.679	89	0.682	47.9

T0848TS067_5-D1.rsa	60.465	0.224	0.03	0.746	100	0.605	37.69
T0848TS145_2-D1.rsa	58.333	0	0.583	0.417	45	1.296	63.19
T0848TS210_1-D1.rsa	57.692	0.321	0.127	0.552	91	0.634	73.58
T0848TS347_2-D1.rsa	57.692	0.255	0.139	0.606	100	0.577	71.36
T0848TS184_3-D1.rsa	57.692	0.206	0.109	0.685	113	0.511	49.52
T0848TS145_5-D1.rsa	57.143	0.031	0.313	0.656	86	0.664	36.64
T0848TS326_4-D1.rsa	55.814	0.201	0	0.799	135	0.413	14.05
T0848TS479_2-D1.rsa	55.769	0.279	0.097	0.624	103	0.541	67.25
T0848TS049_1-D1.rsa	55.769	0.024	0.152	0.824	136	0.41	11.23
T0848TS184_4-D1.rsa	55.769	0.285	0.085	0.63	104	0.536	53.32
T0848TS448_3-D1.rsa	55.682	0.014	0.385	0.601	178	0.313	41.98
T0848TS347_5-D1.rsa	53.846	0.248	0.061	0.691	114	0.472	54.27
T0848TS097_2-D1.rsa	53.846	0.285	0.115	0.6	99	0.544	62.18
T0848TS184_5-D1.rsa	53.571	0	0.374	0.626	82	0.653	54.77
T0848TS296_1-D1.rsa	50	0.354	0.041	0.605	147	0.34	14.56
T0848TS300_4-D1.rsa	48.864	0.037	0.267	0.696	206	0.237	38.18
T0848TS008_1-D1.rsa	47.059	0.366	0.062	0.572	139	0.339	56.67
T0848TS277_4-D1.rsa	46.552	0	0.112	0.888	127	0.367	55.42
T0848TS038_5-D1.rsa	46.154	0.261	0.085	0.655	108	0.427	44.94
T0848TS310_4-D1.rsa	46.154	0.285	0.097	0.618	102	0.452	39.24
T0848TS454_2-D1.rsa	45.833	0.343	0.094	0.563	120	0.382	63.58
T0848TS145_4-D1.rsa	44.231	0.176	0	0.824	136	0.325	25.16
T0848TS445_4-D1.rsa	44.231	0.315	0.097	0.588	97	0.456	54.11
T0848TS160_1-D1.rsa	44.231	0.164	0.03	0.806	133	0.333	11.39
T0848TS282_4-D1.rsa	43.75	0.191	0.059	0.75	102	0.429	38.42
T0848TS157_4-D1.rsa	43.103	0	0.119	0.881	126	0.342	58.04
T0848TS448_2-D1.rsa	43.103	0	0.133	0.867	124	0.348	61.54
T0848TS041_4-D1.rsa	43.103	0	0.112	0.888	127	0.339	58.92
T0848TS358_5-D1.rsa	43.103	0	0.091	0.909	130	0.332	44.23
T0848TS381_4-D1.rsa	42.424	0.191	0.139	0.67	193	0.22	52.43
T0848TS277_5-D1.rsa	42.045	0.037	0.436	0.527	156	0.27	20.69
T0848TS358_2-D1.rsa	42.045	0.064	0.429	0.507	150	0.28	27.79
T0848TS268_5-D1.rsa	40.984	0.024	0.31	0.667	140	0.293	40.17
T0848TS077_2-D1.rsa	40.741	0.239	0	0.761	347	0.117	6.95
T0848TS381_1-D1.rsa	40.678	0.023	0.159	0.818	175	0.232	47.43
T0848TS333_5-D1.rsa	40.625	0.015	0.368	0.618	84	0.484	70.59
T0848TS457_2-D1.rsa	40.278	0.38	0.094	0.526	112	0.36	76.27
T0848TS011_1-D1.rsa	40	0	0.195	0.805	265	0.151	21.43
T0848TS479_3-D1.rsa	39.773	0.054	0.446	0.5	148	0.269	37.58
T0848TS326_1-D1.rsa	39.344	0.029	0.41	0.562	118	0.333	71.39
T0848TS338_2-D1.rsa	39.286	0	0.427	0.573	75	0.524	66.41
T0848TS132_2-D1.rsa	38.983	0.056	0.308	0.636	136	0.287	29.21
T0848TS117_1-D1.rsa	38.889	0.305	0.103	0.592	126	0.309	70.05
T0848TS169_1-D1.rsa	38.889	0.427	0.08	0.493	105	0.37	69.29
T0848TS310_1-D1.rsa	38.889	0.479	0.08	0.441	94	0.414	71.07
T0848TS322_2-D1.rsa	38.636	0.105	0.405	0.49	145	0.266	44.51
T0848TS144_2-D1.rsa	38.636	0.061	0.405	0.534	158	0.245	38.34

T0848TS326_5-D1.rsa	38.596	0.309	0.147	0.544	118	0.327	57.95
T0848TS425_1-D1.rsa	38.372	0.278	0.172	0.55	93	0.413	69.38
T0848TS436_4-D1.rsa	37.895	0.534	0.042	0.424	100	0.379	58.69
T0848TS479_1-D1.rsa	37.719	0.244	0.166	0.59	128	0.295	63.59
T0848TS391_3-D1.rsa	37.705	0.029	0.395	0.576	121	0.312	62.44
T0848TS237_1-D1.rsa	37.5	0.057	0.358	0.584	173	0.217	40.79
T0848TS277_1-D1.rsa	37.5	0.068	0.419	0.514	152	0.247	35.05
T0848TS457_1-D1.rsa	37.5	0.366	0.052	0.582	124	0.302	60.66
T0848TS310_3-D1.rsa	37.5	0.015	0.331	0.654	89	0.421	57.35
T0848TS038_3-D1.rsa	37.288	0.037	0.009	0.953	204	0.183	8.99
T0848TS439_4-D1.rsa	37.143	0.45	0.005	0.545	220	0.169	20.17
T0848TS340_2-D1.rsa	36.842	0.517	0.051	0.432	102	0.361	61.34
T0848TS049_4-D1.rsa	36.571	0.371	0	0.629	254	0.144	8.79
T0848TS333_2-D1.rsa	36.364	0.057	0.412	0.53	157	0.232	39.27
T0848TS097_5-D1.rsa	36.207	0	0.217	0.783	112	0.323	77.62
T0848TS448_5-D1.rsa	36.111	0.343	0.094	0.563	120	0.301	70.56
T0848TS144_3-D1.rsa	35.965	0.253	0.171	0.576	125	0.288	61.64
T0848TS442_3-D1.rsa	35.526	0.139	0.018	0.843	280	0.127	18.9
T0848TS322_3-D1.rsa	35.429	0.332	0.01	0.658	266	0.133	18.13
T0848TS169_2-D1.rsa	35.088	0.304	0.074	0.622	135	0.26	35.25
T0848TS430_1-D1.rsa	34.737	0.504	0.055	0.441	104	0.334	53.6
T0848TS216_5-D1.rsa	34.722	0.268	0.019	0.714	152	0.228	63.58
T0848TS479_5-D1.rsa	34.483	0	0.217	0.783	112	0.308	78.15
T0848TS145_1-D1.rsa	34.375	0.015	0.338	0.647	88	0.391	66.36
T0848TS439_2-D1.rsa	34.286	0.303	0.162	0.534	125	0.274	67.95
T0848TS160_2-D1.rsa	34.091	0.051	0.24	0.709	210	0.162	49.75
T0848TS038_2-D1.rsa	33.766	0.402	0.071	0.527	118	0.286	65.12
T0848TS064_3-D1.rsa	33.333	0.347	0.103	0.549	117	0.285	70.43
T0848TS357_1-D1.rsa	32.955	0.068	0.25	0.682	202	0.163	51.44
T0848TS042_5-D1.rsa	32.955	0	0.176	0.824	244	0.135	6.84
T0848TS492_4-D1.rsa	32.955	0.064	0.311	0.625	185	0.178	51.1
T0848TS210_4-D1.rsa	32.955	0.051	0.416	0.534	158	0.209	42.74
T0848TS153_4-D1.rsa	32.8	0.009	0.383	0.608	200	0.164	55.62
T0848TS420_1-D1.rsa	32.673	0.28	0.15	0.57	183	0.179	51.01
T0848TS184_2-D1.rsa	32.558	0.308	0.201	0.491	83	0.392	72.19
T0848TS483_4-D1.rsa	32.558	0.278	0.178	0.544	92	0.354	72.48
T0848TS437_1-D1.rsa	32.558	0.278	0.189	0.533	90	0.362	65.09
T0848TS310_2-D1.rsa	32.381	0.295	0.231	0.474	111	0.292	69.02
T0848TS080_2-D1.rsa	32.323	0.233	0.073	0.694	200	0.162	41.32
T0848TS216_3-D1.rsa	32.192	0.385	0.113	0.501	186	0.173	78.41
T0848TS042_4-D1.rsa	32	0.012	0.356	0.632	208	0.154	54.33
T0848TS442_2-D1.rsa	32	0.018	0.386	0.596	196	0.163	57.07
T0848TS097_4-D1.rsa	32	0.527	0	0.473	191	0.168	10.03
T0848TS162_4-D1.rsa	31.944	0.31	0.094	0.596	127	0.252	64.59
T0848TS204_1-D1.rsa	31.507	0.369	0.092	0.539	200	0.158	71.8
T0848TS328_5-D1.rsa	31.507	0.361	0.108	0.531	197	0.16	76.84
T0848TS064_5-D1.rsa	31.395	0.272	0.178	0.55	93	0.338	72.63

T0848TS410_5-D1.rsa	31.313	0.118	0.177	0.705	203	0.154	50.26
T0848TS454_5-D1.rsa	31.313	0.16	0.163	0.677	195	0.161	49.91
T0848TS156_3-D1.rsa	31.313	0.208	0.184	0.608	175	0.179	61.02
T0848TS144_4-D1.rsa	31.313	0.247	0.174	0.58	167	0.188	62.07
T0848TS326_2-D1.rsa	31.2	0.009	0.374	0.617	203	0.154	55.77
T0848TS162_2-D1.rsa	31.148	0.029	0.295	0.676	142	0.219	58.95
T0848TS132_5-D1.rsa	31.148	0.024	0.429	0.548	115	0.271	72.76
T0848TS410_1-D1.rsa	30.882	0.337	0.103	0.56	136	0.227	66.56
T0848TS296_5-D1.rsa	30.882	0.342	0.091	0.568	138	0.224	66
T0848TS157_5-D1.rsa	30.857	0.45	0.054	0.495	200	0.154	33.48
T0848TS346_1-D1.rsa	30.822	0.388	0.1	0.512	190	0.162	72.89
T0848TS128_5-D1.rsa	30.769	0.177	0.111	0.712	326	0.094	19.83
T0848TS064_4-D1.rsa	30.476	0.346	0.179	0.474	111	0.275	68.06
T0848TS067_1-D1.rsa	30.476	0.359	0.201	0.44	103	0.296	71.26
T0848TS157_3-D1.rsa	30.4	0.012	0.325	0.663	218	0.139	49.77
T0848TS155_2-D1.rsa	30.4	0	0.316	0.684	225	0.135	37.84
T0848TS235_5-D1.rsa	30.4	0	0.316	0.684	225	0.135	37.77
T0848TS300_1-D1.rsa	30.303	0.153	0.156	0.691	199	0.152	51.48
T0848TS132_1-D1.rsa	30.233	0.29	0.154	0.556	94	0.322	70.27
T0848TS080_5-D1.rsa	30.137	0.375	0.108	0.518	192	0.157	78.34
T0848TS237_5-D1.rsa	29.703	0.271	0.146	0.583	187	0.159	75.62
T0848TS335_2-D1.rsa	29.703	0.283	0.15	0.567	182	0.163	76.09
T0848TS162_3-D1.rsa	29.703	0.268	0.146	0.586	188	0.158	74.84
T0848TS486_1-D1.rsa	29.6	0.012	0.374	0.614	202	0.147	54.94
T0848TS212_1-D1.rsa	29.508	0.038	0.429	0.533	112	0.263	69.28
T0848TS410_4-D1.rsa	29.452	0.372	0.105	0.523	194	0.152	73.71
T0848TS044_2-D1.rsa	29.412	0.399	0.095	0.506	123	0.239	70.33
T0848TS251_4-D1.rsa	29.252	0.378	0.105	0.517	193	0.152	83.89
T0848TS335_3-D1.rsa	29.143	0.45	0.035	0.515	208	0.14	45.98
T0848TS333_1-D1.rsa	29	0.454	0.108	0.438	110	0.264	86.95
T0848TS156_5-D1.rsa	28.8	0.006	0.255	0.739	243	0.119	32.07
T0848TS300_5-D1.rsa	28.8	0.012	0.334	0.653	215	0.134	55.32
T0848TS414_5-D1.rsa	28.8	0.012	0.374	0.614	202	0.143	53.12
T0848TS110_1-D1.rsa	28.8	0.018	0.438	0.544	179	0.161	21.58
T0848TS228_1-D1.rsa	28.571	0.381	0.107	0.512	191	0.15	81.11
T0848TS144_1-D1.rsa	28.571	0.291	0.184	0.526	123	0.232	69.23
T0848TS492_3-D1.rsa	28.283	0.24	0.177	0.583	168	0.168	66.06
T0848TS204_2-D1.rsa	28.283	0.236	0.177	0.587	169	0.167	66.06
T0848TS322_1-D1.rsa	28.283	0.243	0.184	0.573	165	0.171	65.97
T0848TS340_3-D1.rsa	28.283	0.194	0.177	0.628	181	0.156	53.99
T0848TS347_3-D1.rsa	28.205	0.297	0.125	0.578	171	0.165	50.61
T0848TS080_3-D1.rsa	28.082	0.38	0.108	0.512	190	0.148	79.91
T0848TS216_4-D1.rsa	28.082	0.348	0.119	0.534	198	0.142	64.58
T0848TS006_1-D1.rsa	28.082	0.415	0.102	0.482	179	0.157	81.74
T0848TS041_1-D1.rsa	28.082	0.41	0.105	0.485	180	0.156	75.55
T0848TS499_3-D1.rsa	28.082	0.402	0.113	0.485	180	0.156	72.55
T0848TS155_1-D1.rsa	28	0.25	0.02	0.73	295	0.095	25.31

T0848TS439_3-D1.rsa	28	0.024	0.377	0.599	197	0.142	52.43
T0848TS454_1-D1.rsa	27.941	0.412	0.107	0.481	117	0.239	65.78
T0848TS228_3-D1.rsa	27.891	0.378	0.097	0.525	196	0.142	78.06
T0848TS008_3-D1.rsa	27.869	0.048	0.476	0.476	100	0.279	70.9
T0848TS210_2-D1.rsa	27.723	0.28	0.143	0.576	185	0.15	74.53
T0848TS296_2-D1.rsa	27.619	0.372	0.201	0.427	100	0.276	71.69
T0848TS425_5-D1.rsa	27.619	0.333	0.209	0.457	107	0.258	69.44
T0848TS080_1-D1.rsa	27.429	0.54	0.01	0.45	182	0.151	51.8
T0848TS492_1-D1.rsa	27.273	0.215	0.222	0.563	162	0.168	67.8
T0848TS210_3-D1.rsa	27.273	0.167	0.208	0.625	180	0.152	47.66
T0848TS483_1-D1.rsa	27.273	0.26	0.194	0.545	157	0.174	63.02
T0848TS290_5-D1.rsa	27.273	0.243	0.243	0.514	148	0.184	67.36
T0848TS391_1-D1.rsa	27.273	0.42	0.125	0.455	102	0.267	81.67
T0848TS044_5-D1.rsa	27.273	0.397	0.121	0.482	108	0.253	78.93
T0848TS452_2-D1.rsa	27.119	0	0.28	0.72	154	0.176	64.72
T0848TS268_1-D1.rsa	27.119	0.009	0.271	0.72	154	0.176	64.49
T0848TS347_4-D1.rsa	27.119	0.014	0.299	0.687	147	0.184	62.38
T0848TS358_1-D1.rsa	27	0.43	0.124	0.446	112	0.241	87.95
T0848TS032_2-D1.rsa	26.923	0.378	0.111	0.51	151	0.178	58.16
T0848TS290_3-D1.rsa	26.923	0.389	0.128	0.483	143	0.188	59.12
T0848TS160_3-D1.rsa	26.543	0.388	0.09	0.522	238	0.112	55.8
T0848TS349_4-D1.rsa	26.531	0.362	0.11	0.528	197	0.135	80.49
T0848TS080_4-D1.rsa	26.531	0.391	0.107	0.501	187	0.142	84.65
T0848TS116_5-D1.rsa	26.531	0.389	0.11	0.501	187	0.142	81.88
T0848TS049_3-D1.rsa	26.471	0.333	0.107	0.56	136	0.195	66.78
T0848TS335_1-D1.rsa	26.471	0.403	0.095	0.502	122	0.217	69.89
T0848TS499_2-D1.rsa	26.471	0.403	0.107	0.49	119	0.222	70.56
T0848TS110_2-D1.rsa	26.471	0.403	0.103	0.494	120	0.221	73.11
T0848TS216_2-D1.rsa	26.286	0.418	0.03	0.552	223	0.118	45.98
T0848TS345_4-D1.rsa	26.286	0.483	0.05	0.468	189	0.139	39.11
T0848TS335_4-D1.rsa	26.263	0.198	0.16	0.642	185	0.142	62.76
T0848TS452_4-D1.rsa	26.263	0.215	0.188	0.597	172	0.153	69.53
T0848TS073_2-D1.rsa	26.263	0.271	0.212	0.517	149	0.176	73
T0848TS008_4-D1.rsa	26.263	0.253	0.219	0.528	152	0.173	70.92
T0848TS362_3-D1.rsa	26.027	0.367	0.102	0.531	197	0.132	72.34
T0848TS237_2-D1.rsa	26.027	0.402	0.113	0.485	180	0.145	76.16
T0848TS067_4-D1.rsa	26	0.406	0.116	0.478	120	0.217	88.94
T0848TS044_4-D1.rsa	26	0.434	0.127	0.438	110	0.236	85.56
T0848TS457_5-D1.rsa	25.974	0.424	0.121	0.455	102	0.255	82.38
T0848TS169_4-D1.rsa	25.974	0.397	0.156	0.446	100	0.26	72.14
T0848TS006_2-D1.rsa	25.85	0.378	0.102	0.52	194	0.133	84.72
T0848TS349_5-D1.rsa	25.85	0.375	0.107	0.517	193	0.134	82.78
T0848TS492_5-D1.rsa	25.714	0.438	0	0.562	227	0.113	31.81
T0848TS420_4-D1.rsa	25.641	0.318	0.091	0.591	175	0.147	53.3
T0848TS153_2-D1.rsa	25.6	0.012	0.31	0.678	223	0.115	51.22
T0848TS338_5-D1.rsa	25.424	0.019	0.262	0.72	154	0.165	58.29
T0848TS338_1-D1.rsa	25	0.41	0.092	0.498	125	0.2	84.96

T0848TS499_5-D1.rsa	25	0.305	0.041	0.654	159	0.157	56.33
T0848TS347_1-D1.rsa	25	0.354	0.099	0.547	133	0.188	68.11
T0848TS144_5-D1.rsa	25	0.35	0.091	0.56	136	0.184	66.11
T0848TS133_3-D1.rsa	25	0.446	0.12	0.434	109	0.229	83.96
T0848TS251_3-D1.rsa	25	0.374	0.103	0.523	127	0.197	66.11
T0848TS457_3-D1.rsa	25	0.412	0.103	0.486	118	0.212	70.67
T0848TS425_4-D1.rsa	25	0.395	0.115	0.49	119	0.21	66.67
T0848TS442_4-D1.rsa	24.752	0.212	0.109	0.679	218	0.114	70.72
T0848TS044_1-D1.rsa	24.675	0.411	0.08	0.509	114	0.216	61.31
T0848TS381_3-D1.rsa	24.658	0.402	0.108	0.491	182	0.135	80.11
T0848TS235_2-D1.rsa	24.59	0.029	0	0.971	204	0.121	37.19
T0848TS391_2-D1.rsa	24.59	0.024	0.281	0.695	146	0.168	48.76
T0848TS414_4-D1.rsa	24.419	0.378	0.113	0.509	144	0.17	74.56
T0848TS073_1-D1.rsa	24.359	0.378	0.111	0.51	151	0.161	52.78
T0848TS041_3-D1.rsa	24.342	0.307	0.004	0.689	314	0.078	45.72
T0848TS237_4-D1.rsa	24.242	0.233	0.212	0.556	160	0.152	69.01
T0848TS210_5-D1.rsa	24.242	0.267	0.219	0.514	148	0.164	72.14
T0848TS345_2-D1.rsa	24.242	0.264	0.219	0.517	149	0.163	71.01
T0848TS251_5-D1.rsa	24.074	0.406	0.064	0.531	242	0.099	68.27
T0848TS026_1-D1.rsa	24.074	0.434	0.094	0.471	215	0.112	76.38
T0848TS452_3-D1.rsa	24.074	0.386	0.09	0.524	239	0.101	52.98
T0848TS063_3-D1.rsa	24	0.262	0.015	0.723	292	0.082	25.37
T0848TS300_2-D1.rsa	24	0.006	0.277	0.717	236	0.102	36.25
T0848TS290_2-D1.rsa	24	0.402	0.12	0.478	120	0.2	90.24
T0848TS038_1-D1.rsa	24	0.418	0.124	0.458	115	0.209	90.94
T0848TS290_1-D1.rsa	24	0.434	0.108	0.458	115	0.209	88.84
T0848TS340_1-D1.rsa	24	0.446	0.112	0.442	111	0.216	79.28
T0848TS430_2-D1.rsa	24	0.072	0.174	0.754	104	0.231	59.96
T0848TS116_2-D1.rsa	23.81	0.362	0.107	0.531	198	0.12	79.65
T0848TS133_5-D1.rsa	23.81	0.248	0.188	0.564	132	0.18	62.71
T0848TS328_1-D1.rsa	23.762	0.268	0.15	0.583	187	0.127	74.61
T0848TS063_1-D1.rsa	23.684	0.241	0.066	0.693	230	0.103	50.9
T0848TS454_3-D1.rsa	23.529	0.35	0.103	0.547	133	0.177	69.78
T0848TS338_4-D1.rsa	23.529	0.412	0.103	0.486	118	0.199	71.78
T0848TS251_2-D1.rsa	23.457	0.423	0.101	0.476	217	0.108	72.9
T0848TS110_5-D1.rsa	23.429	0.428	0.03	0.542	219	0.107	47.15
T0848TS044_3-D1.rsa	23.404	0.323	0.136	0.541	139	0.168	74.71
T0848TS049_5-D1.rsa	23.377	0.375	0.125	0.5	112	0.209	77.5
T0848TS345_5-D1.rsa	23.256	0.396	0.117	0.488	138	0.169	79.06
T0848TS420_3-D1.rsa	23.256	0.396	0.124	0.481	136	0.171	76.15
T0848TS204_5-D1.rsa	23.232	0.243	0.222	0.535	154	0.151	70.92
T0848TS204_4-D1.rsa	23.2	0.012	0.395	0.593	195	0.119	20.67
T0848TS358_3-D1.rsa	22.951	0.033	0.457	0.51	107	0.214	72.26
T0848TS153_1-D1.rsa	22.857	0.468	0.012	0.52	210	0.109	39.48
T0848TS328_4-D1.rsa	22.857	0.47	0.01	0.52	210	0.109	39.91
T0848TS457_4-D1.rsa	22.807	0.447	0.082	0.47	103	0.221	78.88
T0848TS362_2-D1.rsa	22.603	0.394	0.113	0.493	183	0.124	56.61

T0848TS268_2-D1.rsa	22.286	0.517	0.01	0.473	191	0.117	45.05
T0848TS063_5-D1.rsa	22.222	0.417	0.09	0.493	225	0.099	76.71
T0848TS042_1-D1.rsa	22.222	0.445	0.083	0.471	215	0.103	65.18
T0848TS420_2-D1.rsa	22.093	0.385	0.095	0.519	147	0.15	73.32
T0848TS381_5-D1.rsa	21.918	0.437	0.092	0.472	175	0.125	62.88
T0848TS157_1-D1.rsa	21.795	0.382	0.118	0.5	148	0.147	56.94
T0848TS282_1-D1.rsa	21.714	0.54	0.005	0.455	184	0.118	44.86
T0848TS237_3-D1.rsa	21.212	0.257	0.194	0.549	158	0.134	70.05
T0848TS235_3-D1.rsa	21.053	0.395	0	0.605	276	0.076	61.62
T0848TS169_5-D1.rsa	21	0.418	0.124	0.458	115	0.183	87.85
T0848TS326_3-D1.rsa	20.988	0.425	0.094	0.48	219	0.096	60.6
T0848TS445_3-D1.rsa	20.792	0.259	0.087	0.654	210	0.099	63.47
T0848TS235_1-D1.rsa	20.792	0.243	0.146	0.611	196	0.106	73.36
T0848TS064_2-D1.rsa	20.779	0.411	0.121	0.469	105	0.198	71.79
T0848TS155_4-D1.rsa	20.769	0.242	0.124	0.633	290	0.072	68.56
T0848TS436_5-D1.rsa	20.339	0	0.322	0.678	145	0.14	63.9
T0848TS448_1-D1.rsa	20	0.453	0.027	0.52	210	0.095	40.53
T0848TS042_3-D1.rsa	19.802	0.206	0.09	0.704	226	0.088	55.69
T0848TS445_1-D1.rsa	19.767	0.392	0.113	0.495	140	0.141	74.73
T0848TS153_5-D1.rsa	19.767	0.406	0.113	0.481	136	0.145	79.68
T0848TS023_4-D1.rsa	19.737	0.41	0.022	0.568	259	0.076	50.44
T0848TS132_4-D1.rsa	19.298	0.338	0.059	0.603	132	0.146	64.27
T0848TS049_2-D1.rsa	19.298	0.42	0.046	0.534	117	0.165	75.34
T0848TS358_4-D1.rsa	19.231	0.399	0.091	0.51	151	0.127	56.25
T0848TS349_2-D1.rsa	19.136	0.436	0.094	0.469	214	0.089	71.36
T0848TS023_3-D1.rsa	19.079	0.45	0.033	0.518	236	0.081	61.73
T0848TS499_4-D1.rsa	18.644	0.056	0.22	0.724	155	0.12	54.32
T0848TS442_5-D1.rsa	18.644	0.023	0.318	0.659	141	0.132	62.97
T0848TS023_2-D1.rsa	18.519	0.412	0.092	0.496	226	0.082	67.99
T0848TS228_4-D1.rsa	18.421	0.404	0.024	0.572	261	0.071	61.95
T0848TS042_2-D1.rsa	18.421	0.474	0.024	0.502	229	0.08	64.47
T0848TS155_5-D1.rsa	17.901	0.428	0.103	0.469	214	0.084	78.2
T0848TS338_3-D1.rsa	17.808	0.396	0.108	0.496	184	0.097	55.79
T0848TS300_3-D1.rsa	17.763	0.463	0.024	0.513	234	0.076	64.42
T0848TS483_5-D1.rsa	17.544	0.438	0.082	0.479	105	0.167	82.42
T0848TS162_5-D1.rsa	17.442	0.392	0.11	0.498	141	0.124	79.42
T0848TS499_1-D1.rsa	17.442	0.392	0.11	0.498	141	0.124	74.38
T0848TS414_3-D1.rsa	17.172	0.177	0.073	0.75	216	0.079	10.24
T0848TS132_3-D1.rsa	16.949	0.023	0.215	0.762	163	0.104	63.32
T0848TS011_2-D1.rsa	16.923	0.286	0.179	0.535	245	0.069	70.5
T0848TS322_5-D1.rsa	16.923	0.26	0.225	0.515	236	0.072	77.78
T0848TS439_1-D1.rsa	16.923	0.266	0.223	0.511	234	0.072	73.39
T0848TS436_1-D1.rsa	16.923	0.262	0.236	0.502	230	0.074	74.11
T0848TS442_1-D1.rsa	16.923	0.273	0.216	0.511	234	0.072	66.22
T0848TS362_1-D1.rsa	16.279	0.35	0.106	0.544	154	0.106	78.89
T0848TS335_5-D1.rsa	16.279	0.378	0.12	0.502	142	0.115	81.01
T0848TS333_3-D1.rsa	16.154	0.284	0.159	0.557	255	0.063	74.72

T0848TS041_2-D1.rsa	16.154	0.266	0.221	0.513	235	0.069	73.72
T0848TS228_2-D1.rsa	15.789	0.412	0.024	0.564	257	0.061	60.36
T0848TS345_3-D1.rsa	15.789	0.343	0.117	0.539	179	0.088	99.25
T0848TS425_2-D1.rsa	15.789	0.457	0.091	0.452	99	0.159	79.91
T0848TS041_5-D1.rsa	15.385	0.255	0.225	0.52	238	0.065	77.06
T0848TS160_5-D1.rsa	15.385	0.282	0.234	0.485	222	0.069	71.11
T0848TS279_1-D1.rsa	15.116	0.392	0.117	0.491	139	0.109	81.36
T0848TS425_3-D1.rsa	14.894	0.335	0.183	0.482	124	0.12	81.32
T0848TS445_5-D1.rsa	14.894	0.311	0.195	0.494	127	0.117	79.67
T0848TS436_3-D1.rsa	14.615	0.26	0.223	0.517	237	0.062	75.17
T0848TS362_5-D1.rsa	14.474	0.286	0.114	0.599	199	0.073	97.29
T0848TS116_1-D1.rsa	14.474	0.271	0.108	0.62	206	0.07	67.32
T0848TS157_2-D1.rsa	14.474	0.328	0.157	0.515	171	0.085	99.17
T0848TS145_3-D1.rsa	14.474	0.337	0.123	0.539	179	0.081	99.02
T0848TS448_4-D1.rsa	14.474	0.343	0.13	0.527	175	0.083	98.04
T0848TS006_4-D1.rsa	13.953	0.396	0.113	0.491	139	0.1	77.47
T0848TS116_3-D1.rsa	13.158	0.298	0.087	0.614	204	0.064	96.46
T0848TS328_2-D1.rsa	13.158	0.301	0.139	0.56	186	0.071	94.95
T0848TS414_1-D1.rsa	13.158	0.343	0.139	0.518	172	0.076	81.02
T0848TS479_4-D1.rsa	12.766	0.331	0.195	0.475	122	0.105	79.67
T0848TS038_4-D1.rsa	12.766	0.331	0.183	0.486	125	0.102	82.3
T0848TS362_4-D1.rsa	11.842	0.328	0.123	0.548	182	0.065	99.17
T0848TS110_3-D1.rsa	10.638	0.304	0.066	0.63	162	0.066	83.95
T0848TS410_2-D1.rsa	10.638	0.284	0.152	0.564	145	0.073	75.97
T0848TS310_5-D1.rsa	10.638	0.339	0.183	0.479	123	0.086	81.61
T0848TS268_3-D1.rsa	10.638	0.311	0.183	0.506	130	0.082	85.21
T0822TS317_1-D1.rsa	80	0	0.13	0.87	120	0.667	11.41
T0822TS445_3-D1.rsa	78.947	0.018	0	0.982	109	0.724	14.64
T0822TS067_1-D1.rsa	75	0	0.237	0.763	100	0.75	19.47
T0822TS132_2-D1.rsa	75	0	0.472	0.528	57	1.316	59.95
T0822TS457_5-D1.rsa	72.5	0	0.175	0.825	94	0.771	31.36
T0822TS034_1-D1.rsa	72	0.058	0.239	0.703	97	0.742	18.66
T0822TS336_3-D1.rsa	72	0.014	0.217	0.768	106	0.679	16.3
T0822TS008_4-D1.rsa	69.231	0.279	0.061	0.661	109	0.635	25.16
T0822TS282_3-D1.rsa	67.857	0.015	0.099	0.885	116	0.585	12.79
T0822TS362_1-D1.rsa	67.647	0.556	0	0.444	56	1.208	34.92
T0822TS338_1-D1.rsa	66.667	0	0.583	0.417	45	1.481	77.55
T0822TS499_2-D1.rsa	63.462	0.273	0.085	0.642	106	0.599	56.01
T0822TS054_3-D1.rsa	63.462	0.273	0.158	0.57	94	0.675	65.98
T0822TS011_2-D1.rsa	63.158	0.27	0	0.73	81	0.78	16.67
T0822TS445_5-D1.rsa	62.791	0.157	0.015	0.828	111	0.566	18.47
T0822TS216_4-D1.rsa	62.791	0.239	0.045	0.716	96	0.654	38.99
T0822TS417_5-D1.rsa	61.224	0.263	0.011	0.727	271	0.226	7.01
T0822TS358_2-D1.rsa	59.615	0.273	0.097	0.63	104	0.573	71.04
T0822TS492_3-D1.rsa	59.615	0.267	0.097	0.636	105	0.568	66.77
T0822TS465_5-D1.rsa	58.333	0.046	0.63	0.324	35	1.667	72.92
T0822TS050_1-D1.rsa	58.14	0.328	0.03	0.642	86	0.676	54.1

T0822TS041_4-D1.rsa	57.813	0.059	0	0.941	128	0.452	13.6
T0822TS064_4-D1.rsa	57.692	0.261	0.097	0.642	106	0.544	72.94
T0822TS173_3-D1.rsa	57.692	0.261	0.133	0.606	100	0.577	76.27
T0822TS173_2-D1.rsa	57.692	0.273	0.115	0.612	101	0.571	76.58
T0822TS317_3-D1.rsa	57.692	0.242	0.115	0.642	106	0.544	60.92
T0822TS349_2-D1.rsa	57.576	0.292	0.066	0.642	185	0.311	10.59
T0822TS479_5-D1.rsa	55.814	0.231	0.06	0.709	95	0.588	37.31
T0822TS430_2-D1.rsa	55.814	0	0.187	0.813	109	0.512	13.99
T0822TS156_1-D1.rsa	55.814	0.313	0.075	0.612	82	0.681	36.94
T0822TS155_5-D1.rsa	55.263	0.057	0	0.943	313	0.177	6.93
T0822TS347_3-D1.rsa	55.263	0.234	0.054	0.712	79	0.7	34.46
T0822TS155_1-D1.rsa	55.102	0.177	0	0.823	307	0.179	7.15
T0822TS401_2-D1.rsa	54.412	0.354	0.016	0.63	153	0.356	12.11
T0822TS414_5-D1.rsa	54.237	0.033	0.009	0.958	205	0.265	9.11
T0822TS044_5-D1.rsa	53.846	0.261	0.073	0.667	110	0.49	67.56
T0822TS340_1-D1.rsa	53.521	0.274	0.083	0.643	108	0.496	25.16
T0822TS097_4-D1.rsa	53.488	0.299	0.127	0.575	77	0.695	41.98
T0822TS014_3-D1.rsa	52.632	0.31	0	0.69	229	0.23	11.75
T0822TS403_3-D1.rsa	52.632	0.189	0.126	0.685	76	0.693	37.84
T0822TS479_2-D1.rsa	52.113	0.113	0.042	0.845	142	0.367	14.42
T0822TS417_4-D1.rsa	50	0.057	0.172	0.77	228	0.219	19.17
T0822TS425_2-D1.rsa	50	0.012	0.133	0.855	141	0.355	10.13
T0822TS235_3-D1.rsa	50	0	0.046	0.954	125	0.4	13.93
T0822TS336_1-D1.rsa	50	0	0.445	0.555	61	0.82	49.55
T0822TS160_4-D1.rsa	48.077	0.255	0	0.745	123	0.391	16.77
T0822TS465_2-D1.rsa	47.5	0	0.298	0.702	80	0.594	17.98
T0822TS160_5-D1.rsa	46.591	0	0.314	0.686	203	0.23	28.12
T0822TS326_1-D1.rsa	46.591	0.047	0.355	0.598	177	0.263	42.15
T0822TS064_2-D1.rsa	46.465	0.264	0.139	0.597	172	0.27	11.98
T0822TS425_1-D1.rsa	46.429	0	0.183	0.817	107	0.434	17.18
T0822TS300_1-D1.rsa	45.89	0.148	0.005	0.846	314	0.146	6.68
T0822TS162_5-D1.rsa	45.455	0.198	0.16	0.642	185	0.246	44.44
T0822TS401_3-D1.rsa	44.828	0	0.077	0.923	132	0.34	52.97
T0822TS493_2-D1.rsa	44.737	0.108	0.006	0.886	294	0.152	15.81
T0822TS362_4-D1.rsa	44.737	0.252	0.072	0.676	75	0.596	34.69
T0822TS457_1-D1.rsa	44.643	0.349	0.032	0.619	78	0.572	26.59
T0822TS391_5-D1.rsa	44.444	0.376	0.075	0.549	117	0.38	65.23
T0822TS322_3-D1.rsa	44	0.394	0.015	0.592	239	0.184	32.49
T0822TS155_4-D1.rsa	43.421	0.163	0.06	0.777	258	0.168	30.95
T0822TS345_5-D1.rsa	43.103	0	0.035	0.965	138	0.312	57.17
T0822TS420_2-D1.rsa	43.103	0	0.077	0.923	132	0.327	56.47
T0822TS452_1-D1.rsa	43.103	0.014	0.133	0.853	122	0.353	57.87
T0822TS296_2-D1.rsa	42.857	0	0.321	0.679	89	0.482	58.02
T0822TS216_1-D1.rsa	42.5	0	0.035	0.965	110	0.386	17.76
T0822TS425_3-D1.rsa	42.466	0.714	0	0.286	73	0.582	34.71
T0822TS014_1-D1.rsa	42.254	0.304	0.149	0.548	92	0.459	16.35
T0822TS157_2-D1.rsa	42.105	0.163	0.033	0.804	267	0.158	22.29

T0822TS011_1-D1.rsa	42.105	0.207	0.027	0.766	85	0.495	23.2
T0822TS492_5-D1.rsa	42.045	0.054	0.307	0.639	189	0.222	49.92
T0822TS184_5-D1.rsa	42.045	0.007	0.345	0.649	192	0.219	28.8
T0822TS216_3-D1.rsa	41.379	0	0.217	0.783	112	0.369	78.32
T0822TS132_1-D1.rsa	41.379	0.602	0	0.398	102	0.406	55.18
T0822TS041_3-D1.rsa	40.845	0	0.321	0.679	114	0.358	11.7
T0822TS310_1-D1.rsa	40.351	0.217	0.147	0.636	138	0.292	33.41
T0822TS160_3-D1.rsa	40.278	0.366	0.085	0.549	117	0.344	67
T0822TS483_2-D1.rsa	40	0.07	0.07	0.86	98	0.408	40.35
T0822TS457_4-D1.rsa	39.773	0.074	0.422	0.503	149	0.267	33.53
T0822TS436_3-D1.rsa	39.655	0	0.126	0.874	125	0.317	59.44
T0822TS063_5-D1.rsa	39.474	0.157	0.024	0.819	272	0.145	18.9
T0822TS358_4-D1.rsa	39.474	0.3	0.171	0.53	115	0.343	60.25
T0822TS145_3-D1.rsa	39.437	0.405	0.155	0.44	74	0.533	41.35
T0822TS457_3-D1.rsa	39.286	0	0.115	0.885	116	0.339	16.79
T0822TS064_3-D1.rsa	39.063	0.022	0.051	0.926	126	0.31	55.7
T0822TS279_1-D1.rsa	38.947	0.551	0.042	0.407	96	0.406	58.48
T0822TS145_1-D1.rsa	38.636	0.078	0.297	0.625	185	0.209	36.32
T0822TS065_1-D1.rsa	38.636	0.057	0.456	0.486	144	0.268	46.2
T0822TS290_5-D1.rsa	38.596	0.249	0.161	0.59	128	0.302	61.75
T0822TS054_2-D1.rsa	38.596	0.244	0.226	0.53	115	0.336	63.25
T0822TS328_3-D1.rsa	38.596	0.217	0.147	0.636	138	0.28	33.41
T0822TS465_1-D1.rsa	38.356	0.773	0	0.227	58	0.661	60.98
T0822TS338_5-D1.rsa	38.356	0.741	0	0.259	66	0.581	36.47
T0822TS410_2-D1.rsa	38.286	0.453	0.015	0.532	215	0.178	11.63
T0822TS322_1-D1.rsa	37.931	0	0.133	0.867	124	0.306	59.09
T0822TS145_5-D1.rsa	37.719	0.184	0.12	0.696	151	0.25	10.95
T0822TS362_5-D1.rsa	37.5	0	0.123	0.877	100	0.375	43.42
T0822TS080_5-D1.rsa	37.374	0.198	0.149	0.653	188	0.199	61.72
T0822TS153_3-D1.rsa	36.842	0.295	0.161	0.544	118	0.312	61.06
T0822TS173_1-D1.rsa	36.842	0.281	0.161	0.558	121	0.304	61.52
T0822TS328_4-D1.rsa	36.364	0.088	0.459	0.453	134	0.271	49.41
T0822TS228_4-D1.rsa	36.364	0.061	0.49	0.449	133	0.273	45.78
T0822TS290_4-D1.rsa	36.364	0.108	0.5	0.392	116	0.313	52.03
T0822TS116_2-D1.rsa	36.111	0.319	0.094	0.587	125	0.289	72.08
T0822TS169_3-D1.rsa	35.965	0.258	0.175	0.567	123	0.292	64.98
T0822TS080_1-D1.rsa	35.965	0.258	0.171	0.571	124	0.29	62.21
T0822TS145_4-D1.rsa	35.616	0.737	0	0.263	67	0.532	58.53
T0822TS425_5-D1.rsa	35.429	0.47	0	0.53	214	0.166	32.73
T0822TS403_1-D1.rsa	35.345	0.672	0	0.328	84	0.421	57.72
T0822TS454_4-D1.rsa	35.211	0.482	0.071	0.446	75	0.469	53.53
T0822TS011_4-D1.rsa	35.211	0.524	0.036	0.44	74	0.476	48.4
T0822TS338_4-D1.rsa	35.211	0.28	0.137	0.583	98	0.359	19.23
T0822TS210_2-D1.rsa	35.2	0.012	0.155	0.833	274	0.128	43.77
T0822TS133_5-D1.rsa	35.2	0.021	0.343	0.635	209	0.168	49.54
T0822TS345_3-D1.rsa	35.2	0.006	0.356	0.638	210	0.168	38.37
T0822TS212_1-D1.rsa	35.088	0.311	0.073	0.616	135	0.26	57.08

T0822TS347_1-D1.rsa	35.088	0.253	0.203	0.544	118	0.297	64.63
T0822TS340_3-D1.rsa	35	0.018	0.123	0.86	98	0.357	17.76
T0822TS479_1-D1.rsa	34.722	0.38	0.094	0.526	112	0.31	70.31
T0822TS483_3-D1.rsa	34.722	0.413	0.103	0.484	103	0.337	77.92
T0822TS358_3-D1.rsa	34.722	0.451	0.038	0.512	109	0.319	66.75
T0822TS258_2-D1.rsa	34.653	0.321	0.153	0.526	169	0.205	74.22
T0822TS403_4-D1.rsa	34.483	0.68	0	0.32	82	0.421	67.48
T0822TS034_5-D1.rsa	34.343	0.233	0.191	0.576	166	0.207	39.32
T0822TS403_2-D1.rsa	34.211	0.241	0.5	0.259	28	1.222	94.21
T0822TS335_1-D1.rsa	34.091	0.044	0.304	0.652	193	0.177	41.05
T0822TS428_3-D1.rsa	33.824	0.35	0.091	0.56	136	0.249	65.11
T0822TS290_2-D1.rsa	33.824	0.35	0.107	0.543	132	0.256	62.67
T0822TS340_4-D1.rsa	33.803	0.357	0.107	0.536	90	0.376	37.98
T0822TS335_3-D1.rsa	33.333	0.115	0.163	0.722	208	0.16	50.87
T0822TS442_4-D1.rsa	33.333	0.219	0.188	0.594	171	0.195	54.95
T0822TS118_1-D1.rsa	32.558	0.366	0.03	0.604	81	0.402	53.55
T0822TS064_1-D1.rsa	32.353	0.37	0.095	0.535	130	0.249	60.67
T0822TS067_4-D1.rsa	32.353	0.436	0.099	0.465	113	0.286	64.33
T0822TS345_4-D1.rsa	32.323	0.115	0.156	0.729	210	0.154	49.91
T0822TS433_4-D1.rsa	32.323	0.118	0.174	0.708	204	0.158	49.91
T0822TS290_1-D1.rsa	32.192	0.394	0.102	0.504	187	0.172	73.84
T0822TS360_3-D1.rsa	32.143	0.183	0.238	0.579	73	0.44	57.34
T0822TS260_2-D1.rsa	32	0.015	0.337	0.647	213	0.15	52.13
T0822TS357_1-D1.rsa	32	0.403	0.032	0.564	228	0.14	24.69
T0822TS296_5-D1.rsa	32	0	0.094	0.906	125	0.256	27.36
T0822TS171_4-D1.rsa	31.683	0.246	0.15	0.604	194	0.163	75.08
T0822TS132_5-D1.rsa	31.683	0.249	0.153	0.598	192	0.165	73.52
T0822TS360_2-D1.rsa	31.579	0.25	0.509	0.241	26	1.215	94.68
T0822TS080_4-D1.rsa	31.507	0.383	0.111	0.507	188	0.168	74.11
T0822TS049_3-D1.rsa	31.313	0.198	0.17	0.632	182	0.172	45.92
T0822TS120_4-D1.rsa	31.2	0.012	0.356	0.632	208	0.15	54.71
T0822TS228_2-D1.rsa	31.169	0.393	0.121	0.487	109	0.286	79.05
T0822TS162_1-D1.rsa	31.169	0.415	0.121	0.464	104	0.3	81.31
T0822TS430_1-D1.rsa	31.034	0	0.217	0.783	112	0.277	77.8
T0822TS428_5-D1.rsa	31.034	0.723	0	0.277	71	0.437	78.52
T0822TS014_4-D1.rsa	30.822	0.407	0.108	0.485	180	0.171	79.29
T0822TS258_3-D1.rsa	30.508	0.009	0.243	0.748	160	0.191	59.11
T0822TS336_2-D1.rsa	30.508	0.037	0.299	0.664	142	0.215	52.1
T0822TS118_2-D1.rsa	30.476	0.248	0.158	0.594	139	0.219	69.44
T0822TS296_4-D1.rsa	30.476	0.406	0.15	0.444	104	0.293	69.66
T0822TS263_5-D1.rsa	30.4	0.006	0.301	0.693	228	0.133	46.88
T0822TS268_4-D1.rsa	30.4	0.012	0.313	0.675	222	0.137	54.71
T0822TS282_4-D1.rsa	30.357	0.175	0.349	0.476	60	0.506	51.39
T0822TS433_2-D1.rsa	30.303	0.219	0.094	0.688	198	0.153	45.92
T0822TS428_1-D1.rsa	30.172	0.641	0	0.359	92	0.328	30.18
T0822TS237_2-D1.rsa	30.137	0.372	0.108	0.52	193	0.156	80.38
T0822TS133_4-D1.rsa	30.137	0.399	0.105	0.496	184	0.164	81.61

T0822TS064_5-D1.rsa	30.137	0.388	0.108	0.504	187	0.161	59.95
T0822TS335_4-D1.rsa	29.703	0.259	0.118	0.623	200	0.149	75.78
T0822TS466_1-D1.rsa	29.6	0	0.182	0.818	269	0.11	43.24
T0822TS404_5-D1.rsa	29.6	0.033	0.246	0.72	237	0.125	38.07
T0822TS381_1-D1.rsa	29.452	0.372	0.108	0.52	193	0.153	78.88
T0822TS014_2-D1.rsa	29.452	0.396	0.108	0.496	184	0.16	78.88
T0822TS067_2-D1.rsa	29.412	0.305	0.07	0.626	152	0.193	58.22
T0822TS340_5-D1.rsa	29.412	0.354	0.041	0.605	147	0.2	59.67
T0822TS032_3-D1.rsa	29.412	0.35	0.091	0.56	136	0.216	66
T0822TS008_2-D1.rsa	29.412	0.362	0.099	0.539	131	0.225	68.11
T0822TS414_4-D1.rsa	29.293	0.25	0.174	0.576	166	0.176	69.7
T0822TS401_4-D1.rsa	29.293	0.184	0.201	0.615	177	0.165	52.52
T0822TS448_3-D1.rsa	29.293	0.233	0.191	0.576	166	0.176	63.98
T0822TS493_5-D1.rsa	29.252	0.29	0.054	0.657	245	0.119	42.29
T0822TS260_1-D1.rsa	29.167	0.366	0.094	0.54	115	0.254	53.3
T0822TS156_2-D1.rsa	28.846	0.273	0.097	0.63	104	0.277	18.51
T0822TS439_4-D1.rsa	28.814	0.009	0.276	0.715	153	0.188	62.73
T0822TS049_5-D1.rsa	28.814	0.019	0.28	0.701	150	0.192	60.63
T0822TS492_1-D1.rsa	28.767	0.399	0.108	0.493	183	0.157	81.47
T0822TS097_2-D1.rsa	28.767	0.402	0.113	0.485	180	0.16	76.91
T0822TS347_5-D1.rsa	28.767	0.765	0	0.235	60	0.479	67.84
T0822TS347_4-D1.rsa	28.767	0.749	0	0.251	64	0.449	55.59
T0822TS442_2-D1.rsa	28.571	0.218	0.017	0.765	309	0.092	20.98
T0822TS026_1-D1.rsa	28.571	0.428	0.012	0.559	226	0.126	46.78
T0822TS173_4-D1.rsa	28.571	0.562	0	0.438	177	0.161	38.92
T0822TS483_4-D1.rsa	28.571	0.397	0.116	0.487	109	0.262	82.38
T0822TS362_2-D1.rsa	28.571	0.151	0.222	0.627	79	0.362	43.65
T0822TS391_3-D1.rsa	28.283	0.212	0.128	0.66	190	0.149	64.93
T0822TS448_5-D1.rsa	28.283	0.247	0.163	0.59	170	0.166	46.7
T0822TS465_4-D1.rsa	28.169	0.464	0.077	0.458	77	0.366	49.36
T0822TS317_4-D1.rsa	28.169	0.464	0.161	0.375	63	0.447	62.66
T0822TS336_5-D1.rsa	28.125	0.081	0.029	0.89	121	0.232	15.44
T0822TS049_2-D1.rsa	28	0.015	0.301	0.684	225	0.124	51.29
T0822TS401_1-D1.rsa	28	0.006	0.271	0.723	238	0.118	31.46
T0822TS110_5-D1.rsa	28	0.423	0.027	0.55	222	0.126	46.23
T0822TS452_4-D1.rsa	28	0.012	0.386	0.602	198	0.141	55.09
T0822TS210_5-D1.rsa	27.941	0.354	0.107	0.539	131	0.213	66.33
T0822TS216_5-D1.rsa	27.941	0.379	0.086	0.535	130	0.215	66.78
T0822TS235_5-D1.rsa	27.891	0.37	0.08	0.55	205	0.136	82.92
T0822TS216_2-D1.rsa	27.869	0.038	0.443	0.519	109	0.256	67.29
T0822TS410_4-D1.rsa	27.869	0.038	0.405	0.557	117	0.238	65.8
T0822TS360_4-D1.rsa	27.619	0.346	0.167	0.487	114	0.242	60.68
T0822TS333_5-D1.rsa	27.429	0.443	0.017	0.54	218	0.126	46.41
T0822TS049_4-D1.rsa	27.429	0.443	0.015	0.542	219	0.125	39.54
T0822TS034_2-D1.rsa	27.429	0.574	0.022	0.403	163	0.168	46.78
T0822TS448_4-D1.rsa	27.273	0.212	0.167	0.622	179	0.152	62.67
T0822TS260_3-D1.rsa	27.273	0.208	0.212	0.58	167	0.163	59.9

T0822TS448_2-D1.rsa	27.273	0.24	0.194	0.566	163	0.167	65.8
T0822TS034_3-D1.rsa	27.273	0.278	0.219	0.503	145	0.188	72.48
T0822TS228_5-D1.rsa	27.273	0.411	0.121	0.469	105	0.26	81.79
T0822TS493_3-D1.rsa	27.211	0.354	0.097	0.55	205	0.133	82.99
T0822TS493_1-D1.rsa	27.211	0.381	0.102	0.517	193	0.141	82.85
T0822TS197_1-D1.rsa	27.211	0.381	0.131	0.488	182	0.15	85.69
T0822TS404_4-D1.rsa	27.2	0	0.128	0.872	287	0.095	40.43
T0822TS237_1-D1.rsa	27.2	0.015	0.264	0.72	237	0.115	51.44
T0822TS495_1-D1.rsa	27.119	0.028	0.313	0.659	141	0.192	63.67
T0822TS454_2-D1.rsa	27	0.422	0.124	0.454	114	0.237	89.64
T0822TS044_2-D1.rsa	26.857	0.47	0.035	0.495	200	0.134	41.52
T0822TS116_5-D1.rsa	26.761	0.399	0	0.601	101	0.265	51.12
T0822TS336_4-D1.rsa	26.744	0.254	0.101	0.645	109	0.245	71.3
T0822TS153_4-D1.rsa	26.733	0.274	0.106	0.62	199	0.134	74.06
T0822TS492_2-D1.rsa	26.712	0.391	0.102	0.507	188	0.142	78.54
T0822TS228_3-D1.rsa	26.667	0.325	0.141	0.534	125	0.213	64.42
T0822TS328_2-D1.rsa	26.667	0.389	0.222	0.389	91	0.293	72.22
T0822TS263_2-D1.rsa	26.531	0.349	0.099	0.552	206	0.129	81.32
T0822TS263_1-D1.rsa	26.531	0.375	0.102	0.523	195	0.136	85.21
T0822TS263_3-D1.rsa	26.531	0.383	0.102	0.515	192	0.138	86.04
T0822TS492_4-D1.rsa	26.531	0.378	0.113	0.509	190	0.14	80.9
T0822TS420_1-D1.rsa	26.531	0.359	0.137	0.504	188	0.141	81.81
T0822TS014_5-D1.rsa	26.531	0.381	0.118	0.501	187	0.142	78.26
T0822TS008_1-D1.rsa	26.471	0.342	0.086	0.572	139	0.19	58.89
T0822TS428_4-D1.rsa	26.471	0.37	0.086	0.543	132	0.201	65.89
T0822TS097_3-D1.rsa	26.471	0.407	0.107	0.486	118	0.224	57.33
T0822TS056_1-D1.rsa	26.4	0.027	0.243	0.729	240	0.11	44.3
T0822TS410_3-D1.rsa	26.286	0.448	0.03	0.522	211	0.125	42.08
T0822TS333_2-D1.rsa	26.136	0.051	0.476	0.473	140	0.187	43.75
T0822TS317_2-D1.rsa	26.027	0.388	0.105	0.507	188	0.138	79.7
T0822TS433_3-D1.rsa	26.027	0.404	0.108	0.488	181	0.144	81.2
T0822TS346_1-D1.rsa	26	0.422	0.12	0.458	115	0.226	90.54
T0822TS433_1-D1.rsa	26	0.458	0.139	0.402	101	0.257	70.12
T0822TS044_1-D1.rsa	25.714	0.448	0.01	0.542	219	0.117	34.16
T0822TS349_4-D1.rsa	25.714	0.49	0.052	0.458	185	0.139	39.91
T0822TS034_4-D1.rsa	25.424	0	0.313	0.687	147	0.173	62.38
T0822TS063_1-D1.rsa	25.424	0.037	0.252	0.71	152	0.167	53.85
T0822TS184_2-D1.rsa	25.424	0.028	0.318	0.654	140	0.182	63.44
T0822TS310_2-D1.rsa	25.253	0.229	0.156	0.615	177	0.143	71.18
T0822TS145_2-D1.rsa	25.253	0.156	0.087	0.757	218	0.116	10.59
T0822TS144_5-D1.rsa	25.253	0.247	0.233	0.521	150	0.168	66.41
T0822TS452_3-D1.rsa	25.17	0.405	0.129	0.466	174	0.145	75.9
T0822TS204_1-D1.rsa	25	0.196	0.054	0.75	249	0.1	49.32
T0822TS116_1-D1.rsa	25	0.43	0.048	0.522	131	0.191	88.84
T0822TS368_2-D1.rsa	25	0.43	0.108	0.462	116	0.216	87.65
T0822TS038_4-D1.rsa	25	0.218	0.074	0.708	172	0.145	39.11
T0822TS162_4-D1.rsa	25	0.37	0.082	0.547	133	0.188	66.33

T0822TS268_5-D1.rsa	25	0.458	0.124	0.418	105	0.238	89.64
T0822TS442_5-D1.rsa	24.8	0.012	0.182	0.805	265	0.094	45.59
T0822TS171_2-D1.rsa	24.8	0.012	0.368	0.62	204	0.122	52.43
T0822TS110_2-D1.rsa	24.691	0.406	0.103	0.491	224	0.11	61.87
T0822TS144_3-D1.rsa	24.675	0.393	0.112	0.496	111	0.222	78.45
T0822TS054_1-D1.rsa	24.675	0.388	0.125	0.487	109	0.226	77.98
T0822TS203_3-D1.rsa	24.658	0.407	0.116	0.477	177	0.139	63.28
T0822TS439_5-D1.rsa	24.59	0	0.348	0.652	137	0.179	52.24
T0822TS425_4-D1.rsa	24.59	0.014	0.433	0.552	116	0.212	60.2
T0822TS171_5-D1.rsa	24.49	0.381	0.102	0.517	193	0.127	83.75
T0822TS080_3-D1.rsa	24.359	0.378	0.132	0.49	145	0.168	59.72
T0822TS381_5-D1.rsa	24.242	0.215	0.184	0.601	173	0.14	69.88
T0822TS433_5-D1.rsa	24.242	0.243	0.222	0.535	154	0.157	70.92
T0822TS204_4-D1.rsa	24.074	0.353	0.081	0.566	258	0.093	55.19
T0822TS171_1-D1.rsa	24	0.423	0.02	0.557	225	0.107	41.65
T0822TS008_5-D1.rsa	24	0.515	0.02	0.465	188	0.128	47.77
T0822TS445_2-D1.rsa	23.762	0.249	0.106	0.645	207	0.115	71.34
T0822TS156_3-D1.rsa	23.762	0.262	0.14	0.598	192	0.124	73.91
T0822TS401_5-D1.rsa	23.729	0	0.266	0.734	157	0.151	65.3
T0822TS204_3-D1.rsa	23.684	0.469	0.024	0.507	231	0.103	66.56
T0822TS032_1-D1.rsa	23.529	0.247	0.066	0.687	167	0.141	42.33
T0822TS439_2-D1.rsa	23.529	0.35	0.095	0.556	135	0.174	65.78
T0822TS381_4-D1.rsa	23.429	0.436	0.027	0.537	217	0.108	40.16
T0822TS445_4-D1.rsa	23.429	0.507	0.01	0.483	195	0.12	44.49
T0822TS157_3-D1.rsa	23.256	0.304	0.085	0.611	173	0.134	70.58
T0822TS268_1-D1.rsa	23.232	0.267	0.226	0.507	146	0.159	72.14
T0822TS169_2-D1.rsa	23.077	0.385	0.02	0.595	176	0.131	48.44
T0822TS333_4-D1.rsa	23.077	0.382	0.135	0.483	143	0.161	57.03
T0822TS184_1-D1.rsa	23.077	0.399	0.125	0.476	141	0.164	57.73
T0822TS328_5-D1.rsa	22.951	0.043	0.39	0.567	119	0.193	62.31
T0822TS258_4-D1.rsa	22.951	0.033	0.448	0.519	109	0.211	70.65
T0822TS041_5-D1.rsa	22.857	0.458	0.022	0.52	210	0.109	37.99
T0822TS345_1-D1.rsa	22.857	0.453	0.032	0.515	208	0.11	40.47
T0822TS268_3-D1.rsa	22.857	0.535	0.054	0.411	166	0.138	35.77
T0822TS335_5-D1.rsa	22.84	0.439	0.094	0.467	213	0.107	54.69
T0822TS118_3-D1.rsa	22.807	0.416	0.091	0.493	108	0.211	80.71
T0822TS120_1-D1.rsa	22.4	0.018	0.195	0.787	259	0.086	45.59
T0822TS133_2-D1.rsa	22.368	0.196	0.033	0.771	256	0.087	44.58
T0822TS277_2-D1.rsa	22.286	0.416	0.035	0.55	222	0.1	39.6
T0822TS116_4-D1.rsa	22.286	0.448	0.022	0.53	214	0.104	44.31
T0822TS381_2-D1.rsa	22.286	0.502	0.027	0.47	190	0.117	40.53
T0822TS153_5-D1.rsa	22.222	0.373	0.083	0.544	248	0.09	70.36
T0822TS197_4-D1.rsa	22.222	0.421	0.09	0.489	223	0.1	70.42
T0822TS345_2-D1.rsa	22.093	0.353	0.113	0.534	151	0.146	72.79
T0822TS436_1-D1.rsa	22.093	0.367	0.117	0.516	146	0.151	72.35
T0822TS203_4-D1.rsa	22.059	0.325	0.058	0.617	150	0.147	68.89
T0822TS144_1-D1.rsa	22.059	0.366	0.115	0.519	126	0.175	65.67

T0822TS360_1-D1.rsa	22.059	0.362	0.107	0.531	129	0.171	67.67
T0822TS203_1-D1.rsa	22.034	0	0.299	0.701	150	0.147	63.2
T0822TS391_1-D1.rsa	21.795	0.355	0.098	0.547	162	0.135	50.78
T0822TS381_3-D1.rsa	21.714	0.458	0.022	0.52	210	0.103	44.31
T0822TS118_4-D1.rsa	21.714	0.411	0.054	0.535	216	0.101	35.27
T0822TS349_3-D1.rsa	21.714	0.455	0.05	0.495	200	0.109	39.42
T0822TS203_5-D1.rsa	21.605	0.428	0.096	0.476	217	0.1	69.65
T0822TS445_1-D1.rsa	21.053	0.43	0.029	0.542	247	0.085	60.53
T0822TS368_5-D1.rsa	21.053	0.416	0.068	0.516	113	0.186	77.97
T0822TS210_3-D1.rsa	20.988	0.419	0.09	0.491	224	0.094	73.45
T0822TS120_2-D1.rsa	20.988	0.432	0.11	0.458	209	0.1	72.13
T0822TS414_1-D1.rsa	20.93	0.392	0.078	0.53	150	0.14	75
T0822TS011_3-D1.rsa	20.93	0.239	0	0.761	102	0.205	23.32
T0822TS157_1-D1.rsa	20.571	0.473	0	0.527	213	0.097	46.72
T0822TS410_1-D1.rsa	20.395	0.423	0.02	0.557	254	0.08	63.98
T0822TS417_2-D1.rsa	20.395	0.434	0.029	0.537	245	0.083	63.32
T0822TS038_1-D1.rsa	20.37	0.432	0.09	0.478	218	0.093	72.3
T0822TS171_3-D1.rsa	20.37	0.434	0.086	0.48	219	0.093	64.96
T0822TS310_5-D1.rsa	20	0.051	0.21	0.739	102	0.196	60.33
T0822TS054_4-D1.rsa	19.767	0.371	0.095	0.534	151	0.131	68.11
T0822TS364_1-D1.rsa	19.737	0.412	0.024	0.564	257	0.077	60.47
T0822TS277_3-D1.rsa	19.737	0.425	0	0.575	262	0.075	62.12
T0822TS268_2-D1.rsa	19.737	0.454	0.011	0.535	244	0.081	60.97
T0822TS067_5-D1.rsa	19.429	0.354	0.005	0.641	259	0.075	23.14
T0822TS054_5-D1.rsa	19.298	0.425	0.059	0.516	113	0.171	73.74
T0822TS160_2-D1.rsa	19.192	0.17	0.097	0.733	211	0.091	11.63
T0822TS157_5-D1.rsa	19.136	0.404	0.092	0.504	230	0.083	67.49
T0822TS197_3-D1.rsa	19.079	0.399	0.004	0.596	272	0.07	52.08
T0822TS326_4-D1.rsa	19.079	0.406	0.024	0.57	260	0.073	60.53
T0822TS042_1-D1.rsa	19.079	0.423	0.024	0.553	252	0.076	63.49
T0822TS235_2-D1.rsa	19.079	0.425	0.024	0.55	251	0.076	60.85
T0822TS044_4-D1.rsa	18.605	0.382	0.113	0.505	143	0.13	76.68
T0822TS281_1-D1.rsa	18.605	0.413	0.095	0.491	139	0.134	73.76
T0822TS204_2-D1.rsa	18.519	0.436	0.094	0.469	214	0.087	70.58
T0822TS417_1-D1.rsa	18.519	0.45	0.094	0.456	208	0.089	68.93
T0822TS065_2-D1.rsa	18.421	0.371	0.009	0.621	283	0.065	61.02
T0822TS436_4-D1.rsa	18.182	0.379	0.08	0.54	121	0.15	75
T0822TS206_1-D1.rsa	17.901	0.441	0.101	0.458	209	0.086	80.41
T0822TS438_1-D1.rsa	17.763	0.423	0.024	0.553	252	0.07	65.41
T0822TS063_3-D1.rsa	17.763	0.45	0.029	0.522	238	0.075	64.91
T0822TS282_5-D1.rsa	17.763	0.463	0.011	0.526	240	0.074	63.21
T0822TS326_5-D1.rsa	17.692	0.242	0.188	0.57	261	0.068	72.94
T0822TS118_5-D1.rsa	17.647	0.362	0.016	0.621	151	0.117	18.78
T0822TS439_3-D1.rsa	17.544	0.347	0.064	0.589	129	0.136	73.52
T0822TS044_3-D1.rsa	17.442	0.382	0.113	0.505	143	0.122	80.12
T0822TS032_4-D1.rsa	17.442	0.392	0.099	0.509	144	0.121	77.74
T0822TS173_5-D1.rsa	17.442	0.406	0.113	0.481	136	0.128	75.44

T0822TS133_1-D1.rsa	17.105	0.423	0.015	0.561	256	0.067	58.55
T0822TS110_4-D1.rsa	17.105	0.316	0.117	0.566	188	0.091	90.81
T0822TS437_1-D1.rsa	17.105	0.286	0.096	0.617	205	0.083	51.73
T0822TS368_1-D1.rsa	17.021	0.315	0.195	0.49	126	0.135	85.6
T0822TS277_5-D1.rsa	16.949	0.056	0.229	0.715	153	0.111	53.97
T0822TS499_1-D1.rsa	16.923	0.262	0.216	0.522	239	0.071	71.28
T0822TS067_3-D1.rsa	16.883	0.388	0.08	0.531	119	0.142	76.55
T0822TS499_3-D1.rsa	16.447	0.441	0.024	0.535	244	0.067	68.59
T0822TS277_1-D1.rsa	16.447	0.425	0.015	0.559	255	0.064	58.22
T0822TS080_2-D1.rsa	16.447	0.456	0.015	0.529	241	0.068	64.2
T0822TS117_3-D1.rsa	16.154	0.255	0.166	0.579	265	0.061	68.39
T0822TS235_4-D1.rsa	16.154	0.273	0.183	0.544	249	0.065	74.5
T0822TS326_2-D1.rsa	16.154	0.262	0.227	0.511	234	0.069	77.78
T0822TS133_3-D1.rsa	16.154	0.26	0.221	0.52	238	0.068	74.94
T0822TS434_1-D1.rsa	16.154	0.26	0.236	0.504	231	0.07	71.28
T0822TS184_3-D1.rsa	15.789	0.42	0.087	0.493	108	0.146	78.88
T0822TS479_4-D1.rsa	15.789	0.42	0.096	0.484	106	0.149	80.59
T0822TS436_2-D1.rsa	15.385	0.26	0.218	0.522	239	0.064	78.33
T0822TS282_2-D1.rsa	15.385	0.266	0.229	0.504	231	0.067	75.94
T0822TS290_3-D1.rsa	15.385	0.258	0.238	0.504	231	0.067	74.44
T0822TS482_1-D1.rsa	14.615	0.255	0.221	0.524	240	0.061	75.83
T0822TS120_5-D1.rsa	14.615	0.26	0.21	0.531	243	0.06	77.61
T0822TS277_4-D1.rsa	14.615	0.264	0.218	0.517	237	0.062	74.44
T0822TS404_1-D1.rsa	14.615	0.269	0.242	0.489	224	0.065	76.72
T0822TS237_3-D1.rsa	14.615	0.264	0.238	0.498	228	0.064	72.67
T0822TS404_2-D1.rsa	14.474	0.271	0.072	0.657	218	0.066	98.34
T0822TS391_4-D1.rsa	14.474	0.331	0.142	0.527	175	0.083	99.4
T0822TS008_3-D1.rsa	14.474	0.346	0.123	0.53	176	0.082	98.87
T0822TS335_2-D1.rsa	14.474	0.352	0.123	0.524	174	0.083	98.8
T0822TS162_2-D1.rsa	14.474	0.232	0.117	0.651	216	0.067	48.95
T0822TS153_1-D1.rsa	14.035	0.425	0.082	0.493	108	0.13	78.88
T0822TS428_2-D1.rsa	14.035	0.438	0.082	0.479	105	0.134	81.39
T0822TS338_3-D1.rsa	14.035	0.411	0.105	0.484	106	0.132	78.88
T0822TS251_2-D1.rsa	13.846	0.242	0.19	0.568	260	0.053	71.67
T0822TS282_1-D1.rsa	13.846	0.258	0.223	0.52	238	0.058	78.94
T0822TS276_1-D1.rsa	13.846	0.262	0.212	0.526	241	0.057	75.5
T0822TS116_3-D1.rsa	13.846	0.269	0.24	0.491	225	0.062	70.44
T0822TS203_2-D1.rsa	13.158	0.328	0.117	0.554	184	0.072	98.64
T0822TS169_5-D1.rsa	13.158	0.352	0.123	0.524	174	0.076	98.87
T0822TS310_3-D1.rsa	13.158	0.349	0.142	0.509	169	0.078	99.17
T0822TS483_1-D1.rsa	13.077	0.251	0.214	0.535	245	0.053	72.78
T0822TS144_4-D1.rsa	12.766	0.331	0.086	0.584	150	0.085	81.52
T0822TS333_1-D1.rsa	12.766	0.327	0.191	0.482	124	0.103	73.83
T0822TS439_1-D1.rsa	12	0	0.043	0.957	132	0.091	59.6
T0822TS404_3-D1.rsa	11.842	0.175	0.072	0.753	250	0.047	50.9
T0822TS260_4-D1.rsa	11.842	0.31	0.154	0.536	178	0.067	98.42
T0822TS347_2-D1.rsa	11.842	0.352	0.123	0.524	174	0.068	82

T0822TS420_3-D1.rsa	10.638	0.307	0.14	0.553	142	0.075	85.12
T0822TS340_2-D1.rsa	10.638	0.339	0.183	0.479	123	0.086	81.42
T0822TS358_1-D1.rsa	10.638	0.342	0.187	0.471	121	0.088	82.2
T0822TS153_2-D1.rsa	10.638	0.339	0.198	0.463	119	0.089	84.05
T0822TS296_3-D1.rsa	10.638	0.35	0.183	0.467	120	0.089	81.32
T0822TS349_1-D1.rsa	8.511	0.315	0.179	0.506	130	0.065	79.86
T0822TS454_1-D1.rsa	8.511	0.339	0.183	0.479	123	0.069	84.34
T0822TS333_3-D1.rsa	6.383	0.331	0.183	0.486	125	0.051	82.3
T0861TS464_5-D1.rsa	56.481	0.279	0.013	0.708	221	0.256	17.47
T0861TS321_3-D1.rsa	52.778	0.292	0.026	0.683	213	0.248	8.73
T0861TS321_2-D1.rsa	50.926	0.285	0.019	0.696	217	0.235	8.73
T0861TS321_4-D1.rsa	47.222	0.288	0.038	0.673	210	0.225	10.1
T0861TS464_2-D1.rsa	44.444	0.26	0.013	0.728	227	0.196	17.79
T0861TS321_5-D1.rsa	39.815	0.295	0.042	0.663	207	0.192	9.29
T0861TS321_1-D1.rsa	36.111	0.288	0.019	0.692	216	0.167	8.65
T0861TS451_5-D1.rsa	33.333	0.359	0.016	0.625	195	0.171	36.54
T0861TS451_2-D1.rsa	31.481	0.353	0.013	0.635	198	0.159	34.45
T0861TS451_1-D1.rsa	29.63	0.349	0.048	0.603	188	0.158	39.02
T0861TS432_3-D1.rsa	29.63	0.365	0.077	0.558	174	0.17	39.26
T0861TS434_4-D1.rsa	29.63	0.327	0.006	0.667	208	0.142	13.14
T0861TS451_3-D1.rsa	28.704	0.365	0.026	0.609	190	0.151	31.97
T0861TS434_1-D1.rsa	27.778	0.362	0.006	0.631	197	0.141	18.59
T0861TS434_5-D1.rsa	27.778	0.34	0	0.66	206	0.135	12.42
T0861TS451_4-D1.rsa	26.852	0.362	0.035	0.603	188	0.143	38.62
T0861TS180_5-D1.rsa	26.852	0.295	0.045	0.66	206	0.13	41.11
T0861TS432_5-D1.rsa	25.926	0.375	0.061	0.564	176	0.147	42.71
T0861TS434_3-D1.rsa	23.148	0.343	0	0.657	205	0.113	11.3
T0861TS434_2-D1.rsa	22.222	0.308	0	0.692	216	0.103	12.02
T0861TS284_2-D1.rsa	22.222	0.343	0.131	0.526	164	0.136	70.59
T0861TS180_3-D1.rsa	21.296	0.404	0.125	0.471	147	0.145	87.82
T0861TS382_4-D1.rsa	20.37	0.397	0.128	0.474	148	0.138	71.88
T0861TS432_2-D1.rsa	20.37	0.337	0.061	0.603	188	0.108	37.58
T0861TS432_1-D1.rsa	20.37	0.369	0.09	0.542	169	0.121	42.23
T0861TS382_1-D1.rsa	20.37	0.388	0.128	0.484	151	0.135	72.84
T0861TS382_3-D1.rsa	19.444	0.397	0.128	0.474	148	0.131	72.2
T0861TS026_4-D1.rsa	19.444	0.356	0.138	0.506	158	0.123	79.33
T0861TS432_4-D1.rsa	19.444	0.362	0.096	0.542	169	0.115	36.46
T0861TS357_3-D1.rsa	19.444	0.346	0.013	0.641	200	0.097	89.9
T0861TS382_2-D1.rsa	18.519	0.391	0.131	0.478	149	0.124	71.95
T0861TS421_5-D1.rsa	18.519	0.34	0.115	0.545	170	0.109	75.88
T0861TS357_4-D1.rsa	18.519	0.321	0.026	0.654	204	0.091	89.98
T0861TS357_1-D1.rsa	18.519	0.324	0.013	0.663	207	0.089	91.35
T0861TS430_5-D1.rsa	18.519	0.401	0.093	0.506	158	0.117	92.15
T0861TS284_5-D1.rsa	18.519	0.394	0.135	0.471	147	0.126	91.59
T0861TS275_5-D1.rsa	18.519	0.394	0.128	0.478	149	0.124	80.29
T0861TS220_5-D1.rsa	17.593	0.397	0.103	0.5	156	0.113	92.63
T0861TS284_3-D1.rsa	17.593	0.362	0.131	0.506	158	0.111	78.36

T0861TS284_4-D1.rsa	17.593	0.388	0.131	0.481	150	0.117	91.11
T0861TS467_4-D1.rsa	17.593	0.372	0.128	0.5	156	0.113	89.98
T0861TS382_5-D1.rsa	16.667	0.397	0.131	0.471	147	0.113	72.2
T0861TS026_1-D1.rsa	16.667	0.356	0.112	0.532	166	0.1	91.03
T0861TS407_2-D1.rsa	16.667	0.385	0.125	0.49	153	0.109	96.55
T0861TS275_3-D1.rsa	16.667	0.381	0.128	0.49	153	0.109	81.65
T0861TS026_5-D1.rsa	16.667	0.369	0.128	0.503	157	0.106	91.43
T0861TS357_5-D1.rsa	16.667	0.337	0.006	0.657	205	0.081	90.06
T0861TS425_2-D1.rsa	16.667	0.394	0.131	0.474	148	0.113	93.99
T0861TS467_3-D1.rsa	16.667	0.381	0.131	0.487	152	0.11	73.64
T0861TS425_1-D1.rsa	16.667	0.401	0.131	0.468	146	0.114	94.23
T0861TS430_1-D1.rsa	16.667	0.41	0.135	0.455	142	0.117	95.51
T0861TS349_1-D1.rsa	16.667	0.401	0.128	0.471	147	0.113	93.43
T0861TS407_4-D1.rsa	16.667	0.401	0.128	0.471	147	0.113	95.35
T0861TS275_4-D1.rsa	16.667	0.359	0.128	0.513	160	0.104	79.49
T0861TS077_2-D1.rsa	16.667	0.407	0.128	0.465	145	0.115	93.91
T0861TS220_2-D1.rsa	16.667	0.407	0.131	0.462	144	0.116	95.19
T0861TS077_4-D1.rsa	16.667	0.401	0.135	0.465	145	0.115	94.55
T0861TS180_4-D1.rsa	16.667	0.381	0.125	0.494	154	0.108	88.38
T0861TS180_1-D1.rsa	16.667	0.397	0.128	0.474	148	0.113	90.62
T0861TS357_2-D1.rsa	16.667	0.308	0.035	0.657	205	0.081	91.35
T0861TS345_2-D1.rsa	16.667	0.417	0.128	0.455	142	0.117	95.43
T0861TS479_4-D1.rsa	15.741	0.397	0.125	0.478	149	0.106	94.07
T0861TS183_3-D1.rsa	15.741	0.397	0.128	0.474	148	0.106	95.11
T0861TS421_2-D1.rsa	15.741	0.378	0.125	0.497	155	0.102	97.68
T0861TS430_4-D1.rsa	15.741	0.413	0.128	0.458	143	0.11	95.03
T0861TS183_5-D1.rsa	15.741	0.381	0.096	0.522	163	0.097	84.78
T0861TS421_4-D1.rsa	15.741	0.356	0.096	0.548	171	0.092	91.59
T0861TS495_1-D1.rsa	15.741	0.388	0.138	0.474	148	0.106	91.99
T0861TS479_5-D1.rsa	15.741	0.369	0.087	0.545	170	0.093	83.65
T0861TS444_1-D1.rsa	15.741	0.407	0.119	0.474	148	0.106	89.42
T0861TS421_3-D1.rsa	15.741	0.353	0.099	0.548	171	0.092	92.95
T0861TS220_4-D1.rsa	15.741	0.407	0.138	0.455	142	0.111	94.87
T0861TS275_2-D1.rsa	15.741	0.404	0.125	0.471	147	0.107	99.04
T0861TS444_2-D1.rsa	15.741	0.394	0.131	0.474	148	0.106	89.1
T0861TS455_2-D1.rsa	15.741	0.359	0.109	0.532	166	0.095	80.93
T0861TS446_4-D1.rsa	15.741	0.41	0.128	0.462	144	0.109	91.11
T0861TS236_2-D1.rsa	15.741	0.404	0.128	0.468	146	0.108	96.64
T0861TS180_2-D1.rsa	15.741	0.381	0.128	0.49	153	0.103	79.81
T0861TS425_5-D1.rsa	15.741	0.407	0.131	0.462	144	0.109	93.27
T0861TS077_1-D1.rsa	15.741	0.41	0.135	0.455	142	0.111	93.67
T0861TS430_2-D1.rsa	15.741	0.413	0.125	0.462	144	0.109	95.19
T0861TS430_3-D1.rsa	15.741	0.391	0.135	0.474	148	0.106	94.87
T0861TS345_4-D1.rsa	15.741	0.356	0.119	0.526	164	0.096	63.06
T0861TS479_3-D1.rsa	14.815	0.394	0.125	0.481	150	0.099	93.59
T0861TS183_2-D1.rsa	14.815	0.401	0.128	0.471	147	0.101	92.87
T0861TS359_5-D1.rsa	14.815	0.404	0.122	0.474	148	0.1	98.96

T0861TS444_4-D1.rsa	14.815	0.372	0.128	0.5	156	0.095	87.58
T0861TS313_3-D1.rsa	14.815	0.401	0.122	0.478	149	0.099	93.59
T0861TS407_5-D1.rsa	14.815	0.375	0.135	0.49	153	0.097	95.03
T0861TS313_4-D1.rsa	14.815	0.401	0.128	0.471	147	0.101	93.59
T0861TS287_4-D1.rsa	14.815	0.401	0.135	0.465	145	0.102	97.11
T0861TS467_5-D1.rsa	14.815	0.356	0.125	0.519	162	0.091	79.41
T0861TS250_2-D1.rsa	14.815	0.404	0.122	0.474	148	0.1	92.31
T0861TS313_1-D1.rsa	14.815	0.407	0.128	0.465	145	0.102	93.59
T0861TS077_5-D1.rsa	14.815	0.407	0.125	0.468	146	0.101	93.91
T0861TS425_4-D1.rsa	14.815	0.404	0.128	0.468	146	0.101	94.63
T0861TS464_4-D1.rsa	14.815	0.381	0.131	0.487	152	0.097	90.47
T0861TS287_2-D1.rsa	14.815	0.407	0.128	0.465	145	0.102	97.11
T0861TS236_3-D1.rsa	14.815	0.41	0.135	0.455	142	0.104	96.39
T0861TS287_3-D1.rsa	14.815	0.41	0.128	0.462	144	0.103	97.2
T0861TS359_4-D1.rsa	14.815	0.404	0.125	0.471	147	0.101	98.56
T0861TS345_5-D1.rsa	14.815	0.417	0.128	0.455	142	0.104	95.83
T0861TS250_1-D1.rsa	14.815	0.404	0.122	0.474	148	0.1	92.23
T0861TS345_1-D1.rsa	14.815	0.401	0.128	0.471	147	0.101	96.31
T0861TS236_1-D1.rsa	14.815	0.407	0.128	0.465	145	0.102	98.64
T0861TS236_4-D1.rsa	14.815	0.41	0.128	0.462	144	0.103	98
T0861TS250_4-D1.rsa	14.815	0.404	0.122	0.474	148	0.1	92.39
T0861TS287_5-D1.rsa	14.815	0.41	0.135	0.455	142	0.104	96.8
T0861TS407_3-D1.rsa	13.889	0.388	0.119	0.494	154	0.09	95.75
T0861TS313_5-D1.rsa	13.889	0.401	0.125	0.474	148	0.094	93.75
T0861TS425_3-D1.rsa	13.889	0.404	0.131	0.465	145	0.096	94.23
T0861TS446_2-D1.rsa	13.889	0.381	0.128	0.49	153	0.091	91.67
T0861TS313_2-D1.rsa	13.889	0.401	0.128	0.471	147	0.094	93.75
T0861TS220_3-D1.rsa	13.889	0.397	0.138	0.465	145	0.096	94.87
T0861TS220_1-D1.rsa	13.889	0.413	0.135	0.452	141	0.099	95.27
T0861TS005_5-D1.rsa	13.889	0.404	0.128	0.468	146	0.095	92.47
T0861TS005_3-D1.rsa	13.889	0.41	0.128	0.462	144	0.096	92.95
T0861TS464_1-D1.rsa	13.889	0.388	0.131	0.481	150	0.093	90.7
T0861TS005_4-D1.rsa	13.889	0.401	0.128	0.471	147	0.094	92.63
T0861TS287_1-D1.rsa	13.889	0.407	0.128	0.465	145	0.096	98.64
T0861TS005_1-D1.rsa	13.889	0.407	0.125	0.468	146	0.095	92.71
T0861TS119_1-D1.rsa	13.889	0.401	0.128	0.471	147	0.094	93.43
T0861TS479_1-D1.rsa	12.963	0.401	0.112	0.487	152	0.085	95.83
T0861TS183_4-D1.rsa	12.963	0.407	0.125	0.468	146	0.089	95.35
T0861TS251_5-D1.rsa	12.963	0.394	0.125	0.481	150	0.086	86.54
T0861TS258_2-D1.rsa	12.963	0.365	0.119	0.516	161	0.081	89.9
T0861TS407_1-D1.rsa	12.963	0.394	0.125	0.481	150	0.086	96.8
T0861TS258_1-D1.rsa	12.963	0.381	0.119	0.5	156	0.083	89.58
T0861TS284_1-D1.rsa	12.963	0.394	0.138	0.468	146	0.089	82.21
T0861TS016_1-D1.rsa	12.963	0.41	0.135	0.455	142	0.091	96.95
T0861TS077_3-D1.rsa	12.963	0.407	0.135	0.458	143	0.091	95.03
T0861TS446_5-D1.rsa	12.963	0.407	0.122	0.471	147	0.088	88.62
T0861TS446_1-D1.rsa	12.963	0.381	0.128	0.49	153	0.085	91.59

T0861TS166_1-D1.rsa	12.963	0.404	0.128	0.468	146	0.089	92.55
T0861TS405_4-D1.rsa	12.963	0.41	0.128	0.462	144	0.09	91.91
T0861TS345_3-D1.rsa	12.963	0.391	0.141	0.468	146	0.089	88.62
T0861TS005_2-D1.rsa	12.963	0.41	0.125	0.465	145	0.089	92.79
T0861TS183_1-D1.rsa	12.963	0.404	0.122	0.474	148	0.088	95.99
T0861TS444_5-D1.rsa	12.963	0.372	0.125	0.503	157	0.083	84.78
T0861TS092_1-D1.rsa	12.963	0.394	0.131	0.474	148	0.088	91.19
T0861TS467_2-D1.rsa	12.963	0.397	0.128	0.474	148	0.088	86.78
T0861TS251_4-D1.rsa	12.963	0.394	0.125	0.481	150	0.086	88.22
T0861TS444_3-D1.rsa	12.963	0.381	0.135	0.484	151	0.086	86.7
T0861TS236_5-D1.rsa	12.963	0.413	0.131	0.455	142	0.091	97.68
T0861TS251_3-D1.rsa	12.963	0.388	0.128	0.484	151	0.086	88.14
T0861TS258_4-D1.rsa	12.037	0.369	0.119	0.513	160	0.075	90.39
T0861TS455_5-D1.rsa	12.037	0.385	0.112	0.503	157	0.077	88.62
T0861TS359_2-D1.rsa	12.037	0.404	0.119	0.478	149	0.081	95.83
T0861TS455_4-D1.rsa	12.037	0.349	0.096	0.554	173	0.07	80.61
T0861TS251_2-D1.rsa	12.037	0.404	0.125	0.471	147	0.082	85.18
T0861TS048_1-D1.rsa	12.037	0.404	0.125	0.471	147	0.082	91.91
T0861TS467_1-D1.rsa	12.037	0.372	0.128	0.5	156	0.077	82.85
T0861TS455_3-D1.rsa	12.037	0.375	0.115	0.51	159	0.076	83.49
T0861TS251_1-D1.rsa	12.037	0.397	0.125	0.478	149	0.081	87.02
T0861TS258_3-D1.rsa	11.111	0.369	0.119	0.513	160	0.069	90.47
T0861TS455_1-D1.rsa	11.111	0.404	0.128	0.468	146	0.076	92.31
T0861TS359_1-D1.rsa	11.111	0.41	0.125	0.465	145	0.077	92.31
T0861TS405_3-D1.rsa	11.111	0.397	0.128	0.474	148	0.075	92.47
T0861TS275_1-D1.rsa	11.111	0.404	0.125	0.471	147	0.076	93.03
T0861TS405_2-D1.rsa	11.111	0.404	0.125	0.471	147	0.076	92.55
T0861TS405_1-D1.rsa	11.111	0.404	0.125	0.471	147	0.076	92.71
T0861TS026_2-D1.rsa	11.111	0.394	0.122	0.484	151	0.074	91.43
T0861TS479_2-D1.rsa	10.185	0.404	0.122	0.474	148	0.069	93.59
T0861TS258_5-D1.rsa	10.185	0.381	0.125	0.494	154	0.066	90.55
T0830TS032_1_2-D2.rsa	92.188	0.022	0	0.978	133	0.693	10.29
T0830TS479_2-D2.rsa	92	0.087	0	0.913	126	0.73	18.84
T0830TS368_1-D2.rsa	84.507	0.452	0	0.548	92	0.919	18.43
T0830TS160_4-D2.rsa	77.143	0.428	0	0.572	231	0.334	6.68
T0830TS349_2-D2.rsa	75	0	0.491	0.509	55	1.364	23.15
T0830TS064_2-D2.rsa	74.561	0.217	0.009	0.774	168	0.444	12.33
T0830TS117_5-D2.rsa	69.737	0.006	0	0.994	330	0.211	8.58
T0830TS368_3-D2.rsa	66.667	0.019	0.426	0.556	60	1.111	67.82
T0830TS067_1-D2.rsa	65.116	0.41	0.052	0.537	72	0.904	41.79
T0830TS300_3-D2.rsa	60.769	0.179	0.007	0.814	373	0.163	6.17
T0830TS317_1_2-D2.rsa	60.563	0.161	0	0.839	141	0.43	16.99
T0830TS430_1-D2.rsa	60.465	0.201	0	0.799	107	0.565	40.11
T0830TS197_5-D2.rsa	58.333	0.019	0.519	0.463	50	1.167	65.51
T0830TS026_1-D2.rsa	56.579	0.193	0.004	0.803	366	0.155	6.09
T0830TS439_5-D2.rsa	55.814	0.306	0.06	0.634	85	0.657	42.35
T0830TS206_1-D2.rsa	55.769	0.309	0.139	0.552	91	0.613	74.37

T0830TS118_5-D2.rsa	55.769	0.255	0.139	0.606	100	0.558	74.05
T0830TS436_3-D2.rsa	55.429	0.277	0	0.723	292	0.19	6.87
T0830TS391_2-D2.rsa	54.167	0	0.556	0.444	48	1.128	71.53
T0830TS184_2-D2.rsa	51.923	0.273	0.048	0.679	112	0.464	62.82
T0830TS038_1-D2.rsa	51.163	0.246	0.03	0.724	97	0.527	46.08
T0830TS235_4-D2.rsa	50.769	0.166	0.05	0.784	359	0.141	9.78
T0830TS049_1-D2.rsa	50	0.008	0.008	0.984	239	0.209	7.33
T0830TS144_5-D2.rsa	48.052	0.183	0	0.817	183	0.263	10
T0830TS228_2-D2.rsa	48	0.014	0.246	0.739	102	0.471	30.62
T0830TS454_4-D2.rsa	47.368	0.297	0.108	0.595	66	0.718	39.41
T0830TS169_4-D2.rsa	46.429	0	0.015	0.985	129	0.36	41.6
T0830TS054_1-D2.rsa	45.313	0.015	0.257	0.728	99	0.458	59.56
T0830TS310_4-D2.rsa	44.828	0	0.091	0.909	130	0.345	58.39
T0830TS277_4-D2.rsa	44.828	0	0.07	0.93	133	0.337	55.59
T0830TS277_3-D2.rsa	44.737	0.383	0.03	0.587	195	0.229	10.99
T0830TS145_2-D2.rsa	44.318	0.074	0.429	0.497	147	0.301	36.91
T0830TS340_5-D2.rsa	43.662	0.524	0.119	0.357	60	0.728	34.13
T0830TS064_4-D2.rsa	43.421	0.358	0.024	0.617	205	0.212	8.43
T0830TS080_2-D2.rsa	42.763	0.445	0.015	0.539	246	0.174	16.5
T0830TS425_2-D2.rsa	42.5	0.035	0.5	0.465	53	0.802	20.61
T0830TS499_1-D2.rsa	42.424	0	0.26	0.74	213	0.199	8.42
T0830TS410_2-D2.rsa	42.045	0.068	0.399	0.534	158	0.266	47.97
T0830TS328_5_2-D2.rsa	42.045	0.074	0.382	0.544	161	0.261	25.51
T0830TS340_3-D2.rsa	41.667	0.305	0.009	0.685	146	0.285	62.69
T0830TS347_1-D2.rsa	41.379	0	0.098	0.902	129	0.321	56.12
T0830TS425_3-D2.rsa	40	0.07	0.053	0.877	100	0.4	38.6
T0830TS064_3-D2.rsa	39.773	0.054	0.419	0.527	156	0.255	35.9
T0830TS184_3-D2.rsa	39.773	0.061	0.456	0.483	143	0.278	42.06
T0830TS457_5-D2.rsa	39.655	0.014	0.126	0.86	123	0.322	60.31
T0830TS032_2_2-D2.rsa	39.535	0.157	0	0.843	113	0.35	44.59
T0830TS345_4-D2.rsa	39.535	0.261	0.045	0.694	93	0.425	44.22
T0830TS403_5-D2.rsa	39.429	0.3	0	0.7	283	0.139	7.43
T0830TS080_1-D2.rsa	39.429	0.369	0.005	0.626	253	0.156	7.36
T0830TS483_3-D2.rsa	38.614	0.28	0.159	0.561	180	0.215	72.35
T0830TS204_2-D2.rsa	38.614	0.293	0.14	0.567	182	0.212	45.95
T0830TS296_4-D2.rsa	38.596	0.244	0.203	0.553	120	0.322	64.75
T0830TS317_4_2-D2.rsa	38.384	0.253	0.111	0.635	183	0.21	11.72
T0830TS290_1-D2.rsa	38.158	0.337	0.027	0.636	211	0.181	8.43
T0830TS268_1-D2.rsa	37.5	0.036	0.309	0.655	72	0.521	64.32
T0830TS310_2-D2.rsa	37.374	0.24	0.208	0.552	159	0.235	47.4
T0830TS080_5-D2.rsa	36.842	0.258	0.194	0.548	119	0.31	61.41
T0830TS145_3-D2.rsa	36.842	0.252	0.18	0.568	63	0.585	37.16
T0830TS360_2-D2.rsa	36.634	0.234	0.069	0.698	224	0.164	32.4
T0830TS011_4-D2.rsa	36.571	0.366	0.042	0.592	239	0.153	33.29
T0830TS445_3-D2.rsa	36.364	0.02	0.291	0.689	204	0.178	42.91
T0830TS132_2-D2.rsa	36.066	0.052	0.41	0.538	113	0.319	67.29
T0830TS445_5-D2.rsa	35.789	0.538	0.055	0.407	96	0.373	59.53

T0830TS156_1-D2.rsa	35.354	0.201	0.132	0.667	192	0.184	47.31
T0830TS282_1-D2.rsa	35.2	0.021	0.347	0.632	208	0.169	43.31
T0830TS338_2-D2.rsa	34.483	0	0.21	0.79	113	0.305	77.45
T0830TS345_2-D2.rsa	34.483	0.605	0	0.395	101	0.341	56.84
T0830TS310_1-D2.rsa	34.426	0.019	0.4	0.581	122	0.282	63.31
T0830TS420_2-D2.rsa	34.4	0.006	0.267	0.726	239	0.144	31.69
T0830TS328_3_2-D2.rsa	34.375	0	0.037	0.963	131	0.262	55.7
T0830TS483_5-D2.rsa	34.375	0.015	0.426	0.559	76	0.452	69.67
T0830TS210_4-D2.rsa	34.014	0.357	0.091	0.552	206	0.165	48.61
T0830TS345_5-D2.rsa	33.803	0.333	0.095	0.571	96	0.352	38.46
T0830TS499_2-D2.rsa	33.6	0.006	0.31	0.684	225	0.149	47.34
T0830TS034_1-D2.rsa	33.077	0.12	0.048	0.832	381	0.087	12.22
T0830TS065_4-D2.rsa	32.8	0.012	0.359	0.629	207	0.158	56.08
T0830TS054_4-D2.rsa	32.787	0.033	0.39	0.576	121	0.271	64.3
T0830TS277_1-D2.rsa	32.673	0.29	0.15	0.561	180	0.182	72.66
T0830TS204_3-D2.rsa	32.653	0.378	0.094	0.528	197	0.166	26.18
T0830TS438_1-D2.rsa	32.571	0.498	0.005	0.498	201	0.162	18.38
T0830TS118_1-D2.rsa	32.558	0.308	0.201	0.491	83	0.392	72.63
T0830TS145_4-D2.rsa	32.5	0.018	0.447	0.535	61	0.533	23.68
T0830TS425_5-D2.rsa	32.381	0.355	0.192	0.453	106	0.305	70.41
T0830TS326_5-D2.rsa	32.192	0.361	0.094	0.544	202	0.159	70.44
T0830TS499_4-D2.rsa	32.192	0.394	0.1	0.507	188	0.171	76.77
T0830TS237_3-D2.rsa	32.192	0.375	0.097	0.528	196	0.164	71.59
T0830TS420_5-D2.rsa	32	0.015	0.337	0.647	213	0.15	50.91
T0830TS349_4-D2.rsa	32	0.065	0.246	0.688	95	0.337	65.04
T0830TS420_4-D2.rsa	31.818	0.007	0.236	0.757	224	0.142	8.19
T0830TS042_2-D2.rsa	31.683	0.271	0.137	0.592	190	0.167	74.45
T0830TS160_1-D2.rsa	31.579	0.263	0	0.737	336	0.094	40.08
T0830TS118_3-D2.rsa	31.579	0.555	0.047	0.398	94	0.336	62.5
T0830TS144_2-D2.rsa	31.429	0.274	0.239	0.487	114	0.276	68.48
T0830TS333_2-D2.rsa	31.313	0.264	0.177	0.559	161	0.194	47.92
T0830TS349_3-D2.rsa	31.25	0.064	0.5	0.436	48	0.651	72.95
T0830TS038_5-D2.rsa	31.2	0.021	0.395	0.584	192	0.163	53.19
T0830TS210_3-D2.rsa	30.882	0.346	0.074	0.58	141	0.219	58.33
T0830TS454_3-D2.rsa	30.857	0.463	0.054	0.483	195	0.158	33.97
T0830TS169_3-D2.rsa	30.822	0.385	0.102	0.512	190	0.162	80.25
T0830TS420_3-D2.rsa	30.612	0.279	0.064	0.657	245	0.125	42.36
T0830TS184_4-D2.rsa	30.508	0	0.285	0.715	153	0.199	64.02
T0830TS145_1-D2.rsa	30.357	0.198	0.397	0.405	51	0.595	51.59
T0830TS044_3-D2.rsa	30.286	0.423	0.03	0.547	221	0.137	38.86
T0830TS410_5-D2.rsa	30.263	0.268	0.012	0.72	239	0.127	12.88
T0830TS439_4-D2.rsa	30.172	0.723	0	0.277	71	0.425	78.52
T0830TS235_1-D2.rsa	30.137	0.399	0.105	0.496	184	0.164	77.52
T0830TS300_2-D2.rsa	29.932	0.311	0.021	0.668	249	0.12	64.93
T0830TS038_3-D2.rsa	29.703	0.268	0.146	0.586	188	0.158	75.47
T0830TS410_1-D2.rsa	29.6	0.012	0.301	0.687	226	0.131	50.53
T0830TS296_1-D2.rsa	29.524	0.252	0.188	0.56	131	0.225	53.53

T0830TS157_2-D2.rsa	29.452	0.388	0.084	0.528	196	0.15	72.48
T0830TS210_1-D2.rsa	29.252	0.357	0.099	0.544	203	0.144	83.19
T0830TS328_2_2-D2.rsa	28.814	0	0.29	0.71	152	0.19	58.18
T0830TS132_4-D2.rsa	28.814	0.028	0.313	0.659	141	0.204	63.55
T0830TS049_3-D2.rsa	28.8	0.012	0.35	0.638	210	0.137	37.46
T0830TS160_5-D2.rsa	28.767	0.35	0.119	0.531	197	0.146	76.29
T0830TS448_2-D2.rsa	28.767	0.369	0.1	0.531	197	0.146	78.34
T0830TS340_2-D2.rsa	28.767	0.415	0.084	0.501	186	0.155	78.95
T0830TS237_2-D2.rsa	28.767	0.42	0.092	0.488	181	0.159	79.02
T0830TS263_3-D2.rsa	28.713	0.265	0.14	0.595	191	0.15	74.38
T0830TS483_4-D2.rsa	28.713	0.262	0.159	0.579	186	0.154	74.22
T0830TS368_4-D2.rsa	28.571	0.388	0.107	0.504	113	0.253	77.62
T0830TS042_3-D2.rsa	28.283	0.233	0.16	0.608	175	0.162	59.46
T0830TS403_4-D2.rsa	28.283	0.215	0.181	0.604	174	0.163	60.76
T0830TS317_3_2-D2.rsa	28.205	0.361	0.122	0.517	153	0.184	56.25
T0830TS065_1_2-D2.rsa	28.082	0.396	0.105	0.499	185	0.152	73.36
T0830TS333_4-D2.rsa	28	0.018	0.322	0.66	217	0.129	52.2
T0830TS054_5-D2.rsa	28	0.414	0.12	0.466	117	0.239	90.44
T0830TS358_4-D2.rsa	28	0.051	0.29	0.659	91	0.308	67.75
T0830TS065_5-D2.rsa	27.941	0.374	0.103	0.523	127	0.22	71.44
T0830TS064_1-D2.rsa	27.891	0.37	0.102	0.528	197	0.142	82.71
T0830TS349_1-D2.rsa	27.869	0.024	0.39	0.586	123	0.227	65.55
T0830TS420_1-D2.rsa	27.723	0.265	0.14	0.595	191	0.145	74.45
T0830TS349_5-D2.rsa	27.619	0.376	0.218	0.406	95	0.291	74.25
T0830TS212_2-D2.rsa	27.429	0.292	0.01	0.698	282	0.097	23.52
T0830TS457_4-D2.rsa	27.273	0.226	0.219	0.556	160	0.17	67.71
T0830TS011_5-D2.rsa	27.211	0.37	0.099	0.531	198	0.137	83.96
T0830TS263_1-D2.rsa	27.119	0.009	0.145	0.846	181	0.15	65.07
T0830TS204_4-D2.rsa	27.119	0	0.304	0.696	149	0.182	63.2
T0830TS212_1-D2.rsa	27.119	0	0.299	0.701	150	0.181	59.93
T0830TS290_5-D2.rsa	26.923	0.351	0.101	0.547	162	0.166	56.68
T0830TS268_2-D2.rsa	26.923	0.382	0.108	0.51	151	0.178	56.25
T0830TS041_1-D2.rsa	26.733	0.28	0.115	0.604	194	0.138	76.25
T0830TS326_1-D2.rsa	26.733	0.268	0.14	0.592	190	0.141	74.22
T0830TS338_4-D2.rsa	26.733	0.259	0.14	0.601	193	0.139	74.53
T0830TS310_3-D2.rsa	26.733	0.193	0.128	0.679	218	0.123	32.48
T0830TS448_3-D2.rsa	26.531	0.378	0.102	0.52	194	0.137	84.17
T0830TS157_5-D2.rsa	26.531	0.367	0.11	0.523	195	0.136	80.62
T0830TS097_1-D2.rsa	26.531	0.41	0.107	0.483	180	0.147	87.22
T0830TS160_3-D2.rsa	26.531	0.378	0.097	0.525	196	0.135	81.11
T0830TS197_3-D2.rsa	26.531	0.383	0.11	0.507	189	0.14	79.44
T0830TS023_1-D2.rsa	26.4	0	0.167	0.833	274	0.096	44.98
T0830TS097_4-D2.rsa	26.4	0.015	0.356	0.629	207	0.128	56.76
T0830TS050_1-D2.rsa	26.286	0.483	0.057	0.46	186	0.141	40.53
T0830TS156_2-D2.rsa	26.263	0.201	0.177	0.622	179	0.147	62.33
T0830TS430_2-D2.rsa	26.263	0.215	0.177	0.608	175	0.15	60.76
T0830TS169_2-D2.rsa	26.23	0.038	0.105	0.857	180	0.146	68.41

T0830TS038_4-D2.rsa	26	0.45	0.108	0.442	111	0.234	87.15
T0830TS442_1-D2.rsa	25.926	0.388	0.09	0.522	238	0.109	55.96
T0830TS216_4-D2.rsa	25.85	0.378	0.107	0.515	192	0.135	82.99
T0830TS445_1-D2.rsa	25.743	0.274	0.153	0.573	184	0.14	74.3
T0830TS277_5-D2.rsa	25.714	0.408	0.04	0.552	223	0.115	47.34
T0830TS296_5-D2.rsa	25.641	0.389	0.132	0.48	142	0.181	59.03
T0830TS442_2-D2.rsa	25.385	0.07	0.103	0.828	379	0.067	5.56
T0830TS041_4-D2.rsa	25.253	0.153	0.035	0.813	234	0.108	35.07
T0830TS483_2-D2.rsa	25.253	0.205	0.16	0.635	183	0.138	71.53
T0830TS008_1-D2.rsa	25.253	0.226	0.163	0.611	176	0.143	65.45
T0830TS044_4-D2.rsa	25.253	0.25	0.174	0.576	166	0.152	70.92
T0830TS044_5-D2.rsa	25.17	0.381	0.102	0.517	193	0.13	83.54
T0830TS044_1-D2.rsa	25.17	0.351	0.091	0.558	208	0.121	71.25
T0830TS049_2-D2.rsa	25.17	0.378	0.11	0.512	191	0.132	83.4
T0830TS251_2-D2.rsa	25	0.373	0.004	0.623	284	0.088	43.64
T0830TS160_2-D2.rsa	25	0.199	0.06	0.741	246	0.102	49.02
T0830TS338_3-D2.rsa	25	0.346	0.082	0.572	139	0.18	67.44
T0830TS310_5-D2.rsa	25	0.395	0.066	0.539	131	0.191	65.11
T0830TS326_2-D2.rsa	24.752	0.243	0.121	0.636	204	0.121	74.38
T0830TS276_3-D2.rsa	24.691	0.397	0.094	0.509	232	0.106	62.53
T0830TS097_2-D2.rsa	24.658	0.399	0.078	0.523	194	0.127	63.69
T0830TS184_5-D2.rsa	24.658	0.407	0.094	0.499	185	0.133	66.14
T0830TS155_3-D2.rsa	24.615	0.124	0.17	0.705	323	0.076	57.78
T0830TS425_4-D2.rsa	24.59	0	0.386	0.614	129	0.191	64.3
T0830TS268_4-D2.rsa	24.571	0.411	0.042	0.547	221	0.111	46.84
T0830TS251_1-D2.rsa	24.49	0.37	0.107	0.523	195	0.126	82.64
T0830TS169_1-D2.rsa	24.49	0.391	0.118	0.491	183	0.134	84.86
T0830TS080_4-D2.rsa	24.242	0.191	0.066	0.743	214	0.113	60.07
T0830TS064_5-D2.rsa	24.242	0.188	0.188	0.625	180	0.135	55.56
T0830TS228_5-D2.rsa	24.242	0.194	0.212	0.594	171	0.142	62.76
T0830TS237_4-D2.rsa	24.074	0.443	0.068	0.489	223	0.108	56.24
T0830TS132_5-D2.rsa	24	0.537	0.015	0.448	181	0.133	45.05
T0830TS333_5-D2.rsa	24	0.557	0.022	0.421	170	0.141	49.07
T0830TS345_1-D2.rsa	24	0.058	0.377	0.565	78	0.308	74.09
T0830TS011_3-D2.rsa	23.762	0.255	0.153	0.592	190	0.125	74.3
T0830TS391_4-D2.rsa	23.729	0.023	0.257	0.72	154	0.154	65.3
T0830TS340_1-D2.rsa	23.457	0.342	0.077	0.581	265	0.089	55.24
T0830TS157_3-D2.rsa	23.457	0.384	0.07	0.546	249	0.094	53.53
T0830TS276_4-D2.rsa	23.457	0.408	0.099	0.493	225	0.104	62.58
T0830TS044_2-D2.rsa	23.429	0.441	0.01	0.55	222	0.106	41.34
T0830TS347_4-D2.rsa	23.232	0.271	0.219	0.51	147	0.158	71.79
T0830TS117_4-D2.rsa	23.2	0.012	0.158	0.83	273	0.085	45.59
T0830TS133_3-D2.rsa	23.2	0.015	0.231	0.754	248	0.094	45.67
T0830TS338_5-D2.rsa	23.077	0.399	0.064	0.537	159	0.145	56.94
T0830TS300_1-D2.rsa	23.026	0.408	0.02	0.572	261	0.088	61.73
T0830TS116_5-D2.rsa	23.026	0.439	0.024	0.537	245	0.094	63.32
T0830TS263_4-D2.rsa	23.026	0.439	0.015	0.546	249	0.092	61.62

T0830TS042_5-D2.rsa	22.857	0.455	0.02	0.525	212	0.108	38.24
T0830TS216_5-D2.rsa	22.4	0.024	0.164	0.812	267	0.084	45.52
T0830TS204_1-D2.rsa	22.4	0.012	0.395	0.593	195	0.115	20.67
T0830TS276_1-D2.rsa	22.368	0.211	0.09	0.699	232	0.096	49.7
T0830TS118_2-D2.rsa	22.368	0.196	0.072	0.732	243	0.092	33.51
T0830TS391_1-D2.rsa	22.286	0.502	0	0.498	201	0.111	44.86
T0830TS067_4-D2.rsa	22.222	0.382	0.09	0.529	241	0.092	52.98
T0830TS184_1-D2.rsa	22.222	0.368	0.081	0.55	251	0.089	48.18
T0830TS499_5-D2.rsa	22.222	0.434	0.083	0.482	220	0.101	65.84
T0830TS479_1-D2.rsa	22.093	0.346	0.124	0.53	150	0.147	70.32
T0830TS228_4-D2.rsa	22.093	0.403	0.124	0.473	134	0.165	76.15
T0830TS454_2-D2.rsa	22.059	0.305	0.033	0.663	161	0.137	69.33
T0830TS132_3-D2.rsa	22.034	0	0.313	0.687	147	0.15	64.49
T0830TS038_2-D2.rsa	21.795	0.304	0.101	0.595	176	0.124	53.3
T0830TS445_4-D2.rsa	21.782	0.249	0.14	0.611	196	0.111	74.14
T0830TS042_4-D2.rsa	21.714	0.433	0.02	0.547	221	0.098	45.11
T0830TS358_1-D2.rsa	21.714	0.54	0.005	0.455	184	0.118	44.86
T0830TS011_1-D2.rsa	21.143	0.436	0.032	0.532	215	0.098	39.42
T0830TS011_2-D2.rsa	21.143	0.463	0.015	0.522	211	0.1	38.8
T0830TS346_1-D2.rsa	21.053	0.43	0.026	0.544	248	0.085	65.24
T0830TS282_2-D2.rsa	21.053	0.434	0.024	0.542	247	0.085	57.18
T0830TS128_2-D2.rsa	21.053	0.202	0.048	0.75	249	0.085	50
T0830TS448_1-D2.rsa	21.053	0.487	0.015	0.498	227	0.093	61.84
T0830TS448_5-D2.rsa	21.053	0.485	0.029	0.487	222	0.095	64.8
T0830TS322_1-D2.rsa	21.053	0.214	0.06	0.726	241	0.087	40.44
T0830TS364_1-D2.rsa	21.053	0.343	0.142	0.515	171	0.123	64.08
T0830TS049_5-D2.rsa	21.053	0.461	0.091	0.447	98	0.215	79
T0830TS442_4-D2.rsa	20.988	0.445	0.09	0.465	212	0.099	74.83
T0830TS296_2-D2.rsa	20.93	0.385	0.124	0.491	139	0.151	77.21
T0830TS358_3-D2.rsa	20.779	0.388	0.138	0.473	106	0.196	81.67
T0830TS368_2-D2.rsa	20.571	0.428	0	0.572	231	0.089	46.53
T0830TS235_2-D2.rsa	20.395	0.487	0.022	0.491	224	0.091	62.17
T0830TS499_3-D2.rsa	19.802	0.218	0.146	0.636	204	0.097	73.36
T0830TS144_4-D2.rsa	19.767	0.399	0.092	0.509	144	0.137	77.74
T0830TS290_3-D2.rsa	19.767	0.382	0.117	0.502	142	0.139	69.26
T0830TS153_4-D2.rsa	19.753	0.414	0.061	0.524	239	0.083	66
T0830TS023_2-D2.rsa	19.753	0.414	0.086	0.5	228	0.087	70.53
T0830TS157_1-D2.rsa	19.753	0.436	0.086	0.478	218	0.091	52.43
T0830TS235_5-D2.rsa	19.737	0.447	0.004	0.548	250	0.079	62.72
T0830TS263_5-D2.rsa	19.737	0.434	0.024	0.542	247	0.08	59.92
T0830TS008_5-D2.rsa	19.737	0.436	0.024	0.539	246	0.08	63.05
T0830TS133_4-D2.rsa	19.737	0.443	0.024	0.533	243	0.081	62.45
T0830TS360_4-D2.rsa	19.737	0.13	0.09	0.78	259	0.076	43.98
T0830TS268_5-D2.rsa	19.136	0.421	0.072	0.507	231	0.083	72.68
T0830TS276_2-D2.rsa	19.079	0.474	0.024	0.502	229	0.083	64.91
T0830TS228_3-D2.rsa	19.079	0.445	0.033	0.522	238	0.08	61.62
T0830TS360_1-D2.rsa	18.857	0.478	0	0.522	211	0.089	46.91

T0830TS360_3-D2.rsa	18.812	0.262	0.1	0.639	205	0.092	68.3
T0830TS145_5-D2.rsa	18.75	0.096	0.25	0.654	89	0.211	16.54
T0830TS282_4-D2.rsa	18.644	0.014	0.262	0.724	155	0.12	62.97
T0830TS479_4-D2.rsa	18.605	0.367	0.12	0.512	145	0.128	79.15
T0830TS263_2-D2.rsa	18.519	0.386	0.072	0.542	247	0.075	57.51
T0830TS322_3-D2.rsa	18.519	0.423	0.092	0.485	221	0.084	66.83
T0830TS322_5-D2.rsa	18.421	0.421	0.007	0.572	261	0.071	58.5
T0830TS133_2-D2.rsa	18.421	0.434	0.004	0.561	256	0.072	53.62
T0830TS133_5-D2.rsa	18.421	0.404	0.022	0.575	262	0.07	50.55
T0830TS268_3-D2.rsa	18.421	0.328	0.117	0.554	184	0.1	95.11
T0830TS237_5-D2.rsa	18.421	0.316	0.078	0.605	201	0.092	69.43
T0830TS171_4-D2.rsa	17.901	0.36	0.083	0.557	254	0.07	63.19
T0830TS328_4_2-D2.rsa	17.442	0.413	0.124	0.463	131	0.133	82.95
T0830TS403_3-D2.rsa	17.284	0.375	0.077	0.548	250	0.069	59.27
T0830TS333_1-D2.rsa	17.105	0.268	0.093	0.639	212	0.081	58.58
T0830TS403_1-D2.rsa	16.447	0.439	0.033	0.529	241	0.068	65.08
T0830TS162_1_2-D2.rsa	16.279	0.389	0.117	0.495	140	0.116	81.8
T0830TS116_3-D2.rsa	16.154	0.266	0.207	0.526	241	0.067	64.67
T0830TS228_1-D2.rsa	16.154	0.255	0.236	0.509	233	0.069	70.44
T0830TS410_3-D2.rsa	15.789	0.28	0.081	0.639	212	0.074	96.23
T0830TS008_4-D2.rsa	15.789	0.334	0.114	0.551	183	0.086	98.12
T0830TS482_1-D2.rsa	15.789	0.22	0.111	0.669	222	0.071	47.29
T0830TS439_1-D2.rsa	15.789	0.34	0.117	0.542	180	0.088	94.5
T0830TS153_5-D2.rsa	15.789	0.34	0.123	0.536	178	0.089	99.1
T0830TS197_4-D2.rsa	15.789	0.316	0.123	0.56	186	0.085	93.52
T0830TS197_1-D2.rsa	15.789	0.346	0.139	0.515	171	0.092	98.42
T0830TS483_1-D2.rsa	15.789	0.349	0.142	0.509	169	0.093	98.95
T0830TS279_1-D2.rsa	15.385	0.255	0.205	0.539	247	0.062	71.22
T0830TS442_3-D2.rsa	15.385	0.234	0.218	0.548	251	0.061	73.17
T0830TS326_3-D2.rsa	15.385	0.255	0.242	0.502	230	0.067	70.94
T0830TS277_2-D2.rsa	15.385	0.262	0.242	0.496	227	0.068	72.06
T0830TS067_5-D2.rsa	15.385	0.275	0.223	0.502	230	0.067	71.83
T0830TS317_2_2-D2.rsa	15.116	0.392	0.095	0.512	145	0.104	73.41
T0830TS054_3-D2.rsa	14.894	0.253	0.043	0.704	181	0.082	49.12
T0830TS235_3-D2.rsa	14.615	0.258	0.238	0.504	231	0.063	78.33
T0830TS403_2-D2.rsa	14.615	0.264	0.231	0.504	231	0.063	76
T0830TS056_1-D2.rsa	14.615	0.271	0.225	0.504	231	0.063	71.67
T0830TS251_3-D2.rsa	14.615	0.255	0.247	0.498	228	0.064	71.11
T0830TS290_2-D2.rsa	14.615	0.286	0.234	0.48	220	0.066	70.5
T0830TS133_1-D2.rsa	14.474	0.423	0.015	0.561	256	0.057	58.28
T0830TS439_2-D2.rsa	14.474	0.463	0.02	0.518	236	0.061	60.03
T0830TS041_3-D2.rsa	14.474	0.316	0.123	0.56	186	0.078	99.32
T0830TS237_1-D2.rsa	14.474	0.34	0.123	0.536	178	0.081	98.12
T0830TS153_2-D2.rsa	14.474	0.331	0.133	0.536	178	0.081	99.1
T0830TS479_5-D2.rsa	14.474	0.352	0.148	0.5	166	0.087	99.32
T0830TS204_5-D2.rsa	14.474	0.262	0.015	0.723	240	0.06	9.86
T0830TS118_4-D2.rsa	13.953	0.399	0.141	0.459	130	0.107	71.47

T0830TS063_4-D2.rsa	13.846	0.253	0.17	0.576	264	0.052	70.22
T0830TS442_5-D2.rsa	13.846	0.255	0.245	0.5	229	0.06	74.06
T0830TS169_5-D2.rsa	13.158	0.289	0.108	0.602	200	0.066	96.23
T0830TS322_4-D2.rsa	13.158	0.307	0.127	0.566	188	0.07	92.85
T0830TS080_3-D2.rsa	13.158	0.343	0.123	0.533	177	0.074	98.95
T0830TS156_3-D2.rsa	13.158	0.352	0.123	0.524	174	0.076	99.25
T0830TS210_2-D2.rsa	13.158	0.352	0.123	0.524	174	0.076	97.74
T0830TS210_5-D2.rsa	13.077	0.284	0.177	0.539	247	0.053	71.28
T0830TS347_5-D2.rsa	12.791	0.375	0.124	0.502	142	0.09	80.92
T0830TS460_1-D2.rsa	12.766	0.323	0.183	0.494	127	0.101	86.19
T0830TS338_1-D2.rsa	12.766	0.358	0.179	0.463	119	0.107	83.27
T0830TS128_1-D2.rsa	12.308	0.297	0.133	0.57	261	0.047	72.61
T0830TS008_2-D2.rsa	11.842	0.235	0.09	0.675	224	0.053	55.8
T0830TS042_1-D2.rsa	11.842	0.337	0.13	0.533	177	0.067	98.72
T0830TS360_5-D2.rsa	11.842	0.271	0.136	0.593	197	0.06	84.19
T0830TS067_2-D2.rsa	10.638	0.296	0.148	0.556	143	0.074	85.12
T0830TS216_2-D2.rsa	10.638	0.335	0.163	0.502	129	0.082	81.81
T0830TS410_4-D2.rsa	9.211	0.271	0.069	0.66	219	0.042	92.02
T0830TS132_1-D2.rsa	8.511	0.335	0.183	0.482	124	0.069	82.59
T0807TS073_2-D1.rsa	79.412	0.27	0	0.73	92	0.863	28.57
T0807TS011_1-D1.rsa	76.316	0.27	0.054	0.676	75	1.018	16.67
T0807TS038_4-D1.rsa	71.429	0.275	0.053	0.672	88	0.812	12.79
T0807TS008_1-D1.rsa	68.421	0.144	0.072	0.784	87	0.786	24.55
T0807TS381_5-D1.rsa	67.857	0	0.321	0.679	89	0.762	16.98
T0807TS210_4-D1.rsa	63.158	0.054	0	0.946	105	0.602	15.31
T0807TS212_1-D1.rsa	62.5	0.222	0.151	0.627	79	0.791	24.6
T0807TS216_3-D1.rsa	62.5	0.019	0.546	0.435	47	1.33	71.06
T0807TS117_1-D1.rsa	62.5	0.27	0.016	0.714	90	0.694	17.46
T0807TS008_4-D1.rsa	62.5	0	0.593	0.407	44	1.42	71.3
T0807TS410_2-D1.rsa	61.765	0.548	0	0.452	57	1.084	36.11
T0807TS414_5-D1.rsa	60.714	0	0.298	0.702	92	0.66	13.74
T0807TS184_5-D1.rsa	60.526	0.18	0.027	0.793	88	0.688	17.34
T0807TS436_1-D1.rsa	60.465	0.246	0	0.754	101	0.599	42.72
T0807TS050_1-D1.rsa	60	0.491	0.094	0.415	97	0.619	10.15
T0807TS117_5-D1.rsa	59.211	0.322	0.018	0.66	219	0.27	10.39
T0807TS499_3-D1.rsa	58.929	0.111	0	0.889	112	0.526	15.28
T0807TS133_1-D1.rsa	58.333	0	0.509	0.491	53	1.101	68.29
T0807TS345_2-D1.rsa	58.333	0	0.611	0.389	42	1.389	68.29
T0807TS145_5-D1.rsa	57.746	0.351	0.048	0.601	101	0.572	20.67
T0807TS349_3-D1.rsa	56.338	0.351	0.048	0.601	101	0.558	22.76
T0807TS171_3-D1.rsa	55.882	0.556	0	0.444	56	0.998	49.21
T0807TS237_5-D1.rsa	55.769	0.267	0.036	0.697	115	0.485	28.48
T0807TS171_2-D1.rsa	54.93	0.417	0	0.583	98	0.561	18.11
T0807TS268_2-D1.rsa	54.167	0.018	0.336	0.645	71	0.763	45
T0807TS414_4-D1.rsa	54.167	0	0.593	0.407	44	1.231	71.76
T0807TS492_3-D1.rsa	53.571	0.015	0	0.985	129	0.415	12.4
T0807TS410_4-D1.rsa	53.488	0.336	0.052	0.612	82	0.652	45.9

T0807TS210_2-D1.rsa	50	0	0.175	0.825	94	0.532	39.91
T0807TS210_1-D1.rsa	50	0	0.4	0.6	66	0.758	38.64
T0807TS492_4-D1.rsa	48.837	0.313	0.015	0.672	90	0.543	33.4
T0807TS038_2-D1.rsa	48.214	0.175	0.302	0.524	66	0.731	52.58
T0807TS184_2-D1.rsa	48	0.08	0.29	0.63	87	0.552	35.87
T0807TS335_1-D1.rsa	48	0.072	0.341	0.587	81	0.593	36.05
T0807TS228_5-D1.rsa	47.887	0.119	0	0.881	148	0.324	12.82
T0807TS216_1-D1.rsa	47.887	0.304	0.137	0.56	94	0.509	14.42
T0807TS479_4-D1.rsa	47.5	0.035	0.158	0.807	92	0.516	32.46
T0807TS454_4-D1.rsa	47.5	0.018	0.342	0.64	73	0.651	37.06
T0807TS335_4-D1.rsa	47.368	0.09	0	0.91	101	0.469	22.97
T0807TS420_1-D1.rsa	47.368	0.081	0.081	0.838	93	0.509	19.59
T0807TS216_2-D1.rsa	47.368	0.234	0.09	0.676	75	0.632	37.61
T0807TS073_1-D1.rsa	46.512	0.313	0	0.687	92	0.506	34.89
T0807TS011_2-D1.rsa	46.429	0	0.397	0.603	79	0.588	17.18
T0807TS171_5-D1.rsa	45.833	0	0.436	0.564	62	0.739	59.09
T0807TS210_5-D1.rsa	45	0	0.316	0.684	78	0.577	21.93
T0807TS206_1-D1.rsa	44.737	0.207	0.162	0.631	70	0.639	31.08
T0807TS279_1-D1.rsa	43.836	0.663	0	0.337	86	0.51	40.88
T0807TS041_1-D1.rsa	43.75	0.073	0.391	0.536	59	0.742	56.82
T0807TS011_4-D1.rsa	43.75	0	0.445	0.555	61	0.717	49.32
T0807TS381_4-D1.rsa	43.662	0	0.327	0.673	113	0.386	12.66
T0807TS156_2-D1.rsa	42.857	0	0.29	0.71	93	0.461	60.69
T0807TS335_5-D1.rsa	42.857	0.046	0.015	0.939	123	0.348	12.98
T0807TS268_5-D1.rsa	42.857	0	0.443	0.557	73	0.587	57.82
T0807TS008_3-D1.rsa	42.857	0	0.244	0.756	99	0.433	17.37
T0807TS414_2-D1.rsa	42.857	0.087	0.079	0.833	105	0.408	14.68
T0807TS381_3-D1.rsa	42.5	0.018	0.123	0.86	98	0.434	20.61
T0807TS277_5-D1.rsa	42.188	0.096	0	0.904	123	0.343	14.34
T0807TS349_1-D1.rsa	41.86	0.328	0.015	0.657	88	0.476	47.02
T0807TS268_1-D1.rsa	41.667	0.027	0.373	0.6	66	0.631	62.5
T0807TS210_3-D1.rsa	40.625	0.015	0.456	0.529	72	0.564	70.04
T0807TS349_2-D1.rsa	40	0.035	0.079	0.886	101	0.396	32.67
T0807TS479_1-D1.rsa	40	0.094	0.159	0.746	103	0.388	26.99
T0807TS420_2-D1.rsa	39.474	0.234	0	0.766	85	0.464	38.74
T0807TS479_5-D1.rsa	39.286	0	0.328	0.672	88	0.446	50
T0807TS160_2-D1.rsa	39.063	0.015	0.434	0.551	75	0.521	67.65
T0807TS133_4-D1.rsa	39.063	0.015	0.449	0.537	73	0.535	71.14
T0807TS251_1-D1.rsa	38.889	0.399	0.094	0.507	108	0.36	69.54
T0807TS420_5-D1.rsa	38.793	0.625	0	0.375	96	0.404	41.7
T0807TS452_3-D1.rsa	38.028	0.429	0.071	0.5	84	0.453	33.01
T0807TS499_4-D1.rsa	37.895	0.513	0.059	0.428	101	0.375	50.42
T0807TS216_5-D1.rsa	37.5	0.191	0	0.809	110	0.341	15.99
T0807TS345_4-D1.rsa	37.209	0.231	0.045	0.724	97	0.384	39.74
T0807TS263_1-D1.rsa	37.209	0.396	0.06	0.545	73	0.51	41.42
T0807TS452_4-D1.rsa	36.986	0.718	0	0.282	72	0.514	48.92
T0807TS492_5-D1.rsa	36.538	0.188	0.061	0.752	124	0.295	15.82

T0807TS041_3-D1.rsa	36.047	0.349	0.154	0.497	84	0.429	57.54
T0807TS228_2-D1.rsa	36	0.072	0.254	0.674	93	0.387	23.55
T0807TS133_3-D1.rsa	35.714	0	0.244	0.756	99	0.361	49.62
T0807TS216_4-D1.rsa	35.616	0.667	0	0.333	85	0.419	59.61
T0807TS160_1-D1.rsa	35.417	0	0.145	0.855	94	0.377	51.14
T0807TS038_5-D1.rsa	35	0.088	0.053	0.86	98	0.357	38.16
T0807TS335_3-D1.rsa	34.884	0.194	0.097	0.709	95	0.367	34.89
T0807TS345_3-D1.rsa	34.737	0.534	0.059	0.407	96	0.362	63.56
T0807TS414_3-D1.rsa	34.722	0.502	0.089	0.408	87	0.399	55.2
T0807TS414_1-D1.rsa	34.615	0.273	0.048	0.679	112	0.309	18.99
T0807TS237_2-D1.rsa	34.483	0.613	0	0.387	99	0.348	55.18
T0807TS479_3-D1.rsa	34.375	0.015	0.39	0.596	81	0.424	74.63
T0807TS160_4-D1.rsa	34.247	0.745	0	0.255	65	0.527	60.1
T0807TS184_1-D1.rsa	33.803	0.512	0.048	0.44	74	0.457	51.92
T0807TS145_4-D1.rsa	33.721	0.249	0.142	0.609	103	0.327	47.04
T0807TS251_2-D1.rsa	33.621	0.629	0	0.371	95	0.354	53.61
T0807TS420_4-D1.rsa	33.621	0.68	0	0.32	82	0.41	56.15
T0807TS346_1-D1.rsa	32.759	0.637	0	0.363	93	0.352	57.13
T0807TS160_3-D1.rsa	32.632	0.534	0.059	0.407	96	0.34	62.82
T0807TS454_1-D1.rsa	32.5	0	0.447	0.553	63	0.516	36.4
T0807TS436_3-D1.rsa	32.353	0.342	0.021	0.638	155	0.209	12.22
T0807TS300_5-D1.rsa	31.897	0.641	0	0.359	92	0.347	60.94
T0807TS277_3-D1.rsa	31.579	0.508	0.047	0.445	105	0.301	61.23
T0807TS410_3-D1.rsa	31.579	0.555	0.055	0.39	92	0.343	63.56
T0807TS156_1-D1.rsa	31.579	0.259	0.472	0.269	29	1.089	94.21
T0807TS452_2-D1.rsa	31.507	0.749	0	0.251	64	0.492	66.57
T0807TS251_3-D1.rsa	31.429	0.355	0.205	0.44	103	0.305	70.62
T0807TS133_2-D1.rsa	31.25	0.074	0.309	0.618	84	0.372	64.89
T0807TS277_4-D1.rsa	30.233	0.314	0.16	0.527	89	0.34	71.75
T0807TS041_2-D1.rsa	30.233	0.276	0	0.724	97	0.312	39.74
T0807TS436_5-D1.rsa	30	0.422	0.12	0.458	115	0.261	85.66
T0807TS145_2-D1.rsa	30	0.079	0.026	0.895	102	0.294	35.97
T0807TS492_2-D1.rsa	29.825	0.3	0.161	0.539	117	0.255	34.91
T0807TS499_2-D1.rsa	29.524	0.295	0.192	0.513	120	0.246	66.77
T0807TS117_4-D1.rsa	29.524	0.444	0.209	0.346	81	0.364	70.3
T0807TS300_3-D1.rsa	29.412	0.333	0.045	0.621	151	0.195	18.89
T0807TS160_5-D1.rsa	29.31	0.645	0	0.355	91	0.322	58.2
T0807TS349_4-D1.rsa	29.167	0.39	0.075	0.535	114	0.256	65.99
T0807TS300_2-D1.rsa	29.07	0.32	0.207	0.473	80	0.363	71.3
T0807TS300_1-D1.rsa	28.571	0	0.229	0.771	101	0.283	55.73
T0807TS479_2-D1.rsa	28.421	0.513	0.055	0.432	102	0.279	61.12
T0807TS156_3-D1.rsa	28.421	0.538	0.064	0.398	94	0.302	50.74
T0807TS184_3-D1.rsa	28.125	0.088	0	0.912	124	0.227	13.42
T0807TS171_1-D1.rsa	28	0.072	0.333	0.594	82	0.341	67.03
T0807TS237_1-D1.rsa	28	0.043	0.283	0.674	93	0.301	56.88
T0807TS133_5-D1.rsa	28	0.051	0.283	0.667	92	0.304	56.88
T0807TS345_5-D1.rsa	28	0.036	0.362	0.601	83	0.337	66.49

T0807TS145_3-D1.rsa	28	0.094	0.362	0.543	75	0.373	66.49
T0807TS041_5-D1.rsa	27.907	0.278	0.16	0.562	95	0.294	48.82
T0807TS454_2-D1.rsa	27.5	0	0.202	0.798	91	0.302	39.03
T0807TS349_5-D1.rsa	26.744	0.325	0.16	0.515	87	0.307	73.52
T0807TS454_5-D1.rsa	26.744	0.32	0.195	0.485	82	0.326	70.56
T0807TS499_5-D1.rsa	26.667	0.338	0.201	0.462	108	0.247	71.15
T0807TS420_3-D1.rsa	25.581	0.349	0.172	0.479	81	0.316	74.41
T0807TS499_1-D1.rsa	25	0.366	0.099	0.535	130	0.192	67.22
T0807TS452_5-D1.rsa	25	0.061	0.018	0.921	105	0.238	19.96
T0807TS251_4-D1.rsa	24	0.094	0.377	0.529	73	0.329	68.48
T0807TS008_2-D1.rsa	24	0.109	0.239	0.652	90	0.267	64.67
T0807TS492_1-D1.rsa	22.857	0.385	0.197	0.419	98	0.233	69.12
T0807TS228_3-D1.rsa	22.807	0.47	0.091	0.438	96	0.238	78.88
T0807TS436_4-D1.rsa	22	0.406	0.12	0.474	119	0.185	90.64
T0807TS277_1-D1.rsa	20	0.014	0.406	0.58	80	0.25	75.91
T0807TS300_4-D1.rsa	20	0.058	0.406	0.536	74	0.27	73.19
T0807TS228_4-D1.rsa	19.231	0.416	0.128	0.456	135	0.142	58.42
T0807TS117_2-D1.rsa	18.462	0.262	0.225	0.513	235	0.079	74.17
T0807TS237_3-D1.rsa	14.035	0.393	0.055	0.553	121	0.116	69.52
T0807TS436_2-D1.rsa	12.766	0.346	0.183	0.471	121	0.106	82.3
T0807TS228_1-D1.rsa	12.281	0.452	0.105	0.443	97	0.127	81.85
T0807TS171_4-D1.rsa	12.281	0.452	0.123	0.425	93	0.132	79.57
T0827TS276_1-D1.rsa	86.047	0.657	0.03	0.313	42	2.049	14.18
T0827TS333_5-D1.rsa	81.395	0.537	0.112	0.351	47	1.732	22.02
T0827TS368_3-D1.rsa	78.947	0.486	0	0.514	57	1.385	15.31
T0827TS228_3-D1.rsa	76.563	0.426	0.11	0.463	63	1.215	13.23
T0827TS414_5-D1.rsa	76.316	0.532	0	0.468	52	1.468	19.59
T0827TS452_5-D1.rsa	73.684	0.477	0	0.523	58	1.27	16.22
T0827TS263_3-D1.rsa	73.214	0.579	0.048	0.373	47	1.558	16.67
T0827TS310_1-D1.rsa	71.429	0.492	0	0.508	64	1.116	13.49
T0827TS204_3-D1.rsa	71.053	0.486	0	0.514	57	1.247	17.12
T0827TS216_3-D1.rsa	70	0.412	0	0.588	67	1.045	14.04
T0827TS268_3-D1.rsa	69.643	0.492	0.048	0.46	58	1.201	19.44
T0827TS197_4-D1.rsa	68.421	0.396	0.072	0.532	59	1.16	13.96
T0827TS032_2-D1.rsa	67.857	0.053	0.26	0.687	90	0.754	19.08
T0827TS454_5-D1.rsa	65.789	0.73	0	0.27	30	2.193	15.77
T0827TS457_5-D1.rsa	65.625	0.5	0.015	0.485	66	0.994	10.29
T0827TS212_4-D1.rsa	65.385	0.297	0.024	0.679	112	0.584	19.3
T0827TS335_1-D1.rsa	64.706	0.651	0	0.349	44	1.471	30.95
T0827TS445_2-D1.rsa	63.462	0.648	0	0.352	58	1.094	14.87
T0827TS067_5-D1.rsa	62.5	0	0.519	0.481	52	1.202	22.45
T0827TS326_1-D1.rsa	61.765	0.548	0	0.452	57	1.084	34.52
T0827TS212_1-D1.rsa	61.765	0.524	0	0.476	60	1.029	29.17
T0827TS457_3-D1.rsa	61.765	0.524	0	0.476	60	1.029	29.17
T0827TS210_1-D1.rsa	61.765	0.833	0	0.167	21	2.941	20.64
T0827TS024_1_1-D1.rsa	60.714	0.015	0.42	0.565	74	0.82	57.06
T0827TS212_2-D1.rsa	60.714	0.015	0.42	0.565	74	0.82	56.87

T0827TS157_2-D1.rsa	60.526	0.171	0	0.829	92	0.658	24.77
T0827TS241_1-D1.rsa	60.526	0.288	0.054	0.658	73	0.829	15.54
T0827TS216_4-D1.rsa	58.824	0.444	0	0.556	70	0.84	20.04
T0827TS483_5-D1.rsa	58.333	0	0.227	0.773	85	0.686	18.64
T0827TS008_2-D1.rsa	58.333	0	0.556	0.444	48	1.215	55.56
T0827TS022_3-D1.rsa	57.895	0.424	0	0.576	136	0.426	12.5
T0827TS442_1-D1.rsa	57.813	0.162	0.324	0.515	70	0.826	12.87
T0827TS328_3-D1.rsa	57.143	0.294	0.222	0.484	61	0.937	22.42
T0827TS235_3-D1.rsa	55.769	0.297	0.115	0.588	97	0.575	77.06
T0827TS118_5-D1.rsa	55	0	0.447	0.553	63	0.873	18.64
T0827TS063_2-D1.rsa	54.167	0	0.62	0.38	41	1.321	69.91
T0827TS333_2-D1.rsa	53.571	0.443	0.168	0.389	51	1.05	14.12
T0827TS097_1-D1.rsa	53.488	0.313	0.104	0.582	78	0.686	17.91
T0827TS204_2-D1.rsa	53.488	0.343	0.209	0.448	60	0.891	23.13
T0827TS268_2-D1.rsa	53.125	0.368	0	0.632	86	0.618	10.48
T0827TS338_3-D1.rsa	52.941	0.698	0	0.302	38	1.393	35.12
T0827TS437_1-D1.rsa	52.632	0.225	0.081	0.694	77	0.684	20.72
T0827TS153_2-D1.rsa	52.632	0.432	0	0.568	63	0.835	20.5
T0827TS360_4-D1.rsa	52.632	0.243	0.18	0.577	64	0.822	19.59
T0827TS044_1-D1.rsa	52.5	0.053	0.465	0.482	55	0.955	19.08
T0827TS479_1-D1.rsa	52	0.312	0.167	0.522	72	0.722	12.5
T0827TS263_4-D1.rsa	51.923	0.388	0.079	0.533	88	0.59	18.2
T0827TS156_5-D1.rsa	51.163	0.231	0.09	0.679	91	0.562	18.28
T0827TS442_5-D1.rsa	50	0.378	0.09	0.532	59	0.847	29.05
T0827TS445_4-D1.rsa	50	0.579	0	0.421	53	0.943	32.34
T0827TS452_4-D1.rsa	50	0.714	0	0.286	36	1.389	55.56
T0827TS340_4-D1.rsa	50	0.018	0.518	0.465	53	0.943	27.19
T0827TS277_5-D1.rsa	50	0.413	0.111	0.476	60	0.833	17.66
T0827TS425_3-D1.rsa	50	0	0.518	0.482	55	0.909	17.11
T0827TS155_3-D1.rsa	49.296	0.464	0.107	0.429	72	0.685	21.31
T0827TS042_1-D1.rsa	48.837	0.478	0.03	0.493	66	0.74	28.73
T0827TS300_3-D1.rsa	48.438	0.081	0.287	0.632	86	0.563	12.32
T0827TS008_4-D1.rsa	48.214	0.175	0.254	0.571	72	0.67	23.21
T0827TS438_1-D1.rsa	48.214	0.198	0.238	0.563	71	0.679	22.82
T0827TS368_2-D1.rsa	48.214	0.214	0.222	0.563	71	0.679	25.2
T0827TS479_2-D1.rsa	48.214	0.405	0.063	0.532	67	0.72	19.44
T0827TS345_3-D1.rsa	48.214	0.167	0.421	0.413	52	0.927	40.87
T0827TS197_3-D1.rsa	48.214	0.294	0.23	0.476	60	0.804	23.61
T0827TS452_1-D1.rsa	47.917	0	0.536	0.464	51	0.94	38.64
T0827TS425_4-D1.rsa	47.917	0.327	0.018	0.655	72	0.666	21.59
T0827TS410_2-D1.rsa	47.887	0.506	0.048	0.446	75	0.638	20.03
T0827TS358_4-D1.rsa	47.5	0	0.412	0.588	67	0.709	17.98
T0827TS210_4-D1.rsa	47.5	0.018	0.526	0.456	52	0.913	23.25
T0827TS300_1-D1.rsa	47.059	0.698	0	0.302	38	1.238	42.26
T0827TS452_3-D1.rsa	47.059	0.571	0	0.429	54	0.871	32.54
T0827TS268_1-D1.rsa	46.512	0.291	0.269	0.44	59	0.788	22.76
T0827TS169_3-D1.rsa	46.479	0.476	0.101	0.423	71	0.655	21.47

T0827TS479_5-D1.rsa	46.429	0.452	0.016	0.532	67	0.693	22.22
T0827TS296_5-D1.rsa	46.429	0.349	0.127	0.524	66	0.703	17.46
T0827TS333_4-D1.rsa	46.429	0.294	0.079	0.627	79	0.588	19.44
T0827TS346_1-D1.rsa	46.154	0.255	0.097	0.648	107	0.431	43.51
T0827TS439_2-D1.rsa	45.313	0.096	0.368	0.537	73	0.621	17.1
T0827TS184_2-D1.rsa	45.313	0.154	0.331	0.515	70	0.647	13.05
T0827TS492_2-D1.rsa	45.205	0.761	0	0.239	61	0.741	36.86
T0827TS317_3-D1.rsa	45	0.018	0.465	0.518	59	0.763	27.85
T0827TS290_1-D1.rsa	45	0.018	0.342	0.64	73	0.616	16.67
T0827TS011_2-D1.rsa	45	0	0.351	0.649	74	0.608	17.76
T0827TS097_3-D1.rsa	45	0.018	0.342	0.64	73	0.616	17.98
T0827TS216_1-D1.rsa	45	0	0.404	0.596	68	0.662	20.39
T0827TS368_4-D1.rsa	44.828	0	0.063	0.937	134	0.335	44.58
T0827TS156_3-D1.rsa	44.737	0.259	0.37	0.37	40	1.118	62.96
T0827TS364_1-D1.rsa	44.737	0.231	0.426	0.343	37	1.209	65.28
T0827TS184_4-D1.rsa	44.737	0.269	0.389	0.343	37	1.209	65.74
T0827TS263_1-D1.rsa	44.737	0.222	0.454	0.324	35	1.278	59.72
T0827TS454_1-D1.rsa	44.737	0.278	0.454	0.269	29	1.543	44.91
T0827TS251_2-D1.rsa	44.643	0.159	0.27	0.571	72	0.62	22.02
T0827TS340_1-D1.rsa	44.118	0.556	0	0.444	56	0.788	50.79
T0827TS452_2-D1.rsa	44.118	0.659	0	0.341	43	1.026	37.1
T0827TS362_3-D1.rsa	44	0.196	0.268	0.536	74	0.595	13.77
T0827TS310_4-D1.rsa	43.75	0.059	0.346	0.596	81	0.54	12.5
T0827TS133_4-D1.rsa	43.75	0.074	0.287	0.64	87	0.503	13.05
T0827TS116_4-D1.rsa	42.857	0.103	0.159	0.738	93	0.461	15.68
T0827TS391_2-D1.rsa	42.857	0.079	0.365	0.556	70	0.612	37.5
T0827TS133_1-D1.rsa	42.857	0	0.313	0.687	90	0.476	13.55
T0827TS268_4-D1.rsa	42.5	0	0.351	0.649	74	0.574	18.86
T0827TS368_5-D1.rsa	42.5	0.018	0.482	0.5	57	0.746	29.61
T0827TS290_4-D1.rsa	42.5	0	0.518	0.482	55	0.773	16.67
T0827TS349_4-D1.rsa	42.254	0.5	0.107	0.393	66	0.64	39.1
T0827TS454_2-D1.rsa	42.254	0.464	0.119	0.417	70	0.604	21.47
T0827TS116_3-D1.rsa	42.105	0.279	0.018	0.703	78	0.54	33.33
T0827TS097_4-D1.rsa	42.105	0.252	0.162	0.586	65	0.648	34.23
T0827TS067_1-D1.rsa	42.105	0.297	0.144	0.559	62	0.679	35.59
T0827TS414_2-D1.rsa	41.176	0.651	0	0.349	44	0.936	36.51
T0827TS439_5-D1.rsa	41.096	0.698	0	0.302	77	0.534	36.67
T0827TS169_4-D1.rsa	41.096	0.757	0	0.243	62	0.663	36.86
T0827TS276_5-D1.rsa	41.071	0.389	0.056	0.556	70	0.587	19.84
T0827TS381_5-D1.rsa	40.845	0.446	0.125	0.429	72	0.567	23.4
T0827TS251_4-D1.rsa	40.845	0.482	0.125	0.393	66	0.619	31.57
T0827TS065_1-D1.rsa	40.385	0.715	0	0.285	47	0.859	14.24
T0827TS160_3-D1.rsa	40	0	0.316	0.684	78	0.513	19.08
T0827TS368_1-D1.rsa	40	0	0.447	0.553	63	0.635	27.85
T0827TS439_1-D1.rsa	40	0.035	0.368	0.596	68	0.588	22.59
T0827TS358_3-D1.rsa	40	0	0.342	0.658	75	0.533	19.96
T0827TS153_3-D1.rsa	40	0	0.43	0.57	65	0.615	26.32

T0827TS155_1-D1.rsa	40	0	0.412	0.588	67	0.597	21.05
T0827TS457_1-D1.rsa	40	0	0.43	0.57	65	0.615	26.54
T0827TS360_2-D1.rsa	40	0.018	0.404	0.579	66	0.606	17.98
T0827TS067_2-D1.rsa	40	0.268	0.123	0.609	84	0.476	15.04
T0827TS277_4-D1.rsa	40	0	0.342	0.658	75	0.533	14.25
T0827TS162_4-D1.rsa	39.726	0.761	0	0.239	61	0.651	41.08
T0827TS391_1-D1.rsa	39.726	0.796	0	0.204	52	0.764	33.73
T0827TS032_5-D1.rsa	39.583	0.073	0.273	0.655	72	0.55	58.18
T0827TS210_5-D1.rsa	39.583	0.082	0.427	0.491	54	0.733	34.09
T0827TS499_4-D1.rsa	39.535	0.216	0.134	0.649	87	0.454	19.03
T0827TS041_5-D1.rsa	39.535	0.291	0.127	0.582	78	0.507	17.35
T0827TS155_2-D1.rsa	39.535	0.343	0.149	0.507	68	0.581	27.43
T0827TS454_4-D1.rsa	39.474	0.244	0.175	0.581	126	0.313	35.48
T0827TS235_1-D1.rsa	39.474	0.25	0.352	0.398	43	0.918	73.84
T0827TS300_2-D1.rsa	39.474	0.261	0.018	0.721	80	0.493	32.66
T0827TS044_3-D1.rsa	39.474	0.216	0.063	0.721	80	0.493	19.14
T0827TS414_4-D1.rsa	39.474	0.261	0.135	0.604	67	0.589	29.73
T0827TS499_5-D1.rsa	39.474	0.297	0.054	0.649	72	0.548	25
T0827TS042_2-D1.rsa	39.437	0.452	0.107	0.44	74	0.533	21.47
T0827TS144_3-D1.rsa	39.437	0.524	0.119	0.357	60	0.657	33.97
T0827TS116_1-D1.rsa	39.286	0	0.237	0.763	100	0.393	52.29
T0827TS420_1-D1.rsa	39.286	0.183	0.286	0.532	67	0.586	56.35
T0827TS410_3-D1.rsa	39.286	0.27	0.063	0.667	84	0.468	27.18
T0827TS110_3-D1.rsa	39.063	0.066	0.346	0.588	80	0.488	15.26
T0827TS044_5-D1.rsa	38.462	0.733	0	0.267	44	0.874	14.4
T0827TS160_4-D1.rsa	38.356	0.647	0	0.353	90	0.426	37.16
T0827TS360_1-D1.rsa	38.356	0.784	0	0.216	55	0.697	40.88
T0827TS162_3-D1.rsa	38.235	0.698	0	0.302	38	1.006	42.66
T0827TS263_5-D1.rsa	38.235	0.698	0	0.302	38	1.006	42.06
T0827TS041_2-D1.rsa	38.028	0.405	0.131	0.464	78	0.488	40.22
T0827TS454_3-D1.rsa	38.028	0.494	0.125	0.381	64	0.594	37.02
T0827TS038_5-D1.rsa	37.931	0.664	0	0.336	86	0.441	15.92
T0827TS241_2-D1.rsa	37.5	0.206	0.23	0.563	71	0.528	27.58
T0827TS132_4-D1.rsa	37.5	0.081	0.287	0.632	86	0.436	13.97
T0827TS160_2-D1.rsa	37.5	0.016	0.317	0.667	84	0.446	17.66
T0827TS132_2-D1.rsa	37.5	0.036	0.436	0.527	58	0.647	40
T0827TS338_4-D1.rsa	37.5	0.198	0.325	0.476	60	0.625	49.6
T0827TS011_5-D1.rsa	37.5	0	0.43	0.57	65	0.577	23.25
T0827TS118_3-D1.rsa	37.5	0	0.386	0.614	70	0.536	21.93
T0827TS064_5-D1.rsa	37.5	0	0.439	0.561	64	0.586	26.32
T0827TS263_2-D1.rsa	37.5	0.434	0	0.566	77	0.487	26.29
T0827TS132_5-D1.rsa	37.5	0.19	0.357	0.452	57	0.658	43.65
T0827TS414_3-D1.rsa	37.5	0.19	0.365	0.444	56	0.67	38.89
T0827TS038_4-D1.rsa	37.5	0.044	0.333	0.623	71	0.528	23.03
T0827TS038_1-D1.rsa	36.986	0.773	0	0.227	58	0.638	39.31
T0827TS483_2-D1.rsa	36.986	0.753	0	0.247	63	0.587	29.8
T0827TS063_5-D1.rsa	36.842	0.295	0.157	0.548	119	0.31	31.8

T0827TS403_5-D1.rsa	36.842	0.234	0.189	0.577	64	0.576	39.41
T0827TS133_2-D1.rsa	36.842	0.204	0.398	0.398	43	0.857	65.97
T0827TS328_2-D1.rsa	36.842	0.351	0.036	0.613	68	0.542	27.25
T0827TS251_5-D1.rsa	36.842	0.287	0.481	0.231	25	1.474	61.34
T0827TS011_4-D1.rsa	36.62	0.446	0.149	0.405	68	0.539	44.23
T0827TS133_3-D1.rsa	36.62	0.506	0.155	0.339	57	0.642	50.8
T0827TS169_5-D1.rsa	36.62	0.679	0	0.321	54	0.678	15.71
T0827TS032_4-D1.rsa	36.207	0	0.217	0.783	112	0.323	77.45
T0827TS117_4-D1.rsa	36.111	0.362	0.094	0.545	116	0.311	69.04
T0827TS296_2-D1.rsa	36	0.283	0.152	0.565	78	0.462	14.86
T0827TS436_1-D1.rsa	35.938	0.015	0.449	0.537	73	0.492	70.59
T0827TS044_2-D1.rsa	35.938	0.14	0.243	0.618	84	0.428	13.42
T0827TS420_4-D1.rsa	35.714	0.175	0.325	0.5	63	0.567	51.98
T0827TS216_2-D1.rsa	35.714	0.167	0.373	0.46	58	0.616	52.18
T0827TS349_5-D1.rsa	35.714	0	0.344	0.656	86	0.415	16.79
T0827TS358_1-D1.rsa	35.714	0.015	0.336	0.649	85	0.42	16.79
T0827TS097_2-D1.rsa	35.714	0	0.389	0.611	80	0.446	17.37
T0827TS499_3-D1.rsa	35.714	0.183	0.373	0.444	56	0.638	44.84
T0827TS333_3-D1.rsa	35.714	0.313	0.069	0.618	81	0.441	15.84
T0827TS345_1-D1.rsa	35.616	0.765	0	0.235	60	0.594	40.1
T0827TS483_1-D1.rsa	35.616	0.773	0	0.227	58	0.614	32.45
T0827TS276_2-D1.rsa	35.417	0	0.418	0.582	64	0.553	26.82
T0827TS425_1-D1.rsa	35.417	0	0.436	0.564	62	0.571	27.05
T0827TS276_3-D1.rsa	35.294	0.579	0	0.421	53	0.666	46.03
T0827TS049_4-D1.rsa	35.227	0.071	0.443	0.486	144	0.245	36.91
T0827TS162_2-D1.rsa	35.211	0.494	0.137	0.369	62	0.568	41.19
T0827TS064_3-D1.rsa	35	0	0.289	0.711	81	0.432	19.3
T0827TS492_4-D1.rsa	35	0	0.333	0.667	76	0.461	19.74
T0827TS448_5-D1.rsa	35	0	0.377	0.623	71	0.493	36.62
T0827TS328_1-D1.rsa	35	0.096	0.14	0.763	87	0.402	17.32
T0827TS479_4-D1.rsa	35	0	0.509	0.491	56	0.625	41.89
T0827TS064_2-D1.rsa	35	0.018	0.474	0.509	58	0.603	44.74
T0827TS290_3-D1.rsa	35	0	0.351	0.649	74	0.473	25
T0827TS145_2-D1.rsa	35	0	0.465	0.535	61	0.574	25.44
T0827TS282_2-D1.rsa	35	0	0.439	0.561	64	0.547	26.1
T0827TS322_1-D1.rsa	35	0.026	0.439	0.535	61	0.574	26.32
T0827TS391_4-D1.rsa	35	0.035	0.482	0.482	55	0.636	24.56
T0827TS169_2-D1.rsa	34.884	0.336	0.112	0.552	74	0.471	27.98
T0827TS326_3-D1.rsa	34.737	0.534	0.059	0.407	96	0.362	63.03
T0827TS251_1-D1.rsa	34.615	0.37	0.194	0.436	72	0.481	15.98
T0827TS145_5-D1.rsa	34.375	0.772	0	0.228	31	1.109	12.87
T0827TS184_5-D1.rsa	34.247	0.761	0	0.239	61	0.561	44.02
T0827TS156_4-D1.rsa	34.247	0.757	0	0.243	62	0.552	39.31
T0827TS210_2-D1.rsa	34.247	0.757	0	0.243	62	0.552	39.41
T0827TS436_5-D1.rsa	34.211	0.25	0.491	0.259	28	1.222	94.21
T0827TS310_3-D1.rsa	34.211	0.252	0.189	0.559	62	0.552	37.84
T0827TS228_4-D1.rsa	34.211	0.278	0.481	0.241	26	1.316	85.88

T0827TS410_1-D1.rsa	33.929	0.151	0.262	0.587	74	0.458	40.08
T0827TS228_5-D1.rsa	33.929	0.198	0.262	0.54	68	0.499	32.54
T0827TS391_5-D1.rsa	33.929	0.333	0.079	0.587	74	0.458	18.85
T0827TS042_3-D1.rsa	33.929	0.167	0.333	0.5	63	0.539	44.84
T0827TS483_3-D1.rsa	33.929	0.183	0.389	0.429	54	0.628	45.63
T0827TS153_4-D1.rsa	33.333	0.432	0.052	0.516	110	0.303	66.37
T0827TS080_2-D1.rsa	32.877	0.757	0	0.243	62	0.53	50.98
T0827TS080_4-D1.rsa	32.877	0.71	0	0.29	74	0.444	38.92
T0827TS317_5-D1.rsa	32.813	0.074	0.25	0.676	92	0.357	14.52
T0827TS349_1-D1.rsa	32.759	0.594	0	0.406	104	0.315	54.69
T0827TS362_4-D1.rsa	32.759	0.699	0	0.301	77	0.425	22.56
T0827TS300_5-D1.rsa	32.692	0.364	0.206	0.43	71	0.46	15.66
T0827TS022_4-D1.rsa	32.632	0.517	0.038	0.445	105	0.311	50.11
T0827TS216_5-D1.rsa	32.558	0.306	0.104	0.59	79	0.412	30.04
T0827TS457_4-D1.rsa	32.5	0	0.351	0.649	74	0.439	37.94
T0827TS011_3-D1.rsa	32.5	0.026	0.202	0.772	88	0.369	17.76
T0827TS492_1-D1.rsa	32.5	0.061	0.149	0.789	90	0.361	18.2
T0827TS445_3-D1.rsa	32.5	0	0.316	0.684	78	0.417	19.52
T0827TS277_2-D1.rsa	32.5	0	0.439	0.561	64	0.508	17.32
T0827TS169_1-D1.rsa	32.394	0.494	0.143	0.363	61	0.531	52.08
T0827TS492_5-D1.rsa	32.394	0.488	0.167	0.345	58	0.559	51.28
T0827TS157_1-D1.rsa	32.143	0.159	0.333	0.508	64	0.502	53.77
T0827TS184_3-D1.rsa	32.143	0.175	0.405	0.421	53	0.606	52.98
T0827TS448_3-D1.rsa	32.143	0.167	0.365	0.468	59	0.545	57.14
T0827TS268_5-D1.rsa	32.143	0.151	0.341	0.508	64	0.502	46.63
T0827TS132_1-D1.rsa	32.143	0.183	0.373	0.444	56	0.574	54.56
T0827TS448_4-D1.rsa	32.143	0.175	0.373	0.452	57	0.564	52.18
T0827TS133_5-D1.rsa	32.143	0.151	0.286	0.563	71	0.453	43.06
T0827TS430_1-D1.rsa	32.143	0.063	0.397	0.54	68	0.473	43.85
T0827TS381_4-D1.rsa	32.143	0.143	0.365	0.492	62	0.518	38.49
T0827TS050_1-D1.rsa	32.143	0.183	0.365	0.452	57	0.564	43.06
T0827TS457_2-D1.rsa	32.143	0.214	0.365	0.421	53	0.606	43.85
T0827TS032_3-D1.rsa	32	0.058	0.152	0.79	109	0.294	21.74
T0827TS041_4-D1.rsa	31.579	0.564	0.059	0.377	89	0.355	51.59
T0827TS345_4-D1.rsa	31.579	0.241	0.509	0.25	27	1.17	94.21
T0827TS210_3-D1.rsa	31.579	0.287	0.37	0.343	37	0.853	71.99
T0827TS410_5-D1.rsa	31.507	0.765	0	0.235	60	0.525	53.63
T0827TS042_4-D1.rsa	31.507	0.741	0	0.259	66	0.477	38.82
T0827TS282_3-D1.rsa	31.25	0.125	0.228	0.647	88	0.355	16.18
T0827TS041_1-D1.rsa	30.986	0.512	0.089	0.399	67	0.462	51.6
T0827TS156_2-D1.rsa	30.476	0.423	0.209	0.368	86	0.354	72.54
T0827TS420_5-D1.rsa	30.357	0.167	0.317	0.516	65	0.467	50.4
T0827TS333_1-D1.rsa	30.357	0.167	0.333	0.5	63	0.482	50.79
T0827TS008_1-D1.rsa	30.357	0.175	0.341	0.484	61	0.498	48.61
T0827TS132_3-D1.rsa	30.357	0.175	0.357	0.468	59	0.515	52.38
T0827TS322_2-D1.rsa	30.357	0.175	0.341	0.484	61	0.498	48.41
T0827TS008_3-D1.rsa	30.357	0.151	0.341	0.508	64	0.474	39.29

T0827TS381_1-D1.rsa	30.357	0.175	0.365	0.46	58	0.523	51.19
T0827TS391_3-D1.rsa	30.357	0.19	0.397	0.413	52	0.584	53.37
T0827TS162_5-D1.rsa	30.357	0.175	0.421	0.405	51	0.595	51.98
T0827TS204_1-D1.rsa	30.357	0.175	0.413	0.413	52	0.584	53.97
T0827TS153_1-D1.rsa	30.357	0.151	0.349	0.5	63	0.482	50.4
T0827TS156_1-D1.rsa	30.233	0.306	0.015	0.679	91	0.332	29.1
T0827TS499_1-D1.rsa	30.233	0.276	0.104	0.619	83	0.364	30.78
T0827TS310_2-D1.rsa	30.233	0.328	0.015	0.657	88	0.344	21.45
T0827TS425_2-D1.rsa	30.233	0.291	0.201	0.507	68	0.445	21.08
T0827TS237_2-D1.rsa	30.137	0.369	0.1	0.531	197	0.153	69.28
T0827TS067_4-D1.rsa	30.137	0.765	0	0.235	60	0.502	47.35
T0827TS044_4-D1.rsa	30.137	0.765	0	0.235	60	0.502	46.86
T0827TS492_3-D1.rsa	30	0	0.307	0.693	79	0.38	22.15
T0827TS296_4-D1.rsa	30	0.026	0.342	0.632	72	0.417	18.2
T0827TS251_3-D1.rsa	30	0	0.377	0.623	71	0.423	24.12
T0827TS347_1-D1.rsa	30	0	0.395	0.605	69	0.435	29.39
T0827TS381_2-D1.rsa	29.688	0.081	0.257	0.662	90	0.33	13.6
T0827TS204_5-D1.rsa	29.688	0.096	0.301	0.603	82	0.362	15.81
T0827TS335_5-D1.rsa	29.688	0.096	0.301	0.603	82	0.362	16.18
T0827TS237_1-D1.rsa	29.6	0	0.298	0.702	231	0.128	31.46
T0827TS228_2-D1.rsa	29.577	0.506	0.083	0.411	69	0.429	49.84
T0827TS445_5-D1.rsa	29.474	0.538	0.064	0.398	94	0.314	50.95
T0827TS184_1-D1.rsa	28.947	0.259	0.259	0.481	52	0.557	69.21
T0827TS403_3-D1.rsa	28.767	0.729	0	0.271	69	0.417	39.71
T0827TS340_2-D1.rsa	28.571	0.198	0.317	0.484	61	0.468	50.79
T0827TS479_3-D1.rsa	28.571	0.175	0.373	0.452	57	0.501	51.98
T0827TS347_4-D1.rsa	28.571	0.175	0.381	0.444	56	0.51	51.39
T0827TS414_1-D1.rsa	28.571	0.151	0.389	0.46	58	0.493	50.99
T0827TS241_3-D1.rsa	28.571	0.183	0.405	0.413	52	0.549	49.6
T0827TS290_2-D1.rsa	28.571	0.175	0.413	0.413	52	0.549	53.97
T0827TS360_5-D1.rsa	28.571	0.19	0.373	0.437	55	0.519	43.06
T0827TS277_3-D1.rsa	28.571	0.19	0.389	0.421	53	0.539	43.25
T0827TS110_4-D1.rsa	28.571	0.175	0.437	0.389	49	0.583	53.17
T0827TS144_5-D1.rsa	28.571	0.19	0.373	0.437	55	0.519	42.66
T0827TS049_3-D1.rsa	28.205	0.382	0.135	0.483	143	0.197	57.99
T0827TS328_5-D1.rsa	28.125	0.36	0	0.64	87	0.323	20.96
T0827TS338_2-D1.rsa	28.125	0.096	0.279	0.625	85	0.331	16.91
T0827TS049_1-D1.rsa	28	0.431	0.02	0.55	222	0.126	45.61
T0827TS080_1-D1.rsa	28	0.072	0.348	0.58	80	0.35	54.35
T0827TS063_1-D1.rsa	27.941	0.383	0.082	0.535	130	0.215	67.89
T0827TS212_3-D1.rsa	27.907	0.337	0.207	0.456	77	0.362	72.63
T0827TS026_1-D1.rsa	27.619	0.303	0.184	0.513	120	0.23	70.94
T0827TS118_1-D1.rsa	27.586	0.73	0	0.27	69	0.4	57.32
T0827TS097_5-D1.rsa	27.5	0	0.456	0.544	62	0.444	46.27
T0827TS483_4-D1.rsa	27.5	0.018	0.36	0.623	71	0.387	41.67
T0827TS042_5-D1.rsa	27.5	0	0.254	0.746	85	0.324	19.3
T0827TS162_1-D1.rsa	27.5	0	0.289	0.711	81	0.34	22.81

T0827TS340_3-D1.rsa	27.5	0.018	0.439	0.544	62	0.444	14.91
T0827TS235_2-D1.rsa	27.397	0.749	0	0.251	64	0.428	55.49
T0827TS326_2-D1.rsa	27.397	0.761	0	0.239	61	0.449	51.67
T0827TS442_2-D1.rsa	27.397	0.757	0	0.243	62	0.442	42.94
T0827TS335_4-D1.rsa	27.397	0.765	0	0.235	60	0.457	49.61
T0827TS345_2-D1.rsa	26.786	0.183	0.333	0.484	61	0.439	53.17
T0827TS345_5-D1.rsa	26.786	0.167	0.365	0.468	59	0.454	50
T0827TS362_2-D1.rsa	26.786	0.175	0.397	0.429	54	0.496	53.57
T0827TS310_5-D1.rsa	26.563	0.096	0.279	0.625	85	0.313	16.73
T0827TS317_1-D1.rsa	26.563	0.096	0.294	0.61	83	0.32	16.73
T0827TS118_4-D1.rsa	26.471	0.391	0.082	0.527	128	0.207	69.56
T0827TS034_1-D1.rsa	26.471	0.412	0.103	0.486	118	0.224	71.78
T0827TS279_1-D1.rsa	26.471	0.461	0.086	0.453	110	0.241	67.11
T0827TS063_3-D1.rsa	26.263	0.194	0.215	0.59	170	0.154	65.02
T0827TS117_3-D1.rsa	25.974	0.397	0.098	0.504	113	0.23	82.62
T0827TS193_1-D1.rsa	25.974	0.446	0.116	0.438	98	0.265	81.07
T0827TS349_2-D1.rsa	25.641	0.372	0.132	0.497	147	0.174	60.76
T0827TS011_1-D1.rsa	25	0.183	0.341	0.476	60	0.417	53.17
T0827TS328_4-D1.rsa	25	0.143	0.357	0.5	63	0.397	42.46
T0827TS362_1-D1.rsa	25	0.053	0.184	0.763	87	0.287	24.12
T0827TS381_3-D1.rsa	25	0.175	0.444	0.381	48	0.521	54.76
T0827TS110_2-D1.rsa	25	0.018	0.342	0.64	73	0.342	18.42
T0827TS403_1-D1.rsa	25	0.175	0.389	0.437	55	0.455	53.57
T0827TS317_4-D1.rsa	25	0.096	0.324	0.581	79	0.316	15.99
T0827TS022_2-D1.rsa	24	0.087	0.268	0.645	89	0.27	67.57
T0827TS080_3-D1.rsa	24	0.065	0.333	0.601	83	0.289	67.39
T0827TS157_4-D1.rsa	23.684	0.304	0.099	0.596	198	0.12	43.67
T0827TS204_4-D1.rsa	23.438	0.096	0.301	0.603	82	0.286	16.73
T0827TS347_2-D1.rsa	23.438	0.096	0.287	0.618	84	0.279	16.18
T0827TS499_2-D1.rsa	23.256	0.328	0	0.672	90	0.258	16.05
T0827TS032_1-D1.rsa	23.214	0.119	0.333	0.548	69	0.336	40.48
T0827TS116_5-D1.rsa	23.077	0.412	0.095	0.493	146	0.158	59.2
T0827TS157_5-D1.rsa	22.5	0	0.281	0.719	82	0.274	44.52
T0827TS420_2-D1.rsa	22.5	0.018	0.228	0.754	86	0.262	48.03
T0827TS041_3-D1.rsa	22.5	0.018	0.246	0.737	84	0.268	44.96
T0827TS358_5-D1.rsa	22.5	0	0.132	0.868	99	0.227	28.51
T0827TS235_4-D1.rsa	22.308	0.225	0.129	0.646	296	0.075	34.89
T0827TS282_4-D1.rsa	21.875	0.096	0.316	0.588	80	0.273	16.73
T0827TS434_1-D1.rsa	21.875	0.096	0.301	0.603	82	0.267	16.18
T0827TS118_2-D1.rsa	21.429	0.159	0.175	0.667	84	0.255	21.23
T0827TS110_5-D1.rsa	20.93	0.321	0.045	0.634	85	0.246	26.49
T0827TS482_1-D1.rsa	20	0.044	0.149	0.807	92	0.217	26.1
T0827TS117_5-D1.rsa	19.737	0.441	0.015	0.544	248	0.08	66.5
T0827TS460_2-D1.rsa	19.079	0.45	0.033	0.518	236	0.081	61.68
T0827TS277_1-D1.rsa	17.5	0	0.07	0.93	106	0.165	19.74
T0827TS282_5-D1.rsa	17.5	0	0.281	0.719	82	0.213	37.72
T0827TS237_5-D1.rsa	17.105	0.346	0.123	0.53	176	0.097	99.1

T0827TS436_4-D1.rsa	14.035	0.452	0.078	0.47	103	0.136	81.96
T0827TS460_1-D1.rsa	13.158	0.358	0.123	0.518	172	0.076	98.87
T0827TS049_2-D1.rsa	12.766	0.311	0.183	0.506	130	0.098	80.93
T0827TS335_3-D1.rsa	12	0.051	0.355	0.594	82	0.146	50.54
T0827TS117_1-D1.rsa	10.638	0.327	0.195	0.479	123	0.086	84.14
T0858TS452_4-D1.rsa	75	0	0.065	0.935	101	0.743	17.36
T0858TS479_1-D1.rsa	73.529	0.222	0	0.778	98	0.75	14.48
T0858TS228_2-D1.rsa	72.093	0.187	0	0.813	109	0.661	16.05
T0858TS008_4-D1.rsa	70.833	0	0.509	0.491	53	1.336	52.78
T0858TS345_1-D1.rsa	70.588	0.325	0	0.675	85	0.83	31.94
T0858TS492_2-D1.rsa	68.421	0.135	0	0.865	96	0.713	19.59
T0858TS210_5-D1.rsa	63.158	0.144	0.09	0.766	85	0.743	18.24
T0858TS156_4-D1.rsa	62.5	0	0.546	0.454	49	1.276	58.33
T0858TS436_3-D1.rsa	60.714	0.061	0.366	0.573	75	0.81	53.82
T0858TS420_5-D1.rsa	60	0	0.272	0.728	83	0.723	34.65
T0858TS008_5-D1.rsa	58.333	0.019	0.639	0.343	37	1.577	68.06
T0858TS268_5-D1.rsa	58.333	0	0.509	0.491	53	1.101	58.56
T0858TS216_3-D1.rsa	58.14	0.396	0.015	0.59	79	0.736	41.23
T0858TS011_4-D1.rsa	57.895	0.045	0.045	0.91	101	0.573	20.72
T0858TS212_1-D1.rsa	57.143	0.015	0.443	0.542	71	0.805	56.68
T0858TS156_1-D1.rsa	55.814	0.284	0.045	0.672	90	0.62	41.79
T0858TS300_2-D1.rsa	54.93	0.226	0.101	0.673	113	0.486	23.72
T0858TS410_2-D1.rsa	54.93	0.411	0	0.589	99	0.555	24.68
T0858TS263_2-D1.rsa	54.167	0	0.546	0.454	49	1.105	72.69
T0858TS268_4-D1.rsa	53.521	0.292	0	0.708	119	0.45	13.46
T0858TS410_5-D1.rsa	52.632	0.126	0	0.874	97	0.543	27.7
T0858TS210_4-D1.rsa	52.632	0.252	0.108	0.64	71	0.741	38.06
T0858TS133_1-D1.rsa	52.113	0.173	0.125	0.702	118	0.442	17.15
T0858TS156_5-D1.rsa	51.563	0.118	0.081	0.801	109	0.473	17.83
T0858TS414_2-D1.rsa	51.163	0.224	0.06	0.716	96	0.533	31.34
T0858TS499_5-D1.rsa	50.704	0.012	0.185	0.804	135	0.376	12.34
T0858TS345_5-D1.rsa	50	0.315	0.036	0.648	107	0.467	49.84
T0858TS479_5-D1.rsa	50	0	0.269	0.731	79	0.633	40.05
T0858TS210_1-D1.rsa	50	0	0.137	0.863	113	0.442	14.88
T0858TS454_1-D1.rsa	50	0	0.489	0.511	67	0.746	60.3
T0858TS268_3-D1.rsa	48.837	0.187	0.112	0.701	94	0.52	31.53
T0858TS160_1-D1.rsa	47.917	0.018	0.236	0.745	82	0.584	51.59
T0858TS420_3-D1.rsa	46.875	0.14	0.081	0.779	106	0.442	36.03
T0858TS420_1-D1.rsa	46.512	0.179	0.015	0.806	108	0.431	26.87
T0858TS381_2-D1.rsa	46.512	0.269	0	0.731	98	0.475	23.32
T0858TS414_4-D1.rsa	46.429	0	0.13	0.87	114	0.407	14.5
T0858TS184_3-D1.rsa	46.429	0.015	0.405	0.58	76	0.611	59.16
T0858TS041_5-D1.rsa	44.643	0.111	0.048	0.841	106	0.421	14.88
T0858TS038_1-D1.rsa	44.643	0.151	0.262	0.587	74	0.603	49.8
T0858TS022_4-D1.rsa	44.231	0.024	0.145	0.83	137	0.323	11.23
T0858TS263_1-D1.rsa	44.231	0.012	0.273	0.715	118	0.375	9.34
T0858TS038_3-D1.rsa	44.186	0.351	0.015	0.634	85	0.52	38.62

T0858TS160_2-D1.rsa	44.186	0.381	0.015	0.604	81	0.546	34.52
T0858TS216_2-D1.rsa	44.186	0.388	0.06	0.552	74	0.597	41.05
T0858TS237_3-D1.rsa	43.75	0	0.132	0.868	118	0.371	24.82
T0858TS008_3-D1.rsa	43.75	0.045	0.309	0.645	71	0.616	52.27
T0858TS410_3-D1.rsa	43.662	0.238	0.012	0.75	126	0.347	16.35
T0858TS022_1-D1.rsa	42.857	0	0.328	0.672	88	0.487	59.16
T0858TS381_4-D1.rsa	42.857	0	0.214	0.786	103	0.416	16.6
T0858TS479_2-D1.rsa	42.857	0	0.405	0.595	78	0.549	61.83
T0858TS492_5-D1.rsa	42.857	0.19	0.032	0.778	98	0.437	22.82
T0858TS454_3-D1.rsa	42.5	0.018	0.272	0.711	81	0.525	42.33
T0858TS492_4-D1.rsa	42.105	0.36	0.09	0.55	61	0.69	54.51
T0858TS041_3-D1.rsa	41.86	0.224	0.075	0.701	94	0.445	41.23
T0858TS251_1-D1.rsa	41.379	0	0.161	0.839	120	0.345	50.87
T0858TS454_5-D1.rsa	41.379	0.555	0	0.445	114	0.363	15.43
T0858TS228_5-D1.rsa	39.583	0.091	0.264	0.645	71	0.558	59.77
T0858TS041_1-D1.rsa	39.437	0.423	0	0.577	97	0.407	40.38
T0858TS184_2-D1.rsa	39.437	0	0.28	0.72	121	0.326	12.02
T0858TS335_1-D1.rsa	39.286	0	0.076	0.924	121	0.325	17.37
T0858TS008_2-D1.rsa	39.286	0	0.45	0.55	72	0.546	55.92
T0858TS346_1-D1.rsa	39.286	0	0.458	0.542	71	0.553	55.92
T0858TS300_4-D1.rsa	39.063	0.015	0.331	0.654	89	0.439	62.5
T0858TS345_2-D1.rsa	39.063	0.015	0.419	0.566	77	0.507	71.32
T0858TS381_5-D1.rsa	39.063	0.015	0.463	0.522	71	0.55	69.48
T0858TS022_3-D1.rsa	37.5	0.46	0.08	0.46	98	0.383	76.52
T0858TS237_5-D1.rsa	37.5	0.015	0.346	0.64	87	0.431	64.52
T0858TS268_2-D1.rsa	37.5	0.015	0.456	0.529	72	0.521	72.79
T0858TS117_1-D1.rsa	37.5	0.135	0	0.865	109	0.344	22.42
T0858TS479_4-D1.rsa	37.209	0.328	0	0.672	90	0.413	41.23
T0858TS237_1-D1.rsa	36.842	0.5	0.059	0.441	104	0.354	62.71
T0858TS133_3-D1.rsa	36.842	0.517	0.055	0.428	101	0.365	59.32
T0858TS193_2-D1.rsa	36.842	0.559	0.051	0.39	92	0.4	58.69
T0858TS277_3-D1.rsa	36.207	0	0.189	0.811	116	0.312	58.74
T0858TS263_4-D1.rsa	36.111	0.399	0.094	0.507	108	0.334	78.43
T0858TS237_2-D1.rsa	36.111	0.455	0.094	0.451	96	0.376	75
T0858TS277_1-D1.rsa	35.965	0.267	0.171	0.562	122	0.295	56.45
T0858TS448_2-D1.rsa	35.965	0.272	0.166	0.562	122	0.295	51.61
T0858TS448_1-D1.rsa	35.965	0.258	0.217	0.525	114	0.315	60.83
T0858TS216_1-D1.rsa	35.211	0.387	0.06	0.554	93	0.379	38.94
T0858TS184_1-D1.rsa	34.375	0	0.449	0.551	75	0.458	65.44
T0858TS251_3-D1.rsa	33.721	0.373	0.201	0.426	72	0.468	71.6
T0858TS268_1-D1.rsa	33.621	0.648	0	0.352	90	0.374	58.89
T0858TS133_2-D1.rsa	33.333	0.46	0.103	0.437	93	0.358	71.07
T0858TS216_4-D1.rsa	32.632	0.555	0.059	0.386	91	0.359	63.88
T0858TS492_3-D1.rsa	32.558	0.358	0.097	0.545	73	0.446	53.55
T0858TS228_3-D1.rsa	32.558	0.321	0.045	0.634	85	0.383	42.35
T0858TS448_3-D1.rsa	32.456	0.281	0.161	0.558	121	0.268	51.27
T0858TS349_4-D1.rsa	32.353	0.37	0.099	0.531	129	0.251	58.78

T0858TS263_3-D1.rsa	32	0	0.014	0.986	136	0.235	54.53
T0858TS300_5-D1.rsa	32	0.094	0.362	0.543	75	0.427	67.57
T0858TS277_5-D1.rsa	31.944	0.39	0.094	0.516	110	0.29	70.56
T0858TS022_5-D1.rsa	31.579	0.29	0.189	0.521	113	0.279	61.06
T0858TS050_1-D1.rsa	31.579	0.53	0.025	0.445	105	0.301	53.18
T0858TS011_2-D1.rsa	31.579	0.402	0	0.598	131	0.241	18.61
T0858TS216_5-D1.rsa	31.579	0.547	0.059	0.394	93	0.34	56.14
T0858TS414_3-D1.rsa	31.395	0.367	0.136	0.497	84	0.374	68.49
T0858TS184_5-D1.rsa	31.25	0.015	0.456	0.529	72	0.434	70.4
T0858TS335_4-D1.rsa	31.169	0.379	0.036	0.585	131	0.238	15.83
T0858TS349_1-D1.rsa	30.702	0.258	0.23	0.512	111	0.277	67.51
T0858TS210_3-D1.rsa	30.526	0.504	0.059	0.436	103	0.296	51.16
T0858TS300_3-D1.rsa	30.172	0.586	0	0.414	106	0.285	57.03
T0858TS492_1-D1.rsa	30.172	0.621	0	0.379	97	0.311	65.53
T0858TS193_1-D1.rsa	29.474	0.555	0.059	0.386	91	0.324	51.16
T0858TS160_3-D1.rsa	29.412	0.44	0.099	0.461	112	0.263	67.11
T0858TS499_2-D1.rsa	28.947	0.29	0.175	0.535	116	0.25	62.33
T0858TS349_5-D1.rsa	28.571	0.402	0.121	0.478	107	0.267	75
T0858TS237_4-D1.rsa	28.571	0.411	0.107	0.482	108	0.265	80.48
T0858TS041_2-D1.rsa	28.571	0.372	0.171	0.457	107	0.267	69.02
T0858TS335_5-D1.rsa	28.571	0.393	0.235	0.372	87	0.328	58.12
T0858TS452_3-D1.rsa	28.448	0.645	0	0.355	91	0.313	58.1
T0858TS410_4-D1.rsa	28.421	0.521	0.047	0.432	102	0.279	62.61
T0858TS381_1-D1.rsa	28	0.051	0.362	0.587	81	0.346	68.66
T0858TS436_1-D1.rsa	28	0.058	0.413	0.529	73	0.384	69.2
T0858TS184_4-D1.rsa	28	0.065	0.319	0.616	85	0.329	67.94
T0858TS117_4-D1.rsa	28	0.014	0.362	0.623	86	0.326	64.13
T0858TS452_5-D1.rsa	28	0.065	0.355	0.58	80	0.35	57.97
T0858TS156_2-D1.rsa	28	0.065	0.283	0.652	90	0.311	67.39
T0858TS335_2-D1.rsa	28	0.065	0.37	0.565	78	0.359	73.19
T0858TS228_1-D1.rsa	28	0.072	0.326	0.601	83	0.337	53.99
T0858TS011_3-D1.rsa	28	0.051	0.348	0.601	83	0.337	68.3
T0858TS454_4-D1.rsa	28	0.109	0.246	0.645	89	0.315	60.69
T0858TS011_1-D1.rsa	27.907	0.349	0.213	0.438	74	0.377	71.3
T0858TS277_2-D1.rsa	27.869	0.033	0.443	0.524	110	0.253	71.39
T0858TS381_3-D1.rsa	27.619	0.385	0.239	0.376	88	0.314	75.21
T0858TS160_4-D1.rsa	26.4	0.012	0.374	0.614	202	0.131	51.75
T0858TS345_4-D1.rsa	26.316	0.496	0.042	0.462	109	0.241	62.82
T0858TS263_5-D1.rsa	25.581	0.314	0.207	0.479	81	0.316	70.71
T0858TS251_4-D1.rsa	25	0.442	0.131	0.426	107	0.234	86.65
T0858TS117_3-D1.rsa	25	0	0.149	0.851	97	0.258	38.6
T0858TS171_5-D1.rsa	24.59	0.029	0.424	0.548	115	0.214	61.94
T0858TS349_3-D1.rsa	24.571	0.557	0.022	0.421	170	0.145	49.38
T0858TS251_2-D1.rsa	24.359	0.382	0.128	0.49	145	0.168	57.73
T0858TS171_3-D1.rsa	24.359	0.395	0.142	0.463	137	0.178	58.51
T0858TS156_3-D1.rsa	24	0.036	0.109	0.855	118	0.203	33.7
T0858TS008_1-D1.rsa	24	0.065	0.348	0.587	81	0.296	67.39

T0858TS420_2-D1.rsa	24	0.058	0.406	0.536	74	0.324	73.19
T0858TS038_5-D1.rsa	24	0.043	0.333	0.623	86	0.279	67.75
T0858TS436_4-D1.rsa	22.857	0.431	0.012	0.557	225	0.102	40.59
T0858TS251_5-D1.rsa	22.807	0.429	0.055	0.516	113	0.202	77.74
T0858TS448_4-D1.rsa	20.588	0.362	0.111	0.527	128	0.161	68.22
T0858TS228_4-D1.rsa	20.513	0.426	0.149	0.426	126	0.163	54.51
T0858TS279_1-D1.rsa	20	0.058	0.014	0.928	128	0.156	19.2
T0858TS133_4-D1.rsa	19	0.466	0.139	0.394	99	0.192	72.21
T0858TS117_2-D1.rsa	18.857	0.28	0.015	0.705	285	0.066	23.76
T0858TS436_5-D1.rsa	18.462	0.266	0.24	0.493	226	0.082	68.5
T0858TS171_1-D1.rsa	17.647	0.387	0.07	0.543	132	0.134	54.33
T0858TS022_2-D1.rsa	17.021	0.315	0.179	0.506	130	0.131	60.31
T0858TS206_1-D1.rsa	16.154	0.253	0.238	0.509	233	0.069	77.5
T0858TS436_2-D1.rsa	15.385	0.264	0.223	0.513	235	0.065	76.61
T0858TS499_1-D1.rsa	14.894	0.331	0.167	0.502	129	0.115	56.23
T0858TS277_4-D1.rsa	10.638	0.385	0.179	0.436	112	0.095	81.91
T0795TS032_2-D1.rsa	86.842	0.18	0.054	0.766	85	1.022	19.59
T0795TS457_5-D1.rsa	73.684	0.126	0	0.874	97	0.76	18.92
T0795TS454_5-D1.rsa	73.529	0.468	0	0.532	67	1.097	28.18
T0795TS203_2-D1.rsa	73.438	0.037	0.125	0.838	114	0.644	39.89
T0795TS317_4-D1.rsa	72	0.188	0.072	0.739	102	0.706	12.68
T0795TS049_2-D1.rsa	71.154	0.218	0.036	0.745	123	0.578	19.3
T0795TS340_2-D1.rsa	71.053	0.135	0	0.865	96	0.74	18.24
T0795TS499_5-D1.rsa	69.231	0.273	0.024	0.703	116	0.597	25.79
T0795TS360_5-D1.rsa	65.789	0.036	0	0.964	107	0.615	15.31
T0795TS153_4-D1.rsa	64.789	0.196	0.06	0.744	125	0.518	12.66
T0795TS358_4-D1.rsa	64.286	0	0.046	0.954	125	0.514	16.79
T0795TS360_3-D1.rsa	61.538	0.261	0.073	0.667	110	0.559	64.72
T0795TS116_4-D1.rsa	60.465	0.224	0.045	0.731	98	0.617	42.35
T0795TS054_5-D1.rsa	59.615	0.267	0.109	0.624	103	0.579	80.38
T0795TS277_3-D1.rsa	59.615	0.261	0.085	0.655	108	0.552	61.87
T0795TS296_1-D1.rsa	59.155	0.244	0.083	0.673	113	0.523	25.8
T0795TS054_4-D1.rsa	58.824	0.611	0	0.389	49	1.2	44.44
T0795TS338_1-D1.rsa	58.14	0.231	0.03	0.739	99	0.587	41.23
T0795TS465_5-D1.rsa	57.692	0.297	0.048	0.655	108	0.534	71.52
T0795TS282_3-D1.rsa	57.692	0.291	0.073	0.636	105	0.549	67.72
T0795TS203_3-D1.rsa	57.692	0.321	0.139	0.539	89	0.648	75.16
T0795TS410_5-D1.rsa	57.692	0.333	0.103	0.564	93	0.62	67.25
T0795TS144_4-D1.rsa	57.692	0.279	0.133	0.588	97	0.595	62.66
T0795TS184_4-D1.rsa	57.576	0.125	0	0.875	252	0.228	8.42
T0795TS296_2-D1.rsa	56	0.029	0.13	0.841	116	0.483	14.67
T0795TS032_4-D1.rsa	55.814	0.231	0.015	0.754	101	0.553	36.57
T0795TS203_5-D1.rsa	55.769	0.273	0.085	0.642	106	0.526	75.16
T0795TS049_1-D1.rsa	55.769	0.297	0.182	0.521	86	0.648	60.13
T0795TS336_5-D1.rsa	53.846	0.255	0.048	0.697	115	0.468	57.28
T0795TS241_1-D1.rsa	53.846	0.303	0.139	0.558	92	0.585	58.07
T0795TS011_4-D1.rsa	53.571	0.031	0.069	0.901	118	0.454	24.05

T0795TS157_1-D1.rsa	51.974	0.279	0	0.721	329	0.158	6.36
T0795TS345_1-D1.rsa	51.923	0.261	0.048	0.691	114	0.455	60.76
T0795TS260_1-D1.rsa	51.923	0	0.085	0.915	151	0.344	12.5
T0795TS420_2-D1.rsa	51.923	0.176	0.127	0.697	115	0.452	15.03
T0795TS333_3-D1.rsa	51.923	0.279	0.085	0.636	105	0.495	40.35
T0795TS442_5-D1.rsa	50	0.054	0.253	0.693	205	0.244	21.96
T0795TS414_2-D1.rsa	48.571	0.167	0.068	0.765	179	0.271	19.34
T0795TS162_4-D1.rsa	48.276	0.042	0.168	0.79	113	0.427	29.89
T0795TS358_5-D1.rsa	48.214	0.183	0.103	0.714	90	0.536	48.61
T0795TS006_2-D1.rsa	47.368	0.126	0	0.874	97	0.488	16.89
T0795TS362_3-D1.rsa	46.875	0.103	0.213	0.684	93	0.504	12.13
T0795TS347_4-D1.rsa	46.429	0	0.16	0.84	110	0.422	56.87
T0795TS210_5-D1.rsa	44.828	0	0.07	0.93	133	0.337	59.27
T0795TS479_2-D1.rsa	44.828	0	0.07	0.93	133	0.337	55.07
T0795TS290_2-D1.rsa	44.231	0.206	0.012	0.782	129	0.343	14.56
T0795TS155_2-D1.rsa	43.421	0.157	0.06	0.783	260	0.167	30.8
T0795TS118_2-D1.rsa	43.103	0	0.091	0.909	130	0.332	59.79
T0795TS391_2-D1.rsa	43.103	0	0.154	0.846	121	0.356	60.31
T0795TS326_3-D1.rsa	42.982	0.313	0.074	0.613	133	0.323	54.15
T0795TS322_1-D1.rsa	42.857	0	0.45	0.55	72	0.595	62.59
T0795TS358_3-D1.rsa	42.647	0.358	0.041	0.601	146	0.292	16.44
T0795TS454_3-D1.rsa	42.5	0	0.123	0.877	100	0.425	42.76
T0795TS120_2-D1.rsa	42.286	0.606	0.012	0.381	154	0.275	12.5
T0795TS032_3-D1.rsa	42.254	0	0.161	0.839	141	0.3	12.98
T0795TS425_1-D1.rsa	42.188	0.015	0.36	0.625	85	0.496	70.96
T0795TS162_5-D1.rsa	42.188	0.015	0.449	0.537	73	0.578	72.43
T0795TS008_1-D1.rsa	42.188	0.081	0.338	0.581	79	0.534	59.38
T0795TS162_2-D1.rsa	42.105	0.253	0.203	0.544	118	0.357	64.86
T0795TS360_2-D1.rsa	42.105	0.126	0	0.874	97	0.434	35.59
T0795TS403_1-D1.rsa	41.667	0.352	0.094	0.554	118	0.353	69.42
T0795TS433_1-D1.rsa	41.379	0	0.063	0.937	134	0.309	56.29
T0795TS073_1-D1.rsa	41.379	0	0.126	0.874	125	0.331	60.66
T0795TS326_4-D1.rsa	40.909	0.064	0.426	0.51	151	0.271	43.16
T0795TS479_3-D1.rsa	40.909	0.074	0.432	0.493	146	0.28	36.57
T0795TS296_3-D1.rsa	40.625	0	0.346	0.654	89	0.456	68.2
T0795TS006_1-D1.rsa	40.625	0.088	0.096	0.816	111	0.366	12.87
T0795TS410_2-D1.rsa	40.351	0.313	0.124	0.562	122	0.331	55.76
T0795TS403_3-D1.rsa	40.278	0.366	0.094	0.54	115	0.35	71.83
T0795TS210_2-D1.rsa	40.278	0.455	0.094	0.451	96	0.42	76.4
T0795TS268_4-D1.rsa	39.773	0.051	0.348	0.601	178	0.223	47.13
T0795TS479_4-D1.rsa	39.655	0	0.175	0.825	118	0.336	80.42
T0795TS014_1-D1.rsa	39.655	0.014	0.098	0.888	127	0.312	60.49
T0795TS169_2-D1.rsa	39.655	0	0.14	0.86	123	0.322	58.57
T0795TS381_3-D1.rsa	39.474	0.203	0.097	0.7	152	0.26	63.36
T0795TS241_2-D1.rsa	39.063	0.015	0.39	0.596	81	0.482	70.59
T0795TS216_4-D1.rsa	39.063	0.735	0	0.265	36	1.085	11.58
T0795TS144_1-D1.rsa	38.889	0.376	0.075	0.549	117	0.332	67.89

T0795TS260_5-D1.rsa	38.889	0.366	0.075	0.559	119	0.327	69.16
T0795TS210_4-D1.rsa	38.889	0.352	0.085	0.563	120	0.324	68.4
T0795TS260_4-D1.rsa	38.636	0.064	0.355	0.581	172	0.225	40.96
T0795TS457_1-D1.rsa	38.596	0.272	0.189	0.539	117	0.33	58.87
T0795TS144_3-D1.rsa	38.596	0.369	0.161	0.47	102	0.378	60.25
T0795TS362_5-D1.rsa	38.462	0.152	0.079	0.77	127	0.303	32.59
T0795TS452_2-D1.rsa	38.158	0.346	0.036	0.617	205	0.186	11.14
T0795TS282_4-D1.rsa	37.931	0	0.112	0.888	127	0.299	78.85
T0795TS310_2-D1.rsa	37.931	0	0.14	0.86	123	0.308	60.49
T0795TS155_3-D1.rsa	37.714	0.292	0	0.708	286	0.132	8.11
T0795TS097_2-D1.rsa	37.5	0.3	0.061	0.638	136	0.276	73.48
T0795TS335_3-D1.rsa	37.5	0.064	0.368	0.568	168	0.223	37.67
T0795TS410_4-D1.rsa	37.5	0.427	0.08	0.493	105	0.357	70.05
T0795TS457_2-D1.rsa	37.5	0.029	0.449	0.522	71	0.528	71.51
T0795TS080_3-D1.rsa	37.374	0.215	0.122	0.663	191	0.196	46.7
T0795TS038_1-D1.rsa	37.374	0.229	0.056	0.715	206	0.181	9.38
T0795TS184_5-D1.rsa	37.288	0.019	0.234	0.748	160	0.233	57.71
T0795TS317_3-D1.rsa	36.842	0.508	0.064	0.428	101	0.365	59.32
T0795TS160_5-D1.rsa	36.842	0.25	0.481	0.269	29	1.27	93.06
T0795TS080_2-D1.rsa	36.8	0.03	0.292	0.678	223	0.165	24.7
T0795TS349_2-D1.rsa	36.571	0.339	0.02	0.641	259	0.141	28.59
T0795TS097_4-D1.rsa	36.364	0.068	0.405	0.527	156	0.233	47.3
T0795TS452_1-D1.rsa	36.364	0.057	0.395	0.547	162	0.224	35.98
T0795TS328_3-D1.rsa	36.207	0	0.259	0.741	106	0.342	80.77
T0795TS439_1-D1.rsa	36.111	0.31	0.094	0.596	127	0.284	69.42
T0795TS345_4-D1.rsa	36.066	0.01	0.4	0.59	124	0.291	63.81
T0795TS044_1-D1.rsa	36.066	0.029	0.414	0.557	117	0.308	69.65
T0795TS160_3-D1.rsa	36.066	0	0.405	0.595	125	0.289	45.27
T0795TS044_2-D1.rsa	36	0.567	0.005	0.428	173	0.208	10.77
T0795TS064_1-D1.rsa	35.938	0.015	0.412	0.574	78	0.461	64.52
T0795TS381_1-D1.rsa	35.294	0.362	0.095	0.543	132	0.267	65.33
T0795TS347_1-D1.rsa	35.227	0.081	0.459	0.459	136	0.259	49.83
T0795TS482_1-D1.rsa	35.088	0.244	0.189	0.567	123	0.285	61.64
T0795TS410_1-D1.rsa	35.088	0.313	0.147	0.539	117	0.3	61.64
T0795TS054_1-D1.rsa	35.088	0.29	0.143	0.567	123	0.285	55.53
T0795TS132_3-D1.rsa	35.088	0.286	0.157	0.558	121	0.29	55.3
T0795TS171_2-D1.rsa	34.857	0.582	0	0.418	169	0.206	36.63
T0795TS328_4-D1.rsa	34.722	0.357	0.094	0.549	117	0.297	71.07
T0795TS216_3-D1.rsa	34.615	0.685	0	0.315	52	0.666	15.03
T0795TS452_3-D1.rsa	34.483	0	0.161	0.839	120	0.287	76.75
T0795TS362_1-D1.rsa	34.483	0.574	0	0.426	109	0.316	55.18
T0795TS169_4-D1.rsa	34.426	0	0.333	0.667	140	0.246	64.06
T0795TS333_2-D1.rsa	34.426	0.01	0.414	0.576	121	0.285	59.2
T0795TS301_4-D1.rsa	34.375	0	0.199	0.801	109	0.315	65.99
T0795TS080_4-D1.rsa	34.343	0.174	0.087	0.74	213	0.161	56.42
T0795TS173_3-D1.rsa	34.343	0.247	0.083	0.67	193	0.178	45.57
T0795TS430_1-D1.rsa	34.343	0.233	0.194	0.573	165	0.208	38.54

T0795TS358_2-D1.rsa	34.091	0.068	0.527	0.405	120	0.284	47.04
T0795TS452_5-D1.rsa	33.898	0.047	0.21	0.743	159	0.213	56.08
T0795TS317_2-D1.rsa	33.766	0.424	0.027	0.549	123	0.275	15.24
T0795TS063_2-D1.rsa	33.6	0.012	0.146	0.842	277	0.121	50.76
T0795TS436_3-D1.rsa	33.333	0.253	0.139	0.608	175	0.19	46.88
T0795TS328_1-D1.rsa	32.955	0.074	0.412	0.514	152	0.217	39.1
T0795TS144_2-D1.rsa	32.955	0.088	0.47	0.443	131	0.252	49.75
T0795TS362_4-D1.rsa	32.877	0.741	0	0.259	66	0.498	59.22
T0795TS034_1-D1.rsa	32.8	0.006	0.255	0.739	243	0.135	40.2
T0795TS442_2-D1.rsa	32.8	0.015	0.371	0.614	202	0.162	53.12
T0795TS301_1-D1.rsa	32.787	0	0.457	0.543	114	0.288	72.76
T0795TS340_5-D1.rsa	32.759	0	0.203	0.797	114	0.287	77.97
T0795TS317_5-D1.rsa	32.759	0	0.273	0.727	104	0.315	86.89
T0795TS310_4-D1.rsa	32.759	0	0.217	0.783	112	0.292	76.4
T0795TS290_1-D1.rsa	32.759	0.582	0	0.418	107	0.306	55.08
T0795TS006_5-D1.rsa	32.692	0.236	0.103	0.661	109	0.3	17.25
T0795TS204_1-D1.rsa	32.353	0.383	0.091	0.527	128	0.253	65.67
T0795TS333_5-D1.rsa	32.143	0.167	0.413	0.421	53	0.606	48.02
T0795TS420_1-D1.rsa	32	0.029	0.312	0.659	91	0.352	57.43
T0795TS336_4-D1.rsa	31.944	0.305	0.103	0.592	126	0.254	65.61
T0795TS120_1-D1.rsa	31.579	0.267	0.147	0.585	127	0.249	65.78
T0795TS338_2-D1.rsa	31.429	0.35	0.226	0.423	99	0.317	70.73
T0795TS008_2-D1.rsa	31.395	0.337	0.201	0.462	78	0.403	67.9
T0795TS401_5-D1.rsa	31.2	0.015	0.368	0.617	203	0.154	51.82
T0795TS067_1-D1.rsa	31.148	0.024	0.429	0.548	115	0.271	73.13
T0795TS301_3-D1.rsa	30.882	0.379	0.053	0.568	138	0.224	60.78
T0795TS216_5-D1.rsa	30.857	0.441	0.037	0.522	211	0.146	27.97
T0795TS300_3-D1.rsa	30.822	0.383	0.108	0.509	189	0.163	78.34
T0795TS492_2-D1.rsa	30.822	0.404	0.119	0.477	177	0.174	65.39
T0795TS042_1-D1.rsa	30.4	0.012	0.264	0.723	238	0.128	47.72
T0795TS034_2-D1.rsa	30.4	0.015	0.337	0.647	213	0.143	51.37
T0795TS448_2-D1.rsa	30.4	0.006	0.322	0.672	221	0.138	36.93
T0795TS452_4-D1.rsa	30.303	0.229	0.208	0.563	162	0.187	70.4
T0795TS024_1-D1.rsa	30.303	0.219	0.184	0.597	172	0.176	61.02
T0795TS110_5-D1.rsa	30.303	0.198	0.194	0.608	175	0.173	59.81
T0795TS347_3-D1.rsa	30.263	0.439	0.024	0.537	245	0.124	35.14
T0795TS064_2-D1.rsa	30.172	0.594	0	0.406	104	0.29	55.18
T0795TS026_1-D1.rsa	30.137	0.38	0.113	0.507	188	0.16	79.84
T0795TS492_4-D1.rsa	30.137	0.383	0.113	0.504	187	0.161	75.14
T0795TS110_3-D1.rsa	30	0.343	0.1	0.558	140	0.214	80.78
T0795TS300_1-D1.rsa	29.703	0.24	0.128	0.632	203	0.146	72.66
T0795TS044_3-D1.rsa	29.703	0.262	0.146	0.592	190	0.156	75.7
T0795TS171_1-D1.rsa	29.703	0.277	0.131	0.592	190	0.156	68.15
T0795TS290_5-D1.rsa	29.703	0.324	0.109	0.567	182	0.163	69.31
T0795TS479_1-D1.rsa	29.508	0.048	0.448	0.505	106	0.278	71.27
T0795TS133_3-D1.rsa	29.412	0.346	0.091	0.564	137	0.215	65.22
T0795TS425_4-D1.rsa	29.412	0.354	0.091	0.556	135	0.218	65.67

T0795TS290_4-D1.rsa	29.412	0.346	0.111	0.543	132	0.223	68.11
T0795TS340_4-D1.rsa	29.412	0.354	0.091	0.556	135	0.218	66
T0795TS391_1-D1.rsa	29.412	0.358	0.078	0.564	137	0.215	65.11
T0795TS296_4-D1.rsa	29.412	0.399	0.095	0.506	123	0.239	70.33
T0795TS006_4-D1.rsa	29.412	0.391	0.107	0.502	122	0.241	65.89
T0795TS054_3-D1.rsa	29.293	0.208	0.16	0.632	182	0.161	60.68
T0795TS425_5-D1.rsa	29.293	0.212	0.188	0.601	173	0.169	61.2
T0795TS410_3-D1.rsa	29.293	0.25	0.222	0.528	152	0.193	58.42
T0795TS349_3-D1.rsa	29.143	0.381	0.005	0.614	248	0.118	34.59
T0795TS414_5-D1.rsa	29.143	0.468	0.022	0.51	206	0.141	38.61
T0795TS204_4-D1.rsa	29	0.378	0.112	0.51	128	0.227	88.45
T0795TS268_2-D1.rsa	29	0.323	0.084	0.594	149	0.195	61.35
T0795TS263_1-D1.rsa	28.814	0.037	0.238	0.724	155	0.186	51.64
T0795TS116_1-D1.rsa	28.767	0.38	0.097	0.523	194	0.148	80.31
T0795TS349_5-D1.rsa	28.571	0.362	0.094	0.544	203	0.141	83.06
T0795TS156_5-D1.rsa	28.571	0.401	0.005	0.594	240	0.119	34.78
T0795TS465_3-D1.rsa	28.571	0.366	0.112	0.522	117	0.244	76.43
T0795TS216_2-D1.rsa	28.571	0.19	0.381	0.429	54	0.529	44.05
T0795TS445_2-D1.rsa	28.283	0.222	0.135	0.642	185	0.153	66.41
T0795TS381_2-D1.rsa	28.283	0.184	0.153	0.663	191	0.148	54.34
T0795TS448_1-D1.rsa	28.283	0.215	0.194	0.59	170	0.166	61.72
T0795TS133_5-D1.rsa	28.283	0.267	0.212	0.521	150	0.189	60.42
T0795TS401_3-D1.rsa	28.082	0.41	0.113	0.477	177	0.159	81.27
T0795TS300_4-D1.rsa	28	0.009	0.277	0.714	235	0.119	56.69
T0795TS258_4-D1.rsa	28	0.52	0.057	0.423	171	0.164	29.58
T0795TS216_1-D1.rsa	28	0.051	0.21	0.739	102	0.275	64.31
T0795TS173_1-D1.rsa	27.941	0.354	0.091	0.556	135	0.207	69.44
T0795TS403_5-D1.rsa	27.941	0.374	0.095	0.531	129	0.217	65.89
T0795TS038_4-D1.rsa	27.869	0	0.119	0.881	185	0.151	62.44
T0795TS301_2-D1.rsa	27.869	0.029	0.362	0.61	128	0.218	62.31
T0795TS056_1-D1.rsa	27.869	0.038	0.39	0.571	120	0.232	65.17
T0795TS034_3-D1.rsa	27.723	0.221	0.131	0.648	208	0.133	74.45
T0795TS335_5-D1.rsa	27.723	0.293	0.146	0.561	180	0.154	64.41
T0795TS067_2-D1.rsa	27.619	0.368	0.197	0.436	102	0.271	69.66
T0795TS065_1-D1.rsa	27.429	0.5	0.02	0.48	194	0.141	46.72
T0795TS171_3-D1.rsa	27.397	0.407	0.097	0.496	184	0.149	76.97
T0795TS492_5-D1.rsa	27.397	0.439	0.1	0.461	171	0.16	60.15
T0795TS328_2-D1.rsa	27.273	0.271	0.219	0.51	147	0.186	73.26
T0795TS364_1-D1.rsa	27.273	0.362	0.112	0.527	118	0.231	76.55
T0795TS237_2-D1.rsa	27.211	0.383	0.097	0.52	194	0.14	82.85
T0795TS483_5-D1.rsa	27.2	0.006	0.274	0.72	237	0.115	46.43
T0795TS212_1-D1.rsa	27.2	0.012	0.322	0.666	219	0.124	49.92
T0795TS206_1-D1.rsa	27.119	0.014	0.322	0.664	142	0.191	60.75
T0795TS290_3-D1.rsa	27	0.426	0.124	0.45	113	0.239	89.54
T0795TS006_3-D1.rsa	27	0.434	0.12	0.446	112	0.241	84.36
T0795TS420_5-D1.rsa	26.923	0.378	0.135	0.486	144	0.187	58.85
T0795TS403_4-D1.rsa	26.923	0.412	0.128	0.459	136	0.198	58.07

T0795TS310_5-D1.rsa	26.857	0.423	0.017	0.559	226	0.119	45.42
T0795TS268_5-D1.rsa	26.857	0.532	0.017	0.45	182	0.148	50.99
T0795TS436_5-D1.rsa	26.531	0.378	0.11	0.512	191	0.139	85.69
T0795TS465_2-D1.rsa	26.471	0.383	0.074	0.543	132	0.201	66.56
T0795TS157_3-D1.rsa	26.4	0.018	0.362	0.62	204	0.129	52.13
T0795TS492_1-D1.rsa	26.4	0.012	0.365	0.623	205	0.129	54.26
T0795TS041_2-D1.rsa	26.286	0.495	0.027	0.478	193	0.136	37.81
T0795TS067_3-D1.rsa	26.263	0.198	0.181	0.622	179	0.147	62.67
T0795TS157_5-D1.rsa	26.027	0.407	0.116	0.477	177	0.147	81.74
T0795TS439_3-D1.rsa	26	0.406	0.12	0.474	119	0.218	89.44
T0795TS204_2-D1.rsa	26	0.442	0.116	0.442	111	0.234	88.94
T0795TS011_5-D1.rsa	26	0.466	0.116	0.418	105	0.248	87.45
T0795TS197_5-D1.rsa	25.85	0.378	0.083	0.539	201	0.129	83.96
T0795TS042_3-D1.rsa	25.85	0.367	0.102	0.531	198	0.131	83.75
T0795TS064_4-D1.rsa	25.85	0.381	0.107	0.512	191	0.135	84.72
T0795TS310_3-D1.rsa	25.714	0.401	0.02	0.579	234	0.11	42.08
T0795TS479_5-D1.rsa	25.714	0.505	0.052	0.443	179	0.144	28.22
T0795TS080_5-D1.rsa	25.641	0.318	0.084	0.598	177	0.145	53.3
T0795TS414_3-D1.rsa	25.641	0.392	0.122	0.486	144	0.178	57.55
T0795TS438_1-D1.rsa	25.641	0.378	0.135	0.486	144	0.178	56.34
T0795TS184_2-D1.rsa	25.581	0.32	0.16	0.521	88	0.291	69.08
T0795TS162_3-D1.rsa	25.424	0	0.29	0.71	152	0.167	61.8
T0795TS049_5-D1.rsa	25.263	0.453	0.051	0.496	117	0.216	56.89
T0795TS433_5-D1.rsa	25.253	0.267	0.205	0.528	152	0.166	70.75
T0795TS184_3-D1.rsa	25.253	0.229	0.177	0.594	171	0.148	58.51
T0795TS173_5-D1.rsa	25.253	0.243	0.219	0.538	155	0.163	70.66
T0795TS064_5-D1.rsa	25.143	0.522	0.015	0.463	187	0.134	44.8
T0795TS268_1-D1.rsa	25	0.386	0.112	0.502	126	0.198	88.94
T0795TS277_2-D1.rsa	25	0.402	0.124	0.474	119	0.21	89.34
T0795TS258_5-D1.rsa	25	0.446	0.12	0.434	109	0.229	88.45
T0795TS340_1-D1.rsa	25	0.379	0.091	0.531	129	0.194	69.56
T0795TS360_1-D1.rsa	25	0.395	0.091	0.514	125	0.2	65.44
T0795TS296_5-D1.rsa	25	0.462	0.104	0.434	109	0.229	86.45
T0795TS050_1-D1.rsa	25	0.412	0.103	0.486	118	0.212	69.56
T0795TS011_2-D1.rsa	25	0.342	0.016	0.642	156	0.16	18.89
T0795TS454_1-D1.rsa	25	0	0.096	0.904	103	0.243	28.07
T0795TS251_1-D1.rsa	24.8	0.012	0.207	0.781	257	0.096	55.93
T0795TS263_5-D1.rsa	24.752	0.206	0.1	0.695	223	0.111	70.79
T0795TS277_5-D1.rsa	24.59	0.029	0.386	0.586	123	0.2	66.92
T0795TS499_1-D1.rsa	24.571	0.525	0.022	0.453	183	0.134	43.81
T0795TS368_2-D1.rsa	24.561	0.406	0.082	0.511	112	0.219	77.74
T0795TS228_4-D1.rsa	24.49	0.397	0.126	0.477	178	0.138	88.33
T0795TS282_2-D1.rsa	24.359	0.412	0.084	0.503	149	0.163	59.72
T0795TS336_2-D1.rsa	24.359	0.365	0.145	0.49	145	0.168	57.99
T0795TS014_2-D1.rsa	24.359	0.378	0.132	0.49	145	0.168	59.2
T0795TS340_3-D1.rsa	24.359	0.375	0.139	0.486	144	0.169	58.85
T0795TS434_1-D1.rsa	24.359	0.399	0.132	0.47	139	0.175	59.12

T0795TS403_2-D1.rsa	24.359	0.449	0.081	0.47	139	0.175	58.94
T0795TS465_4-D1.rsa	24.242	0.233	0.212	0.556	160	0.152	69.1
T0795TS171_4-D1.rsa	24.242	0.257	0.215	0.528	152	0.159	70.23
T0795TS483_4-D1.rsa	24.074	0.388	0.072	0.539	246	0.098	53.86
T0795TS204_5-D1.rsa	24	0.443	0.03	0.527	213	0.113	43.69
T0795TS203_4-D1.rsa	24	0.507	0.01	0.483	195	0.123	44.43
T0795TS118_4-D1.rsa	24	0.493	0.03	0.478	193	0.124	38.06
T0795TS011_1-D1.rsa	24	0	0.014	0.986	136	0.176	16.49
T0795TS326_1-D1.rsa	23.81	0.354	0.105	0.542	202	0.118	85.07
T0795TS336_1-D1.rsa	23.81	0.235	0.205	0.56	131	0.182	59.72
T0795TS120_5-D1.rsa	23.729	0.023	0.206	0.771	165	0.144	53.51
T0795TS336_3-D1.rsa	23.729	0.019	0.308	0.673	144	0.165	64.95
T0795TS258_2-D1.rsa	23.729	0.009	0.313	0.678	145	0.164	63.44
T0795TS144_5-D1.rsa	23.729	0	0.304	0.696	149	0.159	62.73
T0795TS445_5-D1.rsa	23.684	0.163	0.045	0.792	263	0.09	35.62
T0795TS133_2-D1.rsa	23.529	0.329	0.078	0.593	144	0.163	70
T0795TS260_3-D1.rsa	23.529	0.337	0.095	0.568	138	0.171	68
T0795TS008_3-D1.rsa	23.529	0.276	0.095	0.63	153	0.154	49.11
T0795TS445_4-D1.rsa	23.457	0.399	0.094	0.507	231	0.102	66.67
T0795TS080_1-D1.rsa	23.429	0.505	0.015	0.48	194	0.121	52.23
T0795TS338_4-D1.rsa	23.429	0.49	0.005	0.505	204	0.115	44.86
T0795TS347_5-D1.rsa	23.377	0.411	0.112	0.478	107	0.218	80.83
T0795TS260_2-D1.rsa	23.377	0.379	0.121	0.5	112	0.209	77.14
T0795TS368_5-D1.rsa	23.256	0.382	0.113	0.505	143	0.163	72.79
T0795TS064_3-D1.rsa	23.232	0.247	0.215	0.538	155	0.15	73.52
T0795TS381_4-D1.rsa	23.232	0.253	0.219	0.528	152	0.153	69.27
T0795TS465_1-D1.rsa	23.232	0.26	0.215	0.524	151	0.154	70.49
T0795TS401_2-D1.rsa	23.2	0.015	0.371	0.614	202	0.115	50.99
T0795TS034_5-D1.rsa	23.077	0.258	0.197	0.546	250	0.092	46.56
T0795TS347_2-D1.rsa	23.077	0.389	0.088	0.524	155	0.149	53.99
T0795TS433_4-D1.rsa	23.077	0.389	0.122	0.49	145	0.159	59.12
T0795TS173_2-D1.rsa	22.857	0.527	0	0.473	191	0.12	45.42
T0795TS368_4-D1.rsa	22.84	0.41	0.09	0.5	228	0.1	77.37
T0795TS038_2-D1.rsa	22.84	0.458	0.096	0.445	203	0.113	53.03
T0795TS042_2-D1.rsa	22.286	0.423	0.027	0.55	222	0.1	40.16
T0795TS054_2-D1.rsa	22.286	0.512	0.02	0.468	189	0.118	42.76
T0795TS414_4-D1.rsa	22.286	0.495	0.042	0.463	187	0.119	38.24
T0795TS499_4-D1.rsa	22.222	0.247	0.184	0.569	164	0.136	59.98
T0795TS454_4-D1.rsa	22.222	0.26	0.205	0.535	154	0.144	64.67
T0795TS008_5-D1.rsa	22.078	0.379	0.116	0.504	113	0.195	83.09
T0795TS169_1-D1.rsa	22.078	0.384	0.125	0.491	110	0.201	77.86
T0795TS345_5-D1.rsa	22.034	0	0.173	0.827	177	0.124	59.46
T0795TS169_3-D1.rsa	22.034	0.019	0.252	0.729	156	0.141	60.75
T0795TS132_4-D1.rsa	22.034	0.009	0.318	0.673	144	0.153	65.54
T0795TS301_5-D1.rsa	21.795	0.372	0.118	0.51	151	0.144	57.12
T0795TS439_4-D1.rsa	21.714	0.567	0.022	0.411	166	0.131	41.83
T0795TS442_1-D1.rsa	21.605	0.421	0.09	0.489	223	0.097	77.54

T0795TS034_4-D1.rsa	21.605	0.43	0.09	0.48	219	0.099	77.04
T0795TS300_5-D1.rsa	21.605	0.43	0.07	0.5	228	0.095	72.68
T0795TS358_1-D1.rsa	21.311	0.029	0.429	0.543	114	0.187	63.31
T0795TS333_1-D1.rsa	21.277	0.315	0.136	0.549	141	0.151	76.17
T0795TS237_4-D1.rsa	21.143	0.166	0.017	0.817	330	0.064	18.01
T0795TS263_3-D1.rsa	21.053	0.434	0.02	0.546	249	0.085	58.66
T0795TS041_5-D1.rsa	21.053	0.482	0.024	0.493	225	0.094	64.2
T0795TS345_2-D1.rsa	20.93	0.346	0.113	0.541	153	0.137	72.08
T0795TS328_5-D1.rsa	20.93	0.417	0.11	0.473	134	0.156	82.86
T0795TS133_4-D1.rsa	20.93	0.378	0.11	0.512	145	0.144	70.14
T0795TS401_1-D1.rsa	20.571	0.374	0.05	0.577	233	0.088	28.59
T0795TS499_2-D1.rsa	20.513	0.331	0.091	0.578	171	0.12	50.95
T0795TS063_3-D1.rsa	20.395	0.423	0.029	0.548	250	0.082	63.76
T0795TS153_5-D1.rsa	20.37	0.386	0.072	0.542	247	0.082	71.08
T0795TS326_2-D1.rsa	20.37	0.382	0.103	0.515	235	0.087	62.25
T0795TS228_1-D1.rsa	20.37	0.404	0.101	0.496	226	0.09	67.88
T0795TS349_1-D1.rsa	20.37	0.432	0.094	0.474	216	0.094	71.3
T0795TS338_3-D1.rsa	20.339	0	0.308	0.692	148	0.137	65.07
T0795TS120_4-D1.rsa	20.339	0.033	0.318	0.65	139	0.146	65.54
T0795TS417_2-D1.rsa	20	0.262	0.116	0.622	285	0.07	62.56
T0795TS118_1-D1.rsa	19.767	0.325	0.11	0.565	160	0.124	66.34
T0795TS499_3-D1.rsa	19.767	0.382	0.099	0.519	147	0.134	63.25
T0795TS235_1-D1.rsa	19.753	0.419	0.09	0.491	224	0.088	72.08
T0795TS228_2-D1.rsa	19.753	0.404	0.092	0.504	230	0.086	68.93
T0795TS038_5-D1.rsa	19.753	0.441	0.09	0.469	214	0.092	33.77
T0795TS368_1-D1.rsa	19.737	0.406	0.029	0.566	258	0.076	61.4
T0795TS300_2-D1.rsa	19.737	0.452	0.02	0.529	241	0.082	66.45
T0795TS425_3-D1.rsa	19.737	0.262	0.117	0.62	206	0.096	58.28
T0795TS162_1-D1.rsa	19.672	0.029	0.352	0.619	130	0.151	56.09
T0795TS282_5-D1.rsa	19.298	0.443	0.082	0.475	104	0.186	79.91
T0795TS042_5-D1.rsa	19.136	0.388	0.096	0.515	235	0.081	67.33
T0795TS153_3-D1.rsa	19.136	0.454	0.088	0.458	209	0.092	72.24
T0795TS317_1-D1.rsa	19.118	0.383	0.107	0.51	124	0.154	64.89
T0795TS116_2-D1.rsa	19.079	0.434	0.015	0.55	251	0.076	62.99
T0795TS433_3-D1.rsa	18.644	0.019	0.192	0.79	169	0.11	65.3
T0795TS110_2-D1.rsa	18.605	0.399	0.113	0.488	138	0.135	81.01
T0795TS335_4-D1.rsa	18.605	0.396	0.117	0.488	138	0.135	82.69
T0795TS349_4-D1.rsa	18.519	0.399	0.079	0.522	238	0.078	64.84
T0795TS117_3-D1.rsa	18.519	0.408	0.099	0.493	225	0.082	70.92
T0795TS197_4-D1.rsa	18.421	0.441	0.029	0.531	242	0.076	62.88
T0795TS457_4-D1.rsa	18.182	0.384	0.129	0.487	109	0.167	75.71
T0795TS436_1-D1.rsa	17.763	0.436	0.024	0.539	246	0.072	56.58
T0795TS157_4-D1.rsa	17.714	0.505	0.022	0.473	191	0.093	41.15
T0795TS277_1-D1.rsa	17.544	0.32	0.073	0.607	133	0.132	70.89
T0795TS439_2-D1.rsa	17.544	0.416	0.091	0.493	108	0.162	81.16
T0795TS049_4-D1.rsa	17.544	0.447	0.087	0.466	102	0.172	81.39
T0795TS133_1-D1.rsa	17.442	0.389	0.117	0.495	140	0.125	81.01

T0795TS132_1-D1.rsa	17.442	0.417	0.12	0.463	131	0.133	77.21
T0795TS132_5-D1.rsa	17.442	0.403	0.117	0.481	136	0.128	82.07
T0795TS258_3-D1.rsa	17.021	0.257	0.051	0.693	178	0.096	48.05
T0795TS155_5-D1.rsa	16.667	0.421	0.096	0.482	220	0.076	79.86
T0795TS417_1-D1.rsa	16.447	0.419	0.029	0.553	252	0.065	65.57
T0795TS442_4-D1.rsa	16.447	0.412	0.024	0.564	257	0.064	60.14
T0795TS448_4-D1.rsa	16.447	0.465	0.024	0.511	233	0.071	61.73
T0795TS097_3-D1.rsa	16.279	0.41	0.12	0.47	133	0.122	77.39
T0795TS118_3-D1.rsa	16.279	0.403	0.124	0.473	134	0.121	82.86
T0795TS251_2-D1.rsa	16.154	0.245	0.194	0.561	257	0.063	54.72
T0795TS442_3-D1.rsa	16.154	0.264	0.229	0.507	232	0.07	73.89
T0795TS448_5-D1.rsa	16.154	0.293	0.236	0.472	216	0.075	70.5
T0795TS155_4-D1.rsa	15.789	0.474	0.011	0.515	235	0.067	63.65
T0795TS436_4-D1.rsa	15.789	0.349	0.096	0.554	184	0.086	98.49
T0795TS436_2-D1.rsa	15.789	0.31	0.136	0.554	184	0.086	96.31
T0795TS116_3-D1.rsa	15.789	0.343	0.13	0.527	175	0.09	98.87
T0795TS160_1-D1.rsa	15.789	0.331	0.127	0.542	180	0.088	97.97
T0795TS333_4-D1.rsa	15.789	0.434	0.082	0.484	106	0.149	79.57
T0795TS132_2-D1.rsa	15.789	0.461	0.082	0.457	100	0.158	80.71
T0795TS173_4-D1.rsa	14.894	0.261	0.035	0.704	181	0.082	48.73
T0795TS425_2-D1.rsa	14.894	0.327	0.175	0.498	128	0.116	81.52
T0795TS042_4-D1.rsa	14.474	0.428	0.015	0.557	254	0.057	58.33
T0795TS038_3-D1.rsa	14.474	0.253	0.051	0.696	231	0.063	53.99
T0795TS160_2-D1.rsa	14.474	0.337	0.117	0.545	181	0.08	99.25
T0795TS401_4-D1.rsa	14.474	0.337	0.123	0.539	179	0.081	98.95
T0795TS044_5-D1.rsa	14.474	0.328	0.142	0.53	176	0.082	98.8
T0795TS197_2-D1.rsa	14.474	0.343	0.157	0.5	166	0.087	99.02
T0795TS032_1-D1.rsa	14.035	0.443	0.096	0.461	101	0.139	81.39
T0795TS360_4-D1.rsa	14.035	0.438	0.105	0.457	100	0.14	80.59
T0795TS153_1-D1.rsa	13.846	0.26	0.227	0.513	235	0.059	78
T0795TS117_5-D1.rsa	13.846	0.262	0.227	0.511	234	0.059	77.44
T0795TS116_5-D1.rsa	13.846	0.266	0.229	0.504	231	0.06	76.22
T0795TS155_1-D1.rsa	13.158	0.325	0.105	0.569	189	0.07	90.74
T0795TS237_5-D1.rsa	13.077	0.277	0.177	0.546	250	0.052	70.61
T0795TS118_5-D1.rsa	12.766	0.3	0.179	0.521	134	0.095	85.31
T0795TS457_3-D1.rsa	12.766	0.307	0.171	0.521	134	0.095	81.03
T0795TS345_3-D1.rsa	12.766	0.339	0.179	0.482	124	0.103	82
T0795TS041_1-D1.rsa	12.766	0.304	0.191	0.506	130	0.098	82.3
T0795TS338_5-D1.rsa	12.766	0.35	0.191	0.459	118	0.108	84.73
T0795TS420_3-D1.rsa	12.766	0.327	0.191	0.482	124	0.103	78.7
T0795TS228_3-D1.rsa	12.308	0.26	0.245	0.496	227	0.054	69.94
T0795TS454_2-D1.rsa	10.638	0.346	0.156	0.498	128	0.083	77.14
T0760TS381_1-D1.rsa	92.105	0.054	0	0.946	105	0.877	14.41
T0760TS184_4-D1.rsa	90.141	0.411	0	0.589	99	0.911	16.83
T0760TS133_5-D1.rsa	79.412	0.294	0	0.706	89	0.892	33.53
T0760TS073_1-D1.rsa	79.412	0.302	0	0.698	88	0.902	32.74
T0760TS008_2-D1.rsa	76.316	0.054	0	0.946	105	0.727	13.96

T0760TS212_1-D1.rsa	73.529	0.383	0.025	0.593	144	0.511	11
T0760TS008_5-D1.rsa	72	0.094	0.043	0.862	119	0.605	16.85
T0760TS414_1-D1.rsa	71.831	0.226	0.071	0.702	118	0.609	20.03
T0760TS277_4-D1.rsa	67.308	0.273	0	0.727	120	0.561	54.59
T0760TS237_3-D1.rsa	64.286	0	0.267	0.733	96	0.67	49.81
T0760TS300_3-D1.rsa	64.286	0.015	0.397	0.588	77	0.835	54.01
T0760TS041_2-D1.rsa	63.38	0.286	0	0.714	120	0.528	15.06
T0760TS117_1-D1.rsa	60.465	0.231	0.03	0.739	99	0.611	45.52
T0760TS381_3-D1.rsa	59.615	0.291	0.061	0.648	107	0.557	75.95
T0760TS335_3-D1.rsa	59.615	0.273	0.097	0.63	104	0.573	53.48
T0760TS008_3-D1.rsa	59.615	0.248	0.085	0.667	110	0.542	47.15
T0760TS038_5-D1.rsa	57.895	0.225	0.054	0.721	80	0.724	35.36
T0760TS022_2-D1.rsa	57.692	0.297	0.109	0.594	98	0.589	77.69
T0760TS279_1-D1.rsa	57.143	0.015	0.374	0.611	80	0.714	56.68
T0760TS349_5-D1.rsa	55.769	0.267	0.036	0.697	115	0.485	69.78
T0760TS448_4-D1.rsa	55.769	0.182	0.115	0.703	116	0.481	64.87
T0760TS193_2-D1.rsa	55.769	0.273	0.085	0.642	106	0.526	66.93
T0760TS171_1-D1.rsa	55.769	0.297	0.139	0.564	93	0.6	60.6
T0760TS184_2-D1.rsa	55.263	0.153	0.036	0.811	90	0.614	32.21
T0760TS133_1-D1.rsa	53.846	0.261	0.139	0.6	99	0.544	77.37
T0760TS041_1-D1.rsa	52	0.043	0.217	0.739	102	0.51	13.04
T0760TS011_2-D1.rsa	51.923	0.182	0.097	0.721	119	0.436	15.19
T0760TS300_5-D1.rsa	51.316	0.557	0	0.443	147	0.349	10.54
T0760TS452_4-D1.rsa	50	0.309	0.048	0.642	106	0.472	17.56
T0760TS492_3-D1.rsa	47.887	0	0.089	0.911	153	0.313	11.38
T0760TS345_5-D1.rsa	47.541	0.019	0	0.981	206	0.231	10.7
T0760TS228_5-D1.rsa	46.552	0	0.098	0.902	129	0.361	59.97
T0760TS349_3-D1.rsa	46.154	0.261	0	0.739	122	0.378	50.48
T0760TS420_5-D1.rsa	45.833	0	0.241	0.759	82	0.559	59.72
T0760TS448_5-D1.rsa	41.379	0	0.091	0.909	130	0.318	56.64
T0760TS436_5-D1.rsa	41.379	0	0.084	0.916	131	0.316	44.06
T0760TS228_2-D1.rsa	41.379	0	0.126	0.874	125	0.331	59.09
T0760TS335_2-D1.rsa	41.379	0.014	0.112	0.874	125	0.331	57.17
T0760TS011_5-D1.rsa	40.625	0.088	0.235	0.676	92	0.442	59.19
T0760TS479_1-D1.rsa	39.773	0.071	0.301	0.628	186	0.214	16.64
T0760TS008_4-D1.rsa	39.286	0	0.16	0.84	110	0.357	55.92
T0760TS436_4-D1.rsa	38.889	0.286	0.085	0.629	134	0.29	51.14
T0760TS300_4-D1.rsa	38.889	0.413	0.108	0.479	102	0.381	70.69
T0760TS156_3-D1.rsa	38.793	0.664	0	0.336	86	0.451	54.69
T0760TS228_4-D1.rsa	38.596	0.338	0.087	0.575	126	0.306	60.39
T0760TS073_2-D1.rsa	38.596	0.309	0.147	0.544	118	0.327	57.95
T0760TS008_1-D1.rsa	37.931	0.021	0.091	0.888	127	0.299	60.31
T0760TS038_3-D1.rsa	37.931	0.021	0.182	0.797	114	0.333	53.5
T0760TS237_1-D1.rsa	37.719	0.258	0.157	0.585	127	0.297	62.79
T0760TS171_2-D1.rsa	37.5	0.362	0.075	0.563	120	0.313	69.67
T0760TS237_4-D1.rsa	37.5	0.352	0.089	0.559	119	0.315	71.95
T0760TS022_1-D1.rsa	37.5	0.385	0.089	0.526	112	0.335	68.78

T0760TS381_4-D1.rsa	37.5	0.074	0.176	0.75	102	0.368	47.61
T0760TS171_5-D1.rsa	36.842	0.249	0.203	0.548	119	0.31	63.83
T0760TS263_4-D1.rsa	36.364	0.229	0.007	0.764	220	0.165	10.07
T0760TS228_3-D1.rsa	36.364	0.068	0.419	0.514	152	0.239	36.82
T0760TS133_4-D1.rsa	35.593	0	0.407	0.593	127	0.28	45.91
T0760TS436_2-D1.rsa	35.227	0.057	0.436	0.507	150	0.235	42.48
T0760TS216_5-D1.rsa	35	0.018	0.272	0.711	81	0.432	46.49
T0760TS041_3-D1.rsa	34.884	0.361	0.195	0.444	75	0.465	70.41
T0760TS277_3-D1.rsa	34.722	0.38	0.108	0.512	109	0.319	71.95
T0760TS414_4-D1.rsa	34.722	0.39	0.085	0.526	112	0.31	67.64
T0760TS216_1-D1.rsa	34.375	0.015	0.463	0.522	71	0.484	70.96
T0760TS277_1-D1.rsa	34.286	0.303	0.158	0.538	126	0.272	63.89
T0760TS414_5-D1.rsa	33.803	0.173	0	0.827	139	0.243	19.87
T0760TS479_5-D1.rsa	33.333	0.267	0.16	0.573	165	0.202	60.24
T0760TS216_2-D1.rsa	32.813	0.015	0.39	0.596	81	0.405	71.88
T0760TS452_2-D1.rsa	32.759	0.582	0	0.418	107	0.306	56.05
T0760TS381_5-D1.rsa	32.632	0.492	0.03	0.479	113	0.289	49.15
T0760TS268_2-D1.rsa	32	0.051	0.413	0.536	74	0.432	70.65
T0760TS011_3-D1.rsa	31.897	0.602	0	0.398	102	0.313	56.25
T0760TS156_1-D1.rsa	31.579	0.295	0.171	0.535	116	0.272	59.1
T0760TS184_5-D1.rsa	31.395	0.284	0.178	0.538	91	0.345	73.22
T0760TS420_2-D1.rsa	30.476	0.333	0.201	0.466	109	0.28	70.83
T0760TS237_2-D1.rsa	29.87	0.362	0.121	0.518	116	0.258	77.5
T0760TS499_5-D1.rsa	29.703	0.274	0.134	0.592	190	0.156	73.83
T0760TS117_3-D1.rsa	29.524	0.35	0.218	0.432	101	0.292	71.37
T0760TS492_4-D1.rsa	29.524	0.329	0.179	0.491	115	0.257	68.7
T0760TS011_1-D1.rsa	29.474	0.551	0.064	0.386	91	0.324	59.22
T0760TS251_1-D1.rsa	29.143	0.47	0.022	0.507	205	0.142	38.55
T0760TS156_2-D1.rsa	29.07	0.296	0.166	0.538	91	0.319	69.97
T0760TS349_1-D1.rsa	28.947	0.258	0.097	0.645	140	0.207	60.14
T0760TS133_2-D1.rsa	28.571	0.329	0.175	0.496	116	0.246	62.29
T0760TS038_4-D1.rsa	28.571	0.372	0.201	0.427	100	0.286	71.47
T0760TS251_4-D1.rsa	28.283	0.25	0.194	0.556	160	0.177	69.36
T0760TS335_4-D1.rsa	28.205	0.412	0.128	0.459	136	0.207	59.38
T0760TS251_2-D1.rsa	28	0.243	0.02	0.738	298	0.094	25.31
T0760TS038_1-D1.rsa	28	0.058	0.413	0.529	73	0.384	68.84
T0760TS452_1-D1.rsa	28	0.065	0.283	0.652	90	0.311	65.04
T0760TS335_5-D1.rsa	27.619	0.363	0.15	0.487	114	0.242	70.62
T0760TS420_4-D1.rsa	27.273	0.415	0.116	0.469	105	0.26	78.57
T0760TS349_4-D1.rsa	27.119	0.019	0.318	0.664	142	0.191	63.9
T0760TS479_3-D1.rsa	27	0.414	0.124	0.462	116	0.233	89.74
T0760TS499_2-D1.rsa	26.857	0.485	0.04	0.475	192	0.14	21.23
T0760TS011_4-D1.rsa	26.667	0.406	0.167	0.427	100	0.267	69.23
T0760TS345_1-D1.rsa	26.471	0.366	0.062	0.572	139	0.19	65.67
T0760TS263_2-D1.rsa	26.471	0.374	0.099	0.527	128	0.207	66.11
T0760TS251_5-D1.rsa	26.263	0.149	0.156	0.694	200	0.131	51.3
T0760TS300_2-D1.rsa	26.23	0.038	0.429	0.533	112	0.234	63.06

T0760TS436_1-D1.rsa	26	0.402	0.124	0.474	119	0.218	90.54
T0760TS216_4-D1.rsa	26	0.454	0.124	0.422	106	0.245	87.35
T0760TS345_4-D1.rsa	25.974	0.379	0.103	0.518	116	0.224	79.41
T0760TS492_5-D1.rsa	25.714	0.333	0.201	0.466	109	0.236	70.3
T0760TS228_1-D1.rsa	25.641	0.392	0.118	0.49	145	0.177	56.34
T0760TS479_4-D1.rsa	25.253	0.247	0.181	0.573	165	0.153	68.23
T0760TS277_5-D1.rsa	25.253	0.215	0.188	0.597	172	0.147	67.36
T0760TS454_1-D1.rsa	25.143	0.498	0	0.502	203	0.124	37.93
T0760TS448_3-D1.rsa	25	0.391	0.095	0.514	125	0.2	69.56
T0760TS345_3-D1.rsa	24.675	0.402	0.121	0.478	107	0.231	81.79
T0760TS216_3-D1.rsa	24.419	0.349	0.219	0.432	73	0.335	73.37
T0760TS414_2-D1.rsa	24.359	0.395	0.142	0.463	137	0.178	57.81
T0760TS263_3-D1.rsa	24.242	0.219	0.201	0.58	167	0.145	68.32
T0760TS263_1-D1.rsa	24	0.448	0.022	0.53	214	0.112	44.31
T0760TS499_3-D1.rsa	24	0.442	0.108	0.45	113	0.212	88.64
T0760TS479_2-D1.rsa	23.729	0.019	0.238	0.743	159	0.149	55.84
T0760TS448_1-D1.rsa	23.377	0.362	0.129	0.509	114	0.205	76.19
T0760TS263_5-D1.rsa	22.286	0.473	0.022	0.505	204	0.109	37.93
T0760TS492_1-D1.rsa	22.286	0.577	0.022	0.401	162	0.138	42.76
T0760TS133_3-D1.rsa	22.059	0.395	0.111	0.494	120	0.184	67.33
T0760TS206_1-D1.rsa	22.059	0.453	0.099	0.449	109	0.202	69.33
T0760TS346_1-D1.rsa	21.795	0.395	0.125	0.48	142	0.153	59.2
T0760TS237_5-D1.rsa	20.93	0.375	0.117	0.509	144	0.145	71.11
T0760TS300_1-D1.rsa	19.767	0.41	0.11	0.481	136	0.145	66.96
T0760TS268_1-D1.rsa	19.298	0.315	0.119	0.566	124	0.156	61.99
T0760TS420_1-D1.rsa	19.298	0.457	0.082	0.461	101	0.191	80.71
T0760TS041_4-D1.rsa	19.079	0.419	0.029	0.553	252	0.076	64.86
T0760TS117_4-D1.rsa	17.544	0.443	0.091	0.466	102	0.172	80.59
T0760TS038_2-D1.rsa	17.544	0.447	0.1	0.452	99	0.177	81.05
T0760TS277_2-D1.rsa	17.442	0.392	0.099	0.509	144	0.121	81.36
T0760TS381_2-D1.rsa	17.021	0.311	0.171	0.518	133	0.128	78.99
T0760TS452_3-D1.rsa	16.883	0.375	0.121	0.504	113	0.149	76.31
T0760TS349_2-D1.rsa	16.279	0.396	0.081	0.523	148	0.11	75.35
T0760TS251_3-D1.rsa	15.789	0.328	0.117	0.554	184	0.086	98.57
T0760TS335_1-D1.rsa	15.789	0.438	0.091	0.47	103	0.153	81.62
T0760TS171_3-D1.rsa	12.766	0.354	0.14	0.506	130	0.098	85.12
T0760TS492_2-D1.rsa	12.766	0.327	0.191	0.482	124	0.103	84.73
T0760TS448_2-D1.rsa	10.638	0.292	0.132	0.576	148	0.072	75
T0760TS499_1-D1.rsa	10.638	0.315	0.191	0.494	127	0.084	83.85
T0760TS022_5-D1.rsa	10.638	0.304	0.195	0.502	129	0.082	86.09
T0760TS436_3-D1.rsa	8.511	0.315	0.187	0.498	128	0.066	82.1
T0760TS420_3-D1.rsa	6.383	0.327	0.187	0.486	125	0.051	85.99
T0766TS381_2-D1.rsa	71.429	0.635	0	0.365	46	1.553	16.67
T0766TS237_2-D1.rsa	71.053	0.396	0	0.604	67	1.06	13.74
T0766TS145_2-D1.rsa	68.421	0.477	0	0.523	58	1.18	16.44
T0766TS171_4-D1.rsa	67.647	0.571	0	0.429	54	1.253	27.58
T0766TS263_2-D1.rsa	67.308	0.648	0	0.352	58	1.16	15.03

T0766TS263_1-D1.rsa	64.706	0.706	0	0.294	37	1.749	28.77
T0766TS335_3-D1.rsa	64.286	0.317	0.254	0.429	54	1.19	13.29
T0766TS171_2-D1.rsa	64	0.13	0.174	0.696	96	0.667	13.95
T0766TS479_4-D1.rsa	60.526	0.369	0	0.631	70	0.865	18.02
T0766TS263_3-D1.rsa	60.526	0.577	0	0.423	47	1.288	20.5
T0766TS335_2-D1.rsa	60.526	0.333	0.148	0.519	56	1.081	18.52
T0766TS160_5-D1.rsa	58.333	0	0.611	0.389	42	1.389	63.89
T0766TS492_5-D1.rsa	58.333	0.019	0.537	0.444	48	1.215	25.23
T0766TS156_5-D1.rsa	57.143	0	0.336	0.664	87	0.657	14.5
T0766TS160_4-D1.rsa	57.143	0.357	0.127	0.516	65	0.879	20.64
T0766TS349_4-D1.rsa	55.882	0.667	0	0.333	42	1.331	44.05
T0766TS492_4-D1.rsa	55.882	0.595	0.016	0.389	49	1.14	14.09
T0766TS156_3-D1.rsa	53.521	0.5	0.048	0.452	76	0.704	20.99
T0766TS479_5-D1.rsa	52.632	0.243	0.018	0.739	82	0.642	18.92
T0766TS420_2-D1.rsa	51.923	0.606	0	0.394	65	0.799	18.67
T0766TS349_2-D1.rsa	50	0.651	0	0.349	44	1.136	36.91
T0766TS011_1-D1.rsa	50	0.468	0	0.532	59	0.847	18.02
T0766TS216_5-D1.rsa	50	0.722	0	0.278	35	1.429	21.03
T0766TS171_5-D1.rsa	49.296	0.482	0.077	0.44	74	0.666	24.04
T0766TS228_5-D1.rsa	48.438	0.059	0.338	0.603	82	0.591	12.68
T0766TS251_3-D1.rsa	48.438	0.103	0.426	0.471	64	0.757	12.87
T0766TS041_2-D1.rsa	48.438	0.118	0.346	0.537	73	0.664	15.62
T0766TS011_4-D1.rsa	47.5	0	0.386	0.614	70	0.679	20.61
T0766TS133_4-D1.rsa	47.368	0.459	0	0.541	60	0.789	18.69
T0766TS381_1-D1.rsa	47.368	0.269	0.481	0.25	27	1.754	48.15
T0766TS171_3-D1.rsa	46.875	0	0.346	0.654	89	0.527	13.05
T0766TS279_1-D1.rsa	46.875	0	0.529	0.471	64	0.732	14.71
T0766TS349_5-D1.rsa	46.575	0.702	0	0.298	76	0.613	28.14
T0766TS156_1-D1.rsa	46.429	0	0.26	0.74	97	0.479	16.79
T0766TS251_1-D1.rsa	46.429	0.015	0.344	0.641	84	0.553	17.94
T0766TS117_4-D1.rsa	45	0.018	0.474	0.509	58	0.776	23.46
T0766TS038_1-D1.rsa	45	0.035	0.456	0.509	58	0.776	19.96
T0766TS381_3-D1.rsa	45	0	0.289	0.711	81	0.556	14.04
T0766TS335_1-D1.rsa	45	0	0.447	0.553	63	0.714	14.91
T0766TS499_4-D1.rsa	44.231	0.345	0.182	0.473	78	0.567	19.46
T0766TS050_1-D1.rsa	44.118	0.563	0	0.437	55	0.802	24.41
T0766TS277_1-D1.rsa	43.75	0.018	0.309	0.673	74	0.591	20.68
T0766TS133_1-D1.rsa	43.75	0.018	0.391	0.591	65	0.673	20.68
T0766TS008_4-D1.rsa	42.857	0.069	0.351	0.58	76	0.564	13.36
T0766TS452_1-D1.rsa	42.857	0.317	0.111	0.571	72	0.595	17.66
T0766TS268_5-D1.rsa	42.857	0.571	0	0.429	54	0.794	16.86
T0766TS156_2-D1.rsa	42.5	0.018	0.377	0.605	69	0.616	38.6
T0766TS335_4-D1.rsa	42.5	0.035	0.509	0.456	52	0.817	29.39
T0766TS216_1-D1.rsa	42.5	0	0.535	0.465	53	0.802	16.89
T0766TS073_2-D1.rsa	42.5	0	0.544	0.456	52	0.817	17.11
T0766TS381_5-D1.rsa	41.667	0.037	0.528	0.435	47	0.887	25.69
T0766TS345_2-D1.rsa	41.176	0.77	0	0.23	29	1.42	47.82

T0766TS156_4-D1.rsa	41.071	0.198	0.087	0.714	90	0.456	18.65
T0766TS038_4-D1.rsa	40.625	0.088	0.279	0.632	86	0.472	12.68
T0766TS263_5-D1.rsa	40.385	0.727	0	0.273	45	0.897	14.24
T0766TS268_2-D1.rsa	40	0	0.474	0.526	60	0.667	16.45
T0766TS452_4-D1.rsa	40	0	0.377	0.623	71	0.563	17.54
T0766TS268_4-D1.rsa	40	0	0.561	0.439	50	0.8	16.67
T0766TS300_3-D1.rsa	39.583	0	0.436	0.564	62	0.638	24.09
T0766TS277_4-D1.rsa	39.535	0.41	0.045	0.545	73	0.542	21.64
T0766TS038_3-D1.rsa	39.286	0.092	0.275	0.634	83	0.473	12.6
T0766TS251_4-D1.rsa	38.356	0.761	0	0.239	61	0.629	42.84
T0766TS454_5-D1.rsa	37.5	0.009	0.421	0.57	65	0.577	50
T0766TS454_1-D1.rsa	37.5	0	0.4	0.6	66	0.568	32.5
T0766TS171_1-D1.rsa	37.5	0.081	0.324	0.596	81	0.463	12.13
T0766TS237_5-D1.rsa	37.5	0	0.368	0.632	72	0.521	19.3
T0766TS345_1-D1.rsa	37.5	0.151	0.381	0.468	59	0.636	34.52
T0766TS410_3-D1.rsa	37.5	0.018	0.263	0.719	82	0.457	19.08
T0766TS499_2-D1.rsa	37.5	0.118	0.331	0.551	75	0.5	13.05
T0766TS184_2-D1.rsa	37.5	0	0.465	0.535	61	0.615	22.59
T0766TS410_1-D1.rsa	37.5	0	0.421	0.579	66	0.568	19.96
T0766TS410_4-D1.rsa	37.5	0	0.5	0.5	57	0.658	16.45
T0766TS011_5-D1.rsa	37.5	0	0.456	0.544	62	0.605	26.1
T0766TS133_3-D1.rsa	37.5	0	0.447	0.553	63	0.595	26.75
T0766TS492_3-D1.rsa	37.5	0	0.5	0.5	57	0.658	16.45
T0766TS414_4-D1.rsa	37.5	0	0.436	0.564	62	0.605	22.95
T0766TS145_1-D1.rsa	36.538	0.436	0	0.564	93	0.393	12.5
T0766TS346_1-D1.rsa	35.938	0.096	0.206	0.699	95	0.378	16.91
T0766TS414_3-D1.rsa	35.938	0.096	0.324	0.581	79	0.455	15.07
T0766TS228_4-D1.rsa	35.714	0.159	0.357	0.484	61	0.585	44.64
T0766TS041_1-D1.rsa	35.714	0.222	0.214	0.563	71	0.503	20.44
T0766TS184_3-D1.rsa	35.714	0.183	0.381	0.437	55	0.649	36.31
T0766TS184_4-D1.rsa	35.714	0.238	0.349	0.413	52	0.687	40.87
T0766TS251_2-D1.rsa	35.616	0.761	0	0.239	61	0.584	44.61
T0766TS038_5-D1.rsa	35	0	0.246	0.754	86	0.407	13.82
T0766TS300_1-D1.rsa	35	0	0.325	0.675	77	0.455	19.96
T0766TS041_3-D1.rsa	35	0.018	0.412	0.57	65	0.538	28.29
T0766TS008_2-D1.rsa	35	0	0.412	0.588	67	0.522	19.74
T0766TS452_3-D1.rsa	35	0	0.588	0.412	47	0.745	31.58
T0766TS454_2-D1.rsa	34.615	0.273	0.121	0.606	100	0.346	19.78
T0766TS145_3-D1.rsa	34.375	0.081	0.228	0.691	94	0.366	13.6
T0766TS237_1-D1.rsa	32.813	0.14	0.169	0.691	94	0.349	13.23
T0766TS300_2-D1.rsa	32.5	0	0.342	0.658	75	0.433	16.89
T0766TS452_5-D1.rsa	32.5	0.018	0.491	0.491	56	0.58	35.75
T0766TS008_5-D1.rsa	32.5	0.018	0.36	0.623	71	0.458	23.03
T0766TS008_1-D1.rsa	32.5	0.026	0.325	0.649	74	0.439	19.08
T0766TS381_4-D1.rsa	32.5	0.018	0.342	0.64	73	0.445	24.78
T0766TS268_1-D1.rsa	32.5	0	0.544	0.456	52	0.625	16.67
T0766TS041_4-D1.rsa	32.394	0.488	0.143	0.369	62	0.522	41.67

T0766TS300_4-D1.rsa	32.353	0.738	0	0.262	33	0.98	56.15
T0766TS479_3-D1.rsa	32.203	0.07	0.252	0.678	145	0.222	39.37
T0766TS499_5-D1.rsa	32.143	0.135	0.333	0.532	67	0.48	47.22
T0766TS349_1-D1.rsa	32.143	0.175	0.397	0.429	54	0.595	55.16
T0766TS145_4-D1.rsa	32.143	0.175	0.413	0.413	52	0.618	53.77
T0766TS237_4-D1.rsa	32.143	0.19	0.357	0.452	57	0.564	42.66
T0766TS041_5-D1.rsa	31.25	0.081	0.331	0.588	80	0.391	12.68
T0766TS251_5-D1.rsa	30.357	0.151	0.333	0.516	65	0.467	47.62
T0766TS436_2-D1.rsa	30.357	0.151	0.349	0.5	63	0.482	42.06
T0766TS216_4-D1.rsa	30.357	0.183	0.357	0.46	58	0.523	49.01
T0766TS184_5-D1.rsa	30.357	0.175	0.429	0.397	50	0.607	52.18
T0766TS073_1-D1.rsa	30.357	0.238	0.373	0.389	49	0.62	41.87
T0766TS414_5-D1.rsa	30	0.018	0.342	0.64	73	0.411	30.04
T0766TS414_2-D1.rsa	30	0	0.439	0.561	64	0.469	24.78
T0766TS436_1-D1.rsa	29.688	0.125	0.25	0.625	85	0.349	15.81
T0766TS193_2-D1.rsa	29.688	0.096	0.368	0.537	73	0.407	17.28
T0766TS499_1-D1.rsa	29.577	0.5	0.167	0.333	56	0.528	51.76
T0766TS454_4-D1.rsa	29.293	0.219	0.167	0.615	177	0.165	60.85
T0766TS216_2-D1.rsa	28.571	0.127	0.365	0.508	64	0.446	39.88
T0766TS228_2-D1.rsa	28.571	0.159	0.357	0.484	61	0.468	42.06
T0766TS277_2-D1.rsa	28.125	0.096	0.294	0.61	83	0.339	16.54
T0766TS414_1-D1.rsa	28.125	0.096	0.331	0.574	78	0.361	16.54
T0766TS160_3-D1.rsa	26.786	0.198	0.294	0.508	64	0.419	49.8
T0766TS499_3-D1.rsa	26.786	0.151	0.341	0.508	64	0.419	41.87
T0766TS228_1-D1.rsa	26.786	0.143	0.389	0.468	59	0.454	48.41
T0766TS011_2-D1.rsa	26.761	0.47	0.173	0.357	60	0.446	43.91
T0766TS160_1-D1.rsa	26.563	0.096	0.265	0.64	87	0.305	17.1
T0766TS160_2-D1.rsa	26.563	0.103	0.265	0.632	86	0.309	16.18
T0766TS277_3-D1.rsa	26.563	0.096	0.301	0.603	82	0.324	16.73
T0766TS263_4-D1.rsa	26.563	0.096	0.265	0.64	87	0.305	15.62
T0766TS228_3-D1.rsa	26.563	0.096	0.294	0.61	83	0.32	15.81
T0766TS420_1-D1.rsa	26.563	0.096	0.316	0.588	80	0.332	16.73
T0766TS335_5-D1.rsa	26.563	0.11	0.316	0.574	78	0.341	16.18
T0766TS268_3-D1.rsa	26.471	0.714	0	0.286	36	0.735	51.98
T0766TS216_3-D1.rsa	26.471	0.738	0	0.262	33	0.802	56.55
T0766TS448_1-D1.rsa	25	0.096	0.309	0.596	81	0.309	16.54
T0766TS277_5-D1.rsa	25	0.11	0.301	0.588	80	0.313	16.18
T0766TS436_5-D1.rsa	25	0.096	0.294	0.61	83	0.301	16.18
T0766TS410_2-D1.rsa	25	0.096	0.294	0.61	83	0.301	15.99
T0766TS345_5-D1.rsa	23.438	0.096	0.265	0.64	87	0.269	17.1
T0766TS410_5-D1.rsa	23.438	0.044	0.294	0.662	90	0.26	15.44
T0766TS184_1-D1.rsa	23.256	0.358	0.067	0.575	77	0.302	32.46
T0766TS420_3-D1.rsa	23.214	0.151	0.349	0.5	63	0.368	48.81
T0766TS038_2-D1.rsa	23.214	0.159	0.349	0.492	62	0.374	49.01
T0766TS448_2-D1.rsa	21.875	0.096	0.301	0.603	82	0.267	17.1
T0766TS300_5-D1.rsa	21.875	0.096	0.316	0.588	80	0.273	16.36
T0766TS454_3-D1.rsa	21.795	0.365	0.108	0.527	156	0.14	51.22

T0766TS479_1-D1.rsa	20.513	0.372	0.132	0.497	147	0.14	57.03
T0766TS133_5-D1.rsa	20	0	0.325	0.675	77	0.26	20.18
T0766TS117_5-D1.rsa	18.421	0.414	0.024	0.561	256	0.072	60.53
T0766TS073_3-D1.rsa	17.5	0	0.298	0.702	80	0.219	36.4
T0766TS117_2-D1.rsa	14.615	0.271	0.24	0.489	224	0.065	76.89
T0867TS464_2-D1.rsa	72.917	0.038	0	0.962	100	0.729	13.46
T0867TS464_5-D1.rsa	72.917	0.192	0.019	0.788	82	0.889	18.27
T0867TS284_3-D1.rsa	62.5	0.087	0	0.913	95	0.658	13.94
T0867TS455_5-D1.rsa	62.5	0.087	0	0.913	95	0.658	15.38
T0867TS321_2-D1.rsa	60.417	0.115	0.077	0.808	84	0.719	17.07
T0867TS455_3-D1.rsa	60.417	0.038	0.058	0.904	94	0.643	18.27
T0867TS321_4-D1.rsa	56.25	0.115	0.038	0.846	88	0.639	17.79
T0867TS452_2-D1.rsa	56.25	0.24	0	0.76	79	0.712	32.93
T0867TS479_3-D1.rsa	54.167	0.51	0	0.49	51	1.062	16.83
T0867TS434_1-D1.rsa	54.167	0.279	0	0.721	75	0.722	19.47
T0867TS434_3-D1.rsa	54.167	0.327	0	0.673	70	0.774	22.36
T0867TS183_3-D1.rsa	52.083	0.529	0	0.471	49	1.063	17.55
T0867TS284_5-D1.rsa	52.083	0.221	0.058	0.721	75	0.694	23.56
T0867TS321_5-D1.rsa	52.083	0.096	0.173	0.731	76	0.685	18.27
T0867TS026_1-D1.rsa	52.083	0.144	0.288	0.567	59	0.883	30.29
T0867TS284_4-D1.rsa	52.083	0.221	0.058	0.721	75	0.694	24.52
T0867TS434_5-D1.rsa	52.083	0.26	0	0.74	77	0.676	20.43
T0867TS180_3-D1.rsa	52.083	0	0.212	0.788	82	0.635	16.59
T0867TS321_3-D1.rsa	50	0.115	0.077	0.808	84	0.595	18.75
T0867TS451_5-D1.rsa	50	0.135	0	0.865	90	0.556	21.39
T0867TS180_1-D1.rsa	50	0	0.231	0.769	80	0.625	20.67
T0867TS321_1-D1.rsa	47.917	0.115	0.077	0.808	84	0.57	17.55
T0867TS467_4-D1.rsa	47.917	0.212	0.154	0.635	66	0.726	26.2
T0867TS357_3-D1.rsa	45.833	0.173	0.077	0.75	78	0.588	25.24
T0867TS016_1-D1.rsa	43.75	0.317	0.154	0.529	55	0.795	23.8
T0867TS467_5-D1.rsa	43.75	0.212	0.24	0.548	57	0.768	21.39
T0867TS357_5-D1.rsa	43.75	0.183	0.019	0.798	83	0.527	25.48
T0867TS434_2-D1.rsa	43.75	0.26	0	0.74	77	0.568	19.95
T0867TS467_1-D1.rsa	43.75	0.26	0.135	0.606	63	0.694	19.23
T0867TS452_3-D1.rsa	41.667	0.317	0.163	0.519	54	0.772	17.79
T0867TS359_4-D1.rsa	41.667	0.077	0.462	0.462	48	0.868	94.23
T0867TS407_3-D1.rsa	41.667	0.077	0.462	0.462	48	0.868	94.95
T0867TS455_4-D1.rsa	41.667	0.058	0	0.942	98	0.425	19.23
T0867TS432_3-D1.rsa	41.667	0.26	0.038	0.702	73	0.571	19.47
T0867TS432_5-D1.rsa	41.667	0.24	0.125	0.635	66	0.631	21.15
T0867TS451_1-D1.rsa	41.667	0.154	0	0.846	88	0.473	21.88
T0867TS467_2-D1.rsa	41.667	0.346	0.183	0.471	49	0.85	24.52
T0867TS026_5-D1.rsa	41.667	0.212	0.25	0.538	56	0.744	28.36
T0867TS357_4-D1.rsa	41.667	0.183	0.058	0.76	79	0.527	26.68
T0867TS455_1-D1.rsa	41.667	0	0.144	0.856	89	0.468	15.87
T0867TS359_3-D1.rsa	39.583	0.077	0.462	0.462	48	0.825	94.47
T0867TS028_1-D1.rsa	39.583	0.067	0.5	0.433	45	0.88	96.64

T0867TS432_4-D1.rsa	39.583	0.26	0.048	0.692	72	0.55	20.91
T0867TS451_4-D1.rsa	39.583	0.144	0	0.856	89	0.445	22.11
T0867TS451_3-D1.rsa	39.583	0.154	0.038	0.808	84	0.471	23.08
T0867TS026_3-D1.rsa	39.583	0.154	0.288	0.558	58	0.682	18.51
T0867TS434_4-D1.rsa	39.583	0.24	0	0.76	79	0.501	19.23
T0867TS495_2-D1.rsa	39.583	0.077	0.394	0.529	55	0.72	92.55
T0867TS251_5-D1.rsa	39.583	0.394	0.058	0.548	57	0.694	22.6
T0867TS180_5-D1.rsa	37.5	0.135	0	0.865	90	0.417	20.91
T0867TS258_5-D1.rsa	37.5	0.077	0.471	0.452	47	0.798	94.95
T0867TS349_1-D1.rsa	37.5	0.077	0.49	0.433	45	0.833	97.11
T0867TS275_4-D1.rsa	37.5	0.077	0.5	0.423	44	0.852	97.11
T0867TS258_4-D1.rsa	37.5	0.077	0.462	0.462	48	0.781	95.43
T0867TS345_4-D1.rsa	37.5	0.067	0.51	0.423	44	0.852	95.91
T0867TS359_1-D1.rsa	37.5	0.077	0.5	0.423	44	0.852	96.39
T0867TS275_2-D1.rsa	37.5	0.077	0.5	0.423	44	0.852	96.88
T0867TS451_2-D1.rsa	37.5	0.135	0.019	0.846	88	0.426	23.32
T0867TS455_2-D1.rsa	37.5	0.038	0.038	0.923	96	0.391	17.07
T0867TS180_2-D1.rsa	37.5	0.067	0.212	0.721	75	0.5	17.07
T0867TS405_4-D1.rsa	35.417	0.077	0.5	0.423	44	0.805	96.15
T0867TS359_5-D1.rsa	35.417	0.077	0.49	0.433	45	0.787	96.88
T0867TS444_1-D1.rsa	35.417	0.077	0.538	0.385	40	0.885	94.95
T0867TS407_1-D1.rsa	35.417	0.077	0.49	0.433	45	0.787	95.19
T0867TS220_3-D1.rsa	35.417	0.067	0.481	0.452	47	0.754	95.67
T0867TS464_3-D1.rsa	35.417	0.077	0.481	0.442	46	0.77	96.64
T0867TS345_5-D1.rsa	35.417	0.077	0.529	0.394	41	0.864	95.19
T0867TS287_2-D1.rsa	35.417	0.077	0.462	0.462	48	0.738	96.39
T0867TS382_4-D1.rsa	35.417	0.077	0.51	0.413	43	0.824	97.36
T0867TS357_1-D1.rsa	35.417	0.173	0.106	0.721	75	0.472	25.96
T0867TS236_1-D1.rsa	35.417	0.077	0.5	0.423	44	0.805	96.88
T0867TS421_1-D1.rsa	35.417	0.077	0.471	0.452	47	0.754	97.36
T0867TS382_5-D1.rsa	35.417	0.067	0.51	0.423	44	0.805	97.36
T0867TS444_3-D1.rsa	35.417	0.077	0.481	0.442	46	0.77	96.15
T0867TS236_2-D1.rsa	35.417	0.077	0.5	0.423	44	0.805	96.39
T0867TS220_1-D1.rsa	35.417	0.067	0.5	0.433	45	0.787	97.11
T0867TS432_2-D1.rsa	35.417	0.24	0.173	0.587	61	0.581	23.8
T0867TS357_2-D1.rsa	35.417	0.183	0.096	0.721	75	0.472	24.52
T0867TS495_1-D1.rsa	35.417	0.077	0.394	0.529	55	0.644	91.83
T0867TS275_3-D1.rsa	35.417	0.077	0.481	0.442	46	0.77	96.88
T0867TS382_3-D1.rsa	35.417	0.077	0.5	0.423	44	0.805	97.6
T0867TS359_2-D1.rsa	33.333	0.077	0.5	0.423	44	0.758	96.88
T0867TS479_5-D1.rsa	33.333	0.279	0.163	0.558	58	0.575	29.81
T0867TS251_1-D1.rsa	33.333	0.077	0.452	0.471	49	0.68	90.14
T0867TS421_4-D1.rsa	33.333	0.077	0.481	0.442	46	0.725	96.64
T0867TS479_4-D1.rsa	33.333	0.308	0.192	0.5	52	0.641	53.12
T0867TS258_2-D1.rsa	33.333	0.077	0.471	0.452	47	0.709	96.88
T0867TS425_1-D1.rsa	33.333	0.077	0.5	0.423	44	0.758	97.36
T0867TS250_2-D1.rsa	33.333	0.077	0.471	0.452	47	0.709	95.91

T0867TS430_3-D1.rsa	33.333	0.067	0.471	0.462	48	0.694	96.88
T0867TS446_4-D1.rsa	33.333	0.067	0.5	0.433	45	0.741	96.88
T0867TS407_2-D1.rsa	33.333	0.077	0.471	0.452	47	0.709	94.71
T0867TS345_1-D1.rsa	33.333	0.077	0.538	0.385	40	0.833	96.15
T0867TS284_1-D1.rsa	33.333	0.077	0.5	0.423	44	0.758	96.64
T0867TS452_5-D1.rsa	33.333	0.183	0.231	0.587	61	0.546	22.36
T0867TS183_5-D1.rsa	33.333	0.288	0.183	0.529	55	0.606	29.57
T0867TS183_4-D1.rsa	33.333	0.269	0.183	0.548	57	0.585	52.4
T0867TS495_3-D1.rsa	33.333	0.077	0.413	0.51	53	0.629	92.07
T0867TS495_4-D1.rsa	33.333	0.077	0.413	0.51	53	0.629	92.31
T0867TS407_5-D1.rsa	33.333	0.077	0.462	0.462	48	0.694	94.71
T0867TS236_4-D1.rsa	33.333	0.077	0.481	0.442	46	0.725	94.71
T0867TS430_5-D1.rsa	33.333	0.067	0.471	0.462	48	0.694	95.43
T0867TS258_3-D1.rsa	33.333	0.077	0.471	0.452	47	0.709	96.39
T0867TS425_4-D1.rsa	33.333	0.077	0.481	0.442	46	0.725	97.11
T0867TS425_3-D1.rsa	33.333	0.077	0.5	0.423	44	0.758	97.11
T0867TS382_2-D1.rsa	33.333	0.077	0.51	0.413	43	0.775	97.11
T0867TS407_4-D1.rsa	33.333	0.077	0.462	0.462	48	0.694	94.23
T0867TS432_1-D1.rsa	33.333	0.24	0.087	0.673	70	0.476	22.6
T0867TS495_5-D1.rsa	33.333	0.077	0.413	0.51	53	0.629	93.03
T0867TS077_1-D1.rsa	33.333	0.077	0.481	0.442	46	0.725	96.88
T0867TS005_1-D1.rsa	33.333	0.077	0.5	0.423	44	0.758	97.6
T0867TS345_3-D1.rsa	33.333	0.077	0.529	0.394	41	0.813	95.19
T0867TS287_4-D1.rsa	33.333	0.077	0.462	0.462	48	0.694	96.39
T0867TS430_2-D1.rsa	33.333	0.077	0.433	0.49	51	0.654	95.67
T0867TS183_1-D1.rsa	31.25	0.077	0.5	0.423	44	0.71	96.39
T0867TS452_1-D1.rsa	31.25	0.077	0.49	0.433	45	0.694	94.71
T0867TS405_5-D1.rsa	31.25	0.077	0.5	0.423	44	0.71	96.64
T0867TS077_2-D1.rsa	31.25	0.077	0.5	0.423	44	0.71	96.88
T0867TS119_1-D1.rsa	31.25	0.077	0.49	0.433	45	0.694	97.11
T0867TS425_2-D1.rsa	31.25	0.077	0.5	0.423	44	0.71	97.11
T0867TS382_1-D1.rsa	31.25	0.077	0.51	0.413	43	0.727	97.6
T0867TS251_3-D1.rsa	31.25	0.298	0.106	0.596	62	0.504	25.96
T0867TS430_4-D1.rsa	31.25	0.067	0.481	0.452	47	0.665	95.91
T0867TS405_3-D1.rsa	31.25	0.077	0.481	0.442	46	0.679	95.91
T0867TS258_1-D1.rsa	31.25	0.077	0.462	0.462	48	0.651	96.39
T0867TS405_2-D1.rsa	31.25	0.077	0.481	0.442	46	0.679	96.39
T0867TS005_5-D1.rsa	31.25	0.077	0.51	0.413	43	0.727	97.36
T0867TS452_4-D1.rsa	31.25	0.173	0.192	0.635	66	0.473	23.08
T0867TS446_3-D1.rsa	31.25	0.077	0.49	0.433	45	0.694	95.67
T0867TS313_4-D1.rsa	31.25	0.077	0.5	0.423	44	0.71	97.36
T0867TS251_2-D1.rsa	31.25	0.077	0.452	0.471	49	0.638	91.11
T0867TS313_5-D1.rsa	31.25	0.077	0.5	0.423	44	0.71	97.6
T0867TS479_1-D1.rsa	29.167	0.077	0.5	0.423	44	0.663	96.64
T0867TS183_2-D1.rsa	29.167	0.058	0.385	0.558	58	0.503	61.06
T0867TS220_4-D1.rsa	29.167	0.077	0.471	0.452	47	0.621	96.39
T0867TS077_4-D1.rsa	29.167	0.077	0.481	0.442	46	0.634	97.36

T0867TS479_2-D1.rsa	29.167	0.058	0.327	0.615	64	0.456	61.3
T0867TS444_4-D1.rsa	29.167	0.077	0.51	0.413	43	0.678	94.71
T0867TS220_2-D1.rsa	29.167	0.077	0.49	0.433	45	0.648	96.15
T0867TS313_3-D1.rsa	29.167	0.077	0.5	0.423	44	0.663	97.11
T0867TS250_3-D1.rsa	29.167	0.077	0.471	0.452	47	0.621	95.43
T0867TS430_1-D1.rsa	29.167	0.077	0.481	0.442	46	0.634	97.11
T0867TS464_1-D1.rsa	27.083	0.077	0.288	0.635	66	0.41	80.53
T0867TS284_2-D1.rsa	27.083	0.077	0.481	0.442	46	0.589	96.88
T0867TS251_4-D1.rsa	27.083	0.288	0.087	0.625	65	0.417	16.83
T0867TS220_5-D1.rsa	27.083	0.077	0.471	0.452	47	0.576	95.67
T0867TS313_2-D1.rsa	27.083	0.077	0.5	0.423	44	0.616	97.6
T0867TS048_1-D1.rsa	27.083	0.077	0.462	0.462	48	0.564	97.6
T0867TS467_3-D1.rsa	25	0.221	0.24	0.538	56	0.446	25.24
T0867TS250_1-D1.rsa	25	0.077	0.471	0.452	47	0.532	95.19
T0764TS436_3-D1.rsa	88.732	0	0.101	0.899	151	0.588	9.62
T0764TS263_3-D1.rsa	82.353	0.373	0	0.627	79	1.042	26.79
T0764TS436_5-D1.rsa	79.412	0.407	0	0.593	144	0.551	8.78
T0764TS410_4-D1.rsa	67.442	0.201	0	0.799	107	0.63	39.92
T0764TS420_5-D1.rsa	65.625	0	0.14	0.86	117	0.561	40.07
T0764TS038_2-D1.rsa	62.791	0.015	0.142	0.843	113	0.556	14.55
T0764TS171_1-D1.rsa	59.615	0.321	0.133	0.545	90	0.662	73.58
T0764TS335_2-D1.rsa	59.615	0.303	0.097	0.6	99	0.602	75.16
T0764TS335_3-D1.rsa	59.615	0.158	0.127	0.715	118	0.505	55.22
T0764TS133_4-D1.rsa	59.615	0.267	0.152	0.582	96	0.621	71.68
T0764TS216_2-D1.rsa	55.769	0.012	0.109	0.879	145	0.385	10.92
T0764TS448_3-D1.rsa	55.769	0.303	0.085	0.612	101	0.552	66.77
T0764TS133_2-D1.rsa	55.172	0	0.112	0.888	127	0.434	48.08
T0764TS171_2-D1.rsa	54.93	0.036	0.024	0.94	158	0.348	18.11
T0764TS133_3-D1.rsa	54.93	0.065	0.036	0.899	151	0.364	17.47
T0764TS237_5-D1.rsa	50	0	0.098	0.902	129	0.388	40.73
T0764TS436_2-D1.rsa	48.276	0	0.098	0.902	129	0.374	59.97
T0764TS008_2-D1.rsa	47.458	0.079	0.243	0.678	145	0.327	22.31
T0764TS436_1-D1.rsa	46.552	0	0.091	0.909	130	0.358	60.66
T0764TS184_2-D1.rsa	46.154	0.248	0.085	0.667	110	0.42	39.24
T0764TS268_3-D1.rsa	45.833	0.019	0.259	0.722	78	0.588	64.35
T0764TS145_2-D1.rsa	45.07	0.018	0.107	0.875	147	0.307	11.22
T0764TS216_1-D1.rsa	44.828	0	0.14	0.86	123	0.364	55.59
T0764TS499_1-D1.rsa	43.182	0.084	0.453	0.463	137	0.315	28.55
T0764TS420_2-D1.rsa	42.188	0.015	0.426	0.559	76	0.555	70.04
T0764TS452_3-D1.rsa	42.105	0.3	0.134	0.567	123	0.342	55.88
T0764TS251_4-D1.rsa	41.379	0	0.112	0.888	127	0.326	57.34
T0764TS448_5-D1.rsa	40.909	0.037	0.48	0.483	143	0.286	35.9
T0764TS011_2-D1.rsa	40.385	0.242	0.073	0.685	113	0.357	47.31
T0764TS452_5-D1.rsa	40.351	0.318	0.078	0.604	131	0.308	54.15
T0764TS492_1-D1.rsa	40.278	0.366	0.103	0.531	113	0.356	70.69
T0764TS268_5-D1.rsa	40	0	0.14	0.86	98	0.408	44.3
T0764TS381_2-D1.rsa	39.474	0.253	0.171	0.576	125	0.316	62.79

T0764TS216_3-D1.rsa	39.437	0	0.042	0.958	161	0.245	10.74
T0764TS268_1-D1.rsa	39.063	0.11	0.294	0.596	81	0.482	65.44
T0764TS410_3-D1.rsa	38.889	0.376	0.085	0.54	115	0.338	66.37
T0764TS410_1-D1.rsa	38.889	0.362	0.085	0.554	118	0.33	63.2
T0764TS499_4-D1.rsa	37.719	0.332	0.111	0.558	121	0.312	57.49
T0764TS263_1-D1.rsa	37.5	0.333	0.07	0.596	127	0.295	63.45
T0764TS410_2-D1.rsa	37.5	0.371	0.07	0.559	119	0.315	59.39
T0764TS008_1-D1.rsa	37.5	0.015	0.368	0.618	84	0.446	64.71
T0764TS133_5-D1.rsa	36.842	0.263	0.166	0.571	124	0.297	61.17
T0764TS479_2-D1.rsa	36.364	0.243	0.101	0.656	189	0.192	43.92
T0764TS277_5-D1.rsa	36.19	0.295	0.214	0.491	115	0.315	69.23
T0764TS300_4-D1.rsa	36.111	0.343	0.094	0.563	120	0.301	72.21
T0764TS335_4-D1.rsa	36.111	0.286	0.103	0.61	130	0.278	64.09
T0764TS008_4-D1.rsa	35.938	0.015	0.426	0.559	76	0.473	68.02
T0764TS414_1-D1.rsa	35.227	0.068	0.436	0.497	147	0.24	32.69
T0764TS414_2-D1.rsa	34.211	0.244	0.175	0.581	126	0.272	61.52
T0764TS117_1-D1.rsa	34.091	0.044	0.392	0.564	167	0.204	35.9
T0764TS492_2-D1.rsa	33.333	0.286	0.138	0.576	125	0.267	49.42
T0764TS420_3-D1.rsa	32.759	0.57	0	0.43	110	0.298	62.79
T0764TS133_1-D1.rsa	32.468	0.406	0.121	0.473	106	0.306	81.19
T0764TS414_4-D1.rsa	32.456	0.253	0.161	0.585	127	0.256	64.06
T0764TS184_5-D1.rsa	32	0.036	0.362	0.601	83	0.386	62.68
T0764TS008_3-D1.rsa	32	0.058	0.196	0.746	103	0.311	66.67
T0764TS050_1-D1.rsa	32	0.051	0.072	0.877	121	0.264	46.2
T0764TS210_1-D1.rsa	31.944	0.408	0.103	0.488	104	0.307	70.31
T0764TS346_1-D1.rsa	31.818	0.061	0.439	0.5	148	0.215	31.76
T0764TS011_4-D1.rsa	31.429	0.316	0.205	0.479	112	0.281	71.15
T0764TS454_2-D1.rsa	31.313	0.208	0.125	0.667	192	0.163	57.55
T0764TS452_4-D1.rsa	30.769	0.392	0.128	0.48	142	0.217	56.77
T0764TS420_4-D1.rsa	30.702	0.249	0.171	0.581	126	0.244	64.86
T0764TS381_1-D1.rsa	29.87	0.339	0.076	0.585	131	0.228	61.67
T0764TS011_3-D1.rsa	29.524	0.278	0.184	0.538	126	0.234	62.07
T0764TS499_5-D1.rsa	29.524	0.338	0.141	0.521	122	0.242	64
T0764TS171_4-D1.rsa	29.412	0.354	0.095	0.551	134	0.219	65
T0764TS436_4-D1.rsa	28	0.574	0.005	0.421	170	0.165	50.31
T0764TS041_5-D1.rsa	28	0.402	0.112	0.486	122	0.23	78.78
T0764TS335_5-D1.rsa	28	0.422	0.116	0.462	116	0.241	87.85
T0764TS041_3-D1.rsa	27.941	0.387	0.095	0.519	126	0.222	66.33
T0764TS212_1-D1.rsa	27.869	0.038	0.41	0.552	116	0.24	68.66
T0764TS228_5-D1.rsa	27.723	0.302	0.137	0.561	180	0.154	69
T0764TS237_1-D1.rsa	27.119	0.009	0.234	0.757	162	0.167	42.06
T0764TS410_5-D1.rsa	27	0.454	0.116	0.43	108	0.25	84.36
T0764TS345_3-D1.rsa	27	0.462	0.1	0.438	110	0.245	86.95
T0764TS251_3-D1.rsa	26.923	0.358	0.111	0.53	157	0.171	55.12
T0764TS228_3-D1.rsa	26.923	0.405	0.115	0.48	142	0.19	58.51
T0764TS184_1-D1.rsa	26.667	0.368	0.201	0.432	101	0.264	72.01
T0764TS073_2-D1.rsa	26.471	0.379	0.091	0.531	129	0.205	68.78

T0764TS349_4-D1.rsa	26.263	0.174	0.007	0.819	236	0.111	14.58
T0764TS011_5-D1.rsa	26	0.418	0.116	0.466	117	0.222	87.95
T0764TS228_1-D1.rsa	26	0.442	0.12	0.438	110	0.236	89.84
T0764TS420_1-D1.rsa	26	0.446	0.135	0.418	105	0.248	85.36
T0764TS345_1-D1.rsa	26	0.466	0.096	0.438	110	0.236	87.35
T0764TS268_2-D1.rsa	25.974	0.379	0.125	0.496	111	0.234	71.79
T0764TS499_2-D1.rsa	25.974	0.42	0.121	0.46	103	0.252	79.52
T0764TS110_5-D1.rsa	25.743	0.308	0.156	0.536	172	0.15	74.22
T0764TS228_4-D1.rsa	25.641	0.422	0.125	0.453	134	0.191	60.94
T0764TS349_5-D1.rsa	25.253	0.226	0.205	0.569	164	0.154	69.18
T0764TS110_2-D1.rsa	25.253	0.208	0.188	0.604	174	0.145	61.55
T0764TS251_5-D1.rsa	25.143	0.515	0.02	0.465	188	0.134	47.77
T0764TS206_1-D1.rsa	25	0.383	0.103	0.514	125	0.2	67.11
T0764TS345_2-D1.rsa	25	0.45	0.135	0.414	104	0.24	88.35
T0764TS414_3-D1.rsa	24.59	0.033	0.424	0.543	114	0.216	71.27
T0764TS345_5-D1.rsa	24.59	0	0.362	0.638	134	0.184	51.49
T0764TS263_2-D1.rsa	24.561	0.434	0.091	0.475	104	0.236	75.69
T0764TS414_5-D1.rsa	24.359	0.395	0.125	0.48	142	0.172	56.6
T0764TS349_1-D1.rsa	24	0.443	0.022	0.535	216	0.111	38.06
T0764TS454_1-D1.rsa	23.729	0	0.285	0.715	153	0.155	55.96
T0764TS038_5-D1.rsa	23.529	0.313	0.095	0.593	144	0.163	60.67
T0764TS277_4-D1.rsa	23.377	0.371	0.125	0.504	113	0.207	71.31
T0764TS160_1-D1.rsa	23.077	0.368	0.111	0.52	154	0.15	56.34
T0764TS041_2-D1.rsa	23.077	0.385	0.118	0.497	147	0.157	58.42
T0764TS300_5-D1.rsa	23.077	0.389	0.128	0.483	143	0.161	59.2
T0764TS228_2-D1.rsa	23.077	0.419	0.115	0.466	138	0.167	58.94
T0764TS268_4-D1.rsa	23.077	0.392	0.132	0.476	141	0.164	60.07
T0764TS145_1-D1.rsa	23.077	0.419	0.118	0.463	137	0.168	59.12
T0764TS479_4-D1.rsa	22.857	0.517	0.01	0.473	191	0.12	51.24
T0764TS160_4-D1.rsa	22.857	0.505	0.015	0.48	194	0.118	50.99
T0764TS277_3-D1.rsa	22.857	0.51	0.01	0.48	194	0.118	9.53
T0764TS184_4-D1.rsa	22.807	0.447	0.046	0.507	111	0.205	77.4
T0764TS117_3-D1.rsa	22.093	0.385	0.11	0.505	143	0.154	74.56
T0764TS251_2-D1.rsa	22.093	0.403	0.092	0.505	143	0.154	61.84
T0764TS145_3-D1.rsa	22.059	0.403	0.107	0.49	119	0.185	71.22
T0764TS011_1-D1.rsa	22.059	0.416	0.107	0.477	116	0.19	69.89
T0764TS300_3-D1.rsa	22.034	0.009	0.154	0.836	179	0.123	41.24
T0764TS381_3-D1.rsa	21.795	0.419	0.132	0.449	133	0.164	61.89
T0764TS038_3-D1.rsa	21.311	0.014	0.433	0.552	116	0.184	70.9
T0764TS237_3-D1.rsa	21.277	0.315	0.136	0.549	141	0.151	76.07
T0764TS110_3-D1.rsa	20.339	0.009	0.294	0.696	149	0.137	66.59
T0764TS448_2-D1.rsa	19.298	0.443	0.078	0.479	105	0.184	81.85
T0764TS349_2-D1.rsa	18.605	0.357	0.095	0.548	155	0.12	70.41
T0764TS110_1-D1.rsa	18.605	0.396	0.124	0.481	136	0.137	82.33
T0764TS448_4-D1.rsa	17.544	0.406	0.046	0.548	120	0.146	76.48
T0764TS277_2-D1.rsa	17.544	0.438	0.091	0.47	103	0.17	77.63
T0764TS263_5-D1.rsa	17.544	0.457	0.082	0.461	101	0.174	80.94

T0764TS117_4-D1.rsa	17.442	0.389	0.113	0.498	141	0.124	82.07
T0764TS300_2-D1.rsa	17.442	0.399	0.11	0.491	139	0.125	82.86
T0764TS171_3-D1.rsa	17.442	0.382	0.11	0.509	144	0.121	76.68
T0764TS041_4-D1.rsa	17.442	0.336	0.113	0.551	156	0.112	66.25
T0764TS454_4-D1.rsa	17.105	0.43	0.015	0.555	253	0.068	59.54
T0764TS160_2-D1.rsa	14.474	0.349	0.123	0.527	175	0.083	99.32
T0764TS349_3-D1.rsa	14.474	0.343	0.123	0.533	177	0.082	98.27
T0764TS160_3-D1.rsa	14.474	0.349	0.12	0.53	176	0.082	98.95
T0764TS452_1-D1.rsa	12.766	0.346	0.198	0.455	117	0.109	85.41
T0764TS251_1-D1.rsa	10.638	0.311	0.16	0.529	136	0.078	79.18
T0764TS381_5-D1.rsa	10.638	0.311	0.179	0.51	131	0.081	82.3
T0764TS073_1-D1.rsa	10.638	0.315	0.195	0.49	126	0.084	85.12
T0764TS277_1-D1.rsa	10.638	0.319	0.183	0.498	128	0.083	78.79
T0764TS448_1-D1.rsa	10.638	0.346	0.198	0.455	117	0.091	85.51
T0817TS300_4-D2.rsa	92.105	0.054	0	0.946	105	0.877	14.87
T0817TS184_1-D2.rsa	71.875	0.353	0	0.647	88	0.817	9.93
T0817TS448_3-D2.rsa	71.053	0.18	0.054	0.766	85	0.836	23.42
T0817TS008_3-D2.rsa	70.833	0.082	0.4	0.518	57	1.243	25.45
T0817TS335_5-D2.rsa	70.588	0.484	0	0.516	65	1.086	35.12
T0817TS210_3-D2.rsa	70	0.228	0	0.772	88	0.795	12.72
T0817TS452_3-D2.rsa	68.421	0.144	0	0.856	95	0.72	18.24
T0817TS346_1-D2.rsa	68	0.333	0	0.667	92	0.739	12.14
T0817TS237_3-D2.rsa	67.647	0.484	0	0.516	65	1.041	34.92
T0817TS300_2-D2.rsa	67.5	0.035	0.158	0.807	92	0.734	35.53
T0817TS206_1-D2.rsa	66.667	0	0.509	0.491	53	1.258	61.81
T0817TS171_3-D2.rsa	65.789	0.216	0	0.784	87	0.756	17.57
T0817TS156_5-D2.rsa	65.789	0.117	0.036	0.847	94	0.7	19.14
T0817TS420_4-D2.rsa	65.789	0.126	0	0.874	97	0.678	14.64
T0817TS160_3-D2.rsa	64.063	0.154	0.015	0.831	113	0.567	11.03
T0817TS228_4-D2.rsa	62.5	0.091	0.327	0.582	64	0.977	25.91
T0817TS381_3-D2.rsa	60.526	0.288	0.081	0.631	70	0.865	34.69
T0817TS268_1-D2.rsa	60.526	0.216	0	0.784	87	0.696	21.17
T0817TS184_3-D2.rsa	60.526	0.189	0.054	0.757	84	0.721	34.69
T0817TS268_5-D2.rsa	58.333	0.037	0.056	0.907	98	0.595	20.37
T0817TS454_2-D2.rsa	58.333	0	0.565	0.435	47	1.241	55.56
T0817TS335_3-D2.rsa	57.895	0.081	0.054	0.865	96	0.603	21.85
T0817TS212_1-D2.rsa	57.895	0.207	0.081	0.712	79	0.733	18.92
T0817TS263_3-D2.rsa	55.769	0.297	0.073	0.63	104	0.536	51.58
T0817TS038_5-D2.rsa	55.263	0.216	0.072	0.712	79	0.7	36.94
T0817TS454_1-D2.rsa	55.263	0.297	0	0.703	78	0.709	17.57
T0817TS381_5-D2.rsa	55	0.053	0.211	0.737	84	0.655	18.86
T0817TS410_2-D2.rsa	54.167	0.037	0.333	0.63	68	0.797	55.56
T0817TS216_4-D2.rsa	54.167	0	0.611	0.389	42	1.29	55.79
T0817TS011_3-D2.rsa	53.571	0.053	0.191	0.756	99	0.541	13.55
T0817TS022_3-D2.rsa	53.521	0.333	0	0.667	112	0.478	19.87
T0817TS184_4-D2.rsa	52.632	0.324	0.099	0.577	64	0.822	39.87
T0817TS268_4-D2.rsa	52.632	0.27	0.108	0.622	69	0.763	39.19

T0817TS448_2-D2.rsa	52.5	0.035	0.149	0.816	93	0.565	35.09
T0817TS011_5-D2.rsa	51.563	0.015	0.243	0.743	101	0.511	15.99
T0817TS452_4-D2.rsa	50	0	0.218	0.782	86	0.581	32.27
T0817TS145_3-D2.rsa	50	0	0.315	0.685	74	0.676	40.05
T0817TS499_1-D2.rsa	50	0	0.382	0.618	81	0.617	17.56
T0817TS022_2-D2.rsa	48.837	0.321	0	0.679	91	0.537	36.75
T0817TS145_2-D2.rsa	48.837	0.343	0.06	0.597	80	0.61	43.28
T0817TS492_3-D2.rsa	48.077	0.248	0.036	0.715	118	0.407	12.5
T0817TS410_1-D2.rsa	47.368	0.045	0.018	0.937	104	0.455	18.69
T0817TS171_5-D2.rsa	46.875	0.015	0.184	0.801	109	0.43	12.87
T0817TS171_2-D2.rsa	46.875	0.081	0.213	0.706	96	0.488	15.62
T0817TS228_1-D2.rsa	46.512	0.351	0	0.649	87	0.535	34.52
T0817TS041_4-D2.rsa	46.479	0.452	0.089	0.458	77	0.604	34.78
T0817TS410_5-D2.rsa	46.429	0	0.229	0.771	101	0.46	16.6
T0817TS414_1-D2.rsa	46.429	0	0.344	0.656	86	0.54	16.22
T0817TS171_4-D2.rsa	46.154	0.061	0.164	0.776	128	0.361	9.97
T0817TS499_5-D2.rsa	45	0	0.307	0.693	79	0.57	17.32
T0817TS381_1-D2.rsa	44.737	0.36	0.054	0.586	65	0.688	54.05
T0817TS117_4-D2.rsa	44.186	0.306	0.045	0.649	87	0.508	43.28
T0817TS156_1-D2.rsa	44.186	0.082	0.075	0.843	113	0.391	14.93
T0817TS420_3-D2.rsa	44.186	0.216	0.187	0.597	80	0.552	20.52
T0817TS277_3-D2.rsa	44.186	0.381	0	0.619	83	0.532	25.56
T0817TS210_4-D2.rsa	43.75	0	0.464	0.536	59	0.742	61.82
T0817TS436_5-D2.rsa	43.75	0	0.436	0.564	62	0.706	59.09
T0817TS420_5-D2.rsa	43.75	0.059	0.191	0.75	102	0.429	13.05
T0817TS133_5-D2.rsa	42.857	0	0.42	0.58	76	0.564	52.29
T0817TS133_3-D2.rsa	42.857	0	0.397	0.603	79	0.542	48.09
T0817TS479_2-D2.rsa	42.308	0.242	0.073	0.685	113	0.374	47.31
T0817TS011_2-D2.rsa	41.86	0.396	0.082	0.522	70	0.598	53.17
T0817TS210_1-D2.rsa	41.667	0.018	0.355	0.627	69	0.604	62.27
T0817TS277_4-D2.rsa	41.667	0.027	0.473	0.5	55	0.758	76.59
T0817TS050_1-D2.rsa	41.071	0.143	0.27	0.587	74	0.555	45.83
T0817TS268_3-D2.rsa	40.625	0.081	0.096	0.824	112	0.363	12.13
T0817TS335_2-D2.rsa	40.385	0.23	0.042	0.727	120	0.337	15.66
T0817TS381_4-D2.rsa	40.26	0.438	0.027	0.536	120	0.335	17.26
T0817TS414_5-D2.rsa	39.535	0.328	0.045	0.627	84	0.471	48.88
T0817TS038_4-D2.rsa	39.474	0.241	0.5	0.259	28	1.41	94.91
T0817TS171_1-D2.rsa	39.437	0.429	0	0.571	96	0.411	33.81
T0817TS345_3-D2.rsa	39.286	0	0.389	0.611	80	0.491	58.21
T0817TS008_5-D2.rsa	39.286	0	0.443	0.557	73	0.538	55.73
T0817TS008_2-D2.rsa	39.286	0	0.13	0.87	114	0.345	15.08
T0817TS414_2-D2.rsa	39.063	0.088	0.36	0.551	75	0.521	62.5
T0817TS237_1-D2.rsa	37.5	0.029	0.382	0.588	80	0.469	70.4
T0817TS448_4-D2.rsa	37.5	0.015	0.331	0.654	89	0.421	60.48
T0817TS022_1-D2.rsa	37.5	0.018	0.272	0.711	81	0.463	45.83
T0817TS216_5-D2.rsa	37.5	0.095	0.056	0.849	107	0.35	23.81
T0817TS133_2-D2.rsa	37.5	0.045	0.264	0.691	76	0.493	53.18

T0817TS420_1-D2.rsa	37.5	0.199	0	0.801	109	0.344	13.23
T0817TS436_1-D2.rsa	36.842	0.538	0.055	0.407	96	0.384	58.58
T0817TS216_1-D2.rsa	36.842	0.045	0.054	0.901	100	0.368	16.89
T0817TS499_2-D2.rsa	36.538	0.279	0.036	0.685	113	0.323	19.62
T0817TS300_5-D2.rsa	36.111	0.46	0.103	0.437	93	0.388	75.89
T0817TS410_3-D2.rsa	35.714	0.143	0.175	0.683	86	0.415	50
T0817TS499_4-D2.rsa	35.714	0.119	0.048	0.833	105	0.34	22.42
T0817TS011_4-D2.rsa	35.616	0.733	0	0.267	68	0.524	59.02
T0817TS156_3-D2.rsa	35.429	0.567	0.03	0.403	163	0.217	11.63
T0817TS193_4-D2.rsa	35.211	0	0.22	0.78	131	0.269	11.06
T0817TS277_2-D2.rsa	35.211	0.494	0.149	0.357	60	0.587	51.92
T0817TS492_2-D2.rsa	35	0	0.123	0.877	100	0.35	53.51
T0817TS448_1-D2.rsa	35	0	0.167	0.833	95	0.368	17.76
T0817TS263_1-D2.rsa	35	0.018	0.158	0.825	94	0.372	30.04
T0817TS454_4-D2.rsa	35	0.026	0.254	0.719	82	0.427	26.32
T0817TS335_4-D2.rsa	34.884	0.299	0.075	0.627	84	0.415	42.16
T0817TS216_2-D2.rsa	34.884	0.216	0.06	0.724	97	0.36	24.44
T0817TS479_4-D2.rsa	34.483	0	0.252	0.748	107	0.322	86.71
T0817TS454_5-D2.rsa	34.483	0.641	0	0.359	92	0.375	56.74
T0817TS237_4-D2.rsa	34.247	0.753	0	0.247	63	0.544	59.61
T0817TS277_5-D2.rsa	34.211	0.198	0	0.802	89	0.384	38.06
T0817TS008_1-D2.rsa	33.929	0.095	0.175	0.73	92	0.369	21.43
T0817TS251_1-D2.rsa	33.333	0.045	0.291	0.664	73	0.457	54.77
T0817TS145_4-D2.rsa	32.877	0.757	0	0.243	62	0.53	60.59
T0817TS414_3-D2.rsa	32.759	0.625	0	0.375	96	0.341	59.28
T0817TS228_5-D2.rsa	32.759	0.703	0	0.297	76	0.431	60.25
T0817TS452_5-D2.rsa	32.558	0.343	0.195	0.462	78	0.417	73.52
T0817TS410_4-D2.rsa	32.558	0.358	0.082	0.56	75	0.434	52.8
T0817TS193_2-D2.rsa	32.558	0.299	0.037	0.664	89	0.366	25.56
T0817TS448_5-D2.rsa	32.143	0.19	0	0.81	102	0.315	20.04
T0817TS345_5-D2.rsa	31.579	0.479	0.051	0.47	111	0.284	52.97
T0817TS436_3-D2.rsa	31.579	0.508	0.055	0.436	103	0.307	58.79
T0817TS277_1-D2.rsa	31.507	0.769	0	0.231	59	0.534	70
T0817TS263_5-D2.rsa	31.395	0.308	0.16	0.533	90	0.349	51.77
T0817TS345_4-D2.rsa	31.395	0.355	0.183	0.462	78	0.403	60.8
T0817TS251_5-D2.rsa	31.25	0.015	0.338	0.647	88	0.355	70.4
T0817TS345_2-D2.rsa	31.25	0.018	0.591	0.391	43	0.727	83.64
T0817TS263_2-D2.rsa	30.882	0.37	0.082	0.547	133	0.232	61.22
T0817TS499_3-D2.rsa	30.357	0.175	0.198	0.627	79	0.384	51.79
T0817TS492_4-D2.rsa	30.233	0.302	0.195	0.503	85	0.356	70.56
T0817TS022_5-D2.rsa	30.233	0.343	0.13	0.527	89	0.34	68.79
T0817TS228_3-D2.rsa	30.172	0.645	0	0.355	91	0.332	57.32
T0817TS210_2-D2.rsa	30.137	0.745	0	0.255	65	0.464	50.2
T0817TS193_1-D2.rsa	30	0.053	0.272	0.675	77	0.39	39.25
T0817TS184_2-D2.rsa	30	0.018	0.377	0.605	69	0.435	47.59
T0817TS117_1-D2.rsa	29.688	0.103	0.331	0.566	77	0.386	60.66
T0817TS452_1-D2.rsa	29.474	0.542	0.051	0.407	96	0.307	63.14

T0817TS479_3-D2.rsa	29.412	0.366	0.091	0.543	132	0.223	66
T0817TS452_2-D2.rsa	28.571	0.444	0.209	0.346	81	0.353	70.62
T0817TS216_3-D2.rsa	28.571	0.159	0.254	0.587	74	0.386	55.95
T0817TS479_5-D2.rsa	28	0.08	0.268	0.652	90	0.311	59.6
T0817TS381_2-D2.rsa	28	0.065	0.304	0.63	87	0.322	55.98
T0817TS237_5-D2.rsa	27.907	0.302	0.201	0.497	84	0.332	72.78
T0817TS193_5-D2.rsa	27.907	0.302	0.195	0.503	85	0.328	74.11
T0817TS436_2-D2.rsa	27.5	0	0.07	0.93	106	0.259	50.22
T0817TS160_2-D2.rsa	27.5	0	0.211	0.789	90	0.306	43.2
T0817TS454_3-D2.rsa	27.5	0	0.149	0.851	97	0.284	28.95
T0817TS160_1-D2.rsa	27.368	0.53	0.051	0.419	99	0.276	57.1
T0817TS041_3-D2.rsa	26.786	0.206	0	0.794	100	0.268	45.04
T0817TS117_5-D2.rsa	25.85	0.402	0.126	0.472	176	0.147	88.89
T0817TS011_1-D2.rsa	25.581	0.355	0.183	0.462	78	0.328	73.08
T0817TS160_5-D2.rsa	24	0.458	0.124	0.418	105	0.229	90.24
T0817TS279_1-D2.rsa	23.077	0.436	0.074	0.49	145	0.159	59.03
T0817TS420_2-D2.rsa	22.5	0	0.289	0.711	81	0.278	44.3
T0817TS263_4-D2.rsa	22.5	0.018	0.333	0.649	74	0.304	42.54
T0817TS193_3-D2.rsa	21.429	0.222	0	0.778	98	0.219	41.67
T0817TS156_2-D2.rsa	20	0.072	0.391	0.536	74	0.27	72.83
T0817TS160_4-D2.rsa	20	0.08	0.341	0.58	80	0.25	62.14
T0817TS300_3-D2.rsa	17.544	0.457	0.091	0.452	99	0.177	81.39
T0817TS038_3-D2.rsa	17.544	0.438	0.096	0.466	102	0.172	51.26
T0817TS251_2-D2.rsa	16	0.022	0.261	0.717	99	0.162	48.37
T0817TS145_1-D2.rsa	16	0.036	0.29	0.674	93	0.172	48.73
T0817TS117_3-D2.rsa	13.077	0.258	0.229	0.513	235	0.056	75.94
T0877TS464_2-D1.rsa	89.063	0.19	0	0.81	115	0.774	13.03
T0877TS464_5-D1.rsa	89.063	0.155	0	0.845	120	0.742	13.73
T0877TS284_2-D1.rsa	81.25	0.169	0.056	0.775	110	0.739	13.38
T0877TS284_1-D1.rsa	78.125	0.211	0.056	0.732	104	0.751	13.56
T0877TS284_3-D1.rsa	75	0.211	0.028	0.761	108	0.694	13.38
T0877TS451_3-D1.rsa	67.188	0.162	0.056	0.782	111	0.605	18.13
T0877TS451_1-D1.rsa	65.625	0.176	0.035	0.789	112	0.586	18.31
T0877TS321_3-D1.rsa	65.625	0.232	0.056	0.711	101	0.65	16.73
T0877TS455_1-D1.rsa	64.063	0.028	0	0.972	138	0.464	12.15
T0877TS321_4-D1.rsa	64.063	0.239	0.056	0.704	100	0.641	22.01
T0877TS451_5-D1.rsa	64.063	0.169	0.042	0.789	112	0.572	19.89
T0877TS321_2-D1.rsa	62.5	0.232	0.056	0.711	101	0.619	16.55
T0877TS451_2-D1.rsa	60.938	0.197	0.07	0.732	104	0.586	25.18
T0877TS284_5-D1.rsa	60.938	0.063	0.07	0.866	123	0.495	12.68
T0877TS451_4-D1.rsa	59.375	0.176	0.028	0.796	113	0.525	19.37
T0877TS455_5-D1.rsa	59.375	0	0.028	0.972	138	0.43	12.15
T0877TS026_5-D1.rsa	56.25	0.134	0.169	0.697	99	0.568	20.42
T0877TS432_3-D1.rsa	51.563	0.211	0.12	0.669	95	0.543	24.65
T0877TS321_5-D1.rsa	50	0.19	0.127	0.683	97	0.515	15.32
T0877TS284_4-D1.rsa	50	0.141	0.077	0.782	111	0.45	16.55
T0877TS452_5-D1.rsa	48.438	0.211	0.12	0.669	95	0.51	21.48

T0877TS467_4-D1.rsa	48.438	0.282	0.07	0.648	92	0.526	17.78
T0877TS321_1-D1.rsa	46.875	0.183	0.127	0.69	98	0.478	15.85
T0877TS455_2-D1.rsa	46.875	0.021	0.021	0.958	136	0.345	11.8
T0877TS432_1-D1.rsa	46.875	0.19	0.204	0.606	86	0.545	20.95
T0877TS434_3-D1.rsa	46.875	0.246	0	0.754	107	0.438	16.2
T0877TS251_5-D1.rsa	45.313	0.303	0.19	0.507	72	0.629	24.3
T0877TS432_2-D1.rsa	45.313	0.204	0.141	0.655	93	0.487	22.01
T0877TS180_4-D1.rsa	43.75	0.366	0.12	0.514	73	0.599	16.02
T0877TS434_5-D1.rsa	43.75	0.239	0	0.761	108	0.405	15.14
T0877TS434_1-D1.rsa	43.75	0.254	0.014	0.732	104	0.421	17.25
T0877TS446_5-D1.rsa	43.75	0.176	0.197	0.627	89	0.492	39.26
T0877TS180_2-D1.rsa	42.188	0.162	0.092	0.746	106	0.398	24.82
T0877TS434_2-D1.rsa	42.188	0.225	0.085	0.69	98	0.43	17.61
T0877TS357_5-D1.rsa	42.188	0.19	0.014	0.796	113	0.373	59.16
T0877TS455_3-D1.rsa	42.188	0.021	0.204	0.775	110	0.384	13.2
T0877TS452_3-D1.rsa	40.625	0.19	0.148	0.662	94	0.432	42.25
T0877TS467_5-D1.rsa	40.625	0.254	0.028	0.718	102	0.398	19.19
T0877TS421_4-D1.rsa	40.625	0.12	0.113	0.768	109	0.373	55.63
T0877TS180_1-D1.rsa	39.063	0.225	0.204	0.57	81	0.482	51.23
T0877TS432_4-D1.rsa	39.063	0.218	0.19	0.592	84	0.465	24.3
T0877TS432_5-D1.rsa	39.063	0.232	0.148	0.62	88	0.444	27.11
T0877TS258_4-D1.rsa	37.5	0.197	0.204	0.599	85	0.441	61.97
T0877TS258_2-D1.rsa	37.5	0.19	0.197	0.613	87	0.431	64.26
T0877TS455_4-D1.rsa	37.5	0	0.014	0.986	140	0.268	13.38
T0877TS407_4-D1.rsa	37.5	0.19	0.169	0.641	91	0.412	62.85
T0877TS005_3-D1.rsa	37.5	0.239	0.254	0.507	72	0.521	68.31
T0877TS467_2-D1.rsa	37.5	0.204	0.155	0.641	91	0.412	61.09
T0877TS467_1-D1.rsa	37.5	0.218	0.183	0.599	85	0.441	62.15
T0877TS452_1-D1.rsa	37.5	0.197	0.197	0.606	86	0.436	62.32
T0877TS357_2-D1.rsa	37.5	0.211	0	0.789	112	0.335	53.17
T0877TS421_3-D1.rsa	35.938	0.169	0.176	0.655	93	0.386	61.44
T0877TS180_3-D1.rsa	35.938	0.232	0.169	0.599	85	0.423	17.43
T0877TS005_4-D1.rsa	35.938	0.225	0.218	0.556	79	0.455	66.02
T0877TS005_1-D1.rsa	35.938	0.232	0.268	0.5	71	0.506	67.08
T0877TS183_1-D1.rsa	35.938	0.204	0.12	0.676	96	0.374	65.14
T0877TS382_1-D1.rsa	35.938	0.225	0.092	0.683	97	0.37	52.29
T0877TS382_4-D1.rsa	35.938	0.239	0.099	0.662	94	0.382	52.47
T0877TS405_3-D1.rsa	35.938	0.19	0.211	0.599	85	0.423	65.14
T0877TS357_1-D1.rsa	35.938	0.19	0	0.81	115	0.313	55.99
T0877TS434_4-D1.rsa	35.938	0.211	0	0.789	112	0.321	18.66
T0877TS444_1-D1.rsa	35.938	0.218	0.19	0.592	84	0.428	53.34
T0877TS220_2-D1.rsa	35.938	0.218	0.232	0.549	78	0.461	66.2
T0877TS382_2-D1.rsa	35.938	0.218	0.099	0.683	97	0.37	51.94
T0877TS407_5-D1.rsa	35.938	0.211	0.162	0.627	89	0.404	65.32
T0877TS183_5-D1.rsa	34.375	0.183	0.148	0.669	95	0.362	43.66
T0877TS077_1-D1.rsa	34.375	0.169	0.085	0.746	106	0.324	56.16
T0877TS077_5-D1.rsa	34.375	0.197	0.077	0.725	103	0.334	55.46

T0877TS446_4-D1.rsa	34.375	0.19	0.218	0.592	84	0.409	60.21
T0877TS250_3-D1.rsa	34.375	0.268	0.134	0.599	85	0.404	67.78
T0877TS452_4-D1.rsa	34.375	0.239	0.169	0.592	84	0.409	57.92
T0877TS345_3-D1.rsa	34.375	0.211	0.211	0.577	82	0.419	64.61
T0877TS236_5-D1.rsa	34.375	0.176	0.204	0.62	88	0.391	66.02
T0877TS236_3-D1.rsa	34.375	0.218	0.183	0.599	85	0.404	66.72
T0877TS275_5-D1.rsa	34.375	0.211	0.127	0.662	94	0.366	59.86
T0877TS236_1-D1.rsa	34.375	0.19	0.239	0.57	81	0.424	67.78
T0877TS005_2-D1.rsa	34.375	0.239	0.254	0.507	72	0.477	69.01
T0877TS251_3-D1.rsa	34.375	0.218	0.19	0.592	84	0.409	62.85
T0877TS382_3-D1.rsa	34.375	0.225	0.092	0.683	97	0.354	51.76
T0877TS357_4-D1.rsa	34.375	0.218	0.014	0.768	109	0.315	57.22
T0877TS313_4-D1.rsa	32.813	0.176	0.197	0.627	89	0.369	64.79
T0877TS479_1-D1.rsa	32.813	0.218	0.148	0.634	90	0.365	65.32
T0877TS220_5-D1.rsa	32.813	0.218	0.19	0.592	84	0.391	69.72
T0877TS479_5-D1.rsa	32.813	0.169	0.204	0.627	89	0.369	44.01
T0877TS446_3-D1.rsa	32.813	0.218	0.183	0.599	85	0.386	60.91
T0877TS251_1-D1.rsa	32.813	0.218	0.204	0.577	82	0.4	61.09
T0877TS421_1-D1.rsa	32.813	0.176	0.169	0.655	93	0.353	62.85
T0877TS345_2-D1.rsa	32.813	0.197	0.197	0.606	86	0.382	66.37
T0877TS077_3-D1.rsa	32.813	0.169	0.085	0.746	106	0.31	54.75
T0877TS251_2-D1.rsa	32.813	0.218	0.19	0.592	84	0.391	63.38
T0877TS349_1-D1.rsa	32.813	0.183	0.169	0.648	92	0.357	63.56
T0877TS220_4-D1.rsa	32.813	0.225	0.197	0.577	82	0.4	69.37
T0877TS421_5-D1.rsa	32.813	0.141	0.056	0.803	114	0.288	54.4
T0877TS405_4-D1.rsa	32.813	0.204	0.19	0.606	86	0.382	63.03
T0877TS258_5-D1.rsa	32.813	0.204	0.204	0.592	84	0.391	63.56
T0877TS407_3-D1.rsa	32.813	0.197	0.169	0.634	90	0.365	64.61
T0877TS258_1-D1.rsa	32.813	0.197	0.183	0.62	88	0.373	67.08
T0877TS357_3-D1.rsa	32.813	0.169	0.014	0.817	116	0.283	56.34
T0877TS026_3-D1.rsa	32.813	0.197	0.19	0.613	87	0.377	63.03
T0877TS250_1-D1.rsa	32.813	0.268	0.134	0.599	85	0.386	67.25
T0877TS382_5-D1.rsa	32.813	0.232	0.077	0.69	98	0.335	52.29
T0877TS313_5-D1.rsa	32.813	0.176	0.197	0.627	89	0.369	64.97
T0877TS005_5-D1.rsa	32.813	0.19	0.225	0.585	83	0.395	67.43
T0877TS026_1-D1.rsa	32.813	0.19	0.19	0.62	88	0.373	63.91
T0877TS421_2-D1.rsa	31.25	0.197	0.204	0.599	85	0.368	62.15
T0877TS183_4-D1.rsa	31.25	0.197	0.155	0.648	92	0.34	43.84
T0877TS479_2-D1.rsa	31.25	0.268	0.155	0.577	82	0.381	59.86
T0877TS345_1-D1.rsa	31.25	0.211	0.204	0.585	83	0.377	68.66
T0877TS258_3-D1.rsa	31.25	0.176	0.197	0.627	89	0.351	63.56
T0877TS236_2-D1.rsa	31.25	0.211	0.204	0.585	83	0.377	67.43
T0877TS495_4-D1.rsa	31.25	0.197	0.12	0.683	97	0.322	65.67
T0877TS220_1-D1.rsa	31.25	0.225	0.19	0.585	83	0.377	70.25
T0877TS446_2-D1.rsa	31.25	0.218	0.183	0.599	85	0.368	61.97
T0877TS220_3-D1.rsa	31.25	0.218	0.232	0.549	78	0.401	69.89
T0877TS275_2-D1.rsa	31.25	0.246	0.239	0.514	73	0.428	64.79

T0877TS251_4-D1.rsa	31.25	0.197	0.183	0.62	88	0.355	59.86
T0877TS444_2-D1.rsa	31.25	0.197	0.197	0.606	86	0.363	60.03
T0877TS407_2-D1.rsa	31.25	0.197	0.183	0.62	88	0.355	62.68
T0877TS287_4-D1.rsa	31.25	0.232	0.211	0.556	79	0.396	66.72
T0877TS048_1-D1.rsa	31.25	0.218	0.183	0.599	85	0.368	67.61
T0877TS446_1-D1.rsa	31.25	0.218	0.183	0.599	85	0.368	61.62
T0877TS077_2-D1.rsa	31.25	0.197	0.141	0.662	94	0.332	58.27
T0877TS467_3-D1.rsa	31.25	0.197	0.162	0.641	91	0.343	58.8
T0877TS430_5-D1.rsa	31.25	0.134	0.225	0.641	91	0.343	44.72
T0877TS180_5-D1.rsa	31.25	0.19	0	0.81	115	0.272	17.25
T0877TS183_2-D1.rsa	29.688	0.275	0.169	0.556	79	0.376	57.75
T0877TS495_2-D1.rsa	29.688	0.218	0.106	0.676	96	0.309	66.37
T0877TS313_2-D1.rsa	29.688	0.176	0.204	0.62	88	0.337	64.97
T0877TS313_3-D1.rsa	29.688	0.176	0.204	0.62	88	0.337	66.02
T0877TS479_4-D1.rsa	29.688	0.218	0.155	0.627	89	0.334	42.78
T0877TS405_1-D1.rsa	29.688	0.19	0.176	0.634	90	0.33	64.97
T0877TS313_1-D1.rsa	29.688	0.176	0.204	0.62	88	0.337	65.32
T0877TS119_1-D1.rsa	29.688	0.183	0.169	0.648	92	0.323	63.56
T0877TS407_1-D1.rsa	29.688	0.19	0.169	0.641	91	0.326	63.73
T0877TS345_4-D1.rsa	29.688	0.225	0.204	0.57	81	0.367	64.61
T0877TS250_4-D1.rsa	29.688	0.261	0.141	0.599	85	0.349	67.43
T0877TS250_5-D1.rsa	29.688	0.254	0.12	0.627	89	0.334	67.43
T0877TS452_2-D1.rsa	29.688	0.19	0.183	0.627	89	0.334	64.97
T0877TS275_3-D1.rsa	29.688	0.19	0.183	0.627	89	0.334	65.32
T0877TS287_1-D1.rsa	29.688	0.218	0.211	0.57	81	0.367	67.78
T0877TS430_2-D1.rsa	29.688	0.183	0.155	0.662	94	0.316	43.84
T0877TS425_5-D1.rsa	28.125	0.19	0.077	0.732	104	0.27	57.75
T0877TS275_1-D1.rsa	28.125	0.204	0.218	0.577	82	0.343	64.61
T0877TS077_4-D1.rsa	28.125	0.19	0.141	0.669	95	0.296	57.22
T0877TS495_3-D1.rsa	28.125	0.197	0.106	0.697	99	0.284	65.49
T0877TS425_4-D1.rsa	28.125	0.204	0.106	0.69	98	0.287	55.81
T0877TS425_2-D1.rsa	28.125	0.211	0.148	0.641	91	0.309	58.8
T0877TS275_4-D1.rsa	28.125	0.204	0.183	0.613	87	0.323	64.97
T0877TS495_5-D1.rsa	28.125	0.197	0.12	0.683	97	0.29	65.14
T0877TS345_5-D1.rsa	28.125	0.204	0.204	0.592	84	0.335	65.84
T0877TS464_3-D1.rsa	28.125	0.169	0.176	0.655	93	0.302	60.39
T0877TS287_5-D1.rsa	28.125	0.183	0.19	0.627	89	0.316	66.55
T0877TS250_2-D1.rsa	28.125	0.246	0.134	0.62	88	0.32	66.9
T0877TS430_3-D1.rsa	28.125	0.169	0.19	0.641	91	0.309	43.31
T0877TS479_3-D1.rsa	26.563	0.204	0.218	0.577	82	0.324	49.65
T0877TS425_3-D1.rsa	26.563	0.169	0.134	0.697	99	0.268	58.98
T0877TS495_1-D1.rsa	26.563	0.204	0.106	0.69	98	0.271	65.14
T0877TS464_1-D1.rsa	26.563	0.169	0.176	0.655	93	0.286	60.56
T0877TS016_1-D1.rsa	26.563	0.204	0.183	0.613	87	0.305	62.85
T0877TS287_3-D1.rsa	26.563	0.176	0.19	0.634	90	0.295	66.72
T0877TS464_4-D1.rsa	26.563	0.169	0.176	0.655	93	0.286	60.21
T0877TS425_1-D1.rsa	25	0.183	0.113	0.704	100	0.25	58.27

T0877TS444_5-D1.rsa	25	0.176	0.183	0.641	91	0.275	57.22
T0877TS444_4-D1.rsa	25	0.218	0.19	0.592	84	0.298	57.22
T0877TS430_4-D1.rsa	25	0.155	0.19	0.655	93	0.269	44.19
T0877TS430_1-D1.rsa	25	0.162	0.176	0.662	94	0.266	43.66
T0877TS183_3-D1.rsa	23.438	0.261	0.183	0.556	79	0.297	49.82
T0877TS236_4-D1.rsa	23.438	0.246	0.19	0.563	80	0.293	68.13
T0877TS444_3-D1.rsa	20.313	0.218	0.204	0.577	82	0.248	57.57
T0851TS492_1-D1.rsa	76.316	0.252	0.036	0.712	79	0.966	25.9
T0851TS216_3-D1.rsa	73.684	0.153	0	0.847	94	0.784	16.44
T0851TS133_4-D1.rsa	70.833	0	0.343	0.657	71	0.998	23.15
T0851TS184_1-D1.rsa	70.588	0.595	0	0.405	51	1.384	33.73
T0851TS251_1-D1.rsa	69.231	0.297	0.024	0.679	112	0.618	24.21
T0851TS210_4-D1.rsa	68.421	0.18	0	0.82	91	0.752	19.14
T0851TS216_5-D1.rsa	65.789	0.288	0.126	0.586	65	1.012	33.33
T0851TS335_1-D1.rsa	65.789	0.252	0.036	0.712	79	0.833	24.55
T0851TS454_5-D1.rsa	64.706	0.579	0	0.421	53	1.221	35.12
T0851TS228_4-D1.rsa	63.38	0.333	0.048	0.619	104	0.609	21.15
T0851TS160_1-D1.rsa	62.791	0.433	0.045	0.522	70	0.897	41.6
T0851TS452_3-D1.rsa	62.5	0	0.306	0.694	75	0.833	19.68
T0851TS381_1-D1.rsa	60.714	0	0.336	0.664	87	0.698	14.31
T0851TS038_4-D1.rsa	60.714	0	0.344	0.656	86	0.706	14.7
T0851TS268_1-D1.rsa	60.526	0.279	0.018	0.703	78	0.776	16.44
T0851TS436_5-D1.rsa	59.615	0.248	0.121	0.63	104	0.573	41.61
T0851TS410_2-D1.rsa	58.333	0.019	0.63	0.352	38	1.535	72.92
T0851TS228_1-D1.rsa	58.333	0	0.546	0.454	49	1.19	65.97
T0851TS414_4-D1.rsa	58.333	0	0.264	0.736	81	0.72	32.73
T0851TS008_5-D1.rsa	58.333	0.176	0	0.824	89	0.655	28.7
T0851TS008_4-D1.rsa	58.333	0.019	0.648	0.333	36	1.62	68.29
T0851TS268_5-D1.rsa	58.333	0	0.528	0.472	51	1.144	62.04
T0851TS041_2-D1.rsa	57.746	0.25	0.125	0.625	105	0.55	16.99
T0851TS237_5-D1.rsa	55.882	0.428	0.033	0.539	131	0.427	21.22
T0851TS452_1-D1.rsa	55.882	0.484	0	0.516	65	0.86	33.33
T0851TS499_1-D1.rsa	54.386	0.29	0.143	0.567	123	0.442	26.5
T0851TS171_2-D1.rsa	53.846	0.158	0.085	0.758	125	0.431	36.23
T0851TS117_4-D1.rsa	53.571	0.015	0.198	0.786	103	0.52	14.5
T0851TS171_5-D1.rsa	53.488	0.201	0	0.799	107	0.5	39.74
T0851TS212_1-D1.rsa	52.941	0.619	0	0.381	48	1.103	52.58
T0851TS454_2-D1.rsa	52.632	0.288	0.108	0.604	67	0.786	39.87
T0851TS184_5-D1.rsa	52.113	0.381	0	0.619	104	0.501	13.3
T0851TS145_4-D1.rsa	51.923	0.012	0.127	0.861	142	0.366	11.23
T0851TS263_4-D1.rsa	51.923	0.279	0.055	0.667	110	0.472	15.98
T0851TS479_4-D1.rsa	50	0	0.321	0.679	89	0.562	15.08
T0851TS145_5-D1.rsa	48.438	0.015	0.287	0.699	95	0.51	42.65
T0851TS420_4-D1.rsa	47.945	0.643	0	0.357	91	0.527	26.08
T0851TS251_3-D1.rsa	47.5	0	0.202	0.798	91	0.522	24.56
T0851TS145_3-D1.rsa	46.429	0.061	0.069	0.87	114	0.407	30.34
T0851TS133_1-D1.rsa	46.429	0.159	0.063	0.778	98	0.474	15.48

T0851TS038_3-D1.rsa	46.429	0.23	0.071	0.698	88	0.528	30.36
T0851TS381_5-D1.rsa	45.313	0.103	0.147	0.75	102	0.444	12.32
T0851TS011_2-D1.rsa	45.07	0.405	0.042	0.554	93	0.485	26.28
T0851TS499_2-D1.rsa	45	0.018	0.123	0.86	98	0.459	35.31
T0851TS414_5-D1.rsa	45	0	0.132	0.868	99	0.455	33.33
T0851TS454_3-D1.rsa	45	0.035	0.202	0.763	87	0.517	34.65
T0851TS420_5-D1.rsa	44.737	0.162	0	0.838	93	0.481	30.41
T0851TS349_2-D1.rsa	44.186	0.03	0.097	0.873	117	0.378	22.39
T0851TS263_3-D1.rsa	44.186	0.246	0.09	0.664	89	0.496	30.22
T0851TS479_3-D1.rsa	43.836	0.722	0	0.278	71	0.617	35.98
T0851TS345_1-D1.rsa	43.75	0.036	0.382	0.582	64	0.684	64.09
T0851TS410_1-D1.rsa	42.857	0	0.527	0.473	62	0.691	56.3
T0851TS300_3-D1.rsa	42.857	0.167	0.143	0.69	87	0.493	34.52
T0851TS160_4-D1.rsa	42.857	0.015	0.206	0.779	102	0.42	19.08
T0851TS335_2-D1.rsa	42.188	0.029	0.169	0.801	109	0.387	13.42
T0851TS277_1-D1.rsa	42.105	0.018	0	0.982	109	0.386	29.5
T0851TS038_2-D1.rsa	41.096	0.706	0	0.294	75	0.548	38.82
T0851TS346_1-D1.rsa	40.845	0.476	0.048	0.476	80	0.511	51.76
T0851TS171_3-D1.rsa	40.278	0.418	0.075	0.507	108	0.373	61.17
T0851TS277_3-D1.rsa	40	0.036	0.167	0.797	110	0.364	18.48
T0851TS448_1-D1.rsa	39.726	0.518	0	0.482	123	0.323	51.27
T0851TS171_4-D1.rsa	39.286	0	0.031	0.969	127	0.309	27.67
T0851TS251_5-D1.rsa	39.063	0.015	0.456	0.529	72	0.543	63.42
T0851TS145_1-D1.rsa	39.063	0.015	0.375	0.61	83	0.471	71.14
T0851TS206_1-D1.rsa	39.063	0.015	0.338	0.647	88	0.444	63.79
T0851TS184_3-D1.rsa	39.063	0.14	0.088	0.772	105	0.372	13.97
T0851TS300_2-D1.rsa	39.063	0.118	0.096	0.787	107	0.365	12.87
T0851TS499_3-D1.rsa	38.028	0.417	0.024	0.56	94	0.405	45.99
T0851TS237_1-D1.rsa	37.5	0.015	0.404	0.581	79	0.475	69.48
T0851TS216_4-D1.rsa	37.5	0.015	0.441	0.544	74	0.507	69.67
T0851TS454_1-D1.rsa	37.5	0.018	0.105	0.877	100	0.375	20.83
T0851TS335_3-D1.rsa	37.5	0.026	0.289	0.684	78	0.481	47.81
T0851TS160_3-D1.rsa	37.5	0.127	0.206	0.667	84	0.446	42.86
T0851TS479_1-D1.rsa	37.209	0.276	0.06	0.664	89	0.418	24.07
T0851TS050_1-D1.rsa	36.207	0.637	0	0.363	93	0.389	52.44
T0851TS410_3-D1.rsa	35.938	0.015	0.441	0.544	74	0.486	67.1
T0851TS492_5-D1.rsa	35.789	0.508	0.059	0.432	102	0.351	49.36
T0851TS008_1-D1.rsa	35.714	0.015	0.427	0.557	73	0.489	54.58
T0851TS216_1-D1.rsa	35.616	0.729	0	0.271	69	0.516	50.29
T0851TS452_4-D1.rsa	35.417	0.036	0.345	0.618	68	0.521	60.91
T0851TS117_1-D1.rsa	35	0	0.053	0.947	108	0.324	25.22
T0851TS492_4-D1.rsa	35	0	0.053	0.947	108	0.324	23.68
T0851TS041_4-D1.rsa	35	0	0.167	0.833	95	0.368	17.98
T0851TS277_5-D1.rsa	34.884	0.284	0.03	0.687	92	0.379	43.1
T0851TS263_1-D1.rsa	34.884	0.328	0.015	0.657	88	0.396	42.16
T0851TS237_4-D1.rsa	34.737	0.492	0.055	0.453	107	0.325	58.05
T0851TS228_5-D1.rsa	33.803	0.22	0.024	0.756	127	0.266	16.03

T0851TS335_5-D1.rsa	33.803	0.31	0	0.69	116	0.291	23.88
T0851TS349_5-D1.rsa	33.684	0.517	0.047	0.436	103	0.327	59.53
T0851TS171_1-D1.rsa	33.684	0.517	0.059	0.424	100	0.337	63.24
T0851TS414_1-D1.rsa	33.684	0.564	0.055	0.381	90	0.374	58.58
T0851TS160_5-D1.rsa	33.684	0.551	0.051	0.398	94	0.358	58.79
T0851TS117_2-D1.rsa	33.333	0.132	0.156	0.712	205	0.163	50.43
T0851TS479_2-D1.rsa	33.333	0	0.055	0.945	104	0.321	22.73
T0851TS184_2-D1.rsa	32.877	0.765	0	0.235	60	0.548	65
T0851TS263_5-D1.rsa	32.877	0.749	0	0.251	64	0.514	63.73
T0851TS237_2-D1.rsa	32.813	0.015	0.397	0.588	80	0.41	70.04
T0851TS133_3-D1.rsa	32.692	0.176	0.073	0.752	124	0.264	15.35
T0851TS041_5-D1.rsa	32.558	0.32	0.178	0.503	85	0.383	69.23
T0851TS499_5-D1.rsa	32.5	0.088	0.035	0.877	100	0.325	37.94
T0851TS452_2-D1.rsa	32.5	0	0.316	0.684	78	0.417	43.86
T0851TS210_3-D1.rsa	32.5	0.018	0.342	0.64	73	0.445	48.03
T0851TS251_4-D1.rsa	32	0.072	0.232	0.696	96	0.333	53.26
T0851TS381_4-D1.rsa	32	0.014	0.188	0.797	110	0.291	17.03
T0851TS268_4-D1.rsa	31.579	0.241	0.481	0.278	30	1.053	94.91
T0851TS038_1-D1.rsa	31.507	0.741	0	0.259	66	0.477	57.65
T0851TS436_2-D1.rsa	31.429	0.363	0.214	0.423	99	0.317	71.05
T0851TS499_4-D1.rsa	31.429	0.359	0.209	0.432	101	0.311	69.02
T0851TS228_3-D1.rsa	31.395	0.331	0.201	0.467	79	0.397	71.3
T0851TS216_2-D1.rsa	30.357	0.19	0.143	0.667	84	0.361	42.66
T0851TS193_1-D1.rsa	30.233	0.261	0.03	0.709	95	0.318	43.1
T0851TS277_2-D1.rsa	30.233	0.269	0.015	0.716	96	0.315	40.3
T0851TS133_5-D1.rsa	30.172	0.613	0	0.387	99	0.305	58.5
T0851TS210_2-D1.rsa	29.577	0.464	0.161	0.375	63	0.469	62.82
T0851TS145_2-D1.rsa	29.508	0.033	0.424	0.543	114	0.259	65.17
T0851TS436_1-D1.rsa	29.508	0.01	0.367	0.624	131	0.225	39.18
T0851TS381_2-D1.rsa	29.31	0.637	0	0.363	93	0.315	57.52
T0851TS492_2-D1.rsa	28.571	0	0.282	0.718	94	0.304	52.86
T0851TS228_2-D1.rsa	28.448	0.664	0	0.336	86	0.331	60.94
T0851TS117_3-D1.rsa	28.421	0.534	0.055	0.411	97	0.293	56.99
T0851TS349_4-D1.rsa	28	0.065	0.239	0.696	96	0.292	65.04
T0851TS349_1-D1.rsa	28	0.094	0.283	0.623	86	0.326	61.77
T0851TS410_5-D1.rsa	28	0.043	0.304	0.652	90	0.311	50.91
T0851TS479_5-D1.rsa	28	0.014	0.232	0.754	104	0.269	47.46
T0851TS008_2-D1.rsa	28	0.08	0.362	0.558	77	0.364	65.76
T0851TS041_1-D1.rsa	28	0.08	0.348	0.572	79	0.354	58.51
T0851TS420_3-D1.rsa	27.5	0	0.36	0.64	73	0.377	46.93
T0851TS436_3-D1.rsa	26.471	0.395	0.074	0.531	129	0.205	67.44
T0851TS210_5-D1.rsa	26.316	0.538	0.059	0.403	95	0.277	55.72
T0851TS041_3-D1.rsa	26.286	0.493	0.015	0.493	199	0.132	39.23
T0851TS117_5-D1.rsa	25.85	0.378	0.107	0.515	192	0.135	84.17
T0851TS492_3-D1.rsa	25.581	0.302	0.201	0.497	84	0.305	71.75
T0851TS335_4-D1.rsa	25.581	0.349	0.219	0.432	73	0.35	69.08
T0851TS414_2-D1.rsa	25	0	0.035	0.965	110	0.227	34.87

T0851TS300_1-D1.rsa	25	0.018	0.555	0.427	47	0.532	83.18
T0851TS300_4-D1.rsa	24	0.072	0.268	0.659	91	0.264	68.12
T0851TS448_3-D1.rsa	23.077	0.416	0.145	0.439	130	0.178	55.56
T0851TS448_4-D1.rsa	21.795	0.409	0.128	0.463	137	0.159	51.48
T0851TS448_2-D1.rsa	21.795	0.466	0.128	0.405	120	0.182	61.72
T0851TS436_4-D1.rsa	20.779	0.366	0.147	0.487	109	0.191	78.33
T0851TS008_3-D1.rsa	20	0.072	0.341	0.587	81	0.247	60.69
T0851TS277_4-D1.rsa	16	0.014	0.203	0.783	108	0.148	47.83
T0851TS420_2-D1.rsa	16	0.036	0.29	0.674	93	0.172	48.19
T0851TS237_3-D1.rsa	15.584	0.344	0.138	0.518	116	0.134	72.98
T0851TS251_2-D1.rsa	14.035	0.457	0.078	0.466	102	0.138	81.28
T0883TS464_5-D1.rsa	70.093	0.083	0.009	0.908	197	0.356	12.79
T0883TS321_4-D1.rsa	67.29	0.051	0.166	0.783	170	0.396	8.41
T0883TS464_2-D1.rsa	62.617	0.106	0.018	0.876	190	0.33	11.06
T0883TS321_5-D1.rsa	62.617	0.065	0.143	0.793	172	0.364	9.22
T0883TS321_2-D1.rsa	57.944	0.065	0.138	0.797	173	0.335	9.91
T0883TS451_2-D1.rsa	57.009	0.083	0.046	0.871	189	0.302	16.13
T0883TS321_1-D1.rsa	55.14	0.074	0.143	0.783	170	0.324	10.71
T0883TS451_4-D1.rsa	54.206	0.083	0.009	0.908	197	0.275	18.2
T0883TS451_3-D1.rsa	54.206	0.088	0.041	0.871	189	0.287	17.51
T0883TS321_3-D1.rsa	53.271	0.069	0.143	0.788	171	0.312	9.56
T0883TS451_5-D1.rsa	52.336	0.083	0.009	0.908	197	0.266	17.97
T0883TS455_5-D1.rsa	51.402	0.018	0.065	0.917	199	0.258	8.06
T0883TS434_5-D1.rsa	49.533	0.097	0.018	0.885	192	0.258	11.64
T0883TS451_1-D1.rsa	48.598	0.083	0.028	0.889	193	0.252	19.01
T0883TS434_4-D1.rsa	43.925	0.083	0.009	0.908	197	0.223	11.98
T0883TS432_5-D1.rsa	41.121	0.143	0.092	0.765	166	0.248	35.25
T0883TS434_2-D1.rsa	41.121	0.101	0.009	0.889	193	0.213	15.55
T0883TS432_4-D1.rsa	38.318	0.134	0.115	0.751	163	0.235	35.48
T0883TS434_1-D1.rsa	38.318	0.101	0.009	0.889	193	0.199	12.9
T0883TS452_5-D1.rsa	37.383	0	0.332	0.668	145	0.258	21.54
T0883TS432_2-D1.rsa	36.449	0.138	0.18	0.682	148	0.246	35.95
T0883TS434_3-D1.rsa	36.449	0.069	0	0.931	202	0.18	14.52
T0883TS432_3-D1.rsa	35.514	0.147	0.065	0.788	171	0.208	30.76
T0883TS432_1-D1.rsa	35.514	0.134	0.138	0.728	158	0.225	37.67
T0883TS455_2-D1.rsa	33.645	0.055	0.189	0.756	164	0.205	53.8
T0883TS455_1-D1.rsa	32.71	0.055	0.29	0.654	142	0.23	73.85
T0883TS236_5-D1.rsa	32.71	0.111	0.267	0.622	135	0.242	86.98
T0883TS287_4-D1.rsa	31.776	0.097	0.286	0.618	134	0.237	86.87
T0883TS236_2-D1.rsa	31.776	0.115	0.267	0.618	134	0.237	88.59
T0883TS479_2-D1.rsa	30.841	0.115	0.207	0.677	147	0.21	89.29
T0883TS425_1-D1.rsa	30.841	0.111	0.286	0.604	131	0.235	87.9
T0883TS251_2-D1.rsa	30.841	0.106	0.258	0.636	138	0.223	86.75
T0883TS287_1-D1.rsa	30.841	0.111	0.281	0.608	132	0.234	88.25
T0883TS345_3-D1.rsa	30.841	0.111	0.318	0.571	124	0.249	88.13
T0883TS236_3-D1.rsa	30.841	0.115	0.267	0.618	134	0.23	88.13
T0883TS345_2-D1.rsa	30.841	0.115	0.332	0.553	120	0.257	86.75

T0883TS345_4-D1.rsa	30.841	0.092	0.332	0.576	125	0.247	87.33
T0883TS183_4-D1.rsa	29.907	0.106	0.286	0.608	132	0.227	89.06
T0883TS183_3-D1.rsa	29.907	0.115	0.226	0.659	143	0.209	88.71
T0883TS251_3-D1.rsa	29.907	0.111	0.258	0.631	137	0.218	87.1
T0883TS479_4-D1.rsa	29.907	0.115	0.244	0.641	139	0.215	88.94
T0883TS077_5-D1.rsa	29.907	0.101	0.29	0.608	132	0.227	88.36
T0883TS313_4-D1.rsa	29.907	0.124	0.253	0.622	135	0.222	86.87
T0883TS345_1-D1.rsa	29.907	0.115	0.3	0.585	127	0.235	87.67
T0883TS180_4-D1.rsa	29.907	0.111	0.276	0.613	133	0.225	45.05
T0883TS287_5-D1.rsa	29.907	0.097	0.29	0.613	133	0.225	86.06
T0883TS287_2-D1.rsa	29.907	0.101	0.276	0.622	135	0.222	88.71
T0883TS467_3-D1.rsa	29.907	0.097	0.276	0.627	136	0.22	79.95
T0883TS251_4-D1.rsa	29.907	0.106	0.267	0.627	136	0.22	82.6
T0883TS026_4-D1.rsa	29.907	0.111	0.244	0.645	140	0.214	85.83
T0883TS464_3-D1.rsa	29.907	0.106	0.263	0.631	137	0.218	87.67
T0883TS251_1-D1.rsa	29.907	0.101	0.24	0.659	143	0.209	83.3
T0883TS026_1-D1.rsa	29.907	0.111	0.267	0.622	135	0.222	84.45
T0883TS026_2-D1.rsa	29.907	0.088	0.244	0.668	145	0.206	85.14
T0883TS345_5-D1.rsa	29.907	0.106	0.327	0.567	123	0.243	87.33
T0883TS220_1-D1.rsa	29.907	0.12	0.29	0.59	128	0.234	89.29
T0883TS183_2-D1.rsa	28.972	0.115	0.203	0.682	148	0.196	87.9
T0883TS077_2-D1.rsa	28.972	0.092	0.272	0.636	138	0.21	88.25
T0883TS077_4-D1.rsa	28.972	0.111	0.267	0.622	135	0.215	88.25
T0883TS236_4-D1.rsa	28.972	0.12	0.276	0.604	131	0.221	87.56
T0883TS275_5-D1.rsa	28.972	0.12	0.267	0.613	133	0.218	88.02
T0883TS180_3-D1.rsa	28.972	0.124	0.24	0.636	138	0.21	57.37
T0883TS275_3-D1.rsa	28.972	0.101	0.276	0.622	135	0.215	87.67
T0883TS287_3-D1.rsa	28.972	0.106	0.281	0.613	133	0.218	88.83
T0883TS407_3-D1.rsa	28.972	0.092	0.244	0.664	144	0.201	80.99
T0883TS407_1-D1.rsa	28.972	0.092	0.198	0.71	154	0.188	82.37
T0883TS275_2-D1.rsa	28.972	0.124	0.267	0.608	132	0.219	88.71
T0883TS452_1-D1.rsa	28.972	0.111	0.281	0.608	132	0.219	88.59
T0883TS180_1-D1.rsa	28.972	0.083	0.267	0.65	141	0.205	67.74
T0883TS220_4-D1.rsa	28.972	0.111	0.29	0.599	130	0.223	89.17
T0883TS382_2-D1.rsa	28.972	0.101	0.281	0.618	134	0.216	84.91
T0883TS425_2-D1.rsa	28.037	0.106	0.295	0.599	130	0.216	88.02
T0883TS258_2-D1.rsa	28.037	0.111	0.189	0.7	152	0.184	86.75
T0883TS405_2-D1.rsa	28.037	0.111	0.276	0.613	133	0.211	87.44
T0883TS425_4-D1.rsa	28.037	0.101	0.263	0.636	138	0.203	88.25
T0883TS077_3-D1.rsa	28.037	0.101	0.286	0.613	133	0.211	88.48
T0883TS452_4-D1.rsa	28.037	0.097	0.29	0.613	133	0.211	81.45
T0883TS425_5-D1.rsa	28.037	0.115	0.276	0.608	132	0.212	88.36
T0883TS048_1-D1.rsa	28.037	0.124	0.281	0.594	129	0.217	85.83
T0883TS407_5-D1.rsa	28.037	0.097	0.198	0.705	153	0.183	81.8
T0883TS407_4-D1.rsa	28.037	0.088	0.217	0.696	151	0.186	81.57
T0883TS405_1-D1.rsa	28.037	0.106	0.272	0.622	135	0.208	88.02
T0883TS467_1-D1.rsa	28.037	0.106	0.272	0.622	135	0.208	82.26

T0883TS382_3-D1.rsa	28.037	0.101	0.281	0.618	134	0.209	85.14
T0883TS220_5-D1.rsa	28.037	0.12	0.286	0.594	129	0.217	88.59
T0883TS183_1-D1.rsa	27.103	0.106	0.24	0.654	142	0.191	88.83
T0883TS479_3-D1.rsa	27.103	0.111	0.203	0.687	149	0.182	87.56
T0883TS258_5-D1.rsa	27.103	0.111	0.263	0.627	136	0.199	86.75
T0883TS119_1-D1.rsa	27.103	0.12	0.253	0.627	136	0.199	87.1
T0883TS258_1-D1.rsa	27.103	0.111	0.212	0.677	147	0.184	86.87
T0883TS425_3-D1.rsa	27.103	0.092	0.281	0.627	136	0.199	87.79
T0883TS452_3-D1.rsa	27.103	0.101	0.286	0.613	133	0.204	88.59
T0883TS455_3-D1.rsa	27.103	0.065	0.3	0.636	138	0.196	81.91
T0883TS313_2-D1.rsa	27.103	0.12	0.253	0.627	136	0.199	86.98
T0883TS464_1-D1.rsa	27.103	0.106	0.272	0.622	135	0.201	89.17
T0883TS250_1-D1.rsa	27.103	0.115	0.286	0.599	130	0.208	87.33
T0883TS479_1-D1.rsa	27.103	0.115	0.263	0.622	135	0.201	88.59
T0883TS236_1-D1.rsa	27.103	0.12	0.286	0.594	129	0.21	89.4
T0883TS467_4-D1.rsa	27.103	0.115	0.286	0.599	130	0.208	82.14
T0883TS251_5-D1.rsa	27.103	0.101	0.253	0.645	140	0.194	85.14
T0883TS275_4-D1.rsa	27.103	0.12	0.272	0.608	132	0.205	89.29
T0883TS467_5-D1.rsa	27.103	0.097	0.276	0.627	136	0.199	76.61
T0883TS313_1-D1.rsa	27.103	0.12	0.253	0.627	136	0.199	87.33
T0883TS180_2-D1.rsa	27.103	0.12	0.258	0.622	135	0.201	65.55
T0883TS382_4-D1.rsa	27.103	0.101	0.281	0.618	134	0.202	85.02
T0883TS220_3-D1.rsa	27.103	0.101	0.295	0.604	131	0.207	88.59
T0883TS220_2-D1.rsa	27.103	0.111	0.281	0.608	132	0.205	87.9
T0883TS077_1-D1.rsa	26.168	0.083	0.29	0.627	136	0.192	87.67
T0883TS405_5-D1.rsa	26.168	0.111	0.272	0.618	134	0.195	87.9
T0883TS357_2-D1.rsa	26.168	0.055	0.009	0.935	203	0.129	85.48
T0883TS313_3-D1.rsa	26.168	0.12	0.253	0.627	136	0.192	87.1
T0883TS005_3-D1.rsa	26.168	0.138	0.295	0.567	123	0.213	87.21
T0883TS467_2-D1.rsa	26.168	0.111	0.281	0.608	132	0.198	82.14
T0883TS464_4-D1.rsa	26.168	0.106	0.272	0.622	135	0.194	88.94
T0883TS016_1-D1.rsa	26.168	0.106	0.286	0.608	132	0.198	89.4
T0883TS005_4-D1.rsa	26.168	0.129	0.295	0.576	125	0.209	89.4
T0883TS382_1-D1.rsa	26.168	0.101	0.281	0.618	134	0.195	85.02
T0883TS183_5-D1.rsa	25.234	0.106	0.23	0.664	144	0.175	88.71
T0883TS357_5-D1.rsa	25.234	0.065	0.037	0.899	195	0.129	85.94
T0883TS250_2-D1.rsa	25.234	0.111	0.286	0.604	131	0.193	87.21
T0883TS455_4-D1.rsa	25.234	0.06	0.166	0.774	168	0.15	58.06
T0883TS250_3-D1.rsa	25.234	0.115	0.286	0.599	130	0.194	87.33
T0883TS444_4-D1.rsa	25.234	0.115	0.286	0.599	130	0.194	83.41
T0883TS258_4-D1.rsa	25.234	0.111	0.221	0.668	145	0.174	88.02
T0883TS357_4-D1.rsa	25.234	0.06	0.046	0.894	194	0.13	86.17
T0883TS382_5-D1.rsa	25.234	0.101	0.281	0.618	134	0.188	84.91
T0883TS258_3-D1.rsa	24.299	0.111	0.23	0.659	143	0.17	87.9
T0883TS479_5-D1.rsa	24.299	0.106	0.23	0.664	144	0.169	88.94
T0883TS250_5-D1.rsa	24.299	0.115	0.286	0.599	130	0.187	87.33
T0883TS357_3-D1.rsa	24.299	0.055	0.023	0.922	200	0.121	85.83

T0883TS407_2-D1.rsa	24.299	0.088	0.249	0.664	144	0.169	83.06
T0883TS275_1-D1.rsa	24.299	0.12	0.286	0.594	129	0.188	89.4
T0883TS444_5-D1.rsa	24.299	0.12	0.313	0.567	123	0.198	85.14
T0883TS444_1-D1.rsa	24.299	0.083	0.3	0.618	134	0.181	80.53
T0883TS452_2-D1.rsa	23.364	0.106	0.29	0.604	131	0.178	87.9
T0883TS180_5-D1.rsa	23.364	0.074	0	0.926	201	0.116	13.71
T0883TS250_4-D1.rsa	23.364	0.111	0.286	0.604	131	0.178	87.1
T0883TS005_1-D1.rsa	23.364	0.12	0.3	0.581	126	0.185	88.94
T0883TS444_2-D1.rsa	23.364	0.111	0.332	0.558	121	0.193	81.22
T0883TS357_1-D1.rsa	23.364	0.06	0.018	0.922	200	0.117	86.41
T0883TS005_5-D1.rsa	23.364	0.12	0.295	0.585	127	0.184	88.25
T0883TS005_2-D1.rsa	23.364	0.12	0.295	0.585	127	0.184	84.91
T0883TS444_3-D1.rsa	21.495	0.115	0.309	0.576	125	0.172	82.95
T0762TS263_1-D1.rsa	91.071	0.111	0.071	0.817	103	0.884	24.01
T0762TS448_1-D1.rsa	83.099	0.494	0	0.506	85	0.978	12.66
T0762TS212_1-D1.rsa	79.412	0.492	0	0.508	64	1.241	28.57
T0762TS041_3-D1.rsa	76.471	0.508	0	0.492	62	1.233	32.74
T0762TS145_1-D1.rsa	70.588	0.587	0	0.413	52	1.357	32.94
T0762TS345_2-D1.rsa	68.75	0.1	0.282	0.618	68	1.011	23.86
T0762TS263_4-D1.rsa	65.789	0.117	0.018	0.865	96	0.685	35.36
T0762TS022_1-D1.rsa	63.462	0.261	0	0.739	122	0.52	14.71
T0762TS237_3-D1.rsa	62.5	0	0.472	0.528	57	1.096	58.56
T0762TS022_2-D1.rsa	60.714	0	0.321	0.679	89	0.682	48.09
T0762TS414_3-D1.rsa	60	0.058	0.196	0.746	103	0.583	14.67
T0762TS452_1-D1.rsa	60	0.044	0.035	0.921	105	0.571	16.23
T0762TS300_5-D1.rsa	58.333	0.019	0.63	0.352	38	1.535	64.12
T0762TS448_2-D1.rsa	58.333	0	0.565	0.435	47	1.241	63.43
T0762TS414_2-D1.rsa	58.333	0	0.537	0.463	50	1.167	54.63
T0762TS184_2-D1.rsa	58.333	0.019	0.593	0.389	42	1.389	71.53
T0762TS479_3-D1.rsa	57.895	0.243	0.045	0.712	79	0.733	35.13
T0762TS263_2-D1.rsa	57.692	0.303	0.085	0.612	101	0.571	72.31
T0762TS156_5-D1.rsa	56.338	0.44	0.071	0.488	82	0.687	18.59
T0762TS300_4-D1.rsa	55.814	0.037	0.276	0.687	92	0.607	14.55
T0762TS228_1-D1.rsa	55.769	0.309	0.097	0.594	98	0.569	78.01
T0762TS499_3-D1.rsa	55.263	0.279	0.036	0.685	76	0.727	30.86
T0762TS499_4-D1.rsa	55.263	0.252	0.027	0.721	80	0.691	38.06
T0762TS184_1-D1.rsa	55	0	0.412	0.588	67	0.821	31.8
T0762TS041_5-D1.rsa	54.688	0.294	0	0.706	96	0.57	9.93
T0762TS300_2-D1.rsa	54.167	0.037	0.62	0.343	37	1.464	71.76
T0762TS206_1-D1.rsa	54.167	0	0.296	0.704	76	0.713	32.41
T0762TS381_2-D1.rsa	53.846	0.345	0.109	0.545	90	0.598	18.35
T0762TS414_5-D1.rsa	53.488	0.194	0.015	0.791	106	0.505	15.67
T0762TS436_4-D1.rsa	52.632	0.081	0	0.919	102	0.516	18.47
T0762TS492_1-D1.rsa	52.632	0.081	0	0.919	102	0.516	19.82
T0762TS346_1-D1.rsa	52.632	0.027	0.054	0.919	102	0.516	20.95
T0762TS452_3-D1.rsa	52.5	0	0.342	0.658	75	0.7	33.77
T0762TS184_3-D1.rsa	52.083	0	0.273	0.727	80	0.651	32.73

T0762TS263_3-D1.rsa	51.923	0.279	0.097	0.624	103	0.504	71.68
T0762TS237_5-D1.rsa	51.163	0.194	0.075	0.731	98	0.522	40.86
T0762TS300_3-D1.rsa	50	0.183	0.103	0.714	90	0.556	24.21
T0762TS011_1-D1.rsa	50	0.081	0.018	0.901	100	0.5	21.85
T0762TS381_5-D1.rsa	50	0.054	0	0.946	105	0.476	19.59
T0762TS381_4-D1.rsa	50	0.261	0.126	0.613	68	0.735	38.29
T0762TS237_4-D1.rsa	48.077	0.242	0.085	0.673	111	0.433	47.47
T0762TS448_3-D1.rsa	47.887	0.452	0.089	0.458	77	0.622	34.78
T0762TS156_3-D1.rsa	47.368	0.045	0	0.955	106	0.447	22.3
T0762TS277_4-D1.rsa	47.368	0.198	0.153	0.649	72	0.658	31.53
T0762TS038_4-D1.rsa	46.429	0	0.221	0.779	102	0.455	16.6
T0762TS038_3-D1.rsa	46.154	0.23	0.091	0.679	112	0.412	12.5
T0762TS008_1-D1.rsa	45.833	0	0.473	0.527	58	0.79	66.36
T0762TS420_1-D1.rsa	45.313	0	0.287	0.713	97	0.467	13.23
T0762TS492_4-D1.rsa	44.186	0.082	0.082	0.836	112	0.395	14.74
T0762TS008_5-D1.rsa	42.857	0	0.351	0.649	85	0.504	56.3
T0762TS216_2-D1.rsa	42.188	0.066	0.213	0.721	98	0.43	15.99
T0762TS492_3-D1.rsa	41.667	0.091	0.191	0.718	79	0.527	51.14
T0762TS277_1-D1.rsa	41.667	0.045	0.391	0.564	62	0.672	61.82
T0762TS008_4-D1.rsa	41.096	0.639	0	0.361	92	0.447	49.22
T0762TS381_3-D1.rsa	41.071	0.159	0.111	0.73	92	0.446	21.82
T0762TS499_2-D1.rsa	40.625	0.088	0.331	0.581	79	0.514	61.03
T0762TS216_5-D1.rsa	40.625	0	0.287	0.713	97	0.419	13.97
T0762TS263_5-D1.rsa	39.655	0	0.231	0.769	110	0.361	78.15
T0762TS184_4-D1.rsa	39.583	0	0.427	0.573	63	0.628	46.36
T0762TS011_5-D1.rsa	39.535	0.306	0	0.694	93	0.425	42.16
T0762TS492_2-D1.rsa	39.535	0.179	0.09	0.731	98	0.403	25.56
T0762TS237_2-D1.rsa	39.063	0.015	0.419	0.566	77	0.507	70.77
T0762TS436_2-D1.rsa	37.5	0.036	0.436	0.527	58	0.647	72.95
T0762TS452_5-D1.rsa	37.5	0.055	0.282	0.664	73	0.514	51.82
T0762TS349_4-D1.rsa	36.047	0.32	0.172	0.509	86	0.419	69.82
T0762TS448_4-D1.rsa	36	0.029	0.109	0.862	119	0.303	14.86
T0762TS349_2-D1.rsa	35.965	0.281	0.203	0.516	112	0.321	66.13
T0762TS345_5-D1.rsa	35.938	0.015	0.456	0.529	72	0.499	71.14
T0762TS300_1-D1.rsa	35.938	0.015	0.412	0.574	78	0.461	58.82
T0762TS492_5-D1.rsa	35.616	0.757	0	0.243	62	0.574	60.49
T0762TS133_2-D1.rsa	35.616	0.769	0	0.231	59	0.604	52.74
T0762TS279_1-D1.rsa	35.345	0.641	0	0.359	92	0.384	57.32
T0762TS228_2-D1.rsa	35.227	0.071	0.497	0.432	128	0.275	33.7
T0762TS479_4-D1.rsa	35.211	0.452	0.101	0.446	75	0.469	52.24
T0762TS156_2-D1.rsa	35.211	0.435	0.024	0.542	91	0.387	47.44
T0762TS073_2-D1.rsa	35	0.018	0.281	0.702	80	0.438	43.2
T0762TS436_3-D1.rsa	34.884	0.187	0.067	0.746	100	0.349	24.44
T0762TS414_1-D1.rsa	34.247	0.737	0	0.263	67	0.511	65.98
T0762TS349_1-D1.rsa	34.211	0.24	0.198	0.562	122	0.28	58.06
T0762TS335_2-D1.rsa	33.929	0.095	0.23	0.675	85	0.399	35.71
T0762TS420_2-D1.rsa	33.803	0.494	0.107	0.399	67	0.505	50.64

T0762TS277_3-D1.rsa	33.684	0.521	0.055	0.424	100	0.337	63.77
T0762TS414_4-D1.rsa	33.333	0	0.364	0.636	70	0.476	65.45
T0762TS117_4-D1.rsa	32.813	0.103	0.353	0.544	74	0.443	61.21
T0762TS479_1-D1.rsa	32.759	0.664	0	0.336	86	0.381	56.54
T0762TS479_5-D1.rsa	32.558	0.299	0.037	0.664	89	0.366	25.75
T0762TS237_1-D1.rsa	32.468	0.406	0.121	0.473	106	0.306	79.64
T0762TS117_1-D1.rsa	32.394	0.256	0.024	0.72	121	0.268	23.24
T0762TS268_4-D1.rsa	32.143	0.175	0.31	0.516	65	0.495	54.76
T0762TS448_5-D1.rsa	31.579	0.072	0	0.928	103	0.307	31.53
T0762TS277_2-D1.rsa	31.579	0.241	0.5	0.259	28	1.128	93.98
T0762TS133_1-D1.rsa	31.579	0.231	0.491	0.278	30	1.053	92.36
T0762TS349_5-D1.rsa	30.476	0.346	0.188	0.466	109	0.28	70.94
T0762TS251_4-D1.rsa	30.263	0.253	0.021	0.726	241	0.126	9.11
T0762TS022_5-D1.rsa	29.07	0.308	0.195	0.497	84	0.346	72.19
T0762TS499_5-D1.rsa	29.07	0.355	0.213	0.432	73	0.398	69.97
T0762TS335_5-D1.rsa	28.947	0.117	0.018	0.865	96	0.302	34.01
T0762TS268_5-D1.rsa	28.947	0.126	0.018	0.856	95	0.305	24.32
T0762TS008_2-D1.rsa	28.947	0.241	0.472	0.287	31	0.934	93.75
T0762TS216_3-D1.rsa	28.846	0.224	0.055	0.721	119	0.242	15.51
T0762TS117_2-D1.rsa	28.571	0.29	0.088	0.622	232	0.123	63.33
T0762TS251_5-D1.rsa	28.205	0.361	0.128	0.51	151	0.187	54.51
T0762TS454_5-D1.rsa	28	0.018	0.383	0.599	197	0.142	52.51
T0762TS011_4-D1.rsa	28	0.014	0.254	0.732	101	0.277	40.4
T0762TS041_2-D1.rsa	28	0.08	0.362	0.558	77	0.364	66.3
T0762TS436_1-D1.rsa	28	0.072	0.355	0.572	79	0.354	54.89
T0762TS499_1-D1.rsa	28	0.065	0.348	0.587	81	0.346	60.87
T0762TS038_2-D1.rsa	28	0.029	0.109	0.862	119	0.235	13.04
T0762TS011_2-D1.rsa	27.907	0.349	0.213	0.438	74	0.377	70.12
T0762TS041_4-D1.rsa	27.907	0.349	0.16	0.491	83	0.336	69.23
T0762TS349_3-D1.rsa	27.619	0.38	0.214	0.406	95	0.291	71.9
T0762TS216_1-D1.rsa	26.786	0.175	0.167	0.659	83	0.323	20.44
T0762TS011_3-D1.rsa	26.744	0.343	0.136	0.521	88	0.304	73.08
T0762TS345_3-D1.rsa	26.744	0.373	0.16	0.467	79	0.339	70.56
T0762TS268_1-D1.rsa	26.316	0.241	0.481	0.278	30	0.877	94.21
T0762TS381_1-D1.rsa	25.862	0.691	0	0.309	79	0.327	68.36
T0762TS454_3-D1.rsa	25.714	0.428	0.05	0.522	211	0.122	46.29
T0762TS479_2-D1.rsa	25	0.44	0.103	0.457	111	0.225	63.78
T0762TS210_1-D1.rsa	25	0.254	0.016	0.73	92	0.272	42.86
T0762TS022_4-D1.rsa	24	0.058	0.341	0.601	83	0.289	70.47
T0762TS022_3-D1.rsa	24	0.094	0.362	0.543	75	0.32	68.48
T0762TS454_2-D1.rsa	23.684	0.399	0.029	0.572	261	0.091	60.97
T0762TS251_1-D1.rsa	23.077	0.368	0.108	0.524	155	0.149	59.2
T0762TS038_1-D1.rsa	20	0.072	0.384	0.543	75	0.267	61.05
T0762TS454_4-D1.rsa	18.421	0.235	0.123	0.642	213	0.086	50
T0762TS117_5-D1.rsa	15.385	0.271	0.229	0.5	229	0.067	72.11
T0762TS452_2-D1.rsa	12	0.029	0.297	0.674	93	0.129	50.73
T0762TS073_1-D1.rsa	12	0.051	0.283	0.667	92	0.13	49.09

T0772TS237_2-D1.rsa	83.562	0.149	0	0.851	217	0.385	8.63
T0772TS160_4-D1.rsa	77.143	0.399	0.015	0.587	237	0.325	6.13
T0772TS263_2-D1.rsa	55.263	0.301	0.018	0.681	226	0.245	13.18
T0772TS133_1-D1.rsa	54.545	0.051	0.311	0.639	189	0.289	44.17
T0772TS414_4-D1.rsa	54.545	0.105	0.287	0.608	180	0.303	23.65
T0772TS228_1-D1.rsa	53.846	0.267	0.085	0.648	107	0.503	68.67
T0772TS499_3-D1.rsa	52	0.431	0	0.569	230	0.226	10.71
T0772TS454_3-D1.rsa	45.455	0.024	0.277	0.699	207	0.22	28.12
T0772TS008_3-D1.rsa	40.909	0.03	0.28	0.689	204	0.201	40.12
T0772TS237_5-D1.rsa	36.364	0.054	0.311	0.635	188	0.193	48.48
T0772TS345_5-D1.rsa	36	0.396	0.017	0.587	237	0.152	15.1
T0772TS436_1-D1.rsa	35.227	0.027	0.331	0.642	190	0.185	25.76
T0772TS448_1-D1.rsa	35.2	0.012	0.35	0.638	210	0.168	50.15
T0772TS479_1-D1.rsa	34.653	0.199	0.109	0.692	222	0.156	39.02
T0772TS452_1-D1.rsa	34.091	0.03	0.389	0.581	172	0.198	42.4
T0772TS216_1-D1.rsa	34.014	0.378	0.094	0.528	197	0.173	26.18
T0772TS228_3-D1.rsa	33.824	0.313	0.099	0.588	143	0.237	64.78
T0772TS133_4-D1.rsa	33.663	0.287	0.15	0.564	181	0.186	71.65
T0772TS145_5-D1.rsa	33.143	0.027	0.381	0.592	239	0.139	6.06
T0772TS184_4-D1.rsa	32.323	0.229	0.042	0.729	210	0.154	50.35
T0772TS073_1-D1.rsa	32.203	0.009	0.28	0.71	152	0.212	65.54
T0772TS212_1-D1.rsa	32.192	0.356	0.105	0.539	200	0.161	72.75
T0772TS349_2-D1.rsa	32.192	0.399	0.102	0.499	185	0.174	80.86
T0772TS268_4-D1.rsa	32.192	0.367	0.119	0.515	191	0.169	62.81
T0772TS160_2-D1.rsa	32	0.015	0.371	0.614	202	0.158	47.26
T0772TS349_1-D1.rsa	32	0.015	0.407	0.578	190	0.168	49.7
T0772TS237_3-D1.rsa	31.507	0.345	0.1	0.555	206	0.153	63.35
T0772TS345_1-D1.rsa	31.507	0.407	0.121	0.472	175	0.18	81
T0772TS216_2-D1.rsa	31.429	0.478	0.005	0.517	209	0.15	37.62
T0772TS268_3-D1.rsa	31.313	0.201	0.142	0.656	189	0.166	53.65
T0772TS335_1-D1.rsa	30.693	0.274	0.137	0.589	189	0.162	74.53
T0772TS171_5-D1.rsa	30.693	0.274	0.14	0.586	188	0.163	74.45
T0772TS038_2-D1.rsa	30.693	0.308	0.153	0.539	173	0.177	75.39
T0772TS263_1-D1.rsa	30.4	0.015	0.325	0.66	217	0.14	48.4
T0772TS492_1-D1.rsa	30.303	0.222	0.135	0.642	185	0.164	62.5
T0772TS452_2-D1.rsa	30.137	0.402	0.108	0.491	182	0.166	79.02
T0772TS193_5-D1.rsa	29.703	0.202	0.115	0.682	219	0.136	48.13
T0772TS454_1-D1.rsa	29.703	0.268	0.146	0.586	188	0.158	75.62
T0772TS160_3-D1.rsa	29.703	0.271	0.15	0.579	186	0.16	75.62
T0772TS008_2-D1.rsa	29.703	0.315	0.153	0.533	171	0.174	75.39
T0772TS492_2-D1.rsa	29.412	0.239	0.103	0.658	160	0.184	60.44
T0772TS452_4-D1.rsa	29.293	0.226	0.167	0.608	175	0.167	61.46
T0772TS237_4-D1.rsa	29.293	0.229	0.208	0.563	162	0.181	70.14
T0772TS335_4-D1.rsa	29.252	0.386	0.107	0.507	189	0.155	44.44
T0772TS499_5-D1.rsa	28.8	0.012	0.255	0.733	241	0.12	25.68
T0772TS277_1-D1.rsa	28.8	0.012	0.374	0.614	202	0.143	52.81
T0772TS268_2-D1.rsa	28.8	0.018	0.413	0.568	187	0.154	57.14

T0772TS237_1-D1.rsa	28.767	0.394	0.102	0.504	187	0.154	73.84
T0772TS171_1-D1.rsa	28.767	0.388	0.119	0.493	183	0.157	76.16
T0772TS300_5-D1.rsa	28.767	0.418	0.105	0.477	177	0.163	75.41
T0772TS420_3-D1.rsa	28.713	0.19	0.109	0.701	225	0.128	39.56
T0772TS346_1-D1.rsa	28.713	0.277	0.153	0.57	183	0.157	74.69
T0772TS335_5-D1.rsa	28.283	0.201	0.17	0.628	181	0.156	70.92
T0772TS073_2-D1.rsa	28.283	0.198	0.142	0.66	190	0.149	55.12
T0772TS300_4-D1.rsa	28.283	0.236	0.177	0.587	169	0.167	66.32
T0772TS133_3-D1.rsa	28.082	0.407	0.108	0.485	180	0.156	82.36
T0772TS345_2-D1.rsa	28.082	0.407	0.108	0.485	180	0.156	81.68
T0772TS041_3-D1.rsa	28.082	0.396	0.1	0.504	187	0.15	75
T0772TS410_4-D1.rsa	28	0.015	0.334	0.65	214	0.131	51.44
T0772TS133_2-D1.rsa	28	0.009	0.374	0.617	203	0.138	47.8
T0772TS414_3-D1.rsa	28	0.48	0.022	0.498	201	0.139	28.46
T0772TS277_5-D1.rsa	27.891	0.349	0.105	0.547	204	0.137	81.88
T0772TS228_5-D1.rsa	27.891	0.378	0.097	0.525	196	0.142	74.79
T0772TS228_4-D1.rsa	27.869	0.014	0.348	0.638	134	0.208	71.64
T0772TS479_5-D1.rsa	27.723	0.215	0.156	0.629	202	0.137	74.84
T0772TS345_4-D1.rsa	27.723	0.265	0.14	0.595	191	0.145	74.53
T0772TS349_4-D1.rsa	26.531	0.389	0.099	0.512	191	0.139	82.01
T0772TS171_2-D1.rsa	26.4	0.006	0.304	0.69	227	0.116	45.52
T0772TS210_2-D1.rsa	26.4	0.046	0.28	0.675	222	0.119	32.22
T0772TS492_3-D1.rsa	26.286	0.45	0.03	0.52	210	0.125	42.82
T0772TS228_2-D1.rsa	26.286	0.478	0.025	0.498	201	0.131	39.48
T0772TS452_3-D1.rsa	26.263	0.25	0.194	0.556	160	0.164	70.31
T0772TS038_4-D1.rsa	26	0.41	0.048	0.542	136	0.191	87.95
T0772TS420_1-D1.rsa	25.85	0.386	0.105	0.509	190	0.136	87.22
T0772TS263_5-D1.rsa	25.85	0.397	0.102	0.501	187	0.138	84.58
T0772TS479_3-D1.rsa	25.85	0.383	0.099	0.517	193	0.134	83.33
T0772TS145_3-D1.rsa	25.743	0.296	0.131	0.573	184	0.14	77.8
T0772TS011_5-D1.rsa	25.743	0.28	0.153	0.567	182	0.141	77.49
T0772TS381_3-D1.rsa	25.743	0.318	0.153	0.53	170	0.151	74.38
T0772TS160_1-D1.rsa	25.424	0	0.294	0.706	151	0.168	63.55
T0772TS335_3-D1.rsa	25.342	0.388	0.086	0.526	195	0.13	64.92
T0772TS436_2-D1.rsa	25.17	0.394	0.11	0.496	185	0.136	80.9
T0772TS479_2-D1.rsa	25	0.247	0.099	0.654	217	0.115	52.26
T0772TS011_4-D1.rsa	24.8	0.015	0.353	0.632	208	0.119	45.97
T0772TS420_4-D1.rsa	24.571	0.441	0.025	0.535	216	0.114	34.28
T0772TS041_1-D1.rsa	24.49	0.375	0.102	0.523	195	0.126	85.69
T0772TS345_3-D1.rsa	24.49	0.386	0.118	0.496	185	0.132	71.6
T0772TS210_4-D1.rsa	24	0.416	0.012	0.572	231	0.104	40.47
T0772TS414_2-D1.rsa	24	0.021	0.377	0.602	198	0.121	52.66
T0772TS184_1-D1.rsa	24	0.47	0.012	0.517	209	0.115	39.11
T0772TS414_1-D1.rsa	24	0.483	0.025	0.493	199	0.121	41.46
T0772TS452_5-D1.rsa	24	0.525	0.02	0.455	184	0.13	49.81
T0772TS492_5-D1.rsa	24	0.515	0.012	0.473	191	0.126	44.49
T0772TS050_1-D1.rsa	24	0.515	0.005	0.48	194	0.124	44.24

T0772TS117_3-D1.rsa	23.762	0.293	0.081	0.626	201	0.118	63.47
T0772TS038_5-D1.rsa	23.729	0.033	0.215	0.752	161	0.147	60.28
T0772TS300_3-D1.rsa	23.684	0.211	0.078	0.711	236	0.1	45.48
T0772TS145_1-D1.rsa	23.256	0.406	0.113	0.481	136	0.171	82.42
T0772TS216_3-D1.rsa	22.772	0.287	0.143	0.57	183	0.124	76.09
T0772TS436_4-D1.rsa	22.368	0.447	0.015	0.537	245	0.091	64.86
T0772TS335_2-D1.rsa	22.286	0.515	0.01	0.475	192	0.116	44.74
T0772TS251_1-D1.rsa	22.222	0.421	0.09	0.489	223	0.1	76.49
T0772TS300_1-D1.rsa	22.034	0	0.21	0.79	169	0.13	60.51
T0772TS499_4-D1.rsa	21.053	0.428	0.02	0.553	252	0.084	65.24
T0772TS454_2-D1.rsa	21.053	0.41	0.024	0.566	258	0.082	57.18
T0772TS499_2-D1.rsa	20.988	0.417	0.081	0.502	229	0.092	74.28
T0772TS277_3-D1.rsa	20.988	0.423	0.101	0.476	217	0.097	70.75
T0772TS008_1-D1.rsa	20.571	0.421	0.027	0.552	223	0.092	42.57
T0772TS210_1-D1.rsa	20.571	0.441	0.03	0.53	214	0.096	42.88
T0772TS011_2-D1.rsa	20.571	0.525	0.01	0.465	188	0.109	51.98
T0772TS216_5-D1.rsa	20.571	0.525	0	0.475	192	0.107	44.49
T0772TS117_4-D1.rsa	20.395	0.487	0.02	0.493	225	0.091	46.93
T0772TS436_3-D1.rsa	20	0.428	0.02	0.552	223	0.09	41.4
T0772TS410_1-D1.rsa	19.753	0.423	0.103	0.474	216	0.091	73.73
T0772TS251_4-D1.rsa	19.737	0.421	0.022	0.557	254	0.078	58.11
T0772TS041_2-D1.rsa	19.737	0.425	0.024	0.55	251	0.079	60.2
T0772TS448_2-D1.rsa	19.737	0.157	0.081	0.762	253	0.078	50.98
T0772TS448_3-D1.rsa	19.737	0.307	0.024	0.669	222	0.089	63.18
T0772TS410_5-D1.rsa	19.737	0.328	0.117	0.554	184	0.107	94.58
T0772TS210_3-D1.rsa	19.079	0.397	0.022	0.581	265	0.072	50.16
T0772TS479_4-D1.rsa	19.079	0.487	0.004	0.509	232	0.082	63.49
T0772TS184_3-D1.rsa	18.605	0.382	0.049	0.569	161	0.116	77.39
T0772TS210_5-D1.rsa	18.421	0.465	0.018	0.518	236	0.078	53.73
T0772TS263_4-D1.rsa	17.105	0.238	0.114	0.648	215	0.08	60.24
T0772TS216_4-D1.rsa	17.105	0.313	0.12	0.566	188	0.091	72.21
T0772TS448_4-D1.rsa	16.923	0.266	0.218	0.515	236	0.072	56.06
T0772TS277_2-D1.rsa	16.447	0.454	0.024	0.522	238	0.069	63.16
T0772TS206_1-D1.rsa	16.279	0.378	0.12	0.502	142	0.115	81.09
T0772TS041_4-D1.rsa	16.154	0.242	0.203	0.555	254	0.064	71.83
T0772TS277_4-D1.rsa	16.154	0.262	0.225	0.513	235	0.069	74.06
T0772TS251_2-D1.rsa	16.154	0.264	0.229	0.507	232	0.07	75.56
T0772TS454_5-D1.rsa	15.789	0.289	0.111	0.599	199	0.079	97.36
T0772TS008_4-D1.rsa	15.789	0.334	0.151	0.515	171	0.092	99.4
T0772TS008_5-D1.rsa	15.789	0.346	0.117	0.536	178	0.089	98.95
T0772TS117_1-D1.rsa	15.789	0.313	0.148	0.539	179	0.088	98.8
T0772TS499_1-D1.rsa	14.615	0.279	0.203	0.517	237	0.062	67.72
T0772TS133_5-D1.rsa	14.474	0.307	0.148	0.545	181	0.08	96.23
T0772TS349_5-D1.rsa	14.474	0.34	0.157	0.503	167	0.087	99.32
T0772TS038_1-D1.rsa	14.474	0.352	0.123	0.524	174	0.083	97.89
T0772TS160_5-D1.rsa	14.474	0.349	0.123	0.527	175	0.083	99.4
T0772TS038_3-D1.rsa	14.474	0.322	0.123	0.554	184	0.079	93.67

T0772TS410_2-D1.rsa	13.158	0.283	0.084	0.633	210	0.063	96.46
T0772TS410_3-D1.rsa	13.158	0.307	0.114	0.578	192	0.069	98.49
T0772TS420_2-D1.rsa	13.158	0.346	0.123	0.53	176	0.075	99.1
T0772TS268_1-D1.rsa	13.158	0.334	0.151	0.515	171	0.077	99.4
T0772TS436_5-D1.rsa	13.077	0.253	0.225	0.522	239	0.055	77.39
T0772TS492_4-D1.rsa	12.766	0.331	0.183	0.486	125	0.102	82.2
T0772TS171_3-D1.rsa	11.842	0.331	0.148	0.521	173	0.068	98.8
T0839TS381_5-D1.rsa	71.053	0.468	0	0.532	59	1.204	21.85
T0839TS448_2-D1.rsa	67.647	0.611	0	0.389	49	1.381	33.73
T0839TS436_2-D1.rsa	67.647	0.611	0	0.389	49	1.381	23.81
T0839TS300_5-D1.rsa	64	0.319	0.181	0.5	69	0.928	12.5
T0839TS038_1-D1.rsa	62.5	0.018	0.474	0.509	58	1.078	17.32
T0839TS277_4-D1.rsa	61.972	0.536	0	0.464	78	0.795	15.22
T0839TS499_5-D1.rsa	60.526	0.342	0.036	0.622	69	0.877	20.5
T0839TS499_2-D1.rsa	60.526	0	0.342	0.658	73	0.829	14.64
T0839TS381_2-D1.rsa	58.824	0.635	0	0.365	46	1.279	37.7
T0839TS041_4-D1.rsa	57.895	0.189	0.072	0.739	82	0.706	34.69
T0839TS160_5-D1.rsa	56.338	0.476	0	0.524	88	0.64	12.98
T0839TS277_5-D1.rsa	55	0.368	0	0.632	72	0.764	14.25
T0839TS479_5-D1.rsa	54.286	0.56	0.064	0.376	88	0.617	10.26
T0839TS171_1-D1.rsa	52.632	0.36	0.108	0.532	59	0.892	36.94
T0839TS008_1-D1.rsa	52.632	0.234	0.099	0.667	74	0.711	16.89
T0839TS210_1-D1.rsa	51.786	0.349	0.04	0.611	77	0.673	16.67
T0839TS452_3-D1.rsa	50	0.059	0.309	0.632	86	0.581	12.87
T0839TS251_1-D1.rsa	50	0.651	0	0.349	44	1.136	37.3
T0839TS420_1-D1.rsa	50	0	0.511	0.489	64	0.781	14.12
T0839TS335_4-D1.rsa	48.837	0.321	0.187	0.493	66	0.74	25.56
T0839TS479_1-D1.rsa	48.214	0.135	0.381	0.484	61	0.79	42.06
T0839TS228_3-D1.rsa	48.214	0.341	0.079	0.579	73	0.66	25
T0839TS160_2-D1.rsa	47.5	0.026	0.298	0.675	77	0.617	18.42
T0839TS448_5-D1.rsa	47.368	0.117	0.198	0.685	76	0.623	16.44
T0839TS011_5-D1.rsa	47.059	0.516	0	0.484	61	0.771	32.94
T0839TS160_1-D1.rsa	46.479	0.464	0.125	0.411	69	0.674	29.49
T0839TS145_1-D1.rsa	46.479	0.506	0.119	0.375	63	0.738	34.3
T0839TS346_1-D1.rsa	46.429	0.373	0.016	0.611	77	0.603	18.65
T0839TS436_5-D1.rsa	45.833	0	0.676	0.324	35	1.31	66.67
T0839TS263_5-D1.rsa	45.833	0	0.545	0.455	50	0.917	41.82
T0839TS436_1-D1.rsa	45.07	0.512	0.113	0.375	63	0.715	33.81
T0839TS349_5-D1.rsa	45.07	0.512	0.071	0.417	70	0.644	21.31
T0839TS349_3-D1.rsa	43.75	0.096	0.301	0.603	82	0.534	15.99
T0839TS279_1-D1.rsa	42.857	0.015	0.359	0.626	82	0.523	17.94
T0839TS420_4-D1.rsa	42.857	0.27	0.095	0.635	80	0.536	22.62
T0839TS452_1-D1.rsa	42.857	0.031	0.313	0.656	86	0.498	16.6
T0839TS117_3-D1.rsa	42.857	0.325	0.063	0.611	77	0.557	25.59
T0839TS145_5-D1.rsa	42.857	0.183	0.31	0.508	64	0.67	42.66
T0839TS110_1-D1.rsa	42.5	0	0.368	0.632	72	0.59	26.54
T0839TS110_2-D1.rsa	42.5	0	0.412	0.588	67	0.634	26.1

T0839TS414_4-D1.rsa	42.5	0	0.281	0.719	82	0.518	18.42
T0839TS454_3-D1.rsa	42.5	0	0.43	0.57	65	0.654	21.27
T0839TS492_3-D1.rsa	42.5	0	0.456	0.544	62	0.685	28.51
T0839TS268_5-D1.rsa	42.5	0.018	0.526	0.456	52	0.817	21.93
T0839TS268_2-D1.rsa	42.5	0.035	0.43	0.535	61	0.697	27.19
T0839TS008_5-D1.rsa	42.5	0.035	0.518	0.447	51	0.833	23.9
T0839TS133_4-D1.rsa	42.188	0.081	0.287	0.632	86	0.491	11.95
T0839TS420_5-D1.rsa	42.105	0.171	0	0.829	92	0.458	18.69
T0839TS237_5-D1.rsa	42.105	0.243	0.108	0.649	72	0.585	37.39
T0839TS436_4-D1.rsa	41.86	0.313	0.075	0.612	82	0.51	25.75
T0839TS448_3-D1.rsa	41.071	0.214	0.135	0.651	82	0.501	17.66
T0839TS410_1-D1.rsa	41.071	0.317	0.079	0.603	76	0.54	17.06
T0839TS228_4-D1.rsa	40.845	0.393	0.149	0.458	77	0.53	40.87
T0839TS268_1-D1.rsa	40.625	0	0.368	0.632	86	0.472	13.05
T0839TS237_3-D1.rsa	40	0.026	0.254	0.719	82	0.488	26.32
T0839TS263_4-D1.rsa	39.583	0.109	0.427	0.464	51	0.776	53.86
T0839TS335_2-D1.rsa	39.535	0.261	0.179	0.56	75	0.527	23.32
T0839TS277_1-D1.rsa	39.535	0.373	0.127	0.5	67	0.59	28.73
T0839TS171_2-D1.rsa	39.474	0.243	0.018	0.739	82	0.481	32.66
T0839TS263_1-D1.rsa	39.437	0.417	0.167	0.417	70	0.563	22.28
T0839TS492_2-D1.rsa	39.437	0.5	0.089	0.411	69	0.572	28.53
T0839TS237_4-D1.rsa	39.286	0.302	0.119	0.579	73	0.538	32.14
T0839TS041_5-D1.rsa	39.286	0.183	0.373	0.444	56	0.702	44.25
T0839TS436_3-D1.rsa	39.063	0.066	0.279	0.654	89	0.439	13.05
T0839TS210_5-D1.rsa	39.063	0.103	0.294	0.603	82	0.476	15.44
T0839TS499_3-D1.rsa	38.028	0.399	0.119	0.482	81	0.469	41.03
T0839TS117_4-D1.rsa	37.5	0	0.412	0.588	67	0.56	32.02
T0839TS410_2-D1.rsa	37.5	0.19	0.325	0.484	61	0.615	43.06
T0839TS117_1-D1.rsa	37.5	0	0.412	0.588	67	0.56	31.8
T0839TS038_4-D1.rsa	37.5	0.175	0.341	0.484	61	0.615	41.47
T0839TS011_1-D1.rsa	37.5	0	0.439	0.561	64	0.586	26.1
T0839TS263_2-D1.rsa	36.986	0.757	0	0.243	62	0.597	43.92
T0839TS212_1-D1.rsa	36.842	0.213	0.25	0.537	58	0.635	63.66
T0839TS216_4-D1.rsa	36.842	0.259	0.306	0.435	47	0.784	69.91
T0839TS300_1-D1.rsa	36.842	0.288	0.072	0.64	71	0.519	25.45
T0839TS499_1-D1.rsa	36.842	0.25	0.509	0.241	26	1.417	85.65
T0839TS381_1-D1.rsa	35.938	0.074	0.279	0.647	88	0.408	14.71
T0839TS184_4-D1.rsa	35.938	0.081	0.309	0.61	83	0.433	12.87
T0839TS184_5-D1.rsa	35.938	0.088	0.368	0.544	74	0.486	13.79
T0839TS251_5-D1.rsa	35.714	0.151	0.238	0.611	77	0.464	21.03
T0839TS216_2-D1.rsa	35.714	0	0.344	0.656	86	0.415	16.6
T0839TS216_5-D1.rsa	35.714	0.053	0.344	0.603	79	0.452	16.41
T0839TS492_4-D1.rsa	35.714	0.23	0.206	0.563	71	0.503	29.56
T0839TS300_4-D1.rsa	35.714	0	0.344	0.656	86	0.415	16.79
T0839TS008_3-D1.rsa	35.714	0.19	0.421	0.389	49	0.729	52.38
T0839TS263_3-D1.rsa	35	0.026	0.202	0.772	88	0.398	17.76
T0839TS050_1-D1.rsa	35	0	0.342	0.658	75	0.467	19.96

T0839TS492_1-D1.rsa	35	0	0.412	0.588	67	0.522	31.8
T0839TS008_2-D1.rsa	35	0	0.368	0.632	72	0.486	23.68
T0839TS008_4-D1.rsa	35	0	0.421	0.579	66	0.53	17.11
T0839TS420_3-D1.rsa	34.884	0.224	0.134	0.642	86	0.406	23.69
T0839TS184_3-D1.rsa	34.247	0.765	0	0.235	60	0.571	39.12
T0839TS184_2-D1.rsa	34.247	0.753	0	0.247	63	0.544	42.94
T0839TS381_4-D1.rsa	34.211	0.27	0.117	0.613	68	0.503	35.81
T0839TS349_4-D1.rsa	34.211	0.27	0.09	0.64	71	0.482	35.13
T0839TS448_1-D1.rsa	33.929	0.167	0.254	0.579	73	0.465	42.06
T0839TS410_5-D1.rsa	33.929	0.19	0.373	0.437	55	0.617	52.58
T0839TS479_4-D1.rsa	33.803	0.518	0.077	0.405	68	0.497	49.84
T0839TS145_4-D1.rsa	33.803	0.429	0.167	0.405	68	0.497	50.16
T0839TS300_3-D1.rsa	33.803	0.47	0.137	0.393	66	0.512	38.94
T0839TS300_2-D1.rsa	33.333	0.055	0.282	0.664	73	0.457	33.41
T0839TS110_4-D1.rsa	32.877	0.698	0	0.302	77	0.427	37.74
T0839TS414_5-D1.rsa	32.558	0.321	0	0.679	91	0.358	23.32
T0839TS011_4-D1.rsa	32.5	0.026	0.289	0.684	78	0.417	28.29
T0839TS041_1-D1.rsa	32.5	0.018	0.298	0.684	78	0.417	26.97
T0839TS038_2-D1.rsa	32.5	0.018	0.395	0.588	67	0.485	31.58
T0839TS345_1-D1.rsa	32.5	0	0.439	0.561	64	0.508	25.66
T0839TS454_2-D1.rsa	32.5	0.342	0.018	0.64	73	0.445	19.52
T0839TS038_5-D1.rsa	32.143	0.175	0.341	0.484	61	0.527	53.57
T0839TS381_3-D1.rsa	32.143	0.175	0.357	0.468	59	0.545	53.37
T0839TS479_2-D1.rsa	32.143	0.159	0.325	0.516	65	0.495	42.86
T0839TS237_2-D1.rsa	31.579	0.259	0.352	0.389	42	0.752	81.25
T0839TS414_2-D1.rsa	31.579	0.241	0.5	0.259	28	1.128	94.21
T0839TS160_4-D1.rsa	31.507	0.753	0	0.247	63	0.5	40.69
T0839TS117_5-D1.rsa	31.429	0.342	0.094	0.564	132	0.238	65.49
T0839TS452_4-D1.rsa	30.357	0.198	0.19	0.611	77	0.394	47.02
T0839TS410_3-D1.rsa	30.357	0.159	0.159	0.683	86	0.353	26.98
T0839TS410_4-D1.rsa	30.357	0.167	0.381	0.452	57	0.533	50.59
T0839TS499_4-D1.rsa	30.357	0.175	0.429	0.397	50	0.607	54.56
T0839TS228_2-D1.rsa	30.137	0.765	0	0.235	60	0.502	54.8
T0839TS448_4-D1.rsa	30.137	0.765	0	0.235	60	0.502	53.14
T0839TS452_2-D1.rsa	30.137	0.765	0	0.235	60	0.502	47.26
T0839TS251_2-D1.rsa	30.137	0.765	0	0.235	60	0.502	46.96
T0839TS420_2-D1.rsa	30	0	0.105	0.895	102	0.294	15.79
T0839TS251_4-D1.rsa	30	0	0.456	0.544	62	0.484	46.05
T0839TS277_2-D1.rsa	30	0.018	0.219	0.763	87	0.345	24.78
T0839TS335_3-D1.rsa	29.688	0.044	0.221	0.735	100	0.297	16.73
T0839TS345_5-D1.rsa	29.412	0.603	0	0.397	50	0.588	57.34
T0839TS133_1-D1.rsa	28.947	0.241	0.5	0.259	28	1.034	93.75
T0839TS206_1-D1.rsa	28.767	0.407	0.092	0.501	186	0.155	78
T0839TS452_5-D1.rsa	28.571	0.127	0.333	0.54	68	0.42	49.41
T0839TS133_5-D1.rsa	28.571	0.183	0.302	0.516	65	0.44	50.79
T0839TS171_5-D1.rsa	28.571	0.159	0.349	0.492	62	0.461	47.42
T0839TS251_3-D1.rsa	28.571	0.151	0.333	0.516	65	0.44	51.98

T0839TS228_1-D1.rsa	28.571	0.183	0.349	0.468	59	0.484	56.15
T0839TS038_3-D1.rsa	28.571	0.175	0.357	0.468	59	0.484	50.99
T0839TS210_3-D1.rsa	28.571	0.167	0.389	0.444	56	0.51	52.18
T0839TS345_2-D1.rsa	28.571	0.175	0.421	0.405	51	0.56	53.97
T0839TS041_3-D1.rsa	28.169	0.458	0.161	0.381	64	0.44	62.34
T0839TS133_3-D1.rsa	28	0.022	0.261	0.717	99	0.283	28.44
T0839TS479_3-D1.rsa	27.907	0.358	0.015	0.627	84	0.332	31.72
T0839TS011_3-D1.rsa	27.586	0.75	0	0.25	64	0.431	66.31
T0839TS041_2-D1.rsa	27.5	0	0.447	0.553	63	0.437	41.67
T0839TS145_3-D1.rsa	27.5	0	0.43	0.57	65	0.423	43.64
T0839TS210_4-D1.rsa	27.397	0.753	0	0.247	63	0.435	46.67
T0839TS345_3-D1.rsa	26.786	0.159	0.357	0.484	61	0.439	48.21
T0839TS133_2-D1.rsa	26.761	0.339	0.024	0.637	107	0.25	16.35
T0839TS216_1-D1.rsa	26.563	0.096	0.287	0.618	84	0.316	15.99
T0839TS345_4-D1.rsa	25.581	0.336	0	0.664	89	0.287	24.44
T0839TS349_2-D1.rsa	25	0.096	0.272	0.632	86	0.291	16.36
T0839TS145_2-D1.rsa	25	0.018	0.333	0.649	74	0.338	26.97
T0839TS492_5-D1.rsa	25	0.132	0.07	0.798	91	0.275	19.08
T0839TS268_4-D1.rsa	25	0	0.298	0.702	80	0.313	28.95
T0839TS268_3-D1.rsa	25	0.11	0.309	0.581	79	0.316	16.36
T0839TS184_1-D1.rsa	25	0.096	0.287	0.618	84	0.298	16.36
T0839TS454_1-D1.rsa	25	0	0.219	0.781	89	0.281	19.52
T0839TS117_2-D1.rsa	16.923	0.275	0.253	0.472	216	0.078	70.11
T0839TS171_4-D1.rsa	15	0	0.167	0.833	95	0.158	40.57

TARGETS: T0885-D1,T0833-D1,T0860-D1,T0845-D2,T0835-D1,T0794-D1,T0811-D1,T0819-D1, T0768-D1,T0803-D1,T0782-D1,T0889-D1,T0770-D1,T0810-D2,T0786-D1

TEST SET

Name	SAAP	HelixFraction	SheetFraction	LoopFraction	Loop	SAAP/Loop	GDT
T0885TS455_5-D1.rsa	81.633	0.219	0	0.781	89	0.917	14.91
T0885TS455_2-D1.rsa	71.429	0.105	0	0.895	102	0.7	14.25
T0885TS464_2-D1.rsa	71.429	0.491	0	0.509	58	1.232	19.52
T0885TS455_1-D1.rsa	65.306	0.404	0	0.596	68	0.96	23.25
T0885TS464_5-D1.rsa	61.224	0.526	0	0.474	54	1.134	29.39
T0885TS446_5-D1.rsa	59.184	0.789	0	0.211	24	2.466	23.9
T0885TS455_3-D1.rsa	55.102	0.254	0	0.746	85	0.648	14.69
T0885TS451_3-D1.rsa	53.061	0.658	0	0.342	39	1.361	57.67
T0885TS284_1-D1.rsa	53.061	0.482	0	0.518	59	0.899	46.71
T0885TS284_3-D1.rsa	53.061	0.491	0	0.509	58	0.915	51.75
T0885TS236_5-D1.rsa	53.061	0.623	0	0.377	43	1.234	60.97
T0885TS455_4-D1.rsa	53.061	0.228	0	0.772	88	0.603	22.37
T0885TS446_1-D1.rsa	51.02	0.868	0	0.132	15	3.401	25.88
T0885TS275_2-D1.rsa	51.02	0.553	0	0.447	51	1	58.33
T0885TS452_5-D1.rsa	51.02	0.632	0	0.368	42	1.215	32.46
T0885TS451_1-D1.rsa	51.02	0.667	0	0.333	38	1.343	52.63
T0885TS446_2-D1.rsa	51.02	0.877	0	0.123	14	3.644	27.19

T0885TS446_3-D1.rsa	48.98	0.798	0	0.202	23	2.13	28.07
T0885TS180_4-D1.rsa	48.98	0.675	0	0.325	37	1.324	49.12
T0885TS321_5-D1.rsa	48.98	0.658	0	0.342	39	1.256	24.12
T0885TS451_4-D1.rsa	48.98	0.667	0	0.333	38	1.289	57.02
T0885TS275_4-D1.rsa	48.98	0.518	0	0.482	55	0.891	57.67
T0885TS451_2-D1.rsa	46.939	0.667	0	0.333	38	1.235	57.46
T0885TS345_4-D1.rsa	46.939	0.711	0	0.289	33	1.422	58.77
T0885TS321_3-D1.rsa	46.939	0.675	0	0.325	37	1.269	26.32
T0885TS345_2-D1.rsa	46.939	0.711	0	0.289	33	1.422	35.31
T0885TS407_2-D1.rsa	46.939	0.553	0	0.447	51	0.92	45.4
T0885TS275_3-D1.rsa	46.939	0.526	0	0.474	54	0.869	59.43
T0885TS359_3-D1.rsa	46.939	0.649	0	0.351	40	1.173	34.21
T0885TS284_2-D1.rsa	44.898	0.605	0	0.395	45	0.998	54.6
T0885TS275_5-D1.rsa	44.898	0.623	0	0.377	43	1.044	50.66
T0885TS183_4-D1.rsa	44.898	0.772	0	0.228	26	1.727	55.92
T0885TS434_5-D1.rsa	44.898	0.588	0	0.412	47	0.955	29.82
T0885TS251_5-D1.rsa	44.898	0.64	0	0.36	41	1.095	56.58
T0885TS451_5-D1.rsa	44.898	0.64	0	0.36	41	1.095	59.43
T0885TS321_1-D1.rsa	44.898	0.675	0	0.325	37	1.213	32.02
T0885TS464_3-D1.rsa	44.898	0.5	0	0.5	57	0.788	49.34
T0885TS464_4-D1.rsa	44.898	0.518	0	0.482	55	0.816	50.22
T0885TS452_2-D1.rsa	44.898	0.596	0	0.404	46	0.976	38.82
T0885TS407_3-D1.rsa	44.898	0.605	0	0.395	45	0.998	44.08
T0885TS407_4-D1.rsa	44.898	0.57	0	0.43	49	0.916	45.17
T0885TS464_1-D1.rsa	44.898	0.518	0	0.482	55	0.816	50
T0885TS275_1-D1.rsa	44.898	0.526	0	0.474	54	0.831	59.43
T0885TS407_1-D1.rsa	44.898	0.623	0	0.377	43	1.044	42.76
T0885TS026_1-D1.rsa	44.898	0.614	0	0.386	44	1.02	55.92
T0885TS321_4-D1.rsa	42.857	0.675	0	0.325	37	1.158	39.69
T0885TS287_2-D1.rsa	42.857	0.693	0	0.307	35	1.224	57.02
T0885TS434_2-D1.rsa	42.857	0.57	0	0.43	49	0.875	32.67
T0885TS016_1-D1.rsa	42.857	0.588	0	0.412	47	0.912	50.44
T0885TS236_4-D1.rsa	42.857	0.719	0	0.281	32	1.339	54.39
T0885TS425_2-D1.rsa	42.857	0.518	0	0.482	55	0.779	58.11
T0885TS251_2-D1.rsa	42.857	0.579	0	0.421	48	0.893	67.11
T0885TS407_5-D1.rsa	42.857	0.614	0	0.386	44	0.974	44.3
T0885TS425_5-D1.rsa	42.857	0.544	0	0.456	52	0.824	57.9
T0885TS077_3-D1.rsa	42.857	0.544	0	0.456	52	0.824	58.99
T0885TS357_3-D1.rsa	42.857	0.509	0	0.491	56	0.765	50.22
T0885TS236_1-D1.rsa	40.816	0.711	0	0.289	33	1.237	58.33
T0885TS321_2-D1.rsa	40.816	0.675	0	0.325	37	1.103	26.75
T0885TS467_2-D1.rsa	40.816	0.632	0	0.368	42	0.972	55.04
T0885TS287_3-D1.rsa	40.816	0.535	0	0.465	53	0.77	59.21
T0885TS405_2-D1.rsa	40.816	0.711	0	0.289	33	1.237	69.96
T0885TS452_1-D1.rsa	40.816	0.667	0	0.333	38	1.074	52.63
T0885TS357_2-D1.rsa	40.816	0.526	0	0.474	54	0.756	53.07
T0885TS251_4-D1.rsa	40.816	0.667	0	0.333	38	1.074	57.9

T0885TS467_3-D1.rsa	40.816	0.711	0	0.289	33	1.237	57.9
T0885TS444_4-D1.rsa	40.816	0.789	0	0.211	24	1.701	60.97
T0885TS444_5-D1.rsa	40.816	0.579	0	0.421	48	0.85	67.54
T0885TS434_4-D1.rsa	40.816	0.614	0	0.386	44	0.928	29.82
T0885TS405_3-D1.rsa	40.816	0.632	0	0.368	42	0.972	70.83
T0885TS180_2-D1.rsa	40.816	0.702	0	0.298	34	1.2	46.27
T0885TS026_5-D1.rsa	40.816	0.632	0	0.368	42	0.972	55.26
T0885TS077_5-D1.rsa	40.816	0.535	0	0.465	53	0.77	57.24
T0885TS382_5-D1.rsa	40.816	0.588	0	0.412	47	0.868	64.25
T0885TS434_1-D1.rsa	40.816	0.596	0	0.404	46	0.887	38.82
T0885TS026_2-D1.rsa	40.816	0.605	0	0.395	45	0.907	51.75
T0885TS432_5-D1.rsa	40.816	0.702	0	0.298	34	1.2	56.14
T0885TS357_1-D1.rsa	40.816	0.509	0	0.491	56	0.729	56.8
T0885TS479_3-D1.rsa	38.776	0.667	0	0.333	38	1.02	62.72
T0885TS405_4-D1.rsa	38.776	0.64	0	0.36	41	0.946	65.13
T0885TS180_3-D1.rsa	38.776	0.684	0	0.316	36	1.077	45.83
T0885TS284_5-D1.rsa	38.776	0.491	0	0.509	58	0.669	51.75
T0885TS287_1-D1.rsa	38.776	0.702	0	0.298	34	1.14	59.21
T0885TS287_5-D1.rsa	38.776	0.561	0	0.439	50	0.776	59.43
T0885TS405_5-D1.rsa	38.776	0.596	0	0.404	46	0.843	61.84
T0885TS444_2-D1.rsa	38.776	0.667	0	0.333	38	1.02	67.33
T0885TS345_5-D1.rsa	38.776	0.588	0	0.412	47	0.825	72.81
T0885TS434_3-D1.rsa	38.776	0.596	0	0.404	46	0.843	30.26
T0885TS444_1-D1.rsa	38.776	0.702	0	0.298	34	1.14	48.47
T0885TS467_4-D1.rsa	38.776	0.693	0	0.307	35	1.108	41.89
T0885TS425_3-D1.rsa	38.776	0.518	0	0.482	55	0.705	58.77
T0885TS345_3-D1.rsa	38.776	0.64	0	0.36	41	0.946	33.77
T0885TS444_3-D1.rsa	38.776	0.684	0	0.316	36	1.077	42.98
T0885TS250_1-D1.rsa	38.776	0.579	0	0.421	48	0.808	59.87
T0885TS313_1-D1.rsa	38.776	0.605	0	0.395	45	0.862	66.23
T0885TS467_5-D1.rsa	38.776	0.579	0	0.421	48	0.808	53.51
T0885TS183_5-D1.rsa	36.735	0.588	0.035	0.377	43	0.854	56.14
T0885TS287_4-D1.rsa	36.735	0.579	0	0.421	48	0.765	59.65
T0885TS180_1-D1.rsa	36.735	0.711	0	0.289	33	1.113	45.17
T0885TS251_1-D1.rsa	36.735	0.57	0	0.43	49	0.75	64.91
T0885TS077_4-D1.rsa	36.735	0.518	0	0.482	55	0.668	56.14
T0885TS432_3-D1.rsa	36.735	0.684	0	0.316	36	1.02	65.79
T0885TS220_4-D1.rsa	36.735	0.711	0	0.289	33	1.113	66.23
T0885TS026_3-D1.rsa	36.735	0.57	0	0.43	49	0.75	53.07
T0885TS452_4-D1.rsa	36.735	0.57	0	0.43	49	0.75	53.95
T0885TS313_4-D1.rsa	36.735	0.605	0	0.395	45	0.816	66.23
T0885TS220_3-D1.rsa	36.735	0.702	0	0.298	34	1.08	67.11
T0885TS425_1-D1.rsa	36.735	0.544	0	0.456	52	0.706	57.02
T0885TS077_1-D1.rsa	36.735	0.579	0	0.421	48	0.765	58.11
T0885TS382_1-D1.rsa	36.735	0.605	0	0.395	45	0.816	64.47
T0885TS357_4-D1.rsa	36.735	0.518	0	0.482	55	0.668	50.44
T0885TS026_4-D1.rsa	36.735	0.588	0	0.412	47	0.782	65.57

T0885TS405_1-D1.rsa	34.694	0.658	0	0.342	39	0.89	72.59
T0885TS258_3-D1.rsa	34.694	0.623	0	0.377	43	0.807	51.97
T0885TS359_1-D1.rsa	34.694	0.719	0	0.281	32	1.084	37.06
T0885TS183_3-D1.rsa	34.694	0.596	0	0.404	46	0.754	48.9
T0885TS345_1-D1.rsa	34.694	0.781	0	0.219	25	1.388	68.42
T0885TS077_2-D1.rsa	34.694	0.535	0	0.465	53	0.655	57.02
T0885TS382_3-D1.rsa	34.694	0.632	0	0.368	42	0.826	64.03
T0885TS250_5-D1.rsa	34.694	0.561	0	0.439	50	0.694	59.21
T0885TS251_3-D1.rsa	34.694	0.579	0	0.421	48	0.723	59.43
T0885TS236_2-D1.rsa	34.694	0.518	0	0.482	55	0.631	60.31
T0885TS258_2-D1.rsa	34.694	0.561	0	0.439	50	0.694	64.03
T0885TS432_1-D1.rsa	34.694	0.684	0	0.316	36	0.964	64.03
T0885TS349_1-D1.rsa	34.694	0.605	0	0.395	45	0.771	65.57
T0885TS183_1-D1.rsa	34.694	0.746	0	0.254	29	1.196	70.17
T0885TS382_4-D1.rsa	34.694	0.596	0	0.404	46	0.754	64.03
T0885TS467_1-D1.rsa	34.694	0.649	0	0.351	40	0.867	27.63
T0885TS250_3-D1.rsa	34.694	0.57	0	0.43	49	0.708	58.99
T0885TS430_1-D1.rsa	34.694	0.684	0	0.316	36	0.964	69.96
T0885TS430_2-D1.rsa	34.694	0.658	0	0.342	39	0.89	56.36
T0885TS250_2-D1.rsa	34.694	0.57	0	0.43	49	0.708	59.21
T0885TS005_1-D1.rsa	34.694	0.693	0	0.307	35	0.991	76.97
T0885TS479_5-D1.rsa	32.653	0.614	0.053	0.333	38	0.859	56.8
T0885TS005_5-D1.rsa	32.653	0.763	0	0.237	27	1.209	83.77
T0885TS258_5-D1.rsa	32.653	0.623	0	0.377	43	0.759	50.66
T0885TS236_3-D1.rsa	32.653	0.518	0	0.482	55	0.594	58.33
T0885TS183_2-D1.rsa	32.653	0.693	0	0.307	35	0.933	61.4
T0885TS425_4-D1.rsa	32.653	0.57	0	0.43	49	0.666	58.77
T0885TS005_4-D1.rsa	32.653	0.728	0	0.272	31	1.053	83.77
T0885TS220_2-D1.rsa	32.653	0.667	0	0.333	38	0.859	55.92
T0885TS430_5-D1.rsa	32.653	0.702	0	0.298	34	0.96	57.67
T0885TS452_3-D1.rsa	32.653	0.614	0	0.386	44	0.742	26.75
T0885TS180_5-D1.rsa	32.653	0.667	0	0.333	38	0.859	32.46
T0885TS432_4-D1.rsa	32.653	0.702	0	0.298	34	0.96	59.87
T0885TS430_4-D1.rsa	32.653	0.693	0	0.307	35	0.933	66.67
T0885TS250_4-D1.rsa	32.653	0.579	0	0.421	48	0.68	58.99
T0885TS479_2-D1.rsa	30.612	0.754	0	0.246	28	1.093	69.3
T0885TS258_4-D1.rsa	30.612	0.605	0	0.395	45	0.68	63.6
T0885TS430_3-D1.rsa	30.612	0.675	0	0.325	37	0.827	68.42
T0885TS048_1-D1.rsa	30.612	0.667	0	0.333	38	0.806	69.74
T0885TS220_1-D1.rsa	30.612	0.693	0	0.307	35	0.875	58.33
T0885TS382_2-D1.rsa	30.612	0.605	0	0.395	45	0.68	62.94
T0885TS220_5-D1.rsa	28.571	0.693	0	0.307	35	0.816	67.76
T0885TS479_4-D1.rsa	28.571	0.693	0	0.307	35	0.816	50
T0885TS258_1-D1.rsa	28.571	0.614	0	0.386	44	0.649	61.18
T0885TS479_1-D1.rsa	28.571	0.711	0	0.289	33	0.866	68.2
T0885TS005_3-D1.rsa	28.571	0.728	0	0.272	31	0.922	84.65
T0885TS313_2-D1.rsa	28.571	0.605	0	0.395	45	0.635	65.79

T0885TS005_2-D1.rsa	26.531	0.667	0	0.333	38	0.698	87.94
T0833TS041_4-D1.rsa	80.357	0.103	0.032	0.865	109	0.737	17.66
T0833TS133_1-D1.rsa	76.471	0.429	0	0.571	72	1.062	19.05
T0833TS349_5-D1.rsa	73.077	0.624	0	0.376	62	1.179	18.2
T0833TS448_4-D1.rsa	73.077	0.618	0	0.382	63	1.16	14.24
T0833TS452_2-D1.rsa	69.767	0.433	0.03	0.537	72	0.969	17.16
T0833TS022_4-D1.rsa	67.188	0.324	0.103	0.574	78	0.861	11.4
T0833TS277_1-D1.rsa	66.667	0.019	0.657	0.324	35	1.905	68.98
T0833TS492_2-D1.rsa	66.667	0.019	0.574	0.407	44	1.515	72.45
T0833TS237_5-D1.rsa	66.667	0	0.556	0.444	48	1.389	63.66
T0833TS410_3-D1.rsa	64.789	0.196	0.06	0.744	125	0.518	14.1
T0833TS268_3-D1.rsa	64.706	0.659	0	0.341	43	1.505	31.35
T0833TS499_5-D1.rsa	63.462	0.285	0.097	0.618	102	0.622	77.53
T0833TS110_3-D1.rsa	62.5	0	0.255	0.745	82	0.762	44.55
T0833TS008_2-D1.rsa	62.5	0	0.236	0.764	84	0.744	18.86
T0833TS008_4-D1.rsa	60.714	0	0.374	0.626	82	0.74	12.6
T0833TS251_1-D1.rsa	60.227	0.061	0.304	0.635	188	0.32	17.4
T0833TS263_1-D1.rsa	59.155	0.214	0.077	0.708	119	0.497	22.92
T0833TS038_1-D1.rsa	58.824	0.286	0	0.714	90	0.654	28.77
T0833TS022_2-D1.rsa	58.333	0	0.537	0.463	50	1.167	46.06
T0833TS216_4-D1.rsa	56.25	0.391	0	0.609	67	0.84	16.36
T0833TS184_1-D1.rsa	55.882	0.635	0	0.365	46	1.215	36.51
T0833TS171_3-D1.rsa	55.814	0.276	0.134	0.59	79	0.707	17.91
T0833TS008_3-D1.rsa	55.814	0.493	0.097	0.41	55	1.015	38.06
T0833TS277_2-D1.rsa	54.167	0	0.528	0.472	51	1.062	57.18
T0833TS038_4-D1.rsa	54.167	0	0.315	0.685	74	0.732	30.32
T0833TS237_4-D1.rsa	54.167	0	0.639	0.361	39	1.389	69.44
T0833TS345_2-D1.rsa	54.167	0.037	0.454	0.509	55	0.985	34.26
T0833TS479_4-D1.rsa	53.571	0.015	0.366	0.618	81	0.661	16.98
T0833TS216_5-D1.rsa	52	0.275	0.196	0.529	73	0.712	12.14
T0833TS133_4-D1.rsa	50	0	0.359	0.641	84	0.595	17.56
T0833TS110_1-D1.rsa	50	0	0.389	0.611	80	0.625	17.94
T0833TS335_4-D1.rsa	50	0.252	0.108	0.64	71	0.704	37.61
T0833TS171_1-D1.rsa	50	0.252	0.108	0.64	71	0.704	37.61
T0833TS436_5-D1.rsa	50	0.552	0	0.448	74	0.676	17.72
T0833TS011_4-D1.rsa	50	0.635	0	0.365	46	1.087	36.51
T0833TS335_2-D1.rsa	48.837	0.239	0.104	0.657	88	0.555	23.13
T0833TS184_3-D1.rsa	48.837	0.418	0.015	0.567	76	0.643	25
T0833TS335_1-D1.rsa	47.5	0	0.333	0.667	76	0.625	20.18
T0833TS420_4-D1.rsa	47.368	0.045	0.045	0.91	101	0.469	22.07
T0833TS346_1-D1.rsa	47.368	0.207	0.171	0.622	69	0.686	31.98
T0833TS184_2-D1.rsa	46.875	0.015	0.125	0.86	117	0.401	13.23
T0833TS145_2-D1.rsa	46.875	0.184	0.081	0.735	100	0.469	36.4
T0833TS279_1-D1.rsa	46.512	0.284	0.082	0.634	85	0.547	18.1
T0833TS160_1-D1.rsa	46.512	0.269	0.097	0.634	85	0.547	18.1
T0833TS156_3-D1.rsa	46.429	0	0.107	0.893	117	0.397	16.41
T0833TS216_3-D1.rsa	45.833	0.018	0.382	0.6	66	0.694	20.68

T0833TS420_3-D1.rsa	45	0	0.342	0.658	75	0.6	21.71
T0833TS251_4-D1.rsa	44.8	0.012	0.334	0.653	215	0.208	23.94
T0833TS133_2-D1.rsa	44.737	0.252	0.153	0.595	66	0.678	31.98
T0833TS448_5-D1.rsa	44.737	0.27	0.09	0.64	71	0.63	28.6
T0833TS193_1-D1.rsa	44.643	0	0.286	0.714	90	0.496	18.25
T0833TS448_3-D1.rsa	44.643	0.159	0.325	0.516	65	0.687	47.42
T0833TS414_1-D1.rsa	44.643	0.23	0.238	0.532	67	0.666	20.24
T0833TS420_2-D1.rsa	43.75	0.015	0.324	0.662	90	0.486	13.42
T0833TS420_5-D1.rsa	43.75	0.082	0.436	0.482	53	0.825	55.91
T0833TS268_2-D1.rsa	42.857	0.27	0.143	0.587	74	0.579	22.62
T0833TS436_2-D1.rsa	42.105	0.25	0.519	0.231	25	1.684	87.04
T0833TS212_1-D1.rsa	41.86	0.224	0.015	0.761	102	0.41	22.95
T0833TS156_2-D1.rsa	41.667	0.018	0.364	0.618	68	0.613	56.82
T0833TS335_3-D1.rsa	41.667	0.273	0.164	0.564	62	0.672	41.59
T0833TS210_2-D1.rsa	39.773	0.071	0.416	0.514	152	0.262	39.1
T0833TS492_3-D1.rsa	39.583	0	0.218	0.782	86	0.46	62.27
T0833TS160_3-D1.rsa	39.394	0.229	0	0.771	222	0.177	25.35
T0833TS263_3-D1.rsa	39.286	0	0.382	0.618	81	0.485	36.26
T0833TS452_3-D1.rsa	38.356	0.678	0	0.322	82	0.468	41.37
T0833TS414_5-D1.rsa	38.356	0.737	0	0.263	67	0.572	35.78
T0833TS277_4-D1.rsa	37.5	0.404	0.094	0.502	107	0.35	76.65
T0833TS133_5-D1.rsa	37.5	0.14	0.088	0.772	105	0.357	13.97
T0833TS171_2-D1.rsa	37.5	0	0.175	0.825	94	0.399	33.55
T0833TS479_2-D1.rsa	37.5	0.143	0.357	0.5	63	0.595	45.83
T0833TS022_3-D1.rsa	37.5	0.019	0.565	0.417	45	0.833	53.01
T0833TS184_5-D1.rsa	37.5	0.035	0.412	0.553	63	0.595	36.4
T0833TS414_4-D1.rsa	36.842	0.222	0.324	0.454	49	0.752	65.28
T0833TS041_5-D1.rsa	36.765	0.226	0.086	0.687	167	0.22	48.44
T0833TS171_4-D1.rsa	36.62	0.405	0.131	0.464	78	0.469	41.03
T0833TS454_3-D1.rsa	35.938	0.081	0.309	0.61	83	0.433	13.23
T0833TS454_1-D1.rsa	35.714	0.175	0.349	0.476	60	0.595	40.87
T0833TS216_1-D1.rsa	35.714	0.167	0.373	0.46	58	0.616	43.85
T0833TS133_3-D1.rsa	35.417	0.064	0.264	0.673	74	0.479	52.27
T0833TS145_5-D1.rsa	34.884	0.216	0.157	0.627	84	0.415	23.88
T0833TS156_4-D1.rsa	34.884	0.313	0.134	0.552	74	0.471	29.66
T0833TS345_5-D1.rsa	34.375	0.015	0.463	0.522	71	0.484	66.36
T0833TS452_5-D1.rsa	34.211	0.225	0.126	0.649	72	0.475	34.69
T0833TS022_1-D1.rsa	33.929	0.19	0.222	0.587	74	0.458	27.98
T0833TS381_3-D1.rsa	33.803	0.488	0.065	0.446	75	0.451	50.96
T0833TS381_4-D1.rsa	33.803	0.482	0.137	0.381	64	0.528	40.87
T0833TS011_2-D1.rsa	33.803	0.494	0.143	0.363	61	0.554	39.58
T0833TS228_1-D1.rsa	33.333	0.356	0.082	0.562	123	0.271	58.56
T0833TS499_1-D1.rsa	32.877	0.741	0	0.259	66	0.498	65.39
T0833TS011_3-D1.rsa	32.558	0.291	0.127	0.582	78	0.417	18.84
T0833TS237_3-D1.rsa	32.5	0	0.404	0.596	68	0.478	47.81
T0833TS349_4-D1.rsa	32.394	0.512	0.077	0.411	69	0.469	50
T0833TS436_3-D1.rsa	32.143	0.167	0.302	0.532	67	0.48	44.84

T0833TS237_1-D1.rsa	32.143	0.167	0.357	0.476	60	0.536	48.61
T0833TS448_1-D1.rsa	32.143	0.167	0.325	0.508	64	0.502	43.85
T0833TS345_4-D1.rsa	31.897	0.656	0	0.344	88	0.362	57.52
T0833TS210_5-D1.rsa	31.683	0.268	0.143	0.589	189	0.168	74.61
T0833TS410_5-D1.rsa	31.579	0.564	0.055	0.381	90	0.351	58.26
T0833TS349_1-D1.rsa	31.579	0.241	0.472	0.287	31	1.019	93.52
T0833TS022_5-D1.rsa	31.579	0.216	0	0.784	87	0.363	38.96
T0833TS381_2-D1.rsa	31.579	0.25	0.444	0.306	33	0.957	82.41
T0833TS452_4-D1.rsa	31.507	0.722	0	0.278	71	0.444	35
T0833TS011_5-D1.rsa	31.507	0.745	0	0.255	65	0.485	36.18
T0833TS011_1-D1.rsa	31.507	0.765	0	0.235	60	0.525	39.51
T0833TS268_4-D1.rsa	31.25	0.096	0.265	0.64	87	0.359	15.44
T0833TS414_2-D1.rsa	30.986	0.506	0.089	0.405	68	0.456	52.4
T0833TS335_5-D1.rsa	30.682	0.061	0.51	0.429	127	0.242	47.04
T0833TS156_1-D1.rsa	30.357	0.19	0	0.81	102	0.298	29.96
T0833TS008_5-D1.rsa	30.357	0.167	0.325	0.508	64	0.474	57.14
T0833TS499_2-D1.rsa	30.357	0.175	0.373	0.452	57	0.533	51.59
T0833TS216_2-D1.rsa	30.357	0.167	0.349	0.484	61	0.498	50.99
T0833TS499_4-D1.rsa	30.233	0.337	0.219	0.444	75	0.403	73.22
T0833TS117_4-D1.rsa	30.137	0.399	0.105	0.496	184	0.164	80.79
T0833TS448_2-D1.rsa	30	0	0.307	0.693	79	0.38	25.66
T0833TS436_4-D1.rsa	29.577	0.494	0.113	0.393	66	0.448	50.16
T0833TS184_4-D1.rsa	29.577	0.524	0.036	0.44	74	0.4	38.46
T0833TS117_1-D1.rsa	29.293	0.198	0.16	0.642	185	0.158	63.28
T0833TS410_1-D1.rsa	29.07	0.308	0.201	0.491	83	0.35	72.19
T0833TS041_2-D1.rsa	28.947	0.241	0.5	0.259	28	1.034	93.52
T0833TS479_1-D1.rsa	28.947	0.25	0.5	0.25	27	1.072	94.44
T0833TS499_3-D1.rsa	28.947	0.25	0.426	0.324	35	0.827	92.82
T0833TS237_2-D1.rsa	28.947	0.25	0.509	0.241	26	1.113	93.98
T0833TS210_3-D1.rsa	28.767	0.418	0.108	0.474	176	0.163	84.81
T0833TS145_1-D1.rsa	28.571	0.372	0.218	0.41	96	0.298	71.37
T0833TS454_2-D1.rsa	28.571	0.151	0.381	0.468	59	0.484	50.79
T0833TS038_2-D1.rsa	28.169	0.339	0.089	0.571	96	0.293	36.7
T0833TS436_1-D1.rsa	28	0.43	0.12	0.45	113	0.248	81.67
T0833TS410_2-D1.rsa	28	0.058	0.312	0.63	87	0.322	68.84
T0833TS038_3-D1.rsa	27.5	0	0.307	0.693	79	0.348	41.89
T0833TS156_5-D1.rsa	27.5	0	0.298	0.702	80	0.344	42.54
T0833TS349_3-D1.rsa	27.5	0	0.158	0.842	96	0.286	18.86
T0833TS381_1-D1.rsa	27.5	0.035	0.342	0.623	71	0.387	46.27
T0833TS268_1-D1.rsa	27.397	0.769	0	0.231	59	0.464	66.18
T0833TS277_5-D1.rsa	27.273	0.205	0.142	0.653	188	0.145	54.25
T0833TS228_2-D1.rsa	27.273	0.233	0.16	0.608	175	0.156	65.97
T0833TS041_1-D1.rsa	26.786	0.175	0.325	0.5	63	0.425	50.99
T0833TS454_5-D1.rsa	26.733	0.24	0.1	0.66	212	0.126	70.09
T0833TS410_4-D1.rsa	26.027	0.6	0	0.4	102	0.255	40.29
T0833TS251_2-D1.rsa	25.85	0.381	0.097	0.523	195	0.133	82.43
T0833TS228_4-D1.rsa	24.571	0.51	0.01	0.48	194	0.127	42.95

T0833TS145_3-D1.rsa	24.359	0.321	0.081	0.598	177	0.138	52.26
T0833TS263_5-D1.rsa	24	0.418	0.012	0.569	230	0.104	40.59
T0833TS345_1-D1.rsa	24	0.072	0.246	0.681	94	0.255	67.94
T0833TS349_2-D1.rsa	24	0.014	0.145	0.841	116	0.207	18.84
T0833TS251_5-D1.rsa	23.457	0.406	0.09	0.504	230	0.102	75.77
T0833TS160_4-D1.rsa	23.288	0.431	0.108	0.461	171	0.136	84.06
T0833TS228_3-D1.rsa	22.286	0.485	0	0.515	208	0.107	36.88
T0833TS210_4-D1.rsa	20.988	0.406	0.088	0.507	231	0.091	75.11
T0833TS110_5-D1.rsa	20.93	0.413	0.117	0.47	133	0.157	82.77
T0833TS228_5-D1.rsa	19.737	0.307	0.084	0.608	202	0.098	47.89
T0833TS251_3-D1.rsa	19.298	0.452	0.091	0.457	100	0.193	81.16
T0833TS263_4-D1.rsa	17.544	0.452	0.082	0.466	102	0.172	80.59
T0833TS479_3-D1.rsa	16	0.036	0.261	0.703	97	0.165	48.01
T0833TS210_1-D1.rsa	15.116	0.403	0.102	0.495	140	0.108	79.95
T0833TS110_4-D1.rsa	14.894	0.323	0.187	0.49	126	0.118	86.38
T0833TS300_5-D1.rsa	13.846	0.247	0.201	0.552	253	0.055	68.11
T0833TS454_4-D1.rsa	12.766	0.346	0.175	0.479	123	0.104	81.91
T0833TS345_3-D1.rsa	12.281	0.429	0.1	0.47	103	0.119	47.49
T0833TS050_1-D1.rsa	12	0	0.268	0.732	101	0.119	38.23
T0833TS117_3-D1.rsa	11.842	0.331	0.142	0.527	175	0.068	99.17
T0860TS464_5-D1.rsa	79.688	0.022	0.044	0.934	127	0.627	11.58
T0860TS464_2-D1.rsa	75	0.066	0	0.934	127	0.591	11.03
T0860TS284_3-D1.rsa	71.875	0	0.022	0.978	133	0.54	12.68
T0860TS464_3-D1.rsa	70.313	0.088	0	0.912	124	0.567	32.54
T0860TS407_3-D1.rsa	70.313	0.118	0.162	0.721	98	0.717	48.35
T0860TS451_5-D1.rsa	68.75	0	0.103	0.897	122	0.564	16.18
T0860TS407_2-D1.rsa	67.188	0.125	0.176	0.699	95	0.707	49.82
T0860TS451_4-D1.rsa	64.063	0	0.044	0.956	130	0.493	16.18
T0860TS451_2-D1.rsa	64.063	0	0.125	0.875	119	0.538	18.02
T0860TS407_4-D1.rsa	64.063	0.103	0.199	0.699	95	0.674	47.43
T0860TS284_5-D1.rsa	62.5	0.088	0.199	0.713	97	0.644	14.71
T0860TS284_4-D1.rsa	60.938	0.066	0.191	0.743	101	0.603	15.81
T0860TS451_1-D1.rsa	60.938	0	0.044	0.956	130	0.469	17.46
T0860TS444_5-D1.rsa	59.375	0.191	0.118	0.691	94	0.632	11.58
T0860TS284_2-D1.rsa	59.375	0.11	0.191	0.699	95	0.625	15.07
T0860TS444_4-D1.rsa	59.375	0.191	0.176	0.632	86	0.69	11.4
T0860TS407_1-D1.rsa	59.375	0.125	0.184	0.691	94	0.632	49.45
T0860TS432_5-D1.rsa	59.375	0.118	0.118	0.765	104	0.571	14.71
T0860TS451_3-D1.rsa	57.813	0	0.044	0.956	130	0.445	18.93
T0860TS455_3-D1.rsa	56.25	0.029	0.074	0.897	122	0.461	13.6
T0860TS464_1-D1.rsa	56.25	0.074	0	0.926	126	0.446	31.8
T0860TS467_2-D1.rsa	54.688	0.059	0.279	0.662	90	0.608	13.79
T0860TS455_5-D1.rsa	54.688	0	0.14	0.86	117	0.467	13.23
T0860TS464_4-D1.rsa	54.688	0.074	0.029	0.897	122	0.448	32.54
T0860TS321_5-D1.rsa	54.688	0.125	0.206	0.669	91	0.601	13.97
T0860TS321_3-D1.rsa	54.688	0.125	0.206	0.669	91	0.601	13.05
T0860TS016_1-D1.rsa	53.125	0	0.294	0.706	96	0.553	13.05

T0860TS434_2-D1.rsa	53.125	0.169	0.029	0.801	109	0.487	13.05
T0860TS421_4-D1.rsa	53.125	0	0.015	0.985	134	0.396	12.87
T0860TS444_2-D1.rsa	53.125	0.14	0.118	0.743	101	0.526	11.58
T0860TS321_1-D1.rsa	53.125	0.096	0.228	0.676	92	0.577	16.54
T0860TS455_1-D1.rsa	51.563	0.029	0.14	0.831	113	0.456	13.6
T0860TS026_3-D1.rsa	51.563	0	0.228	0.772	105	0.491	12.32
T0860TS321_4-D1.rsa	51.563	0.096	0.221	0.684	93	0.554	13.6
T0860TS444_3-D1.rsa	51.563	0.199	0.14	0.662	90	0.573	11.4
T0860TS467_5-D1.rsa	51.563	0	0.235	0.765	104	0.496	17.1
T0860TS357_4-D1.rsa	51.563	0.015	0.316	0.669	91	0.567	42.1
T0860TS180_1-D1.rsa	50	0	0.272	0.728	99	0.505	14.89
T0860TS026_1-D1.rsa	50	0.044	0.279	0.676	92	0.543	12.13
T0860TS357_1-D1.rsa	50	0.015	0.309	0.676	92	0.543	41.54
T0860TS432_2-D1.rsa	50	0.096	0.184	0.721	98	0.51	11.58
T0860TS357_3-D1.rsa	50	0	0.221	0.779	106	0.472	44.3
T0860TS357_2-D1.rsa	50	0	0.228	0.772	105	0.476	44.67
T0860TS180_3-D1.rsa	48.438	0.015	0.272	0.713	97	0.499	16.54
T0860TS455_4-D1.rsa	48.438	0	0.029	0.971	132	0.367	11.77
T0860TS434_1-D1.rsa	46.875	0.162	0.118	0.721	98	0.478	12.87
T0860TS434_5-D1.rsa	46.875	0.162	0	0.838	114	0.411	11.77
T0860TS446_3-D1.rsa	46.875	0.169	0.419	0.412	56	0.837	12.68
T0860TS345_5-D1.rsa	46.875	0.081	0.191	0.728	99	0.473	33.46
T0860TS357_5-D1.rsa	46.875	0.015	0.257	0.728	99	0.473	44.12
T0860TS434_4-D1.rsa	46.875	0.162	0.029	0.809	110	0.426	14.34
T0860TS434_3-D1.rsa	46.875	0.147	0.015	0.838	114	0.411	13.42
T0860TS180_4-D1.rsa	45.313	0.074	0.191	0.735	100	0.453	13.42
T0860TS446_4-D1.rsa	45.313	0.206	0.346	0.449	61	0.743	12.5
T0860TS495_2-D1.rsa	45.313	0.015	0.287	0.699	95	0.477	63.97
T0860TS455_2-D1.rsa	43.75	0	0.044	0.956	130	0.337	11.03
T0860TS425_1-D1.rsa	43.75	0.015	0.493	0.493	67	0.653	76.65
T0860TS446_5-D1.rsa	43.75	0.184	0.324	0.493	67	0.653	12.68
T0860TS430_5-D1.rsa	43.75	0	0.537	0.463	63	0.694	79.23
T0860TS275_4-D1.rsa	42.188	0.015	0.434	0.551	75	0.563	77.57
T0860TS077_3-D1.rsa	42.188	0.015	0.478	0.507	69	0.611	77.39
T0860TS345_1-D1.rsa	42.188	0.015	0.493	0.493	67	0.63	69.3
T0860TS430_4-D1.rsa	42.188	0	0.485	0.515	70	0.603	79.41
T0860TS005_4-D1.rsa	42.188	0.029	0.5	0.471	64	0.659	79.41
T0860TS092_1-D1.rsa	42.188	0.015	0.471	0.515	70	0.603	68.57
T0860TS220_5-D1.rsa	42.188	0.015	0.5	0.485	66	0.639	78.12
T0860TS005_5-D1.rsa	42.188	0.015	0.507	0.478	65	0.649	81.62
T0860TS432_4-D1.rsa	42.188	0.11	0.132	0.757	103	0.41	15.62
T0860TS467_1-D1.rsa	40.625	0	0.346	0.654	89	0.456	15.44
T0860TS258_1-D1.rsa	40.625	0.015	0.426	0.559	76	0.535	70.04
T0860TS258_2-D1.rsa	40.625	0.015	0.434	0.551	75	0.542	69.85
T0860TS479_5-D1.rsa	40.625	0.125	0.213	0.662	90	0.451	16.91
T0860TS425_3-D1.rsa	40.625	0.015	0.449	0.537	73	0.557	77.94
T0860TS479_4-D1.rsa	40.625	0.015	0.162	0.824	112	0.363	18.57

T0860TS421_3-D1.rsa	40.625	0.015	0.426	0.559	76	0.535	69.67
T0860TS479_1-D1.rsa	40.625	0.015	0.441	0.544	74	0.549	72.61
T0860TS425_5-D1.rsa	40.625	0.015	0.434	0.551	75	0.542	76.47
T0860TS275_2-D1.rsa	40.625	0.015	0.441	0.544	74	0.549	77.39
T0860TS405_3-D1.rsa	40.625	0.015	0.441	0.544	74	0.549	71.51
T0860TS287_5-D1.rsa	40.625	0.015	0.463	0.522	71	0.572	70.77
T0860TS287_4-D1.rsa	40.625	0.015	0.478	0.507	69	0.589	70.59
T0860TS345_4-D1.rsa	40.625	0.015	0.507	0.478	65	0.625	72.61
T0860TS220_2-D1.rsa	40.625	0.029	0.449	0.522	71	0.572	78.31
T0860TS432_1-D1.rsa	40.625	0.103	0.221	0.676	92	0.442	13.79
T0860TS220_1-D1.rsa	40.625	0.015	0.471	0.515	70	0.58	79.41
T0860TS359_5-D1.rsa	39.063	0.015	0.449	0.537	73	0.535	77.21
T0860TS284_1-D1.rsa	39.063	0.015	0.441	0.544	74	0.528	72.43
T0860TS425_2-D1.rsa	39.063	0.015	0.478	0.507	69	0.566	76.1
T0860TS425_4-D1.rsa	39.063	0.015	0.426	0.559	76	0.514	77.76
T0860TS258_3-D1.rsa	39.063	0.015	0.449	0.537	73	0.535	69.3
T0860TS359_2-D1.rsa	39.063	0.015	0.456	0.529	72	0.543	77.39
T0860TS183_1-D1.rsa	39.063	0.015	0.404	0.581	79	0.494	72.43
T0860TS495_1-D1.rsa	39.063	0.015	0.39	0.596	81	0.482	71.51
T0860TS359_1-D1.rsa	39.063	0.015	0.485	0.5	68	0.574	76.65
T0860TS077_1-D1.rsa	39.063	0.015	0.463	0.522	71	0.55	77.94
T0860TS287_1-D1.rsa	39.063	0.015	0.5	0.485	66	0.592	70.4
T0860TS005_3-D1.rsa	39.063	0.015	0.397	0.588	80	0.488	75.92
T0860TS077_5-D1.rsa	39.063	0.015	0.456	0.529	72	0.543	77.21
T0860TS321_2-D1.rsa	39.063	0.096	0.213	0.691	94	0.416	12.32
T0860TS077_4-D1.rsa	39.063	0.015	0.478	0.507	69	0.566	77.39
T0860TS349_1-D1.rsa	39.063	0.015	0.434	0.551	75	0.521	77.94
T0860TS180_2-D1.rsa	39.063	0.272	0.176	0.551	75	0.521	14.52
T0860TS287_3-D1.rsa	39.063	0.015	0.441	0.544	74	0.528	70.59
T0860TS251_3-D1.rsa	39.063	0.029	0.478	0.493	67	0.583	68.38
T0860TS430_1-D1.rsa	39.063	0	0.493	0.507	69	0.566	79.96
T0860TS183_3-D1.rsa	39.063	0.015	0.162	0.824	112	0.349	17.83
T0860TS236_3-D1.rsa	39.063	0.015	0.382	0.603	82	0.476	70.22
T0860TS287_2-D1.rsa	39.063	0.015	0.36	0.625	85	0.46	70.4
T0860TS432_3-D1.rsa	39.063	0.118	0.14	0.743	101	0.387	17.28
T0860TS421_2-D1.rsa	37.5	0.015	0.434	0.551	75	0.5	69.3
T0860TS421_1-D1.rsa	37.5	0.015	0.39	0.596	81	0.463	70.04
T0860TS359_3-D1.rsa	37.5	0.015	0.441	0.544	74	0.507	77.39
T0860TS479_2-D1.rsa	37.5	0.015	0.412	0.574	78	0.481	71.32
T0860TS183_2-D1.rsa	37.5	0.015	0.397	0.588	80	0.469	72.24
T0860TS250_3-D1.rsa	37.5	0.015	0.419	0.566	77	0.487	72.43
T0860TS275_1-D1.rsa	37.5	0.015	0.456	0.529	72	0.521	76.65
T0860TS220_4-D1.rsa	37.5	0.015	0.485	0.5	68	0.551	78.68
T0860TS236_2-D1.rsa	37.5	0.015	0.478	0.507	69	0.543	69.67
T0860TS236_1-D1.rsa	37.5	0.015	0.493	0.493	67	0.56	70.77
T0860TS382_2-D1.rsa	37.5	0.015	0.397	0.588	80	0.469	71.69
T0860TS382_3-D1.rsa	37.5	0.015	0.375	0.61	83	0.452	71.88

T0860TS382_4-D1.rsa	37.5	0.015	0.397	0.588	80	0.469	71.88
T0860TS275_3-D1.rsa	37.5	0.015	0.456	0.529	72	0.521	78.12
T0860TS313_1-D1.rsa	37.5	0.015	0.426	0.559	76	0.493	78.31
T0860TS250_1-D1.rsa	37.5	0.015	0.412	0.574	78	0.481	72.61
T0860TS077_2-D1.rsa	37.5	0.015	0.463	0.522	71	0.528	77.39
T0860TS275_5-D1.rsa	37.5	0.015	0.471	0.515	70	0.536	77.94
T0860TS444_1-D1.rsa	37.5	0	0.478	0.522	71	0.528	67.28
T0860TS236_4-D1.rsa	37.5	0.015	0.463	0.522	71	0.528	68.75
T0860TS495_3-D1.rsa	37.5	0.015	0.346	0.64	87	0.431	70.77
T0860TS405_1-D1.rsa	37.5	0.015	0.507	0.478	65	0.577	72.79
T0860TS430_2-D1.rsa	37.5	0	0.449	0.551	75	0.5	79.78
T0860TS005_1-D1.rsa	37.5	0.015	0.478	0.507	69	0.543	81.8
T0860TS430_3-D1.rsa	37.5	0	0.478	0.522	71	0.528	79.41
T0860TS495_4-D1.rsa	37.5	0.015	0.39	0.596	81	0.463	72.61
T0860TS026_5-D1.rsa	37.5	0.044	0.309	0.647	88	0.426	11.58
T0860TS258_5-D1.rsa	35.938	0.015	0.397	0.588	80	0.449	69.3
T0860TS359_4-D1.rsa	35.938	0.015	0.419	0.566	77	0.467	72.98
T0860TS446_1-D1.rsa	35.938	0.015	0.419	0.566	77	0.467	71.32
T0860TS250_5-D1.rsa	35.938	0.015	0.412	0.574	78	0.461	72.43
T0860TS313_2-D1.rsa	35.938	0.015	0.434	0.551	75	0.479	78.68
T0860TS313_4-D1.rsa	35.938	0.015	0.426	0.559	76	0.473	78.86
T0860TS345_2-D1.rsa	35.938	0	0.529	0.471	64	0.562	68.57
T0860TS236_5-D1.rsa	35.938	0.015	0.456	0.529	72	0.499	70.4
T0860TS382_1-D1.rsa	35.938	0.015	0.397	0.588	80	0.449	71.69
T0860TS005_2-D1.rsa	35.938	0.015	0.5	0.485	66	0.545	77.39
T0860TS119_1-D1.rsa	35.938	0.015	0.434	0.551	75	0.479	77.94
T0860TS313_5-D1.rsa	35.938	0.015	0.426	0.559	76	0.473	79.04
T0860TS313_3-D1.rsa	35.938	0.015	0.434	0.551	75	0.479	79.04
T0860TS251_1-D1.rsa	35.938	0.029	0.426	0.544	74	0.486	63.79
T0860TS251_5-D1.rsa	35.938	0.015	0.426	0.559	76	0.473	65.81
T0860TS251_2-D1.rsa	35.938	0.015	0.426	0.559	76	0.473	67.46
T0860TS220_3-D1.rsa	35.938	0.015	0.456	0.529	72	0.499	78.31
T0860TS467_3-D1.rsa	35.938	0	0.228	0.772	105	0.342	13.23
T0860TS048_1-D1.rsa	35.938	0.015	0.471	0.515	70	0.513	68.38
T0860TS467_4-D1.rsa	35.938	0	0.235	0.765	104	0.346	13.05
T0860TS251_4-D1.rsa	35.938	0.015	0.434	0.551	75	0.479	68.38
T0860TS258_4-D1.rsa	34.375	0.015	0.397	0.588	80	0.43	70.77
T0860TS495_5-D1.rsa	34.375	0.015	0.434	0.551	75	0.458	72.98
T0860TS405_2-D1.rsa	34.375	0.015	0.419	0.566	77	0.446	72.06
T0860TS250_2-D1.rsa	34.375	0.015	0.412	0.574	78	0.441	72.79
T0860TS026_4-D1.rsa	34.375	0	0.272	0.728	99	0.347	14.52
T0860TS183_4-D1.rsa	32.813	0.103	0.162	0.735	100	0.328	17.1
T0860TS250_4-D1.rsa	32.813	0.015	0.419	0.566	77	0.426	72.61
T0860TS345_3-D1.rsa	32.813	0	0.529	0.471	64	0.513	69.85
T0860TS479_3-D1.rsa	31.25	0.015	0.213	0.772	105	0.298	12.5
T0860TS183_5-D1.rsa	31.25	0.081	0.147	0.772	105	0.298	12.68
T0845TS008_4-D2.rsa	75	0.194	0.024	0.782	129	0.581	21.68

T0845TS268_2-D2.rsa	64.912	0.242	0	0.758	166	0.391	11.99
T0845TS008_3-D2.rsa	63.462	0.279	0.024	0.697	115	0.552	69.46
T0845TS452_2-D2.rsa	61.538	0.261	0.097	0.642	106	0.581	68.35
T0845TS038_2-D2.rsa	61.538	0.279	0.073	0.648	107	0.575	62.18
T0845TS452_5-D2.rsa	61.538	0.279	0.152	0.57	94	0.655	77.06
T0845TS184_4-D2.rsa	59.615	0.297	0.127	0.576	95	0.628	77.37
T0845TS279_1-D2.rsa	59.048	0.209	0	0.791	185	0.319	10.36
T0845TS420_5-D2.rsa	57.759	0.273	0	0.727	186	0.311	8.4
T0845TS184_1-D2.rsa	55.882	0.35	0.091	0.56	136	0.411	42.78
T0845TS414_2-D2.rsa	55.769	0.285	0.115	0.6	99	0.563	76.42
T0845TS436_3-D2.rsa	54.545	0.068	0.209	0.723	214	0.255	19.43
T0845TS011_3-D2.rsa	53.846	0.242	0.048	0.709	117	0.46	58.07
T0845TS145_2-D2.rsa	53.846	0.273	0.085	0.642	106	0.508	65.03
T0845TS041_2-D2.rsa	51.923	0.273	0.085	0.642	106	0.49	63.92
T0845TS492_3-D2.rsa	51.136	0.074	0.216	0.709	210	0.244	19.17
T0845TS050_1-D2.rsa	48.529	0.177	0.012	0.811	197	0.246	10.11
T0845TS420_3-D2.rsa	48.276	0	0.098	0.902	129	0.374	52.1
T0845TS145_1-D2.rsa	46.512	0.187	0	0.813	109	0.427	37.87
T0845TS008_2-D2.rsa	44.828	0	0.098	0.902	129	0.348	52.1
T0845TS452_3-D2.rsa	44.737	0.318	0.115	0.567	123	0.364	58.76
T0845TS268_5-D2.rsa	42.857	0	0.069	0.931	122	0.351	61.26
T0845TS381_1-D2.rsa	42.105	0.272	0.129	0.599	130	0.324	52.19
T0845TS011_5-D2.rsa	41.379	0	0.056	0.944	135	0.307	58.74
T0845TS410_2-D2.rsa	41.379	0	0.119	0.881	126	0.328	60.84
T0845TS133_5-D2.rsa	41.176	0.403	0	0.597	145	0.284	45.33
T0845TS145_3-D2.rsa	41.176	0.395	0.074	0.531	129	0.319	25.11
T0845TS171_2-D2.rsa	40.404	0.215	0.149	0.635	183	0.221	43.4
T0845TS436_1-D2.rsa	39.773	0.071	0.402	0.527	156	0.255	35.56
T0845TS216_3-D2.rsa	38.636	0.057	0.456	0.486	144	0.268	42.23
T0845TS216_5-D2.rsa	37.719	0.249	0.171	0.581	126	0.299	64.4
T0845TS335_3-D2.rsa	37.705	0.038	0.386	0.576	121	0.312	62.31
T0845TS345_5-D2.rsa	36.842	0.334	0.012	0.654	217	0.17	10.47
T0845TS145_4-D2.rsa	36.842	0.24	0.175	0.585	127	0.29	57.26
T0845TS160_4-D2.rsa	36.364	0.108	0.426	0.466	138	0.264	47.47
T0845TS216_2-D2.rsa	35.938	0	0.184	0.816	111	0.324	66.18
T0845TS349_5-D2.rsa	35.294	0.333	0.099	0.568	138	0.256	60.33
T0845TS184_3-D2.rsa	34.722	0.319	0.094	0.587	125	0.278	72.97
T0845TS381_5-D2.rsa	34.426	0.01	0.39	0.6	126	0.273	63.56
T0845TS156_4-D2.rsa	34.4	0.015	0.368	0.617	203	0.169	49.77
T0845TS499_1-D2.rsa	33.824	0.358	0.091	0.551	134	0.252	65.78
T0845TS499_2-D2.rsa	33.333	0.181	0.083	0.736	212	0.157	42.88
T0845TS448_4-D2.rsa	32.895	0.364	0.039	0.596	198	0.166	38.7
T0845TS479_2-D2.rsa	32.8	0.012	0.419	0.568	187	0.175	54.86
T0845TS237_4-D2.rsa	32.787	0	0.367	0.633	133	0.247	64.06
T0845TS008_1-D2.rsa	32.353	0.337	0.062	0.601	146	0.222	57.67
T0845TS454_3-D2.rsa	32.353	0.432	0.132	0.436	106	0.305	64.89
T0845TS041_1-D2.rsa	31.579	0.343	0.024	0.633	210	0.15	11.07

T0845TS263_2-D2.rsa	31.313	0.236	0.174	0.59	170	0.184	58.68
T0845TS216_1-D2.rsa	31.034	0	0.07	0.93	133	0.233	50.87
T0845TS277_5-D2.rsa	30.822	0.337	0.049	0.615	228	0.135	58.58
T0845TS263_5-D2.rsa	30.693	0.255	0.134	0.611	196	0.157	74.84
T0845TS038_4-D2.rsa	30.508	0.009	0.252	0.738	158	0.193	60.05
T0845TS479_5-D2.rsa	30.4	0.012	0.274	0.714	235	0.129	28.65
T0845TS448_5-D2.rsa	30.4	0.012	0.398	0.59	194	0.157	54.48
T0845TS492_2-D2.rsa	29.703	0.259	0.134	0.607	195	0.152	74.38
T0845TS210_1-D2.rsa	29.6	0.015	0.419	0.565	186	0.159	56.38
T0845TS452_4-D2.rsa	29.545	0.068	0.48	0.453	134	0.22	51.69
T0845TS414_4-D2.rsa	29.508	0.024	0.429	0.548	115	0.257	65.3
T0845TS156_3-D2.rsa	29.452	0.394	0.097	0.509	189	0.156	73.03
T0845TS420_4-D2.rsa	29.452	0.404	0.111	0.485	180	0.164	73.71
T0845TS216_4-D2.rsa	29.412	0.403	0.103	0.494	120	0.245	64.22
T0845TS492_1-D2.rsa	29.293	0.184	0.125	0.691	199	0.147	60.16
T0845TS171_5-D2.rsa	29.293	0.201	0.191	0.608	175	0.167	63.45
T0845TS436_4-D2.rsa	29.252	0.351	0.094	0.555	207	0.141	81.67
T0845TS160_3-D2.rsa	29.252	0.386	0.113	0.501	187	0.156	73.19
T0845TS117_4-D2.rsa	28.814	0.019	0.28	0.701	150	0.192	60.51
T0845TS171_4-D2.rsa	28.8	0.049	0.283	0.669	220	0.131	31.99
T0845TS237_2-D2.rsa	28.571	0.5	0.015	0.485	196	0.146	39.6
T0845TS263_1-D2.rsa	28.082	0.388	0.113	0.499	185	0.152	78.41
T0845TS277_1-D2.rsa	28.082	0.38	0.086	0.534	198	0.142	65.46
T0845TS228_1-D2.rsa	28.082	0.394	0.108	0.499	185	0.152	78.41
T0845TS237_5-D2.rsa	27.941	0.362	0.07	0.568	138	0.202	69.56
T0845TS145_5-D2.rsa	27.941	0.436	0.103	0.461	112	0.249	67.11
T0845TS228_3-D2.rsa	27.632	0.211	0.081	0.708	235	0.118	35.32
T0845TS448_3-D2.rsa	27.632	0.283	0.06	0.657	218	0.127	10.17
T0845TS171_3-D2.rsa	27.429	0.443	0.035	0.522	211	0.13	40.1
T0845TS228_4-D2.rsa	27.397	0.41	0.111	0.48	178	0.154	71.53
T0845TS117_1-D2.rsa	27.273	0.243	0.181	0.576	166	0.164	70.57
T0845TS300_4-D2.rsa	27.211	0.381	0.102	0.517	193	0.141	85.76
T0845TS410_1-D2.rsa	27.119	0	0.285	0.715	153	0.177	55.26
T0845TS011_4-D2.rsa	27	0.398	0.116	0.486	122	0.221	88.84
T0845TS277_4-D2.rsa	26.712	0.394	0.108	0.499	185	0.144	81.27
T0845TS414_5-D2.rsa	26.471	0.374	0.091	0.535	130	0.204	65.89
T0845TS454_4-D2.rsa	26.471	0.449	0.07	0.481	117	0.226	61.33
T0845TS251_2-D2.rsa	26.316	0.274	0.03	0.696	231	0.114	47.14
T0845TS041_4-D2.rsa	26.286	0.587	0.012	0.401	162	0.162	51.05
T0845TS410_4-D2.rsa	26.263	0.229	0.215	0.556	160	0.164	69.44
T0845TS117_3-D2.rsa	26.027	0.437	0.102	0.461	171	0.152	66.28
T0845TS335_4-D2.rsa	26	0.402	0.112	0.486	122	0.213	88.05
T0845TS479_1-D2.rsa	25.85	0.394	0.126	0.48	179	0.144	86.81
T0845TS237_3-D2.rsa	25.743	0.324	0.128	0.548	176	0.146	77.57
T0845TS277_3-D2.rsa	25.714	0.416	0.04	0.545	220	0.117	45.05
T0845TS300_2-D2.rsa	25.309	0.382	0.075	0.544	248	0.102	53.31
T0845TS492_4-D2.rsa	25.17	0.397	0.107	0.496	185	0.136	83.89

T0845TS492_5-D2.rsa	25.143	0.431	0.017	0.552	223	0.113	46.16
T0845TS038_5-D2.rsa	25.143	0.431	0.054	0.515	208	0.121	42.76
T0845TS436_2-D2.rsa	25	0.333	0.082	0.584	142	0.176	61.22
T0845TS038_1-D2.rsa	24.752	0.299	0.128	0.573	184	0.135	78.11
T0845TS448_2-D2.rsa	24.571	0.473	0.005	0.522	211	0.116	42.7
T0845TS251_3-D2.rsa	24.49	0.373	0.102	0.525	196	0.125	84.58
T0845TS041_3-D2.rsa	24.359	0.361	0.108	0.53	157	0.155	56.42
T0845TS479_3-D2.rsa	24	0.438	0.025	0.537	217	0.111	41.95
T0845TS499_4-D2.rsa	24	0.067	0.292	0.641	211	0.114	31.84
T0845TS452_1-D2.rsa	24	0.394	0.12	0.486	122	0.197	88.55
T0845TS160_1-D2.rsa	24	0.402	0.12	0.478	120	0.2	90.34
T0845TS381_3-D2.rsa	24	0.426	0.12	0.454	114	0.211	90.24
T0845TS335_5-D2.rsa	23.729	0	0.332	0.668	143	0.166	63.67
T0845TS133_2-D2.rsa	23.529	0.374	0.066	0.56	136	0.173	64.67
T0845TS268_1-D2.rsa	23.529	0.395	0.066	0.539	131	0.18	68.56
T0845TS349_2-D2.rsa	23.429	0.5	0.01	0.49	198	0.118	41.34
T0845TS133_3-D2.rsa	23.429	0.564	0	0.436	176	0.133	34.34
T0845TS345_4-D2.rsa	22.951	0.048	0.086	0.867	182	0.126	52.24
T0845TS228_5-D2.rsa	22.84	0.43	0.088	0.482	220	0.104	34.22
T0845TS210_2-D2.rsa	22.772	0.318	0.156	0.526	169	0.135	75.39
T0845TS110_5-D2.rsa	22.368	0.41	0.024	0.566	258	0.087	64.97
T0845TS414_3-D2.rsa	22.286	0.5	0.047	0.453	183	0.122	27.97
T0845TS041_5-D2.rsa	22.093	0.389	0.11	0.502	142	0.156	72.79
T0845TS414_1-D2.rsa	22.059	0.333	0.07	0.597	145	0.152	57.67
T0845TS171_1-D2.rsa	22.034	0	0.187	0.813	174	0.127	56.78
T0845TS011_1-D2.rsa	21.714	0.507	0.015	0.478	193	0.113	43.63
T0845TS335_2-D2.rsa	21.714	0.522	0.027	0.45	182	0.119	44.74
T0845TS436_5-D2.rsa	21.605	0.414	0.094	0.491	224	0.096	62.64
T0845TS160_5-D2.rsa	21.053	0.324	0.055	0.621	136	0.155	72.94
T0845TS193_1-D2.rsa	20.93	0.403	0.088	0.509	144	0.145	75
T0845TS479_4-D2.rsa	20.571	0.438	0.01	0.552	223	0.092	42.88
T0845TS410_3-D2.rsa	20.571	0.525	0.01	0.465	188	0.109	51.36
T0845TS110_2-D2.rsa	20.395	0.404	0.022	0.575	262	0.078	50.49
T0845TS300_3-D2.rsa	20.37	0.454	0.088	0.458	209	0.097	72.74
T0845TS381_4-D2.rsa	20.339	0.009	0.238	0.752	161	0.126	64.25
T0845TS381_2-D2.rsa	20	0.502	0.005	0.493	199	0.101	9.96
T0845TS410_5-D2.rsa	19.767	0.346	0.113	0.541	153	0.129	77.03
T0845TS237_1-D2.rsa	18.644	0.056	0.229	0.715	153	0.122	54.09
T0845TS345_3-D2.rsa	18.605	0.396	0.106	0.498	141	0.132	79.59
T0845TS335_1-D2.rsa	18.605	0.399	0.113	0.488	138	0.135	77.56
T0845TS454_1-D2.rsa	18.605	0.389	0.124	0.488	138	0.135	83.13
T0845TS300_1-D2.rsa	18.519	0.417	0.088	0.496	226	0.082	73.56
T0845TS110_3-D2.rsa	18.462	0.258	0.214	0.528	242	0.076	52.61
T0845TS156_5-D2.rsa	17.901	0.436	0.099	0.465	212	0.084	70.92
T0845TS110_4-D2.rsa	17.692	0.255	0.192	0.552	253	0.07	70.83
T0845TS448_1-D2.rsa	17.105	0.355	0.123	0.521	173	0.099	99.32
T0845TS300_5-D2.rsa	16.923	0.255	0.229	0.515	236	0.072	76

T0845TS263_3-D2.rsa	15.385	0.26	0.225	0.515	236	0.065	78.28
T0845TS210_4-D2.rsa	15.385	0.262	0.216	0.522	239	0.064	73.72
T0845TS212_1-D2.rsa	15.116	0.385	0.12	0.495	140	0.108	79.33
T0845TS346_1-D2.rsa	14.894	0.292	0.109	0.599	154	0.097	74.22
T0845TS110_1-D2.rsa	14.615	0.264	0.236	0.5	229	0.064	78.39
T0845TS263_4-D2.rsa	14.474	0.319	0.105	0.575	191	0.076	98.95
T0845TS210_5-D2.rsa	14.474	0.349	0.123	0.527	175	0.083	98.64
T0845TS349_3-D2.rsa	13.158	0.313	0.111	0.575	191	0.069	93.83
T0845TS228_2-D2.rsa	13.158	0.334	0.117	0.548	182	0.072	98.64
T0845TS160_2-D2.rsa	13.158	0.352	0.123	0.524	174	0.076	98.95
T0845TS133_4-D2.rsa	12.766	0.327	0.167	0.506	130	0.098	78.41
T0845TS268_3-D2.rsa	12.766	0.35	0.183	0.467	120	0.106	78.11
T0845TS345_2-D2.rsa	11.842	0.334	0.123	0.542	180	0.066	99.32
T0845TS345_1-D2.rsa	11.842	0.322	0.117	0.56	186	0.064	98.27
T0845TS156_2-D2.rsa	9.211	0.334	0.117	0.548	182	0.051	98.34
T0835TS228_5-D1.rsa	94.737	0.045	0	0.955	106	0.894	10.59
T0835TS067_3-D1.rsa	92.105	0.216	0	0.784	87	1.059	14.19
T0835TS251_5-D1.rsa	79.412	0.373	0	0.627	79	1.005	26.79
T0835TS155_1-D1.rsa	76.471	0.333	0	0.667	84	0.91	14.09
T0835TS197_2-D1.rsa	76.471	0.302	0	0.698	88	0.869	16.67
T0835TS410_2-D1.rsa	75	0.111	0.148	0.741	80	0.938	30.32
T0835TS317_5-D1.rsa	73.077	0.327	0.012	0.661	109	0.67	11.55
T0835TS436_5-D1.rsa	72.093	0.306	0	0.694	93	0.775	21.45
T0835TS145_3-D1.rsa	71.875	0.382	0.015	0.603	82	0.877	11.77
T0835TS160_5-D1.rsa	70.833	0	0.528	0.472	51	1.389	59.26
T0835TS204_5-D1.rsa	70.588	0.27	0	0.73	92	0.767	29.96
T0835TS333_4-D1.rsa	70.423	0.464	0.036	0.5	84	0.838	20.51
T0835TS216_3-D1.rsa	68.421	0.279	0.036	0.685	76	0.9	23.87
T0835TS479_1-D1.rsa	67.857	0	0.229	0.771	101	0.672	26.14
T0835TS445_2-D1.rsa	67.857	0.282	0.038	0.679	89	0.762	12.6
T0835TS042_3-D1.rsa	67.647	0.532	0	0.468	59	1.147	33.73
T0835TS358_1-D1.rsa	67.647	0.571	0.04	0.389	49	1.381	33.53
T0835TS368_4-D1.rsa	67.308	0.261	0.109	0.63	104	0.647	17.41
T0835TS169_3-D1.rsa	64.706	0.548	0	0.452	57	1.135	30.95
T0835TS347_5-D1.rsa	64.286	0	0.359	0.641	84	0.765	56.87
T0835TS452_3-D1.rsa	62.791	0.149	0	0.851	114	0.551	17.72
T0835TS290_4-D1.rsa	62.5	0	0.611	0.389	42	1.488	63.89
T0835TS173_3-D1.rsa	61.765	0.437	0	0.563	71	0.87	26.59
T0835TS300_2-D1.rsa	61.538	0.291	0.091	0.618	102	0.603	50.48
T0835TS032_2-D1.rsa	60.465	0.194	0.067	0.739	99	0.611	40.48
T0835TS425_3-D1.rsa	60	0.014	0.051	0.935	129	0.465	12.32
T0835TS347_2-D1.rsa	60	0.087	0.283	0.63	87	0.69	15.58
T0835TS345_5-D1.rsa	59.375	0.074	0.199	0.728	99	0.6	11.95
T0835TS358_2-D1.rsa	59.155	0.399	0.119	0.482	81	0.73	21.31
T0835TS486_1-D1.rsa	58.333	0	0.676	0.324	35	1.667	72.69
T0835TS322_2-D1.rsa	58.333	0.037	0.361	0.602	65	0.897	53.47
T0835TS391_2-D1.rsa	58.333	0.019	0.676	0.306	33	1.768	70.14

T0835TS436_1-D1.rsa	58.333	0	0.454	0.546	59	0.989	59.72
T0835TS296_2-D1.rsa	58.333	0	0.611	0.389	42	1.389	67.36
T0835TS277_5-D1.rsa	58.14	0.284	0.172	0.545	73	0.796	18.66
T0835TS011_5-D1.rsa	57.895	0.099	0	0.901	100	0.579	18.02
T0835TS204_1-D1.rsa	57.895	0.252	0.045	0.703	78	0.742	19.59
T0835TS425_1-D1.rsa	57.895	0.243	0.054	0.703	78	0.742	21.85
T0835TS276_4-D1.rsa	57.895	0.234	0.171	0.595	66	0.877	29.5
T0835TS479_4-D1.rsa	57.143	0.015	0.427	0.557	73	0.783	58.78
T0835TS290_3-D1.rsa	57.143	0.238	0.016	0.746	94	0.608	17.66
T0835TS368_2-D1.rsa	55.882	0.508	0	0.492	62	0.901	33.33
T0835TS347_1-D1.rsa	55.814	0.448	0.09	0.463	62	0.9	37.13
T0835TS011_4-D1.rsa	55.263	0.297	0.054	0.649	72	0.768	33.78
T0835TS042_4-D1.rsa	55.263	0.306	0.09	0.604	67	0.825	36.94
T0835TS038_3-D1.rsa	55	0	0.333	0.667	76	0.724	32.46
T0835TS251_4-D1.rsa	54.93	0.381	0.071	0.548	92	0.597	28.05
T0835TS197_5-D1.rsa	54.93	0.268	0.131	0.601	101	0.544	13.3
T0835TS317_1-D1.rsa	54.93	0.458	0	0.542	91	0.604	19.71
T0835TS452_2-D1.rsa	54.167	0	0.361	0.639	69	0.785	55.32
T0835TS263_4-D1.rsa	54.167	0.019	0.556	0.426	46	1.178	68.29
T0835TS169_2-D1.rsa	54.167	0	0.565	0.435	47	1.152	51.62
T0835TS008_5-D1.rsa	53.846	0.212	0.048	0.739	122	0.441	16.14
T0835TS133_3-D1.rsa	53.846	0.285	0.139	0.576	95	0.567	16.46
T0835TS008_1-D1.rsa	53.571	0.015	0.229	0.756	99	0.541	14.5
T0835TS184_4-D1.rsa	53.571	0.084	0.237	0.679	89	0.602	17.18
T0835TS483_1-D1.rsa	53.571	0.294	0.111	0.595	75	0.714	21.23
T0835TS032_1-D1.rsa	52.5	0	0.07	0.93	106	0.495	28.51
T0835TS310_2-D1.rsa	52.113	0.375	0	0.625	105	0.496	14.1
T0835TS237_1-D1.rsa	51.923	0.291	0.085	0.624	103	0.504	58.54
T0835TS173_2-D1.rsa	51.923	0.291	0.152	0.558	92	0.564	14.4
T0835TS080_1-D1.rsa	51.786	0.254	0.032	0.714	90	0.575	16.47
T0835TS414_1-D1.rsa	51.563	0.081	0.029	0.89	121	0.426	12.87
T0835TS452_1-D1.rsa	50.704	0	0.208	0.792	133	0.381	11.7
T0835TS381_1-D1.rsa	50	0.015	0.405	0.58	76	0.658	58.97
T0835TS184_2-D1.rsa	50	0.225	0.108	0.667	74	0.676	38.96
T0835TS358_4-D1.rsa	50	0.037	0.454	0.509	55	0.909	59.26
T0835TS118_3-D1.rsa	50	0.018	0.377	0.605	69	0.725	33.77
T0835TS430_1-D1.rsa	50	0	0.366	0.634	83	0.602	17.56
T0835TS216_1-D1.rsa	50	0.238	0.127	0.635	80	0.625	22.22
T0835TS338_1-D1.rsa	50	0.135	0.153	0.712	79	0.633	20.95
T0835TS335_3-D1.rsa	48.077	0.242	0.085	0.673	111	0.433	46.99
T0835TS417_3-D1.rsa	48.077	0.279	0.048	0.673	111	0.433	48.89
T0835TS310_3-D1.rsa	48.077	0	0.418	0.582	96	0.501	11.08
T0835TS381_5-D1.rsa	48	0.043	0.072	0.884	122	0.393	32.79
T0835TS439_4-D1.rsa	47.887	0.375	0.036	0.589	99	0.484	16.03
T0835TS204_2-D1.rsa	47.5	0	0.281	0.719	82	0.579	16.67
T0835TS118_4-D1.rsa	46.552	0.035	0.217	0.748	107	0.435	30.07
T0835TS282_5-D1.rsa	46.479	0.405	0	0.595	100	0.465	39.74

T0835TS156_5-D1.rsa	46.429	0	0.168	0.832	109	0.426	58.4
T0835TS067_2-D1.rsa	46.429	0.151	0.063	0.786	99	0.469	18.85
T0835TS454_2-D1.rsa	46.429	0.254	0.063	0.683	86	0.54	20.24
T0835TS097_1-D1.rsa	46.154	0.315	0.097	0.588	97	0.476	55.06
T0835TS326_4-D1.rsa	46.154	0.218	0.024	0.758	125	0.369	21.04
T0835TS322_5-D1.rsa	45.833	0	0.309	0.691	76	0.603	44.77
T0835TS064_3-D1.rsa	45	0	0.211	0.789	90	0.5	16.23
T0835TS358_3-D1.rsa	44.737	0.216	0.126	0.658	73	0.613	33.11
T0835TS328_4-D1.rsa	44.737	0.279	0.108	0.613	68	0.658	38.51
T0835TS184_3-D1.rsa	44.643	0.159	0.23	0.611	77	0.58	49.8
T0835TS277_1-D1.rsa	44.643	0.087	0.135	0.778	98	0.456	14.48
T0835TS414_2-D1.rsa	44.231	0.248	0.073	0.679	112	0.395	46.99
T0835TS436_4-D1.rsa	44.186	0.134	0.082	0.784	105	0.421	27.24
T0835TS155_3-D1.rsa	44	0.029	0.21	0.761	105	0.419	14.86
T0835TS445_3-D1.rsa	43.75	0	0.331	0.669	91	0.481	13.23
T0835TS237_5-D1.rsa	43.662	0.107	0	0.893	150	0.291	15.71
T0835TS160_4-D1.rsa	42.857	0.071	0.063	0.865	109	0.393	18.45
T0835TS445_5-D1.rsa	42.857	0.143	0.071	0.786	99	0.433	20.04
T0835TS067_5-D1.rsa	42.857	0.183	0.198	0.619	78	0.549	20.83
T0835TS044_3-D1.rsa	42.5	0.061	0.333	0.605	69	0.616	28.07
T0835TS228_3-D1.rsa	42.308	0.248	0.061	0.691	114	0.371	46.68
T0835TS420_1-D1.rsa	42.308	0.364	0.036	0.6	99	0.427	48.58
T0835TS276_2-D1.rsa	42.254	0.387	0.119	0.494	83	0.509	22.76
T0835TS132_3-D1.rsa	42.188	0.059	0.235	0.706	96	0.439	16.18
T0835TS118_2-D1.rsa	42.105	0.259	0.361	0.38	41	1.027	72.45
T0835TS328_5-D1.rsa	42.105	0.297	0	0.703	78	0.54	28.83
T0835TS038_2-D1.rsa	41.86	0.351	0.03	0.619	83	0.504	42.72
T0835TS044_4-D1.rsa	41.86	0.328	0.097	0.575	77	0.544	39.74
T0835TS156_2-D1.rsa	41.86	0.373	0.06	0.567	76	0.551	40.67
T0835TS145_1-D1.rsa	41.86	0.149	0.149	0.701	94	0.445	18.28
T0835TS080_2-D1.rsa	41.667	0.019	0.574	0.407	44	0.947	72.45
T0835TS483_2-D1.rsa	41.667	0	0.382	0.618	68	0.613	30.45
T0835TS153_5-D1.rsa	41.379	0.021	0.07	0.909	130	0.318	47.03
T0835TS006_3-D1.rsa	41.379	0	0.098	0.902	129	0.321	44.58
T0835TS345_1-D1.rsa	41.379	0.035	0.133	0.832	119	0.348	29.72
T0835TS006_2-D1.rsa	41.228	0.258	0.18	0.562	122	0.338	61.98
T0835TS133_5-D1.rsa	41.071	0.095	0.087	0.817	103	0.399	19.44
T0835TS097_3-D1.rsa	41.071	0.214	0.048	0.738	93	0.442	19.25
T0835TS492_5-D1.rsa	40.845	0.024	0.179	0.798	134	0.305	12.98
T0835TS157_5-D1.rsa	40.678	0.056	0.308	0.636	136	0.299	29.21
T0835TS064_1-D1.rsa	40.625	0.176	0.235	0.588	80	0.508	13.42
T0835TS064_5-D1.rsa	40.625	0.154	0.14	0.706	96	0.423	11.03
T0835TS133_4-D1.rsa	40	0.145	0.101	0.754	104	0.385	18.3
T0835TS044_1-D1.rsa	40	0	0.351	0.649	74	0.541	19.96
T0835TS171_5-D1.rsa	39.773	0.037	0.446	0.517	153	0.26	33.7
T0835TS335_2-D1.rsa	39.655	0.59	0	0.41	105	0.378	54.2
T0835TS445_4-D1.rsa	39.535	0.313	0.06	0.627	84	0.471	51.31

T0835TS197_1-D1.rsa	39.535	0.276	0.067	0.657	88	0.449	24.07
T0835TS340_1-D1.rsa	39.535	0.261	0.082	0.657	88	0.449	24.63
T0835TS169_5-D1.rsa	39.535	0.284	0	0.716	96	0.412	21.27
T0835TS184_1-D1.rsa	39.535	0.343	0.142	0.515	69	0.573	49.81
T0835TS049_5-D1.rsa	39.474	0.263	0.212	0.525	114	0.346	65.55
T0835TS340_2-D1.rsa	39.474	0.244	0.189	0.567	123	0.321	62.79
T0835TS345_3-D1.rsa	39.474	0.045	0	0.955	106	0.372	22.52
T0835TS322_4-D1.rsa	39.474	0	0.018	0.982	109	0.362	23.65
T0835TS268_5-D1.rsa	39.474	0.297	0.081	0.622	69	0.572	40.77
T0835TS425_4-D1.rsa	39.437	0.411	0.143	0.446	75	0.526	40.87
T0835TS032_5-D1.rsa	39.286	0	0.015	0.985	129	0.305	16.98
T0835TS483_3-D1.rsa	39.286	0	0.145	0.855	112	0.351	20.23
T0835TS358_5-D1.rsa	39.286	0	0.389	0.611	80	0.491	58.21
T0835TS042_2-D1.rsa	39.286	0	0.382	0.618	81	0.485	56.87
T0835TS290_2-D1.rsa	39.286	0.175	0.27	0.556	70	0.561	50.59
T0835TS322_3-D1.rsa	39.063	0.015	0.309	0.676	92	0.425	64.34
T0835TS326_5-D1.rsa	39.063	0.015	0.346	0.64	87	0.449	71.69
T0835TS335_4-D1.rsa	38.947	0.517	0.038	0.445	105	0.371	58.26
T0835TS235_4-D1.rsa	38.889	0.408	0.089	0.502	107	0.363	74.49
T0835TS479_3-D1.rsa	38.889	0.432	0.085	0.484	103	0.378	71.83
T0835TS381_3-D1.rsa	38.636	0.064	0.459	0.476	141	0.274	34.21
T0835TS346_1-D1.rsa	38.636	0.078	0.51	0.412	122	0.317	40.12
T0835TS420_5-D1.rsa	38.356	0.745	0	0.255	65	0.59	58.23
T0835TS145_2-D1.rsa	38.356	0.753	0	0.247	63	0.609	49.71
T0835TS263_5-D1.rsa	38.028	0.161	0.012	0.827	139	0.274	15.71
T0835TS282_4-D1.rsa	37.931	0.586	0	0.414	106	0.358	53.12
T0835TS032_3-D1.rsa	37.895	0.547	0.038	0.415	98	0.387	59.11
T0835TS160_1-D1.rsa	37.895	0.568	0.059	0.373	88	0.431	51.06
T0835TS277_3-D1.rsa	37.719	0.263	0.161	0.576	125	0.302	51.61
T0835TS235_3-D1.rsa	37.719	0.318	0.147	0.535	116	0.325	51.84
T0835TS391_4-D1.rsa	37.5	0.39	0.085	0.526	112	0.335	71.07
T0835TS290_5-D1.rsa	37.5	0.315	0.099	0.587	125	0.3	59.39
T0835TS026_1-D1.rsa	37.5	0.413	0.085	0.502	107	0.35	71.19
T0835TS414_4-D1.rsa	37.5	0.399	0.085	0.516	110	0.341	65.36
T0835TS155_5-D1.rsa	37.5	0.055	0.291	0.655	72	0.521	51.82
T0835TS065_1-D1.rsa	37.5	0.127	0.31	0.563	71	0.528	43.25
T0835TS118_5-D1.rsa	37.5	0.175	0.23	0.595	75	0.5	47.82
T0835TS132_4-D1.rsa	37.5	0	0.518	0.482	53	0.708	62.27
T0835TS282_3-D1.rsa	37.209	0.269	0.03	0.701	94	0.396	42.54
T0835TS340_3-D1.rsa	37.209	0.299	0.03	0.672	90	0.413	42.91
T0835TS403_3-D1.rsa	37.209	0.343	0.09	0.567	76	0.49	42.72
T0835TS173_5-D1.rsa	37.209	0.269	0.127	0.604	81	0.459	22.02
T0835TS184_5-D1.rsa	37.209	0.381	0.045	0.575	77	0.483	26.31
T0835TS144_1-D1.rsa	36.986	0.749	0	0.251	64	0.578	66.28
T0835TS296_1-D1.rsa	36.986	0.733	0	0.267	68	0.544	65.59
T0835TS116_5-D1.rsa	36.842	0.479	0.059	0.462	109	0.338	61.44
T0835TS133_2-D1.rsa	36.842	0.429	0.129	0.442	96	0.384	39.05

T0835TS349_2-D1.rsa	36.842	0.241	0.5	0.259	28	1.316	93.06
T0835TS049_3-D1.rsa	36.571	0.574	0.025	0.401	162	0.226	12.44
T0835TS049_4-D1.rsa	36.364	0.091	0.493	0.416	123	0.296	45.61
T0835TS128_2-D1.rsa	36.207	0	0.161	0.839	120	0.302	80.25
T0835TS425_5-D1.rsa	36.207	0	0.14	0.86	123	0.294	54.72
T0835TS414_5-D1.rsa	36.111	0.46	0.094	0.446	95	0.38	75.89
T0835TS335_5-D1.rsa	36.111	0.366	0.085	0.549	117	0.309	59.52
T0835TS228_1-D1.rsa	35.938	0.096	0.294	0.61	83	0.433	63.23
T0835TS117_3-D1.rsa	35.789	0.462	0.059	0.479	113	0.317	55.3
T0835TS454_5-D1.rsa	35.616	0.722	0	0.278	71	0.502	41.27
T0835TS204_3-D1.rsa	35.417	0	0.055	0.945	104	0.341	25
T0835TS347_4-D1.rsa	35.417	0	0.291	0.709	78	0.454	62.05
T0835TS042_1-D1.rsa	35.417	0.045	0.336	0.618	68	0.521	62.05
T0835TS169_4-D1.rsa	35	0	0.456	0.544	62	0.565	47.37
T0835TS347_3-D1.rsa	35	0.018	0.307	0.675	77	0.455	42.33
T0835TS044_2-D1.rsa	35	0	0.149	0.851	97	0.361	14.47
T0835TS011_3-D1.rsa	34.884	0.231	0.06	0.709	95	0.367	22.2
T0835TS263_1-D1.rsa	34.737	0.517	0.055	0.428	101	0.344	56.46
T0835TS492_4-D1.rsa	34.737	0.517	0.059	0.424	100	0.347	63.77
T0835TS251_3-D1.rsa	34.483	0.035	0.168	0.797	114	0.302	52.27
T0835TS162_3-D1.rsa	34.483	0.582	0	0.418	107	0.322	56.05
T0835TS410_5-D1.rsa	34.483	0.602	0	0.398	102	0.338	56.54
T0835TS296_3-D1.rsa	34.375	0.176	0.051	0.772	105	0.327	13.42
T0835TS216_4-D1.rsa	34.375	0.096	0.074	0.831	113	0.304	16.18
T0835TS499_2-D1.rsa	34.375	0.059	0.059	0.882	120	0.286	11.95
T0835TS317_4-D1.rsa	34.247	0.757	0	0.243	62	0.552	55.1
T0835TS197_4-D1.rsa	34.211	0.25	0.435	0.315	34	1.006	93.75
T0835TS145_5-D1.rsa	34.211	0.25	0.491	0.259	28	1.222	94.21
T0835TS338_4-D1.rsa	34.211	0.278	0.454	0.269	29	1.18	93.29
T0835TS454_4-D1.rsa	34.211	0.25	0.5	0.25	27	1.267	93.98
T0835TS492_3-D1.rsa	33.929	0.151	0.151	0.698	88	0.386	41.27
T0835TS282_2-D1.rsa	33.929	0.135	0.317	0.548	69	0.492	52.18
T0835TS276_5-D1.rsa	33.803	0.494	0.107	0.399	67	0.505	50.64
T0835TS116_3-D1.rsa	33.333	0.028	0.007	0.965	278	0.12	7.03
T0835TS417_2-D1.rsa	33.333	0.305	0.103	0.592	126	0.265	65.74
T0835TS128_3-D1.rsa	32.955	0.007	0.172	0.821	243	0.136	6.84
T0835TS128_1-D1.rsa	32.955	0.064	0.307	0.628	186	0.177	40.79
T0835TS349_4-D1.rsa	32.877	0.737	0	0.263	67	0.491	59.51
T0835TS499_5-D1.rsa	32.813	0.015	0.441	0.544	74	0.443	72.43
T0835TS268_4-D1.rsa	32.813	0.015	0.382	0.603	82	0.4	45.77
T0835TS436_2-D1.rsa	32.787	0.01	0.405	0.586	123	0.267	63.18
T0835TS204_4-D1.rsa	32.759	0.555	0	0.445	114	0.287	52.44
T0835TS212_1-D1.rsa	32.759	0.59	0	0.41	105	0.312	56.25
T0835TS454_1-D1.rsa	32.692	0.23	0.012	0.758	125	0.262	12.5
T0835TS439_5-D1.rsa	32.558	0.302	0.148	0.55	93	0.35	72.48
T0835TS156_3-D1.rsa	32.558	0.246	0.06	0.694	93	0.35	24.25
T0835TS160_2-D1.rsa	32.558	0.216	0.157	0.627	84	0.388	19.4

T0835TS282_1-D1.rsa	32.5	0	0.096	0.904	103	0.316	49.12
T0835TS144_2-D1.rsa	32.5	0	0.333	0.667	76	0.428	48.9
T0835TS310_4-D1.rsa	32.5	0	0.211	0.789	90	0.361	33.33
T0835TS328_1-D1.rsa	32.5	0.026	0.246	0.728	83	0.392	19.52
T0835TS268_1-D1.rsa	32.143	0.183	0.27	0.548	69	0.466	50
T0835TS032_4-D1.rsa	32	0.043	0.319	0.638	88	0.364	55.98
T0835TS345_4-D1.rsa	32	0.08	0.326	0.594	82	0.39	53.99
T0835TS391_1-D1.rsa	32	0.087	0.362	0.551	76	0.421	66.12
T0835TS290_1-D1.rsa	32	0.094	0	0.906	125	0.256	14.49
T0835TS333_5-D1.rsa	31.897	0.699	0	0.301	77	0.414	60.74
T0835TS251_2-D1.rsa	31.579	0.581	0.025	0.394	93	0.34	55.3
T0835TS080_4-D1.rsa	31.579	0.241	0.491	0.269	29	1.089	93.98
T0835TS276_1-D1.rsa	31.579	0.241	0.5	0.259	28	1.128	93.06
T0835TS153_3-D1.rsa	31.507	0.402	0.1	0.499	185	0.17	75.82
T0835TS414_3-D1.rsa	31.429	0.346	0.218	0.436	102	0.308	70.94
T0835TS410_1-D1.rsa	31.25	0.015	0.419	0.566	77	0.406	68.57
T0835TS044_5-D1.rsa	31.25	0.015	0.426	0.559	76	0.411	74.08
T0835TS008_3-D1.rsa	31.034	0.613	0	0.387	99	0.313	56.74
T0835TS038_1-D1.rsa	31.034	0.613	0	0.387	99	0.313	55.27
T0835TS160_3-D1.rsa	31.034	0.715	0	0.285	73	0.425	62.79
T0835TS333_3-D1.rsa	30.986	0.464	0.161	0.375	63	0.492	63.14
T0835TS483_5-D1.rsa	30.986	0.506	0.071	0.423	71	0.436	53.37
T0835TS064_4-D1.rsa	30.986	0.494	0.107	0.399	67	0.462	50.96
T0835TS381_4-D1.rsa	30.702	0.276	0.221	0.502	109	0.282	70.16
T0835TS162_5-D1.rsa	30.702	0.3	0.171	0.53	115	0.267	60.14
T0835TS326_2-D1.rsa	30.556	0.385	0.075	0.54	115	0.266	65.61
T0835TS237_2-D1.rsa	30.4	0.024	0.41	0.565	186	0.163	50.46
T0835TS268_2-D1.rsa	30.357	0.159	0.04	0.802	101	0.301	31.15
T0835TS197_3-D1.rsa	30.357	0.206	0.127	0.667	84	0.361	29.96
T0835TS349_5-D1.rsa	30.357	0.183	0.357	0.46	58	0.523	54.37
T0835TS328_3-D1.rsa	30.357	0.183	0.206	0.611	77	0.394	26.98
T0835TS023_1-D1.rsa	30.233	0.32	0.219	0.462	78	0.388	72.48
T0835TS042_5-D1.rsa	30.233	0.355	0.13	0.515	87	0.348	68.49
T0835TS080_5-D1.rsa	30.233	0.358	0.067	0.575	77	0.393	52.8
T0835TS067_4-D1.rsa	30.137	0.71	0	0.29	74	0.407	39.41
T0835TS144_5-D1.rsa	30	0	0.36	0.64	73	0.411	48.9
T0835TS097_5-D1.rsa	30	0	0.36	0.64	73	0.411	48.03
T0835TS333_1-D1.rsa	30	0	0.482	0.518	59	0.508	48.68
T0835TS157_1-D1.rsa	29.87	0.397	0.121	0.482	108	0.277	79.05
T0835TS310_1-D1.rsa	29.688	0.118	0.162	0.721	98	0.303	13.97
T0835TS333_2-D1.rsa	29.577	0.47	0.161	0.369	62	0.477	62.82
T0835TS041_3-D1.rsa	29.524	0.368	0.179	0.453	106	0.279	70.62
T0835TS263_3-D1.rsa	29.474	0.53	0.059	0.411	97	0.304	51.38
T0835TS479_2-D1.rsa	29.412	0.395	0.091	0.514	125	0.235	66.44
T0835TS439_2-D1.rsa	29.412	0.358	0.025	0.617	150	0.196	13
T0835TS410_3-D1.rsa	29.07	0.308	0.189	0.503	85	0.342	69.08
T0835TS439_3-D1.rsa	28.947	0.29	0.217	0.493	107	0.271	62.67

T0835TS338_5-D1.rsa	28.947	0.241	0.5	0.259	28	1.034	94.44
T0835TS326_1-D1.rsa	28.571	0.342	0.201	0.457	107	0.267	71.58
T0835TS133_1-D1.rsa	28.571	0.046	0.137	0.817	107	0.267	15.46
T0835TS145_4-D1.rsa	28.571	0.175	0.302	0.524	66	0.433	38.89
T0835TS486_2-D1.rsa	28.07	0.219	0.096	0.685	150	0.187	18.95
T0835TS006_4-D1.rsa	28	0.466	0.068	0.466	117	0.239	87.95
T0835TS162_1-D1.rsa	28	0.014	0.225	0.761	105	0.267	53.8
T0835TS117_4-D1.rsa	28	0.036	0.333	0.63	87	0.322	71.56
T0835TS499_4-D1.rsa	28	0.072	0.377	0.551	76	0.368	68.66
T0835TS251_1-D1.rsa	28	0.072	0.348	0.58	80	0.35	59.06
T0835TS050_1-D1.rsa	28	0.072	0.326	0.601	83	0.337	53.99
T0835TS300_3-D1.rsa	27.907	0.32	0.16	0.521	88	0.317	73.37
T0835TS118_1-D1.rsa	27.907	0.325	0.201	0.473	80	0.349	72.04
T0835TS322_1-D1.rsa	27.907	0.367	0.178	0.456	77	0.362	72.19
T0835TS216_5-D1.rsa	27.907	0.367	0.213	0.42	71	0.393	69.67
T0835TS420_4-D1.rsa	27.619	0.338	0.184	0.479	112	0.247	67.63
T0835TS038_4-D1.rsa	27.619	0.389	0.205	0.406	95	0.291	72.76
T0835TS403_1-D1.rsa	27.586	0.695	0	0.305	78	0.354	61.43
T0835TS276_3-D1.rsa	27.5	0.018	0.386	0.596	68	0.404	46.27
T0835TS403_5-D1.rsa	27.5	0	0.263	0.737	84	0.327	45.83
T0835TS155_4-D1.rsa	27.5	0.044	0.368	0.588	67	0.41	46.49
T0835TS153_1-D1.rsa	27.273	0.205	0.146	0.649	187	0.146	53.99
T0835TS063_2-D1.rsa	27.273	0.208	0.222	0.569	164	0.166	68.66
T0835TS442_2-D1.rsa	27.211	0.287	0.064	0.649	242	0.112	40.83
T0835TS300_5-D1.rsa	27	0.442	0.12	0.438	110	0.245	81.28
T0835TS063_4-D1.rsa	26.923	0.365	0.108	0.527	156	0.173	53.38
T0835TS210_1-D1.rsa	26.744	0.302	0.183	0.515	87	0.307	72.78
T0835TS349_3-D1.rsa	26.744	0.325	0.201	0.473	80	0.334	70.56
T0835TS041_2-D1.rsa	26.471	0.457	0.107	0.436	106	0.25	67.56
T0835TS116_1-D1.rsa	26.316	0.265	0.093	0.642	213	0.124	50.75
T0835TS296_5-D1.rsa	25.974	0.379	0.143	0.478	107	0.243	70.71
T0835TS436_3-D1.rsa	25.641	0.348	0.111	0.541	160	0.16	57.03
T0835TS300_4-D1.rsa	25.581	0.26	0.172	0.568	96	0.266	70.71
T0835TS403_4-D1.rsa	25.581	0.361	0.189	0.45	76	0.337	69.82
T0835TS171_1-D1.rsa	25	0.35	0.095	0.556	135	0.185	68.67
T0835TS237_4-D1.rsa	25	0.438	0.12	0.442	111	0.225	88.45
T0835TS479_5-D1.rsa	25	0.383	0.095	0.523	127	0.197	71.11
T0835TS157_4-D1.rsa	25	0.383	0.103	0.514	125	0.2	67.22
T0835TS499_3-D1.rsa	25	0.42	0.107	0.473	115	0.217	66.78
T0835TS483_4-D1.rsa	25	0.466	0.124	0.41	103	0.243	86.75
T0835TS144_4-D1.rsa	25	0.19	0	0.81	102	0.245	42.66
T0835TS430_2-D1.rsa	25	0	0.184	0.816	93	0.269	39.69
T0835TS349_1-D1.rsa	25	0	0.184	0.816	93	0.269	39.69
T0835TS326_3-D1.rsa	24.675	0.402	0.098	0.5	112	0.22	69.64
T0835TS128_4-D1.rsa	24.658	0.404	0.113	0.482	179	0.138	79.7
T0835TS452_5-D1.rsa	24.59	0.038	0.438	0.524	110	0.224	66.29
T0835TS008_4-D1.rsa	24.419	0.343	0.172	0.485	82	0.298	73.08

T0835TS357_1-D1.rsa	24.359	0.409	0.115	0.476	141	0.173	59.63
T0835TS038_5-D1.rsa	24	0	0.043	0.957	132	0.182	17.21
T0835TS317_2-D1.rsa	24	0.014	0.377	0.609	84	0.286	64.13
T0835TS439_1-D1.rsa	24	0.036	0.326	0.638	88	0.273	58.15
T0835TS228_2-D1.rsa	24	0.087	0.319	0.594	82	0.293	68.12
T0835TS420_3-D1.rsa	24	0.08	0.362	0.558	77	0.312	57.06
T0835TS132_1-D1.rsa	24	0.13	0.152	0.717	99	0.242	22.46
T0835TS171_3-D1.rsa	23.729	0.009	0.206	0.785	168	0.141	42.29
T0835TS417_1-D1.rsa	23.529	0.37	0.111	0.519	126	0.187	65.33
T0835TS049_2-D1.rsa	23.529	0.366	0.099	0.535	130	0.181	67.22
T0835TS162_4-D1.rsa	23.377	0.415	0.125	0.46	103	0.227	77.26
T0835TS228_4-D1.rsa	23.377	0.406	0.129	0.464	104	0.225	78.81
T0835TS067_1-D1.rsa	23.256	0.296	0.178	0.527	89	0.261	70.12
T0835TS237_3-D1.rsa	23.077	0.361	0.139	0.5	148	0.156	56.68
T0835TS445_1-D1.rsa	22.5	0	0.158	0.842	96	0.234	44.96
T0835TS171_4-D1.rsa	22.059	0.379	0.062	0.56	136	0.162	68.44
T0835TS381_2-D1.rsa	22.059	0.346	0.103	0.551	134	0.165	60
T0835TS156_1-D1.rsa	22	0.45	0.124	0.426	107	0.206	87.25
T0835TS277_4-D1.rsa	21.795	0.453	0.128	0.419	124	0.176	62.15
T0835TS063_5-D1.rsa	21.605	0.434	0.081	0.485	221	0.098	31.24
T0835TS116_2-D1.rsa	21.053	0.265	0.093	0.642	213	0.099	49.92
T0835TS157_3-D1.rsa	21.053	0.393	0.078	0.53	116	0.181	69.29
T0835TS049_1-D1.rsa	21.053	0.429	0.096	0.475	104	0.202	81.16
T0835TS442_3-D1.rsa	20.988	0.399	0.077	0.524	239	0.088	67.05
T0835TS442_4-D1.rsa	20.988	0.414	0.086	0.5	228	0.092	75.5
T0835TS097_4-D1.rsa	20	0.065	0.333	0.601	83	0.241	66.49
T0835TS132_2-D1.rsa	20	0.029	0.304	0.667	92	0.217	45.65
T0835TS153_2-D1.rsa	19.753	0.432	0.077	0.491	224	0.088	72.57
T0835TS117_1-D1.rsa	19.481	0.353	0.138	0.509	114	0.171	75.24
T0835TS041_4-D1.rsa	19.079	0.436	0.024	0.539	246	0.078	60.36
T0835TS442_1-D1.rsa	19.079	0.445	0.015	0.539	246	0.078	54.55
T0835TS041_1-D1.rsa	18.182	0.362	0.085	0.554	124	0.147	68.21
T0835TS442_5-D1.rsa	17.692	0.26	0.225	0.515	236	0.075	74.11
T0835TS162_2-D1.rsa	17.544	0.443	0.082	0.475	104	0.169	80.59
T0835TS279_1-D1.rsa	17.544	0.443	0.087	0.47	103	0.17	77.97
T0835TS006_5-D1.rsa	17.442	0.371	0.113	0.516	146	0.119	70.94
T0835TS212_2-D1.rsa	14.035	0.457	0.078	0.466	102	0.138	80.94
T0835TS117_2-D1.rsa	13.846	0.258	0.221	0.522	239	0.058	77.72
T0835TS157_2-D1.rsa	12.766	0.327	0.175	0.498	128	0.1	79.67
T0835TS454_3-D1.rsa	12	0.029	0.326	0.645	89	0.135	48.19
T0835TS171_2-D1.rsa	10.638	0.292	0.183	0.525	135	0.079	76.65
T0835TS335_1-D1.rsa	10.638	0.342	0.206	0.451	116	0.092	84.53
T0794TS391_1-D1.rsa	90.141	0.357	0	0.643	108	0.835	17.15
T0794TS290_4-D1.rsa	79.412	0.254	0	0.746	94	0.845	29.17
T0794TS445_1-D1.rsa	78.125	0	0.118	0.882	120	0.651	39.89
T0794TS184_4-D1.rsa	76.471	0.294	0	0.706	89	0.859	32.74
T0794TS482_1-D1.rsa	76.471	0.262	0	0.738	93	0.822	29.17

T0794TS333_5-D1.rsa	75	0	0.333	0.667	72	1.042	21.53
T0794TS425_2-D1.rsa	68.421	0.189	0.018	0.793	88	0.778	36.71
T0794TS454_3-D1.rsa	68.421	0.18	0.054	0.766	85	0.805	24.77
T0794TS041_5-D1.rsa	67.442	0.201	0	0.799	107	0.63	40.3
T0794TS425_5-D1.rsa	63.158	0.18	0.036	0.784	87	0.726	36.71
T0794TS011_3-D1.rsa	62.791	0.239	0.045	0.716	96	0.654	38.81
T0794TS117_1-D1.rsa	62.791	0.239	0.045	0.716	96	0.654	39.37
T0794TS347_2-D1.rsa	62.791	0.201	0	0.799	107	0.587	36.19
T0794TS038_1-D1.rsa	62.791	0.209	0.015	0.776	104	0.604	36.57
T0794TS133_3-D1.rsa	61.538	0.291	0.139	0.57	94	0.655	74.53
T0794TS492_1-D1.rsa	61.538	0.297	0.139	0.564	93	0.662	60.6
T0794TS162_4-D1.rsa	60.714	0	0.137	0.863	113	0.537	16.6
T0794TS310_4-D1.rsa	60.465	0.231	0.06	0.709	95	0.636	41.23
T0794TS204_2-D1.rsa	59.615	0.279	0.097	0.624	103	0.579	77.06
T0794TS144_1-D1.rsa	59.615	0.297	0.085	0.618	102	0.584	60.76
T0794TS282_3-D1.rsa	59.615	0.291	0.091	0.618	102	0.584	50.48
T0794TS011_4-D1.rsa	59.615	0.273	0.073	0.655	108	0.552	53.01
T0794TS228_5-D1.rsa	59.302	0.3	0	0.7	198	0.3	8.13
T0794TS204_3-D1.rsa	57.813	0	0.206	0.794	108	0.535	40.81
T0794TS044_4-D1.rsa	57.746	0.345	0.048	0.607	102	0.566	21.47
T0794TS417_3-D1.rsa	57.692	0.242	0.103	0.655	108	0.534	77.37
T0794TS492_2-D1.rsa	57.692	0.218	0.097	0.685	113	0.511	43.04
T0794TS346_1-D1.rsa	57.692	0.255	0.145	0.6	99	0.583	49.52
T0794TS358_5-D1.rsa	57.692	0.218	0	0.782	129	0.447	15.35
T0794TS296_5-D1.rsa	55.769	0.255	0.097	0.648	107	0.521	67.25
T0794TS011_5-D1.rsa	55.769	0.267	0.097	0.636	105	0.531	69.3
T0794TS032_1-D1.rsa	55.769	0.255	0.115	0.63	104	0.536	64.87
T0794TS210_4-D1.rsa	53.846	0.279	0.073	0.648	107	0.503	72.94
T0794TS301_4-D1.rsa	53.846	0.303	0.085	0.612	101	0.533	17.09
T0794TS097_1-D1.rsa	53.571	0.061	0.168	0.771	101	0.53	14.7
T0794TS358_1-D1.rsa	53.409	0.088	0.419	0.493	146	0.366	21.96
T0794TS155_4-D1.rsa	52.632	0.265	0.018	0.717	238	0.221	10.47
T0794TS216_1-D1.rsa	52.083	0	0.309	0.691	76	0.685	45
T0794TS310_3-D1.rsa	52	0.101	0.058	0.841	116	0.448	12.68
T0794TS414_1-D1.rsa	51.923	0.261	0.073	0.667	110	0.472	74.84
T0794TS492_3-D1.rsa	51.923	0.321	0.048	0.63	104	0.499	46.36
T0794TS277_2-D1.rsa	50.505	0.271	0.118	0.611	176	0.287	16.15
T0794TS452_3-D1.rsa	50	0	0.038	0.962	126	0.397	41.79
T0794TS391_2-D1.rsa	50	0	0.015	0.985	129	0.388	15.08
T0794TS184_5-D1.rsa	50	0	0.382	0.618	81	0.617	61.45
T0794TS169_5-D1.rsa	50	0	0.229	0.771	101	0.495	16.6
T0794TS160_3-D1.rsa	49.495	0.302	0.142	0.556	160	0.309	12.24
T0794TS430_1-D1.rsa	46.286	0.532	0	0.468	189	0.245	21.29
T0794TS333_3-D1.rsa	46.154	0.061	0.164	0.776	128	0.361	10.44
T0794TS349_3-D1.rsa	46.053	0.361	0.006	0.633	210	0.219	11.37
T0794TS268_3-D1.rsa	44.828	0	0.238	0.762	109	0.411	51.22
T0794TS333_2-D1.rsa	44.762	0.376	0.158	0.466	109	0.411	44.44

T0794TS080_4-D1.rsa	44.079	0.434	0.015	0.55	251	0.176	20.39
T0794TS038_2-D1.rsa	43.158	0.475	0.042	0.483	114	0.379	57.52
T0794TS340_1-D1.rsa	43.158	0.517	0.059	0.424	100	0.432	57.2
T0794TS448_1-D1.rsa	43.103	0	0.091	0.909	130	0.332	60.84
T0794TS210_2-D1.rsa	42.857	0	0.282	0.718	94	0.456	60.5
T0794TS216_3-D1.rsa	42.857	0.015	0.076	0.908	119	0.36	20.04
T0794TS317_2_1-D1.rsa	42.857	0	0.359	0.641	84	0.51	58.4
T0794TS338_4-D1.rsa	42.857	0	0.405	0.595	78	0.549	62.21
T0794TS169_4-D1.rsa	42.857	0	0.382	0.618	81	0.529	58.02
T0794TS097_2-D1.rsa	42.254	0.333	0.107	0.56	94	0.45	42.15
T0794TS417_2-D1.rsa	42.105	0.377	0.024	0.599	199	0.212	9.04
T0794TS301_1-D1.rsa	42.045	0.105	0.372	0.524	155	0.271	21.79
T0794TS317_3_1-D1.rsa	41.86	0.321	0.015	0.664	89	0.47	41.98
T0794TS041_4-D1.rsa	41.667	0.38	0.094	0.526	112	0.372	70.94
T0794TS410_3-D1.rsa	41.667	0.39	0.08	0.531	113	0.369	69.92
T0794TS358_2-D1.rsa	41.667	0.362	0.085	0.554	118	0.353	63.07
T0794TS038_4-D1.rsa	41.667	0.39	0.085	0.526	112	0.372	68.66
T0794TS439_1-D1.rsa	41.379	0	0.112	0.888	127	0.326	57.69
T0794TS073_1-D1.rsa	41.379	0.014	0.098	0.888	127	0.326	55.94
T0794TS347_1-D1.rsa	41.379	0	0.112	0.888	127	0.326	56.47
T0794TS345_4-D1.rsa	40.845	0	0.024	0.976	164	0.249	11.7
T0794TS358_3-D1.rsa	40.845	0.25	0	0.75	126	0.324	15.38
T0794TS155_2-D1.rsa	40	0.006	0.295	0.699	230	0.174	31.91
T0794TS197_3-D1.rsa	40	0.014	0.268	0.717	99	0.404	48.01
T0794TS008_5-D1.rsa	39.773	0.074	0.432	0.493	146	0.272	27.62
T0794TS310_5-D1.rsa	39.655	0	0.147	0.853	122	0.325	56.82
T0794TS282_4-D1.rsa	39.655	0	0.126	0.874	125	0.317	59.44
T0794TS197_4-D1.rsa	39.474	0.286	0.143	0.571	124	0.318	52.99
T0794TS349_1-D1.rsa	39.394	0	0.247	0.753	217	0.182	9.72
T0794TS067_1-D1.rsa	39.286	0	0.374	0.626	82	0.479	58.02
T0794TS064_3-D1.rsa	39.063	0.015	0.279	0.706	96	0.407	63.97
T0794TS184_2-D1.rsa	39.063	0.015	0.463	0.522	71	0.55	65.62
T0794TS118_1-D1.rsa	38.889	0.352	0.094	0.554	118	0.33	68.66
T0794TS145_2-D1.rsa	38.636	0.071	0.493	0.436	129	0.3	43.33
T0794TS414_2-D1.rsa	38.596	0.24	0.171	0.59	128	0.302	63.25
T0794TS032_2_1-D1.rsa	38.596	0.309	0.147	0.544	118	0.327	57.95
T0794TS345_3-D1.rsa	38.596	0.309	0.147	0.544	118	0.327	57.83
T0794TS251_2-D1.rsa	38.384	0.24	0.174	0.587	169	0.227	54.6
T0794TS300_2-D1.rsa	37.931	0	0.154	0.846	121	0.313	70.45
T0794TS317_4_1-D1.rsa	37.931	0.598	0	0.402	103	0.368	54.98
T0794TS300_3-D1.rsa	37.719	0.244	0.184	0.571	124	0.304	61.29
T0794TS133_1-D1.rsa	37.719	0.295	0.111	0.594	129	0.292	43.55
T0794TS067_4-D1.rsa	37.5	0.305	0	0.695	148	0.253	52.54
T0794TS448_3-D1.rsa	37.5	0.071	0.459	0.47	139	0.27	48.23
T0794TS445_4-D1.rsa	37.5	0.362	0.085	0.554	118	0.318	67.13
T0794TS067_2-D1.rsa	37.5	0.39	0.085	0.526	112	0.335	64.85
T0794TS391_4-D1.rsa	37.5	0.446	0.089	0.465	99	0.379	74.87

T0794TS425_1-D1.rsa	37.5	0.015	0.36	0.625	85	0.441	64.71
T0794TS216_5-D1.rsa	37.5	0	0.471	0.529	72	0.521	66.18
T0794TS310_1-D1.rsa	37.5	0.176	0.029	0.794	108	0.347	36.4
T0794TS080_1-D1.rsa	37.288	0.009	0.308	0.682	146	0.255	58.53
T0794TS436_4-D1.rsa	37.288	0.051	0.192	0.757	162	0.23	34.46
T0794TS153_1-D1.rsa	37.143	0.455	0.022	0.522	211	0.176	7.8
T0794TS011_2-D1.rsa	36.842	0.267	0.171	0.562	122	0.302	57.14
T0794TS317_1_1-D1.rsa	36.842	0.521	0.059	0.419	99	0.372	63.66
T0794TS290_3-D1.rsa	36.207	0.014	0.14	0.846	121	0.299	79.2
T0794TS008_4-D1.rsa	36.207	0	0.196	0.804	115	0.315	76.57
T0794TS073_2-D1.rsa	36.207	0	0.084	0.916	131	0.276	59.27
T0794TS410_1-D1.rsa	36.111	0.38	0.089	0.531	113	0.32	76.4
T0794TS499_1-D1.rsa	36.111	0.352	0.094	0.554	118	0.306	74.87
T0794TS024_1_1-D1.rsa	36.066	0.029	0.41	0.562	118	0.306	70.03
T0794TS347_5-D1.rsa	36.047	0.29	0.142	0.568	96	0.375	53.4
T0794TS368_2-D1.rsa	35.965	0.253	0.166	0.581	126	0.285	56.34
T0794TS420_2-D1.rsa	35.965	0.313	0.147	0.539	117	0.307	61.75
T0794TS216_2-D1.rsa	35.938	0.015	0.353	0.632	86	0.418	67.83
T0794TS132_5-D1.rsa	35.789	0.542	0.038	0.419	99	0.362	58.16
T0794TS184_3-D1.rsa	35.789	0.513	0.055	0.432	102	0.351	64.62
T0794TS064_2-D1.rsa	35.789	0.504	0.059	0.436	103	0.347	50.74
T0794TS251_4-D1.rsa	35.526	0.298	0.048	0.654	217	0.164	8.66
T0794TS067_5-D1.rsa	35.345	0.57	0	0.43	110	0.321	56.74
T0794TS347_3-D1.rsa	35	0	0.158	0.842	96	0.365	44.96
T0794TS064_5-D1.rsa	34.884	0.266	0.166	0.568	96	0.363	65.83
T0794TS452_1-D1.rsa	34.857	0.53	0	0.47	190	0.183	9.41
T0794TS038_5-D1.rsa	34.722	0.385	0.094	0.521	111	0.313	75.51
T0794TS492_5-D1.rsa	34.615	0.127	0.061	0.812	134	0.258	37.5
T0794TS381_2-D1.rsa	34.426	0.052	0.386	0.562	118	0.292	71.27
T0794TS300_4-D1.rsa	34.426	0.019	0.424	0.557	117	0.294	73.51
T0794TS157_3-D1.rsa	34.4	0.024	0.252	0.723	238	0.145	53.95
T0794TS282_5-D1.rsa	34.091	0.068	0.439	0.493	146	0.233	38.6
T0794TS357_1-D1.rsa	33.714	0.473	0.042	0.485	196	0.172	26.18
T0794TS064_1-D1.rsa	33.621	0.578	0	0.422	108	0.311	54.59
T0794TS282_1-D1.rsa	33.333	0.278	0.231	0.491	115	0.29	68.91
T0794TS296_1-D1.rsa	33.333	0.371	0.103	0.526	112	0.298	70.81
T0794TS204_5-D1.rsa	32.955	0.007	0.392	0.601	178	0.185	45.1
T0794TS133_5-D1.rsa	32.955	0.061	0.392	0.547	162	0.203	33.62
T0794TS216_4-D1.rsa	32.813	0.015	0.397	0.588	80	0.41	72.06
T0794TS333_4-D1.rsa	32.759	0.59	0	0.41	105	0.312	57.23
T0794TS338_2-D1.rsa	32.632	0.521	0.055	0.424	100	0.326	59
T0794TS301_3-D1.rsa	32.558	0.278	0.195	0.527	89	0.366	72.93
T0794TS425_4-D1.rsa	32.558	0.172	0.015	0.813	109	0.299	43.28
T0794TS008_3-D1.rsa	32.456	0.281	0.101	0.618	134	0.242	55.3
T0794TS335_1-D1.rsa	32.381	0.286	0.162	0.551	129	0.251	68.91
T0794TS210_5-D1.rsa	32.381	0.282	0.179	0.538	126	0.257	69.12
T0794TS438_1-D1.rsa	32	0.094	0.326	0.58	80	0.4	61.23

T0794TS157_4-D1.rsa	31.973	0.375	0.094	0.531	198	0.161	77.78
T0794TS132_2-D1.rsa	31.944	0.474	0.07	0.455	97	0.329	55.96
T0794TS169_2-D1.rsa	31.897	0.613	0	0.387	99	0.322	57.32
T0794TS290_5-D1.rsa	31.897	0.605	0	0.395	101	0.316	55.47
T0794TS235_1-D1.rsa	31.507	0.345	0.105	0.55	204	0.154	70.98
T0794TS364_1-D1.rsa	31.429	0.363	0.231	0.406	95	0.331	61.11
T0794TS483_3-D1.rsa	31.395	0.302	0.178	0.521	88	0.357	72.34
T0794TS080_2-D1.rsa	31.313	0.257	0.16	0.583	168	0.186	46.18
T0794TS118_3-D1.rsa	31.148	0.01	0.295	0.695	146	0.213	55.97
T0794TS381_5-D1.rsa	31.148	0.024	0.429	0.548	115	0.271	73.01
T0794TS277_1-D1.rsa	31.034	0	0.217	0.783	112	0.277	77.8
T0794TS144_2-D1.rsa	30.882	0.399	0.095	0.506	123	0.251	69.33
T0794TS414_5-D1.rsa	30.882	0.358	0.095	0.547	133	0.232	65.11
T0794TS483_2-D1.rsa	30.882	0.428	0.103	0.469	114	0.271	64.22
T0794TS235_5-D1.rsa	30.822	0.385	0.086	0.528	196	0.157	73.84
T0794TS483_5-D1.rsa	30.702	0.24	0.138	0.622	135	0.227	64.75
T0794TS011_1-D1.rsa	30.476	0.308	0.162	0.53	124	0.246	65.7
T0794TS132_3-D1.rsa	30.476	0.286	0.214	0.5	117	0.26	69.77
T0794TS326_1-D1.rsa	30.137	0.385	0.105	0.509	189	0.159	73.5
T0794TS340_3-D1.rsa	29.87	0.362	0.067	0.571	128	0.233	72.14
T0794TS420_1-D1.rsa	29.87	0.402	0.121	0.478	107	0.279	79.52
T0794TS155_5-D1.rsa	29.6	0.018	0.374	0.608	200	0.148	53.42
T0794TS228_1-D1.rsa	29.6	0.018	0.383	0.599	197	0.15	53.19
T0794TS492_4-D1.rsa	29.524	0.359	0.197	0.444	104	0.284	70.94
T0794TS479_2-D1.rsa	29.508	0.052	0.324	0.624	131	0.225	68.91
T0794TS228_2-D1.rsa	29.452	0.394	0.108	0.499	185	0.159	81.95
T0794TS349_5-D1.rsa	29.293	0.149	0.16	0.691	199	0.147	51.04
T0794TS117_4-D1.rsa	29.293	0.288	0.233	0.479	138	0.212	49.48
T0794TS483_4-D1.rsa	29.167	0.305	0.085	0.61	130	0.224	64.85
T0794TS197_5-D1.rsa	29	0.394	0.12	0.486	122	0.238	78.59
T0794TS425_3-D1.rsa	29	0.45	0.108	0.442	111	0.261	81.67
T0794TS153_3-D1.rsa	28.814	0.014	0.327	0.659	141	0.204	64.02
T0794TS335_3-D1.rsa	28.814	0.009	0.299	0.692	148	0.195	59.23
T0794TS034_1-D1.rsa	28.814	0.009	0.285	0.706	151	0.191	42.76
T0794TS263_3-D1.rsa	28.8	0.012	0.41	0.578	190	0.152	53.34
T0794TS157_5-D1.rsa	28.713	0.265	0.146	0.589	189	0.152	75.86
T0794TS326_5-D1.rsa	28.713	0.28	0.143	0.576	185	0.155	74.61
T0794TS049_2-D1.rsa	28.713	0.268	0.137	0.595	191	0.15	74.14
T0794TS268_5-D1.rsa	28.571	0.402	0.107	0.491	110	0.26	78.45
T0794TS360_4-D1.rsa	28.571	0.393	0.12	0.487	114	0.251	69.12
T0794TS276_1-D1.rsa	28.082	0.396	0.108	0.496	184	0.153	81.4
T0794TS326_4-D1.rsa	28.082	0.388	0.113	0.499	185	0.152	78.34
T0794TS235_3-D1.rsa	28	0.049	0.267	0.684	225	0.124	31.99
T0794TS317_5_1-D1.rsa	28	0.426	0.135	0.438	110	0.255	88.05
T0794TS335_5-D1.rsa	28	0.446	0.1	0.454	114	0.246	81.57
T0794TS296_3-D1.rsa	27.941	0.35	0.062	0.588	143	0.195	59.11
T0794TS434_1-D1.rsa	27.941	0.403	0.119	0.477	116	0.241	70.22

T0794TS276_3-D1.rsa	27.891	0.381	0.099	0.52	194	0.144	81.04
T0794TS448_4-D1.rsa	27.869	0.024	0.295	0.681	143	0.195	70.52
T0794TS144_3-D1.rsa	27.869	0.024	0.352	0.624	131	0.213	57.96
T0794TS368_1-D1.rsa	27.869	0.019	0.414	0.567	119	0.234	65.3
T0794TS251_1-D1.rsa	27.273	0.219	0.177	0.604	174	0.157	60.24
T0794TS162_1_1-D1.rsa	27.273	0.379	0.103	0.518	116	0.235	79.41
T0794TS442_2-D1.rsa	27.211	0.357	0.078	0.566	211	0.129	81.74
T0794TS277_4-D1.rsa	27.119	0	0.318	0.682	146	0.186	62.38
T0794TS197_1-D1.rsa	27	0.378	0.108	0.514	129	0.209	88.75
T0794TS448_5-D1.rsa	26.923	0.318	0.118	0.564	167	0.161	49.57
T0794TS448_2-D1.rsa	26.923	0.331	0.125	0.544	161	0.167	53.47
T0794TS042_4-D1.rsa	26.923	0.378	0.111	0.51	151	0.178	57.55
T0794TS228_3-D1.rsa	26.531	0.37	0.105	0.525	196	0.135	80.62
T0794TS157_1-D1.rsa	26.531	0.375	0.134	0.491	183	0.145	84.51
T0794TS212_1-D1.rsa	26.471	0.395	0.099	0.506	123	0.215	71.22
T0794TS345_2-D1.rsa	26.471	0.399	0.095	0.506	123	0.215	70.44
T0794TS290_1-D1.rsa	26.471	0.432	0.107	0.461	112	0.236	70.22
T0794TS145_4-D1.rsa	26.471	0.395	0.107	0.498	121	0.219	58
T0794TS326_2-D1.rsa	26.4	0.015	0.38	0.605	199	0.133	52.96
T0794TS237_5-D1.rsa	26.316	0.349	0.012	0.639	212	0.124	10.02
T0794TS322_1-D1.rsa	26.263	0.201	0.184	0.615	177	0.148	62.59
T0794TS479_4-D1.rsa	26.263	0.215	0.222	0.563	162	0.162	67.71
T0794TS296_4-D1.rsa	26.23	0.014	0.414	0.571	120	0.219	63.93
T0794TS338_1-D1.rsa	26.23	0.01	0.4	0.59	124	0.212	63.43
T0794TS049_4-D1.rsa	26.027	0.415	0.1	0.485	180	0.145	75.55
T0794TS237_3-D1.rsa	26	0.41	0.116	0.474	119	0.218	89.74
T0794TS445_5-D1.rsa	26	0.43	0.108	0.462	116	0.224	88.55
T0794TS436_2-D1.rsa	26	0.398	0.139	0.462	116	0.224	83.86
T0794TS160_5-D1.rsa	25.974	0.402	0.121	0.478	107	0.243	79.64
T0794TS063_1-D1.rsa	25.85	0.37	0.105	0.525	196	0.132	84.58
T0794TS204_4-D1.rsa	25.714	0.475	0.032	0.493	199	0.129	36.82
T0794TS479_1-D1.rsa	25.641	0.382	0.111	0.507	150	0.171	59.81
T0794TS436_1-D1.rsa	25.641	0.382	0.132	0.486	144	0.178	59.03
T0794TS144_4-D1.rsa	25.641	0.399	0.128	0.473	140	0.183	58.33
T0794TS263_2-D1.rsa	25.641	0.405	0.125	0.47	139	0.184	58.42
T0794TS080_5-D1.rsa	25.6	0.015	0.38	0.605	199	0.129	55.17
T0794TS237_1-D1.rsa	25.424	0.037	0.145	0.818	175	0.145	51.99
T0794TS436_5-D1.rsa	25.253	0.16	0.007	0.833	240	0.105	9.98
T0794TS206_1-D1.rsa	25.17	0.383	0.139	0.477	178	0.141	80.35
T0794TS479_5-D1.rsa	25	0.321	0.091	0.588	143	0.175	68.44
T0794TS237_4-D1.rsa	25	0.346	0.086	0.568	138	0.181	67.33
T0794TS381_3-D1.rsa	25	0.41	0.116	0.474	119	0.21	89.54
T0794TS439_2-D1.rsa	25	0.354	0.066	0.58	141	0.177	66.44
T0794TS153_5-D1.rsa	24.762	0.312	0.15	0.538	126	0.197	64.1
T0794TS300_5-D1.rsa	24.59	0	0.381	0.619	130	0.189	63.93
T0794TS097_3-D1.rsa	24.59	0.038	0.476	0.486	102	0.241	67.54
T0794TS155_3-D1.rsa	24.49	0.375	0.097	0.528	197	0.124	82.29

T0794TS381_4-D1.rsa	24.359	0.378	0.125	0.497	147	0.166	58.94
T0794TS360_1-D1.rsa	24.359	0.348	0.128	0.524	155	0.157	59.72
T0794TS439_3-D1.rsa	24.359	0.385	0.132	0.483	143	0.17	59.55
T0794TS235_2-D1.rsa	24.242	0.198	0.076	0.726	209	0.116	60.16
T0794TS049_1-D1.rsa	24.242	0.132	0.063	0.806	232	0.104	30.47
T0794TS153_2-D1.rsa	24.242	0.167	0.014	0.819	236	0.103	11.02
T0794TS276_5-D1.rsa	24.074	0.414	0.088	0.498	227	0.106	51.33
T0794TS251_3-D1.rsa	24	0.438	0.025	0.537	217	0.111	43.87
T0794TS263_4-D1.rsa	24	0.512	0.017	0.47	190	0.126	44.31
T0794TS499_4-D1.rsa	24	0.402	0.12	0.478	120	0.2	89.54
T0794TS300_1-D1.rsa	24	0.45	0.112	0.438	110	0.218	89.84
T0794TS347_4-D1.rsa	24	0	0.319	0.681	94	0.255	61.23
T0794TS310_2-D1.rsa	24	0.101	0.377	0.522	72	0.333	67.57
T0794TS160_2-D1.rsa	23.729	0.014	0.332	0.654	140	0.169	63.55
T0794TS276_2-D1.rsa	23.684	0.452	0.015	0.533	243	0.097	50
T0794TS454_4-D1.rsa	23.529	0.366	0.095	0.539	131	0.18	64.89
T0794TS251_5-D1.rsa	23.429	0.426	0.025	0.55	222	0.106	46.1
T0794TS268_4-D1.rsa	23.377	0.362	0.138	0.5	112	0.209	77.98
T0794TS268_2-D1.rsa	23.377	0.379	0.116	0.504	113	0.207	71.31
T0794TS358_4-D1.rsa	23.232	0.212	0.212	0.576	166	0.14	57.73
T0794TS301_5-D1.rsa	23.232	0.253	0.222	0.524	151	0.154	69.36
T0794TS263_5-D1.rsa	23.077	0.409	0.142	0.449	133	0.174	60.5
T0794TS204_1-D1.rsa	23.077	0.395	0.132	0.473	140	0.165	57.64
T0794TS410_2-D1.rsa	23.077	0.399	0.105	0.497	147	0.157	54.34
T0794TS145_3-D1.rsa	23.077	0.389	0.139	0.473	140	0.165	54.77
T0794TS420_5-D1.rsa	23	0.454	0.131	0.414	104	0.221	89.14
T0794TS145_1-D1.rsa	22.857	0.52	0.015	0.465	188	0.122	9.9
T0794TS162_5-D1.rsa	22.807	0.406	0.05	0.543	119	0.192	71.35
T0794TS276_4-D1.rsa	22.368	0.196	0.072	0.732	243	0.092	33.51
T0794TS118_4-D1.rsa	22.286	0.515	0.027	0.458	185	0.12	45.05
T0794TS117_2-D1.rsa	22.222	0.393	0.099	0.509	232	0.096	72.74
T0794TS049_3-D1.rsa	22.222	0.118	0.111	0.771	222	0.1	36.11
T0794TS338_5-D1.rsa	22.222	0.247	0.219	0.535	154	0.144	70.4
T0794TS162_3-D1.rsa	22.059	0.383	0.115	0.502	122	0.181	64.44
T0794TS452_5-D1.rsa	22	0.406	0.124	0.47	118	0.186	90.04
T0794TS360_3-D1.rsa	21.795	0.358	0.152	0.49	145	0.15	57.55
T0794TS322_3-D1.rsa	21.714	0.433	0.022	0.545	220	0.099	43.81
T0794TS414_4-D1.rsa	21.311	0.014	0.405	0.581	122	0.175	69.53
T0794TS499_2-D1.rsa	21.311	0.033	0.367	0.6	126	0.169	59.58
T0794TS237_2-D1.rsa	21.053	0.384	0.064	0.553	121	0.174	73.06
T0794TS391_3-D1.rsa	21.053	0.438	0.082	0.479	105	0.201	81.05
T0794TS145_5-D1.rsa	21.053	0.452	0.082	0.466	102	0.206	80.25
T0794TS067_3-D1.rsa	21.053	0.434	0.096	0.47	103	0.204	81.05
T0794TS340_2-D1.rsa	21.053	0.402	0.091	0.507	111	0.19	74.77
T0794TS445_2-D1.rsa	21.053	0.457	0.091	0.452	99	0.213	79.57
T0794TS160_4-D1.rsa	20.93	0.406	0.12	0.473	134	0.156	80.56
T0794TS162_2-D1.rsa	20.779	0.375	0.107	0.518	116	0.179	81.55

T0794TS133_2-D1.rsa	20.779	0.384	0.125	0.491	110	0.189	79.41
T0794TS065_1_1-D1.rsa	20.779	0.388	0.125	0.487	109	0.191	78.93
T0794TS439_5-D1.rsa	20.588	0.342	0.07	0.588	143	0.144	68.22
T0794TS118_5-D1.rsa	20.588	0.383	0.111	0.506	123	0.167	65.22
T0794TS349_4-D1.rsa	20.571	0.515	0.005	0.48	194	0.106	44.8
T0794TS439_4-D1.rsa	19.767	0.382	0.113	0.505	143	0.138	77.12
T0794TS063_2-D1.rsa	19.737	0.434	0.004	0.561	256	0.077	58.94
T0794TS368_4-D1.rsa	19.737	0.346	0.018	0.636	211	0.094	14.16
T0794TS132_1-D1.rsa	19.481	0.388	0.08	0.531	119	0.164	75.24
T0794TS420_3-D1.rsa	19.481	0.371	0.129	0.5	112	0.174	77.5
T0794TS117_3-D1.rsa	19.481	0.402	0.094	0.504	113	0.172	81.67
T0794TS038_3-D1.rsa	19.481	0.393	0.125	0.482	108	0.18	80.71
T0794TS326_3-D1.rsa	19.429	0.458	0	0.542	219	0.089	46.66
T0794TS210_3-D1.rsa	19.298	0.434	0.078	0.489	107	0.18	81.62
T0794TS050_1-D1.rsa	19.298	0.429	0.096	0.475	104	0.186	78.31
T0794TS064_4-D1.rsa	19.298	0.457	0.078	0.466	102	0.189	80.59
T0794TS184_1-D1.rsa	19.149	0.381	0.163	0.455	117	0.164	58.66
T0794TS235_4-D1.rsa	19.136	0.436	0.099	0.465	212	0.09	71.63
T0794TS279_1-D1.rsa	19.118	0.379	0.115	0.506	123	0.155	65.11
T0794TS228_4-D1.rsa	18.644	0.009	0.154	0.836	179	0.104	63.32
T0794TS414_3-D1.rsa	18.644	0.037	0.238	0.724	155	0.12	62.97
T0794TS381_1-D1.rsa	18.605	0.385	0.12	0.495	140	0.133	81.18
T0794TS442_1-D1.rsa	18.519	0.399	0.066	0.535	244	0.076	69.48
T0794TS157_2-D1.rsa	18.421	0.328	0.111	0.56	186	0.099	94.58
T0794TS296_2-D1.rsa	18.033	0.014	0.433	0.552	116	0.155	70.15
T0794TS417_1-D1.rsa	17.822	0.206	0.106	0.688	221	0.081	50.08
T0794TS263_1-D1.rsa	17.442	0.403	0.11	0.488	138	0.126	83.22
T0794TS042_2-D1.rsa	17.442	0.403	0.117	0.481	136	0.128	82.07
T0794TS063_3-D1.rsa	17.105	0.443	0.004	0.553	252	0.068	63.16
T0794TS080_3-D1.rsa	17.105	0.307	0.154	0.539	179	0.096	97.44
T0794TS063_5-D1.rsa	16.923	0.24	0.197	0.563	258	0.066	72.94
T0794TS442_5-D1.rsa	16.923	0.255	0.231	0.513	235	0.072	76.22
T0794TS277_5-D1.rsa	16.883	0.379	0.089	0.531	119	0.142	76.43
T0794TS437_1-D1.rsa	16.279	0.382	0.117	0.502	142	0.115	79.68
T0794TS042_5-D1.rsa	16.279	0.406	0.113	0.481	136	0.12	81.98
T0794TS132_4-D1.rsa	16	0.058	0.341	0.601	83	0.193	68.66
T0794TS340_4-D1.rsa	15.116	0.41	0.117	0.473	134	0.113	82.51
T0794TS042_1-D1.rsa	14.894	0.292	0.148	0.56	144	0.103	78.7
T0794TS442_4-D1.rsa	14.615	0.264	0.216	0.52	238	0.061	76.22
T0794TS197_2-D1.rsa	14.035	0.443	0.082	0.475	104	0.135	81.62
T0794TS063_4-D1.rsa	13.077	0.26	0.24	0.5	229	0.057	77.78
T0794TS335_2-D1.rsa	12.766	0.331	0.183	0.486	125	0.102	83.66
T0794TS056_1-D1.rsa	12.766	0.327	0.195	0.479	123	0.104	79.47
T0794TS420_4-D1.rsa	12.766	0.362	0.202	0.436	112	0.114	83.46
T0794TS268_1-D1.rsa	12.766	0.362	0.198	0.44	113	0.113	83.07
T0794TS368_5-D1.rsa	12.766	0.35	0.202	0.447	115	0.111	79.18
T0794TS322_5-D1.rsa	11.842	0.325	0.13	0.545	181	0.065	99.02

T0794TS097_5-D1.rsa	10.638	0.319	0.187	0.494	127	0.084	81.23
T0794TS345_1-D1.rsa	10.638	0.346	0.198	0.455	117	0.091	84.73
T0794TS410_5-D1.rsa	10.638	0.292	0.183	0.525	135	0.079	77.24
T0794TS282_2-D1.rsa	10.638	0.339	0.198	0.463	119	0.089	85.41
T0794TS338_3-D1.rsa	10.638	0.335	0.179	0.486	125	0.085	79.38
T0794TS044_2-D1.rsa	10.638	0.389	0.195	0.416	107	0.099	81.42
T0811TS237_1-D1.rsa	76.316	0.18	0.054	0.766	85	0.898	23.65
T0811TS228_5-D1.rsa	71.053	0.054	0	0.946	105	0.677	15.77
T0811TS038_2-D1.rsa	70.833	0.139	0.324	0.537	58	1.221	18.98
T0811TS160_2-D1.rsa	70.588	0.548	0.04	0.413	52	1.357	33.93
T0811TS193_3-D1.rsa	65.789	0.279	0.126	0.595	66	0.997	34.23
T0811TS228_3-D1.rsa	62.5	0.037	0.454	0.509	55	1.136	50.93
T0811TS133_5-D1.rsa	61.765	0.579	0	0.421	53	1.165	34.72
T0811TS410_1-D1.rsa	60.714	0	0.237	0.763	100	0.607	17.37
T0811TS263_2-D1.rsa	59.615	0.218	0.085	0.697	115	0.518	43.99
T0811TS050_1-D1.rsa	59.375	0	0.36	0.64	87	0.682	12.68
T0811TS008_5-D1.rsa	58.333	0.037	0.472	0.491	53	1.101	71.76
T0811TS237_2-D1.rsa	58.14	0	0.179	0.821	110	0.529	14.18
T0811TS452_4-D1.rsa	57.895	0.297	0	0.703	78	0.742	27.03
T0811TS499_4-D1.rsa	57.895	0.045	0.198	0.757	84	0.689	22.07
T0811TS038_4-D1.rsa	57.143	0.254	0.222	0.524	66	0.866	22.82
T0811TS381_4-D1.rsa	56	0.036	0.203	0.761	105	0.533	20.65
T0811TS410_2-D1.rsa	55.882	0.603	0	0.397	50	1.118	42.66
T0811TS156_3-D1.rsa	54.167	0	0.63	0.37	40	1.354	65.51
T0811TS184_3-D1.rsa	53.125	0.015	0.338	0.647	88	0.604	12.68
T0811TS011_2-D1.rsa	51.786	0.095	0.103	0.802	101	0.513	15.87
T0811TS414_5-D1.rsa	50	0.19	0.27	0.54	68	0.735	54.17
T0811TS300_2-D1.rsa	50	0	0.359	0.641	84	0.595	17.56
T0811TS184_2-D1.rsa	50	0.018	0.227	0.755	83	0.602	32.5
T0811TS206_1-D1.rsa	50	0.297	0.108	0.595	66	0.758	38.74
T0811TS133_2-D1.rsa	50	0	0.397	0.603	79	0.633	17.18
T0811TS479_3-D1.rsa	48.077	0.297	0.024	0.679	112	0.429	16.77
T0811TS381_2-D1.rsa	47.887	0.31	0.077	0.613	103	0.465	16.99
T0811TS251_4-D1.rsa	47.368	0.225	0.162	0.613	68	0.697	31.53
T0811TS448_3-D1.rsa	46.512	0.306	0	0.694	93	0.5	28.92
T0811TS228_2-D1.rsa	46.479	0.411	0.03	0.56	94	0.494	18.59
T0811TS008_3-D1.rsa	46.429	0.135	0.143	0.722	91	0.51	20.44
T0811TS171_1-D1.rsa	46.429	0	0.374	0.626	82	0.566	17.56
T0811TS038_3-D1.rsa	46.429	0	0.351	0.649	85	0.546	17.56
T0811TS454_1-D1.rsa	46.429	0.214	0.103	0.683	86	0.54	19.25
T0811TS073_2-D1.rsa	45.833	0	0.436	0.564	62	0.739	48.18
T0811TS492_3-D1.rsa	45.07	0.226	0.155	0.619	104	0.433	17.15
T0811TS345_4-D1.rsa	45	0	0.377	0.623	71	0.634	28.07
T0811TS145_2-D1.rsa	44.737	0.27	0.108	0.622	69	0.648	38.51
T0811TS349_3-D1.rsa	44.643	0.254	0.016	0.73	92	0.485	16.47
T0811TS216_3-D1.rsa	44.643	0.183	0.143	0.675	85	0.525	22.02
T0811TS160_3-D1.rsa	44.186	0.388	0.03	0.582	78	0.566	19.96

T0811TS349_1-D1.rsa	44	0.065	0.355	0.58	80	0.55	35.33
T0811TS145_4-D1.rsa	42.188	0.081	0.25	0.669	91	0.464	15.07
T0811TS210_1-D1.rsa	42.105	0.198	0	0.802	89	0.473	17.79
T0811TS499_1-D1.rsa	42.105	0.234	0.189	0.577	64	0.658	37.84
T0811TS345_1-D1.rsa	42.105	0.306	0.09	0.604	67	0.628	36.71
T0811TS452_2-D1.rsa	41.86	0.231	0.119	0.649	87	0.481	24.44
T0811TS381_3-D1.rsa	41.176	0.424	0.099	0.477	116	0.355	11.44
T0811TS420_4-D1.rsa	41.176	0.516	0	0.484	61	0.675	46.83
T0811TS268_2-D1.rsa	40.625	0.029	0.191	0.779	106	0.383	13.42
T0811TS160_4-D1.rsa	40.517	0.613	0	0.387	99	0.409	22.27
T0811TS251_1-D1.rsa	40.278	0.446	0.094	0.46	98	0.411	70.05
T0811TS156_4-D1.rsa	40	0.018	0.228	0.754	86	0.465	46.05
T0811TS300_3-D1.rsa	40	0.145	0.101	0.754	104	0.385	18.3
T0811TS216_1-D1.rsa	39.726	0.702	0	0.298	76	0.523	36.77
T0811TS479_5-D1.rsa	39.535	0.358	0.045	0.597	80	0.494	52.24
T0811TS436_4-D1.rsa	39.535	0.291	0	0.709	95	0.416	39.92
T0811TS349_4-D1.rsa	39.535	0.224	0.052	0.724	97	0.408	21.08
T0811TS452_1-D1.rsa	39.286	0	0.16	0.84	110	0.357	13.74
T0811TS117_1-D1.rsa	39.063	0.14	0.074	0.787	107	0.365	13.23
T0811TS251_3-D1.rsa	37.5	0.091	0.264	0.645	71	0.528	48.41
T0811TS346_1-D1.rsa	37.209	0.276	0.06	0.664	89	0.418	23.88
T0811TS041_2-D1.rsa	37.209	0.276	0.082	0.642	86	0.433	23.51
T0811TS022_1-D1.rsa	36.986	0.745	0	0.255	65	0.569	55.2
T0811TS011_3-D1.rsa	36.842	0.063	0	0.937	104	0.354	21.17
T0811TS216_2-D1.rsa	36.842	0.27	0.081	0.649	72	0.512	36.71
T0811TS041_5-D1.rsa	36.538	0.218	0.091	0.691	114	0.321	14.24
T0811TS454_3-D1.rsa	36.207	0.703	0	0.297	76	0.476	44.63
T0811TS237_4-D1.rsa	36.111	0.399	0.085	0.516	110	0.328	65.48
T0811TS145_1-D1.rsa	35.938	0.096	0.221	0.684	93	0.386	12.87
T0811TS335_4-D1.rsa	35.714	0.175	0.278	0.548	69	0.518	57.34
T0811TS228_1-D1.rsa	35.714	0.079	0.389	0.532	67	0.533	52.18
T0811TS184_5-D1.rsa	35.714	0.127	0.254	0.619	78	0.458	42.26
T0811TS335_1-D1.rsa	35.714	0.16	0	0.84	110	0.325	22.71
T0811TS041_1-D1.rsa	35.616	0.753	0	0.247	63	0.565	60
T0811TS492_1-D1.rsa	35.616	0.718	0	0.282	72	0.495	35.2
T0811TS448_4-D1.rsa	35.345	0.598	0	0.402	103	0.343	55.47
T0811TS133_4-D1.rsa	35.211	0.399	0.131	0.47	79	0.446	40.87
T0811TS117_4-D1.rsa	34.884	0.299	0.015	0.687	92	0.379	27.24
T0811TS011_4-D1.rsa	34.884	0.313	0	0.687	92	0.379	43.47
T0811TS011_1-D1.rsa	34.884	0.224	0.06	0.716	96	0.363	21.45
T0811TS452_3-D1.rsa	34.884	0.284	0	0.716	96	0.363	27.43
T0811TS008_4-D1.rsa	34.247	0.741	0	0.259	66	0.519	59.8
T0811TS011_5-D1.rsa	34.211	0.072	0	0.928	103	0.332	20.95
T0811TS492_2-D1.rsa	34.211	0.241	0.454	0.306	33	1.037	93.06
T0811TS454_5-D1.rsa	33.929	0.19	0.254	0.556	70	0.485	48.61
T0811TS454_2-D1.rsa	33.929	0.167	0.357	0.476	60	0.565	49.6
T0811TS448_2-D1.rsa	33.824	0.412	0.086	0.502	122	0.277	23.56

T0811TS448_1-D1.rsa	33.684	0.5	0.059	0.441	104	0.324	49.36
T0811TS499_2-D1.rsa	32.877	0.737	0	0.263	67	0.491	58.82
T0811TS349_2-D1.rsa	32.877	0.761	0	0.239	61	0.539	62.06
T0811TS008_1-D1.rsa	32.877	0.757	0	0.243	62	0.53	57.45
T0811TS349_5-D1.rsa	32.877	0.729	0	0.271	69	0.476	50.39
T0811TS492_4-D1.rsa	32.877	0.761	0	0.239	61	0.539	55.29
T0811TS251_2-D1.rsa	32.759	0.617	0	0.383	98	0.334	55.66
T0811TS184_4-D1.rsa	32.558	0.284	0.097	0.619	83	0.392	36.01
T0811TS268_1-D1.rsa	32.558	0.373	0.142	0.485	65	0.501	50.93
T0811TS263_1-D1.rsa	32.5	0	0.158	0.842	96	0.339	20.18
T0811TS345_3-D1.rsa	32.5	0.018	0.439	0.544	62	0.524	49.56
T0811TS279_1-D1.rsa	32	0.181	0.123	0.696	96	0.333	17.03
T0811TS184_1-D1.rsa	31.579	0.25	0.509	0.241	26	1.215	93.98
T0811TS420_2-D1.rsa	31.579	0.25	0.5	0.25	27	1.17	94.21
T0811TS277_1-D1.rsa	31.579	0.241	0.5	0.259	28	1.128	93.98
T0811TS216_5-D1.rsa	31.579	0.534	0.059	0.407	96	0.329	37.39
T0811TS499_3-D1.rsa	31.579	0.25	0.509	0.241	26	1.215	93.98
T0811TS420_1-D1.rsa	31.507	0.757	0	0.243	62	0.508	66.57
T0811TS237_3-D1.rsa	31.034	0.59	0	0.41	105	0.296	60.74
T0811TS156_1-D1.rsa	30.986	0.339	0.107	0.554	93	0.333	38.3
T0811TS171_5-D1.rsa	30.526	0.525	0.055	0.419	99	0.308	42.48
T0811TS212_1-D1.rsa	30.526	0.542	0.064	0.394	93	0.328	49.15
T0811TS499_5-D1.rsa	30.357	0.278	0.032	0.69	87	0.349	27.98
T0811TS277_4-D1.rsa	30.357	0.175	0.262	0.563	71	0.428	43.85
T0811TS216_4-D1.rsa	30.233	0.224	0	0.776	104	0.291	14.18
T0811TS335_5-D1.rsa	30.137	0.733	0	0.267	68	0.443	58.53
T0811TS171_4-D1.rsa	30.137	0.749	0	0.251	64	0.471	56.27
T0811TS156_2-D1.rsa	30	0.035	0.228	0.737	84	0.357	43.64
T0811TS171_2-D1.rsa	30	0	0.053	0.947	108	0.278	21.93
T0811TS420_3-D1.rsa	30	0	0.474	0.526	60	0.5	48.03
T0811TS145_5-D1.rsa	30	0	0.386	0.614	70	0.429	42.98
T0811TS041_4-D1.rsa	29.688	0.096	0.081	0.824	112	0.265	13.23
T0811TS414_4-D1.rsa	29.474	0.564	0.042	0.394	93	0.317	56.46
T0811TS436_3-D1.rsa	29	0.422	0.108	0.47	118	0.246	77.89
T0811TS251_5-D1.rsa	28.947	0.231	0.5	0.269	29	0.998	94.21
T0811TS008_2-D1.rsa	28.947	0.259	0.481	0.259	28	1.034	93.98
T0811TS345_2-D1.rsa	28.947	0.216	0.126	0.658	73	0.397	38.74
T0811TS038_1-D1.rsa	28.767	0.749	0	0.251	64	0.449	55.49
T0811TS277_3-D1.rsa	28.421	0.525	0.059	0.415	98	0.29	32.52
T0811TS479_1-D1.rsa	28	0.036	0.333	0.63	87	0.322	59.24
T0811TS436_2-D1.rsa	28	0.123	0.072	0.804	111	0.252	12.86
T0811TS156_5-D1.rsa	25.581	0.308	0.195	0.497	84	0.305	72.19
T0811TS038_5-D1.rsa	25	0	0.342	0.658	75	0.333	43.64
T0811TS410_3-D1.rsa	24	0.051	0.326	0.623	86	0.279	52.17
T0811TS492_5-D1.rsa	23.81	0.389	0.209	0.402	94	0.253	62.71
T0811TS436_1-D1.rsa	23.529	0.412	0.086	0.502	122	0.193	67.44
T0811TS133_3-D1.rsa	23.256	0.402	0.201	0.396	67	0.347	69.53

T0811TS479_2-D1.rsa	22.5	0	0.167	0.833	95	0.237	50.22
T0811TS381_5-D1.rsa	20	0.08	0.29	0.63	87	0.23	67.21
T0811TS171_3-D1.rsa	20	0.022	0.312	0.667	92	0.217	41.12
T0811TS452_5-D1.rsa	19.118	0.42	0.008	0.572	139	0.138	12
T0811TS160_5-D1.rsa	12	0.022	0.312	0.667	92	0.13	49.82
T0811TS410_4-D1.rsa	8	0.036	0.268	0.696	96	0.083	49.82
T0819TS268_1-D1.rsa	81.579	0.279	0.054	0.667	74	1.102	16.67
T0819TS171_2-D1.rsa	79.412	0.286	0	0.714	90	0.882	27.38
T0819TS492_3-D1.rsa	79.167	0.019	0.491	0.491	53	1.494	61.11
T0819TS237_2-D1.rsa	74.648	0.327	0	0.673	113	0.661	13.78
T0819TS381_5-D1.rsa	73.684	0.081	0	0.919	102	0.722	16.44
T0819TS414_5-D1.rsa	73.684	0.162	0.054	0.784	87	0.847	15.54
T0819TS184_1-D1.rsa	73.529	0.532	0	0.468	59	1.246	28.18
T0819TS156_2-D1.rsa	71.053	0.252	0	0.748	83	0.856	17.12
T0819TS156_3-D1.rsa	70.588	0.325	0	0.675	85	0.83	31.55
T0819TS454_5-D1.rsa	68.421	0.243	0.054	0.703	78	0.877	16.89
T0819TS492_2-D1.rsa	65.789	0.117	0	0.883	98	0.671	17.79
T0819TS216_1-D1.rsa	64.706	0.54	0	0.46	58	1.116	31.94
T0819TS237_3-D1.rsa	64.286	0	0.366	0.634	83	0.775	58.59
T0819TS345_2-D1.rsa	64	0	0.391	0.609	84	0.762	21.2
T0819TS160_3-D1.rsa	62.791	0.239	0.015	0.746	100	0.628	14.93
T0819TS414_1-D1.rsa	60.714	0.262	0.175	0.563	71	0.855	25.2
T0819TS345_3-D1.rsa	60.526	0.243	0.054	0.703	78	0.776	35.36
T0819TS300_1-D1.rsa	60.526	0.162	0.054	0.784	87	0.696	27.03
T0819TS171_5-D1.rsa	60.465	0.41	0.045	0.545	73	0.828	39.37
T0819TS479_2-D1.rsa	58.333	0	0.546	0.454	49	1.19	71.53
T0819TS345_4-D1.rsa	56	0.051	0.225	0.725	100	0.56	13.59
T0819TS110_4-D1.rsa	55.814	0.022	0.276	0.701	94	0.594	14.55
T0819TS335_4-D1.rsa	55.263	0.252	0.09	0.658	73	0.757	33.78
T0819TS184_5-D1.rsa	55	0	0.351	0.649	74	0.743	32.24
T0819TS210_1-D1.rsa	54.167	0	0.463	0.537	58	0.934	61.34
T0819TS452_2-D1.rsa	54.167	0	0.583	0.417	45	1.204	75.46
T0819TS420_3-D1.rsa	54.167	0	0.583	0.417	45	1.204	77.08
T0819TS335_3-D1.rsa	54.167	0.056	0.63	0.315	34	1.593	75.46
T0819TS145_4-D1.rsa	53.571	0.053	0.153	0.794	104	0.515	12.98
T0819TS156_4-D1.rsa	53.571	0.167	0.048	0.786	99	0.541	21.63
T0819TS038_1-D1.rsa	53.521	0.244	0.173	0.583	98	0.546	13.46
T0819TS041_1-D1.rsa	52.632	0.171	0.072	0.757	84	0.627	23.65
T0819TS038_3-D1.rsa	52	0.08	0.391	0.529	73	0.712	36.05
T0819TS237_4-D1.rsa	51.163	0.246	0.09	0.664	89	0.575	42.72
T0819TS110_1-D1.rsa	51.163	0.276	0.045	0.679	91	0.562	41.79
T0819TS479_1-D1.rsa	50	0.135	0.246	0.619	78	0.641	49.01
T0819TS452_5-D1.rsa	50	0	0.556	0.444	48	1.042	68.29
T0819TS216_3-D1.rsa	50	0.238	0.143	0.619	78	0.641	22.22
T0819TS300_2-D1.rsa	50	0	0.366	0.634	83	0.602	17.18
T0819TS420_2-D1.rsa	48.837	0.381	0.06	0.56	75	0.651	43.1
T0819TS041_4-D1.rsa	48.438	0.096	0	0.904	123	0.394	38.42

T0819TS492_1-D1.rsa	47.917	0.064	0.291	0.645	71	0.675	54.55
T0819TS448_3-D1.rsa	47.887	0	0.208	0.792	133	0.36	11.22
T0819TS171_4-D1.rsa	47.5	0	0.158	0.842	96	0.495	41.45
T0819TS414_3-D1.rsa	47.5	0	0.272	0.728	83	0.572	30.04
T0819TS210_2-D1.rsa	46.512	0.082	0.097	0.821	110	0.423	14.74
T0819TS345_5-D1.rsa	46.512	0.336	0.06	0.604	81	0.574	44.96
T0819TS414_4-D1.rsa	46.512	0.284	0.082	0.634	85	0.547	42.35
T0819TS499_4-D1.rsa	46.429	0	0.427	0.573	75	0.619	48.66
T0819TS008_3-D1.rsa	45.833	0	0.4	0.6	66	0.694	59.77
T0819TS117_3-D1.rsa	44.737	0.063	0	0.937	104	0.43	23.42
T0819TS335_2-D1.rsa	44.737	0.036	0.018	0.946	105	0.426	17.12
T0819TS381_4-D1.rsa	44.643	0.238	0	0.762	96	0.465	17.86
T0819TS268_4-D1.rsa	44.186	0.213	0.118	0.669	113	0.391	17.6
T0819TS499_3-D1.rsa	44.186	0.336	0.06	0.604	81	0.546	43.28
T0819TS436_2-D1.rsa	43.103	0	0.091	0.909	130	0.332	55.59
T0819TS277_5-D1.rsa	43.103	0.049	0.21	0.741	106	0.407	31.64
T0819TS145_2-D1.rsa	42.857	0	0.366	0.634	83	0.516	58.59
T0819TS237_5-D1.rsa	42.857	0	0.405	0.595	78	0.549	62.21
T0819TS011_4-D1.rsa	41.86	0.291	0.045	0.664	89	0.47	42.72
T0819TS268_2-D1.rsa	41.86	0.396	0.067	0.537	72	0.581	46.27
T0819TS263_1-D1.rsa	41.667	0.091	0.182	0.727	80	0.521	51.36
T0819TS349_3-D1.rsa	40.625	0.015	0.412	0.574	78	0.521	70.96
T0819TS452_4-D1.rsa	40.625	0.015	0.478	0.507	69	0.589	70.77
T0819TS420_4-D1.rsa	40.385	0.242	0.133	0.624	103	0.392	18.35
T0819TS184_2-D1.rsa	40.385	0.267	0.121	0.612	101	0.4	15.98
T0819TS133_5-D1.rsa	40	0.029	0.029	0.942	130	0.308	13.95
T0819TS184_4-D1.rsa	40	0	0.377	0.623	71	0.563	48.47
T0819TS454_2-D1.rsa	40	0.116	0.152	0.732	101	0.396	16.49
T0819TS452_3-D1.rsa	39.583	0	0.282	0.718	79	0.501	60
T0819TS448_1-D1.rsa	39.437	0.25	0	0.75	126	0.313	23.24
T0819TS499_5-D1.rsa	39.437	0.417	0	0.583	98	0.402	42.47
T0819TS041_5-D1.rsa	39.286	0.048	0	0.952	120	0.327	12.3
T0819TS381_3-D1.rsa	39.286	0	0.328	0.672	88	0.446	61.64
T0819TS171_3-D1.rsa	39.286	0	0.344	0.656	86	0.457	52.86
T0819TS008_2-D1.rsa	39.286	0.175	0.071	0.754	95	0.414	35.91
T0819TS436_4-D1.rsa	38.028	0.363	0.071	0.565	95	0.4	38.94
T0819TS277_4-D1.rsa	37.5	0.015	0.404	0.581	79	0.475	70.22
T0819TS145_1-D1.rsa	37.5	0.015	0.441	0.544	74	0.507	70.77
T0819TS160_1-D1.rsa	37.5	0.015	0.382	0.603	82	0.457	60.29
T0819TS349_4-D1.rsa	37.209	0.291	0.075	0.634	85	0.438	44.59
T0819TS251_2-D1.rsa	37.209	0.299	0	0.701	94	0.396	40.48
T0819TS414_2-D1.rsa	36.842	0.25	0.491	0.259	28	1.316	93.98
T0819TS410_1-D1.rsa	36.111	0.432	0.085	0.484	103	0.351	67.39
T0819TS133_3-D1.rsa	35.938	0.015	0.434	0.551	75	0.479	69.67
T0819TS237_1-D1.rsa	35.789	0.521	0.055	0.424	100	0.358	59
T0819TS499_1-D1.rsa	35.345	0.664	0	0.336	86	0.411	61.62
T0819TS410_4-D1.rsa	35.227	0.074	0.524	0.402	119	0.296	42.4

T0819TS268_5-D1.rsa	35	0	0.377	0.623	71	0.493	48.68
T0819TS110_3-D1.rsa	35	0	0.333	0.667	76	0.461	48.47
T0819TS008_1-D1.rsa	35	0	0.018	0.982	112	0.313	16.01
T0819TS216_4-D1.rsa	34.884	0.388	0.03	0.582	78	0.447	53.92
T0819TS228_1-D1.rsa	34.737	0.53	0.055	0.415	98	0.354	59.43
T0819TS251_3-D1.rsa	34.375	0.015	0.353	0.632	86	0.4	64.89
T0819TS410_3-D1.rsa	34.375	0.015	0.456	0.529	72	0.477	70.77
T0819TS038_5-D1.rsa	34.247	0.749	0	0.251	64	0.535	59.02
T0819TS008_4-D1.rsa	33.684	0.525	0.059	0.415	98	0.344	57.31
T0819TS133_2-D1.rsa	33.621	0.676	0	0.324	83	0.405	66.31
T0819TS410_2-D1.rsa	33.333	0.413	0.08	0.507	108	0.309	61.42
T0819TS117_4-D1.rsa	33.333	0.045	0.218	0.736	81	0.412	55.68
T0819TS011_5-D1.rsa	33.333	0.055	0.173	0.773	85	0.392	51.59
T0819TS345_1-D1.rsa	32.813	0.015	0.471	0.515	70	0.469	65.62
T0819TS160_2-D1.rsa	32.759	0.676	0	0.324	83	0.395	63.38
T0819TS436_5-D1.rsa	32.632	0.564	0.059	0.377	89	0.367	59.43
T0819TS110_5-D1.rsa	32.5	0	0.079	0.921	105	0.31	17.11
T0819TS381_1-D1.rsa	32.143	0.151	0	0.849	107	0.3	23.02
T0819TS448_4-D1.rsa	32	0.014	0.232	0.754	104	0.308	47.65
T0819TS454_3-D1.rsa	31.507	0.773	0	0.227	58	0.543	66.96
T0819TS454_4-D1.rsa	31.25	0.037	0.463	0.5	68	0.46	67.1
T0819TS300_4-D1.rsa	31.034	0.582	0	0.418	107	0.29	55.76
T0819TS212_1-D1.rsa	30.556	0.376	0.075	0.549	117	0.261	41.24
T0819TS335_5-D1.rsa	30.526	0.53	0.059	0.411	97	0.315	58.37
T0819TS346_1-D1.rsa	30.357	0.175	0.246	0.579	73	0.416	40.87
T0819TS251_4-D1.rsa	30.233	0.325	0.189	0.485	82	0.369	70.86
T0819TS228_4-D1.rsa	30.233	0.331	0.172	0.497	84	0.36	73.52
T0819TS041_3-D1.rsa	30.233	0.385	0.154	0.462	78	0.388	69.23
T0819TS216_5-D1.rsa	30.172	0.637	0	0.363	93	0.324	57.32
T0819TS452_1-D1.rsa	30	0	0.158	0.842	96	0.313	46.05
T0819TS184_3-D1.rsa	30	0	0.36	0.64	73	0.411	46.49
T0819TS050_1-D1.rsa	29.07	0.308	0.195	0.497	84	0.346	69.38
T0819TS277_1-D1.rsa	29.07	0.32	0.166	0.515	87	0.334	55.62
T0819TS117_5-D1.rsa	28.767	0.399	0.108	0.493	183	0.157	81.61
T0819TS011_1-D1.rsa	28.448	0.715	0	0.285	73	0.39	77.54
T0819TS156_5-D1.rsa	28	0	0.362	0.638	88	0.318	62.68
T0819TS349_2-D1.rsa	28	0.058	0.196	0.746	103	0.272	36.96
T0819TS117_1-D1.rsa	28	0.065	0.326	0.609	84	0.333	59.06
T0819TS499_2-D1.rsa	28	0.072	0.196	0.732	101	0.277	36.23
T0819TS279_1-D1.rsa	27.907	0.314	0.195	0.491	83	0.336	71.15
T0819TS268_3-D1.rsa	27.907	0.367	0.219	0.414	70	0.399	72.19
T0819TS300_3-D1.rsa	27.368	0.538	0.059	0.403	95	0.288	55.3
T0819TS381_2-D1.rsa	26.724	0.648	0	0.352	90	0.297	65.14
T0819TS145_3-D1.rsa	26.471	0.44	0.103	0.457	111	0.238	64.44
T0819TS479_3-D1.rsa	26.316	0.542	0.055	0.403	95	0.277	57.1
T0819TS133_4-D1.rsa	25.581	0.296	0.207	0.497	84	0.305	72.34
T0819TS277_3-D1.rsa	24	0.058	0.413	0.529	73	0.329	69.02

T0819TS479_5-D1.rsa	24	0.08	0.333	0.587	81	0.296	70.83
T0819TS335_1-D1.rsa	24	0.087	0.348	0.565	78	0.308	57.79
T0819TS171_1-D1.rsa	24	0.065	0.333	0.601	83	0.289	67.57
T0819TS008_5-D1.rsa	24	0.072	0.341	0.587	81	0.296	51.99
T0819TS349_5-D1.rsa	23.81	0.41	0.154	0.436	102	0.233	68.16
T0819TS436_1-D1.rsa	23	0.398	0.124	0.478	120	0.192	89.74
T0819TS156_1-D1.rsa	22.807	0.443	0.046	0.511	112	0.204	79.91
T0819TS420_1-D1.rsa	22.5	0	0.158	0.842	96	0.234	44.52
T0819TS410_5-D1.rsa	21.053	0.447	0.068	0.484	106	0.199	81.05
T0819TS145_5-D1.rsa	20.779	0.379	0.138	0.482	108	0.192	77.74
T0819TS251_5-D1.rsa	19.298	0.447	0.087	0.466	102	0.189	81.85
T0819TS228_3-D1.rsa	19.298	0.443	0.091	0.466	102	0.189	80.82
T0819TS216_2-D1.rsa	19.118	0.424	0.008	0.568	138	0.139	13.67
T0819TS436_3-D1.rsa	17.105	0.322	0.139	0.539	179	0.096	66.72
T0819TS041_2-D1.rsa	15.789	0.47	0.078	0.452	99	0.159	81.16
T0768TS436_3-D1.rsa	82.857	0.384	0	0.616	249	0.333	6.99
T0768TS452_3-D1.rsa	74	0.06	0	0.94	236	0.314	6.97
T0768TS073_1-D1.rsa	50.617	0.292	0	0.708	323	0.157	6.84
T0768TS268_2-D1.rsa	47.368	0.063	0	0.937	311	0.152	5.35
T0768TS041_2-D1.rsa	46.053	0.133	0.048	0.819	272	0.169	10.02
T0768TS492_2-D1.rsa	44.318	0	0.071	0.929	275	0.161	35.64
T0768TS117_4-D1.rsa	40.789	0.428	0	0.572	261	0.156	21.49
T0768TS492_5-D1.rsa	40.571	0.168	0.005	0.827	334	0.121	5.63
T0768TS499_2-D1.rsa	39.429	0.317	0.007	0.676	273	0.144	31.44
T0768TS452_5-D1.rsa	34.857	0.366	0.025	0.609	246	0.142	38.92
T0768TS073_2-D1.rsa	30.4	0.006	0.14	0.854	281	0.108	44.07
T0768TS156_4-D1.rsa	29.932	0.365	0.097	0.539	201	0.149	81.67
T0768TS038_1-D1.rsa	29.6	0.006	0.289	0.705	232	0.128	49.16
T0768TS145_5-D1.rsa	29.6	0.009	0.337	0.653	215	0.138	43.54
T0768TS268_4-D1.rsa	28.8	0.012	0.216	0.772	254	0.113	44.38
T0768TS041_5-D1.rsa	28.571	0.218	0.017	0.765	309	0.092	21.1
T0768TS171_1-D1.rsa	28.289	0.395	0	0.605	276	0.102	42.76
T0768TS436_2-D1.rsa	28	0.012	0.255	0.733	241	0.116	50.68
T0768TS145_1-D1.rsa	28	0.027	0.283	0.69	227	0.123	45.59
T0768TS452_1-D1.rsa	27.429	0.376	0.01	0.614	248	0.111	34.22
T0768TS335_4-D1.rsa	27.211	0.357	0.094	0.55	205	0.133	79.44
T0768TS499_1-D1.rsa	27.2	0.003	0.28	0.717	236	0.115	52.36
T0768TS251_3-D1.rsa	26.974	0.325	0	0.675	308	0.088	42.54
T0768TS145_4-D1.rsa	26.531	0.378	0.102	0.52	194	0.137	85.83
T0768TS381_4-D1.rsa	26.531	0.37	0.107	0.523	195	0.136	84.51
T0768TS263_3-D1.rsa	25.85	0.365	0.102	0.534	199	0.13	82.5
T0768TS454_2-D1.rsa	25.85	0.375	0.102	0.523	195	0.133	87.01
T0768TS038_4-D1.rsa	25.85	0.383	0.102	0.515	192	0.135	86.18
T0768TS038_3-D1.rsa	25.6	0.015	0.222	0.763	251	0.102	51.52
T0768TS156_5-D1.rsa	24.8	0.027	0.267	0.705	232	0.107	52.66
T0768TS335_2-D1.rsa	24.691	0.382	0.09	0.529	241	0.102	55.74
T0768TS436_4-D1.rsa	22.857	0.181	0.025	0.795	321	0.071	19.86

T0768TS452_4-D1.rsa	22.84	0.423	0.086	0.491	224	0.102	76.44
T0768TS206_1-D1.rsa	22.368	0.419	0	0.581	265	0.084	49.4
T0768TS171_2-D1.rsa	22.286	0.181	0	0.819	331	0.067	10.95
T0768TS414_1-D1.rsa	22.222	0.428	0.094	0.478	218	0.102	75.83
T0768TS452_2-D1.rsa	22.222	0.439	0.072	0.489	223	0.1	76.16
T0768TS184_3-D1.rsa	22.222	0.412	0.092	0.496	226	0.098	58.94
T0768TS381_2-D1.rsa	21.605	0.434	0.075	0.491	224	0.096	70.92
T0768TS414_2-D1.rsa	20.988	0.412	0.088	0.5	228	0.092	68.6
T0768TS156_1-D1.rsa	20.988	0.43	0.094	0.476	217	0.097	73.56
T0768TS041_4-D1.rsa	20.988	0.434	0.099	0.467	213	0.099	70.58
T0768TS414_5-D1.rsa	19.753	0.404	0.092	0.504	230	0.086	66.28
T0768TS263_4-D1.rsa	19.737	0.401	0.024	0.575	262	0.075	58.72
T0768TS492_4-D1.rsa	19.737	0.417	0	0.583	266	0.074	53.4
T0768TS038_5-D1.rsa	19.737	0.425	0.004	0.57	260	0.076	54.17
T0768TS145_2-D1.rsa	19.079	0.423	0.029	0.548	250	0.076	63.71
T0768TS228_5-D1.rsa	19.079	0.417	0.009	0.575	262	0.073	56.14
T0768TS335_1-D1.rsa	19.079	0.45	0.033	0.518	236	0.081	61.68
T0768TS210_1-D1.rsa	18.857	0.156	0.01	0.834	337	0.056	16.21
T0768TS263_1-D1.rsa	18.421	0.463	0.024	0.513	234	0.079	61.95
T0768TS414_3-D1.rsa	17.763	0.452	0.024	0.524	239	0.074	61.57
T0768TS345_3-D1.rsa	17.692	0.266	0.231	0.502	230	0.077	70.33
T0768TS381_1-D1.rsa	17.692	0.273	0.24	0.487	223	0.079	70.94
T0768TS381_3-D1.rsa	17.105	0.271	0.117	0.611	203	0.084	98.04
T0768TS041_1-D1.rsa	16.923	0.24	0.194	0.566	259	0.065	71.33
T0768TS277_3-D1.rsa	16.923	0.262	0.238	0.5	229	0.074	74.17
T0768TS345_2-D1.rsa	16.447	0.417	0.015	0.568	259	0.064	59.32
T0768TS345_1-D1.rsa	16.447	0.471	0.004	0.524	239	0.069	62.88
T0768TS277_1-D1.rsa	16.154	0.271	0.162	0.568	260	0.062	75.17
T0768TS345_5-D1.rsa	16.154	0.264	0.223	0.513	235	0.069	78.17
T0768TS436_5-D1.rsa	16.154	0.262	0.223	0.515	236	0.068	73.61
T0768TS345_4-D1.rsa	16.154	0.264	0.245	0.491	225	0.072	74.61
T0768TS216_1-D1.rsa	15.789	0.313	0.117	0.569	189	0.084	97.36
T0768TS216_3-D1.rsa	15.385	0.245	0.168	0.587	269	0.057	75.17
T0768TS499_5-D1.rsa	15.385	0.258	0.225	0.517	237	0.065	78.22
T0768TS381_5-D1.rsa	15.385	0.271	0.207	0.522	239	0.064	63
T0768TS414_4-D1.rsa	15.385	0.271	0.234	0.496	227	0.068	72.78
T0768TS277_4-D1.rsa	14.615	0.249	0.21	0.541	248	0.059	71.17
T0768TS145_3-D1.rsa	14.474	0.313	0.117	0.569	189	0.077	99.17
T0768TS268_1-D1.rsa	13.846	0.251	0.229	0.52	238	0.058	77.61
T0768TS050_1-D1.rsa	13.158	0.241	0.075	0.684	227	0.058	62.58
T0803TS345_2-D1.rsa	92	0.072	0	0.928	128	0.719	14.67
T0803TS358_1-D1.rsa	75	0	0.333	0.667	72	1.042	21.53
T0803TS197_2-D1.rsa	73.684	0.252	0.072	0.676	75	0.982	25.9
T0803TS157_2-D1.rsa	72.5	0.219	0.07	0.711	81	0.895	14.91
T0803TS479_1-D1.rsa	68.421	0.198	0.036	0.766	85	0.805	20.5
T0803TS279_1-D1.rsa	66.197	0.107	0.012	0.881	148	0.447	16.35
T0803TS479_2-D1.rsa	65.116	0.201	0	0.799	107	0.609	37.69

T0803TS301_4-D1.rsa	63.158	0.18	0.117	0.703	78	0.81	17.34
T0803TS479_5-D1.rsa	60.714	0	0.137	0.863	113	0.537	13.74
T0803TS328_1-D1.rsa	60	0.022	0.101	0.877	121	0.496	15.04
T0803TS116_1-D1.rsa	59.615	0.267	0.073	0.661	109	0.547	68.99
T0803TS290_1-D1.rsa	59.615	0.273	0.158	0.57	94	0.634	58.54
T0803TS268_2-D1.rsa	59.574	0.163	0.008	0.829	213	0.28	9.44
T0803TS433_4-D1.rsa	58.14	0.179	0	0.821	110	0.529	34.33
T0803TS110_4-D1.rsa	57.714	0.426	0.015	0.559	226	0.255	11.08
T0803TS364_1-D1.rsa	57.692	0.291	0.085	0.624	103	0.56	77.69
T0803TS097_2-D1.rsa	57.692	0.261	0.097	0.642	106	0.544	71.36
T0803TS300_3-D1.rsa	56.579	0.19	0	0.81	269	0.21	11.67
T0803TS132_5-D1.rsa	55.769	0.042	0	0.958	158	0.353	10.6
T0803TS097_4-D1.rsa	54.795	0.286	0.082	0.631	161	0.34	24.71
T0803TS144_5-D1.rsa	54.688	0.096	0.015	0.89	121	0.452	43.02
T0803TS032_2-D1.rsa	54.167	0	0.602	0.398	43	1.26	66.44
T0803TS360_4-D1.rsa	54.167	0	0.574	0.426	46	1.178	58.33
T0803TS116_4-D1.rsa	51.923	0.285	0.061	0.655	108	0.481	66.14
T0803TS210_3-D1.rsa	51.923	0.018	0.17	0.812	134	0.387	10.44
T0803TS241_3-D1.rsa	50	0	0.125	0.875	119	0.42	45.22
T0803TS132_2-D1.rsa	50	0	0.037	0.963	104	0.481	35.42
T0803TS479_4-D1.rsa	50	0.261	0.085	0.655	108	0.463	47.94
T0803TS042_3-D1.rsa	48.864	0.057	0.24	0.703	208	0.235	19.93
T0803TS276_5-D1.rsa	48.864	0	0.301	0.699	207	0.236	28.8
T0803TS204_1-D1.rsa	48.718	0.179	0.007	0.814	241	0.202	7.9
T0803TS097_5-D1.rsa	46.552	0	0.098	0.902	129	0.361	58.39
T0803TS290_2-D1.rsa	46.479	0.101	0.042	0.857	144	0.323	16.19
T0803TS360_2-D1.rsa	45.07	0.161	0.119	0.72	121	0.372	13.14
T0803TS204_2-D1.rsa	44.828	0	0.063	0.937	134	0.335	55.24
T0803TS006_3-D1.rsa	44.186	0.328	0.06	0.612	82	0.539	46.45
T0803TS454_3-D1.rsa	44	0.029	0.239	0.732	101	0.436	35.87
T0803TS116_2-D1.rsa	43.836	0.718	0	0.282	72	0.609	38.73
T0803TS063_2-D1.rsa	43.429	0.389	0.03	0.582	235	0.185	36.45
T0803TS338_1-D1.rsa	42.982	0.318	0.078	0.604	131	0.328	54.26
T0803TS333_2-D1.rsa	42.982	0.3	0.138	0.562	122	0.352	57.03
T0803TS228_1-D1.rsa	42.424	0.128	0.153	0.719	207	0.205	42.88
T0803TS358_2-D1.rsa	42.308	0	0.133	0.867	143	0.296	11.08
T0803TS038_1-D1.rsa	42.254	0.226	0.06	0.714	120	0.352	15.54
T0803TS044_2-D1.rsa	42.105	0.221	0.041	0.737	160	0.263	56.45
T0803TS391_2-D1.rsa	40.909	0.057	0.436	0.507	150	0.273	42.4
T0803TS097_3-D1.rsa	40.845	0	0.036	0.964	162	0.252	11.7
T0803TS080_2-D1.rsa	40.278	0.319	0.066	0.615	131	0.307	71.83
T0803TS483_1-D1.rsa	40	0.428	0.005	0.567	229	0.175	11.76
T0803TS258_2-D1.rsa	39.773	0.041	0.355	0.605	179	0.222	40.2
T0803TS333_3-D1.rsa	39.655	0	0.105	0.895	128	0.31	60.14
T0803TS145_3-D1.rsa	39.394	0.253	0.128	0.618	178	0.221	50.95
T0803TS277_5-D1.rsa	39.344	0.019	0.362	0.619	130	0.303	62.81
T0803TS454_4-D1.rsa	38.983	0.047	0.21	0.743	159	0.245	19.16

T0803TS056_1-D1.rsa	38.889	0.3	0.099	0.601	128	0.304	52.16
T0803TS317_3-D1.rsa	38.636	0.027	0.321	0.652	193	0.2	6.84
T0803TS120_1-D1.rsa	38.636	0.081	0.443	0.476	141	0.274	45.1
T0803TS184_5-D1.rsa	38.596	0.23	0.175	0.594	129	0.299	64.29
T0803TS110_2-D1.rsa	38.286	0.428	0.017	0.554	224	0.171	35.95
T0803TS336_2-D1.rsa	37.931	0	0.161	0.839	120	0.316	59.44
T0803TS277_3-D1.rsa	37.705	0	0.41	0.59	124	0.304	62.56
T0803TS145_4-D1.rsa	37.6	0	0.331	0.669	220	0.171	13.98
T0803TS433_1-D1.rsa	37.5	0.057	0.334	0.608	180	0.208	34.71
T0803TS144_4-D1.rsa	37.5	0.282	0.085	0.634	135	0.278	54.57
T0803TS338_3-D1.rsa	37.5	0.088	0.443	0.47	139	0.27	48.9
T0803TS216_1-D1.rsa	37.5	0.39	0.07	0.54	115	0.326	59.14
T0803TS326_1-D1.rsa	37.374	0.135	0.097	0.767	221	0.169	41.93
T0803TS499_3-D1.rsa	37.374	0.188	0.16	0.653	188	0.199	63.02
T0803TS237_1-D1.rsa	36.842	0.262	0.009	0.729	242	0.152	18.37
T0803TS006_4-D1.rsa	36.842	0.606	0.059	0.335	79	0.466	54.02
T0803TS454_2-D1.rsa	36.62	0.208	0.024	0.768	129	0.284	17.79
T0803TS157_1-D1.rsa	36.571	0.394	0.047	0.559	226	0.162	35.58
T0803TS420_3-D1.rsa	36.571	0.572	0	0.428	173	0.211	25.74
T0803TS358_5-D1.rsa	36.111	0.427	0.103	0.469	100	0.361	71.32
T0803TS044_3-D1.rsa	36.066	0.019	0.314	0.667	140	0.258	59.7
T0803TS064_2-D1.rsa	36.066	0.029	0.414	0.557	117	0.308	69.65
T0803TS080_1-D1.rsa	35.965	0.23	0.171	0.599	130	0.277	63.13
T0803TS080_5-D1.rsa	35.965	0.263	0.166	0.571	124	0.29	62.9
T0803TS241_2-D1.rsa	35.965	0.235	0.217	0.548	119	0.302	61.17
T0803TS336_1-D1.rsa	35.593	0	0.327	0.673	144	0.247	62.38
T0803TS439_1-D1.rsa	35.417	0.082	0.191	0.727	80	0.443	57.95
T0803TS210_4-D1.rsa	35.417	0.018	0.245	0.736	81	0.437	27.5
T0803TS346_1-D1.rsa	35.354	0.104	0.014	0.882	254	0.139	9.98
T0803TS110_5-D1.rsa	35.354	0.181	0.094	0.726	209	0.169	55.99
T0803TS173_4-D1.rsa	35.354	0.201	0.219	0.58	167	0.212	60.16
T0803TS391_5-D1.rsa	35.294	0.366	0.086	0.547	133	0.265	65.89
T0803TS216_4-D1.rsa	35.294	0.603	0	0.397	50	0.706	46.43
T0803TS014_5-D1.rsa	35.227	0.068	0.443	0.49	145	0.243	43.07
T0803TS335_1-D1.rsa	35.2	0	0.271	0.729	240	0.147	32.83
T0803TS251_4-D1.rsa	35.2	0	0.331	0.669	220	0.16	40.2
T0803TS153_2-D1.rsa	34.483	0	0.133	0.867	124	0.278	69.23
T0803TS260_2-D1.rsa	34.426	0.019	0.357	0.624	131	0.263	62.19
T0803TS132_4-D1.rsa	34.426	0.029	0.429	0.543	114	0.302	66.05
T0803TS258_3-D1.rsa	34.343	0.163	0.021	0.816	235	0.146	10.68
T0803TS483_3-D1.rsa	34.286	0.48	0.015	0.505	204	0.168	32.43
T0803TS210_5-D1.rsa	34.247	0.773	0	0.227	58	0.59	65.1
T0803TS251_2-D1.rsa	34.091	0.064	0.284	0.652	193	0.177	49.32
T0803TS260_5-D1.rsa	33.824	0.309	0.037	0.654	159	0.213	45.44
T0803TS216_2-D1.rsa	33.803	0.494	0.167	0.339	57	0.593	50.64
T0803TS155_2-D1.rsa	33.621	0.551	0	0.449	115	0.292	59.08
T0803TS258_4-D1.rsa	33.6	0	0.374	0.626	206	0.163	46.28

T0803TS063_1-D1.rsa	33.333	0.391	0.102	0.507	189	0.176	40.83
T0803TS204_5-D1.rsa	33.333	0.219	0.194	0.587	169	0.197	54.25
T0803TS216_3-D1.rsa	33.333	0.366	0.085	0.549	117	0.285	71.57
T0803TS347_3-D1.rsa	33.333	0.249	0.212	0.539	117	0.285	65.32
T0803TS442_1-D1.rsa	32.8	0.012	0.401	0.587	193	0.17	55.17
T0803TS258_1-D1.rsa	32.787	0.01	0.386	0.605	127	0.258	64.43
T0803TS439_3-D1.rsa	32.759	0.605	0	0.395	101	0.324	53.22
T0803TS251_5-D1.rsa	32.673	0.252	0.125	0.623	200	0.163	69.31
T0803TS155_5-D1.rsa	32.632	0.492	0.055	0.453	107	0.305	57.95
T0803TS420_1-D1.rsa	32.558	0.302	0.189	0.509	86	0.379	73.37
T0803TS032_1-D1.rsa	32.558	0.325	0.178	0.497	84	0.388	73.52
T0803TS301_1-D1.rsa	32.456	0.29	0.161	0.548	119	0.273	57.72
T0803TS282_3-D1.rsa	32.456	0.286	0.184	0.53	115	0.282	58.18
T0803TS499_4-D1.rsa	32.323	0.212	0.194	0.594	171	0.189	61.2
T0803TS169_5-D1.rsa	32.203	0.019	0.145	0.836	179	0.18	46.61
T0803TS064_3-D1.rsa	32.203	0.061	0.257	0.682	146	0.221	39.02
T0803TS006_1-D1.rsa	32.143	0.175	0.389	0.437	55	0.584	49.6
T0803TS145_5-D1.rsa	32	0.378	0.108	0.514	129	0.248	78.09
T0803TS479_3-D1.rsa	31.897	0.578	0	0.422	108	0.295	59.28
T0803TS276_4-D1.rsa	31.683	0.271	0.131	0.598	192	0.165	73.91
T0803TS301_2-D1.rsa	31.579	0.241	0.481	0.278	30	1.053	94.44
T0803TS110_3-D1.rsa	31.507	0.356	0.102	0.542	201	0.157	71.94
T0803TS067_3-D1.rsa	31.507	0.377	0.108	0.515	191	0.165	73.57
T0803TS006_2-D1.rsa	31.507	0.769	0	0.231	59	0.534	40.39
T0803TS338_2-D1.rsa	31.429	0.376	0.205	0.419	98	0.321	72.11
T0803TS414_1-D1.rsa	31.313	0.181	0.108	0.712	205	0.153	53.91
T0803TS296_5-D1.rsa	31.313	0.139	0.16	0.701	202	0.155	55.12
T0803TS347_4-D1.rsa	31.313	0.253	0.108	0.639	184	0.17	60.59
T0803TS155_4-D1.rsa	31.148	0.024	0.357	0.619	130	0.24	66.29
T0803TS132_3-D1.rsa	31.034	0	0.217	0.783	112	0.277	78.67
T0803TS362_1-D1.rsa	31.034	0	0.21	0.79	113	0.275	78.5
T0803TS080_3-D1.rsa	31	0.41	0.124	0.466	117	0.265	87.25
T0803TS038_3-D1.rsa	30.882	0.374	0.082	0.543	132	0.234	65.11
T0803TS064_5-D1.rsa	30.882	0.383	0.082	0.535	130	0.238	65
T0803TS445_3-D1.rsa	30.857	0.468	0.01	0.522	211	0.146	24.88
T0803TS349_4-D1.rsa	30.822	0.394	0.108	0.499	185	0.167	82.83
T0803TS063_3-D1.rsa	30.822	0.402	0.108	0.491	182	0.169	80.86
T0803TS067_5-D1.rsa	30.822	0.404	0.105	0.491	182	0.169	77.25
T0803TS160_3-D1.rsa	30.693	0.287	0.14	0.573	184	0.167	55.92
T0803TS160_4-D1.rsa	30.303	0.149	0.156	0.694	200	0.152	51.22
T0803TS300_1-D1.rsa	30.303	0.205	0.125	0.67	193	0.157	54.6
T0803TS042_5-D1.rsa	30.286	0.485	0.02	0.495	200	0.151	37.56
T0803TS328_5-D1.rsa	30.233	0.308	0.183	0.509	86	0.352	69.23
T0803TS445_2-D1.rsa	30.233	0.337	0.172	0.491	83	0.364	66.72
T0803TS368_4-D1.rsa	30.137	0.353	0.105	0.542	201	0.15	72.07
T0803TS333_1-D1.rsa	30.137	0.372	0.105	0.523	194	0.155	71.19
T0803TS317_4-D1.rsa	30.137	0.383	0.094	0.523	194	0.155	71.19

T0803TS360_5-D1.rsa	30	0	0.474	0.526	60	0.5	41.45
T0803TS120_3-D1.rsa	29.714	0.493	0	0.507	205	0.145	31
T0803TS034_3-D1.rsa	29.703	0.255	0.131	0.614	197	0.151	75.16
T0803TS034_2-D1.rsa	29.703	0.277	0.156	0.567	182	0.163	75.86
T0803TS448_5-D1.rsa	29.703	0.271	0.15	0.579	186	0.16	76.79
T0803TS268_4-D1.rsa	29.703	0.299	0.159	0.542	174	0.171	73.68
T0803TS436_5-D1.rsa	29.6	0.012	0.295	0.693	228	0.13	50.46
T0803TS011_4-D1.rsa	29.6	0.015	0.386	0.599	197	0.15	48.48
T0803TS439_5-D1.rsa	29.508	0.057	0.438	0.505	106	0.278	75.12
T0803TS116_5-D1.rsa	29.508	0.019	0.405	0.576	121	0.244	72.14
T0803TS153_1-D1.rsa	29.452	0.404	0.086	0.509	189	0.156	78
T0803TS067_1-D1.rsa	29.452	0.402	0.111	0.488	181	0.163	75.07
T0803TS340_2-D1.rsa	29.412	0.3	0.078	0.621	151	0.195	52.11
T0803TS044_4-D1.rsa	29.31	0.617	0	0.383	98	0.299	66.31
T0803TS391_1-D1.rsa	29.293	0.243	0.135	0.622	179	0.164	58.07
T0803TS425_5-D1.rsa	29.293	0.219	0.139	0.642	185	0.158	42.45
T0803TS439_2-D1.rsa	29.143	0.48	0.035	0.485	196	0.149	40.22
T0803TS044_5-D1.rsa	29.143	0.527	0	0.473	191	0.153	30.45
T0803TS347_5-D1.rsa	28.814	0	0.285	0.715	153	0.188	54.56
T0803TS410_4-D1.rsa	28.8	0.015	0.328	0.657	216	0.133	46.58
T0803TS041_3-D1.rsa	28.8	0.012	0.356	0.632	208	0.138	53.65
T0803TS333_4-D1.rsa	28.8	0.018	0.389	0.593	195	0.148	54.79
T0803TS160_1-D1.rsa	28.8	0.006	0.383	0.611	201	0.143	47.8
T0803TS263_5-D1.rsa	28.767	0.412	0.102	0.485	180	0.16	82.36
T0803TS251_3-D1.rsa	28.713	0.268	0.146	0.586	188	0.153	74.53
T0803TS034_4-D1.rsa	28.571	0.397	0.118	0.485	181	0.158	80.14
T0803TS425_1-D1.rsa	28.571	0.406	0.121	0.473	106	0.27	81.07
T0803TS347_1-D1.rsa	28.571	0.372	0.209	0.419	98	0.292	71.15
T0803TS483_2-D1.rsa	28.448	0.668	0	0.332	85	0.335	67.58
T0803TS153_3-D1.rsa	28.421	0.547	0.059	0.394	93	0.306	58.58
T0803TS263_4-D1.rsa	28.409	0.064	0.416	0.52	154	0.184	42.06
T0803TS336_4-D1.rsa	28.283	0.219	0.174	0.608	175	0.162	60.42
T0803TS118_3-D1.rsa	28.283	0.247	0.191	0.563	162	0.175	63.28
T0803TS368_2-D1.rsa	28.283	0.267	0.212	0.521	150	0.189	73.09
T0803TS080_4-D1.rsa	28.205	0.365	0.128	0.507	150	0.188	54.95
T0803TS237_5-D1.rsa	28.082	0.407	0.113	0.48	178	0.158	75.48
T0803TS162_2-D1.rsa	28	0.53	0.02	0.45	182	0.154	50.62
T0803TS290_4-D1.rsa	28	0.422	0.116	0.462	116	0.241	87.85
T0803TS290_3-D1.rsa	28	0.434	0.12	0.446	112	0.25	81.38
T0803TS360_1-D1.rsa	28	0.45	0.12	0.43	108	0.259	81.38
T0803TS328_2-D1.rsa	27.869	0.024	0.424	0.552	116	0.24	67.29
T0803TS184_1-D1.rsa	27.869	0.014	0.41	0.576	121	0.23	67.16
T0803TS228_5-D1.rsa	27.869	0.024	0.39	0.586	123	0.227	64.18
T0803TS338_5-D1.rsa	27.869	0.052	0.405	0.543	114	0.244	64.3
T0803TS349_3-D1.rsa	27.429	0.441	0.025	0.535	216	0.127	46.41
T0803TS260_3-D1.rsa	27.429	0.455	0.054	0.49	198	0.139	40.04
T0803TS041_1-D1.rsa	27.397	0.383	0.089	0.528	196	0.14	76.97

T0803TS162_3-D1.rsa	27.273	0.194	0.188	0.618	178	0.153	61.55
T0803TS317_5-D1.rsa	27.273	0.411	0.121	0.469	105	0.26	82.86
T0803TS454_1-D1.rsa	27.273	0.384	0.121	0.496	111	0.246	65.12
T0803TS452_3-D1.rsa	27.211	0.375	0.086	0.539	201	0.135	83.75
T0803TS162_5-D1.rsa	27.211	0.373	0.105	0.523	195	0.14	83.4
T0803TS322_5-D1.rsa	27.211	0.378	0.097	0.525	196	0.139	76.11
T0803TS117_3-D1.rsa	27.2	0.012	0.374	0.614	202	0.135	51.6
T0803TS014_1-D1.rsa	27.119	0	0.262	0.738	158	0.172	62.97
T0803TS362_4-D1.rsa	26.923	0.405	0.078	0.517	153	0.176	59.63
T0803TS499_1-D1.rsa	26.857	0.416	0.02	0.564	228	0.118	42.33
T0803TS403_5-D1.rsa	26.857	0.535	0.03	0.436	176	0.153	50.93
T0803TS235_1-D1.rsa	26.857	0.587	0.012	0.401	162	0.166	51.05
T0803TS492_3-D1.rsa	26.744	0.343	0.102	0.555	157	0.17	58.66
T0803TS290_5-D1.rsa	26.733	0.308	0.162	0.53	170	0.157	74.38
T0803TS038_5-D1.rsa	26.733	0.327	0.159	0.514	165	0.162	74.92
T0803TS157_3-D1.rsa	26.531	0.375	0.107	0.517	193	0.137	86.25
T0803TS414_5-D1.rsa	26.531	0.37	0.075	0.555	207	0.128	77.57
T0803TS433_3-D1.rsa	26.531	0.394	0.102	0.504	188	0.141	84.86
T0803TS326_5-D1.rsa	26.531	0.375	0.099	0.525	196	0.135	74.03
T0803TS326_3-D1.rsa	26.531	0.357	0.118	0.525	196	0.135	80.07
T0803TS008_4-D1.rsa	26.531	0.399	0.118	0.483	180	0.147	88.19
T0803TS277_2-D1.rsa	26.531	0.397	0.102	0.501	187	0.142	82.71
T0803TS336_5-D1.rsa	26.471	0.251	0.091	0.658	160	0.165	68.33
T0803TS228_2-D1.rsa	26.471	0.374	0.091	0.535	130	0.204	65.78
T0803TS317_2-D1.rsa	26.471	0.354	0.107	0.539	131	0.202	69
T0803TS144_1-D1.rsa	26.471	0.366	0.099	0.535	130	0.204	68.89
T0803TS144_3-D1.rsa	26.471	0.354	0.091	0.556	135	0.196	65.89
T0803TS347_2-D1.rsa	26.471	0.366	0.041	0.593	144	0.184	59.67
T0803TS360_3-D1.rsa	26.471	0.412	0.099	0.49	119	0.222	57.56
T0803TS410_1-D1.rsa	26.4	0.036	0.307	0.657	216	0.122	49.09
T0803TS410_5-D1.rsa	26.4	0.012	0.334	0.653	215	0.123	52.36
T0803TS448_3-D1.rsa	26.4	0.015	0.38	0.605	199	0.133	55.24
T0803TS120_2-D1.rsa	26.286	0.438	0.02	0.542	219	0.12	41.09
T0803TS203_3-D1.rsa	26.286	0.465	0.025	0.51	206	0.128	39.48
T0803TS403_1-D1.rsa	26.286	0.48	0.05	0.47	190	0.138	39.54
T0803TS368_3-D1.rsa	26.286	0.574	0.005	0.421	170	0.155	50.31
T0803TS492_1-D1.rsa	26.23	0.014	0.433	0.552	116	0.226	62.19
T0803TS228_4-D1.rsa	26.23	0	0.381	0.619	130	0.202	50.62
T0803TS300_4-D1.rsa	26.027	0.396	0.1	0.504	187	0.139	71.46
T0803TS445_1-D1.rsa	26.027	0.399	0.105	0.496	184	0.141	75.34
T0803TS073_2-D1.rsa	25.974	0.424	0.121	0.455	102	0.255	79.64
T0803TS171_5-D1.rsa	25.926	0.368	0.088	0.544	248	0.105	53.86
T0803TS414_3-D1.rsa	25.926	0.377	0.079	0.544	248	0.105	54.14
T0803TS322_2-D1.rsa	25.862	0.492	0	0.508	130	0.199	50.2
T0803TS235_4-D1.rsa	25.85	0.34	0.099	0.56	209	0.124	81.53
T0803TS133_2-D1.rsa	25.85	0.365	0.099	0.536	200	0.129	84.17
T0803TS349_5-D1.rsa	25.85	0.391	0.102	0.507	189	0.137	85.42

T0803TS381_5-D1.rsa	25.85	0.367	0.097	0.536	200	0.129	80.97
T0803TS067_4-D1.rsa	25.743	0.265	0.156	0.579	186	0.138	77.73
T0803TS263_1-D1.rsa	25.743	0.283	0.174	0.542	174	0.148	73.44
T0803TS014_2-D1.rsa	25.743	0.312	0.174	0.514	165	0.156	74.22
T0803TS117_1-D1.rsa	25.714	0.438	0.005	0.557	225	0.114	34.72
T0803TS203_1-D1.rsa	25.641	0.348	0.098	0.554	164	0.156	57.12
T0803TS210_2-D1.rsa	25.641	0.426	0.139	0.436	129	0.199	58.59
T0803TS263_2-D1.rsa	25.253	0.219	0.167	0.615	177	0.143	60.5
T0803TS357_1-D1.rsa	25.253	0.257	0.215	0.528	152	0.166	71.96
T0803TS117_5-D1.rsa	25.17	0.375	0.094	0.531	198	0.127	84.38
T0803TS133_3-D1.rsa	25.17	0.373	0.099	0.528	197	0.128	84.38
T0803TS118_4-D1.rsa	25.17	0.359	0.102	0.539	201	0.125	71.81
T0803TS041_5-D1.rsa	25.143	0.416	0.02	0.564	228	0.11	42.27
T0803TS260_1-D1.rsa	25.143	0.416	0.05	0.535	216	0.116	42.95
T0803TS268_5-D1.rsa	25.143	0.53	0.025	0.446	180	0.14	51.86
T0803TS011_1-D1.rsa	25.143	0.478	0.035	0.488	197	0.128	42.14
T0803TS420_4-D1.rsa	25.143	0.485	0.025	0.49	198	0.127	34.34
T0803TS011_2-D1.rsa	25	0.361	0.006	0.633	210	0.119	16.27
T0803TS391_4-D1.rsa	25	0.361	0	0.639	212	0.118	9.56
T0803TS425_2-D1.rsa	25	0.412	0.103	0.486	118	0.212	71.78
T0803TS169_4-D1.rsa	25	0.424	0.107	0.469	114	0.219	66.67
T0803TS173_1-D1.rsa	24.762	0.316	0.15	0.534	125	0.198	64.1
T0803TS197_1-D1.rsa	24.752	0.268	0.162	0.57	183	0.135	76.32
T0803TS042_4-D1.rsa	24.752	0.262	0.178	0.561	180	0.138	74.84
T0803TS276_1-D1.rsa	24.752	0.265	0.115	0.62	199	0.124	41.04
T0803TS260_4-D1.rsa	24.571	0.428	0.005	0.567	229	0.107	45.73
T0803TS282_4-D1.rsa	24.571	0.512	0.01	0.478	193	0.127	50.31
T0803TS162_1-D1.rsa	24.571	0.507	0.01	0.483	195	0.126	43.69
T0803TS492_4-D1.rsa	24.242	0.208	0.205	0.587	169	0.143	67.45
T0803TS345_4-D1.rsa	24.242	0.236	0.212	0.552	159	0.152	69.01
T0803TS433_2-D1.rsa	24	0.505	0.01	0.485	196	0.122	43.13
T0803TS008_2-D1.rsa	24	0.41	0.112	0.478	120	0.2	89.14
T0803TS362_3-D1.rsa	24	0.422	0.12	0.458	115	0.209	87.05
T0803TS153_5-D1.rsa	24	0.454	0.12	0.426	107	0.224	89.34
T0803TS499_2-D1.rsa	24	0	0.152	0.848	117	0.205	57.43
T0803TS277_1-D1.rsa	23.973	0.412	0.102	0.485	180	0.133	78.95
T0803TS117_4-D1.rsa	23.81	0.394	0.107	0.499	186	0.128	85.56
T0803TS433_5-D1.rsa	23.762	0.277	0.146	0.576	185	0.128	74.84
T0803TS442_2-D1.rsa	23.729	0	0.285	0.715	153	0.155	51.28
T0803TS425_3-D1.rsa	23.729	0	0.336	0.664	142	0.167	61.68
T0803TS197_5-D1.rsa	23.729	0.019	0.318	0.664	142	0.167	62.5
T0803TS157_4-D1.rsa	23.684	0.401	0.029	0.57	260	0.091	61.95
T0803TS204_4-D1.rsa	23.529	0.333	0.107	0.56	136	0.173	70
T0803TS042_1-D1.rsa	23.529	0.16	0.099	0.741	180	0.131	33.33
T0803TS436_1-D1.rsa	23.457	0.408	0.094	0.498	227	0.103	75.22
T0803TS277_4-D1.rsa	23.429	0.428	0.01	0.562	227	0.103	39.17
T0803TS197_4-D1.rsa	23.429	0.463	0.022	0.515	208	0.113	38.61

T0803TS173_2-D1.rsa	23.429	0.502	0.03	0.468	189	0.124	44.68
T0803TS442_5-D1.rsa	23.429	0.502	0.027	0.47	190	0.123	43.81
T0803TS403_4-D1.rsa	23.429	0.51	0.022	0.468	189	0.124	45.3
T0803TS038_4-D1.rsa	23.377	0.371	0.125	0.504	113	0.207	72.26
T0803TS322_3-D1.rsa	23.232	0.108	0.076	0.816	235	0.099	32.81
T0803TS120_5-D1.rsa	23.232	0.208	0.215	0.576	166	0.14	57.12
T0803TS171_2-D1.rsa	23.026	0.386	0.013	0.601	274	0.084	58.11
T0803TS132_1-D1.rsa	23	0.406	0.124	0.47	118	0.195	90.74
T0803TS237_4-D1.rsa	22.857	0.438	0.022	0.54	218	0.105	40.59
T0803TS445_4-D1.rsa	22.857	0.49	0.005	0.505	204	0.112	45.36
T0803TS322_4-D1.rsa	22.857	0.507	0.025	0.468	189	0.121	48.82
T0803TS064_1-D1.rsa	22.857	0.507	0.047	0.446	180	0.127	22.9
T0803TS445_5-D1.rsa	22.857	0.507	0.047	0.446	180	0.127	22.77
T0803TS381_4-D1.rsa	22.84	0.423	0.09	0.487	222	0.103	75
T0803TS197_3-D1.rsa	22.772	0.299	0.156	0.545	175	0.13	78.5
T0803TS483_4-D1.rsa	22.772	0.299	0.156	0.545	175	0.13	78.5
T0803TS063_4-D1.rsa	22.286	0.428	0.045	0.527	213	0.105	46.84
T0803TS171_3-D1.rsa	22.222	0.393	0.081	0.526	240	0.093	68.65
T0803TS452_2-D1.rsa	22.222	0.421	0.09	0.489	223	0.1	74.06
T0803TS338_4-D1.rsa	22.059	0.333	0.062	0.605	147	0.15	57.67
T0803TS116_3-D1.rsa	22.059	0.379	0.091	0.531	129	0.171	67.78
T0803TS228_3-D1.rsa	21.795	0.372	0.139	0.49	145	0.15	58.68
T0803TS173_3-D1.rsa	21.795	0.389	0.132	0.48	142	0.153	58.68
T0803TS014_4-D1.rsa	21.782	0.246	0.115	0.639	205	0.106	74.14
T0803TS203_4-D1.rsa	21.782	0.249	0.143	0.607	195	0.112	76.25
T0803TS160_5-D1.rsa	21.782	0.28	0.159	0.561	180	0.121	69.08
T0803TS063_5-D1.rsa	21.605	0.414	0.09	0.496	226	0.096	77.81
T0803TS133_1-D1.rsa	21.605	0.417	0.09	0.493	225	0.096	73.51
T0803TS212_1-D1.rsa	21.605	0.428	0.083	0.489	223	0.097	64.51
T0803TS014_3-D1.rsa	21.605	0.436	0.105	0.458	209	0.103	67.88
T0803TS073_1-D1.rsa	21.311	0.014	0.424	0.562	118	0.181	73.63
T0803TS034_1-D1.rsa	21.233	0.399	0.105	0.496	184	0.115	75.41
T0803TS492_2-D1.rsa	21.143	0.47	0.017	0.512	207	0.102	37.81
T0803TS301_3-D1.rsa	21.143	0.507	0.01	0.483	195	0.108	8.85
T0803TS133_5-D1.rsa	21.053	0.19	0.024	0.786	261	0.081	44.65
T0803TS067_2-D1.rsa	21.053	0.22	0.06	0.72	239	0.088	44.95
T0803TS008_5-D1.rsa	21.053	0.265	0.069	0.666	221	0.095	56.25
T0803TS358_3-D1.rsa	21.053	0.356	0.091	0.553	121	0.174	57.42
T0803TS381_3-D1.rsa	20.988	0.419	0.09	0.491	224	0.094	74.12
T0803TS436_2-D1.rsa	20.988	0.434	0.09	0.476	217	0.097	75.5
T0803TS041_4-D1.rsa	20.988	0.423	0.088	0.489	223	0.094	65.07
T0803TS203_2-D1.rsa	20.988	0.447	0.09	0.463	211	0.099	54.41
T0803TS263_3-D1.rsa	20.93	0.375	0.11	0.516	146	0.143	72
T0803TS145_2-D1.rsa	20.588	0.21	0.029	0.761	185	0.111	40
T0803TS317_1-D1.rsa	20.571	0.465	0.005	0.53	214	0.096	46.78
T0803TS268_3-D1.rsa	20.571	0.507	0.005	0.488	197	0.104	47.59
T0803TS049_3-D1.rsa	20.395	0.447	0.02	0.533	243	0.084	65.08

T0803TS349_1-D1.rsa	20.37	0.425	0.103	0.471	215	0.095	73.4
T0803TS184_3-D1.rsa	20.339	0	0.327	0.673	144	0.141	64.72
T0803TS296_4-D1.rsa	19.802	0.212	0.121	0.667	214	0.093	52.18
T0803TS050_1-D1.rsa	19.767	0.392	0.124	0.484	137	0.144	83.3
T0803TS117_2-D1.rsa	19.753	0.384	0.072	0.544	248	0.08	62.86
T0803TS358_4-D1.rsa	19.298	0.452	0.068	0.479	105	0.184	79.68
T0803TS328_3-D1.rsa	19.298	0.429	0.1	0.47	103	0.187	78.2
T0803TS268_1-D1.rsa	18.644	0.056	0.22	0.724	155	0.12	54.09
T0803TS237_3-D1.rsa	18.519	0.419	0.094	0.487	222	0.083	66.28
T0803TS452_1-D1.rsa	18.421	0.441	0.024	0.535	244	0.075	57.51
T0803TS157_5-D1.rsa	18.421	0.304	0.114	0.581	193	0.095	97.89
T0803TS420_5-D1.rsa	18.421	0.322	0.117	0.56	186	0.099	94.95
T0803TS097_1-D1.rsa	17.822	0.212	0.112	0.676	217	0.082	52.8
T0803TS133_4-D1.rsa	17.763	0.452	0.024	0.524	239	0.074	61.51
T0803TS452_4-D1.rsa	17.763	0.447	0.029	0.524	239	0.074	64.69
T0803TS335_4-D1.rsa	17.692	0.262	0.236	0.502	230	0.077	70.28
T0803TS410_2-D1.rsa	17.284	0.432	0.092	0.476	217	0.08	70.53
T0803TS326_4-D1.rsa	17.105	0.419	0.029	0.553	252	0.068	65.02
T0803TS452_5-D1.rsa	17.105	0.436	0.029	0.535	244	0.07	63.32
T0803TS237_2-D1.rsa	17.105	0.34	0.102	0.557	185	0.092	98.27
T0803TS300_2-D1.rsa	17.105	0.238	0.108	0.654	217	0.079	60.32
T0803TS448_2-D1.rsa	17.105	0.346	0.123	0.53	176	0.097	98.27
T0803TS328_4-D1.rsa	16.949	0	0.248	0.752	161	0.105	24.88
T0803TS041_2-D1.rsa	16.923	0.271	0.186	0.544	249	0.068	75.22
T0803TS410_3-D1.rsa	16.667	0.384	0.096	0.52	237	0.07	49.89
T0803TS049_4-D1.rsa	16.447	0.43	0.011	0.559	255	0.064	66.12
T0803TS042_2-D1.rsa	16.279	0.392	0.078	0.53	150	0.109	73.59
T0803TS049_5-D1.rsa	16.154	0.262	0.212	0.526	241	0.067	70.78
T0803TS381_1-D1.rsa	16.154	0.269	0.245	0.487	223	0.072	76.5
T0803TS282_5-D1.rsa	15.789	0.346	0.123	0.53	176	0.09	98.8
T0803TS162_4-D1.rsa	15.789	0.343	0.148	0.509	169	0.093	99.17
T0803TS349_2-D1.rsa	15.789	0.349	0.142	0.509	169	0.093	98.95
T0803TS296_1-D1.rsa	15.789	0.301	0.057	0.642	213	0.074	49.32
T0803TS171_4-D1.rsa	15.385	0.234	0.218	0.548	251	0.061	73.22
T0803TS049_1-D1.rsa	15.385	0.253	0.229	0.517	237	0.065	74.94
T0803TS335_5-D1.rsa	15.385	0.271	0.225	0.504	231	0.067	73.5
T0803TS414_4-D1.rsa	15.385	0.262	0.238	0.5	229	0.067	74.17
T0803TS235_3-D1.rsa	15.385	0.266	0.24	0.493	226	0.068	70.39
T0803TS155_3-D1.rsa	15.254	0.019	0.243	0.738	158	0.097	63.32
T0803TS282_2-D1.rsa	14.894	0.327	0.198	0.475	122	0.122	84.92
T0803TS492_5-D1.rsa	14.894	0.331	0.167	0.502	129	0.115	82.1
T0803TS206_1-D1.rsa	14.474	0.352	0.123	0.524	174	0.083	99.25
T0803TS008_1-D1.rsa	14.035	0.457	0.091	0.452	99	0.142	78.65
T0803TS006_5-D1.rsa	14.035	0.452	0.1	0.447	98	0.143	81.39
T0803TS381_2-D1.rsa	13.846	0.266	0.227	0.507	232	0.06	78.22
T0803TS335_3-D1.rsa	13.846	0.269	0.234	0.498	228	0.061	74.44
T0803TS235_2-D1.rsa	13.158	0.289	0.108	0.602	200	0.066	96.69

T0803TS448_4-D1.rsa	13.158	0.328	0.108	0.563	187	0.07	99.4
T0803TS420_2-D1.rsa	13.158	0.343	0.105	0.551	183	0.072	90.14
T0803TS434_1-D1.rsa	13.158	0.346	0.117	0.536	178	0.074	99.4
T0803TS296_3-D1.rsa	13.158	0.337	0.123	0.539	179	0.074	98.19
T0803TS499_5-D1.rsa	13.158	0.349	0.142	0.509	169	0.078	94.95
T0803TS011_3-D1.rsa	13.158	0.343	0.136	0.521	173	0.076	94.73
T0803TS335_2-D1.rsa	13.077	0.262	0.249	0.489	224	0.058	76.11
T0803TS160_2-D1.rsa	12.766	0.307	0.175	0.518	133	0.096	82
T0803TS241_1-D1.rsa	12.766	0.35	0.156	0.494	127	0.101	85.21
T0803TS044_1-D1.rsa	12.766	0.35	0.191	0.459	118	0.108	84.92
T0803TS171_1-D1.rsa	12.308	0.255	0.218	0.526	241	0.051	70.83
T0803TS011_5-D1.rsa	11.842	0.316	0.084	0.599	199	0.06	86.14
T0803TS430_1-D1.rsa	11.842	0.346	0.117	0.536	178	0.067	98.19
T0803TS362_5-D1.rsa	11.842	0.319	0.142	0.539	179	0.066	98.57
T0803TS212_2-D1.rsa	11.842	0.331	0.136	0.533	177	0.067	99.02
T0803TS296_2-D1.rsa	11.842	0.355	0.123	0.521	173	0.068	97.21
T0803TS483_5-D1.rsa	10.638	0.319	0.175	0.506	130	0.082	85.31
T0803TS442_4-D1.rsa	8.511	0.331	0.183	0.486	125	0.068	82.1
T0803TS425_4-D1.rsa	6.383	0.339	0.187	0.475	122	0.052	80.84
T0782TS345_2-D1.rsa	85.294	0.286	0	0.714	90	0.948	27.38
T0782TS184_2-D1.rsa	65.789	0.261	0	0.739	82	0.802	20.5
T0782TS420_5-D1.rsa	65.385	0.248	0.036	0.715	118	0.554	65.51
T0782TS011_4-D1.rsa	65.116	0.201	0.015	0.784	105	0.62	40.67
T0782TS268_4-D1.rsa	64.789	0.196	0.012	0.792	133	0.487	13.14
T0782TS216_4-D1.rsa	63.38	0.202	0.03	0.768	129	0.491	12.66
T0782TS335_5-D1.rsa	61.538	0.291	0.139	0.57	94	0.655	74.84
T0782TS479_2-D1.rsa	60.417	0	0.182	0.818	90	0.671	43.64
T0782TS216_5-D1.rsa	57.895	0.288	0	0.712	79	0.733	16.22
T0782TS479_4-D1.rsa	57.692	0.273	0.115	0.612	101	0.571	76.58
T0782TS216_3-D1.rsa	55.769	0.261	0.012	0.727	120	0.465	62.02
T0782TS452_4-D1.rsa	55.769	0.309	0.085	0.606	100	0.558	78.17
T0782TS452_3-D1.rsa	55.769	0.267	0.085	0.648	107	0.521	71.36
T0782TS335_3-D1.rsa	55.769	0.321	0.133	0.545	90	0.62	72.94
T0782TS160_2-D1.rsa	51.163	0.163	0.018	0.82	232	0.221	8.22
T0782TS156_4-D1.rsa	50.286	0.448	0	0.552	223	0.225	10.89
T0782TS448_2-D1.rsa	49.351	0.143	0	0.857	192	0.257	10
T0782TS212_1-D1.rsa	48.837	0.119	0.097	0.784	105	0.465	28.17
T0782TS184_1-D1.rsa	48.438	0.059	0.316	0.625	85	0.57	12.68
T0782TS145_3-D1.rsa	48	0.058	0.275	0.667	92	0.522	36.05
T0782TS184_3-D1.rsa	47.5	0	0.368	0.632	72	0.66	21.71
T0782TS073_1-D1.rsa	46.875	0.015	0.147	0.838	114	0.411	52.57
T0782TS420_3-D1.rsa	46.809	0.183	0.012	0.805	207	0.226	9.14
T0782TS492_2-D1.rsa	46.552	0	0.098	0.902	129	0.361	59.62
T0782TS492_3-D1.rsa	46.552	0	0.098	0.902	129	0.361	44.41
T0782TS038_2-D1.rsa	46.429	0	0.153	0.847	111	0.418	51.34
T0782TS268_1-D1.rsa	46.429	0	0.214	0.786	103	0.451	51.15
T0782TS345_3-D1.rsa	46.154	0.303	0.097	0.6	99	0.466	54.59

T0782TS117_1-D1.rsa	45.6	0	0.17	0.83	273	0.167	21.66
T0782TS454_2-D1.rsa	45	0	0.316	0.684	78	0.577	22.15
T0782TS436_3-D1.rsa	42.105	0.323	0.055	0.622	135	0.312	57.83
T0782TS410_1-D1.rsa	41.667	0.46	0.085	0.455	97	0.43	65.1
T0782TS145_1-D1.rsa	41.667	0.091	0.182	0.727	80	0.521	51.14
T0782TS381_1-D1.rsa	41.379	0	0.133	0.867	124	0.334	59.44
T0782TS228_1-D1.rsa	40.278	0.362	0.075	0.563	120	0.336	64.72
T0782TS171_5-D1.rsa	40	0.012	0.228	0.76	250	0.16	16.72
T0782TS041_4-D1.rsa	40	0.577	0.015	0.408	165	0.242	11.32
T0782TS210_2-D1.rsa	39.655	0	0.14	0.86	123	0.322	58.39
T0782TS145_2-D1.rsa	39.655	0.028	0.245	0.727	104	0.381	50.17
T0782TS410_2-D1.rsa	39.655	0.566	0	0.434	111	0.357	53.12
T0782TS448_1-D1.rsa	38.983	0.033	0.266	0.701	150	0.26	56.08
T0782TS335_1-D1.rsa	38.889	0.38	0.085	0.535	114	0.341	69.67
T0782TS110_4-D1.rsa	37.714	0.327	0	0.673	272	0.139	8.11
T0782TS228_3-D1.rsa	37.5	0.31	0.103	0.587	125	0.3	69.8
T0782TS011_3-D1.rsa	37.5	0.38	0.07	0.549	117	0.321	61.55
T0782TS216_2-D1.rsa	37.209	0.29	0.13	0.58	98	0.38	65.24
T0782TS448_5-D1.rsa	36.842	0.263	0.157	0.581	126	0.292	61.98
T0782TS184_4-D1.rsa	36	0.014	0.348	0.638	88	0.409	51.09
T0782TS216_1-D1.rsa	34.884	0.321	0.082	0.597	80	0.436	45.15
T0782TS041_5-D1.rsa	34.426	0.019	0.371	0.61	128	0.269	62.56
T0782TS448_3-D1.rsa	34.286	0.535	0.005	0.46	186	0.184	9.1
T0782TS349_3-D1.rsa	34.091	0.074	0.402	0.524	155	0.22	37.42
T0782TS160_3-D1.rsa	33.333	0.413	0.085	0.502	107	0.312	70.94
T0782TS110_2-D1.rsa	32.8	0	0.307	0.693	228	0.144	40.2
T0782TS452_5-D1.rsa	32.8	0.015	0.392	0.593	195	0.168	53.04
T0782TS011_1-D1.rsa	32.787	0.043	0.476	0.481	101	0.325	71.89
T0782TS479_5-D1.rsa	32.759	0.609	0	0.391	100	0.328	57.23
T0782TS251_5-D1.rsa	32.673	0.218	0.131	0.651	209	0.156	75.23
T0782TS414_1-D1.rsa	32.571	0.579	0	0.421	170	0.192	33.35
T0782TS420_2-D1.rsa	32.192	0.369	0.105	0.526	195	0.165	71.19
T0782TS210_3-D1.rsa	32	0.579	0.005	0.416	168	0.19	27.6
T0782TS133_3-D1.rsa	31.507	0.385	0.113	0.501	186	0.169	78.27
T0782TS492_1-D1.rsa	30.882	0.374	0.082	0.543	132	0.234	65.11
T0782TS038_3-D1.rsa	30.682	0.054	0.341	0.605	179	0.171	35.22
T0782TS050_1-D1.rsa	30.526	0.517	0.059	0.424	100	0.305	57.2
T0782TS492_4-D1.rsa	30.476	0.325	0.171	0.504	118	0.258	70.51
T0782TS110_3-D1.rsa	30.4	0.012	0.267	0.72	237	0.128	28.65
T0782TS454_5-D1.rsa	30.233	0.269	0.037	0.694	93	0.325	39.37
T0782TS349_4-D1.rsa	29.703	0.296	0.187	0.517	166	0.179	73.91
T0782TS145_4-D1.rsa	29.524	0.376	0.15	0.474	111	0.266	67.42
T0782TS228_2-D1.rsa	29.293	0.243	0.184	0.573	165	0.178	65.97
T0782TS277_5-D1.rsa	29.293	0.233	0.188	0.58	167	0.175	67.62
T0782TS210_5-D1.rsa	29	0.382	0.112	0.506	127	0.228	88.45
T0782TS454_1-D1.rsa	28.947	0.176	0.25	0.574	62	0.467	65.28
T0782TS414_3-D1.rsa	28.814	0.009	0.304	0.687	147	0.196	62.73

T0782TS263_4-D1.rsa	28.713	0.252	0.137	0.611	196	0.146	73.75
T0782TS277_2-D1.rsa	28.571	0.299	0.205	0.496	116	0.246	70.62
T0782TS499_3-D1.rsa	28.283	0.194	0.215	0.59	170	0.166	65.02
T0782TS008_3-D1.rsa	28.283	0.222	0.167	0.611	176	0.161	60.68
T0782TS420_1-D1.rsa	28.283	0.271	0.226	0.503	145	0.195	73
T0782TS410_4-D1.rsa	28.082	0.396	0.108	0.496	184	0.153	70.03
T0782TS436_5-D1.rsa	28	0.309	0.01	0.681	275	0.102	23.58
T0782TS210_4-D1.rsa	27.869	0.038	0.419	0.543	114	0.244	69.53
T0782TS300_5-D1.rsa	27.723	0.215	0.168	0.617	198	0.14	74.61
T0782TS160_5-D1.rsa	27.5	0	0.018	0.982	112	0.246	15.79
T0782TS237_3-D1.rsa	27.368	0.453	0.051	0.496	117	0.234	56.78
T0782TS277_3-D1.rsa	27.273	0.208	0.215	0.576	166	0.164	68.66
T0782TS499_1-D1.rsa	27.273	0.25	0.198	0.552	159	0.172	69.53
T0782TS452_1-D1.rsa	27.273	0.233	0.191	0.576	166	0.164	61.98
T0782TS335_2-D1.rsa	27.273	0.233	0.198	0.569	164	0.166	65.97
T0782TS251_4-D1.rsa	27.211	0.373	0.094	0.534	199	0.137	83.75
T0782TS008_2-D1.rsa	27.211	0.386	0.097	0.517	193	0.141	84.24
T0782TS038_5-D1.rsa	27.2	0.036	0.258	0.705	232	0.117	32.07
T0782TS171_2-D1.rsa	27.2	0.012	0.374	0.614	202	0.135	53.95
T0782TS011_2-D1.rsa	26.923	0.389	0.132	0.48	142	0.19	58.85
T0782TS171_3-D1.rsa	26.531	0.399	0.102	0.499	186	0.143	86.88
T0782TS156_3-D1.rsa	26.531	0.391	0.107	0.501	187	0.142	85.14
T0782TS277_4-D1.rsa	26.4	0.006	0.392	0.602	198	0.133	56.16
T0782TS499_5-D1.rsa	26.263	0.247	0.208	0.545	157	0.167	69.88
T0782TS038_1-D1.rsa	26.23	0.01	0.4	0.59	124	0.212	63.31
T0782TS279_1-D1.rsa	26.027	0.423	0.111	0.466	173	0.15	79.63
T0782TS041_3-D1.rsa	26	0.402	0.12	0.478	120	0.217	90.04
T0782TS133_4-D1.rsa	25.85	0.383	0.115	0.501	187	0.138	79.03
T0782TS300_1-D1.rsa	25.85	0.391	0.118	0.491	183	0.141	76.81
T0782TS300_3-D1.rsa	25.743	0.283	0.034	0.682	219	0.118	72.2
T0782TS410_5-D1.rsa	25.743	0.321	0.137	0.542	174	0.148	78.04
T0782TS436_4-D1.rsa	25.6	0.024	0.292	0.684	225	0.114	52.05
T0782TS381_2-D1.rsa	25.342	0.396	0.108	0.496	184	0.138	79.43
T0782TS499_4-D1.rsa	25.253	0.267	0.219	0.514	148	0.171	72.05
T0782TS277_1-D1.rsa	25	0.354	0.082	0.564	137	0.182	65.89
T0782TS479_3-D1.rsa	25	0.371	0.089	0.54	115	0.217	51.27
T0782TS156_2-D1.rsa	24.752	0.265	0.15	0.586	188	0.132	72.35
T0782TS268_3-D1.rsa	24.561	0.356	0.091	0.553	121	0.203	61.99
T0782TS448_4-D1.rsa	24.49	0.402	0.131	0.466	174	0.141	73.54
T0782TS160_4-D1.rsa	24.242	0.236	0.212	0.552	159	0.152	69.1
T0782TS171_4-D1.rsa	23.684	0.211	0.09	0.699	232	0.102	48.34
T0782TS414_2-D1.rsa	23.429	0.532	0.015	0.453	183	0.128	44.68
T0782TS349_2-D1.rsa	23.377	0.384	0.112	0.504	113	0.207	79.29
T0782TS381_3-D1.rsa	23.377	0.379	0.121	0.5	112	0.209	78.33
T0782TS410_3-D1.rsa	22.093	0.392	0.113	0.495	140	0.158	82.16
T0782TS479_1-D1.rsa	22.078	0.384	0.125	0.491	110	0.201	77.62
T0782TS237_1-D1.rsa	22.059	0.436	0.107	0.457	111	0.199	67.78

T0782TS346_1-D1.rsa	22	0.442	0.116	0.442	111	0.198	88.25
T0782TS492_5-D1.rsa	21.795	0.378	0.128	0.493	146	0.149	57.38
T0782TS171_1-D1.rsa	21.605	0.362	0.088	0.55	251	0.086	47.85
T0782TS237_2-D1.rsa	21.605	0.401	0.092	0.507	231	0.094	54.41
T0782TS110_5-D1.rsa	21.143	0.319	0.02	0.661	267	0.079	23.33
T0782TS436_1-D1.rsa	20.93	0.389	0.113	0.498	141	0.148	79.95
T0782TS008_1-D1.rsa	20.588	0.37	0.095	0.535	130	0.158	68.22
T0782TS268_5-D1.rsa	20.513	0.405	0.132	0.463	137	0.15	59.03
T0782TS145_5-D1.rsa	20	0.051	0.304	0.645	89	0.225	63.04
T0782TS156_5-D1.rsa	19.802	0.237	0.14	0.623	200	0.099	73.68
T0782TS436_2-D1.rsa	19.767	0.367	0.085	0.548	155	0.128	76.41
T0782TS251_3-D1.rsa	19.079	0.476	0.024	0.5	228	0.084	64.36
T0782TS210_1-D1.rsa	18.605	0.371	0.124	0.505	143	0.13	73.85
T0782TS452_2-D1.rsa	18.605	0.406	0.117	0.477	135	0.138	81.8
T0782TS160_1-D1.rsa	18.421	0.343	0.117	0.539	179	0.103	93.83
T0782TS381_5-D1.rsa	18.182	0.366	0.121	0.513	115	0.158	76.55
T0782TS345_5-D1.rsa	17.544	0.434	0.078	0.489	107	0.164	79.8
T0782TS011_5-D1.rsa	17.544	0.429	0.091	0.479	105	0.167	81.62
T0782TS300_2-D1.rsa	17.105	0.434	0.013	0.553	252	0.068	61.13
T0782TS133_5-D1.rsa	17.105	0.301	0.142	0.557	185	0.092	94.73
T0782TS117_4-D1.rsa	16.923	0.266	0.153	0.581	266	0.064	73.39
T0782TS300_4-D1.rsa	16.154	0.24	0.203	0.557	255	0.063	72.56
T0782TS237_4-D1.rsa	15.385	0.247	0.188	0.566	259	0.059	71.28
T0782TS420_4-D1.rsa	14.894	0.311	0.093	0.595	153	0.097	80.16
T0782TS117_3-D1.rsa	14.615	0.264	0.229	0.507	232	0.063	75
T0782TS414_4-D1.rsa	14.474	0.325	0.123	0.551	183	0.079	99.4
T0782TS133_1-D1.rsa	13.158	0.316	0.123	0.56	186	0.071	93.75
T0782TS268_2-D1.rsa	12.766	0.323	0.152	0.525	135	0.095	76.07
T0782TS349_5-D1.rsa	10.638	0.342	0.183	0.475	122	0.087	85.7
T0782TS414_5-D1.rsa	10.169	0	0.168	0.832	178	0.057	54.44
T0889TS464_2-D1.rsa	89.32	0.13	0	0.87	208	0.429	10.36
T0889TS464_5-D1.rsa	75.728	0.364	0	0.636	152	0.498	11.82
T0889TS321_1-D1.rsa	53.398	0.431	0.038	0.531	127	0.42	15.79
T0889TS321_3-D1.rsa	52.427	0.435	0.038	0.527	126	0.416	15.06
T0889TS321_5-D1.rsa	52.427	0.452	0.038	0.51	122	0.43	15.38
T0889TS321_4-D1.rsa	50.485	0.427	0.054	0.519	124	0.407	16.32
T0889TS321_2-D1.rsa	50.485	0.435	0.038	0.527	126	0.401	14.96
T0889TS451_2-D1.rsa	45.631	0.418	0.084	0.498	119	0.383	57.43
T0889TS451_4-D1.rsa	41.748	0.431	0.088	0.481	115	0.363	54.71
T0889TS451_5-D1.rsa	40.777	0.431	0.096	0.473	113	0.361	54.6
T0889TS451_1-D1.rsa	40.777	0.427	0.088	0.485	116	0.352	55.23
T0889TS455_5-D1.rsa	38.835	0.46	0.109	0.431	103	0.377	67.89
T0889TS434_3-D1.rsa	37.864	0.418	0	0.582	139	0.272	20.92
T0889TS434_2-D1.rsa	37.864	0.46	0	0.54	129	0.294	17.36
T0889TS434_4-D1.rsa	37.864	0.452	0	0.548	131	0.289	16.95
T0889TS451_3-D1.rsa	37.864	0.431	0.096	0.473	113	0.335	56.59
T0889TS180_5-D1.rsa	33.981	0.423	0.042	0.536	128	0.265	44.04

T0889TS455_4-D1.rsa	32.039	0.23	0.117	0.653	156	0.205	56.7
T0889TS452_1-D1.rsa	31.068	0.464	0.121	0.414	99	0.314	84.94
T0889TS434_1-D1.rsa	31.068	0.402	0	0.598	143	0.217	17.57
T0889TS275_5-D1.rsa	30.097	0.46	0.121	0.418	100	0.301	85.46
T0889TS425_1-D1.rsa	29.126	0.435	0.134	0.431	103	0.283	78.35
T0889TS452_4-D1.rsa	29.126	0.452	0.121	0.427	102	0.286	80.23
T0889TS183_2-D1.rsa	29.126	0.444	0.088	0.469	112	0.26	80.75
T0889TS359_1-D1.rsa	28.155	0.456	0.121	0.423	101	0.279	84.73
T0889TS275_4-D1.rsa	28.155	0.435	0.121	0.444	106	0.266	83.68
T0889TS275_1-D1.rsa	28.155	0.46	0.13	0.41	98	0.287	83.68
T0889TS455_3-D1.rsa	28.155	0.469	0.117	0.414	99	0.284	72.8
T0889TS405_1-D1.rsa	28.155	0.469	0.121	0.41	98	0.287	85.04
T0889TS421_1-D1.rsa	28.155	0.427	0.121	0.452	108	0.261	75
T0889TS077_5-D1.rsa	28.155	0.435	0.134	0.431	103	0.273	78.35
T0889TS313_4-D1.rsa	28.155	0.444	0.088	0.469	112	0.251	80.75
T0889TS479_2-D1.rsa	28.155	0.431	0.105	0.464	111	0.254	80.13
T0889TS425_5-D1.rsa	28.155	0.41	0.134	0.456	109	0.258	78.35
T0889TS425_4-D1.rsa	28.155	0.435	0.134	0.431	103	0.273	77.72
T0889TS275_3-D1.rsa	28.155	0.456	0.138	0.406	97	0.29	83.68
T0889TS425_3-D1.rsa	28.155	0.406	0.134	0.46	110	0.256	78.66
T0889TS251_2-D1.rsa	28.155	0.427	0.117	0.456	109	0.258	82.74
T0889TS382_2-D1.rsa	28.155	0.414	0.126	0.46	110	0.256	66.53
T0889TS434_5-D1.rsa	28.155	0.381	0	0.619	148	0.19	23.12
T0889TS048_1-D1.rsa	27.184	0.469	0.126	0.406	97	0.28	80.86
T0889TS026_4-D1.rsa	27.184	0.439	0.13	0.431	103	0.264	74.27
T0889TS313_5-D1.rsa	27.184	0.444	0.088	0.469	112	0.243	81.28
T0889TS464_1-D1.rsa	27.184	0.444	0.134	0.423	101	0.269	79.6
T0889TS026_1-D1.rsa	27.184	0.452	0.109	0.439	105	0.259	84.73
T0889TS452_2-D1.rsa	27.184	0.431	0.121	0.448	107	0.254	75.11
T0889TS357_1-D1.rsa	27.184	0.372	0	0.628	150	0.181	75.52
T0889TS464_4-D1.rsa	27.184	0.444	0.142	0.414	99	0.275	79.39
T0889TS258_2-D1.rsa	27.184	0.464	0.126	0.41	98	0.277	77.09
T0889TS077_1-D1.rsa	27.184	0.414	0.134	0.452	108	0.252	78.24
T0889TS407_3-D1.rsa	27.184	0.452	0.134	0.414	99	0.275	79.29
T0889TS345_1-D1.rsa	27.184	0.473	0.134	0.393	94	0.289	79.71
T0889TS382_1-D1.rsa	27.184	0.402	0.121	0.477	114	0.238	66.74
T0889TS357_5-D1.rsa	26.214	0.377	0	0.623	149	0.176	75.52
T0889TS464_3-D1.rsa	26.214	0.435	0.142	0.423	101	0.26	78.45
T0889TS359_2-D1.rsa	26.214	0.464	0.13	0.406	97	0.27	81.17
T0889TS405_4-D1.rsa	26.214	0.469	0.121	0.41	98	0.267	84.94
T0889TS183_5-D1.rsa	26.214	0.423	0.088	0.49	117	0.224	79.92
T0889TS275_2-D1.rsa	26.214	0.444	0.13	0.427	102	0.257	84.73
T0889TS077_2-D1.rsa	26.214	0.431	0.134	0.435	104	0.252	78.03
T0889TS119_1-D1.rsa	26.214	0.444	0.092	0.464	111	0.236	79.71
T0889TS236_5-D1.rsa	26.214	0.456	0.13	0.414	99	0.265	80.33
T0889TS405_5-D1.rsa	26.214	0.423	0.13	0.448	107	0.245	87.34
T0889TS357_3-D1.rsa	26.214	0.364	0.017	0.619	148	0.177	75.31

T0889TS005_3-D1.rsa	26.214	0.464	0.138	0.397	95	0.276	82.32
T0889TS183_3-D1.rsa	26.214	0.452	0.113	0.435	104	0.252	80.23
T0889TS467_3-D1.rsa	26.214	0.452	0.117	0.431	103	0.255	78.03
T0889TS407_5-D1.rsa	26.214	0.452	0.126	0.423	101	0.26	79.18
T0889TS382_5-D1.rsa	26.214	0.423	0.105	0.473	113	0.232	66.21
T0889TS183_4-D1.rsa	25.243	0.427	0.109	0.464	111	0.227	80.02
T0889TS258_4-D1.rsa	25.243	0.452	0.1	0.448	107	0.236	76.67
T0889TS479_4-D1.rsa	25.243	0.435	0.092	0.473	113	0.223	80.23
T0889TS077_3-D1.rsa	25.243	0.444	0.134	0.423	101	0.25	78.24
T0889TS479_5-D1.rsa	25.243	0.427	0.084	0.49	117	0.216	82.11
T0889TS452_3-D1.rsa	25.243	0.464	0.142	0.393	94	0.269	82.74
T0889TS313_3-D1.rsa	25.243	0.448	0.088	0.464	111	0.227	80.86
T0889TS005_4-D1.rsa	25.243	0.456	0.138	0.406	97	0.26	81.38
T0889TS313_2-D1.rsa	25.243	0.448	0.088	0.464	111	0.227	80.75
T0889TS467_1-D1.rsa	25.243	0.456	0.113	0.431	103	0.245	79.71
T0889TS287_4-D1.rsa	25.243	0.464	0.134	0.402	96	0.263	81.17
T0889TS236_3-D1.rsa	25.243	0.473	0.13	0.397	95	0.266	81.59
T0889TS382_3-D1.rsa	25.243	0.414	0.121	0.464	111	0.227	67.26
T0889TS180_3-D1.rsa	25.243	0.49	0.126	0.385	92	0.274	77.2
T0889TS180_2-D1.rsa	25.243	0.477	0.134	0.389	93	0.271	74.89
T0889TS357_4-D1.rsa	25.243	0.385	0	0.615	147	0.172	75.31
T0889TS028_1-D1.rsa	25.243	0.46	0.13	0.41	98	0.258	78.03
T0889TS407_1-D1.rsa	25.243	0.418	0.138	0.444	106	0.238	78.66
T0889TS220_1-D1.rsa	25.243	0.469	0.13	0.402	96	0.263	82.53
T0889TS251_1-D1.rsa	25.243	0.439	0.117	0.444	106	0.238	82.74
T0889TS220_5-D1.rsa	25.243	0.456	0.13	0.414	99	0.255	81.59
T0889TS382_4-D1.rsa	25.243	0.41	0.109	0.481	115	0.22	66.74
T0889TS220_2-D1.rsa	25.243	0.46	0.13	0.41	98	0.258	81.17
T0889TS220_4-D1.rsa	25.243	0.444	0.142	0.414	99	0.255	81.48
T0889TS455_2-D1.rsa	24.272	0.364	0.079	0.556	133	0.182	63.39
T0889TS250_4-D1.rsa	24.272	0.456	0.121	0.423	101	0.24	86.09
T0889TS258_5-D1.rsa	24.272	0.469	0.1	0.431	103	0.236	76.88
T0889TS183_1-D1.rsa	24.272	0.469	0.138	0.393	94	0.258	85.67
T0889TS349_1-D1.rsa	24.272	0.444	0.092	0.464	111	0.219	79.71
T0889TS479_1-D1.rsa	24.272	0.456	0.13	0.414	99	0.245	84.94
T0889TS313_1-D1.rsa	24.272	0.452	0.088	0.46	110	0.221	80.65
T0889TS407_2-D1.rsa	24.272	0.448	0.121	0.431	103	0.236	78.77
T0889TS287_3-D1.rsa	24.272	0.464	0.134	0.402	96	0.253	80.54
T0889TS016_1-D1.rsa	24.272	0.427	0.121	0.452	108	0.225	86.19
T0889TS077_4-D1.rsa	24.272	0.427	0.13	0.444	106	0.229	78.66
T0889TS345_2-D1.rsa	24.272	0.464	0.134	0.402	96	0.253	79.71
T0889TS287_1-D1.rsa	24.272	0.464	0.134	0.402	96	0.253	80.96
T0889TS405_3-D1.rsa	24.272	0.439	0.13	0.431	103	0.236	87.55
T0889TS251_5-D1.rsa	24.272	0.464	0.121	0.414	99	0.245	72.59
T0889TS220_3-D1.rsa	24.272	0.456	0.13	0.414	99	0.245	81.69
T0889TS357_2-D1.rsa	24.272	0.389	0	0.611	146	0.166	75.21
T0889TS345_5-D1.rsa	24.272	0.469	0.134	0.397	95	0.255	78.77

T0889TS005_2-D1.rsa	24.272	0.46	0.142	0.397	95	0.255	83.58
T0889TS026_3-D1.rsa	24.272	0.431	0.109	0.46	110	0.221	73.43
T0889TS180_4-D1.rsa	24.272	0.464	0.126	0.41	98	0.248	75.84
T0889TS444_2-D1.rsa	23.301	0.477	0.121	0.402	96	0.243	83.16
T0889TS479_3-D1.rsa	23.301	0.46	0.092	0.448	107	0.218	80.65
T0889TS250_1-D1.rsa	23.301	0.456	0.121	0.423	101	0.231	85.98
T0889TS452_5-D1.rsa	23.301	0.464	0.126	0.41	98	0.238	80.96
T0889TS421_4-D1.rsa	23.301	0.46	0.105	0.435	104	0.224	75.21
T0889TS421_3-D1.rsa	23.301	0.431	0.092	0.477	114	0.204	77.72
T0889TS005_1-D1.rsa	23.301	0.477	0.138	0.385	92	0.253	83.05
T0889TS258_3-D1.rsa	23.301	0.431	0.126	0.444	106	0.22	75.63
T0889TS250_2-D1.rsa	23.301	0.456	0.121	0.423	101	0.231	86.19
T0889TS467_2-D1.rsa	23.301	0.473	0.126	0.402	96	0.243	71.55
T0889TS425_2-D1.rsa	23.301	0.423	0.134	0.444	106	0.22	78.45
T0889TS236_2-D1.rsa	23.301	0.469	0.13	0.402	96	0.243	80.44
T0889TS287_5-D1.rsa	23.301	0.481	0.134	0.385	92	0.253	81.17
T0889TS421_2-D1.rsa	23.301	0.435	0.105	0.46	110	0.212	75.84
T0889TS236_1-D1.rsa	23.301	0.473	0.13	0.397	95	0.245	80.44
T0889TS236_4-D1.rsa	23.301	0.469	0.13	0.402	96	0.243	82.32
T0889TS407_4-D1.rsa	23.301	0.448	0.142	0.41	98	0.238	79.71
T0889TS444_4-D1.rsa	22.33	0.473	0.121	0.406	97	0.23	79.39
T0889TS444_3-D1.rsa	22.33	0.477	0.13	0.393	94	0.238	81.69
T0889TS444_5-D1.rsa	22.33	0.477	0.13	0.393	94	0.238	79.08
T0889TS250_3-D1.rsa	22.33	0.46	0.121	0.418	100	0.223	85.98
T0889TS345_4-D1.rsa	22.33	0.464	0.142	0.393	94	0.238	79.29
T0889TS287_2-D1.rsa	22.33	0.469	0.13	0.402	96	0.233	80.86
T0889TS250_5-D1.rsa	22.33	0.456	0.121	0.423	101	0.221	86.19
T0889TS026_5-D1.rsa	22.33	0.444	0.117	0.439	105	0.213	73.95
T0889TS345_3-D1.rsa	22.33	0.464	0.142	0.393	94	0.238	79.39
T0889TS005_5-D1.rsa	22.33	0.46	0.134	0.406	97	0.23	83.16
T0889TS467_5-D1.rsa	22.33	0.255	0.13	0.615	147	0.152	81.8
T0889TS251_4-D1.rsa	22.33	0.452	0.105	0.444	106	0.211	75.94
T0889TS455_1-D1.rsa	21.359	0.397	0.079	0.523	125	0.171	69.14
T0889TS421_5-D1.rsa	21.359	0.406	0.113	0.481	115	0.186	75.42
T0889TS467_4-D1.rsa	21.359	0.439	0.126	0.435	104	0.205	76.15
T0889TS258_1-D1.rsa	20.388	0.477	0.13	0.393	94	0.217	78.87
T0889TS444_1-D1.rsa	20.388	0.473	0.134	0.393	94	0.217	80.86
T0889TS180_1-D1.rsa	19.417	0.494	0.126	0.381	91	0.213	75.42
T0770TS184_2-D1.rsa	81.579	0.234	0.054	0.712	79	1.033	16.67
T0770TS381_2-D1.rsa	79.412	0.246	0	0.754	95	0.836	29.56
T0770TS041_2-D1.rsa	76.471	0.325	0	0.675	85	0.9	31.55
T0770TS420_4-D1.rsa	76	0.029	0	0.971	134	0.567	9.96
T0770TS414_2-D1.rsa	74.419	0.306	0	0.694	93	0.8	38.25
T0770TS237_4-D1.rsa	71.053	0.108	0.018	0.874	97	0.733	29.73
T0770TS228_2-D1.rsa	70.833	0	0.145	0.855	94	0.754	29.55
T0770TS335_2-D1.rsa	67.442	0.231	0.03	0.739	99	0.681	41.79
T0770TS499_4-D1.rsa	61.765	0.381	0	0.619	78	0.792	34.52

T0770TS156_1-D1.rsa	60.526	0.225	0.099	0.676	75	0.807	34.46
T0770TS436_1-D1.rsa	60.465	0.194	0	0.806	108	0.56	38.81
T0770TS117_1-D1.rsa	59.615	0.273	0.079	0.648	107	0.557	58.7
T0770TS454_1-D1.rsa	59.615	0.309	0.048	0.642	106	0.562	53.01
T0770TS479_5-D1.rsa	59.615	0.279	0.073	0.648	107	0.557	55.85
T0770TS345_3-D1.rsa	58.333	0	0.102	0.898	97	0.601	35.19
T0770TS133_2-D1.rsa	58.14	0.231	0.09	0.679	91	0.639	29.48
T0770TS268_1-D1.rsa	57.746	0.214	0.06	0.726	122	0.473	24.52
T0770TS448_5-D1.rsa	57.692	0.297	0.139	0.564	93	0.62	61.08
T0770TS448_2-D1.rsa	57.692	0.012	0.176	0.812	134	0.431	10.92
T0770TS381_3-D1.rsa	57.143	0.015	0.427	0.557	73	0.783	57.25
T0770TS237_1-D1.rsa	56.338	0.012	0.119	0.869	146	0.386	12.02
T0770TS277_1-D1.rsa	56.25	0	0.3	0.7	77	0.731	44.77
T0770TS349_3-D1.rsa	55.814	0.261	0.037	0.701	94	0.594	42.35
T0770TS171_1-D1.rsa	55.769	0.291	0.127	0.582	96	0.581	65.51
T0770TS228_4-D1.rsa	54.167	0	0.574	0.426	46	1.178	75.46
T0770TS184_5-D1.rsa	53.571	0	0.267	0.733	96	0.558	16.98
T0770TS216_1-D1.rsa	52.632	0.288	0.108	0.604	67	0.786	38.29
T0770TS277_2-D1.rsa	51.563	0.015	0.324	0.662	90	0.573	39.89
T0770TS237_2-D1.rsa	50.704	0	0.202	0.798	134	0.378	10.58
T0770TS349_4-D1.rsa	50	0	0.405	0.595	78	0.641	60.69
T0770TS420_5-D1.rsa	50	0	0.397	0.603	79	0.633	58.21
T0770TS038_2-D1.rsa	50	0.092	0.061	0.847	111	0.45	13.93
T0770TS160_5-D1.rsa	50	0	0.267	0.733	96	0.521	17.94
T0770TS228_1-D1.rsa	50	0	0.221	0.779	102	0.49	16.41
T0770TS216_3-D1.rsa	49.296	0.393	0.048	0.56	94	0.524	22.6
T0770TS436_2-D1.rsa	46.429	0	0.389	0.611	80	0.58	52.48
T0770TS145_1-D1.rsa	46.429	0	0.321	0.679	89	0.522	51.91
T0770TS277_3-D1.rsa	45.313	0.081	0	0.919	125	0.363	13.79
T0770TS133_5-D1.rsa	45.07	0.286	0	0.714	120	0.376	21.64
T0770TS133_4-D1.rsa	43.662	0.048	0.185	0.768	129	0.338	12.66
T0770TS011_5-D1.rsa	42.982	0.3	0.152	0.548	119	0.361	56.34
T0770TS452_1-D1.rsa	42.857	0	0.313	0.687	90	0.476	58.21
T0770TS345_2-D1.rsa	42.5	0	0.035	0.965	110	0.386	40.57
T0770TS133_1-D1.rsa	42.254	0.208	0.06	0.732	123	0.344	15.38
T0770TS268_3-D1.rsa	41.86	0.269	0.052	0.679	91	0.46	43.84
T0770TS251_3-D1.rsa	41.667	0.347	0.085	0.568	121	0.344	70.56
T0770TS346_1-D1.rsa	41.071	0.198	0	0.802	101	0.407	35.12
T0770TS410_2-D1.rsa	40.278	0.357	0.07	0.573	122	0.33	64.59
T0770TS263_1-D1.rsa	39.706	0.313	0.033	0.654	159	0.25	14.11
T0770TS479_2-D1.rsa	39.655	0.014	0.154	0.832	119	0.333	44.23
T0770TS210_1-D1.rsa	39.535	0.358	0.045	0.597	80	0.494	52.42
T0770TS268_5-D1.rsa	39.286	0	0.359	0.641	84	0.468	64.12
T0770TS499_1-D1.rsa	39.286	0	0.405	0.595	78	0.504	65.46
T0770TS381_1-D1.rsa	38.889	0.399	0.094	0.507	108	0.36	69.16
T0770TS050_1-D1.rsa	38.596	0.327	0.147	0.525	114	0.339	49.54
T0770TS410_5-D1.rsa	38.028	0.173	0	0.827	139	0.274	20.19

T0770TS300_1-D1.rsa	37.931	0	0.035	0.965	138	0.275	21.5
T0770TS011_3-D1.rsa	37.719	0.258	0.189	0.553	120	0.314	63.94
T0770TS251_2-D1.rsa	37.5	0.371	0.094	0.535	114	0.329	70.81
T0770TS268_2-D1.rsa	37.5	0.015	0.368	0.618	84	0.446	72.06
T0770TS228_5-D1.rsa	36.842	0.508	0.055	0.436	103	0.358	61.65
T0770TS300_5-D1.rsa	36.207	0.035	0.14	0.825	118	0.307	43.18
T0770TS171_5-D1.rsa	36.111	0.385	0.085	0.531	113	0.32	71.32
T0770TS008_5-D1.rsa	36	0.022	0.319	0.659	91	0.396	58.15
T0770TS160_1-D1.rsa	35.965	0.3	0.129	0.571	124	0.29	42.74
T0770TS038_5-D1.rsa	35.938	0	0.449	0.551	75	0.479	64.34
T0770TS452_5-D1.rsa	35.714	0	0.107	0.893	117	0.305	55.34
T0770TS479_4-D1.rsa	35.088	0.253	0.212	0.535	116	0.302	61.29
T0770TS300_4-D1.rsa	35.088	0.198	0.152	0.65	141	0.249	32.37
T0770TS184_1-D1.rsa	34.884	0.379	0.148	0.473	80	0.436	65.09
T0770TS216_2-D1.rsa	34.884	0.239	0.045	0.716	96	0.363	24.07
T0770TS145_4-D1.rsa	34.884	0.366	0.015	0.619	83	0.42	33.21
T0770TS268_4-D1.rsa	34.426	0.014	0.386	0.6	126	0.273	32.59
T0770TS349_1-D1.rsa	33.333	0.371	0.085	0.545	116	0.287	52.41
T0770TS420_1-D1.rsa	32.955	0.074	0.524	0.402	119	0.277	47.13
T0770TS038_1-D1.rsa	32.558	0.308	0.148	0.544	92	0.354	68.64
T0770TS335_5-D1.rsa	32	0.014	0.087	0.899	124	0.258	28.08
T0770TS335_1-D1.rsa	31.944	0.394	0.085	0.521	111	0.288	71.7
T0770TS492_3-D1.rsa	31.944	0.493	0.103	0.404	86	0.371	71.57
T0770TS454_3-D1.rsa	31.579	0.24	0.161	0.599	130	0.243	55.76
T0770TS160_3-D1.rsa	31.579	0.517	0.055	0.428	101	0.313	60.59
T0770TS300_2-D1.rsa	31.429	0.333	0.222	0.444	104	0.302	69.66
T0770TS038_3-D1.rsa	30.233	0.249	0.166	0.586	99	0.305	63.91
T0770TS420_3-D1.rsa	30.233	0.266	0.189	0.544	92	0.329	70.86
T0770TS133_3-D1.rsa	30.172	0.594	0	0.406	104	0.29	56.05
T0770TS117_5-D1.rsa	30.137	0.407	0.108	0.485	180	0.167	81.4
T0770TS263_4-D1.rsa	29.87	0.362	0.121	0.518	116	0.258	76.19
T0770TS171_2-D1.rsa	29.87	0.402	0.121	0.478	107	0.279	81.31
T0770TS479_1-D1.rsa	29.524	0.372	0.222	0.406	95	0.311	71.47
T0770TS454_2-D1.rsa	29.412	0.37	0.086	0.543	132	0.223	70
T0770TS410_1-D1.rsa	29.31	0.609	0	0.391	100	0.293	65.14
T0770TS206_1-D1.rsa	29.07	0.302	0.201	0.497	84	0.346	72.93
T0770TS381_4-D1.rsa	28.947	0.276	0.235	0.488	106	0.273	69.36
T0770TS117_3-D1.rsa	28.571	0.366	0.121	0.513	115	0.248	78.21
T0770TS160_2-D1.rsa	28.571	0.278	0.205	0.517	121	0.236	69.23
T0770TS345_4-D1.rsa	28.571	0.415	0.121	0.464	104	0.275	79.41
T0770TS499_5-D1.rsa	28.571	0.299	0.205	0.496	116	0.246	70.62
T0770TS448_3-D1.rsa	28.571	0.402	0.121	0.478	107	0.267	79.52
T0770TS041_5-D1.rsa	28	0.014	0.246	0.739	102	0.275	54.17
T0770TS038_4-D1.rsa	28	0.065	0.268	0.667	92	0.304	55.25
T0770TS212_1-D1.rsa	27.907	0.249	0.095	0.657	111	0.251	62.43
T0770TS479_3-D1.rsa	27.907	0.308	0.207	0.485	82	0.34	71.3
T0770TS008_4-D1.rsa	27.907	0.302	0.195	0.503	85	0.328	72.78

T0770TS436_5-D1.rsa	27.619	0.321	0.184	0.496	116	0.238	62.93
T0770TS454_4-D1.rsa	27.429	0.507	0	0.493	199	0.138	45.17
T0770TS251_4-D1.rsa	27	0.414	0.116	0.47	118	0.229	87.45
T0770TS263_3-D1.rsa	26.389	0.31	0.07	0.62	132	0.2	65.99
T0770TS263_2-D1.rsa	25.974	0.384	0.121	0.496	111	0.234	83.45
T0770TS277_5-D1.rsa	25.974	0.379	0.125	0.496	111	0.234	71.55
T0770TS414_3-D1.rsa	25.974	0.442	0.103	0.455	102	0.255	80.36
T0770TS251_5-D1.rsa	25.424	0	0.327	0.673	144	0.177	65.54
T0770TS454_5-D1.rsa	25.17	0.386	0.102	0.512	191	0.132	80.56
T0770TS263_5-D1.rsa	25	0.407	0.103	0.49	119	0.21	70.89
T0770TS499_3-D1.rsa	25	0.42	0.115	0.465	113	0.221	71.11
T0770TS448_4-D1.rsa	24.675	0.393	0.112	0.496	111	0.222	78.09
T0770TS414_1-D1.rsa	24.675	0.379	0.125	0.496	111	0.222	71.67
T0770TS452_4-D1.rsa	24	0	0.225	0.775	107	0.224	61.05
T0770TS216_4-D1.rsa	24	0.051	0.167	0.783	108	0.222	41.85
T0770TS436_3-D1.rsa	23.729	0.014	0.322	0.664	142	0.167	63.32
T0770TS171_4-D1.rsa	23.529	0.403	0.103	0.494	120	0.196	72.67
T0770TS041_1-D1.rsa	23.529	0.506	0.103	0.391	95	0.248	65.56
T0770TS436_4-D1.rsa	23.429	0.446	0.035	0.52	210	0.112	43.94
T0770TS414_5-D1.rsa	23.404	0.342	0.156	0.502	129	0.181	42.51
T0770TS011_4-D1.rsa	23.077	0.382	0.132	0.486	144	0.16	59.12
T0770TS410_4-D1.rsa	22.807	0.425	0.082	0.493	108	0.211	82.31
T0770TS448_1-D1.rsa	22.807	0.411	0.096	0.493	108	0.211	78.54
T0770TS452_3-D1.rsa	22.078	0.379	0.129	0.491	110	0.201	77.86
T0770TS349_5-D1.rsa	21.795	0.416	0.139	0.446	132	0.165	59.2
T0770TS279_1-D1.rsa	21	0.458	0.139	0.402	101	0.208	83.67
T0770TS008_3-D1.rsa	20.779	0.379	0.125	0.496	111	0.187	77.86
T0770TS492_2-D1.rsa	20.779	0.362	0.134	0.504	113	0.184	73.21
T0770TS345_1-D1.rsa	20.588	0.337	0.07	0.593	144	0.143	43.44
T0770TS277_4-D1.rsa	20.513	0.389	0.135	0.476	141	0.145	56.08
T0770TS160_4-D1.rsa	20.513	0.416	0.139	0.446	132	0.155	58.94
T0770TS414_4-D1.rsa	19.481	0.402	0.08	0.518	116	0.168	76.19
T0770TS381_5-D1.rsa	19.298	0.475	0.082	0.443	97	0.199	80.59
T0770TS335_3-D1.rsa	17.544	0.461	0.078	0.461	101	0.174	81.96
T0770TS492_4-D1.rsa	17.544	0.452	0.082	0.466	102	0.172	82.65
T0770TS349_2-D1.rsa	17.021	0.335	0.175	0.49	126	0.135	56.32
T0770TS117_2-D1.rsa	16.923	0.266	0.212	0.522	239	0.071	63.5
T0770TS156_4-D1.rsa	15.789	0.411	0.087	0.502	110	0.144	83.45
T0770TS492_5-D1.rsa	15.789	0.397	0.064	0.539	118	0.134	77.85
T0770TS499_2-D1.rsa	10.638	0.346	0.198	0.455	117	0.091	85.89
T0770TS251_1-D1.rsa	10.638	0.335	0.183	0.482	124	0.086	78.89
T0770TS420_2-D1.rsa	10.638	0.37	0.191	0.44	113	0.094	82.88
T0770TS008_2-D1.rsa	10.638	0.346	0.191	0.463	119	0.089	84.44
T0770TS008_1-D1.rsa	10.638	0.342	0.187	0.471	121	0.088	82.39
T0770TS011_1-D1.rsa	6.383	0.315	0.191	0.494	127	0.05	84.92
T0810TS301_1-D2.rsa	79.412	0.381	0	0.619	78	1.018	31.15
T0810TS216_5-D2.rsa	76.471	0.508	0.016	0.476	60	1.275	28.57

T0810TS118_5-D2.rsa	75	0	0.333	0.667	72	1.042	21.07
T0810TS184_5-D2.rsa	75	0.132	0.169	0.699	95	0.789	11.77
T0810TS340_3-D2.rsa	73.684	0.394	0	0.606	143	0.515	12.39
T0810TS326_1-D2.rsa	71.429	0.015	0.031	0.954	125	0.571	14.31
T0810TS381_1-D2.rsa	69.767	0.328	0	0.672	90	0.775	42.16
T0810TS197_4-D2.rsa	68.421	0.099	0.036	0.865	96	0.713	20.95
T0810TS358_5-D2.rsa	67.606	0.411	0	0.589	99	0.683	15.87
T0810TS482_1-D2.rsa	67.5	0	0.105	0.895	102	0.662	34.65
T0810TS041_3-D2.rsa	66.667	0	0.528	0.472	51	1.307	65.74
T0810TS204_1-D2.rsa	66.667	0	0.463	0.537	58	1.149	60.65
T0810TS160_1-D2.rsa	65.789	0.135	0	0.865	96	0.685	17.57
T0810TS442_4-D2.rsa	65.385	0.279	0.073	0.648	107	0.611	76.27
T0810TS276_4-D2.rsa	64.286	0	0.321	0.679	89	0.722	46.56
T0810TS210_4-D2.rsa	64.063	0	0.353	0.647	88	0.728	14.52
T0810TS483_1-D2.rsa	63.462	0.297	0.164	0.539	89	0.713	59.81
T0810TS301_5-D2.rsa	63.158	0.054	0	0.946	105	0.602	15.77
T0810TS349_3-D2.rsa	63.158	0.279	0.018	0.703	78	0.81	35.59
T0810TS144_3-D2.rsa	62.791	0.201	0.015	0.784	105	0.598	14.74
T0810TS206_1-D2.rsa	62.5	0.103	0	0.897	113	0.553	19.05
T0810TS425_4-D2.rsa	62.5	0	0.611	0.389	42	1.488	65.51
T0810TS290_4-D2.rsa	62.5	0.019	0.63	0.352	38	1.645	62.73
T0810TS171_4-D2.rsa	61.972	0.274	0.101	0.625	105	0.59	25.16
T0810TS362_4-D2.rsa	61.538	0.273	0.109	0.618	102	0.603	65.03
T0810TS263_1-D2.rsa	61.538	0.309	0.139	0.552	91	0.676	74.21
T0810TS008_1-D2.rsa	60.714	0	0.229	0.771	101	0.601	51.53
T0810TS454_3-D2.rsa	60.714	0	0.321	0.679	89	0.682	16.79
T0810TS216_2-D2.rsa	60.526	0.198	0	0.802	89	0.68	16.67
T0810TS277_4-D2.rsa	60.465	0.276	0	0.724	97	0.623	41.23
T0810TS364_1-D2.rsa	60.465	0.261	0	0.739	99	0.611	37.69
T0810TS445_2-D2.rsa	60	0	0.188	0.812	112	0.536	13.95
T0810TS448_4-D2.rsa	58.333	0	0.528	0.472	51	1.144	71.76
T0810TS445_3-D2.rsa	58.333	0	0.602	0.398	43	1.357	72.92
T0810TS410_4-D2.rsa	58.14	0.216	0.03	0.754	101	0.576	42.54
T0810TS064_1-D2.rsa	57.895	0.09	0.18	0.73	81	0.715	29.73
T0810TS310_4-D2.rsa	57.895	0.144	0.072	0.784	87	0.665	28.83
T0810TS454_4-D2.rsa	57.895	0.018	0.135	0.847	94	0.616	19.14
T0810TS116_4-D2.rsa	57.692	0.248	0.097	0.655	108	0.534	73.58
T0810TS235_3-D2.rsa	57.692	0.261	0.073	0.667	110	0.524	67.72
T0810TS276_1-D2.rsa	57.692	0.291	0.073	0.636	105	0.549	68.83
T0810TS276_3-D2.rsa	57.692	0.285	0.097	0.618	102	0.566	70.89
T0810TS144_1-D2.rsa	57.143	0	0.206	0.794	104	0.549	14.7
T0810TS310_1-D2.rsa	57.143	0	0.328	0.672	88	0.649	15.84
T0810TS064_2-D2.rsa	57.143	0.206	0.135	0.659	83	0.688	14.68
T0810TS362_5-D2.rsa	56.897	0.266	0	0.734	188	0.303	10.94
T0810TS204_3-D2.rsa	55.814	0.231	0.03	0.739	99	0.564	40.11
T0810TS133_1-D2.rsa	55.814	0.321	0.067	0.612	82	0.681	34.14
T0810TS006_1-D2.rsa	55.769	0.2	0.085	0.715	118	0.473	50.95

T0810TS056_1-D2.rsa	55.357	0.214	0.032	0.754	95	0.583	18.65
T0810TS169_1-D2.rsa	55.263	0.225	0.036	0.739	82	0.674	20.72
T0810TS492_3-D2.rsa	54.167	0	0.611	0.389	42	1.29	71.99
T0810TS410_1-D2.rsa	54.167	0	0.583	0.417	45	1.204	66.67
T0810TS296_4-D2.rsa	54.167	0.037	0.648	0.315	34	1.593	70.37
T0810TS448_2-D2.rsa	53.846	0.273	0.121	0.606	100	0.538	64.24
T0810TS345_2-D2.rsa	53.846	0.255	0.048	0.697	115	0.468	49.21
T0810TS044_1-D2.rsa	53.846	0.115	0.152	0.733	121	0.445	11.55
T0810TS117_1-D2.rsa	53.571	0	0.183	0.817	107	0.501	34.73
T0810TS338_5-D2.rsa	53.571	0.053	0.115	0.832	109	0.491	13.36
T0810TS228_1-D2.rsa	53.521	0.351	0.077	0.571	96	0.558	23.08
T0810TS064_4-D2.rsa	53.488	0.015	0.172	0.813	109	0.491	14.18
T0810TS171_5-D2.rsa	52.632	0.063	0	0.937	104	0.506	22.75
T0810TS197_3-D2.rsa	52.632	0.189	0	0.811	90	0.585	28.83
T0810TS499_4-D2.rsa	52.083	0	0.291	0.709	78	0.668	42.05
T0810TS228_5-D2.rsa	52	0.051	0.225	0.725	100	0.52	13.77
T0810TS483_5-D2.rsa	51.923	0	0.461	0.539	89	0.583	11.39
T0810TS338_3-D2.rsa	51.563	0.051	0.184	0.765	104	0.496	12.13
T0810TS479_4-D2.rsa	50	0.279	0.085	0.636	105	0.476	56.33
T0810TS326_2-D2.rsa	50	0	0.031	0.969	127	0.394	17.37
T0810TS008_5-D2.rsa	50	0.067	0	0.933	154	0.325	10.29
T0810TS277_2-D2.rsa	50	0.279	0.012	0.709	117	0.427	46.84
T0810TS445_1-D2.rsa	50	0.281	0.12	0.599	130	0.385	18.89
T0810TS038_3-D2.rsa	50	0.297	0.182	0.521	86	0.581	60.13
T0810TS162_5-D2.rsa	50	0.084	0	0.916	120	0.417	23.66
T0810TS425_3-D2.rsa	50	0.045	0.018	0.937	104	0.481	19.59
T0810TS034_1-D2.rsa	50	0	0.537	0.463	50	1	65.74
T0810TS263_2-D2.rsa	50	0.018	0.321	0.661	109	0.459	9.34
T0810TS210_1-D2.rsa	50	0.243	0.099	0.658	73	0.685	37.84
T0810TS296_1-D2.rsa	50	0.27	0.108	0.622	69	0.725	39.19
T0810TS410_2-D2.rsa	50	0.252	0.108	0.64	71	0.704	38.29
T0810TS044_5-D2.rsa	50	0.225	0.108	0.667	74	0.676	38.51
T0810TS210_2-D2.rsa	50	0.579	0	0.421	53	0.943	37.1
T0810TS117_4-D2.rsa	49.296	0.256	0.03	0.714	120	0.411	27.08
T0810TS279_1-D2.rsa	48.837	0.172	0.112	0.716	96	0.509	23.13
T0810TS436_5-D2.rsa	48.214	0.095	0.143	0.762	96	0.502	15.87
T0810TS263_3-D2.rsa	47.917	0.018	0.355	0.627	69	0.694	42.73
T0810TS296_5-D2.rsa	47.368	0.27	0.036	0.694	77	0.615	34.91
T0810TS237_2-D2.rsa	46.575	0.537	0	0.463	118	0.395	39.12
T0810TS442_1-D2.rsa	46.552	0	0.175	0.825	118	0.395	64.86
T0810TS145_4-D2.rsa	46.512	0.358	0.06	0.582	78	0.596	46.27
T0810TS410_3-D2.rsa	46.512	0.396	0.067	0.537	72	0.646	46.08
T0810TS132_2-D2.rsa	46.479	0.351	0	0.649	109	0.426	21.15
T0810TS479_5-D2.rsa	46.429	0	0.115	0.885	116	0.4	56.87
T0810TS360_2-D2.rsa	46.429	0	0.298	0.702	92	0.505	17.56
T0810TS338_2-D2.rsa	46.154	0.248	0.176	0.576	95	0.486	16.93
T0810TS044_4-D2.rsa	46.154	0.309	0.152	0.539	89	0.519	18.2

T0810TS171_2-D2.rsa	45.833	0.073	0.309	0.618	68	0.674	60.45
T0810TS276_2-D2.rsa	44.828	0.035	0.091	0.874	125	0.359	47.55
T0810TS118_2-D2.rsa	44.828	0.098	0.112	0.79	113	0.397	38.64
T0810TS038_5-D2.rsa	44.231	0.261	0.03	0.709	117	0.378	17.72
T0810TS044_2-D2.rsa	44.186	0.276	0.052	0.672	90	0.491	40.3
T0810TS403_5-D2.rsa	44	0	0.167	0.833	115	0.383	14.67
T0810TS064_3-D2.rsa	43.75	0	0.118	0.882	120	0.365	12.5
T0810TS049_5-D2.rsa	42.857	0	0.023	0.977	128	0.335	18.32
T0810TS277_5-D2.rsa	42.857	0	0.389	0.611	80	0.536	60.12
T0810TS483_4-D2.rsa	42.857	0	0.26	0.74	97	0.442	42.94
T0810TS073_2-D2.rsa	42.857	0	0.321	0.679	89	0.482	58.21
T0810TS216_3-D2.rsa	42.857	0	0.374	0.626	82	0.523	59.16
T0810TS038_1-D2.rsa	42.254	0.149	0.012	0.839	141	0.3	17.79
T0810TS434_1-D2.rsa	42.254	0.357	0.071	0.571	96	0.44	35.26
T0810TS145_1-D2.rsa	42.188	0.074	0.118	0.809	110	0.384	13.97
T0810TS301_3-D2.rsa	41.86	0.246	0	0.754	101	0.414	24.25
T0810TS442_2-D2.rsa	41.379	0	0.084	0.916	131	0.316	58.74
T0810TS345_4-D2.rsa	41.379	0	0.07	0.93	133	0.311	58.92
T0810TS347_4-D2.rsa	41.379	0.056	0.056	0.888	127	0.326	43.01
T0810TS403_3-D2.rsa	41.071	0.143	0.135	0.722	91	0.451	20.64
T0810TS333_2-D2.rsa	41.053	0.513	0.055	0.432	102	0.402	57.95
T0810TS452_4-D2.rsa	40.385	0.248	0.073	0.679	112	0.361	47.47
T0810TS228_3-D2.rsa	40	0.018	0.228	0.754	86	0.465	45.83
T0810TS162_4-D2.rsa	40	0	0.351	0.649	74	0.541	26.75
T0810TS362_3-D2.rsa	39.655	0	0.084	0.916	131	0.303	51.22
T0810TS345_5-D2.rsa	39.655	0	0.133	0.867	124	0.32	51.92
T0810TS097_2-D2.rsa	39.655	0.014	0.07	0.916	131	0.303	46.15
T0810TS403_2-D2.rsa	39.583	0.055	0.264	0.682	75	0.528	55.68
T0810TS347_5-D2.rsa	39.583	0.064	0.436	0.5	55	0.72	73.86
T0810TS439_4-D2.rsa	39.583	0.036	0.355	0.609	67	0.591	63.64
T0810TS310_2-D2.rsa	39.535	0.179	0.075	0.746	100	0.395	25.37
T0810TS169_2-D2.rsa	39.535	0.261	0.06	0.679	91	0.434	38.99
T0810TS282_5-D2.rsa	39.474	0.249	0.147	0.604	131	0.301	53.11
T0810TS368_3-D2.rsa	39.474	0.332	0.111	0.558	121	0.326	60.71
T0810TS204_2-D2.rsa	39.474	0.304	0.166	0.53	115	0.343	56.45
T0810TS132_3-D2.rsa	39.474	0.212	0.069	0.719	156	0.253	11.41
T0810TS436_4-D2.rsa	39.437	0	0.333	0.667	112	0.352	12.66
T0810TS169_3-D2.rsa	39.437	0.345	0.012	0.643	108	0.365	16.19
T0810TS038_2-D2.rsa	39.286	0	0.29	0.71	93	0.422	64.12
T0810TS347_1-D2.rsa	39.286	0.031	0.237	0.733	96	0.409	58.4
T0810TS282_4-D2.rsa	39.286	0	0.084	0.916	120	0.327	13.93
T0810TS145_5-D2.rsa	39.286	0	0.16	0.84	110	0.357	14.31
T0810TS067_5-D2.rsa	39.286	0.015	0.427	0.557	73	0.538	54.58
T0810TS251_1-D2.rsa	39.063	0.015	0.397	0.588	80	0.488	77.76
T0810TS184_1-D2.rsa	39.063	0	0.221	0.779	106	0.369	13.42
T0810TS425_2-D2.rsa	38.983	0.07	0.308	0.621	133	0.293	29.44
T0810TS346_1-D2.rsa	38.889	0.455	0.094	0.451	96	0.405	70.69

T0810TS118_4-D2.rsa	38.596	0.309	0.147	0.544	118	0.327	57.6
T0810TS162_2-D2.rsa	38.596	0.171	0.157	0.673	146	0.264	14.4
T0810TS403_1-D2.rsa	38.235	0.407	0.086	0.506	123	0.311	24
T0810TS117_5-D2.rsa	38.158	0.184	0.024	0.792	263	0.145	21.01
T0810TS420_3-D2.rsa	38.028	0.161	0	0.839	141	0.27	14.9
T0810TS326_5-D2.rsa	37.931	0	0.231	0.769	110	0.345	77.97
T0810TS155_4-D2.rsa	37.931	0	0.126	0.874	125	0.303	58.74
T0810TS042_4-D2.rsa	37.719	0.244	0.184	0.571	124	0.304	61.98
T0810TS049_4-D2.rsa	37.719	0.332	0.138	0.53	115	0.328	58.99
T0810TS067_3-D2.rsa	37.5	0.38	0.085	0.535	114	0.329	63.2
T0810TS358_4-D2.rsa	37.5	0.455	0.08	0.465	99	0.379	61.67
T0810TS358_3-D2.rsa	37.5	0.015	0.331	0.654	89	0.421	62.68
T0810TS282_3-D2.rsa	37.5	0.063	0.286	0.651	82	0.457	43.45
T0810TS169_4-D2.rsa	37.5	0	0.491	0.509	58	0.647	39.91
T0810TS483_2-D2.rsa	36.842	0.487	0.055	0.458	108	0.341	54.98
T0810TS228_2-D2.rsa	36.842	0.521	0.055	0.424	100	0.368	52.54
T0810TS184_3-D2.rsa	36.842	0.5	0.055	0.445	105	0.351	51.91
T0810TS296_3-D2.rsa	36.842	0.231	0.491	0.278	30	1.228	92.36
T0810TS340_2-D2.rsa	36.62	0.429	0.071	0.5	84	0.436	33.01
T0810TS097_1-D2.rsa	36.364	0.253	0.115	0.632	182	0.2	50.87
T0810TS023_1-D2.rsa	36.364	0.095	0.47	0.436	129	0.282	54.73
T0810TS157_2-D2.rsa	36.111	0.385	0.08	0.535	114	0.317	65.1
T0810TS076_5-D2.rsa	36.111	0.338	0.103	0.559	119	0.303	63.83
T0810TS349_5-D2.rsa	36	0.014	0.174	0.812	112	0.321	42.03
T0810TS349_2-D2.rsa	35.938	0.015	0.228	0.757	103	0.349	65.07
T0810TS322_2-D2.rsa	35.714	0.015	0.305	0.679	89	0.401	49.05
T0810TS296_2-D2.rsa	35.714	0.222	0	0.778	98	0.364	30.56
T0810TS445_5-D2.rsa	35.714	0.175	0.389	0.437	55	0.649	50.59
T0810TS439_3-D2.rsa	35.593	0.037	0.257	0.706	151	0.236	56.42
T0810TS301_2-D2.rsa	35.227	0.068	0.5	0.432	128	0.275	46.37
T0810TS445_4-D2.rsa	35.211	0.161	0	0.839	141	0.25	14.9
T0810TS145_3-D2.rsa	35	0	0.237	0.763	87	0.402	45.4
T0810TS076_4-D2.rsa	34.722	0.408	0.08	0.512	109	0.319	69.67
T0810TS338_4-D2.rsa	34.722	0.423	0.103	0.474	101	0.344	70.18
T0810TS368_2-D2.rsa	34.722	0.474	0.066	0.46	98	0.354	65.99
T0810TS349_4-D2.rsa	34.722	0.474	0.094	0.432	92	0.377	72.84
T0810TS277_3-D2.rsa	34.426	0.043	0.433	0.524	110	0.313	71.64
T0810TS008_3-D2.rsa	34.426	0.024	0.41	0.567	119	0.289	45.15
T0810TS144_5-D2.rsa	34.375	0.015	0.5	0.485	66	0.521	63.97
T0810TS436_3-D2.rsa	34.247	0.675	0	0.325	83	0.413	39.51
T0810TS340_1-D2.rsa	34.247	0.753	0	0.247	63	0.544	51.67
T0810TS345_1-D2.rsa	34.211	0.258	0.152	0.59	128	0.267	36.06
T0810TS210_3-D2.rsa	34.211	0.287	0.398	0.315	34	1.006	85.88
T0810TS041_2-D2.rsa	33.824	0.239	0.058	0.704	171	0.198	55
T0810TS171_1-D2.rsa	33.721	0.349	0.207	0.444	75	0.45	71.01
T0810TS073_1-D2.rsa	33.684	0.475	0.059	0.466	110	0.306	52.01
T0810TS216_1-D2.rsa	33.684	0.525	0.055	0.419	99	0.34	59.11

T0810TS263_4-D2.rsa	33.333	0.299	0.209	0.491	115	0.29	69.66
T0810TS452_2-D2.rsa	33.333	0.24	0.184	0.576	125	0.267	58.18
T0810TS290_3-D2.rsa	33.333	0.327	0.147	0.525	114	0.292	61.29
T0810TS116_5-D2.rsa	32.955	0.081	0.466	0.453	134	0.246	47.89
T0810TS011_1-D2.rsa	32.813	0	0.199	0.801	109	0.301	63.97
T0810TS237_4-D2.rsa	32.813	0	0.228	0.772	105	0.313	66.91
T0810TS145_2-D2.rsa	32.813	0.015	0.353	0.632	86	0.382	65.81
T0810TS492_5-D2.rsa	32.759	0.028	0.203	0.769	110	0.298	45.8
T0810TS391_3-D2.rsa	32.632	0.521	0.059	0.419	99	0.33	57.95
T0810TS041_4-D2.rsa	32.558	0.26	0.136	0.604	102	0.319	64.94
T0810TS080_5-D2.rsa	32.456	0.258	0.23	0.512	111	0.292	67.51
T0810TS097_3-D2.rsa	32.394	0.155	0	0.845	142	0.228	15.22
T0810TS228_4-D2.rsa	32.394	0.31	0.089	0.601	101	0.321	32.69
T0810TS197_1-D2.rsa	32.381	0.355	0.179	0.466	109	0.297	62.5
T0810TS349_1-D2.rsa	32.143	0	0.427	0.573	75	0.429	54.58
T0810TS368_1-D2.rsa	32	0.051	0.326	0.623	86	0.372	63.95
T0810TS076_2-D2.rsa	31.897	0.672	0	0.328	84	0.38	55.66
T0810TS360_1-D2.rsa	31.818	0.068	0.52	0.412	122	0.261	47.38
T0810TS153_3-D2.rsa	31.683	0.283	0.159	0.558	179	0.177	47.2
T0810TS403_4-D2.rsa	31.579	0.241	0.417	0.343	37	0.853	91.2
T0810TS439_1-D2.rsa	31.507	0.761	0	0.239	61	0.517	57.45
T0810TS011_3-D2.rsa	31.429	0.35	0.226	0.423	99	0.317	70.62
T0810TS439_2-D2.rsa	31.25	0.081	0.118	0.801	109	0.287	13.6
T0810TS197_5-D2.rsa	31.25	0.055	0.282	0.664	73	0.428	34.09
T0810TS155_1-D2.rsa	31.169	0.379	0.138	0.482	108	0.289	79.29
T0810TS153_4-D2.rsa	31.148	0.019	0.329	0.652	137	0.227	67.79
T0810TS300_5-D2.rsa	31.148	0.033	0.429	0.538	113	0.276	67.29
T0810TS310_3-D2.rsa	31.148	0.057	0.443	0.5	105	0.297	68.28
T0810TS310_5-D2.rsa	31.034	0.688	0	0.313	80	0.388	66.6
T0810TS391_4-D2.rsa	30.769	0.103	0.036	0.861	142	0.217	13.92
T0810TS391_2-D2.rsa	30.702	0.272	0.157	0.571	124	0.248	33.99
T0810TS116_1-D2.rsa	30.693	0.277	0.153	0.57	183	0.168	44.24
T0810TS063_2-D2.rsa	30.612	0.37	0.099	0.531	198	0.155	77.85
T0810TS251_4-D2.rsa	30.556	0.357	0.094	0.549	117	0.261	70.43
T0810TS251_2-D2.rsa	30.508	0.009	0.294	0.696	149	0.205	24.77
T0810TS006_2-D2.rsa	30.476	0.355	0.154	0.491	115	0.265	69.02
T0810TS080_4-D2.rsa	30.303	0.222	0.184	0.594	171	0.177	67.62
T0810TS041_1-D2.rsa	30.303	0.253	0.198	0.549	158	0.192	73.18
T0810TS358_1-D2.rsa	30.233	0.343	0.166	0.491	83	0.364	68.64
T0810TS067_1-D2.rsa	30	0	0.439	0.561	64	0.469	46.05
T0810TS251_3-D2.rsa	29.825	0.29	0.184	0.525	114	0.262	61.29
T0810TS436_2-D2.rsa	29.577	0.44	0.161	0.399	67	0.441	62.18
T0810TS436_1-D2.rsa	29.577	0.464	0.161	0.375	63	0.469	62.5
T0810TS368_4-D2.rsa	29.524	0.316	0.184	0.5	117	0.252	68.59
T0810TS452_5-D2.rsa	29.524	0.359	0.179	0.462	108	0.273	68.38
T0810TS391_1-D2.rsa	29.524	0.368	0.222	0.41	96	0.308	70.83
T0810TS360_3-D2.rsa	29.524	0.444	0.209	0.346	81	0.364	71.15

T0810TS157_1-D2.rsa	29.412	0.366	0.091	0.543	132	0.223	65.56
T0810TS263_5-D2.rsa	29.412	0.395	0.074	0.531	129	0.228	62.11
T0810TS041_5-D2.rsa	29.31	0	0.07	0.93	133	0.22	50.52
T0810TS448_5-D2.rsa	29.31	0.602	0	0.398	102	0.287	61.43
T0810TS492_4-D2.rsa	29.31	0.625	0	0.375	96	0.305	62.5
T0810TS420_5-D2.rsa	29.07	0.302	0.201	0.497	84	0.346	71.75
T0810TS276_5-D2.rsa	28.947	0.24	0.171	0.59	128	0.226	57.37
T0810TS067_4-D2.rsa	28.947	0.241	0.444	0.315	34	0.851	91.67
T0810TS063_4-D2.rsa	28.713	0.255	0.159	0.586	188	0.153	74.38
T0810TS483_3-D2.rsa	28.448	0.742	0	0.258	66	0.431	78.22
T0810TS133_3-D2.rsa	28.421	0.521	0.042	0.436	103	0.276	63.66
T0810TS044_3-D2.rsa	28.421	0.517	0.038	0.445	105	0.271	60.38
T0810TS326_3-D2.rsa	28.205	0.351	0.101	0.547	162	0.174	58.16
T0810TS277_1-D2.rsa	28	0.072	0.37	0.558	77	0.364	69.2
T0810TS425_1-D2.rsa	28	0.014	0.304	0.681	94	0.298	64.13
T0810TS118_1-D2.rsa	28	0.036	0.312	0.652	90	0.311	59.96
T0810TS210_5-D2.rsa	28	0.058	0.333	0.609	84	0.333	66.67
T0810TS492_1-D2.rsa	28	0.014	0.275	0.71	98	0.286	43.66
T0810TS132_1-D2.rsa	28	0.138	0.014	0.848	117	0.239	13.95
T0810TS414_5-D2.rsa	27.941	0.428	0.107	0.465	113	0.247	70.78
T0810TS290_1-D2.rsa	27.907	0.296	0.178	0.527	89	0.314	69.23
T0810TS326_4-D2.rsa	27.869	0.019	0.39	0.59	124	0.225	68.28
T0810TS268_5-D2.rsa	27.869	0	0.381	0.619	130	0.214	51.49
T0810TS452_1-D2.rsa	27.619	0.342	0.197	0.462	108	0.256	70.51
T0810TS160_2-D2.rsa	27.619	0.393	0.201	0.406	95	0.291	71.9
T0810TS097_4-D2.rsa	27.619	0.338	0.218	0.444	104	0.266	69.77
T0810TS391_5-D2.rsa	27.619	0.385	0.209	0.406	95	0.291	72.01
T0810TS184_4-D2.rsa	27.619	0.38	0.239	0.38	89	0.31	74.79
T0810TS290_5-D2.rsa	27.619	0.423	0.214	0.363	85	0.325	71.47
T0810TS050_1-D2.rsa	27.5	0	0.158	0.842	96	0.286	26.97
T0810TS160_3-D2.rsa	27.368	0.521	0.051	0.428	101	0.271	62.29
T0810TS076_3-D2.rsa	27.273	0.247	0.194	0.559	161	0.169	67.19
T0810TS006_4-D2.rsa	27.273	0.247	0.181	0.573	165	0.165	68.23
T0810TS362_2-D2.rsa	27	0.422	0.124	0.454	114	0.237	89.34
T0810TS212_1-D2.rsa	27	0.466	0.116	0.418	105	0.257	83.96
T0810TS300_4-D2.rsa	26.857	0.483	0.054	0.463	187	0.144	40.28
T0810TS133_4-D2.rsa	26.744	0.284	0.154	0.562	95	0.282	69.97
T0810TS144_4-D2.rsa	26.667	0.346	0.205	0.449	105	0.254	72.01
T0810TS049_1-D2.rsa	26.471	0.403	0.086	0.51	124	0.213	71.89
T0810TS335_4-D2.rsa	26.471	0.403	0.099	0.498	121	0.219	67.22
T0810TS417_1-D2.rsa	26.471	0.412	0.103	0.486	118	0.224	57.22
T0810TS300_3-D2.rsa	26.263	0.247	0.198	0.556	160	0.164	69.27
T0810TS420_1-D2.rsa	26.23	0.029	0.443	0.529	111	0.236	64.43
T0810TS268_2-D2.rsa	26.23	0.024	0.438	0.538	113	0.232	68.53
T0810TS153_1-D2.rsa	26.027	0.412	0.092	0.496	184	0.141	71.87
T0810TS160_4-D2.rsa	26	0.434	0.139	0.426	107	0.243	88.55
T0810TS268_3-D2.rsa	26	0.458	0.135	0.406	102	0.255	81.67

T0810TS290_2-D2.rsa	25.974	0.388	0.103	0.509	114	0.228	80
T0810TS042_3-D2.rsa	25.974	0.411	0.116	0.473	106	0.245	81.79
T0810TS357_1-D2.rsa	25.974	0.42	0.121	0.46	103	0.252	79.88
T0810TS282_1-D2.rsa	25.862	0.668	0	0.332	85	0.304	62.7
T0810TS438_1-D2.rsa	25.714	0.376	0.128	0.496	116	0.222	68.27
T0810TS049_2-D2.rsa	25.641	0.432	0.135	0.432	128	0.2	54.34
T0810TS360_5-D2.rsa	25.581	0.32	0.195	0.485	82	0.312	72.48
T0810TS420_2-D2.rsa	25.424	0.042	0.285	0.673	144	0.177	34.11
T0810TS454_5-D2.rsa	25.352	0.452	0.06	0.488	82	0.309	38.94
T0810TS006_5-D2.rsa	25.253	0.201	0.17	0.628	181	0.14	64.5
T0810TS235_1-D2.rsa	25.253	0.229	0.219	0.552	159	0.159	69.27
T0810TS235_2-D2.rsa	25	0.37	0.078	0.551	134	0.187	69.44
T0810TS448_1-D2.rsa	25	0.412	0.103	0.486	118	0.212	69.44
T0810TS335_1-D2.rsa	25	0.362	0.075	0.563	120	0.208	54.57
T0810TS300_1-D2.rsa	24.59	0.043	0.362	0.595	125	0.197	60.32
T0810TS157_4-D2.rsa	24.359	0.348	0.091	0.561	166	0.147	55.99
T0810TS268_4-D2.rsa	24	0.414	0.068	0.518	130	0.185	38.84
T0810TS492_2-D2.rsa	24	0.036	0.362	0.601	83	0.289	63.41
T0810TS338_1-D2.rsa	24	0.101	0.377	0.522	72	0.333	67.57
T0810TS097_5-D2.rsa	24	0.036	0.283	0.681	94	0.255	36.96
T0810TS235_4-D2.rsa	23.529	0.35	0.086	0.564	137	0.172	67.89
T0810TS442_3-D2.rsa	23.429	0.53	0.02	0.45	182	0.129	50.12
T0810TS042_1-D2.rsa	23.077	0.405	0.071	0.524	155	0.149	58.85
T0810TS080_1-D2.rsa	23	0.375	0.104	0.522	131	0.176	86.06
T0810TS160_5-D2.rsa	22.857	0.475	0.047	0.478	193	0.118	40.9
T0810TS381_4-D2.rsa	22.857	0.321	0.158	0.521	122	0.187	64.21
T0810TS155_5-D2.rsa	22.807	0.416	0.1	0.484	106	0.215	78.77
T0810TS042_2-D2.rsa	22.807	0.443	0.082	0.475	104	0.219	80.71
T0810TS157_5-D2.rsa	22.222	0.243	0.229	0.528	152	0.146	70.23
T0810TS347_3-D2.rsa	22.105	0.547	0.055	0.398	94	0.235	57.31
T0810TS479_3-D2.rsa	22.078	0.357	0.071	0.571	128	0.172	72.74
T0810TS333_3-D2.rsa	21.795	0.422	0.108	0.47	139	0.157	53.82
T0810TS301_4-D2.rsa	21.429	0.23	0.016	0.754	95	0.226	41.67
T0810TS479_1-D2.rsa	21.311	0.029	0.319	0.652	137	0.156	66.79
T0810TS026_1-D2.rsa	21.311	0.038	0.4	0.562	118	0.181	69.03
T0810TS063_5-D2.rsa	21.053	0.434	0.024	0.542	247	0.085	57.24
T0810TS333_1-D2.rsa	21.053	0.393	0.096	0.511	112	0.188	76.26
T0810TS360_4-D2.rsa	21.053	0.47	0.078	0.452	99	0.213	80.82
T0810TS499_3-D2.rsa	20.779	0.406	0.134	0.46	103	0.202	79.64
T0810TS237_1-D2.rsa	20.779	0.371	0.138	0.491	110	0.189	79.05
T0810TS381_5-D2.rsa	20.779	0.362	0.138	0.5	112	0.186	77.38
T0810TS235_5-D2.rsa	20.571	0.49	0.017	0.493	199	0.103	41.09
T0810TS153_5-D2.rsa	20.202	0.264	0.226	0.51	147	0.137	69.7
T0810TS080_2-D2.rsa	19.767	0.403	0.12	0.477	135	0.146	73.94
T0810TS116_2-D2.rsa	19.737	0.259	0.093	0.648	215	0.092	44.88
T0810TS011_2-D2.rsa	19.481	0.411	0.121	0.469	105	0.186	80.59
T0810TS499_5-D2.rsa	19.298	0.461	0.078	0.461	101	0.191	80.94

T0810TS362_1-D2.rsa	19.231	0.372	0.122	0.507	150	0.128	59.72
T0810TS155_3-D2.rsa	19.192	0.163	0.083	0.753	217	0.088	9.29
T0810TS157_3-D2.rsa	18.605	0.392	0.074	0.534	151	0.123	80.39
T0810TS439_5-D2.rsa	18.605	0.392	0.12	0.488	138	0.135	82.69
T0810TS499_2-D2.rsa	18.605	0.42	0.134	0.445	126	0.148	73.23
T0810TS117_3-D2.rsa	18.182	0.371	0.147	0.482	108	0.168	77.86
T0810TS042_5-D2.rsa	17.544	0.361	0.096	0.543	119	0.147	74.31
T0810TS049_3-D2.rsa	17.544	0.443	0.091	0.466	102	0.172	81.05
T0810TS011_4-D2.rsa	17.544	0.461	0.1	0.438	96	0.183	73.97
T0810TS197_2-D2.rsa	17.544	0.466	0.087	0.447	98	0.179	81.85
T0810TS080_3-D2.rsa	17.105	0.331	0.163	0.506	168	0.102	68.98
T0810TS118_3-D2.rsa	16.279	0.456	0.124	0.42	119	0.137	78.53
T0810TS322_1-D2.rsa	16	0.051	0.254	0.696	96	0.167	60.15
T0810TS184_2-D2.rsa	16	0.036	0.312	0.652	90	0.178	40.94
T0810TS063_3-D2.rsa	15.789	0.31	0.12	0.569	189	0.084	99.1
T0810TS011_5-D2.rsa	15.789	0.457	0.073	0.47	103	0.153	81.62
T0810TS381_2-D2.rsa	15.789	0.406	0.091	0.502	110	0.144	73.06
T0810TS345_3-D2.rsa	15.789	0.452	0.087	0.461	101	0.156	82.99
T0810TS063_1-D2.rsa	14.474	0.352	0.123	0.524	174	0.083	99.1
T0810TS425_5-D2.rsa	14.035	0.452	0.1	0.447	98	0.143	79
T0810TS368_5-D2.rsa	14.035	0.447	0.087	0.466	102	0.138	78.08
T0810TS479_2-D2.rsa	12.766	0.346	0.198	0.455	117	0.109	84.83
T0810TS420_4-D2.rsa	12.766	0.366	0.198	0.436	112	0.114	81.71
T0810TS442_5-D2.rsa	12.766	0.335	0.183	0.482	124	0.103	82.39
T0810TS132_4-D2.rsa	12.281	0.457	0.068	0.475	104	0.118	80.94
T0810TS006_3-D2.rsa	10.638	0.28	0.163	0.556	143	0.074	76.75
T0810TS008_2-D2.rsa	10.638	0.346	0.183	0.471	121	0.088	81.81
T0810TS414_2-D2.rsa	10.638	0.342	0.183	0.475	122	0.087	82.69
T0810TS335_3-D2.rsa	8.511	0.346	0.183	0.471	121	0.07	85.41
T0810TS322_3-D2.rsa	8.511	0.354	0.183	0.463	119	0.072	85.21
T0786TS228_1-D1.rsa	82.353	0.317	0	0.683	86	0.958	27.18
T0786TS492_2-D1.rsa	82.143	0.076	0	0.924	121	0.679	18.89
T0786TS145_1-D1.rsa	76.471	0.321	0	0.679	165	0.463	9.11
T0786TS277_3-D1.rsa	75	0.224	0.036	0.739	122	0.615	17.72
T0786TS492_1-D1.rsa	70.588	0.27	0	0.73	92	0.767	32.54
T0786TS193_2-D1.rsa	69.767	0.224	0.045	0.731	98	0.712	38.62
T0786TS268_1-D1.rsa	69.767	0.03	0	0.97	130	0.537	17.16
T0786TS184_3-D1.rsa	69.014	0.363	0	0.637	107	0.645	15.06
T0786TS156_5-D1.rsa	68.75	0.096	0.147	0.757	103	0.667	40.07
T0786TS216_5-D1.rsa	66.667	0	0.5	0.5	54	1.235	65.74
T0786TS479_1-D1.rsa	65.116	0.201	0	0.799	107	0.609	37.31
T0786TS414_2-D1.rsa	65.116	0.216	0.03	0.754	101	0.645	43.1
T0786TS335_2-D1.rsa	64.286	0.015	0.038	0.947	124	0.518	16.6
T0786TS300_2-D1.rsa	64.286	0.015	0.076	0.908	119	0.54	14.7
T0786TS008_2-D1.rsa	60.714	0.015	0.344	0.641	84	0.723	56.68
T0786TS210_4-D1.rsa	60	0.043	0.203	0.754	104	0.577	13.59
T0786TS216_2-D1.rsa	60	0.065	0.188	0.746	103	0.583	13.41

T0786TS171_5-D1.rsa	59.155	0.244	0.083	0.673	113	0.523	25.8
T0786TS133_5-D1.rsa	58.904	0.439	0	0.561	143	0.412	10.29
T0786TS160_1-D1.rsa	58.14	0.179	0	0.821	110	0.529	34.52
T0786TS300_1-D1.rsa	57.692	0.285	0.097	0.618	102	0.566	52.53
T0786TS454_4-D1.rsa	56.338	0.304	0.048	0.649	109	0.517	21.47
T0786TS414_3-D1.rsa	53.846	0.012	0.103	0.885	146	0.369	10.92
T0786TS410_3-D1.rsa	53.488	0.321	0.03	0.649	87	0.615	43.1
T0786TS349_2-D1.rsa	51.923	0.285	0.073	0.642	106	0.49	56.8
T0786TS011_2-D1.rsa	50.704	0.31	0.012	0.679	114	0.445	20.99
T0786TS011_1-D1.rsa	50	0.042	0.028	0.93	133	0.376	12.06
T0786TS038_3-D1.rsa	50	0	0.405	0.595	78	0.641	58.4
T0786TS184_2-D1.rsa	50	0.255	0.055	0.691	114	0.439	12.5
T0786TS171_3-D1.rsa	48.077	0.267	0.097	0.636	105	0.458	42.25
T0786TS228_2-D1.rsa	46.552	0.063	0	0.937	134	0.347	12.94
T0786TS454_1-D1.rsa	46.512	0.313	0.09	0.597	80	0.581	44.03
T0786TS008_1-D1.rsa	46.429	0	0.29	0.71	93	0.499	58.4
T0786TS011_5-D1.rsa	46.429	0	0.275	0.725	95	0.489	54.2
T0786TS410_4-D1.rsa	44.231	0.248	0.073	0.679	112	0.395	48.26
T0786TS420_1-D1.rsa	44.231	0.315	0.097	0.588	97	0.456	54.11
T0786TS133_3-D1.rsa	44	0	0.13	0.87	120	0.367	42.03
T0786TS237_4-D1.rsa	43.103	0	0.07	0.93	133	0.324	59.09
T0786TS050_1-D1.rsa	42.857	0	0.336	0.664	87	0.493	58.78
T0786TS184_1-D1.rsa	41.86	0.351	0	0.649	87	0.481	46.83
T0786TS349_5-D1.rsa	41.379	0	0.098	0.902	129	0.321	55.94
T0786TS210_3-D1.rsa	40	0.483	0.055	0.462	109	0.367	57.1
T0786TS038_1-D1.rsa	40	0.018	0.254	0.728	83	0.482	45.61
T0786TS414_1-D1.rsa	39.655	0	0.119	0.881	126	0.315	55.77
T0786TS073_1-D1.rsa	39.535	0.246	0	0.754	101	0.391	28.17
T0786TS216_1-D1.rsa	39.535	0.361	0.13	0.509	86	0.46	48.96
T0786TS277_1-D1.rsa	39.437	0.292	0.089	0.619	104	0.379	40.87
T0786TS228_5-D1.rsa	39.286	0.19	0.032	0.778	98	0.401	48.21
T0786TS479_2-D1.rsa	37.931	0	0.231	0.769	110	0.345	79.02
T0786TS237_5-D1.rsa	37.719	0.263	0.161	0.576	125	0.302	65.09
T0786TS216_3-D1.rsa	37.662	0.393	0.036	0.571	128	0.294	15.24
T0786TS436_5-D1.rsa	37.5	0.211	0.009	0.779	166	0.226	51.9
T0786TS349_4-D1.rsa	37.143	0.592	0.03	0.379	153	0.243	33.11
T0786TS145_3-D1.rsa	36.842	0.244	0.184	0.571	124	0.297	62.21
T0786TS499_1-D1.rsa	36.364	0.064	0.456	0.48	142	0.256	35.39
T0786TS335_4-D1.rsa	36.207	0	0.217	0.783	112	0.323	77.1
T0786TS008_4-D1.rsa	36.207	0.582	0	0.418	107	0.338	55.18
T0786TS263_4-D1.rsa	35.789	0.538	0.059	0.403	95	0.377	63.14
T0786TS237_2-D1.rsa	35.227	0.071	0.476	0.453	134	0.263	46.28
T0786TS268_3-D1.rsa	35.088	0.361	0.073	0.566	124	0.283	58.56
T0786TS410_5-D1.rsa	35.088	0.374	0.073	0.553	121	0.29	57.53
T0786TS335_1-D1.rsa	34.722	0.385	0.075	0.54	115	0.302	65.1
T0786TS251_2-D1.rsa	34.483	0	0.231	0.769	110	0.313	78.67
T0786TS210_5-D1.rsa	34.483	0	0.147	0.853	122	0.283	58.92

T0786TS345_1-D1.rsa	34.211	0.267	0.175	0.558	121	0.283	56.22
T0786TS492_4-D1.rsa	33.333	0.258	0.103	0.638	136	0.245	62.94
T0786TS300_3-D1.rsa	33.333	0.418	0.094	0.488	104	0.321	73.48
T0786TS452_1-D1.rsa	32.955	0.057	0.497	0.446	132	0.25	48.31
T0786TS335_5-D1.rsa	32.955	0.078	0.426	0.497	147	0.224	36.99
T0786TS452_3-D1.rsa	32.787	0	0.443	0.557	117	0.28	59.83
T0786TS228_4-D1.rsa	32.759	0.574	0	0.426	109	0.301	54.88
T0786TS156_4-D1.rsa	32.759	0.578	0	0.422	108	0.303	52.44
T0786TS184_5-D1.rsa	32.632	0.53	0.064	0.407	96	0.34	54.13
T0786TS184_4-D1.rsa	32.558	0.373	0	0.627	84	0.388	40.48
T0786TS011_4-D1.rsa	32.394	0	0.065	0.935	157	0.206	10.9
T0786TS436_3-D1.rsa	32.323	0.233	0.021	0.747	215	0.15	38.11
T0786TS073_2-D1.rsa	31.579	0.551	0.042	0.407	96	0.329	58.79
T0786TS210_1-D1.rsa	31.507	0.745	0	0.255	65	0.485	59.31
T0786TS117_4-D1.rsa	31.429	0.295	0.214	0.491	115	0.273	69.12
T0786TS345_5-D1.rsa	31.148	0.014	0.395	0.59	124	0.251	72.76
T0786TS263_3-D1.rsa	31.148	0.038	0.452	0.51	107	0.291	73.51
T0786TS436_2-D1.rsa	31.148	0.038	0.438	0.524	110	0.283	66.92
T0786TS345_4-D1.rsa	30.882	0.407	0	0.593	144	0.214	26.11
T0786TS345_3-D1.rsa	30.702	0.258	0.226	0.516	112	0.274	66.94
T0786TS263_2-D1.rsa	30.702	0.3	0.171	0.53	115	0.267	60.14
T0786TS277_2-D1.rsa	30.476	0.385	0.218	0.397	93	0.328	70.73
T0786TS041_4-D1.rsa	30.233	0.308	0.201	0.491	83	0.364	71.89
T0786TS038_5-D1.rsa	30.172	0.594	0	0.406	104	0.29	62.5
T0786TS499_4-D1.rsa	29.825	0.272	0.221	0.507	110	0.271	69.93
T0786TS041_2-D1.rsa	29.703	0.29	0.146	0.564	181	0.164	72.9
T0786TS160_4-D1.rsa	29.703	0.265	0.156	0.579	186	0.16	75.7
T0786TS479_3-D1.rsa	29.508	0.033	0.443	0.524	110	0.268	74.25
T0786TS479_4-D1.rsa	29.412	0.313	0.086	0.601	146	0.201	50.78
T0786TS133_4-D1.rsa	29.167	0.39	0.085	0.526	112	0.26	65.23
T0786TS279_1-D1.rsa	29.167	0.46	0.094	0.446	95	0.307	69.8
T0786TS171_1-D1.rsa	29.07	0.349	0.189	0.462	78	0.373	74.56
T0786TS346_1-D1.rsa	28.571	0.333	0.205	0.462	108	0.265	70.51
T0786TS263_1-D1.rsa	28.571	0.372	0.205	0.423	99	0.289	71.8
T0786TS038_4-D1.rsa	28.571	0.385	0.239	0.376	88	0.325	74.47
T0786TS436_4-D1.rsa	28.283	0.184	0.125	0.691	199	0.142	60.16
T0786TS454_2-D1.rsa	28.169	0.524	0.071	0.405	68	0.414	60.42
T0786TS499_3-D1.rsa	28	0.47	0.12	0.41	103	0.272	86.85
T0786TS410_2-D1.rsa	28	0.065	0.29	0.645	89	0.315	55.8
T0786TS381_1-D1.rsa	27.941	0.362	0.107	0.531	129	0.217	68.11
T0786TS300_5-D1.rsa	27.941	0.387	0.082	0.531	129	0.217	62.89
T0786TS479_5-D1.rsa	27.941	0.403	0.095	0.502	122	0.229	66.56
T0786TS216_4-D1.rsa	27.907	0.337	0.16	0.503	85	0.328	69.97
T0786TS448_3-D1.rsa	27.869	0.024	0.433	0.543	114	0.244	63.18
T0786TS452_2-D1.rsa	27.869	0.024	0.395	0.581	122	0.228	63.56
T0786TS160_3-D1.rsa	27.397	0.418	0.108	0.474	176	0.156	84.47
T0786TS145_2-D1.rsa	27.273	0.24	0.194	0.566	163	0.167	66.49

T0786TS420_5-D1.rsa	27.273	0.393	0.103	0.504	113	0.241	79.64
T0786TS349_1-D1.rsa	27.119	0	0.318	0.682	146	0.186	64.37
T0786TS499_5-D1.rsa	26.471	0.337	0.099	0.564	137	0.193	68.78
T0786TS448_2-D1.rsa	26.471	0.35	0.107	0.543	132	0.201	65
T0786TS492_5-D1.rsa	26.471	0.379	0.099	0.523	127	0.208	67.11
T0786TS268_4-D1.rsa	26.471	0.428	0.107	0.465	113	0.234	66.67
T0786TS349_3-D1.rsa	26.23	0.019	0.362	0.619	130	0.202	65.17
T0786TS212_1-D1.rsa	26.23	0.071	0.448	0.481	101	0.26	68.03
T0786TS268_2-D1.rsa	25.974	0.42	0.121	0.46	103	0.252	82.38
T0786TS041_3-D1.rsa	25.85	0.397	0.11	0.493	184	0.14	84.38
T0786TS345_2-D1.rsa	25.714	0.338	0.167	0.496	116	0.222	64
T0786TS237_3-D1.rsa	25	0.346	0.078	0.576	140	0.179	67.56
T0786TS251_4-D1.rsa	25	0.387	0.095	0.519	126	0.198	67.33
T0786TS237_1-D1.rsa	25	0.366	0.099	0.535	130	0.192	67
T0786TS414_5-D1.rsa	25	0.403	0.103	0.494	120	0.208	67
T0786TS335_3-D1.rsa	24.675	0.384	0.112	0.504	113	0.218	79.64
T0786TS448_4-D1.rsa	24.359	0.368	0.111	0.52	154	0.158	56.6
T0786TS041_5-D1.rsa	24.359	0.385	0.115	0.5	148	0.165	53.65
T0786TS381_4-D1.rsa	24.359	0.382	0.145	0.473	140	0.174	58.94
T0786TS050_2-D1.rsa	24	0.014	0.406	0.58	80	0.3	75.91
T0786TS492_3-D1.rsa	23.529	0.317	0.107	0.576	140	0.168	68.11
T0786TS381_2-D1.rsa	23.529	0.412	0.107	0.481	117	0.201	71.56
T0786TS452_4-D1.rsa	23.529	0.379	0.091	0.531	129	0.182	65.44
T0786TS117_3-D1.rsa	23.529	0.412	0.107	0.481	117	0.201	71.22
T0786TS414_4-D1.rsa	23.077	0.385	0.122	0.493	146	0.158	57.73
T0786TS452_5-D1.rsa	23	0.414	0.12	0.466	117	0.197	85.96
T0786TS381_5-D1.rsa	23	0.43	0.124	0.446	112	0.205	89.84
T0786TS448_5-D1.rsa	22.857	0.572	0.022	0.406	164	0.139	43.01
T0786TS420_4-D1.rsa	22.078	0.379	0.134	0.487	109	0.203	76.91
T0786TS420_3-D1.rsa	22.059	0.399	0.078	0.523	127	0.174	68.11
T0786TS381_3-D1.rsa	21.795	0.361	0.128	0.51	151	0.144	56.6
T0786TS277_4-D1.rsa	21.795	0.402	0.118	0.48	142	0.153	57.9
T0786TS160_5-D1.rsa	21.233	0.391	0.108	0.501	186	0.114	58.65
T0786TS263_5-D1.rsa	20.588	0.403	0.082	0.514	125	0.165	64.78
T0786TS300_4-D1.rsa	20	0.051	0.167	0.783	108	0.185	58.15
T0786TS410_1-D1.rsa	19.298	0.425	0.082	0.493	108	0.179	81.51
T0786TS251_3-D1.rsa	18.605	0.332	0.11	0.558	158	0.118	71.03
T0786TS448_1-D1.rsa	18.605	0.42	0.12	0.459	130	0.143	80.65
T0786TS251_1-D1.rsa	17.544	0.411	0.05	0.539	118	0.149	80.14
T0786TS145_4-D1.rsa	16.279	0.385	0.127	0.488	138	0.118	81.71
T0786TS117_5-D1.rsa	15.789	0.474	0.015	0.511	233	0.068	63.65
T0786TS206_1-D1.rsa	15.789	0.337	0.123	0.539	179	0.088	98.95
T0786TS117_1-D1.rsa	15.789	0.434	0.082	0.484	106	0.149	81.96
T0786TS133_1-D1.rsa	15.789	0.457	0.091	0.452	99	0.159	83.68
T0786TS133_2-D1.rsa	15.789	0.447	0.082	0.47	103	0.153	79.68
T0786TS145_5-D1.rsa	15.254	0	0.159	0.841	180	0.085	59.58

CV1 Dataset	CV2 Dataset	CV3 Dataset
T0819-D1	T0827-D1	T0871-D1
T0835-D1	T0796-D1	T0760-D1
T0803-D1	T0849-D1	T0766-D1
T0845-D2	T0770-D1	T0860-D1
T0881-D1	T0772-D1	T0762-D1
T0852-D1	T0795-D1	T0821-D1
T0877-D1	T0794-D1	T0867-D1
T0826-D2	T0817-D2	T0764-D1
T0811-D1	T0879-D1	T0823-D1
T0838-D1	T0782-D1	T0848-D1
T0805-D1	T0830-D2	T0873-D1
T0801-D1	T0885-D1	T0808-D1
T0889-D1	T0783-D2	T0861-D1
T0851-D1	T0883-D1	T0799D4
T0891-D1	T0822-D1	T0839-D1
T0786-D1	T0847-D1	T0858-D1
T0833-D1	T0768-D1	T0810-D2
T0776-D1	T0807-D1	

Supplementary Table S3. GDT Prediction of SAAPp scoring function for domain based targets from train, test and blind set

TRAIN SET TARGETS			
**Targets**	**Best GDT**	**Selected GDT (Top 1)**	**GDT Loss**
T0852-D1	99.4	99.4	0
T0873-D1	83.5	82.63	0.87
T0826-D2	99.32	99.32	0
T0776-D1	93.98	93.98	0
T0881-D1	69.43	65.59	3.84
T0796-D1	98.87	98.87	0
T0871-D1	62.7	59.48	3.22
T0801-D1	83.17	83.17	0
T0808-D1	99.4	99.4	0
T0879-D1	79.2	76.25	2.95
T0838-D1	99.4	99.25	0.15
T0823-D1	94.44	94.44	0
T0783-D2	99.32	99.32	0
T0891-D1	91.74	86.38	5.36
T0799-D4	99.4	99.32	0.08
T0805-D1	92.82	92.82	0
T0847-D1	98.72	98.72	0
T0821-D1	88.35	83.46	4.89
T0849-D1	94.68	94.44	0.24
T0848-D1	99.25	99.02	0.23
T0822-D1	99.4	98.87	0.53
T0861-D1	99.04	98.64	0.4
T0830-D2	99.32	98.12	1.2
T0807-D1	94.21	94.21	0
T0827-D1	99.1	94.21	4.89
T0858-D1	86.65	86.65	0
T0795-D1	99.25	98.95	0.3
T0760-D1	98.57	98.57	0
T0766-D1	76.89	76.89	0
T0867-D1	97.6	97.6	0
T0764-D1	99.32	99.32	0
T0817-D2	94.91	94.91	0
T0877-D1	70.25	70.25	0
T0851-D1	94.91	94.91	0
T0883-D1	89.4	88.59	0.81
T0762-D1	94.21	93.98	0.23
T0772-D1	99.4	99.4	0
T0839-D1	94.21	93.75	0.46
**Average GDT Loss**			**0.806579**

TEST SET TARGETS			
Targets	Best GDT	Selected GDT (Top 1)	GDT Loss
T0885-D1	87.94	69.3	18.64
T0833-D1	99.17	99.17	0
T0860-D1	81.8	77.39	4.41
T0845-D2	99.32	98.64	0.68
T0835-D1	94.44	94.44	0
T0794-D1	99.02	99.02	0
T0811-D1	94.21	94.21	0
T0819-D1	93.98	93.98	0
T0768-D1	99.17	99.17	0
T0803-D1	99.4	99.25	0.15
T0782-D1	99.4	99.4	0
T0889-D1	87.55	82.74	4.81
T0770-D1	87.45	85.89	1.56
T0810-D2	99.1	99.1	0
T0786-D1	98.95	98.95	0
**Average GDT Loss**			**2.02**
**BLIND SET TARGETS**			
**Targets**	**Best GDT**	**Selected GDT (Top 1)**	**GDT Loss**
T0893-D1	87.28	79.73	7.55
T0895-D1	75.42	71.88	3.54
T0902-D1	60.71	57.36	3.35
T0906-D1	95.19	91.22	3.97
T0910-D1	90.69	88.09	2.6
T0911-D1	65.99	62.5	3.49
T0912-D1	66.42	58.21	8.31
T0913-D1	68.56	66.79	1.77
T0917-D1	85.68	76.32	9.36
T0920-D1	79.21	72.97	6.24
T0921-D1	70.65	63.59	7.06
T0928-D1	63.27	44.06	19.21
T0920-D2	79.68	66.67	13.01
T0943-D2	68.85	64.82	4.03
T0944-D1	76.28	65.81	10.47
T0946-D1	63.8	60.14	3.66
T0947-D1	66.43	60.43	6
T0948-D1	76.68	75	1.68
**Average GDT Loss**			**6.405556**

Supplementary Table S4. Correlation coefficient (PCC) for each target domains in train, test and blind set.

Train Targets	PCC
T0852-D1	0.964
T0873-D1	0.984
T0826-D2	0.960
T0776-D1	0.953
T0881-D1	0.955
T0796-D1	0.968
T0871-D1	0.941
T0801-D1	0.936
T0808-D1	0.959
T0879-D1	0.970
T0838-D1	0.967
T0823-D1	0.947
T0783-D2	0.966
T0891-D1	0.939
T0799-D4	0.968
T0805-D1	0.954
T0847-D1	0.950
T0821-D1	0.900
T0849-D1	0.972
T0848-D1	0.962
T0822-D1	0.966
T0861-D1	0.977
T0830-D2	0.967
T0807-D1	0.957
T0827-D1	0.943
T0858-D1	0.946
T0795-D1	0.959
T0760-D1	0.959
T0766-D1	0.905
T0867-D1	0.993
T0764-D1	0.961
T0817-D2	0.952
T0877-D1	0.951
T0851-D1	0.954
T0883-D1	0.977
T0762-D1	0.959
T0772-D1	0.976
T0839-D1	0.956

Test Targets	PCC
T0833-D1	0.905
T0860-D1	0.832
T0845-D2	0.887
T0835-D1	0.872
T0794-D1	0.885
T0811-D1	0.840
T0819-D1	0.854
T0768-D1	0.952
T0803-D1	0.919
T0782-D1	0.898
T0889-D1	0.819
T0770-D1	0.894
T0810-D2	0.870
T0786-D1	0.890
T0885-D1	0.577

Blind Set (CASP)	PCC
T0893-D2	0.740
T0895-D1	0.830
T0902-D1	0.820
T0906-D1	0.900
T0910-D1	0.870
T0911-D1	0.750
T0912-D1	0.740
T0913-D1	0.800
T0917-D1	0.580
T0920-D2	0.870
T0921-D1	0.860
T0928-D1	0.810
T0942-D1	0.690
T0943-D2	0.820
T0944-D1	0.810
T0946-D2	0.800
T0947-D1	0.620
T0948-D1	0.590

Blind Set (CAMEO)	PCC
5TXR_A	0.830
5XCA_A	0.722
5U7Z_C	0.402
5Z4G_B	0.789
5XVS_B	0.561
5VGU_F	0.879
5OUN_A	0.729
5XB6_L	0.972
6AU1_B	0.862
5W35_B	0.825
5YH0_L	0.776
5XDY_A	0.853
5WEE_D	0.520
5XJV_B	0.681
5OJY_A	0.537
5Z9Y_B	0.819
5TOS_B	0.470
5VH2_D	0.785
5Z68_D	0.700
6CK0_B	0.756
5XOM_B	0.917
5OVY_A	0.691
5ZHZ_A	0.838
5V8C_A	0.810
5X7Y_D	0.853
5UD7_F	0.390
5XD6_B	0.840
5O6C_A	0.438
5XBV_A	0.957
5XPW_A	0.475
5U84_B	0.923
5NVA_A	0.408
6CKG_B	0.810
6CKP_A	0.705
5Y8E_A	0.903
5VFX_H	0.569
5XFL_D	0.675
5U7Z_D	0.837
5OV3_B	0.506
5WJD_A	0.589
5WLY_A	0.847

5Y4B_A	0.627
5XEP_F	0.538
5ZB8_E	0.601
5XEO_B	0.815
5U81_A	0.837
5MM8_A	0.424
5ZI9_D	0.923
5VG2_C	0.691
5X2B_L	0.664
5Z11_B	0.614

Supplementary Table S5. Models domain list of 18 targets of CASP 12 and CAMEO used in blind dataset for testing SAAP scoring function, 6 Features used for prediction is listed for each models with their corresponding GDT.

CASP12							
**Name**	**SAAP**	**Helix Fraction**	**Sheet Fraction**	**Loop Fraction**	**Loop**	**SAAPLoop**	**GDT**
T0893TS005_1-D2.pdb	29.09	0.33	0.23	0.44	74	0.39	87.28
T0893TS005_2-D2.pdb	29.09	0.31	0.22	0.46	78	0.37	79.73
T0893TS005_3-D2.pdb	34.55	0.31	0.25	0.44	75	0.46	83.73
T0893TS005_4-D2.pdb	29.09	0.31	0.21	0.47	80	0.36	76.78
T0893TS005_5-D2.pdb	32.73	0.34	0.2	0.46	78	0.42	77.37
T0893TS016_1-D2.pdb	32.73	0.29	0.21	0.5	85	0.39	75.44
T0893TS026_1-D2.pdb	30.91	0.3	0.2	0.5	85	0.36	75.15
T0893TS026_2-D2.pdb	32.73	0.28	0.2	0.52	88	0.37	76.48
T0893TS026_5-D2.pdb	32.73	0.26	0.24	0.5	85	0.39	74.56
T0893TS028_1-D2.pdb	32.73	0.27	0.22	0.51	86	0.38	74.11
T0893TS048_1-D2.pdb	32.73	0.33	0.22	0.46	77	0.43	79.44
T0893TS077_1-D2.pdb	36.36	0.29	0.22	0.49	82	0.44	73.97
T0893TS077_2-D2.pdb	32.73	0.28	0.2	0.51	87	0.38	69.23
T0893TS077_3-D2.pdb	34.55	0.3	0.21	0.49	83	0.42	74.7
T0893TS077_4-D2.pdb	43.64	0.31	0.21	0.49	82	0.53	63.91
T0893TS077_5-D2.pdb	36.36	0.3	0.21	0.5	84	0.43	71.3
T0893TS119_1-D2.pdb	34.55	0.29	0.21	0.5	84	0.41	79.73
T0893TS180_1-D2.pdb	36.36	0.27	0.2	0.53	90	0.4	56.66
T0893TS180_2-D2.pdb	40	0.3	0.22	0.49	82	0.49	45.71
T0893TS180_3-D2.pdb	45.45	0.31	0.22	0.47	79	0.58	43.64
T0893TS180_4-D2.pdb	36.36	0.25	0.24	0.51	86	0.42	60.06
T0893TS180_5-D2.pdb	30.91	0.25	0.02	0.72	122	0.25	49.85
T0893TS183_1-D2.pdb	36.36	0.31	0.2	0.49	83	0.44	84.61
T0893TS183_2-D2.pdb	27.27	0.27	0.21	0.51	87	0.31	81.51
T0893TS183_3-D2.pdb	34.55	0.28	0.2	0.51	87	0.4	82.84
T0893TS183_4-D2.pdb	34.55	0.33	0.2	0.48	81	0.43	76.78
T0893TS183_5-D2.pdb	36.36	0.36	0.15	0.49	82	0.44	80.47
T0893TS220_1-D2.pdb	30.91	0.34	0.21	0.44	75	0.41	77.07
T0893TS220_2-D2.pdb	34.55	0.33	0.19	0.49	82	0.42	75.44
T0893TS220_3-D2.pdb	32.73	0.33	0.24	0.44	74	0.44	85.21
T0893TS220_4-D2.pdb	30.91	0.34	0.22	0.44	74	0.42	84.17
T0893TS220_5-D2.pdb	32.73	0.33	0.22	0.45	76	0.43	87.28
T0893TS236_1-D2.pdb	38.18	0.3	0.22	0.48	81	0.47	76.33
T0893TS236_3-D2.pdb	36.36	0.25	0.24	0.51	87	0.42	75.59
T0893TS236_4-D2.pdb	32.73	0.27	0.25	0.49	82	0.4	79.88
T0893TS236_5-D2.pdb	34.55	0.23	0.22	0.54	92	0.38	76.33
T0893TS250_1-D2.pdb	34.55	0.28	0.22	0.49	83	0.42	78.4
T0893TS250_2-D2.pdb	29.09	0.28	0.22	0.49	83	0.35	78.4
T0893TS250_3-D2.pdb	32.73	0.28	0.22	0.49	83	0.39	77.96

T0893TS250_4-D2.pdb	30.91	0.28	0.22	0.49	83	0.37	78.7
T0893TS250_5-D2.pdb	29.09	0.28	0.22	0.49	83	0.35	78.55
T0893TS251_2-D2.pdb	30.91	0.26	0.23	0.51	86	0.36	71.89
T0893TS251_3-D2.pdb	34.55	0.29	0.24	0.47	79	0.44	76.33
T0893TS251_4-D2.pdb	34.55	0.29	0.2	0.51	87	0.4	68.05
T0893TS251_5-D2.pdb	32.73	0.3	0.22	0.47	80	0.41	74.7
T0893TS258_1-D2.pdb	34.55	0.32	0.22	0.46	78	0.44	75.74
T0893TS258_2-D2.pdb	27.27	0.28	0.22	0.49	83	0.33	74.7
T0893TS258_3-D2.pdb	30.91	0.3	0.19	0.51	86	0.36	76.03
T0893TS258_4-D2.pdb	30.91	0.33	0.24	0.43	73	0.42	75.89
T0893TS258_5-D2.pdb	30.91	0.32	0.22	0.46	77	0.4	75
T0893TS275_1-D2.pdb	36.36	0.29	0.21	0.5	85	0.43	72.34
T0893TS275_2-D2.pdb	34.55	0.29	0.22	0.49	82	0.42	75
T0893TS275_3-D2.pdb	34.55	0.3	0.23	0.47	79	0.44	77.81
T0893TS275_4-D2.pdb	34.55	0.31	0.23	0.46	78	0.44	79.29
T0893TS275_5-D2.pdb	36.36	0.31	0.24	0.46	77	0.47	78.85
T0893TS284_1-D2.pdb	32.73	0.32	0.22	0.46	77	0.43	66.57
T0893TS284_2-D2.pdb	38.18	0.31	0.21	0.48	81	0.47	62.28
T0893TS284_3-D2.pdb	34.55	0.29	0.24	0.47	80	0.43	75.15
T0893TS284_4-D2.pdb	36.36	0.27	0.22	0.51	86	0.42	73.22
T0893TS284_5-D2.pdb	36.36	0.31	0.18	0.5	85	0.43	65.09
T0893TS287_2-D2.pdb	38.18	0.29	0.21	0.5	84	0.45	70.41
T0893TS287_3-D2.pdb	36.36	0.29	0.24	0.47	80	0.45	76.48
T0893TS287_4-D2.pdb	38.18	0.29	0.25	0.46	77	0.5	80.18
T0893TS287_5-D2.pdb	32.73	0.26	0.23	0.51	86	0.38	76.18
T0893TS313_1-D2.pdb	32.73	0.29	0.2	0.51	87	0.38	81.21
T0893TS313_3-D2.pdb	32.73	0.26	0.2	0.54	92	0.36	80.92
T0893TS313_4-D2.pdb	30.91	0.29	0.2	0.51	87	0.36	81.36
T0893TS313_5-D2.pdb	32.73	0.27	0.2	0.53	90	0.36	80.77
T0893TS321_1-D2.pdb	63.64	0.22	0.14	0.63	107	0.59	16.86
T0893TS321_2-D2.pdb	47.27	0.25	0.14	0.61	103	0.46	18.05
T0893TS321_3-D2.pdb	70.91	0.24	0.13	0.63	106	0.67	17.9
T0893TS321_4-D2.pdb	76.36	0.27	0.11	0.62	104	0.73	15.09
T0893TS321_5-D2.pdb	69.09	0.22	0.14	0.64	108	0.64	16.12
T0893TS345_1-D2.pdb	38.18	0.28	0.24	0.47	80	0.48	74.26
T0893TS345_2-D2.pdb	40	0.27	0.22	0.5	85	0.47	73.37
T0893TS345_3-D2.pdb	34.55	0.3	0.22	0.48	81	0.43	76.18
T0893TS345_4-D2.pdb	32.73	0.3	0.25	0.46	77	0.43	75.59
T0893TS345_5-D2.pdb	32.73	0.3	0.25	0.46	77	0.43	77.07
T0893TS349_1-D2.pdb	38.18	0.29	0.21	0.5	84	0.45	79.73
T0893TS357_1-D2.pdb	40	0.27	0.05	0.67	114	0.35	60.65
T0893TS357_2-D2.pdb	41.82	0.27	0.05	0.68	115	0.36	61.69
T0893TS357_3-D2.pdb	38.18	0.28	0.01	0.71	120	0.32	61.54
T0893TS357_4-D2.pdb	40	0.27	0	0.73	123	0.33	61.39
T0893TS357_5-D2.pdb	38.18	0.28	0	0.72	121	0.32	60.65
T0893TS359_1-D2.pdb	36.36	0.29	0.22	0.49	82	0.44	77.96
T0893TS359_3-D2.pdb	34.55	0.29	0.22	0.49	82	0.42	78.11

T0893TS359_5-D2.pdb	34.55	0.29	0.26	0.45	76	0.45	77.66
T0893TS382_1-D2.pdb	36.36	0.33	0.17	0.5	85	0.43	61.09
T0893TS382_2-D2.pdb	36.36	0.31	0.17	0.51	87	0.42	61.09
T0893TS382_3-D2.pdb	38.18	0.31	0.18	0.5	85	0.45	61.24
T0893TS382_4-D2.pdb	34.55	0.31	0.17	0.51	87	0.4	60.65
T0893TS405_1-D2.pdb	40	0.32	0.2	0.48	81	0.49	76.92
T0893TS405_2-D2.pdb	34.55	0.31	0.24	0.45	76	0.45	76.92
T0893TS405_3-D2.pdb	36.36	0.29	0.24	0.47	80	0.45	80.77
T0893TS405_4-D2.pdb	30.91	0.26	0.2	0.54	91	0.34	83.43
T0893TS405_5-D2.pdb	34.55	0.31	0.2	0.49	83	0.42	78.99
T0893TS407_1-D2.pdb	38.18	0.27	0.22	0.51	86	0.44	76.92
T0893TS407_2-D2.pdb	34.55	0.28	0.14	0.58	98	0.35	74.56
T0893TS407_3-D2.pdb	30.91	0.27	0.11	0.63	106	0.29	77.07
T0893TS407_4-D2.pdb	30.91	0.3	0.17	0.54	91	0.34	74.26
T0893TS407_5-D2.pdb	40	0.29	0.12	0.59	100	0.4	75.3
T0893TS421_1-D2.pdb	32.73	0.25	0.09	0.66	111	0.29	60.5
T0893TS421_2-D2.pdb	30.91	0.25	0.08	0.67	113	0.27	70.86
T0893TS421_3-D2.pdb	27.27	0.27	0.16	0.57	97	0.28	70.71
T0893TS421_4-D2.pdb	32.73	0.3	0.21	0.49	83	0.39	74.56
T0893TS421_5-D2.pdb	32.73	0.25	0.12	0.63	106	0.31	67.6
T0893TS425_1-D2.pdb	36.36	0.29	0.25	0.46	78	0.47	73.97
T0893TS425_2-D2.pdb	38.18	0.3	0.24	0.47	79	0.48	72.19
T0893TS425_3-D2.pdb	36.36	0.28	0.22	0.5	84	0.43	68.79
T0893TS425_4-D2.pdb	34.55	0.28	0.25	0.47	79	0.44	79.59
T0893TS425_5-D2.pdb	34.55	0.28	0.22	0.5	85	0.41	73.08
T0893TS430_1-D2.pdb	32.73	0.24	0.22	0.54	91	0.36	71.3
T0893TS430_2-D2.pdb	34.55	0.27	0.21	0.52	88	0.39	70.86
T0893TS430_3-D2.pdb	34.55	0.3	0.2	0.5	85	0.41	65.83
T0893TS430_4-D2.pdb	34.55	0.32	0.19	0.49	83	0.42	80.18
T0893TS430_5-D2.pdb	36.36	0.31	0.21	0.48	81	0.45	79.29
T0893TS432_1-D2.pdb	34.55	0.34	0.2	0.47	79	0.44	67.6
T0893TS432_2-D2.pdb	34.55	0.33	0.22	0.44	75	0.46	55.77
T0893TS432_3-D2.pdb	34.55	0.34	0.16	0.5	84	0.41	59.02
T0893TS432_4-D2.pdb	34.55	0.33	0.18	0.5	84	0.41	61.24
T0893TS434_1-D2.pdb	56.36	0.3	0.04	0.66	112	0.5	27.37
T0893TS434_2-D2.pdb	47.27	0.29	0.02	0.69	117	0.4	18.79
T0893TS434_3-D2.pdb	45.45	0.28	0	0.72	121	0.38	27.07
T0893TS434_4-D2.pdb	41.82	0.28	0	0.72	121	0.35	27.81
T0893TS434_5-D2.pdb	56.36	0.25	0.01	0.73	124	0.45	21.6
T0893TS444_1-D2.pdb	32.73	0.36	0.25	0.39	66	0.5	78.11
T0893TS444_2-D2.pdb	30.91	0.32	0.22	0.46	78	0.4	76.48
T0893TS444_3-D2.pdb	30.91	0.28	0.22	0.49	83	0.37	73.67
T0893TS444_4-D2.pdb	30.91	0.27	0.22	0.51	86	0.36	75.89
T0893TS444_5-D2.pdb	29.09	0.32	0.26	0.42	71	0.41	73.22
T0893TS446_1-D2.pdb	32.73	0.3	0.21	0.49	82	0.4	69.82
T0893TS446_2-D2.pdb	27.27	0.34	0.22	0.44	75	0.36	72.78
T0893TS446_3-D2.pdb	36.36	0.25	0.23	0.52	88	0.41	73.97

T0893TS446_4-D2.pdb	34.55	0.26	0.2	0.54	91	0.38	68.64
T0893TS446_5-D2.pdb	32.73	0.33	0.21	0.47	79	0.41	79.14
T0893TS451_1-D2.pdb	45.45	0.27	0.17	0.56	94	0.48	60.35
T0893TS451_2-D2.pdb	45.45	0.27	0.14	0.59	100	0.45	57.69
T0893TS451_3-D2.pdb	38.18	0.27	0.15	0.58	98	0.39	60.95
T0893TS451_4-D2.pdb	41.82	0.27	0.18	0.55	93	0.45	60.5
T0893TS451_5-D2.pdb	40	0.28	0.17	0.56	94	0.43	59.62
T0893TS452_1-D2.pdb	34.55	0.3	0.22	0.48	81	0.43	75.89
T0893TS452_2-D2.pdb	34.55	0.29	0.23	0.48	81	0.43	74.26
T0893TS452_3-D2.pdb	34.55	0.27	0.24	0.49	83	0.42	77.07
T0893TS452_4-D2.pdb	36.36	0.3	0.21	0.5	84	0.43	73.52
T0893TS452_5-D2.pdb	32.73	0.26	0.24	0.5	84	0.39	72.63
T0893TS455_1-D2.pdb	45.45	0.33	0.01	0.66	111	0.41	13.46
T0893TS455_2-D2.pdb	60	0.08	0.02	0.89	151	0.4	12.57
T0893TS455_3-D2.pdb	63.64	0.07	0.02	0.91	153	0.42	9.32
T0893TS455_4-D2.pdb	43.64	0.05	0.07	0.88	149	0.29	16.57
T0893TS455_5-D2.pdb	56.36	0.17	0	0.83	140	0.4	11.39
T0893TS464_1-D2.pdb	30.91	0.3	0.18	0.53	89	0.35	73.67
T0893TS464_2-D2.pdb	76.36	0.27	0.01	0.72	121	0.63	15.68
T0893TS464_3-D2.pdb	32.73	0.3	0.18	0.53	89	0.37	73.22
T0893TS464_4-D2.pdb	32.73	0.3	0.18	0.53	89	0.37	73.37
T0893TS464_5-D2.pdb	80	0.23	0	0.77	130	0.62	16.27
T0893TS467_1-D2.pdb	32.73	0.29	0.24	0.47	80	0.41	80.33
T0893TS467_2-D2.pdb	32.73	0.3	0.22	0.48	81	0.4	64.79
T0893TS467_3-D2.pdb	34.55	0.26	0.22	0.52	88	0.39	69.67
T0893TS467_4-D2.pdb	36.36	0.3	0.19	0.51	86	0.42	59.62
T0893TS467_5-D2.pdb	34.55	0.31	0.24	0.46	77	0.45	61.09
T0893TS479_1-D2.pdb	40	0.31	0.2	0.5	84	0.48	83.73
T0893TS479_2-D2.pdb	34.55	0.27	0.2	0.53	90	0.38	80.77
T0893TS479_3-D2.pdb	29.09	0.3	0.2	0.5	85	0.34	77.22
T0893TS479_4-D2.pdb	32.73	0.33	0.22	0.46	77	0.43	83.14
T0893TS479_5-D2.pdb	30.91	0.28	0.19	0.53	90	0.34	80.62
T0893TS495_1-D2.pdb	32.73	0.3	0.22	0.48	81	0.4	75.89
T0893TS495_2-D2.pdb	36.36	0.34	0.23	0.43	73	0.5	65.98
T0893TS495_3-D2.pdb	30.91	0.29	0.22	0.49	82	0.38	75.74
T0893TS495_4-D2.pdb	34.55	0.33	0.21	0.46	77	0.45	66.27
T0893TS495_5-D2.pdb	38.18	0.33	0.2	0.47	79	0.48	62.28
T0895TS001_1-D1.pdb	40.63	0.31	0.08	0.61	73	0.56	50.42
T0895TS001_2-D1.pdb	32.81	0.48	0.13	0.39	47	0.7	68.54
T0895TS001_3-D1.pdb	32.81	0.48	0.13	0.4	48	0.68	68.54
T0895TS001_4-D1.pdb	32.81	0.41	0.13	0.47	56	0.59	70.21
T0895TS001_5-D1.pdb	31.25	0.41	0.14	0.45	54	0.58	70.42
T0895TS004_1-D1.pdb	37.5	0.42	0.13	0.45	54	0.69	72.08
T0895TS004_2-D1.pdb	37.5	0.44	0.03	0.53	63	0.6	65.21
T0895TS004_3-D1.pdb	34.38	0.41	0.04	0.55	66	0.52	22.71
T0895TS004_4-D1.pdb	40.63	0.42	0.02	0.57	68	0.6	57.71
T0895TS004_5-D1.pdb	39.06	0.46	0.05	0.49	59	0.66	42.29

T0895TS005_1-D1.pdb	32.81	0.41	0.13	0.46	55	0.6	70.62
T0895TS005_2-D1.pdb	37.5	0.43	0.12	0.45	54	0.69	65.83
T0895TS005_3-D1.pdb	29.69	0.44	0.09	0.47	56	0.53	72.08
T0895TS005_4-D1.pdb	32.81	0.45	0.13	0.43	51	0.64	66.46
T0895TS005_5-D1.pdb	35.94	0.5	0.11	0.39	47	0.76	66.25
T0895TS011_1-D1.pdb	35.94	0.54	0.13	0.33	39	0.92	72.5
T0895TS011_2-D1.pdb	48.44	0.47	0.07	0.47	56	0.86	29.58
T0895TS011_3-D1.pdb	43.75	0.47	0.03	0.51	61	0.72	35.62
T0895TS011_4-D1.pdb	46.88	0.47	0.05	0.48	58	0.81	32.5
T0895TS011_5-D1.pdb	39.06	0.46	0.08	0.47	56	0.7	35
T0895TS016_1-D1.pdb	37.5	0.51	0.05	0.44	53	0.71	64.17
T0895TS017_1-D1.pdb	28.13	0.44	0.12	0.44	53	0.53	71.67
T0895TS017_2-D1.pdb	34.38	0.44	0.12	0.44	53	0.65	72.5
T0895TS017_3-D1.pdb	34.38	0.44	0.12	0.44	53	0.65	71.67
T0895TS017_4-D1.pdb	34.38	0.42	0.12	0.47	56	0.61	72.08
T0895TS017_5-D1.pdb	31.25	0.42	0.16	0.43	51	0.61	72.29
T0895TS019_1-D1.pdb	29.69	0.47	0.16	0.38	45	0.66	70.21
T0895TS023_1-D1.pdb	31.25	0.45	0.16	0.39	47	0.66	72.29
T0895TS023_2-D1.pdb	28.13	0.45	0.12	0.43	52	0.54	71.88
T0895TS023_3-D1.pdb	28.13	0.44	0.16	0.4	48	0.59	73.33
T0895TS023_4-D1.pdb	29.69	0.44	0.12	0.44	53	0.56	71.88
T0895TS023_5-D1.pdb	31.25	0.44	0.12	0.44	53	0.59	73.33
T0895TS026_1-D1.pdb	67.19	0.29	0.05	0.66	79	0.85	18.33
T0895TS026_2-D1.pdb	59.38	0.4	0	0.6	72	0.82	25.83
T0895TS026_3-D1.pdb	54.69	0.23	0.08	0.69	83	0.66	21.88
T0895TS040_1-D1.pdb	51.56	0.44	0.08	0.48	58	0.89	21.67
T0895TS040_2-D1.pdb	53.13	0.42	0.02	0.57	68	0.78	21.46
T0895TS040_3-D1.pdb	56.25	0.42	0.12	0.47	56	1	21.88
T0895TS040_4-D1.pdb	59.38	0.39	0.13	0.48	57	1.04	21.67
T0895TS040_5-D1.pdb	50	0.42	0.02	0.57	68	0.74	19.17
T0895TS048_1-D1.pdb	37.5	0.48	0.12	0.4	48	0.78	66.04
T0895TS060_2-D1.pdb	37.5	0.44	0.05	0.51	61	0.61	66.88
T0895TS060_3-D1.pdb	32.81	0.4	0.05	0.55	66	0.5	65.21
T0895TS060_4-D1.pdb	34.38	0.41	0.05	0.54	65	0.53	64.79
T0895TS060_5-D1.pdb	35.94	0.41	0.04	0.55	66	0.54	64.79
T0895TS064_1-D1.pdb	35.94	0.43	0.11	0.46	55	0.65	71.04
T0895TS066_1-D1.pdb	57.81	0.39	0.02	0.59	71	0.81	16.04
T0895TS066_2-D1.pdb	50	0.52	0	0.48	58	0.86	26.25
T0895TS066_3-D1.pdb	37.5	0.38	0.05	0.58	69	0.54	20.62
T0895TS066_4-D1.pdb	39.06	0.36	0.07	0.58	69	0.57	22.92
T0895TS073_1-D1.pdb	31.25	0.45	0.11	0.44	53	0.59	68.96
T0895TS073_2-D1.pdb	31.25	0.43	0.12	0.46	55	0.57	74.17
T0895TS073_3-D1.pdb	34.38	0.43	0.12	0.46	55	0.63	73.96
T0895TS073_4-D1.pdb	32.81	0.43	0.12	0.46	55	0.6	72.29
T0895TS073_5-D1.pdb	34.38	0.4	0.08	0.53	63	0.55	65.21
T0895TS077_1-D1.pdb	56.25	0.43	0.1	0.47	56	1	27.08
T0895TS077_3-D1.pdb	54.69	0.39	0.08	0.53	63	0.87	24.79

T0895TS077_4-D1.pdb	57.81	0.41	0.08	0.51	61	0.95	26.25
T0895TS077_5-D1.pdb	54.69	0.39	0.1	0.51	61	0.9	24.79
T0895TS079_1-D1.pdb	34.38	0.44	0.1	0.46	55	0.63	70.83
T0895TS079_3-D1.pdb	32.81	0.42	0.12	0.47	56	0.59	72.08
T0895TS083_1-D1.pdb	42.19	0.53	0.05	0.42	50	0.84	27.29
T0895TS083_2-D1.pdb	35.94	0.48	0.11	0.41	49	0.73	25.21
T0895TS083_3-D1.pdb	37.5	0.48	0.02	0.51	61	0.61	25.62
T0895TS083_4-D1.pdb	48.44	0.34	0.14	0.52	62	0.78	19.38
T0895TS083_5-D1.pdb	45.31	0.43	0	0.57	68	0.67	21.46
T0895TS101_1-D1.pdb	37.5	0.33	0.13	0.53	64	0.59	64.38
T0895TS101_2-D1.pdb	48.44	0.39	0	0.61	73	0.66	33.96
T0895TS101_3-D1.pdb	43.75	0.42	0	0.58	70	0.63	33.54
T0895TS101_5-D1.pdb	45.31	0.43	0.05	0.53	63	0.72	31.46
T0895TS102_1-D1.pdb	35.94	0.49	0.13	0.38	45	0.8	72.08
T0895TS102_2-D1.pdb	34.38	0.51	0.13	0.37	44	0.78	70.21
T0895TS102_3-D1.pdb	35.94	0.49	0.18	0.33	40	0.9	72.92
T0895TS102_4-D1.pdb	34.38	0.48	0.13	0.38	46	0.75	69.38
T0895TS102_5-D1.pdb	29.69	0.48	0.09	0.43	51	0.58	71.67
T0895TS126_1-D1.pdb	43.75	0.47	0.07	0.47	56	0.78	27.71
T0895TS126_2-D1.pdb	43.75	0.51	0.02	0.48	57	0.77	34.38
T0895TS126_3-D1.pdb	46.88	0.48	0.05	0.47	56	0.84	33.75
T0895TS126_4-D1.pdb	46.88	0.52	0.07	0.42	50	0.94	25
T0895TS126_5-D1.pdb	46.88	0.48	0.08	0.44	53	0.88	31.88
T0895TS162_1-D1.pdb	35.94	0.45	0.1	0.45	54	0.67	21.25
T0895TS162_2-D1.pdb	37.5	0.37	0.02	0.62	74	0.51	22.71
T0895TS162_3-D1.pdb	43.75	0.44	0.05	0.51	61	0.72	23.75
T0895TS162_4-D1.pdb	48.44	0.33	0.08	0.58	70	0.69	22.29
T0895TS162_5-D1.pdb	46.88	0.36	0.03	0.61	73	0.64	22.29
T0895TS171_1-D1.pdb	62.5	0.62	0.09	0.29	35	1.79	19.17
T0895TS171_2-D1.pdb	59.38	0.35	0.16	0.49	59	1.01	15.62
T0895TS171_3-D1.pdb	64.06	0.28	0.08	0.63	76	0.84	16.25
T0895TS171_4-D1.pdb	56.25	0.24	0.02	0.74	89	0.63	16.67
T0895TS171_5-D1.pdb	60.94	0.23	0.02	0.76	91	0.67	18.12
T0895TS173_1-D1.pdb	23.44	0.46	0.13	0.42	50	0.47	69.38
T0895TS173_2-D1.pdb	21.88	0.47	0.12	0.42	50	0.44	69.17
T0895TS173_3-D1.pdb	21.88	0.43	0.12	0.45	54	0.41	64.17
T0895TS173_4-D1.pdb	25	0.43	0.11	0.47	56	0.45	64.17
T0895TS173_5-D1.pdb	25	0.44	0.11	0.45	54	0.46	70.21
T0895TS179_1-D1.pdb	32.81	0.53	0.11	0.37	44	0.75	71.88
T0895TS179_2-D1.pdb	28.13	0.49	0.16	0.35	42	0.67	72.29
T0895TS179_3-D1.pdb	32.81	0.52	0.11	0.38	45	0.73	71.88
T0895TS179_4-D1.pdb	31.25	0.47	0.15	0.38	46	0.68	72.08
T0895TS179_5-D1.pdb	32.81	0.53	0.13	0.34	41	0.8	72.29
T0895TS180_1-D1.pdb	32.81	0.47	0.13	0.4	48	0.68	65.83
T0895TS180_2-D1.pdb	40.63	0.22	0.17	0.62	74	0.55	34.17
T0895TS180_3-D1.pdb	51.56	0.08	0.25	0.67	80	0.64	24.38
T0895TS180_4-D1.pdb	56.25	0.26	0.08	0.67	80	0.7	33.12

T0895TS180_5-D1.pdb	46.88	0.36	0.03	0.62	74	0.63	29.79
T0895TS182_1-D1.pdb	34.38	0.2	0	0.8	96	0.36	27.71
T0895TS182_2-D1.pdb	42.19	0.21	0	0.79	95	0.44	22.71
T0895TS182_3-D1.pdb	37.5	0.35	0.02	0.63	76	0.49	21.88
T0895TS182_4-D1.pdb	40.63	0.28	0	0.73	87	0.47	27.92
T0895TS182_5-D1.pdb	39.06	0.33	0	0.67	80	0.49	25.62
T0895TS183_2-D1.pdb	39.06	0.43	0.03	0.53	64	0.61	23.12
T0895TS183_3-D1.pdb	43.75	0.37	0.08	0.56	67	0.65	23.54
T0895TS183_4-D1.pdb	48.44	0.31	0.07	0.63	75	0.65	21.25
T0895TS183_5-D1.pdb	51.56	0.54	0	0.46	55	0.94	23.12
T0895TS203_1-D1.pdb	31.25	0.44	0.13	0.43	52	0.6	67.71
T0895TS207_1-D1.pdb	35.94	0.38	0.11	0.51	61	0.59	56.25
T0895TS207_2-D1.pdb	37.5	0.31	0	0.69	83	0.45	24.17
T0895TS207_3-D1.pdb	39.06	0.29	0.08	0.63	75	0.52	55.42
T0895TS207_4-D1.pdb	40.63	0.29	0.05	0.66	79	0.51	28.75
T0895TS207_5-D1.pdb	51.56	0.18	0	0.82	98	0.53	24.17
T0895TS220_4-D1.pdb	39.06	0.48	0.13	0.39	47	0.83	72.5
T0895TS230_1-D1.pdb	51.56	0.47	0.09	0.44	53	0.97	37.71
T0895TS230_2-D1.pdb	46.88	0.47	0.09	0.44	53	0.88	37.08
T0895TS230_3-D1.pdb	43.75	0.46	0.09	0.45	54	0.81	36.46
T0895TS230_4-D1.pdb	43.75	0.48	0.08	0.44	53	0.83	36.88
T0895TS230_5-D1.pdb	40.63	0.47	0.09	0.44	53	0.77	37.08
T0895TS232_3-D1.pdb	28.13	0.44	0.1	0.46	55	0.51	71.88
T0895TS232_5-D1.pdb	32.81	0.42	0.16	0.43	51	0.64	72.29
T0895TS236_1-D1.pdb	32.81	0.42	0.16	0.43	51	0.64	69.58
T0895TS236_2-D1.pdb	34.38	0.42	0.12	0.47	56	0.61	69.38
T0895TS236_4-D1.pdb	37.5	0.44	0.15	0.41	49	0.77	66.46
T0895TS236_5-D1.pdb	34.38	0.43	0.08	0.5	60	0.57	67.71
T0895TS239_1-D1.pdb	39.06	0.45	0.13	0.42	50	0.78	71.25
T0895TS239_2-D1.pdb	35.94	0.45	0.16	0.39	47	0.76	72.29
T0895TS239_3-D1.pdb	31.25	0.45	0.12	0.43	52	0.6	72.29
T0895TS239_4-D1.pdb	34.38	0.45	0.16	0.39	47	0.73	72.29
T0895TS239_5-D1.pdb	35.94	0.47	0.12	0.42	50	0.72	72.08
T0895TS243_1-D1.pdb	29.69	0.52	0.09	0.39	47	0.63	74.17
T0895TS243_3-D1.pdb	29.69	0.52	0.11	0.38	45	0.66	73.54
T0895TS243_4-D1.pdb	32.81	0.49	0.13	0.38	46	0.71	72.29
T0895TS243_5-D1.pdb	28.13	0.52	0.11	0.38	45	0.63	72.71
T0895TS247_1-D1.pdb	37.5	0.48	0.13	0.4	48	0.78	68.12
T0895TS247_2-D1.pdb	28.13	0.48	0.13	0.4	48	0.59	67.5
T0895TS247_3-D1.pdb	34.38	0.48	0.11	0.42	50	0.69	68.54
T0895TS247_4-D1.pdb	29.69	0.49	0.11	0.4	48	0.62	69.79
T0895TS247_5-D1.pdb	29.69	0.52	0.13	0.36	43	0.69	68.96
T0895TS251_1-D1.pdb	34.38	0.44	0.16	0.4	48	0.72	70.62
T0895TS251_2-D1.pdb	28.13	0.43	0.12	0.45	54	0.52	72.71
T0895TS251_3-D1.pdb	40.63	0.43	0.13	0.44	53	0.77	68.96
T0895TS251_4-D1.pdb	32.81	0.43	0.13	0.43	52	0.63	71.04
T0895TS251_5-D1.pdb	34.38	0.43	0.13	0.43	52	0.66	68.54

T0895TS252_1-D1.pdb	34.38	0.41	0.13	0.46	55	0.63	67.08
T0895TS252_2-D1.pdb	32.81	0.42	0.13	0.45	54	0.61	70.21
T0895TS252_3-D1.pdb	34.38	0.45	0.12	0.43	52	0.66	65.21
T0895TS252_4-D1.pdb	37.5	0.41	0.08	0.52	62	0.6	70.21
T0895TS252_5-D1.pdb	29.69	0.5	0.12	0.38	46	0.65	68.54
T0895TS258_2-D1.pdb	29.69	0.45	0.12	0.43	52	0.57	72.08
T0895TS258_3-D1.pdb	28.13	0.44	0.09	0.47	56	0.5	72.08
T0895TS258_4-D1.pdb	28.13	0.45	0.1	0.45	54	0.52	71.46
T0895TS258_5-D1.pdb	32.81	0.43	0.1	0.47	56	0.59	71.04
T0895TS264_1-D1.pdb	43.75	0.36	0	0.64	77	0.57	28.75
T0895TS264_2-D1.pdb	54.69	0.35	0	0.65	78	0.7	22.92
T0895TS264_3-D1.pdb	54.69	0.37	0	0.63	76	0.72	22.71
T0895TS264_4-D1.pdb	53.13	0.38	0	0.63	75	0.71	23.75
T0895TS264_5-D1.pdb	48.44	0.4	0	0.6	72	0.67	27.92
T0895TS275_1-D1.pdb	37.5	0.47	0.11	0.43	51	0.74	62.71
T0895TS275_2-D1.pdb	32.81	0.47	0.12	0.42	50	0.66	63.12
T0895TS275_3-D1.pdb	42.19	0.44	0.1	0.46	55	0.77	68.54
T0895TS275_5-D1.pdb	35.94	0.46	0.13	0.42	50	0.72	62.29
T0895TS282_1-D1.pdb	45.31	0.45	0.11	0.44	53	0.85	29.17
T0895TS282_2-D1.pdb	50	0.44	0	0.56	67	0.75	26.88
T0895TS282_3-D1.pdb	39.06	0.51	0.08	0.41	49	0.8	33.12
T0895TS282_4-D1.pdb	40.63	0.43	0.1	0.47	56	0.73	49.38
T0895TS282_5-D1.pdb	43.75	0.47	0.02	0.52	62	0.71	27.92
T0895TS287_1-D1.pdb	31.25	0.43	0.15	0.43	51	0.61	71.25
T0895TS287_3-D1.pdb	32.81	0.4	0.12	0.48	58	0.57	71.04
T0895TS287_4-D1.pdb	34.38	0.43	0.14	0.43	51	0.67	65.21
T0895TS287_5-D1.pdb	31.25	0.44	0.16	0.4	48	0.65	71.46
T0895TS303_1-D1.pdb	32.81	0.43	0.12	0.45	54	0.61	67.92
T0895TS303_2-D1.pdb	32.81	0.47	0.08	0.45	54	0.61	73.96
T0895TS303_3-D1.pdb	34.38	0.46	0.16	0.38	46	0.75	66.88
T0895TS303_4-D1.pdb	29.69	0.48	0.13	0.39	47	0.63	70
T0895TS303_5-D1.pdb	37.5	0.38	0.17	0.45	54	0.69	67.71
T0895TS313_1-D1.pdb	32.81	0.46	0.04	0.5	60	0.55	72.71
T0895TS313_2-D1.pdb	32.81	0.46	0.04	0.5	60	0.55	72.29
T0895TS313_3-D1.pdb	34.38	0.48	0.04	0.48	58	0.59	72.08
T0895TS313_4-D1.pdb	31.25	0.48	0.04	0.48	58	0.54	71.88
T0895TS313_5-D1.pdb	34.38	0.47	0.04	0.49	59	0.58	71.67
T0895TS321_1-D1.pdb	82.81	0.42	0.02	0.57	68	1.22	27.29
T0895TS321_2-D1.pdb	60.94	0.43	0	0.57	68	0.9	23.33
T0895TS321_3-D1.pdb	51.56	0.4	0	0.6	72	0.72	22.92
T0895TS321_4-D1.pdb	75	0.43	0	0.57	68	1.1	22.5
T0895TS321_5-D1.pdb	62.5	0.4	0	0.6	72	0.87	24.38
T0895TS322_1-D1.pdb	37.5	0.43	0.03	0.54	65	0.58	25.21
T0895TS322_2-D1.pdb	40.63	0.43	0.06	0.52	62	0.66	29.38
T0895TS322_3-D1.pdb	45.31	0.4	0.03	0.57	68	0.67	27.71
T0895TS322_4-D1.pdb	42.19	0.4	0.02	0.58	70	0.6	28.12
T0895TS322_5-D1.pdb	48.44	0.44	0.07	0.49	59	0.82	25.83

T0895TS324_1-D1.pdb	34.38	0.43	0.13	0.43	52	0.66	71.25
T0895TS324_2-D1.pdb	31.25	0.44	0.13	0.43	51	0.61	69.58
T0895TS324_3-D1.pdb	34.38	0.48	0.15	0.38	45	0.76	74.38
T0895TS324_4-D1.pdb	34.38	0.41	0.13	0.46	55	0.63	70.62
T0895TS324_5-D1.pdb	35.94	0.43	0.13	0.44	53	0.68	69.58
T0895TS325_1-D1.pdb	35.94	0.46	0.17	0.38	45	0.8	70.42
T0895TS325_2-D1.pdb	35.94	0.48	0.13	0.39	47	0.76	65.62
T0895TS325_3-D1.pdb	45.31	0.49	0.02	0.49	59	0.77	25.42
T0895TS325_4-D1.pdb	32.81	0.53	0.09	0.38	46	0.71	66.46
T0895TS325_5-D1.pdb	34.38	0.53	0.13	0.35	42	0.82	66.25
T0895TS330_1-D1.pdb	45.31	0.48	0.07	0.46	55	0.82	21.04
T0895TS330_2-D1.pdb	34.38	0.44	0.12	0.44	53	0.65	45.62
T0895TS330_3-D1.pdb	48.44	0.44	0.03	0.53	63	0.77	27.08
T0895TS330_4-D1.pdb	37.5	0.38	0.08	0.53	64	0.59	25
T0895TS330_5-D1.pdb	46.88	0.42	0.13	0.46	55	0.85	37.92
T0895TS345_1-D1.pdb	29.69	0.48	0.12	0.4	48	0.62	67.92
T0895TS345_2-D1.pdb	31.25	0.49	0.12	0.39	47	0.66	68.33
T0895TS345_3-D1.pdb	34.38	0.49	0.13	0.38	45	0.76	67.29
T0895TS345_4-D1.pdb	35.94	0.47	0.12	0.42	50	0.72	70
T0895TS345_5-D1.pdb	35.94	0.48	0.12	0.4	48	0.75	65.42
T0895TS349_1-D1.pdb	29.69	0.47	0.06	0.48	57	0.52	70.83
T0895TS356_1-D1.pdb	34.38	0.5	0.13	0.38	45	0.76	65.21
T0895TS356_2-D1.pdb	35.94	0.49	0.15	0.36	43	0.84	64.58
T0895TS356_3-D1.pdb	35.94	0.48	0.12	0.4	48	0.75	64.58
T0895TS356_4-D1.pdb	45.31	0.41	0.15	0.44	53	0.85	32.08
T0895TS356_5-D1.pdb	50	0.39	0.18	0.43	51	0.98	32.29
T0895TS357_1-D1.pdb	51.56	0.23	0	0.78	93	0.55	20.42
T0895TS357_2-D1.pdb	53.13	0.25	0	0.75	90	0.59	21.25
T0895TS357_3-D1.pdb	56.25	0.23	0	0.77	92	0.61	20.62
T0895TS357_4-D1.pdb	53.13	0.25	0	0.75	90	0.59	19.38
T0895TS357_5-D1.pdb	53.13	0.27	0	0.73	88	0.6	19.79
T0895TS359_5-D1.pdb	32.81	0.46	0	0.54	65	0.5	56.25
T0895TS363_5-D1.pdb	39.06	0.48	0.14	0.38	46	0.85	68.12
T0895TS382_1-D1.pdb	46.88	0.12	0	0.88	106	0.44	21.46
T0895TS382_3-D1.pdb	43.75	0.18	0	0.83	99	0.44	21.67
T0895TS382_4-D1.pdb	51.56	0.18	0	0.82	98	0.53	21.04
T0895TS382_5-D1.pdb	50	0.22	0	0.78	94	0.53	21.46
T0895TS384_1-D1.pdb	32.81	0.49	0.1	0.41	49	0.67	71.04
T0895TS384_2-D1.pdb	32.81	0.49	0.16	0.35	42	0.78	71.04
T0895TS384_3-D1.pdb	34.38	0.48	0.13	0.39	47	0.73	72.5
T0895TS384_4-D1.pdb	35.94	0.49	0.16	0.35	42	0.86	72.92
T0895TS393_1-D1.pdb	31.25	0.44	0.12	0.44	53	0.59	71.67
T0895TS393_2-D1.pdb	29.69	0.48	0.13	0.4	48	0.62	71.25
T0895TS393_3-D1.pdb	31.25	0.47	0.16	0.38	45	0.69	71.88
T0895TS393_4-D1.pdb	34.38	0.48	0.12	0.41	49	0.7	73.33
T0895TS393_5-D1.pdb	32.81	0.43	0.17	0.41	49	0.67	68.96
T0895TS396_1-D1.pdb	34.38	0.43	0.1	0.47	56	0.61	70.83

T0895TS396_2-D1.pdb	34.38	0.41	0.12	0.48	57	0.6	72.08
T0895TS396_3-D1.pdb	43.75	0.35	0.08	0.58	69	0.63	23.54
T0895TS396_4-D1.pdb	35.94	0.43	0.08	0.48	58	0.62	24.38
T0895TS396_5-D1.pdb	39.06	0.43	0.05	0.52	62	0.63	23.12
T0895TS399_1-D1.pdb	56.25	0.39	0	0.61	73	0.77	25.21
T0895TS399_2-D1.pdb	57.81	0.37	0	0.63	76	0.76	25.42
T0895TS399_3-D1.pdb	59.38	0.42	0	0.58	70	0.85	26.25
T0895TS399_4-D1.pdb	59.38	0.39	0	0.61	73	0.81	24.17
T0895TS399_5-D1.pdb	68.75	0.43	0	0.58	69	1	29.17
T0895TS405_2-D1.pdb	34.38	0.41	0.08	0.51	61	0.56	71.04
T0895TS405_3-D1.pdb	35.94	0.41	0.09	0.5	60	0.6	66.67
T0895TS405_4-D1.pdb	34.38	0.44	0.12	0.44	53	0.65	65.21
T0895TS405_5-D1.pdb	35.94	0.44	0.12	0.44	53	0.68	71.04
T0895TS407_1-D1.pdb	48.44	0.42	0.1	0.48	58	0.84	59.38
T0895TS407_2-D1.pdb	48.44	0.4	0.09	0.51	61	0.79	61.67
T0895TS407_3-D1.pdb	42.19	0.4	0.08	0.52	62	0.68	59.17
T0895TS407_4-D1.pdb	43.75	0.39	0.1	0.51	61	0.72	61.04
T0895TS407_5-D1.pdb	48.44	0.44	0.08	0.48	58	0.84	60.42
T0895TS411_1-D1.pdb	31.25	0.43	0.11	0.47	56	0.56	68.33
T0895TS411_2-D1.pdb	37.5	0.43	0.13	0.43	52	0.72	71.46
T0895TS411_3-D1.pdb	34.38	0.43	0.12	0.46	55	0.63	75.42
T0895TS411_4-D1.pdb	35.94	0.43	0.12	0.46	55	0.65	63.75
T0895TS411_5-D1.pdb	29.69	0.45	0.16	0.39	47	0.63	70.83
T0895TS420_2-D1.pdb	48.44	0.48	0.05	0.48	57	0.85	29.58
T0895TS420_3-D1.pdb	51.56	0.35	0.07	0.58	70	0.74	17.92
T0895TS420_4-D1.pdb	51.56	0.51	0.03	0.46	55	0.94	22.29
T0895TS425_1-D1.pdb	48.44	0.38	0.08	0.53	64	0.76	24.79
T0895TS425_2-D1.pdb	56.25	0.41	0.08	0.51	61	0.92	26.04
T0895TS425_3-D1.pdb	56.25	0.42	0.07	0.52	62	0.91	25.21
T0895TS425_4-D1.pdb	56.25	0.42	0.08	0.5	60	0.94	25.42
T0895TS425_5-D1.pdb	59.38	0.37	0.07	0.57	68	0.87	25
T0895TS432_1-D1.pdb	45.31	0.45	0.05	0.5	60	0.76	34.58
T0895TS432_2-D1.pdb	40.63	0.44	0.07	0.49	59	0.69	31.04
T0895TS432_3-D1.pdb	42.19	0.45	0.03	0.52	62	0.68	35.21
T0895TS432_4-D1.pdb	43.75	0.44	0.04	0.52	62	0.71	28.54
T0895TS432_5-D1.pdb	42.19	0.48	0.02	0.51	61	0.69	27.5
T0895TS434_1-D1.pdb	40.63	0.43	0	0.58	69	0.59	40
T0895TS434_2-D1.pdb	43.75	0.48	0	0.53	63	0.69	38.33
T0895TS434_3-D1.pdb	34.38	0.38	0	0.63	75	0.46	27.08
T0895TS434_4-D1.pdb	40.63	0.38	0	0.62	74	0.55	28.96
T0895TS434_5-D1.pdb	46.88	0.45	0	0.55	66	0.71	25.83
T0895TS436_1-D1.pdb	34.38	0.43	0.02	0.56	67	0.51	20.21
T0895TS436_2-D1.pdb	42.19	0.39	0.07	0.54	65	0.65	21.46
T0895TS436_3-D1.pdb	37.5	0.4	0.11	0.49	59	0.64	27.92
T0895TS436_4-D1.pdb	37.5	0.39	0.07	0.54	65	0.58	27.29
T0895TS436_5-D1.pdb	45.31	0.38	0.03	0.58	70	0.65	23.75
T0895TS439_1-D1.pdb	32.81	0.51	0.17	0.33	39	0.84	71.04

T0895TS439_2-D1.pdb	37.5	0.45	0.13	0.42	50	0.75	72.08
T0895TS439_3-D1.pdb	37.5	0.41	0.12	0.48	57	0.66	71.25
T0895TS439_4-D1.pdb	39.06	0.45	0.1	0.45	54	0.72	69.17
T0895TS439_5-D1.pdb	43.75	0.46	0.05	0.49	59	0.74	33.96
T0895TS441_1-D1.pdb	26.56	0.46	0.12	0.43	51	0.52	70.62
T0895TS441_4-D1.pdb	23.44	0.41	0.14	0.45	54	0.43	64.58
T0895TS441_5-D1.pdb	31.25	0.47	0.12	0.42	50	0.63	67.08
T0895TS443_1-D1.pdb	35.94	0.44	0.13	0.43	52	0.69	61.67
T0895TS443_3-D1.pdb	35.94	0.47	0.13	0.41	49	0.73	58.12
T0895TS443_4-D1.pdb	37.5	0.47	0.13	0.41	49	0.77	60
T0895TS443_5-D1.pdb	32.81	0.47	0.13	0.41	49	0.67	63.33
T0895TS444_2-D1.pdb	34.38	0.48	0.08	0.43	52	0.66	60.83
T0895TS444_3-D1.pdb	65.63	0.75	0	0.25	30	2.19	22.92
T0895TS444_4-D1.pdb	59.38	0.73	0	0.27	32	1.86	23.33
T0895TS444_5-D1.pdb	57.81	0.73	0	0.27	32	1.81	22.5
T0895TS446_2-D1.pdb	78.13	0.7	0	0.3	36	2.17	21.04
T0895TS446_3-D1.pdb	76.56	0.73	0	0.28	33	2.32	23.33
T0895TS446_5-D1.pdb	65.63	0.72	0	0.28	34	1.93	21.67
T0895TS450_1-D1.pdb	32.81	0.5	0.12	0.38	46	0.71	73.33
T0895TS450_2-D1.pdb	34.38	0.53	0.13	0.35	42	0.82	73.12
T0895TS450_3-D1.pdb	34.38	0.5	0.12	0.38	46	0.75	73.96
T0895TS450_4-D1.pdb	35.94	0.47	0.11	0.43	51	0.7	71.25
T0895TS450_5-D1.pdb	35.94	0.44	0.11	0.45	54	0.67	71.46
T0895TS451_1-D1.pdb	50	0.37	0.06	0.58	69	0.72	32.71
T0895TS451_2-D1.pdb	53.13	0.37	0.05	0.58	70	0.76	31.67
T0895TS451_3-D1.pdb	54.69	0.37	0.03	0.6	72	0.76	31.04
T0895TS451_4-D1.pdb	45.31	0.37	0.05	0.58	70	0.65	35.21
T0895TS451_5-D1.pdb	54.69	0.38	0.05	0.57	68	0.8	28.96
T0895TS452_1-D1.pdb	39.06	0.43	0.13	0.43	52	0.75	71.46
T0895TS452_2-D1.pdb	35.94	0.38	0.07	0.55	66	0.54	23.12
T0895TS452_3-D1.pdb	39.06	0.41	0.06	0.53	64	0.61	20.83
T0895TS452_4-D1.pdb	51.56	0.52	0	0.48	58	0.89	26.04
T0895TS452_5-D1.pdb	62.5	0.39	0.02	0.59	71	0.88	16.67
T0895TS455_1-D1.pdb	65.63	0.19	0	0.81	97	0.68	14.17
T0895TS455_2-D1.pdb	60.94	0.15	0	0.85	102	0.6	17.5
T0895TS455_3-D1.pdb	64.06	0.13	0	0.88	105	0.61	15
T0895TS455_4-D1.pdb	50	0.1	0.02	0.88	106	0.47	16.67
T0895TS455_5-D1.pdb	59.38	0.1	0	0.9	108	0.55	13.75
T0895TS456_1-D1.pdb	29.69	0.45	0.12	0.43	52	0.57	66.04
T0895TS456_2-D1.pdb	34.38	0.48	0.12	0.41	49	0.7	67.71
T0895TS456_4-D1.pdb	28.13	0.45	0.13	0.43	51	0.55	63.96
T0895TS456_5-D1.pdb	34.38	0.43	0.11	0.47	56	0.61	67.08
T0895TS464_1-D1.pdb	42.19	0.38	0.07	0.55	66	0.64	63.33
T0895TS464_2-D1.pdb	76.56	0.35	0	0.65	78	0.98	18.75
T0895TS464_3-D1.pdb	43.75	0.36	0.07	0.58	69	0.63	60.83
T0895TS464_4-D1.pdb	40.63	0.38	0.07	0.55	66	0.62	63.75
T0895TS464_5-D1.pdb	71.88	0.33	0	0.68	81	0.89	24.38

T0895TS467_3-D1.pdb	42.19	0.23	0.02	0.75	90	0.47	19.58
T0895TS467_4-D1.pdb	54.69	0.61	0	0.39	47	1.16	23.54
T0895TS467_5-D1.pdb	53.13	0.43	0	0.58	69	0.77	18.12
T0895TS475_1-D1.pdb	31.25	0.48	0.11	0.42	50	0.63	66.67
T0895TS475_2-D1.pdb	32.81	0.42	0.13	0.46	55	0.6	71.04
T0895TS475_3-D1.pdb	31.25	0.48	0.12	0.41	49	0.64	68.75
T0895TS475_4-D1.pdb	34.38	0.48	0.15	0.37	44	0.78	58.75
T0895TS479_2-D1.pdb	35.94	0.43	0.06	0.51	61	0.59	24.38
T0895TS479_3-D1.pdb	54.69	0.5	0	0.5	60	0.91	29.58
T0895TS479_4-D1.pdb	43.75	0.4	0.08	0.52	62	0.71	35.62
T0895TS480_1-D1.pdb	34.38	0.44	0.11	0.45	54	0.64	63.96
T0895TS480_2-D1.pdb	51.56	0.16	0.02	0.83	99	0.52	15.83
T0895TS480_3-D1.pdb	54.69	0.41	0	0.59	71	0.77	17.92
T0895TS480_4-D1.pdb	31.25	0.43	0.11	0.47	56	0.56	71.46
T0895TS480_5-D1.pdb	53.13	0.2	0.04	0.76	91	0.58	16.25
T0895TS483_1-D1.pdb	54.69	0.58	0	0.43	51	1.07	24.79
T0895TS483_2-D1.pdb	46.88	0.57	0	0.43	52	0.9	27.71
T0895TS483_3-D1.pdb	48.44	0.43	0.02	0.56	67	0.72	20.62
T0895TS483_4-D1.pdb	59.38	0.49	0	0.51	61	0.97	24.58
T0895TS483_5-D1.pdb	45.31	0.56	0	0.44	53	0.85	22.5
T0895TS486_2-D1.pdb	39.06	0.46	0.05	0.49	59	0.66	32.71
T0895TS486_3-D1.pdb	35.94	0.36	0.02	0.63	75	0.48	65.42
T0895TS486_4-D1.pdb	34.38	0.43	0.04	0.53	64	0.54	61.25
T0895TS486_5-D1.pdb	32.81	0.38	0.02	0.61	73	0.45	60.62
T0895TS489_1-D1.pdb	40.63	0.36	0.03	0.61	73	0.56	20.42
T0895TS498_1-D1.pdb	31.25	0.44	0.12	0.44	53	0.59	71.04
T0895TS498_2-D1.pdb	39.06	0.47	0.12	0.42	50	0.78	70
T0895TS498_4-D1.pdb	31.25	0.45	0.13	0.43	51	0.61	66.46
T0895TS498_5-D1.pdb	31.25	0.43	0.06	0.51	61	0.51	24.38
T0902TS005_1-D1.pdb	24.72	0.29	0.24	0.47	109	0.23	60.61
T0902TS005_2-D1.pdb	29.21	0.29	0.24	0.47	108	0.27	59.52
T0902TS005_3-D1.pdb	23.6	0.28	0.24	0.48	112	0.21	58.87
T0902TS005_4-D1.pdb	26.97	0.29	0.25	0.45	105	0.26	60.71
T0902TS005_5-D1.pdb	25.84	0.28	0.24	0.48	112	0.23	56.93
T0902TS016_1-D1.pdb	32.58	0.25	0.23	0.53	122	0.27	54.11
T0902TS026_1-D1.pdb	30.34	0.19	0.2	0.6	139	0.22	54.11
T0902TS026_2-D1.pdb	26.97	0.23	0.23	0.54	124	0.22	54.87
T0902TS026_3-D1.pdb	33.71	0.22	0.23	0.55	127	0.27	55.3
T0902TS026_5-D1.pdb	30.34	0.23	0.19	0.58	133	0.23	46
T0902TS048_1-D1.pdb	32.58	0.23	0.24	0.53	122	0.27	55.74
T0902TS077_1-D1.pdb	24.72	0.23	0.22	0.55	128	0.19	55.41
T0902TS077_2-D1.pdb	25.84	0.2	0.23	0.57	131	0.2	55.84
T0902TS077_3-D1.pdb	31.46	0.21	0.2	0.58	135	0.23	54.33
T0902TS077_4-D1.pdb	28.09	0.22	0.22	0.57	131	0.21	52.38
T0902TS077_5-D1.pdb	28.09	0.22	0.23	0.56	129	0.22	56.17
T0902TS119_1-D1.pdb	29.21	0.14	0.15	0.71	163	0.18	52.27
T0902TS166_1-D1.pdb	32.58	0.26	0.22	0.53	122	0.27	54.44

T0902TS180_1-D1.pdb	30.34	0.24	0.23	0.53	123	0.25	48.16
T0902TS180_2-D1.pdb	24.72	0.24	0.23	0.53	123	0.2	46.54
T0902TS180_3-D1.pdb	37.08	0.23	0.23	0.54	125	0.3	28.68
T0902TS180_4-D1.pdb	37.08	0.23	0.22	0.55	128	0.29	29.22
T0902TS180_5-D1.pdb	33.71	0.22	0.01	0.77	177	0.19	25.11
T0902TS183_1-D1.pdb	30.34	0.28	0.22	0.5	116	0.26	57.36
T0902TS183_2-D1.pdb	30.34	0.29	0.14	0.58	133	0.23	58.01
T0902TS183_3-D1.pdb	34.83	0.24	0.13	0.63	146	0.24	44.16
T0902TS183_4-D1.pdb	29.21	0.23	0.11	0.66	152	0.19	46.32
T0902TS183_5-D1.pdb	33.71	0.24	0.17	0.59	137	0.25	51.3
T0902TS220_1-D1.pdb	28.09	0.29	0.24	0.47	109	0.26	55.09
T0902TS220_2-D1.pdb	28.09	0.28	0.24	0.48	112	0.25	56.38
T0902TS220_3-D1.pdb	29.21	0.27	0.22	0.51	118	0.25	56.49
T0902TS220_4-D1.pdb	33.71	0.29	0.23	0.48	110	0.31	57.03
T0902TS220_5-D1.pdb	32.58	0.24	0.19	0.57	132	0.25	55.3
T0902TS236_1-D1.pdb	33.71	0.24	0.2	0.55	128	0.26	57.58
T0902TS236_3-D1.pdb	29.21	0.25	0.22	0.53	123	0.24	53.68
T0902TS236_5-D1.pdb	30.34	0.28	0.24	0.48	111	0.27	57.25
T0902TS250_1-D1.pdb	28.09	0.25	0.23	0.52	119	0.24	56.28
T0902TS250_2-D1.pdb	29.21	0.23	0.23	0.53	123	0.24	57.58
T0902TS250_3-D1.pdb	28.09	0.23	0.22	0.56	129	0.22	54.87
T0902TS250_4-D1.pdb	26.97	0.22	0.23	0.55	126	0.21	55.63
T0902TS250_5-D1.pdb	28.09	0.26	0.23	0.51	117	0.24	54.55
T0902TS251_1-D1.pdb	31.46	0.28	0.24	0.48	112	0.28	49.46
T0902TS251_2-D1.pdb	33.71	0.26	0.24	0.5	116	0.29	50.43
T0902TS251_4-D1.pdb	32.58	0.28	0.23	0.49	113	0.29	48.48
T0902TS258_1-D1.pdb	29.21	0.2	0.23	0.57	131	0.22	54.33
T0902TS258_2-D1.pdb	29.21	0.24	0.24	0.52	121	0.24	55.41
T0902TS258_3-D1.pdb	26.97	0.18	0.21	0.61	140	0.19	55.74
T0902TS258_4-D1.pdb	32.58	0.21	0.23	0.55	128	0.25	51.73
T0902TS258_5-D1.pdb	28.09	0.23	0.23	0.54	125	0.22	52.27
T0902TS275_1-D1.pdb	35.96	0.25	0.2	0.55	128	0.28	44.7
T0902TS275_2-D1.pdb	30.34	0.25	0.23	0.52	120	0.25	52.71
T0902TS275_3-D1.pdb	32.58	0.22	0.23	0.55	128	0.25	52.71
T0902TS275_4-D1.pdb	30.34	0.26	0.23	0.51	118	0.26	54
T0902TS275_5-D1.pdb	32.58	0.19	0.23	0.58	133	0.24	50.22
T0902TS287_1-D1.pdb	32.58	0.23	0.2	0.57	131	0.25	56.28
T0902TS287_3-D1.pdb	26.97	0.24	0.22	0.54	124	0.22	54.11
T0902TS287_5-D1.pdb	31.46	0.25	0.22	0.53	123	0.26	51.95
T0902TS313_2-D1.pdb	29.21	0.14	0.15	0.71	163	0.18	51.95
T0902TS313_3-D1.pdb	25.84	0.15	0.15	0.7	161	0.16	51.73
T0902TS313_4-D1.pdb	25.84	0.17	0.15	0.68	156	0.17	51.95
T0902TS313_5-D1.pdb	26.97	0.16	0.15	0.68	158	0.17	52.27
T0902TS345_1-D1.pdb	29.21	0.27	0.23	0.51	117	0.25	55.09
T0902TS345_2-D1.pdb	32.58	0.27	0.24	0.49	113	0.29	58.44
T0902TS345_4-D1.pdb	31.46	0.27	0.22	0.51	118	0.27	53.03
T0902TS345_5-D1.pdb	37.08	0.26	0.23	0.51	118	0.31	52.27

T0902TS357_1-D1.pdb	35.96	0.16	0.06	0.78	181	0.2	54.11
T0902TS357_2-D1.pdb	31.46	0.13	0.1	0.77	177	0.18	54.11
T0902TS357_3-D1.pdb	31.46	0.14	0.05	0.81	187	0.17	54.11
T0902TS357_4-D1.pdb	33.71	0.14	0.05	0.81	187	0.18	52.6
T0902TS357_5-D1.pdb	34.83	0.2	0.05	0.75	173	0.2	53.46
T0902TS359_2-D1.pdb	34.83	0.23	0.18	0.59	137	0.25	47.84
T0902TS359_4-D1.pdb	34.83	0.12	0.15	0.73	169	0.21	44.37
T0902TS382_1-D1.pdb	34.83	0.18	0.16	0.66	153	0.23	52.16
T0902TS382_2-D1.pdb	28.09	0.05	0.1	0.85	196	0.14	51.95
T0902TS382_3-D1.pdb	26.97	0.08	0.16	0.76	175	0.15	50.87
T0902TS382_4-D1.pdb	32.58	0.05	0.15	0.8	184	0.18	51.62
T0902TS382_5-D1.pdb	26.97	0.09	0.13	0.78	180	0.15	52.81
T0902TS405_1-D1.pdb	29.21	0.23	0.23	0.53	123	0.24	50
T0902TS407_1-D1.pdb	30.34	0.21	0.16	0.63	145	0.21	54.22
T0902TS407_2-D1.pdb	33.71	0.23	0.16	0.6	139	0.24	54.98
T0902TS407_3-D1.pdb	30.34	0.2	0.19	0.61	141	0.22	53.14
T0902TS407_4-D1.pdb	31.46	0.2	0.17	0.63	146	0.22	53.03
T0902TS407_5-D1.pdb	29.21	0.2	0.17	0.63	145	0.2	53.35
T0902TS421_1-D1.pdb	28.09	0.13	0.13	0.74	170	0.17	49.35
T0902TS421_2-D1.pdb	29.21	0.11	0.14	0.75	174	0.17	51.52
T0902TS421_3-D1.pdb	25.84	0.2	0.14	0.65	151	0.17	53.9
T0902TS421_4-D1.pdb	29.21	0.17	0.19	0.64	148	0.2	53.46
T0902TS421_5-D1.pdb	32.58	0.16	0.13	0.71	163	0.2	54.76
T0902TS425_1-D1.pdb	30.34	0.21	0.23	0.56	129	0.24	54.44
T0902TS425_2-D1.pdb	28.09	0.22	0.23	0.55	128	0.22	55.41
T0902TS425_3-D1.pdb	34.83	0.23	0.22	0.55	128	0.27	53.25
T0902TS425_4-D1.pdb	29.21	0.2	0.23	0.57	132	0.22	53.25
T0902TS425_5-D1.pdb	28.09	0.22	0.23	0.55	128	0.22	57.25
T0902TS432_1-D1.pdb	32.58	0.3	0.02	0.68	158	0.21	16.45
T0902TS432_2-D1.pdb	40.45	0.29	0.06	0.65	151	0.27	13.74
T0902TS432_3-D1.pdb	30.34	0.26	0.04	0.69	160	0.19	14.83
T0902TS432_4-D1.pdb	37.08	0.3	0.04	0.66	153	0.24	13.74
T0902TS432_5-D1.pdb	44.94	0.31	0.01	0.68	157	0.29	14.07
T0902TS434_1-D1.pdb	40.45	0.18	0	0.82	189	0.21	10.82
T0902TS434_2-D1.pdb	48.31	0.19	0	0.81	188	0.26	13.2
T0902TS434_3-D1.pdb	41.57	0.14	0	0.86	198	0.21	10.06
T0902TS434_4-D1.pdb	62.92	0.23	0	0.77	179	0.35	12.01
T0902TS434_5-D1.pdb	55.06	0.16	0	0.84	193	0.29	9.74
T0902TS444_1-D1.pdb	23.6	0.24	0.21	0.55	127	0.19	50.33
T0902TS444_2-D1.pdb	28.09	0.23	0.23	0.54	125	0.22	50.11
T0902TS444_3-D1.pdb	32.58	0.21	0.22	0.57	132	0.25	50.33
T0902TS444_4-D1.pdb	30.34	0.22	0.23	0.56	129	0.24	50.54
T0902TS444_5-D1.pdb	24.72	0.22	0.22	0.56	130	0.19	51.3
T0902TS446_1-D1.pdb	32.58	0.26	0.15	0.59	137	0.24	42.53
T0902TS446_2-D1.pdb	38.2	0.26	0.16	0.58	134	0.29	42.64
T0902TS446_4-D1.pdb	39.33	0.2	0.16	0.64	147	0.27	46.43
T0902TS451_1-D1.pdb	46.07	0.21	0.04	0.75	173	0.27	21.32

T0902TS451_2-D1.pdb	44.94	0.21	0.04	0.75	174	0.26	23.81
T0902TS451_3-D1.pdb	46.07	0.2	0.11	0.69	160	0.29	21.54
T0902TS451_4-D1.pdb	43.82	0.21	0.1	0.69	159	0.28	26.08
T0902TS451_5-D1.pdb	40.45	0.19	0.06	0.74	171	0.24	19.8
T0902TS452_1-D1.pdb	33.71	0.23	0.2	0.57	132	0.26	53.14
T0902TS452_2-D1.pdb	33.71	0.19	0.16	0.65	150	0.22	41.02
T0902TS452_3-D1.pdb	34.83	0.16	0.13	0.71	164	0.21	43.4
T0902TS452_4-D1.pdb	28.09	0.22	0.17	0.61	142	0.2	42.97
T0902TS452_5-D1.pdb	29.21	0.25	0.17	0.58	133	0.22	37.34
T0902TS455_1-D1.pdb	56.18	0.03	0	0.97	225	0.25	7.79
T0902TS455_2-D1.pdb	42.7	0.08	0.04	0.88	204	0.21	10.61
T0902TS455_3-D1.pdb	58.43	0.06	0.01	0.93	214	0.27	8.33
T0902TS455_4-D1.pdb	48.31	0.05	0	0.95	220	0.22	7.9
T0902TS455_5-D1.pdb	44.94	0.06	0.06	0.88	203	0.22	8.01
T0902TS464_1-D1.pdb	24.72	0.24	0.2	0.56	130	0.19	43.51
T0902TS464_2-D1.pdb	67.42	0.16	0.05	0.79	182	0.37	9.96
T0902TS464_3-D1.pdb	29.21	0.21	0.17	0.62	143	0.2	38.53
T0902TS464_4-D1.pdb	24.72	0.24	0.19	0.57	132	0.19	44.48
T0902TS464_5-D1.pdb	70.79	0.12	0.03	0.84	195	0.36	11.26
T0902TS467_1-D1.pdb	34.83	0.24	0.16	0.6	138	0.25	38.2
T0902TS467_2-D1.pdb	29.21	0.2	0.22	0.58	134	0.22	53.57
T0902TS467_3-D1.pdb	35.96	0.43	0	0.57	131	0.27	10.17
T0902TS467_4-D1.pdb	40.45	0.13	0.18	0.68	158	0.26	8.87
T0902TS467_5-D1.pdb	39.33	0.37	0.07	0.56	129	0.3	11.04
T0902TS479_1-D1.pdb	26.97	0.27	0.22	0.51	118	0.23	57.68
T0902TS479_2-D1.pdb	30.34	0.26	0.2	0.54	124	0.24	56.17
T0902TS479_3-D1.pdb	25.84	0.26	0.19	0.56	129	0.2	51.73
T0902TS479_4-D1.pdb	34.83	0.26	0.16	0.58	135	0.26	44.26
T0902TS479_5-D1.pdb	28.09	0.23	0.12	0.65	151	0.19	46.65
T0906TS005_1-D1.pdb	18.97	0.25	0.25	0.5	165	0.11	94.22
T0906TS005_2-D1.pdb	20.69	0.25	0.26	0.49	164	0.13	94.67
T0906TS005_3-D1.pdb	19.83	0.25	0.26	0.49	164	0.12	94.75
T0906TS005_4-D1.pdb	18.97	0.26	0.26	0.49	163	0.12	94.75
T0906TS005_5-D1.pdb	19.83	0.25	0.26	0.5	166	0.12	94.67
T0906TS016_1-D1.pdb	18.1	0.26	0.26	0.48	160	0.11	92.49
T0906TS026_1-D1.pdb	22.41	0.25	0.22	0.53	177	0.13	90.69
T0906TS026_3-D1.pdb	22.41	0.26	0.21	0.53	177	0.13	90.39
T0906TS026_5-D1.pdb	21.55	0.26	0.19	0.55	183	0.12	92.12
T0906TS028_1-D1.pdb	20.69	0.25	0.24	0.51	169	0.12	93.17
T0906TS048_1-D1.pdb	19.83	0.26	0.25	0.49	163	0.12	93.84
T0906TS077_1-D1.pdb	20.69	0.26	0.24	0.5	167	0.12	93.69
T0906TS077_2-D1.pdb	21.55	0.27	0.25	0.49	162	0.13	93.77
T0906TS077_3-D1.pdb	19.83	0.26	0.25	0.49	163	0.12	93.69
T0906TS077_4-D1.pdb	22.41	0.26	0.25	0.49	164	0.14	93.77
T0906TS077_5-D1.pdb	20.69	0.27	0.25	0.49	162	0.13	93.62
T0906TS119_1-D1.pdb	21.55	0.24	0.21	0.55	184	0.12	93.92
T0906TS166_1-D1.pdb	22.41	0.26	0.24	0.5	167	0.13	92.72

T0906TS180_1-D1.pdb	26.72	0.23	0.21	0.56	188	0.14	59.46
T0906TS180_2-D1.pdb	24.14	0.24	0.2	0.56	185	0.13	67.12
T0906TS180_3-D1.pdb	21.55	0.26	0.2	0.53	177	0.12	56.08
T0906TS180_4-D1.pdb	32.76	0.01	0.26	0.74	245	0.13	9.69
T0906TS180_5-D1.pdb	29.31	0.2	0	0.8	266	0.11	12.69
T0906TS183_1-D1.pdb	19.83	0.26	0.22	0.52	173	0.11	92.79
T0906TS183_2-D1.pdb	22.41	0.25	0.22	0.53	177	0.13	90.24
T0906TS183_3-D1.pdb	20.69	0.28	0.23	0.5	166	0.12	93.54
T0906TS183_4-D1.pdb	19.83	0.25	0.22	0.53	175	0.11	92.12
T0906TS183_5-D1.pdb	20.69	0.26	0.21	0.53	178	0.12	93.24
T0906TS220_1-D1.pdb	18.97	0.26	0.26	0.47	158	0.12	95.12
T0906TS220_2-D1.pdb	18.1	0.26	0.26	0.48	161	0.11	94.97
T0906TS220_3-D1.pdb	19.83	0.26	0.26	0.49	162	0.12	95.05
T0906TS220_4-D1.pdb	18.97	0.25	0.26	0.49	164	0.12	95.19
T0906TS220_5-D1.pdb	19.83	0.26	0.24	0.5	168	0.12	95.19
T0906TS236_1-D1.pdb	21.55	0.26	0.26	0.48	159	0.14	92.72
T0906TS236_2-D1.pdb	19.83	0.26	0.26	0.48	159	0.12	92.19
T0906TS236_3-D1.pdb	22.41	0.26	0.24	0.5	165	0.14	90.84
T0906TS236_4-D1.pdb	20.69	0.27	0.26	0.47	158	0.13	92.72
T0906TS236_5-D1.pdb	20.69	0.26	0.24	0.5	168	0.12	88.96
T0906TS250_1-D1.pdb	23.28	0.25	0.25	0.51	169	0.14	93.32
T0906TS250_2-D1.pdb	21.55	0.25	0.25	0.51	169	0.13	93.54
T0906TS250_3-D1.pdb	22.41	0.25	0.25	0.5	168	0.13	93.24
T0906TS250_4-D1.pdb	22.41	0.24	0.24	0.51	171	0.13	93.32
T0906TS250_5-D1.pdb	21.55	0.25	0.25	0.51	169	0.13	93.32
T0906TS251_1-D1.pdb	21.55	0.25	0.26	0.49	163	0.13	88.36
T0906TS251_2-D1.pdb	20.69	0.26	0.26	0.48	159	0.13	88.14
T0906TS251_3-D1.pdb	24.14	0.25	0.25	0.5	166	0.15	90.09
T0906TS251_4-D1.pdb	22.41	0.25	0.25	0.5	167	0.13	89.94
T0906TS258_1-D1.pdb	22.41	0.27	0.22	0.51	171	0.13	91.44
T0906TS258_2-D1.pdb	21.55	0.26	0.22	0.52	173	0.12	92.94
T0906TS258_3-D1.pdb	21.55	0.26	0.21	0.53	178	0.12	92.79
T0906TS258_4-D1.pdb	24.14	0.25	0.22	0.53	176	0.14	91.22
T0906TS258_5-D1.pdb	21.55	0.26	0.22	0.52	173	0.12	93.24
T0906TS275_1-D1.pdb	21.55	0.26	0.25	0.5	165	0.13	91.37
T0906TS275_2-D1.pdb	21.55	0.26	0.25	0.5	166	0.13	91.37
T0906TS275_3-D1.pdb	19.83	0.26	0.25	0.5	166	0.12	91.59
T0906TS275_4-D1.pdb	20.69	0.26	0.25	0.49	163	0.13	93.47
T0906TS275_5-D1.pdb	21.55	0.27	0.25	0.49	162	0.13	93.24
T0906TS284_1-D1.pdb	20.69	0.26	0.25	0.49	162	0.13	93.24
T0906TS284_2-D1.pdb	21.55	0.25	0.25	0.5	167	0.13	90.84
T0906TS287_1-D1.pdb	23.28	0.26	0.24	0.5	168	0.14	91.97
T0906TS287_2-D1.pdb	23.28	0.26	0.24	0.5	166	0.14	92.34
T0906TS287_3-D1.pdb	23.28	0.26	0.24	0.5	166	0.14	91.74
T0906TS287_4-D1.pdb	22.41	0.26	0.24	0.5	168	0.13	91.97
T0906TS287_5-D1.pdb	23.28	0.26	0.24	0.5	168	0.14	92.04
T0906TS313_1-D1.pdb	21.55	0.26	0.23	0.52	173	0.12	94.14

T0906TS313_2-D1.pdb	21.55	0.25	0.22	0.53	177	0.12	94.07
T0906TS313_3-D1.pdb	21.55	0.25	0.22	0.53	175	0.12	94.22
T0906TS313_4-D1.pdb	20.69	0.25	0.21	0.54	181	0.11	93.77
T0906TS313_5-D1.pdb	20.69	0.26	0.22	0.53	175	0.12	94.44
T0906TS321_1-D1.pdb	66.38	0.21	0.11	0.69	229	0.29	9.46
T0906TS321_2-D1.pdb	72.41	0.21	0.1	0.68	228	0.32	9.01
T0906TS321_3-D1.pdb	60.34	0.21	0.1	0.69	229	0.26	9.46
T0906TS321_4-D1.pdb	72.41	0.21	0.1	0.69	230	0.31	9.46
T0906TS321_5-D1.pdb	62.07	0.2	0.08	0.72	240	0.26	9.84
T0906TS345_1-D1.pdb	21.55	0.26	0.27	0.47	158	0.14	89.56
T0906TS345_3-D1.pdb	22.41	0.25	0.27	0.48	160	0.14	90.69
T0906TS345_4-D1.pdb	23.28	0.25	0.26	0.49	162	0.14	88.96
T0906TS349_1-D1.pdb	19.83	0.24	0.21	0.55	184	0.11	93.92
T0906TS357_1-D1.pdb	22.41	0.22	0.07	0.71	236	0.09	91.97
T0906TS357_2-D1.pdb	22.41	0.2	0.05	0.74	248	0.09	91.22
T0906TS357_3-D1.pdb	23.28	0.22	0.01	0.77	256	0.09	90.69
T0906TS357_4-D1.pdb	25.86	0.21	0.05	0.74	246	0.11	90.99
T0906TS357_5-D1.pdb	24.14	0.21	0.02	0.77	255	0.09	91.07
T0906TS359_1-D1.pdb	21.55	0.26	0.25	0.49	164	0.13	92.04
T0906TS359_3-D1.pdb	19.83	0.25	0.24	0.5	168	0.12	91.07
T0906TS359_4-D1.pdb	20.69	0.25	0.25	0.5	166	0.12	90.17
T0906TS359_5-D1.pdb	20.69	0.26	0.24	0.5	167	0.12	90.02
T0906TS382_1-D1.pdb	18.1	0.26	0.24	0.5	166	0.11	93.32
T0906TS382_2-D1.pdb	20.69	0.27	0.24	0.49	162	0.13	93.17
T0906TS382_3-D1.pdb	22.41	0.26	0.24	0.5	167	0.13	92.87
T0906TS382_4-D1.pdb	23.28	0.27	0.25	0.48	160	0.15	93.09
T0906TS382_5-D1.pdb	21.55	0.26	0.25	0.49	163	0.13	93.39
T0906TS405_1-D1.pdb	22.41	0.25	0.25	0.5	167	0.13	90.24
T0906TS407_1-D1.pdb	21.55	0.24	0.23	0.53	176	0.12	90.69
T0906TS407_2-D1.pdb	21.55	0.24	0.23	0.53	176	0.12	90.77
T0906TS407_3-D1.pdb	24.14	0.26	0.22	0.53	175	0.14	91.52
T0906TS407_4-D1.pdb	19.83	0.24	0.23	0.52	174	0.11	89.72
T0906TS407_5-D1.pdb	21.55	0.25	0.22	0.53	176	0.12	89.19
T0906TS421_1-D1.pdb	21.55	0.26	0.2	0.54	181	0.12	92.27
T0906TS421_2-D1.pdb	21.55	0.26	0.21	0.54	179	0.12	93.09
T0906TS421_3-D1.pdb	21.55	0.24	0.23	0.53	177	0.12	89.19
T0906TS421_4-D1.pdb	19.83	0.25	0.21	0.55	182	0.11	91.89
T0906TS421_5-D1.pdb	20.69	0.25	0.2	0.55	184	0.11	91.22
T0906TS425_1-D1.pdb	21.55	0.26	0.25	0.5	166	0.13	93.99
T0906TS425_2-D1.pdb	20.69	0.26	0.24	0.5	165	0.13	94.07
T0906TS425_3-D1.pdb	23.28	0.27	0.25	0.49	162	0.14	93.62
T0906TS425_4-D1.pdb	20.69	0.26	0.25	0.49	164	0.13	93.77
T0906TS425_5-D1.pdb	19.83	0.26	0.25	0.49	164	0.12	93.77
T0906TS430_1-D1.pdb	17.24	0.25	0.25	0.5	165	0.1	64.04
T0906TS430_2-D1.pdb	17.24	0.24	0.25	0.51	170	0.1	67.64
T0906TS430_3-D1.pdb	18.1	0.24	0.24	0.52	173	0.1	70.95
T0906TS430_4-D1.pdb	18.97	0.22	0.25	0.54	179	0.11	66.82

T0906TS430_5-D1.pdb	22.41	0.23	0.24	0.53	176	0.13	75.38
T0906TS432_1-D1.pdb	47.41	0.3	0.11	0.59	195	0.24	13.29
T0906TS432_2-D1.pdb	52.59	0.28	0.07	0.65	215	0.24	10.96
T0906TS432_3-D1.pdb	49.14	0.29	0.06	0.65	215	0.23	11.34
T0906TS432_4-D1.pdb	44.83	0.29	0.08	0.64	212	0.21	10.06
T0906TS432_5-D1.pdb	47.41	0.27	0.07	0.66	221	0.21	13.74
T0906TS434_1-D1.pdb	34.48	0.24	0.01	0.75	250	0.14	9.23
T0906TS434_2-D1.pdb	33.62	0.21	0.02	0.77	255	0.13	10.29
T0906TS434_3-D1.pdb	37.07	0.2	0.01	0.79	264	0.14	10.29
T0906TS434_4-D1.pdb	34.48	0.19	0.01	0.8	267	0.13	8.86
T0906TS434_5-D1.pdb	41.38	0.2	0	0.8	267	0.15	15.99
T0906TS444_1-D1.pdb	18.1	0.27	0.25	0.48	159	0.11	91.37
T0906TS444_2-D1.pdb	20.69	0.27	0.25	0.48	160	0.13	90.92
T0906TS444_3-D1.pdb	19.83	0.27	0.25	0.49	162	0.12	89.86
T0906TS444_4-D1.pdb	18.97	0.27	0.25	0.48	161	0.12	91.37
T0906TS444_5-D1.pdb	19.83	0.27	0.23	0.5	165	0.12	84.91
T0906TS446_1-D1.pdb	18.97	0.26	0.25	0.49	163	0.12	90.39
T0906TS446_2-D1.pdb	19.83	0.25	0.25	0.5	166	0.12	92.19
T0906TS446_3-D1.pdb	20.69	0.25	0.24	0.5	168	0.12	89.64
T0906TS451_1-D1.pdb	37.07	0.22	0.02	0.76	252	0.15	15.77
T0906TS451_2-D1.pdb	49.14	0.22	0.06	0.71	238	0.21	13.96
T0906TS451_3-D1.pdb	41.38	0.23	0.08	0.69	231	0.18	12.99
T0906TS451_4-D1.pdb	47.41	0.21	0.04	0.75	250	0.19	24.93
T0906TS451_5-D1.pdb	39.66	0.23	0.06	0.71	237	0.17	28.3
T0906TS452_1-D1.pdb	20.69	0.26	0.26	0.48	161	0.13	92.94
T0906TS452_2-D1.pdb	21.55	0.25	0.26	0.49	164	0.13	90.24
T0906TS452_3-D1.pdb	35.34	0.17	0.12	0.71	236	0.15	8.11
T0906TS452_4-D1.pdb	30.17	0.23	0.06	0.71	238	0.13	8.41
T0906TS452_5-D1.pdb	25.86	0.16	0.18	0.66	221	0.12	11.19
T0906TS455_1-D1.pdb	22.41	0.25	0.26	0.49	164	0.14	88.89
T0906TS455_2-D1.pdb	26.72	0.16	0.18	0.66	220	0.12	49.7
T0906TS455_3-D1.pdb	19.83	0.22	0.26	0.52	173	0.11	87.31
T0906TS455_4-D1.pdb	25	0.19	0.18	0.63	209	0.12	51.13
T0906TS455_5-D1.pdb	27.59	0.2	0	0.8	266	0.1	9.31
T0906TS464_1-D1.pdb	20.69	0.26	0.25	0.49	163	0.13	92.64
T0906TS464_2-D1.pdb	77.59	0.14	0	0.86	288	0.27	6.98
T0906TS464_4-D1.pdb	18.97	0.26	0.24	0.49	164	0.12	92.49
T0906TS464_5-D1.pdb	78.45	0.11	0	0.89	296	0.27	8.41
T0906TS467_1-D1.pdb	22.41	0.24	0.25	0.51	169	0.13	82.28
T0906TS467_2-D1.pdb	18.97	0.24	0.25	0.5	168	0.11	86.94
T0906TS467_3-D1.pdb	24.14	0.25	0.23	0.53	176	0.14	85.14
T0906TS467_4-D1.pdb	31.03	0.24	0.08	0.68	228	0.14	10.21
T0906TS467_5-D1.pdb	26.72	0.27	0.04	0.69	229	0.12	7.66
T0906TS479_1-D1.pdb	18.97	0.27	0.24	0.49	164	0.12	92.94
T0906TS479_2-D1.pdb	21.55	0.26	0.24	0.5	168	0.13	90.02
T0906TS479_3-D1.pdb	19.83	0.28	0.21	0.5	168	0.12	93.17
T0906TS479_4-D1.pdb	20.69	0.26	0.24	0.5	165	0.13	92.04

T0906TS479_5-D1.pdb	20.69	0.25	0.25	0.5	166	0.12	93.62
T0906TS495_1-D1.pdb	22.41	0.26	0.24	0.51	169	0.13	91.29
T0906TS495_2-D1.pdb	21.55	0.25	0.24	0.51	170	0.13	90.54
T0906TS495_3-D1.pdb	20.69	0.24	0.25	0.51	170	0.12	92.27
T0906TS495_4-D1.pdb	21.55	0.24	0.25	0.51	170	0.13	92.34
T0906TS495_5-D1.pdb	25	0.25	0.25	0.51	169	0.15	90.54
T0910TS005_1-D1.pdb	23.28	0.34	0.13	0.53	168	0.14	89.51
T0910TS005_2-D1.pdb	22.41	0.34	0.13	0.53	169	0.13	90.54
T0910TS005_3-D1.pdb	23.28	0.34	0.13	0.53	167	0.14	90.06
T0910TS005_4-D1.pdb	22.41	0.34	0.13	0.52	166	0.14	89.83
T0910TS005_5-D1.pdb	21.55	0.34	0.13	0.53	169	0.13	90.22
T0910TS016_1-D1.pdb	21.55	0.34	0.13	0.53	168	0.13	88.01
T0910TS026_1-D1.pdb	28.45	0.32	0.12	0.56	177	0.16	83.6
T0910TS026_2-D1.pdb	36.21	0.31	0.1	0.59	186	0.19	74.29
T0910TS026_3-D1.pdb	25	0.31	0.11	0.58	185	0.14	85.65
T0910TS026_4-D1.pdb	25.86	0.32	0.12	0.56	178	0.15	85.41
T0910TS026_5-D1.pdb	24.14	0.32	0.13	0.55	175	0.14	84.31
T0910TS028_1-D1.pdb	21.55	0.33	0.13	0.54	171	0.13	85.96
T0910TS048_1-D1.pdb	24.14	0.32	0.13	0.56	176	0.14	87.46
T0910TS077_1-D1.pdb	25	0.33	0.13	0.53	169	0.15	87.7
T0910TS077_2-D1.pdb	22.41	0.33	0.13	0.54	170	0.13	87.54
T0910TS077_3-D1.pdb	20.69	0.33	0.13	0.54	170	0.12	86.99
T0910TS077_4-D1.pdb	24.14	0.34	0.13	0.53	168	0.14	87.78
T0910TS077_5-D1.pdb	25	0.33	0.13	0.53	169	0.15	87.46
T0910TS119_1-D1.pdb	21.55	0.33	0.12	0.55	173	0.12	86.12
T0910TS166_1-D1.pdb	20.69	0.33	0.12	0.55	173	0.12	87.54
T0910TS180_1-D1.pdb	26.72	0.32	0.13	0.55	175	0.15	69.01
T0910TS180_2-D1.pdb	28.45	0.33	0.11	0.56	178	0.16	75.55
T0910TS180_3-D1.pdb	32.76	0.32	0.12	0.56	179	0.18	67.03
T0910TS180_4-D1.pdb	31.9	0.27	0.13	0.6	191	0.17	70.98
T0910TS180_5-D1.pdb	31.9	0.26	0.01	0.73	230	0.14	20.98
T0910TS183_1-D1.pdb	22.41	0.33	0.13	0.54	171	0.13	89.59
T0910TS183_2-D1.pdb	23.28	0.33	0.14	0.53	169	0.14	85.49
T0910TS183_3-D1.pdb	23.28	0.33	0.14	0.52	166	0.14	82.26
T0910TS183_4-D1.pdb	22.41	0.32	0.13	0.55	175	0.13	85.65
T0910TS183_5-D1.pdb	21.55	0.33	0.12	0.55	174	0.12	85.25
T0910TS220_1-D1.pdb	24.14	0.33	0.13	0.54	170	0.14	87.3
T0910TS220_2-D1.pdb	26.72	0.33	0.13	0.54	171	0.16	88.56
T0910TS220_3-D1.pdb	26.72	0.35	0.14	0.52	164	0.16	88.49
T0910TS220_4-D1.pdb	25	0.32	0.13	0.55	174	0.14	90.69
T0910TS220_5-D1.pdb	25.86	0.32	0.13	0.54	172	0.15	90.14
T0910TS236_1-D1.pdb	21.55	0.33	0.13	0.54	171	0.13	89.04
T0910TS236_2-D1.pdb	23.28	0.34	0.13	0.53	169	0.14	88.64
T0910TS236_3-D1.pdb	24.14	0.34	0.13	0.54	170	0.14	87.54
T0910TS236_4-D1.pdb	23.28	0.32	0.13	0.55	173	0.13	89.04
T0910TS236_5-D1.pdb	23.28	0.34	0.13	0.53	168	0.14	88.17
T0910TS250_1-D1.pdb	19.83	0.34	0.13	0.53	167	0.12	85.88

T0910TS250_2-D1.pdb	20.69	0.34	0.13	0.53	169	0.12	85.96
T0910TS250_3-D1.pdb	22.41	0.34	0.13	0.53	169	0.13	85.02
T0910TS250_4-D1.pdb	21.55	0.34	0.13	0.53	169	0.13	85.02
T0910TS250_5-D1.pdb	20.69	0.34	0.13	0.53	169	0.12	85.88
T0910TS251_1-D1.pdb	20.69	0.34	0.13	0.53	169	0.12	74.61
T0910TS251_2-D1.pdb	25	0.32	0.12	0.56	179	0.14	75.95
T0910TS251_3-D1.pdb	22.41	0.32	0.13	0.56	177	0.13	81.31
T0910TS251_4-D1.pdb	31.03	0.32	0.13	0.55	175	0.18	63.01
T0910TS251_5-D1.pdb	19.83	0.3	0.12	0.59	186	0.11	77.84
T0910TS275_1-D1.pdb	26.72	0.28	0.13	0.59	186	0.14	72.56
T0910TS275_2-D1.pdb	26.72	0.3	0.13	0.57	182	0.15	72.56
T0910TS275_3-D1.pdb	32.76	0.31	0.13	0.56	178	0.18	70.58
T0910TS275_4-D1.pdb	29.31	0.3	0.12	0.57	182	0.16	73.27
T0910TS275_5-D1.pdb	24.14	0.3	0.14	0.57	180	0.13	75.08
T0910TS287_1-D1.pdb	20.69	0.33	0.13	0.54	171	0.12	88.96
T0910TS287_2-D1.pdb	17.24	0.33	0.13	0.55	173	0.1	88.09
T0910TS287_3-D1.pdb	17.24	0.33	0.13	0.54	172	0.1	88.25
T0910TS287_4-D1.pdb	19.83	0.33	0.13	0.55	173	0.11	88.8
T0910TS287_5-D1.pdb	20.69	0.33	0.13	0.55	173	0.12	87.7
T0910TS313_1-D1.pdb	25	0.33	0.12	0.55	174	0.14	85.33
T0910TS313_2-D1.pdb	22.41	0.33	0.12	0.55	173	0.13	85.25
T0910TS313_3-D1.pdb	22.41	0.33	0.12	0.55	174	0.13	85.33
T0910TS313_4-D1.pdb	23.28	0.33	0.12	0.55	173	0.13	86.04
T0910TS313_5-D1.pdb	20.69	0.33	0.12	0.55	173	0.12	85.33
T0910TS321_1-D1.pdb	38.79	0.3	0.13	0.57	182	0.21	10.25
T0910TS321_2-D1.pdb	49.14	0.31	0.12	0.58	183	0.27	9.7
T0910TS321_3-D1.pdb	48.28	0.29	0.11	0.6	190	0.25	10.33
T0910TS321_4-D1.pdb	55.17	0.32	0.12	0.57	180	0.31	9.07
T0910TS321_5-D1.pdb	55.17	0.31	0.12	0.57	182	0.3	8.91
T0910TS345_1-D1.pdb	21.55	0.33	0.14	0.53	169	0.13	86.67
T0910TS345_2-D1.pdb	18.1	0.34	0.13	0.53	169	0.11	87.07
T0910TS345_3-D1.pdb	20.69	0.34	0.14	0.52	166	0.12	86.75
T0910TS345_4-D1.pdb	19.83	0.34	0.14	0.52	165	0.12	87.62
T0910TS345_5-D1.pdb	20.69	0.35	0.14	0.52	164	0.13	87.22
T0910TS349_1-D1.pdb	20.69	0.33	0.12	0.55	173	0.12	86.12
T0910TS357_1-D1.pdb	27.59	0.29	0.05	0.66	209	0.13	79.97
T0910TS357_2-D1.pdb	23.28	0.27	0.04	0.69	220	0.11	80.05
T0910TS357_3-D1.pdb	25.86	0.28	0.01	0.71	225	0.11	80.36
T0910TS357_4-D1.pdb	26.72	0.27	0.03	0.7	221	0.12	80.13
T0910TS357_5-D1.pdb	25.86	0.28	0.04	0.68	215	0.12	80.36
T0910TS382_1-D1.pdb	38.79	0.28	0.11	0.62	195	0.2	52.21
T0910TS382_2-D1.pdb	34.48	0.27	0.1	0.63	199	0.17	53.78
T0910TS382_3-D1.pdb	35.34	0.26	0.09	0.64	203	0.17	52.45
T0910TS382_4-D1.pdb	36.21	0.24	0.11	0.65	205	0.18	52.84
T0910TS382_5-D1.pdb	31.9	0.27	0.11	0.62	196	0.16	50.71
T0910TS405_1-D1.pdb	22.41	0.32	0.13	0.55	173	0.13	86.44
T0910TS405_4-D1.pdb	21.55	0.33	0.13	0.54	172	0.13	88.8

T0910TS405_5-D1.pdb	20.69	0.33	0.12	0.55	173	0.12	86.28
T0910TS407_1-D1.pdb	24.14	0.32	0.14	0.54	170	0.14	80.92
T0910TS407_2-D1.pdb	23.28	0.31	0.12	0.58	183	0.13	82.18
T0910TS407_3-D1.pdb	25	0.33	0.12	0.55	174	0.14	81.78
T0910TS407_4-D1.pdb	25	0.32	0.09	0.59	188	0.13	79.97
T0910TS407_5-D1.pdb	25	0.32	0.12	0.56	176	0.14	80.05
T0910TS421_1-D1.pdb	26.72	0.32	0.12	0.57	180	0.15	86.91
T0910TS421_2-D1.pdb	24.14	0.31	0.1	0.59	186	0.13	88.41
T0910TS421_3-D1.pdb	21.55	0.32	0.11	0.57	181	0.12	87.38
T0910TS421_4-D1.pdb	25	0.31	0.11	0.57	182	0.14	85.65
T0910TS421_5-D1.pdb	24.14	0.31	0.09	0.6	191	0.13	87.93
T0910TS425_1-D1.pdb	24.14	0.34	0.13	0.53	168	0.14	87.62
T0910TS425_2-D1.pdb	23.28	0.34	0.13	0.53	168	0.14	87.86
T0910TS425_3-D1.pdb	22.41	0.33	0.13	0.53	169	0.13	87.54
T0910TS425_5-D1.pdb	20.69	0.33	0.13	0.54	171	0.12	87.62
T0910TS432_1-D1.pdb	27.59	0.32	0.09	0.58	185	0.15	34.94
T0910TS432_2-D1.pdb	35.34	0.34	0.05	0.61	194	0.18	26.66
T0910TS432_3-D1.pdb	28.45	0.28	0.01	0.7	223	0.13	24.05
T0910TS432_4-D1.pdb	31.9	0.27	0.03	0.7	222	0.14	20.27
T0910TS434_1-D1.pdb	37.07	0.29	0	0.71	224	0.17	11.36
T0910TS434_2-D1.pdb	39.66	0.28	0	0.72	228	0.17	12.38
T0910TS434_3-D1.pdb	48.28	0.31	0	0.69	219	0.22	12.46
T0910TS434_4-D1.pdb	46.55	0.27	0	0.73	230	0.2	14.35
T0910TS434_5-D1.pdb	39.66	0.3	0.02	0.68	215	0.18	11.91
T0910TS444_1-D1.pdb	21.55	0.32	0.12	0.56	177	0.12	82.97
T0910TS444_2-D1.pdb	19.83	0.32	0.13	0.54	172	0.12	80.2
T0910TS444_3-D1.pdb	19.83	0.32	0.13	0.55	175	0.11	79.57
T0910TS444_4-D1.pdb	20.69	0.32	0.11	0.57	180	0.11	84.46
T0910TS444_5-D1.pdb	19.83	0.33	0.13	0.54	171	0.12	75.47
T0910TS446_1-D1.pdb	18.97	0.33	0.13	0.54	170	0.11	83.99
T0910TS446_2-D1.pdb	16.38	0.33	0.13	0.54	171	0.1	86.59
T0910TS446_5-D1.pdb	18.97	0.32	0.14	0.54	172	0.11	87.86
T0910TS451_1-D1.pdb	38.79	0.28	0.01	0.71	225	0.17	32.18
T0910TS451_2-D1.pdb	40.52	0.27	0	0.73	232	0.17	35.41
T0910TS451_3-D1.pdb	38.79	0.28	0.04	0.68	214	0.18	33.28
T0910TS451_4-D1.pdb	39.66	0.25	0.05	0.7	221	0.18	31.86
T0910TS451_5-D1.pdb	34.48	0.26	0.01	0.73	231	0.15	31.23
T0910TS452_3-D1.pdb	31.03	0.32	0.13	0.55	174	0.18	67.98
T0910TS452_4-D1.pdb	27.59	0.32	0.13	0.56	177	0.16	72.24
T0910TS452_5-D1.pdb	22.41	0.31	0.13	0.56	176	0.13	79.34
T0910TS455_1-D1.pdb	23.28	0.34	0.13	0.53	169	0.14	85.33
T0910TS455_2-D1.pdb	28.45	0.29	0.09	0.62	195	0.15	74.53
T0910TS455_3-D1.pdb	22.41	0.3	0.11	0.59	186	0.12	74.53
T0910TS455_4-D1.pdb	33.62	0.28	0.1	0.62	196	0.17	66.8
T0910TS455_5-D1.pdb	21.55	0.32	0.13	0.55	174	0.12	81.31
T0910TS464_1-D1.pdb	24.14	0.33	0.13	0.54	170	0.14	82.18
T0910TS464_2-D1.pdb	63.79	0.19	0	0.81	258	0.25	9.07

T0910TS464_3-D1.pdb	24.14	0.33	0.13	0.54	170	0.14	81.23
T0910TS464_4-D1.pdb	24.14	0.32	0.13	0.55	175	0.14	82.26
T0910TS464_5-D1.pdb	64.66	0.19	0	0.81	257	0.25	10.88
T0910TS467_1-D1.pdb	19.83	0.33	0.13	0.54	171	0.12	78.55
T0910TS467_2-D1.pdb	23.28	0.32	0.12	0.56	178	0.13	78.55
T0910TS467_3-D1.pdb	25	0.31	0.14	0.56	176	0.14	71.53
T0910TS467_4-D1.pdb	22.41	0.33	0.1	0.57	180	0.12	67.67
T0910TS467_5-D1.pdb	23.28	0.32	0.12	0.55	175	0.13	73.66
T0910TS479_1-D1.pdb	22.41	0.33	0.12	0.55	174	0.13	89.27
T0910TS479_2-D1.pdb	24.14	0.34	0.11	0.55	174	0.14	86.36
T0910TS479_3-D1.pdb	22.41	0.33	0.11	0.56	178	0.13	84.39
T0910TS479_4-D1.pdb	19.83	0.33	0.13	0.54	172	0.12	84.86
T0910TS479_5-D1.pdb	19.83	0.33	0.11	0.55	175	0.11	86.59
T0911TS001_1-D1.pdb	33.55	0.48	0	0.52	215	0.16	13.36
T0911TS004_1-D1.pdb	31.6	0.84	0	0.16	66	0.48	61.4
T0911TS004_2-D1.pdb	27.04	0.85	0	0.15	61	0.44	57.78
T0911TS004_3-D1.pdb	31.92	0.81	0	0.19	81	0.39	56.74
T0911TS004_4-D1.pdb	28.01	0.83	0	0.17	71	0.39	65.2
T0911TS004_5-D1.pdb	31.6	0.83	0	0.17	72	0.44	63.91
T0911TS005_1-D1.pdb	26.38	0.84	0	0.16	65	0.41	58.4
T0911TS005_2-D1.pdb	29.64	0.84	0	0.16	67	0.44	60.54
T0911TS005_3-D1.pdb	29.32	0.83	0	0.17	70	0.42	61.09
T0911TS005_4-D1.pdb	28.01	0.84	0	0.16	67	0.42	62.01
T0911TS005_5-D1.pdb	30.29	0.88	0	0.12	50	0.61	58.09
T0911TS011_1-D1.pdb	31.27	0.85	0	0.15	64	0.49	62.32
T0911TS011_2-D1.pdb	31.27	0.83	0	0.17	69	0.45	63.54
T0911TS011_3-D1.pdb	30.62	0.83	0	0.17	71	0.43	62.56
T0911TS011_4-D1.pdb	31.27	0.82	0	0.18	77	0.41	61.52
T0911TS011_5-D1.pdb	29.64	0.81	0	0.19	79	0.38	63.36
T0911TS016_1-D1.pdb	31.92	0.75	0	0.25	104	0.31	53.31
T0911TS017_1-D1.pdb	31.92	0.76	0	0.24	99	0.32	52.63
T0911TS017_2-D1.pdb	31.92	0.75	0	0.25	104	0.31	52.51
T0911TS017_3-D1.pdb	31.92	0.76	0	0.24	98	0.33	52.63
T0911TS017_4-D1.pdb	33.55	0.76	0	0.24	102	0.33	52.27
T0911TS017_5-D1.pdb	31.92	0.76	0	0.24	102	0.31	52.57
T0911TS019_1-D1.pdb	30.29	0.82	0	0.18	76	0.4	51.72
T0911TS022_1-D1.pdb	37.46	0.33	0	0.67	279	0.13	6.99
T0911TS022_2-D1.pdb	33.22	0.3	0	0.7	292	0.11	6.43
T0911TS022_4-D1.pdb	33.88	0.27	0	0.73	303	0.11	6.74
T0911TS022_5-D1.pdb	39.41	0.33	0	0.67	278	0.14	6.37
T0911TS023_1-D1.pdb	31.6	0.74	0	0.26	108	0.29	57.84
T0911TS023_2-D1.pdb	31.6	0.75	0	0.25	105	0.3	57.41
T0911TS023_3-D1.pdb	29.97	0.71	0	0.29	121	0.25	48.59
T0911TS023_4-D1.pdb	28.99	0.71	0	0.29	121	0.24	48.65
T0911TS023_5-D1.pdb	31.92	0.79	0	0.21	87	0.37	52.15
T0911TS026_1-D1.pdb	35.83	0.73	0	0.27	111	0.32	43.32
T0911TS026_2-D1.pdb	32.9	0.63	0	0.37	155	0.21	9.74

T0911TS026_4-D1.pdb	31.92	0.59	0	0.41	173	0.18	9.99
T0911TS026_5-D1.pdb	29.64	0.6	0	0.4	166	0.18	11.58
T0911TS028_1-D1.pdb	33.88	0.74	0	0.26	107	0.32	55.39
T0911TS040_1-D1.pdb	55.7	0.76	0	0.24	98	0.57	10.48
T0911TS040_2-D1.pdb	61.24	0.79	0	0.21	86	0.71	8.95
T0911TS040_3-D1.pdb	42.35	0.78	0	0.22	92	0.46	9.25
T0911TS040_4-D1.pdb	66.12	0.78	0	0.22	91	0.73	9.07
T0911TS040_5-D1.pdb	50.49	0.77	0	0.23	94	0.54	9.87
T0911TS042_1-D1.pdb	26.71	0.84	0	0.16	65	0.41	58.27
T0911TS042_2-D1.pdb	30.62	0.74	0	0.26	109	0.28	54.53
T0911TS042_3-D1.pdb	33.88	0.77	0	0.23	95	0.36	53.74
T0911TS042_4-D1.pdb	31.92	0.74	0	0.26	107	0.3	65.93
T0911TS042_5-D1.pdb	30.62	0.76	0	0.24	99	0.31	53.25
T0911TS048_1-D1.pdb	33.88	0.8	0	0.2	85	0.4	53.37
T0911TS060_1-D1.pdb	31.27	0.76	0	0.24	102	0.31	51.78
T0911TS060_2-D1.pdb	30.29	0.75	0	0.25	106	0.29	53.19
T0911TS060_3-D1.pdb	28.34	0.73	0	0.27	112	0.25	52.82
T0911TS060_4-D1.pdb	28.99	0.72	0	0.28	118	0.25	53
T0911TS060_5-D1.pdb	31.92	0.72	0	0.28	118	0.27	53.37
T0911TS064_1-D1.pdb	28.99	0.88	0	0.12	48	0.6	58.33
T0911TS066_1-D1.pdb	33.88	0.66	0	0.34	140	0.24	50
T0911TS066_2-D1.pdb	32.25	0.64	0	0.36	152	0.21	45.9
T0911TS066_3-D1.pdb	36.48	0.76	0	0.24	102	0.36	44.91
T0911TS066_4-D1.pdb	35.18	0.74	0	0.26	109	0.32	52.45
T0911TS066_5-D1.pdb	33.88	0.71	0	0.29	119	0.28	54.84
T0911TS067_2-D1.pdb	26.71	0.84	0	0.16	66	0.4	61.03
T0911TS067_4-D1.pdb	29.97	0.81	0	0.19	79	0.38	62.01
T0911TS067_5-D1.pdb	27.04	0.82	0	0.18	75	0.36	61.03
T0911TS073_1-D1.pdb	25.73	0.85	0	0.15	64	0.4	56.43
T0911TS073_2-D1.pdb	25.41	0.82	0	0.18	74	0.34	59.74
T0911TS073_3-D1.pdb	27.36	0.82	0	0.18	75	0.36	61.89
T0911TS073_4-D1.pdb	29.97	0.84	0	0.16	67	0.45	56.8
T0911TS073_5-D1.pdb	29.32	0.86	0	0.14	60	0.49	61.46
T0911TS077_1-D1.pdb	28.01	0.79	0	0.21	87	0.32	53.25
T0911TS077_2-D1.pdb	30.29	0.77	0	0.23	94	0.32	52.88
T0911TS077_3-D1.pdb	28.01	0.77	0	0.23	97	0.29	53.43
T0911TS077_4-D1.pdb	29.32	0.78	0	0.22	92	0.32	52.88
T0911TS077_5-D1.pdb	29.32	0.77	0	0.23	95	0.31	53.98
T0911TS079_2-D1.pdb	32.25	0.75	0	0.25	104	0.31	53.49
T0911TS079_3-D1.pdb	32.9	0.81	0	0.19	78	0.42	60.85
T0911TS079_4-D1.pdb	31.27	0.81	0	0.19	81	0.39	60.54
T0911TS079_5-D1.pdb	33.55	0.79	0	0.21	87	0.39	54.29
T0911TS101_1-D1.pdb	32.57	0.76	0	0.24	101	0.32	54.29
T0911TS101_2-D1.pdb	31.6	0.78	0	0.22	91	0.35	54.41
T0911TS102_5-D1.pdb	27.04	0.7	0	0.3	126	0.21	50.55
T0911TS119_1-D1.pdb	35.18	0.67	0	0.33	139	0.25	55.7
T0911TS126_1-D1.pdb	31.27	0.8	0	0.2	83	0.38	12.07

T0911TS126_2-D1.pdb	34.2	0.82	0	0.18	75	0.46	14.15
T0911TS126_3-D1.pdb	39.74	0.83	0	0.17	71	0.56	14.58
T0911TS126_4-D1.pdb	34.2	0.8	0	0.2	84	0.41	14.64
T0911TS126_5-D1.pdb	42.35	0.82	0	0.18	75	0.56	13.91
T0911TS162_1-D1.pdb	24.1	0.47	0	0.53	220	0.11	9.5
T0911TS162_2-D1.pdb	22.48	0.45	0	0.55	228	0.1	9.31
T0911TS162_3-D1.pdb	22.15	0.45	0	0.55	228	0.1	9.5
T0911TS162_4-D1.pdb	24.76	0.5	0	0.5	210	0.12	8.15
T0911TS162_5-D1.pdb	21.82	0.45	0	0.54	227	0.1	6.99
T0911TS166_1-D1.pdb	34.85	0.74	0	0.26	110	0.32	52.94
T0911TS171_1-D1.pdb	35.18	0.72	0	0.28	118	0.3	53.98
T0911TS171_2-D1.pdb	35.83	0.73	0	0.27	111	0.32	53.8
T0911TS173_1-D1.pdb	25.08	0.84	0	0.16	67	0.37	57.41
T0911TS173_2-D1.pdb	23.78	0.82	0	0.18	77	0.31	57.17
T0911TS173_3-D1.pdb	24.1	0.82	0	0.18	73	0.33	59.87
T0911TS173_4-D1.pdb	24.1	0.81	0	0.19	79	0.31	58.15
T0911TS173_5-D1.pdb	23.78	0.77	0	0.23	95	0.25	60.42
T0911TS179_1-D1.pdb	30.29	0.75	0	0.25	104	0.29	48.1
T0911TS179_2-D1.pdb	30.29	0.79	0	0.21	87	0.35	49.51
T0911TS179_3-D1.pdb	28.99	0.74	0	0.26	109	0.27	47.18
T0911TS179_4-D1.pdb	32.25	0.75	0	0.25	103	0.31	49.02
T0911TS179_5-D1.pdb	31.6	0.78	0	0.22	93	0.34	55.21
T0911TS180_1-D1.pdb	34.85	0.77	0	0.23	97	0.36	48.77
T0911TS180_2-D1.pdb	31.27	0.74	0	0.26	109	0.29	33.64
T0911TS180_3-D1.pdb	37.13	0.73	0	0.27	112	0.33	38.17
T0911TS180_4-D1.pdb	33.22	0.73	0	0.27	112	0.3	43.2
T0911TS180_5-D1.pdb	24.1	0.75	0	0.25	104	0.23	13.36
T0911TS182_1-D1.pdb	23.78	0.57	0	0.43	180	0.13	10.42
T0911TS182_2-D1.pdb	23.13	0.52	0	0.48	202	0.11	18.02
T0911TS182_3-D1.pdb	19.87	0.5	0	0.5	207	0.1	12.87
T0911TS182_4-D1.pdb	24.1	0.44	0	0.56	233	0.1	13.91
T0911TS182_5-D1.pdb	23.78	0.55	0	0.45	187	0.13	11.52
T0911TS183_2-D1.pdb	34.85	0.83	0	0.17	72	0.48	58.09
T0911TS183_3-D1.pdb	33.22	0.77	0	0.23	94	0.35	56.74
T0911TS183_4-D1.pdb	31.27	0.79	0	0.21	88	0.36	59.62
T0911TS183_5-D1.pdb	33.88	0.74	0	0.26	108	0.31	58.64
T0911TS188_1-D1.pdb	33.88	0.72	0	0.28	117	0.29	54.66
T0911TS192_1-D1.pdb	41.04	0.75	0	0.25	103	0.4	25.31
T0911TS218_1-D1.pdb	33.88	0.71	0	0.29	120	0.28	55.88
T0911TS220_4-D1.pdb	29.64	0.83	0	0.17	71	0.42	65.26
T0911TS220_5-D1.pdb	29.64	0.82	0	0.18	75	0.4	64.71
T0911TS230_1-D1.pdb	37.46	0.74	0	0.26	109	0.34	30.76
T0911TS230_2-D1.pdb	36.16	0.74	0	0.26	108	0.33	30.94
T0911TS230_3-D1.pdb	36.48	0.73	0	0.27	114	0.32	31.37
T0911TS230_4-D1.pdb	35.5	0.73	0	0.27	113	0.31	30.58
T0911TS230_5-D1.pdb	36.81	0.74	0	0.26	107	0.34	30.88
T0911TS232_3-D1.pdb	33.55	0.67	0	0.33	139	0.24	54.9

T0911TS232_4-D1.pdb	32.9	0.68	0	0.32	134	0.25	56.92
T0911TS236_1-D1.pdb	30.94	0.78	0	0.22	92	0.34	49.69
T0911TS236_2-D1.pdb	29.64	0.76	0	0.24	98	0.3	50.49
T0911TS236_4-D1.pdb	31.92	0.76	0	0.24	99	0.32	63.36
T0911TS236_5-D1.pdb	30.29	0.76	0	0.24	99	0.31	55.02
T0911TS239_3-D1.pdb	26.06	0.84	0	0.16	66	0.39	61.4
T0911TS239_4-D1.pdb	26.38	0.85	0	0.15	64	0.41	58.64
T0911TS239_5-D1.pdb	27.36	0.84	0	0.16	67	0.41	59.13
T0911TS243_1-D1.pdb	28.01	0.89	0	0.11	44	0.64	57.97
T0911TS243_2-D1.pdb	27.04	0.9	0	0.1	43	0.63	57.66
T0911TS243_3-D1.pdb	27.36	0.87	0	0.13	55	0.5	57.78
T0911TS243_4-D1.pdb	27.36	0.87	0	0.13	54	0.51	57.41
T0911TS243_5-D1.pdb	28.66	0.9	0	0.1	43	0.67	57.66
T0911TS247_1-D1.pdb	27.69	0.84	0	0.16	67	0.41	58.09
T0911TS247_2-D1.pdb	27.69	0.84	0	0.16	65	0.43	56.86
T0911TS247_3-D1.pdb	26.71	0.81	0	0.19	79	0.34	53.25
T0911TS247_4-D1.pdb	28.66	0.83	0	0.17	72	0.4	54.53
T0911TS247_5-D1.pdb	26.71	0.82	0	0.18	74	0.36	59.8
T0911TS250_1-D1.pdb	32.25	0.75	0	0.25	105	0.31	56.98
T0911TS250_2-D1.pdb	32.9	0.75	0	0.25	106	0.31	56.86
T0911TS250_3-D1.pdb	31.6	0.75	0	0.25	106	0.3	57.54
T0911TS250_4-D1.pdb	31.27	0.75	0	0.25	105	0.3	57.17
T0911TS250_5-D1.pdb	33.55	0.76	0	0.24	101	0.33	57.05
T0911TS251_1-D1.pdb	32.9	0.74	0	0.26	108	0.3	52.88
T0911TS251_2-D1.pdb	35.18	0.73	0	0.27	114	0.31	45.59
T0911TS252_1-D1.pdb	28.01	0.83	0	0.17	71	0.39	63.23
T0911TS252_2-D1.pdb	27.04	0.84	0	0.16	67	0.4	59.44
T0911TS252_3-D1.pdb	28.66	0.81	0	0.19	78	0.37	62.62
T0911TS252_4-D1.pdb	26.71	0.81	0	0.19	81	0.33	59.68
T0911TS252_5-D1.pdb	29.97	0.83	0	0.17	71	0.42	62.07
T0911TS258_1-D1.pdb	28.01	0.72	0	0.28	117	0.24	42.4
T0911TS258_2-D1.pdb	29.32	0.74	0	0.26	108	0.27	43.2
T0911TS258_3-D1.pdb	28.66	0.76	0	0.24	100	0.29	45.34
T0911TS258_4-D1.pdb	28.01	0.74	0	0.26	109	0.26	44.98
T0911TS258_5-D1.pdb	28.66	0.76	0	0.24	98	0.29	44.42
T0911TS264_1-D1.pdb	42.02	0.74	0	0.26	109	0.39	14.46
T0911TS264_2-D1.pdb	49.84	0.72	0	0.28	116	0.43	11.4
T0911TS264_3-D1.pdb	44.3	0.72	0	0.28	117	0.38	9.93
T0911TS264_4-D1.pdb	43.97	0.65	0	0.35	144	0.31	7.84
T0911TS264_5-D1.pdb	52.44	0.73	0	0.27	114	0.46	9.68
T0911TS275_1-D1.pdb	55.05	0.45	0	0.55	231	0.24	24.75
T0911TS275_2-D1.pdb	54.4	0.45	0	0.55	231	0.24	24.94
T0911TS275_3-D1.pdb	57	0.47	0	0.53	219	0.26	25.8
T0911TS275_4-D1.pdb	56.35	0.45	0	0.55	228	0.25	23.96
T0911TS275_5-D1.pdb	56.35	0.48	0	0.52	215	0.26	25.37
T0911TS287_2-D1.pdb	32.57	0.77	0	0.23	97	0.34	65.99
T0911TS287_3-D1.pdb	33.22	0.74	0	0.26	107	0.31	54.17

T0911TS287_4-D1.pdb	29.64	0.77	0	0.23	95	0.31	50.61
T0911TS287_5-D1.pdb	31.92	0.71	0	0.29	120	0.27	57.9
T0911TS300_1-D1.pdb	20.2	0.22	0	0.78	325	0.06	8.03
T0911TS300_2-D1.pdb	23.13	0.25	0.02	0.73	305	0.08	6.8
T0911TS300_3-D1.pdb	21.17	0.28	0.01	0.71	297	0.07	6.8
T0911TS300_4-D1.pdb	24.43	0.28	0	0.71	298	0.08	7.84
T0911TS300_5-D1.pdb	24.76	0.22	0	0.78	324	0.08	7.35
T0911TS303_1-D1.pdb	27.69	0.85	0	0.15	64	0.43	61.15
T0911TS303_2-D1.pdb	30.94	0.82	0	0.18	74	0.42	64.83
T0911TS303_3-D1.pdb	28.01	0.82	0	0.18	76	0.37	62.32
T0911TS303_4-D1.pdb	27.69	0.85	0	0.15	62	0.45	58.7
T0911TS303_5-D1.pdb	27.36	0.82	0	0.18	75	0.36	61.58
T0911TS313_2-D1.pdb	31.92	0.69	0	0.31	130	0.25	56.62
T0911TS313_3-D1.pdb	33.55	0.68	0	0.32	134	0.25	56.92
T0911TS313_4-D1.pdb	33.55	0.68	0	0.32	132	0.25	56.98
T0911TS313_5-D1.pdb	31.92	0.67	0	0.33	136	0.23	56.68
T0911TS320_1-D1.pdb	29.32	0.8	0	0.2	85	0.34	53.98
T0911TS320_2-D1.pdb	28.99	0.75	0	0.25	106	0.27	52.88
T0911TS320_4-D1.pdb	28.99	0.76	0	0.24	99	0.29	54.1
T0911TS321_1-D1.pdb	41.69	0.74	0	0.26	107	0.39	8.7
T0911TS321_2-D1.pdb	58.63	0.72	0	0.28	116	0.51	7.9
T0911TS321_3-D1.pdb	41.69	0.7	0	0.3	125	0.33	10.85
T0911TS321_4-D1.pdb	54.72	0.74	0	0.26	110	0.5	7.9
T0911TS321_5-D1.pdb	47.56	0.72	0	0.28	116	0.41	9.62
T0911TS324_1-D1.pdb	28.34	0.83	0	0.17	70	0.4	63.91
T0911TS324_2-D1.pdb	27.36	0.85	0	0.15	64	0.43	63.3
T0911TS324_3-D1.pdb	27.04	0.84	0	0.16	67	0.4	64.15
T0911TS324_4-D1.pdb	28.01	0.82	0	0.18	73	0.38	63.91
T0911TS324_5-D1.pdb	27.69	0.84	0	0.16	65	0.43	63.97
T0911TS325_1-D1.pdb	30.29	0.87	0	0.13	54	0.56	61.95
T0911TS325_2-D1.pdb	28.99	0.85	0	0.15	62	0.47	62.44
T0911TS325_3-D1.pdb	28.34	0.85	0	0.15	63	0.45	61.27
T0911TS325_4-D1.pdb	29.97	0.84	0	0.16	68	0.44	59.74
T0911TS325_5-D1.pdb	29.64	0.85	0	0.15	61	0.49	60.11
T0911TS345_1-D1.pdb	30.62	0.79	0	0.21	87	0.35	50.55
T0911TS345_3-D1.pdb	31.6	0.77	0	0.23	95	0.33	47.12
T0911TS345_4-D1.pdb	33.55	0.78	0	0.22	93	0.36	54.96
T0911TS345_5-D1.pdb	32.57	0.78	0	0.22	91	0.36	58.21
T0911TS349_1-D1.pdb	36.16	0.67	0	0.33	139	0.26	55.7
T0911TS357_1-D1.pdb	35.18	0.6	0	0.4	168	0.21	54.41
T0911TS357_2-D1.pdb	35.83	0.58	0	0.42	177	0.2	54.29
T0911TS357_3-D1.pdb	36.81	0.55	0	0.45	187	0.2	54.29
T0911TS357_4-D1.pdb	37.46	0.56	0	0.44	183	0.2	54.47
T0911TS357_5-D1.pdb	33.88	0.56	0	0.44	182	0.19	54.96
T0911TS359_1-D1.pdb	34.53	0.76	0	0.24	99	0.35	37.13
T0911TS359_2-D1.pdb	31.27	0.74	0	0.26	109	0.29	55.45
T0911TS359_5-D1.pdb	31.92	0.69	0	0.31	129	0.25	48.65

T0911TS363_4-D1.pdb	28.34	0.72	0	0.28	116	0.24	45.47
T0911TS382_1-D1.pdb	30.94	0.74	0	0.26	110	0.28	59.5
T0911TS382_2-D1.pdb	29.97	0.74	0	0.26	107	0.28	59.5
T0911TS382_3-D1.pdb	30.62	0.71	0	0.29	121	0.25	59.25
T0911TS382_4-D1.pdb	29.32	0.75	0	0.25	103	0.28	60.23
T0911TS382_5-D1.pdb	31.6	0.73	0	0.27	114	0.28	61.21
T0911TS384_1-D1.pdb	30.29	0.88	0	0.12	51	0.59	58.27
T0911TS384_4-D1.pdb	29.32	0.84	0	0.16	65	0.45	61.83
T0911TS384_5-D1.pdb	30.94	0.84	0	0.16	66	0.47	61.03
T0911TS393_1-D1.pdb	26.06	0.84	0	0.16	68	0.38	60.72
T0911TS393_2-D1.pdb	26.06	0.83	0	0.17	72	0.36	63.79
T0911TS393_3-D1.pdb	26.71	0.83	0	0.17	72	0.37	62.32
T0911TS393_4-D1.pdb	24.76	0.84	0	0.16	66	0.38	58.52
T0911TS393_5-D1.pdb	24.76	0.83	0	0.17	71	0.35	60.97
T0911TS396_1-D1.pdb	31.6	0.8	0	0.2	82	0.39	60.78
T0911TS396_2-D1.pdb	32.57	0.81	0	0.19	81	0.4	60.66
T0911TS396_3-D1.pdb	30.94	0.76	0	0.24	98	0.32	59.62
T0911TS396_4-D1.pdb	32.57	0.82	0	0.18	76	0.43	57.9
T0911TS396_5-D1.pdb	34.85	0.83	0	0.17	72	0.48	58.15
T0911TS399_1-D1.pdb	42.67	0.71	0	0.29	119	0.36	12.13
T0911TS399_2-D1.pdb	43.32	0.73	0	0.27	113	0.38	12.01
T0911TS399_3-D1.pdb	42.35	0.71	0	0.29	119	0.36	12.07
T0911TS399_4-D1.pdb	44.95	0.72	0	0.28	116	0.39	12.26
T0911TS399_5-D1.pdb	42.02	0.71	0	0.29	122	0.34	12.01
T0911TS405_1-D1.pdb	33.55	0.75	0	0.25	104	0.32	52.02
T0911TS405_3-D1.pdb	33.22	0.76	0	0.24	101	0.33	51.84
T0911TS405_4-D1.pdb	33.55	0.76	0	0.24	102	0.33	51.35
T0911TS405_5-D1.pdb	34.2	0.77	0	0.23	97	0.35	53.74
T0911TS407_1-D1.pdb	33.22	0.78	0	0.22	92	0.36	57.72
T0911TS407_2-D1.pdb	31.92	0.75	0	0.25	103	0.31	58.46
T0911TS407_3-D1.pdb	32.57	0.74	0	0.26	107	0.3	58.64
T0911TS407_4-D1.pdb	33.88	0.79	0	0.21	88	0.38	59.8
T0911TS407_5-D1.pdb	34.53	0.76	0	0.24	99	0.35	56.86
T0911TS411_1-D1.pdb	28.01	0.82	0	0.18	74	0.38	63.3
T0911TS411_2-D1.pdb	27.04	0.83	0	0.17	70	0.39	65.62
T0911TS411_3-D1.pdb	29.64	0.82	0	0.18	73	0.41	60.78
T0911TS411_4-D1.pdb	31.27	0.83	0	0.17	70	0.45	60.72
T0911TS411_5-D1.pdb	26.38	0.82	0	0.18	76	0.35	60.6
T0911TS420_1-D1.pdb	33.55	0.57	0	0.43	181	0.19	7.9
T0911TS420_2-D1.pdb	36.16	0.52	0	0.48	202	0.18	9.5
T0911TS420_3-D1.pdb	30.62	0.54	0	0.46	190	0.16	9.74
T0911TS420_4-D1.pdb	34.85	0.59	0	0.41	169	0.21	10.48
T0911TS420_5-D1.pdb	34.2	0.44	0.05	0.51	214	0.16	8.09
T0911TS421_2-D1.pdb	33.55	0.66	0	0.34	140	0.24	55.64
T0911TS421_5-D1.pdb	32.57	0.66	0.01	0.33	139	0.23	47.24
T0911TS425_3-D1.pdb	28.01	0.77	0	0.23	97	0.29	53.19
T0911TS425_4-D1.pdb	29.97	0.78	0	0.22	90	0.33	53.31

T0911TS432_1-D1.pdb	34.2	0.82	0	0.18	74	0.46	33.64
T0911TS432_2-D1.pdb	38.11	0.78	0	0.22	90	0.42	33.64
T0911TS432_3-D1.pdb	34.85	0.76	0	0.24	102	0.34	31.56
T0911TS432_4-D1.pdb	34.53	0.81	0	0.19	81	0.43	36.95
T0911TS432_5-D1.pdb	38.11	0.77	0	0.23	95	0.4	26.9
T0911TS434_1-D1.pdb	34.53	0.57	0	0.43	178	0.19	10.23
T0911TS434_4-D1.pdb	37.13	0.53	0	0.47	195	0.19	10.54
T0911TS434_5-D1.pdb	46.58	0.58	0	0.42	175	0.27	10.29
T0911TS439_1-D1.pdb	30.29	0.87	0	0.13	54	0.56	58.58
T0911TS439_2-D1.pdb	25.41	0.85	0	0.15	61	0.42	62.81
T0911TS439_3-D1.pdb	29.97	0.84	0	0.16	65	0.46	61.95
T0911TS439_4-D1.pdb	28.34	0.71	0	0.29	123	0.23	50.18
T0911TS439_5-D1.pdb	29.32	0.76	0	0.24	102	0.29	50.37
T0911TS441_1-D1.pdb	26.06	0.82	0	0.18	77	0.34	58.21
T0911TS441_2-D1.pdb	24.76	0.83	0	0.17	70	0.35	58.15
T0911TS441_4-D1.pdb	25.08	0.82	0	0.18	76	0.33	60.17
T0911TS441_5-D1.pdb	25.08	0.81	0	0.19	80	0.31	57.84
T0911TS443_1-D1.pdb	30.94	0.81	0	0.19	80	0.39	42.71
T0911TS443_2-D1.pdb	32.25	0.81	0	0.19	79	0.41	32.35
T0911TS443_3-D1.pdb	32.25	0.8	0	0.2	84	0.38	33.76
T0911TS443_4-D1.pdb	30.29	0.81	0	0.19	78	0.39	27.51
T0911TS443_5-D1.pdb	34.2	0.76	0	0.24	98	0.35	28.55
T0911TS444_1-D1.pdb	28.01	0.82	0	0.18	77	0.36	53.19
T0911TS444_4-D1.pdb	31.27	0.7	0	0.3	126	0.25	56.43
T0911TS444_5-D1.pdb	29.64	0.71	0	0.29	122	0.24	44.06
T0911TS446_1-D1.pdb	29.64	0.8	0	0.2	84	0.35	59.44
T0911TS446_2-D1.pdb	29.97	0.81	0	0.19	79	0.38	59.38
T0911TS446_3-D1.pdb	36.48	0.73	0	0.27	114	0.32	52.57
T0911TS446_4-D1.pdb	33.22	0.77	0	0.23	96	0.35	49.88
T0911TS450_1-D1.pdb	30.62	0.85	0	0.15	63	0.49	62.93
T0911TS450_2-D1.pdb	29.97	0.82	0	0.18	73	0.41	60.11
T0911TS450_3-D1.pdb	30.62	0.83	0	0.17	72	0.43	62.5
T0911TS450_4-D1.pdb	29.97	0.85	0	0.15	63	0.48	63.11
T0911TS450_5-D1.pdb	30.62	0.83	0	0.17	71	0.43	62.19
T0911TS451_1-D1.pdb	39.74	0.82	0	0.18	77	0.52	49.08
T0911TS451_2-D1.pdb	38.11	0.83	0	0.17	71	0.54	50.67
T0911TS451_3-D1.pdb	38.44	0.81	0	0.19	81	0.47	50.61
T0911TS451_4-D1.pdb	40.07	0.81	0	0.19	80	0.5	47.3
T0911TS451_5-D1.pdb	41.37	0.8	0	0.2	82	0.5	48.59
T0911TS452_1-D1.pdb	33.55	0.73	0	0.27	113	0.3	54.72
T0911TS452_2-D1.pdb	33.88	0.74	0	0.26	107	0.32	52.27
T0911TS452_3-D1.pdb	35.5	0.75	0	0.25	103	0.34	44.85
T0911TS452_4-D1.pdb	29.97	0.66	0	0.34	141	0.21	45.9
T0911TS452_5-D1.pdb	36.16	0.68	0	0.32	135	0.27	49.76
T0911TS456_1-D1.pdb	28.66	0.85	0	0.15	63	0.45	59.62
T0911TS456_2-D1.pdb	27.69	0.84	0	0.16	66	0.42	56.8
T0911TS456_3-D1.pdb	27.36	0.82	0	0.18	75	0.36	59.44

T0911TS456_4-D1.pdb	29.97	0.86	0	0.14	60	0.5	60.05
T0911TS456_5-D1.pdb	25.73	0.82	0	0.18	77	0.33	59.8
T0911TS464_1-D1.pdb	29.32	0.75	0	0.25	105	0.28	56.19
T0911TS464_2-D1.pdb	68.08	0.55	0	0.44	184	0.37	8.03
T0911TS464_3-D1.pdb	28.34	0.75	0	0.25	104	0.27	56.25
T0911TS464_4-D1.pdb	28.34	0.75	0	0.25	104	0.27	56.19
T0911TS464_5-D1.pdb	73.94	0.68	0	0.32	132	0.56	9.44
T0911TS467_1-D1.pdb	33.55	0.65	0	0.35	144	0.23	43.08
T0911TS467_3-D1.pdb	29.64	0.71	0	0.29	120	0.25	48.41
T0911TS475_1-D1.pdb	26.71	0.87	0	0.13	53	0.5	58.95
T0911TS475_2-D1.pdb	28.34	0.85	0	0.15	61	0.46	58.82
T0911TS475_4-D1.pdb	29.64	0.85	0	0.15	62	0.48	58.58
T0911TS475_5-D1.pdb	27.04	0.83	0	0.17	70	0.39	57.35
T0911TS479_2-D1.pdb	33.88	0.82	0	0.18	76	0.45	57.78
T0911TS479_3-D1.pdb	29.97	0.76	0	0.24	99	0.3	59.07
T0911TS479_4-D1.pdb	31.6	0.77	0	0.23	96	0.33	59.74
T0911TS479_5-D1.pdb	32.57	0.76	0	0.24	101	0.32	56.37
T0911TS480_1-D1.pdb	33.22	0.74	0	0.26	108	0.31	54.72
T0911TS480_2-D1.pdb	32.25	0.78	0	0.22	92	0.35	42.65
T0911TS480_3-D1.pdb	35.18	0.58	0	0.42	175	0.2	9.99
T0911TS480_4-D1.pdb	32.25	0.86	0	0.14	58	0.56	61.15
T0911TS483_1-D1.pdb	30.29	0.71	0	0.29	123	0.25	48.22
T0911TS483_2-D1.pdb	32.9	0.58	0	0.42	175	0.19	8.46
T0911TS483_3-D1.pdb	37.79	0.59	0	0.41	173	0.22	7.29
T0911TS483_4-D1.pdb	36.48	0.58	0	0.42	175	0.21	7.17
T0911TS483_5-D1.pdb	37.46	0.63	0	0.37	153	0.24	7.9
T0911TS486_2-D1.pdb	29.64	0.78	0	0.22	91	0.33	62.74
T0911TS486_3-D1.pdb	27.69	0.76	0	0.24	98	0.28	55.88
T0911TS486_4-D1.pdb	28.66	0.75	0	0.25	106	0.27	51.16
T0911TS486_5-D1.pdb	28.66	0.72	0	0.28	118	0.24	52.08
T0911TS489_1-D1.pdb	43	0.52	0.01	0.47	198	0.22	7.11
T0911TS498_1-D1.pdb	27.69	0.81	0	0.19	79	0.35	62.01
T0911TS498_2-D1.pdb	25.73	0.84	0	0.16	65	0.4	58.4
T0911TS498_3-D1.pdb	30.94	0.74	0	0.26	107	0.29	58.64
T0911TS498_4-D1.pdb	29.64	0.88	0	0.12	50	0.59	58.09
T0911TS498_5-D1.pdb	26.06	0.84	0	0.16	66	0.39	61.03
T0912TS001_1-D1.pdb	37.5	0.06	0.15	0.79	327	0.11	8.7
T0912TS004_1-D1.pdb	16.18	0.04	0.15	0.81	336	0.05	59.36
T0912TS004_2-D1.pdb	15.44	0.04	0.17	0.79	327	0.05	64.25
T0912TS004_3-D1.pdb	22.06	0.03	0.17	0.8	332	0.07	66.24
T0912TS004_4-D1.pdb	19.12	0.03	0.17	0.8	332	0.06	66.42
T0912TS004_5-D1.pdb	18.38	0.01	0.17	0.81	337	0.05	58.63
T0912TS005_1-D1.pdb	22.79	0.05	0.22	0.72	300	0.08	63.53
T0912TS005_2-D1.pdb	23.53	0.04	0.29	0.67	276	0.09	59.78
T0912TS005_3-D1.pdb	25	0.08	0.23	0.69	286	0.09	60.51
T0912TS005_4-D1.pdb	19.12	0.05	0.23	0.72	299	0.06	62.08
T0912TS005_5-D1.pdb	21.32	0.06	0.28	0.67	276	0.08	59.6

T0912TS011_1-D1.pdb	19.85	0.07	0.21	0.72	297	0.07	64.31
T0912TS011_2-D1.pdb	26.47	0.07	0.19	0.74	307	0.09	49.58
T0912TS011_3-D1.pdb	21.32	0.07	0.2	0.73	303	0.07	60.51
T0912TS011_4-D1.pdb	19.85	0.06	0.2	0.75	310	0.06	56.94
T0912TS011_5-D1.pdb	22.79	0.06	0.18	0.76	314	0.07	55.98
T0912TS016_1-D1.pdb	22.79	0.06	0.21	0.74	305	0.07	38.59
T0912TS017_1-D1.pdb	22.79	0.03	0.24	0.73	303	0.08	60.45
T0912TS017_2-D1.pdb	22.79	0.03	0.21	0.76	315	0.07	60.99
T0912TS017_3-D1.pdb	23.53	0.03	0.22	0.75	310	0.08	60.99
T0912TS017_4-D1.pdb	23.53	0.03	0.21	0.76	315	0.07	60.93
T0912TS017_5-D1.pdb	23.53	0.03	0.21	0.76	315	0.07	60.87
T0912TS019_1-D1.pdb	35.29	0.03	0.18	0.79	327	0.11	9.12
T0912TS022_1-D1.pdb	47.79	0.3	0	0.7	289	0.17	4.41
T0912TS022_2-D1.pdb	49.26	0.35	0	0.65	268	0.18	4.53
T0912TS022_3-D1.pdb	50	0.35	0	0.65	268	0.19	4.53
T0912TS022_4-D1.pdb	47.79	0.36	0	0.64	264	0.18	5.07
T0912TS022_5-D1.pdb	44.85	0.31	0	0.69	284	0.16	4.11
T0912TS023_1-D1.pdb	18.38	0.05	0.21	0.74	306	0.06	55.86
T0912TS023_2-D1.pdb	20.59	0.05	0.2	0.75	309	0.07	55.56
T0912TS023_3-D1.pdb	19.85	0.05	0.21	0.75	309	0.06	54.95
T0912TS023_4-D1.pdb	19.12	0.06	0.21	0.74	305	0.06	55.13
T0912TS023_5-D1.pdb	19.85	0.06	0.2	0.74	308	0.06	54.29
T0912TS026_1-D1.pdb	26.47	0.01	0.15	0.84	347	0.08	39.01
T0912TS026_2-D1.pdb	41.18	0.03	0.12	0.85	353	0.12	31.22
T0912TS026_4-D1.pdb	38.97	0.02	0.1	0.88	364	0.11	24.76
T0912TS026_5-D1.pdb	33.82	0	0.14	0.86	355	0.1	31.64
T0912TS040_1-D1.pdb	46.32	0.07	0.04	0.9	371	0.12	8.09
T0912TS040_2-D1.pdb	45.59	0.07	0.05	0.89	367	0.12	6.88
T0912TS040_3-D1.pdb	41.91	0.06	0.06	0.88	364	0.12	8.39
T0912TS040_4-D1.pdb	46.32	0.06	0.01	0.92	382	0.12	6.04
T0912TS040_5-D1.pdb	44.85	0.07	0.02	0.91	376	0.12	6.52
T0912TS042_1-D1.pdb	21.32	0.01	0.24	0.74	308	0.07	56.46
T0912TS042_2-D1.pdb	22.79	0.04	0.29	0.66	275	0.08	59.6
T0912TS042_4-D1.pdb	25	0.08	0.23	0.69	286	0.09	60.63
T0912TS042_5-D1.pdb	21.32	0.02	0.22	0.76	313	0.07	56.16
T0912TS048_1-D1.pdb	27.94	0.12	0.21	0.67	278	0.1	52.9
T0912TS060_1-D1.pdb	22.79	0.06	0.11	0.83	343	0.07	32.25
T0912TS060_2-D1.pdb	24.26	0.07	0.14	0.79	326	0.07	39.31
T0912TS060_3-D1.pdb	17.65	0.06	0.19	0.76	314	0.06	57.25
T0912TS060_4-D1.pdb	19.12	0.06	0.19	0.75	311	0.06	55.92
T0912TS060_5-D1.pdb	24.26	0.07	0.14	0.79	327	0.07	38.77
T0912TS064_1-D1.pdb	19.85	0.04	0.18	0.78	322	0.06	57.49
T0912TS066_1-D1.pdb	35.29	0.03	0.19	0.78	324	0.11	17.15
T0912TS066_2-D1.pdb	32.35	0.02	0.14	0.84	346	0.09	45.35
T0912TS066_3-D1.pdb	33.82	0.05	0.21	0.74	308	0.11	33.58
T0912TS066_4-D1.pdb	27.21	0.03	0.26	0.71	295	0.09	19.2
T0912TS066_5-D1.pdb	16.18	0.03	0.22	0.75	312	0.05	53.14

T0912TS067_1-D1.pdb	69.85	0.08	0	0.92	379	0.18	6.22
T0912TS067_4-D1.pdb	16.91	0.04	0.14	0.83	342	0.05	61.11
T0912TS073_1-D1.pdb	23.53	0.03	0.18	0.79	326	0.07	60.93
T0912TS073_2-D1.pdb	22.79	0.03	0.17	0.79	329	0.07	53.2
T0912TS073_3-D1.pdb	27.21	0.05	0.3	0.66	272	0.1	59.24
T0912TS073_4-D1.pdb	27.21	0.08	0.24	0.68	283	0.1	58.88
T0912TS073_5-D1.pdb	19.85	0.05	0.22	0.73	302	0.07	60.93
T0912TS077_1-D1.pdb	39.71	0.01	0.15	0.84	347	0.11	43.18
T0912TS077_2-D1.pdb	38.24	0.01	0.14	0.85	350	0.11	44.2
T0912TS077_3-D1.pdb	36.76	0.01	0.16	0.82	341	0.11	41.55
T0912TS077_4-D1.pdb	38.24	0.01	0.19	0.8	330	0.12	41.85
T0912TS077_5-D1.pdb	38.97	0.01	0.15	0.84	346	0.11	42.03
T0912TS079_4-D1.pdb	21.32	0.05	0.16	0.79	329	0.06	54.17
T0912TS079_5-D1.pdb	22.06	0.02	0.24	0.75	309	0.07	56.52
T0912TS101_1-D1.pdb	27.21	0.12	0.21	0.67	278	0.1	33.58
T0912TS101_2-D1.pdb	27.21	0.11	0.18	0.71	293	0.09	34
T0912TS102_2-D1.pdb	21.32	0.03	0.17	0.79	329	0.06	55.25
T0912TS102_4-D1.pdb	36.03	0.18	0.16	0.66	272	0.13	4.71
T0912TS114_3-D1.pdb	29.41	0.02	0.19	0.79	327	0.09	34.36
T0912TS114_5-D1.pdb	27.94	0.03	0.2	0.77	320	0.09	35.02
T0912TS119_1-D1.pdb	16.18	0.03	0.11	0.86	354	0.05	57.97
T0912TS126_1-D1.pdb	58.82	0.06	0.05	0.89	368	0.16	5.98
T0912TS126_2-D1.pdb	71.32	0.05	0.05	0.9	374	0.19	7.13
T0912TS126_3-D1.pdb	59.56	0.03	0.11	0.86	354	0.17	7.61
T0912TS126_4-D1.pdb	58.82	0.02	0.04	0.94	390	0.15	5.8
T0912TS126_5-D1.pdb	58.82	0.03	0.04	0.93	384	0.15	5.43
T0912TS145_1-D1.pdb	22.79	0.09	0.15	0.77	317	0.07	7.67
T0912TS145_2-D1.pdb	28.68	0.11	0.08	0.82	338	0.08	7.25
T0912TS145_3-D1.pdb	27.94	0.09	0.1	0.8	333	0.08	6.76
T0912TS145_4-D1.pdb	25	0.11	0.14	0.76	314	0.08	6.1
T0912TS145_5-D1.pdb	22.06	0.12	0.07	0.81	335	0.07	7
T0912TS162_1-D1.pdb	19.85	0.06	0.09	0.86	354	0.06	7.61
T0912TS162_2-D1.pdb	18.38	0.05	0.13	0.82	339	0.05	6.04
T0912TS162_3-D1.pdb	19.12	0.06	0.1	0.84	348	0.05	5.74
T0912TS162_4-D1.pdb	21.32	0.06	0.09	0.84	349	0.06	6.28
T0912TS162_5-D1.pdb	27.94	0.06	0.08	0.86	356	0.08	6.34
T0912TS171_1-D1.pdb	37.5	0.43	0.05	0.53	218	0.17	5.86
T0912TS171_2-D1.pdb	49.26	0.12	0.02	0.86	356	0.14	5.43
T0912TS171_3-D1.pdb	38.97	0.22	0.05	0.73	303	0.13	5.62
T0912TS171_4-D1.pdb	52.21	0.2	0.06	0.75	309	0.17	5.5
T0912TS171_5-D1.pdb	34.56	0.25	0.15	0.6	247	0.14	5.56
T0912TS173_1-D1.pdb	22.06	0.06	0.26	0.68	281	0.08	63.1
T0912TS173_2-D1.pdb	20.59	0.04	0.25	0.71	294	0.07	61.41
T0912TS173_3-D1.pdb	13.97	0.06	0.22	0.71	296	0.05	59.48
T0912TS173_4-D1.pdb	17.65	0.05	0.23	0.71	295	0.06	61.35
T0912TS173_5-D1.pdb	21.32	0.05	0.29	0.66	274	0.08	61.23
T0912TS180_5-D1.pdb	38.24	0.05	0	0.95	393	0.1	12.86

T0912TS182_1-D1.pdb	17.65	0.07	0.08	0.85	352	0.05	11.47
T0912TS182_2-D1.pdb	22.79	0.08	0.06	0.86	354	0.06	11.05
T0912TS182_3-D1.pdb	22.06	0.06	0.07	0.87	361	0.06	9
T0912TS182_4-D1.pdb	16.91	0.05	0.08	0.86	358	0.05	8.27
T0912TS182_5-D1.pdb	24.26	0.06	0.08	0.86	357	0.07	11.17
T0912TS183_2-D1.pdb	32.35	0.09	0.08	0.83	343	0.09	38.65
T0912TS183_3-D1.pdb	15.44	0.02	0.1	0.87	362	0.04	50.91
T0912TS183_4-D1.pdb	15.44	0.01	0.11	0.88	363	0.04	56.04
T0912TS183_5-D1.pdb	23.53	0.03	0.13	0.84	349	0.07	51.75
T0912TS203_1-D1.pdb	24.26	0.03	0.18	0.79	327	0.07	61.05
T0912TS220_3-D1.pdb	23.53	0.02	0.13	0.85	350	0.07	51.27
T0912TS220_4-D1.pdb	21.32	0.03	0.15	0.82	339	0.06	41.97
T0912TS220_5-D1.pdb	27.21	0.03	0.15	0.82	339	0.08	40.28
T0912TS230_1-D1.pdb	38.24	0.05	0.19	0.77	317	0.12	10.21
T0912TS230_2-D1.pdb	39.71	0.04	0.19	0.77	318	0.12	10.39
T0912TS230_3-D1.pdb	36.76	0.05	0.2	0.75	310	0.12	10.87
T0912TS230_4-D1.pdb	37.5	0.04	0.18	0.78	324	0.12	10.69
T0912TS230_5-D1.pdb	40.44	0.04	0.23	0.73	302	0.13	10.63
T0912TS232_5-D1.pdb	44.85	0.02	0.2	0.78	323	0.14	5.19
T0912TS236_1-D1.pdb	19.85	0.02	0.24	0.75	309	0.06	56.34
T0912TS236_2-D1.pdb	23.53	0.01	0.17	0.82	338	0.07	51.45
T0912TS236_3-D1.pdb	25	0.01	0.22	0.77	317	0.08	51.87
T0912TS236_5-D1.pdb	21.32	0	0.19	0.81	337	0.06	50.73
T0912TS243_1-D1.pdb	25.74	0.12	0.22	0.66	272	0.09	56.4
T0912TS243_2-D1.pdb	24.26	0.12	0.21	0.67	278	0.09	54.29
T0912TS243_3-D1.pdb	25	0.12	0.22	0.66	273	0.09	54.83
T0912TS243_4-D1.pdb	25	0.12	0.22	0.66	273	0.09	54.59
T0912TS243_5-D1.pdb	25.74	0.12	0.22	0.66	273	0.09	54.35
T0912TS247_1-D1.pdb	21.32	0.06	0.28	0.67	276	0.08	59.96
T0912TS247_2-D1.pdb	18.38	0.06	0.28	0.66	274	0.07	58.21
T0912TS247_3-D1.pdb	19.85	0.06	0.29	0.65	270	0.07	59.24
T0912TS247_4-D1.pdb	19.85	0.06	0.29	0.64	267	0.07	59.36
T0912TS247_5-D1.pdb	21.32	0.07	0.28	0.65	271	0.08	59.18
T0912TS250_1-D1.pdb	22.79	0.03	0.19	0.78	321	0.07	36.53
T0912TS250_2-D1.pdb	22.79	0.03	0.18	0.79	327	0.07	36.77
T0912TS250_3-D1.pdb	22.06	0.03	0.2	0.77	320	0.07	36.35
T0912TS250_4-D1.pdb	22.79	0.03	0.22	0.75	311	0.07	37.38
T0912TS250_5-D1.pdb	21.32	0.03	0.2	0.77	319	0.07	36.17
T0912TS251_1-D1.pdb	20.59	0.06	0.21	0.73	303	0.07	39.49
T0912TS251_2-D1.pdb	18.38	0.06	0.22	0.71	296	0.06	38.95
T0912TS251_3-D1.pdb	27.94	0.03	0.2	0.77	318	0.09	38.53
T0912TS251_4-D1.pdb	26.47	0.05	0.18	0.77	320	0.08	40.82
T0912TS251_5-D1.pdb	21.32	0.06	0.15	0.79	326	0.07	40.64
T0912TS252_1-D1.pdb	25.74	0.07	0.24	0.7	288	0.09	60.33
T0912TS252_2-D1.pdb	25	0.05	0.25	0.7	289	0.09	63.83
T0912TS252_3-D1.pdb	22.06	0.06	0.24	0.7	290	0.08	61.84
T0912TS252_4-D1.pdb	25	0.05	0.27	0.68	281	0.09	58.94

T0912TS252_5-D1.pdb	22.06	0.04	0.23	0.73	302	0.07	58.88
T0912TS258_1-D1.pdb	36.03	0.04	0.17	0.79	328	0.11	27.11
T0912TS258_2-D1.pdb	37.5	0.04	0.16	0.79	329	0.11	26.81
T0912TS258_3-D1.pdb	36.76	0.04	0.16	0.79	329	0.11	26.87
T0912TS258_4-D1.pdb	36.03	0.04	0.17	0.79	328	0.11	26.93
T0912TS258_5-D1.pdb	36.03	0.04	0.15	0.82	338	0.11	27.17
T0912TS275_1-D1.pdb	29.41	0.03	0.19	0.78	323	0.09	37.8
T0912TS275_2-D1.pdb	34.56	0.04	0.19	0.77	319	0.11	37.56
T0912TS275_3-D1.pdb	31.62	0.02	0.13	0.86	354	0.09	36.05
T0912TS275_4-D1.pdb	33.82	0.03	0.23	0.74	305	0.11	38.28
T0912TS275_5-D1.pdb	45.59	0.04	0.23	0.73	302	0.15	33.51
T0912TS287_2-D1.pdb	20.59	0.01	0.18	0.8	333	0.06	53.2
T0912TS287_3-D1.pdb	25.74	0.01	0.18	0.81	334	0.08	51.27
T0912TS287_4-D1.pdb	24.26	0.01	0.21	0.77	319	0.08	51.93
T0912TS287_5-D1.pdb	22.79	0	0.21	0.79	329	0.07	50.42
T0912TS300_1-D1.pdb	19.12	0.07	0.12	0.81	335	0.06	19.93
T0912TS300_2-D1.pdb	13.24	0.08	0.08	0.84	346	0.04	10.69
T0912TS300_3-D1.pdb	22.06	0.06	0.1	0.84	349	0.06	12.2
T0912TS300_4-D1.pdb	16.18	0.06	0.13	0.81	337	0.05	9.54
T0912TS300_5-D1.pdb	14.71	0.07	0.06	0.87	361	0.04	10.39
T0912TS303_1-D1.pdb	22.79	0.05	0.27	0.68	283	0.08	64.13
T0912TS303_2-D1.pdb	22.79	0.03	0.2	0.77	319	0.07	61.05
T0912TS303_3-D1.pdb	27.94	0.08	0.25	0.67	277	0.1	60.69
T0912TS303_4-D1.pdb	21.32	0.05	0.23	0.72	298	0.07	62.44
T0912TS303_5-D1.pdb	25	0.04	0.31	0.64	266	0.09	59.96
T0912TS313_1-D1.pdb	19.12	0.03	0.14	0.82	340	0.06	55.19
T0912TS313_2-D1.pdb	17.65	0.03	0.16	0.8	332	0.05	55.31
T0912TS313_3-D1.pdb	19.12	0.03	0.15	0.81	336	0.06	55.19
T0912TS313_4-D1.pdb	16.18	0.03	0.14	0.82	340	0.05	55.37
T0912TS313_5-D1.pdb	18.38	0.03	0.14	0.83	344	0.05	54.65
T0912TS320_4-D1.pdb	18.38	0.03	0.09	0.88	364	0.05	57.97
T0912TS320_5-D1.pdb	19.12	0.03	0.09	0.88	365	0.05	53.14
T0912TS321_1-D1.pdb	50.74	0.05	0.12	0.82	341	0.15	5.13
T0912TS321_2-D1.pdb	50.74	0.05	0.12	0.83	342	0.15	5.25
T0912TS321_3-D1.pdb	55.15	0.06	0.11	0.83	344	0.16	5.37
T0912TS321_4-D1.pdb	50.74	0.06	0.12	0.82	341	0.15	5.19
T0912TS321_5-D1.pdb	55.15	0.06	0.11	0.83	343	0.16	5.43
T0912TS324_1-D1.pdb	27.94	0.09	0.22	0.69	285	0.1	53.5
T0912TS324_2-D1.pdb	25.74	0.13	0.22	0.65	270	0.1	52.78
T0912TS324_3-D1.pdb	17.65	0.04	0.2	0.76	314	0.06	55.86
T0912TS324_4-D1.pdb	25	0.04	0.2	0.76	316	0.08	55.98
T0912TS324_5-D1.pdb	25.74	0.13	0.21	0.67	276	0.09	53.02
T0912TS325_1-D1.pdb	23.53	0.08	0.27	0.65	270	0.09	58.27
T0912TS325_2-D1.pdb	25.74	0.07	0.22	0.71	294	0.09	59.78
T0912TS325_3-D1.pdb	29.41	0.04	0.21	0.75	312	0.09	56.34
T0912TS325_4-D1.pdb	22.06	0.06	0.25	0.69	284	0.08	59.36
T0912TS325_5-D1.pdb	19.85	0.06	0.23	0.71	293	0.07	61.84

T0912TS345_1-D1.pdb	26.47	0.06	0.17	0.77	319	0.08	41.12
T0912TS345_2-D1.pdb	21.32	0.01	0.26	0.73	301	0.07	51.69
T0912TS345_3-D1.pdb	23.53	0.06	0.16	0.78	324	0.07	40.94
T0912TS345_4-D1.pdb	25	0.04	0.2	0.77	318	0.08	38.28
T0912TS345_5-D1.pdb	31.62	0.06	0.24	0.71	293	0.11	36.59
T0912TS349_1-D1.pdb	17.65	0.03	0.11	0.86	354	0.05	57.97
T0912TS357_1-D1.pdb	22.06	0.01	0	0.99	408	0.05	53.44
T0912TS357_2-D1.pdb	24.26	0.01	0.01	0.98	404	0.06	53.8
T0912TS357_3-D1.pdb	22.06	0.02	0	0.98	405	0.05	52.96
T0912TS357_4-D1.pdb	22.79	0.01	0	0.99	410	0.06	52.9
T0912TS357_5-D1.pdb	27.94	0.01	0	0.99	410	0.07	52.78
T0912TS359_3-D1.pdb	28.68	0.02	0.09	0.89	369	0.08	16.18
T0912TS359_5-D1.pdb	27.94	0.01	0.06	0.93	385	0.07	20.11
T0912TS363_5-D1.pdb	28.68	0.02	0.2	0.78	323	0.09	34.72
T0912TS382_1-D1.pdb	21.32	0	0.14	0.86	358	0.06	36.59
T0912TS382_2-D1.pdb	18.38	0	0.16	0.84	348	0.05	35.33
T0912TS382_3-D1.pdb	17.65	0	0.13	0.87	359	0.05	35.45
T0912TS382_4-D1.pdb	23.53	0	0.13	0.87	362	0.06	38.53
T0912TS382_5-D1.pdb	25	0	0.15	0.85	353	0.07	36.29
T0912TS384_1-D1.pdb	26.47	0.12	0.21	0.67	276	0.1	53.68
T0912TS384_2-D1.pdb	24.26	0.05	0.22	0.73	303	0.08	65.28
T0912TS384_3-D1.pdb	26.47	0.08	0.23	0.69	286	0.09	61.41
T0912TS384_4-D1.pdb	21.32	0.06	0.27	0.67	277	0.08	60.87
T0912TS384_5-D1.pdb	19.85	0.05	0.23	0.72	300	0.07	62.98
T0912TS393_1-D1.pdb	23.53	0.05	0.26	0.69	286	0.08	63.89
T0912TS393_2-D1.pdb	21.32	0.03	0.21	0.76	313	0.07	60.51
T0912TS393_3-D1.pdb	27.94	0.08	0.22	0.69	287	0.1	59.78
T0912TS393_4-D1.pdb	19.12	0.05	0.23	0.72	297	0.06	62.02
T0912TS393_5-D1.pdb	23.53	0.04	0.28	0.68	282	0.08	59.9
T0912TS396_1-D1.pdb	16.91	0.04	0.12	0.84	348	0.05	61.05
T0912TS396_2-D1.pdb	21.32	0.05	0.16	0.79	327	0.07	54.11
T0912TS396_3-D1.pdb	25	0.08	0.13	0.79	328	0.08	38.28
T0912TS396_4-D1.pdb	31.62	0.09	0.09	0.81	337	0.09	38.65
T0912TS396_5-D1.pdb	18.38	0.03	0.06	0.91	375	0.05	53.44
T0912TS399_1-D1.pdb	47.79	0.03	0	0.97	400	0.12	5.13
T0912TS399_2-D1.pdb	47.79	0.03	0	0.97	402	0.12	4.95
T0912TS399_3-D1.pdb	54.41	0.03	0	0.97	400	0.14	5.01
T0912TS399_4-D1.pdb	52.94	0.03	0	0.97	403	0.13	5.01
T0912TS399_5-D1.pdb	51.47	0.01	0	0.99	409	0.13	4.83
T0912TS405_1-D1.pdb	22.06	0.06	0.18	0.76	315	0.07	41.18
T0912TS405_2-D1.pdb	19.85	0.05	0.2	0.75	312	0.06	41.18
T0912TS405_3-D1.pdb	22.06	0.06	0.19	0.75	312	0.07	42.33
T0912TS407_1-D1.pdb	20.59	0.04	0.1	0.86	355	0.06	30.86
T0912TS407_2-D1.pdb	19.12	0.04	0.06	0.91	375	0.05	32.31
T0912TS407_3-D1.pdb	25	0.05	0.16	0.8	330	0.08	27.9
T0912TS407_4-D1.pdb	18.38	0.03	0.09	0.87	362	0.05	27.05
T0912TS407_5-D1.pdb	19.85	0.05	0.05	0.9	374	0.05	27.11

T0912TS411_1-D1.pdb	23.53	0.03	0.19	0.78	322	0.07	60.51
T0912TS411_2-D1.pdb	20.59	0.05	0.17	0.79	325	0.06	46.01
T0912TS411_3-D1.pdb	19.85	0.04	0.13	0.83	343	0.06	52.41
T0912TS411_4-D1.pdb	19.85	0.03	0.17	0.8	330	0.06	55.68
T0912TS411_5-D1.pdb	22.06	0.01	0.11	0.87	361	0.06	49.82
T0912TS420_3-D1.pdb	36.03	0	0.24	0.76	314	0.11	6.28
T0912TS420_4-D1.pdb	32.35	0.03	0.24	0.74	305	0.11	23.07
T0912TS420_5-D1.pdb	33.82	0.03	0.18	0.79	327	0.1	17.33
T0912TS425_1-D1.pdb	37.5	0.01	0.17	0.81	336	0.11	41.06
T0912TS425_2-D1.pdb	36.76	0.01	0.16	0.82	341	0.11	43.42
T0912TS425_3-D1.pdb	38.24	0.01	0.15	0.83	345	0.11	42.94
T0912TS425_4-D1.pdb	37.5	0.01	0.17	0.81	337	0.11	42.33
T0912TS425_5-D1.pdb	40.44	0.01	0.14	0.84	348	0.12	42.15
T0912TS432_1-D1.pdb	33.82	0.01	0.01	0.98	405	0.08	13.47
T0912TS432_2-D1.pdb	30.15	0.01	0.02	0.97	402	0.07	8.88
T0912TS432_3-D1.pdb	32.35	0	0	1	412	0.08	14.67
T0912TS432_4-D1.pdb	29.41	0	0.03	0.97	403	0.07	8.63
T0912TS432_5-D1.pdb	36.03	0.02	0.02	0.96	398	0.09	8.82
T0912TS434_1-D1.pdb	69.85	0.06	0	0.94	388	0.18	6.1
T0912TS434_3-D1.pdb	55.88	0.07	0	0.93	385	0.15	6.34
T0912TS434_4-D1.pdb	60.29	0.07	0	0.92	382	0.16	6.76
T0912TS434_5-D1.pdb	63.24	0.08	0	0.92	380	0.17	5.8
T0912TS439_1-D1.pdb	27.21	0.12	0.22	0.66	273	0.1	53.74
T0912TS439_3-D1.pdb	20.59	0.03	0.17	0.79	329	0.06	55.56
T0912TS439_4-D1.pdb	22.06	0.03	0.17	0.8	330	0.07	61.23
T0912TS439_5-D1.pdb	23.53	0.03	0.18	0.79	326	0.07	36.53
T0912TS441_1-D1.pdb	17.65	0.04	0.23	0.73	302	0.06	57.31
T0912TS441_2-D1.pdb	21.32	0.06	0.24	0.7	291	0.07	61.84
T0912TS441_5-D1.pdb	19.12	0.06	0.23	0.71	293	0.07	61.72
T0912TS444_1-D1.pdb	25.74	0.1	0.16	0.74	306	0.08	40.16
T0912TS444_3-D1.pdb	27.94	0.04	0.21	0.76	314	0.09	34.78
T0912TS446_1-D1.pdb	27.21	0.07	0.21	0.72	297	0.09	30.86
T0912TS450_1-D1.pdb	25	0.06	0.24	0.7	290	0.09	54.53
T0912TS450_2-D1.pdb	21.32	0.05	0.18	0.76	316	0.07	49.94
T0912TS450_3-D1.pdb	22.79	0.05	0.19	0.76	315	0.07	51.33
T0912TS450_4-D1.pdb	26.47	0.06	0.17	0.77	320	0.08	48.49
T0912TS450_5-D1.pdb	23.53	0.06	0.2	0.74	307	0.08	55.01
T0912TS452_1-D1.pdb	13.24	0.03	0.21	0.76	316	0.04	53.8
T0912TS452_2-D1.pdb	27.94	0.03	0.25	0.73	301	0.09	19.14
T0912TS452_3-D1.pdb	36.03	0.04	0.22	0.74	307	0.12	33.7
T0912TS452_4-D1.pdb	33.09	0.02	0.15	0.82	341	0.1	45.83
T0912TS455_1-D1.pdb	39.71	0.01	0.04	0.95	394	0.1	6.22
T0912TS455_2-D1.pdb	33.09	0	0.03	0.97	402	0.08	5.5
T0912TS455_3-D1.pdb	45.59	0	0.03	0.97	400	0.11	4.95
T0912TS455_4-D1.pdb	29.41	0	0.03	0.97	400	0.07	5.92
T0912TS455_5-D1.pdb	35.29	0	0.1	0.9	372	0.09	5.92
T0912TS456_1-D1.pdb	25	0.06	0.28	0.67	276	0.09	61.77

T0912TS456_2-D1.pdb	25	0.06	0.23	0.71	292	0.09	57.19
T0912TS456_3-D1.pdb	25.74	0.05	0.23	0.72	297	0.09	62.08
T0912TS456_4-D1.pdb	26.47	0.05	0.26	0.7	288	0.09	60.87
T0912TS456_5-D1.pdb	28.68	0.05	0.24	0.71	292	0.1	62.02
T0912TS464_1-D1.pdb	27.21	0.03	0.19	0.79	325	0.08	42.51
T0912TS464_3-D1.pdb	27.94	0.03	0.19	0.78	324	0.09	42.39
T0912TS464_4-D1.pdb	25	0.03	0.19	0.78	323	0.08	42.45
T0912TS464_5-D1.pdb	73.53	0.05	0	0.95	393	0.19	4.65
T0912TS467_1-D1.pdb	36.76	0.04	0.19	0.77	317	0.12	11.78
T0912TS467_4-D1.pdb	38.24	0.04	0.18	0.78	323	0.12	14.25
T0912TS475_1-D1.pdb	25	0.05	0.23	0.72	298	0.08	56.22
T0912TS475_2-D1.pdb	29.41	0.04	0.22	0.74	306	0.1	55.37
T0912TS475_3-D1.pdb	24.26	0.05	0.24	0.71	295	0.08	58.94
T0912TS475_4-D1.pdb	25.74	0.06	0.24	0.7	288	0.09	58.58
T0912TS475_5-D1.pdb	24.26	0.05	0.23	0.72	299	0.08	56.16
T0912TS479_2-D1.pdb	24.26	0.08	0.14	0.78	321	0.08	38.65
T0912TS479_3-D1.pdb	14.71	0.02	0.12	0.86	354	0.04	51.63
T0912TS480_1-D1.pdb	33.09	0.01	0.23	0.75	311	0.11	27.23
T0912TS480_2-D1.pdb	21.32	0.01	0.18	0.8	332	0.06	49.22
T0912TS480_3-D1.pdb	37.5	0.04	0.18	0.79	325	0.12	33.76
T0912TS480_4-D1.pdb	20.59	0.04	0.21	0.75	309	0.07	58.03
T0912TS480_5-D1.pdb	31.62	0.02	0.29	0.69	285	0.11	26.03
T0912TS483_1-D1.pdb	38.24	0.05	0.23	0.72	298	0.13	19.38
T0912TS483_2-D1.pdb	33.09	0.03	0.21	0.76	314	0.11	33.63
T0912TS483_3-D1.pdb	36.76	0	0.23	0.76	316	0.12	6.1
T0912TS483_4-D1.pdb	35.29	0.03	0.31	0.66	273	0.13	6.28
T0912TS483_5-D1.pdb	40.44	0.17	0.18	0.65	271	0.15	4.35
T0912TS486_2-D1.pdb	28.68	0	0.1	0.89	370	0.08	28.44
T0912TS486_3-D1.pdb	21.32	0.02	0.04	0.93	386	0.06	47.22
T0912TS486_4-D1.pdb	22.79	0.03	0.06	0.91	375	0.06	45.89
T0912TS498_2-D1.pdb	26.47	0.02	0.19	0.79	327	0.08	34.36
T0912TS498_3-D1.pdb	19.12	0.03	0.17	0.79	329	0.06	55.25
T0912TS498_5-D1.pdb	28.68	0.12	0.21	0.67	278	0.1	52.9
T0913TS001_3-D1.pdb	18.02	0.25	0.11	0.64	215	0.08	61.24
T0913TS001_4-D1.pdb	18.92	0.3	0.16	0.53	180	0.11	65.75
T0913TS001_5-D1.pdb	14.41	0.34	0.16	0.5	169	0.09	66.79
T0913TS004_1-D1.pdb	18.02	0.23	0.14	0.63	212	0.08	61.83
T0913TS004_2-D1.pdb	21.62	0.23	0.15	0.62	211	0.1	58.65
T0913TS004_3-D1.pdb	18.92	0.27	0.15	0.58	197	0.1	61.54
T0913TS004_4-D1.pdb	19.82	0.25	0.14	0.61	206	0.1	58.21
T0913TS004_5-D1.pdb	16.22	0.28	0.14	0.58	196	0.08	62.35
T0913TS005_1-D1.pdb	19.82	0.31	0.16	0.53	179	0.11	66.2
T0913TS005_2-D1.pdb	14.41	0.35	0.16	0.49	165	0.09	66.57
T0913TS005_3-D1.pdb	19.82	0.29	0.15	0.55	187	0.11	63.61
T0913TS005_4-D1.pdb	21.62	0.31	0.16	0.52	177	0.12	62.2
T0913TS005_5-D1.pdb	19.82	0.29	0.17	0.54	182	0.11	62.43
T0913TS011_1-D1.pdb	19.82	0.32	0.15	0.53	180	0.11	65.83

T0913TS011_2-D1.pdb	23.42	0.33	0.14	0.54	181	0.13	66.35
T0913TS011_3-D1.pdb	20.72	0.33	0.15	0.52	176	0.12	66.86
T0913TS011_4-D1.pdb	19.82	0.33	0.14	0.53	179	0.11	66.35
T0913TS011_5-D1.pdb	18.92	0.33	0.16	0.51	171	0.11	66.72
T0913TS016_1-D1.pdb	23.42	0.2	0.17	0.63	214	0.11	54.44
T0913TS017_1-D1.pdb	17.12	0.28	0.13	0.59	201	0.09	62.06
T0913TS017_2-D1.pdb	17.12	0.22	0.13	0.64	218	0.08	61.91
T0913TS017_3-D1.pdb	17.12	0.22	0.13	0.65	219	0.08	61.91
T0913TS017_5-D1.pdb	17.12	0.28	0.13	0.6	202	0.08	61.98
T0913TS019_1-D1.pdb	21.62	0.25	0.16	0.59	199	0.11	57.03
T0913TS022_1-D1.pdb	41.44	0.3	0	0.7	235	0.18	5.92
T0913TS022_2-D1.pdb	34.23	0.34	0	0.66	223	0.15	7.69
T0913TS022_3-D1.pdb	35.14	0.34	0	0.66	223	0.16	7.69
T0913TS022_4-D1.pdb	41.44	0.36	0	0.64	215	0.19	8.43
T0913TS022_5-D1.pdb	36.94	0.31	0	0.69	234	0.16	7.03
T0913TS026_1-D1.pdb	18.02	0.25	0.13	0.62	210	0.09	55.62
T0913TS026_3-D1.pdb	26.13	0.2	0.13	0.67	228	0.11	52.81
T0913TS026_4-D1.pdb	23.42	0.22	0.14	0.64	217	0.11	53.18
T0913TS026_5-D1.pdb	21.62	0.2	0.13	0.66	224	0.1	53.7
T0913TS040_1-D1.pdb	34.23	0.24	0.07	0.69	233	0.15	7.84
T0913TS040_2-D1.pdb	30.63	0.23	0.07	0.7	236	0.13	7.62
T0913TS040_3-D1.pdb	32.43	0.24	0.07	0.7	235	0.14	7.91
T0913TS040_4-D1.pdb	29.73	0.26	0.04	0.71	239	0.12	9.1
T0913TS040_5-D1.pdb	25.23	0.27	0.09	0.64	217	0.12	8.36
T0913TS042_1-D1.pdb	18.92	0.31	0.17	0.53	178	0.11	65.9
T0913TS042_2-D1.pdb	20.72	0.29	0.17	0.54	183	0.11	62.43
T0913TS042_3-D1.pdb	20.72	0.23	0.17	0.59	201	0.1	58.21
T0913TS042_4-D1.pdb	16.22	0.25	0.13	0.62	210	0.08	62.06
T0913TS042_5-D1.pdb	20.72	0.25	0.14	0.61	206	0.1	63.39
T0913TS048_1-D1.pdb	22.52	0.27	0.14	0.6	202	0.11	63.02
T0913TS060_1-D1.pdb	18.92	0.26	0.14	0.6	203	0.09	61.76
T0913TS060_2-D1.pdb	19.82	0.29	0.15	0.56	188	0.11	63.09
T0913TS060_3-D1.pdb	14.41	0.21	0.11	0.67	228	0.06	61.76
T0913TS060_4-D1.pdb	17.12	0.15	0.11	0.74	251	0.07	61.76
T0913TS060_5-D1.pdb	18.92	0.25	0.12	0.63	212	0.09	62.65
T0913TS064_1-D1.pdb	18.02	0.37	0.16	0.47	160	0.11	66.12
T0913TS066_1-D1.pdb	24.32	0.21	0.15	0.63	214	0.11	57.91
T0913TS066_2-D1.pdb	26.13	0.25	0.14	0.6	203	0.13	53.11
T0913TS066_3-D1.pdb	19.82	0.2	0.12	0.68	230	0.09	53.99
T0913TS066_4-D1.pdb	23.42	0.21	0.14	0.64	217	0.11	50.15
T0913TS066_5-D1.pdb	26.13	0.15	0.13	0.72	242	0.11	49.33
T0913TS067_2-D1.pdb	19.82	0.3	0.16	0.54	181	0.11	61.98
T0913TS067_3-D1.pdb	20.72	0.32	0.16	0.53	178	0.12	60.95
T0913TS067_4-D1.pdb	15.32	0.25	0.13	0.62	208	0.07	61.17
T0913TS067_5-D1.pdb	21.62	0.27	0.13	0.6	202	0.11	58.73
T0913TS073_1-D1.pdb	18.92	0.25	0.15	0.59	200	0.09	59.17
T0913TS073_2-D1.pdb	20.72	0.23	0.14	0.63	212	0.1	62.13

T0913TS073_3-D1.pdb	22.52	0.28	0.13	0.58	197	0.11	57.62
T0913TS073_4-D1.pdb	19.82	0.24	0.15	0.61	206	0.1	59.25
T0913TS073_5-D1.pdb	19.82	0.26	0.12	0.63	212	0.09	57.47
T0913TS077_1-D1.pdb	19.82	0.22	0.17	0.61	206	0.1	59.17
T0913TS077_2-D1.pdb	20.72	0.23	0.17	0.6	204	0.1	58.95
T0913TS077_3-D1.pdb	19.82	0.22	0.16	0.62	208	0.1	58.58
T0913TS077_4-D1.pdb	22.52	0.23	0.17	0.6	204	0.11	59.17
T0913TS077_5-D1.pdb	20.72	0.21	0.16	0.63	213	0.1	58.28
T0913TS092_1-D1.pdb	19.82	0.23	0.14	0.62	211	0.09	60.8
T0913TS101_1-D1.pdb	27.93	0.18	0.1	0.72	243	0.11	49.78
T0913TS101_2-D1.pdb	27.93	0.18	0.09	0.73	248	0.11	49.19
T0913TS101_3-D1.pdb	26.13	0.21	0.14	0.65	219	0.12	49.04
T0913TS101_4-D1.pdb	23.42	0.22	0.07	0.71	241	0.1	47.26
T0913TS101_5-D1.pdb	22.52	0.2	0.09	0.7	238	0.09	48.15
T0913TS102_5-D1.pdb	19.82	0.27	0.17	0.57	191	0.1	57.99
T0913TS114_2-D1.pdb	18.02	0.25	0.15	0.6	204	0.09	56.66
T0913TS114_4-D1.pdb	18.02	0.24	0.14	0.61	207	0.09	56.51
T0913TS119_1-D1.pdb	19.82	0.2	0.15	0.65	221	0.09	59.91
T0913TS126_1-D1.pdb	35.14	0.36	0.11	0.53	180	0.2	13.09
T0913TS126_2-D1.pdb	35.14	0.29	0.1	0.61	205	0.17	15.9
T0913TS126_3-D1.pdb	27.93	0.33	0.12	0.55	187	0.15	17.23
T0913TS126_4-D1.pdb	32.43	0.32	0.09	0.59	199	0.16	13.61
T0913TS126_5-D1.pdb	36.94	0.33	0.12	0.55	186	0.2	18.64
T0913TS162_1-D1.pdb	14.41	0.21	0.06	0.73	246	0.06	9.98
T0913TS162_2-D1.pdb	15.32	0.18	0.04	0.77	261	0.06	7.69
T0913TS162_3-D1.pdb	18.92	0.19	0.06	0.75	255	0.07	10.36
T0913TS162_4-D1.pdb	11.71	0.18	0.03	0.79	267	0.04	8.21
T0913TS162_5-D1.pdb	13.51	0.18	0.08	0.75	252	0.05	9.17
T0913TS171_1-D1.pdb	27.93	0.3	0.14	0.55	187	0.15	34.62
T0913TS171_2-D1.pdb	27.03	0.31	0.14	0.55	186	0.15	34.39
T0913TS171_3-D1.pdb	29.73	0.3	0.14	0.56	188	0.16	34.54
T0913TS171_4-D1.pdb	28.83	0.31	0.14	0.55	187	0.15	34.25
T0913TS171_5-D1.pdb	27.93	0.29	0.13	0.57	194	0.14	33.58
T0913TS173_1-D1.pdb	18.02	0.35	0.17	0.49	164	0.11	65.24
T0913TS173_2-D1.pdb	15.32	0.32	0.14	0.54	184	0.08	61.32
T0913TS173_3-D1.pdb	13.51	0.33	0.16	0.5	170	0.08	62.94
T0913TS173_4-D1.pdb	18.92	0.3	0.17	0.54	182	0.1	62.5
T0913TS173_5-D1.pdb	13.51	0.3	0.17	0.53	179	0.08	64.05
T0913TS179_1-D1.pdb	13.51	0.37	0.18	0.45	153	0.09	65.46
T0913TS179_2-D1.pdb	16.22	0.32	0.18	0.5	170	0.1	63.31
T0913TS179_3-D1.pdb	18.92	0.28	0.15	0.57	192	0.1	59.98
T0913TS179_4-D1.pdb	20.72	0.32	0.17	0.52	175	0.12	62.94
T0913TS179_5-D1.pdb	18.92	0.29	0.15	0.55	187	0.1	60.43
T0913TS180_1-D1.pdb	26.13	0.33	0.12	0.56	188	0.14	36.24
T0913TS180_3-D1.pdb	28.83	0.32	0.1	0.57	194	0.15	33.88
T0913TS180_4-D1.pdb	26.13	0.2	0.11	0.69	234	0.11	34.25
T0913TS180_5-D1.pdb	18.92	0.21	0.01	0.79	266	0.07	13.46

T0913TS182_1-D1.pdb	15.32	0.21	0.09	0.7	236	0.06	19.82
T0913TS182_2-D1.pdb	14.41	0.22	0.08	0.7	237	0.06	9.25
T0913TS182_3-D1.pdb	18.02	0.21	0.05	0.74	250	0.07	11.46
T0913TS182_4-D1.pdb	16.22	0.25	0.08	0.67	226	0.07	10.43
T0913TS182_5-D1.pdb	17.12	0.22	0.01	0.78	262	0.07	11.61
T0913TS183_1-D1.pdb	18.92	0.25	0.12	0.63	213	0.09	60.87
T0913TS183_2-D1.pdb	18.92	0.25	0.14	0.61	206	0.09	60.87
T0913TS183_3-D1.pdb	19.82	0.2	0.12	0.67	227	0.09	58.8
T0913TS183_4-D1.pdb	20.72	0.23	0.12	0.65	219	0.09	61.09
T0913TS203_1-D1.pdb	19.82	0.3	0.16	0.54	182	0.11	60.43
T0913TS203_2-D1.pdb	18.92	0.32	0.16	0.52	176	0.11	61.24
T0913TS203_4-D1.pdb	17.12	0.26	0.13	0.61	207	0.08	61.02
T0913TS207_3-D1.pdb	41.44	0.26	0.07	0.67	227	0.18	18.05
T0913TS236_1-D1.pdb	24.32	0.24	0.12	0.64	216	0.11	55.7
T0913TS236_2-D1.pdb	21.62	0.21	0.14	0.66	222	0.1	55.7
T0913TS236_3-D1.pdb	20.72	0.22	0.17	0.61	207	0.1	57.54
T0913TS236_4-D1.pdb	20.72	0.21	0.16	0.63	212	0.1	57.54
T0913TS236_5-D1.pdb	21.62	0.2	0.15	0.65	220	0.1	56.21
T0913TS239_1-D1.pdb	20.72	0.27	0.14	0.58	197	0.11	60.43
T0913TS239_2-D1.pdb	16.22	0.28	0.14	0.58	196	0.08	61.02
T0913TS239_3-D1.pdb	19.82	0.27	0.16	0.57	194	0.1	57.4
T0913TS239_4-D1.pdb	18.02	0.28	0.15	0.57	191	0.09	61.02
T0913TS239_5-D1.pdb	18.92	0.28	0.15	0.57	193	0.1	60.5
T0913TS243_1-D1.pdb	16.22	0.28	0.15	0.57	192	0.08	61.24
T0913TS243_2-D1.pdb	15.32	0.28	0.14	0.57	193	0.08	61.32
T0913TS243_3-D1.pdb	15.32	0.29	0.14	0.57	191	0.08	61.98
T0913TS243_4-D1.pdb	17.12	0.28	0.15	0.57	192	0.09	61.24
T0913TS243_5-D1.pdb	17.12	0.28	0.14	0.58	197	0.09	61.76
T0913TS247_1-D1.pdb	20.72	0.28	0.17	0.54	183	0.11	65.98
T0913TS247_2-D1.pdb	16.22	0.31	0.18	0.51	173	0.09	61.09
T0913TS247_4-D1.pdb	15.32	0.31	0.17	0.52	177	0.09	66.57
T0913TS247_5-D1.pdb	20.72	0.27	0.17	0.56	190	0.11	60.21
T0913TS250_1-D1.pdb	17.12	0.23	0.15	0.62	210	0.08	56.88
T0913TS250_3-D1.pdb	17.12	0.23	0.15	0.62	209	0.08	57.17
T0913TS250_5-D1.pdb	18.92	0.22	0.15	0.62	211	0.09	56.88
T0913TS251_1-D1.pdb	23.42	0.23	0.15	0.62	209	0.11	54.96
T0913TS251_2-D1.pdb	18.02	0.24	0.2	0.56	189	0.1	54.73
T0913TS251_3-D1.pdb	21.62	0.24	0.17	0.59	200	0.11	54.66
T0913TS251_4-D1.pdb	20.72	0.22	0.17	0.61	207	0.1	54.36
T0913TS251_5-D1.pdb	22.52	0.23	0.17	0.59	201	0.11	55.1
T0913TS252_1-D1.pdb	21.62	0.31	0.17	0.51	174	0.12	65.09
T0913TS252_2-D1.pdb	19.82	0.31	0.17	0.52	177	0.11	62.65
T0913TS252_3-D1.pdb	22.52	0.32	0.16	0.52	176	0.13	60.43
T0913TS252_4-D1.pdb	19.82	0.35	0.17	0.49	164	0.12	63.61
T0913TS264_1-D1.pdb	51.35	0.22	0.05	0.73	247	0.21	12.5
T0913TS264_2-D1.pdb	51.35	0.21	0.04	0.75	254	0.2	11.91
T0913TS264_3-D1.pdb	61.26	0.24	0.01	0.75	253	0.24	8.73

T0913TS264_4-D1.pdb	60.36	0.2	0.05	0.75	255	0.24	11.32
T0913TS264_5-D1.pdb	51.35	0.22	0.02	0.76	258	0.2	8.58
T0913TS275_1-D1.pdb	34.23	0.25	0.12	0.63	214	0.16	38.91
T0913TS275_2-D1.pdb	36.94	0.24	0.12	0.64	217	0.17	39.13
T0913TS275_3-D1.pdb	34.23	0.24	0.11	0.65	220	0.16	39.2
T0913TS275_4-D1.pdb	35.14	0.25	0.13	0.62	209	0.17	38.91
T0913TS275_5-D1.pdb	36.04	0.25	0.13	0.62	209	0.17	38.91
T0913TS287_1-D1.pdb	18.92	0.22	0.12	0.66	224	0.08	56.29
T0913TS287_2-D1.pdb	20.72	0.22	0.13	0.65	220	0.09	57.32
T0913TS287_3-D1.pdb	19.82	0.23	0.17	0.61	205	0.1	57.32
T0913TS287_4-D1.pdb	22.52	0.21	0.14	0.64	218	0.1	55.84
T0913TS287_5-D1.pdb	21.62	0.24	0.15	0.61	205	0.11	55.77
T0913TS300_1-D1.pdb	14.41	0.14	0.07	0.78	264	0.05	17.01
T0913TS300_2-D1.pdb	19.82	0.17	0.06	0.77	261	0.08	14.5
T0913TS300_3-D1.pdb	27.03	0.15	0.07	0.78	263	0.1	11.69
T0913TS300_4-D1.pdb	17.12	0.19	0.02	0.79	268	0.06	12.13
T0913TS300_5-D1.pdb	16.22	0.13	0.05	0.82	276	0.06	10.43
T0913TS303_1-D1.pdb	18.02	0.21	0.15	0.64	215	0.08	58.51
T0913TS303_3-D1.pdb	19.82	0.22	0.15	0.62	211	0.09	59.32
T0913TS303_5-D1.pdb	15.32	0.32	0.17	0.5	170	0.09	61.09
T0913TS313_1-D1.pdb	20.72	0.2	0.14	0.66	223	0.09	60.65
T0913TS313_2-D1.pdb	19.82	0.2	0.15	0.66	222	0.09	60.06
T0913TS313_3-D1.pdb	20.72	0.21	0.15	0.64	218	0.1	60.21
T0913TS313_4-D1.pdb	20.72	0.2	0.15	0.65	220	0.09	60.21
T0913TS313_5-D1.pdb	21.62	0.21	0.15	0.64	218	0.1	60.65
T0913TS321_1-D1.pdb	47.75	0.23	0.08	0.7	235	0.2	9.47
T0913TS321_2-D1.pdb	52.25	0.23	0.07	0.69	234	0.22	8.8
T0913TS321_3-D1.pdb	54.05	0.21	0.07	0.71	241	0.22	9.02
T0913TS321_4-D1.pdb	49.55	0.21	0.07	0.71	241	0.21	9.69
T0913TS321_5-D1.pdb	55.86	0.21	0.07	0.71	241	0.23	9.47
T0913TS324_1-D1.pdb	20.72	0.3	0.17	0.53	179	0.12	65.9
T0913TS324_2-D1.pdb	19.82	0.3	0.17	0.54	182	0.11	64.28
T0913TS324_3-D1.pdb	20.72	0.27	0.16	0.57	191	0.11	57.91
T0913TS324_4-D1.pdb	18.02	0.27	0.17	0.57	192	0.09	58.65
T0913TS324_5-D1.pdb	18.02	0.3	0.16	0.54	184	0.1	59.25
T0913TS325_1-D1.pdb	22.52	0.33	0.17	0.5	169	0.13	67.23
T0913TS325_2-D1.pdb	18.92	0.34	0.17	0.49	165	0.11	64.28
T0913TS325_3-D1.pdb	18.92	0.3	0.15	0.55	186	0.1	63.46
T0913TS325_4-D1.pdb	21.62	0.32	0.15	0.54	181	0.12	59.54
T0913TS325_5-D1.pdb	23.42	0.31	0.15	0.54	182	0.13	59.47
T0913TS345_1-D1.pdb	20.72	0.23	0.17	0.6	204	0.1	56.95
T0913TS345_2-D1.pdb	21.62	0.22	0.17	0.6	204	0.11	58.14
T0913TS345_3-D1.pdb	20.72	0.22	0.17	0.61	205	0.1	57.32
T0913TS345_4-D1.pdb	19.82	0.22	0.17	0.61	207	0.1	57.91
T0913TS345_5-D1.pdb	25.23	0.22	0.17	0.62	208	0.12	57.03
T0913TS356_1-D1.pdb	33.33	0.3	0.13	0.57	194	0.17	13.54
T0913TS356_2-D1.pdb	34.23	0.3	0.15	0.55	185	0.19	13.61

T0913TS356_3-D1.pdb	31.53	0.29	0.09	0.62	209	0.15	17.97
T0913TS356_4-D1.pdb	32.43	0.3	0.09	0.61	207	0.16	18.2
T0913TS356_5-D1.pdb	36.04	0.3	0.1	0.6	202	0.18	18.34
T0913TS357_1-D1.pdb	22.52	0.15	0.04	0.81	273	0.08	54.22
T0913TS357_2-D1.pdb	21.62	0.14	0.01	0.86	290	0.07	53.99
T0913TS357_3-D1.pdb	24.32	0.03	0.02	0.95	322	0.08	54.07
T0913TS357_4-D1.pdb	21.62	0.13	0.04	0.83	280	0.08	53.85
T0913TS357_5-D1.pdb	20.72	0.14	0.03	0.83	281	0.07	53.77
T0913TS363_5-D1.pdb	19.82	0.26	0.13	0.61	205	0.1	60.87
T0913TS367_1-D1.pdb	18.02	0.19	0.12	0.69	234	0.08	52.07
T0913TS367_2-D1.pdb	38.74	0.36	0.07	0.58	195	0.2	12.2
T0913TS382_1-D1.pdb	33.33	0.17	0.11	0.71	241	0.14	33.58
T0913TS382_2-D1.pdb	27.93	0.17	0.18	0.66	222	0.13	36.09
T0913TS382_3-D1.pdb	27.03	0.14	0.15	0.71	241	0.11	37.13
T0913TS382_4-D1.pdb	25.23	0.15	0.16	0.7	235	0.11	38.39
T0913TS382_5-D1.pdb	24.32	0.14	0.15	0.71	239	0.1	37.65
T0913TS384_1-D1.pdb	18.92	0.31	0.16	0.53	179	0.11	61.02
T0913TS384_2-D1.pdb	18.02	0.31	0.17	0.52	175	0.1	60.5
T0913TS384_3-D1.pdb	14.41	0.33	0.17	0.51	172	0.08	62.2
T0913TS384_4-D1.pdb	18.02	0.32	0.16	0.52	175	0.1	61.91
T0913TS384_5-D1.pdb	17.12	0.36	0.17	0.48	162	0.11	67.38
T0913TS393_1-D1.pdb	17.12	0.22	0.16	0.62	210	0.08	57.47
T0913TS393_2-D1.pdb	15.32	0.36	0.15	0.48	163	0.09	66.49
T0913TS393_3-D1.pdb	20.72	0.24	0.14	0.62	208	0.1	57.91
T0913TS393_4-D1.pdb	19.82	0.33	0.15	0.51	173	0.11	59.84
T0913TS393_5-D1.pdb	16.22	0.33	0.17	0.5	169	0.1	60.87
T0913TS396_1-D1.pdb	18.02	0.24	0.12	0.64	216	0.08	60.8
T0913TS396_2-D1.pdb	19.82	0.25	0.16	0.6	202	0.1	60.95
T0913TS396_3-D1.pdb	18.02	0.26	0.13	0.61	206	0.09	61.17
T0913TS396_4-D1.pdb	18.92	0.25	0.14	0.61	206	0.09	60.95
T0913TS396_5-D1.pdb	18.92	0.21	0.11	0.68	230	0.08	59.47
T0913TS399_1-D1.pdb	56.76	0.25	0	0.75	252	0.23	9.76
T0913TS399_2-D1.pdb	59.46	0.25	0	0.75	252	0.24	9.62
T0913TS399_3-D1.pdb	60.36	0.25	0	0.75	252	0.24	9.76
T0913TS399_4-D1.pdb	61.26	0.25	0	0.75	252	0.24	9.62
T0913TS399_5-D1.pdb	58.56	0.25	0	0.75	252	0.23	10.06
T0913TS405_1-D1.pdb	22.52	0.23	0.15	0.62	208	0.11	57.84
T0913TS405_3-D1.pdb	22.52	0.23	0.15	0.62	209	0.11	57.17
T0913TS405_5-D1.pdb	27.03	0.24	0.16	0.6	203	0.13	57.69
T0913TS407_1-D1.pdb	22.52	0.22	0.12	0.67	225	0.1	54.81
T0913TS407_2-D1.pdb	20.72	0.17	0.12	0.7	237	0.09	55.4
T0913TS407_3-D1.pdb	18.92	0.22	0.08	0.7	235	0.08	56.06
T0913TS407_4-D1.pdb	23.42	0.2	0.09	0.71	239	0.1	52.52
T0913TS407_5-D1.pdb	24.32	0.2	0.09	0.71	240	0.1	53.92
T0913TS411_1-D1.pdb	22.52	0.25	0.14	0.62	209	0.11	57.54
T0913TS411_2-D1.pdb	25.23	0.28	0.13	0.59	200	0.13	60.58
T0913TS411_3-D1.pdb	19.82	0.25	0.15	0.61	205	0.1	61.32

T0913TS411_4-D1.pdb	20.72	0.22	0.15	0.63	214	0.1	62.43
T0913TS411_5-D1.pdb	17.12	0.25	0.12	0.62	210	0.08	60.65
T0913TS420_3-D1.pdb	33.33	0.22	0.04	0.74	249	0.13	18.12
T0913TS420_4-D1.pdb	36.04	0.25	0.04	0.71	240	0.15	18.34
T0913TS420_5-D1.pdb	27.03	0.27	0.07	0.66	222	0.12	18.93
T0913TS425_1-D1.pdb	19.82	0.22	0.16	0.61	207	0.1	58.14
T0913TS425_2-D1.pdb	24.32	0.22	0.16	0.62	209	0.12	59.1
T0913TS425_3-D1.pdb	20.72	0.2	0.16	0.65	219	0.09	58.21
T0913TS425_4-D1.pdb	18.02	0.21	0.15	0.63	214	0.08	58.36
T0913TS425_5-D1.pdb	21.62	0.22	0.16	0.61	207	0.1	58.14
T0913TS432_2-D1.pdb	40.54	0.24	0.06	0.7	237	0.17	23
T0913TS434_1-D1.pdb	34.23	0.23	0	0.77	259	0.13	11.39
T0913TS434_2-D1.pdb	35.14	0.25	0	0.75	254	0.14	10.28
T0913TS434_3-D1.pdb	42.34	0.24	0.01	0.75	252	0.17	10.65
T0913TS434_4-D1.pdb	42.34	0.24	0.02	0.73	248	0.17	11.32
T0913TS434_5-D1.pdb	40.54	0.22	0.01	0.76	258	0.16	9.54
T0913TS439_1-D1.pdb	18.02	0.34	0.17	0.5	168	0.11	62.87
T0913TS439_2-D1.pdb	18.92	0.35	0.17	0.48	161	0.12	61.32
T0913TS439_3-D1.pdb	18.92	0.35	0.17	0.49	165	0.11	60.95
T0913TS439_4-D1.pdb	20.72	0.24	0.17	0.59	198	0.1	55.1
T0913TS439_5-D1.pdb	18.02	0.23	0.17	0.61	205	0.09	57.54
T0913TS441_1-D1.pdb	18.02	0.3	0.16	0.55	185	0.1	61.54
T0913TS441_3-D1.pdb	14.41	0.31	0.16	0.52	177	0.08	60.43
T0913TS441_4-D1.pdb	17.12	0.3	0.17	0.54	181	0.09	61.54
T0913TS441_5-D1.pdb	14.41	0.3	0.16	0.54	184	0.08	62.72
T0913TS443_1-D1.pdb	36.94	0.19	0.12	0.69	232	0.16	23.08
T0913TS443_2-D1.pdb	33.33	0.19	0.12	0.69	234	0.14	25.15
T0913TS443_3-D1.pdb	32.43	0.19	0.12	0.69	234	0.14	25
T0913TS443_4-D1.pdb	35.14	0.19	0.12	0.69	234	0.15	23.37
T0913TS443_5-D1.pdb	36.04	0.19	0.12	0.69	234	0.15	23.08
T0913TS444_1-D1.pdb	21.62	0.26	0.17	0.57	193	0.11	58.06
T0913TS444_4-D1.pdb	16.22	0.27	0.13	0.6	203	0.08	61.69
T0913TS444_5-D1.pdb	17.12	0.25	0.16	0.59	198	0.09	56.95
T0913TS450_1-D1.pdb	17.12	0.33	0.17	0.5	169	0.1	67.83
T0913TS450_2-D1.pdb	18.92	0.33	0.17	0.5	168	0.11	67.53
T0913TS450_3-D1.pdb	17.12	0.33	0.17	0.5	169	0.1	68.19
T0913TS450_4-D1.pdb	17.12	0.31	0.16	0.53	179	0.1	68.56
T0913TS450_5-D1.pdb	16.22	0.33	0.18	0.49	167	0.1	68.27
T0913TS451_1-D1.pdb	36.94	0.23	0.03	0.74	250	0.15	12.13
T0913TS451_2-D1.pdb	41.44	0.22	0.03	0.75	255	0.16	24.48
T0913TS451_3-D1.pdb	39.64	0.22	0.03	0.75	253	0.16	18.12
T0913TS451_4-D1.pdb	36.94	0.22	0.01	0.78	262	0.14	18.12
T0913TS451_5-D1.pdb	38.74	0.23	0.01	0.76	257	0.15	17.6
T0913TS452_1-D1.pdb	27.03	0.12	0.14	0.73	248	0.11	48.96
T0913TS452_2-D1.pdb	21.62	0.23	0.13	0.63	214	0.1	50.3
T0913TS452_3-D1.pdb	22.52	0.17	0.12	0.71	239	0.09	53.55
T0913TS452_4-D1.pdb	22.52	0.27	0.16	0.58	195	0.12	53.25

T0913TS452_5-D1.pdb	27.03	0.25	0.15	0.61	205	0.13	57.62
T0913TS455_1-D1.pdb	39.64	0.14	0.05	0.81	273	0.15	25.07
T0913TS455_2-D1.pdb	27.93	0.2	0.08	0.72	245	0.11	36.32
T0913TS455_3-D1.pdb	44.14	0.1	0.05	0.86	289	0.15	6.8
T0913TS455_4-D1.pdb	36.04	0.07	0	0.93	313	0.12	8.06
T0913TS455_5-D1.pdb	36.94	0.09	0.08	0.83	280	0.13	7.17
T0913TS456_1-D1.pdb	19.82	0.33	0.15	0.52	175	0.11	64.64
T0913TS456_2-D1.pdb	18.92	0.32	0.16	0.52	175	0.11	63.39
T0913TS456_3-D1.pdb	16.22	0.36	0.17	0.47	160	0.1	63.68
T0913TS456_4-D1.pdb	21.62	0.33	0.17	0.49	167	0.13	63.39
T0913TS456_5-D1.pdb	23.42	0.33	0.15	0.52	176	0.13	62.5
T0913TS464_1-D1.pdb	22.52	0.22	0.15	0.64	215	0.1	56.88
T0913TS464_2-D1.pdb	55.86	0.11	0	0.89	301	0.19	6.66
T0913TS464_4-D1.pdb	21.62	0.22	0.15	0.64	215	0.1	56.88
T0913TS464_5-D1.pdb	64.86	0.17	0	0.83	280	0.23	8.51
T0913TS467_1-D1.pdb	20.72	0.24	0.16	0.6	202	0.1	55.47
T0913TS467_2-D1.pdb	20.72	0.23	0.16	0.6	204	0.1	51.77
T0913TS467_3-D1.pdb	22.52	0.15	0.12	0.73	246	0.09	34.84
T0913TS467_4-D1.pdb	19.82	0.2	0.13	0.67	225	0.09	45.93
T0913TS467_5-D1.pdb	27.03	0.2	0.12	0.67	228	0.12	30.99
T0913TS475_1-D1.pdb	22.52	0.28	0.14	0.59	198	0.11	56.29
T0913TS475_2-D1.pdb	21.62	0.35	0.15	0.51	171	0.13	56.66
T0913TS475_3-D1.pdb	21.62	0.32	0.14	0.54	181	0.12	59.32
T0913TS475_4-D1.pdb	17.12	0.33	0.16	0.51	172	0.1	59.32
T0913TS475_5-D1.pdb	23.42	0.3	0.14	0.56	189	0.12	57.91
T0913TS479_3-D1.pdb	18.92	0.22	0.11	0.67	228	0.08	59.39
T0913TS479_4-D1.pdb	19.82	0.25	0.16	0.59	201	0.1	56.21
T0913TS479_5-D1.pdb	16.22	0.25	0.13	0.62	210	0.08	61.98
T0913TS480_1-D1.pdb	24.32	0.28	0.15	0.57	192	0.13	55.77
T0913TS480_2-D1.pdb	22.52	0.25	0.14	0.62	208	0.11	55.55
T0913TS480_4-D1.pdb	14.41	0.27	0.13	0.61	205	0.07	54.59
T0913TS483_1-D1.pdb	18.02	0.22	0.14	0.64	217	0.08	54.88
T0913TS483_2-D1.pdb	18.92	0.24	0.17	0.59	199	0.1	56.06
T0913TS483_3-D1.pdb	18.02	0.23	0.14	0.62	210	0.09	54.14
T0913TS483_4-D1.pdb	36.04	0.3	0.02	0.68	230	0.16	8.21
T0913TS483_5-D1.pdb	28.83	0.14	0.1	0.75	255	0.11	9.62
T0913TS486_2-D1.pdb	20.72	0.26	0.07	0.67	225	0.09	56.73
T0913TS486_3-D1.pdb	16.22	0.2	0.04	0.75	255	0.06	53.92
T0913TS486_4-D1.pdb	15.32	0.23	0.08	0.69	234	0.07	57.47
T0913TS486_5-D1.pdb	18.92	0.2	0.06	0.74	251	0.08	54.81
T0913TS489_1-D1.pdb	44.14	0.31	0.04	0.64	217	0.2	8.65
T0913TS495_1-D1.pdb	19.82	0.24	0.14	0.62	208	0.1	57.1
T0913TS495_2-D1.pdb	18.02	0.25	0.15	0.6	204	0.09	56.88
T0913TS495_3-D1.pdb	17.12	0.24	0.14	0.63	212	0.08	57.25
T0913TS495_4-D1.pdb	16.22	0.23	0.14	0.63	213	0.08	57.62
T0913TS495_5-D1.pdb	17.12	0.23	0.13	0.64	215	0.08	57.25
T0913TS498_2-D1.pdb	18.02	0.32	0.16	0.53	178	0.1	60.95

T0913TS498_3-D1.pdb	14.41	0.25	0.13	0.62	208	0.07	61.17
T0913TS498_4-D1.pdb	18.92	0.3	0.16	0.54	182	0.1	59.84
T0917TS005_1-D1.pdb	17.8	0.51	0.07	0.41	162	0.11	83.18
T0917TS005_2-D1.pdb	22.03	0.52	0.06	0.41	161	0.14	85.49
T0917TS005_3-D1.pdb	19.49	0.53	0.08	0.39	153	0.13	84.53
T0917TS005_4-D1.pdb	19.49	0.53	0.07	0.4	157	0.12	84.21
T0917TS005_5-D1.pdb	22.03	0.53	0.07	0.4	158	0.14	85.68
T0917TS016_1-D1.pdb	22.03	0.52	0.06	0.42	163	0.14	72.19
T0917TS026_1-D1.pdb	21.19	0.49	0.08	0.43	169	0.13	69.95
T0917TS026_2-D1.pdb	19.49	0.49	0.06	0.44	173	0.11	74.81
T0917TS026_3-D1.pdb	22.03	0.47	0.07	0.46	180	0.12	72.12
T0917TS026_4-D1.pdb	21.19	0.45	0.08	0.47	183	0.12	70.78
T0917TS028_1-D1.pdb	19.49	0.51	0.07	0.42	166	0.12	70.46
T0917TS048_1-D1.pdb	21.19	0.55	0.07	0.38	147	0.14	79.48
T0917TS077_1-D1.pdb	20.34	0.51	0.06	0.42	165	0.12	74.62
T0917TS077_2-D1.pdb	19.49	0.52	0.06	0.42	163	0.12	74.74
T0917TS077_3-D1.pdb	22.03	0.51	0.07	0.42	163	0.14	74.3
T0917TS077_4-D1.pdb	17.8	0.52	0.07	0.41	162	0.11	74.74
T0917TS077_5-D1.pdb	17.8	0.53	0.06	0.41	160	0.11	74.17
T0917TS119_1-D1.pdb	19.49	0.53	0.05	0.42	164	0.12	76.73
T0917TS166_1-D1.pdb	21.19	0.49	0.06	0.44	173	0.12	70.59
T0917TS180_1-D1.pdb	23.73	0.52	0.07	0.41	161	0.15	73.59
T0917TS180_2-D1.pdb	23.73	0.51	0.07	0.42	163	0.15	70.01
T0917TS180_3-D1.pdb	23.73	0.51	0.08	0.41	159	0.15	69.05
T0917TS180_4-D1.pdb	24.58	0.49	0.07	0.44	171	0.14	57.23
T0917TS180_5-D1.pdb	16.1	0.43	0	0.57	222	0.07	31.71
T0917TS183_1-D1.pdb	21.19	0.51	0.03	0.46	179	0.12	73.53
T0917TS183_2-D1.pdb	20.34	0.51	0.06	0.42	166	0.12	73.15
T0917TS183_3-D1.pdb	22.88	0.52	0.06	0.42	166	0.14	70.08
T0917TS183_4-D1.pdb	21.19	0.54	0.05	0.41	159	0.13	75
T0917TS183_5-D1.pdb	22.03	0.51	0.06	0.43	168	0.13	71.48
T0917TS220_1-D1.pdb	22.03	0.51	0.08	0.41	160	0.14	80.37
T0917TS220_2-D1.pdb	23.73	0.51	0.09	0.4	158	0.15	80.05
T0917TS220_3-D1.pdb	19.49	0.52	0.08	0.4	156	0.12	79.22
T0917TS220_4-D1.pdb	22.03	0.54	0.07	0.39	154	0.14	79.35
T0917TS220_5-D1.pdb	21.19	0.55	0.08	0.37	145	0.15	78.84
T0917TS236_1-D1.pdb	22.03	0.53	0.07	0.39	154	0.14	74.49
T0917TS236_2-D1.pdb	22.88	0.51	0.07	0.42	165	0.14	74.81
T0917TS236_4-D1.pdb	20.34	0.54	0.07	0.38	149	0.14	73.85
T0917TS236_5-D1.pdb	21.19	0.51	0.08	0.4	157	0.13	79.86
T0917TS250_1-D1.pdb	19.49	0.53	0.07	0.4	157	0.12	72.57
T0917TS250_2-D1.pdb	18.64	0.53	0.07	0.4	157	0.12	72.7
T0917TS250_3-D1.pdb	17.8	0.53	0.07	0.4	157	0.11	72.51
T0917TS250_4-D1.pdb	19.49	0.53	0.07	0.4	158	0.12	72.31
T0917TS250_5-D1.pdb	16.95	0.52	0.07	0.41	159	0.11	72.51
T0917TS251_1-D1.pdb	21.19	0.51	0.07	0.41	162	0.13	71.23
T0917TS251_3-D1.pdb	22.03	0.47	0.07	0.46	180	0.12	71.48

T0917TS251_5-D1.pdb	21.19	0.51	0.08	0.41	159	0.13	71.04
T0917TS258_1-D1.pdb	21.19	0.49	0.07	0.44	172	0.12	76.28
T0917TS258_2-D1.pdb	20.34	0.49	0.06	0.45	177	0.11	75.58
T0917TS258_3-D1.pdb	16.95	0.47	0.07	0.47	182	0.09	71.04
T0917TS258_4-D1.pdb	19.49	0.5	0.06	0.43	170	0.11	75.96
T0917TS258_5-D1.pdb	18.64	0.5	0.06	0.44	171	0.11	75.83
T0917TS275_1-D1.pdb	22.88	0.52	0.08	0.4	156	0.15	72.89
T0917TS275_2-D1.pdb	20.34	0.53	0.08	0.39	153	0.13	79.48
T0917TS275_3-D1.pdb	20.34	0.52	0.07	0.4	157	0.13	76.09
T0917TS275_4-D1.pdb	18.64	0.53	0.08	0.4	155	0.12	75.7
T0917TS275_5-D1.pdb	17.8	0.51	0.06	0.43	168	0.11	71.93
T0917TS284_1-D1.pdb	20.34	0.5	0.07	0.43	168	0.12	72.95
T0917TS284_2-D1.pdb	23.73	0.51	0.07	0.41	161	0.15	73.66
T0917TS284_3-D1.pdb	21.19	0.51	0.08	0.41	161	0.13	72.95
T0917TS284_4-D1.pdb	26.27	0.41	0.07	0.51	201	0.13	60.04
T0917TS284_5-D1.pdb	28.81	0.45	0.08	0.47	184	0.16	52.24
T0917TS287_1-D1.pdb	22.03	0.54	0.07	0.39	151	0.15	74.3
T0917TS287_2-D1.pdb	21.19	0.54	0.07	0.39	152	0.14	74.36
T0917TS287_3-D1.pdb	22.03	0.54	0.07	0.38	150	0.15	74.3
T0917TS287_4-D1.pdb	22.03	0.54	0.07	0.39	151	0.15	74.55
T0917TS287_5-D1.pdb	20.34	0.54	0.08	0.38	150	0.14	74.49
T0917TS313_1-D1.pdb	21.19	0.51	0.05	0.44	172	0.12	77.88
T0917TS313_2-D1.pdb	22.03	0.51	0.05	0.44	172	0.13	77.88
T0917TS313_3-D1.pdb	22.03	0.51	0.05	0.44	172	0.13	78.2
T0917TS313_5-D1.pdb	22.03	0.52	0.05	0.43	169	0.13	78.58
T0917TS321_1-D1.pdb	42.37	0.46	0.07	0.47	185	0.23	9.21
T0917TS321_2-D1.pdb	45.76	0.46	0.05	0.49	192	0.24	8.63
T0917TS321_3-D1.pdb	48.31	0.46	0.05	0.5	194	0.25	9.85
T0917TS321_4-D1.pdb	38.14	0.47	0.05	0.48	187	0.2	9.72
T0917TS321_5-D1.pdb	38.98	0.46	0.05	0.48	189	0.21	9.78
T0917TS345_1-D1.pdb	22.88	0.54	0.08	0.38	150	0.15	74.17
T0917TS345_2-D1.pdb	22.88	0.54	0.08	0.38	150	0.15	73.59
T0917TS345_3-D1.pdb	22.03	0.55	0.08	0.37	144	0.15	75
T0917TS345_4-D1.pdb	19.49	0.54	0.07	0.39	152	0.13	80.88
T0917TS345_5-D1.pdb	21.19	0.53	0.08	0.4	155	0.14	80.5
T0917TS349_1-D1.pdb	22.88	0.53	0.05	0.42	164	0.14	76.73
T0917TS357_1-D1.pdb	23.73	0.42	0	0.58	228	0.1	72.51
T0917TS357_2-D1.pdb	24.58	0.41	0.01	0.58	227	0.11	70.97
T0917TS357_3-D1.pdb	22.03	0.4	0.01	0.59	231	0.1	71.61
T0917TS357_4-D1.pdb	23.73	0.39	0.01	0.6	236	0.1	71.93
T0917TS357_5-D1.pdb	22.88	0.41	0.02	0.57	224	0.1	71.36
T0917TS359_1-D1.pdb	21.19	0.49	0.08	0.43	169	0.13	75.58
T0917TS359_3-D1.pdb	25.42	0.5	0.07	0.43	169	0.15	73.08
T0917TS359_4-D1.pdb	26.27	0.49	0.07	0.44	171	0.15	76.53
T0917TS359_5-D1.pdb	22.88	0.51	0.06	0.42	165	0.14	81.01
T0917TS382_1-D1.pdb	27.12	0.41	0.09	0.5	196	0.14	47.57
T0917TS382_2-D1.pdb	27.97	0.4	0.09	0.51	200	0.14	46.23

T0917TS382_3-D1.pdb	31.36	0.39	0.09	0.52	204	0.15	47.95
T0917TS382_4-D1.pdb	27.97	0.41	0.09	0.5	197	0.14	47.44
T0917TS382_5-D1.pdb	28.81	0.4	0.09	0.51	201	0.14	47.95
T0917TS405_1-D1.pdb	22.03	0.53	0.07	0.4	157	0.14	73.53
T0917TS405_2-D1.pdb	25.42	0.52	0.08	0.39	154	0.17	72.44
T0917TS405_4-D1.pdb	20.34	0.52	0.08	0.41	159	0.13	71.93
T0917TS407_1-D1.pdb	19.49	0.5	0.06	0.44	171	0.11	75.51
T0917TS407_2-D1.pdb	20.34	0.51	0.06	0.42	166	0.12	72.25
T0917TS407_3-D1.pdb	21.19	0.52	0.06	0.41	162	0.13	75
T0917TS407_4-D1.pdb	21.19	0.49	0.06	0.45	176	0.12	73.85
T0917TS407_5-D1.pdb	22.03	0.51	0.06	0.43	170	0.13	74.36
T0917TS421_1-D1.pdb	21.19	0.47	0.07	0.46	178	0.12	75.89
T0917TS421_2-D1.pdb	17.8	0.47	0.05	0.48	186	0.1	70.78
T0917TS421_3-D1.pdb	25.42	0.48	0.03	0.49	192	0.13	71.55
T0917TS421_4-D1.pdb	21.19	0.49	0.05	0.46	178	0.12	73.53
T0917TS421_5-D1.pdb	21.19	0.46	0.05	0.49	193	0.11	70.14
T0917TS425_1-D1.pdb	22.03	0.53	0.06	0.41	161	0.14	74.42
T0917TS425_2-D1.pdb	22.03	0.51	0.07	0.41	162	0.14	74.3
T0917TS425_3-D1.pdb	19.49	0.52	0.06	0.42	164	0.12	74.81
T0917TS425_4-D1.pdb	21.19	0.51	0.07	0.41	162	0.13	75.38
T0917TS425_5-D1.pdb	22.03	0.52	0.06	0.41	161	0.14	75.13
T0917TS430_1-D1.pdb	21.19	0.5	0.08	0.42	165	0.13	75.89
T0917TS430_2-D1.pdb	22.88	0.51	0.08	0.41	160	0.14	80.31
T0917TS430_3-D1.pdb	22.88	0.51	0.07	0.42	164	0.14	78.52
T0917TS430_4-D1.pdb	23.73	0.51	0.08	0.41	159	0.15	78.71
T0917TS430_5-D1.pdb	22.03	0.53	0.07	0.4	156	0.14	78.84
T0917TS432_1-D1.pdb	25.42	0.49	0.05	0.46	181	0.14	40.66
T0917TS432_2-D1.pdb	27.12	0.5	0.06	0.45	174	0.16	38.04
T0917TS432_3-D1.pdb	26.27	0.49	0.04	0.47	184	0.14	36.64
T0917TS432_4-D1.pdb	27.12	0.46	0.05	0.5	194	0.14	41.37
T0917TS432_5-D1.pdb	20.34	0.53	0.03	0.44	172	0.12	38.62
T0917TS434_1-D1.pdb	39.83	0.46	0	0.54	211	0.19	12.6
T0917TS434_2-D1.pdb	43.22	0.42	0	0.58	226	0.19	12.28
T0917TS434_3-D1.pdb	29.66	0.43	0	0.57	223	0.13	15.22
T0917TS434_4-D1.pdb	38.14	0.48	0.02	0.51	199	0.19	10.68
T0917TS434_5-D1.pdb	34.75	0.46	0	0.54	213	0.16	9.85
T0917TS444_1-D1.pdb	17.8	0.45	0.06	0.49	192	0.09	72.63
T0917TS444_2-D1.pdb	21.19	0.45	0.07	0.48	187	0.11	75.45
T0917TS444_3-D1.pdb	20.34	0.48	0.07	0.45	176	0.12	72.63
T0917TS444_4-D1.pdb	22.03	0.49	0.06	0.45	175	0.13	73.85
T0917TS444_5-D1.pdb	19.49	0.49	0.08	0.42	166	0.12	74.94
T0917TS446_1-D1.pdb	15.25	0.54	0.08	0.38	150	0.1	80.56
T0917TS446_2-D1.pdb	18.64	0.52	0.07	0.41	159	0.12	77.94
T0917TS446_3-D1.pdb	22.03	0.5	0.09	0.41	161	0.14	72.76
T0917TS446_4-D1.pdb	19.49	0.5	0.07	0.43	168	0.12	75.58
T0917TS446_5-D1.pdb	22.88	0.5	0.07	0.42	166	0.14	73.27
T0917TS451_1-D1.pdb	27.97	0.47	0.02	0.51	200	0.14	33.89

T0917TS451_2-D1.pdb	25.42	0.46	0.01	0.53	208	0.12	35.61
T0917TS451_3-D1.pdb	26.27	0.48	0.01	0.51	198	0.13	36.19
T0917TS451_4-D1.pdb	28.81	0.47	0.01	0.52	203	0.14	34.14
T0917TS451_5-D1.pdb	24.58	0.46	0.03	0.51	200	0.12	35.93
T0917TS452_1-D1.pdb	22.03	0.5	0.07	0.43	170	0.13	70.59
T0917TS452_2-D1.pdb	18.64	0.51	0.08	0.41	162	0.12	75
T0917TS452_3-D1.pdb	18.64	0.52	0.06	0.41	161	0.12	69.25
T0917TS452_4-D1.pdb	21.19	0.52	0.06	0.42	164	0.13	72.63
T0917TS452_5-D1.pdb	21.19	0.48	0.09	0.44	171	0.12	71.42
T0917TS455_1-D1.pdb	22.03	0.45	0.06	0.49	190	0.12	71.93
T0917TS455_2-D1.pdb	25.42	0.34	0.07	0.58	228	0.11	58.44
T0917TS455_3-D1.pdb	29.66	0.42	0.06	0.52	203	0.15	60.61
T0917TS455_4-D1.pdb	28.81	0.35	0.04	0.62	241	0.12	51.73
T0917TS464_1-D1.pdb	22.88	0.51	0.07	0.42	166	0.14	74.17
T0917TS464_2-D1.pdb	55.08	0.28	0.02	0.7	275	0.2	6.59
T0917TS464_3-D1.pdb	21.19	0.5	0.07	0.42	165	0.13	74.11
T0917TS464_4-D1.pdb	21.19	0.5	0.07	0.43	167	0.13	73.4
T0917TS464_5-D1.pdb	64.41	0.32	0	0.68	264	0.24	6.71
T0917TS467_1-D1.pdb	17.8	0.5	0.08	0.42	165	0.11	68.16
T0917TS467_2-D1.pdb	17.8	0.51	0.07	0.41	161	0.11	69.05
T0917TS467_3-D1.pdb	20.34	0.5	0.08	0.41	162	0.13	69.56
T0917TS467_4-D1.pdb	22.03	0.49	0.06	0.45	177	0.12	73.47
T0917TS467_5-D1.pdb	19.49	0.47	0.07	0.46	178	0.11	73.59
T0917TS479_1-D1.pdb	21.19	0.52	0.06	0.42	165	0.13	73.72
T0917TS479_2-D1.pdb	23.73	0.51	0.06	0.43	169	0.14	73.27
T0917TS479_3-D1.pdb	21.19	0.5	0.06	0.43	169	0.13	70.33
T0917TS479_4-D1.pdb	24.58	0.51	0.06	0.43	167	0.15	71.48
T0917TS479_5-D1.pdb	18.64	0.52	0.04	0.43	169	0.11	75.13
T0917TS495_1-D1.pdb	19.49	0.51	0.08	0.41	161	0.12	72.7
T0917TS495_2-D1.pdb	19.49	0.51	0.07	0.42	164	0.12	76.98
T0917TS495_3-D1.pdb	20.34	0.51	0.08	0.4	158	0.13	73.78
T0917TS495_4-D1.pdb	21.19	0.51	0.08	0.41	160	0.13	76.22
T0917TS495_5-D1.pdb	21.19	0.52	0.08	0.4	157	0.13	77.49
T0920TS005_1-D1.pdb	20.17	0.44	0.19	0.38	121	0.17	70.41
T0920TS005_1-D2.pdb	27.72	0.31	0.29	0.4	97	0.29	67.12
T0920TS005_2-D1.pdb	20.17	0.42	0.18	0.4	127	0.16	71.11
T0920TS005_2-D2.pdb	34.65	0.31	0.31	0.38	92	0.38	69.29
T0920TS005_3-D1.pdb	20.17	0.41	0.17	0.41	133	0.15	71.03
T0920TS005_3-D2.pdb	34.65	0.29	0.31	0.41	98	0.35	70.43
T0920TS005_4-D1.pdb	21.01	0.42	0.18	0.4	128	0.16	73.36
T0920TS005_4-D2.pdb	31.68	0.31	0.27	0.42	101	0.31	70.32
T0920TS005_5-D1.pdb	18.49	0.4	0.17	0.42	136	0.14	74.69
T0920TS005_5-D2.pdb	35.64	0.29	0.29	0.41	99	0.36	71.8
T0920TS016_1-D1.pdb	22.69	0.4	0.17	0.42	136	0.17	70.87
T0920TS016_1-D2.pdb	29.7	0.28	0.22	0.5	120	0.25	65.64
T0920TS026_1-D1.pdb	25.21	0.41	0.15	0.44	142	0.18	51.32
T0920TS026_1-D2.pdb	46.53	0.16	0	0.84	203	0.23	10.27

T0920TS026_2-D1.pdb	25.21	0.39	0.15	0.46	149	0.17	67.29
T0920TS026_2-D2.pdb	37.62	0	0.01	0.99	239	0.16	9.25
T0920TS026_3-D1.pdb	20.17	0.4	0.15	0.45	145	0.14	65.73
T0920TS026_3-D2.pdb	54.46	0.1	0	0.9	218	0.25	10.16
T0920TS026_4-D1.pdb	22.69	0.39	0.12	0.49	158	0.14	65.11
T0920TS026_4-D2.pdb	49.5	0.08	0	0.92	221	0.22	8.45
T0920TS026_5-D1.pdb	27.73	0.36	0.12	0.51	164	0.17	47.04
T0920TS026_5-D2.pdb	63.37	0.09	0	0.91	219	0.29	10.5
T0920TS028_1-D1.pdb	27.73	0.41	0.16	0.43	139	0.2	52.73
T0920TS048_1-D1.pdb	21.01	0.41	0.17	0.42	135	0.16	73.75
T0920TS048_1-D2.pdb	31.68	0.27	0.23	0.5	121	0.26	68.95
T0920TS077_1-D1.pdb	20.17	0.41	0.18	0.41	132	0.15	71.81
T0920TS077_1-D2.pdb	33.66	0.29	0.22	0.5	120	0.28	68.04
T0920TS077_2-D1.pdb	19.33	0.41	0.17	0.42	136	0.14	71.34
T0920TS077_2-D2.pdb	33.66	0.32	0.26	0.43	103	0.33	66.89
T0920TS077_3-D1.pdb	21.01	0.41	0.17	0.42	134	0.16	73.52
T0920TS077_3-D2.pdb	33.66	0.3	0.24	0.45	109	0.31	65.41
T0920TS077_4-D1.pdb	21.01	0.42	0.18	0.4	128	0.16	72.2
T0920TS077_4-D2.pdb	36.63	0.3	0.23	0.47	113	0.32	66.1
T0920TS077_5-D1.pdb	21.85	0.4	0.18	0.42	135	0.16	70.87
T0920TS077_5-D2.pdb	37.62	0.28	0.24	0.48	116	0.32	65.3
T0920TS119_1-D1.pdb	26.05	0.42	0.15	0.43	138	0.19	71.18
T0920TS119_1-D2.pdb	38.61	0.23	0.17	0.6	145	0.27	67.92
T0920TS166_1-D1.pdb	24.37	0.42	0.16	0.42	135	0.18	69.55
T0920TS180_1-D1.pdb	40.34	0.07	0.08	0.84	271	0.15	10.12
T0920TS180_1-D2.pdb	39.6	0.05	0.04	0.9	218	0.18	9.36
T0920TS180_2-D1.pdb	36.97	0.43	0.11	0.47	150	0.25	11.14
T0920TS180_3-D1.pdb	32.77	0	0.16	0.84	271	0.12	6.85
T0920TS180_3-D2.pdb	44.55	0	0.2	0.8	194	0.23	10.5
T0920TS180_4-D1.pdb	31.09	0.07	0.18	0.75	242	0.13	10.75
T0920TS180_4-D2.pdb	50.5	0.06	0.07	0.87	209	0.24	9.59
T0920TS180_5-D1.pdb	36.13	0.33	0	0.67	215	0.17	8.96
T0920TS180_5-D2.pdb	51.49	0.32	0	0.68	163	0.32	16.78
T0920TS183_1-D1.pdb	22.69	0.41	0.13	0.45	145	0.16	68.77
T0920TS183_1-D2.pdb	26.73	0.32	0.2	0.49	117	0.23	71.35
T0920TS183_2-D1.pdb	24.37	0.42	0.15	0.43	138	0.18	54.44
T0920TS183_2-D2.pdb	31.68	0.28	0.18	0.54	129	0.25	62.56
T0920TS183_3-D1.pdb	25.21	0.42	0.12	0.46	148	0.17	51.95
T0920TS183_3-D2.pdb	30.69	0.28	0.23	0.49	119	0.26	62.1
T0920TS183_4-D1.pdb	22.69	0.4	0.1	0.5	162	0.14	61.99
T0920TS183_4-D2.pdb	26.73	0.27	0.07	0.66	158	0.17	62.9
T0920TS183_5-D1.pdb	26.05	0.43	0.12	0.45	145	0.18	51.25
T0920TS183_5-D2.pdb	34.65	0.27	0.17	0.56	135	0.26	67.12
T0920TS220_1-D1.pdb	24.37	0.44	0.17	0.4	128	0.19	62.3
T0920TS220_1-D2.pdb	37.62	0.3	0.27	0.43	104	0.36	77.51
T0920TS220_2-D1.pdb	23.53	0.43	0.17	0.4	128	0.18	63.01
T0920TS220_2-D2.pdb	33.66	0.34	0.27	0.39	94	0.36	76.71

T0920TS220_3-D1.pdb	21.01	0.42	0.17	0.41	133	0.16	79.21
T0920TS220_3-D2.pdb	37.62	0.32	0.24	0.44	105	0.36	77.63
T0920TS220_4-D1.pdb	21.01	0.45	0.18	0.37	120	0.18	79.13
T0920TS220_4-D2.pdb	36.63	0.33	0.23	0.44	106	0.35	79.68
T0920TS220_5-D1.pdb	24.37	0.42	0.17	0.41	132	0.18	56.31
T0920TS220_5-D2.pdb	32.67	0.31	0.24	0.44	107	0.31	70.09
T0920TS236_1-D1.pdb	23.53	0.4	0.16	0.43	139	0.17	74.69
T0920TS236_1-D2.pdb	56.44	0.18	0.16	0.66	159	0.35	24.43
T0920TS236_2-D1.pdb	21.85	0.41	0.17	0.43	137	0.16	73.75
T0920TS236_2-D2.pdb	43.56	0.19	0.18	0.63	152	0.29	45.2
T0920TS236_3-D1.pdb	20.17	0.41	0.12	0.47	152	0.13	72.97
T0920TS236_3-D2.pdb	47.52	0.19	0.15	0.66	160	0.3	48.06
T0920TS236_4-D1.pdb	26.05	0.4	0.16	0.44	142	0.18	74.3
T0920TS236_4-D2.pdb	68.32	0.17	0.15	0.68	163	0.42	18.72
T0920TS236_5-D1.pdb	21.85	0.4	0.16	0.44	142	0.15	76.17
T0920TS236_5-D2.pdb	40.59	0.18	0.2	0.62	150	0.27	54.22
T0920TS250_1-D1.pdb	22.69	0.43	0.17	0.4	128	0.18	73.91
T0920TS250_1-D2.pdb	29.7	0.23	0.26	0.51	123	0.24	64.73
T0920TS250_2-D1.pdb	22.69	0.43	0.17	0.4	129	0.18	74.22
T0920TS250_2-D2.pdb	26.73	0.24	0.26	0.5	121	0.22	64.61
T0920TS250_3-D1.pdb	19.33	0.43	0.17	0.4	128	0.15	73.83
T0920TS250_3-D2.pdb	26.73	0.22	0.26	0.51	124	0.22	63.81
T0920TS250_4-D1.pdb	20.17	0.43	0.16	0.41	132	0.15	73.13
T0920TS250_4-D2.pdb	28.71	0.24	0.26	0.5	121	0.24	64.5
T0920TS250_5-D1.pdb	21.85	0.43	0.17	0.4	130	0.17	73.36
T0920TS250_5-D2.pdb	26.73	0.24	0.26	0.5	121	0.22	64.16
T0920TS251_1-D1.pdb	26.05	0.45	0.16	0.4	127	0.21	69
T0920TS251_2-D1.pdb	25.21	0.42	0.16	0.42	135	0.19	64.88
T0920TS251_3-D1.pdb	30.25	0.41	0.15	0.44	141	0.21	55.06
T0920TS251_4-D1.pdb	25.21	0.41	0.17	0.41	132	0.19	68.92
T0920TS251_5-D1.pdb	24.37	0.41	0.19	0.4	130	0.19	69.31
T0920TS258_1-D1.pdb	28.57	0.31	0.09	0.6	193	0.15	53.66
T0920TS258_1-D2.pdb	34.65	0.09	0.02	0.89	215	0.16	11.3
T0920TS258_2-D1.pdb	26.89	0.32	0.1	0.58	185	0.15	52.88
T0920TS258_2-D2.pdb	32.67	0.1	0.03	0.87	210	0.16	11.07
T0920TS258_3-D1.pdb	25.21	0.31	0.08	0.61	195	0.13	49.06
T0920TS258_3-D2.pdb	34.65	0.1	0.02	0.88	213	0.16	11.19
T0920TS258_4-D1.pdb	26.05	0.32	0.1	0.58	187	0.14	54.98
T0920TS258_4-D2.pdb	30.69	0.1	0.01	0.89	215	0.14	11.19
T0920TS258_5-D1.pdb	27.73	0.32	0.1	0.58	185	0.15	52.49
T0920TS258_5-D2.pdb	33.66	0.09	0.03	0.88	211	0.16	11.07
T0920TS275_1-D1.pdb	25.21	0.4	0.14	0.45	145	0.17	57.94
T0920TS275_1-D2.pdb	64.36	0.25	0.16	0.59	142	0.45	51.94
T0920TS275_2-D1.pdb	26.05	0.4	0.15	0.44	142	0.18	58.1
T0920TS275_2-D2.pdb	61.39	0.23	0.14	0.63	153	0.4	51.48
T0920TS275_3-D1.pdb	24.37	0.39	0.14	0.47	150	0.16	57.87
T0920TS275_3-D2.pdb	56.44	0.25	0.15	0.6	145	0.39	50

T0920TS275_4-D1.pdb	27.73	0.4	0.16	0.44	141	0.2	62.85
T0920TS275_4-D2.pdb	60.4	0.24	0.16	0.6	145	0.42	53.99
T0920TS275_5-D1.pdb	28.57	0.41	0.15	0.44	142	0.2	52.34
T0920TS275_5-D2.pdb	55.45	0.24	0.17	0.59	143	0.39	52.4
T0920TS287_1-D1.pdb	23.53	0.4	0.18	0.42	136	0.17	74.38
T0920TS287_1-D2.pdb	53.47	0.19	0.16	0.65	157	0.34	25.11
T0920TS287_2-D1.pdb	21.01	0.41	0.18	0.41	131	0.16	75.08
T0920TS287_2-D2.pdb	67.33	0.17	0.17	0.66	159	0.42	16.44
T0920TS287_3-D1.pdb	22.69	0.4	0.18	0.41	133	0.17	75.55
T0920TS287_3-D2.pdb	69.31	0.16	0.17	0.68	163	0.43	14.84
T0920TS287_4-D1.pdb	22.69	0.41	0.18	0.41	131	0.17	75.55
T0920TS287_4-D2.pdb	66.34	0.18	0.15	0.67	162	0.41	18.72
T0920TS287_5-D1.pdb	20.17	0.41	0.17	0.42	135	0.15	73.13
T0920TS287_5-D2.pdb	43.56	0.2	0.15	0.65	157	0.28	45.09
T0920TS313_1-D1.pdb	25.21	0.39	0.15	0.46	147	0.17	69.86
T0920TS313_1-D2.pdb	40.59	0.23	0.18	0.59	142	0.29	66.44
T0920TS313_2-D1.pdb	25.21	0.39	0.15	0.46	149	0.17	69.94
T0920TS313_2-D2.pdb	40.59	0.23	0.18	0.59	142	0.29	66.89
T0920TS313_3-D1.pdb	27.73	0.39	0.16	0.45	145	0.19	69.86
T0920TS313_3-D2.pdb	40.59	0.23	0.2	0.56	136	0.3	67.35
T0920TS313_4-D1.pdb	26.89	0.38	0.15	0.47	151	0.18	70.02
T0920TS313_4-D2.pdb	38.61	0.23	0.18	0.59	142	0.27	67.47
T0920TS313_5-D1.pdb	26.05	0.38	0.16	0.46	147	0.18	69.94
T0920TS321_1-D1.pdb	57.98	0.35	0.1	0.55	175	0.33	9.03
T0920TS321_1-D2.pdb	66.34	0.3	0.12	0.58	140	0.47	14.04
T0920TS321_2-D1.pdb	51.26	0.32	0.11	0.57	183	0.28	8.8
T0920TS321_2-D2.pdb	72.28	0.29	0.1	0.61	147	0.49	12.21
T0920TS321_3-D1.pdb	54.62	0.34	0.1	0.56	179	0.31	9.97
T0920TS321_3-D2.pdb	65.35	0.28	0.12	0.6	145	0.45	13.13
T0920TS321_4-D1.pdb	57.14	0.33	0.1	0.57	183	0.31	9.66
T0920TS321_4-D2.pdb	70.3	0.3	0.12	0.58	140	0.5	13.24
T0920TS321_5-D1.pdb	55.46	0.35	0.1	0.55	178	0.31	10.98
T0920TS321_5-D2.pdb	63.37	0.3	0.12	0.58	140	0.45	13.24
T0920TS345_1-D1.pdb	21.01	0.41	0.2	0.39	126	0.17	74.22
T0920TS345_1-D2.pdb	31.68	0.3	0.23	0.47	114	0.28	62.22
T0920TS345_2-D1.pdb	23.53	0.43	0.2	0.38	121	0.19	69.47
T0920TS345_2-D2.pdb	35.64	0.28	0.28	0.44	106	0.34	60.5
T0920TS345_3-D1.pdb	23.53	0.41	0.18	0.41	133	0.18	75.31
T0920TS345_3-D2.pdb	39.6	0.27	0.25	0.48	116	0.34	59.59
T0920TS345_4-D1.pdb	22.69	0.41	0.17	0.42	135	0.17	70.02
T0920TS345_4-D2.pdb	37.62	0.27	0.27	0.47	113	0.33	59.93
T0920TS345_5-D1.pdb	24.37	0.4	0.17	0.43	138	0.18	73.52
T0920TS345_5-D2.pdb	36.63	0.27	0.24	0.49	118	0.31	61.99
T0920TS349_1-D1.pdb	23.53	0.42	0.15	0.43	138	0.17	71.18
T0920TS349_1-D2.pdb	40.59	0.23	0.17	0.6	145	0.28	67.92
T0920TS357_1-D1.pdb	26.89	0.36	0.02	0.63	201	0.13	70.56
T0920TS357_2-D1.pdb	24.37	0.36	0.01	0.64	204	0.12	69.94

T0920TS357_3-D1.pdb	24.37	0.31	0.01	0.68	218	0.11	70.09
T0920TS357_4-D1.pdb	26.05	0.35	0	0.65	208	0.13	70.56
T0920TS357_5-D1.pdb	27.73	0.35	0.06	0.6	192	0.14	71.03
T0920TS359_1-D1.pdb	24.37	0.38	0.17	0.45	144	0.17	68.77
T0920TS359_2-D1.pdb	24.37	0.39	0.15	0.46	148	0.16	60.75
T0920TS359_3-D1.pdb	27.73	0.42	0.14	0.43	139	0.2	70.56
T0920TS359_4-D1.pdb	25.21	0.39	0.17	0.45	144	0.18	67.52
T0920TS382_1-D1.pdb	25.21	0.38	0.15	0.47	151	0.17	66.75
T0920TS382_1-D2.pdb	28.71	0.25	0.25	0.49	119	0.24	66.44
T0920TS382_2-D1.pdb	27.73	0.39	0.14	0.48	153	0.18	66.12
T0920TS382_2-D2.pdb	29.7	0.27	0.24	0.48	116	0.26	64.5
T0920TS382_3-D1.pdb	24.37	0.39	0.14	0.47	151	0.16	65.42
T0920TS382_3-D2.pdb	31.68	0.26	0.24	0.5	121	0.26	60.96
T0920TS382_4-D1.pdb	22.69	0.41	0.13	0.46	148	0.15	66.51
T0920TS382_4-D2.pdb	29.7	0.25	0.25	0.5	120	0.25	63.81
T0920TS382_5-D1.pdb	26.89	0.4	0.14	0.47	150	0.18	67.37
T0920TS382_5-D2.pdb	33.66	0.28	0.23	0.49	118	0.29	63.58
T0920TS405_1-D1.pdb	23.53	0.41	0.17	0.42	135	0.17	71.34
T0920TS405_3-D1.pdb	23.53	0.41	0.17	0.42	134	0.18	66.82
T0920TS405_4-D1.pdb	23.53	0.41	0.15	0.44	141	0.17	69.47
T0920TS405_5-D1.pdb	23.53	0.41	0.16	0.43	138	0.17	69.7
T0920TS407_1-D1.pdb	20.17	0.4	0.13	0.46	149	0.14	70.48
T0920TS407_1-D2.pdb	32.67	0.22	0.19	0.59	143	0.23	56.62
T0920TS407_2-D1.pdb	22.69	0.4	0.13	0.46	149	0.15	69.63
T0920TS407_2-D2.pdb	43.56	0.15	0.18	0.67	162	0.27	45.55
T0920TS407_3-D1.pdb	26.89	0.41	0.11	0.48	153	0.18	66.43
T0920TS407_3-D2.pdb	34.65	0.22	0.15	0.62	150	0.23	59.02
T0920TS407_4-D1.pdb	21.85	0.38	0.15	0.47	151	0.14	67.99
T0920TS407_4-D2.pdb	36.63	0.19	0.19	0.62	150	0.24	45.55
T0920TS407_5-D1.pdb	26.89	0.36	0.11	0.53	171	0.16	61.84
T0920TS407_5-D2.pdb	38.61	0.22	0.16	0.62	150	0.26	57.88
T0920TS421_1-D1.pdb	21.01	0.4	0.14	0.46	148	0.14	68.54
T0920TS421_2-D1.pdb	21.01	0.37	0.12	0.5	162	0.13	65.89
T0920TS421_3-D1.pdb	22.69	0.37	0.14	0.49	158	0.14	66.04
T0920TS421_4-D1.pdb	27.73	0.38	0.11	0.51	165	0.17	67.06
T0920TS421_5-D1.pdb	19.33	0.37	0.14	0.48	155	0.12	70.48
T0920TS425_1-D1.pdb	19.33	0.42	0.17	0.4	130	0.15	71.18
T0920TS425_1-D2.pdb	31.68	0.32	0.23	0.46	110	0.29	66.67
T0920TS425_2-D1.pdb	20.17	0.41	0.17	0.42	136	0.15	71.18
T0920TS425_2-D2.pdb	33.66	0.31	0.22	0.46	112	0.3	69.64
T0920TS425_3-D1.pdb	24.37	0.41	0.17	0.42	134	0.18	71.89
T0920TS425_3-D2.pdb	29.7	0.3	0.23	0.46	112	0.27	68.72
T0920TS425_4-D1.pdb	18.49	0.42	0.18	0.4	130	0.14	71.18
T0920TS425_4-D2.pdb	32.67	0.3	0.24	0.46	111	0.29	68.72
T0920TS425_5-D1.pdb	21.85	0.41	0.17	0.42	135	0.16	71.96
T0920TS425_5-D2.pdb	34.65	0.3	0.24	0.46	110	0.32	65.64
T0920TS432_1-D1.pdb	29.41	0.25	0.05	0.71	227	0.13	27.18

T0920TS432_1-D2.pdb	71.29	0.35	0.02	0.63	152	0.47	14.84
T0920TS432_2-D1.pdb	26.89	0.27	0.04	0.69	220	0.12	30.68
T0920TS432_2-D2.pdb	64.36	0.38	0.01	0.61	148	0.43	15.64
T0920TS432_3-D1.pdb	25.21	0.29	0.06	0.65	209	0.12	30.14
T0920TS432_3-D2.pdb	75.25	0.34	0	0.66	160	0.47	14.84
T0920TS432_4-D1.pdb	29.41	0.28	0.05	0.68	217	0.14	33.49
T0920TS432_4-D2.pdb	75.25	0.32	0.02	0.65	157	0.48	14.15
T0920TS432_5-D1.pdb	29.41	0.29	0.1	0.61	195	0.15	34.58
T0920TS432_5-D2.pdb	67.33	0.28	0.02	0.71	170	0.4	14.15
T0920TS434_1-D1.pdb	44.54	0.4	0	0.6	191	0.23	13.32
T0920TS434_1-D2.pdb	80.2	0.31	0	0.69	166	0.48	13.81
T0920TS434_2-D1.pdb	52.94	0.4	0	0.6	194	0.27	13.55
T0920TS434_2-D2.pdb	76.24	0.31	0	0.69	167	0.46	11.3
T0920TS434_3-D1.pdb	55.46	0.38	0	0.62	198	0.28	13.32
T0920TS434_3-D2.pdb	63.37	0.31	0	0.69	167	0.38	13.24
T0920TS434_4-D1.pdb	52.94	0.41	0	0.59	188	0.28	14.33
T0920TS434_4-D2.pdb	78.22	0.29	0	0.71	172	0.45	9.93
T0920TS434_5-D1.pdb	52.1	0.41	0	0.59	189	0.28	13.4
T0920TS434_5-D2.pdb	69.31	0.31	0	0.69	167	0.42	17.35
T0920TS444_1-D1.pdb	21.85	0.41	0.17	0.42	134	0.16	67.21
T0920TS444_1-D2.pdb	31.68	0.31	0.22	0.47	114	0.28	63.81
T0920TS444_2-D1.pdb	26.89	0.4	0.15	0.44	142	0.19	64.72
T0920TS444_2-D2.pdb	24.75	0.29	0.24	0.47	113	0.22	62.33
T0920TS444_3-D1.pdb	24.37	0.41	0.13	0.45	145	0.17	63.4
T0920TS444_3-D2.pdb	28.71	0.26	0.26	0.48	116	0.25	64.16
T0920TS444_4-D1.pdb	21.01	0.44	0.16	0.41	131	0.16	69.08
T0920TS444_4-D2.pdb	31.68	0.32	0.21	0.48	115	0.28	59.48
T0920TS444_5-D1.pdb	19.33	0.45	0.15	0.41	131	0.15	63.24
T0920TS444_5-D2.pdb	32.67	0.26	0.26	0.48	116	0.28	64.84
T0920TS446_1-D1.pdb	19.33	0.42	0.17	0.42	134	0.14	69.16
T0920TS446_2-D1.pdb	29.41	0.41	0.16	0.43	138	0.21	55.69
T0920TS446_3-D1.pdb	24.37	0.4	0.17	0.43	138	0.18	65.81
T0920TS446_4-D1.pdb	22.69	0.39	0.17	0.45	144	0.16	64.49
T0920TS446_5-D1.pdb	24.37	0.41	0.17	0.42	135	0.18	70.64
T0920TS451_1-D1.pdb	39.5	0.4	0.05	0.54	174	0.23	28.97
T0920TS451_1-D2.pdb	55.45	0.28	0.11	0.61	146	0.38	27.4
T0920TS451_2-D1.pdb	36.97	0.41	0.05	0.54	174	0.21	30.37
T0920TS451_2-D2.pdb	53.47	0.29	0.07	0.65	156	0.34	34.36
T0920TS451_3-D1.pdb	38.66	0.4	0.11	0.49	158	0.24	35.2
T0920TS451_3-D2.pdb	56.44	0.29	0.05	0.66	160	0.35	32.19
T0920TS451_4-D1.pdb	34.45	0.4	0.06	0.54	172	0.2	30.92
T0920TS451_4-D2.pdb	53.47	0.29	0.06	0.65	156	0.34	29.57
T0920TS451_5-D1.pdb	36.97	0.4	0.05	0.54	174	0.21	34.81
T0920TS451_5-D2.pdb	51.49	0.29	0.09	0.62	149	0.35	37.9
T0920TS452_1-D1.pdb	21.85	0.41	0.16	0.43	138	0.16	66.59
T0920TS452_1-D2.pdb	35.64	0.27	0.25	0.49	117	0.3	60.05
T0920TS452_2-D1.pdb	22.69	0.42	0.16	0.42	136	0.17	68.38

T0920TS452_2-D2.pdb	66.34	0.38	0	0.62	149	0.45	13.47
T0920TS452_3-D1.pdb	26.05	0.4	0.18	0.42	135	0.19	50
T0920TS452_3-D2.pdb	69.31	0.37	0	0.63	152	0.46	12.1
T0920TS452_4-D1.pdb	26.89	0.4	0.14	0.46	147	0.18	66.28
T0920TS452_4-D2.pdb	66.34	0.36	0.01	0.63	152	0.44	14.04
T0920TS452_5-D1.pdb	26.05	0.42	0.16	0.42	134	0.19	69.16
T0920TS452_5-D2.pdb	52.48	0.37	0.01	0.62	149	0.35	12.67
T0920TS455_1-D1.pdb	42.86	0.12	0.03	0.85	274	0.16	7.01
T0920TS455_1-D2.pdb	58.42	0.02	0.04	0.94	226	0.26	9.59
T0920TS455_2-D1.pdb	27.73	0.02	0.01	0.98	314	0.09	8.96
T0920TS455_2-D2.pdb	52.48	0.01	0.06	0.93	225	0.23	8.79
T0920TS455_3-D1.pdb	41.18	0.07	0.03	0.9	289	0.14	6.7
T0920TS455_3-D2.pdb	36.63	0.1	0.02	0.87	210	0.17	7.76
T0920TS455_4-D1.pdb	28.57	0.03	0.01	0.95	306	0.09	8.1
T0920TS455_4-D2.pdb	55.45	0.01	0.06	0.93	225	0.25	9.7
T0920TS455_5-D1.pdb	47.06	0.03	0.05	0.92	295	0.16	7.17
T0920TS455_5-D2.pdb	61.39	0.02	0.02	0.95	230	0.27	9.25
T0920TS464_1-D1.pdb	23.53	0.38	0.16	0.45	146	0.16	64.8
T0920TS464_1-D2.pdb	77.23	0.17	0	0.83	201	0.38	9.82
T0920TS464_2-D1.pdb	72.27	0.27	0.01	0.72	230	0.31	7.71
T0920TS464_2-D2.pdb	79.21	0.11	0	0.89	215	0.37	9.59
T0920TS464_3-D1.pdb	21.01	0.39	0.16	0.45	145	0.14	64.56
T0920TS464_3-D2.pdb	79.21	0.17	0	0.83	200	0.4	9.36
T0920TS464_4-D1.pdb	23.53	0.38	0.16	0.46	148	0.16	65.34
T0920TS464_4-D2.pdb	74.26	0.2	0	0.8	192	0.39	11.53
T0920TS464_5-D1.pdb	68.07	0.27	0	0.73	233	0.29	7.55
T0920TS464_5-D2.pdb	76.24	0.08	0	0.92	221	0.34	10.85
T0920TS479_1-D1.pdb	22.69	0.43	0.14	0.43	138	0.16	70.25
T0920TS479_1-D2.pdb	29.7	0.28	0.17	0.54	131	0.23	71.46
T0920TS479_2-D1.pdb	23.53	0.42	0.13	0.45	143	0.16	54.21
T0920TS479_2-D2.pdb	28.71	0.25	0.12	0.63	151	0.19	63.24
T0920TS479_3-D1.pdb	21.01	0.44	0.15	0.41	132	0.16	51.79
T0920TS479_3-D2.pdb	26.73	0.27	0.18	0.54	131	0.2	61.99
T0920TS479_4-D1.pdb	23.53	0.4	0.11	0.49	157	0.15	61.53
T0920TS479_4-D2.pdb	30.69	0.25	0.16	0.59	142	0.22	62.56
T0920TS479_5-D1.pdb	26.89	0.43	0.14	0.43	139	0.19	51.95
T0920TS479_5-D2.pdb	30.69	0.26	0.24	0.5	120	0.26	67.35
T0921TS005_1-D1.pdb	28	0	0.5	0.5	69	0.41	67.03
T0921TS005_2-D1.pdb	24	0	0.47	0.53	73	0.33	70.47
T0921TS005_5-D1.pdb	26	0	0.49	0.51	70	0.37	68.48
T0921TS016_1-D1.pdb	30	0	0.43	0.57	79	0.38	66.67
T0921TS026_1-D1.pdb	32	0.01	0.52	0.46	64	0.5	62.5
T0921TS026_2-D1.pdb	36	0	0.43	0.57	79	0.46	62.32
T0921TS026_5-D1.pdb	30	0.01	0.46	0.53	73	0.41	60.87
T0921TS048_1-D1.pdb	28	0	0.46	0.54	75	0.37	64.67
T0921TS077_1-D1.pdb	26	0	0.51	0.49	67	0.39	68.12
T0921TS077_2-D1.pdb	18	0	0.49	0.51	70	0.26	67.94

T0921TS077_3-D1.pdb	26	0	0.49	0.51	70	0.37	67.03
T0921TS077_4-D1.pdb	24	0	0.46	0.54	75	0.32	67.94
T0921TS077_5-D1.pdb	24	0	0.51	0.49	68	0.35	67.94
T0921TS166_1-D1.pdb	28	0.03	0.37	0.6	83	0.34	50.54
T0921TS180_1-D1.pdb	48	0	0.38	0.62	86	0.56	17.03
T0921TS180_2-D1.pdb	40	0	0.38	0.62	86	0.47	14.67
T0921TS180_3-D1.pdb	48	0	0.36	0.64	88	0.55	15.4
T0921TS180_4-D1.pdb	52	0	0.25	0.75	103	0.5	14.67
T0921TS180_5-D1.pdb	58	0	0.11	0.89	123	0.47	13.22
T0921TS183_1-D1.pdb	26	0	0.43	0.57	78	0.33	66.12
T0921TS183_2-D1.pdb	26	0.01	0.39	0.59	82	0.32	63.59
T0921TS183_3-D1.pdb	30	0	0.41	0.59	82	0.37	62.5
T0921TS183_4-D1.pdb	24	0	0.4	0.6	83	0.29	63.59
T0921TS183_5-D1.pdb	20	0.01	0.45	0.54	74	0.27	63.59
T0921TS220_1-D1.pdb	36	0	0.46	0.54	74	0.49	69.02
T0921TS220_2-D1.pdb	30	0	0.47	0.53	73	0.41	70.65
T0921TS220_3-D1.pdb	34	0	0.44	0.56	77	0.44	68.84
T0921TS220_4-D1.pdb	30	0	0.46	0.54	74	0.41	68.66
T0921TS220_5-D1.pdb	40	0	0.44	0.56	77	0.52	59.6
T0921TS236_1-D1.pdb	26	0	0.55	0.45	62	0.42	64.86
T0921TS236_2-D1.pdb	28	0.01	0.51	0.48	66	0.42	67.03
T0921TS236_3-D1.pdb	32	0	0.53	0.47	65	0.49	63.23
T0921TS250_1-D1.pdb	22	0	0.53	0.47	65	0.34	67.03
T0921TS250_2-D1.pdb	22	0	0.53	0.47	65	0.34	67.21
T0921TS250_3-D1.pdb	24	0	0.51	0.49	67	0.36	67.21
T0921TS250_4-D1.pdb	26	0	0.51	0.49	67	0.39	67.03
T0921TS250_5-D1.pdb	22	0	0.53	0.47	65	0.34	67.57
T0921TS251_1-D1.pdb	26	0	0.51	0.49	68	0.38	62.5
T0921TS251_2-D1.pdb	28	0	0.54	0.46	63	0.44	67.75
T0921TS251_3-D1.pdb	26	0	0.53	0.47	65	0.4	63.59
T0921TS251_4-D1.pdb	28	0	0.54	0.46	64	0.44	57.61
T0921TS251_5-D1.pdb	30	0	0.5	0.5	69	0.43	62.5
T0921TS258_1-D1.pdb	30	0	0.47	0.53	73	0.41	66.12
T0921TS258_2-D1.pdb	26	0	0.43	0.57	79	0.33	65.76
T0921TS258_3-D1.pdb	30	0	0.43	0.57	78	0.38	66.12
T0921TS258_4-D1.pdb	26	0	0.43	0.57	78	0.33	66.85
T0921TS258_5-D1.pdb	32	0	0.48	0.52	72	0.44	62.68
T0921TS275_1-D1.pdb	26	0	0.51	0.49	67	0.39	66.67
T0921TS275_2-D1.pdb	26	0	0.47	0.53	73	0.36	62.86
T0921TS275_3-D1.pdb	28	0	0.53	0.47	65	0.43	65.76
T0921TS275_4-D1.pdb	26	0	0.48	0.52	72	0.36	67.03
T0921TS275_5-D1.pdb	28	0	0.52	0.48	66	0.42	66.49
T0921TS284_1-D1.pdb	28	0	0.41	0.59	81	0.35	63.04
T0921TS284_2-D1.pdb	34	0	0.5	0.5	69	0.49	64.31
T0921TS284_3-D1.pdb	56	0	0.13	0.87	120	0.47	14.49
T0921TS284_4-D1.pdb	28	0	0.48	0.52	72	0.39	65.58
T0921TS284_5-D1.pdb	26	0.01	0.33	0.66	91	0.29	29.89

T0921TS287_1-D1.pdb	30	0	0.54	0.46	63	0.48	67.75
T0921TS287_2-D1.pdb	26	0	0.53	0.47	65	0.4	66.3
T0921TS287_3-D1.pdb	28	0	0.54	0.46	63	0.44	66.67
T0921TS287_4-D1.pdb	28	0	0.54	0.46	64	0.44	66.85
T0921TS287_5-D1.pdb	24	0.01	0.51	0.48	66	0.36	64.86
T0921TS313_1-D1.pdb	30	0	0.49	0.51	70	0.43	68.66
T0921TS313_2-D1.pdb	28	0	0.47	0.53	73	0.38	69.2
T0921TS313_3-D1.pdb	28	0	0.49	0.51	70	0.4	69.38
T0921TS313_4-D1.pdb	26	0	0.47	0.53	73	0.36	70.11
T0921TS313_5-D1.pdb	30	0	0.49	0.51	70	0.43	69.2
T0921TS321_1-D1.pdb	64	0	0.28	0.72	100	0.64	12.68
T0921TS321_2-D1.pdb	54	0	0.28	0.72	99	0.55	14.49
T0921TS321_3-D1.pdb	50	0	0.3	0.7	97	0.52	12.32
T0921TS321_4-D1.pdb	60	0	0.35	0.65	90	0.67	13.59
T0921TS321_5-D1.pdb	60	0	0.32	0.68	94	0.64	14.13
T0921TS345_1-D1.pdb	28	0	0.51	0.49	67	0.42	63.59
T0921TS345_2-D1.pdb	30	0	0.53	0.47	65	0.46	63.23
T0921TS345_3-D1.pdb	28	0.01	0.53	0.46	63	0.44	65.04
T0921TS345_4-D1.pdb	28	0	0.57	0.43	59	0.47	64.67
T0921TS345_5-D1.pdb	28	0	0.54	0.46	64	0.44	64.67
T0921TS349_1-D1.pdb	28	0	0.5	0.5	69	0.41	66.85
T0921TS357_1-D1.pdb	30	0	0.12	0.88	122	0.25	56.34
T0921TS357_2-D1.pdb	34	0	0.12	0.88	121	0.28	61.59
T0921TS357_3-D1.pdb	40	0	0.07	0.93	128	0.31	57.97
T0921TS357_4-D1.pdb	36	0	0.04	0.96	132	0.27	58.88
T0921TS357_5-D1.pdb	40	0	0.07	0.93	129	0.31	58.88
T0921TS359_1-D1.pdb	28	0.01	0.43	0.56	77	0.36	60.51
T0921TS382_1-D1.pdb	32	0	0.46	0.54	74	0.43	65.58
T0921TS382_2-D1.pdb	32	0	0.5	0.5	69	0.46	66.12
T0921TS382_3-D1.pdb	28	0	0.45	0.55	76	0.37	67.39
T0921TS382_4-D1.pdb	28	0	0.44	0.56	77	0.36	67.21
T0921TS382_5-D1.pdb	30	0	0.41	0.59	81	0.37	66.3
T0921TS405_1-D1.pdb	28	0	0.53	0.47	65	0.43	63.77
T0921TS405_2-D1.pdb	26	0	0.52	0.48	66	0.39	63.95
T0921TS405_3-D1.pdb	28	0.01	0.42	0.57	78	0.36	63.59
T0921TS405_4-D1.pdb	30	0.01	0.46	0.52	72	0.42	64.31
T0921TS405_5-D1.pdb	26	0	0.48	0.52	72	0.36	64.13
T0921TS407_1-D1.pdb	34	0	0.43	0.57	79	0.43	63.04
T0921TS407_2-D1.pdb	34	0	0.4	0.6	83	0.41	62.68
T0921TS407_3-D1.pdb	32	0	0.43	0.57	78	0.41	62.32
T0921TS407_4-D1.pdb	34	0	0.38	0.62	85	0.4	63.77
T0921TS407_5-D1.pdb	30	0	0.4	0.6	83	0.36	63.77
T0921TS421_1-D1.pdb	32	0	0.33	0.67	93	0.34	63.41
T0921TS425_1-D1.pdb	26	0	0.51	0.49	68	0.38	67.75
T0921TS425_2-D1.pdb	26	0	0.49	0.51	70	0.37	68.66
T0921TS425_3-D1.pdb	24	0	0.43	0.57	78	0.31	67.39
T0921TS425_4-D1.pdb	26	0	0.51	0.49	68	0.38	67.03

T0921TS425_5-D1.pdb	28	0	0.46	0.54	74	0.38	66.85
T0921TS432_1-D1.pdb	28	0.01	0.14	0.84	116	0.24	30.43
T0921TS432_2-D1.pdb	42	0	0.16	0.84	116	0.36	19.57
T0921TS432_3-D1.pdb	34	0.01	0.17	0.82	113	0.3	17.75
T0921TS432_4-D1.pdb	36	0.02	0.18	0.8	110	0.33	19.75
T0921TS432_5-D1.pdb	32	0.05	0.23	0.72	99	0.32	17.57
T0921TS434_1-D1.pdb	30	0.07	0	0.93	128	0.23	17.57
T0921TS434_2-D1.pdb	42	0.1	0	0.9	124	0.34	18.84
T0921TS434_3-D1.pdb	30	0.08	0	0.92	127	0.24	15.76
T0921TS434_4-D1.pdb	52	0.09	0	0.91	125	0.42	16.67
T0921TS434_5-D1.pdb	48	0.12	0.08	0.8	111	0.43	18.84
T0921TS444_1-D1.pdb	20	0	0.56	0.44	61	0.33	69.2
T0921TS444_2-D1.pdb	20	0	0.51	0.49	67	0.3	67.39
T0921TS444_3-D1.pdb	16	0	0.49	0.51	70	0.23	65.94
T0921TS444_4-D1.pdb	24	0	0.49	0.51	70	0.34	65.76
T0921TS444_5-D1.pdb	24	0	0.49	0.51	70	0.34	67.94
T0921TS446_1-D1.pdb	26	0	0.46	0.54	74	0.35	69.02
T0921TS446_2-D1.pdb	24	0	0.51	0.49	68	0.35	66.3
T0921TS446_3-D1.pdb	22	0	0.47	0.53	73	0.3	66.3
T0921TS446_4-D1.pdb	22	0.01	0.49	0.49	68	0.32	64.49
T0921TS446_5-D1.pdb	26	0	0.49	0.51	71	0.37	63.77
T0921TS451_1-D1.pdb	56	0.03	0.08	0.89	123	0.46	25.18
T0921TS451_2-D1.pdb	62	0	0.12	0.88	122	0.51	30.07
T0921TS451_3-D1.pdb	54	0.03	0.04	0.93	128	0.42	23.01
T0921TS451_4-D1.pdb	56	0.02	0.09	0.89	123	0.46	24.82
T0921TS451_5-D1.pdb	58	0.03	0.03	0.94	130	0.45	22.83
T0921TS452_1-D1.pdb	38	0	0.46	0.54	74	0.51	64.67
T0921TS452_2-D1.pdb	26	0.02	0.44	0.54	74	0.35	60.69
T0921TS452_3-D1.pdb	30	0.01	0.51	0.47	65	0.46	63.41
T0921TS452_4-D1.pdb	32	0	0.4	0.6	83	0.39	60.69
T0921TS452_5-D1.pdb	30	0	0.49	0.51	71	0.42	62.86
T0921TS455_1-D1.pdb	46	0	0.14	0.86	118	0.39	14.67
T0921TS455_2-D1.pdb	48	0	0.09	0.91	126	0.38	12.14
T0921TS455_3-D1.pdb	52	0.09	0.06	0.85	117	0.44	13.59
T0921TS455_4-D1.pdb	42	0.01	0.03	0.96	132	0.32	12.68
T0921TS455_5-D1.pdb	42	0.02	0.14	0.83	115	0.37	11.96
T0921TS464_1-D1.pdb	28	0	0.35	0.65	90	0.31	67.21
T0921TS464_2-D1.pdb	76	0.07	0	0.93	128	0.59	12.14
T0921TS464_3-D1.pdb	26	0	0.4	0.6	83	0.31	66.85
T0921TS464_4-D1.pdb	26	0	0.39	0.61	84	0.31	67.39
T0921TS464_5-D1.pdb	66	0.04	0.03	0.93	129	0.51	13.22
T0921TS467_1-D1.pdb	26	0.01	0.48	0.51	70	0.37	63.41
T0921TS467_2-D1.pdb	20	0.02	0.45	0.53	73	0.27	66.12
T0921TS467_3-D1.pdb	24	0	0.42	0.58	80	0.3	55.62
T0921TS467_4-D1.pdb	32	0.01	0.43	0.56	77	0.42	53.8
T0921TS467_5-D1.pdb	28	0	0.41	0.59	82	0.34	63.04
T0921TS479_1-D1.pdb	26	0	0.34	0.66	91	0.29	66.85

T0921TS479_2-D1.pdb	20	0.01	0.43	0.55	76	0.26	64.49
T0921TS479_3-D1.pdb	26	0	0.44	0.56	77	0.34	60.69
T0921TS479_4-D1.pdb	22	0	0.3	0.7	96	0.23	63.95
T0921TS479_5-D1.pdb	28	0.01	0.42	0.57	78	0.36	63.41
T0921TS495_1-D1.pdb	20	0	0.53	0.47	65	0.31	67.03
T0928TS005_1-D1.pdb	21.17	0.01	0.37	0.61	210	0.1	53.89
T0928TS005_2-D1.pdb	24.82	0.01	0.39	0.6	206	0.12	53.67
T0928TS005_3-D1.pdb	21.9	0.03	0.38	0.59	201	0.11	55.13
T0928TS005_4-D1.pdb	23.36	0.02	0.36	0.62	212	0.11	58.94
T0928TS005_5-D1.pdb	28.47	0.02	0.31	0.66	228	0.12	22.65
T0928TS016_1-D1.pdb	26.28	0.01	0.25	0.74	254	0.1	38.64
T0928TS026_1-D1.pdb	37.96	0	0.38	0.62	213	0.18	34.02
T0928TS026_3-D1.pdb	27.74	0.01	0.31	0.69	236	0.12	54.91
T0928TS026_4-D1.pdb	22.63	0	0.33	0.67	231	0.1	48.02
T0928TS026_5-D1.pdb	21.17	0	0.34	0.66	226	0.09	45.6
T0928TS048_1-D1.pdb	24.09	0.02	0.42	0.57	194	0.12	51.83
T0928TS077_1-D1.pdb	24.82	0	0.34	0.66	227	0.11	56.67
T0928TS077_2-D1.pdb	26.28	0.01	0.33	0.66	228	0.12	56.6
T0928TS077_3-D1.pdb	26.28	0	0.33	0.67	231	0.11	56.6
T0928TS077_4-D1.pdb	24.82	0	0.36	0.64	218	0.11	55.94
T0928TS077_5-D1.pdb	21.9	0	0.36	0.64	221	0.1	55.35
T0928TS119_1-D1.pdb	25.55	0	0.33	0.67	229	0.11	63.27
T0928TS166_1-D1.pdb	24.82	0	0.38	0.62	212	0.12	57.84
T0928TS180_1-D1.pdb	30.66	0	0.31	0.69	235	0.13	46.85
T0928TS180_2-D1.pdb	18.98	0	0.33	0.67	229	0.08	46.41
T0928TS180_3-D1.pdb	30.66	0	0.34	0.66	225	0.14	46.26
T0928TS180_4-D1.pdb	22.63	0	0.34	0.66	227	0.1	45.38
T0928TS180_5-D1.pdb	44.53	0	0.04	0.96	329	0.14	13.78
T0928TS183_1-D1.pdb	24.09	0.01	0.22	0.77	263	0.09	49.56
T0928TS183_2-D1.pdb	24.09	0	0.27	0.73	251	0.1	49.71
T0928TS183_3-D1.pdb	24.82	0.01	0.22	0.77	265	0.09	46.41
T0928TS183_4-D1.pdb	18.98	0.01	0.29	0.7	241	0.08	57.62
T0928TS183_5-D1.pdb	24.82	0	0.14	0.86	296	0.08	40.03
T0928TS220_1-D1.pdb	27.01	0	0.35	0.65	223	0.12	51.39
T0928TS220_2-D1.pdb	27.01	0	0.34	0.66	228	0.12	57.92
T0928TS220_3-D1.pdb	23.36	0	0.34	0.66	225	0.1	57.77
T0928TS220_4-D1.pdb	21.17	0.01	0.36	0.64	218	0.1	58.94
T0928TS220_5-D1.pdb	20.44	0	0.32	0.68	233	0.09	58.36
T0928TS236_1-D1.pdb	27.74	0	0.37	0.63	215	0.13	47.87
T0928TS236_2-D1.pdb	25.55	0	0.34	0.66	226	0.11	48.61
T0928TS236_3-D1.pdb	29.2	0	0.34	0.66	225	0.13	44.65
T0928TS236_4-D1.pdb	24.82	0.01	0.31	0.68	233	0.11	46.63
T0928TS236_5-D1.pdb	26.28	0.01	0.35	0.64	221	0.12	48.53
T0928TS250_1-D1.pdb	32.85	0	0.34	0.66	227	0.14	38.93
T0928TS250_2-D1.pdb	29.93	0	0.34	0.66	226	0.13	38.78
T0928TS250_3-D1.pdb	30.66	0	0.34	0.66	225	0.14	38.86
T0928TS250_4-D1.pdb	32.12	0.01	0.34	0.65	223	0.14	38.42

T0928TS250_5-D1.pdb	29.2	0	0.34	0.66	225	0.13	39.08
T0928TS251_1-D1.pdb	27.74	0.01	0.36	0.62	214	0.13	40.91
T0928TS251_2-D1.pdb	36.5	0	0.31	0.69	235	0.16	41.86
T0928TS251_3-D1.pdb	37.96	0.01	0.32	0.67	230	0.17	39.08
T0928TS251_4-D1.pdb	37.96	0	0.32	0.68	233	0.16	41.79
T0928TS251_5-D1.pdb	23.36	0	0.38	0.62	212	0.11	42.89
T0928TS275_1-D1.pdb	25.55	0.01	0.3	0.69	238	0.11	46.92
T0928TS275_2-D1.pdb	26.28	0.01	0.32	0.67	230	0.11	49.27
T0928TS275_3-D1.pdb	25.55	0	0.33	0.67	229	0.11	50.37
T0928TS275_4-D1.pdb	24.09	0.01	0.31	0.68	233	0.1	49.93
T0928TS275_5-D1.pdb	25.55	0	0.34	0.66	226	0.11	50.66
T0928TS287_1-D1.pdb	24.82	0.01	0.34	0.65	223	0.11	48.53
T0928TS287_2-D1.pdb	26.28	0.01	0.32	0.68	232	0.11	49.27
T0928TS287_3-D1.pdb	26.28	0.01	0.33	0.66	227	0.12	48.31
T0928TS287_4-D1.pdb	27.74	0.01	0.34	0.66	225	0.12	45.97
T0928TS287_5-D1.pdb	21.17	0	0.25	0.75	256	0.08	46.33
T0928TS313_1-D1.pdb	18.98	0	0.33	0.67	231	0.08	62.39
T0928TS313_2-D1.pdb	18.98	0	0.32	0.68	232	0.08	62.46
T0928TS313_3-D1.pdb	18.25	0	0.33	0.67	231	0.08	62.83
T0928TS313_4-D1.pdb	19.71	0	0.35	0.65	224	0.09	62.68
T0928TS313_5-D1.pdb	21.17	0	0.33	0.67	231	0.09	62.9
T0928TS321_1-D1.pdb	54.74	0	0.36	0.64	218	0.25	8.72
T0928TS321_2-D1.pdb	53.28	0	0.36	0.64	218	0.24	8.43
T0928TS321_3-D1.pdb	51.82	0	0.36	0.64	218	0.24	8.43
T0928TS321_4-D1.pdb	55.47	0	0.36	0.64	218	0.25	8.58
T0928TS321_5-D1.pdb	54.74	0	0.36	0.64	218	0.25	8.58
T0928TS345_1-D1.pdb	22.63	0	0.38	0.62	211	0.11	44.94
T0928TS345_2-D1.pdb	27.01	0.01	0.36	0.64	218	0.12	47.29
T0928TS345_3-D1.pdb	27.74	0	0.36	0.64	218	0.13	45.31
T0928TS345_4-D1.pdb	22.63	0	0.36	0.64	221	0.1	43.48
T0928TS345_5-D1.pdb	25.55	0.01	0.36	0.63	215	0.12	43.84
T0928TS349_1-D1.pdb	23.36	0	0.33	0.67	229	0.1	63.27
T0928TS357_1-D1.pdb	27.01	0.01	0.01	0.98	337	0.08	50.73
T0928TS357_2-D1.pdb	24.82	0.01	0.02	0.97	331	0.07	50.44
T0928TS357_3-D1.pdb	29.2	0.01	0.03	0.96	330	0.09	51.32
T0928TS357_4-D1.pdb	29.93	0.01	0.02	0.97	333	0.09	50.88
T0928TS357_5-D1.pdb	27.74	0	0.05	0.95	327	0.08	51.69
T0928TS382_1-D1.pdb	25.55	0.01	0.25	0.74	254	0.1	51.76
T0928TS382_2-D1.pdb	30.66	0.01	0.2	0.8	273	0.11	49.27
T0928TS382_3-D1.pdb	27.74	0.01	0.29	0.71	243	0.11	52.13
T0928TS382_4-D1.pdb	27.74	0	0.27	0.73	252	0.11	51.54
T0928TS382_5-D1.pdb	28.47	0.01	0.25	0.74	253	0.11	49.93
T0928TS405_1-D1.pdb	22.63	0	0.39	0.61	209	0.11	52.64
T0928TS405_2-D1.pdb	29.2	0.01	0.38	0.62	212	0.14	42.45
T0928TS405_3-D1.pdb	27.74	0.01	0.32	0.67	231	0.12	43.84
T0928TS405_4-D1.pdb	25.55	0.01	0.32	0.67	230	0.11	43.26
T0928TS405_5-D1.pdb	26.28	0	0.36	0.64	218	0.12	41.5

T0928TS407_4-D1.pdb	19.71	0	0.17	0.83	285	0.07	41.35
T0928TS407_5-D1.pdb	23.36	0	0.16	0.84	288	0.08	42.3
T0928TS421_3-D1.pdb	49.64	0.01	0.18	0.81	279	0.18	20.02
T0928TS421_5-D1.pdb	46.72	0.01	0.19	0.8	275	0.17	19.94
T0928TS425_1-D1.pdb	24.82	0	0.34	0.66	225	0.11	55.94
T0928TS425_2-D1.pdb	18.98	0	0.32	0.68	232	0.08	55.06
T0928TS425_3-D1.pdb	24.82	0.01	0.34	0.65	223	0.11	57.04
T0928TS425_4-D1.pdb	26.28	0	0.34	0.66	226	0.12	55.79
T0928TS425_5-D1.pdb	23.36	0	0.37	0.63	217	0.11	55.72
T0928TS432_1-D1.pdb	32.12	0.02	0.11	0.87	298	0.11	12.54
T0928TS432_2-D1.pdb	36.5	0	0.17	0.83	284	0.13	16.35
T0928TS432_3-D1.pdb	35.77	0.01	0.12	0.87	299	0.12	18.04
T0928TS432_4-D1.pdb	39.42	0.01	0.17	0.82	282	0.14	15.84
T0928TS432_5-D1.pdb	42.34	0.02	0.24	0.74	254	0.17	17.82
T0928TS434_1-D1.pdb	40.88	0	0.04	0.96	330	0.12	9.6
T0928TS434_3-D1.pdb	37.96	0	0.04	0.96	330	0.12	8.14
T0928TS434_4-D1.pdb	44.53	0	0.02	0.98	337	0.13	7.99
T0928TS434_5-D1.pdb	42.34	0	0.01	0.99	341	0.12	10.34
T0928TS444_1-D1.pdb	29.2	0.02	0.36	0.62	212	0.14	44.58
T0928TS444_2-D1.pdb	24.09	0	0.34	0.66	225	0.11	42.3
T0928TS444_3-D1.pdb	21.9	0	0.3	0.7	241	0.09	45.23
T0928TS444_4-D1.pdb	22.63	0.01	0.36	0.64	218	0.1	50.73
T0928TS444_5-D1.pdb	27.74	0.01	0.35	0.65	222	0.12	30.5
T0928TS446_1-D1.pdb	25.55	0.03	0.37	0.6	207	0.12	43.77
T0928TS446_2-D1.pdb	24.09	0.03	0.37	0.6	206	0.12	45.6
T0928TS446_4-D1.pdb	23.36	0.01	0.4	0.59	203	0.12	52.93
T0928TS451_1-D1.pdb	48.18	0	0.04	0.96	329	0.15	14.08
T0928TS451_2-D1.pdb	58.39	0	0.1	0.9	309	0.19	12.32
T0928TS451_3-D1.pdb	54.01	0	0.08	0.92	316	0.17	10.78
T0928TS451_4-D1.pdb	53.28	0	0.05	0.95	327	0.16	13.42
T0928TS451_5-D1.pdb	48.91	0	0.1	0.9	309	0.16	16.86
T0928TS452_1-D1.pdb	21.17	0	0.39	0.61	209	0.1	44.65
T0928TS452_2-D1.pdb	29.2	0.02	0.28	0.71	242	0.12	42.59
T0928TS452_5-D1.pdb	29.93	0.01	0.28	0.71	243	0.12	40.32
T0928TS455_1-D1.pdb	48.18	0.01	0.07	0.92	314	0.15	6.01
T0928TS455_3-D1.pdb	49.64	0.01	0.02	0.98	335	0.15	6.08
T0928TS455_4-D1.pdb	47.45	0.01	0.05	0.94	324	0.15	5.94
T0928TS455_5-D1.pdb	51.82	0	0.12	0.88	301	0.17	6.38
T0928TS464_1-D1.pdb	25.55	0	0.25	0.75	256	0.1	45.45
T0928TS464_2-D1.pdb	81.75	0.02	0.02	0.95	327	0.25	4.69
T0928TS464_3-D1.pdb	25.55	0	0.25	0.75	257	0.1	44.72
T0928TS464_4-D1.pdb	27.01	0	0.26	0.74	254	0.11	45.45
T0928TS464_5-D1.pdb	82.48	0.02	0.02	0.96	329	0.25	5.35
T0928TS467_1-D1.pdb	29.2	0.01	0.34	0.66	225	0.13	40.76
T0928TS467_2-D1.pdb	30.66	0.01	0.37	0.62	214	0.14	45.09
T0928TS467_3-D1.pdb	24.82	0.01	0.3	0.69	236	0.11	44.06
T0928TS467_4-D1.pdb	24.09	0.01	0.32	0.67	231	0.1	48.17

T0928TS467_5-D1.pdb	23.36	0.01	0.27	0.72	246	0.09	46.26
T0928TS479_1-D1.pdb	24.82	0	0.28	0.72	246	0.1	50.95
T0928TS479_2-D1.pdb	24.82	0.01	0.21	0.78	266	0.09	51.39
T0928TS479_3-D1.pdb	26.28	0.01	0.21	0.79	270	0.1	46.7
T0928TS479_4-D1.pdb	20.44	0.01	0.2	0.8	273	0.07	60.34
T0928TS479_5-D1.pdb	20.44	0.01	0.21	0.78	269	0.08	41.06
T0943TS005_1-D2.pdb	27.72	0.35	0.17	0.49	219	0.13	68.23
T0943TS005_2-D2.pdb	28.8	0.35	0.16	0.49	220	0.13	68.85
T0943TS005_3-D2.pdb	28.26	0.34	0.15	0.51	230	0.12	67.34
T0943TS005_4-D2.pdb	28.26	0.35	0.17	0.48	215	0.13	67.45
T0943TS005_5-D2.pdb	28.8	0.38	0.17	0.45	204	0.14	64.82
T0943TS016_1-D2.pdb	30.43	0.24	0.15	0.61	273	0.11	41.95
T0943TS026_1-D2.pdb	30.98	0.27	0.12	0.62	278	0.11	52.46
T0943TS026_3-D2.pdb	34.78	0.27	0.05	0.68	308	0.11	6.32
T0943TS026_4-D2.pdb	37.5	0.22	0.11	0.67	301	0.12	41.78
T0943TS026_5-D2.pdb	42.39	0.22	0.06	0.72	325	0.13	6.04
T0943TS048_1-D2.pdb	27.17	0.36	0.15	0.49	223	0.12	56.49
T0943TS077_1-D2.pdb	30.43	0.3	0.16	0.54	244	0.12	46.53
T0943TS077_2-D2.pdb	23.37	0.31	0.14	0.55	248	0.09	46.81
T0943TS077_3-D2.pdb	27.17	0.3	0.15	0.55	250	0.11	47.54
T0943TS077_4-D2.pdb	28.26	0.3	0.15	0.55	247	0.11	43.96
T0943TS077_5-D2.pdb	29.89	0.31	0.16	0.53	241	0.12	45.36
T0943TS119_1-D2.pdb	27.72	0.26	0.05	0.69	311	0.09	36.07
T0943TS180_1-D2.pdb	39.67	0.04	0.18	0.77	349	0.11	5.71
T0943TS180_2-D2.pdb	32.07	0	0.39	0.61	276	0.12	5.09
T0943TS180_3-D2.pdb	58.15	0.35	0.14	0.51	229	0.25	6.94
T0943TS180_4-D2.pdb	45.11	0.01	0.11	0.88	398	0.11	4.36
T0943TS180_5-D2.pdb	30.43	0.28	0	0.72	325	0.09	7.66
T0943TS183_1-D2.pdb	30.98	0.31	0.15	0.54	242	0.13	52.96
T0943TS183_2-D2.pdb	28.8	0.32	0.14	0.54	243	0.12	53.02
T0943TS183_3-D2.pdb	27.17	0.32	0.12	0.56	253	0.11	53.3
T0943TS183_4-D2.pdb	28.26	0.33	0.14	0.53	241	0.12	53.24
T0943TS183_5-D2.pdb	28.26	0.33	0.11	0.56	254	0.11	48.94
T0943TS220_1-D2.pdb	27.17	0.35	0.15	0.5	227	0.12	58.61
T0943TS220_2-D2.pdb	29.35	0.35	0.14	0.5	227	0.13	59.28
T0943TS220_3-D2.pdb	28.26	0.36	0.15	0.49	221	0.13	58.78
T0943TS220_4-D2.pdb	29.89	0.35	0.16	0.49	221	0.14	62.47
T0943TS220_5-D2.pdb	31.52	0.35	0.16	0.49	220	0.14	61.86
T0943TS236_1-D2.pdb	28.8	0.27	0.14	0.58	263	0.11	49.33
T0943TS236_2-D2.pdb	29.89	0.29	0.14	0.56	253	0.12	49.61
T0943TS236_3-D2.pdb	27.72	0.26	0.15	0.59	266	0.1	49.83
T0943TS236_4-D2.pdb	36.41	0.26	0.14	0.6	272	0.13	47.76
T0943TS236_5-D2.pdb	35.33	0.26	0.15	0.59	266	0.13	44.91
T0943TS250_1-D2.pdb	34.78	0.31	0.1	0.58	262	0.13	54.14
T0943TS250_2-D2.pdb	35.33	0.3	0.1	0.6	270	0.13	50.45
T0943TS250_3-D2.pdb	35.87	0.31	0.1	0.59	266	0.13	48.77
T0943TS250_4-D2.pdb	35.33	0.31	0.1	0.59	266	0.13	49.33

T0943TS250_5-D2.pdb	34.78	0.3	0.1	0.59	268	0.13	46.87
T0943TS251_1-D2.pdb	28.8	0.25	0.12	0.63	284	0.1	49.55
T0943TS251_2-D2.pdb	30.43	0.27	0.12	0.6	271	0.11	46.37
T0943TS251_3-D2.pdb	30.98	0.27	0.12	0.61	276	0.11	46.03
T0943TS251_4-D2.pdb	27.72	0.25	0.15	0.6	270	0.1	48.43
T0943TS251_5-D2.pdb	41.85	0.26	0.1	0.64	290	0.14	48.88
T0943TS275_1-D2.pdb	27.72	0.29	0.14	0.57	255	0.11	51.68
T0943TS275_2-D2.pdb	28.8	0.3	0.15	0.55	248	0.12	55.26
T0943TS275_3-D2.pdb	32.61	0.3	0.14	0.55	250	0.13	51.9
T0943TS275_4-D2.pdb	30.43	0.29	0.15	0.55	249	0.12	53.36
T0943TS275_5-D2.pdb	32.61	0.3	0.15	0.55	250	0.13	54.98
T0943TS287_1-D2.pdb	27.17	0.27	0.14	0.58	263	0.1	49.33
T0943TS287_2-D2.pdb	30.98	0.26	0.1	0.64	288	0.11	48.99
T0943TS287_4-D2.pdb	34.24	0.27	0.15	0.58	262	0.13	45.36
T0943TS287_5-D2.pdb	32.61	0.26	0.14	0.6	269	0.12	47.82
T0943TS313_1-D2.pdb	29.35	0.27	0.04	0.7	314	0.09	35.85
T0943TS313_2-D2.pdb	29.35	0.27	0.04	0.69	310	0.09	35.52
T0943TS313_3-D2.pdb	30.98	0.27	0.04	0.7	314	0.1	35.52
T0943TS313_5-D2.pdb	29.35	0.27	0.04	0.7	314	0.09	35.91
T0943TS321_1-D2.pdb	58.7	0.28	0.07	0.65	294	0.2	6.49
T0943TS321_2-D2.pdb	59.78	0.26	0.07	0.68	305	0.2	6.54
T0943TS321_3-D2.pdb	54.89	0.27	0.06	0.67	303	0.18	6.77
T0943TS321_4-D2.pdb	54.89	0.27	0.07	0.66	296	0.19	6.6
T0943TS321_5-D2.pdb	53.26	0.27	0.06	0.67	300	0.18	5.76
T0943TS345_1-D2.pdb	38.04	0.27	0.17	0.55	249	0.15	44.69
T0943TS345_2-D2.pdb	38.04	0.23	0.17	0.61	273	0.14	44.97
T0943TS345_3-D2.pdb	29.35	0.29	0.14	0.57	255	0.12	45.86
T0943TS345_4-D2.pdb	39.13	0.16	0.09	0.75	339	0.12	41.05
T0943TS345_5-D2.pdb	40.22	0.19	0.15	0.66	298	0.13	45.3
T0943TS349_1-D2.pdb	29.89	0.26	0.05	0.69	311	0.1	36.07
T0943TS357_1-D2.pdb	34.78	0.23	0.02	0.75	339	0.1	50.78
T0943TS357_2-D2.pdb	28.26	0.23	0.02	0.75	339	0.08	51.06
T0943TS357_3-D2.pdb	32.07	0.19	0.01	0.8	362	0.09	50.67
T0943TS357_4-D2.pdb	29.35	0.19	0	0.8	362	0.08	51.34
T0943TS357_5-D2.pdb	32.61	0.24	0.01	0.75	340	0.1	51.17
T0943TS382_1-D2.pdb	32.61	0.28	0.16	0.56	251	0.13	39.32
T0943TS382_2-D2.pdb	32.07	0.29	0.17	0.55	247	0.13	38.81
T0943TS382_3-D2.pdb	34.78	0.27	0.14	0.58	263	0.13	35.01
T0943TS382_4-D2.pdb	33.7	0.27	0.16	0.58	260	0.13	38.65
T0943TS382_5-D2.pdb	31.52	0.27	0.17	0.56	253	0.12	43.62
T0943TS405_1-D2.pdb	33.15	0.28	0.12	0.59	268	0.12	49.61
T0943TS405_2-D2.pdb	33.7	0.3	0.13	0.57	256	0.13	53.08
T0943TS405_3-D2.pdb	29.89	0.28	0.14	0.58	261	0.11	46.81
T0943TS407_1-D2.pdb	38.59	0.28	0.06	0.66	298	0.13	44.74
T0943TS407_2-D2.pdb	37.5	0.27	0.05	0.68	306	0.12	43.4
T0943TS407_3-D2.pdb	32.61	0.34	0.06	0.6	270	0.12	43.29
T0943TS407_4-D2.pdb	36.41	0.29	0.08	0.63	283	0.13	43.51

T0943TS407_5-D2.pdb	35.87	0.23	0.07	0.7	317	0.11	43.74
T0943TS421_2-D2.pdb	28.8	0.25	0.09	0.66	298	0.1	48.94
T0943TS425_1-D2.pdb	26.63	0.31	0.15	0.54	244	0.11	47.88
T0943TS425_2-D2.pdb	28.8	0.29	0.14	0.56	254	0.11	45.8
T0943TS425_3-D2.pdb	29.89	0.31	0.16	0.53	239	0.13	45.75
T0943TS425_4-D2.pdb	28.26	0.28	0.16	0.55	250	0.11	46.59
T0943TS425_5-D2.pdb	28.26	0.28	0.16	0.55	250	0.11	46.7
T0943TS432_1-D2.pdb	50.54	0.26	0.02	0.72	323	0.16	7.77
T0943TS432_2-D2.pdb	54.89	0.27	0.06	0.67	302	0.18	10.07
T0943TS432_3-D2.pdb	52.17	0.25	0.02	0.73	327	0.16	9.56
T0943TS432_4-D2.pdb	56.52	0.24	0.03	0.73	328	0.17	10.68
T0943TS432_5-D2.pdb	44.02	0.28	0.02	0.7	315	0.14	12.3
T0943TS434_1-D2.pdb	67.39	0.34	0	0.66	296	0.23	6.54
T0943TS434_2-D2.pdb	67.93	0.35	0	0.65	294	0.23	5.71
T0943TS434_3-D2.pdb	81.52	0.36	0	0.64	289	0.28	6.38
T0943TS434_4-D2.pdb	78.8	0.36	0	0.64	287	0.27	5.82
T0943TS434_5-D2.pdb	76.63	0.36	0	0.64	290	0.26	6.6
T0943TS444_1-D2.pdb	32.07	0.34	0.12	0.54	242	0.13	43.9
T0943TS444_2-D2.pdb	30.98	0.33	0.12	0.55	246	0.13	43.51
T0943TS444_3-D2.pdb	33.7	0.34	0.11	0.55	249	0.14	43.68
T0943TS444_4-D2.pdb	29.35	0.33	0.11	0.56	252	0.12	44.46
T0943TS444_5-D2.pdb	30.43	0.34	0.12	0.54	244	0.12	43.85
T0943TS446_1-D2.pdb	29.35	0.33	0.14	0.54	242	0.12	47.59
T0943TS446_2-D2.pdb	28.8	0.32	0.15	0.53	239	0.12	47.26
T0943TS446_5-D2.pdb	29.89	0.33	0.15	0.53	237	0.13	48.27
T0943TS451_1-D2.pdb	41.3	0.3	0.03	0.68	305	0.14	20.58
T0943TS451_2-D2.pdb	39.13	0.27	0.03	0.7	316	0.12	18.07
T0943TS451_3-D2.pdb	41.3	0.28	0.02	0.7	314	0.13	25.28
T0943TS451_4-D2.pdb	46.2	0.31	0.03	0.66	298	0.16	16.44
T0943TS451_5-D2.pdb	43.48	0.29	0.01	0.7	314	0.14	19.24
T0943TS452_1-D2.pdb	27.72	0.29	0.14	0.57	258	0.11	39.71
T0943TS452_2-D2.pdb	40.22	0.2	0.13	0.67	301	0.13	45.97
T0943TS452_3-D2.pdb	38.04	0.24	0.02	0.74	335	0.11	6.49
T0943TS452_4-D2.pdb	44.02	0.2	0.04	0.76	341	0.13	6.94
T0943TS452_5-D2.pdb	47.28	0.34	0.09	0.57	256	0.18	6.49
T0943TS455_1-D2.pdb	41.85	0.22	0	0.77	349	0.12	5.42
T0943TS455_2-D2.pdb	44.02	0.1	0	0.89	403	0.11	5.31
T0943TS455_3-D2.pdb	44.02	0.1	0.01	0.89	400	0.11	5.26
T0943TS455_4-D2.pdb	48.37	0.09	0	0.91	411	0.12	5.48
T0943TS455_5-D2.pdb	32.61	0.14	0.04	0.82	372	0.09	4.98
T0943TS464_1-D2.pdb	29.89	0.29	0.13	0.58	261	0.11	62.75
T0943TS464_2-D2.pdb	79.89	0.14	0	0.86	388	0.21	4.75
T0943TS464_3-D2.pdb	31.52	0.29	0.14	0.57	256	0.12	63.09
T0943TS464_4-D2.pdb	30.43	0.29	0.13	0.58	261	0.12	61.35
T0943TS464_5-D2.pdb	77.17	0.25	0	0.75	336	0.23	5.31
T0943TS467_1-D2.pdb	42.39	0.5	0	0.5	226	0.19	6.6
T0943TS467_2-D2.pdb	48.37	0.47	0.01	0.52	235	0.21	6.43

T0943TS467_3-D2.pdb	41.3	0.53	0	0.47	213	0.19	6.32
T0943TS467_4-D2.pdb	44.57	0.5	0	0.5	226	0.2	5.82
T0943TS467_5-D2.pdb	43.48	0.54	0	0.46	207	0.21	6.43
T0943TS479_1-D2.pdb	28.26	0.32	0.12	0.56	253	0.11	53.02
T0943TS479_2-D2.pdb	28.26	0.33	0.12	0.54	245	0.12	52.8
T0943TS479_3-D2.pdb	29.35	0.33	0.12	0.55	247	0.12	53.58
T0943TS479_4-D2.pdb	27.72	0.33	0.12	0.56	251	0.11	53.36
T0943TS479_5-D2.pdb	28.26	0.33	0.14	0.53	241	0.12	57.22
T0944TS001_1-D1.pdb	41.43	0.11	0.11	0.78	197	0.21	30.93
T0944TS004_1-D1.pdb	20	0.21	0.18	0.61	155	0.13	73.72
T0944TS004_2-D1.pdb	22.86	0.2	0.2	0.6	152	0.15	70.16
T0944TS004_3-D1.pdb	22.86	0.19	0.21	0.6	152	0.15	74.7
T0944TS004_4-D1.pdb	18.57	0.23	0.22	0.56	141	0.13	72.63
T0944TS004_5-D1.pdb	20	0.16	0.19	0.65	165	0.12	57.12
T0944TS005_1-D1.pdb	18.57	0.19	0.23	0.58	147	0.13	66.8
T0944TS005_2-D1.pdb	21.43	0.16	0.24	0.6	151	0.14	73.72
T0944TS005_4-D1.pdb	17.14	0.19	0.24	0.57	143	0.12	73.72
T0944TS005_5-D1.pdb	21.43	0.17	0.25	0.59	149	0.14	74.01
T0944TS011_1-D1.pdb	24.29	0.25	0.26	0.49	124	0.2	73.72
T0944TS011_2-D1.pdb	25.71	0.19	0.22	0.58	148	0.17	73.32
T0944TS011_3-D1.pdb	24.29	0.19	0.25	0.56	142	0.17	74.7
T0944TS011_4-D1.pdb	22.86	0.21	0.25	0.54	137	0.17	74.31
T0944TS011_5-D1.pdb	20	0.18	0.25	0.57	143	0.14	74.7
T0944TS016_1-D1.pdb	21.43	0.19	0.23	0.58	147	0.15	66.11
T0944TS017_1-D1.pdb	22.86	0.16	0.26	0.58	146	0.16	73.22
T0944TS017_2-D1.pdb	22.86	0.16	0.26	0.58	146	0.16	73.12
T0944TS017_5-D1.pdb	20	0.19	0.26	0.55	139	0.14	72.83
T0944TS019_1-D1.pdb	21.43	0.21	0.27	0.53	133	0.16	67.79
T0944TS022_1-D1.pdb	37.14	0.38	0	0.62	157	0.24	7.81
T0944TS022_2-D1.pdb	45.71	0.38	0	0.62	157	0.29	7.02
T0944TS022_3-D1.pdb	47.14	0.38	0	0.62	157	0.3	7.12
T0944TS022_4-D1.pdb	47.14	0.25	0	0.75	191	0.25	7.21
T0944TS022_5-D1.pdb	42.86	0.37	0	0.63	159	0.27	7.61
T0944TS023_1-D1.pdb	21.43	0.16	0.21	0.63	160	0.13	70.16
T0944TS023_2-D1.pdb	20	0.15	0.2	0.65	165	0.12	57.71
T0944TS023_3-D1.pdb	17.14	0.15	0.22	0.63	159	0.11	70.36
T0944TS023_4-D1.pdb	21.43	0.15	0.2	0.65	164	0.13	57.81
T0944TS023_5-D1.pdb	21.43	0.17	0.25	0.58	147	0.15	64.72
T0944TS026_1-D1.pdb	24.29	0.09	0.17	0.74	187	0.13	54.05
T0944TS026_3-D1.pdb	24.29	0.17	0.17	0.66	167	0.15	66.11
T0944TS026_4-D1.pdb	30	0.1	0.23	0.66	168	0.18	50.49
T0944TS026_5-D1.pdb	24.29	0.12	0.17	0.7	178	0.14	60.57
T0944TS028_1-D1.pdb	15.71	0.15	0.21	0.63	160	0.1	69.66
T0944TS040_1-D1.pdb	51.43	0.14	0.02	0.84	212	0.24	9.68
T0944TS040_2-D1.pdb	57.14	0.13	0.02	0.85	216	0.26	10.87
T0944TS040_3-D1.pdb	50	0.11	0.02	0.87	219	0.23	8.3
T0944TS040_4-D1.pdb	52.86	0.11	0.03	0.86	218	0.24	9.39

T0944TS040_5-D1.pdb	40	0.1	0.02	0.88	223	0.18	10.97
T0944TS042_1-D1.pdb	20	0.19	0.23	0.58	147	0.14	66.6
T0944TS042_2-D1.pdb	24.29	0.17	0.26	0.57	143	0.17	73.02
T0944TS042_3-D1.pdb	22.86	0.13	0.22	0.65	164	0.14	71.64
T0944TS042_4-D1.pdb	22.86	0.16	0.25	0.59	149	0.15	71.25
T0944TS042_5-D1.pdb	18.57	0.19	0.24	0.57	145	0.13	73.52
T0944TS048_1-D1.pdb	24.29	0.22	0.22	0.56	142	0.17	70.26
T0944TS060_1-D1.pdb	20	0.17	0.25	0.58	146	0.14	71.74
T0944TS060_2-D1.pdb	17.14	0.19	0.21	0.6	153	0.11	71.15
T0944TS060_3-D1.pdb	20	0.16	0.2	0.64	162	0.12	70.95
T0944TS060_4-D1.pdb	25.71	0.18	0.19	0.63	160	0.16	71.15
T0944TS060_5-D1.pdb	21.43	0.21	0.19	0.59	150	0.14	70.06
T0944TS064_1-D1.pdb	18.57	0.23	0.24	0.53	134	0.14	73.91
T0944TS067_5-D1.pdb	17.14	0.23	0.23	0.54	136	0.13	65.81
T0944TS073_1-D1.pdb	20	0.18	0.24	0.58	147	0.14	70.16
T0944TS073_2-D1.pdb	15.71	0.14	0.26	0.6	151	0.1	70.06
T0944TS073_3-D1.pdb	18.57	0.15	0.22	0.63	160	0.12	69.76
T0944TS073_4-D1.pdb	21.43	0.19	0.22	0.59	149	0.14	70.06
T0944TS073_5-D1.pdb	20	0.16	0.26	0.58	146	0.14	73.42
T0944TS077_1-D1.pdb	24.29	0.15	0.26	0.58	148	0.16	71.34
T0944TS077_2-D1.pdb	25.71	0.18	0.25	0.57	145	0.18	71.64
T0944TS077_3-D1.pdb	21.43	0.1	0.26	0.64	161	0.13	69.56
T0944TS077_4-D1.pdb	21.43	0.17	0.26	0.58	146	0.15	72.73
T0944TS077_5-D1.pdb	22.86	0.11	0.23	0.66	166	0.14	70.65
T0944TS079_3-D1.pdb	20	0.1	0.23	0.67	170	0.12	70.06
T0944TS079_4-D1.pdb	20	0.2	0.22	0.58	147	0.14	69.47
T0944TS079_5-D1.pdb	24.29	0.18	0.28	0.54	136	0.18	70.75
T0944TS083_1-D1.pdb	24.29	0.14	0.25	0.61	154	0.16	14.92
T0944TS083_2-D1.pdb	25.71	0.19	0.21	0.6	151	0.17	13.14
T0944TS083_3-D1.pdb	20	0.21	0.19	0.6	152	0.13	12.45
T0944TS083_4-D1.pdb	21.43	0.18	0.16	0.66	167	0.13	11.46
T0944TS083_5-D1.pdb	22.86	0.22	0.18	0.6	152	0.15	16.9
T0944TS102_1-D1.pdb	28.57	0.07	0.09	0.84	213	0.13	65.81
T0944TS102_2-D1.pdb	28.57	0.05	0.05	0.9	227	0.13	66.01
T0944TS102_3-D1.pdb	20	0.09	0.04	0.86	218	0.09	66.7
T0944TS102_4-D1.pdb	21.43	0.06	0.06	0.88	223	0.1	66.7
T0944TS102_5-D1.pdb	24.29	0.08	0.08	0.85	214	0.11	66.4
T0944TS114_3-D1.pdb	21.43	0.14	0.26	0.6	151	0.14	74.11
T0944TS114_4-D1.pdb	17.14	0.17	0.2	0.64	161	0.11	69.56
T0944TS119_1-D1.pdb	24.29	0.14	0.15	0.7	178	0.14	65.51
T0944TS126_1-D1.pdb	37.14	0.21	0.08	0.71	179	0.21	13.54
T0944TS126_2-D1.pdb	38.57	0.21	0.1	0.7	176	0.22	16.7
T0944TS126_3-D1.pdb	38.57	0.21	0.09	0.7	177	0.22	16.9
T0944TS126_4-D1.pdb	44.29	0.22	0.1	0.68	172	0.26	11.36
T0944TS126_5-D1.pdb	31.43	0.18	0.12	0.7	178	0.18	20.26
T0944TS145_1-D1.pdb	18.57	0.14	0.1	0.76	192	0.1	10.97
T0944TS145_2-D1.pdb	14.29	0.2	0.11	0.69	175	0.08	10.28

T0944TS145_3-D1.pdb	10	0.11	0.11	0.78	197	0.05	10.67
T0944TS145_4-D1.pdb	15.71	0.17	0.1	0.73	185	0.08	9.98
T0944TS145_5-D1.pdb	11.43	0.17	0.08	0.75	190	0.06	10.97
T0944TS171_1-D1.pdb	18.57	0.11	0.2	0.69	174	0.11	50
T0944TS171_2-D1.pdb	24.29	0.17	0.17	0.67	169	0.14	50.1
T0944TS171_3-D1.pdb	30	0.15	0.11	0.74	188	0.16	33.3
T0944TS171_4-D1.pdb	22.86	0.18	0.19	0.64	161	0.14	57.41
T0944TS171_5-D1.pdb	27.14	0.18	0.19	0.64	161	0.17	36.76
T0944TS173_1-D1.pdb	17.14	0.17	0.24	0.6	151	0.11	65.61
T0944TS173_2-D1.pdb	11.43	0.18	0.24	0.58	147	0.08	69.76
T0944TS173_3-D1.pdb	11.43	0.19	0.23	0.58	148	0.08	69.07
T0944TS173_4-D1.pdb	12.86	0.16	0.22	0.62	158	0.08	68.68
T0944TS173_5-D1.pdb	12.86	0.19	0.25	0.57	144	0.09	70.16
T0944TS179_2-D1.pdb	18.57	0.23	0.23	0.54	136	0.14	66.8
T0944TS179_4-D1.pdb	18.57	0.23	0.22	0.56	141	0.13	68.68
T0944TS180_1-D1.pdb	22.86	0.2	0.14	0.66	167	0.14	50.59
T0944TS180_2-D1.pdb	21.43	0.16	0.17	0.68	171	0.13	50.4
T0944TS180_3-D1.pdb	27.14	0.18	0.1	0.72	182	0.15	42.98
T0944TS180_4-D1.pdb	32.86	0.11	0.14	0.75	191	0.17	38.04
T0944TS180_5-D1.pdb	30	0.08	0.02	0.91	230	0.13	11.56
T0944TS182_1-D1.pdb	14.29	0.09	0.04	0.87	221	0.06	12.85
T0944TS182_2-D1.pdb	17.14	0.13	0.09	0.78	197	0.09	27.17
T0944TS182_3-D1.pdb	17.14	0.09	0.1	0.81	206	0.08	10.97
T0944TS182_4-D1.pdb	20	0.12	0.06	0.82	208	0.1	17.19
T0944TS182_5-D1.pdb	18.57	0.06	0.1	0.83	211	0.09	13.83
T0944TS183_1-D1.pdb	20	0.24	0.19	0.57	145	0.14	70.26
T0944TS183_3-D1.pdb	20	0.18	0.2	0.62	158	0.13	70.45
T0944TS183_4-D1.pdb	18.57	0.19	0.17	0.65	164	0.11	68.48
T0944TS183_5-D1.pdb	17.14	0.19	0.19	0.62	158	0.11	61.27
T0944TS187_1-D1.pdb	20	0.21	0.21	0.58	147	0.14	65.32
T0944TS187_2-D1.pdb	21.43	0.19	0.21	0.6	153	0.14	65.02
T0944TS187_3-D1.pdb	21.43	0.22	0.21	0.57	143	0.15	66.7
T0944TS187_4-D1.pdb	20	0.21	0.21	0.58	146	0.14	63.93
T0944TS187_5-D1.pdb	17.14	0.2	0.24	0.56	142	0.12	63.14
T0944TS188_2-D1.pdb	22.86	0.17	0.17	0.66	167	0.14	66.01
T0944TS203_1-D1.pdb	22.86	0.17	0.26	0.57	143	0.16	72.92
T0944TS203_4-D1.pdb	24.29	0.16	0.23	0.61	154	0.16	73.42
T0944TS203_5-D1.pdb	18.57	0.22	0.22	0.56	142	0.13	67
T0944TS207_1-D1.pdb	20	0.14	0.13	0.74	186	0.11	62.35
T0944TS207_2-D1.pdb	27.14	0.17	0.15	0.68	171	0.16	51.28
T0944TS207_3-D1.pdb	17.14	0.16	0.13	0.71	179	0.1	54.35
T0944TS207_4-D1.pdb	38.57	0.21	0.06	0.74	187	0.21	42.79
T0944TS207_5-D1.pdb	48.57	0.23	0	0.77	195	0.25	7.61
T0944TS220_3-D1.pdb	17.14	0.17	0.25	0.58	146	0.12	71.54
T0944TS220_4-D1.pdb	24.29	0.2	0.22	0.58	147	0.17	73.62
T0944TS230_1-D1.pdb	18.57	0.19	0.25	0.56	142	0.13	70.45
T0944TS230_2-D1.pdb	20	0.2	0.23	0.58	146	0.14	68.38

T0944TS230_3-D1.pdb	15.71	0.21	0.22	0.57	145	0.11	71.84
T0944TS230_4-D1.pdb	20	0.2	0.22	0.58	147	0.14	69.07
T0944TS230_5-D1.pdb	18.57	0.21	0.25	0.55	138	0.13	70.55
T0944TS236_1-D1.pdb	20	0.17	0.25	0.58	146	0.14	69.96
T0944TS236_2-D1.pdb	18.57	0.16	0.23	0.6	153	0.12	69.07
T0944TS236_3-D1.pdb	21.43	0.19	0.22	0.59	149	0.14	68.18
T0944TS236_4-D1.pdb	25.71	0.19	0.21	0.59	150	0.17	67.39
T0944TS236_5-D1.pdb	24.29	0.14	0.25	0.61	154	0.16	69.27
T0944TS239_1-D1.pdb	22.86	0.19	0.25	0.57	144	0.16	71.74
T0944TS239_2-D1.pdb	17.14	0.19	0.26	0.55	138	0.12	72.73
T0944TS239_3-D1.pdb	18.57	0.19	0.25	0.57	144	0.13	75.79
T0944TS239_4-D1.pdb	21.43	0.17	0.24	0.59	150	0.14	75.39
T0944TS239_5-D1.pdb	22.86	0.17	0.25	0.59	149	0.15	73.32
T0944TS243_1-D1.pdb	18.57	0.2	0.26	0.53	135	0.14	73.72
T0944TS243_2-D1.pdb	20	0.2	0.26	0.54	136	0.15	73.42
T0944TS243_4-D1.pdb	18.57	0.2	0.26	0.53	135	0.14	74.51
T0944TS243_5-D1.pdb	20	0.19	0.27	0.54	136	0.15	73.62
T0944TS250_3-D1.pdb	22.86	0.14	0.2	0.66	167	0.14	58.4
T0944TS250_4-D1.pdb	22.86	0.15	0.2	0.65	164	0.14	57.91
T0944TS250_5-D1.pdb	22.86	0.14	0.2	0.66	166	0.14	58
T0944TS251_1-D1.pdb	24.29	0.15	0.22	0.63	159	0.15	56.52
T0944TS251_2-D1.pdb	25.71	0.15	0.2	0.65	164	0.16	57.12
T0944TS251_3-D1.pdb	24.29	0.17	0.21	0.62	156	0.16	57.61
T0944TS251_4-D1.pdb	27.14	0.15	0.2	0.64	163	0.17	57.31
T0944TS251_5-D1.pdb	27.14	0.17	0.23	0.61	154	0.18	56.23
T0944TS252_1-D1.pdb	15.71	0.16	0.24	0.6	153	0.1	72.63
T0944TS252_2-D1.pdb	21.43	0.17	0.23	0.6	151	0.14	69.47
T0944TS252_3-D1.pdb	18.57	0.19	0.22	0.59	150	0.12	68.68
T0944TS252_4-D1.pdb	14.29	0.17	0.23	0.61	154	0.09	73.42
T0944TS252_5-D1.pdb	18.57	0.17	0.24	0.59	149	0.12	72.04
T0944TS264_1-D1.pdb	52.86	0.09	0.04	0.87	221	0.24	9.29
T0944TS264_2-D1.pdb	51.43	0.1	0.04	0.86	218	0.24	10.38
T0944TS264_3-D1.pdb	44.29	0.09	0.05	0.86	218	0.2	8.89
T0944TS264_4-D1.pdb	47.14	0.08	0.05	0.87	220	0.21	9.78
T0944TS264_5-D1.pdb	57.14	0.09	0.04	0.87	221	0.26	9.19
T0944TS275_1-D1.pdb	28.57	0.18	0.25	0.58	146	0.2	68.28
T0944TS275_2-D1.pdb	27.14	0.19	0.21	0.6	152	0.18	68.08
T0944TS275_3-D1.pdb	27.14	0.19	0.24	0.57	145	0.19	69.37
T0944TS275_4-D1.pdb	30	0.15	0.24	0.61	154	0.19	68.68
T0944TS275_5-D1.pdb	27.14	0.19	0.22	0.59	149	0.18	69.07
T0944TS287_1-D1.pdb	24.29	0.16	0.25	0.59	149	0.16	69.47
T0944TS287_2-D1.pdb	21.43	0.17	0.23	0.6	152	0.14	69.07
T0944TS287_3-D1.pdb	21.43	0.19	0.22	0.59	149	0.14	68.38
T0944TS287_4-D1.pdb	21.43	0.19	0.24	0.57	144	0.15	70.75
T0944TS287_5-D1.pdb	20	0.17	0.25	0.58	148	0.14	70.06
T0944TS295_2-D1.pdb	21.43	0.21	0.21	0.58	148	0.14	62.65
T0944TS295_3-D1.pdb	18.57	0.2	0.22	0.58	148	0.13	62.35

T0944TS295_4-D1.pdb	21.43	0.21	0.22	0.57	143	0.15	65.81
T0944TS295_5-D1.pdb	17.14	0.2	0.23	0.57	145	0.12	66.01
T0944TS303_1-D1.pdb	21.43	0.17	0.26	0.56	142	0.15	72.92
T0944TS303_2-D1.pdb	27.14	0.16	0.27	0.57	145	0.19	74.21
T0944TS303_3-D1.pdb	21.43	0.17	0.26	0.56	142	0.15	67.09
T0944TS303_4-D1.pdb	24.29	0.19	0.25	0.56	141	0.17	74.01
T0944TS303_5-D1.pdb	22.86	0.16	0.21	0.62	158	0.14	69.96
T0944TS313_1-D1.pdb	21.43	0.13	0.14	0.73	184	0.12	65.02
T0944TS313_2-D1.pdb	21.43	0.13	0.14	0.74	186	0.12	64.92
T0944TS313_3-D1.pdb	22.86	0.14	0.14	0.72	182	0.13	65.22
T0944TS313_4-D1.pdb	21.43	0.13	0.14	0.74	186	0.12	65.61
T0944TS313_5-D1.pdb	20	0.13	0.14	0.73	184	0.11	65.71
T0944TS320_4-D1.pdb	21.43	0.17	0.25	0.58	148	0.14	70.85
T0944TS321_1-D1.pdb	64.29	0.11	0.09	0.8	202	0.32	9.68
T0944TS321_2-D1.pdb	71.43	0.11	0.09	0.8	202	0.35	9.49
T0944TS321_3-D1.pdb	68.57	0.1	0.09	0.8	203	0.34	9.88
T0944TS321_4-D1.pdb	68.57	0.11	0.11	0.78	197	0.35	9.49
T0944TS321_5-D1.pdb	74.29	0.1	0.11	0.79	200	0.37	9.09
T0944TS324_1-D1.pdb	22.86	0.2	0.25	0.55	140	0.16	73.91
T0944TS324_2-D1.pdb	20	0.19	0.26	0.55	140	0.14	75.1
T0944TS324_3-D1.pdb	22.86	0.21	0.26	0.53	135	0.17	76.28
T0944TS324_4-D1.pdb	18.57	0.17	0.26	0.57	143	0.13	74.31
T0944TS324_5-D1.pdb	22.86	0.2	0.26	0.54	137	0.17	74.11
T0944TS325_1-D1.pdb	17.14	0.18	0.25	0.58	146	0.12	76.19
T0944TS325_2-D1.pdb	20	0.15	0.23	0.62	156	0.13	75.99
T0944TS325_3-D1.pdb	14.29	0.19	0.22	0.59	150	0.1	68.18
T0944TS325_4-D1.pdb	20	0.19	0.22	0.59	150	0.13	66.6
T0944TS325_5-D1.pdb	17.14	0.2	0.23	0.57	145	0.12	67.39
T0944TS330_1-D1.pdb	21.43	0.15	0.25	0.6	152	0.14	67.79
T0944TS330_2-D1.pdb	21.43	0.13	0.27	0.6	153	0.14	55.24
T0944TS330_3-D1.pdb	18.57	0.18	0.17	0.65	165	0.11	47.92
T0944TS330_4-D1.pdb	25.71	0.21	0.23	0.56	141	0.18	63.14
T0944TS330_5-D1.pdb	34.29	0.17	0.19	0.63	160	0.21	11.36
T0944TS345_1-D1.pdb	25.71	0.19	0.23	0.57	145	0.18	68.38
T0944TS345_2-D1.pdb	27.14	0.19	0.22	0.59	150	0.18	67.79
T0944TS345_3-D1.pdb	27.14	0.19	0.21	0.6	152	0.18	65.02
T0944TS345_4-D1.pdb	27.14	0.18	0.22	0.6	151	0.18	64.92
T0944TS345_5-D1.pdb	22.86	0.14	0.24	0.62	156	0.15	60.67
T0944TS349_1-D1.pdb	22.86	0.14	0.15	0.7	178	0.13	65.51
T0944TS359_1-D1.pdb	24.29	0.1	0.23	0.67	169	0.14	67
T0944TS359_2-D1.pdb	22.86	0.13	0.25	0.61	155	0.15	68.38
T0944TS359_3-D1.pdb	22.86	0.15	0.25	0.61	154	0.15	69.76
T0944TS359_5-D1.pdb	24.29	0.16	0.21	0.63	160	0.15	69.07
T0944TS363_5-D1.pdb	21.43	0.21	0.23	0.56	142	0.15	68.58
T0944TS382_1-D1.pdb	24.29	0.19	0.22	0.59	149	0.16	68.78
T0944TS382_2-D1.pdb	24.29	0.19	0.24	0.57	145	0.17	68.08
T0944TS382_3-D1.pdb	20	0.19	0.2	0.61	155	0.13	68.58

T0944TS382_4-D1.pdb	22.86	0.19	0.21	0.6	152	0.15	68.87
T0944TS382_5-D1.pdb	25.71	0.17	0.21	0.62	157	0.16	68.48
T0944TS384_1-D1.pdb	24.29	0.23	0.22	0.55	139	0.17	73.72
T0944TS384_2-D1.pdb	17.14	0.2	0.23	0.57	144	0.12	67.09
T0944TS384_3-D1.pdb	21.43	0.23	0.23	0.54	137	0.16	67
T0944TS384_4-D1.pdb	17.14	0.18	0.26	0.56	141	0.12	73.12
T0944TS384_5-D1.pdb	20	0.19	0.23	0.58	146	0.14	73.81
T0944TS393_1-D1.pdb	21.43	0.18	0.27	0.55	140	0.15	72.13
T0944TS393_2-D1.pdb	22.86	0.16	0.25	0.59	150	0.15	73.32
T0944TS393_3-D1.pdb	20	0.22	0.26	0.52	131	0.15	65.81
T0944TS393_4-D1.pdb	21.43	0.17	0.21	0.62	156	0.14	71.64
T0944TS393_5-D1.pdb	21.43	0.16	0.21	0.62	158	0.14	69.76
T0944TS396_1-D1.pdb	20	0.24	0.19	0.57	145	0.14	69.96
T0944TS396_2-D1.pdb	17.14	0.17	0.22	0.61	155	0.11	69.66
T0944TS396_3-D1.pdb	20	0.21	0.19	0.6	153	0.13	69.17
T0944TS396_4-D1.pdb	15.71	0.18	0.2	0.62	157	0.1	67.49
T0944TS396_5-D1.pdb	20	0.2	0.23	0.57	145	0.14	69.27
T0944TS399_1-D1.pdb	54.29	0.14	0	0.86	218	0.25	10.28
T0944TS399_2-D1.pdb	60	0.14	0	0.86	218	0.28	10.38
T0944TS399_3-D1.pdb	52.86	0.14	0	0.86	218	0.24	10.97
T0944TS399_4-D1.pdb	44.29	0.14	0	0.86	218	0.2	10.47
T0944TS399_5-D1.pdb	48.57	0.14	0	0.86	218	0.22	10.28
T0944TS405_3-D1.pdb	21.43	0.17	0.22	0.61	155	0.14	70.16
T0944TS405_5-D1.pdb	20	0.17	0.23	0.6	153	0.13	70.55
T0944TS407_1-D1.pdb	22.86	0.11	0.14	0.75	191	0.12	62.65
T0944TS407_2-D1.pdb	22.86	0.16	0.13	0.71	179	0.13	63.54
T0944TS407_3-D1.pdb	28.57	0.17	0.14	0.7	176	0.16	63.04
T0944TS407_4-D1.pdb	25.71	0.15	0.13	0.72	182	0.14	64.23
T0944TS407_5-D1.pdb	21.43	0.2	0.16	0.64	162	0.13	61.86
T0944TS411_1-D1.pdb	20	0.18	0.26	0.55	140	0.14	71.34
T0944TS411_2-D1.pdb	20	0.18	0.23	0.58	148	0.14	66.9
T0944TS411_3-D1.pdb	24.29	0.16	0.25	0.59	149	0.16	74.01
T0944TS411_4-D1.pdb	24.29	0.19	0.21	0.6	153	0.16	68.38
T0944TS411_5-D1.pdb	18.57	0.16	0.24	0.6	152	0.12	73.72
T0944TS421_1-D1.pdb	27.14	0.11	0.15	0.74	188	0.14	62.85
T0944TS425_1-D1.pdb	20	0.11	0.23	0.66	167	0.12	68.78
T0944TS425_2-D1.pdb	22.86	0.14	0.21	0.65	165	0.14	70.45
T0944TS425_3-D1.pdb	20	0.17	0.26	0.56	142	0.14	72.33
T0944TS425_4-D1.pdb	21.43	0.14	0.25	0.61	155	0.14	69.27
T0944TS432_1-D1.pdb	25.71	0.16	0.09	0.74	188	0.14	30.63
T0944TS432_2-D1.pdb	28.57	0.13	0.17	0.7	176	0.16	35.67
T0944TS432_3-D1.pdb	31.43	0.16	0.08	0.76	193	0.16	23.12
T0944TS432_4-D1.pdb	30	0.15	0.07	0.78	197	0.15	23.91
T0944TS432_5-D1.pdb	25.71	0.16	0.08	0.75	191	0.13	25.1
T0944TS434_1-D1.pdb	32.86	0.15	0	0.85	216	0.15	11.66
T0944TS434_2-D1.pdb	42.86	0.19	0	0.81	205	0.21	13.73
T0944TS434_3-D1.pdb	41.43	0.18	0	0.82	208	0.2	11.66

T0944TS434_4-D1.pdb	37.14	0.19	0	0.81	206	0.18	11.76
T0944TS434_5-D1.pdb	45.71	0.19	0	0.81	204	0.22	9.09
T0944TS439_1-D1.pdb	27.14	0.18	0.26	0.57	143	0.19	72.63
T0944TS439_2-D1.pdb	17.14	0.22	0.25	0.53	135	0.13	72.33
T0944TS439_3-D1.pdb	20	0.13	0.23	0.65	164	0.12	70.65
T0944TS439_4-D1.pdb	24.29	0.15	0.22	0.63	159	0.15	70.65
T0944TS439_5-D1.pdb	20	0.21	0.26	0.53	135	0.15	71.15
T0944TS441_1-D1.pdb	10	0.19	0.24	0.57	144	0.07	68.58
T0944TS441_3-D1.pdb	11.43	0.17	0.24	0.59	149	0.08	66.9
T0944TS441_4-D1.pdb	12.86	0.17	0.23	0.6	151	0.09	70.85
T0944TS441_5-D1.pdb	12.86	0.18	0.21	0.61	154	0.08	69.07
T0944TS443_1-D1.pdb	20	0.16	0.24	0.6	151	0.13	73.62
T0944TS443_2-D1.pdb	20	0.17	0.24	0.58	148	0.14	68.38
T0944TS443_3-D1.pdb	22.86	0.16	0.24	0.6	151	0.15	65.32
T0944TS443_4-D1.pdb	21.43	0.2	0.24	0.57	143	0.15	65.91
T0944TS443_5-D1.pdb	22.86	0.22	0.27	0.51	130	0.18	61.17
T0944TS446_1-D1.pdb	22.86	0.21	0.23	0.57	144	0.16	60.47
T0944TS446_2-D1.pdb	18.57	0.25	0.25	0.51	128	0.15	61.27
T0944TS446_4-D1.pdb	27.14	0.18	0.22	0.6	153	0.18	66.11
T0944TS446_5-D1.pdb	28.57	0.17	0.18	0.65	164	0.17	55.73
T0944TS450_1-D1.pdb	18.57	0.13	0.25	0.62	158	0.12	76.19
T0944TS450_2-D1.pdb	21.43	0.19	0.24	0.57	143	0.15	74.51
T0944TS450_3-D1.pdb	21.43	0.17	0.24	0.6	151	0.14	74.51
T0944TS450_4-D1.pdb	21.43	0.2	0.24	0.56	142	0.15	73.81
T0944TS450_5-D1.pdb	21.43	0.18	0.25	0.57	144	0.15	73.72
T0944TS451_1-D1.pdb	38.57	0.08	0.08	0.84	212	0.18	34.78
T0944TS451_2-D1.pdb	37.14	0.08	0.08	0.85	214	0.17	34.98
T0944TS451_3-D1.pdb	38.57	0.09	0.08	0.83	211	0.18	37.45
T0944TS451_4-D1.pdb	38.57	0.07	0.09	0.83	211	0.18	35.97
T0944TS451_5-D1.pdb	42.86	0.09	0.11	0.8	202	0.21	33.2
T0944TS452_1-D1.pdb	22.86	0.21	0.22	0.57	144	0.16	67.79
T0944TS452_2-D1.pdb	21.43	0.16	0.21	0.63	159	0.13	61.46
T0944TS452_3-D1.pdb	30	0.13	0.19	0.67	170	0.18	53.56
T0944TS452_4-D1.pdb	30	0.13	0.17	0.7	176	0.17	49.51
T0944TS452_5-D1.pdb	22.86	0.1	0.22	0.68	172	0.13	53.85
T0944TS455_1-D1.pdb	52.86	0.04	0.05	0.92	232	0.23	6.92
T0944TS455_2-D1.pdb	38.57	0.04	0.01	0.96	242	0.16	7.81
T0944TS455_3-D1.pdb	60	0.04	0.03	0.93	235	0.26	7.71
T0944TS455_4-D1.pdb	34.29	0.02	0.01	0.97	245	0.14	8.6
T0944TS455_5-D1.pdb	44.29	0.09	0.01	0.91	229	0.19	7.91
T0944TS456_1-D1.pdb	12.86	0.21	0.25	0.55	139	0.09	73.32
T0944TS456_2-D1.pdb	18.57	0.17	0.23	0.6	153	0.12	70.55
T0944TS456_3-D1.pdb	15.71	0.2	0.25	0.55	140	0.11	71.44
T0944TS456_4-D1.pdb	15.71	0.22	0.24	0.54	136	0.12	66.3
T0944TS456_5-D1.pdb	20	0.17	0.22	0.62	156	0.13	68.08
T0944TS464_1-D1.pdb	21.43	0.15	0.22	0.62	158	0.14	71.44
T0944TS464_2-D1.pdb	80	0.1	0.01	0.89	226	0.35	7.21

T0944TS464_3-D1.pdb	21.43	0.14	0.22	0.64	161	0.13	71.05
T0944TS464_4-D1.pdb	22.86	0.14	0.22	0.64	162	0.14	70.26
T0944TS464_5-D1.pdb	80	0.05	0	0.95	240	0.33	7.71
T0944TS467_1-D1.pdb	22.86	0.19	0.21	0.6	152	0.15	65.42
T0944TS467_2-D1.pdb	18.57	0.16	0.17	0.67	170	0.11	54.25
T0944TS467_3-D1.pdb	21.43	0.14	0.16	0.7	177	0.12	51.68
T0944TS467_4-D1.pdb	35.71	0.09	0.15	0.75	191	0.19	48.12
T0944TS467_5-D1.pdb	22.86	0.15	0.17	0.68	171	0.13	44.56
T0944TS474_1-D1.pdb	18.57	0.21	0.26	0.53	135	0.14	65.02
T0944TS474_2-D1.pdb	20	0.2	0.26	0.54	137	0.15	64.82
T0944TS475_1-D1.pdb	15.71	0.15	0.23	0.62	157	0.1	70.36
T0944TS475_2-D1.pdb	18.57	0.19	0.22	0.59	149	0.12	68.48
T0944TS475_3-D1.pdb	20	0.18	0.22	0.6	152	0.13	68.78
T0944TS475_4-D1.pdb	20	0.2	0.23	0.57	143	0.14	70.85
T0944TS475_5-D1.pdb	21.43	0.15	0.24	0.6	153	0.14	71.94
T0944TS479_2-D1.pdb	20	0.21	0.16	0.62	158	0.13	68.97
T0944TS479_3-D1.pdb	17.14	0.19	0.19	0.62	156	0.11	67.19
T0944TS479_4-D1.pdb	18.57	0.19	0.17	0.64	163	0.11	70.85
T0944TS479_5-D1.pdb	18.57	0.16	0.19	0.65	164	0.11	58.5
T0944TS480_1-D1.pdb	25.71	0.22	0.22	0.56	142	0.18	67.49
T0944TS480_2-D1.pdb	22.86	0.17	0.18	0.65	164	0.14	60.28
T0944TS480_3-D1.pdb	27.14	0.12	0.13	0.75	191	0.14	51.68
T0944TS480_4-D1.pdb	18.57	0.11	0.21	0.68	172	0.11	54.64
T0944TS480_5-D1.pdb	24.29	0.13	0.22	0.65	164	0.15	54.94
T0944TS483_1-D1.pdb	24.29	0.17	0.19	0.64	161	0.15	58
T0944TS483_2-D1.pdb	22.86	0.15	0.14	0.71	180	0.13	51.88
T0944TS483_3-D1.pdb	20	0.11	0.17	0.72	183	0.11	56.42
T0944TS483_4-D1.pdb	22.86	0.15	0.19	0.66	167	0.14	45.75
T0944TS483_5-D1.pdb	28.57	0.12	0.14	0.74	188	0.15	42.79
T0944TS486_2-D1.pdb	21.43	0.12	0.19	0.69	174	0.12	66.3
T0944TS486_3-D1.pdb	15.71	0.11	0.09	0.8	202	0.08	61.86
T0944TS486_4-D1.pdb	22.86	0.14	0.16	0.7	177	0.13	61.36
T0944TS486_5-D1.pdb	18.57	0.13	0.09	0.78	198	0.09	62.25
T0944TS489_1-D1.pdb	55.71	0.17	0.13	0.7	177	0.31	8.6
T0944TS498_1-D1.pdb	17.14	0.19	0.24	0.57	145	0.12	73.42
T0944TS498_2-D1.pdb	20	0.17	0.25	0.59	149	0.13	74.01
T0944TS498_3-D1.pdb	24.29	0.15	0.17	0.68	171	0.14	44.56
T0944TS498_5-D1.pdb	20	0.22	0.22	0.56	142	0.14	67
T0946TS001_1-D2.pdb	25	0.34	0.03	0.63	133	0.19	55.54
T0946TS001_2-D2.pdb	22.06	0.38	0.06	0.56	118	0.19	60.26
T0946TS001_3-D2.pdb	19.12	0.38	0.06	0.56	119	0.16	61.56
T0946TS001_4-D2.pdb	27.94	0.43	0.05	0.52	111	0.25	58.73
T0946TS001_5-D2.pdb	23.53	0.41	0.06	0.53	113	0.21	56.13
T0946TS004_1-D2.pdb	19.12	0.39	0.09	0.52	111	0.17	63.8
T0946TS004_2-D2.pdb	22.06	0.29	0.08	0.63	133	0.17	59.08
T0946TS004_3-D2.pdb	25	0.37	0.07	0.56	118	0.21	57.55
T0946TS004_4-D2.pdb	20.59	0.36	0.09	0.55	116	0.18	56.72

T0946TS004_5-D2.pdb	17.65	0.42	0.07	0.5	107	0.16	57.43
T0946TS005_1-D2.pdb	25	0.42	0.08	0.51	108	0.23	55.66
T0946TS005_3-D2.pdb	22.06	0.4	0.07	0.53	112	0.2	59.08
T0946TS005_4-D2.pdb	26.47	0.47	0.07	0.47	99	0.27	58.26
T0946TS005_5-D2.pdb	23.53	0.48	0.06	0.47	99	0.24	56.13
T0946TS011_1-D2.pdb	26.47	0.39	0.06	0.56	118	0.22	61.67
T0946TS011_2-D2.pdb	25	0.4	0.08	0.53	112	0.22	61.56
T0946TS011_3-D2.pdb	23.53	0.33	0.08	0.59	126	0.19	51.18
T0946TS011_4-D2.pdb	25	0.39	0.06	0.55	117	0.21	50.47
T0946TS011_5-D2.pdb	25	0.34	0.05	0.61	129	0.19	51.3
T0946TS016_1-D2.pdb	25	0.25	0.08	0.67	142	0.18	51.06
T0946TS017_1-D2.pdb	22.06	0.39	0.07	0.54	114	0.19	61.2
T0946TS017_2-D2.pdb	20.59	0.39	0.07	0.54	115	0.18	60.85
T0946TS017_3-D2.pdb	22.06	0.39	0.07	0.54	114	0.19	61.09
T0946TS017_4-D2.pdb	20.59	0.39	0.07	0.54	114	0.18	60.73
T0946TS017_5-D2.pdb	20.59	0.39	0.07	0.54	115	0.18	61.09
T0946TS019_1-D2.pdb	26.47	0.39	0.08	0.53	113	0.23	43.04
T0946TS022_1-D2.pdb	48.53	0.25	0	0.75	159	0.31	10.14
T0946TS022_2-D2.pdb	47.06	0.32	0	0.68	144	0.33	10.73
T0946TS022_3-D2.pdb	47.06	0.28	0	0.72	152	0.31	9.79
T0946TS022_4-D2.pdb	44.12	0.31	0	0.69	147	0.3	9.67
T0946TS022_5-D2.pdb	47.06	0.3	0	0.7	149	0.32	9.55
T0946TS023_1-D2.pdb	23.53	0.37	0.08	0.55	116	0.2	46.11
T0946TS023_2-D2.pdb	22.06	0.37	0.11	0.52	110	0.2	46.7
T0946TS023_3-D2.pdb	25	0.37	0.08	0.55	116	0.22	45.99
T0946TS023_4-D2.pdb	26.47	0.37	0.08	0.55	117	0.23	45.76
T0946TS023_5-D2.pdb	26.47	0.37	0.1	0.52	111	0.24	46.23
T0946TS026_1-D2.pdb	45.59	0.25	0.06	0.69	146	0.31	19.34
T0946TS026_2-D2.pdb	35.29	0.22	0.03	0.75	160	0.22	39.27
T0946TS026_5-D2.pdb	38.24	0.33	0.05	0.62	131	0.29	20.05
T0946TS040_1-D2.pdb	52.94	0.34	0.01	0.65	137	0.39	14.03
T0946TS040_2-D2.pdb	45.59	0.4	0.01	0.59	126	0.36	12.74
T0946TS040_3-D2.pdb	38.24	0.37	0	0.63	134	0.29	13.21
T0946TS040_4-D2.pdb	45.59	0.35	0	0.65	137	0.33	12.5
T0946TS040_5-D2.pdb	45.59	0.39	0	0.61	130	0.35	13.32
T0946TS042_1-D2.pdb	25	0.37	0.07	0.56	119	0.21	61.44
T0946TS042_2-D2.pdb	23.53	0.46	0.08	0.46	98	0.24	60.85
T0946TS042_3-D2.pdb	26.47	0.3	0.05	0.65	137	0.19	48.82
T0946TS042_4-D2.pdb	26.47	0.38	0.08	0.53	113	0.23	53.07
T0946TS042_5-D2.pdb	27.94	0.35	0.07	0.58	123	0.23	59.67
T0946TS060_1-D2.pdb	20.59	0.31	0.09	0.6	128	0.16	48.23
T0946TS060_2-D2.pdb	30.88	0.32	0.08	0.6	128	0.24	48.94
T0946TS060_3-D2.pdb	30.88	0.33	0.08	0.59	125	0.25	50.35
T0946TS060_4-D2.pdb	29.41	0.31	0.07	0.62	131	0.22	48.23
T0946TS060_5-D2.pdb	25	0.28	0.09	0.63	133	0.19	46.82
T0946TS064_1-D2.pdb	23.53	0.48	0.08	0.44	93	0.25	61.2
T0946TS067_3-D2.pdb	29.41	0.38	0.08	0.53	113	0.26	53.3

T0946TS073_1-D2.pdb	23.53	0.38	0.09	0.53	113	0.21	53.07
T0946TS073_2-D2.pdb	27.94	0.36	0.05	0.59	125	0.22	49.29
T0946TS073_3-D2.pdb	25	0.32	0.07	0.61	130	0.19	50.71
T0946TS073_4-D2.pdb	25	0.38	0.09	0.52	111	0.23	51.18
T0946TS073_5-D2.pdb	25	0.42	0.08	0.51	108	0.23	55.9
T0946TS079_3-D2.pdb	22.06	0.35	0.08	0.57	120	0.18	56.01
T0946TS079_4-D2.pdb	27.94	0.31	0.06	0.63	134	0.21	56.37
T0946TS079_5-D2.pdb	30.88	0.33	0.06	0.62	131	0.24	57.43
T0946TS102_1-D2.pdb	30.88	0.1	0	0.9	191	0.16	18.28
T0946TS102_2-D2.pdb	30.88	0.26	0	0.74	156	0.2	53.54
T0946TS102_3-D2.pdb	32.35	0.24	0	0.76	161	0.2	54.84
T0946TS102_5-D2.pdb	25	0.39	0.05	0.56	119	0.21	42.81
T0946TS126_1-D2.pdb	45.59	0.43	0.03	0.54	115	0.4	20.52
T0946TS126_2-D2.pdb	32.35	0.45	0.03	0.52	111	0.29	22.17
T0946TS126_3-D2.pdb	44.12	0.47	0.03	0.5	106	0.42	16.75
T0946TS126_4-D2.pdb	32.35	0.47	0	0.53	112	0.29	15.57
T0946TS126_5-D2.pdb	44.12	0.41	0	0.59	126	0.35	15.57
T0946TS162_1-D2.pdb	41.18	0.14	0	0.86	182	0.23	12.15
T0946TS162_2-D2.pdb	48.53	0.17	0.01	0.83	175	0.28	10.73
T0946TS162_3-D2.pdb	50	0.14	0.01	0.85	181	0.28	11.56
T0946TS162_4-D2.pdb	41.18	0.11	0.01	0.88	187	0.22	12.85
T0946TS162_5-D2.pdb	44.12	0.18	0.01	0.81	171	0.26	12.74
T0946TS171_1-D2.pdb	52.94	0.83	0.08	0.08	18	2.94	11.2
T0946TS171_2-D2.pdb	48.53	0.38	0.08	0.54	114	0.43	9.32
T0946TS171_3-D2.pdb	48.53	0.43	0.02	0.55	117	0.41	10.38
T0946TS171_4-D2.pdb	47.06	0.35	0.07	0.58	124	0.38	14.62
T0946TS171_5-D2.pdb	48.53	0.75	0	0.25	52	0.93	11.2
T0946TS173_1-D2.pdb	25	0.47	0.07	0.46	98	0.26	58.84
T0946TS173_2-D2.pdb	20.59	0.42	0.08	0.51	108	0.19	52.01
T0946TS173_3-D2.pdb	19.12	0.47	0.06	0.47	100	0.19	55.19
T0946TS173_4-D2.pdb	17.65	0.44	0.07	0.49	104	0.17	53.3
T0946TS173_5-D2.pdb	22.06	0.43	0.07	0.5	106	0.21	58.96
T0946TS179_1-D2.pdb	22.06	0.32	0.07	0.62	131	0.17	52.95
T0946TS179_2-D2.pdb	22.06	0.39	0.08	0.53	112	0.2	59.32
T0946TS179_3-D2.pdb	20.59	0.41	0.11	0.48	102	0.2	56.84
T0946TS179_4-D2.pdb	27.94	0.33	0.09	0.58	122	0.23	49.29
T0946TS179_5-D2.pdb	23.53	0.43	0.09	0.48	102	0.23	55.42
T0946TS180_1-D2.pdb	27.94	0.39	0.07	0.54	115	0.24	16.16
T0946TS180_2-D2.pdb	22.06	0.26	0.08	0.66	139	0.16	20.16
T0946TS180_3-D2.pdb	30.88	0.41	0.04	0.55	117	0.26	15.92
T0946TS180_4-D2.pdb	52.94	0.26	0.09	0.65	137	0.39	17.93
T0946TS180_5-D2.pdb	29.41	0.33	0	0.67	142	0.21	15.57
T0946TS182_1-D2.pdb	26.47	0.28	0.03	0.69	146	0.18	24.88
T0946TS182_2-D2.pdb	22.06	0.28	0.07	0.65	138	0.16	23.94
T0946TS182_3-D2.pdb	32.35	0.27	0	0.73	155	0.21	13.8
T0946TS182_4-D2.pdb	22.06	0.28	0.01	0.71	151	0.15	16.04
T0946TS182_5-D2.pdb	30.88	0.32	0.04	0.65	137	0.23	15.21

T0946TS183_1-D2.pdb	25	0.34	0.07	0.59	125	0.2	59.32
T0946TS183_3-D2.pdb	29.41	0.31	0.09	0.59	126	0.23	49.29
T0946TS183_4-D2.pdb	32.35	0.35	0	0.65	138	0.23	48.47
T0946TS183_5-D2.pdb	20.59	0.38	0.08	0.54	114	0.18	49.29
T0946TS187_2-D2.pdb	20.59	0.38	0.07	0.55	117	0.18	61.67
T0946TS187_5-D2.pdb	26.47	0.39	0.05	0.56	119	0.22	42.81
T0946TS188_1-D2.pdb	47.06	0.25	0.05	0.7	149	0.32	19.34
T0946TS188_3-D2.pdb	33.82	0.34	0.06	0.6	128	0.26	20.05
T0946TS188_4-D2.pdb	39.71	0.31	0.06	0.63	134	0.3	17.34
T0946TS192_1-D2.pdb	45.59	0.07	0.18	0.75	158	0.29	10.85
T0946TS203_2-D2.pdb	29.41	0.39	0.08	0.53	112	0.26	54.01
T0946TS203_5-D2.pdb	33.82	0.38	0.07	0.56	118	0.29	42.1
T0946TS207_1-D2.pdb	39.71	0.33	0	0.67	142	0.28	17.34
T0946TS207_2-D2.pdb	64.71	0.3	0.02	0.68	144	0.45	20.99
T0946TS207_3-D2.pdb	52.94	0.33	0.02	0.64	136	0.39	25.71
T0946TS207_4-D2.pdb	76.47	0.32	0	0.68	145	0.53	10.61
T0946TS207_5-D2.pdb	51.47	0.3	0	0.7	149	0.35	16.75
T0946TS220_4-D2.pdb	30.88	0.39	0.05	0.56	119	0.26	41.04
T0946TS220_5-D2.pdb	33.82	0.37	0.04	0.59	125	0.27	47.05
T0946TS230_1-D2.pdb	41.18	0.37	0.11	0.52	110	0.37	12.38
T0946TS230_2-D2.pdb	42.65	0.33	0.11	0.56	118	0.36	12.5
T0946TS230_3-D2.pdb	44.12	0.35	0.1	0.55	116	0.38	12.26
T0946TS230_4-D2.pdb	44.12	0.34	0.1	0.55	117	0.38	12.38
T0946TS230_5-D2.pdb	42.65	0.33	0.11	0.56	118	0.36	12.38
T0946TS232_3-D2.pdb	39.71	0.3	0.04	0.66	140	0.28	25.12
T0946TS232_5-D2.pdb	20.59	0.42	0.06	0.51	109	0.19	50.12
T0946TS236_1-D2.pdb	30.88	0.27	0.09	0.64	135	0.23	53.77
T0946TS236_2-D2.pdb	26.47	0.34	0.08	0.58	122	0.22	53.54
T0946TS236_3-D2.pdb	29.41	0.31	0.08	0.61	130	0.23	53.54
T0946TS236_4-D2.pdb	23.53	0.27	0.07	0.66	140	0.17	53.42
T0946TS236_5-D2.pdb	27.94	0.28	0.06	0.66	140	0.2	53.54
T0946TS239_1-D2.pdb	22.06	0.4	0.08	0.52	110	0.2	62.03
T0946TS239_2-D2.pdb	25	0.37	0.08	0.54	115	0.22	61.2
T0946TS239_3-D2.pdb	30.88	0.49	0.09	0.42	89	0.35	58.61
T0946TS239_4-D2.pdb	26.47	0.4	0.08	0.53	112	0.24	61.79
T0946TS239_5-D2.pdb	26.47	0.38	0.08	0.54	114	0.23	61.91
T0946TS243_1-D2.pdb	22.06	0.48	0.08	0.45	95	0.23	61.44
T0946TS243_2-D2.pdb	22.06	0.48	0.08	0.44	94	0.23	61.56
T0946TS243_3-D2.pdb	23.53	0.48	0.08	0.44	94	0.25	62.5
T0946TS243_4-D2.pdb	23.53	0.48	0.08	0.45	95	0.25	61.44
T0946TS243_5-D2.pdb	22.06	0.48	0.08	0.45	95	0.23	61.67
T0946TS250_1-D2.pdb	41.18	0.32	0.08	0.6	128	0.32	41.16
T0946TS250_2-D2.pdb	36.76	0.33	0.08	0.6	127	0.29	42.34
T0946TS250_3-D2.pdb	38.24	0.3	0.08	0.62	132	0.29	41.74
T0946TS250_4-D2.pdb	33.82	0.31	0.08	0.61	130	0.26	41.86
T0946TS250_5-D2.pdb	39.71	0.34	0.08	0.58	124	0.32	41.51
T0946TS251_1-D2.pdb	32.35	0.34	0.07	0.58	124	0.26	52.01

T0946TS251_2-D2.pdb	27.94	0.32	0.08	0.6	127	0.22	52.83
T0946TS251_3-D2.pdb	26.47	0.3	0.07	0.63	134	0.2	52.95
T0946TS251_4-D2.pdb	30.88	0.3	0.06	0.64	136	0.23	51.3
T0946TS251_5-D2.pdb	29.41	0.33	0.06	0.6	128	0.23	51.89
T0946TS252_1-D2.pdb	20.59	0.39	0.07	0.54	115	0.18	62.38
T0946TS252_2-D2.pdb	23.53	0.38	0.06	0.56	118	0.2	60.49
T0946TS252_3-D2.pdb	23.53	0.41	0.08	0.52	110	0.21	57.9
T0946TS252_4-D2.pdb	27.94	0.47	0.08	0.46	97	0.29	58.96
T0946TS252_5-D2.pdb	22.06	0.38	0.05	0.57	121	0.18	58.73
T0946TS258_1-D2.pdb	36.76	0.3	0.05	0.65	138	0.27	40.33
T0946TS258_2-D2.pdb	36.76	0.23	0.05	0.73	154	0.24	39.51
T0946TS258_3-D2.pdb	38.24	0.25	0.03	0.72	153	0.25	38.8
T0946TS258_4-D2.pdb	35.29	0.3	0.05	0.66	139	0.25	39.62
T0946TS258_5-D2.pdb	42.65	0.26	0.05	0.69	146	0.29	39.98
T0946TS264_1-D2.pdb	70.59	0.31	0	0.69	147	0.48	12.38
T0946TS264_2-D2.pdb	60.29	0.33	0.03	0.64	136	0.44	14.03
T0946TS264_3-D2.pdb	54.41	0.37	0.01	0.62	132	0.41	13.8
T0946TS264_4-D2.pdb	57.35	0.34	0	0.66	139	0.41	12.74
T0946TS264_5-D2.pdb	47.06	0.3	0.02	0.68	144	0.33	14.03
T0946TS275_1-D2.pdb	26.47	0.28	0.08	0.64	136	0.19	55.78
T0946TS275_2-D2.pdb	29.41	0.27	0.1	0.63	133	0.22	56.25
T0946TS275_3-D2.pdb	25	0.26	0.09	0.65	138	0.18	54.6
T0946TS275_4-D2.pdb	23.53	0.31	0.09	0.6	128	0.18	55.54
T0946TS275_5-D2.pdb	26.47	0.28	0.09	0.63	134	0.2	55.9
T0946TS287_1-D2.pdb	25	0.32	0.07	0.61	130	0.19	52.95
T0946TS287_2-D2.pdb	27.94	0.25	0.08	0.67	142	0.2	56.25
T0946TS287_3-D2.pdb	33.82	0.27	0.08	0.65	138	0.25	54.48
T0946TS287_5-D2.pdb	25	0.3	0.06	0.64	135	0.19	52.36
T0946TS295_3-D2.pdb	33.82	0.36	0.02	0.62	132	0.26	22.17
T0946TS303_1-D2.pdb	27.94	0.4	0.08	0.52	111	0.25	53.54
T0946TS303_2-D2.pdb	29.41	0.4	0.08	0.52	110	0.27	54.6
T0946TS303_3-D2.pdb	22.06	0.44	0.08	0.48	101	0.22	60.97
T0946TS303_4-D2.pdb	32.35	0.39	0.07	0.55	116	0.28	42.57
T0946TS303_5-D2.pdb	25	0.38	0.09	0.53	113	0.22	62.03
T0946TS313_1-D2.pdb	35.29	0.33	0.04	0.63	133	0.27	56.37
T0946TS313_2-D2.pdb	32.35	0.35	0.05	0.59	126	0.26	55.66
T0946TS313_3-D2.pdb	33.82	0.35	0.04	0.61	130	0.26	56.37
T0946TS313_4-D2.pdb	33.82	0.34	0.04	0.62	131	0.26	56.37
T0946TS320_2-D2.pdb	27.94	0.36	0.06	0.58	122	0.23	59.08
T0946TS320_5-D2.pdb	20.59	0.37	0.11	0.52	111	0.19	58.96
T0946TS321_1-D2.pdb	61.76	0.37	0.03	0.6	128	0.48	13.09
T0946TS321_2-D2.pdb	61.76	0.37	0.03	0.6	128	0.48	12.97
T0946TS321_3-D2.pdb	64.71	0.39	0	0.61	129	0.5	12.85
T0946TS321_4-D2.pdb	60.29	0.39	0	0.61	129	0.47	12.5
T0946TS321_5-D2.pdb	64.71	0.37	0	0.63	133	0.49	13.32
T0946TS324_1-D2.pdb	27.94	0.39	0.08	0.54	114	0.25	61.09
T0946TS324_2-D2.pdb	22.06	0.41	0.08	0.52	110	0.2	60.02

T0946TS324_3-D2.pdb	20.59	0.42	0.08	0.5	106	0.19	63.8
T0946TS324_4-D2.pdb	25	0.37	0.08	0.54	115	0.22	60.26
T0946TS324_5-D2.pdb	25	0.41	0.08	0.52	110	0.23	61.2
T0946TS325_1-D2.pdb	26.47	0.45	0.07	0.48	102	0.26	59.79
T0946TS325_2-D2.pdb	20.59	0.46	0.06	0.49	103	0.2	59.79
T0946TS325_3-D2.pdb	26.47	0.42	0.06	0.52	110	0.24	58.73
T0946TS325_4-D2.pdb	26.47	0.4	0.08	0.51	109	0.24	55.9
T0946TS325_5-D2.pdb	26.47	0.41	0.08	0.51	108	0.25	55.66
T0946TS345_1-D2.pdb	33.82	0.32	0.07	0.61	130	0.26	47.99
T0946TS345_2-D2.pdb	33.82	0.33	0.09	0.58	122	0.28	52.36
T0946TS345_3-D2.pdb	30.88	0.33	0.08	0.58	124	0.25	44.81
T0946TS345_4-D2.pdb	30.88	0.33	0.07	0.6	127	0.24	53.18
T0946TS345_5-D2.pdb	29.41	0.3	0.06	0.64	136	0.22	45.64
T0946TS356_1-D2.pdb	58.82	0.2	0.06	0.74	156	0.38	14.15
T0946TS356_2-D2.pdb	63.24	0.2	0.06	0.74	156	0.41	14.03
T0946TS356_3-D2.pdb	26.47	0.35	0.05	0.6	127	0.21	48.23
T0946TS356_4-D2.pdb	26.47	0.37	0.08	0.56	118	0.22	53.18
T0946TS356_5-D2.pdb	27.94	0.38	0.08	0.55	116	0.24	56.01
T0946TS357_1-D2.pdb	30.88	0.22	0.03	0.75	159	0.19	54.01
T0946TS357_2-D2.pdb	32.35	0.21	0.01	0.78	165	0.2	52.83
T0946TS359_2-D2.pdb	48.53	0.23	0.08	0.68	145	0.33	10.49
T0946TS367_1-D2.pdb	26.47	0.37	0.05	0.58	123	0.22	43.87
T0946TS382_1-D2.pdb	35.29	0.31	0.06	0.63	134	0.26	26.18
T0946TS382_2-D2.pdb	30.88	0.29	0.03	0.68	144	0.21	22.76
T0946TS382_3-D2.pdb	30.88	0.27	0.05	0.68	144	0.21	27.36
T0946TS382_5-D2.pdb	32.35	0.3	0	0.7	148	0.22	19.22
T0946TS384_1-D2.pdb	23.53	0.47	0.08	0.45	96	0.25	61.56
T0946TS384_2-D2.pdb	27.94	0.4	0.09	0.51	109	0.26	53.77
T0946TS384_3-D2.pdb	23.53	0.47	0.06	0.47	100	0.24	56.72
T0946TS384_4-D2.pdb	26.47	0.42	0.08	0.5	106	0.25	55.54
T0946TS384_5-D2.pdb	23.53	0.41	0.07	0.52	111	0.21	59.08
T0946TS393_1-D2.pdb	26.47	0.38	0.08	0.55	116	0.23	53.18
T0946TS393_2-D2.pdb	23.53	0.4	0.08	0.52	111	0.21	56.13
T0946TS393_3-D2.pdb	25	0.44	0.07	0.49	103	0.24	61.09
T0946TS393_4-D2.pdb	30.88	0.4	0.07	0.54	114	0.27	41.63
T0946TS393_5-D2.pdb	23.53	0.38	0.07	0.55	117	0.2	62.62
T0946TS396_1-D2.pdb	19.12	0.37	0.07	0.56	118	0.16	61.56
T0946TS396_3-D2.pdb	26.47	0.32	0.08	0.59	126	0.21	49.53
T0946TS396_4-D2.pdb	20.59	0.37	0.08	0.55	116	0.18	49.29
T0946TS396_5-D2.pdb	29.41	0.35	0.06	0.59	126	0.23	58.96
T0946TS399_1-D2.pdb	60.29	0.3	0	0.7	148	0.41	13.68
T0946TS399_2-D2.pdb	45.59	0.26	0	0.74	157	0.29	13.09
T0946TS399_3-D2.pdb	60.29	0.29	0	0.71	151	0.4	12.15
T0946TS399_4-D2.pdb	54.41	0.29	0	0.71	151	0.36	13.44
T0946TS399_5-D2.pdb	51.47	0.3	0	0.7	149	0.35	13.44
T0946TS405_1-D2.pdb	25	0.31	0.06	0.63	134	0.19	49.76
T0946TS405_2-D2.pdb	25	0.28	0.06	0.66	140	0.18	48.7

T0946TS405_4-D2.pdb	29.41	0.3	0.07	0.64	135	0.22	48.82
T0946TS405_5-D2.pdb	30.88	0.36	0.07	0.57	120	0.26	47.29
T0946TS407_2-D2.pdb	36.76	0.37	0	0.63	134	0.27	22.05
T0946TS407_3-D2.pdb	38.24	0.27	0.03	0.7	149	0.26	22.17
T0946TS407_4-D2.pdb	38.24	0.35	0.01	0.64	135	0.28	22.64
T0946TS407_5-D2.pdb	35.29	0.32	0.01	0.67	142	0.25	22.41
T0946TS411_1-D2.pdb	26.47	0.38	0.08	0.54	114	0.23	55.19
T0946TS411_2-D2.pdb	26.47	0.38	0.07	0.55	116	0.23	57.55
T0946TS411_3-D2.pdb	22.06	0.42	0.07	0.51	109	0.2	54.95
T0946TS411_4-D2.pdb	26.47	0.38	0.1	0.51	109	0.24	55.9
T0946TS411_5-D2.pdb	25	0.38	0.1	0.51	109	0.23	56.01
T0946TS434_1-D2.pdb	36.76	0.34	0	0.66	140	0.26	12.74
T0946TS434_2-D2.pdb	42.65	0.35	0	0.65	138	0.31	16.75
T0946TS434_3-D2.pdb	44.12	0.37	0	0.63	134	0.33	14.51
T0946TS434_4-D2.pdb	41.18	0.38	0	0.62	131	0.31	14.27
T0946TS434_5-D2.pdb	42.65	0.4	0	0.6	128	0.33	17.45
T0946TS439_1-D2.pdb	22.06	0.38	0.07	0.55	116	0.19	61.56
T0946TS439_2-D2.pdb	19.12	0.46	0.08	0.46	97	0.2	60.14
T0946TS439_3-D2.pdb	29.41	0.41	0.05	0.55	116	0.25	41.27
T0946TS439_4-D2.pdb	19.12	0.38	0.1	0.51	109	0.18	59.67
T0946TS439_5-D2.pdb	32.35	0.34	0.04	0.62	131	0.25	55.9
T0946TS441_1-D2.pdb	22.06	0.42	0.08	0.5	105	0.21	58.84
T0946TS441_3-D2.pdb	20.59	0.43	0.06	0.5	107	0.19	58.73
T0946TS441_4-D2.pdb	22.06	0.4	0.06	0.54	115	0.19	59.2
T0946TS441_5-D2.pdb	22.06	0.42	0.06	0.52	111	0.2	53.42
T0946TS443_1-D2.pdb	48.53	0.46	0.06	0.48	102	0.48	39.74
T0946TS443_2-D2.pdb	20.59	0.45	0.07	0.48	102	0.2	50.12
T0946TS443_3-D2.pdb	26.47	0.41	0.08	0.51	108	0.25	52.83
T0946TS443_4-D2.pdb	25	0.42	0.08	0.5	106	0.24	42.81
T0946TS443_5-D2.pdb	23.53	0.47	0.05	0.48	102	0.23	49.53
T0946TS444_3-D2.pdb	36.76	0.35	0.05	0.6	127	0.29	30.9
T0946TS444_4-D2.pdb	35.29	0.39	0.05	0.57	120	0.29	37.97
T0946TS444_5-D2.pdb	32.35	0.4	0.12	0.49	103	0.31	35.85
T0946TS446_1-D2.pdb	48.53	0.53	0.06	0.42	88	0.55	18.16
T0946TS446_3-D2.pdb	48.53	0.5	0.03	0.47	99	0.49	14.03
T0946TS446_5-D2.pdb	38.24	0.44	0.05	0.51	108	0.35	25.94
T0946TS450_1-D2.pdb	22.06	0.42	0.08	0.5	107	0.21	59.67
T0946TS450_2-D2.pdb	27.94	0.42	0.07	0.51	109	0.26	58.37
T0946TS450_3-D2.pdb	22.06	0.43	0.06	0.51	108	0.2	58.61
T0946TS450_4-D2.pdb	22.06	0.42	0.08	0.5	107	0.21	60.61
T0946TS450_5-D2.pdb	26.47	0.4	0.09	0.51	109	0.24	61.67
T0946TS451_1-D2.pdb	35.29	0.33	0.05	0.62	131	0.27	42.1
T0946TS451_2-D2.pdb	32.35	0.33	0.07	0.6	128	0.25	43.87
T0946TS451_3-D2.pdb	36.76	0.33	0.07	0.6	127	0.29	41.51
T0946TS451_4-D2.pdb	30.88	0.34	0.04	0.62	131	0.24	44.1
T0946TS451_5-D2.pdb	36.76	0.35	0.04	0.6	128	0.29	41.98
T0946TS452_1-D2.pdb	30.88	0.27	0.04	0.69	146	0.21	35.61

T0946TS452_2-D2.pdb	32.35	0.3	0.04	0.66	140	0.23	46.58
T0946TS452_3-D2.pdb	33.82	0.27	0.02	0.71	151	0.22	20.64
T0946TS452_4-D2.pdb	41.18	0.24	0.03	0.73	155	0.27	24.06
T0946TS452_5-D2.pdb	33.82	0.27	0.05	0.68	144	0.23	48.59
T0946TS455_1-D2.pdb	54.41	0.08	0.04	0.88	186	0.29	8.49
T0946TS455_2-D2.pdb	64.71	0.04	0.03	0.93	197	0.33	9.55
T0946TS455_3-D2.pdb	57.35	0.13	0.01	0.86	182	0.32	10.02
T0946TS455_4-D2.pdb	52.94	0.04	0.01	0.95	202	0.26	11.2
T0946TS455_5-D2.pdb	41.18	0.09	0.1	0.81	172	0.24	12.62
T0946TS456_1-D2.pdb	27.94	0.42	0.06	0.52	110	0.25	53.66
T0946TS456_2-D2.pdb	25	0.42	0.07	0.51	108	0.23	52.59
T0946TS456_3-D2.pdb	20.59	0.4	0.07	0.53	113	0.18	62.5
T0946TS456_4-D2.pdb	25	0.39	0.05	0.56	118	0.21	50.12
T0946TS456_5-D2.pdb	22.06	0.39	0.06	0.55	116	0.19	60.49
T0946TS464_1-D2.pdb	26.47	0.27	0.07	0.66	139	0.19	50.94
T0946TS464_2-D2.pdb	72.06	0.13	0	0.87	184	0.39	11.56
T0946TS464_3-D2.pdb	27.94	0.28	0.07	0.66	139	0.2	50.94
T0946TS464_4-D2.pdb	25	0.27	0.08	0.65	138	0.18	50.59
T0946TS464_5-D2.pdb	60.29	0.2	0.01	0.79	167	0.36	11.2
T0946TS467_1-D2.pdb	42.65	0.31	0.06	0.64	135	0.32	12.5
T0946TS467_2-D2.pdb	27.94	0.3	0.07	0.64	135	0.21	38.56
T0946TS467_3-D2.pdb	38.24	0.41	0.07	0.53	112	0.34	21.82
T0946TS467_4-D2.pdb	30.88	0.33	0.07	0.6	127	0.24	24.06
T0946TS467_5-D2.pdb	35.29	0.41	0.08	0.51	109	0.32	19.93
T0946TS474_1-D2.pdb	25	0.45	0.08	0.48	101	0.25	60.73
T0946TS474_2-D2.pdb	25	0.45	0.08	0.47	100	0.25	61.91
T0946TS474_3-D2.pdb	25	0.44	0.08	0.48	102	0.25	53.3
T0946TS475_1-D2.pdb	25	0.45	0.08	0.47	99	0.25	58.84
T0946TS475_2-D2.pdb	17.65	0.38	0.08	0.55	116	0.15	55.9
T0946TS475_3-D2.pdb	26.47	0.44	0.08	0.48	101	0.26	59.08
T0946TS475_4-D2.pdb	30.88	0.46	0.07	0.47	100	0.31	59.91
T0946TS475_5-D2.pdb	25	0.45	0.07	0.49	103	0.24	61.56
T0946TS479_3-D2.pdb	26.47	0.31	0.08	0.6	128	0.21	49.65
T0946TS479_5-D2.pdb	20.59	0.39	0	0.61	129	0.16	47.99
T0946TS480_1-D2.pdb	26.47	0.28	0.08	0.63	134	0.2	45.16
T0946TS480_2-D2.pdb	30.88	0.33	0.06	0.6	128	0.24	42.45
T0946TS480_3-D2.pdb	23.53	0.29	0.06	0.65	138	0.17	45.64
T0946TS480_4-D2.pdb	26.47	0.48	0.08	0.44	94	0.28	59.79
T0946TS480_5-D2.pdb	36.76	0.25	0.03	0.72	152	0.24	41.63
T0946TS483_1-D2.pdb	29.41	0.28	0.07	0.65	138	0.21	19.46
T0946TS483_2-D2.pdb	29.41	0.28	0.09	0.63	133	0.22	41.51
T0946TS483_3-D2.pdb	41.18	0.35	0.09	0.56	118	0.35	24.06
T0946TS483_4-D2.pdb	45.59	0.44	0.03	0.52	111	0.41	16.16
T0946TS483_5-D2.pdb	27.94	0.31	0.07	0.62	132	0.21	25.59
T0946TS486_2-D2.pdb	25	0.35	0.05	0.59	126	0.2	41.27
T0946TS489_1-D2.pdb	52.94	0.35	0	0.65	138	0.38	12.26
T0946TS495_1-D2.pdb	26.47	0.73	0.1	0.17	36	0.74	52.12

T0946TS495_2-D2.pdb	27.94	0.29	0.09	0.62	131	0.21	47.17
T0946TS495_3-D2.pdb	26.47	0.26	0.1	0.64	135	0.2	44.93
T0946TS495_4-D2.pdb	41.18	0.33	0.08	0.59	126	0.33	42.45
T0946TS495_5-D2.pdb	35.29	0.32	0.08	0.59	126	0.28	41.74
T0946TS498_1-D2.pdb	23.53	0.38	0.08	0.53	113	0.21	53.3
T0946TS498_2-D2.pdb	22.06	0.37	0.11	0.52	111	0.2	58.96
T0946TS498_4-D2.pdb	23.53	0.46	0.08	0.46	98	0.24	60.73
T0946TS498_5-D2.pdb	22.06	0.38	0.07	0.55	117	0.19	61.67
T0947TS001_1-D1.pdb	41.94	0.17	0.19	0.63	111	0.38	51.57
T0947TS004_1-D1.pdb	33.87	0.31	0.18	0.5	88	0.38	65.14
T0947TS004_2-D1.pdb	22.58	0.23	0.13	0.64	112	0.2	52.86
T0947TS004_3-D1.pdb	25.81	0.24	0.21	0.55	97	0.27	49.57
T0947TS004_4-D1.pdb	33.87	0.11	0.22	0.67	117	0.29	59
T0947TS004_5-D1.pdb	33.87	0.23	0.17	0.6	105	0.32	59.14
T0947TS005_1-D1.pdb	35.48	0.23	0.22	0.55	97	0.37	61.71
T0947TS005_2-D1.pdb	35.48	0.27	0.23	0.49	86	0.41	60
T0947TS005_3-D1.pdb	37.1	0.26	0.22	0.53	92	0.4	63.14
T0947TS005_4-D1.pdb	38.71	0.22	0.21	0.57	99	0.39	60.43
T0947TS005_5-D1.pdb	41.94	0.26	0.22	0.51	90	0.47	60.57
T0947TS011_1-D1.pdb	37.1	0.31	0.24	0.45	78	0.48	65.86
T0947TS011_2-D1.pdb	37.1	0.3	0.22	0.49	85	0.44	65.71
T0947TS011_3-D1.pdb	38.71	0.29	0.23	0.47	83	0.47	66
T0947TS011_4-D1.pdb	37.1	0.24	0.23	0.53	93	0.4	51
T0947TS011_5-D1.pdb	35.48	0.25	0.22	0.53	93	0.38	52.29
T0947TS016_1-D1.pdb	37.1	0.13	0.15	0.72	126	0.29	48.71
T0947TS017_1-D1.pdb	29.03	0.22	0.2	0.58	102	0.28	63.29
T0947TS017_3-D1.pdb	29.03	0.22	0.2	0.58	102	0.28	63.43
T0947TS017_4-D1.pdb	29.03	0.21	0.2	0.59	104	0.28	63.29
T0947TS022_1-D1.pdb	40.32	0.31	0	0.69	120	0.34	10.43
T0947TS022_2-D1.pdb	53.23	0.35	0	0.65	114	0.47	9.86
T0947TS022_3-D1.pdb	35.48	0.27	0	0.73	128	0.28	10.71
T0947TS022_4-D1.pdb	32.26	0.34	0	0.66	115	0.28	11.86
T0947TS022_5-D1.pdb	46.77	0.27	0	0.73	127	0.37	11.14
T0947TS023_1-D1.pdb	29.03	0.25	0.19	0.55	97	0.3	63.14
T0947TS023_2-D1.pdb	29.03	0.25	0.19	0.55	97	0.3	62.71
T0947TS023_3-D1.pdb	29.03	0.25	0.19	0.55	97	0.3	63.29
T0947TS023_4-D1.pdb	25.81	0.25	0.19	0.55	97	0.27	62.71
T0947TS023_5-D1.pdb	32.26	0.18	0.19	0.63	110	0.29	62.43
T0947TS026_1-D1.pdb	30.65	0.05	0.21	0.74	130	0.24	55
T0947TS026_2-D1.pdb	27.42	0.12	0.19	0.69	121	0.23	47.57
T0947TS026_3-D1.pdb	40.32	0.05	0.19	0.76	133	0.3	42.57
T0947TS026_5-D1.pdb	24.19	0.05	0.21	0.74	130	0.19	43.71
T0947TS028_1-D1.pdb	75.81	0.24	0.06	0.7	122	0.62	16.86
T0947TS028_2-D1.pdb	72.58	0.25	0.06	0.69	121	0.6	17.29
T0947TS028_3-D1.pdb	74.19	0.26	0.06	0.68	119	0.62	17
T0947TS028_4-D1.pdb	77.42	0.23	0.06	0.7	123	0.63	17.14
T0947TS028_5-D1.pdb	74.19	0.23	0.05	0.71	125	0.59	17.14

T0947TS040_1-D1.pdb	38.71	0.23	0.08	0.69	120	0.32	16.57
T0947TS040_2-D1.pdb	41.94	0.22	0.07	0.71	124	0.34	13.29
T0947TS040_3-D1.pdb	35.48	0.19	0.01	0.79	139	0.26	13
T0947TS040_4-D1.pdb	40.32	0.22	0.04	0.74	129	0.31	14.29
T0947TS040_5-D1.pdb	50	0.23	0.07	0.7	123	0.41	12.86
T0947TS042_1-D1.pdb	32.26	0.26	0.23	0.5	88	0.37	60
T0947TS042_2-D1.pdb	33.87	0.21	0.22	0.57	100	0.34	64.57
T0947TS042_3-D1.pdb	33.87	0.25	0.21	0.55	96	0.35	63.14
T0947TS042_4-D1.pdb	35.48	0.23	0.15	0.62	109	0.33	57.71
T0947TS042_5-D1.pdb	33.87	0.1	0.25	0.65	113	0.3	50
T0947TS048_1-D1.pdb	33.87	0.13	0.3	0.57	100	0.34	52.57
T0947TS060_1-D1.pdb	24.19	0.25	0.17	0.58	102	0.24	56.57
T0947TS060_2-D1.pdb	29.03	0.2	0.15	0.65	113	0.26	55.71
T0947TS060_3-D1.pdb	37.1	0.25	0.17	0.58	102	0.36	55
T0947TS060_4-D1.pdb	35.48	0.15	0.22	0.63	111	0.32	55.57
T0947TS060_5-D1.pdb	27.42	0.17	0.21	0.62	108	0.25	60.57
T0947TS064_1-D1.pdb	19.35	0.22	0.15	0.63	111	0.17	48.43
T0947TS073_1-D1.pdb	40.32	0.27	0.17	0.55	97	0.42	57.86
T0947TS073_3-D1.pdb	35.48	0.21	0.21	0.58	101	0.35	64.43
T0947TS073_4-D1.pdb	33.87	0.25	0.22	0.54	94	0.36	59
T0947TS073_5-D1.pdb	33.87	0.21	0.22	0.58	101	0.34	60.29
T0947TS077_1-D1.pdb	29.03	0.05	0.24	0.71	124	0.23	48.43
T0947TS077_2-D1.pdb	37.1	0.05	0.25	0.7	123	0.3	48.43
T0947TS077_3-D1.pdb	29.03	0.05	0.19	0.75	132	0.22	48.57
T0947TS077_4-D1.pdb	32.26	0.05	0.19	0.75	132	0.24	49.14
T0947TS077_5-D1.pdb	32.26	0.05	0.21	0.74	130	0.25	48.71
T0947TS079_3-D1.pdb	30.65	0.09	0.14	0.77	135	0.23	59.14
T0947TS079_4-D1.pdb	32.26	0.08	0.15	0.77	135	0.24	59.57
T0947TS079_5-D1.pdb	27.42	0.19	0.17	0.64	112	0.24	60.43
T0947TS083_1-D1.pdb	32.26	0.25	0.21	0.55	96	0.34	14.57
T0947TS083_2-D1.pdb	33.87	0.27	0.14	0.59	103	0.33	19.71
T0947TS083_3-D1.pdb	35.48	0.27	0.25	0.49	85	0.42	17
T0947TS083_4-D1.pdb	37.1	0.33	0.13	0.55	96	0.39	14.57
T0947TS083_5-D1.pdb	32.26	0.3	0.18	0.52	91	0.35	13.43
T0947TS102_1-D1.pdb	33.87	0.03	0.06	0.91	159	0.21	49.29
T0947TS119_1-D1.pdb	32.26	0.09	0.14	0.77	135	0.24	59.14
T0947TS126_1-D1.pdb	41.94	0.25	0.1	0.65	113	0.37	22
T0947TS126_2-D1.pdb	45.16	0.26	0.09	0.66	115	0.39	18.29
T0947TS126_3-D1.pdb	40.32	0.25	0.08	0.67	117	0.34	17
T0947TS126_4-D1.pdb	54.84	0.28	0.14	0.58	101	0.54	19.57
T0947TS126_5-D1.pdb	45.16	0.25	0.09	0.66	116	0.39	19.29
T0947TS145_1-D1.pdb	32.26	0.28	0.04	0.68	119	0.27	15.57
T0947TS145_2-D1.pdb	27.42	0.33	0.06	0.61	107	0.26	13.14
T0947TS145_3-D1.pdb	29.03	0.3	0.01	0.69	120	0.24	15.86
T0947TS145_4-D1.pdb	33.87	0.27	0.05	0.69	120	0.28	13.43
T0947TS145_5-D1.pdb	45.16	0.24	0.01	0.75	131	0.34	15.57
T0947TS171_1-D1.pdb	40.32	0.19	0.18	0.63	111	0.36	31.14

T0947TS171_2-D1.pdb	64.52	0.26	0.15	0.59	103	0.63	9.43
T0947TS171_3-D1.pdb	40.32	0.19	0.18	0.63	111	0.36	31.29
T0947TS173_1-D1.pdb	19.35	0.23	0.23	0.54	95	0.2	52.43
T0947TS173_2-D1.pdb	32.26	0.27	0.19	0.54	94	0.34	60
T0947TS173_3-D1.pdb	22.58	0.23	0.25	0.52	91	0.25	57.57
T0947TS173_4-D1.pdb	24.19	0.2	0.21	0.59	103	0.23	56.86
T0947TS173_5-D1.pdb	27.42	0.24	0.22	0.54	94	0.29	51.29
T0947TS179_1-D1.pdb	29.03	0.11	0.23	0.66	116	0.25	49.14
T0947TS179_2-D1.pdb	35.48	0.09	0.14	0.77	135	0.26	49
T0947TS179_3-D1.pdb	22.58	0.1	0.3	0.6	105	0.22	48.14
T0947TS179_4-D1.pdb	32.26	0.09	0.25	0.66	116	0.28	43.14
T0947TS179_5-D1.pdb	29.03	0.22	0.19	0.59	103	0.28	54.43
T0947TS180_1-D1.pdb	32.26	0.06	0.27	0.67	117	0.28	49.71
T0947TS180_2-D1.pdb	32.26	0.08	0.21	0.71	125	0.26	47.71
T0947TS180_3-D1.pdb	33.87	0.11	0.2	0.69	121	0.28	52.29
T0947TS180_4-D1.pdb	38.71	0.05	0.17	0.78	136	0.28	45.57
T0947TS180_5-D1.pdb	27.42	0.2	0.03	0.77	135	0.2	16.43
T0947TS182_1-D1.pdb	29.03	0.25	0.09	0.66	116	0.25	21.86
T0947TS182_2-D1.pdb	37.1	0.22	0.09	0.69	121	0.31	14.86
T0947TS182_3-D1.pdb	27.42	0.22	0.1	0.67	118	0.23	16.14
T0947TS182_4-D1.pdb	27.42	0.17	0.06	0.78	136	0.2	16.71
T0947TS182_5-D1.pdb	27.42	0.24	0.05	0.71	125	0.22	15.43
T0947TS183_3-D1.pdb	27.42	0.26	0.19	0.55	96	0.29	57.43
T0947TS183_4-D1.pdb	32.26	0.22	0.15	0.63	111	0.29	58
T0947TS183_5-D1.pdb	37.1	0.12	0.16	0.72	126	0.29	57.71
T0947TS187_1-D1.pdb	30.65	0.23	0.24	0.53	92	0.33	55
T0947TS187_2-D1.pdb	29.03	0.23	0.25	0.52	91	0.32	54.71
T0947TS187_3-D1.pdb	35.48	0.27	0.26	0.47	82	0.43	56
T0947TS187_4-D1.pdb	32.26	0.14	0.21	0.65	113	0.29	44.14
T0947TS187_5-D1.pdb	32.26	0.21	0.26	0.53	92	0.35	54.71
T0947TS188_5-D1.pdb	27.42	0.11	0.21	0.67	118	0.23	47.57
T0947TS207_1-D1.pdb	24.19	0.24	0.09	0.67	118	0.21	50.29
T0947TS207_2-D1.pdb	27.42	0.14	0.11	0.74	130	0.21	46.86
T0947TS207_3-D1.pdb	40.32	0.16	0.13	0.71	124	0.33	31.14
T0947TS207_4-D1.pdb	59.68	0.21	0	0.79	139	0.43	12.57
T0947TS207_5-D1.pdb	24.19	0.22	0.03	0.75	132	0.18	39.43
T0947TS220_2-D1.pdb	30.65	0.18	0.17	0.65	113	0.27	51.29
T0947TS220_3-D1.pdb	33.87	0.15	0.24	0.61	106	0.32	54.14
T0947TS220_4-D1.pdb	30.65	0.05	0.27	0.67	118	0.26	51.57
T0947TS220_5-D1.pdb	35.48	0.17	0.21	0.62	109	0.33	47.57
T0947TS230_1-D1.pdb	41.94	0.17	0.24	0.59	103	0.41	17.14
T0947TS230_2-D1.pdb	41.94	0.17	0.23	0.6	105	0.4	17.14
T0947TS230_3-D1.pdb	43.55	0.17	0.24	0.59	103	0.42	18.14
T0947TS230_4-D1.pdb	38.71	0.17	0.23	0.6	105	0.37	17.29
T0947TS230_5-D1.pdb	40.32	0.17	0.25	0.58	101	0.4	17.71
T0947TS232_3-D1.pdb	33.87	0.21	0.15	0.64	112	0.3	53
T0947TS232_5-D1.pdb	29.03	0.12	0.25	0.63	110	0.26	48.57

T0947TS236_1-D1.pdb	35.48	0.13	0.29	0.59	103	0.34	48.86
T0947TS236_2-D1.pdb	33.87	0.11	0.26	0.63	111	0.31	49.71
T0947TS236_3-D1.pdb	35.48	0.13	0.25	0.63	110	0.32	48.71
T0947TS236_4-D1.pdb	29.03	0.06	0.34	0.6	105	0.28	48.86
T0947TS236_5-D1.pdb	27.42	0.06	0.29	0.65	114	0.24	49.43
T0947TS239_1-D1.pdb	32.26	0.24	0.27	0.49	86	0.38	59.14
T0947TS239_2-D1.pdb	37.1	0.29	0.23	0.49	85	0.44	56.86
T0947TS239_3-D1.pdb	30.65	0.28	0.22	0.5	88	0.35	57.71
T0947TS239_4-D1.pdb	30.65	0.26	0.22	0.53	92	0.33	64.43
T0947TS239_5-D1.pdb	32.26	0.25	0.22	0.54	94	0.34	65.29
T0947TS243_1-D1.pdb	37.1	0.29	0.19	0.51	90	0.41	57.43
T0947TS243_2-D1.pdb	33.87	0.3	0.2	0.5	87	0.39	57
T0947TS243_3-D1.pdb	38.71	0.3	0.2	0.5	87	0.44	57.57
T0947TS243_4-D1.pdb	35.48	0.3	0.2	0.5	87	0.41	57
T0947TS243_5-D1.pdb	35.48	0.29	0.19	0.52	91	0.39	57.29
T0947TS250_1-D1.pdb	35.48	0.14	0.19	0.67	118	0.3	53.14
T0947TS250_2-D1.pdb	33.87	0.16	0.19	0.65	114	0.3	54.14
T0947TS250_3-D1.pdb	33.87	0.15	0.19	0.66	115	0.29	54
T0947TS250_4-D1.pdb	29.03	0.16	0.19	0.65	114	0.25	53.71
T0947TS250_5-D1.pdb	37.1	0.15	0.19	0.66	115	0.32	52.71
T0947TS251_1-D1.pdb	32.26	0.12	0.18	0.7	123	0.26	42.71
T0947TS251_2-D1.pdb	32.26	0.09	0.18	0.73	128	0.25	45.14
T0947TS251_3-D1.pdb	30.65	0.05	0.18	0.77	134	0.23	49
T0947TS251_4-D1.pdb	30.65	0.16	0.17	0.67	117	0.26	48.86
T0947TS251_5-D1.pdb	43.55	0.17	0.17	0.66	116	0.38	57.29
T0947TS252_1-D1.pdb	30.65	0.25	0.22	0.54	94	0.33	59.71
T0947TS252_2-D1.pdb	29.03	0.25	0.23	0.51	90	0.32	60.14
T0947TS252_3-D1.pdb	33.87	0.25	0.25	0.51	89	0.38	61.86
T0947TS252_4-D1.pdb	35.48	0.22	0.22	0.56	98	0.36	59.71
T0947TS252_5-D1.pdb	35.48	0.24	0.22	0.54	95	0.37	57.86
T0947TS258_1-D1.pdb	29.03	0.1	0.24	0.66	116	0.25	48
T0947TS258_2-D1.pdb	29.03	0.1	0.25	0.65	113	0.26	47.86
T0947TS258_3-D1.pdb	29.03	0.1	0.23	0.67	117	0.25	48.43
T0947TS258_5-D1.pdb	27.42	0.1	0.19	0.71	124	0.22	48.43
T0947TS275_1-D1.pdb	32.26	0.15	0.18	0.67	117	0.28	58.43
T0947TS275_2-D1.pdb	37.1	0.18	0.17	0.65	114	0.33	57.14
T0947TS275_3-D1.pdb	37.1	0.19	0.16	0.65	113	0.33	56.43
T0947TS275_4-D1.pdb	27.42	0.15	0.16	0.69	120	0.23	57.57
T0947TS275_5-D1.pdb	33.87	0.17	0.17	0.66	116	0.29	58.14
T0947TS287_1-D1.pdb	37.1	0.13	0.29	0.59	103	0.36	48.86
T0947TS287_3-D1.pdb	33.87	0.05	0.3	0.65	114	0.3	49.57
T0947TS287_4-D1.pdb	30.65	0.06	0.29	0.65	114	0.27	49.29
T0947TS287_5-D1.pdb	33.87	0.05	0.23	0.72	126	0.27	48.71
T0947TS295_1-D1.pdb	25.81	0.22	0.21	0.58	101	0.26	46.29
T0947TS295_2-D1.pdb	33.87	0.26	0.25	0.5	87	0.39	54.86
T0947TS295_3-D1.pdb	29.03	0.15	0.19	0.65	114	0.25	44.43
T0947TS295_4-D1.pdb	30.65	0.26	0.27	0.47	82	0.37	56

T0947TS303_1-D1.pdb	35.48	0.29	0.21	0.5	88	0.4	57.43
T0947TS303_2-D1.pdb	35.48	0.23	0.22	0.55	96	0.37	62.43
T0947TS303_3-D1.pdb	37.1	0.27	0.22	0.5	88	0.42	63.14
T0947TS303_4-D1.pdb	33.87	0.21	0.22	0.58	101	0.34	65.14
T0947TS303_5-D1.pdb	30.65	0.24	0.25	0.51	90	0.34	59.14
T0947TS313_1-D1.pdb	33.87	0.08	0.14	0.78	136	0.25	59
T0947TS313_2-D1.pdb	30.65	0.08	0.15	0.77	135	0.23	58.71
T0947TS313_3-D1.pdb	25.81	0.08	0.13	0.79	138	0.19	58.86
T0947TS313_5-D1.pdb	29.03	0.08	0.13	0.79	138	0.21	58.57
T0947TS321_1-D1.pdb	48.39	0.25	0.13	0.62	109	0.44	13.71
T0947TS321_2-D1.pdb	46.77	0.23	0.12	0.65	113	0.41	13.29
T0947TS321_3-D1.pdb	70.97	0.22	0.12	0.66	116	0.61	11.86
T0947TS321_4-D1.pdb	48.39	0.26	0.13	0.61	107	0.45	13.57
T0947TS321_5-D1.pdb	64.52	0.21	0.13	0.66	115	0.56	12
T0947TS324_1-D1.pdb	37.1	0.27	0.17	0.55	97	0.38	58
T0947TS324_2-D1.pdb	29.03	0.23	0.23	0.54	94	0.31	61.57
T0947TS324_3-D1.pdb	29.03	0.25	0.21	0.55	96	0.3	58.71
T0947TS324_4-D1.pdb	32.26	0.27	0.23	0.49	86	0.38	59.57
T0947TS324_5-D1.pdb	30.65	0.27	0.22	0.51	90	0.34	57.29
T0947TS325_1-D1.pdb	35.48	0.21	0.22	0.57	100	0.35	61.71
T0947TS325_2-D1.pdb	33.87	0.25	0.23	0.52	91	0.37	62.29
T0947TS325_3-D1.pdb	33.87	0.26	0.25	0.5	87	0.39	59.57
T0947TS325_4-D1.pdb	29.03	0.25	0.19	0.56	98	0.3	58.29
T0947TS325_5-D1.pdb	32.26	0.26	0.18	0.57	99	0.33	58.57
T0947TS330_1-D1.pdb	32.26	0.25	0.26	0.49	85	0.38	52.57
T0947TS330_2-D1.pdb	38.71	0.27	0.23	0.5	87	0.44	48.43
T0947TS330_3-D1.pdb	30.65	0.19	0.26	0.54	95	0.32	45.86
T0947TS330_4-D1.pdb	37.1	0.26	0.25	0.5	87	0.43	58.43
T0947TS330_5-D1.pdb	32.26	0.29	0.23	0.48	84	0.38	31.71
T0947TS345_1-D1.pdb	32.26	0.06	0.34	0.6	105	0.31	46.86
T0947TS345_2-D1.pdb	32.26	0.05	0.39	0.55	97	0.33	47.43
T0947TS345_3-D1.pdb	32.26	0.11	0.23	0.66	116	0.28	48.57
T0947TS345_4-D1.pdb	35.48	0.11	0.27	0.62	109	0.33	48.71
T0947TS345_5-D1.pdb	29.03	0.06	0.2	0.74	129	0.23	46.43
T0947TS357_2-D1.pdb	35.48	0.03	0.08	0.89	155	0.23	49.43
T0947TS357_3-D1.pdb	29.03	0.01	0.07	0.92	161	0.18	49.43
T0947TS357_4-D1.pdb	35.48	0.02	0.12	0.86	151	0.23	49.29
T0947TS357_5-D1.pdb	38.71	0.02	0.07	0.9	158	0.24	49.71
T0947TS382_1-D1.pdb	29.03	0.18	0.22	0.6	105	0.28	49
T0947TS382_2-D1.pdb	29.03	0.18	0.23	0.59	103	0.28	44.14
T0947TS382_3-D1.pdb	48.39	0.19	0.26	0.55	96	0.5	31
T0947TS382_4-D1.pdb	38.71	0.18	0.25	0.57	99	0.39	35.86
T0947TS382_5-D1.pdb	59.68	0.18	0.16	0.66	116	0.51	22
T0947TS384_1-D1.pdb	35.48	0.28	0.17	0.55	97	0.37	58.29
T0947TS384_2-D1.pdb	35.48	0.28	0.23	0.49	86	0.41	60
T0947TS384_3-D1.pdb	35.48	0.26	0.23	0.51	89	0.4	59
T0947TS384_5-D1.pdb	37.1	0.21	0.14	0.65	114	0.33	53.29

T0947TS393_1-D1.pdb	35.48	0.29	0.15	0.57	99	0.36	56.43
T0947TS393_2-D1.pdb	35.48	0.22	0.21	0.57	100	0.35	62.71
T0947TS393_3-D1.pdb	35.48	0.27	0.21	0.52	91	0.39	62.14
T0947TS393_4-D1.pdb	30.65	0.23	0.19	0.58	102	0.3	63.43
T0947TS393_5-D1.pdb	29.03	0.25	0.25	0.5	88	0.33	57.71
T0947TS396_1-D1.pdb	41.94	0.27	0.18	0.54	95	0.44	57.86
T0947TS396_2-D1.pdb	33.87	0.23	0.21	0.55	97	0.35	58.71
T0947TS396_3-D1.pdb	33.87	0.21	0.17	0.62	109	0.31	53
T0947TS396_4-D1.pdb	27.42	0.19	0.16	0.65	114	0.24	60.86
T0947TS396_5-D1.pdb	33.87	0.21	0.22	0.57	100	0.34	60.29
T0947TS399_2-D1.pdb	54.84	0.26	0	0.74	129	0.43	13.71
T0947TS399_3-D1.pdb	61.29	0.26	0	0.74	129	0.48	14.57
T0947TS399_4-D1.pdb	61.29	0.25	0	0.75	131	0.47	11.57
T0947TS399_5-D1.pdb	58.06	0.26	0	0.74	129	0.45	14.29
T0947TS405_1-D1.pdb	38.71	0.18	0.23	0.58	102	0.38	56.29
T0947TS405_2-D1.pdb	38.71	0.13	0.2	0.67	118	0.33	57.71
T0947TS405_4-D1.pdb	30.65	0.11	0.22	0.67	117	0.26	59.14
T0947TS405_5-D1.pdb	29.03	0.18	0.2	0.62	109	0.27	57.43
T0947TS407_1-D1.pdb	30.65	0.07	0.25	0.69	120	0.26	49.86
T0947TS407_2-D1.pdb	37.1	0.12	0.21	0.67	118	0.31	49.29
T0947TS407_4-D1.pdb	38.71	0.11	0.18	0.71	125	0.31	48.57
T0947TS407_5-D1.pdb	38.71	0.13	0.17	0.7	122	0.32	47.43
T0947TS411_1-D1.pdb	41.94	0.27	0.17	0.56	98	0.43	56
T0947TS411_2-D1.pdb	37.1	0.25	0.22	0.54	94	0.39	56.57
T0947TS411_3-D1.pdb	41.94	0.26	0.23	0.5	88	0.48	60.86
T0947TS411_4-D1.pdb	33.87	0.23	0.21	0.56	98	0.35	61.43
T0947TS411_5-D1.pdb	33.87	0.21	0.21	0.58	102	0.33	63.86
T0947TS421_1-D1.pdb	27.42	0.06	0.14	0.79	139	0.2	55.43
T0947TS425_1-D1.pdb	33.87	0.05	0.22	0.73	127	0.27	48.43
T0947TS425_2-D1.pdb	29.03	0.07	0.19	0.74	129	0.23	49
T0947TS425_3-D1.pdb	33.87	0.05	0.25	0.7	123	0.28	49.29
T0947TS425_4-D1.pdb	30.65	0.05	0.21	0.74	129	0.24	48.86
T0947TS425_5-D1.pdb	38.71	0.06	0.25	0.69	121	0.32	49.14
T0947TS434_1-D1.pdb	50	0.16	0.05	0.79	139	0.36	15.14
T0947TS434_2-D1.pdb	56.45	0.15	0.02	0.83	145	0.39	15.43
T0947TS434_3-D1.pdb	48.39	0.17	0.03	0.8	140	0.35	14.86
T0947TS434_4-D1.pdb	67.74	0.2	0.01	0.79	138	0.49	13.43
T0947TS434_5-D1.pdb	67.74	0.19	0	0.81	142	0.48	11.57
T0947TS439_1-D1.pdb	30.65	0.23	0.22	0.55	96	0.32	62.14
T0947TS439_2-D1.pdb	32.26	0.3	0.23	0.47	82	0.39	60.43
T0947TS439_3-D1.pdb	30.65	0.18	0.21	0.61	107	0.29	48
T0947TS439_4-D1.pdb	32.26	0.26	0.23	0.51	89	0.36	58.29
T0947TS439_5-D1.pdb	35.48	0.14	0.22	0.63	111	0.32	54.29
T0947TS441_2-D1.pdb	24.19	0.19	0.23	0.57	100	0.24	59
T0947TS441_3-D1.pdb	25.81	0.24	0.22	0.54	95	0.27	57.71
T0947TS441_4-D1.pdb	32.26	0.24	0.24	0.52	91	0.35	52.14
T0947TS443_1-D1.pdb	33.87	0.26	0.22	0.52	91	0.37	60.43

T0947TS443_2-D1.pdb	32.26	0.27	0.22	0.51	89	0.36	61.71
T0947TS443_3-D1.pdb	35.48	0.27	0.19	0.54	94	0.38	65.71
T0947TS443_4-D1.pdb	40.32	0.25	0.19	0.55	97	0.42	65.29
T0947TS443_5-D1.pdb	33.87	0.27	0.21	0.52	91	0.37	63.71
T0947TS444_1-D1.pdb	20.97	0.09	0.23	0.68	119	0.18	52.57
T0947TS444_2-D1.pdb	20.97	0.11	0.21	0.67	118	0.18	48.71
T0947TS444_3-D1.pdb	25.81	0.14	0.18	0.68	119	0.22	46.43
T0947TS444_4-D1.pdb	32.26	0.23	0.08	0.69	121	0.27	18.86
T0947TS444_5-D1.pdb	32.26	0.13	0.18	0.69	120	0.27	46.14
T0947TS446_1-D1.pdb	40.32	0.23	0.22	0.54	95	0.42	66.43
T0947TS446_2-D1.pdb	43.55	0.22	0.25	0.53	92	0.47	60.43
T0947TS446_4-D1.pdb	40.32	0.14	0.25	0.61	106	0.38	48.29
T0947TS446_5-D1.pdb	40.32	0.18	0.21	0.61	107	0.38	47.71
T0947TS450_1-D1.pdb	35.48	0.21	0.23	0.55	97	0.37	63.43
T0947TS450_2-D1.pdb	33.87	0.23	0.24	0.53	92	0.37	63.57
T0947TS450_3-D1.pdb	35.48	0.21	0.24	0.55	96	0.37	64.29
T0947TS450_4-D1.pdb	32.26	0.22	0.25	0.54	94	0.34	62.14
T0947TS450_5-D1.pdb	37.1	0.23	0.24	0.53	92	0.4	60.86
T0947TS451_1-D1.pdb	38.71	0.19	0.05	0.76	133	0.29	33.86
T0947TS451_2-D1.pdb	41.94	0.19	0.1	0.71	125	0.34	30.14
T0947TS451_3-D1.pdb	45.16	0.17	0.07	0.76	133	0.34	16.14
T0947TS451_4-D1.pdb	45.16	0.2	0.09	0.71	125	0.36	36.57
T0947TS451_5-D1.pdb	37.1	0.19	0.04	0.77	134	0.28	16.86
T0947TS452_1-D1.pdb	29.03	0.16	0.13	0.71	124	0.23	43.43
T0947TS452_2-D1.pdb	30.65	0.11	0.17	0.71	125	0.25	51.86
T0947TS452_3-D1.pdb	43.55	0.18	0.17	0.65	113	0.39	54.71
T0947TS455_1-D1.pdb	59.68	0.19	0.05	0.76	133	0.45	10.14
T0947TS455_2-D1.pdb	48.39	0.07	0	0.93	163	0.3	11.43
T0947TS455_3-D1.pdb	45.16	0.17	0.02	0.81	142	0.32	10.86
T0947TS455_4-D1.pdb	48.39	0.09	0.03	0.89	155	0.31	11.57
T0947TS455_5-D1.pdb	40.32	0.15	0.07	0.77	135	0.3	12.71
T0947TS456_1-D1.pdb	33.87	0.25	0.21	0.55	96	0.35	55
T0947TS456_2-D1.pdb	32.26	0.26	0.23	0.51	89	0.36	62
T0947TS456_3-D1.pdb	38.71	0.26	0.2	0.54	95	0.41	57.29
T0947TS456_4-D1.pdb	37.1	0.23	0.21	0.56	98	0.38	61.14
T0947TS456_5-D1.pdb	35.48	0.29	0.19	0.53	92	0.39	58.71
T0947TS464_1-D1.pdb	37.1	0.09	0.22	0.69	121	0.31	51.57
T0947TS464_2-D1.pdb	77.42	0.09	0	0.91	159	0.49	10.86
T0947TS464_4-D1.pdb	37.1	0.09	0.2	0.71	124	0.3	51.57
T0947TS464_5-D1.pdb	87.1	0.21	0	0.79	138	0.63	10.29
T0947TS467_1-D1.pdb	46.77	0.22	0.11	0.67	117	0.4	12.29
T0947TS467_2-D1.pdb	48.39	0.15	0.12	0.73	128	0.38	13.86
T0947TS467_3-D1.pdb	40.32	0.15	0.13	0.71	125	0.32	15.14
T0947TS467_4-D1.pdb	40.32	0.33	0.03	0.64	112	0.36	14.14
T0947TS467_5-D1.pdb	41.94	0.17	0.17	0.67	117	0.36	13.86
T0947TS475_1-D1.pdb	35.48	0.24	0.19	0.57	99	0.36	61.29
T0947TS475_2-D1.pdb	30.65	0.25	0.22	0.53	93	0.33	60.57

T0947TS475_3-D1.pdb	35.48	0.24	0.21	0.55	96	0.37	62.14
T0947TS475_4-D1.pdb	27.42	0.25	0.25	0.51	89	0.31	59.71
T0947TS475_5-D1.pdb	37.1	0.25	0.22	0.54	94	0.39	56.14
T0947TS479_2-D1.pdb	24.19	0.26	0.13	0.61	106	0.23	53.57
T0947TS479_4-D1.pdb	40.32	0.18	0.16	0.66	116	0.35	58
T0947TS480_1-D1.pdb	29.03	0.21	0.22	0.58	101	0.29	47.86
T0947TS480_2-D1.pdb	27.42	0.19	0.25	0.56	98	0.28	48.14
T0947TS480_3-D1.pdb	33.87	0.17	0.22	0.61	107	0.32	49.14
T0947TS480_4-D1.pdb	38.71	0.26	0.22	0.52	91	0.43	61.43
T0947TS480_5-D1.pdb	35.48	0.19	0.24	0.57	100	0.35	48.86
T0947TS483_2-D1.pdb	35.48	0.17	0.2	0.63	111	0.32	11.43
T0947TS483_3-D1.pdb	48.39	0.22	0.12	0.66	115	0.42	12
T0947TS483_4-D1.pdb	35.48	0.01	0.35	0.63	111	0.32	16.29
T0947TS483_5-D1.pdb	41.94	0.16	0.14	0.7	123	0.34	14.71
T0947TS486_1-D1.pdb	37.1	0.15	0.22	0.63	111	0.33	54.86
T0947TS489_1-D1.pdb	50	0.25	0.07	0.68	119	0.42	14.71
T0947TS495_1-D1.pdb	30.65	0.19	0.19	0.62	108	0.28	54
T0947TS495_2-D1.pdb	29.03	0.11	0.31	0.58	102	0.28	51.29
T0947TS495_3-D1.pdb	29.03	0.06	0.22	0.71	125	0.23	48.71
T0947TS495_4-D1.pdb	32.26	0.19	0.19	0.61	107	0.3	53.29
T0947TS495_5-D1.pdb	30.65	0.2	0.19	0.61	107	0.29	55.71
T0947TS498_1-D1.pdb	37.1	0.21	0.22	0.58	101	0.37	60.29
T0947TS498_2-D1.pdb	30.65	0.1	0.27	0.62	109	0.28	48
T0947TS498_4-D1.pdb	35.48	0.15	0.24	0.61	106	0.33	54.14
T0947TS498_5-D1.pdb	33.87	0.05	0.39	0.55	97	0.35	47.43
T0948TS001_1-D1.pdb	53.7	0.67	0	0.33	49	1.1	18.96
T0948TS004_1-D1.pdb	29.63	0.79	0	0.21	31	0.96	72.65
T0948TS004_2-D1.pdb	27.78	0.71	0	0.29	43	0.65	69.46
T0948TS004_3-D1.pdb	27.78	0.82	0	0.18	27	1.03	75.5
T0948TS004_4-D1.pdb	33.33	0.67	0	0.33	49	0.68	70.13
T0948TS004_5-D1.pdb	31.48	0.7	0	0.3	45	0.7	75.67
T0948TS005_1-D1.pdb	25.93	0.74	0	0.26	39	0.66	71.48
T0948TS005_3-D1.pdb	27.78	0.74	0	0.26	39	0.71	73.49
T0948TS005_4-D1.pdb	22.22	0.7	0	0.3	44	0.51	73.66
T0948TS005_5-D1.pdb	35.19	0.71	0	0.29	43	0.82	20.47
T0948TS011_1-D1.pdb	31.48	0.74	0	0.26	38	0.83	73.83
T0948TS011_2-D1.pdb	29.63	0.74	0	0.26	39	0.76	71.98
T0948TS011_3-D1.pdb	31.48	0.78	0	0.22	33	0.95	74.83
T0948TS011_4-D1.pdb	44.44	0.84	0	0.16	24	1.85	20.3
T0948TS011_5-D1.pdb	42.59	0.8	0	0.2	30	1.42	20.64
T0948TS016_1-D1.pdb	35.19	0.72	0	0.28	41	0.86	18.96
T0948TS017_1-D1.pdb	29.63	0.79	0	0.21	32	0.93	71.64
T0948TS017_2-D1.pdb	29.63	0.78	0	0.22	33	0.9	71.98
T0948TS017_3-D1.pdb	29.63	0.78	0	0.22	33	0.9	71.81
T0948TS017_4-D1.pdb	29.63	0.79	0	0.21	32	0.93	71.81
T0948TS019_1-D1.pdb	40.74	0.91	0	0.09	14	2.91	21.31
T0948TS022_1-D1.pdb	33.33	0.3	0	0.7	104	0.32	14.43

T0948TS022_2-D1.pdb	44.44	0.3	0	0.7	105	0.42	11.58
T0948TS022_3-D1.pdb	42.59	0.36	0	0.64	95	0.45	15.6
T0948TS022_4-D1.pdb	38.89	0.46	0	0.54	80	0.49	12.92
T0948TS022_5-D1.pdb	46.3	0.23	0	0.77	115	0.4	16.44
T0948TS023_1-D1.pdb	37.04	0.77	0	0.23	35	1.06	70.13
T0948TS023_2-D1.pdb	35.19	0.74	0	0.26	38	0.93	71.98
T0948TS023_3-D1.pdb	40.74	0.72	0	0.28	41	0.99	69.13
T0948TS023_4-D1.pdb	37.04	0.77	0	0.23	34	1.09	70.13
T0948TS023_5-D1.pdb	33.33	0.73	0	0.27	40	0.83	70.13
T0948TS026_1-D1.pdb	42.59	0.69	0	0.31	46	0.93	19.13
T0948TS026_2-D1.pdb	44.44	0.63	0	0.37	55	0.81	20.8
T0948TS026_3-D1.pdb	48.15	0.68	0	0.32	48	1	16.61
T0948TS026_4-D1.pdb	40.74	0.66	0	0.34	51	0.8	19.97
T0948TS040_1-D1.pdb	50	0.8	0	0.2	30	1.67	22.99
T0948TS040_2-D1.pdb	57.41	0.8	0	0.2	30	1.91	21.48
T0948TS040_3-D1.pdb	70.37	0.79	0	0.21	32	2.2	20.47
T0948TS040_4-D1.pdb	46.3	0.81	0	0.19	29	1.6	22.82
T0948TS040_5-D1.pdb	77.78	0.79	0	0.21	31	2.51	18.29
T0948TS042_1-D1.pdb	51.85	0.74	0	0.26	38	1.36	25.84
T0948TS042_2-D1.pdb	37.04	0.7	0	0.3	44	0.84	21.14
T0948TS042_3-D1.pdb	35.19	0.71	0	0.29	43	0.82	20.3
T0948TS042_4-D1.pdb	38.89	0.72	0	0.28	41	0.95	19.46
T0948TS042_5-D1.pdb	25.93	0.74	0	0.26	38	0.68	71.64
T0948TS048_1-D1.pdb	25.93	0.68	0	0.32	47	0.55	71.31
T0948TS060_1-D1.pdb	40.74	0.72	0	0.28	42	0.97	72.31
T0948TS060_2-D1.pdb	38.89	0.71	0	0.29	43	0.9	69.63
T0948TS060_3-D1.pdb	33.33	0.72	0	0.28	41	0.81	70.97
T0948TS060_4-D1.pdb	37.04	0.7	0	0.3	45	0.82	70.47
T0948TS060_5-D1.pdb	44.44	0.74	0	0.26	38	1.17	72.82
T0948TS064_1-D1.pdb	27.78	0.77	0	0.23	34	0.82	73.83
T0948TS073_2-D1.pdb	29.63	0.71	0	0.29	43	0.69	69.46
T0948TS073_3-D1.pdb	24.07	0.79	0	0.21	31	0.78	73.15
T0948TS073_4-D1.pdb	31.48	0.69	0	0.31	46	0.68	68.12
T0948TS073_5-D1.pdb	27.78	0.72	0	0.28	42	0.66	69.63
T0948TS077_1-D1.pdb	29.63	0.66	0	0.34	50	0.59	67.78
T0948TS077_2-D1.pdb	31.48	0.68	0	0.32	48	0.66	67.78
T0948TS077_3-D1.pdb	33.33	0.64	0	0.36	53	0.63	66.95
T0948TS077_4-D1.pdb	33.33	0.66	0	0.34	51	0.65	66.78
T0948TS077_5-D1.pdb	29.63	0.66	0	0.34	51	0.58	67.78
T0948TS083_1-D1.pdb	31.48	0.72	0	0.28	41	0.77	19.63
T0948TS083_2-D1.pdb	38.89	0.68	0	0.32	47	0.83	20.3
T0948TS083_3-D1.pdb	33.33	0.76	0	0.24	36	0.93	20.97
T0948TS083_4-D1.pdb	27.78	0.87	0	0.13	19	1.46	22.32
T0948TS083_5-D1.pdb	38.89	0.74	0	0.26	39	1	20.3
T0948TS102_1-D1.pdb	64.81	0.46	0	0.54	81	0.8	17.28
T0948TS102_2-D1.pdb	25.93	0.81	0	0.19	28	0.93	73.66
T0948TS102_4-D1.pdb	31.48	0.79	0	0.21	32	0.98	72.15

T0948TS102_5-D1.pdb	29.63	0.79	0	0.21	32	0.93	75.17
T0948TS119_1-D1.pdb	64.81	0.6	0	0.4	59	1.1	20.97
T0948TS126_1-D1.pdb	38.89	0.73	0	0.27	40	0.97	27.85
T0948TS126_2-D1.pdb	40.74	0.79	0	0.21	32	1.27	23.32
T0948TS126_3-D1.pdb	31.48	0.77	0	0.23	34	0.93	24.66
T0948TS126_4-D1.pdb	37.04	0.79	0	0.21	32	1.16	25.34
T0948TS126_5-D1.pdb	31.48	0.76	0	0.24	36	0.87	27.18
T0948TS145_1-D1.pdb	29.63	0.73	0	0.27	40	0.74	22.65
T0948TS145_2-D1.pdb	27.78	0.72	0	0.28	41	0.68	20.3
T0948TS145_3-D1.pdb	25.93	0.77	0	0.23	35	0.74	25.84
T0948TS145_4-D1.pdb	27.78	0.77	0	0.23	35	0.79	20.13
T0948TS145_5-D1.pdb	25.93	0.78	0	0.22	33	0.79	18.29
T0948TS171_1-D1.pdb	48.15	0.66	0	0.34	51	0.94	55.03
T0948TS171_2-D1.pdb	44.44	0.66	0	0.34	51	0.87	54.2
T0948TS171_3-D1.pdb	50	0.66	0	0.34	51	0.98	54.7
T0948TS171_5-D1.pdb	68.52	0.5	0.01	0.48	72	0.95	27.01
T0948TS173_1-D1.pdb	18.52	0.77	0	0.23	34	0.54	72.48
T0948TS173_2-D1.pdb	18.52	0.73	0	0.27	40	0.46	69.46
T0948TS173_3-D1.pdb	18.52	0.73	0	0.27	40	0.46	74.83
T0948TS173_4-D1.pdb	18.52	0.76	0	0.24	36	0.51	72.82
T0948TS173_5-D1.pdb	24.07	0.75	0	0.25	37	0.65	70.3
T0948TS179_1-D1.pdb	24.07	0.9	0	0.1	15	1.6	72.31
T0948TS179_2-D1.pdb	31.48	0.83	0	0.17	26	1.21	75
T0948TS179_3-D1.pdb	22.22	0.78	0	0.22	33	0.67	71.31
T0948TS179_4-D1.pdb	22.22	0.75	0	0.25	37	0.6	72.48
T0948TS179_5-D1.pdb	29.63	0.82	0	0.18	27	1.1	23.15
T0948TS180_1-D1.pdb	33.33	0.75	0	0.25	37	0.9	26.17
T0948TS180_2-D1.pdb	29.63	0.75	0	0.25	37	0.8	24.5
T0948TS180_3-D1.pdb	38.89	0.66	0	0.34	50	0.78	21.31
T0948TS180_4-D1.pdb	38.89	0.74	0	0.26	38	1.02	19.8
T0948TS180_5-D1.pdb	35.19	0.71	0	0.29	43	0.82	21.81
T0948TS182_1-D1.pdb	35.19	0.64	0	0.36	54	0.65	17.62
T0948TS182_2-D1.pdb	33.33	0.48	0	0.52	77	0.43	19.13
T0948TS182_3-D1.pdb	27.78	0.6	0	0.4	59	0.47	21.48
T0948TS182_4-D1.pdb	37.04	0.46	0	0.54	81	0.46	22.82
T0948TS182_5-D1.pdb	29.63	0.54	0	0.46	69	0.43	20.13
T0948TS183_1-D1.pdb	33.33	0.68	0	0.32	48	0.69	67.95
T0948TS183_3-D1.pdb	31.48	0.89	0	0.11	17	1.85	70.97
T0948TS187_1-D1.pdb	25.93	0.75	0	0.25	37	0.7	73.15
T0948TS187_2-D1.pdb	25.93	0.73	0	0.27	40	0.65	72.31
T0948TS187_3-D1.pdb	22.22	0.74	0	0.26	38	0.58	73.99
T0948TS187_5-D1.pdb	37.04	0.93	0	0.07	10	3.7	19.63
T0948TS188_2-D1.pdb	38.89	0.62	0	0.38	56	0.69	66.28
T0948TS188_3-D1.pdb	42.59	0.7	0	0.3	44	0.97	19.13
T0948TS188_4-D1.pdb	37.04	0.66	0	0.34	50	0.74	19.97
T0948TS188_5-D1.pdb	44.44	0.62	0	0.38	56	0.79	20.8
T0948TS192_1-D1.pdb	42.59	0.66	0	0.34	51	0.84	53.19

T0948TS203_1-D1.pdb	37.04	0.77	0	0.23	34	1.09	74.5
T0948TS203_3-D1.pdb	33.33	0.78	0	0.22	33	1.01	74.33
T0948TS207_1-D1.pdb	59.26	0.52	0	0.48	71	0.83	35.91
T0948TS207_2-D1.pdb	61.11	0.4	0	0.6	90	0.68	19.63
T0948TS207_3-D1.pdb	59.26	0.46	0	0.54	80	0.74	20.64
T0948TS207_4-D1.pdb	59.26	0.48	0	0.52	77	0.77	34.56
T0948TS207_5-D1.pdb	64.81	0.43	0	0.57	85	0.76	18.79
T0948TS220_2-D1.pdb	29.63	0.78	0	0.22	33	0.9	75.5
T0948TS220_3-D1.pdb	33.33	0.79	0	0.21	31	1.08	73.99
T0948TS232_1-D1.pdb	33.33	0.7	0	0.3	45	0.74	70.3
T0948TS236_1-D1.pdb	24.07	0.66	0	0.34	50	0.48	65.77
T0948TS236_3-D1.pdb	33.33	0.65	0	0.35	52	0.64	63.76
T0948TS236_5-D1.pdb	29.63	0.68	0	0.32	48	0.62	64.43
T0948TS239_1-D1.pdb	29.63	0.79	0	0.21	31	0.96	72.82
T0948TS239_2-D1.pdb	27.78	0.78	0	0.22	33	0.84	71.64
T0948TS239_3-D1.pdb	29.63	0.79	0	0.21	32	0.93	71.48
T0948TS239_4-D1.pdb	31.48	0.76	0	0.24	36	0.87	72.82
T0948TS239_5-D1.pdb	33.33	0.79	0	0.21	32	1.04	72.65
T0948TS243_1-D1.pdb	27.78	0.89	0	0.11	16	1.74	74.66
T0948TS243_2-D1.pdb	27.78	0.81	0	0.19	29	0.96	75.17
T0948TS243_3-D1.pdb	27.78	0.8	0	0.2	30	0.93	74.66
T0948TS247_2-D1.pdb	38.89	0.79	0	0.21	32	1.22	34.23
T0948TS247_3-D1.pdb	31.48	0.76	0	0.24	36	0.87	26.17
T0948TS247_4-D1.pdb	27.78	0.77	0	0.23	34	0.82	22.82
T0948TS250_2-D1.pdb	37.04	0.75	0	0.25	37	1	69.63
T0948TS250_3-D1.pdb	35.19	0.74	0	0.26	39	0.9	70.13
T0948TS250_4-D1.pdb	33.33	0.73	0	0.27	40	0.83	70.64
T0948TS250_5-D1.pdb	37.04	0.74	0	0.26	39	0.95	69.63
T0948TS251_1-D1.pdb	35.19	0.68	0	0.32	47	0.75	76.68
T0948TS251_2-D1.pdb	37.04	0.69	0	0.31	46	0.81	71.48
T0948TS251_3-D1.pdb	37.04	0.68	0	0.32	47	0.79	68.79
T0948TS251_4-D1.pdb	37.04	0.68	0	0.32	47	0.79	67.62
T0948TS251_5-D1.pdb	33.33	0.93	0	0.07	10	3.33	20.3
T0948TS252_1-D1.pdb	27.78	0.75	0	0.25	37	0.75	71.31
T0948TS252_2-D1.pdb	27.78	0.77	0	0.23	35	0.79	69.13
T0948TS252_3-D1.pdb	29.63	0.72	0	0.28	41	0.72	66.95
T0948TS252_4-D1.pdb	25.93	0.73	0	0.27	40	0.65	68.79
T0948TS252_5-D1.pdb	24.07	0.75	0	0.25	37	0.65	72.99
T0948TS258_2-D1.pdb	35.19	0.68	0	0.32	47	0.75	68.12
T0948TS258_3-D1.pdb	31.48	0.65	0	0.35	52	0.61	68.29
T0948TS258_4-D1.pdb	25.93	0.68	0	0.32	47	0.55	67.62
T0948TS258_5-D1.pdb	31.48	0.69	0	0.31	46	0.68	67.95
T0948TS275_1-D1.pdb	59.26	0.6	0	0.4	60	0.99	17.11
T0948TS275_2-D1.pdb	62.96	0.64	0	0.36	53	1.19	15.27
T0948TS275_3-D1.pdb	64.81	0.62	0	0.38	57	1.14	16.78
T0948TS275_4-D1.pdb	64.81	0.63	0	0.37	55	1.18	17.62
T0948TS275_5-D1.pdb	79.63	0.66	0	0.34	50	1.59	19.97

T0948TS287_1-D1.pdb	27.78	0.65	0	0.35	52	0.53	64.93
T0948TS287_3-D1.pdb	33.33	0.64	0	0.36	53	0.63	63.76
T0948TS287_5-D1.pdb	29.63	0.66	0	0.34	50	0.59	64.93
T0948TS295_1-D1.pdb	37.04	0.94	0	0.06	9	4.12	20.3
T0948TS295_2-D1.pdb	38.89	0.91	0	0.09	13	2.99	19.46
T0948TS295_3-D1.pdb	33.33	0.9	0	0.1	15	2.22	19.3
T0948TS295_4-D1.pdb	25.93	0.76	0	0.24	36	0.72	70.81
T0948TS295_5-D1.pdb	38.89	0.77	0	0.23	34	1.14	19.8
T0948TS303_1-D1.pdb	29.63	0.77	0	0.23	35	0.85	70.97
T0948TS303_2-D1.pdb	27.78	0.75	0	0.25	37	0.75	72.15
T0948TS303_3-D1.pdb	35.19	0.8	0	0.2	30	1.17	75.67
T0948TS303_4-D1.pdb	29.63	0.74	0	0.26	38	0.78	73.15
T0948TS303_5-D1.pdb	27.78	0.72	0	0.28	42	0.66	73.66
T0948TS313_1-D1.pdb	66.67	0.61	0	0.39	58	1.15	21.31
T0948TS313_2-D1.pdb	61.11	0.61	0	0.39	58	1.05	21.48
T0948TS313_3-D1.pdb	61.11	0.61	0	0.39	58	1.05	21.14
T0948TS313_4-D1.pdb	62.96	0.61	0	0.39	58	1.09	21.48
T0948TS313_5-D1.pdb	59.26	0.61	0	0.39	58	1.02	20.8
T0948TS320_3-D1.pdb	24.07	0.71	0	0.29	43	0.56	68.62
T0948TS321_1-D1.pdb	50	0.76	0	0.24	36	1.39	20.47
T0948TS321_2-D1.pdb	42.59	0.77	0	0.23	34	1.25	23.32
T0948TS321_3-D1.pdb	51.85	0.68	0	0.32	47	1.1	21.48
T0948TS321_4-D1.pdb	37.04	0.74	0	0.26	39	0.95	29.53
T0948TS321_5-D1.pdb	57.41	0.76	0	0.24	36	1.59	30.54
T0948TS324_1-D1.pdb	25.93	0.77	0	0.23	35	0.74	72.65
T0948TS324_2-D1.pdb	27.78	0.77	0	0.23	34	0.82	71.98
T0948TS324_3-D1.pdb	33.33	0.74	0	0.26	38	0.88	73.83
T0948TS324_4-D1.pdb	25.93	0.78	0	0.22	33	0.79	72.99
T0948TS324_5-D1.pdb	25.93	0.77	0	0.23	34	0.76	72.99
T0948TS325_1-D1.pdb	25.93	0.75	0	0.25	37	0.7	72.65
T0948TS325_2-D1.pdb	25.93	0.77	0	0.23	35	0.74	74.5
T0948TS325_3-D1.pdb	44.44	0.77	0	0.23	34	1.31	20.47
T0948TS325_4-D1.pdb	24.07	0.75	0	0.25	37	0.65	69.97
T0948TS325_5-D1.pdb	25.93	0.77	0	0.23	34	0.76	69.97
T0948TS330_1-D1.pdb	31.48	0.73	0	0.27	40	0.79	74.66
T0948TS330_2-D1.pdb	37.04	0.73	0	0.27	40	0.93	20.97
T0948TS330_3-D1.pdb	31.48	0.71	0	0.29	43	0.73	22.99
T0948TS330_4-D1.pdb	29.63	0.77	0	0.23	35	0.85	60.4
T0948TS330_5-D1.pdb	29.63	0.72	0	0.28	41	0.72	59.9
T0948TS345_1-D1.pdb	29.63	0.69	0	0.31	46	0.64	64.26
T0948TS345_2-D1.pdb	31.48	0.66	0	0.34	50	0.63	64.26
T0948TS345_3-D1.pdb	33.33	0.66	0	0.34	51	0.65	64.93
T0948TS345_4-D1.pdb	31.48	0.68	0	0.32	48	0.66	63.59
T0948TS345_5-D1.pdb	29.63	0.66	0	0.34	51	0.58	64.26
T0948TS349_1-D1.pdb	68.52	0.6	0	0.4	59	1.16	20.97
T0948TS357_2-D1.pdb	61.11	0.46	0	0.54	81	0.75	15.6
T0948TS357_3-D1.pdb	61.11	0.4	0	0.6	89	0.69	16.11

T0948TS357_4-D1.pdb	59.26	0.38	0	0.62	92	0.64	15.94
T0948TS357_5-D1.pdb	61.11	0.41	0	0.59	88	0.69	15.77
T0948TS359_1-D1.pdb	50	0.77	0	0.23	34	1.47	18.12
T0948TS382_1-D1.pdb	72.22	0.78	0	0.22	33	2.19	15.6
T0948TS382_2-D1.pdb	74.07	0.8	0	0.2	30	2.47	16.11
T0948TS382_3-D1.pdb	77.78	0.79	0	0.21	31	2.51	16.78
T0948TS382_4-D1.pdb	72.22	0.79	0	0.21	32	2.26	17.95
T0948TS382_5-D1.pdb	81.48	0.79	0	0.21	31	2.63	15.6
T0948TS384_1-D1.pdb	25.93	0.81	0	0.19	28	0.93	73.99
T0948TS384_2-D1.pdb	27.78	0.74	0	0.26	39	0.71	71.98
T0948TS384_4-D1.pdb	29.63	0.88	0	0.12	18	1.65	70.81
T0948TS384_5-D1.pdb	22.22	0.71	0	0.29	43	0.52	73.83
T0948TS393_1-D1.pdb	25.93	0.74	0	0.26	39	0.66	71.31
T0948TS393_2-D1.pdb	27.78	0.74	0	0.26	39	0.71	71.48
T0948TS393_3-D1.pdb	29.63	0.8	0	0.2	30	0.99	74.83
T0948TS393_4-D1.pdb	25.93	0.74	0	0.26	39	0.66	72.31
T0948TS393_5-D1.pdb	22.22	0.71	0	0.29	43	0.52	73.66
T0948TS396_2-D1.pdb	33.33	0.68	0	0.32	48	0.69	68.12
T0948TS396_3-D1.pdb	31.48	0.78	0	0.22	33	0.95	72.31
T0948TS396_4-D1.pdb	25.93	0.77	0	0.23	35	0.74	71.14
T0948TS396_5-D1.pdb	31.48	0.88	0	0.12	18	1.75	70.81
T0948TS399_1-D1.pdb	46.3	0.89	0	0.11	17	2.72	18.12
T0948TS399_2-D1.pdb	57.41	0.88	0	0.12	18	3.19	17.95
T0948TS399_3-D1.pdb	50	0.89	0	0.11	16	3.13	17.45
T0948TS399_4-D1.pdb	61.11	0.89	0	0.11	16	3.82	18.29
T0948TS399_5-D1.pdb	48.15	0.86	0	0.14	21	2.29	19.13
T0948TS405_2-D1.pdb	31.48	0.65	0	0.35	52	0.61	64.26
T0948TS405_4-D1.pdb	35.19	0.65	0	0.35	52	0.68	65.27
T0948TS407_1-D1.pdb	40.74	0.72	0	0.28	41	0.99	20.64
T0948TS407_2-D1.pdb	44.44	0.73	0	0.27	40	1.11	20.64
T0948TS407_3-D1.pdb	48.15	0.72	0	0.28	42	1.15	19.63
T0948TS407_4-D1.pdb	44.44	0.72	0	0.28	42	1.06	19.13
T0948TS407_5-D1.pdb	48.15	0.7	0	0.3	45	1.07	20.47
T0948TS411_1-D1.pdb	31.48	0.77	0	0.23	35	0.9	75
T0948TS411_2-D1.pdb	27.78	0.78	0	0.22	33	0.84	72.48
T0948TS411_3-D1.pdb	29.63	0.8	0	0.2	30	0.99	70.13
T0948TS411_4-D1.pdb	27.78	0.73	0	0.27	40	0.69	74.83
T0948TS411_5-D1.pdb	27.78	0.71	0	0.29	43	0.65	75.17
T0948TS421_2-D1.pdb	33.33	0.42	0	0.58	87	0.38	21.98
T0948TS421_3-D1.pdb	29.63	0.7	0	0.3	44	0.67	21.81
T0948TS421_5-D1.pdb	25.93	0.68	0	0.32	48	0.54	21.81
T0948TS425_1-D1.pdb	31.48	0.66	0	0.34	50	0.63	67.28
T0948TS425_2-D1.pdb	29.63	0.66	0	0.34	50	0.59	67.28
T0948TS425_3-D1.pdb	24.07	0.67	0	0.33	49	0.49	67.11
T0948TS425_4-D1.pdb	25.93	0.66	0	0.34	50	0.52	67.62
T0948TS425_5-D1.pdb	25.93	0.66	0	0.34	50	0.52	68.29
T0948TS434_1-D1.pdb	31.48	0.78	0	0.22	33	0.95	22.15

T0948TS434_2-D1.pdb	33.33	0.84	0	0.16	24	1.39	24.66
T0948TS434_3-D1.pdb	38.89	0.79	0	0.21	31	1.25	22.99
T0948TS434_4-D1.pdb	46.3	0.74	0	0.26	38	1.22	25.67
T0948TS434_5-D1.pdb	35.19	0.78	0	0.22	33	1.07	24.5
T0948TS439_1-D1.pdb	25.93	0.77	0	0.23	34	0.76	72.15
T0948TS439_2-D1.pdb	27.78	0.79	0	0.21	32	0.87	72.31
T0948TS439_3-D1.pdb	35.19	0.73	0	0.27	40	0.88	71.31
T0948TS439_4-D1.pdb	35.19	0.68	0	0.32	47	0.75	72.31
T0948TS439_5-D1.pdb	37.04	0.72	0	0.28	41	0.9	20.64
T0948TS441_1-D1.pdb	24.07	0.72	0	0.28	41	0.59	76.01
T0948TS441_2-D1.pdb	24.07	0.7	0	0.3	45	0.53	70.47
T0948TS441_4-D1.pdb	18.52	0.75	0	0.25	37	0.5	70.13
T0948TS441_5-D1.pdb	24.07	0.71	0	0.29	43	0.56	72.65
T0948TS443_1-D1.pdb	48.15	0.82	0	0.18	27	1.78	21.64
T0948TS443_2-D1.pdb	25.93	0.76	0	0.24	36	0.72	62.08
T0948TS443_3-D1.pdb	42.59	0.81	0	0.19	28	1.52	28.19
T0948TS443_4-D1.pdb	44.44	0.82	0	0.18	27	1.65	28.69
T0948TS443_5-D1.pdb	37.04	0.77	0	0.23	35	1.06	39.09
T0948TS444_1-D1.pdb	27.78	0.5	0	0.5	74	0.38	43.96
T0948TS444_2-D1.pdb	33.33	0.59	0	0.41	61	0.55	44.46
T0948TS444_3-D1.pdb	25.93	0.52	0	0.48	71	0.37	42.95
T0948TS444_4-D1.pdb	35.19	0.56	0	0.44	66	0.53	44.63
T0948TS444_5-D1.pdb	38.89	0.56	0	0.44	65	0.6	44.3
T0948TS446_1-D1.pdb	35.19	0.7	0	0.3	44	0.8	42.62
T0948TS446_2-D1.pdb	38.89	0.7	0	0.3	45	0.86	43.96
T0948TS450_1-D1.pdb	29.63	0.81	0	0.19	28	1.06	72.65
T0948TS450_2-D1.pdb	25.93	0.77	0	0.23	34	0.76	76.51
T0948TS450_3-D1.pdb	29.63	0.79	0	0.21	31	0.96	72.15
T0948TS450_4-D1.pdb	24.07	0.79	0	0.21	32	0.75	75.5
T0948TS450_5-D1.pdb	25.93	0.76	0	0.24	36	0.72	73.83
T0948TS451_1-D1.pdb	42.59	0.67	0	0.33	49	0.87	34.23
T0948TS451_2-D1.pdb	44.44	0.68	0	0.32	47	0.95	34.06
T0948TS451_3-D1.pdb	40.74	0.66	0	0.34	51	0.8	34.9
T0948TS451_4-D1.pdb	38.89	0.66	0	0.34	50	0.78	28.86
T0948TS451_5-D1.pdb	48.15	0.66	0	0.34	50	0.96	32.55
T0948TS452_1-D1.pdb	33.33	0.7	0	0.3	44	0.76	16.78
T0948TS452_2-D1.pdb	40.74	0.77	0	0.23	35	1.16	18.79
T0948TS452_3-D1.pdb	38.89	0.8	0	0.2	30	1.3	22.32
T0948TS452_4-D1.pdb	35.19	0.79	0	0.21	32	1.1	19.8
T0948TS452_5-D1.pdb	38.89	0.77	0	0.23	35	1.11	20.3
T0948TS455_1-D1.pdb	53.7	0.29	0	0.71	106	0.51	14.09
T0948TS455_2-D1.pdb	48.15	0.21	0	0.79	118	0.41	15.77
T0948TS455_3-D1.pdb	50	0.36	0	0.64	96	0.52	14.43
T0948TS455_4-D1.pdb	62.96	0.17	0	0.83	123	0.51	15.27
T0948TS455_5-D1.pdb	46.3	0.32	0	0.68	102	0.45	15.94
T0948TS456_1-D1.pdb	25.93	0.77	0	0.23	34	0.76	71.31
T0948TS456_2-D1.pdb	24.07	0.74	0	0.26	38	0.63	72.82

T0948TS456_3-D1.pdb	25.93	0.74	0	0.26	38	0.68	68.79
T0948TS456_4-D1.pdb	27.78	0.75	0	0.25	37	0.75	69.3
T0948TS456_5-D1.pdb	27.78	0.69	0	0.31	46	0.6	73.15
T0948TS464_1-D1.pdb	57.41	0.62	0	0.38	57	1.01	16.78
T0948TS464_2-D1.pdb	70.37	0.52	0	0.48	72	0.98	15.6
T0948TS464_3-D1.pdb	81.48	0.64	0	0.36	54	1.51	15.6
T0948TS464_4-D1.pdb	62.96	0.59	0	0.41	61	1.03	16.78
T0948TS464_5-D1.pdb	81.48	0.7	0	0.3	44	1.85	16.44
T0948TS467_1-D1.pdb	53.7	0.79	0	0.21	32	1.68	22.15
T0948TS467_2-D1.pdb	46.3	0.62	0	0.38	56	0.83	18.62
T0948TS467_3-D1.pdb	51.85	0.68	0	0.32	47	1.1	20.13
T0948TS467_4-D1.pdb	42.59	0.77	0	0.23	34	1.25	19.3
T0948TS467_5-D1.pdb	38.89	0.71	0	0.29	43	0.9	18.29
T0948TS474_1-D1.pdb	31.48	0.7	0	0.3	44	0.72	65.44
T0948TS474_2-D1.pdb	29.63	0.71	0	0.29	43	0.69	70.47
T0948TS475_1-D1.pdb	29.63	0.77	0	0.23	35	0.85	72.99
T0948TS475_2-D1.pdb	27.78	0.71	0	0.29	43	0.65	71.64
T0948TS475_3-D1.pdb	27.78	0.68	0	0.32	47	0.59	69.46
T0948TS475_4-D1.pdb	22.22	0.74	0	0.26	39	0.57	72.48
T0948TS475_5-D1.pdb	24.07	0.73	0	0.27	40	0.6	70.13
T0948TS479_2-D1.pdb	25.93	0.77	0	0.23	34	0.76	70.81
T0948TS479_4-D1.pdb	33.33	0.7	0	0.3	44	0.76	68.12
T0948TS480_1-D1.pdb	33.33	0.75	0	0.25	37	0.9	66.11
T0948TS480_2-D1.pdb	29.63	0.61	0	0.39	58	0.51	65.6
T0948TS480_3-D1.pdb	25.93	0.74	0	0.26	38	0.68	66.78
T0948TS480_5-D1.pdb	35.19	0.74	0	0.26	38	0.93	65.94
T0948TS483_1-D1.pdb	59.26	0.79	0	0.21	32	1.85	22.15
T0948TS483_2-D1.pdb	42.59	0.76	0	0.24	36	1.18	21.48
T0948TS483_3-D1.pdb	46.3	0.76	0	0.24	36	1.29	18.62
T0948TS483_4-D1.pdb	44.44	0.66	0	0.34	51	0.87	20.3
T0948TS483_5-D1.pdb	42.59	0.68	0	0.32	47	0.91	18.29
T0948TS486_5-D1.pdb	42.59	0.64	0	0.36	54	0.79	20.47
T0948TS489_1-D1.pdb	40.74	0.73	0	0.27	40	1.02	22.32
T0948TS498_1-D1.pdb	25.93	0.78	0	0.22	33	0.79	75.5
T0948TS498_3-D1.pdb	25.93	0.73	0	0.27	40	0.65	71.48
T0948TS498_5-D1.pdb	31.48	0.65	0	0.35	52	0.61	65.27

**CAMEO**							
5MM8_A-servers-server11-model-1	28.44	0.08	0.34	0.57	117	0.24	91.05
5MM8_A-servers-server11-model-2	28.44	0.08	0.35	0.57	116	0.25	91.42
5MM8_A-servers-server11-model-3	29.36	0.08	0.35	0.57	116	0.25	90.56
5MM8_A-servers-server11-model-4	30.28	0.09	0.35	0.56	114	0.27	90.32
5MM8_A-servers-server11-model-5	28.44	0.08	0.35	0.56	115	0.25	91.30
5MM8_A-servers-server20-model-1	27.52	0.07	0.31	0.62	125	0.22	88.97
5MM8_A-servers-server20-model-2	35.78	0.06	0.40	0.54	107	0.33	51.47
5MM8_A-servers-server22-model-1	26.61	0.10	0.33	0.57	116	0.23	89.22
5MM8_A-servers-server30-model-1	27.52	0.09	0.32	0.59	121	0.23	89.46

5MM8_A-servers-server30-model-2	28.44	0.11	0.31	0.57	117	0.24	84.19
5MM8_A-servers-server30-model-3	30.28	0.09	0.32	0.59	120	0.25	84.56
5MM8_A-servers-server30-model-4	26.61	0.09	0.34	0.57	116	0.23	84.31
5MM8_A-servers-server30-model-5	29.36	0.07	0.27	0.66	135	0.22	73.78
5MM8_A-servers-server33-model-1	24.77	0.09	0.33	0.58	118	0.21	91.18
5MM8_A-servers-server33-model-2	27.52	0.10	0.35	0.55	113	0.24	90.56
5MM8_A-servers-server33-model-3	27.52	0.10	0.35	0.55	113	0.24	90.56
5MM8_A-servers-server33-model-4	27.52	0.10	0.35	0.55	113	0.24	90.56
5MM8_A-servers-server33-model-5	27.52	0.10	0.35	0.55	113	0.24	90.56
5MM8_A-servers-server36-model-1	26.61	0.08	0.33	0.58	117	0.23	88.60
5MM8_A-servers-server4-model-1	29.36	0.08	0.29	0.63	128	0.23	90.81
5MM8_A-servers-server61-model-1	30.28	0.09	0.34	0.57	115	0.26	88.85
5MM8_A-servers-server61-model-2	30.28	0.09	0.34	0.57	115	0.26	88.85
5MM8_A-servers-server62-model-1	28.44	0.09	0.34	0.57	115	0.25	89.58
5MM8_A-servers-server62-model-2	28.44	0.09	0.34	0.57	115	0.25	89.58
5MM8_A-servers-server63-model-1	28.44	0.10	0.33	0.57	116	0.25	82.97
5MM8_A-servers-server63-model-2	28.44	0.10	0.33	0.57	116	0.25	82.97
5MM8_A-servers-server64-model-1	28.44	0.10	0.33	0.57	116	0.25	82.97
5MM8_A-servers-server64-model-2	28.44	0.10	0.33	0.57	116	0.25	82.97
5MM8_A-servers-server65-model-1	30.28	0.09	0.34	0.57	115	0.26	88.85
5MM8_A-servers-server65-model-2	30.28	0.09	0.34	0.57	115	0.26	88.85
5MM8_A-servers-server70-model-1	28.44	0.09	0.33	0.58	117	0.24	89.58
5NVA_A-servers-server11-model-1	25.79	0.74	0.00	0.26	128	0.20	57.51
5NVA_A-servers-server11-model-2	25.53	0.72	0.00	0.28	139	0.18	57.21
5NVA_A-servers-server11-model-3	25.26	0.76	0.00	0.24	121	0.21	58.52
5NVA_A-servers-server11-model-4	24.74	0.74	0.00	0.26	131	0.19	57.76
5NVA_A-servers-server11-model-5	27.11	0.76	0.00	0.24	120	0.23	56.55
5NVA_A-servers-server17-model-1	33.78	0.76	0.00	0.24	114	0.30	47.43
5NVA_A-servers-server20-model-1	32.62	0.69	0.00	0.31	148	0.22	49.55
5NVA_A-servers-server20-model-2	91.30	0.58	0.00	0.42	16	5.71	4.44
5NVA_A-servers-server22-model-1	29.74	0.73	0.00	0.27	136	0.22	52.32
5NVA_A-servers-server36-model-1	32.84	0.72	0.00	0.28	123	0.27	43.04
5NVA_A-servers-server4-model-1	32.89	0.67	0.00	0.33	166	0.20	49.70
5NVA_A-servers-server61-model-1	30.83	0.70	0.00	0.30	146	0.21	48.19
5NVA_A-servers-server61-model-2	30.83	0.70	0.00	0.30	146	0.21	48.19
5NVA_A-servers-server62-model-1	30.38	0.68	0.00	0.32	150	0.20	44.10
5NVA_A-servers-server62-model-2	30.38	0.68	0.00	0.32	150	0.20	44.10
5NVA_A-servers-server63-model-1	31.72	0.72	0.00	0.28	133	0.24	45.72
5NVA_A-servers-server63-model-2	31.72	0.72	0.00	0.28	133	0.24	45.72
5NVA_A-servers-server64-model-1	30.83	0.70	0.00	0.30	146	0.21	48.19
5NVA_A-servers-server64-model-2	30.83	0.70	0.00	0.30	146	0.21	48.19
5NVA_A-servers-server65-model-1	30.83	0.70	0.00	0.30	146	0.21	48.19
5NVA_A-servers-server65-model-2	30.83	0.70	0.00	0.30	146	0.21	48.19
5NVA_A-servers-server70-model-1	33.42	0.71	0.00	0.29	143	0.23	49.45
5O6C_A-servers-server11-model-1	30.34	0.41	0.07	0.52	137	0.22	17.68
5O6C_A-servers-server11-model-2	37.08	0.38	0.06	0.56	146	0.25	17.11
5O6C_A-servers-server11-model-3	25.84	0.39	0.07	0.54	141	0.18	18.54

5O6C_A-servers-server11-model-4	33.71	0.41	0.06	0.53	140	0.24	14.26
5O6C_A-servers-server11-model-5	26.97	0.37	0.08	0.55	145	0.19	13.69
5O6C_A-servers-server17-model-1	66.67	0.28	0.07	0.65	139	0.48	13.69
5O6C_A-servers-server20-model-1	49.09	0.26	0.04	0.71	97	0.51	13.50
5O6C_A-servers-server20-model-2	48.78	0.20	0.05	0.75	63	0.77	15.11
5O6C_A-servers-server22-model-1	42.70	0.29	0.08	0.64	168	0.25	17.21
5O6C_A-servers-server30-model-1	58.43	0.33	0.02	0.66	173	0.34	17.21
5O6C_A-servers-server30-model-2	31.46	0.37	0.02	0.62	163	0.19	12.07
5O6C_A-servers-server30-model-3	39.33	0.32	0.03	0.65	170	0.23	12.26
5O6C_A-servers-server30-model-4	35.96	0.31	0.02	0.67	177	0.20	9.22
5O6C_A-servers-server30-model-5	34.83	0.17	0.05	0.78	206	0.17	16.07
5O6C_A-servers-server36-model-1	55.88	0.20	0.00	0.80	52	1.07	13.02
5O6C_A-servers-server4-model-1	48.31	0.15	0.08	0.77	203	0.24	16.16
5O6C_A-servers-server58-model-1	44.94	0.22	0.02	0.76	201	0.22	13.12
5O6C_A-servers-server58-model-2	39.33	0.13	0.02	0.86	225	0.17	12.17
5O6C_A-servers-server58-model-3	49.44	0.27	0.03	0.70	183	0.27	14.54
5O6C_A-servers-server58-model-4	43.82	0.15	0.02	0.83	219	0.20	13.31
5O6C_A-servers-server58-model-5	46.07	0.13	0.03	0.85	223	0.21	12.45
5O6C_A-servers-server61-model-1	60.98	0.18	0.06	0.76	55	1.11	13.97
5O6C_A-servers-server61-model-2	60.98	0.18	0.06	0.76	55	1.11	13.97
5O6C_A-servers-server62-model-1	63.89	0.14	0.09	0.77	50	1.28	15.30
5O6C_A-servers-server62-model-2	63.89	0.14	0.09	0.77	50	1.28	15.30
5O6C_A-servers-server63-model-1	88.57	0.15	0.00	0.85	149	0.59	5.80
5O6C_A-servers-server63-model-2	88.57	0.15	0.00	0.85	149	0.59	5.80
5O6C_A-servers-server64-model-1	90.24	0.71	0.00	0.29	28	3.22	10.65
5O6C_A-servers-server64-model-2	90.24	0.71	0.00	0.29	28	3.22	10.65
5O6C_A-servers-server65-model-1	60.98	0.18	0.06	0.76	55	1.11	13.97
5O6C_A-servers-server65-model-2	60.98	0.18	0.06	0.76	55	1.11	13.97
5O6C_A-servers-server70-model-1	81.25	0.31	0.00	0.69	52	1.56	6.08
5OJY_A-servers-server11-model-1	23.94	0.61	0.00	0.39	97	0.25	60.04
5OJY_A-servers-server11-model-2	25.35	0.62	0.00	0.38	94	0.27	64.17
5OJY_A-servers-server11-model-3	29.58	0.62	0.00	0.38	94	0.31	63.48
5OJY_A-servers-server11-model-4	26.76	0.61	0.00	0.39	96	0.28	64.67
5OJY_A-servers-server11-model-5	29.58	0.65	0.00	0.35	86	0.34	62.30
5OJY_A-servers-server17-model-1	33.80	0.63	0.00	0.37	84	0.40	62.11
5OJY_A-servers-server20-model-1	32.39	0.55	0.00	0.45	100	0.32	62.80
5OJY_A-servers-server22-model-1	30.99	0.66	0.00	0.34	87	0.36	64.27
5OJY_A-servers-server30-model-1	30.99	0.61	0.01	0.38	97	0.32	61.12
5OJY_A-servers-server36-model-1	35.21	0.63	0.00	0.37	83	0.42	57.68
5OJY_A-servers-server4-model-1	32.39	0.59	0.00	0.41	104	0.31	64.27
5OJY_A-servers-server61-model-1	32.39	0.65	0.00	0.35	80	0.40	62.60
5OJY_A-servers-server61-model-2	32.39	0.65	0.00	0.35	80	0.40	62.60
5OJY_A-servers-server62-model-1	29.23	0.65	0.00	0.35	76	0.38	59.55
5OJY_A-servers-server62-model-2	29.23	0.65	0.00	0.35	76	0.38	59.55
5OJY_A-servers-server63-model-1	28.17	0.61	0.00	0.39	82	0.34	43.60
5OJY_A-servers-server63-model-2	28.17	0.61	0.00	0.39	82	0.34	43.60
5OJY_A-servers-server64-model-1	35.21	0.59	0.00	0.41	90	0.39	38.98

5OJY_A-servers-server64-model-2	35.21	0.59	0.00	0.41	90	0.39	38.98
5OJY_A-servers-server65-model-1	32.39	0.65	0.00	0.35	80	0.40	62.60
5OJY_A-servers-server65-model-2	32.39	0.65	0.00	0.35	80	0.40	62.60
5OJY_A-servers-server70-model-1	33.80	0.64	0.00	0.36	84	0.40	62.21
5OUN_A-servers-server11-model-1	31.43	0.08	0.40	0.51	55	0.57	60.51
5OUN_A-servers-server11-model-2	31.43	0.12	0.40	0.48	51	0.62	61.92
5OUN_A-servers-server11-model-3	28.57	0.10	0.38	0.51	55	0.52	61.68
5OUN_A-servers-server11-model-4	31.43	0.13	0.40	0.47	50	0.63	62.15
5OUN_A-servers-server11-model-5	31.43	0.10	0.40	0.50	53	0.59	61.68
5OUN_A-servers-server20-model-1	34.29	0.09	0.22	0.70	71	0.48	46.26
5OUN_A-servers-server22-model-1	28.57	0.20	0.27	0.53	57	0.50	56.08
5OUN_A-servers-server30-model-1	31.43	0.08	0.32	0.60	64	0.49	59.58
5OUN_A-servers-server30-model-2	45.71	0.04	0.37	0.59	63	0.73	33.18
5OUN_A-servers-server30-model-3	37.14	0.10	0.35	0.55	59	0.63	61.92
5OUN_A-servers-server30-model-4	37.14	0.12	0.15	0.73	78	0.48	22.43
5OUN_A-servers-server30-model-5	37.14	0.08	0.20	0.72	77	0.48	18.69
5OUN_A-servers-server33-model-1	28.57	0.10	0.39	0.50	54	0.53	61.92
5OUN_A-servers-server33-model-2	28.57	0.10	0.39	0.50	54	0.53	61.92
5OUN_A-servers-server33-model-3	28.57	0.10	0.39	0.50	54	0.53	61.92
5OUN_A-servers-server33-model-4	28.57	0.10	0.39	0.50	54	0.53	61.92
5OUN_A-servers-server33-model-5	28.57	0.10	0.39	0.50	54	0.53	61.92
5OUN_A-servers-server36-model-1	25.71	0.09	0.28	0.63	64	0.40	55.14
5OUN_A-servers-server4-model-1	28.57	0.07	0.32	0.61	65	0.44	62.85
5OUN_A-servers-server58-model-1	28.57	0.08	0.37	0.54	58	0.49	61.45
5OUN_A-servers-server58-model-2	25.71	0.08	0.39	0.52	56	0.46	62.38
5OUN_A-servers-server58-model-3	25.71	0.08	0.39	0.52	56	0.46	62.38
5OUN_A-servers-server58-model-4	25.71	0.08	0.39	0.52	56	0.46	62.38
5OUN_A-servers-server58-model-5	31.43	0.10	0.36	0.53	57	0.55	60.75
5OUN_A-servers-server61-model-1	31.43	0.08	0.25	0.66	71	0.44	57.24
5OUN_A-servers-server61-model-2	31.43	0.08	0.25	0.66	71	0.44	57.24
5OUN_A-servers-server62-model-1	40.00	0.09	0.15	0.77	79	0.51	46.96
5OUN_A-servers-server62-model-2	40.00	0.09	0.15	0.77	79	0.51	46.96
5OUN_A-servers-server63-model-1	39.39	0.10	0.38	0.52	46	0.86	59.11
5OUN_A-servers-server63-model-2	39.39	0.10	0.38	0.52	46	0.86	59.11
5OUN_A-servers-server64-model-1	39.39	0.10	0.38	0.52	46	0.86	59.58
5OUN_A-servers-server64-model-2	39.39	0.10	0.38	0.52	46	0.86	59.58
5OUN_A-servers-server65-model-1	31.43	0.08	0.25	0.66	71	0.44	57.24
5OUN_A-servers-server65-model-2	31.43	0.08	0.25	0.66	71	0.44	57.24
5OUN_A-servers-server70-model-1	34.29	0.08	0.30	0.62	66	0.52	55.61
5OVY_A-servers-server11-model-1	30.00	0.68	0.00	0.32	72	0.42	47.07
5OVY_A-servers-server11-model-2	30.00	0.66	0.00	0.34	76	0.39	49.89
5OVY_A-servers-server11-model-3	38.75	0.67	0.00	0.33	73	0.53	46.40
5OVY_A-servers-server11-model-4	36.25	0.69	0.00	0.31	68	0.53	47.86
5OVY_A-servers-server11-model-5	35.00	0.67	0.00	0.33	73	0.48	49.66
5OVY_A-servers-server20-model-1	35.90	0.67	0.00	0.33	61	0.59	39.41
5OVY_A-servers-server22-model-1	21.25	0.69	0.00	0.31	68	0.31	46.62
5OVY_A-servers-server30-model-1	33.75	0.66	0.00	0.34	75	0.45	45.16

5OVY_A-servers-server30-model-2	35.00	0.62	0.00	0.38	85	0.41	46.40
5OVY_A-servers-server30-model-3	33.75	0.59	0.00	0.41	90	0.38	57.32
5OVY_A-servers-server30-model-4	32.50	0.56	0.00	0.44	98	0.33	49.55
5OVY_A-servers-server30-model-5	30.00	0.63	0.00	0.37	83	0.36	39.08
5OVY_A-servers-server33-model-1	37.50	0.61	0.00	0.39	87	0.43	52.93
5OVY_A-servers-server33-model-2	37.50	0.61	0.00	0.39	87	0.43	52.93
5OVY_A-servers-server33-model-3	37.50	0.61	0.00	0.39	87	0.43	52.93
5OVY_A-servers-server33-model-4	37.50	0.61	0.00	0.39	87	0.43	52.93
5OVY_A-servers-server33-model-5	37.50	0.61	0.00	0.39	87	0.43	52.93
5OVY_A-servers-server36-model-1	28.21	0.74	0.00	0.26	45	0.63	42.79
5OVY_A-servers-server4-model-1	37.50	0.62	0.00	0.38	85	0.44	46.85
5OVY_A-servers-server61-model-1	34.62	0.71	0.00	0.29	50	0.69	43.24
5OVY_A-servers-server61-model-2	34.62	0.71	0.00	0.29	50	0.69	43.24
5OVY_A-servers-server62-model-1	37.18	0.72	0.00	0.28	49	0.76	43.47
5OVY_A-servers-server62-model-2	37.18	0.72	0.00	0.28	49	0.76	43.47
5OVY_A-servers-server63-model-1	37.66	0.56	0.00	0.44	75	0.50	40.20
5OVY_A-servers-server63-model-2	37.66	0.56	0.00	0.44	75	0.50	40.20
5OVY_A-servers-server64-model-1	52.50	0.43	0.00	0.57	118	0.44	29.73
5OVY_A-servers-server64-model-2	52.50	0.43	0.00	0.57	118	0.44	29.73
5OVY_A-servers-server65-model-1	34.62	0.71	0.00	0.29	50	0.69	43.24
5OVY_A-servers-server65-model-2	34.62	0.71	0.00	0.29	50	0.69	43.24
5OVY_A-servers-server70-model-1	33.33	0.75	0.00	0.25	45	0.74	49.32
5TOS_B-servers-server11-model-1	28.03	0.32	0.11	0.58	228	0.12	53.61
5TOS_B-servers-server11-model-2	19.70	0.33	0.10	0.57	227	0.09	54.62
5TOS_B-servers-server11-model-3	26.52	0.30	0.10	0.59	235	0.11	54.37
5TOS_B-servers-server11-model-4	22.73	0.32	0.10	0.58	228	0.10	54.56
5TOS_B-servers-server11-model-5	23.48	0.32	0.10	0.58	230	0.10	54.24
5TOS_B-servers-server17-model-1	30.36	0.26	0.06	0.68	183	0.17	33.61
5TOS_B-servers-server20-model-1	26.19	0.35	0.13	0.51	153	0.17	45.76
5TOS_B-servers-server22-model-1	25.00	0.39	0.10	0.51	202	0.12	50.89
5TOS_B-servers-server30-model-1	22.73	0.27	0.06	0.67	263	0.09	47.15
5TOS_B-servers-server30-model-2	24.24	0.27	0.07	0.66	259	0.09	52.53
5TOS_B-servers-server30-model-3	24.24	0.30	0.09	0.60	238	0.10	50.51
5TOS_B-servers-server30-model-4	25.76	0.12	0.06	0.81	321	0.08	48.17
5TOS_B-servers-server30-model-5	25.00	0.30	0.10	0.60	238	0.11	46.39
5TOS_B-servers-server36-model-1	28.57	0.33	0.09	0.58	172	0.17	48.42
5TOS_B-servers-server4-model-1	27.27	0.29	0.09	0.61	242	0.11	55.13
5TOS_B-servers-server58-model-1	26.52	0.28	0.09	0.64	251	0.11	52.15
5TOS_B-servers-server58-model-2	31.06	0.29	0.08	0.63	250	0.12	50.51
5TOS_B-servers-server58-model-3	31.06	0.29	0.08	0.63	250	0.12	50.51
5TOS_B-servers-server58-model-4	31.06	0.29	0.08	0.63	250	0.12	50.51
5TOS_B-servers-server58-model-5	31.06	0.29	0.08	0.63	250	0.12	50.51
5TOS_B-servers-server61-model-1	24.60	0.38	0.11	0.51	159	0.15	45.63
5TOS_B-servers-server61-model-2	24.60	0.38	0.11	0.51	159	0.15	45.63
5TOS_B-servers-server62-model-1	26.98	0.36	0.12	0.52	162	0.17	46.39
5TOS_B-servers-server62-model-2	26.98	0.36	0.12	0.52	162	0.17	46.39
5TOS_B-servers-server63-model-1	31.82	0.27	0.12	0.61	215	0.15	40.06

5TOS_B-servers-server63-model-2	31.82	0.27	0.12	0.61	215	0.15	40.06
5TOS_B-servers-server64-model-1	29.55	0.26	0.11	0.63	224	0.13	39.87
5TOS_B-servers-server64-model-2	29.55	0.26	0.11	0.63	224	0.13	39.87
5TOS_B-servers-server65-model-1	24.60	0.38	0.11	0.51	159	0.15	45.63
5TOS_B-servers-server65-model-2	24.60	0.38	0.11	0.51	159	0.15	45.63
5TOS_B-servers-server70-model-1	32.54	0.35	0.13	0.52	158	0.21	52.66
5TXR_A-servers-server11-model-1	25.81	0.37	0.09	0.54	263	0.10	76.07
5TXR_A-servers-server11-model-2	27.19	0.37	0.10	0.53	262	0.10	75.76
5TXR_A-servers-server11-model-3	27.19	0.38	0.10	0.53	258	0.11	76.17
5TXR_A-servers-server11-model-4	27.65	0.37	0.09	0.54	264	0.10	76.12
5TXR_A-servers-server11-model-5	26.73	0.38	0.10	0.52	254	0.11	77.34
5TXR_A-servers-server20-model-1	26.40	0.39	0.11	0.50	205	0.13	74.75
5TXR_A-servers-server22-model-1	26.73	0.42	0.09	0.49	240	0.11	76.07
5TXR_A-servers-server30-model-1	23.50	0.36	0.10	0.55	268	0.09	75.15
5TXR_A-servers-server30-model-2	27.19	0.34	0.10	0.56	276	0.10	63.75
5TXR_A-servers-server30-model-3	25.35	0.37	0.10	0.53	262	0.10	65.68
5TXR_A-servers-server30-model-4	25.81	0.37	0.09	0.54	266	0.10	65.28
5TXR_A-servers-server30-model-5	25.35	0.37	0.10	0.53	258	0.10	63.90
5TXR_A-servers-server33-model-1	32.72	0.33	0.09	0.58	284	0.12	75.61
5TXR_A-servers-server33-model-2	32.72	0.33	0.09	0.57	282	0.12	72.76
5TXR_A-servers-server33-model-3	32.72	0.33	0.09	0.57	282	0.12	72.76
5TXR_A-servers-server33-model-4	29.03	0.33	0.09	0.57	282	0.10	75.51
5TXR_A-servers-server33-model-5	32.26	0.34	0.08	0.58	286	0.11	74.80
5TXR_A-servers-server36-model-1	26.90	0.39	0.11	0.49	202	0.13	73.78
5TXR_A-servers-server4-model-1	28.11	0.32	0.11	0.57	281	0.10	73.27
5TXR_A-servers-server61-model-1	29.65	0.40	0.11	0.50	206	0.14	75.26
5TXR_A-servers-server61-model-2	29.65	0.40	0.11	0.50	206	0.14	75.26
5TXR_A-servers-server62-model-1	28.14	0.39	0.11	0.50	208	0.14	75.10
5TXR_A-servers-server62-model-2	28.14	0.39	0.11	0.50	208	0.14	75.10
5TXR_A-servers-server65-model-1	29.65	0.40	0.11	0.50	206	0.14	75.26
5TXR_A-servers-server65-model-2	29.65	0.40	0.11	0.50	206	0.14	75.26
5TXR_A-servers-server70-model-1	26.63	0.39	0.09	0.52	215	0.12	74.08
5U7Z_C-servers-server11-model-1	40.58	0.55	0.00	0.45	54	0.75	45.16
5U7Z_C-servers-server11-model-2	37.68	0.55	0.00	0.45	54	0.70	45.16
5U7Z_C-servers-server11-model-3	40.58	0.59	0.00	0.41	50	0.81	44.19
5U7Z_C-servers-server11-model-4	36.23	0.57	0.00	0.43	52	0.70	46.32
5U7Z_C-servers-server11-model-5	49.28	0.55	0.00	0.45	55	0.90	23.45
5U7Z_C-servers-server20-model-1	45.16	0.71	0.00	0.29	26	1.74	46.51
5U7Z_C-servers-server20-model-2	69.09	0.32	0.00	0.68	65	1.06	15.50
5U7Z_C-servers-server20-model-3	81.40	0.62	0.00	0.38	26	3.13	15.31
5U7Z_C-servers-server22-model-1	39.13	0.53	0.00	0.47	60	0.65	47.09
5U7Z_C-servers-server30-model-1	49.28	0.54	0.00	0.46	59	0.84	47.67
5U7Z_C-servers-server30-model-2	63.77	0.41	0.00	0.59	76	0.84	19.19
5U7Z_C-servers-server30-model-3	56.52	0.45	0.00	0.55	71	0.80	20.54
5U7Z_C-servers-server30-model-4	56.52	0.49	0.00	0.51	66	0.86	23.45
5U7Z_C-servers-server30-model-5	49.28	0.49	0.00	0.51	66	0.75	22.29
5U7Z_C-servers-server33-model-1	55.07	0.51	0.00	0.49	63	0.87	48.64

5U7Z_C-servers-server33-model-2	55.07	0.51	0.00	0.49	63	0.87	48.64
5U7Z_C-servers-server33-model-3	50.72	0.48	0.00	0.52	67	0.76	49.03
5U7Z_C-servers-server33-model-4	50.72	0.48	0.00	0.52	67	0.76	49.03
5U7Z_C-servers-server33-model-5	50.72	0.48	0.00	0.52	67	0.76	49.03
5U7Z_C-servers-server36-model-1	56.82	0.08	0.18	0.74	48	1.18	13.76
5U7Z_C-servers-server4-model-1	55.07	0.41	0.00	0.59	76	0.72	48.06
5U7Z_C-servers-server61-model-1	43.48	0.02	0.13	0.86	108	0.40	13.18
5U7Z_C-servers-server61-model-2	43.48	0.02	0.13	0.86	108	0.40	13.18
5U7Z_C-servers-server62-model-1	52.38	0.02	0.10	0.89	109	0.48	12.02
5U7Z_C-servers-server62-model-2	52.38	0.02	0.10	0.89	109	0.48	12.02
5U7Z_C-servers-server63-model-1	60.00	0.22	0.16	0.62	58	1.03	14.34
5U7Z_C-servers-server63-model-2	60.00	0.22	0.16	0.62	58	1.03	14.34
5U7Z_C-servers-server64-model-1	50.00	0.51	0.00	0.49	37	1.35	33.53
5U7Z_C-servers-server64-model-2	50.00	0.51	0.00	0.49	37	1.35	33.53
5U7Z_C-servers-server65-model-1	43.48	0.02	0.13	0.86	108	0.40	13.18
5U7Z_C-servers-server65-model-2	43.48	0.02	0.13	0.86	108	0.40	13.18
5U7Z_C-servers-server70-model-1	51.56	0.66	0.00	0.34	31	1.66	24.81
5U7Z_D-servers-server11-model-1	28.00	0.17	0.29	0.54	137	0.20	58.14
5U7Z_D-servers-server11-model-2	31.20	0.20	0.27	0.53	134	0.23	62.84
5U7Z_D-servers-server11-model-3	27.20	0.20	0.29	0.50	128	0.21	60.10
5U7Z_D-servers-server11-model-4	28.00	0.20	0.29	0.52	132	0.21	58.82
5U7Z_D-servers-server11-model-5	31.20	0.18	0.27	0.55	141	0.22	59.41
5U7Z_D-servers-server20-model-1	33.87	0.16	0.24	0.60	148	0.23	47.06
5U7Z_D-servers-server20-model-2	36.59	0.16	0.25	0.59	150	0.24	50.49
5U7Z_D-servers-server20-model-3	65.52	0.05	0.27	0.68	30	2.18	5.69
5U7Z_D-servers-server20-model-4	75.00	0.27	0.00	0.73	33	2.27	7.35
5U7Z_D-servers-server22-model-1	29.60	0.15	0.26	0.58	149	0.20	52.16
5U7Z_D-servers-server30-model-1	36.80	0.15	0.24	0.61	155	0.24	54.51
5U7Z_D-servers-server33-model-1	36.80	0.15	0.23	0.63	160	0.23	41.37
5U7Z_D-servers-server33-model-2	36.80	0.15	0.23	0.63	160	0.23	41.37
5U7Z_D-servers-server33-model-3	31.20	0.14	0.27	0.59	150	0.21	40.59
5U7Z_D-servers-server33-model-4	31.20	0.14	0.27	0.59	150	0.21	40.59
5U7Z_D-servers-server33-model-5	33.60	0.14	0.26	0.60	153	0.22	39.61
5U7Z_D-servers-server4-model-1	32.80	0.14	0.22	0.64	164	0.20	63.92
5U7Z_D-servers-server61-model-1	53.60	0.25	0.00	0.75	192	0.28	9.12
5U7Z_D-servers-server61-model-2	53.60	0.25	0.00	0.75	192	0.28	9.12
5U7Z_D-servers-server62-model-1	58.82	0.24	0.00	0.76	179	0.33	7.84
5U7Z_D-servers-server62-model-2	58.82	0.24	0.00	0.76	179	0.33	7.84
5U7Z_D-servers-server63-model-1	37.60	0.13	0.22	0.66	168	0.22	38.53
5U7Z_D-servers-server63-model-2	37.60	0.13	0.22	0.66	168	0.22	38.53
5U7Z_D-servers-server64-model-1	39.20	0.16	0.18	0.66	163	0.24	27.06
5U7Z_D-servers-server64-model-2	39.20	0.16	0.18	0.66	163	0.24	27.06
5U7Z_D-servers-server65-model-1	53.60	0.25	0.00	0.75	192	0.28	9.12
5U7Z_D-servers-server65-model-2	53.60	0.25	0.00	0.75	192	0.28	9.12
5U7Z_D-servers-server70-model-1	32.52	0.14	0.09	0.78	190	0.17	11.37
5U81_A-servers-server11-model-1	19.89	0.32	0.19	0.49	184	0.11	53.06
5U81_A-servers-server11-model-2	20.44	0.33	0.20	0.47	177	0.12	53.84

5U81_A-servers-server11-model-3	20.99	0.29	0.18	0.52	197	0.11	52.15
5U81_A-servers-server11-model-4	19.89	0.32	0.17	0.51	191	0.10	52.41
5U81_A-servers-server11-model-5	21.55	0.31	0.19	0.51	191	0.11	52.34
5U81_A-servers-server20-model-1	27.84	0.31	0.18	0.51	170	0.16	48.18
5U81_A-servers-server20-model-2	25.83	0.17	0.20	0.63	150	0.17	29.88
5U81_A-servers-server22-model-1	19.89	0.35	0.17	0.48	185	0.11	50.13
5U81_A-servers-server30-model-1	25.97	0.30	0.17	0.52	201	0.13	45.57
5U81_A-servers-server33-model-1	25.41	0.28	0.17	0.54	208	0.12	44.73
5U81_A-servers-server33-model-2	25.41	0.28	0.17	0.54	208	0.12	44.73
5U81_A-servers-server33-model-3	26.52	0.28	0.17	0.55	210	0.13	44.47
5U81_A-servers-server33-model-4	26.52	0.28	0.17	0.55	210	0.13	44.47
5U81_A-servers-server33-model-5	26.52	0.28	0.17	0.55	210	0.13	44.47
5U81_A-servers-server36-model-1	82.76	0.61	0.00	0.39	21	3.94	4.62
5U81_A-servers-server4-model-1	26.52	0.25	0.13	0.62	239	0.11	51.50
5U81_A-servers-server61-model-1	52.58	0.51	0.04	0.46	74	0.71	6.32
5U81_A-servers-server61-model-2	52.58	0.51	0.04	0.46	74	0.71	6.32
5U81_A-servers-server62-model-1	46.97	0.19	0.02	0.79	213	0.22	6.12
5U81_A-servers-server62-model-2	46.97	0.19	0.02	0.79	213	0.22	6.12
5U81_A-servers-server63-model-1	57.34	0.23	0.06	0.71	202	0.28	12.04
5U81_A-servers-server63-model-2	57.34	0.23	0.06	0.71	202	0.28	12.04
5U81_A-servers-server64-model-1	29.59	0.30	0.17	0.53	171	0.17	25.13
5U81_A-servers-server64-model-2	29.59	0.30	0.17	0.53	171	0.17	25.13
5U81_A-servers-server65-model-1	52.58	0.51	0.04	0.46	74	0.71	6.32
5U81_A-servers-server65-model-2	52.58	0.51	0.04	0.46	74	0.71	6.32
5U84_B-servers-server11-model-1	20.20	0.31	0.19	0.50	188	0.11	53.06
5U84_B-servers-server11-model-2	19.70	0.30	0.19	0.51	191	0.10	53.19
5U84_B-servers-server11-model-3	22.66	0.29	0.19	0.52	197	0.12	53.52
5U84_B-servers-server11-model-4	19.70	0.32	0.19	0.49	184	0.11	56.06
5U84_B-servers-server11-model-5	24.14	0.27	0.19	0.54	202	0.12	50.78
5U84_B-servers-server20-model-1	26.26	0.30	0.20	0.50	167	0.16	47.92
5U84_B-servers-server20-model-2	27.94	0.15	0.23	0.62	148	0.19	28.26
5U84_B-servers-server22-model-1	21.67	0.34	0.16	0.50	191	0.11	48.18
5U84_B-servers-server30-model-1	23.65	0.29	0.20	0.51	195	0.12	44.60
5U84_B-servers-server33-model-1	25.62	0.28	0.18	0.54	209	0.12	44.01
5U84_B-servers-server33-model-2	24.63	0.27	0.16	0.57	218	0.11	44.79
5U84_B-servers-server33-model-3	25.12	0.28	0.16	0.56	214	0.12	44.40
5U84_B-servers-server33-model-4	25.12	0.28	0.16	0.56	214	0.12	44.40
5U84_B-servers-server33-model-5	25.12	0.28	0.16	0.56	214	0.12	44.40
5U84_B-servers-server36-model-1	79.41	0.39	0.00	0.61	34	2.34	4.95
5U84_B-servers-server4-model-1	25.12	0.28	0.15	0.57	218	0.12	50.98
5U84_B-servers-server61-model-1	30.46	0.35	0.02	0.63	201	0.15	6.58
5U84_B-servers-server61-model-2	30.46	0.35	0.02	0.63	201	0.15	6.58
5U84_B-servers-server62-model-1	38.15	0.36	0.00	0.64	193	0.20	6.12
5U84_B-servers-server62-model-2	38.15	0.36	0.00	0.64	193	0.20	6.12
5U84_B-servers-server63-model-1	29.10	0.26	0.12	0.61	213	0.14	23.63
5U84_B-servers-server63-model-2	29.10	0.26	0.12	0.61	213	0.14	23.63
5U84_B-servers-server64-model-1	34.46	0.33	0.07	0.59	179	0.19	23.96

5U84_B-servers-server64-model-2	34.46	0.33	0.07	0.59	179	0.19	23.96
5U84_B-servers-server65-model-1	30.46	0.35	0.02	0.63	201	0.15	6.58
5U84_B-servers-server65-model-2	30.46	0.35	0.02	0.63	201	0.15	6.58
5UD7_F-servers-server11-model-1	48.28	0.09	0.28	0.64	104	0.46	47.34
5UD7_F-servers-server11-model-2	41.38	0.05	0.30	0.65	106	0.39	47.93
5UD7_F-servers-server11-model-3	48.28	0.12	0.26	0.62	101	0.48	46.89
5UD7_F-servers-server11-model-4	48.28	0.22	0.26	0.52	85	0.57	46.60
5UD7_F-servers-server11-model-5	41.38	0.04	0.17	0.79	129	0.32	26.33
5UD7_F-servers-server17-model-1	55.17	0.04	0.46	0.50	56	0.99	64.50
5UD7_F-servers-server20-model-1	51.72	0.04	0.46	0.50	56	0.92	63.91
5UD7_F-servers-server22-model-1	51.72	0.04	0.30	0.66	111	0.47	63.31
5UD7_F-servers-server36-model-1	48.28	0.04	0.47	0.49	55	0.88	64.50
5UD7_F-servers-server4-model-1	48.28	0.04	0.25	0.72	121	0.40	53.11
5UD7_F-servers-server61-model-1	41.38	0.05	0.16	0.79	133	0.31	41.27
5UD7_F-servers-server61-model-2	41.38	0.05	0.16	0.79	133	0.31	41.27
5UD7_F-servers-server62-model-1	48.28	0.00	0.17	0.83	140	0.34	42.31
5UD7_F-servers-server62-model-2	48.28	0.00	0.17	0.83	140	0.34	42.31
5UD7_F-servers-server63-model-1	59.26	0.03	0.44	0.53	59	1.00	49.70
5UD7_F-servers-server63-model-2	59.26	0.03	0.44	0.53	59	1.00	49.70
5UD7_F-servers-server64-model-1	48.28	0.02	0.39	0.59	69	0.70	48.96
5UD7_F-servers-server64-model-2	48.28	0.02	0.39	0.59	69	0.70	48.96
5UD7_F-servers-server65-model-1	41.38	0.05	0.16	0.79	133	0.31	41.27
5UD7_F-servers-server65-model-2	41.38	0.05	0.16	0.79	133	0.31	41.27
5UD7_F-servers-server70-model-1	48.28	0.01	0.23	0.76	128	0.38	39.65
5V8C_A-servers-server11-model-1	15.12	0.34	0.16	0.49	145	0.10	48.72
5V8C_A-servers-server11-model-2	15.12	0.31	0.13	0.56	163	0.09	48.55
5V8C_A-servers-server11-model-3	12.79	0.32	0.14	0.54	157	0.08	48.55
5V8C_A-servers-server11-model-4	18.60	0.31	0.14	0.55	161	0.12	48.98
5V8C_A-servers-server11-model-5	18.60	0.32	0.13	0.55	161	0.12	47.87
5V8C_A-servers-server17-model-1	20.93	0.39	0.19	0.42	106	0.20	45.22
5V8C_A-servers-server20-model-1	22.09	0.35	0.12	0.53	142	0.16	49.15
5V8C_A-servers-server22-model-1	13.95	0.31	0.17	0.52	153	0.09	50.00
5V8C_A-servers-server30-model-1	18.60	0.32	0.14	0.54	159	0.12	47.95
5V8C_A-servers-server33-model-1	15.12	0.30	0.15	0.55	161	0.09	48.55
5V8C_A-servers-server33-model-2	18.60	0.31	0.14	0.56	163	0.11	47.01
5V8C_A-servers-server33-model-3	18.60	0.31	0.14	0.56	163	0.11	47.01
5V8C_A-servers-server33-model-4	18.60	0.31	0.14	0.56	163	0.11	47.01
5V8C_A-servers-server33-model-5	18.60	0.29	0.17	0.54	159	0.12	47.36
5V8C_A-servers-server36-model-1	51.92	0.29	0.13	0.58	70	0.74	25.34
5V8C_A-servers-server4-model-1	18.60	0.29	0.16	0.55	161	0.12	48.55
5V8C_A-servers-server58-model-1	17.44	0.28	0.15	0.57	166	0.11	47.18
5V8C_A-servers-server58-model-2	17.44	0.27	0.12	0.61	179	0.10	46.67
5V8C_A-servers-server58-model-3	17.44	0.27	0.12	0.61	179	0.10	46.67
5V8C_A-servers-server58-model-4	22.09	0.29	0.14	0.57	167	0.13	47.36
5V8C_A-servers-server58-model-5	22.09	0.29	0.14	0.57	167	0.13	47.36
5V8C_A-servers-server61-model-1	27.91	0.23	0.15	0.63	181	0.15	33.36
5V8C_A-servers-server61-model-2	27.91	0.23	0.15	0.63	181	0.15	33.36

5V8C_A-servers-server62-model-1	22.09	0.21	0.09	0.71	192	0.12	38.40
5V8C_A-servers-server62-model-2	22.09	0.21	0.09	0.71	192	0.12	38.40
5V8C_A-servers-server63-model-1	30.95	0.26	0.11	0.63	165	0.19	37.88
5V8C_A-servers-server63-model-2	30.95	0.26	0.11	0.63	165	0.19	37.88
5V8C_A-servers-server64-model-1	32.05	0.27	0.16	0.57	143	0.22	33.70
5V8C_A-servers-server64-model-2	32.05	0.27	0.16	0.57	143	0.22	33.70
5V8C_A-servers-server65-model-1	27.91	0.23	0.15	0.63	181	0.15	33.36
5V8C_A-servers-server65-model-2	27.91	0.23	0.15	0.63	181	0.15	33.36
5V8C_A-servers-server70-model-1	17.44	0.30	0.16	0.54	159	0.11	49.57
5VFX_H-servers-server11-model-1	38.00	0.49	0.15	0.36	39	0.97	54.91
5VFX_H-servers-server11-model-2	40.00	0.50	0.07	0.43	46	0.87	34.11
5VFX_H-servers-server11-model-3	38.00	0.50	0.09	0.41	44	0.86	40.65
5VFX_H-servers-server11-model-4	48.00	0.48	0.12	0.40	43	1.12	37.15
5VFX_H-servers-server11-model-5	54.00	0.51	0.15	0.34	36	1.50	25.94
5VFX_H-servers-server17-model-1	59.46	0.30	0.22	0.48	33	1.80	37.15
5VFX_H-servers-server20-model-1	54.55	0.34	0.10	0.55	32	1.70	33.65
5VFX_H-servers-server20-model-2	94.44	0.63	0.00	0.37	11	8.59	22.90
5VFX_H-servers-server22-model-1	34.00	0.41	0.13	0.46	49	0.69	47.43
5VFX_H-servers-server30-model-1	50.00	0.22	0.17	0.61	65	0.77	19.63
5VFX_H-servers-server30-model-2	38.00	0.36	0.06	0.59	63	0.60	25.70
5VFX_H-servers-server30-model-3	48.00	0.41	0.06	0.53	57	0.84	22.43
5VFX_H-servers-server30-model-4	60.00	0.44	0.02	0.54	58	1.03	20.79
5VFX_H-servers-server30-model-5	50.00	0.41	0.07	0.51	55	0.91	24.77
5VFX_H-servers-server36-model-1	85.71	0.00	0.30	0.70	45	1.90	10.98
5VFX_H-servers-server4-model-1	62.00	0.39	0.11	0.50	53	1.17	23.83
5VFX_H-servers-server58-model-1	32.00	0.49	0.04	0.48	51	0.63	53.27
5VFX_H-servers-server58-model-2	30.00	0.52	0.04	0.44	47	0.64	56.54
5VFX_H-servers-server58-model-3	34.00	0.50	0.00	0.50	54	0.63	53.74
5VFX_H-servers-server58-model-4	44.00	0.36	0.11	0.53	57	0.77	51.64
5VFX_H-servers-server58-model-5	32.00	0.43	0.05	0.52	56	0.57	44.16
5VFX_H-servers-server61-model-1	56.00	0.05	0.39	0.56	60	0.93	13.08
5VFX_H-servers-server61-model-2	56.00	0.05	0.39	0.56	60	0.93	13.08
5VFX_H-servers-server62-model-1	92.31	0.00	0.00	1.00	49	1.88	12.62
5VFX_H-servers-server62-model-2	92.31	0.00	0.00	1.00	49	1.88	12.62
5VFX_H-servers-server63-model-1	80.77	0.47	0.00	0.53	30	2.69	14.95
5VFX_H-servers-server63-model-2	80.77	0.47	0.00	0.53	30	2.69	14.95
5VFX_H-servers-server64-model-1	64.00	0.49	0.00	0.51	55	1.16	18.46
5VFX_H-servers-server64-model-2	64.00	0.49	0.00	0.51	55	1.16	18.46
5VFX_H-servers-server65-model-1	56.00	0.05	0.39	0.56	60	0.93	13.08
5VFX_H-servers-server65-model-2	56.00	0.05	0.39	0.56	60	0.93	13.08
5VFX_H-servers-server70-model-1	40.00	0.36	0.00	0.64	65	0.62	20.33
5VG2_C-servers-server11-model-1	22.95	0.07	0.24	0.69	153	0.15	62.12
5VG2_C-servers-server11-model-2	25.41	0.05	0.23	0.71	159	0.16	62.23
5VG2_C-servers-server11-model-3	22.13	0.09	0.24	0.66	148	0.15	59.83
5VG2_C-servers-server11-model-4	26.23	0.09	0.22	0.70	155	0.17	62.01
5VG2_C-servers-server11-model-5	27.87	0.11	0.26	0.63	140	0.20	61.46
5VG2_C-servers-server17-model-1	32.04	0.03	0.34	0.63	97	0.33	55.90

5VG2_C-servers-server20-model-1	30.36	0.03	0.26	0.71	140	0.22	57.21
5VG2_C-servers-server20-model-2	36.26	0.06	0.23	0.71	118	0.31	36.35
5VG2_C-servers-server20-model-3	52.17	0.02	0.20	0.77	65	0.80	16.16
5VG2_C-servers-server22-model-1	29.51	0.10	0.22	0.68	155	0.19	59.83
5VG2_C-servers-server30-model-1	27.87	0.02	0.24	0.74	170	0.16	56.99
5VG2_C-servers-server30-model-2	22.95	0.04	0.20	0.76	174	0.13	47.05
5VG2_C-servers-server30-model-3	27.05	0.04	0.22	0.74	169	0.16	42.69
5VG2_C-servers-server30-model-4	39.34	0.07	0.17	0.76	174	0.23	47.82
5VG2_C-servers-server30-model-5	26.23	0.00	0.22	0.78	178	0.15	54.26
5VG2_C-servers-server36-model-1	38.46	0.00	0.15	0.85	110	0.35	40.50
5VG2_C-servers-server4-model-1	33.61	0.04	0.18	0.78	178	0.19	58.41
5VG2_C-servers-server58-model-1	29.51	0.01	0.23	0.76	175	0.17	60.48
5VG2_C-servers-server58-model-2	28.69	0.01	0.21	0.78	179	0.16	56.11
5VG2_C-servers-server58-model-3	29.51	0.02	0.21	0.77	177	0.17	56.77
5VG2_C-servers-server58-model-4	29.51	0.03	0.20	0.77	177	0.17	56.88
5VG2_C-servers-server58-model-5	24.59	0.01	0.20	0.79	180	0.14	54.59
5VG2_C-servers-server61-model-1	31.25	0.02	0.27	0.71	120	0.26	44.43
5VG2_C-servers-server61-model-2	31.25	0.02	0.27	0.71	120	0.26	44.43
5VG2_C-servers-server62-model-1	34.09	0.03	0.21	0.76	121	0.28	43.67
5VG2_C-servers-server62-model-2	34.09	0.03	0.21	0.76	121	0.28	43.67
5VG2_C-servers-server63-model-1	30.33	0.01	0.18	0.80	178	0.17	37.45
5VG2_C-servers-server63-model-2	30.33	0.01	0.18	0.80	178	0.17	37.45
5VG2_C-servers-server64-model-1	39.34	0.05	0.23	0.72	146	0.27	39.74
5VG2_C-servers-server64-model-2	39.34	0.05	0.23	0.72	146	0.27	39.74
5VG2_C-servers-server65-model-1	31.25	0.02	0.27	0.71	120	0.26	44.43
5VG2_C-servers-server65-model-2	31.25	0.02	0.27	0.71	120	0.26	44.43
5VG2_C-servers-server70-model-1	27.87	0.03	0.22	0.75	165	0.17	52.62
5VGU_F-servers-server11-model-1	31.76	0.35	0.22	0.43	54	0.59	80.00
5VGU_F-servers-server11-model-2	30.59	0.32	0.24	0.44	55	0.56	80.00
5VGU_F-servers-server11-model-3	34.12	0.33	0.24	0.43	54	0.63	81.80
5VGU_F-servers-server11-model-4	32.94	0.38	0.22	0.40	50	0.66	81.00
5VGU_F-servers-server11-model-5	35.29	0.38	0.22	0.40	50	0.71	80.40
5VGU_F-servers-server17-model-1	32.50	0.41	0.28	0.31	32	1.02	81.00
5VGU_F-servers-server20-model-1	28.21	0.41	0.25	0.33	34	0.83	80.20
5VGU_F-servers-server22-model-1	31.76	0.42	0.24	0.34	42	0.76	81.80
5VGU_F-servers-server30-model-1	29.41	0.34	0.24	0.42	52	0.57	81.60
5VGU_F-servers-server30-model-2	31.76	0.34	0.24	0.42	53	0.60	73.20
5VGU_F-servers-server30-model-3	36.47	0.29	0.24	0.47	59	0.62	69.20
5VGU_F-servers-server30-model-4	36.47	0.34	0.23	0.42	53	0.69	72.00
5VGU_F-servers-server30-model-5	34.12	0.36	0.22	0.42	52	0.66	69.00
5VGU_F-servers-server33-model-1	29.41	0.31	0.23	0.46	57	0.52	80.40
5VGU_F-servers-server33-model-2	29.41	0.31	0.23	0.46	57	0.52	80.40
5VGU_F-servers-server33-model-3	29.41	0.31	0.23	0.46	57	0.52	80.40
5VGU_F-servers-server33-model-4	29.41	0.31	0.23	0.46	57	0.52	80.40
5VGU_F-servers-server33-model-5	29.41	0.31	0.23	0.46	57	0.52	80.40
5VGU_F-servers-server36-model-1	30.00	0.37	0.31	0.32	30	1.00	73.20
5VGU_F-servers-server4-model-1	34.12	0.33	0.23	0.44	55	0.62	82.20

5VGU_F-servers-server58-model-1	31.76	0.34	0.23	0.43	54	0.59	81.80
5VGU_F-servers-server58-model-2	31.76	0.30	0.23	0.46	58	0.55	80.60
5VGU_F-servers-server58-model-3	31.76	0.30	0.23	0.46	58	0.55	80.60
5VGU_F-servers-server58-model-4	31.76	0.30	0.23	0.46	58	0.55	80.60
5VGU_F-servers-server58-model-5	31.76	0.30	0.23	0.46	58	0.55	80.60
5VGU_F-servers-server61-model-1	29.41	0.38	0.29	0.32	33	0.89	79.20
5VGU_F-servers-server61-model-2	29.41	0.38	0.29	0.32	33	0.89	79.20
5VGU_F-servers-server62-model-1	32.43	0.43	0.18	0.40	36	0.90	71.80
5VGU_F-servers-server62-model-2	32.43	0.43	0.18	0.40	36	0.90	71.80
5VGU_F-servers-server63-model-1	35.71	0.37	0.32	0.31	29	1.23	69.00
5VGU_F-servers-server63-model-2	35.71	0.37	0.32	0.31	29	1.23	69.00
5VGU_F-servers-server64-model-1	34.15	0.35	0.28	0.37	37	0.92	68.20
5VGU_F-servers-server64-model-2	34.15	0.35	0.28	0.37	37	0.92	68.20
5VGU_F-servers-server65-model-1	29.41	0.38	0.29	0.32	33	0.89	79.20
5VGU_F-servers-server65-model-2	29.41	0.38	0.29	0.32	33	0.89	79.20
5VGU_F-servers-server70-model-1	34.12	0.41	0.28	0.31	34	1.00	81.80
5W35_B-servers-server11-model-1	20.74	0.47	0.12	0.41	133	0.16	57.15
5W35_B-servers-server11-model-2	21.48	0.49	0.13	0.39	126	0.17	60.08
5W35_B-servers-server11-model-3	25.93	0.47	0.12	0.41	132	0.20	57.08
5W35_B-servers-server11-model-4	25.19	0.47	0.13	0.40	130	0.19	60.15
5W35_B-servers-server11-model-5	24.44	0.47	0.13	0.40	131	0.19	61.85
5W35_B-servers-server20-model-1	24.63	0.46	0.13	0.41	130	0.19	59.00
5W35_B-servers-server20-model-2	20.43	0.29	0.13	0.58	128	0.16	28.92
5W35_B-servers-server22-model-1	23.70	0.49	0.11	0.40	129	0.18	64.69
5W35_B-servers-server30-model-1	22.22	0.45	0.11	0.43	141	0.16	57.23
5W35_B-servers-server30-model-2	19.26	0.47	0.09	0.44	142	0.14	65.31
5W35_B-servers-server30-model-3	23.70	0.46	0.12	0.42	138	0.17	63.23
5W35_B-servers-server30-model-4	41.48	0.32	0.04	0.65	210	0.20	9.54
5W35_B-servers-server30-model-5	30.37	0.34	0.04	0.62	201	0.15	8.62
5W35_B-servers-server33-model-1	22.96	0.46	0.11	0.42	137	0.17	60.46
5W35_B-servers-server33-model-2	24.44	0.47	0.11	0.42	136	0.18	58.62
5W35_B-servers-server33-model-3	22.22	0.45	0.11	0.44	144	0.15	59.46
5W35_B-servers-server33-model-4	22.96	0.45	0.11	0.44	143	0.16	58.69
5W35_B-servers-server33-model-5	23.70	0.46	0.11	0.43	139	0.17	59.46
5W35_B-servers-server36-model-1	23.31	0.46	0.11	0.43	135	0.17	55.77
5W35_B-servers-server4-model-1	25.19	0.46	0.09	0.46	148	0.17	65.77
5W35_B-servers-server61-model-1	22.22	0.45	0.11	0.44	139	0.16	56.46
5W35_B-servers-server61-model-2	22.22	0.45	0.11	0.44	139	0.16	56.46
5W35_B-servers-server62-model-1	22.39	0.45	0.11	0.44	139	0.16	56.77
5W35_B-servers-server62-model-2	22.39	0.45	0.11	0.44	139	0.16	56.77
5W35_B-servers-server63-model-1	22.58	0.43	0.12	0.45	134	0.17	53.15
5W35_B-servers-server63-model-2	22.58	0.43	0.12	0.45	134	0.17	53.15
5W35_B-servers-server64-model-1	22.22	0.45	0.11	0.44	139	0.16	56.46
5W35_B-servers-server64-model-2	22.22	0.45	0.11	0.44	139	0.16	56.46
5W35_B-servers-server65-model-1	22.22	0.45	0.11	0.44	139	0.16	56.46
5W35_B-servers-server65-model-2	22.22	0.45	0.11	0.44	139	0.16	56.46
5W35_B-servers-server70-model-1	22.96	0.48	0.12	0.41	131	0.18	63.92

5WEE_D-servers-server11-model-1	34.74	0.28	0.14	0.57	112	0.31	78.72
5WEE_D-servers-server11-model-2	30.53	0.30	0.14	0.56	109	0.28	79.36
5WEE_D-servers-server11-model-3	34.74	0.31	0.11	0.57	112	0.31	74.62
5WEE_D-servers-server11-model-4	32.63	0.33	0.10	0.57	111	0.29	77.69
5WEE_D-servers-server11-model-5	33.68	0.28	0.11	0.61	118	0.29	79.49
5WEE_D-servers-server20-model-1	37.23	0.29	0.14	0.57	106	0.35	69.49
5WEE_D-servers-server22-model-1	34.74	0.32	0.15	0.52	102	0.34	70.77
5WEE_D-servers-server30-model-1	31.58	0.28	0.14	0.57	112	0.28	69.10
5WEE_D-servers-server30-model-2	34.74	0.27	0.12	0.61	119	0.29	60.90
5WEE_D-servers-server30-model-3	29.47	0.25	0.12	0.64	124	0.24	60.13
5WEE_D-servers-server30-model-4	27.37	0.25	0.11	0.64	125	0.22	54.10
5WEE_D-servers-server30-model-5	35.79	0.27	0.12	0.61	119	0.30	68.33
5WEE_D-servers-server33-model-1	31.58	0.28	0.14	0.58	113	0.28	77.82
5WEE_D-servers-server33-model-2	28.42	0.28	0.13	0.59	115	0.25	77.95
5WEE_D-servers-server33-model-3	31.58	0.28	0.13	0.58	114	0.28	75.51
5WEE_D-servers-server33-model-4	36.84	0.28	0.14	0.58	113	0.33	75.64
5WEE_D-servers-server33-model-5	35.79	0.28	0.13	0.59	115	0.31	76.03
5WEE_D-servers-server36-model-1	35.11	0.29	0.15	0.56	105	0.33	68.85
5WEE_D-servers-server4-model-1	35.79	0.31	0.17	0.52	102	0.35	78.85
5WEE_D-servers-server61-model-1	38.95	0.31	0.17	0.52	101	0.39	70.13
5WEE_D-servers-server61-model-2	38.95	0.31	0.17	0.52	101	0.39	70.13
5WEE_D-servers-server62-model-1	35.79	0.30	0.16	0.54	101	0.35	70.00
5WEE_D-servers-server62-model-2	35.79	0.30	0.16	0.54	101	0.35	70.00
5WEE_D-servers-server63-model-1	39.36	0.31	0.12	0.57	106	0.37	69.62
5WEE_D-servers-server63-model-2	39.36	0.31	0.12	0.57	106	0.37	69.62
5WEE_D-servers-server64-model-1	34.04	0.32	0.13	0.54	102	0.33	69.49
5WEE_D-servers-server64-model-2	34.04	0.32	0.13	0.54	102	0.33	69.49
5WEE_D-servers-server65-model-1	38.95	0.31	0.17	0.52	101	0.39	70.13
5WEE_D-servers-server65-model-2	38.95	0.31	0.17	0.52	101	0.39	70.13
5WEE_D-servers-server70-model-1	37.89	0.31	0.14	0.55	106	0.36	70.90
5WJD_A-servers-server11-model-1	37.04	0.31	0.26	0.43	69	0.54	67.77
5WJD_A-servers-server11-model-2	40.74	0.30	0.26	0.43	69	0.59	70.60
5WJD_A-servers-server11-model-3	40.74	0.32	0.23	0.45	71	0.57	66.20
5WJD_A-servers-server11-model-4	37.04	0.33	0.28	0.39	62	0.60	66.35
5WJD_A-servers-server11-model-5	41.98	0.34	0.28	0.38	60	0.70	66.98
5WJD_A-servers-server20-model-1	37.33	0.34	0.19	0.47	63	0.59	56.29
5WJD_A-servers-server20-model-2	50.00	0.23	0.21	0.56	87	0.57	50.63
5WJD_A-servers-server22-model-1	32.10	0.31	0.31	0.38	60	0.53	64.62
5WJD_A-servers-server30-model-1	38.27	0.35	0.21	0.43	69	0.55	57.70
5WJD_A-servers-server30-model-2	38.27	0.30	0.16	0.53	85	0.45	61.79
5WJD_A-servers-server30-model-3	29.63	0.26	0.21	0.53	84	0.35	51.73
5WJD_A-servers-server30-model-4	34.57	0.31	0.20	0.48	77	0.45	55.50
5WJD_A-servers-server30-model-5	34.57	0.29	0.21	0.50	79	0.44	58.65
5WJD_A-servers-server33-model-1	34.57	0.28	0.16	0.57	90	0.38	64.94
5WJD_A-servers-server33-model-2	39.51	0.30	0.24	0.46	73	0.54	65.88
5WJD_A-servers-server33-model-3	34.57	0.30	0.19	0.51	81	0.43	63.84
5WJD_A-servers-server33-model-4	34.57	0.30	0.19	0.51	81	0.43	63.84

5WJD_A-servers-server33-model-5	34.57	0.26	0.21	0.53	84	0.41	63.68
5WJD_A-servers-server36-model-1	62.50	0.45	0.00	0.55	30	2.08	28.46
5WJD_A-servers-server4-model-1	39.51	0.35	0.21	0.44	70	0.56	60.69
5WJD_A-servers-server61-model-1	54.79	0.31	0.22	0.47	63	0.87	38.52
5WJD_A-servers-server61-model-2	54.79	0.31	0.22	0.47	63	0.87	38.52
5WJD_A-servers-server62-model-1	39.66	0.21	0.25	0.54	57	0.70	43.40
5WJD_A-servers-server62-model-2	39.66	0.21	0.25	0.54	57	0.70	43.40
5WJD_A-servers-server63-model-1	41.10	0.23	0.24	0.53	75	0.55	48.90
5WJD_A-servers-server63-model-2	41.10	0.23	0.24	0.53	75	0.55	48.90
5WJD_A-servers-server64-model-1	40.26	0.28	0.25	0.48	74	0.54	52.20
5WJD_A-servers-server64-model-2	40.26	0.28	0.25	0.48	74	0.54	52.20
5WJD_A-servers-server65-model-1	54.79	0.31	0.22	0.47	63	0.87	38.52
5WJD_A-servers-server65-model-2	54.79	0.31	0.22	0.47	63	0.87	38.52
5WJD_A-servers-server70-model-1	40.79	0.25	0.18	0.57	81	0.50	48.90
5WLY_A-servers-server11-model-1	21.77	0.36	0.19	0.44	107	0.20	74.29
5WLY_A-servers-server11-model-2	23.13	0.36	0.21	0.43	103	0.22	74.09
5WLY_A-servers-server11-model-3	23.13	0.36	0.20	0.43	105	0.22	74.09
5WLY_A-servers-server11-model-4	24.49	0.36	0.21	0.43	104	0.24	73.89
5WLY_A-servers-server11-model-5	22.45	0.36	0.20	0.44	106	0.21	73.99
5WLY_A-servers-server17-model-1	22.92	0.36	0.19	0.45	108	0.21	72.28
5WLY_A-servers-server20-model-1	28.08	0.37	0.21	0.43	102	0.28	72.98
5WLY_A-servers-server22-model-1	26.53	0.36	0.18	0.46	114	0.23	73.29
5WLY_A-servers-server30-model-1	25.17	0.15	0.16	0.70	173	0.15	41.13
5WLY_A-servers-server30-model-2	25.17	0.21	0.15	0.63	157	0.16	40.22
5WLY_A-servers-server30-model-3	24.49	0.13	0.15	0.73	181	0.14	40.42
5WLY_A-servers-server30-model-4	25.85	0.21	0.17	0.61	152	0.17	34.38
5WLY_A-servers-server30-model-5	25.85	0.10	0.17	0.74	183	0.14	40.73
5WLY_A-servers-server33-model-1	24.49	0.36	0.19	0.44	110	0.22	73.29
5WLY_A-servers-server33-model-2	24.49	0.36	0.19	0.44	110	0.22	73.29
5WLY_A-servers-server33-model-3	24.49	0.36	0.19	0.44	110	0.22	73.29
5WLY_A-servers-server33-model-4	24.49	0.36	0.19	0.44	110	0.22	73.29
5WLY_A-servers-server33-model-5	24.49	0.36	0.19	0.44	110	0.22	73.29
5WLY_A-servers-server36-model-1	26.12	0.41	0.12	0.47	102	0.26	62.60
5WLY_A-servers-server4-model-1	21.09	0.21	0.12	0.67	167	0.13	49.70
5WLY_A-servers-server58-model-1	23.81	0.34	0.19	0.46	115	0.21	73.59
5WLY_A-servers-server58-model-2	25.85	0.36	0.20	0.44	108	0.24	73.29
5WLY_A-servers-server58-model-3	25.85	0.36	0.20	0.44	108	0.24	73.29
5WLY_A-servers-server58-model-4	23.81	0.35	0.21	0.44	109	0.22	73.59
5WLY_A-servers-server58-model-5	23.13	0.36	0.18	0.46	113	0.20	73.29
5WLY_A-servers-server61-model-1	28.08	0.38	0.20	0.43	102	0.28	72.58
5WLY_A-servers-server61-model-2	28.08	0.38	0.20	0.43	102	0.28	72.58
5WLY_A-servers-server62-model-1	28.08	0.38	0.20	0.43	102	0.28	72.58
5WLY_A-servers-server62-model-2	28.08	0.38	0.20	0.43	102	0.28	72.58
5WLY_A-servers-server63-model-1	24.82	0.38	0.14	0.48	109	0.23	65.63
5WLY_A-servers-server63-model-2	24.82	0.38	0.14	0.48	109	0.23	65.63
5WLY_A-servers-server64-model-1	25.85	0.37	0.15	0.48	116	0.22	65.93
5WLY_A-servers-server64-model-2	25.85	0.37	0.15	0.48	116	0.22	65.93

5WLY_A-servers-server65-model-1	28.08	0.38	0.20	0.43	102	0.28	72.58
5WLY_A-servers-server65-model-2	28.08	0.38	0.20	0.43	102	0.28	72.58
5WLY_A-servers-server70-model-1	22.45	0.36	0.20	0.44	110	0.20	73.19
5X2B_L-servers-server11-model-1	28.15	0.43	0.07	0.49	140	0.20	74.82
5X2B_L-servers-server11-model-2	26.67	0.43	0.07	0.50	142	0.19	74.56
5X2B_L-servers-server11-model-3	28.89	0.42	0.08	0.51	143	0.20	73.76
5X2B_L-servers-server11-model-4	24.44	0.43	0.07	0.49	140	0.17	74.21
5X2B_L-servers-server11-model-5	28.89	0.43	0.07	0.50	142	0.20	74.03
5X2B_L-servers-server20-model-1	25.93	0.42	0.07	0.51	141	0.18	71.73
5X2B_L-servers-server22-model-1	27.41	0.45	0.07	0.48	137	0.20	76.41
5X2B_L-servers-server30-model-1	26.67	0.40	0.07	0.53	149	0.18	72.70
5X2B_L-servers-server30-model-2	26.67	0.43	0.08	0.49	139	0.19	71.47
5X2B_L-servers-server30-model-3	28.15	0.43	0.07	0.50	142	0.20	71.38
5X2B_L-servers-server30-model-4	29.63	0.40	0.07	0.53	150	0.20	74.03
5X2B_L-servers-server30-model-5	25.93	0.41	0.07	0.52	146	0.18	71.73
5X2B_L-servers-server33-model-1	30.37	0.41	0.07	0.52	148	0.21	73.15
5X2B_L-servers-server33-model-2	30.37	0.41	0.07	0.52	148	0.21	73.15
5X2B_L-servers-server33-model-3	30.37	0.41	0.07	0.52	148	0.21	73.15
5X2B_L-servers-server33-model-4	28.89	0.42	0.08	0.50	141	0.20	74.21
5X2B_L-servers-server33-model-5	27.41	0.41	0.07	0.52	147	0.19	74.21
5X2B_L-servers-server36-model-1	37.19	0.39	0.07	0.54	134	0.28	69.44
5X2B_L-servers-server4-model-1	29.63	0.40	0.08	0.53	149	0.20	77.74
5X2B_L-servers-server61-model-1	31.85	0.44	0.07	0.49	134	0.24	75.27
5X2B_L-servers-server61-model-2	31.85	0.44	0.07	0.49	134	0.24	75.27
5X2B_L-servers-server62-model-1	34.33	0.44	0.08	0.48	129	0.27	76.68
5X2B_L-servers-server62-model-2	34.33	0.44	0.08	0.48	129	0.27	76.68
5X2B_L-servers-server63-model-1	26.12	0.44	0.08	0.48	131	0.20	69.17
5X2B_L-servers-server63-model-2	26.12	0.44	0.08	0.48	131	0.20	69.17
5X2B_L-servers-server64-model-1	28.15	0.43	0.07	0.51	140	0.20	68.91
5X2B_L-servers-server64-model-2	28.15	0.43	0.07	0.51	140	0.20	68.91
5X2B_L-servers-server65-model-1	31.85	0.44	0.07	0.49	134	0.24	75.27
5X2B_L-servers-server65-model-2	31.85	0.44	0.07	0.49	134	0.24	75.27
5X2B_L-servers-server70-model-1	29.63	0.42	0.07	0.51	141	0.21	74.29
5X7Y_D-servers-server11-model-1	22.81	0.14	0.37	0.50	88	0.26	83.48
5X7Y_D-servers-server11-model-2	21.05	0.14	0.39	0.47	84	0.25	83.33
5X7Y_D-servers-server11-model-3	17.54	0.15	0.37	0.49	86	0.20	83.62
5X7Y_D-servers-server11-model-4	17.54	0.14	0.37	0.49	87	0.20	83.33
5X7Y_D-servers-server11-model-5	19.30	0.14	0.37	0.49	87	0.22	83.62
5X7Y_D-servers-server17-model-1	17.54	0.12	0.40	0.49	79	0.22	84.46
5X7Y_D-servers-server20-model-1	21.05	0.12	0.40	0.48	79	0.27	83.62
5X7Y_D-servers-server22-model-1	24.56	0.14	0.36	0.50	88	0.28	85.73
5X7Y_D-servers-server36-model-1	19.30	0.13	0.37	0.49	80	0.24	83.76
5X7Y_D-servers-server4-model-1	21.05	0.12	0.32	0.55	98	0.21	83.76
5X7Y_D-servers-server63-model-1	22.81	0.15	0.40	0.45	72	0.32	83.90
5X7Y_D-servers-server63-model-2	22.81	0.15	0.40	0.45	72	0.32	83.90
5X7Y_D-servers-server64-model-1	22.81	0.15	0.40	0.45	72	0.32	83.90
5X7Y_D-servers-server64-model-2	22.81	0.15	0.40	0.45	72	0.32	83.90

5X7Y_D-servers-server70-model-1	21.05	0.16	0.40	0.44	71	0.30	86.02
5XB6_L-servers-server11-model-1	23.36	0.37	0.17	0.46	141	0.17	82.68
5XB6_L-servers-server11-model-2	26.17	0.38	0.17	0.45	138	0.19	80.39
5XB6_L-servers-server11-model-3	23.36	0.39	0.17	0.45	137	0.17	82.11
5XB6_L-servers-server11-model-4	27.10	0.39	0.17	0.45	137	0.20	82.60
5XB6_L-servers-server11-model-5	23.36	0.39	0.17	0.44	135	0.17	83.50
5XB6_L-servers-server17-model-1	26.17	0.34	0.16	0.50	149	0.18	76.72
5XB6_L-servers-server20-model-1	22.43	0.38	0.15	0.47	144	0.16	76.39
5XB6_L-servers-server20-model-2	24.30	0.30	0.14	0.56	169	0.14	52.53
5XB6_L-servers-server20-model-3	30.84	0.29	0.13	0.58	170	0.18	50.00
5XB6_L-servers-server22-model-1	26.17	0.36	0.16	0.48	147	0.18	81.37
5XB6_L-servers-server30-model-1	28.04	0.37	0.18	0.44	136	0.21	81.62
5XB6_L-servers-server30-model-2	26.17	0.27	0.14	0.59	181	0.14	54.49
5XB6_L-servers-server30-model-3	26.17	0.25	0.14	0.62	189	0.14	55.31
5XB6_L-servers-server30-model-4	24.30	0.34	0.09	0.56	172	0.14	49.51
5XB6_L-servers-server30-model-5	28.97	0.36	0.10	0.54	166	0.17	48.45
5XB6_L-servers-server33-model-1	27.10	0.37	0.14	0.49	151	0.18	80.96
5XB6_L-servers-server33-model-2	27.10	0.37	0.14	0.49	151	0.18	80.96
5XB6_L-servers-server33-model-3	27.10	0.37	0.14	0.49	151	0.18	80.96
5XB6_L-servers-server33-model-4	27.10	0.37	0.14	0.49	151	0.18	80.96
5XB6_L-servers-server33-model-5	27.10	0.37	0.14	0.49	151	0.18	80.96
5XB6_L-servers-server36-model-1	21.50	0.33	0.14	0.53	161	0.13	76.80
5XB6_L-servers-server4-model-1	27.10	0.33	0.14	0.52	160	0.17	81.78
5XB6_L-servers-server58-model-1	27.10	0.37	0.15	0.48	147	0.18	81.62
5XB6_L-servers-server58-model-2	27.10	0.37	0.15	0.48	147	0.18	81.62
5XB6_L-servers-server58-model-3	27.10	0.37	0.15	0.48	147	0.18	81.62
5XB6_L-servers-server58-model-4	27.10	0.37	0.15	0.48	147	0.18	81.62
5XB6_L-servers-server58-model-5	27.10	0.37	0.15	0.48	147	0.18	81.62
5XB6_L-servers-server61-model-1	24.30	0.34	0.17	0.48	148	0.16	80.72
5XB6_L-servers-server61-model-2	24.30	0.34	0.17	0.48	148	0.16	80.72
5XB6_L-servers-server62-model-1	26.92	0.35	0.13	0.52	149	0.18	75.82
5XB6_L-servers-server62-model-2	26.92	0.35	0.13	0.52	149	0.18	75.82
5XB6_L-servers-server63-model-1	37.36	0.42	0.08	0.51	133	0.28	69.94
5XB6_L-servers-server63-model-2	37.36	0.42	0.08	0.51	133	0.28	69.94
5XB6_L-servers-server64-model-1	24.30	0.34	0.17	0.48	148	0.16	80.72
5XB6_L-servers-server64-model-2	24.30	0.34	0.17	0.48	148	0.16	80.72
5XB6_L-servers-server65-model-1	24.30	0.34	0.17	0.48	148	0.16	80.72
5XB6_L-servers-server65-model-2	24.30	0.34	0.17	0.48	148	0.16	80.72
5XB6_L-servers-server70-model-1	27.10	0.37	0.18	0.45	139	0.19	80.39
5XBV_A-servers-server11-model-1	31.58	0.38	0.13	0.48	72	0.44	63.09
5XBV_A-servers-server11-model-2	38.60	0.41	0.11	0.48	71	0.54	64.77
5XBV_A-servers-server11-model-3	35.09	0.41	0.10	0.49	73	0.48	63.09
5XBV_A-servers-server11-model-4	36.84	0.37	0.10	0.53	79	0.47	56.88
5XBV_A-servers-server11-model-5	36.84	0.38	0.09	0.53	79	0.47	60.91
5XBV_A-servers-server20-model-1	36.36	0.40	0.11	0.49	57	0.64	71.81
5XBV_A-servers-server22-model-1	38.60	0.42	0.10	0.48	71	0.54	72.32
5XBV_A-servers-server30-model-1	35.09	0.33	0.02	0.65	97	0.36	36.75

5XBV_A-servers-server30-model-2	43.86	0.32	0.06	0.62	92	0.48	36.75
5XBV_A-servers-server30-model-3	47.37	0.30	0.05	0.64	96	0.49	35.91
5XBV_A-servers-server30-model-4	33.33	0.32	0.18	0.50	75	0.44	24.66
5XBV_A-servers-server30-model-5	36.84	0.38	0.01	0.60	90	0.41	21.14
5XBV_A-servers-server33-model-1	38.60	0.32	0.10	0.58	86	0.45	65.27
5XBV_A-servers-server33-model-2	38.60	0.32	0.10	0.58	86	0.45	65.27
5XBV_A-servers-server33-model-3	38.60	0.32	0.10	0.58	86	0.45	65.27
5XBV_A-servers-server33-model-4	38.60	0.32	0.10	0.58	86	0.45	65.27
5XBV_A-servers-server33-model-5	38.60	0.32	0.10	0.58	86	0.45	65.27
5XBV_A-servers-server36-model-1	45.83	0.39	0.09	0.52	50	0.92	57.38
5XBV_A-servers-server4-model-1	45.61	0.36	0.08	0.56	83	0.55	35.40
5XBV_A-servers-server61-model-1	41.51	0.38	0.15	0.47	54	0.77	66.44
5XBV_A-servers-server61-model-2	41.51	0.38	0.15	0.47	54	0.77	66.44
5XBV_A-servers-server62-model-1	39.62	0.43	0.10	0.47	54	0.73	67.95
5XBV_A-servers-server62-model-2	39.62	0.43	0.10	0.47	54	0.73	67.95
5XBV_A-servers-server63-model-1	49.02	0.44	0.04	0.53	57	0.86	32.89
5XBV_A-servers-server63-model-2	49.02	0.44	0.04	0.53	57	0.86	32.89
5XBV_A-servers-server64-model-1	50.00	0.41	0.14	0.46	34	1.47	21.98
5XBV_A-servers-server64-model-2	50.00	0.41	0.14	0.46	34	1.47	21.98
5XBV_A-servers-server65-model-1	41.51	0.38	0.15	0.47	54	0.77	66.44
5XBV_A-servers-server65-model-2	41.51	0.38	0.15	0.47	54	0.77	66.44
5XBV_A-servers-server70-model-1	33.93	0.40	0.12	0.48	58	0.58	72.48
5XCA_A-servers-server11-model-1	21.21	0.08	0.18	0.74	136	0.16	72.90
5XCA_A-servers-server11-model-2	21.21	0.11	0.18	0.71	130	0.16	70.00
5XCA_A-servers-server11-model-3	27.27	0.09	0.14	0.78	143	0.19	62.90
5XCA_A-servers-server11-model-4	27.27	0.11	0.18	0.71	131	0.21	65.66
5XCA_A-servers-server11-model-5	27.27	0.10	0.13	0.77	142	0.19	62.90
5XCA_A-servers-server20-model-1	21.21	0.09	0.23	0.68	118	0.18	83.68
5XCA_A-servers-server20-model-2	43.33	0.19	0.15	0.66	67	0.65	26.71
5XCA_A-servers-server22-model-1	24.24	0.12	0.20	0.68	130	0.19	79.47
5XCA_A-servers-server30-model-1	27.27	0.09	0.18	0.73	138	0.20	79.74
5XCA_A-servers-server33-model-1	21.21	0.07	0.20	0.73	138	0.15	82.63
5XCA_A-servers-server33-model-2	21.21	0.07	0.20	0.73	138	0.15	82.63
5XCA_A-servers-server33-model-3	21.21	0.07	0.20	0.73	138	0.15	80.66
5XCA_A-servers-server33-model-4	21.21	0.07	0.20	0.73	138	0.15	80.66
5XCA_A-servers-server33-model-5	21.21	0.07	0.20	0.73	138	0.15	80.66
5XCA_A-servers-server36-model-1	24.24	0.07	0.23	0.70	115	0.21	77.63
5XCA_A-servers-server4-model-1	21.21	0.06	0.18	0.75	143	0.15	84.74
5XCA_A-servers-server61-model-1	21.21	0.09	0.22	0.69	120	0.18	81.18
5XCA_A-servers-server61-model-2	21.21	0.09	0.22	0.69	120	0.18	81.18
5XCA_A-servers-server62-model-1	27.27	0.08	0.23	0.69	116	0.24	80.79
5XCA_A-servers-server62-model-2	27.27	0.08	0.23	0.69	116	0.24	80.79
5XCA_A-servers-server63-model-1	24.24	0.07	0.23	0.70	118	0.21	80.40
5XCA_A-servers-server63-model-2	24.24	0.07	0.23	0.70	118	0.21	80.40
5XCA_A-servers-server64-model-1	21.21	0.09	0.22	0.69	120	0.18	81.18
5XCA_A-servers-server64-model-2	21.21	0.09	0.22	0.69	120	0.18	81.18
5XCA_A-servers-server65-model-1	21.21	0.09	0.22	0.69	120	0.18	81.18

5XCA_A-servers-server65-model-2	21.21	0.09	0.22	0.69	120	0.18	81.18
5XCA_A-servers-server70-model-1	21.21	0.09	0.22	0.69	120	0.18	79.47
5XD6_B-servers-server11-model-1	29.27	0.37	0.15	0.49	146	0.20	73.20
5XD6_B-servers-server11-model-2	23.58	0.37	0.15	0.49	147	0.16	74.18
5XD6_B-servers-server11-model-3	26.02	0.36	0.14	0.50	150	0.17	73.77
5XD6_B-servers-server11-model-4	23.58	0.38	0.15	0.47	142	0.17	73.36
5XD6_B-servers-server11-model-5	28.46	0.37	0.15	0.49	146	0.19	74.67
5XD6_B-servers-server20-model-1	29.51	0.35	0.13	0.53	156	0.19	71.48
5XD6_B-servers-server22-model-1	29.27	0.36	0.15	0.50	151	0.19	71.23
5XD6_B-servers-server36-model-1	29.91	0.36	0.13	0.51	146	0.20	67.62
5XD6_B-servers-server4-model-1	26.83	0.37	0.12	0.51	157	0.17	73.77
5XD6_B-servers-server61-model-1	26.23	0.36	0.13	0.51	153	0.17	70.98
5XD6_B-servers-server61-model-2	26.23	0.36	0.13	0.51	153	0.17	70.98
5XD6_B-servers-server62-model-1	29.27	0.36	0.12	0.52	157	0.19	69.26
5XD6_B-servers-server62-model-2	29.27	0.36	0.12	0.52	157	0.19	69.26
5XD6_B-servers-server63-model-1	29.91	0.31	0.13	0.56	163	0.18	56.97
5XD6_B-servers-server63-model-2	29.91	0.31	0.13	0.56	163	0.18	56.97
5XD6_B-servers-server64-model-1	32.52	0.30	0.13	0.57	173	0.19	54.34
5XD6_B-servers-server64-model-2	32.52	0.30	0.13	0.57	173	0.19	54.34
5XD6_B-servers-server65-model-1	26.23	0.36	0.13	0.51	153	0.17	70.98
5XD6_B-servers-server65-model-2	26.23	0.36	0.13	0.51	153	0.17	70.98
5XD6_B-servers-server70-model-1	29.51	0.37	0.13	0.50	149	0.20	73.53
5XDY_A-servers-server11-model-1	32.11	0.17	0.33	0.50	164	0.20	62.54
5XDY_A-servers-server11-model-2	30.28	0.18	0.33	0.50	165	0.18	63.43
5XDY_A-servers-server11-model-3	30.28	0.16	0.34	0.50	166	0.18	65.43
5XDY_A-servers-server11-model-4	29.36	0.18	0.31	0.51	169	0.17	63.06
5XDY_A-servers-server11-model-5	31.19	0.18	0.30	0.53	174	0.18	61.05
5XDY_A-servers-server17-model-1	30.61	0.14	0.36	0.50	144	0.21	57.86
5XDY_A-servers-server20-model-1	24.49	0.13	0.34	0.53	159	0.15	62.98
5XDY_A-servers-server20-model-2	32.65	0.13	0.31	0.56	167	0.20	50.37
5XDY_A-servers-server20-model-3	34.69	0.15	0.32	0.53	146	0.24	60.16
5XDY_A-servers-server20-model-4	28.57	0.13	0.32	0.55	167	0.17	49.48
5XDY_A-servers-server20-model-5	31.63	0.09	0.36	0.55	168	0.19	47.26
5XDY_A-servers-server22-model-1	31.19	0.18	0.29	0.53	179	0.17	63.35
5XDY_A-servers-server36-model-1	33.67	0.15	0.32	0.53	146	0.23	60.68
5XDY_A-servers-server4-model-1	36.70	0.13	0.33	0.54	181	0.20	68.69
5XDY_A-servers-server61-model-1	30.61	0.13	0.35	0.52	155	0.20	61.72
5XDY_A-servers-server61-model-2	30.61	0.13	0.35	0.52	155	0.20	61.72
5XDY_A-servers-server62-model-1	29.59	0.14	0.26	0.60	180	0.16	57.42
5XDY_A-servers-server62-model-2	29.59	0.14	0.26	0.60	180	0.16	57.42
5XDY_A-servers-server63-model-1	27.55	0.13	0.29	0.58	174	0.16	59.72
5XDY_A-servers-server63-model-2	27.55	0.13	0.29	0.58	174	0.16	59.72
5XDY_A-servers-server64-model-1	30.61	0.13	0.35	0.52	155	0.20	61.72
5XDY_A-servers-server64-model-2	30.61	0.13	0.35	0.52	155	0.20	61.72
5XDY_A-servers-server65-model-1	30.61	0.13	0.35	0.52	155	0.20	61.72
5XDY_A-servers-server65-model-2	30.61	0.13	0.35	0.52	155	0.20	61.72
5XDY_A-servers-server70-model-1	25.00	0.15	0.32	0.53	172	0.15	63.95

5XEO_B-servers-server11-model-1	22.43	0.39	0.13	0.48	150	0.15	90.89
5XEO_B-servers-server11-model-2	20.56	0.40	0.13	0.47	147	0.14	91.45
5XEO_B-servers-server11-model-3	22.43	0.41	0.13	0.47	145	0.15	91.53
5XEO_B-servers-server11-model-4	20.56	0.38	0.13	0.49	152	0.14	91.94
5XEO_B-servers-server11-model-5	23.36	0.41	0.13	0.46	143	0.16	91.29
5XEO_B-servers-server17-model-1	23.36	0.37	0.13	0.50	152	0.15	83.63
5XEO_B-servers-server20-model-1	25.23	0.38	0.13	0.49	149	0.17	87.66
5XEO_B-servers-server22-model-1	24.30	0.39	0.13	0.49	151	0.16	88.31
5XEO_B-servers-server30-model-1	29.91	0.40	0.10	0.50	154	0.19	84.44
5XEO_B-servers-server30-model-2	27.10	0.38	0.12	0.50	154	0.18	82.34
5XEO_B-servers-server30-model-3	28.97	0.38	0.13	0.49	151	0.19	78.71
5XEO_B-servers-server30-model-4	27.10	0.42	0.13	0.46	142	0.19	84.60
5XEO_B-servers-server30-model-5	25.23	0.41	0.13	0.46	144	0.18	82.50
5XEO_B-servers-server33-model-1	26.17	0.39	0.13	0.48	150	0.17	86.69
5XEO_B-servers-server33-model-2	25.23	0.40	0.13	0.48	148	0.17	85.89
5XEO_B-servers-server33-model-3	25.23	0.39	0.13	0.48	148	0.17	85.89
5XEO_B-servers-server33-model-4	27.10	0.38	0.13	0.49	152	0.18	85.57
5XEO_B-servers-server33-model-5	27.10	0.38	0.13	0.49	152	0.18	85.57
5XEO_B-servers-server36-model-1	31.78	0.36	0.12	0.52	155	0.21	61.29
5XEO_B-servers-server4-model-1	25.23	0.40	0.13	0.48	148	0.17	85.89
5XEO_B-servers-server58-model-1	25.23	0.39	0.13	0.49	151	0.17	85.48
5XEO_B-servers-server58-model-2	23.36	0.40	0.13	0.47	146	0.16	86.37
5XEO_B-servers-server58-model-3	24.30	0.39	0.13	0.48	150	0.16	86.13
5XEO_B-servers-server58-model-4	24.30	0.38	0.13	0.49	152	0.16	86.37
5XEO_B-servers-server58-model-5	22.43	0.39	0.13	0.48	150	0.15	86.21
5XEO_B-servers-server61-model-1	35.51	0.36	0.13	0.51	151	0.24	64.76
5XEO_B-servers-server61-model-2	35.51	0.36	0.13	0.51	151	0.24	64.76
5XEO_B-servers-server62-model-1	34.58	0.37	0.13	0.51	149	0.23	64.36
5XEO_B-servers-server62-model-2	34.58	0.37	0.13	0.51	149	0.23	64.36
5XEO_B-servers-server63-model-1	26.17	0.41	0.13	0.46	138	0.19	83.07
5XEO_B-servers-server63-model-2	26.17	0.41	0.13	0.46	138	0.19	83.07
5XEO_B-servers-server64-model-1	27.10	0.41	0.13	0.47	140	0.19	83.95
5XEO_B-servers-server64-model-2	27.10	0.41	0.13	0.47	140	0.19	83.95
5XEO_B-servers-server65-model-1	35.51	0.36	0.13	0.51	151	0.24	64.76
5XEO_B-servers-server65-model-2	35.51	0.36	0.13	0.51	151	0.24	64.76
5XEO_B-servers-server70-model-1	23.36	0.39	0.13	0.48	149	0.16	89.11
5XEP_F-servers-server11-model-1	19.08	0.36	0.19	0.45	170	0.11	90.68
5XEP_F-servers-server11-model-2	19.69	0.35	0.18	0.47	178	0.11	90.42
5XEP_F-servers-server11-model-3	17.54	0.36	0.19	0.45	172	0.10	90.42
5XEP_F-servers-server11-model-4	21.23	0.36	0.19	0.46	174	0.12	91.08
5XEP_F-servers-server11-model-5	19.08	0.36	0.19	0.46	174	0.11	90.49
5XEP_F-servers-server17-model-1	16.67	0.32	0.20	0.48	172	0.10	86.55
5XEP_F-servers-server20-model-1	17.76	0.32	0.20	0.48	174	0.10	91.54
5XEP_F-servers-server22-model-1	20.62	0.35	0.19	0.45	173	0.12	90.35
5XEP_F-servers-server30-model-1	18.46	0.34	0.18	0.48	183	0.10	90.75
5XEP_F-servers-server30-model-2	15.08	0.34	0.18	0.48	184	0.08	86.55
5XEP_F-servers-server30-model-3	17.85	0.36	0.19	0.45	171	0.10	87.73

5XEP_F-servers-server30-model-4	20.31	0.32	0.19	0.49	187	0.11	85.11
5XEP_F-servers-server30-model-5	19.38	0.36	0.17	0.48	181	0.11	79.33
5XEP_F-servers-server36-model-1	16.45	0.34	0.20	0.46	164	0.10	91.34
5XEP_F-servers-server4-model-1	24.62	0.33	0.18	0.49	188	0.13	90.68
5XEP_F-servers-server58-model-1	20.00	0.33	0.18	0.49	187	0.11	90.95
5XEP_F-servers-server58-model-2	20.62	0.33	0.16	0.51	194	0.11	90.95
5XEP_F-servers-server58-model-3	20.62	0.33	0.16	0.51	194	0.11	90.95
5XEP_F-servers-server58-model-4	19.08	0.33	0.17	0.50	191	0.10	91.14
5XEP_F-servers-server58-model-5	19.69	0.33	0.18	0.49	186	0.11	88.91
5XEP_F-servers-server61-model-1	16.45	0.33	0.19	0.48	172	0.10	91.47
5XEP_F-servers-server61-model-2	16.45	0.33	0.19	0.48	172	0.10	91.47
5XEP_F-servers-server62-model-1	17.11	0.33	0.20	0.48	171	0.10	91.86
5XEP_F-servers-server62-model-2	17.11	0.33	0.20	0.48	171	0.10	91.86
5XEP_F-servers-server63-model-1	15.41	0.32	0.20	0.48	175	0.09	84.97
5XEP_F-servers-server63-model-2	15.41	0.32	0.20	0.48	175	0.09	84.97
5XEP_F-servers-server64-model-1	17.38	0.32	0.19	0.49	178	0.10	83.53
5XEP_F-servers-server64-model-2	17.38	0.32	0.19	0.49	178	0.10	83.53
5XEP_F-servers-server65-model-1	16.45	0.33	0.19	0.48	172	0.10	91.47
5XEP_F-servers-server65-model-2	16.45	0.33	0.19	0.48	172	0.10	91.47
5XEP_F-servers-server70-model-1	17.76	0.34	0.19	0.47	169	0.11	90.22
5XFL_D-servers-server11-model-1	40.56	0.79	0.00	0.21	80	0.51	77.53
5XFL_D-servers-server11-model-2	39.86	0.80	0.00	0.20	74	0.54	76.93
5XFL_D-servers-server11-model-3	41.26	0.80	0.00	0.20	74	0.56	76.67
5XFL_D-servers-server11-model-4	39.86	0.80	0.00	0.20	74	0.54	76.87
5XFL_D-servers-server11-model-5	39.86	0.80	0.00	0.20	76	0.52	76.67
5XFL_D-servers-server20-model-1	38.46	0.77	0.00	0.23	84	0.46	77.07
5XFL_D-servers-server20-model-2	44.90	0.74	0.00	0.26	56	0.80	20.20
5XFL_D-servers-server22-model-1	41.26	0.78	0.00	0.22	82	0.50	75.53
5XFL_D-servers-server30-model-1	40.56	0.78	0.00	0.22	81	0.50	56.80
5XFL_D-servers-server30-model-2	43.36	0.70	0.00	0.30	113	0.38	22.20
5XFL_D-servers-server30-model-3	44.06	0.70	0.00	0.30	114	0.39	20.93
5XFL_D-servers-server30-model-4	41.26	0.68	0.00	0.32	119	0.35	13.73
5XFL_D-servers-server30-model-5	30.07	0.63	0.00	0.37	138	0.22	10.80
5XFL_D-servers-server33-model-1	37.76	0.78	0.00	0.22	82	0.46	76.00
5XFL_D-servers-server33-model-2	37.06	0.78	0.00	0.22	82	0.45	74.67
5XFL_D-servers-server33-model-3	37.06	0.78	0.00	0.22	82	0.45	74.67
5XFL_D-servers-server33-model-4	37.06	0.78	0.00	0.22	82	0.45	74.67
5XFL_D-servers-server33-model-5	38.46	0.78	0.00	0.22	83	0.46	75.40
5XFL_D-servers-server36-model-1	39.16	0.79	0.00	0.21	79	0.50	69.67
5XFL_D-servers-server4-model-1	41.26	0.74	0.00	0.26	96	0.43	63.80
5XFL_D-servers-server61-model-1	37.06	0.78	0.00	0.22	84	0.44	78.73
5XFL_D-servers-server61-model-2	37.06	0.78	0.00	0.22	84	0.44	78.73
5XFL_D-servers-server62-model-1	45.74	0.78	0.00	0.22	74	0.62	71.87
5XFL_D-servers-server62-model-2	45.74	0.78	0.00	0.22	74	0.62	71.87
5XFL_D-servers-server63-model-1	46.02	0.78	0.00	0.22	71	0.65	67.07
5XFL_D-servers-server63-model-2	46.02	0.78	0.00	0.22	71	0.65	67.07
5XFL_D-servers-server64-model-1	37.06	0.78	0.00	0.22	84	0.44	78.73

5XFL_D-servers-server64-model-2	37.06	0.78	0.00	0.22	84	0.44	78.73
5XFL_D-servers-server65-model-1	37.06	0.78	0.00	0.22	84	0.44	78.73
5XFL_D-servers-server65-model-2	37.06	0.78	0.00	0.22	84	0.44	78.73
5XFL_D-servers-server70-model-1	41.26	0.77	0.00	0.23	86	0.48	77.33
5XJV_B-servers-server11-model-1	25.33	0.41	0.09	0.50	93	0.27	83.92
5XJV_B-servers-server11-model-2	25.33	0.40	0.09	0.51	95	0.27	84.05
5XJV_B-servers-server11-model-3	24.00	0.41	0.09	0.51	94	0.26	84.32
5XJV_B-servers-server11-model-4	25.33	0.44	0.09	0.48	88	0.29	84.60
5XJV_B-servers-server11-model-5	26.67	0.41	0.09	0.51	94	0.28	83.92
5XJV_B-servers-server17-model-1	33.78	0.45	0.14	0.41	58	0.58	66.62
5XJV_B-servers-server20-model-1	28.00	0.43	0.10	0.48	79	0.35	77.03
5XJV_B-servers-server20-model-2	30.67	0.43	0.10	0.47	78	0.39	76.35
5XJV_B-servers-server20-model-3	36.00	0.46	0.11	0.43	64	0.56	63.24
5XJV_B-servers-server22-model-1	30.67	0.45	0.09	0.46	86	0.36	76.62
5XJV_B-servers-server30-model-1	25.33	0.41	0.09	0.51	94	0.27	83.65
5XJV_B-servers-server30-model-2	25.33	0.40	0.09	0.51	95	0.27	77.43
5XJV_B-servers-server30-model-3	24.00	0.41	0.09	0.51	94	0.26	80.27
5XJV_B-servers-server30-model-4	24.00	0.38	0.12	0.50	92	0.26	66.49
5XJV_B-servers-server30-model-5	26.67	0.36	0.08	0.56	103	0.26	67.97
5XJV_B-servers-server33-model-1	21.33	0.40	0.09	0.51	95	0.22	83.78
5XJV_B-servers-server33-model-2	21.33	0.40	0.09	0.51	95	0.22	83.78
5XJV_B-servers-server33-model-3	26.67	0.39	0.09	0.52	96	0.28	82.84
5XJV_B-servers-server33-model-4	21.33	0.40	0.09	0.51	95	0.22	82.97
5XJV_B-servers-server33-model-5	25.33	0.40	0.09	0.51	95	0.27	81.76
5XJV_B-servers-server36-model-1	26.67	0.44	0.13	0.44	73	0.37	77.43
5XJV_B-servers-server4-model-1	28.00	0.41	0.08	0.52	96	0.29	84.73
5XJV_B-servers-server58-model-1	24.00	0.38	0.09	0.53	98	0.24	83.92
5XJV_B-servers-server58-model-2	24.00	0.38	0.09	0.53	98	0.24	83.92
5XJV_B-servers-server58-model-3	24.00	0.38	0.09	0.53	98	0.24	83.92
5XJV_B-servers-server58-model-4	25.33	0.39	0.09	0.52	96	0.26	84.19
5XJV_B-servers-server58-model-5	25.33	0.39	0.09	0.52	96	0.26	85.27
5XJV_B-servers-server61-model-1	28.00	0.46	0.10	0.45	75	0.37	77.03
5XJV_B-servers-server61-model-2	28.00	0.46	0.10	0.45	75	0.37	77.03
5XJV_B-servers-server62-model-1	28.00	0.42	0.10	0.48	80	0.35	76.22
5XJV_B-servers-server62-model-2	28.00	0.42	0.10	0.48	80	0.35	76.22
5XJV_B-servers-server63-model-1	29.33	0.35	0.11	0.54	77	0.38	71.08
5XJV_B-servers-server63-model-2	29.33	0.35	0.11	0.54	77	0.38	71.08
5XJV_B-servers-server64-model-1	26.67	0.44	0.09	0.46	79	0.34	77.84
5XJV_B-servers-server64-model-2	26.67	0.44	0.09	0.46	79	0.34	77.84
5XJV_B-servers-server65-model-1	28.00	0.46	0.10	0.45	75	0.37	77.03
5XJV_B-servers-server65-model-2	28.00	0.46	0.10	0.45	75	0.37	77.03
5XJV_B-servers-server70-model-1	26.67	0.42	0.09	0.49	83	0.32	77.16
5XOM_B-servers-server11-model-1	24.48	0.37	0.10	0.53	208	0.12	75.70
5XOM_B-servers-server11-model-2	27.27	0.35	0.12	0.54	211	0.13	74.87
5XOM_B-servers-server11-model-3	25.17	0.35	0.11	0.54	213	0.12	73.98
5XOM_B-servers-server11-model-4	27.27	0.36	0.11	0.53	207	0.13	69.21
5XOM_B-servers-server11-model-5	23.78	0.33	0.10	0.57	225	0.11	72.65

5XOM_B-servers-server20-model-1	30.07	0.36	0.13	0.51	190	0.16	67.88
5XOM_B-servers-server20-model-2	82.93	0.26	0.05	0.69	60	1.38	8.84
5XOM_B-servers-server22-model-1	27.97	0.38	0.11	0.51	200	0.14	74.05
5XOM_B-servers-server30-model-1	26.57	0.34	0.10	0.55	218	0.12	75.38
5XOM_B-servers-server30-model-2	42.66	0.28	0.06	0.66	261	0.16	18.58
5XOM_B-servers-server30-model-3	43.36	0.31	0.06	0.63	247	0.18	20.99
5XOM_B-servers-server30-model-4	31.47	0.24	0.07	0.68	269	0.12	7.63
5XOM_B-servers-server30-model-5	35.66	0.39	0.04	0.57	225	0.16	15.90
5XOM_B-servers-server36-model-1	32.17	0.35	0.10	0.54	187	0.17	70.80
5XOM_B-servers-server4-model-1	28.67	0.32	0.10	0.58	227	0.13	75.13
5XOM_B-servers-server58-model-1	30.07	0.37	0.12	0.51	202	0.15	66.92
5XOM_B-servers-server58-model-2	30.07	0.37	0.12	0.51	202	0.15	66.92
5XOM_B-servers-server58-model-3	30.07	0.37	0.12	0.51	202	0.15	66.92
5XOM_B-servers-server58-model-4	30.07	0.37	0.12	0.51	202	0.15	66.92
5XOM_B-servers-server58-model-5	30.07	0.39	0.12	0.49	194	0.15	65.84
5XOM_B-servers-server61-model-1	28.67	0.33	0.11	0.57	223	0.13	75.64
5XOM_B-servers-server61-model-2	28.67	0.33	0.11	0.57	223	0.13	75.64
5XOM_B-servers-server62-model-1	34.31	0.31	0.10	0.59	216	0.16	63.74
5XOM_B-servers-server62-model-2	34.31	0.31	0.10	0.59	216	0.16	63.74
5XOM_B-servers-server63-model-1	33.58	0.31	0.11	0.58	210	0.16	68.89
5XOM_B-servers-server63-model-2	33.58	0.31	0.11	0.58	210	0.16	68.89
5XOM_B-servers-server64-model-1	25.87	0.31	0.11	0.59	230	0.11	70.29
5XOM_B-servers-server64-model-2	25.87	0.31	0.11	0.59	230	0.11	70.29
5XOM_B-servers-server65-model-1	28.67	0.33	0.11	0.57	223	0.13	75.64
5XOM_B-servers-server65-model-2	28.67	0.33	0.11	0.57	223	0.13	75.64
5XOM_B-servers-server70-model-1	32.17	0.31	0.10	0.60	234	0.14	70.61
5XPW_A-servers-server11-model-1	40.91	0.04	0.44	0.52	94	0.44	60.14
5XPW_A-servers-server11-model-2	37.88	0.04	0.48	0.48	86	0.44	65.00
5XPW_A-servers-server11-model-3	40.91	0.03	0.46	0.51	92	0.44	61.11
5XPW_A-servers-server11-model-4	42.42	0.03	0.46	0.51	92	0.46	64.31
5XPW_A-servers-server11-model-5	37.88	0.03	0.48	0.49	88	0.43	60.97
5XPW_A-servers-server17-model-1	48.48	0.05	0.54	0.41	70	0.69	30.14
5XPW_A-servers-server20-model-1	36.92	0.08	0.40	0.52	93	0.40	36.81
5XPW_A-servers-server20-model-2	40.91	0.01	0.34	0.65	116	0.35	50.97
5XPW_A-servers-server20-model-3	37.88	0.03	0.30	0.66	119	0.32	37.50
5XPW_A-servers-server22-model-1	36.36	0.02	0.46	0.52	94	0.39	59.03
5XPW_A-servers-server30-model-1	48.48	0.04	0.34	0.61	110	0.44	51.94
5XPW_A-servers-server30-model-2	45.45	0.00	0.35	0.65	117	0.39	53.06
5XPW_A-servers-server30-model-3	40.91	0.02	0.36	0.62	112	0.37	46.39
5XPW_A-servers-server30-model-4	39.39	0.02	0.28	0.69	125	0.32	37.50
5XPW_A-servers-server30-model-5	42.42	0.01	0.36	0.63	114	0.37	44.31
5XPW_A-servers-server36-model-1	53.85	0.03	0.37	0.60	71	0.76	43.75
5XPW_A-servers-server4-model-1	36.36	0.02	0.19	0.78	141	0.26	40.83
5XPW_A-servers-server58-model-1	50.00	0.02	0.37	0.61	110	0.45	59.44
5XPW_A-servers-server58-model-2	31.82	0.02	0.42	0.56	100	0.32	59.58
5XPW_A-servers-server58-model-3	31.82	0.02	0.42	0.56	100	0.32	59.58
5XPW_A-servers-server58-model-4	36.36	0.01	0.41	0.58	104	0.35	63.61

5XPW_A-servers-server58-model-5	45.45	0.02	0.37	0.61	109	0.42	56.94
5XPW_A-servers-server61-model-1	50.00	0.03	0.31	0.66	118	0.42	44.17
5XPW_A-servers-server61-model-2	50.00	0.03	0.31	0.66	118	0.42	44.17
5XPW_A-servers-server62-model-1	45.45	0.02	0.39	0.58	105	0.43	57.78
5XPW_A-servers-server62-model-2	45.45	0.02	0.39	0.58	105	0.43	57.78
5XPW_A-servers-server63-model-1	57.81	0.02	0.23	0.75	124	0.47	26.94
5XPW_A-servers-server63-model-2	57.81	0.02	0.23	0.75	124	0.47	26.94
5XPW_A-servers-server64-model-1	51.52	0.01	0.31	0.68	122	0.42	35.00
5XPW_A-servers-server64-model-2	51.52	0.01	0.31	0.68	122	0.42	35.00
5XPW_A-servers-server65-model-1	50.00	0.03	0.31	0.66	118	0.42	44.17
5XPW_A-servers-server65-model-2	50.00	0.03	0.31	0.66	118	0.42	44.17
5XPW_A-servers-server70-model-1	48.78	0.02	0.37	0.61	51	0.96	31.53
5XVS_B-servers-server11-model-1	25.93	0.44	0.13	0.43	164	0.16	80.01
5XVS_B-servers-server11-model-2	25.19	0.43	0.13	0.44	167	0.15	76.45
5XVS_B-servers-server11-model-3	27.41	0.41	0.12	0.46	176	0.16	75.46
5XVS_B-servers-server11-model-4	27.41	0.41	0.12	0.47	178	0.15	77.18
5XVS_B-servers-server11-model-5	25.19	0.44	0.13	0.42	161	0.16	77.18
5XVS_B-servers-server20-model-1	27.41	0.40	0.13	0.48	179	0.15	78.83
5XVS_B-servers-server20-model-2	27.48	0.41	0.10	0.49	176	0.16	52.57
5XVS_B-servers-server20-model-3	34.33	0.34	0.12	0.54	197	0.17	27.24
5XVS_B-servers-server22-model-1	31.11	0.44	0.11	0.45	170	0.18	77.70
5XVS_B-servers-server30-model-1	28.15	0.39	0.10	0.50	191	0.15	63.65
5XVS_B-servers-server30-model-2	38.52	0.41	0.11	0.47	180	0.21	49.14
5XVS_B-servers-server30-model-3	28.89	0.40	0.11	0.49	186	0.16	57.98
5XVS_B-servers-server30-model-4	32.59	0.40	0.11	0.49	185	0.18	54.16
5XVS_B-servers-server30-model-5	27.41	0.41	0.12	0.47	178	0.15	43.21
5XVS_B-servers-server33-model-1	27.41	0.40	0.12	0.48	183	0.15	75.92
5XVS_B-servers-server33-model-2	27.41	0.39	0.13	0.48	183	0.15	75.59
5XVS_B-servers-server33-model-3	28.15	0.42	0.12	0.46	173	0.16	80.87
5XVS_B-servers-server33-model-4	25.93	0.40	0.12	0.47	179	0.14	78.50
5XVS_B-servers-server33-model-5	24.44	0.40	0.12	0.48	181	0.14	76.85
5XVS_B-servers-server36-model-1	25.56	0.40	0.11	0.50	184	0.14	77.18
5XVS_B-servers-server4-model-1	30.37	0.39	0.11	0.50	190	0.16	54.42
5XVS_B-servers-server58-model-1	25.19	0.41	0.12	0.47	177	0.14	80.67
5XVS_B-servers-server58-model-2	25.19	0.42	0.13	0.45	169	0.15	81.14
5XVS_B-servers-server58-model-3	27.41	0.42	0.12	0.46	175	0.16	81.27
5XVS_B-servers-server58-model-4	24.44	0.40	0.12	0.47	180	0.14	78.56
5XVS_B-servers-server58-model-5	28.89	0.42	0.13	0.45	169	0.17	81.73
5XVS_B-servers-server61-model-1	27.41	0.39	0.12	0.49	183	0.15	77.77
5XVS_B-servers-server61-model-2	27.41	0.39	0.12	0.49	183	0.15	77.77
5XVS_B-servers-server62-model-1	23.48	0.40	0.09	0.50	187	0.13	70.19
5XVS_B-servers-server62-model-2	23.48	0.40	0.09	0.50	187	0.13	70.19
5XVS_B-servers-server63-model-1	34.59	0.45	0.10	0.45	169	0.20	56.00
5XVS_B-servers-server63-model-2	34.59	0.45	0.10	0.45	169	0.20	56.00
5XVS_B-servers-server64-model-1	31.85	0.42	0.10	0.47	178	0.18	54.22
5XVS_B-servers-server64-model-2	31.85	0.42	0.10	0.47	178	0.18	54.22
5XVS_B-servers-server65-model-1	27.41	0.39	0.12	0.49	183	0.15	77.77

5XVS_B-servers-server65-model-2	27.41	0.39	0.12	0.49	183	0.15	77.77
5XVS_B-servers-server70-model-1	25.93	0.39	0.12	0.49	184	0.14	77.77
5Y4B_A-servers-server11-model-1	43.90	0.43	0.23	0.34	30	1.46	62.10
5Y4B_A-servers-server11-model-2	48.78	0.43	0.20	0.38	33	1.48	63.71
5Y4B_A-servers-server11-model-3	41.46	0.41	0.23	0.36	31	1.34	64.79
5Y4B_A-servers-server11-model-4	41.46	0.45	0.23	0.32	28	1.48	62.63
5Y4B_A-servers-server11-model-5	41.46	0.39	0.23	0.38	33	1.26	63.17
5Y4B_A-servers-server20-model-1	35.14	0.43	0.19	0.38	31	1.13	54.57
5Y4B_A-servers-server22-model-1	31.71	0.35	0.22	0.43	40	0.79	63.44
5Y4B_A-servers-server30-model-1	36.59	0.40	0.25	0.35	33	1.11	63.71
5Y4B_A-servers-server30-model-2	51.22	0.37	0.15	0.48	45	1.14	64.79
5Y4B_A-servers-server30-model-3	43.90	0.38	0.23	0.40	37	1.19	69.09
5Y4B_A-servers-server30-model-4	39.02	0.43	0.19	0.38	35	1.11	56.45
5Y4B_A-servers-server30-model-5	31.71	0.37	0.18	0.45	42	0.75	61.56
5Y4B_A-servers-server33-model-1	36.59	0.37	0.22	0.42	39	0.94	62.37
5Y4B_A-servers-server33-model-2	36.59	0.37	0.22	0.42	39	0.94	62.37
5Y4B_A-servers-server33-model-3	36.59	0.37	0.22	0.42	39	0.94	62.37
5Y4B_A-servers-server33-model-4	36.59	0.37	0.22	0.42	39	0.94	62.37
5Y4B_A-servers-server33-model-5	36.59	0.37	0.22	0.42	39	0.94	62.37
5Y4B_A-servers-server36-model-1	48.65	0.42	0.21	0.37	28	1.74	48.93
5Y4B_A-servers-server4-model-1	46.34	0.38	0.17	0.45	42	1.10	65.86
5Y4B_A-servers-server61-model-1	41.46	0.44	0.19	0.36	32	1.30	55.65
5Y4B_A-servers-server61-model-2	41.46	0.44	0.19	0.36	32	1.30	55.65
5Y4B_A-servers-server62-model-1	41.46	0.45	0.23	0.32	28	1.48	56.18
5Y4B_A-servers-server62-model-2	41.46	0.45	0.23	0.32	28	1.48	56.18
5Y4B_A-servers-server63-model-1	44.12	0.32	0.21	0.47	35	1.26	55.65
5Y4B_A-servers-server63-model-2	44.12	0.32	0.21	0.47	35	1.26	55.65
5Y4B_A-servers-server64-model-1	41.18	0.31	0.19	0.50	39	1.06	55.38
5Y4B_A-servers-server64-model-2	41.18	0.31	0.19	0.50	39	1.06	55.38
5Y4B_A-servers-server65-model-1	41.46	0.44	0.19	0.36	32	1.30	55.65
5Y4B_A-servers-server65-model-2	41.46	0.44	0.19	0.36	32	1.30	55.65
5Y4B_A-servers-server70-model-1	43.59	0.40	0.24	0.36	31	1.41	61.56
5Y8E_A-servers-server11-model-1	17.07	0.43	0.09	0.48	74	0.23	63.96
5Y8E_A-servers-server11-model-2	12.20	0.43	0.10	0.47	73	0.17	61.85
5Y8E_A-servers-server11-model-3	19.51	0.45	0.10	0.45	69	0.28	65.75
5Y8E_A-servers-server11-model-4	19.51	0.41	0.08	0.51	78	0.25	64.45
5Y8E_A-servers-server11-model-5	14.63	0.42	0.09	0.49	76	0.19	62.50
5Y8E_A-servers-server20-model-1	19.51	0.45	0.11	0.44	64	0.30	65.42
5Y8E_A-servers-server20-model-2	47.06	0.37	0.03	0.60	81	0.58	23.54
5Y8E_A-servers-server22-model-1	17.07	0.38	0.09	0.53	82	0.21	61.04
5Y8E_A-servers-server36-model-1	64.71	0.34	0.00	0.66	85	0.76	19.48
5Y8E_A-servers-server4-model-1	24.39	0.36	0.08	0.55	85	0.29	64.94
5Y8E_A-servers-server61-model-1	56.41	0.31	0.07	0.61	92	0.61	19.48
5Y8E_A-servers-server61-model-2	56.41	0.31	0.07	0.61	92	0.61	19.48
5Y8E_A-servers-server62-model-1	65.22	0.18	0.00	0.83	66	0.99	10.71
5Y8E_A-servers-server62-model-2	65.22	0.18	0.00	0.83	66	0.99	10.71
5Y8E_A-servers-server63-model-1	24.39	0.35	0.12	0.53	73	0.33	48.38

5Y8E_A-servers-server63-model-2	24.39	0.35	0.12	0.53	73	0.33	48.38
5Y8E_A-servers-server64-model-1	24.39	0.22	0.09	0.69	105	0.23	39.94
5Y8E_A-servers-server64-model-2	24.39	0.22	0.09	0.69	105	0.23	39.94
5Y8E_A-servers-server65-model-1	56.41	0.31	0.07	0.61	92	0.61	19.48
5Y8E_A-servers-server65-model-2	56.41	0.31	0.07	0.61	92	0.61	19.48
5YH0_L-servers-server11-model-1	30.22	0.41	0.08	0.51	285	0.11	61.25
5YH0_L-servers-server11-model-2	30.94	0.37	0.08	0.55	305	0.10	60.18
5YH0_L-servers-server11-model-3	30.58	0.37	0.08	0.55	303	0.10	61.16
5YH0_L-servers-server11-model-4	33.09	0.40	0.08	0.53	292	0.11	60.18
5YH0_L-servers-server11-model-5	30.58	0.39	0.08	0.54	299	0.10	60.40
5YH0_L-servers-server20-model-1	28.85	0.37	0.09	0.54	223	0.13	51.34
5YH0_L-servers-server20-model-2	28.21	0.33	0.11	0.57	225	0.13	56.83
5YH0_L-servers-server20-model-3	78.05	0.25	0.10	0.65	55	1.42	6.30
5YH0_L-servers-server22-model-1	32.94	0.37	0.11	0.52	231	0.14	53.80
5YH0_L-servers-server30-model-1	34.98	0.37	0.08	0.56	311	0.11	55.85
5YH0_L-servers-server30-model-2	54.77	0.35	0.05	0.60	336	0.16	18.04
5YH0_L-servers-server30-model-3	44.52	0.28	0.06	0.66	369	0.12	5.85
5YH0_L-servers-server30-model-4	38.52	0.31	0.04	0.65	363	0.11	5.22
5YH0_L-servers-server30-model-5	45.58	0.44	0.00	0.56	313	0.15	6.07
5YH0_L-servers-server36-model-1	28.85	0.39	0.09	0.51	213	0.14	50.09
5YH0_L-servers-server4-model-1	36.51	0.24	0.08	0.67	376	0.10	57.01
5YH0_L-servers-server61-model-1	33.07	0.32	0.11	0.57	242	0.14	55.22
5YH0_L-servers-server61-model-2	33.07	0.32	0.11	0.57	242	0.14	55.22
5YH0_L-servers-server62-model-1	31.37	0.28	0.08	0.64	318	0.10	55.98
5YH0_L-servers-server62-model-2	31.37	0.28	0.08	0.64	318	0.10	55.98
5YH0_L-servers-server63-model-1	34.51	0.27	0.08	0.64	312	0.11	53.75
5YH0_L-servers-server63-model-2	34.51	0.27	0.08	0.64	312	0.11	53.75
5YH0_L-servers-server64-model-1	30.71	0.34	0.10	0.56	237	0.13	57.05
5YH0_L-servers-server64-model-2	30.71	0.34	0.10	0.56	237	0.13	57.05
5YH0_L-servers-server65-model-1	33.07	0.32	0.11	0.57	242	0.14	55.22
5YH0_L-servers-server65-model-2	33.07	0.32	0.11	0.57	242	0.14	55.22
5YH0_L-servers-server70-model-1	29.64	0.40	0.11	0.49	203	0.15	53.84
5Z11_B-servers-server11-model-1	36.90	0.00	0.36	0.64	82	0.45	54.88
5Z11_B-servers-server11-model-2	30.95	0.04	0.41	0.55	70	0.44	55.27
5Z11_B-servers-server11-model-3	35.71	0.04	0.38	0.59	75	0.48	58.01
5Z11_B-servers-server11-model-4	29.76	0.02	0.40	0.58	74	0.40	58.20
5Z11_B-servers-server11-model-5	36.90	0.00	0.37	0.63	81	0.46	58.01
5Z11_B-servers-server17-model-1	32.05	0.05	0.28	0.67	67	0.48	52.54
5Z11_B-servers-server20-model-1	33.33	0.03	0.41	0.57	64	0.52	55.47
5Z11_B-servers-server22-model-1	39.29	0.02	0.33	0.66	84	0.47	52.15
5Z11_B-servers-server30-model-1	38.10	0.02	0.41	0.57	73	0.52	50.78
5Z11_B-servers-server30-model-2	38.10	0.03	0.38	0.59	75	0.51	59.96
5Z11_B-servers-server30-model-3	36.90	0.00	0.38	0.63	80	0.46	54.10
5Z11_B-servers-server30-model-4	29.76	0.02	0.33	0.66	84	0.35	54.49
5Z11_B-servers-server30-model-5	38.10	0.02	0.33	0.66	84	0.45	54.69
5Z11_B-servers-server33-model-1	32.14	0.02	0.38	0.60	77	0.42	55.27
5Z11_B-servers-server33-model-2	32.14	0.02	0.38	0.60	77	0.42	55.27

5Z11_B-servers-server33-model-3	33.33	0.03	0.37	0.60	77	0.43	56.06
5Z11_B-servers-server33-model-4	33.33	0.03	0.37	0.60	77	0.43	56.06
5Z11_B-servers-server33-model-5	30.95	0.02	0.41	0.57	73	0.42	56.25
5Z11_B-servers-server36-model-1	35.06	0.02	0.37	0.61	71	0.49	54.10
5Z11_B-servers-server4-model-1	42.86	0.02	0.31	0.66	85	0.50	52.93
5Z11_B-servers-server58-model-1	34.52	0.04	0.39	0.57	73	0.47	55.86
5Z11_B-servers-server58-model-2	33.33	0.02	0.41	0.58	74	0.45	55.27
5Z11_B-servers-server58-model-3	33.33	0.02	0.41	0.58	74	0.45	55.27
5Z11_B-servers-server58-model-4	36.90	0.02	0.41	0.57	73	0.51	56.25
5Z11_B-servers-server58-model-5	36.90	0.02	0.41	0.57	73	0.51	56.25
5Z11_B-servers-server61-model-1	38.55	0.02	0.35	0.63	80	0.48	56.06
5Z11_B-servers-server61-model-2	38.55	0.02	0.35	0.63	80	0.48	56.06
5Z11_B-servers-server62-model-1	31.33	0.02	0.40	0.58	73	0.43	55.86
5Z11_B-servers-server62-model-2	31.33	0.02	0.40	0.58	73	0.43	55.86
5Z11_B-servers-server63-model-1	35.71	0.02	0.39	0.60	65	0.55	56.45
5Z11_B-servers-server63-model-2	35.71	0.02	0.39	0.60	65	0.55	56.45
5Z11_B-servers-server64-model-1	33.33	0.02	0.36	0.62	68	0.49	54.49
5Z11_B-servers-server64-model-2	33.33	0.02	0.36	0.62	68	0.49	54.49
5Z11_B-servers-server65-model-1	38.55	0.02	0.35	0.63	80	0.48	56.06
5Z11_B-servers-server65-model-2	38.55	0.02	0.35	0.63	80	0.48	56.06
5Z11_B-servers-server70-model-1	34.52	0.00	0.41	0.59	75	0.46	56.25
5Z4G_B-servers-server11-model-1	31.82	0.10	0.46	0.44	67	0.47	71.43
5Z4G_B-servers-server11-model-2	36.36	0.11	0.44	0.45	68	0.53	70.65
5Z4G_B-servers-server11-model-3	36.36	0.15	0.45	0.40	61	0.60	69.10
5Z4G_B-servers-server11-model-4	40.91	0.15	0.46	0.39	59	0.69	65.68
5Z4G_B-servers-server11-model-5	31.82	0.12	0.44	0.44	66	0.48	70.50
5Z4G_B-servers-server17-model-1	20.00	0.10	0.56	0.34	46	0.43	65.84
5Z4G_B-servers-server20-model-1	22.73	0.13	0.48	0.40	56	0.41	71.27
5Z4G_B-servers-server22-model-1	22.73	0.12	0.41	0.47	75	0.30	69.26
5Z4G_B-servers-server30-model-1	40.91	0.09	0.39	0.52	84	0.49	51.40
5Z4G_B-servers-server30-model-2	45.45	0.11	0.39	0.50	80	0.57	56.99
5Z4G_B-servers-server30-model-3	27.27	0.06	0.35	0.58	94	0.29	49.22
5Z4G_B-servers-server30-model-4	36.36	0.10	0.38	0.52	84	0.43	57.14
5Z4G_B-servers-server30-model-5	18.18	0.12	0.39	0.50	80	0.23	57.92
5Z4G_B-servers-server33-model-1	27.27	0.08	0.45	0.47	76	0.36	66.15
5Z4G_B-servers-server33-model-2	31.82	0.11	0.43	0.46	74	0.43	64.44
5Z4G_B-servers-server33-model-3	31.82	0.11	0.47	0.43	69	0.46	66.15
5Z4G_B-servers-server33-model-4	31.82	0.09	0.47	0.44	71	0.45	65.37
5Z4G_B-servers-server33-model-5	18.18	0.08	0.47	0.45	72	0.25	65.68
5Z4G_B-servers-server36-model-1	22.73	0.08	0.48	0.44	63	0.36	70.03
5Z4G_B-servers-server4-model-1	22.73	0.15	0.42	0.43	69	0.33	62.11
5Z4G_B-servers-server58-model-1	31.82	0.09	0.48	0.43	69	0.46	66.15
5Z4G_B-servers-server58-model-2	22.73	0.11	0.46	0.43	70	0.32	65.68
5Z4G_B-servers-server58-model-3	36.36	0.09	0.40	0.50	81	0.45	59.78
5Z4G_B-servers-server58-model-4	22.73	0.09	0.45	0.45	73	0.31	64.75
5Z4G_B-servers-server58-model-5	27.27	0.08	0.48	0.43	70	0.39	65.99
5Z4G_B-servers-server61-model-1	50.00	0.14	0.00	0.86	138	0.36	12.42

5Z4G_B-servers-server61-model-2	50.00	0.14	0.00	0.86	138	0.36	12.42
5Z4G_B-servers-server62-model-1	53.33	0.17	0.17	0.67	73	0.73	11.34
5Z4G_B-servers-server62-model-2	53.33	0.17	0.17	0.67	73	0.73	11.34
5Z4G_B-servers-server63-model-1	31.82	0.09	0.47	0.44	60	0.53	58.70
5Z4G_B-servers-server63-model-2	31.82	0.09	0.47	0.44	60	0.53	58.70
5Z4G_B-servers-server64-model-1	22.73	0.10	0.43	0.47	64	0.36	59.47
5Z4G_B-servers-server64-model-2	22.73	0.10	0.43	0.47	64	0.36	59.47
5Z4G_B-servers-server65-model-1	50.00	0.14	0.00	0.86	138	0.36	12.42
5Z4G_B-servers-server65-model-2	50.00	0.14	0.00	0.86	138	0.36	12.42
5Z4G_B-servers-server70-model-1	22.73	0.12	0.10	0.78	114	0.20	21.27
5Z9Y_B-servers-server11-model-1	25.34	0.41	0.13	0.46	116	0.22	90.08
5Z9Y_B-servers-server11-model-2	22.62	0.41	0.13	0.46	117	0.19	89.48
5Z9Y_B-servers-server11-model-3	23.08	0.42	0.13	0.46	115	0.20	89.29
5Z9Y_B-servers-server11-model-4	23.53	0.40	0.13	0.47	119	0.20	90.18
5Z9Y_B-servers-server11-model-5	24.43	0.40	0.13	0.47	118	0.21	89.39
5Z9Y_B-servers-server20-model-1	25.69	0.36	0.10	0.54	135	0.19	84.82
5Z9Y_B-servers-server20-model-2	46.80	0.32	0.06	0.62	132	0.35	25.89
5Z9Y_B-servers-server22-model-1	26.70	0.36	0.12	0.53	133	0.20	85.62
5Z9Y_B-servers-server30-model-1	27.15	0.37	0.11	0.53	133	0.20	84.23
5Z9Y_B-servers-server30-model-2	30.77	0.36	0.10	0.54	135	0.23	76.29
5Z9Y_B-servers-server30-model-3	25.79	0.37	0.12	0.52	131	0.20	86.31
5Z9Y_B-servers-server30-model-4	31.67	0.35	0.11	0.54	135	0.23	84.43
5Z9Y_B-servers-server30-model-5	32.58	0.33	0.09	0.58	145	0.22	21.73
5Z9Y_B-servers-server36-model-1	28.44	0.37	0.12	0.51	124	0.23	84.23
5Z9Y_B-servers-server4-model-1	25.79	0.36	0.12	0.53	133	0.19	89.09
5Z9Y_B-servers-server58-model-1	24.43	0.36	0.12	0.52	132	0.19	86.11
5Z9Y_B-servers-server58-model-2	22.62	0.35	0.12	0.53	134	0.17	86.31
5Z9Y_B-servers-server58-model-3	28.05	0.37	0.12	0.52	131	0.21	87.20
5Z9Y_B-servers-server58-model-4	28.05	0.37	0.12	0.52	130	0.22	87.40
5Z9Y_B-servers-server58-model-5	28.05	0.37	0.12	0.52	130	0.22	87.40
5Z9Y_B-servers-server61-model-1	26.27	0.37	0.12	0.51	121	0.22	84.33
5Z9Y_B-servers-server61-model-2	26.27	0.37	0.12	0.51	121	0.22	84.33
5Z9Y_B-servers-server62-model-1	30.23	0.39	0.10	0.51	119	0.25	83.53
5Z9Y_B-servers-server62-model-2	30.23	0.39	0.10	0.51	119	0.25	83.53
5Z9Y_B-servers-server63-model-1	27.06	0.41	0.13	0.47	108	0.25	82.44
5Z9Y_B-servers-server63-model-2	27.06	0.41	0.13	0.47	108	0.25	82.44
5Z9Y_B-servers-server64-model-1	26.48	0.38	0.11	0.51	121	0.22	84.82
5Z9Y_B-servers-server64-model-2	26.48	0.38	0.11	0.51	121	0.22	84.82
5Z9Y_B-servers-server65-model-1	26.27	0.37	0.12	0.51	121	0.22	84.33
5Z9Y_B-servers-server65-model-2	26.27	0.37	0.12	0.51	121	0.22	84.33
5Z9Y_B-servers-server70-model-1	23.29	0.36	0.12	0.53	132	0.18	86.41
5ZB8_E-servers-server11-model-1	41.96	0.43	0.07	0.50	200	0.21	28.81
5ZB8_E-servers-server11-model-2	44.64	0.39	0.07	0.54	217	0.21	26.81
5ZB8_E-servers-server11-model-3	34.82	0.43	0.06	0.51	205	0.17	28.25
5ZB8_E-servers-server11-model-4	34.82	0.46	0.06	0.49	194	0.18	26.63
5ZB8_E-servers-server11-model-5	47.32	0.45	0.05	0.50	201	0.24	25.75
5ZB8_E-servers-server17-model-1	18.75	0.31	0.10	0.58	111	0.17	27.88

5ZB8_E-servers-server20-model-1	44.64	0.33	0.08	0.59	221	0.20	26.00
5ZB8_E-servers-server20-model-2	34.09	0.29	0.12	0.59	137	0.25	30.06
5ZB8_E-servers-server20-model-3	47.73	0.26	0.09	0.65	145	0.33	22.88
5ZB8_E-servers-server20-model-4	50.68	0.25	0.13	0.62	145	0.35	9.75
5ZB8_E-servers-server20-model-5	57.14	0.31	0.07	0.62	70	0.82	8.19
5ZB8_E-servers-server22-model-1	34.82	0.39	0.06	0.55	221	0.16	27.50
5ZB8_E-servers-server36-model-1	64.29	0.11	0.16	0.73	33	1.95	7.31
5ZB8_E-servers-server4-model-1	38.39	0.24	0.08	0.68	273	0.14	35.63
5ZB8_E-servers-server61-model-1	36.79	0.15	0.07	0.78	295	0.12	7.19
5ZB8_E-servers-server61-model-2	36.79	0.15	0.07	0.78	295	0.12	7.19
5ZB8_E-servers-server62-model-1	50.00	0.15	0.10	0.76	222	0.23	5.81
5ZB8_E-servers-server62-model-2	50.00	0.15	0.10	0.76	222	0.23	5.81
5ZB8_E-servers-server63-model-1	53.57	0.32	0.01	0.68	259	0.21	8.00
5ZB8_E-servers-server63-model-2	53.57	0.32	0.01	0.68	259	0.21	8.00
5ZB8_E-servers-server64-model-1	48.21	0.30	0.06	0.64	254	0.19	17.38
5ZB8_E-servers-server64-model-2	48.21	0.30	0.06	0.64	254	0.19	17.38
5ZB8_E-servers-server65-model-1	36.79	0.15	0.07	0.78	295	0.12	7.19
5ZB8_E-servers-server65-model-2	36.79	0.15	0.07	0.78	295	0.12	7.19
5ZHZ_A-servers-server11-model-1	20.25	0.38	0.12	0.50	125	0.16	80.23
5ZHZ_A-servers-server11-model-2	21.52	0.40	0.12	0.48	121	0.18	77.81
5ZHZ_A-servers-server11-model-3	18.99	0.38	0.13	0.49	124	0.15	80.81
5ZHZ_A-servers-server11-model-4	18.99	0.40	0.10	0.50	126	0.15	77.04
5ZHZ_A-servers-server11-model-5	17.72	0.39	0.12	0.50	125	0.14	78.88
5ZHZ_A-servers-server17-model-1	22.78	0.34	0.13	0.53	130	0.18	64.44
5ZHZ_A-servers-server20-model-1	22.08	0.29	0.11	0.60	148	0.15	57.95
5ZHZ_A-servers-server22-model-1	21.52	0.38	0.11	0.51	132	0.16	73.06
5ZHZ_A-servers-server36-model-1	24.05	0.32	0.11	0.57	143	0.17	60.37
5ZHZ_A-servers-server4-model-1	22.78	0.29	0.12	0.59	152	0.15	72.19
5ZHZ_A-servers-server61-model-1	20.25	0.25	0.12	0.62	161	0.13	64.63
5ZHZ_A-servers-server61-model-2	20.25	0.25	0.12	0.62	161	0.13	64.63
5ZHZ_A-servers-server62-model-1	25.64	0.27	0.13	0.60	155	0.17	65.02
5ZHZ_A-servers-server62-model-2	25.64	0.27	0.13	0.60	155	0.17	65.02
5ZHZ_A-servers-server63-model-1	21.79	0.34	0.11	0.54	136	0.16	62.60
5ZHZ_A-servers-server63-model-2	21.79	0.34	0.11	0.54	136	0.16	62.60
5ZHZ_A-servers-server64-model-1	35.44	0.36	0.09	0.56	144	0.25	40.70
5ZHZ_A-servers-server64-model-2	35.44	0.36	0.09	0.56	144	0.25	40.70
5ZHZ_A-servers-server65-model-1	20.25	0.25	0.12	0.62	161	0.13	64.63
5ZHZ_A-servers-server65-model-2	20.25	0.25	0.12	0.62	161	0.13	64.63
5ZI9_D-servers-server11-model-1	22.03	0.43	0.09	0.48	121	0.18	74.04
5ZI9_D-servers-server11-model-2	20.34	0.45	0.09	0.45	115	0.18	74.33
5ZI9_D-servers-server11-model-3	30.51	0.46	0.09	0.45	114	0.27	72.89
5ZI9_D-servers-server11-model-4	21.19	0.44	0.09	0.46	118	0.18	71.92
5ZI9_D-servers-server11-model-5	22.03	0.46	0.09	0.44	112	0.20	72.79
5ZI9_D-servers-server17-model-1	29.91	0.48	0.13	0.39	78	0.38	67.98
5ZI9_D-servers-server20-model-1	28.81	0.46	0.13	0.40	81	0.36	69.23
5ZI9_D-servers-server22-model-1	22.03	0.52	0.09	0.39	101	0.22	73.94
5ZI9_D-servers-server30-model-1	26.27	0.27	0.09	0.64	166	0.16	68.65

5ZI9_D-servers-server30-model-2	28.81	0.44	0.09	0.47	121	0.24	53.27
5ZI9_D-servers-server30-model-3	32.20	0.38	0.09	0.53	138	0.23	46.44
5ZI9_D-servers-server30-model-4	24.58	0.45	0.08	0.47	122	0.20	50.58
5ZI9_D-servers-server30-model-5	24.58	0.30	0.10	0.60	157	0.16	47.89
5ZI9_D-servers-server36-model-1	30.51	0.49	0.11	0.40	84	0.36	73.46
5ZI9_D-servers-server4-model-1	28.81	0.44	0.10	0.46	119	0.24	68.56
5ZI9_D-servers-server58-model-1	28.81	0.40	0.09	0.51	133	0.22	75.87
5ZI9_D-servers-server58-model-2	28.81	0.40	0.09	0.51	133	0.22	75.87
5ZI9_D-servers-server58-model-3	28.81	0.40	0.09	0.51	133	0.22	75.87
5ZI9_D-servers-server58-model-4	28.81	0.40	0.09	0.51	133	0.22	75.87
5ZI9_D-servers-server58-model-5	27.97	0.40	0.09	0.51	133	0.21	76.44
5ZI9_D-servers-server61-model-1	26.27	0.50	0.13	0.38	78	0.34	69.23
5ZI9_D-servers-server61-model-2	26.27	0.50	0.13	0.38	78	0.34	69.23
5ZI9_D-servers-server62-model-1	27.12	0.47	0.13	0.41	84	0.32	69.42
5ZI9_D-servers-server62-model-2	27.12	0.47	0.13	0.41	84	0.32	69.42
5ZI9_D-servers-server63-model-1	29.66	0.46	0.13	0.41	84	0.35	69.42
5ZI9_D-servers-server63-model-2	29.66	0.46	0.13	0.41	84	0.35	69.42
5ZI9_D-servers-server64-model-1	26.27	0.50	0.13	0.38	78	0.34	69.23
5ZI9_D-servers-server64-model-2	26.27	0.50	0.13	0.38	78	0.34	69.23
5ZI9_D-servers-server65-model-1	26.27	0.50	0.13	0.38	78	0.34	69.23
5ZI9_D-servers-server65-model-2	26.27	0.50	0.13	0.38	78	0.34	69.23
5ZI9_D-servers-server70-model-1	27.97	0.48	0.11	0.40	87	0.32	72.31
6AU1_B-servers-server11-model-1	17.11	0.40	0.11	0.49	176	0.10	87.61
6AU1_B-servers-server11-model-2	18.42	0.38	0.10	0.51	183	0.10	88.66
6AU1_B-servers-server11-model-3	17.11	0.42	0.10	0.48	172	0.10	88.17
6AU1_B-servers-server11-model-4	15.79	0.40	0.10	0.50	179	0.09	86.49
6AU1_B-servers-server11-model-5	17.11	0.41	0.10	0.49	174	0.10	89.15
6AU1_B-servers-server17-model-1	14.47	0.39	0.10	0.50	177	0.08	84.31
6AU1_B-servers-server20-model-1	17.11	0.39	0.11	0.50	176	0.10	85.85
6AU1_B-servers-server20-model-2	50.00	0.33	0.10	0.57	35	1.43	6.93
6AU1_B-servers-server22-model-1	17.11	0.40	0.11	0.49	176	0.10	86.28
6AU1_B-servers-server30-model-1	17.11	0.43	0.10	0.47	167	0.10	75.35
6AU1_B-servers-server30-model-2	21.05	0.34	0.08	0.58	208	0.10	46.71
6AU1_B-servers-server30-model-3	28.95	0.27	0.08	0.64	230	0.13	31.44
6AU1_B-servers-server30-model-4	27.63	0.26	0.09	0.66	234	0.12	31.65
6AU1_B-servers-server30-model-5	19.74	0.36	0.06	0.58	206	0.10	46.85
6AU1_B-servers-server36-model-1	19.74	0.40	0.11	0.49	170	0.12	82.77
6AU1_B-servers-server4-model-1	19.74	0.40	0.11	0.49	176	0.11	88.80
6AU1_B-servers-server58-model-1	19.74	0.40	0.10	0.50	178	0.11	76.89
6AU1_B-servers-server58-model-2	19.74	0.39	0.10	0.51	181	0.11	87.89
6AU1_B-servers-server58-model-3	14.47	0.40	0.10	0.50	179	0.08	77.17
6AU1_B-servers-server58-model-4	17.11	0.43	0.10	0.48	170	0.10	79.41
6AU1_B-servers-server58-model-5	25.00	0.39	0.10	0.51	181	0.14	75.28
6AU1_B-servers-server61-model-1	18.42	0.40	0.10	0.49	174	0.11	84.17
6AU1_B-servers-server61-model-2	18.42	0.40	0.10	0.49	174	0.11	84.17
6AU1_B-servers-server62-model-1	23.68	0.41	0.11	0.48	166	0.14	84.31
6AU1_B-servers-server62-model-2	23.68	0.41	0.11	0.48	166	0.14	84.31

6AU1_B-servers-server63-model-1	22.97	0.41	0.10	0.49	169	0.14	86.42
6AU1_B-servers-server63-model-2	22.97	0.41	0.10	0.49	169	0.14	86.42
6AU1_B-servers-server64-model-1	19.74	0.41	0.10	0.49	174	0.11	87.75
6AU1_B-servers-server64-model-2	19.74	0.41	0.10	0.49	174	0.11	87.75
6AU1_B-servers-server65-model-1	18.42	0.40	0.10	0.49	174	0.11	84.17
6AU1_B-servers-server65-model-2	18.42	0.40	0.10	0.49	174	0.11	84.17
6AU1_B-servers-server70-model-1	17.11	0.40	0.10	0.50	179	0.10	86.63
6CK0_B-servers-server11-model-1	18.75	0.26	0.26	0.48	101	0.19	67.39
6CK0_B-servers-server11-model-2	17.50	0.27	0.29	0.43	92	0.19	67.96
6CK0_B-servers-server11-model-3	16.25	0.26	0.29	0.44	94	0.17	67.73
6CK0_B-servers-server11-model-4	20.00	0.25	0.24	0.51	109	0.18	64.77
6CK0_B-servers-server11-model-5	21.25	0.29	0.25	0.46	97	0.22	64.66
6CK0_B-servers-server17-model-1	23.38	0.23	0.27	0.49	97	0.24	51.48
6CK0_B-servers-server20-model-1	20.51	0.22	0.28	0.50	102	0.20	63.30
6CK0_B-servers-server22-model-1	21.25	0.28	0.30	0.43	94	0.23	60.57
6CK0_B-servers-server36-model-1	36.00	0.20	0.28	0.52	66	0.55	46.02
6CK0_B-servers-server4-model-1	20.00	0.23	0.25	0.52	114	0.18	68.07
6CK0_B-servers-server61-model-1	20.00	0.25	0.14	0.61	135	0.15	52.84
6CK0_B-servers-server61-model-2	20.00	0.25	0.14	0.61	135	0.15	52.84
6CK0_B-servers-server62-model-1	20.78	0.19	0.19	0.62	131	0.16	53.98
6CK0_B-servers-server62-model-2	20.78	0.19	0.19	0.62	131	0.16	53.98
6CK0_B-servers-server63-model-1	25.00	0.23	0.24	0.53	111	0.23	54.43
6CK0_B-servers-server63-model-2	25.00	0.23	0.24	0.53	111	0.23	54.43
6CK0_B-servers-server64-model-1	30.00	0.19	0.19	0.62	128	0.23	38.75
6CK0_B-servers-server64-model-2	30.00	0.19	0.19	0.62	128	0.23	38.75
6CK0_B-servers-server65-model-1	20.00	0.25	0.14	0.61	135	0.15	52.84
6CK0_B-servers-server65-model-2	20.00	0.25	0.14	0.61	135	0.15	52.84
6CKG_B-servers-server11-model-1	35.29	0.43	0.14	0.43	139	0.25	46.82
6CKG_B-servers-server11-model-2	36.27	0.42	0.10	0.48	156	0.23	49.32
6CKG_B-servers-server11-model-3	38.24	0.45	0.10	0.45	144	0.27	49.32
6CKG_B-servers-server11-model-4	31.37	0.43	0.12	0.45	144	0.22	52.27
6CKG_B-servers-server11-model-5	28.43	0.46	0.12	0.43	137	0.21	46.29
6CKG_B-servers-server17-model-1	25.29	0.43	0.15	0.41	110	0.23	58.33
6CKG_B-servers-server20-model-1	32.35	0.35	0.11	0.54	169	0.19	58.11
6CKG_B-servers-server20-model-2	38.36	0.29	0.14	0.57	133	0.29	27.05
6CKG_B-servers-server20-model-3	57.89	0.35	0.05	0.60	90	0.64	9.92
6CKG_B-servers-server22-model-1	27.45	0.41	0.12	0.48	158	0.17	60.53
6CKG_B-servers-server36-model-1	27.84	0.35	0.15	0.50	152	0.18	52.50
6CKG_B-servers-server4-model-1	30.39	0.35	0.10	0.55	182	0.17	63.18
6CKG_B-servers-server61-model-1	29.41	0.34	0.11	0.55	169	0.17	55.83
6CKG_B-servers-server61-model-2	29.41	0.34	0.11	0.55	169	0.17	55.83
6CKG_B-servers-server62-model-1	28.71	0.37	0.15	0.49	150	0.19	57.73
6CKG_B-servers-server62-model-2	28.71	0.37	0.15	0.49	150	0.19	57.73
6CKG_B-servers-server63-model-1	30.69	0.36	0.13	0.51	157	0.20	58.26
6CKG_B-servers-server63-model-2	30.69	0.36	0.13	0.51	157	0.20	58.26
6CKG_B-servers-server64-model-1	29.41	0.34	0.11	0.55	169	0.17	55.83
6CKG_B-servers-server64-model-2	29.41	0.34	0.11	0.55	169	0.17	55.83

6CKG_B-servers-server65-model-1	29.41	0.34	0.11	0.55	169	0.17	55.83
6CKG_B-servers-server65-model-2	29.41	0.34	0.11	0.55	169	0.17	55.83
6CKP_A-servers-server11-model-1	25.71	0.32	0.20	0.48	58	0.44	82.23
6CKP_A-servers-server11-model-2	22.86	0.32	0.20	0.48	58	0.39	82.42
6CKP_A-servers-server11-model-3	25.71	0.32	0.20	0.48	58	0.44	83.20
6CKP_A-servers-server11-model-4	28.57	0.32	0.20	0.48	58	0.49	83.01
6CKP_A-servers-server11-model-5	25.71	0.36	0.20	0.44	53	0.49	81.64
6CKP_A-servers-server17-model-1	25.71	0.37	0.22	0.42	44	0.58	70.90
6CKP_A-servers-server20-model-1	28.57	0.31	0.21	0.48	52	0.55	78.13
6CKP_A-servers-server22-model-1	22.86	0.43	0.17	0.40	51	0.45	81.25
6CKP_A-servers-server36-model-1	28.57	0.38	0.22	0.40	42	0.68	77.34
6CKP_A-servers-server4-model-1	31.43	0.30	0.19	0.52	66	0.48	80.47
6CKP_A-servers-server61-model-1	34.29	0.39	0.22	0.39	41	0.84	77.54
6CKP_A-servers-server61-model-2	34.29	0.39	0.22	0.39	41	0.84	77.54
6CKP_A-servers-server62-model-1	28.57	0.38	0.22	0.41	43	0.66	77.73
6CKP_A-servers-server62-model-2	28.57	0.38	0.22	0.41	43	0.66	77.73
6CKP_A-servers-server63-model-1	31.43	0.37	0.21	0.42	45	0.70	74.61
6CKP_A-servers-server63-model-2	31.43	0.37	0.21	0.42	45	0.70	74.61
6CKP_A-servers-server64-model-1	28.57	0.38	0.20	0.42	45	0.63	75.00
6CKP_A-servers-server64-model-2	28.57	0.38	0.20	0.42	45	0.63	75.00
6CKP_A-servers-server65-model-1	34.29	0.39	0.22	0.39	41	0.84	77.54
6CKP_A-servers-server65-model-2	34.29	0.39	0.22	0.39	41	0.84	77.54
5VH2_D-servers-server11-model-1	38.71	0.06	0.37	0.57	122	0.32	65.00
5VH2_D-servers-server11-model-2	33.33	0.06	0.37	0.57	122	0.27	65.80
5VH2_D-servers-server11-model-3	38.71	0.02	0.37	0.60	129	0.30	65.00
5VH2_D-servers-server11-model-4	34.41	0.05	0.35	0.60	129	0.27	41.93
5VH2_D-servers-server11-model-5	36.56	0.02	0.35	0.63	135	0.27	41.93
5VH2_D-servers-server17-model-1	40.91	0.01	0.40	0.59	120	0.34	38.86
5VH2_D-servers-server20-model-1	41.94	0.01	0.40	0.59	125	0.34	62.61
5VH2_D-servers-server22-model-1	36.56	0.03	0.36	0.60	133	0.27	57.96
5VH2_D-servers-server30-model-1	31.18	0.01	0.35	0.65	142	0.22	63.64
5VH2_D-servers-server30-model-2	33.33	0.03	0.36	0.60	133	0.25	54.89
5VH2_D-servers-server30-model-3	36.56	0.02	0.35	0.62	137	0.27	58.07
5VH2_D-servers-server30-model-4	39.78	0.03	0.36	0.60	133	0.30	56.82
5VH2_D-servers-server30-model-5	34.41	0.02	0.35	0.62	137	0.25	60.91
5VH2_D-servers-server36-model-1	36.56	0.02	0.38	0.59	124	0.29	59.09
5VH2_D-servers-server4-model-1	36.56	0.03	0.28	0.69	151	0.24	68.07
5VH2_D-servers-server58-model-1	36.56	0.01	0.37	0.62	137	0.27	58.64
5VH2_D-servers-server58-model-2	33.33	0.03	0.40	0.57	125	0.27	65.23
5VH2_D-servers-server58-model-3	37.63	0.03	0.36	0.61	135	0.28	62.84
5VH2_D-servers-server58-model-4	33.33	0.02	0.36	0.61	135	0.25	66.48
5VH2_D-servers-server58-model-5	39.78	0.04	0.35	0.61	134	0.30	60.68
5VH2_D-servers-server61-model-1	35.48	0.01	0.37	0.62	130	0.27	58.18
5VH2_D-servers-server61-model-2	35.48	0.01	0.37	0.62	130	0.27	58.18
5VH2_D-servers-server62-model-1	41.94	0.04	0.31	0.65	137	0.31	51.48
5VH2_D-servers-server62-model-2	41.94	0.04	0.31	0.65	137	0.31	51.48
5VH2_D-servers-server63-model-1	37.63	0.02	0.39	0.59	125	0.30	54.55

5VH2_D-servers-server63-model-2	37.63	0.02	0.39	0.59	125	0.30	54.55
5VH2_D-servers-server64-model-1	36.56	0.02	0.42	0.56	119	0.31	53.07
5VH2_D-servers-server64-model-2	36.56	0.02	0.42	0.56	119	0.31	53.07
5VH2_D-servers-server65-model-1	35.48	0.01	0.37	0.62	130	0.27	58.18
5VH2_D-servers-server65-model-2	35.48	0.01	0.37	0.62	130	0.27	58.18
5VH2_D-servers-server70-model-1	41.30	0.02	0.36	0.62	129	0.32	48.64
5Z68_D-servers-server11-model-1	29.19	0.44	0.17	0.40	146	0.20	73.53
5Z68_D-servers-server11-model-2	28.57	0.43	0.18	0.38	142	0.20	56.90
5Z68_D-servers-server11-model-3	32.30	0.43	0.17	0.41	151	0.21	62.80
5Z68_D-servers-server11-model-4	34.78	0.42	0.17	0.41	151	0.23	66.42
5Z68_D-servers-server11-model-5	28.57	0.38	0.15	0.47	172	0.17	57.78
5Z68_D-servers-server17-model-1	34.39	0.43	0.20	0.37	129	0.27	61.80
5Z68_D-servers-server20-model-1	34.18	0.38	0.17	0.45	163	0.21	65.55
5Z68_D-servers-server20-model-2	51.95	0.34	0.13	0.53	193	0.27	24.67
5Z68_D-servers-server20-model-3	36.42	0.35	0.16	0.49	177	0.21	30.50
5Z68_D-servers-server20-model-4	33.99	0.35	0.17	0.48	172	0.20	35.79
5Z68_D-servers-server20-model-5	34.19	0.32	0.15	0.52	190	0.18	35.59
5Z68_D-servers-server22-model-1	31.68	0.40	0.19	0.41	152	0.21	68.23
5Z68_D-servers-server30-model-1	32.30	0.38	0.17	0.45	167	0.19	67.43
5Z68_D-servers-server30-model-2	36.02	0.32	0.17	0.51	191	0.19	39.48
5Z68_D-servers-server30-model-3	39.75	0.26	0.16	0.58	218	0.18	34.65
5Z68_D-servers-server30-model-4	35.40	0.29	0.18	0.53	198	0.18	37.80
5Z68_D-servers-server30-model-5	37.27	0.18	0.13	0.69	258	0.14	39.21
5Z68_D-servers-server36-model-1	34.06	0.45	0.13	0.42	140	0.24	54.96
5Z68_D-servers-server4-model-1	31.68	0.38	0.15	0.47	177	0.18	65.62
5Z68_D-servers-server58-model-1	29.81	0.38	0.18	0.43	162	0.18	67.43
5Z68_D-servers-server58-model-2	29.81	0.38	0.18	0.43	162	0.18	67.43
5Z68_D-servers-server58-model-3	29.81	0.38	0.18	0.43	162	0.18	67.43
5Z68_D-servers-server58-model-4	29.81	0.38	0.18	0.43	162	0.18	67.43
5Z68_D-servers-server58-model-5	31.06	0.38	0.18	0.44	165	0.19	64.81
5Z68_D-servers-server61-model-1	33.13	0.41	0.18	0.41	150	0.22	62.27
5Z68_D-servers-server61-model-2	33.13	0.41	0.18	0.41	150	0.22	62.27
5Z68_D-servers-server62-model-1	30.00	0.39	0.19	0.42	154	0.19	63.34
5Z68_D-servers-server62-model-2	30.00	0.39	0.19	0.42	154	0.19	63.34
5Z68_D-servers-server63-model-1	30.00	0.38	0.14	0.48	177	0.17	57.11
5Z68_D-servers-server63-model-2	30.00	0.38	0.14	0.48	177	0.17	57.11
5Z68_D-servers-server64-model-1	33.13	0.41	0.18	0.41	150	0.22	62.27
5Z68_D-servers-server64-model-2	33.13	0.41	0.18	0.41	150	0.22	62.27
5Z68_D-servers-server65-model-1	33.13	0.41	0.18	0.41	150	0.22	62.27
5Z68_D-servers-server65-model-2	33.13	0.41	0.18	0.41	150	0.22	62.27
5Z68_D-servers-server70-model-1	30.63	0.40	0.19	0.42	154	0.20	66.22
5OV3_B-servers-server11-model-1	23.56	0.03	0.35	0.62	236	0.10	57.55
5OV3_B-servers-server11-model-2	25.86	0.04	0.36	0.61	231	0.11	59.51
5OV3_B-servers-server11-model-3	21.84	0.06	0.33	0.61	234	0.09	57.15
5OV3_B-servers-server11-model-4	20.11	0.04	0.35	0.60	230	0.09	56.82
5OV3_B-servers-server11-model-5	25.86	0.03	0.34	0.63	240	0.11	56.76
5OV3_B-servers-server20-model-1	31.40	0.01	0.40	0.60	191	0.16	41.67

5OV3_B-servers-server20-model-2	39.77	0.01	0.39	0.60	184	0.22	34.19
5OV3_B-servers-server20-model-3	33.33	0.03	0.40	0.57	179	0.19	33.99
5OV3_B-servers-server20-model-4	36.31	0.01	0.35	0.64	203	0.18	36.88
5OV3_B-servers-server20-model-5	36.31	0.01	0.38	0.61	202	0.18	43.57
5OV3_B-servers-server22-model-1	26.44	0.04	0.36	0.59	202	0.13	54.66
5OV3_B-servers-server30-model-1	22.41	0.01	0.35	0.64	243	0.09	52.43
5OV3_B-servers-server30-model-2	22.99	0.00	0.34	0.66	251	0.09	42.32
5OV3_B-servers-server30-model-3	21.84	0.01	0.32	0.67	255	0.09	49.28
5OV3_B-servers-server30-model-4	21.26	0.00	0.33	0.67	256	0.08	49.28
5OV3_B-servers-server30-model-5	21.84	0.01	0.32	0.68	258	0.08	49.54
5OV3_B-servers-server33-model-1	26.44	0.00	0.33	0.67	256	0.10	54.27
5OV3_B-servers-server33-model-2	26.44	0.00	0.33	0.67	256	0.10	54.27
5OV3_B-servers-server33-model-3	26.44	0.00	0.33	0.67	256	0.10	54.27
5OV3_B-servers-server33-model-4	26.44	0.00	0.33	0.67	256	0.10	54.27
5OV3_B-servers-server36-model-1	28.85	0.00	0.39	0.61	174	0.17	42.85
5OV3_B-servers-server4-model-1	22.41	0.00	0.35	0.65	249	0.09	53.35
5OV3_B-servers-server61-model-1	29.76	0.00	0.35	0.65	195	0.15	47.31
5OV3_B-servers-server61-model-2	29.76	0.00	0.35	0.65	195	0.15	47.31
5OV3_B-servers-server62-model-1	33.55	0.00	0.16	0.84	253	0.13	41.86
5OV3_B-servers-server62-model-2	33.55	0.00	0.16	0.84	253	0.13	41.86
5OV3_B-servers-server65-model-1	29.76	0.00	0.35	0.65	195	0.15	47.31
5OV3_B-servers-server65-model-2	29.76	0.00	0.35	0.65	195	0.15	47.31
5OV3_B-servers-server70-model-1	28.07	0.00	0.38	0.62	194	0.14	43.83

Supplementary Table S6. List of 52 CAMEO targets.

**Target Name**	**Sequence Length**
5MM8_A	204
5NVA_A	496
5O6C_A	263
5OJY_A	254
5OUN_A	107
5OV3_B	381
5OVY_A	222
5TOS_B	395
5TXR_A	491
5U7Z_C	129
5U7Z_D	255
5U81_A	384
5U84_B	384
5UD7_F	169
5V8C_A	293
5VFX_H	107
5VG2_C	229
5VGU_F	125
5VH2_D	220
5W35_B	325
5WEE_D	195
5WJD_A	159
5WLY_A	283
5X2B_L	283
5X7Y_D	177
5XB6_L	306
5XBV_A	149
5XCA_A	190
5XD6_B	305
5XDY_A	337
5XEO_B	310
5XEP_F	381
5XFL_D	375
5XJV_B	185
5XOM_B	393
5XPW_A	180
5XVS_B	379
5Y4B_A	93
5Y8E_A	154
5YH0_L	560

5Z11_B	128
5Z4G_B	161
5Z68_D	373
5Z9Y_B	252
5ZB8_E	400
5ZHZ_A	258
5ZI9_D	260
6AU1_B	357
6CK0_B	220
6CKG_B	330
6CKP_A	128
